# Supplementary material for: Divergent evolution of the PRPS enzymes across the tree of life
Source: bioRxiv. 2026 Jun 3:2026.06.01.728777. Preprint. [Version 1] doi: 10.64898/2026.06.01.728777 (PMC13252144; doi:10.64898/2026.06.01.728777)
Supplement: Supplement 10 [file NIHPP2026.06.01.728777v1-supplement-10.pdf]

bioRxiv preprint doi: https://doi.org/10.64898/2026.06.01.728777; this version posted June 3, 2026. The copyright holder for this preprint (which was not certified by peer review) is the author/funder, who has granted bioRxiv a license to display the preprint in perpetuity. It is made available under a CC-BY 4.0 International license.

Supplementary Fig. 1

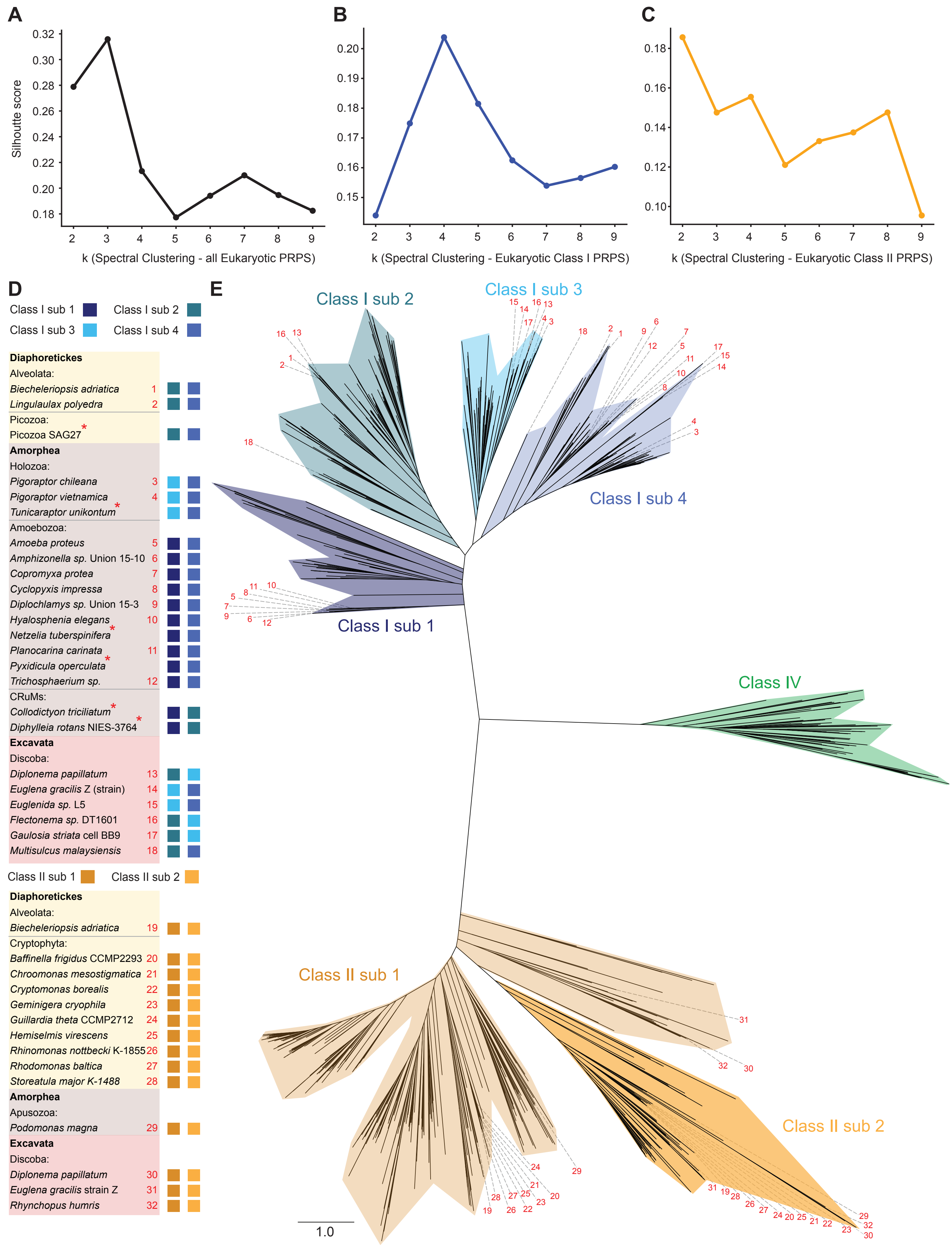

bioRxiv preprint doi: https://doi.org/10.64898/2026.06.01.728777; this version posted June 3, 2026. The copyright holder for this preprint (which was not certified by peer review) is the author/funder, who has granted bioRxiv a license to display the preprint in perpetuity. It is made available under a CC-BY 4.0 International license.

Supplementary Figure 1. Robust clustering and subclass co-existence validate duplications of ancestral eukaryotic PRPS genes.

(A-C) Silhouette score analysis of spectral clustering on a pairwise distance matrix of all eukaryotic PRPS sequences (A), eukaryotic Class I PRPS sequences (B), and eukaryotic Class II PRPS sequences (C) across k = 2-9. **(D)** Distribution of PRPS subclasses across eukaryotic lineages. Presence of subclasses is shown across major eukaryotic supergroups, highlighting species encoding multiple PRPS subclasses. Asterisks denote species for which one or more PRPS sequences were partial and excluded from the gene tree analysis in Figure 1C. **(E)** Positions of PRPS homologs from multi-subclass species across the inferred eukaryotic PRPS gene tree, with numbered labels corresponding to species shown in (D).

bioRxiv preprint doi: https://doi.org/10.64898/2026.06.01.728777; this version posted June 3, 2026. The copyright holder for this preprint (which was not certified by peer review) is the author/funder, who has granted bioRxiv a license to display the preprint in perpetuity. It is made available under a CC-BY 4.0 International license.

Supplementary Fig. 2

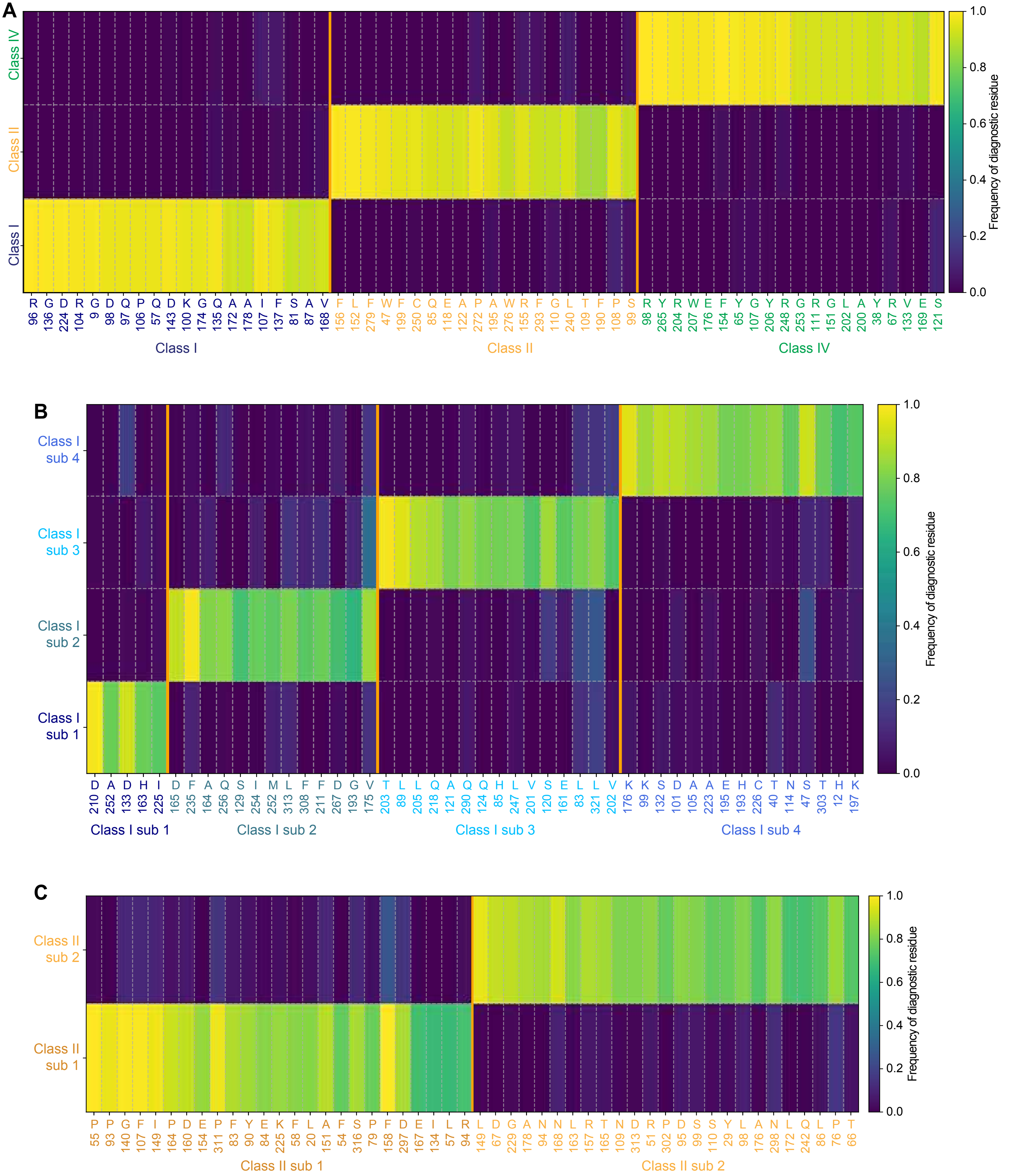

bioRxiv preprint doi: https://doi.org/10.64898/2026.06.01.728777; this version posted June 3, 2026. The copyright holder for this preprint (which was not certified by peer review) is the author/funder, who has granted bioRxiv a license to display the preprint in perpetuity. It is made available under a CC-BY 4.0 International license.

Supplementary Figure 2. Diagnostic residue signatures define distinct PRPS classes and subclasses in eukaryotes.

(A-C) Heatmaps showing diagnostic residues distinguishing eukaryotic PRPS classes (A), eukaryotic Class I subclasses (B), and eukaryotic Class II subclasses (C). Rows correspond to classes or subclasses, and columns represent alignment positions meeting stringent diagnostic criteria (frequency, coverage, and statistical criteria – see Methods). Cells indicate residue frequency (0-1) within each group, calculated from non-gap residues at each position. Only the top diagnostic sites per class are shown. Diagnostic site positions for Class I, Class II, and Class IV PRPS shown in (A), for Class I subclasses shown in (B), and for Class II subclasses shown in (C) correspond to representative organism sequences shown in Supplementary Figure 3. Group sizes for (A): Class I (n = 417), Class II (n = 422), Class IV (n = 68). Group sizes for (B): Class I sub 1 (n = 71), sub 2 (n = 155), sub 3 (n = 84), sub 4 (n = 107). Group sizes for (C): Class II sub 1 (n = 341), sub 2 (n = 81).

bioRxiv preprint doi: https://doi.org/10.64898/2026.06.01.728777; this version posted June 3, 2026. The copyright holder for this preprint (which was not certified by peer review) is the author/funder, who has granted bioRxiv a license to display the preprint in perpetuity. It is made available under a CC-BY 4.0 International license.

Supplementary Fig. 3

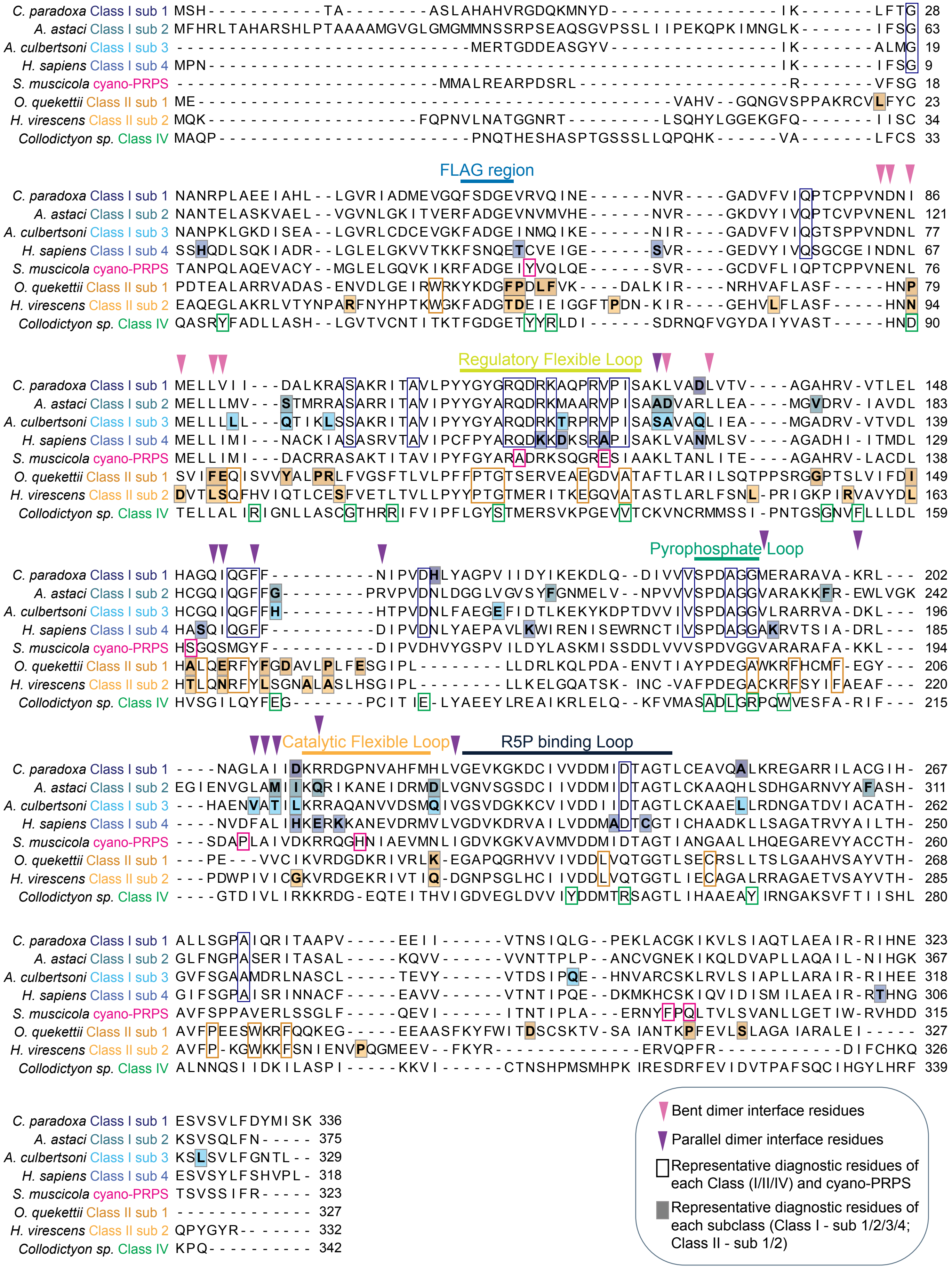

bioRxiv preprint doi: https://doi.org/10.64898/2026.06.01.728777; this version posted June 3, 2026. The copyright holder for this preprint (which was not certified by peer review) is the author/funder, who has granted bioRxiv a license to display the preprint in perpetuity. It is made available under a CC-BY 4.0 International license.

Supplementary Figure 3. Diagnostic residues map to both established functional regions and residues of undetermined significance across PRPS classes/subclasses.

Aligned PRPS sequences from representative eukaryotic species across classes and subclasses, with diagnostic residues highlighted. Functional regions, including the FLAG region, regulatory flexible loop, pyrophosphate loop, catalytic flexible loop, and R5P-binding loop, are shown. Residues contributing to parallel and bent dimer interfaces are indicated. Diagnostic residues correspond to class-level (Class I, II, IV, and cyano-PRPS) and subclass-level signatures shown in Supplementary Figure 2.

bioRxiv preprint doi: https://doi.org/10.64898/2026.06.01.728777; this version posted June 3, 2026. The copyright holder for this preprint (which was not certified by peer review) is the author/funder, who has granted bioRxiv a license to display the preprint in perpetuity. It is made available under a CC-BY 4.0 International license.

Supplementary Fig. 4

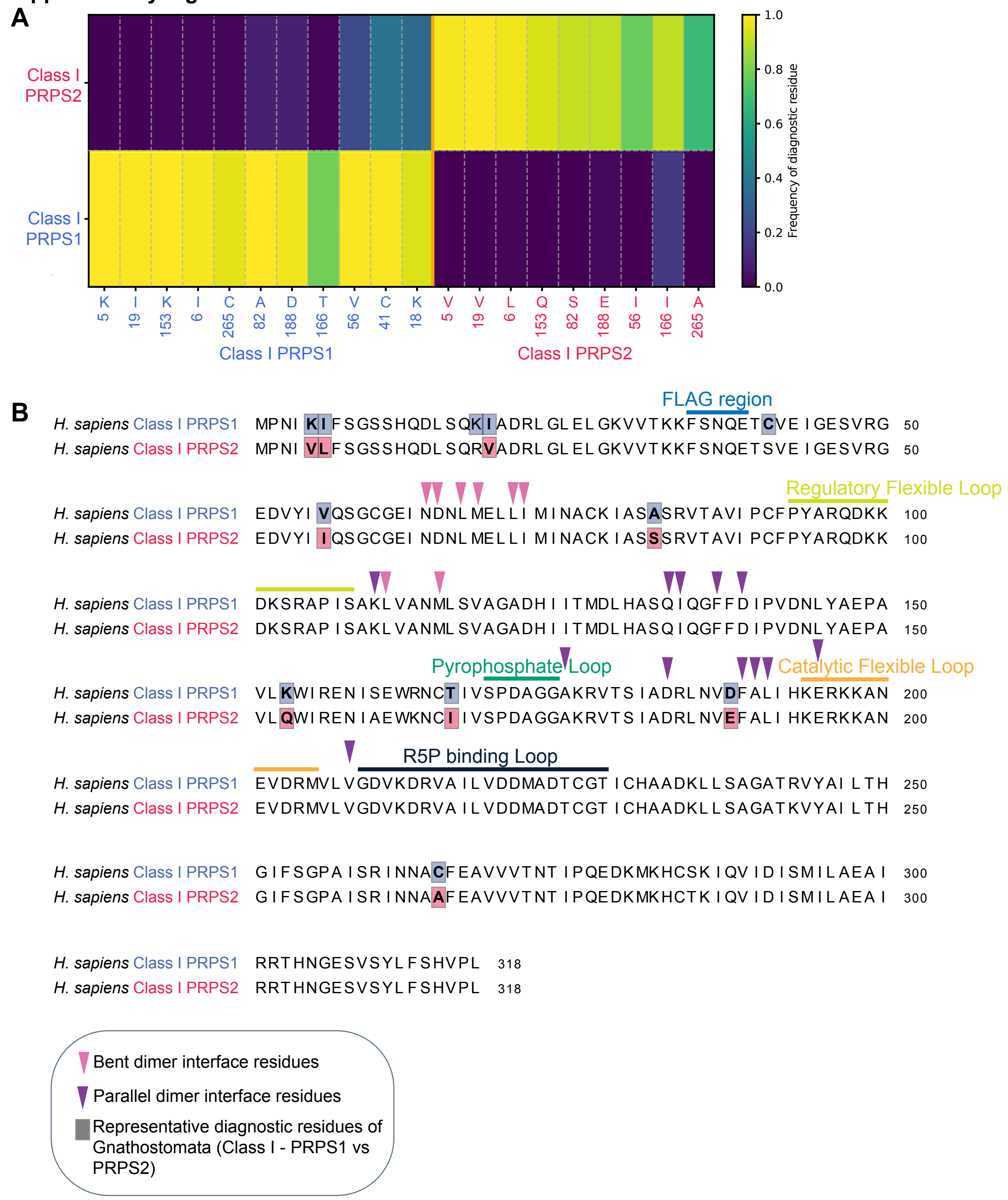

bioRxiv preprint doi: https://doi.org/10.64898/2026.06.01.728777; this version posted June 3, 2026. The copyright holder for this preprint (which was not certified by peer review) is the author/funder, who has granted bioRxiv a license to display the preprint in perpetuity. It is made available under a CC-BY 4.0 International license.

Supplementary Figure 4. Diagnostic residues distinguish Gnathostomata PRPS1 and PRPS2.

(A) Heatmap of diagnostic residues distinguishing PRPS1 and PRPS2. Rows correspond to PRPS1 and PRPS2, and columns represent alignment positions meeting stringent diagnostic criteria (frequency, coverage, and statistical criteria – see Methods). Cells indicate residue frequency (0-1) within each group, calculated from non-gap residues at each position. Diagnostic site positions for PRPS1 and PRPS2 shown correspond to human PRPS1 and PRPS2, respectively shown in (B). Group sizes: PRPS1 = 150, PRPS2 = 150. **(B)** Representative sequence alignment of human PRPS1 and PRPS2 with highlighted diagnostic residues identified in (A). Functional regions, including the FLAG region, regulatory flexible loop, pyrophosphate loop, catalytic flexible loop, and R5P-binding loop, are shown. Residues contributing to parallel and bent dimer interfaces are indicated.

bioRxiv preprint doi: https://doi.org/10.64898/2026.06.01.728777; this version posted June 3, 2026. The copyright holder for this preprint (which was not certified by peer review) is the author/funder, who has granted bioRxiv a license to display the preprint in perpetuity. It is made available under a CC-BY 4.0 International license.

Supplementary Fig. 5

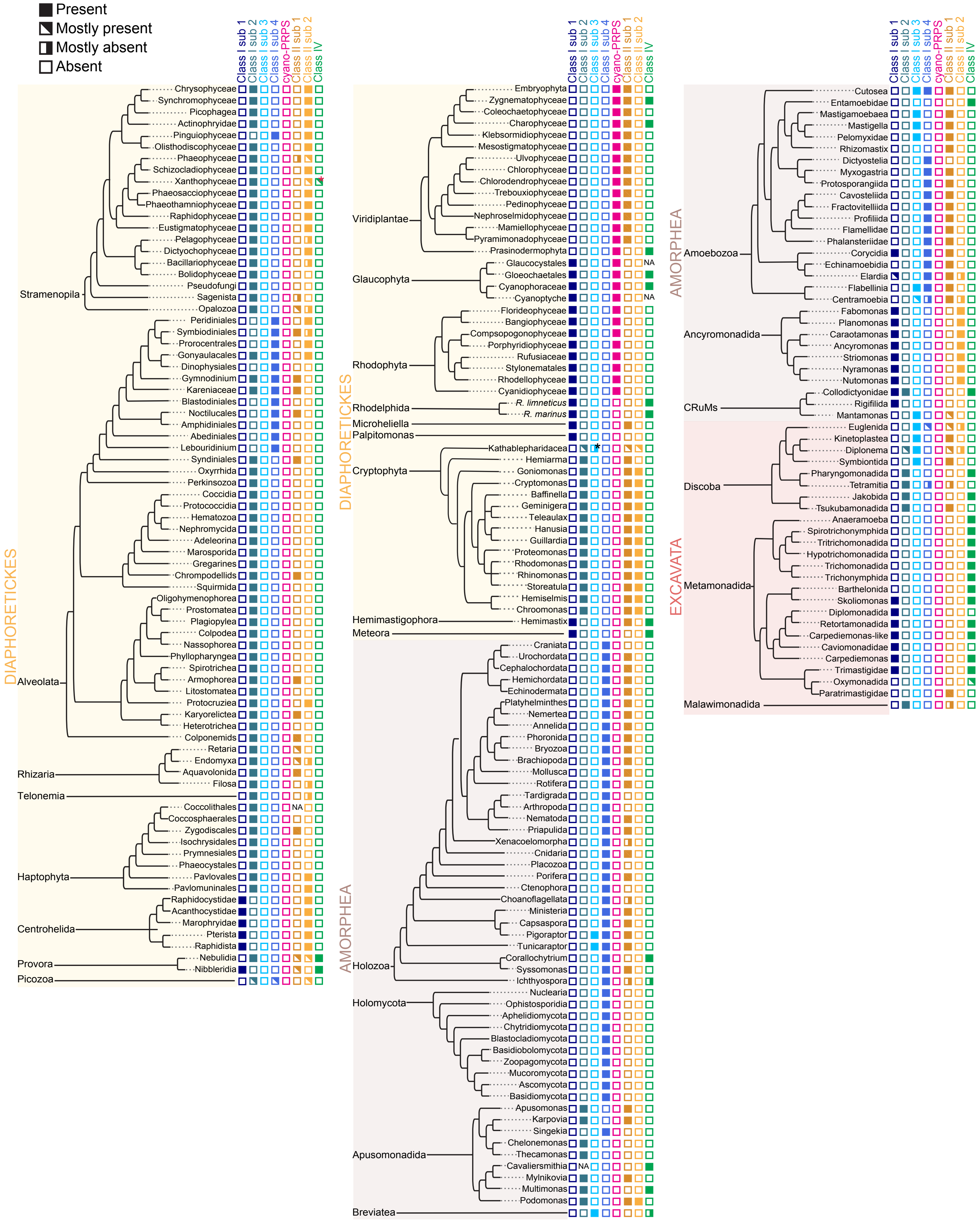

bioRxiv preprint doi: https://doi.org/10.64898/2026.06.01.728777; this version posted June 3, 2026. The copyright holder for this preprint (which was not certified by peer review) is the author/funder, who has granted bioRxiv a license to display the preprint in perpetuity. It is made available under a CC-BY 4.0 International license.

Supplementary Figure 5. Lineage-specific PRPS gene losses drive heterogeneous distribution of PRPS classes/subclasses across eukaryotes.

Expanded eukaryotic phylogenetic tree showing distribution of PRPS classes across major and minor lineages. Presence and absence are indicated using a categorical scheme (present, mostly present, mostly absent, absent) based on the proportion of sampled taxa within each lineage. "NA" denotes lineages for which presence/absence could not be determined due to incomplete or unavailable datasets. Lineages are organized by eukaryotic supergroups – Diaphoretickes, Amorphea, and Excavata – with expanded taxonomic resolution relative to Figure 1D. Colored annotations correspond to PRPS subclasses (Class I subclasses 1-4, cyano-PRPS, Class II subclasses 1-2, and Class IV). Black asterisk denotes Class II PRPS restricted to a single Cryptophyta species (uncultured Katablepharidaceae), consistent with horizontal gene transfer, and red asterisk indicates Class IV PRPS in a limited number of Stramenopila (three Xanthophyceae species), also suggestive of horizontal gene transfer.

bioRxiv preprint doi: https://doi.org/10.64898/2026.06.01.728777; this version posted June 3, 2026. The copyright holder for this preprint (which was not certified by peer review) is the author/funder, who has granted bioRxiv a license to display the preprint in perpetuity. It is made available under a CC-BY 4.0 International license.

Supplementary Fig. 6

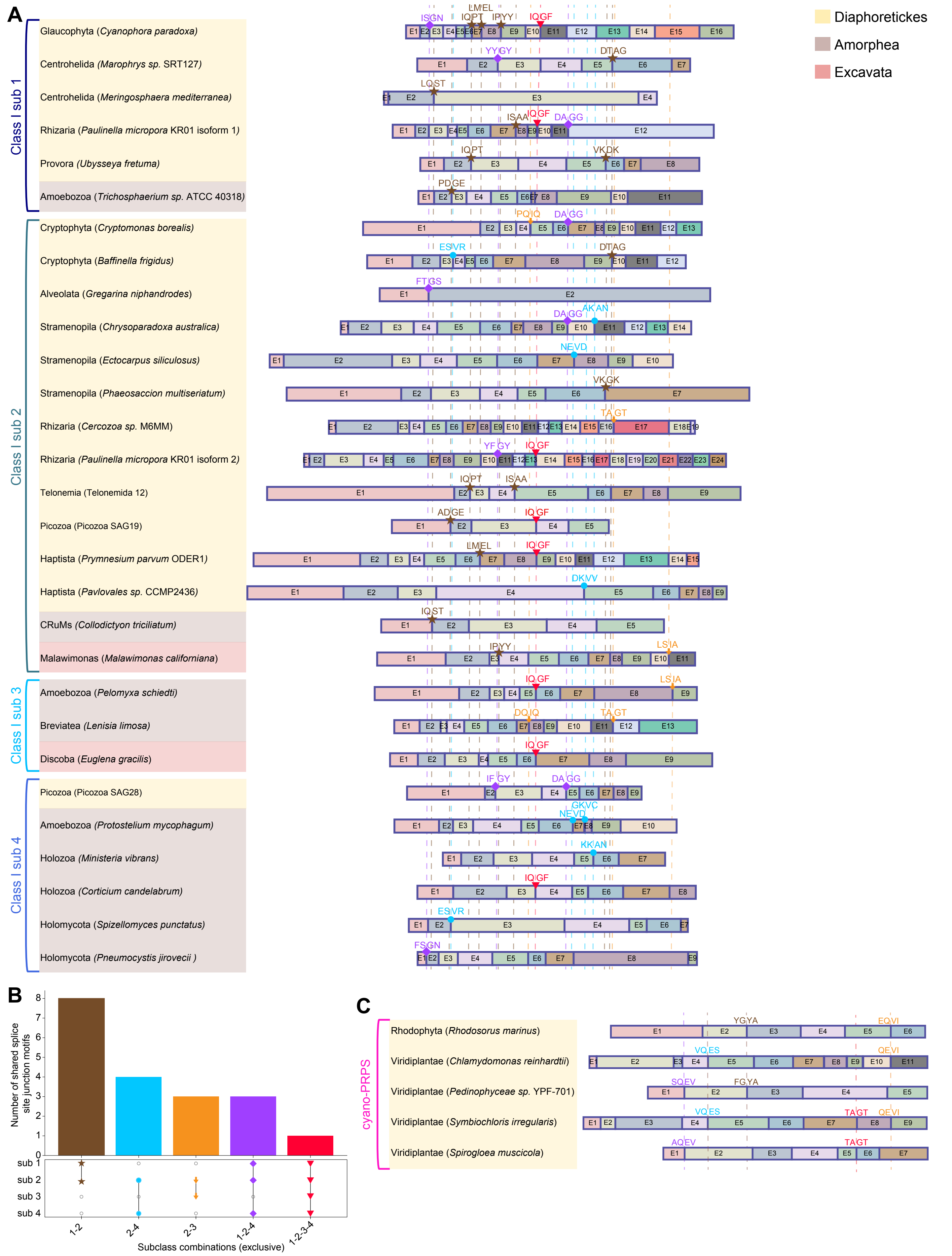

bioRxiv preprint doi: https://doi.org/10.64898/2026.06.01.728777; this version posted June 3, 2026. The copyright holder for this preprint (which was not certified by peer review) is the author/funder, who has granted bioRxiv a license to display the preprint in perpetuity. It is made available under a CC-BY 4.0 International license.

Supplementary Figure 6. Conserved splice junctions reveal shared ancestry of Class I PRPS genes and of cyanobacteria-derived PRPS genes.

(A) Exon organization of Class I PRPS subclasses (subclasses 1-4) across representative eukaryotic lineages, shown as colored boxes with lengths proportional to the number of amino acid residues per exon; introns are not displayed. Sequences are grouped by supergroups – Diaphoretickes, Amorphea, and Excavata. Conserved splice site junctions revealed via multiple sequence alignments of translated sequences are indicated at exon boundaries, with amino acids flanking each junction (two residues on either side) displayed. Symbols denote shared splice site junction categorized by subclass combinations as defined in (B). **(B)** Number of shared splice site junctions across Class I subclasses. Bars represent counts of conserved junctions, with subclass combinations indicated below. **(C)** Exon organization of cyano-PRPS across representative Archaeplastida species. Conserved splice site junctions revealed via multiple sequence alignments of translated sequences are indicated at exon boundaries, with amino acids flanking each junction (two residues on either side) displayed.

bioRxiv preprint doi: https://doi.org/10.64898/2026.06.01.728777; this version posted June 3, 2026. The copyright holder for this preprint (which was not certified by peer review) is the author/funder, who has granted bioRxiv a license to display the preprint in perpetuity. It is made available under a CC-BY 4.0 International license.

Supplementary Fig. 7

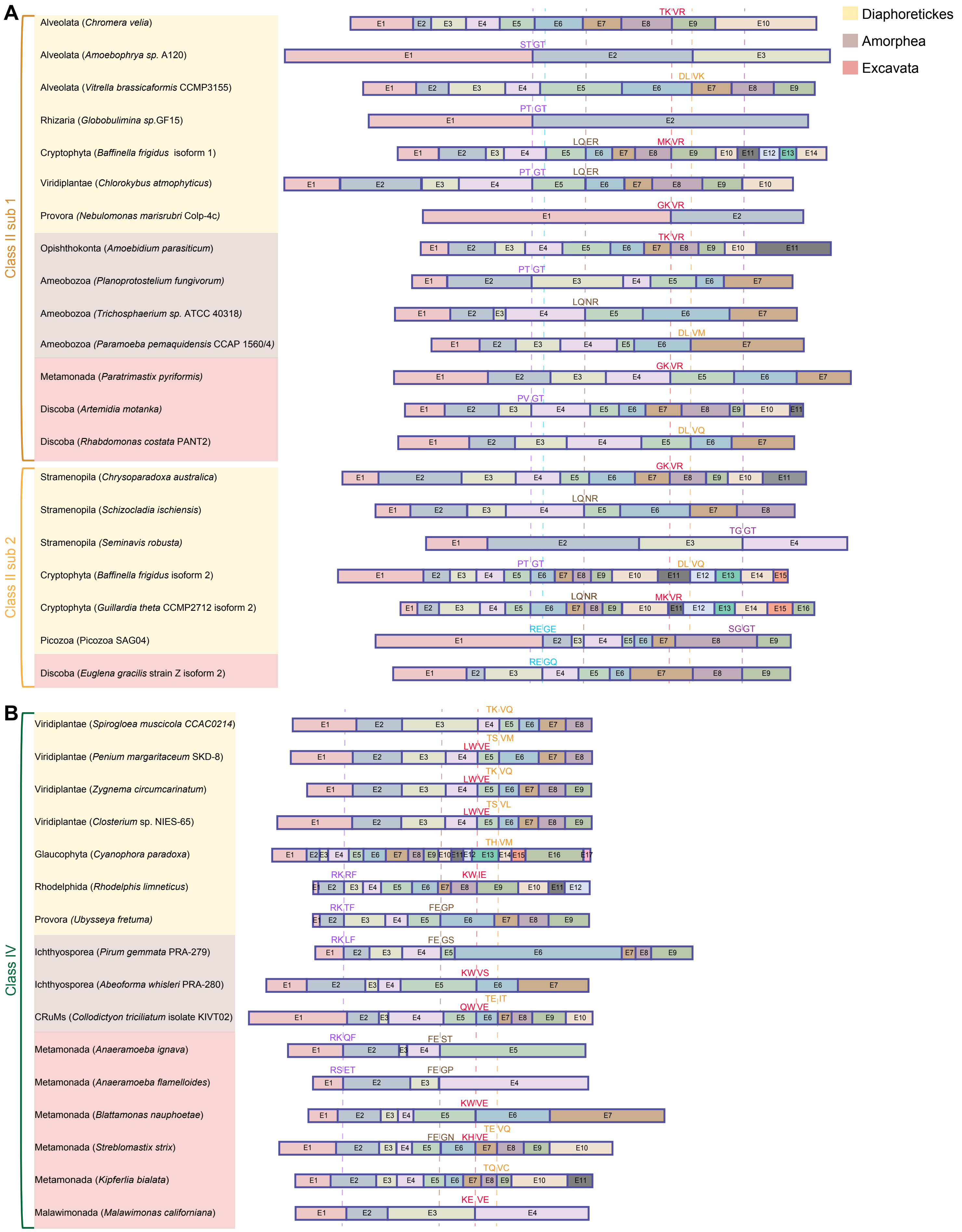

bioRxiv preprint doi: https://doi.org/10.64898/2026.06.01.728777; this version posted June 3, 2026. The copyright holder for this preprint (which was not certified by peer review) is the author/funder, who has granted bioRxiv a license to display the preprint in perpetuity. It is made available under a CC-BY 4.0 International license.

Supplementary Figure 7. Conserved splice junctions reveal shared ancestry of Class II PRPS genes and of Class IV PRPS genes.

(A-B) Exon organization of Class II PRPS subclasses (subclasses 1-2) (A) and Class IV PRPS (B) across representative eukaryotic lineages, shown as colored boxes with lengths proportional to the number of amino acid residues per exon; introns are not displayed. Sequences are grouped by major supergroups (Diaphoretickes, Amorphea, and Excavata). Conserved splice site junctions revealed via multiple sequence alignments of translated sequences are indicated at exon boundaries, with amino acids flanking each junction (two residues on either side) displayed.

bioRxiv preprint doi: https://doi.org/10.64898/2026.06.01.728777; this version posted June 3, 2026. The copyright holder for this preprint (which was not certified by peer review) is the author/funder, who has granted bioRxiv a license to display the preprint in perpetuity. It is made available under a CC-BY 4.0 International license.

Supplementary Fig. 8

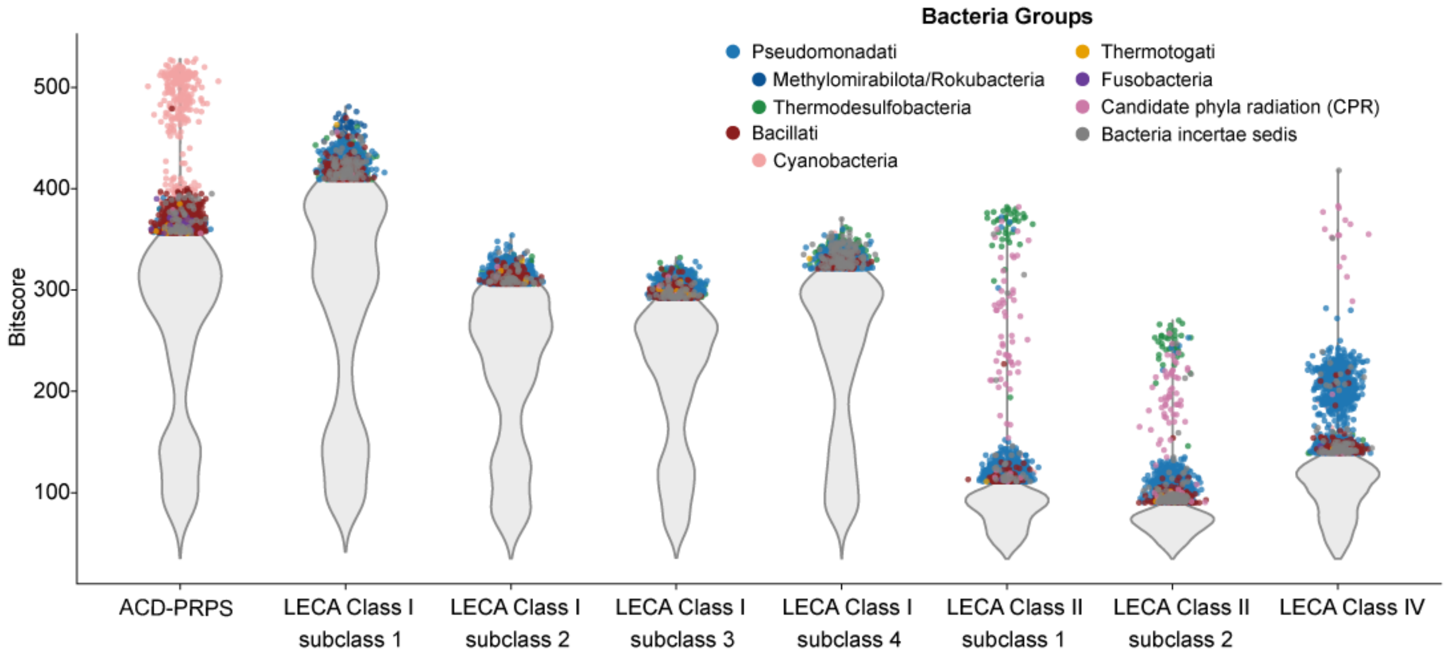

bioRxiv preprint doi: https://doi.org/10.64898/2026.06.01.728777; this version posted June 3, 2026. The copyright holder for this preprint (which was not certified by peer review) is the author/funder, who has granted bioRxiv a license to display the preprint in perpetuity. It is made available under a CC-BY 4.0 International license.

Supplementary Figure 8. BLASTP profiling of ancestral PRPS reconstructions support chimeric bacterial origins of the pre-LECA eukaryotic PRPS repertoire.

Violin plots showing the distribution of BLASTP bit scores between ancestrally reconstructed PRPS queries and a curated database of 33,248 non-redundant prokaryotic PRPS sequences (28,560 bacterial and 4,688 archaeal). Queries include ACD-PRPS, LECA Class I subclasses (subclasses 1-4), LECA Class II subclasses (subclasses 1-2), and LECA Class IV. Ancestral sequence reconstruction (ASR) is described in Methods. For each query, the top 5% of highest-scoring sequences (≥ 95th percentile) are overlaid as individual points and colored by taxonomic classification (Pseudomonadati, Methylomirabilota/Rokubacteria, Bacillati, Cyanobacteria, Thermotogati, Fusobacteria, Candidate Phyla Radiation (CPR), and Bacteria incertae sedis).

bioRxiv preprint doi: https://doi.org/10.64898/2026.06.01.728777; this version posted June 3, 2026. The copyright holder for this preprint (which was not certified by peer review) is the author/funder, who has granted bioRxiv a license to display the preprint in perpetuity. It is made available under a CC-BY 4.0 International license.

Supplementary Fig. 9

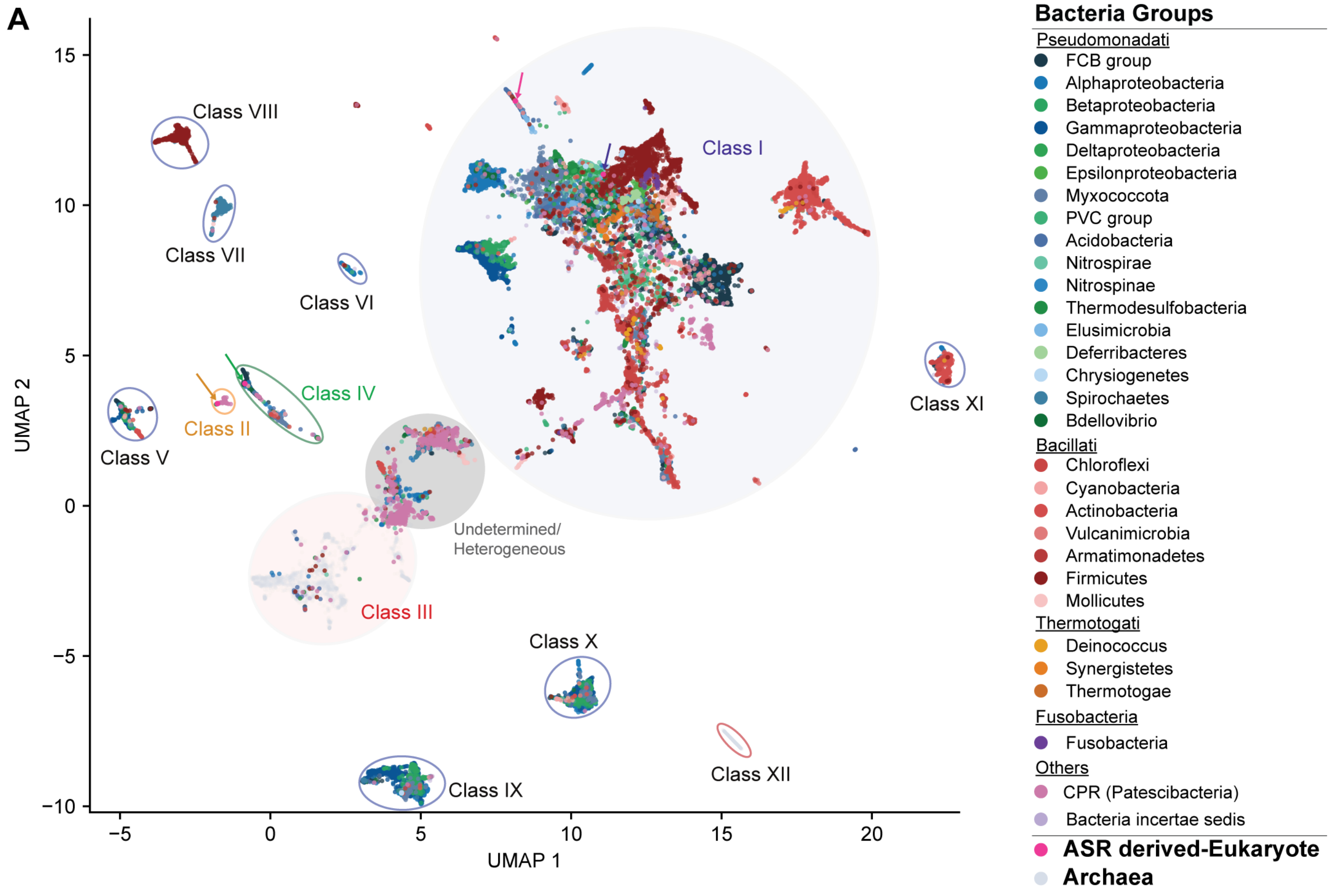

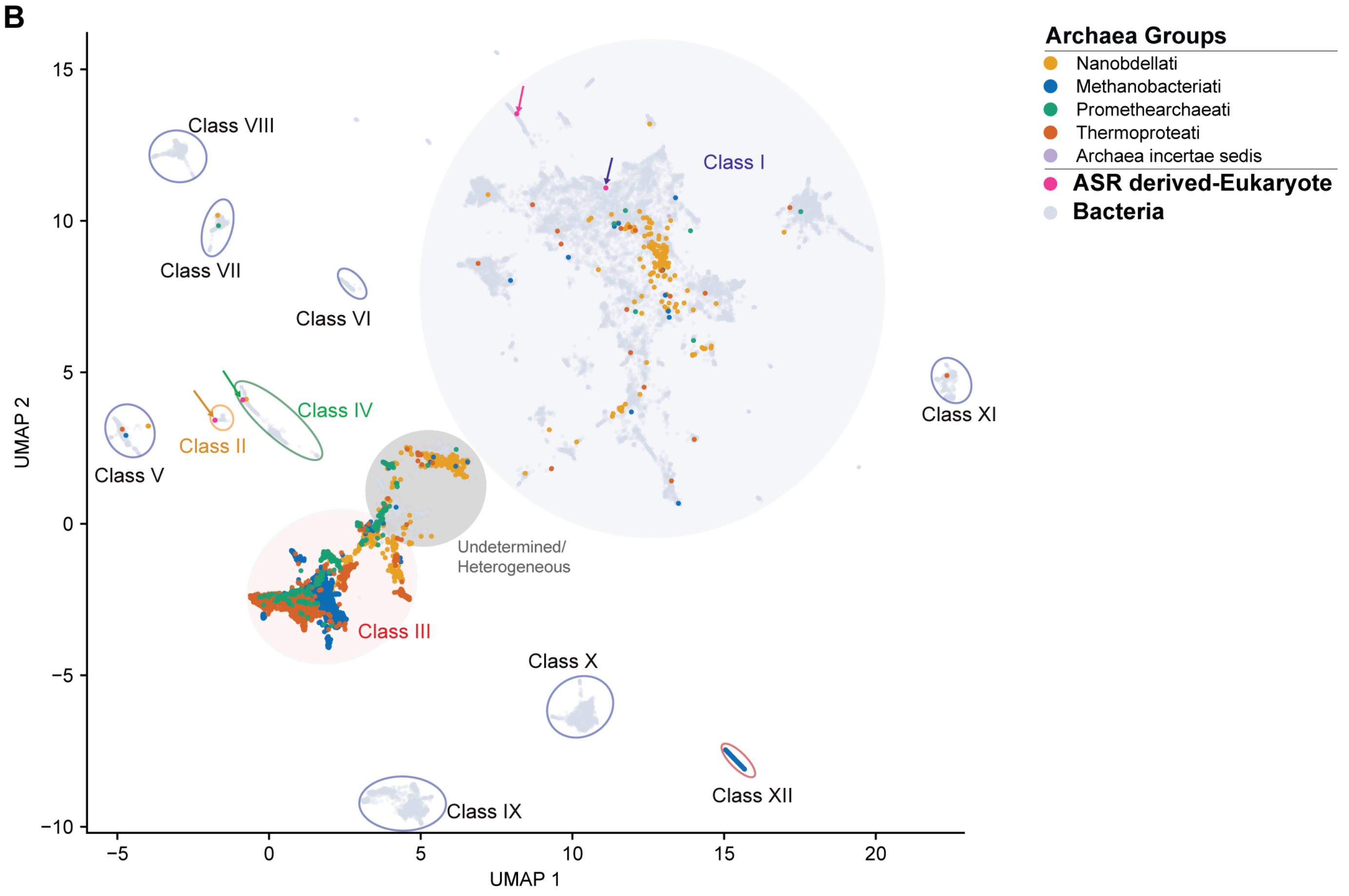

bioRxiv preprint doi: https://doi.org/10.64898/2026.06.01.728777; this version posted June 3, 2026. The copyright holder for this preprint (which was not certified by peer review) is the author/funder, who has granted bioRxiv a license to display the preprint in perpetuity. It is made available under a CC-BY 4.0 International license.

Supplementary Figure 9. Class I and Class III PRPS have near universal distribution in Bacteria and Archaea, respectively, whereas other classes are more sporadically distributed.

(A-B) Two-dimensional UMAP of ESM-2-derived embeddings for prokaryotic PRPS sequences (same embedding as Figure 2A). Sequences are colored by kingdom/phylum-level groupings for bacterial lineage in (A) and archaeal lineage in (B), respectively, indicating the relative distribution of sequences across major bacterial and archaeal groups. Different prokaryotic PRPS classes are indicated and circled. Colored arrows denote ancestrally reconstructed eukaryotic sequences (blue, LECA Class I sub 1; pink, ACD-PRPS; orange, LECA Class II sub 1; green, LECA Class IV).

bioRxiv preprint doi: https://doi.org/10.64898/2026.06.01.728777; this version posted June 3, 2026. The copyright holder for this preprint (which was not certified by peer review) is the author/funder, who has granted bioRxiv a license to display the preprint in perpetuity. It is made available under a CC-BY 4.0 International license.

Supplementary Fig. 10

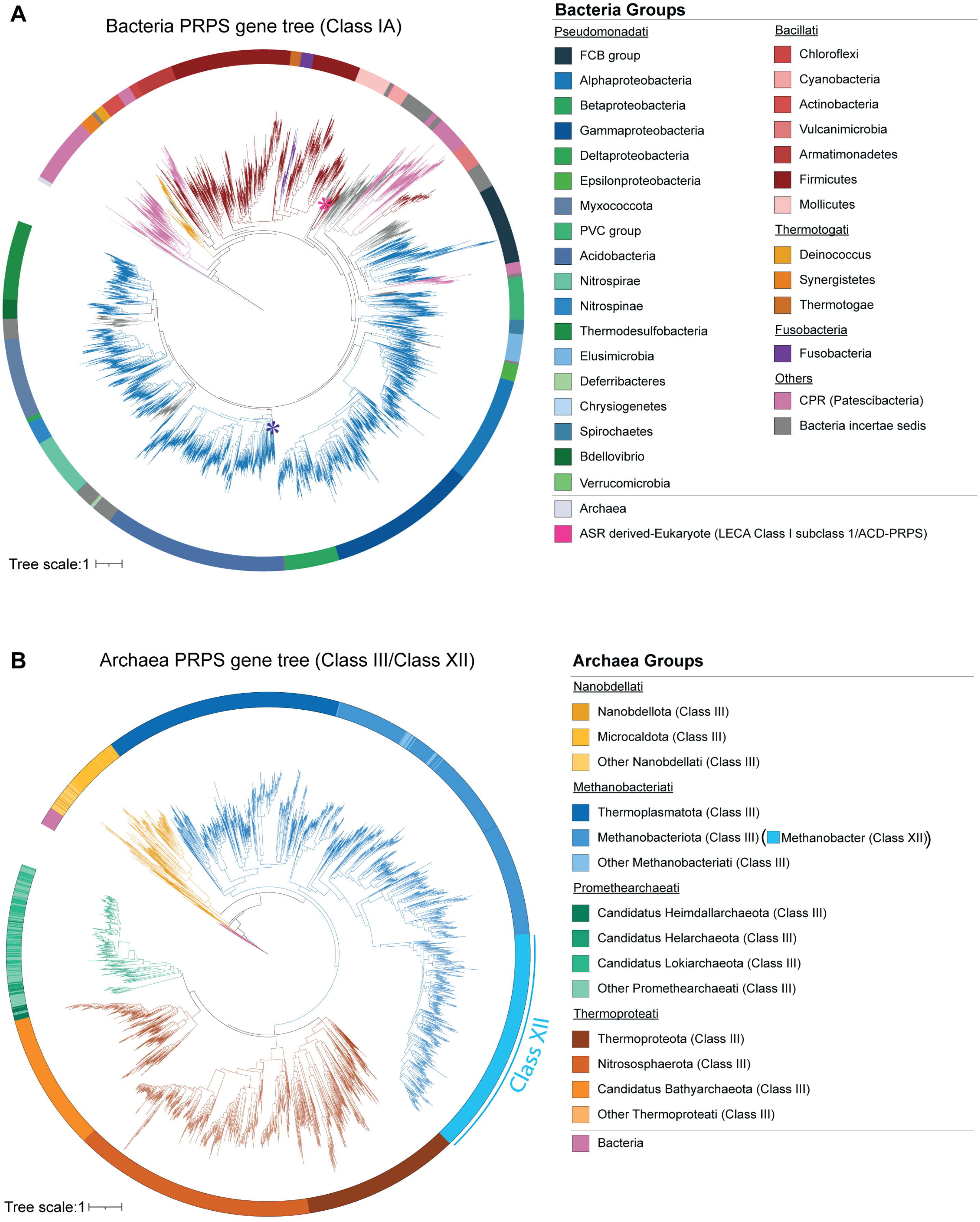

bioRxiv preprint doi: https://doi.org/10.64898/2026.06.01.728777; this version posted June 3, 2026. The copyright holder for this preprint (which was not certified by peer review) is the author/funder, who has granted bioRxiv a license to display the preprint in perpetuity. It is made available under a CC-BY 4.0 International license.

Supplementary Figure 10. Phylogenetics indicate vertical inheritance of the most prevalent bacterial (Class I) and archaeal (Class III) PRPS classes.

(A) Circular maximum likelihood gene tree of bacterial Class I PRPS sequences, inferred using IQ-TREE under the LG+F+R10 model. Branches are colored by major bacterial lineages, and the outer circular color strip denotes kingdom/phylum-level classification. Archaeal sequences are included as an outgroup. The most prevalent Class I PRPS sequences that follow a pattern of vertical inheritance within bacteria are hereafter referred to as Class IA. **(B)** Circular maximum likelihood gene tree of archaeal PRPS sequences, including Class III and Class XII classes, inferred using IQ-TREE under the LG+F+R10 model. Branches are colored by major archaeal lineages, and the outer circular color strip denotes kingdom/phylum-level classification. Class XII sequences are labeled in the circular color strip. Bacterial Class IA sequences are included as an outgroup. The full tree for (A) and (B) is provided in rectangular format with bootstrap support values in Supplementary File 2 and 3, respectively, and in Newick format in the Figshare repository.

bioRxiv preprint doi: https://doi.org/10.64898/2026.06.01.728777; this version posted June 3, 2026. The copyright holder for this preprint (which was not certified by peer review) is the author/funder, who has granted bioRxiv a license to display the preprint in perpetuity. It is made available under a CC-BY 4.0 International license.

Supplementary Fig. 11

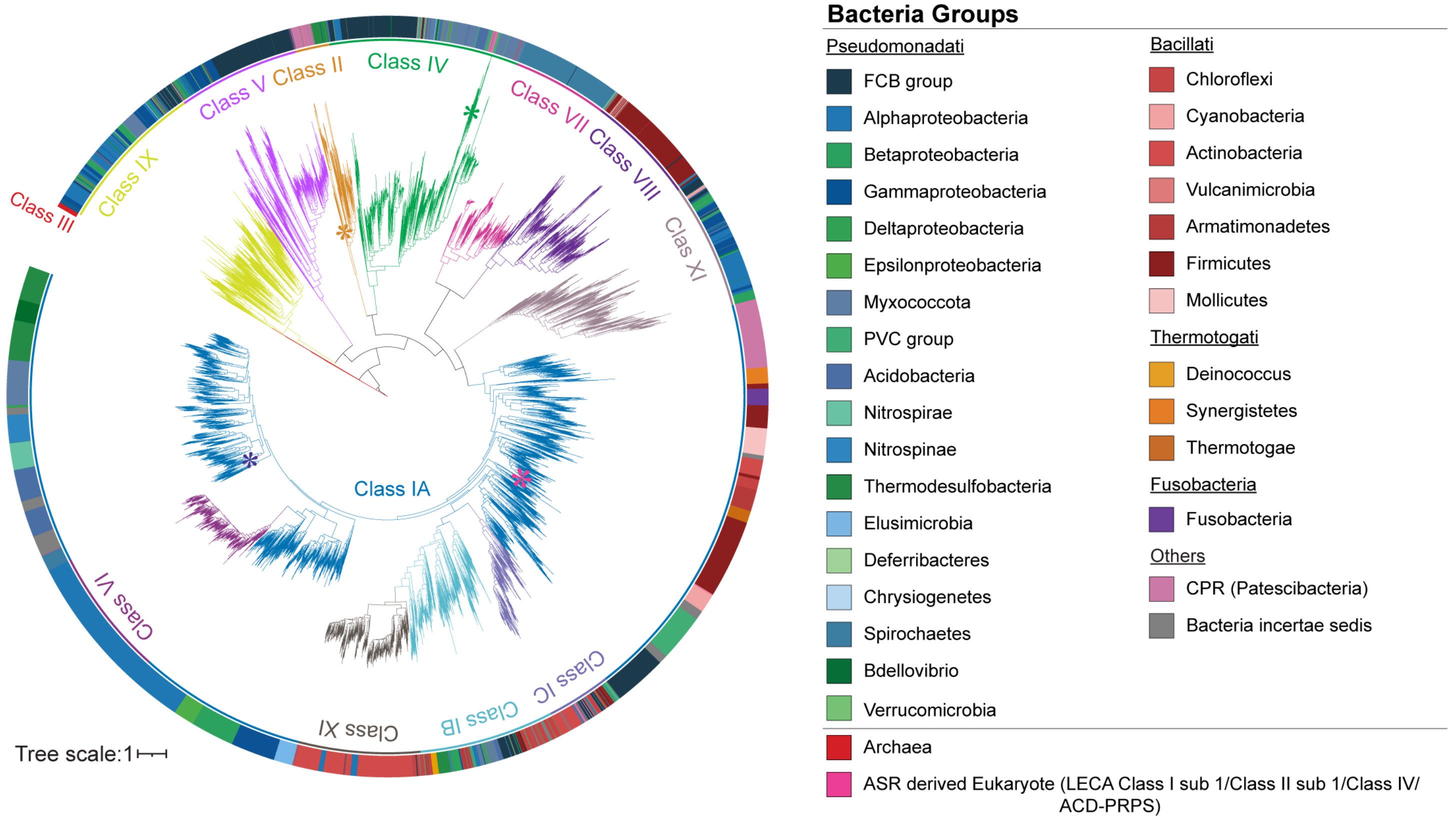

bioRxiv preprint doi: https://doi.org/10.64898/2026.06.01.728777; this version posted June 3, 2026. The copyright holder for this preprint (which was not certified by peer review) is the author/funder, who has granted bioRxiv a license to display the preprint in perpetuity. It is made available under a CC-BY 4.0 International license.

Supplementary Figure 11. Expanded gene tree reveals relationships among prokaryotic PRPS classes.

Circular maximum likelihood gene tree of diverse bacterial (Class I (A, B, C), II, IV-XI) and archaeal (Class III) PRPS sequences inferred using IQ-TREE under the LG+F+R10 model (same as Figure 2C). Branches are colored based on PRPS classes as indicated. Outer circular color strip denotes kingdom/phylum-level classification. Asterisks mark positions of ASR-derived ancestral sequences (blue, LECA Class I sub 1; pink, ACD-PRPS; green, LECA Class IV; orange, LECA Class II sub 1). The full tree is provided in rectangular format with bootstrap support values in Supplementary File 5, and in Newick format in the Figshare repository.

bioRxiv preprint doi: https://doi.org/10.64898/2026.06.01.728777; this version posted June 3, 2026. The copyright holder for this preprint (which was not certified by peer review) is the author/funder, who has granted bioRxiv a license to display the preprint in perpetuity. It is made available under a CC-BY 4.0 International license.

Supplementary Fig. 12

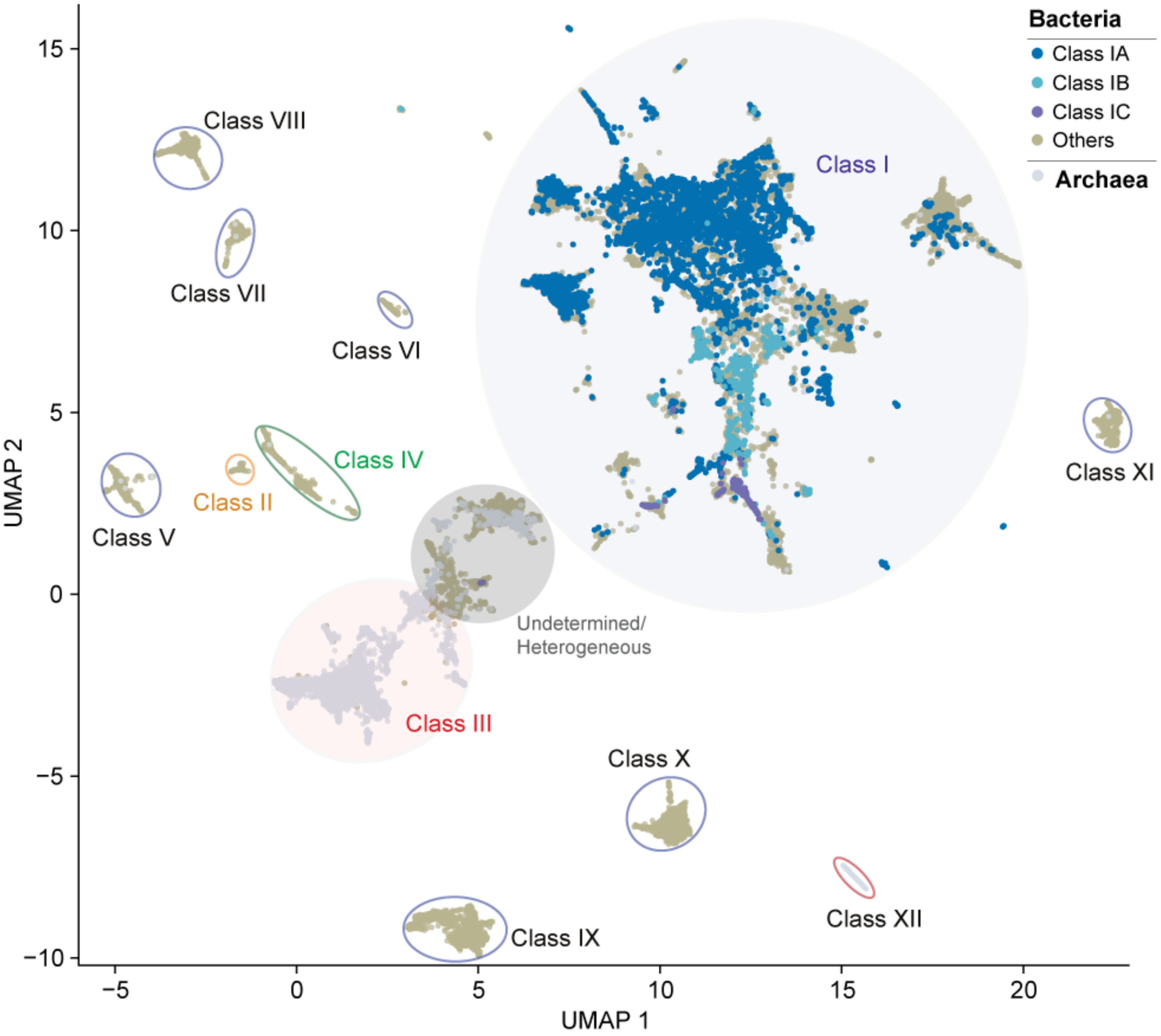

bioRxiv preprint doi: https://doi.org/10.64898/2026.06.01.728777; this version posted June 3, 2026. The copyright holder for this preprint (which was not certified by peer review) is the author/funder, who has granted bioRxiv a license to display the preprint in perpetuity. It is made available under a CC-BY 4.0 International license.

Supplementary Figure 12. Bacterial Class I subclasses occupy distinct regions within the broader Class I embedding landscape.

Two-dimensional UMAP of ESM-2-derived embeddings for prokaryotic PRPS sequences (same embedding as Figure 2A) highlighting bacterial Class I subclass – Class IA (n = 5,224), IB (n = 699), and IC (n = 174). Different prokaryotic PRPS classes are indicated and circled.

bioRxiv preprint doi: https://doi.org/10.64898/2026.06.01.728777; this version posted June 3, 2026. The copyright holder for this preprint (which was not certified by peer review) is the author/funder, who has granted bioRxiv a license to display the preprint in perpetuity. It is made available under a CC-BY 4.0 International license.

Supplementary Fig. 13

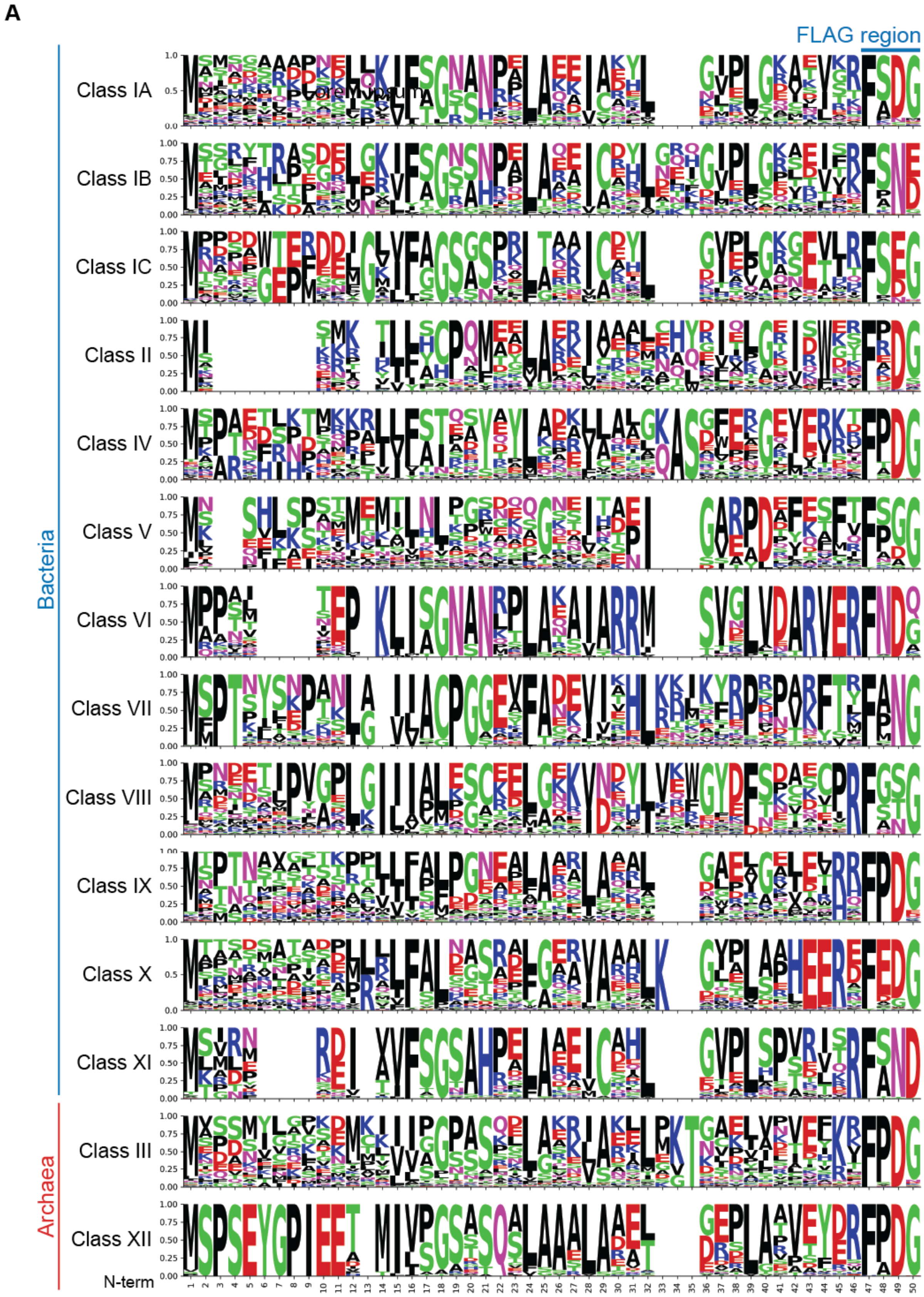

bioRxiv preprint doi: https://doi.org/10.64898/2026.06.01.728777; this version posted June 3, 2026. The copyright holder for this preprint (which was not certified by peer review) is the author/funder, who has granted bioRxiv a license to display the preprint in perpetuity. It is made available under a CC-BY 4.0 International license.

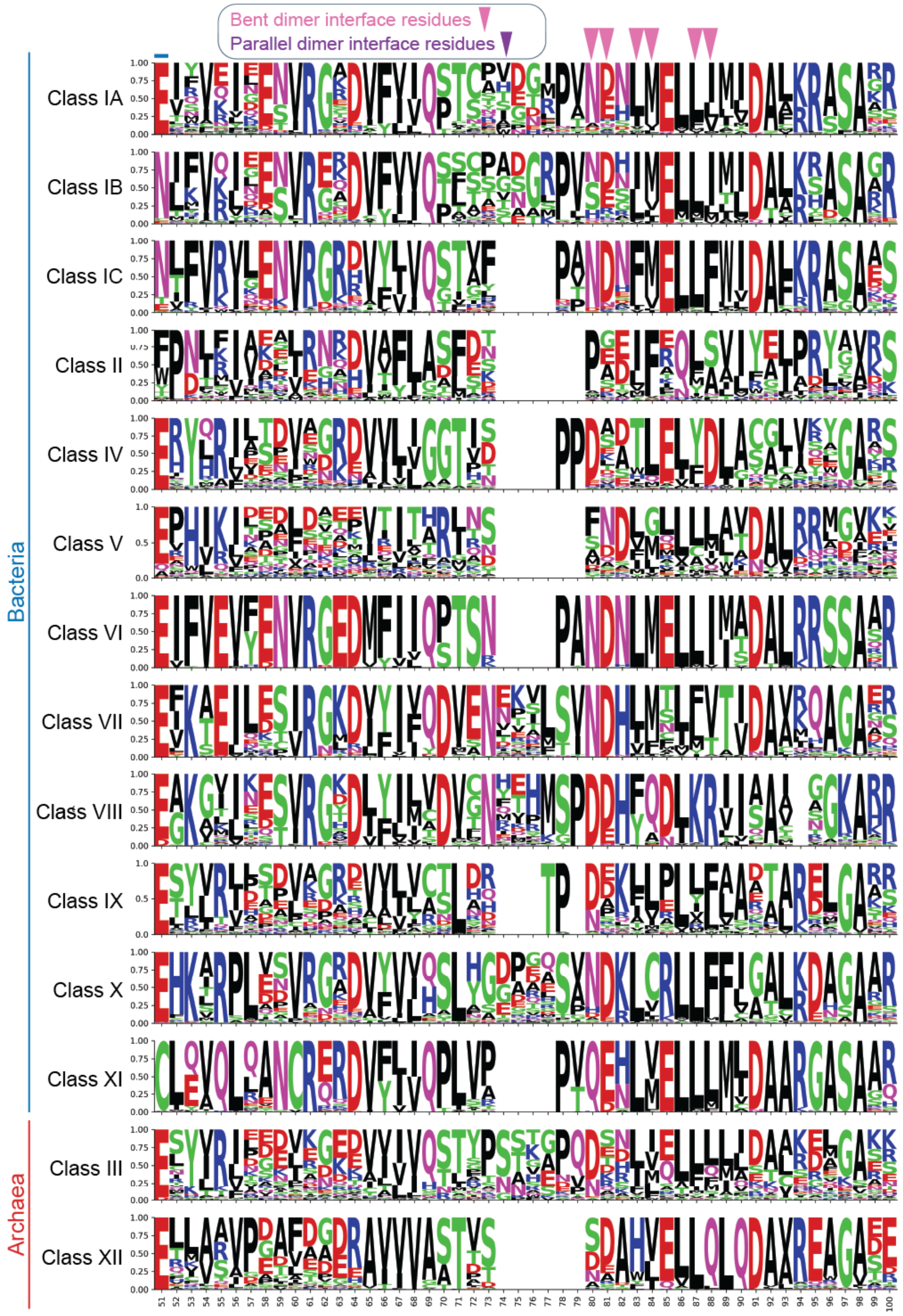

bioRxiv preprint doi: https://doi.org/10.64898/2026.06.01.728777; this version posted June 3, 2026. The copyright holder for this preprint (which was not certified by peer review) is the author/funder, who has granted bioRxiv a license to display the preprint in perpetuity. It is made available under a CC-BY 4.0 International license.

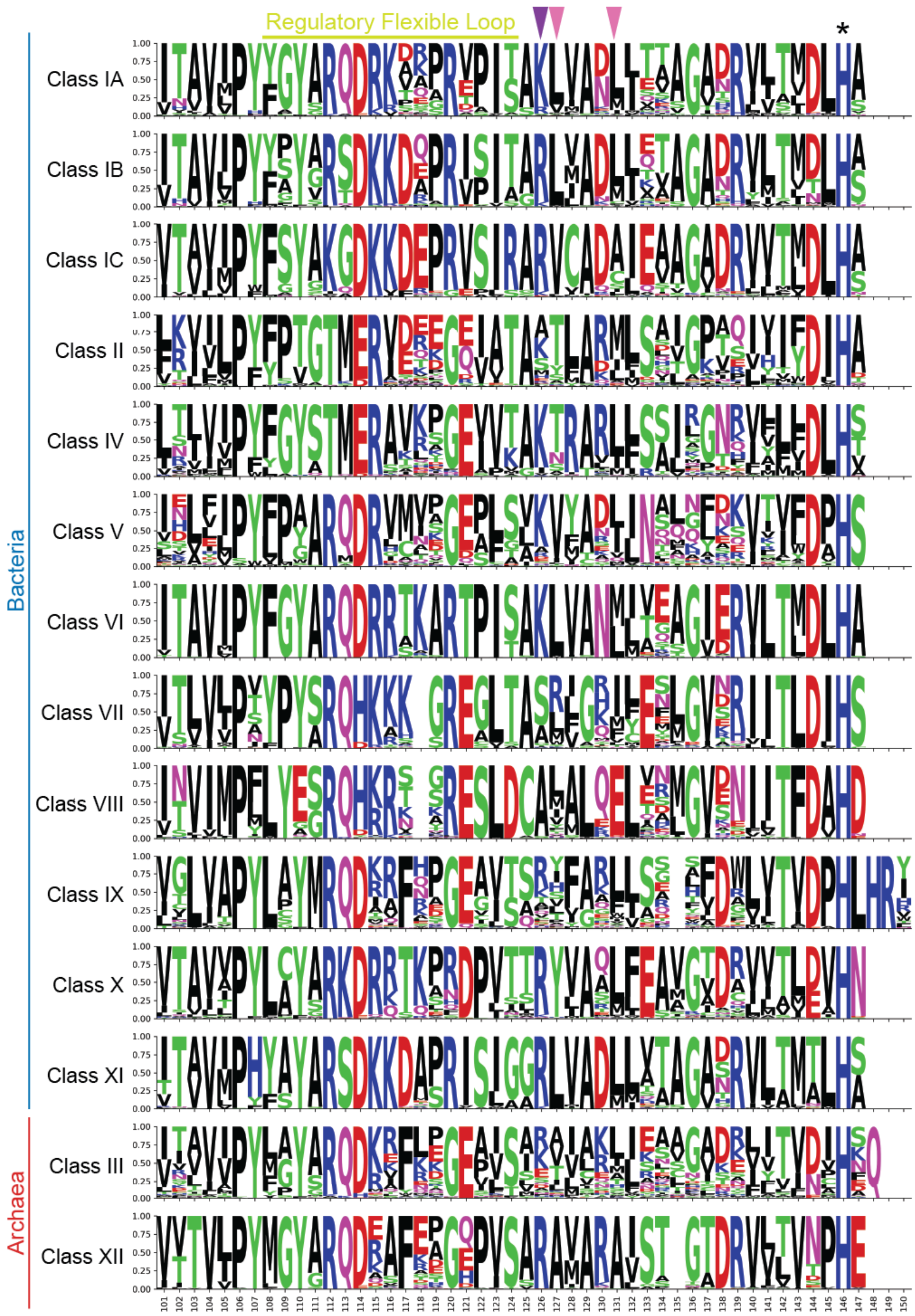

bioRxiv preprint doi: https://doi.org/10.64898/2026.06.01.728777; this version posted June 3, 2026. The copyright holder for this preprint (which was not certified by peer review) is the author/funder, who has granted bioRxiv a license to display the preprint in perpetuity. It is made available under a CC-BY 4.0 International license.

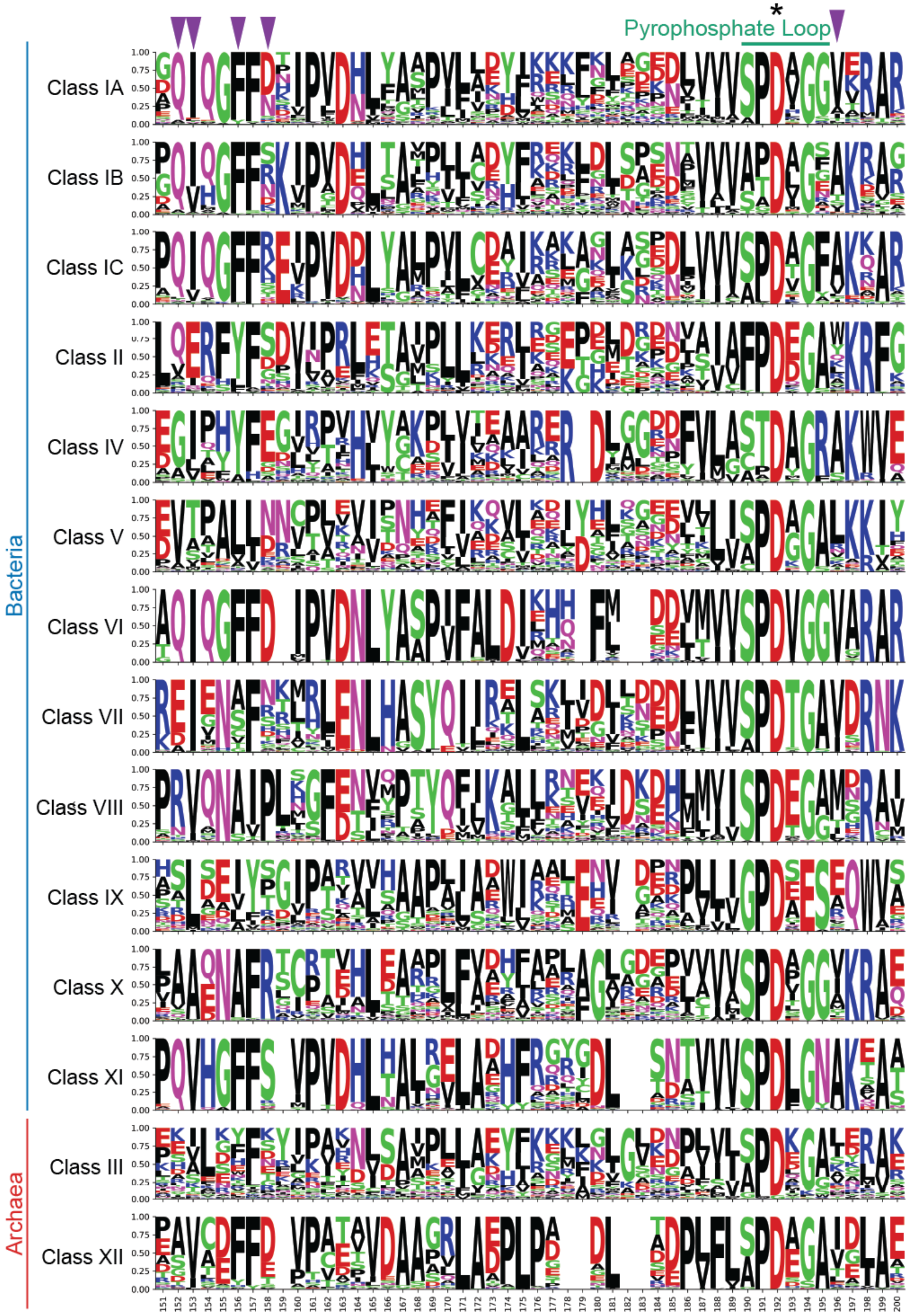

bioRxiv preprint doi: https://doi.org/10.64898/2026.06.01.728777; this version posted June 3, 2026. The copyright holder for this preprint (which was not certified by peer review) is the author/funder, who has granted bioRxiv a license to display the preprint in perpetuity. It is made available under a CC-BY 4.0 International license.

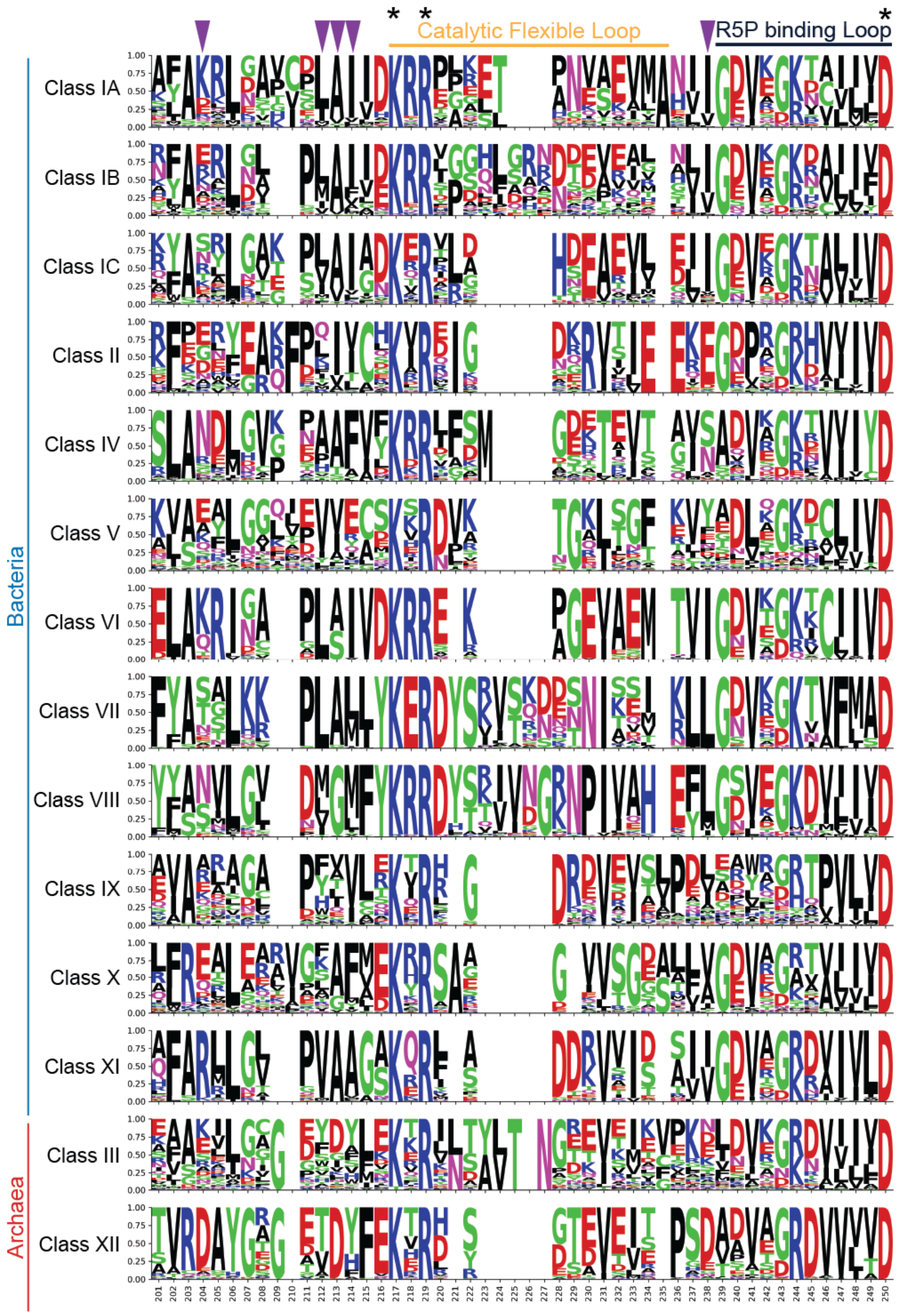

bioRxiv preprint doi: https://doi.org/10.64898/2026.06.01.728777; this version posted June 3, 2026. The copyright holder for this preprint (which was not certified by peer review) is the author/funder, who has granted bioRxiv a license to display the preprint in perpetuity. It is made available under a CC-BY 4.0 International license.

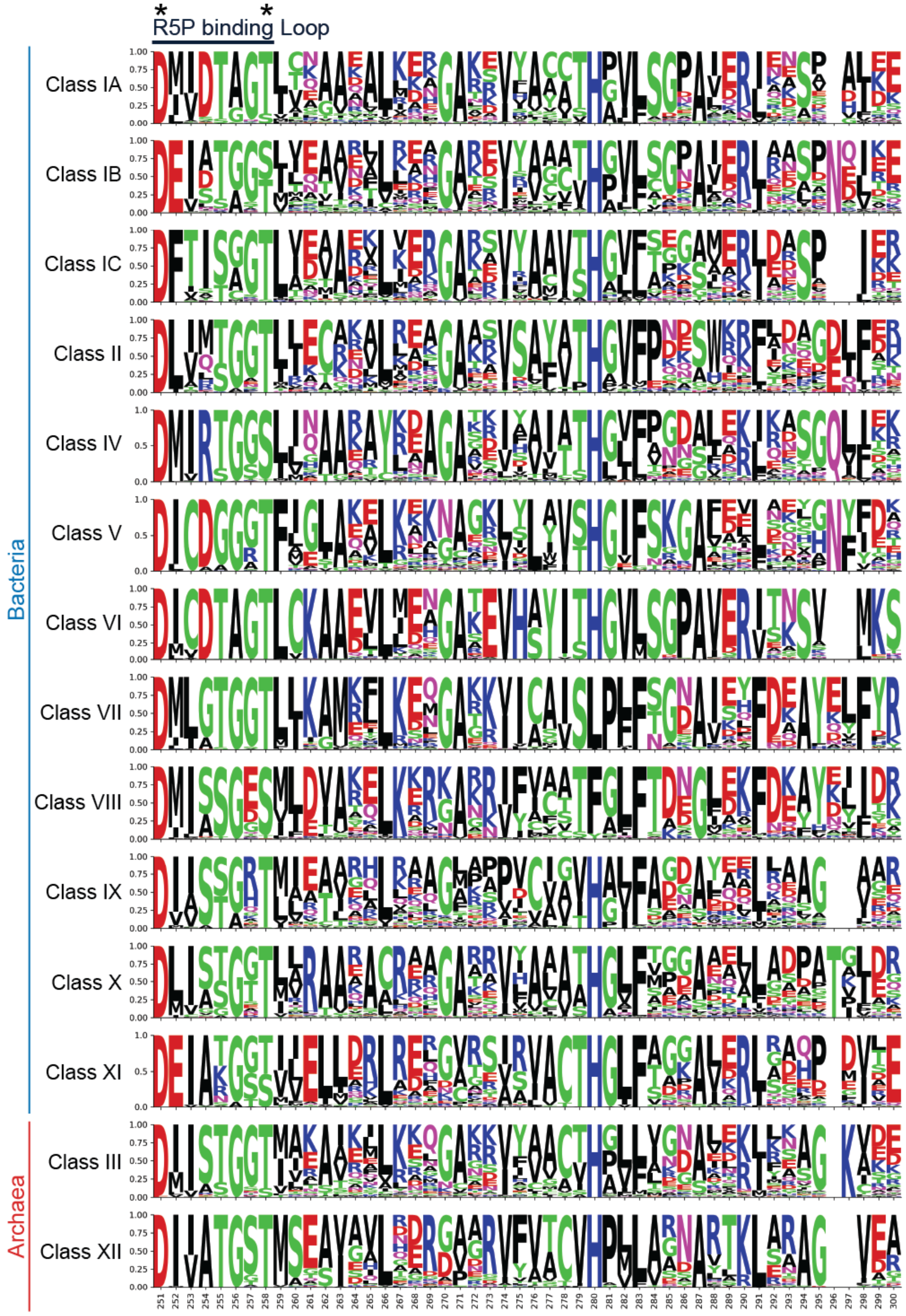

bioRxiv preprint doi: https://doi.org/10.64898/2026.06.01.728777; this version posted June 3, 2026. The copyright holder for this preprint (which was not certified by peer review) is the author/funder, who has granted bioRxiv a license to display the preprint in perpetuity. It is made available under a CC-BY 4.0 International license.

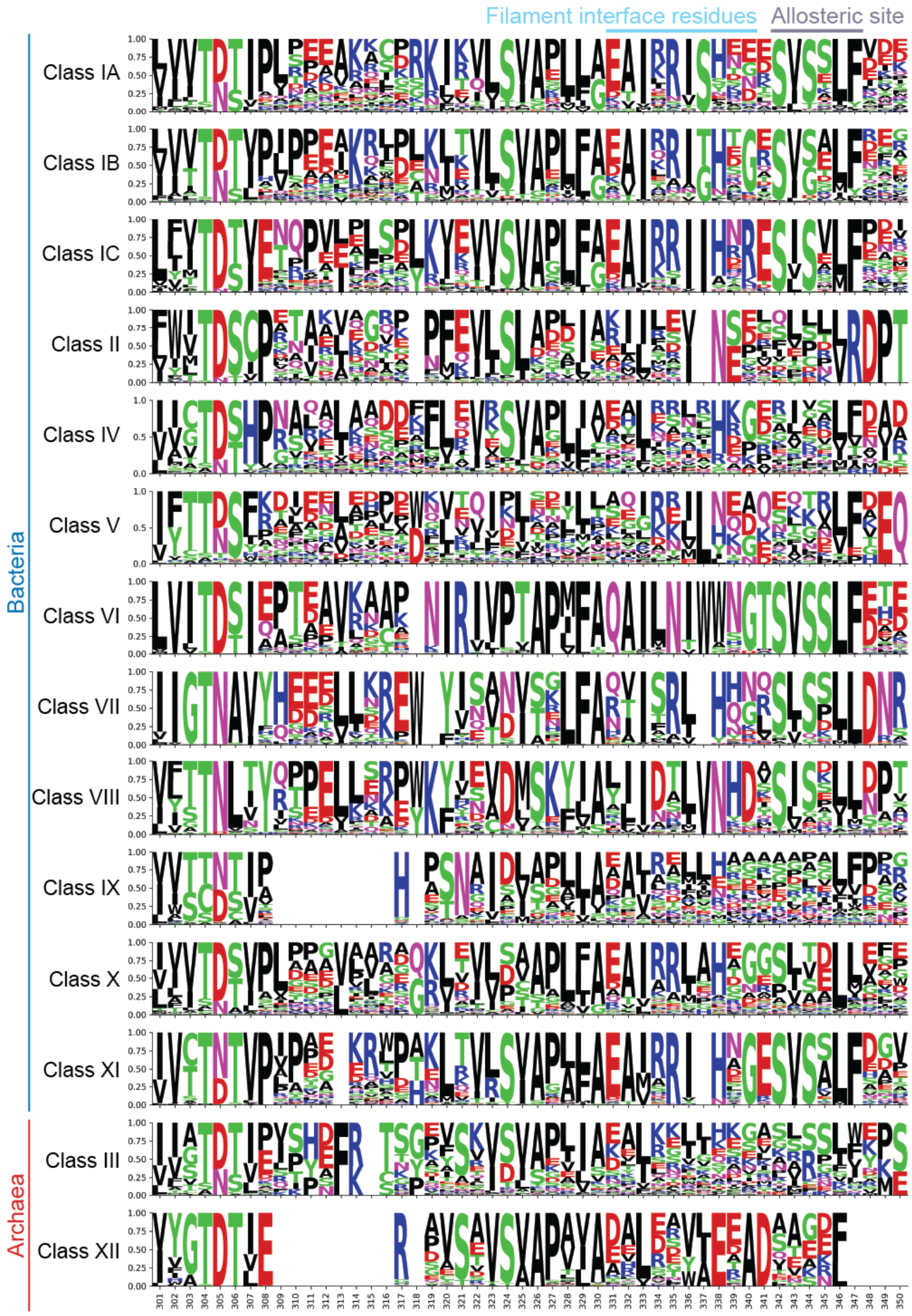

bioRxiv preprint doi: https://doi.org/10.64898/2026.06.01.728777; this version posted June 3, 2026. The copyright holder for this preprint (which was not certified by peer review) is the author/funder, who has granted bioRxiv a license to display the preprint in perpetuity. It is made available under a CC-BY 4.0 International license.

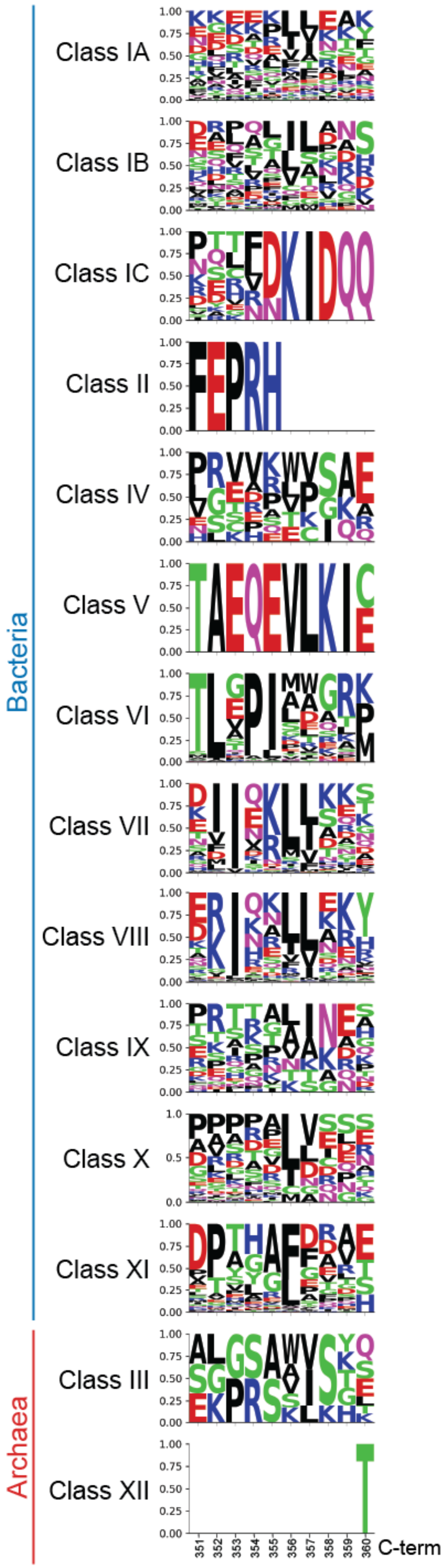

bioRxiv preprint doi: https://doi.org/10.64898/2026.06.01.728777; this version posted June 3, 2026. The copyright holder for this preprint (which was not certified by peer review) is the author/funder, who has granted bioRxiv a license to display the preprint in perpetuity. It is made available under a CC-BY 4.0 International license.

Supplementary Figure 13. Global sequence conservation and divergence across prokaryotic PRPS classes

WebLogo depicting full-length multiple sequence alignment for prokaryotic PRPS sequences. Logos represent amino acid frequencies at each alignment position, calculated independently for each group using non-gap residues. The height of each letter reflects its relative frequency at that position. Asterisks denote universally conserved residues critical for enzyme function. Group sizes for bacterial PRPS classes: Class IA (n = 3251), Class IB (n = 732), Class IC (n = 224), Class II (n = 109), Class IV (n = 763), Class V (n = 511), Class VI (n = 459), Class VII (n = 445), Class VIII (n = 726), Class IX (n = 1368), Class X (n = 929), Class XI (n = 699). Group sizes for archaeal PRPS classes: Class III (n = 1156), Class XII (n = 410).

bioRxiv preprint doi: https://doi.org/10.64898/2026.06.01.728777; this version posted June 3, 2026. The copyright holder for this preprint (which was not certified by peer review) is the author/funder, who has granted bioRxiv a license to display the preprint in perpetuity. It is made available under a CC-BY 4.0 International license.

Supplementary Fig. 14

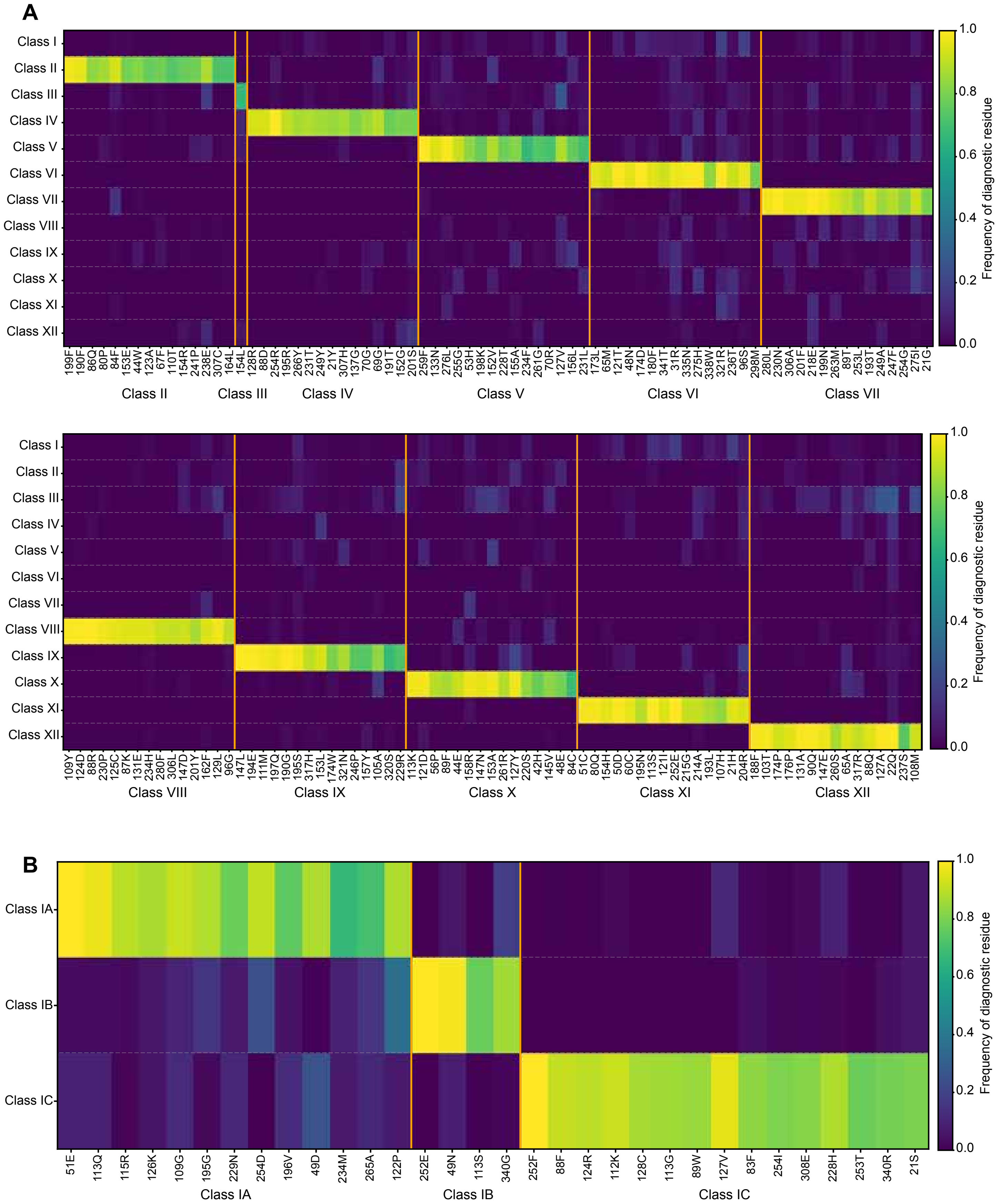

bioRxiv preprint doi: https://doi.org/10.64898/2026.06.01.728777; this version posted June 3, 2026. The copyright holder for this preprint (which was not certified by peer review) is the author/funder, who has granted bioRxiv a license to display the preprint in perpetuity. It is made available under a CC-BY 4.0 International license.

Supplementary Figure 14. Diagnostic residue signatures define distinct PRPS classes across prokaryotes

(A-B) Heatmaps showing diagnostic residues distinguishing PRPS classes across Bacteria and Archaea (A) and across bacterial Class I subclasses (B). Rows correspond to PRPS classes and columns represent alignment positions meeting stringent diagnostic criteria (frequency, coverage, and statistical criteria – see Methods). Cells indicate residue frequency (0-1) within each group, calculated from non-gap residues at each position. Only the top diagnostic sites per class are shown. Diagnostic site positions for each class are referenced to amino acid position shown in Supplementary Figure 13. Group sizes for (A): Class I (n = 4171), Class II (n = 109), Class III (n = 1156), Class IV (n = 763), Class V (n = 511), Class VI (n = 459), Class VII (n = 445), Class VIII (n = 726), Class IX (n = 1368), Class X (n = 929), Class XI (n = 699), Class XII (n = 410). Group sizes for (B): Class IA (n = 3251), Class IB (n = 732), Class IC (n = 224).

bioRxiv preprint doi: https://doi.org/10.64898/2026.06.01.728777; this version posted June 3, 2026. The copyright holder for this preprint (which was not certified by peer review) is the author/funder, who has granted bioRxiv a license to display the preprint in perpetuity. It is made available under a CC-BY 4.0 International license.

Supplementary Fig. 15

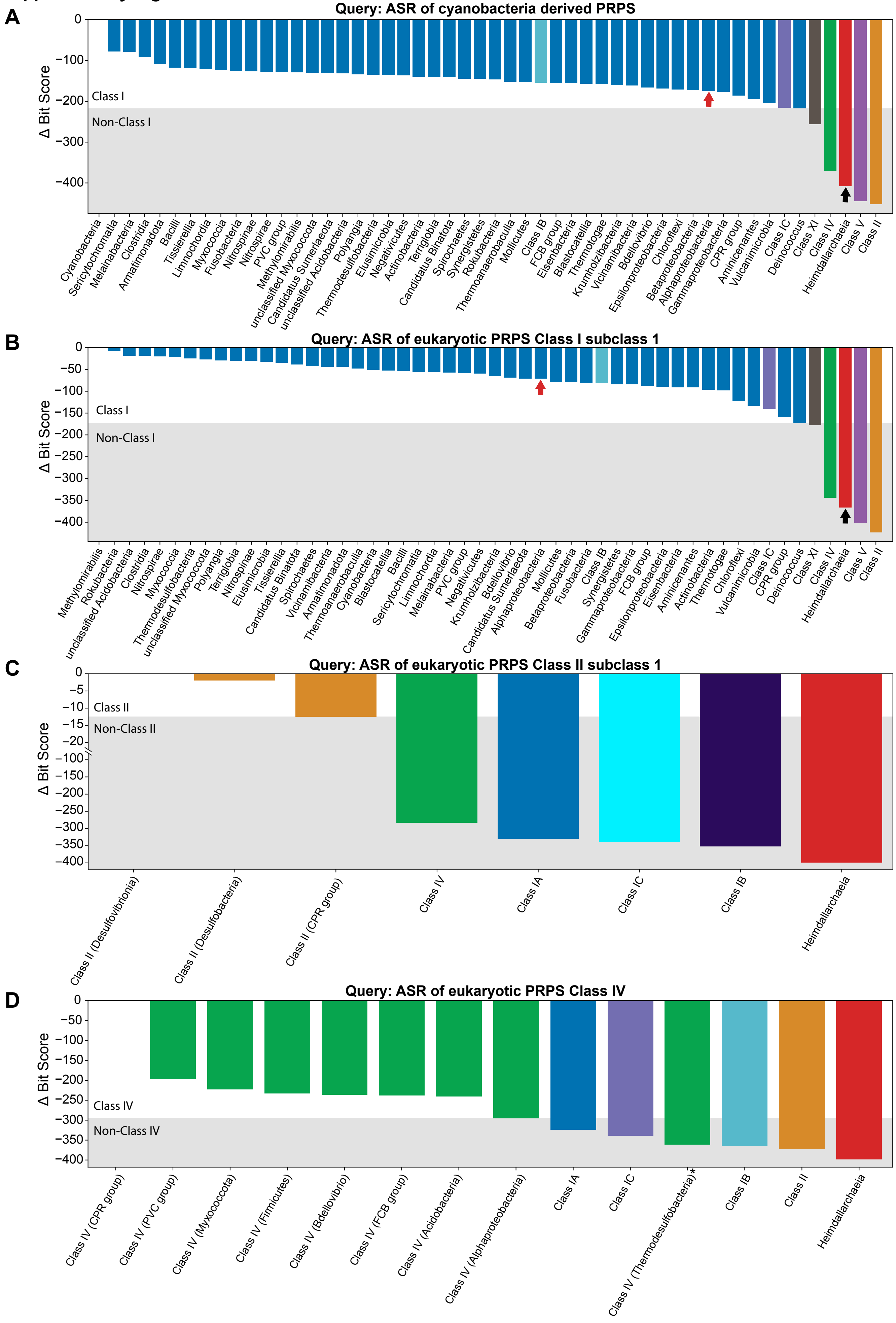

bioRxiv preprint doi: https://doi.org/10.64898/2026.06.01.728777; this version posted June 3, 2026. The copyright holder for this preprint (which was not certified by peer review) is the author/funder, who has granted bioRxiv a license to display the preprint in perpetuity. It is made available under a CC-BY 4.0 International license.

Supplementary Figure 15. Ancestrally reconstructed eukaryotic PRPS sequences nominate distinct bacterial lineages as HGT donors via HMM profiling.

(A-D) Waterfall plots showing Δ bit score profiles for ancestrally reconstructed ACD-PRPS (A), LECA Class I subclass 1 (B), LECA Class II subclass 1 (C), and LECA Class IV (D) sequences against a panel of lineage-specific profile Hidden Markov Models (HMMs). For each query, scores were normalized to the top-scoring model ($\Delta = 0$), and bars are ordered by decreasing bit score. The shaded region denotes non-Class I PRPS. In (A) and (B), ACD-PRPS and LECA Class I show highest-scoring matches to profile HMMs of Cyanobacteria and Methylomirabilota respectively. Alphaproteobacterial HMM (red arrows) and archaeal HMM (black arrows) are not among the top-scoring profiles suggesting that they are unlikely to be the donors to stem eukaryote. In (C) and (D), LECA Class II and LECA Class IV show highest-scoring matches to profile HMMs of Thermodesulfobacteria and Candidate Phyla Radiation (CPR), respectively. Archaea ranks lowest among the profiles compared. Black asterisk denotes a divergent Class IV sequence in Thermodesulfobacteria. Ancestral sequence reconstruction (ASR) is described in Methods.

bioRxiv preprint doi: https://doi.org/10.64898/2026.06.01.728777; this version posted June 3, 2026. The copyright holder for this preprint (which was not certified by peer review) is the author/funder, who has granted bioRxiv a license to display the preprint in perpetuity. It is made available under a CC-BY 4.0 International license.

Supplementary Fig. 16

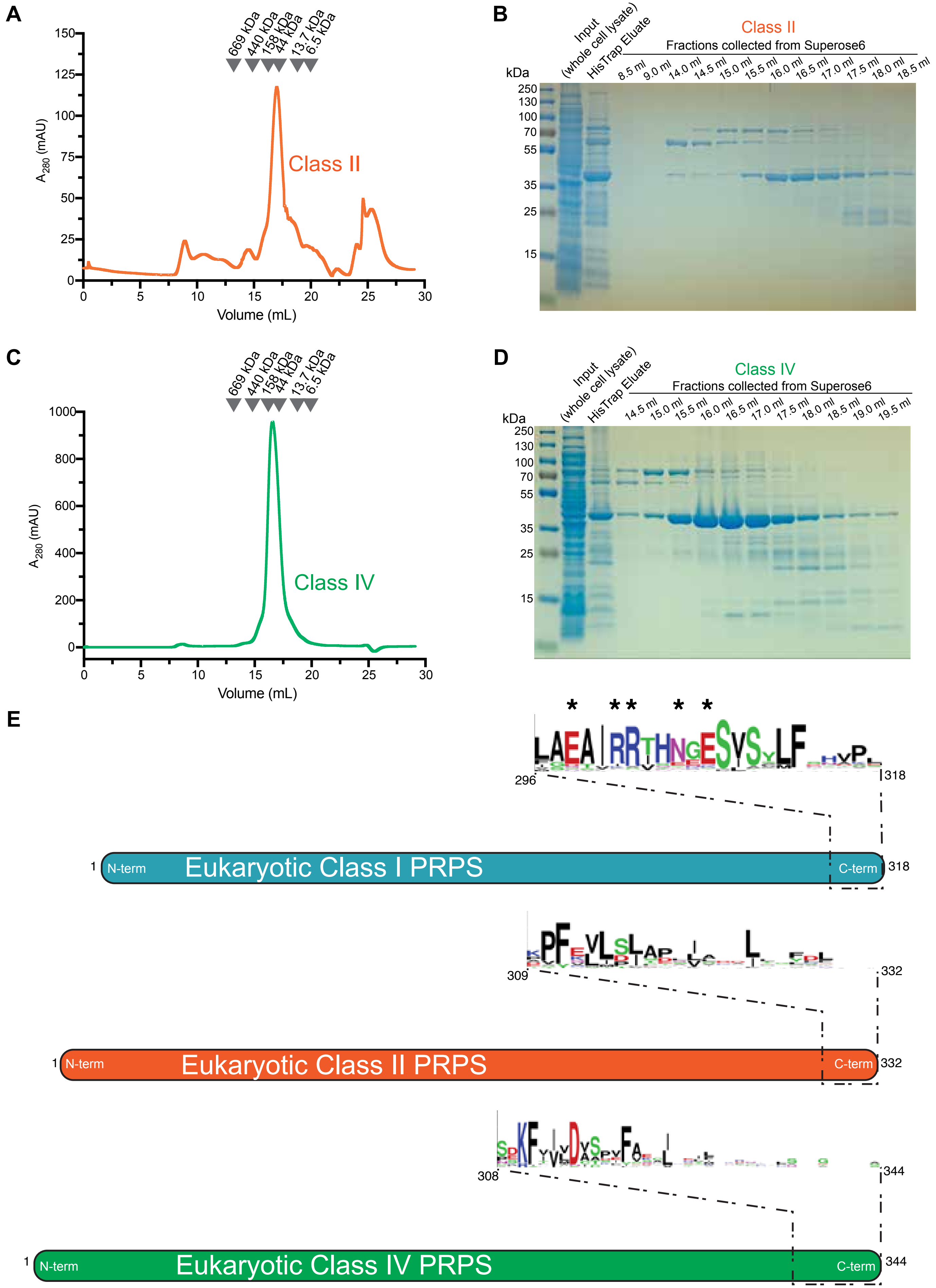

bioRxiv preprint doi: https://doi.org/10.64898/2026.06.01.728777; this version posted June 3, 2026. The copyright holder for this preprint (which was not certified by peer review) is the author/funder, who has granted bioRxiv a license to display the preprint in perpetuity. It is made available under a CC-BY 4.0 International license.

Supplementary Figure 16. Purification and determination of oligomeric state of recombinant Class II and Class IV PRPS.

(A) Size-exclusion chromatography (SEC) elution profile of recombinant protein purified from bacteria for *Branchiostoma lanceolatum* Class II PRPS on a Superose 6 Increase 10/300 GL column; molecular weight standards are shown. **(B)** SDS-PAGE followed by Coomassie staining of fractions collected from the SEC run in (A), showing enrichment and purity of Class II PRPS in peak fractions. **(C)** SEC elution profile of recombinant protein purified from bacteria for *Corallochytrium limacisporum* Class IV PRPS on a Superose 6 Increase 10/300 GL column; molecular weight standards are shown. **(D)** SDS- PAGE followed by Coomassie staining of fractions collected from the SEC run in (C), showing enrichment and purity of Class IV PRPS in peak fractions. **(E)** WebLogo representations of conserved residues at the C-terminal region of eukaryotic PRPS, including Class I (n = 408), Class IV (n = 76), and Class II (n = 475). Residue positions are referenced to representative sequences shown in each schematic. Asterisks denote residues implicated in hexamer stacking at the filament interface for Class I PRPS[64]. These residues are conserved in Class I PRPS but are not conserved or are absent in Class IV and Class II PRPS, consistent with loss of filament-forming capability in these classes.

bioRxiv preprint doi: https://doi.org/10.64898/2026.06.01.728777; this version posted June 3, 2026. The copyright holder for this preprint (which was not certified by peer review) is the author/funder, who has granted bioRxiv a license to display the preprint in perpetuity. It is made available under a CC-BY 4.0 International license.

Supplementary Fig. 17

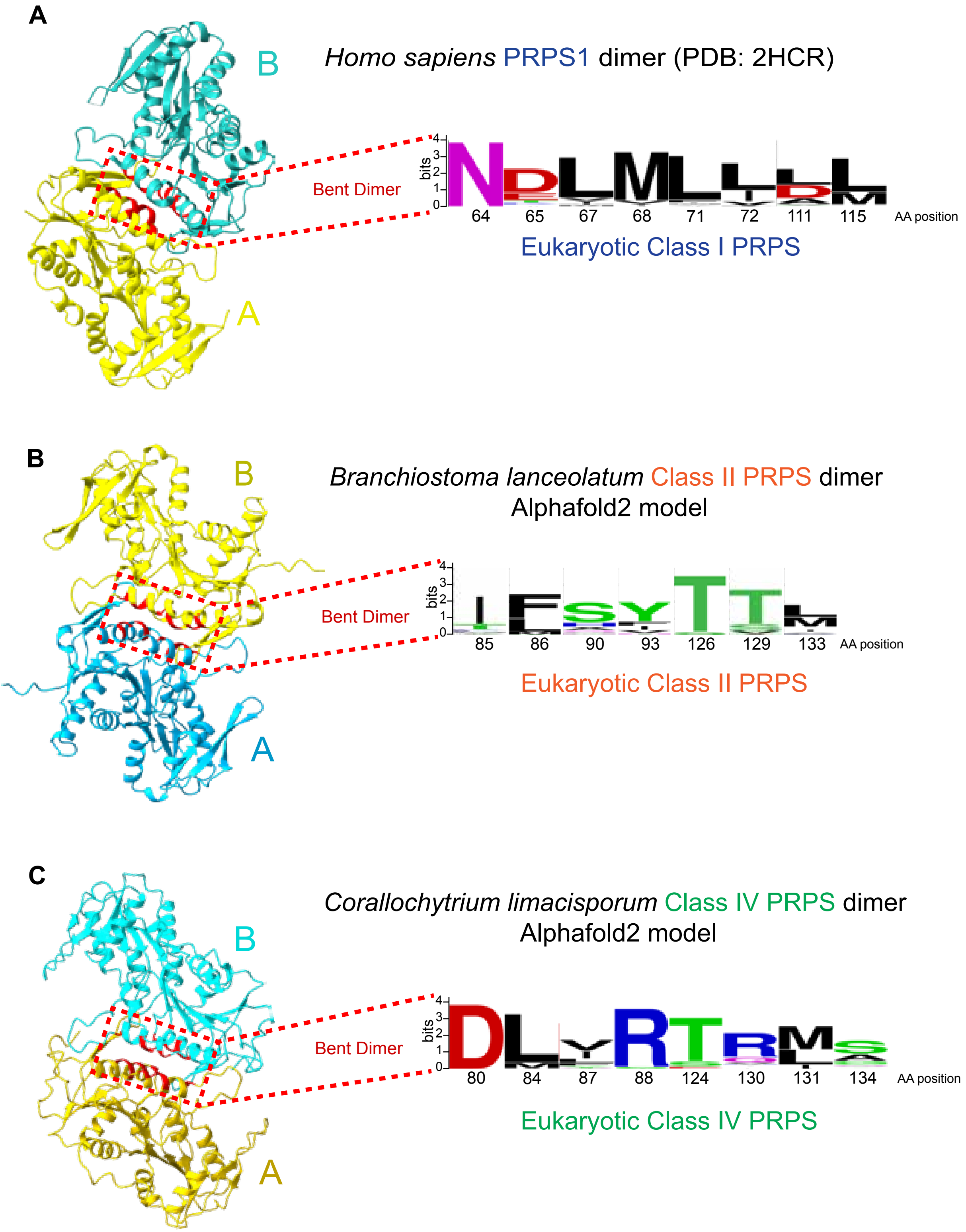

bioRxiv preprint doi: https://doi.org/10.64898/2026.06.01.728777; this version posted June 3, 2026. The copyright holder for this preprint (which was not certified by peer review) is the author/funder, who has granted bioRxiv a license to display the preprint in perpetuity. It is made available under a CC-BY 4.0 International license.

Supplementary Figure 17. Sequence divergence at the dimer interface correspond to class-specific assembly of Class I, II, and IV PRPS.

(A) Dimeric structure of human Class I PRPS1 (PDB: 2HCR), with the bent dimer interface highlighted (dashed box). Residues contributing to the interface between subunits A and B are shown in red. WebLogo derived from multiple sequence alignment of eukaryotic Class I PRPS sequences (n = 408) illustrates conservation of interface residues, primarily comprising helices α2 and α3, with positions referenced to *Homo sapiens* PRPS1. **(B, C)** Predicted dimeric structures of *Branchiostoma lanceolatum* Class II PRPS (B) and *Corallochytrium limacisporum* Class IV PRPS (C), generated using AlphaFold2. Interface residues from corresponding positions as shown in (A) are highlighted. WebLogo representations from eukaryotic sequences (Class IV, n = 76; Class II, n = 475) show class-specific conservation patterns within the interface region. Residue positions are referenced to the respective representative sequences.

bioRxiv preprint doi: https://doi.org/10.64898/2026.06.01.728777; this version posted June 3, 2026. The copyright holder for this preprint (which was not certified by peer review) is the author/funder, who has granted bioRxiv a license to display the preprint in perpetuity. It is made available under a CC-BY 4.0 International license.

Supplementary Fig. 18

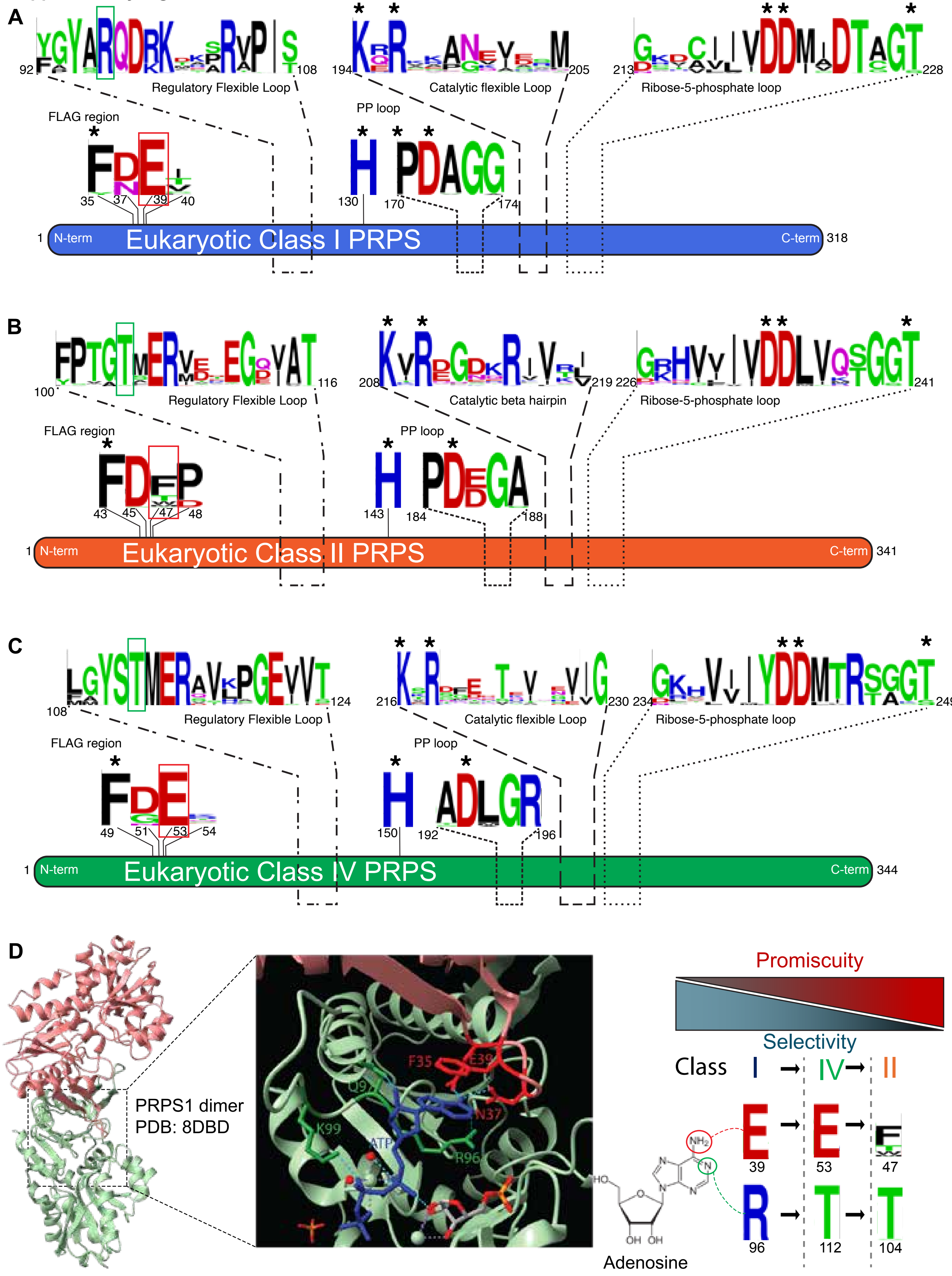

bioRxiv preprint doi: https://doi.org/10.64898/2026.06.01.728777; this version posted June 3, 2026. The copyright holder for this preprint (which was not certified by peer review) is the author/funder, who has granted bioRxiv a license to display the preprint in perpetuity. It is made available under a CC-BY 4.0 International license.

Supplementary Figure 18: Neofunctionalization toward substrate promiscuity in the evolution of PRPS Class II.

(A-C) WebLogo representations of conserved motifs derived from multiple sequence alignments of eukaryotic PRPS, including Class I (A; n = 408), Class II (C; n = 475), and Class IV (B; n = 76). Conserved residues within key functional regions – the FLAG region, regulatory flexible (RF) loop, pyrophosphate (PP) loop, catalytic flexible (CF) loop (or catalytic β-hairpin in Class II), and ribose-5-phosphate (R5P) binding loop are indicated. Residue positions refer to the following representative sequences: *Homo sapiens* PRPS1 for Class I, *Amoebidium parasiticum* PRPS for Class II, and *Corallochytrium limacisporum* PRPS for Class IV. Asterisks denote conserved catalytic residues shared across PRPS classes. Comparative motif composition highlights divergence in regulatory and catalytic regions, with Class II exhibiting distinct sequence features consistent with altered substrate interactions. Green and red boxes highlight key residues in the RF loop and FLAG region that confer ATP specificity. **(D)** Structural basis of catalytic site divergence. A PRPS1 dimer (PDB: 8DBD) is shown (left) with monomers colored in light green and salmon. A zoomed-in view of the active site highlights interactions of ATP with FLAG and RF loop residues, with key side chains labeled (middle). Structure of adenosine is shown to emphasize specific interactions with key Class I residues and a stepwise shift away from ATP specificity (right). The gradient illustrates a conceptual shift from a specialist enzyme specific to ATP as a diphosphoryl donor to a generalist enzyme with increased substrate promiscuity (Class II), with Class IV exhibiting intermediate characteristics.

bioRxiv preprint doi: https://doi.org/10.64898/2026.06.01.728777; this version posted June 3, 2026. The copyright holder for this preprint (which was not certified by peer review) is the author/funder, who has granted bioRxiv a license to display the preprint in perpetuity. It is made available under a CC-BY 4.0 International license.

Supplementary Fig. 19

A

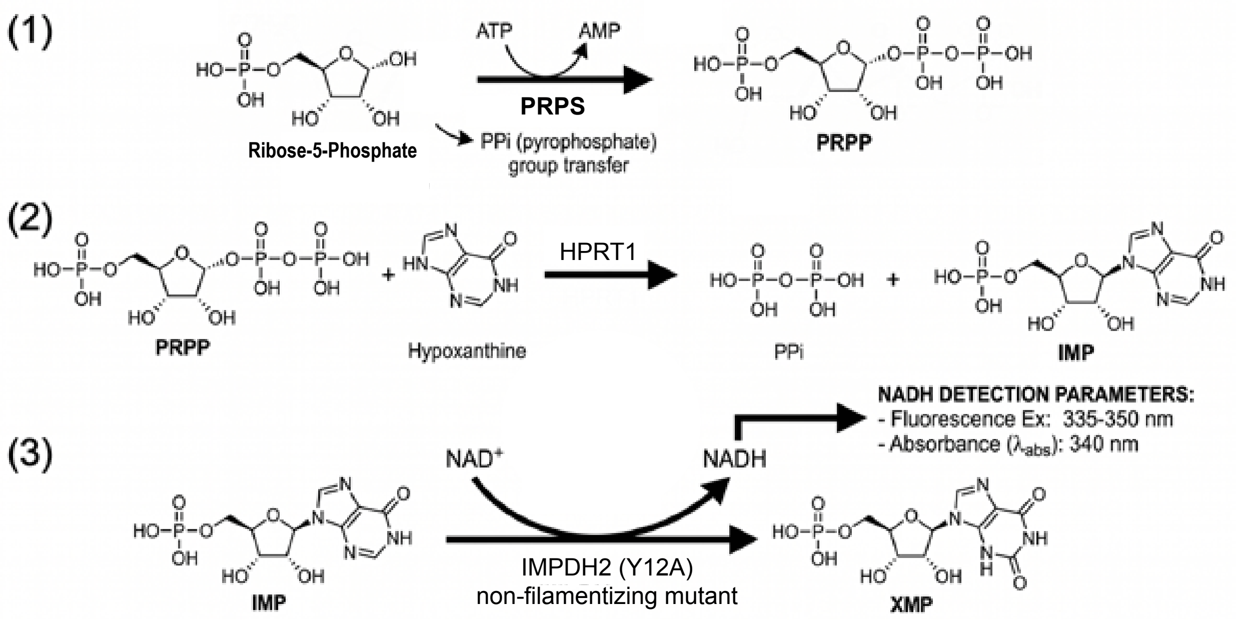

B

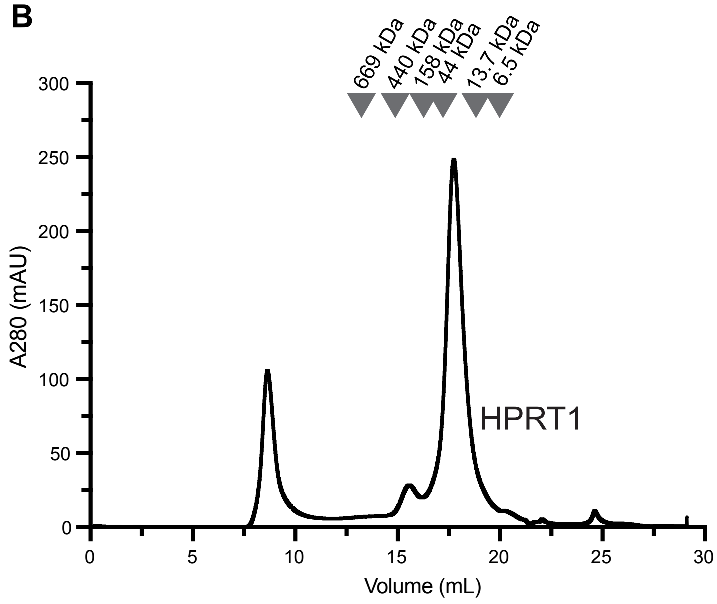

C

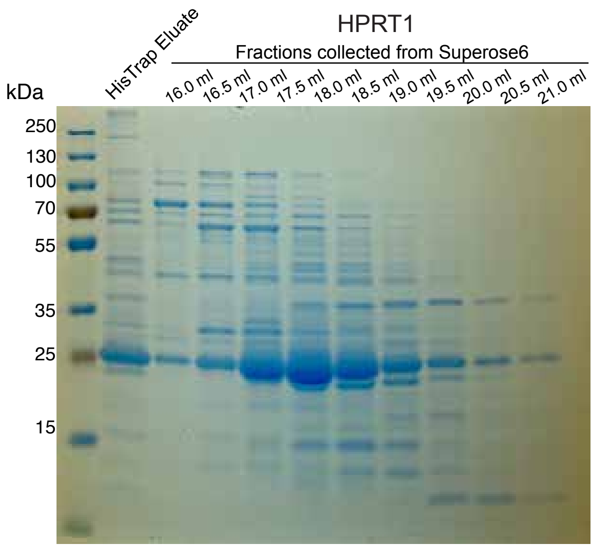

D

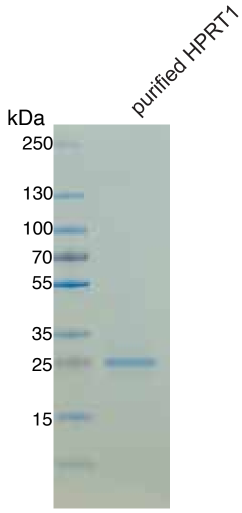

bioRxiv preprint doi: https://doi.org/10.64898/2026.06.01.728777; this version posted June 3, 2026. The copyright holder for this preprint (which was not certified by peer review) is the author/funder, who has granted bioRxiv a license to display the preprint in perpetuity. It is made available under a CC-BY 4.0 International license.

Supplementary Figure 19. PRPS enzyme activity assay and purification of HPRT1. (A) Schematic of the PRPS activity assay. PRPS activity is measured through a coupled reaction in which PRPP generated by PRPS from ATP and ribose-5-phosphate is then converted by HPRT1 to IMP, followed by oxidation by IMPDH2 (Y12A; non-filamentizing mutant)[91]. This reaction produces NADH from NAD^+, which is monitored continuously by fluorescence (excitation 335-350 nm, emission 470 nm). **(B)** Size-exclusion chromatography (SEC) elution profile of purified human HPRT1 on a Superose 6 Increase 10/300 GL column. **(C)** SDS-PAGE followed by Coomassie staining of fractions collected from the SEC run in (B), showing enrichment of HPRT1 in peak fractions. **(D)** SDS-PAGE followed by Coomassie staining of purified HPRT1 peak fraction used in activity assays.

bioRxiv preprint doi: https://doi.org/10.64898/2026.06.01.728777; this version posted June 3, 2026. The copyright holder for this preprint (which was not certified by peer review) is the author/funder, who has granted bioRxiv a license to display the preprint in perpetuity. It is made available under a CC-BY 4.0 International license.

Supplementary Fig. 20

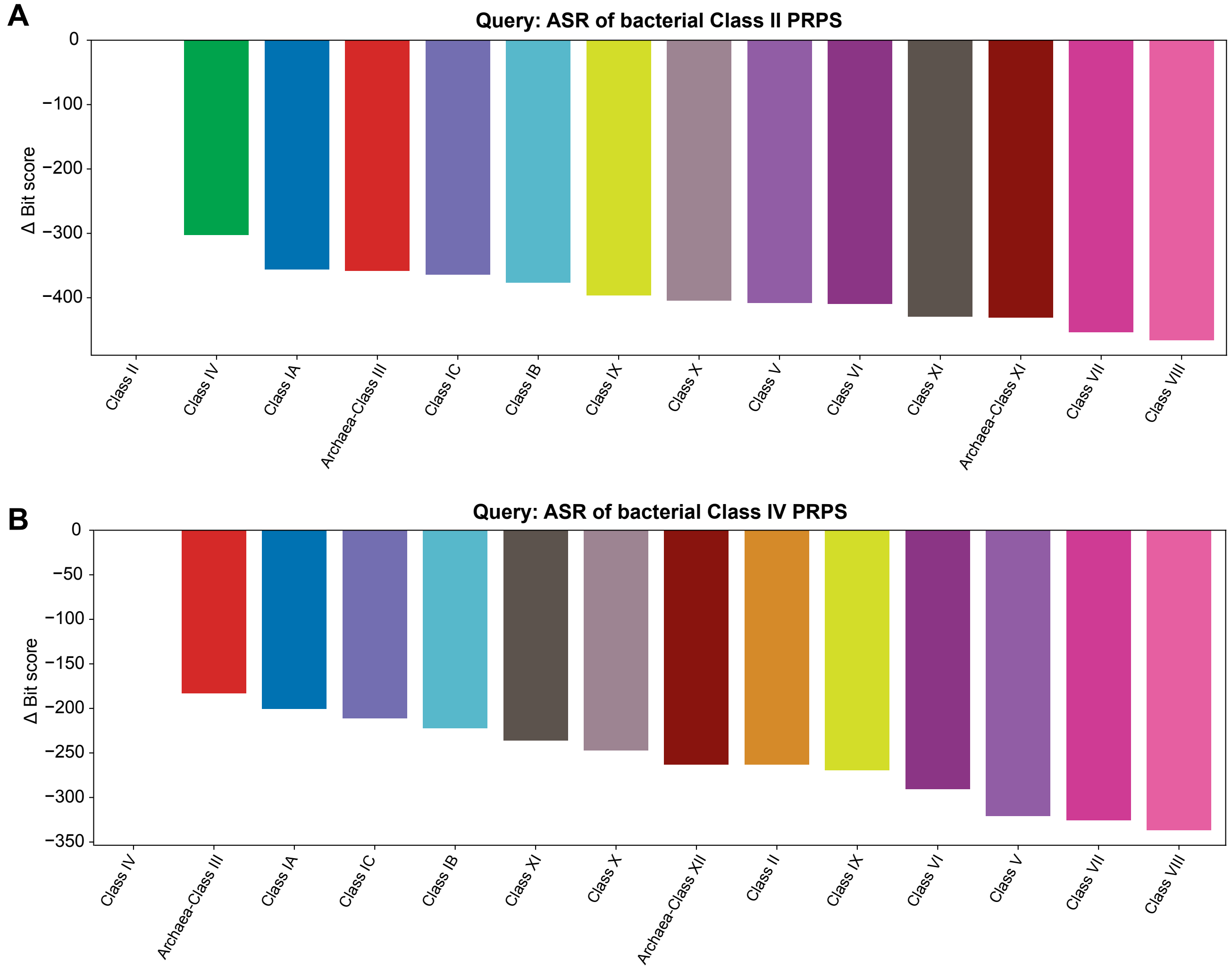

bioRxiv preprint doi: https://doi.org/10.64898/2026.06.01.728777; this version posted June 3, 2026. The copyright holder for this preprint (which was not certified by peer review) is the author/funder, who has granted bioRxiv a license to display the preprint in perpetuity. It is made available under a CC-BY 4.0 International license.

Supplementary Figure 20. Ancestrally reconstructed bacterial sequences distinguish Class II and Class IV from other PRPS classes via HMM profiling.

(A, B) Waterfall plots showing Δ bit scores for ancestrally reconstructed bacterial Class II (A) and Class IV (B) PRPS sequences queried against a panel of prokaryotic class-level PRPS profile HMMs. For each query, Δ bit scores are calculated relative to the top-scoring model ($\Delta = 0$). Ancestral sequence reconstruction is described in Methods. The bacterial Class II ancestor scored highest against the Class II profile, with Class IV as the next closest class-level profile, supporting a close relationship between Class II and Class IV. In contrast, the bacterial Class IV ancestor scored highest against the Class IV profile and showed closer similarity to Class IA/Class III-associated profiles than with the Class II profile. Together, these patterns are consistent with a model in which Class IV represents an intermediate PRPS class that diverged from a Class I/III-like ancestor before subsequent emergence of Class II from a Class IV-like precursor.
