# Supplementary material for: Divergent evolution of the PRPS enzymes across the tree of life
Source: bioRxiv. 2026 Jun 3:2026.06.01.728777. Preprint. [Version 1] doi: 10.64898/2026.06.01.728777 (PMC13252144; doi:10.64898/2026.06.01.728777)
Supplement: Supplement 1 [file media-1.pdf]

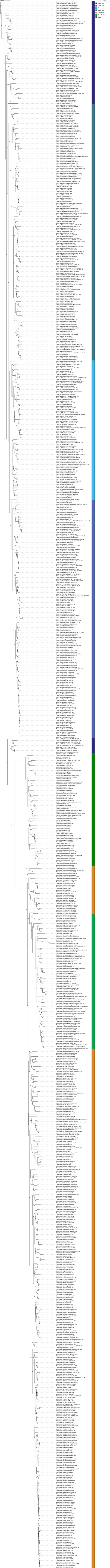

Eukaryotic PRPS Classes
Class I sub1
Class I sub2
Class I sub3
Class I sub4
Class II sub1
Class II sub2
Class IV
