# Supplementary material for: Divergent evolution of the PRPS enzymes across the tree of life
Source: bioRxiv. 2026 Jun 3:2026.06.01.728777. Preprint. [Version 1] doi: 10.64898/2026.06.01.728777 (PMC13252144; doi:10.64898/2026.06.01.728777)
Supplement: Supplement 2 [file media-2.pdf]

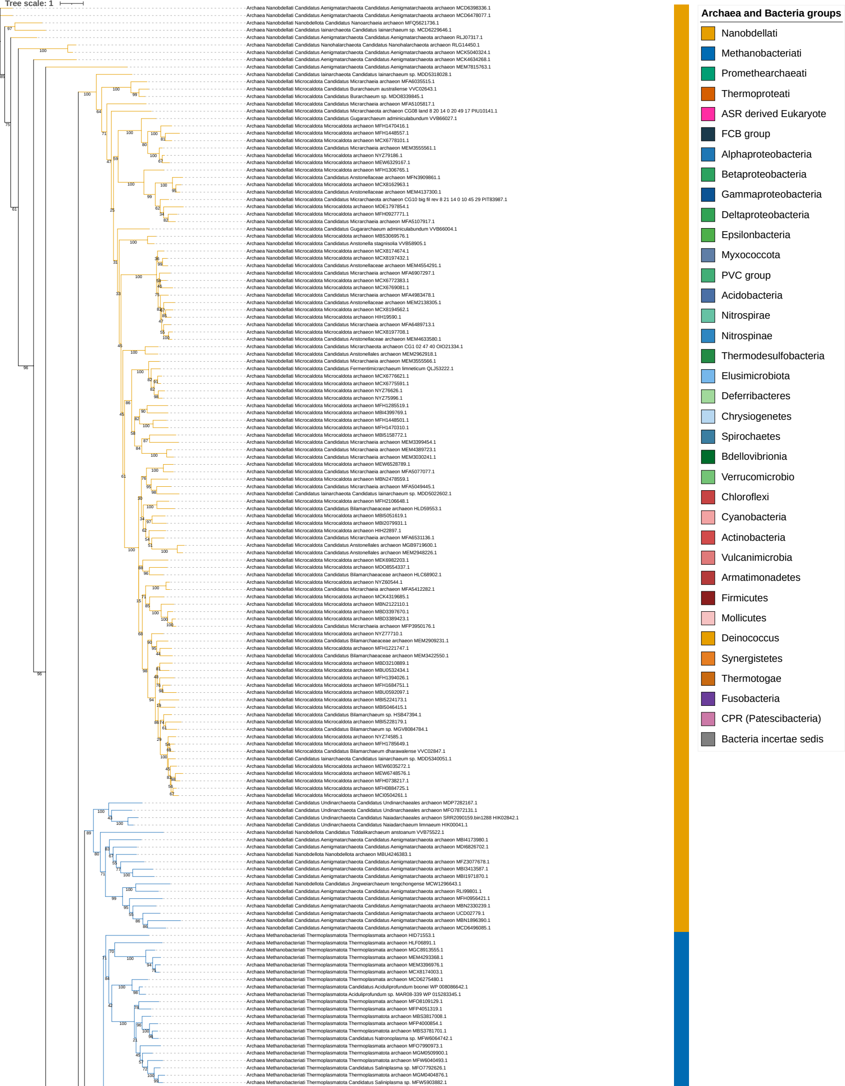
Tree scale: 1
Archaea Nanobdellati Candidatus Aenigmatarchaeota Candidatus Aenigmatarchaeota archaeon MCD6398336.1
Archaea Nanobdellati Candidatus Aenigmatarchaeota Candidatus Aenigmatarchaeota archaeon MCD6478077.1
Archaea Nanobdellati Nanobdellota Candidatus Nanoarchaeia archaeon MFQ5621736.1
Archaea Nanobdellati Candidatus Iainarchaeota Candidatus Iainarchaeum sp. MCD6229646.1
Archaea Nanobdellati Candidatus Aenigmatarchaeota Candidatus Aenigmatarchaeota archaeon RLJ07317.1
Archaea Nanobdellati Candidatus Nanohalarchaeota Candidatus Nanohalarchaeota archaeon RLG14450.1
Archaea Nanobdellati Candidatus Aenigmatarchaeota Candidatus Aenigmatarchaeota archaeon MCK5040324.1
Archaea Nanobdellati Candidatus Aenigmatarchaeota Candidatus Aenigmatarchaeota archaeon MCK4634268.1
Archaea Nanobdellati Candidatus Aenigmatarchaeota Candidatus Aenigmatarchaeota archaeon MEM7815763.1
Archaea Nanobdellati Candidatus Iainarchaeota Candidatus Iainarchaeum sp. MDD5318028.1
Archaea Nanobdellati Microcaldota Candidatus Micrarchaeia archaeon MFA6035515.1
Archaea Nanobdellati Microcaldota Candidatus Burarchaeum australiense VVC02643.1
Archaea Nanobdellati Microcaldota Candidatus Burarchaeum sp. MDO8339845.1
Archaea Nanobdellati Microcaldota Candidatus Micrarchaeia archaeon MFA5105817.1
Archaea Nanobdellati Microcaldota Candidatus Micrarchaeota archaeon CG08 land 8 20 14 0 20 49 17 PIU10141.1
Archaea Nanobdellati Microcaldota Candidatus Gugararchaeum adminiculabundum VVB66027.1
Archaea Nanobdellati Microcaldota Microcaldota archaeon MFH1470416.1
Archaea Nanobdellati Microcaldota Microcaldota archaeon MFH1448557.1
Archaea Nanobdellati Microcaldota Microcaldota archaeon MCX6778101.1
Archaea Nanobdellati Microcaldota Candidatus Micrarchaeia archaeon MEM3555561.1
Archaea Nanobdellati Microcaldota Microcaldota archaeon NYZ79186.1
Archaea Nanobdellati Microcaldota Microcaldota archaeon MEW6329167.1
Archaea Nanobdellati Microcaldota Microcaldota archaeon MFH1306765.1
Archaea Nanobdellati Microcaldota Candidatus Anstonellaceae archaeon MFN3909861.1
Archaea Nanobdellati Microcaldota Microcaldota archaeon MCX8162963.1
Archaea Nanobdellati Microcaldota Candidatus Anstonellaceae archaeon MEM4137300.1
Archaea Nanobdellati Microcaldota Candidatus Micrarchaeota archaeon CG10 big fil rev 8 21 14 0 10 45 29 PIT83987.1
Archaea Nanobdellati Microcaldota Microcaldota archaeon MDE1797854.1
Archaea Nanobdellati Microcaldota Microcaldota archaeon MFH0927771.1
Archaea Nanobdellati Microcaldota Candidatus Micrarchaeia archaeon MFA5107917.1
Archaea Nanobdellati Microcaldota Candidatus Gugararchaeum adminiculabundum VVB66004.1
Archaea Nanobdellati Microcaldota Microcaldota archaeon MBS3069576.1
Archaea Nanobdellati Microcaldota Candidatus Anstonella stagnisolia VVB58905.1
Archaea Nanobdellati Microcaldota Microcaldota archaeon MCX8174674.1
Archaea Nanobdellati Microcaldota Microcaldota archaeon MCX8197432.1
Archaea Nanobdellati Microcaldota Candidatus Anstonellaceae archaeon MEM4554291.1
Archaea Nanobdellati Microcaldota Candidatus Micrarchaeia archaeon MFA6907297.1
Archaea Nanobdellati Microcaldota Microcaldota archaeon MCX6772383.1
Archaea Nanobdellati Microcaldota Microcaldota archaeon MCX6769081.1
Archaea Nanobdellati Microcaldota Candidatus Micrarchaeia archaeon MFA4983478.1
Archaea Nanobdellati Microcaldota Candidatus Anstonellaceae archaeon MEM2138305.1
Archaea Nanobdellati Microcaldota Microcaldota archaeon MCX8194562.1
Archaea Nanobdellati Microcaldota Microcaldota archaeon HIH19590.1
Archaea Nanobdellati Microcaldota Candidatus Micrarchaeia archaeon MFA6489713.1
Archaea Nanobdellati Microcaldota Microcaldota archaeon MCX8197708.1
Archaea Nanobdellati Microcaldota Candidatus Anstonellaceae archaeon MEM4633580.1
Archaea Nanobdellati Microcaldota Candidatus Micrarchaeota archaeon CG1 02 47 40 OIO21334.1
Archaea Nanobdellati Microcaldota Candidatus Anstonellales archaeon MEM2962918.1
Archaea Nanobdellati Microcaldota Candidatus Micrarchaeia archaeon MEM3555566.1
Archaea Nanobdellati Microcaldota Candidatus Fermentimicrarchaeum limneticum QLJ53222.1
Archaea Nanobdellati Microcaldota Microcaldota archaeon MCX6776621.1
Archaea Nanobdellati Microcaldota Microcaldota archaeon MCX6775591.1
Archaea Nanobdellati Microcaldota Microcaldota archaeon NYZ76626.1
Archaea Nanobdellati Microcaldota Microcaldota archaeon NYZ75996.1
Archaea Nanobdellati Microcaldota Microcaldota archaeon MFH1285519.1
Archaea Nanobdellati Microcaldota Microcaldota archaeon MBI4399769.1
Archaea Nanobdellati Microcaldota Microcaldota archaeon MFH1448501.1
Archaea Nanobdellati Microcaldota Microcaldota archaeon MFH1470310.1
Archaea Nanobdellati Microcaldota Microcaldota archaeon MBI5158772.1
Archaea Nanobdellati Microcaldota Candidatus Micrarchaeia archaeon MEM3399454.1
Archaea Nanobdellati Microcaldota Candidatus Micrarchaeia archaeon MEM4389723.1
Archaea Nanobdellati Microcaldota Candidatus Micrarchaeia archaeon MEM3030241.1
Archaea Nanobdellati Microcaldota Microcaldota archaeon MEW6528789.1
Archaea Nanobdellati Microcaldota Candidatus Micrarchaeia archaeon MFA5077077.1
Archaea Nanobdellati Microcaldota Microcaldota archaeon MBN2478559.1
Archaea Nanobdellati Microcaldota Candidatus Micrarchaeia archaeon MFA5049445.1
Archaea Nanobdellati Candidatus Iainarchaeota Candidatus Iainarchaeum sp. MDD5022602.1
Archaea Nanobdellati Microcaldota Microcaldota archaeon MFH2106648.1
Archaea Nanobdellati Microcaldota Candidatus Bilamarchaeaceae archaeon HLD59553.1
Archaea Nanobdellati Microcaldota Microcaldota archaeon MBI5051619.1
Archaea Nanobdellati Microcaldota Microcaldota archaeon MBI2079931.1
Archaea Nanobdellati Microcaldota Microcaldota archaeon HIH22897.1
Archaea Nanobdellati Microcaldota Candidatus Micrarchaeia archaeon MFA6531136.1
Archaea Nanobdellati Microcaldota Candidatus Anstonellales archaeon MGB9719600.1
Archaea Nanobdellati Microcaldota Candidatus Anstonellales archaeon MEM2948226.1
Archaea Nanobdellati Microcaldota Microcaldota archaeon MEK6982203.1
Archaea Nanobdellati Microcaldota Microcaldota archaeon MDO8554337.1
Archaea Nanobdellati Microcaldota Candidatus Bilamarchaeaceae archaeon HLC68902.1
Archaea Nanobdellati Microcaldota Microcaldota archaeon NYZ60544.1
Archaea Nanobdellati Microcaldota Candidatus Micrarchaeia archaeon MFA5412282.1
Archaea Nanobdellati Microcaldota Microcaldota archaeon MCK4319685.1
Archaea Nanobdellati Microcaldota Microcaldota archaeon MBN2122110.1
Archaea Nanobdellati Microcaldota Microcaldota archaeon MBD3397670.1
Archaea Nanobdellati Microcaldota Microcaldota archaeon MBD3389423.1
Archaea Nanobdellati Microcaldota Candidatus Micrarchaeia archaeon MFP3950176.1
Archaea Nanobdellati Microcaldota Microcaldota archaeon NYZ77710.1
Archaea Nanobdellati Microcaldota Candidatus Bilamarchaeaceae archaeon MEM2909231.1
Archaea Nanobdellati Microcaldota Microcaldota archaeon MFH1221747.1
Archaea Nanobdellati Microcaldota Candidatus Bilamarchaeaceae archaeon MEM3422550.1
Archaea Nanobdellati Microcaldota Microcaldota archaeon MBD3210889.1
Archaea Nanobdellati Microcaldota Microcaldota archaeon MBU0532434.1
Archaea Nanobdellati Microcaldota Microcaldota archaeon MFH1394026.1
Archaea Nanobdellati Microcaldota Microcaldota archaeon MFH1684751.1
Archaea Nanobdellati Microcaldota Microcaldota archaeon MBU0592097.1
Archaea Nanobdellati Microcaldota Microcaldota archaeon MBI5224173.1
Archaea Nanobdellati Microcaldota Microcaldota archaeon MBI5046415.1
Archaea Nanobdellati Microcaldota Candidatus Bilamarchaeum sp. HSB47394.1
Archaea Nanobdellati Microcaldota Microcaldota archaeon MBI5228179.1
Archaea Nanobdellati Microcaldota Candidatus Bilamarchaeum sp. MGV8084784.1
Archaea Nanobdellati Microcaldota Microcaldota archaeon NYZ74585.1
Archaea Nanobdellati Microcaldota Microcaldota archaeon MFH1785649.1
Archaea Nanobdellati Microcaldota Candidatus Bilamarchaeum dharawalense VVC02847.1
Archaea Nanobdellati Candidatus Iainarchaeota Candidatus Iainarchaeum sp. MDD5340051.1
Archaea Nanobdellati Microcaldota Microcaldota archaeon MEW6035272.1
Archaea Nanobdellati Microcaldota Microcaldota archaeon MEW6748576.1
Archaea Nanobdellati Microcaldota Microcaldota archaeon MFH0738217.1
Archaea Nanobdellati Microcaldota Microcaldota archaeon MFH0884725.1
Archaea Nanobdellati Microcaldota Microcaldota archaeon MCI0504261.1
Archaea Nanobdellati Candidatus Undinarchaeota Candidatus Undinarchaeales archaeon MDP7282167.1
Archaea Nanobdellati Candidatus Undinarchaeota Candidatus Undinarchaeales archaeon MFO7872131.1
Archaea Nanobdellati Candidatus Undinarchaeota Candidatus Naiadarchaeales archaeon SRR2090159.bin1288 HIK02842.1
Archaea Nanobdellati Candidatus Undinarchaeota Candidatus Naiadarchaeum limnaeum HIK00041.1
Archaea Nanobdellati Nanobdellota Candidatus Tiddalikarchaeum anstoanum VVB75522.1
Archaea Nanobdellati Candidatus Aenigmatarchaeota Candidatus Aenigmatarchaeota archaeon MBI4173980.1
Archaea Nanobdellati Candidatus Aenigmatarchaeota Candidatus Aenigmatarchaeota archaeon MDI6826702.1
Archaea Nanobdellati Nanobdellota Nanobdellota archaeon MBU4246383.1
Archaea Nanobdellati Candidatus Aenigmatarchaeota Candidatus Aenigmatarchaeota archaeon MFZ3077678.1
Archaea Nanobdellati Candidatus Aenigmatarchaeota Candidatus Aenigmatarchaeota archaeon MBI3413587.1
Archaea Nanobdellati Candidatus Aenigmatarchaeota Candidatus Aenigmatarchaeota archaeon MBI1971870.1
Archaea Nanobdellati Nanobdellota Candidatus Jingweiarchaeum tengchongense MCW1296643.1
Archaea Nanobdellati Candidatus Aenigmatarchaeota Candidatus Aenigmatarchaeota archaeon RLI99801.1
Archaea Nanobdellati Candidatus Aenigmatarchaeota Candidatus Aenigmatarchaeota archaeon MFH0956421.1
Archaea Nanobdellati Candidatus Aenigmatarchaeota Candidatus Aenigmatarchaeota archaeon MBN2330239.1
Archaea Nanobdellati Candidatus Aenigmatarchaeota Candidatus Aenigmatarchaeota archaeon UCD02779.1
Archaea Nanobdellati Candidatus Aenigmatarchaeota Candidatus Aenigmatarchaeota archaeon MBN1896390.1
Archaea Nanobdellati Candidatus Aenigmatarchaeota Candidatus Aenigmatarchaeota archaeon MCD6496085.1
Archaea Methanobacteriati Thermoplasmatota Thermoplasmata archaeon HID71553.1
Archaea Methanobacteriati Thermoplasmatota Thermoplasmata archaeon HLF06891.1
Archaea Methanobacteriati Thermoplasmatota Thermoplasmata archaeon MGC8913555.1
Archaea Methanobacteriati Thermoplasmatota Thermoplasmata archaeon MEM4293368.1
Archaea Methanobacteriati Thermoplasmatota Thermoplasmata archaeon MEM3396976.1
Archaea Methanobacteriati Thermoplasmatota Thermoplasmata archaeon MCX8174003.1
Archaea Methanobacteriati Thermoplasmatota Thermoplasmata archaeon MCD6275480.1
Archaea Methanobacteriati Thermoplasmatota Thermoplasmata Candidatus Aciduliprofundum boonei WP 008086642.1
Archaea Methanobacteriati Thermoplasmatota Thermoplasmata Aciduliprofundum sp. MAR08-339 WP 015283345.1
Archaea Methanobacteriati Thermoplasmatota Thermoplasmata archaeon MFO8109129.1
Archaea Methanobacteriati Thermoplasmatota Thermoplasmata archaeon MFP4051319.1
Archaea Methanobacteriati Thermoplasmatota Thermoplasmatota archaeon MBS3817008.1
Archaea Methanobacteriati Thermoplasmatota Thermoplasmata archaeon MFP4000854.1
Archaea Methanobacteriati Thermoplasmatota Thermoplasmatota archaeon MBS3781701.1
Archaea Methanobacteriati Thermoplasmatota Thermoplasmata Candidatus Natronoplasma sp. MFW6064742.1
Archaea Methanobacteriati Thermoplasmatota Thermoplasmata archaeon MFO7990973.1
Archaea Methanobacteriati Thermoplasmatota Thermoplasmatota archaeon MGM0509900.1
Archaea Methanobacteriati Thermoplasmatota Thermoplasmatota archaeon MFW6040493.1
Archaea Methanobacteriati Thermoplasmatota Thermoplasmata Candidatus Saliniplasma sp. MFO7792626.1
Archaea Methanobacteriati Thermoplasmatota Thermoplasmatota archaeon MGM0404876.1
Archaea Methanobacteriati Thermoplasmatota Thermoplasmata Candidatus Saliniplasma sp. MFW5903882.1
Archaea and Bacteria groups
Nanobdellati
Methanobacteriati
Promethearchaeati
Thermoproteati
ASR derived Eukaryote
FCB group
Alphaproteobacteria
Betaproteobacteria
Gammaproteobacteria
Deltaproteobacteria
Epsilonbacteria
Myxococcota
PVC group
Acidobacteria
Nitrospirae
Nitrospinae
Thermodesulfobacteria
Elusimicrobiota
Deferribacteres
Chrysiogenetes
Spirochaetes
Bdellovibrionia
Verrucomicrobio
Chloroflexi
Cyanobacteria
Actinobacteria
Vulcanimicrobia
Armatimonadetes
Firmicutes
Mollicutes
Deinococcus
Synergistetes
Thermotogae
Fusobacteria
CPR (Patescibacteria)
Bacteria incertae sedis

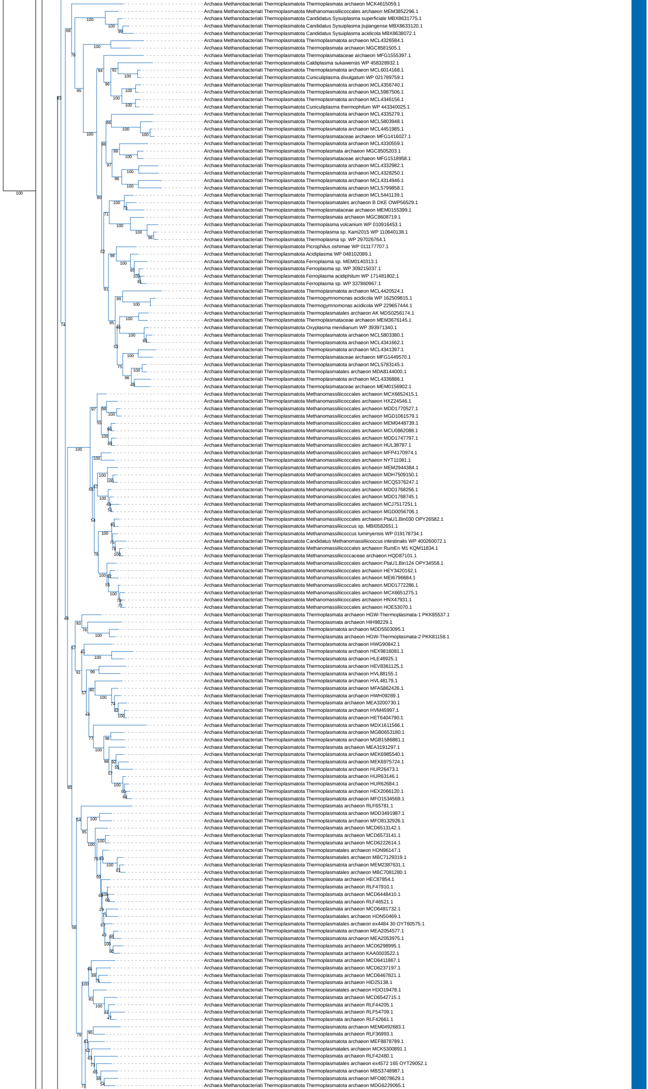
Archaea Methanobacteriati Thermoplasmatota Thermoplasmata archaeon MCK4615059.1
Archaea Methanobacteriati Thermoplasmatota Methanomassiliicoccales archaeon MEM3852296.1
Archaea Methanobacteriati Thermoplasmatota Candidatus Sysuiplasma superficiale MBX8631775.1
Archaea Methanobacteriati Thermoplasmatota Candidatus Sysuiplasma jiujiangense MBX8633120.1
Archaea Methanobacteriati Thermoplasmatota Candidatus Sysuiplasma acidicola MBX8638072.1
Archaea Methanobacteriati Thermoplasmatota Thermoplasmatota archaeon MCL4326584.1
Archaea Methanobacteriati Thermoplasmatota Thermoplasmata archaeon MGC8581505.1
Archaea Methanobacteriati Thermoplasmatota Thermoplasmataceae archaeon MFG1555397.1
Archaea Methanobacteriati Thermoplasmatota Caldiplasma sukawensis WP 458328932.1
Archaea Methanobacteriati Thermoplasmatota Thermoplasmatota archaeon MCL6014168.1
Archaea Methanobacteriati Thermoplasmatota Cuniculiplasma divulgatum WP 021789759.1
Archaea Methanobacteriati Thermoplasmatota Thermoplasmatota archaeon MCL4356740.1
Archaea Methanobacteriati Thermoplasmatota Thermoplasmatota archaeon MCL5987506.1
Archaea Methanobacteriati Thermoplasmatota Thermoplasmatota archaeon MCL4346156.1
Archaea Methanobacteriati Thermoplasmatota Cuniculiplasma thermophilum WP 443340025.1
Archaea Methanobacteriati Thermoplasmatota Thermoplasmatota archaeon MCL4335279.1
Archaea Methanobacteriati Thermoplasmatota Thermoplasmatota archaeon MCL5803948.1
Archaea Methanobacteriati Thermoplasmatota Thermoplasmatota archaeon MCL4451985.1
Archaea Methanobacteriati Thermoplasmatota Thermoplasmataceae archaeon MFG1416027.1
Archaea Methanobacteriati Thermoplasmatota Thermoplasmatota archaeon MCL4330559.1
Archaea Methanobacteriati Thermoplasmatota Thermoplasmata archaeon MGC8505203.1
Archaea Methanobacteriati Thermoplasmatota Thermoplasmataceae archaeon MFG1518958.1
Archaea Methanobacteriati Thermoplasmatota Thermoplasmatota archaeon MCL4332982.1
Archaea Methanobacteriati Thermoplasmatota Thermoplasmatota archaeon MCL4328250.1
Archaea Methanobacteriati Thermoplasmatota Thermoplasmatota archaeon MCL4314946.1
Archaea Methanobacteriati Thermoplasmatota Thermoplasmatota archaeon MCL5799858.1
Archaea Methanobacteriati Thermoplasmatota Thermoplasmatota archaeon MCL5441139.1
Archaea Methanobacteriati Thermoplasmatota Thermoplasmatales archaeon B DKE OWP56529.1
Archaea Methanobacteriati Thermoplasmatota Thermoplasmataceae archaeon MEM0155399.1
Archaea Methanobacteriati Thermoplasmatota Thermoplasmata archaeon MGC8608719.1
Archaea Methanobacteriati Thermoplasmatota Thermoplasma volcanium WP 010916453.1
Archaea Methanobacteriati Thermoplasmatota Thermoplasma sp. Kam2015 WP 110640138.1
Archaea Methanobacteriati Thermoplasmatota Thermoplasma sp. WP 297026764.1
Archaea Methanobacteriati Thermoplasmatota Picrophilus oshimae WP 011177707.1
Archaea Methanobacteriati Thermoplasmatota Acidiplasma WP 048102089.1
Archaea Methanobacteriati Thermoplasmatota Ferroplasma sp. MEM0140313.1
Archaea Methanobacteriati Thermoplasmatota Ferroplasma sp. WP 309215037.1
Archaea Methanobacteriati Thermoplasmatota Ferroplasma acidiphilum WP 171481802.1
Archaea Methanobacteriati Thermoplasmatota Ferroplasma sp. WP 337860967.1
Archaea Methanobacteriati Thermoplasmatota Thermoplasmatota archaeon MCL4420524.1
Archaea Methanobacteriati Thermoplasmatota Thermogymnomonas acidicola WP 162509815.1
Archaea Methanobacteriati Thermoplasmatota Thermogymnomonas acidicola WP 229657444.1
Archaea Methanobacteriati Thermoplasmatota Thermoplasmatales archaeon AK MDS0256174.1
Archaea Methanobacteriati Thermoplasmatota Thermoplasmataceae archaeon MEM3676145.1
Archaea Methanobacteriati Thermoplasmatota Oxyplasma meridianum WP 393971340.1
Archaea Methanobacteriati Thermoplasmatota Thermoplasmatota archaeon MCL5803380.1
Archaea Methanobacteriati Thermoplasmatota Thermoplasmatota archaeon MCL4341662.1
Archaea Methanobacteriati Thermoplasmatota Thermoplasmatota archaeon MCL4341397.1
Archaea Methanobacteriati Thermoplasmatota Thermoplasmataceae archaeon MFG1449570.1
Archaea Methanobacteriati Thermoplasmatota Thermoplasmatota archaeon MCL5783145.1
Archaea Methanobacteriati Thermoplasmatota Thermoplasmatales archaeon MDA8144000.1
Archaea Methanobacteriati Thermoplasmatota Thermoplasmatota archaeon MCL4336886.1
Archaea Methanobacteriati Thermoplasmatota Thermoplasmataceae archaeon MEM0156902.1
Archaea Methanobacteriati Thermoplasmatota Methanomassiliicoccales archaeon MCX6652415.1
Archaea Methanobacteriati Thermoplasmatota Methanomassiliicoccales archaeon HXZ24546.1
Archaea Methanobacteriati Thermoplasmatota Methanomassiliicoccales archaeon MDD1770527.1
Archaea Methanobacteriati Thermoplasmatota Methanomassiliicoccales archaeon MGD1061579.1
Archaea Methanobacteriati Thermoplasmatota Methanomassiliicoccales archaeon MEM0448739.1
Archaea Methanobacteriati Thermoplasmatota Methanomassiliicoccales archaeon MCU0862088.1
Archaea Methanobacteriati Thermoplasmatota Methanomassiliicoccales archaeon MDD1747797.1
Archaea Methanobacteriati Thermoplasmatota Methanomassiliicoccales archaeon HUL38787.1
Archaea Methanobacteriati Thermoplasmatota Methanomassiliicoccales archaeon MFP4170974.1
Archaea Methanobacteriati Thermoplasmatota Methanomassiliicoccales archaeon NYT11081.1
Archaea Methanobacteriati Thermoplasmatota Methanomassiliicoccales archaeon MEM2944384.1
Archaea Methanobacteriati Thermoplasmatota Methanomassiliicoccales archaeon MDH7509150.1
Archaea Methanobacteriati Thermoplasmatota Methanomassiliicoccales archaeon MCQ5376247.1
Archaea Methanobacteriati Thermoplasmatota Methanomassiliicoccales archaeon MDD1768256.1
Archaea Methanobacteriati Thermoplasmatota Methanomassiliicoccales archaeon MDD1768745.1
Archaea Methanobacteriati Thermoplasmatota Methanomassiliicoccales archaeon MCJ7517251.1
Archaea Methanobacteriati Thermoplasmatota Methanomassiliicoccales archaeon MGD0056706.1
Archaea Methanobacteriati Thermoplasmatota Methanomassiliicoccales archaeon PtaU1.Bin030 OPY26582.1
Archaea Methanobacteriati Thermoplasmatota Methanomassiliicoccus sp. MBI0582651.1
Archaea Methanobacteriati Thermoplasmatota Methanomassiliicoccus luminyensis WP 019178734.1
Archaea Methanobacteriati Thermoplasmatota Candidatus Methanomassiliicoccus intestinalis WP 400260072.1
Archaea Methanobacteriati Thermoplasmatota Methanomassiliicoccales archaeon RumEn M1 KQM11834.1
Archaea Methanobacteriati Thermoplasmatota Methanomassiliicoccaceae archaeon HQD87101.1
Archaea Methanobacteriati Thermoplasmatota Methanomassiliicoccales archaeon PtaU1.Bin124 OPY34558.1
Archaea Methanobacteriati Thermoplasmatota Methanomassiliicoccales archaeon HEY3420162.1
Archaea Methanobacteriati Thermoplasmatota Methanomassiliicoccales archaeon MEI6796684.1
Archaea Methanobacteriati Thermoplasmatota Methanomassiliicoccales archaeon MDD1772286.1
Archaea Methanobacteriati Thermoplasmatota Methanomassiliicoccales archaeon MCX6651275.1
Archaea Methanobacteriati Thermoplasmatota Methanomassiliicoccales archaeon HNX47931.1
Archaea Methanobacteriati Thermoplasmatota Methanomassiliicoccales archaeon HOE53070.1
Archaea Methanobacteriati Thermoplasmatota Thermoplasmata archaeon HGW-Thermoplasmata-1 PKK85537.1
Archaea Methanobacteriati Thermoplasmatota Thermoplasmata archaeon HIH98229.1
Archaea Methanobacteriati Thermoplasmatota Thermoplasmatota archaeon MDD5503095.1
Archaea Methanobacteriati Thermoplasmatota Thermoplasmata archaeon HGW-Thermoplasmata-2 PKK81158.1
Archaea Methanobacteriati Thermoplasmatota Thermoplasmatota archaeon HWG90842.1
Archaea Methanobacteriati Thermoplasmatota Thermoplasmatota archaeon HEX9816081.1
Archaea Methanobacteriati Thermoplasmatota Thermoplasmatota archaeon HLE46925.1
Archaea Methanobacteriati Thermoplasmatota Thermoplasmatota archaeon HEV8361125.1
Archaea Methanobacteriati Thermoplasmatota Thermoplasmatota archaeon HVL88155.1
Archaea Methanobacteriati Thermoplasmatota Thermoplasmatota archaeon HVL48179.1
Archaea Methanobacteriati Thermoplasmatota Thermoplasmatota archaeon MFA5862426.1
Archaea Methanobacteriati Thermoplasmatota Thermoplasmatota archaeon HWH09289.1
Archaea Methanobacteriati Thermoplasmatota Thermoplasmata archaeon MEA3200730.1
Archaea Methanobacteriati Thermoplasmatota Thermoplasmatota archaeon HVM45997.1
Archaea Methanobacteriati Thermoplasmatota Thermoplasmatota archaeon HET6404790.1
Archaea Methanobacteriati Thermoplasmatota Thermoplasmatota archaeon MDX1611566.1
Archaea Methanobacteriati Thermoplasmatota Thermoplasmatota archaeon MGB0653180.1
Archaea Methanobacteriati Thermoplasmatota Thermoplasmatota archaeon MGB1586861.1
Archaea Methanobacteriati Thermoplasmatota Thermoplasmata archaeon MEA3191297.1
Archaea Methanobacteriati Thermoplasmatota Thermoplasmatota archaeon MEK6985540.1
Archaea Methanobacteriati Thermoplasmatota Thermoplasmatota archaeon MEK6975724.1
Archaea Methanobacteriati Thermoplasmatota Thermoplasmatota archaeon HUR26473.1
Archaea Methanobacteriati Thermoplasmatota Thermoplasmatota archaeon HUR63146.1
Archaea Methanobacteriati Thermoplasmatota Thermoplasmatota archaeon HUR62684.1
Archaea Methanobacteriati Thermoplasmatota Thermoplasmatota archaeon HEX2066120.1
Archaea Methanobacteriati Thermoplasmatota Thermoplasmatota archaeon MFO1534569.1
Archaea Methanobacteriati Thermoplasmatota Thermoplasmata archaeon RLF65781.1
Archaea Methanobacteriati Thermoplasmatota Thermoplasmatota archaeon MDD3491987.1
Archaea Methanobacteriati Thermoplasmatota Thermoplasmatota archaeon MFO8132926.1
Archaea Methanobacteriati Thermoplasmatota Thermoplasmata archaeon MCD6513142.1
Archaea Methanobacteriati Thermoplasmatota Thermoplasmata archaeon MCD6573141.1
Archaea Methanobacteriati Thermoplasmatota Thermoplasmata archaeon MCD6222614.1
Archaea Methanobacteriati Thermoplasmatota Thermoplasmatales archaeon HDN96147.1
Archaea Methanobacteriati Thermoplasmatota Thermoplasmatales archaeon MBC7129319.1
Archaea Methanobacteriati Thermoplasmatota Thermoplasmatota archaeon MEM2387631.1
Archaea Methanobacteriati Thermoplasmatota Thermoplasmatales archaeon MBC7081280.1
Archaea Methanobacteriati Thermoplasmatota Thermoplasmata archaeon HEC87854.1
Archaea Methanobacteriati Thermoplasmatota Thermoplasmata archaeon RLF47910.1
Archaea Methanobacteriati Thermoplasmatota Thermoplasmata archaeon MCD6448410.1
Archaea Methanobacteriati Thermoplasmatota Thermoplasmata archaeon RLF46521.1
Archaea Methanobacteriati Thermoplasmatota Thermoplasmata archaeon MCD6481732.1
Archaea Methanobacteriati Thermoplasmatota Thermoplasmatales archaeon HDN50469.1
Archaea Methanobacteriati Thermoplasmatota Thermoplasmatales archaeon ex4484 30 OYT60575.1
Archaea Methanobacteriati Thermoplasmatota Thermoplasmata archaeon MEA2054577.1
Archaea Methanobacteriati Thermoplasmatota Thermoplasmata archaeon MEA2053975.1
Archaea Methanobacteriati Thermoplasmatota Thermoplasmata archaeon MCD6298995.1
Archaea Methanobacteriati Thermoplasmatota Thermoplasmata archaeon KAA0003522.1
Archaea Methanobacteriati Thermoplasmatota Thermoplasmata archaeon MCD6411867.1
Archaea Methanobacteriati Thermoplasmatota Thermoplasmata archaeon MCD6237197.1
Archaea Methanobacteriati Thermoplasmatota Thermoplasmata archaeon MCD6467821.1
Archaea Methanobacteriati Thermoplasmatota Thermoplasmata archaeon HID25138.1
Archaea Methanobacteriati Thermoplasmatota Thermoplasmatales archaeon HDO19478.1
Archaea Methanobacteriati Thermoplasmatota Thermoplasmata archaeon MCD6542715.1
Archaea Methanobacteriati Thermoplasmatota Thermoplasmata archaeon RLF44205.1
Archaea Methanobacteriati Thermoplasmatota Thermoplasmata archaeon RLF54709.1
Archaea Methanobacteriati Thermoplasmatota Thermoplasmata archaeon RLF42661.1
Archaea Methanobacteriati Thermoplasmatota Thermoplasmata archaeon MEM0492683.1
Archaea Methanobacteriati Thermoplasmatota Thermoplasmata archaeon RLF36993.1
Archaea Methanobacteriati Thermoplasmatota Thermoplasmatota archaeon MEF8878789.1
Archaea Methanobacteriati Thermoplasmatota Thermoplasmatales archaeon MCK5300891.1
Archaea Methanobacteriati Thermoplasmatota Thermoplasmata archaeon RLF42480.1
Archaea Methanobacteriati Thermoplasmatota Thermoplasmatales archaeon ex4572 165 OYT29052.1
Archaea Methanobacteriati Thermoplasmatota Thermoplasmatota archaeon MBS3748987.1
Archaea Methanobacteriati Thermoplasmatota Thermoplasmatota archaeon MFO8078629.1
Archaea Methanobacteriati Thermoplasmatota Thermoplasmatota archaeon MDG6229065.1

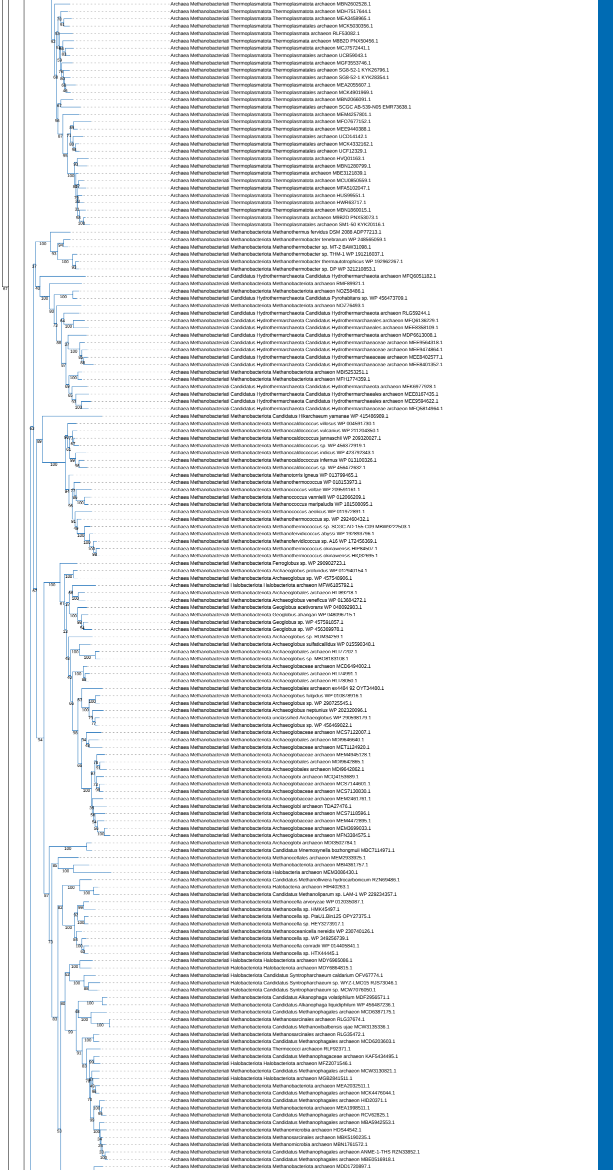
Archaea Methanobacteriati Thermoplasmatota Thermoplasmatota archaeon MBN2602528.1
Archaea Methanobacteriati Thermoplasmatota Thermoplasmatota archaeon MDH7517644.1
Archaea Methanobacteriati Thermoplasmatota Thermoplasmatota archaeon MEA3458965.1
Archaea Methanobacteriati Thermoplasmatota Thermoplasmatales archaeon MCK5030356.1
Archaea Methanobacteriati Thermoplasmatota Thermoplasmata archaeon RLF53082.1
Archaea Methanobacteriati Thermoplasmatota Thermoplasmata archaeon M8B2D PNX50456.1
Archaea Methanobacteriati Thermoplasmatota Thermoplasmatota archaeon MCJ7572441.1
Archaea Methanobacteriati Thermoplasmatota Thermoplasmatales archaeon UCB59043.1
Archaea Methanobacteriati Thermoplasmatota Thermoplasmatota archaeon MGF3553746.1
Archaea Methanobacteriati Thermoplasmatota Thermoplasmatales archaeon SG8-52-1 KYK26796.1
Archaea Methanobacteriati Thermoplasmatota Thermoplasmatales archaeon SG8-52-1 KYK28354.1
Archaea Methanobacteriati Thermoplasmatota Thermoplasmatota archaeon MEA2055607.1
Archaea Methanobacteriati Thermoplasmatota Thermoplasmatales archaeon MCK4901969.1
Archaea Methanobacteriati Thermoplasmatota Thermoplasmatota archaeon MBN2066091.1
Archaea Methanobacteriati Thermoplasmatota Thermoplasmatales archaeon SCGC AB-539-N05 EMR73638.1
Archaea Methanobacteriati Thermoplasmatota Thermoplasmatota archaeon MEM4257801.1
Archaea Methanobacteriati Thermoplasmatota Thermoplasmatota archaeon MFO7677152.1
Archaea Methanobacteriati Thermoplasmatota Thermoplasmatota archaeon MEE9440388.1
Archaea Methanobacteriati Thermoplasmatota Thermoplasmatales archaeon UCD14142.1
Archaea Methanobacteriati Thermoplasmatota Thermoplasmatales archaeon MCK4332162.1
Archaea Methanobacteriati Thermoplasmatota Thermoplasmatales archaeon UCF12329.1
Archaea Methanobacteriati Thermoplasmatota Thermoplasmatota archaeon HVQ01163.1
Archaea Methanobacteriati Thermoplasmatota Thermoplasmatota archaeon MBN1280799.1
Archaea Methanobacteriati Thermoplasmatota Thermoplasmata archaeon MBE3121839.1
Archaea Methanobacteriati Thermoplasmatota Thermoplasmatota archaeon MCU0850559.1
Archaea Methanobacteriati Thermoplasmatota Thermoplasmatota archaeon MFA5102047.1
Archaea Methanobacteriati Thermoplasmatota Thermoplasmatota archaeon HUS99551.1
Archaea Methanobacteriati Thermoplasmatota Thermoplasmatota archaeon HWR63717.1
Archaea Methanobacteriati Thermoplasmatota Thermoplasmatota archaeon MBN1860015.1
Archaea Methanobacteriati Thermoplasmatota Thermoplasmata archaeon M9B2D PNX53073.1
Archaea Methanobacteriati Thermoplasmatota Thermoplasmatales archaeon SM1-50 KYK20116.1
Archaea Methanobacteriati Methanobacteriota Methanothermus fervidus DSM 2088 ADP77213.1
Archaea Methanobacteriati Methanobacteriota Methanothermobacter tenebrarum WP 248565059.1
Archaea Methanobacteriati Methanobacteriota Methanothermobacter sp. MT-2 BAW31098.1
Archaea Methanobacteriati Methanobacteriota Methanothermobacter sp. THM-1 WP 191216037.1
Archaea Methanobacteriati Methanobacteriota Methanothermobacter thermautotrophicus WP 192962267.1
Archaea Methanobacteriati Methanobacteriota Methanothermobacter sp. DP WP 321210853.1
Archaea Methanobacteriati Candidatus Hydrothermarchaeota Candidatus Hydrothermarchaeota archaeon MFQ6051182.1
Archaea Methanobacteriati Methanobacteriota Methanobacteriota archaeon RMF89921.1
Archaea Methanobacteriati Methanobacteriota Methanobacteriota archaeon NOZ58486.1
Archaea Methanobacteriati Candidatus Hydrothermarchaeota Candidatus Pyrohabitans sp. WP 456473709.1
Archaea Methanobacteriati Methanobacteriota Methanobacteriota archaeon NOZ76493.1
Archaea Methanobacteriati Candidatus Hydrothermarchaeota Candidatus Hydrothermarchaeota archaeon RLG59244.1
Archaea Methanobacteriati Candidatus Hydrothermarchaeota Candidatus Hydrothermarchaeales archaeon MFQ6136229.1
Archaea Methanobacteriati Candidatus Hydrothermarchaeota Candidatus Hydrothermarchaeales archaeon MEE8358109.1
Archaea Methanobacteriati Candidatus Hydrothermarchaeota Candidatus Hydrothermarchaeota archaeon MDP6613008.1
Archaea Methanobacteriati Candidatus Hydrothermarchaeota Candidatus Hydrothermarchaeaceae archaeon MEE9564318.1
Archaea Methanobacteriati Candidatus Hydrothermarchaeota Candidatus Hydrothermarchaeaceae archaeon MEE9474864.1
Archaea Methanobacteriati Candidatus Hydrothermarchaeota Candidatus Hydrothermarchaeaceae archaeon MEE8402577.1
Archaea Methanobacteriati Candidatus Hydrothermarchaeota Candidatus Hydrothermarchaeaceae archaeon MEE8401352.1
Archaea Methanobacteriati Methanobacteriota Methanobacteriota archaeon MBI5253251.1
Archaea Methanobacteriati Methanobacteriota Methanobacteriota archaeon MFH1774359.1
Archaea Methanobacteriati Candidatus Hydrothermarchaeota Candidatus Hydrothermarchaeota archaeon MEK6977928.1
Archaea Methanobacteriati Candidatus Hydrothermarchaeota Candidatus Hydrothermarchaeales archaeon MEE8167435.1
Archaea Methanobacteriati Candidatus Hydrothermarchaeota Candidatus Hydrothermarchaeales archaeon MEE9594622.1
Archaea Methanobacteriati Candidatus Hydrothermarchaeota Candidatus Hydrothermarchaeaceae archaeon MFQ5814964.1
Archaea Methanobacteriati Methanobacteriota Candidatus Hikarchaeum yamanae WP 415486989.1
Archaea Methanobacteriati Methanobacteriota Methanocaldococcus villosus WP 004591730.1
Archaea Methanobacteriati Methanobacteriota Methanocaldococcus vulcanius WP 211204350.1
Archaea Methanobacteriati Methanobacteriota Methanocaldococcus jannaschii WP 209320027.1
Archaea Methanobacteriati Methanobacteriota Methanocaldococcus sp. WP 456372919.1
Archaea Methanobacteriati Methanobacteriota Methanocaldococcus indicus WP 423792343.1
Archaea Methanobacteriati Methanobacteriota Methanocaldococcus infernus WP 013100326.1
Archaea Methanobacteriati Methanobacteriota Methanocaldococcus sp. WP 456472632.1
Archaea Methanobacteriati Methanobacteriota Methanotorris igneus WP 013799465.1
Archaea Methanobacteriati Methanobacteriota Methanothermococcus WP 018153973.1
Archaea Methanobacteriati Methanobacteriota Methanococcus voltae WP 209591161.1
Archaea Methanobacteriati Methanobacteriota Methanococcus vannielii WP 012066209.1
Archaea Methanobacteriati Methanobacteriota Methanococcus maripaludis WP 181508095.1
Archaea Methanobacteriati Methanobacteriota Methanococcus aeolicus WP 011972891.1
Archaea Methanobacteriati Methanobacteriota Methanothermococcus sp. WP 292460432.1
Archaea Methanobacteriati Methanobacteriota Methanothermococcus sp. SCGC AD-155-C09 MBW9222503.1
Archaea Methanobacteriati Methanobacteriota Methanofervidicoccus abyssi WP 192893796.1
Archaea Methanobacteriati Methanobacteriota Methanofervidicoccus sp. A16 WP 172456369.1
Archaea Methanobacteriati Methanobacteriota Methanothermococcus okinawensis HIP84507.1
Archaea Methanobacteriati Methanobacteriota Methanothermococcus okinawensis HIQ32695.1
Archaea Methanobacteriati Methanobacteriota Ferroglobus sp. WP 290902723.1
Archaea Methanobacteriati Methanobacteriota Archaeoglobus profundus WP 012940154.1
Archaea Methanobacteriati Methanobacteriota Archaeoglobus sp. WP 457548906.1
Archaea Methanobacteriati Halobacteriota Halobacteriota archaeon MFW6185792.1
Archaea Methanobacteriati Methanobacteriota Archaeoglobales archaeon RLI89218.1
Archaea Methanobacteriati Methanobacteriota Archaeoglobus veneficus WP 013684272.1
Archaea Methanobacteriati Methanobacteriota Geoglobus acetivorans WP 048092983.1
Archaea Methanobacteriati Methanobacteriota Geoglobus ahangari WP 048096715.1
Archaea Methanobacteriati Methanobacteriota Geoglobus sp. WP 457591857.1
Archaea Methanobacteriati Methanobacteriota Geoglobus sp. WP 456369978.1
Archaea Methanobacteriati Methanobacteriota Archaeoglobus sp. RUM34259.1
Archaea Methanobacteriati Methanobacteriota Archaeoglobus sulfaticallidus WP 015590348.1
Archaea Methanobacteriati Methanobacteriota Archaeoglobales archaeon RLI77202.1
Archaea Methanobacteriati Methanobacteriota Archaeoglobus sp. MBO8183108.1
Archaea Methanobacteriati Methanobacteriota Archaeoglobaceae archaeon MCD6494002.1
Archaea Methanobacteriati Methanobacteriota Archaeoglobales archaeon RLI74991.1
Archaea Methanobacteriati Methanobacteriota Archaeoglobales archaeon RLI78050.1
Archaea Methanobacteriati Methanobacteriota Archaeoglobales archaeon ex4484 92 OYT34480.1
Archaea Methanobacteriati Methanobacteriota Archaeoglobus fulgidus WP 010878916.1
Archaea Methanobacteriati Methanobacteriota Archaeoglobus sp. WP 290725545.1
Archaea Methanobacteriati Methanobacteriota Archaeoglobus neptunius WP 202320096.1
Archaea Methanobacteriati Methanobacteriota unclassified Archaeoglobus WP 290598179.1
Archaea Methanobacteriati Methanobacteriota Archaeoglobus sp. WP 456469022.1
Archaea Methanobacteriati Methanobacteriota Archaeoglobaceae archaeon MCS7122007.1
Archaea Methanobacteriati Methanobacteriota Archaeoglobales archaeon MDI9646640.1
Archaea Methanobacteriati Methanobacteriota Archaeoglobaceae archaeon MET1124920.1
Archaea Methanobacteriati Methanobacteriota Archaeoglobaceae archaeon MEM4945128.1
Archaea Methanobacteriati Methanobacteriota Archaeoglobales archaeon MDI9642865.1
Archaea Methanobacteriati Methanobacteriota Archaeoglobales archaeon MDI9642862.1
Archaea Methanobacteriati Methanobacteriota Archaeoglobi archaeon MCQ4153689.1
Archaea Methanobacteriati Methanobacteriota Archaeoglobaceae archaeon MCS7144601.1
Archaea Methanobacteriati Methanobacteriota Archaeoglobaceae archaeon MCS7130830.1
Archaea Methanobacteriati Methanobacteriota Archaeoglobaceae archaeon MEM2461761.1
Archaea Methanobacteriati Methanobacteriota Archaeoglobi archaeon TDA27476.1
Archaea Methanobacteriati Methanobacteriota Archaeoglobaceae archaeon MCS7118596.1
Archaea Methanobacteriati Methanobacteriota Archaeoglobaceae archaeon MEM4472895.1
Archaea Methanobacteriati Methanobacteriota Archaeoglobaceae archaeon MEM3699033.1
Archaea Methanobacteriati Methanobacteriota Archaeoglobaceae archaeon MFN3384575.1
Archaea Methanobacteriati Methanobacteriota Archaeoglobi archaeon MDI3502784.1
Archaea Methanobacteriati Methanobacteriota Candidatus Mnemosynella bozhongmuii MBC7114971.1
Archaea Methanobacteriati Methanobacteriota Methanocellales archaeon MEM2933925.1
Archaea Methanobacteriati Methanobacteriota Methanobacteriota archaeon MBI4361757.1
Archaea Methanobacteriati Methanobacteriota Halobacteria archaeon MEM3086430.1
Archaea Methanobacteriati Methanobacteriota Candidatus Methanolliviera hydrocarbonicum RZN69486.1
Archaea Methanobacteriati Methanobacteriota Halobacteria archaeon HIH40263.1
Archaea Methanobacteriati Methanobacteriota Candidatus Methanoliparum sp. LAM-1 WP 229234357.1
Archaea Methanobacteriati Methanobacteriota Methanocella arvoryzae WP 012035087.1
Archaea Methanobacteriati Methanobacteriota Methanocella sp. HMK45497.1
Archaea Methanobacteriati Methanobacteriota Methanocella sp. PtaU1.Bin125 OPY27375.1
Archaea Methanobacteriati Methanobacteriota Methanocella sp. HEY3273917.1
Archaea Methanobacteriati Methanobacteriota Methanooceanicella nereidis WP 230740126.1
Archaea Methanobacteriati Methanobacteriota Methanocella sp. WP 349256739.1
Archaea Methanobacteriati Methanobacteriota Methanocella conradii WP 014405841.1
Archaea Methanobacteriati Methanobacteriota Methanocella sp. HTX44445.1
Archaea Methanobacteriati Halobacteriota Halobacteriota archaeon MDY6965086.1
Archaea Methanobacteriati Halobacteriota Halobacteriota archaeon MDY6864815.1
Archaea Methanobacteriati Halobacteriota Candidatus Syntropharchaeum caldarium OFV67774.1
Archaea Methanobacteriati Halobacteriota Candidatus Syntropharchaeum sp. WYZ-LMO15 RJS73046.1
Archaea Methanobacteriati Halobacteriota Candidatus Syntropharchaeum sp. MCW7076050.1
Archaea Methanobacteriati Methanobacteriota Candidatus Alkanophaga volatiphilum MDF2956571.1
Archaea Methanobacteriati Methanobacteriota Candidatus Alkanophaga liquidiphilum WP 456487236.1
Archaea Methanobacteriati Methanobacteriota Candidatus Methanophagales archaeon MCD6387175.1
Archaea Methanobacteriati Methanobacteriota Methanosarcinales archaeon RLG37674.1
Archaea Methanobacteriati Methanobacteriota Candidatus Methanoxibalbensis ujae MCW3135336.1
Archaea Methanobacteriati Methanobacteriota Methanosarcinales archaeon RLG35472.1
Archaea Methanobacteriati Methanobacteriota Candidatus Methanophagales archaeon MCD6203603.1
Archaea Methanobacteriati Methanobacteriota Thermococci archaeon RLF92371.1
Archaea Methanobacteriati Methanobacteriota Candidatus Methanophagaceae archaeon KAF5434495.1
Archaea Methanobacteriati Halobacteriota Halobacteriota archaeon MFZ2071546.1
Archaea Methanobacteriati Methanobacteriota Candidatus Methanophagales archaeon MCW3130821.1
Archaea Methanobacteriati Halobacteriota Halobacteriota archaeon MGB2841511.1
Archaea Methanobacteriati Methanobacteriota Methanobacteriota archaeon MEA2032511.1
Archaea Methanobacteriati Methanobacteriota Candidatus Methanophagales archaeon MCK4476044.1
Archaea Methanobacteriati Methanobacteriota Candidatus Methanophagales archaeon HID20371.1
Archaea Methanobacteriati Methanobacteriota Methanobacteriota archaeon MEA1998511.1
Archaea Methanobacteriati Methanobacteriota Candidatus Methanophagales archaeon RCV62825.1
Archaea Methanobacteriati Methanobacteriota Candidatus Methanophagales archaeon MBA5942553.1
Archaea Methanobacteriati Methanobacteriota Methanomicrobia archaeon HDS44542.1
Archaea Methanobacteriati Methanobacteriota Methanosarcinales archaeon MBK5190235.1
Archaea Methanobacteriati Methanobacteriota Methanomicrobia archaeon MBN1761572.1
Archaea Methanobacteriati Methanobacteriota Candidatus Methanophagales archaeon ANME-1-THS RZN33852.1
Archaea Methanobacteriati Methanobacteriota Candidatus Methanophagales archaeon MBE0516918.1
Archaea Methanobacteriati Methanobacteriota Methanobacteriota archaeon MDD1720897.1

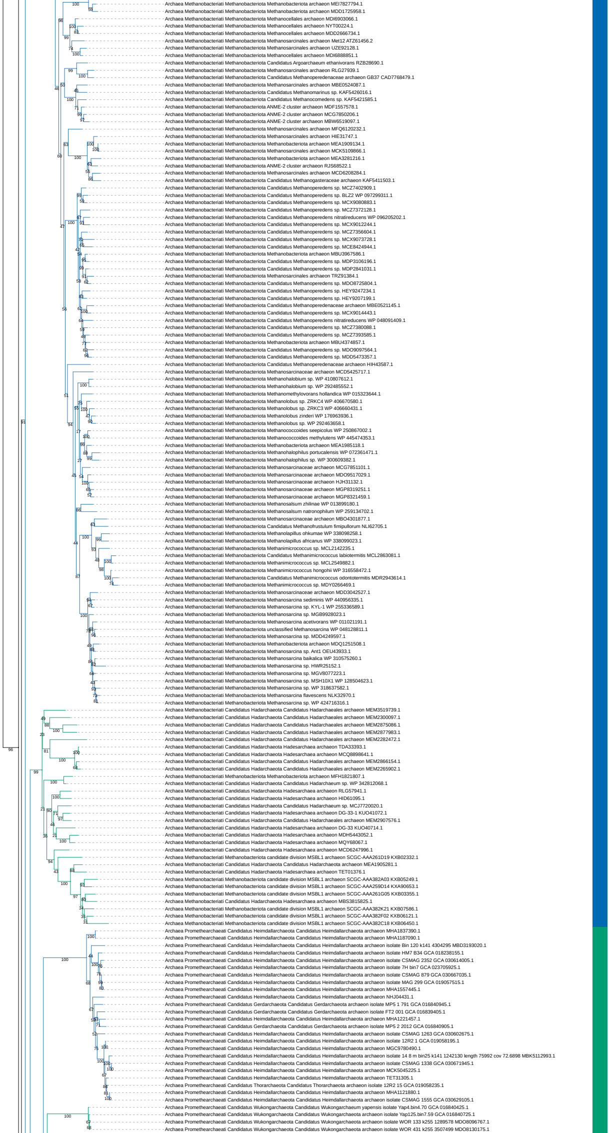
Archaea Methanobacteriati Methanobacteriota Methanobacteriota archaeon MEI7827794.1
Archaea Methanobacteriati Methanobacteriota Methanobacteriota archaeon MDD1725958.1
Archaea Methanobacteriati Methanobacteriota Methanocellales archaeon MDI6903066.1
Archaea Methanobacteriati Methanobacteriota Methanocellales archaeon NYT00224.1
Archaea Methanobacteriati Methanobacteriota Methanocellales archaeon MDD2666734.1
Archaea Methanobacteriati Methanobacteriota Methanosarcinales archaeon Met12 ATZ61456.2
Archaea Methanobacteriati Methanobacteriota Methanosarcinales archaeon UZE92128.1
Archaea Methanobacteriati Methanobacteriota Methanocellales archaeon MDI6888851.1
Archaea Methanobacteriati Methanobacteriota Candidatus Argoarchaeum ethanivorans RZB28690.1
Archaea Methanobacteriati Methanobacteriota Methanosarcinales archaeon RLG27939.1
Archaea Methanobacteriati Methanobacteriota Candidatus Methanoperedenaceae archaeon GB37 CAD7768479.1
Archaea Methanobacteriati Methanobacteriota Methanosarcinales archaeon MBE0524087.1
Archaea Methanobacteriati Methanobacteriota Candidatus Methanomarinus sp. KAF5426016.1
Archaea Methanobacteriati Methanobacteriota Candidatus Methanocomedens sp. KAF5421585.1
Archaea Methanobacteriati Methanobacteriota ANME-2 cluster archaeon MDF1557578.1
Archaea Methanobacteriati Methanobacteriota ANME-2 cluster archaeon MCG7850206.1
Archaea Methanobacteriati Methanobacteriota ANME-2 cluster archaeon MBW6519097.1
Archaea Methanobacteriati Methanobacteriota Methanosarcinales archaeon MFQ6120232.1
Archaea Methanobacteriati Methanobacteriota Methanosarcinales archaeon HIE31747.1
Archaea Methanobacteriati Methanobacteriota Methanobacteriota archaeon MEA1909134.1
Archaea Methanobacteriati Methanobacteriota Methanosarcinales archaeon MCK5109866.1
Archaea Methanobacteriati Methanobacteriota Methanobacteriota archaeon MEA3281216.1
Archaea Methanobacteriati Methanobacteriota ANME-2 cluster archaeon RJS68522.1
Archaea Methanobacteriati Methanobacteriota Methanosarcinales archaeon MCD6208284.1
Archaea Methanobacteriati Methanobacteriota Candidatus Methanogasteraceae archaeon KAF5411503.1
Archaea Methanobacteriati Methanobacteriota Candidatus Methanoperedens sp. MCZ7402909.1
Archaea Methanobacteriati Methanobacteriota Candidatus Methanoperedens sp. BLZ2 WP 097299311.1
Archaea Methanobacteriati Methanobacteriota Candidatus Methanoperedens sp. MCX9080883.1
Archaea Methanobacteriati Methanobacteriota Candidatus Methanoperedens sp. MCZ7372128.1
Archaea Methanobacteriati Methanobacteriota Candidatus Methanoperedens nitratireducens WP 096205202.1
Archaea Methanobacteriati Methanobacteriota Candidatus Methanoperedens sp. MCX9012244.1
Archaea Methanobacteriati Methanobacteriota Candidatus Methanoperedens sp. MCZ7356604.1
Archaea Methanobacteriati Methanobacteriota Candidatus Methanoperedens sp. MCX9073728.1
Archaea Methanobacteriati Methanobacteriota Candidatus Methanoperedens sp. MCE8424944.1
Archaea Methanobacteriati Methanobacteriota Methanobacteriota archaeon MBU3967586.1
Archaea Methanobacteriati Methanobacteriota Candidatus Methanoperedens sp. MDP3106196.1
Archaea Methanobacteriati Methanobacteriota Candidatus Methanoperedens sp. MDP2841031.1
Archaea Methanobacteriati Methanobacteriota Methanosarcinales archaeon TRZ91384.1
Archaea Methanobacteriati Methanobacteriota Candidatus Methanoperedens sp. MDO8725804.1
Archaea Methanobacteriati Methanobacteriota Candidatus Methanoperedens sp. HEY9247234.1
Archaea Methanobacteriati Methanobacteriota Candidatus Methanoperedens sp. HEY9207199.1
Archaea Methanobacteriati Methanobacteriota Candidatus Methanoperedenaceae archaeon MBE0521145.1
Archaea Methanobacteriati Methanobacteriota Candidatus Methanoperedens sp. MCX9014443.1
Archaea Methanobacteriati Methanobacteriota Candidatus Methanoperedens nitratireducens WP 048091409.1
Archaea Methanobacteriati Methanobacteriota Candidatus Methanoperedens sp. MCZ7380088.1
Archaea Methanobacteriati Methanobacteriota Candidatus Methanoperedens sp. MCZ7393585.1
Archaea Methanobacteriati Methanobacteriota Methanobacteriota archaeon MBU4374857.1
Archaea Methanobacteriati Methanobacteriota Candidatus Methanoperedens sp. MDO9097564.1
Archaea Methanobacteriati Methanobacteriota Candidatus Methanoperedens sp. MDD5473357.1
Archaea Methanobacteriati Methanobacteriota Candidatus Methanoperedenaceae archaeon HIH43587.1
Archaea Methanobacteriati Methanobacteriota Methanosarcinaceae archaeon MCD5425717.1
Archaea Methanobacteriati Methanobacteriota Methanohalobium sp. WP 410807612.1
Archaea Methanobacteriati Methanobacteriota Methanohalobium sp. WP 292485552.1
Archaea Methanobacteriati Methanobacteriota Methanomethylovorans hollandica WP 015323644.1
Archaea Methanobacteriati Methanobacteriota Methanolobus sp. ZRKC4 WP 406670580.1
Archaea Methanobacteriati Methanobacteriota Methanolobus sp. ZRKC3 WP 406660431.1
Archaea Methanobacteriati Methanobacteriota Methanolobus zinderi WP 176963936.1
Archaea Methanobacteriati Methanobacteriota Methanolobus sp. WP 292463658.1
Archaea Methanobacteriati Methanobacteriota Methanococcoides seepicolus WP 250867002.1
Archaea Methanobacteriati Methanobacteriota Methanococcoides methylutens WP 445474353.1
Archaea Methanobacteriati Methanobacteriota Methanobacteriota archaeon MEA1985118.1
Archaea Methanobacteriati Methanobacteriota Methanohalophilus portucalensis WP 072361471.1
Archaea Methanobacteriati Methanobacteriota Methanohalophilus sp. WP 300609382.1
Archaea Methanobacteriati Methanobacteriota Methanosarcinaceae archaeon MCG7851101.1
Archaea Methanobacteriati Methanobacteriota Methanosarcinaceae archaeon MDO9517029.1
Archaea Methanobacteriati Methanobacteriota Methanosarcinaceae archaeon HJH31132.1
Archaea Methanobacteriati Methanobacteriota Methanosarcinaceae archaeon MGP8319251.1
Archaea Methanobacteriati Methanobacteriota Methanosarcinaceae archaeon MGP8321459.1
Archaea Methanobacteriati Methanobacteriota Methanosalsum zhilinae WP 013899180.1
Archaea Methanobacteriati Methanobacteriota Methanosalsum natronophilum WP 259134702.1
Archaea Methanobacteriati Methanobacteriota Methanosarcinaceae archaeon MBO4301877.1
Archaea Methanobacteriati Methanobacteriota Candidatus Methanofrustulum fimipullorum NLI62705.1
Archaea Methanobacteriati Methanobacteriota Methanolapillus ohkumae WP 338098258.1
Archaea Methanobacteriati Methanobacteriota Methanolapillus africanus WP 338099023.1
Archaea Methanobacteriati Methanobacteriota Methanimicrococcus sp. MCL2142235.1
Archaea Methanobacteriati Methanobacteriota Candidatus Methanimicrococcus labiotermitis MCL2863081.1
Archaea Methanobacteriati Methanobacteriota Methanimicrococcus sp. MCL2549882.1
Archaea Methanobacteriati Methanobacteriota Methanimicrococcus hongohii WP 316558472.1
Archaea Methanobacteriati Methanobacteriota Candidatus Methanimicrococcus odontotermitis MDR2943614.1
Archaea Methanobacteriati Methanobacteriota Methanimicrococcus sp. MDY0266469.1
Archaea Methanobacteriati Methanobacteriota Methanosarcinaceae archaeon MDD3042527.1
Archaea Methanobacteriati Methanobacteriota Methanosarcina sediminis WP 440956335.1
Archaea Methanobacteriati Methanobacteriota Methanosarcina sp. KYL-1 WP 255336589.1
Archaea Methanobacteriati Methanobacteriota Methanosarcina sp. MGB9928023.1
Archaea Methanobacteriati Methanobacteriota Methanosarcina acetivorans WP 011021191.1
Archaea Methanobacteriati Methanobacteriota unclassified Methanosarcina WP 048128811.1
Archaea Methanobacteriati Methanobacteriota Methanosarcina sp. MDD4249597.1
Archaea Methanobacteriati Methanobacteriota Methanobacteriota archaeon MDQ1251508.1
Archaea Methanobacteriati Methanobacteriota Methanosarcina sp. Ant1 OEU43933.1
Archaea Methanobacteriati Methanobacteriota Methanosarcina baikalica WP 310575260.1
Archaea Methanobacteriati Methanobacteriota Methanosarcina sp. HWR25152.1
Archaea Methanobacteriati Methanobacteriota Methanosarcina sp. MGV8077223.1
Archaea Methanobacteriati Methanobacteriota Methanosarcina sp. MSH10X1 WP 128504623.1
Archaea Methanobacteriati Methanobacteriota Methanosarcina sp. WP 318637582.1
Archaea Methanobacteriati Methanobacteriota Methanosarcina flavescens NLK32970.1
Archaea Methanobacteriati Methanobacteriota Methanosarcina sp. WP 424716316.1
Archaea Methanobacteriati Candidatus Hadarchaeota Candidatus Hadarchaeales archaeon MEM3519739.1
Archaea Methanobacteriati Candidatus Hadarchaeota Candidatus Hadarchaeales archaeon MEM2300097.1
Archaea Methanobacteriati Candidatus Hadarchaeota Candidatus Hadarchaeales archaeon MEM2875086.1
Archaea Methanobacteriati Candidatus Hadarchaeota Candidatus Hadarchaeales archaeon MEM2877983.1
Archaea Methanobacteriati Candidatus Hadarchaeota Candidatus Hadarchaeales archaeon MEM2282472.1
Archaea Methanobacteriati Candidatus Hadarchaeota Hadesarchaea archaeon TDA33393.1
Archaea Methanobacteriati Candidatus Hadarchaeota Hadesarchaea archaeon MCQ8898641.1
Archaea Methanobacteriati Candidatus Hadarchaeota Candidatus Hadarchaeales archaeon MEM2866154.1
Archaea Methanobacteriati Candidatus Hadarchaeota Candidatus Hadarchaeales archaeon MEM2265902.1
Archaea Methanobacteriati Methanobacteriota Methanobacteriota archaeon MFH1821807.1
Archaea Methanobacteriati Candidatus Hadarchaeota Candidatus Hadarchaeum sp. WP 342812068.1
Archaea Methanobacteriati Candidatus Hadarchaeota Hadesarchaea archaeon RLG57941.1
Archaea Methanobacteriati Candidatus Hadarchaeota Hadesarchaea archaeon HID61095.1
Archaea Methanobacteriati Candidatus Hadarchaeota Candidatus Hadarchaeum sp. MCJ7720020.1
Archaea Methanobacteriati Candidatus Hadarchaeota Hadesarchaea archaeon DG-33-1 KUO41072.1
Archaea Methanobacteriati Candidatus Hadarchaeota Candidatus Hadarchaeales archaeon MEM2907576.1
Archaea Methanobacteriati Candidatus Hadarchaeota Hadesarchaea archaeon DG-33 KUO40714.1
Archaea Methanobacteriati Candidatus Hadarchaeota Hadesarchaea archaeon MDH5443052.1
Archaea Methanobacteriati Candidatus Hadarchaeota Hadesarchaea archaeon MQY68067.1
Archaea Methanobacteriati Candidatus Hadarchaeota Hadesarchaea archaeon MCD6247996.1
Archaea Methanobacteriati Methanobacteriota candidate division MSBL1 archaeon SCGC-AAA261D19 KXB02332.1
Archaea Methanobacteriati Candidatus Hadarchaeota Candidatus Hadarchaeota archaeon MEA1905281.1
Archaea Methanobacteriati Candidatus Hadarchaeota Hadesarchaea archaeon TET01376.1
Archaea Methanobacteriati Methanobacteriota candidate division MSBL1 archaeon SCGC-AAA382A03 KXB05249.1
Archaea Methanobacteriati Methanobacteriota candidate division MSBL1 archaeon SCGC-AAA259D14 KXA90653.1
Archaea Methanobacteriati Methanobacteriota candidate division MSBL1 archaeon SCGC-AAA261G05 KXB03355.1
Archaea Methanobacteriati Candidatus Hadarchaeota Hadesarchaea archaeon MBS3815825.1
Archaea Methanobacteriati Methanobacteriota candidate division MSBL1 archaeon SCGC-AAA382K21 KXB07586.1
Archaea Methanobacteriati Methanobacteriota candidate division MSBL1 archaeon SCGC-AAA382F02 KXB06121.1
Archaea Methanobacteriati Methanobacteriota candidate division MSBL1 archaeon SCGC-AAA382C18 KXB06450.1
Archaea Promethearchaeati Candidatus Heimdallarchaeota Candidatus Heimdallarchaeota archaeon MHA1837390.1
Archaea Promethearchaeati Candidatus Heimdallarchaeota Candidatus Heimdallarchaeota archaeon MHA1187090.1
Archaea Promethearchaeati Candidatus Heimdallarchaeota Candidatus Heimdallarchaeota archaeon isolate Bin 120 k141 4304295 MBD3193020.1
Archaea Promethearchaeati Candidatus Heimdallarchaeota Candidatus Heimdallarchaeota archaeon isolate HM7 B34 GCA 018238155.1
Archaea Promethearchaeati Candidatus Heimdallarchaeota Candidatus Heimdallarchaeota archaeon isolate CSMAG 2352 GCA 030614005.1
Archaea Promethearchaeati Candidatus Heimdallarchaeota Candidatus Heimdallarchaeota archaeon isolate 7H bin7 GCA 023705925.1
Archaea Promethearchaeati Candidatus Heimdallarchaeota Candidatus Heimdallarchaeota archaeon isolate CSMAG 879 GCA 030667035.1
Archaea Promethearchaeati Candidatus Heimdallarchaeota Candidatus Heimdallarchaeota archaeon isolate MAG 299 GCA 019057515.1
Archaea Promethearchaeati Candidatus Heimdallarchaeota Candidatus Heimdallarchaeota archaeon MHA1557445.1
Archaea Promethearchaeati Candidatus Heimdallarchaeota Candidatus Heimdallarchaeota archaeon NHJ04431.1
Archaea Promethearchaeati Candidatus Gerdarchaeota Candidatus Gerdarchaeota archaeon isolate MP5 1 791 GCA 016840945.1
Archaea Promethearchaeati Candidatus Gerdarchaeota Candidatus Gerdarchaeota archaeon isolate FT2 001 GCA 016839405.1
Archaea Promethearchaeati Candidatus Heimdallarchaeota Candidatus Heimdallarchaeota archaeon MHA1221457.1
Archaea Promethearchaeati Candidatus Gerdarchaeota Candidatus Gerdarchaeota archaeon isolate MP5 2 2012 GCA 016840905.1
Archaea Promethearchaeati Candidatus Heimdallarchaeota Candidatus Heimdallarchaeota archaeon isolate CSMAG 1263 GCA 030602675.1
Archaea Promethearchaeati Candidatus Heimdallarchaeota Candidatus Heimdallarchaeota archaeon isolate 12R2 1 GCA 019058195.1
Archaea Promethearchaeati Candidatus Heimdallarchaeota Candidatus Heimdallarchaeota archaeon MGC9780490.1
Archaea Promethearchaeati Candidatus Heimdallarchaeota Candidatus Heimdallarchaeota archaeon isolate 14 8 m bin25 k141 1242130 length 75992 cov 72.6898 MBK5112993.1
Archaea Promethearchaeati Candidatus Heimdallarchaeota Candidatus Heimdallarchaeota archaeon isolate CSMAG 1338 GCA 030671945.1
Archaea Promethearchaeati Candidatus Heimdallarchaeota Candidatus Heimdallarchaeota archaeon MCK5045225.1
Archaea Promethearchaeati Candidatus Heimdallarchaeota Candidatus Heimdallarchaeota archaeon TET31305.1
Archaea Promethearchaeati Candidatus Thorarchaeota Candidatus Thorarchaeota archaeon isolate 12R2 15 GCA 019058235.1
Archaea Promethearchaeati Candidatus Heimdallarchaeota Candidatus Heimdallarchaeota archaeon MHA1121880.1
Archaea Promethearchaeati Candidatus Heimdallarchaeota Candidatus Heimdallarchaeota archaeon isolate CSMAG 1555 GCA 030629105.1
Archaea Promethearchaeati Candidatus Wukongarchaeota Candidatus Wukongarchaeum yapensis isolate Yap4.bin4.70 GCA 016840425.1
Archaea Promethearchaeati Candidatus Wukongarchaeota Candidatus Wukongarchaeota archaeon isolate Yap125.bin7.59 GCA 016840725.1
Archaea Promethearchaeati Candidatus Wukongarchaeota Candidatus Wukongarchaeota archaeon isolate WOR 133 k255 1289578 MDO8096767.1
Archaea Promethearchaeati Candidatus Wukongarchaeota Candidatus Wukongarchaeota archaeon isolate WOR 431 k255 3507499 MDO8130175.1

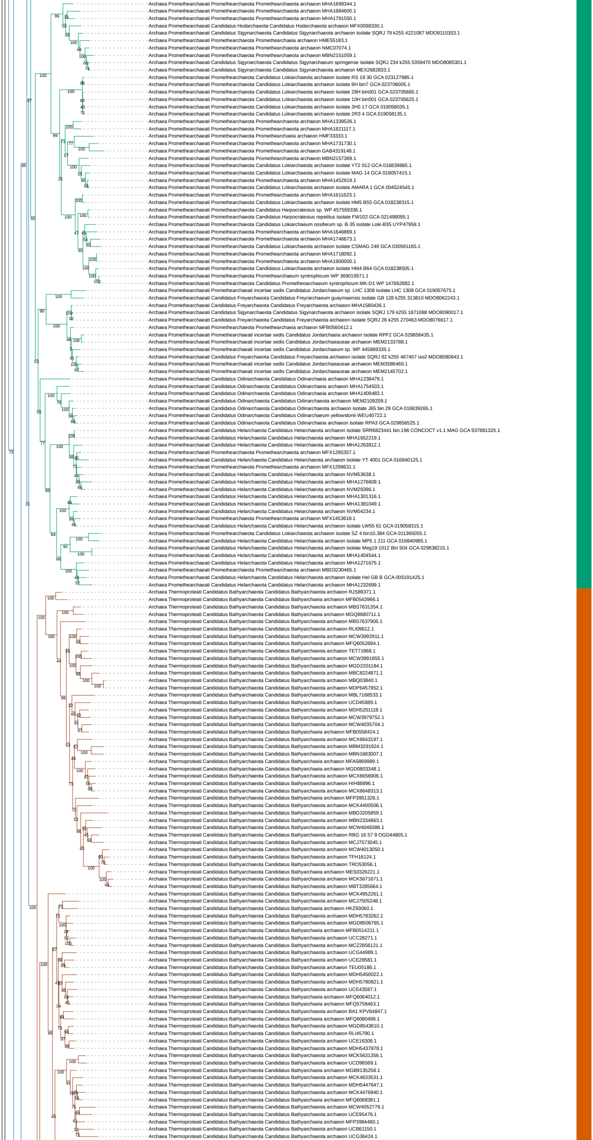
Archaea Promethearchaeati Promethearchaeota Promethearchaeota archaeon MHA1699344.1
Archaea Promethearchaeati Promethearchaeota Promethearchaeota archaeon MHA1684600.1
Archaea Promethearchaeati Promethearchaeota Promethearchaeota archaeon MHA1791550.1
Archaea Promethearchaeati Candidatus Hodarchaeota Candidatus Hodarchaeota archaeon MFX0098330.1
Archaea Promethearchaeati Candidatus Sigynarchaeota Candidatus Sigynarchaeota archaeon isolate SQRJ 79 k255 4221087 MDO8110353.1
Archaea Promethearchaeati Promethearchaeota Promethearchaeia archaeon HME55183.1
Archaea Promethearchaeati Promethearchaeota Promethearchaeota archaeon NMC07074.1
Archaea Promethearchaeati Promethearchaeota Promethearchaeota archaeon MBN2151059.1
Archaea Promethearchaeati Candidatus Sigynarchaeota Candidatus Sigynarchaeum springense isolate SQRJ 234 k255 5359470 MDO8085301.1
Archaea Promethearchaeati Candidatus Sigynarchaeota Candidatus Sigynarchaeota archaeon MEX2682833.1
Archaea Promethearchaeati Promethearchaeota Candidatus Lokiarchaeota archaeon isolate RS 19 30 GCA 023127985.1
Archaea Promethearchaeati Promethearchaeota Candidatus Lokiarchaeota archaeon isolate 8H bin7 GCA 023706005.1
Archaea Promethearchaeati Promethearchaeota Candidatus Lokiarchaeota archaeon isolate 28H bin001 GCA 023705665.1
Archaea Promethearchaeati Promethearchaeota Candidatus Lokiarchaeota archaeon isolate 10H bin001 GCA 023705625.1
Archaea Promethearchaeati Promethearchaeota Candidatus Lokiarchaeota archaeon isolate 3H5 17 GCA 019058035.1
Archaea Promethearchaeati Promethearchaeota Candidatus Lokiarchaeota archaeon isolate 2R3 4 GCA 019058135.1
Archaea Promethearchaeati Promethearchaeota Promethearchaeota archaeon MHA1339526.1
Archaea Promethearchaeati Promethearchaeota Promethearchaeota archaeon MHA1821117.1
Archaea Promethearchaeati Promethearchaeota Promethearchaeia archaeon HMF33333.1
Archaea Promethearchaeati Promethearchaeota Promethearchaeota archaeon MHA1731730.1
Archaea Promethearchaeati Promethearchaeota Promethearchaeota archaeon GAB4319148.1
Archaea Promethearchaeati Promethearchaeota Promethearchaeota archaeon MBN2157369.1
Archaea Promethearchaeati Promethearchaeota Candidatus Lokiarchaeota archaeon isolate YT2 012 GCA 016839865.1
Archaea Promethearchaeati Promethearchaeota Candidatus Lokiarchaeota archaeon isolate MAG 14 GCA 019057415.1
Archaea Promethearchaeati Promethearchaeota Promethearchaeota archaeon MHA1452919.1
Archaea Promethearchaeati Promethearchaeota Candidatus Lokiarchaeota archaeon isolate AMARA 1 GCA 004524545.1
Archaea Promethearchaeati Promethearchaeota Promethearchaeota archaeon MHA1611623.1
Archaea Promethearchaeati Promethearchaeota Candidatus Lokiarchaeota archaeon isolate HM5 B55 GCA 018238315.1
Archaea Promethearchaeati Promethearchaeota Candidatus Harpocratesius sp. WP 457559336.1
Archaea Promethearchaeati Promethearchaeota Candidatus Harpocratesius repetitus isolate FW102 GCA 021498095.1
Archaea Promethearchaeati Promethearchaeota Candidatus Lokiarchaeum ossiferum sp. B-35 isolate Loki-B35 UYP47959.1
Archaea Promethearchaeati Promethearchaeota Promethearchaeota archaeon MHA1646869.1
Archaea Promethearchaeati Promethearchaeota Promethearchaeota archaeon MHA1746673.1
Archaea Promethearchaeati Promethearchaeota Candidatus Lokiarchaeota archaeon isolate CSMAG 249 GCA 030591165.1
Archaea Promethearchaeati Promethearchaeota Promethearchaeota archaeon MHA1718092.1
Archaea Promethearchaeati Promethearchaeota Promethearchaeota archaeon MHA1900000.1
Archaea Promethearchaeati Promethearchaeota Candidatus Lokiarchaeota archaeon isolate HM4 B64 GCA 018238505.1
Archaea Promethearchaeati Promethearchaeota Promethearchaeum syntrophicum WP 369019571.1
Archaea Promethearchaeati Promethearchaeota Candidatus Prometheoarchaeum syntrophicum MK-D1 WP 147662882.1
Archaea Promethearchaeati Promethearchaeati incertae sedis Candidatus Jordarchaeum sp. LHC 1308 isolate LHC 1308 GCA 019057675.1
Archaea Promethearchaeati Candidatus Freyarchaeota Candidatus Freyrarchaeum guaymaensis isolate GB 128 k255 313810 MDO8062243.1
Archaea Promethearchaeati Candidatus Freyarchaeota Candidatus Freyarchaeota archaeon MHA1580436.1
Archaea Promethearchaeati Candidatus Sigynarchaeota Candidatus Sigynarchaeota archaeon isolate SQRJ 179 k255 1871088 MDO8090017.1
Archaea Promethearchaeati Candidatus Freyarchaeota Candidatus Freyarchaeota archaeon isolate SQRJ 26 k255 270463 MDO8076617.1
Archaea Promethearchaeati Promethearchaeota Promethearchaeia archaeon MFB0560412.1
Archaea Promethearchaeati Promethearchaeati incertae sedis Candidatus Jordarchaeia archaeon isolate RPF2 GCA 029856435.1
Archaea Promethearchaeati Promethearchaeati incertae sedis Candidatus Jordarchaeaceae archaeon MEM2133788.1
Archaea Promethearchaeati Promethearchaeati incertae sedis Candidatus Jordarchaeum sp. WP 445869335.1
Archaea Promethearchaeati Candidatus Freyarchaeota Candidatus Freyarchaeota archaeon isolate SQRJ 82 k255 467457 iso2 MDO8080843.1
Archaea Promethearchaeati Promethearchaeati incertae sedis Candidatus Jordarchaeaceae archaeon MEM3586469.1
Archaea Promethearchaeati Promethearchaeati incertae sedis Candidatus Jordarchaeaceae archaeon MEM2145702.1
Archaea Promethearchaeati Candidatus Odinarchaeota Candidatus Odinarchaeia archaeon MHA1238479.1
Archaea Promethearchaeati Candidatus Odinarchaeota Candidatus Odinarchaeia archaeon MHA1754503.1
Archaea Promethearchaeati Candidatus Odinarchaeota Candidatus Odinarchaeia archaeon MHA1409483.1
Archaea Promethearchaeati Candidatus Odinarchaeota Candidatus Odinarchaeia archaeon MEM2109209.1
Archaea Promethearchaeati Candidatus Odinarchaeota Candidatus Odinarchaeota archaeon isolate J65 bin.29 GCA 016839265.1
Archaea Promethearchaeati Candidatus Odinarchaeota Candidatus Odinarchaeum yellowstonii WEU40722.1
Archaea Promethearchaeati Candidatus Odinarchaeota Candidatus Odinarchaeia archaeon isolate RPA3 GCA 029856525.1
Archaea Promethearchaeati Candidatus Helarchaeota Candidatus Helarchaeota archaeon isolate SRR6823441 bin.196 CONCOCT v1.1 MAG GCA 937881325.1
Archaea Promethearchaeati Candidatus Helarchaeota Candidatus Helarchaeota archaeon MHA1652219.1
Archaea Promethearchaeati Candidatus Helarchaeota Candidatus Helarchaeota archaeon MHA1263812.1
Archaea Promethearchaeati Promethearchaeota Promethearchaeota archaeon MFX1295357.1
Archaea Promethearchaeati Candidatus Helarchaeota Candidatus Helarchaeota archaeon isolate YT 4001 GCA 016840125.1
Archaea Promethearchaeati Promethearchaeota Promethearchaeota archaeon MFX1298631.1
Archaea Promethearchaeati Candidatus Helarchaeota Candidatus Helarchaeota archaeon NVM53638.1
Archaea Promethearchaeati Candidatus Helarchaeota Candidatus Helarchaeota archaeon MHA1276609.1
Archaea Promethearchaeati Candidatus Helarchaeota Candidatus Helarchaeota archaeon NVM29396.1
Archaea Promethearchaeati Candidatus Helarchaeota Candidatus Helarchaeota archaeon MHA1301316.1
Archaea Promethearchaeati Candidatus Helarchaeota Candidatus Helarchaeota archaeon MHA1381049.1
Archaea Promethearchaeati Candidatus Helarchaeota Candidatus Helarchaeota archaeon NVM04234.1
Archaea Promethearchaeati Promethearchaeota Promethearchaeota archaeon MFX1453618.1
Archaea Promethearchaeati Candidatus Helarchaeota Candidatus Helarchaeota archaeon isolate LW55 61 GCA 019058315.1
Archaea Promethearchaeati Promethearchaeota Candidatus Lokiarchaeota archaeon isolate SZ 4 bin10.384 GCA 011365055.1
Archaea Promethearchaeati Candidatus Helarchaeota Candidatus Helarchaeota archaeon isolate MP5 1 211 GCA 016840985.1
Archaea Promethearchaeati Candidatus Helarchaeota Candidatus Helarchaeota archaeon isolate Meg19 1012 Bin 504 GCA 029838215.1
Archaea Promethearchaeati Candidatus Helarchaeota Candidatus Helarchaeota archaeon MHA1404544.1
Archaea Promethearchaeati Candidatus Helarchaeota Candidatus Helarchaeota archaeon MHA1271675.1
Archaea Promethearchaeati Promethearchaeota Promethearchaeota archaeon MBD3230465.1
Archaea Promethearchaeati Candidatus Helarchaeota Candidatus Helarchaeota archaeon isolate Hel GB B GCA 005191425.1
Archaea Promethearchaeati Candidatus Helarchaeota Candidatus Helarchaeota archaeon MHA1232699.1
Archaea Thermoproteati Candidatus Bathyarchaeota Candidatus Bathyarchaeota archaeon RJS89371.1
Archaea Thermoproteati Candidatus Bathyarchaeota Candidatus Bathyarchaeota archaeon MFB0543966.1
Archaea Thermoproteati Candidatus Bathyarchaeota Candidatus Bathyarchaeota archaeon MBS7631354.1
Archaea Thermoproteati Candidatus Bathyarchaeota Candidatus Bathyarchaeota archaeon MGQ9680711.1
Archaea Thermoproteati Candidatus Bathyarchaeota Candidatus Bathyarchaeota archaeon MBS7637905.1
Archaea Thermoproteati Candidatus Bathyarchaeota Candidatus Bathyarchaeota archaeon RLI09612.1
Archaea Thermoproteati Candidatus Bathyarchaeota Candidatus Bathyarchaeota archaeon MCW3992911.1
Archaea Thermoproteati Candidatus Bathyarchaeota Candidatus Bathyarchaeota archaeon MFQ6052694.1
Archaea Thermoproteati Candidatus Bathyarchaeota Candidatus Bathyarchaeota archaeon TET71868.1
Archaea Thermoproteati Candidatus Bathyarchaeota Candidatus Bathyarchaeota archaeon MCW3991855.1
Archaea Thermoproteati Candidatus Bathyarchaeota Candidatus Bathyarchaeota archaeon MGD2201184.1
Archaea Thermoproteati Candidatus Bathyarchaeota Candidatus Bathyarchaeota archaeon MBC8224871.1
Archaea Thermoproteati Candidatus Bathyarchaeota Candidatus Bathyarchaeota archaeon MBQ03840.1
Archaea Thermoproteati Candidatus Bathyarchaeota Candidatus Bathyarchaeota archaeon MDP6457852.1
Archaea Thermoproteati Candidatus Bathyarchaeota Candidatus Bathyarchaeota archaeon MBL7168533.1
Archaea Thermoproteati Candidatus Bathyarchaeota Candidatus Bathyarchaeota archaeon UCD45889.1
Archaea Thermoproteati Candidatus Bathyarchaeota Candidatus Bathyarchaeota archaeon MDH5201118.1
Archaea Thermoproteati Candidatus Bathyarchaeota Candidatus Bathyarchaeota archaeon MCW3979752.1
Archaea Thermoproteati Candidatus Bathyarchaeota Candidatus Bathyarchaeota archaeon MCW4035704.1
Archaea Thermoproteati Candidatus Bathyarchaeota Candidatus Bathyarchaeota archaeon MFB0558424.1
Archaea Thermoproteati Candidatus Bathyarchaeota Candidatus Bathyarchaeota archaeon MCX6643197.1
Archaea Thermoproteati Candidatus Bathyarchaeota Candidatus Bathyarchaeota archaeon MBM3291924.1
Archaea Thermoproteati Candidatus Bathyarchaeota Candidatus Bathyarchaeota archaeon MBN1683007.1
Archaea Thermoproteati Candidatus Bathyarchaeota Candidatus Bathyarchaeia archaeon MFA5869989.1
Archaea Thermoproteati Candidatus Bathyarchaeota Candidatus Bathyarchaeia archaeon MGD0803348.1
Archaea Thermoproteati Candidatus Bathyarchaeota Candidatus Bathyarchaeota archaeon MCX6656906.1
Archaea Thermoproteati Candidatus Bathyarchaeota Candidatus Bathyarchaeota archaeon HIH88896.1
Archaea Thermoproteati Candidatus Bathyarchaeota Candidatus Bathyarchaeota archaeon MCX6648313.1
Archaea Thermoproteati Candidatus Bathyarchaeota Candidatus Bathyarchaeia archaeon MFP3951326.1
Archaea Thermoproteati Candidatus Bathyarchaeota Candidatus Bathyarchaeota archaeon MCK4400506.1
Archaea Thermoproteati Candidatus Bathyarchaeota Candidatus Bathyarchaeota archaeon MBD3205859.1
Archaea Thermoproteati Candidatus Bathyarchaeota Candidatus Bathyarchaeota archaeon MBN2334863.1
Archaea Thermoproteati Candidatus Bathyarchaeota Candidatus Bathyarchaeota archaeon MCW4048386.1
Archaea Thermoproteati Candidatus Bathyarchaeota Candidatus Bathyarchaeota archaeon RBG 16 57 9 OGD44805.1
Archaea Thermoproteati Candidatus Bathyarchaeota Candidatus Bathyarchaeota archaeon MCJ7573045.1
Archaea Thermoproteati Candidatus Bathyarchaeota Candidatus Bathyarchaeota archaeon MCW4013050.1
Archaea Thermoproteati Candidatus Bathyarchaeota Candidatus Bathyarchaeota archaeon TFH16124.1
Archaea Thermoproteati Candidatus Bathyarchaeota Candidatus Bathyarchaeota archaeon TRO53056.1
Archaea Thermoproteati Candidatus Bathyarchaeota Candidatus Bathyarchaeia archaeon MES0326221.1
Archaea Thermoproteati Candidatus Bathyarchaeota Candidatus Bathyarchaeota archaeon MCK5671671.1
Archaea Thermoproteati Candidatus Bathyarchaeota Candidatus Bathyarchaeota archaeon MBT3285664.1
Archaea Thermoproteati Candidatus Bathyarchaeota Candidatus Bathyarchaeota archaeon MCK4952261.1
Archaea Thermoproteati Candidatus Bathyarchaeota Candidatus Bathyarchaeota archaeon MCJ7505248.1
Archaea Thermoproteati Candidatus Bathyarchaeota Candidatus Bathyarchaeota archaeon HKZ93060.1
Archaea Thermoproteati Candidatus Bathyarchaeota Candidatus Bathyarchaeota archaeon MDH5783262.1
Archaea Thermoproteati Candidatus Bathyarchaeota Candidatus Bathyarchaeota archaeon MGD8506765.1
Archaea Thermoproteati Candidatus Bathyarchaeota Candidatus Bathyarchaeia archaeon MFB0514211.1
Archaea Thermoproteati Candidatus Bathyarchaeota Candidatus Bathyarchaeota archaeon UCC28271.1
Archaea Thermoproteati Candidatus Bathyarchaeota Candidatus Bathyarchaeota archaeon MCZ2856121.1
Archaea Thermoproteati Candidatus Bathyarchaeota Candidatus Bathyarchaeota archaeon UCG44989.1
Archaea Thermoproteati Candidatus Bathyarchaeota Candidatus Bathyarchaeota archaeon UCE28581.1
Archaea Thermoproteati Candidatus Bathyarchaeota Candidatus Bathyarchaeota archaeon TEU05186.1
Archaea Thermoproteati Candidatus Bathyarchaeota Candidatus Bathyarchaeota archaeon MDH5450022.1
Archaea Thermoproteati Candidatus Bathyarchaeota Candidatus Bathyarchaeota archaeon MDH5780821.1
Archaea Thermoproteati Candidatus Bathyarchaeota Candidatus Bathyarchaeota archaeon UCE43587.1
Archaea Thermoproteati Candidatus Bathyarchaeota Candidatus Bathyarchaeota archaeon MFQ6064012.1
Archaea Thermoproteati Candidatus Bathyarchaeota Candidatus Bathyarchaeota archaeon MFQ5758463.1
Archaea Thermoproteati Candidatus Bathyarchaeota Candidatus Bathyarchaeota archaeon BA1 KPV64847.1
Archaea Thermoproteati Candidatus Bathyarchaeota Candidatus Bathyarchaeota archaeon MFQ6080496.1
Archaea Thermoproteati Candidatus Bathyarchaeota Candidatus Bathyarchaeota archaeon MGD8543610.1
Archaea Thermoproteati Candidatus Bathyarchaeota Candidatus Bathyarchaeota archaeon RLI45790.1
Archaea Thermoproteati Candidatus Bathyarchaeota Candidatus Bathyarchaeota archaeon UCE16306.1
Archaea Thermoproteati Candidatus Bathyarchaeota Candidatus Bathyarchaeota archaeon MDH5437878.1
Archaea Thermoproteati Candidatus Bathyarchaeota Candidatus Bathyarchaeota archaeon MCK5631356.1
Archaea Thermoproteati Candidatus Bathyarchaeota Candidatus Bathyarchaeota archaeon UCD96569.1
Archaea Thermoproteati Candidatus Bathyarchaeota Candidatus Bathyarchaeia archaeon MGB9135258.1
Archaea Thermoproteati Candidatus Bathyarchaeota Candidatus Bathyarchaeota archaeon MCK4633531.1
Archaea Thermoproteati Candidatus Bathyarchaeota Candidatus Bathyarchaeota archaeon MDH5447647.1
Archaea Thermoproteati Candidatus Bathyarchaeota Candidatus Bathyarchaeota archaeon MCK4476940.1
Archaea Thermoproteati Candidatus Bathyarchaeota Candidatus Bathyarchaeia archaeon MFQ6068381.1
Archaea Thermoproteati Candidatus Bathyarchaeota Candidatus Bathyarchaeota archaeon MCW4052779.1
Archaea Thermoproteati Candidatus Bathyarchaeota Candidatus Bathyarchaeota archaeon UCE95476.1
Archaea Thermoproteati Candidatus Bathyarchaeota Candidatus Bathyarchaeia archaeon MFP3984480.1
Archaea Thermoproteati Candidatus Bathyarchaeota Candidatus Bathyarchaeota archaeon UCB61150.1
Archaea Thermoproteati Candidatus Bathyarchaeota Candidatus Bathyarchaeota archaeon UCG36424.1

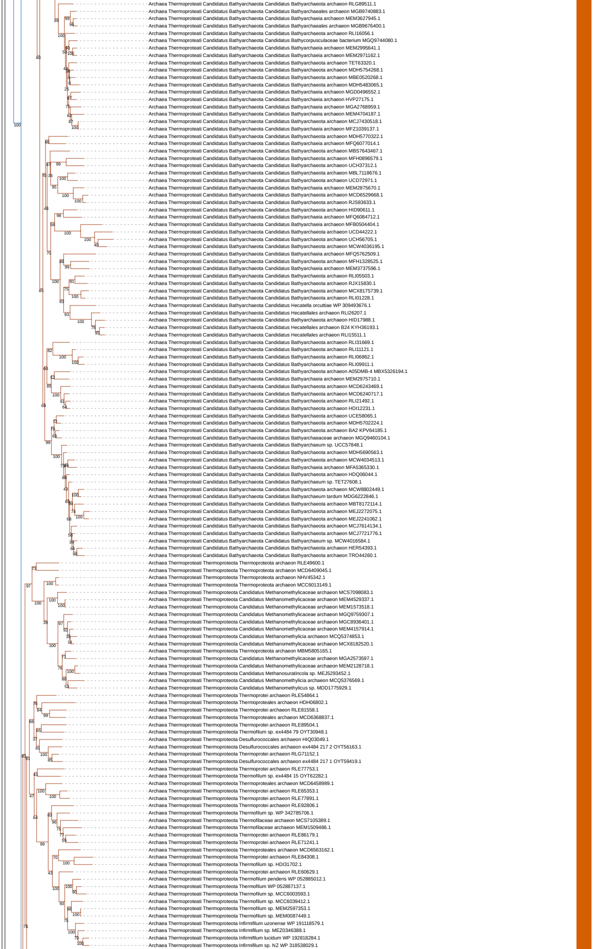
Archaea Thermoproteati Candidatus Bathyarchaeota Candidatus Bathyarchaeota archaeon RLG89511.1
Archaea Thermoproteati Candidatus Bathyarchaeota Candidatus Bathyarchaeales archaeon MGB9740883.1
Archaea Thermoproteati Candidatus Bathyarchaeota Candidatus Bathyarchaeia archaeon MEM3627945.1
Archaea Thermoproteati Candidatus Bathyarchaeota Candidatus Bathyarchaeales archaeon MGB9676400.1
Archaea Thermoproteati Candidatus Bathyarchaeota Candidatus Bathyarchaeota archaeon RLI16056.1
Archaea Thermoproteati Candidatus Bathyarchaeota Candidatus Bathycorpusculaceae bacterium MGQ9744080.1
Archaea Thermoproteati Candidatus Bathyarchaeota Candidatus Bathyarchaeia archaeon MEM2995641.1
Archaea Thermoproteati Candidatus Bathyarchaeota Candidatus Bathyarchaeia archaeon MEM2971162.1
Archaea Thermoproteati Candidatus Bathyarchaeota Candidatus Bathyarchaeota archaeon TET63320.1
Archaea Thermoproteati Candidatus Bathyarchaeota Candidatus Bathyarchaeota archaeon MDH5754268.1
Archaea Thermoproteati Candidatus Bathyarchaeota Candidatus Bathyarchaeota archaeon MBE0520268.1
Archaea Thermoproteati Candidatus Bathyarchaeota Candidatus Bathyarchaeota archaeon MDH5483065.1
Archaea Thermoproteati Candidatus Bathyarchaeota Candidatus Bathyarchaeia archaeon MGD0496552.1
Archaea Thermoproteati Candidatus Bathyarchaeota Candidatus Bathyarchaeia archaeon HVP27175.1
Archaea Thermoproteati Candidatus Bathyarchaeota Candidatus Bathyarchaeia archaeon MGA2768959.1
Archaea Thermoproteati Candidatus Bathyarchaeota Candidatus Bathyarchaeia archaeon MEM4704187.1
Archaea Thermoproteati Candidatus Bathyarchaeota Candidatus Bathyarchaeota archaeon MCJ7430518.1
Archaea Thermoproteati Candidatus Bathyarchaeota Candidatus Bathyarchaeia archaeon MFZ1039137.1
Archaea Thermoproteati Candidatus Bathyarchaeota Candidatus Bathyarchaeota archaeon MDH5770322.1
Archaea Thermoproteati Candidatus Bathyarchaeota Candidatus Bathyarchaeia archaeon MFQ6077014.1
Archaea Thermoproteati Candidatus Bathyarchaeota Candidatus Bathyarchaeota archaeon MBS7643467.1
Archaea Thermoproteati Candidatus Bathyarchaeota Candidatus Bathyarchaeia archaeon MFH0896579.1
Archaea Thermoproteati Candidatus Bathyarchaeota Candidatus Bathyarchaeota archaeon UCH37312.1
Archaea Thermoproteati Candidatus Bathyarchaeota Candidatus Bathyarchaeota archaeon MBL7118676.1
Archaea Thermoproteati Candidatus Bathyarchaeota Candidatus Bathyarchaeota archaeon UCD72971.1
Archaea Thermoproteati Candidatus Bathyarchaeota Candidatus Bathyarchaeia archaeon MEM2875670.1
Archaea Thermoproteati Candidatus Bathyarchaeota Candidatus Bathyarchaeota archaeon MCD6529668.1
Archaea Thermoproteati Candidatus Bathyarchaeota Candidatus Bathyarchaeota archaeon RJS83633.1
Archaea Thermoproteati Candidatus Bathyarchaeota Candidatus Bathyarchaeota archaeon HID90611.1
Archaea Thermoproteati Candidatus Bathyarchaeota Candidatus Bathyarchaeia archaeon MFQ6084712.1
Archaea Thermoproteati Candidatus Bathyarchaeota Candidatus Bathyarchaeia archaeon MFB0504404.1
Archaea Thermoproteati Candidatus Bathyarchaeota Candidatus Bathyarchaeota archaeon UCD44222.1
Archaea Thermoproteati Candidatus Bathyarchaeota Candidatus Bathyarchaeota archaeon UCH56705.1
Archaea Thermoproteati Candidatus Bathyarchaeota Candidatus Bathyarchaeota archaeon MCW4036195.1
Archaea Thermoproteati Candidatus Bathyarchaeota Candidatus Bathyarchaeia archaeon MFQ5762509.1
Archaea Thermoproteati Candidatus Bathyarchaeota Candidatus Bathyarchaeia archaeon MFH1328525.1
Archaea Thermoproteati Candidatus Bathyarchaeota Candidatus Bathyarchaeia archaeon MEM3737596.1
Archaea Thermoproteati Candidatus Bathyarchaeota Candidatus Bathyarchaeota archaeon RLI05503.1
Archaea Thermoproteati Candidatus Bathyarchaeota Candidatus Bathyarchaeota archaeon RJX15830.1
Archaea Thermoproteati Candidatus Bathyarchaeota Candidatus Bathyarchaeota archaeon MCX8175739.1
Archaea Thermoproteati Candidatus Bathyarchaeota Candidatus Bathyarchaeota archaeon RLI01228.1
Archaea Thermoproteati Candidatus Bathyarchaeota Candidatus Hecatella orcuttiae WP 309493676.1
Archaea Thermoproteati Candidatus Bathyarchaeota Candidatus Hecatellales archaeon RLI26207.1
Archaea Thermoproteati Candidatus Bathyarchaeota Candidatus Bathyarchaeota archaeon HID17988.1
Archaea Thermoproteati Candidatus Bathyarchaeota Candidatus Hecatellales archaeon B24 KYH36193.1
Archaea Thermoproteati Candidatus Bathyarchaeota Candidatus Hecatellales archaeon RLI15511.1
Archaea Thermoproteati Candidatus Bathyarchaeota Candidatus Bathyarchaeota archaeon RLI31669.1
Archaea Thermoproteati Candidatus Bathyarchaeota Candidatus Bathyarchaeota archaeon RLI11121.1
Archaea Thermoproteati Candidatus Bathyarchaeota Candidatus Bathyarchaeota archaeon RLI06862.1
Archaea Thermoproteati Candidatus Bathyarchaeota Candidatus Bathyarchaeota archaeon RLI09911.1
Archaea Thermoproteati Candidatus Bathyarchaeota Candidatus Bathyarchaeota archaeon A05DMB-4 MBX5326194.1
Archaea Thermoproteati Candidatus Bathyarchaeota Candidatus Bathyarchaeia archaeon MEM2975710.1
Archaea Thermoproteati Candidatus Bathyarchaeota Candidatus Bathyarchaeota archaeon MCD6243469.1
Archaea Thermoproteati Candidatus Bathyarchaeota Candidatus Bathyarchaeota archaeon MCD6240717.1
Archaea Thermoproteati Candidatus Bathyarchaeota Candidatus Bathyarchaeota archaeon RLI21492.1
Archaea Thermoproteati Candidatus Bathyarchaeota Candidatus Bathyarchaeota archaeon HDI12231.1
Archaea Thermoproteati Candidatus Bathyarchaeota Candidatus Bathyarchaeota archaeon UCE58065.1
Archaea Thermoproteati Candidatus Bathyarchaeota Candidatus Bathyarchaeota archaeon MDH5702224.1
Archaea Thermoproteati Candidatus Bathyarchaeota Candidatus Bathyarchaeota archaeon BA2 KPV64185.1
Archaea Thermoproteati Candidatus Bathyarchaeota Candidatus Bathyarchaeaceae archaeon MGQ9460104.1
Archaea Thermoproteati Candidatus Bathyarchaeota Candidatus Bathyarchaeum sp. UCC57848.1
Archaea Thermoproteati Candidatus Bathyarchaeota Candidatus Bathyarchaeota archaeon MDH5690563.1
Archaea Thermoproteati Candidatus Bathyarchaeota Candidatus Bathyarchaeota archaeon MCW4034513.1
Archaea Thermoproteati Candidatus Bathyarchaeota Candidatus Bathyarchaeota archaeon MFA5365330.1
Archaea Thermoproteati Candidatus Bathyarchaeota Candidatus Bathyarchaeota archaeon HDQ06044.1
Archaea Thermoproteati Candidatus Bathyarchaeota Candidatus Bathyarchaeum sp. TET27608.1
Archaea Thermoproteati Candidatus Bathyarchaeota Candidatus Bathyarchaeota archaeon MCW8802449.1
Archaea Thermoproteati Candidatus Bathyarchaeota Candidatus Bathyarchaeum tardum MDG6222846.1
Archaea Thermoproteati Candidatus Bathyarchaeota Candidatus Bathyarchaeota archaeon MBT8172114.1
Archaea Thermoproteati Candidatus Bathyarchaeota Candidatus Bathyarchaeota archaeon MEJ2272075.1
Archaea Thermoproteati Candidatus Bathyarchaeota Candidatus Bathyarchaeota archaeon MEJ2241062.1
Archaea Thermoproteati Candidatus Bathyarchaeota Candidatus Bathyarchaeota archaeon MCJ7614134.1
Archaea Thermoproteati Candidatus Bathyarchaeota Candidatus Bathyarchaeota archaeon MCJ7721776.1
Archaea Thermoproteati Candidatus Bathyarchaeota Candidatus Bathyarchaeum sp. MCW4016584.1
Archaea Thermoproteati Candidatus Bathyarchaeota Candidatus Bathyarchaeota archaeon HER54393.1
Archaea Thermoproteati Candidatus Bathyarchaeota Candidatus Bathyarchaeota archaeon TRO44260.1
Archaea Thermoproteati Thermoproteota Thermoproteota archaeon RLE49600.1
Archaea Thermoproteati Thermoproteota Thermoproteota archaeon MCD6409045.1
Archaea Thermoproteati Thermoproteota Thermoproteota archaeon NHV45342.1
Archaea Thermoproteati Thermoproteota Thermoproteota archaeon MCC6013149.1
Archaea Thermoproteati Thermoproteota Candidatus Methanomethylicaceae archaeon MCS7098083.1
Archaea Thermoproteati Thermoproteota Candidatus Methanomethylicaceae archaeon MEM4529337.1
Archaea Thermoproteati Thermoproteota Candidatus Methanomethylicaceae archaeon MEM1573518.1
Archaea Thermoproteati Thermoproteota Candidatus Methanomethylicaceae archaeon MGQ9759307.1
Archaea Thermoproteati Thermoproteota Candidatus Methanomethylicaceae archaeon MGC8936401.1
Archaea Thermoproteati Thermoproteota Candidatus Methanomethylicaceae archaeon MEM4157914.1
Archaea Thermoproteati Thermoproteota Candidatus Methanomethylicia archaeon MCQ5374853.1
Archaea Thermoproteati Thermoproteota Candidatus Methanomethylicaceae archaeon MCX8182520.1
Archaea Thermoproteati Thermoproteota Thermoproteota archaeon MBM5805165.1
Archaea Thermoproteati Thermoproteota Candidatus Methanomethylicaceae archaeon MGA2573597.1
Archaea Thermoproteati Thermoproteota Candidatus Methanomethylicaceae archaeon MEM2128718.1
Archaea Thermoproteati Thermoproteota Candidatus Methanosuratincola sp. MEJ5293452.1
Archaea Thermoproteati Thermoproteota Candidatus Methanomethylicia archaeon MCQ5376569.1
Archaea Thermoproteati Thermoproteota Candidatus Methanomethylicus sp. MDD1775929.1
Archaea Thermoproteati Thermoproteota Thermoprotei archaeon RLE54864.1
Archaea Thermoproteati Thermoproteota Thermoproteales archaeon HDH06802.1
Archaea Thermoproteati Thermoproteota Thermoprotei archaeon RLE81558.1
Archaea Thermoproteati Thermoproteota Thermoproteales archaeon MCD6368837.1
Archaea Thermoproteati Thermoproteota Thermoprotei archaeon RLE89504.1
Archaea Thermoproteati Thermoproteota Thermofilum sp. ex4484 79 OYT30948.1
Archaea Thermoproteati Thermoproteota Desulfurococcales archaeon HIQ03049.1
Archaea Thermoproteati Thermoproteota Desulfurococcales archaeon ex4484 217 2 OYT56163.1
Archaea Thermoproteati Thermoproteota Thermoprotei archaeon RLG71152.1
Archaea Thermoproteati Thermoproteota Desulfurococcales archaeon ex4484 217 1 OYT59419.1
Archaea Thermoproteati Thermoproteota Thermoprotei archaeon RLE77753.1
Archaea Thermoproteati Thermoproteota Thermofilum sp. ex4484 15 OYT62282.1
Archaea Thermoproteati Thermoproteota Thermoproteales archaeon MCD6458989.1
Archaea Thermoproteati Thermoproteota Thermoprotei archaeon RLE65353.1
Archaea Thermoproteati Thermoproteota Thermoprotei archaeon RLE77891.1
Archaea Thermoproteati Thermoproteota Thermoprotei archaeon RLE92806.1
Archaea Thermoproteati Thermoproteota Thermofilum sp. WP 342785706.1
Archaea Thermoproteati Thermoproteota Thermofilaceae archaeon MCS7105389.1
Archaea Thermoproteati Thermoproteota Thermofilaceae archaeon MEM1509486.1
Archaea Thermoproteati Thermoproteota Thermoprotei archaeon RLE86179.1
Archaea Thermoproteati Thermoproteota Thermoprotei archaeon RLE71241.1
Archaea Thermoproteati Thermoproteota Thermoproteales archaeon MCD6563162.1
Archaea Thermoproteati Thermoproteota Thermoprotei archaeon RLE84308.1
Archaea Thermoproteati Thermoproteota Thermofilum sp. HDI31702.1
Archaea Thermoproteati Thermoproteota Thermoprotei archaeon RLE60629.1
Archaea Thermoproteati Thermoproteota Thermofilum pendens WP 052885012.1
Archaea Thermoproteati Thermoproteota Thermofilum WP 052887137.1
Archaea Thermoproteati Thermoproteota Thermofilum sp. MCC6003593.1
Archaea Thermoproteati Thermoproteota Thermofilum sp. MCC6039412.1
Archaea Thermoproteati Thermoproteota Thermofilum sp. MEM2597353.1
Archaea Thermoproteati Thermoproteota Thermofilum sp. MEM0087449.1
Archaea Thermoproteati Thermoproteota Infirmifilum uzonense WP 191118579.1
Archaea Thermoproteati Thermoproteota Infirmifilum sp. MEZ0346388.1
Archaea Thermoproteati Thermoproteota Infirmifilum lucidum WP 192818284.1
Archaea Thermoproteati Thermoproteota Infirmifilum sp. NZ WP 318538029.1

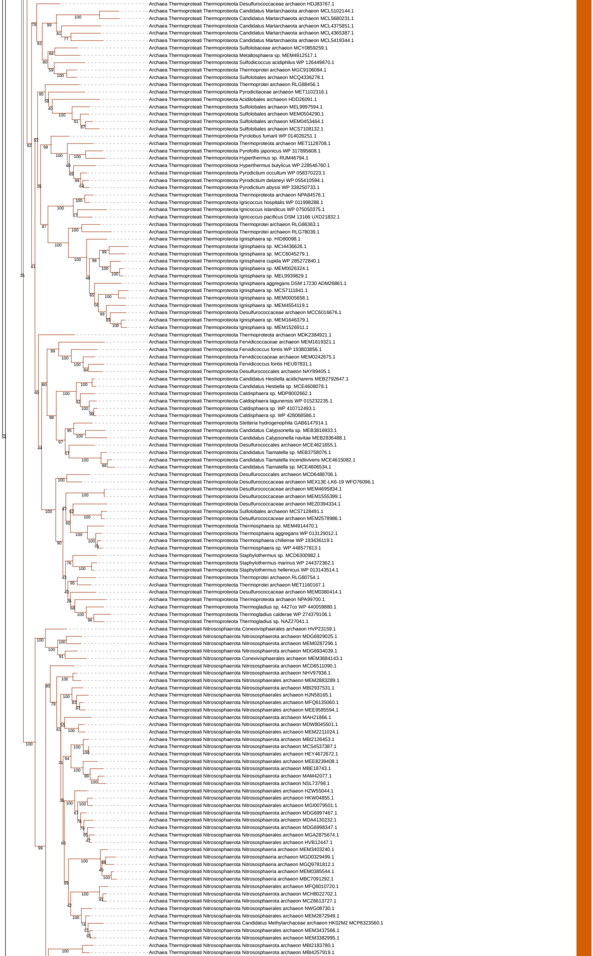
Archaea Thermoproteati Thermoproteota Desulfurococcaceae archaeon HDJ83767.1
Archaea Thermoproteati Thermoproteota Candidatus Martarchaeota archaeon MCL5102144.1
Archaea Thermoproteati Thermoproteota Candidatus Martarchaeota archaeon MCL5680231.1
Archaea Thermoproteati Thermoproteota Candidatus Martarchaeota archaeon MCL4375851.1
Archaea Thermoproteati Thermoproteota Candidatus Martarchaeota archaeon MCL4365387.1
Archaea Thermoproteati Thermoproteota Candidatus Martarchaeota archaeon MCL5419344.1
Archaea Thermoproteati Thermoproteota Sulfolobaceae archaeon MCY0859259.1
Archaea Thermoproteati Thermoproteota Metallosphaera sp. MEM4912517.1
Archaea Thermoproteati Thermoproteota Sulfodiicoccus acidiphilus WP 126449870.1
Archaea Thermoproteati Thermoproteota Thermoprotei archaeon MGC9106084.1
Archaea Thermoproteati Thermoproteota Sulfolobales archaeon MCQ4336278.1
Archaea Thermoproteati Thermoproteota Thermoprotei archaeon RLG88456.1
Archaea Thermoproteati Thermoproteota Pyrodictiaceae archaeon MET1102316.1
Archaea Thermoproteati Thermoproteota Acidilobales archaeon HDD26091.1
Archaea Thermoproteati Thermoproteota Sulfolobales archaeon MEL9997594.1
Archaea Thermoproteati Thermoproteota Sulfolobales archaeon MEM0504290.1
Archaea Thermoproteati Thermoproteota Sulfolobales archaeon MEM0453464.1
Archaea Thermoproteati Thermoproteota Sulfolobales archaeon MCS7108132.1
Archaea Thermoproteati Thermoproteota Pyrolobus fumarii WP 014026251.1
Archaea Thermoproteati Thermoproteota Thermoproteota archaeon MET1128708.1
Archaea Thermoproteati Thermoproteota Pyrofollis japonicus WP 317895608.1
Archaea Thermoproteati Thermoproteota Hyperthermus sp. RUM46794.1
Archaea Thermoproteati Thermoproteota Hyperthermus butylicus WP 228546760.1
Archaea Thermoproteati Thermoproteota Pyrodictium occultum WP 058370223.1
Archaea Thermoproteati Thermoproteota Pyrodictium delaneyi WP 055410594.1
Archaea Thermoproteati Thermoproteota Pyrodictium abyssi WP 338250733.1
Archaea Thermoproteati Thermoproteota Thermoproteota archaeon NPA84576.1
Archaea Thermoproteati Thermoproteota Ignicoccus hospitalis WP 011998288.1
Archaea Thermoproteati Thermoproteota Ignicoccus islandicus WP 075050375.1
Archaea Thermoproteati Thermoproteota Ignicoccus pacificus DSM 13166 UXD21832.1
Archaea Thermoproteati Thermoproteota Thermoprotei archaeon RLG86363.1
Archaea Thermoproteati Thermoproteota Thermoprotei archaeon RLG78039.1
Archaea Thermoproteati Thermoproteota Ignisphaera sp. HID80098.1
Archaea Thermoproteati Thermoproteota Ignisphaera sp. MCI4436626.1
Archaea Thermoproteati Thermoproteota Ignisphaera sp. MCC6045279.1
Archaea Thermoproteati Thermoproteota Ignisphaera cupida WP 285272840.1
Archaea Thermoproteati Thermoproteota Ignisphaera sp. MEM0026324.1
Archaea Thermoproteati Thermoproteota Ignisphaera sp. MEL9939829.1
Archaea Thermoproteati Thermoproteota Ignisphaera aggregans DSM 17230 ADM26861.1
Archaea Thermoproteati Thermoproteota Ignisphaera sp. MCS7111841.1
Archaea Thermoproteati Thermoproteota Ignisphaera sp. MEM0005658.1
Archaea Thermoproteati Thermoproteota Ignisphaera sp. MEM4554119.1
Archaea Thermoproteati Thermoproteota Desulfurococcaceae archaeon MCC6016676.1
Archaea Thermoproteati Thermoproteota Ignisphaera sp. MEM1646379.1
Archaea Thermoproteati Thermoproteota Ignisphaera sp. MEM1526911.1
Archaea Thermoproteati Thermoproteota Thermoproteota archaeon MDK2384921.1
Archaea Thermoproteati Thermoproteota Fervidicoccaceae archaeon MEM1619321.1
Archaea Thermoproteati Thermoproteota Fervidicoccus fontis WP 193803856.1
Archaea Thermoproteati Thermoproteota Fervidicoccaceae archaeon MEM0242675.1
Archaea Thermoproteati Thermoproteota Fervidicoccus fontis HEU97831.1
Archaea Thermoproteati Thermoproteota Desulfurococcales archaeon NAY89405.1
Archaea Thermoproteati Thermoproteota Candidatus Hestiella acidicharens MEB2792647.1
Archaea Thermoproteati Thermoproteota Candidatus Hestiella sp. MCE4608078.1
Archaea Thermoproteati Thermoproteota Caldisphaera sp. MDP8002662.1
Archaea Thermoproteati Thermoproteota Caldisphaera lagunensis WP 015232235.1
Archaea Thermoproteati Thermoproteota Caldisphaera sp. WP 410712493.1
Archaea Thermoproteati Thermoproteota Caldisphaera sp. WP 428068586.1
Archaea Thermoproteati Thermoproteota Stetteria hydrogenophila GAB6147914.1
Archaea Thermoproteati Thermoproteota Candidatus Calypsonella sp. MEB3816933.1
Archaea Thermoproteati Thermoproteota Candidatus Calypsonella navitae MEB2836488.1
Archaea Thermoproteati Thermoproteota Desulfurococcales archaeon MCE4621655.1
Archaea Thermoproteati Thermoproteota Candidatus Tiamatella sp. MEB3758076.1
Archaea Thermoproteati Thermoproteota Candidatus Tiamatella incendiivivens MCE4615082.1
Archaea Thermoproteati Thermoproteota Candidatus Tiamatella sp. MCE4606534.1
Archaea Thermoproteati Thermoproteota Desulfurococcales archaeon MCD6488706.1
Archaea Thermoproteati Thermoproteota Desulfurococcaceae archaeon MEX13E-LK6-19 WFO76096.1
Archaea Thermoproteati Thermoproteota Desulfurococcaceae archaeon MEM4695834.1
Archaea Thermoproteati Thermoproteota Desulfurococcaceae archaeon MEM1555399.1
Archaea Thermoproteati Thermoproteota Desulfurococcaceae archaeon MEZ0394334.1
Archaea Thermoproteati Thermoproteota Sulfolobales archaeon MCS7128491.1
Archaea Thermoproteati Thermoproteota Desulfurococcaceae archaeon MEM2578986.1
Archaea Thermoproteati Thermoproteota Thermosphaera sp. MEM4914470.1
Archaea Thermoproteati Thermoproteota Thermosphaera aggregans WP 013129012.1
Archaea Thermoproteati Thermoproteota Thermosphaera chiliense WP 193436119.1
Archaea Thermoproteati Thermoproteota Thermosphaera sp. WP 448577813.1
Archaea Thermoproteati Thermoproteota Staphylothermus sp. MCD6300982.1
Archaea Thermoproteati Thermoproteota Staphylothermus marinus WP 244372362.1
Archaea Thermoproteati Thermoproteota Staphylothermus hellenicus WP 013143514.1
Archaea Thermoproteati Thermoproteota Thermoprotei archaeon RLG80754.1
Archaea Thermoproteati Thermoproteota Thermoprotei archaeon MET1160167.1
Archaea Thermoproteati Thermoproteota Desulfurococcaceae archaeon MEM0380414.1
Archaea Thermoproteati Thermoproteota Thermoproteota archaeon NPA99700.1
Archaea Thermoproteati Thermoproteota Thermogladius sp. 4427co WP 440059880.1
Archaea Thermoproteati Thermoproteota Thermogladius calderae WP 274379106.1
Archaea Thermoproteati Thermoproteota Thermogladius sp. NAZ27041.1
Archaea Thermoproteati Nitrososphaerota Conexivisphaerales archaeon HVP23159.1
Archaea Thermoproteati Nitrososphaerota Nitrososphaerota archaeon MDG6929025.1
Archaea Thermoproteati Nitrososphaerota Nitrososphaerota archaeon MEM0287296.1
Archaea Thermoproteati Nitrososphaerota Nitrososphaerota archaeon MDG6934039.1
Archaea Thermoproteati Nitrososphaerota Conexivisphaerales archaeon MEM3684143.1
Archaea Thermoproteati Nitrososphaerota Nitrososphaerota archaeon MCD6511090.1
Archaea Thermoproteati Nitrososphaerota Nitrososphaerota archaeon NHV97936.1
Archaea Thermoproteati Nitrososphaerota Nitrososphaerales archaeon MEM2883289.1
Archaea Thermoproteati Nitrososphaerota Nitrososphaerota archaeon MBI2937531.1
Archaea Thermoproteati Nitrososphaerota Nitrososphaerales archaeon HJN58165.1
Archaea Thermoproteati Nitrososphaerota Nitrososphaerales archaeon MFQ6135060.1
Archaea Thermoproteati Nitrososphaerota Nitrososphaerales archaeon MEE9585594.1
Archaea Thermoproteati Nitrososphaerota Nitrososphaerota archaeon MAH21866.1
Archaea Thermoproteati Nitrososphaerota Nitrososphaerales archaeon MDW8045501.1
Archaea Thermoproteati Nitrososphaerota Nitrososphaerales archaeon MEM2211024.1
Archaea Thermoproteati Nitrososphaerota Nitrososphaerota archaeon MBI2126453.1
Archaea Thermoproteati Nitrososphaerota Nitrososphaerota archaeon MCS4537387.1
Archaea Thermoproteati Nitrososphaerota Nitrososphaerales archaeon HEY4672672.1
Archaea Thermoproteati Nitrososphaerota Nitrososphaerales archaeon MEE8239408.1
Archaea Thermoproteati Nitrososphaerota Nitrososphaerota archaeon MBE18743.1
Archaea Thermoproteati Nitrososphaerota Nitrososphaerota archaeon MAM42077.1
Archaea Thermoproteati Nitrososphaerota Nitrososphaerota archaeon NSL73798.1
Archaea Thermoproteati Nitrososphaerota Nitrososphaerales archaeon HZW55044.1
Archaea Thermoproteati Nitrososphaerota Nitrososphaerales archaeon HKW04855.1
Archaea Thermoproteati Nitrososphaerota Nitrososphaerales archaeon MGI0079501.1
Archaea Thermoproteati Nitrososphaerota Nitrososphaerota archaeon MDG6997467.1
Archaea Thermoproteati Nitrososphaerota Nitrososphaerota archaeon MDA4130232.1
Archaea Thermoproteati Nitrososphaerota Nitrososphaerota archaeon MDG6998347.1
Archaea Thermoproteati Nitrososphaerota Nitrososphaerales archaeon MGA2875674.1
Archaea Thermoproteati Nitrososphaerota Nitrososphaerales archaeon HVB12447.1
Archaea Thermoproteati Nitrososphaerota Nitrososphaeria archaeon MEM3403240.1
Archaea Thermoproteati Nitrososphaerota Nitrososphaeria archaeon MGD0329499.1
Archaea Thermoproteati Nitrososphaerota Nitrososphaeria archaeon MGQ9781812.1
Archaea Thermoproteati Nitrososphaerota Nitrososphaeria archaeon MEM0385544.1
Archaea Thermoproteati Nitrososphaerota Nitrososphaeria archaeon MBC7091292.1
Archaea Thermoproteati Nitrososphaerota Nitrososphaerales archaeon MFQ6010720.1
Archaea Thermoproteati Nitrososphaerota Nitrososphaerota archaeon MCH8022702.1
Archaea Thermoproteati Nitrososphaerota Nitrososphaerota archaeon MCZ6613727.1
Archaea Thermoproteati Nitrososphaerota Nitrososphaerales archaeon NWG08730.1
Archaea Thermoproteati Nitrososphaerota Nitrososphaerales archaeon MEM2872949.1
Archaea Thermoproteati Nitrososphaerota Candidatus Methylarchaceae archaeon HK02M2 MCP8323560.1
Archaea Thermoproteati Nitrososphaerota Nitrososphaerales archaeon MEM3437566.1
Archaea Thermoproteati Nitrososphaerota Nitrososphaerales archaeon MEM3382995.1
Archaea Thermoproteati Nitrososphaerota Nitrososphaerota archaeon MBI2183780.1
Archaea Thermoproteati Nitrososphaerota Nitrososphaerota archaeon MBI4257919.1

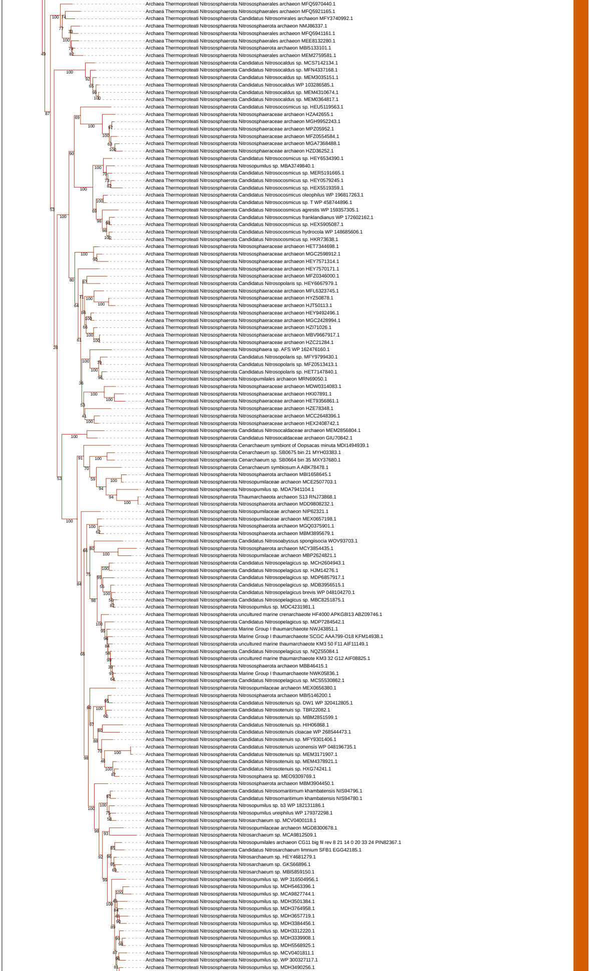

Archaea Thermoproteati Nitrososphaerota Nitrososphaerales archaeon MFQ5970440.1
Archaea Thermoproteati Nitrososphaerota Nitrososphaerales archaeon MFQ5921165.1
Archaea Thermoproteati Nitrososphaerota Candidatus Nitrosomirales archaeon MFY3740992.1
Archaea Thermoproteati Nitrososphaerota Nitrososphaerota archaeon NMJ86337.1
Archaea Thermoproteati Nitrososphaerota Nitrososphaerales archaeon MFQ5941161.1
Archaea Thermoproteati Nitrososphaerota Nitrososphaerales archaeon MEE8132280.1
Archaea Thermoproteati Nitrososphaerota Nitrososphaerota archaeon MBI5133101.1
Archaea Thermoproteati Nitrososphaerota Nitrososphaerales archaeon MEM2759581.1
Archaea Thermoproteati Nitrososphaerota Candidatus Nitrosocaldus sp. MCS7142134.1
Archaea Thermoproteati Nitrososphaerota Candidatus Nitrosocaldus sp. MFN4337168.1
Archaea Thermoproteati Nitrososphaerota Candidatus Nitrosocaldus sp. MEM3035151.1
Archaea Thermoproteati Nitrososphaerota Candidatus Nitrosocaldus WP 103286585.1
Archaea Thermoproteati Nitrososphaerota Candidatus Nitrosocaldus sp. MEM4310674.1
Archaea Thermoproteati Nitrososphaerota Candidatus Nitrosocaldus sp. MEM0364817.1
Archaea Thermoproteati Nitrososphaerota Candidatus Nitrosocosmicus sp. HEU5119563.1
Archaea Thermoproteati Nitrososphaerota Nitrososphaeraceae archaeon HZA42655.1
Archaea Thermoproteati Nitrososphaerota Nitrososphaeraceae archaeon MGH9952243.1
Archaea Thermoproteati Nitrososphaerota Nitrososphaeraceae archaeon MPZ05952.1
Archaea Thermoproteati Nitrososphaerota Nitrososphaeraceae archaeon MFZ0554584.1
Archaea Thermoproteati Nitrososphaerota Nitrososphaeraceae archaeon MGA7368488.1
Archaea Thermoproteati Nitrososphaerota Nitrososphaeraceae archaeon HZD36252.1
Archaea Thermoproteati Nitrososphaerota Candidatus Nitrosocosmicus sp. HEY6534390.1
Archaea Thermoproteati Nitrososphaerota Nitrosopumilus sp. MBA3749840.1
Archaea Thermoproteati Nitrososphaerota Candidatus Nitrosocosmicus sp. MER5191665.1
Archaea Thermoproteati Nitrososphaerota Candidatus Nitrosocosmicus sp. HEY0579245.1
Archaea Thermoproteati Nitrososphaerota Candidatus Nitrosocosmicus sp. HEX5519359.1
Archaea Thermoproteati Nitrososphaerota Candidatus Nitrosocosmicus oleophilus WP 196817263.1
Archaea Thermoproteati Nitrososphaerota Candidatus Nitrosocosmicus sp. T WP 458744896.1
Archaea Thermoproteati Nitrososphaerota Candidatus Nitrosocosmicus agrestis WP 159357305.1
Archaea Thermoproteati Nitrososphaerota Candidatus Nitrosocosmicus franklandianus WP 172602162.1
Archaea Thermoproteati Nitrososphaerota Candidatus Nitrosocosmicus sp. HEX5905087.1
Archaea Thermoproteati Nitrososphaerota Candidatus Nitrosocosmicus hydrocola WP 148685606.1
Archaea Thermoproteati Nitrososphaerota Candidatus Nitrosocosmicus sp. HKR73638.1
Archaea Thermoproteati Nitrososphaerota Nitrososphaeraceae archaeon HET7344698.1
Archaea Thermoproteati Nitrososphaerota Nitrososphaeraceae archaeon MGC2598912.1
Archaea Thermoproteati Nitrososphaerota Nitrososphaeraceae archaeon HEY7571314.1
Archaea Thermoproteati Nitrososphaerota Nitrososphaeraceae archaeon HEY7570171.1
Archaea Thermoproteati Nitrososphaerota Nitrososphaeraceae archaeon MFZ0346000.1
Archaea Thermoproteati Nitrososphaerota Candidatus Nitrosopolaris sp. HEY6667979.1
Archaea Thermoproteati Nitrososphaerota Nitrososphaeraceae archaeon MFL6323745.1
Archaea Thermoproteati Nitrososphaerota Nitrososphaeraceae archaeon HYZ50878.1
Archaea Thermoproteati Nitrososphaerota Nitrososphaeraceae archaeon HJT50113.1
Archaea Thermoproteati Nitrososphaerota Nitrososphaeraceae archaeon HEY9492496.1
Archaea Thermoproteati Nitrososphaerota Nitrososphaeraceae archaeon MGC2428994.1
Archaea Thermoproteati Nitrososphaerota Nitrososphaeraceae archaeon HZI71026.1
Archaea Thermoproteati Nitrososphaerota Nitrososphaeraceae archaeon MBV9667917.1
Archaea Thermoproteati Nitrososphaerota Nitrososphaeraceae archaeon HZC21284.1
Archaea Thermoproteati Nitrososphaerota Nitrososphaera sp. AFS WP 162476160.1
Archaea Thermoproteati Nitrososphaerota Candidatus Nitrosopolaris sp. MFY9799430.1
Archaea Thermoproteati Nitrososphaerota Candidatus Nitrosopolaris sp. MFZ0513413.1
Archaea Thermoproteati Nitrososphaerota Candidatus Nitrosopolaris sp. HET7147840.1
Archaea Thermoproteati Nitrososphaerota Nitrosopumilales archaeon MRN69050.1
Archaea Thermoproteati Nitrososphaerota Nitrososphaeraceae archaeon MDW0314083.1
Archaea Thermoproteati Nitrososphaerota Nitrososphaeraceae archaeon HKI07891.1
Archaea Thermoproteati Nitrososphaerota Nitrososphaeraceae archaeon HET9356861.1
Archaea Thermoproteati Nitrososphaerota Nitrososphaeraceae archaeon HZE78348.1
Archaea Thermoproteati Nitrososphaerota Nitrososphaeraceae archaeon MCC2648396.1
Archaea Thermoproteati Nitrososphaerota Nitrososphaeraceae archaeon HEX2408742.1
Archaea Thermoproteati Nitrososphaerota Candidatus Nitrosocaldaceae archaeon MEM2856804.1
Archaea Thermoproteati Nitrososphaerota Candidatus Nitrosocaldaceae archaeon GIU70842.1
Archaea Thermoproteati Nitrososphaerota Cenarchaeum symbiont of Oopsacas minuta MDI1494939.1
Archaea Thermoproteati Nitrososphaerota Cenarchaeum sp. SB0675 bin 21 MYH03383.1
Archaea Thermoproteati Nitrososphaerota Cenarchaeum sp. SB0664 bin 35 MXY37680.1
Archaea Thermoproteati Nitrososphaerota Cenarchaeum symbiosum A ABK78478.1
Archaea Thermoproteati Nitrososphaerota Nitrososphaerota archaeon MBI1658645.1
Archaea Thermoproteati Nitrososphaerota Nitrosopumilaceae archaeon MCE2507703.1
Archaea Thermoproteati Nitrososphaerota Nitrosopumilus sp. MDA7941104.1
Archaea Thermoproteati Nitrososphaerota Thaumarchaeota archaeon S13 RNJ73868.1
Archaea Thermoproteati Nitrososphaerota Nitrososphaerota archaeon MDD9808232.1
Archaea Thermoproteati Nitrososphaerota Nitrosopumilaceae archaeon NIP62321.1
Archaea Thermoproteati Nitrososphaerota Nitrosopumilaceae archaeon MEX0657198.1
Archaea Thermoproteati Nitrososphaerota Nitrososphaerota archaeon MGQ0375901.1
Archaea Thermoproteati Nitrososphaerota Nitrososphaerota archaeon MBM3895679.1
Archaea Thermoproteati Nitrososphaerota Candidatus Nitrosoabyssus spongiisocia WOV93703.1
Archaea Thermoproteati Nitrososphaerota Nitrososphaerota archaeon MCY3854435.1
Archaea Thermoproteati Nitrososphaerota Nitrosopumilaceae archaeon MBP2624821.1
Archaea Thermoproteati Nitrososphaerota Candidatus Nitrosopelagicus sp. MCH2604943.1
Archaea Thermoproteati Nitrososphaerota Candidatus Nitrosopelagicus sp. HJM14276.1
Archaea Thermoproteati Nitrososphaerota Candidatus Nitrosopelagicus sp. MDP6857917.1
Archaea Thermoproteati Nitrososphaerota Candidatus Nitrosopelagicus sp. MDB3956515.1
Archaea Thermoproteati Nitrososphaerota Candidatus Nitrosopelagicus brevis WP 048104270.1
Archaea Thermoproteati Nitrososphaerota Candidatus Nitrosopelagicus sp. MBC8251875.1
Archaea Thermoproteati Nitrososphaerota Nitrosopumilus sp. MDC4231981.1
Archaea Thermoproteati Nitrososphaerota uncultured marine crenarchaeote HF4000 APKG8I13 ABZ09746.1
Archaea Thermoproteati Nitrososphaerota Candidatus Nitrosopelagicus sp. MDP7284542.1
Archaea Thermoproteati Nitrososphaerota Marine Group I thaumarchaeote NWJ43851.1
Archaea Thermoproteati Nitrososphaerota Marine Group I thaumarchaeote SCGC AAA799-O18 KFM14938.1
Archaea Thermoproteati Nitrososphaerota uncultured marine thaumarchaeote KM3 50 F11 AIF11149.1
Archaea Thermoproteati Nitrososphaerota Candidatus Nitrosopelagicus sp. NQZ55084.1
Archaea Thermoproteati Nitrososphaerota uncultured marine thaumarchaeote KM3 32 G12 AIF08825.1
Archaea Thermoproteati Nitrososphaerota Nitrososphaerota archaeon MBB46415.1
Archaea Thermoproteati Nitrososphaerota Marine Group I thaumarchaeote NWK05836.1
Archaea Thermoproteati Nitrososphaerota Candidatus Nitrosopelagicus sp. MCS5530862.1
Archaea Thermoproteati Nitrososphaerota Nitrosopumilaceae archaeon MEX0656380.1
Archaea Thermoproteati Nitrososphaerota Nitrososphaerota archaeon MBI5146200.1
Archaea Thermoproteati Nitrososphaerota Candidatus Nitrosotenuis sp. DW1 WP 320412805.1
Archaea Thermoproteati Nitrososphaerota Candidatus Nitrosotenuis sp. TBR22082.1
Archaea Thermoproteati Nitrososphaerota Candidatus Nitrosotenuis sp. MBM2851599.1
Archaea Thermoproteati Nitrososphaerota Candidatus Nitrosotenuis sp. HIH06868.1
Archaea Thermoproteati Nitrososphaerota Candidatus Nitrosotenuis cloacae WP 268544473.1
Archaea Thermoproteati Nitrososphaerota Candidatus Nitrosotenuis sp. MFY9301406.1
Archaea Thermoproteati Nitrososphaerota Candidatus Nitrosotenuis uzonensis WP 048196735.1
Archaea Thermoproteati Nitrososphaerota Candidatus Nitrosotenuis sp. MEM3171907.1
Archaea Thermoproteati Nitrososphaerota Candidatus Nitrosotenuis sp. MEM4378921.1
Archaea Thermoproteati Nitrososphaerota Candidatus Nitrosotenuis sp. HXG74241.1
Archaea Thermoproteati Nitrososphaerota Nitrososphaera sp. MEO9309769.1
Archaea Thermoproteati Nitrososphaerota Nitrososphaerota archaeon MBM3904450.1
Archaea Thermoproteati Nitrososphaerota Candidatus Nitrosomaritimum khambatensis NIS94796.1
Archaea Thermoproteati Nitrososphaerota Candidatus Nitrosomaritimum khambatensis NIS94780.1
Archaea Thermoproteati Nitrososphaerota Nitrosopumilus sp. b3 WP 182131186.1
Archaea Thermoproteati Nitrososphaerota Nitrosopumilus ureiphilus WP 179372298.1
Archaea Thermoproteati Nitrososphaerota Nitrosarchaeum sp. MCV0400118.1
Archaea Thermoproteati Nitrososphaerota Nitrosopumilaceae archaeon MGD8300678.1
Archaea Thermoproteati Nitrososphaerota Nitrosarchaeum sp. MCA9812509.1
Archaea Thermoproteati Nitrososphaerota Nitrosopumilales archaeon CG11 big fil rev 8 21 14 0 20 33 24 PIN82367.1
Archaea Thermoproteati Nitrososphaerota Candidatus Nitrosarchaeum limnium SFB1 EGG42185.1
Archaea Thermoproteati Nitrososphaerota Nitrosarchaeum sp. HEY4681279.1
Archaea Thermoproteati Nitrososphaerota Nitrosarchaeum sp. GKS66896.1
Archaea Thermoproteati Nitrososphaerota Nitrosarchaeum sp. MBI5859150.1
Archaea Thermoproteati Nitrososphaerota Nitrosopumilus sp. WP 316504956.1
Archaea Thermoproteati Nitrososphaerota Nitrosopumilus sp. MDH5463396.1
Archaea Thermoproteati Nitrososphaerota Nitrosopumilus sp. MCA9827744.1
Archaea Thermoproteati Nitrososphaerota Nitrosopumilus sp. MDH3501384.1
Archaea Thermoproteati Nitrososphaerota Nitrosopumilus sp. MDH3764958.1
Archaea Thermoproteati Nitrososphaerota Nitrosopumilus sp. MDH3657719.1
Archaea Thermoproteati Nitrososphaerota Nitrosopumilus sp. MDH3384456.1
Archaea Thermoproteati Nitrososphaerota Nitrosopumilus sp. MDH3312220.1
Archaea Thermoproteati Nitrososphaerota Nitrosopumilus sp. MDH3339908.1
Archaea Thermoproteati Nitrososphaerota Nitrosopumilus sp. MDH5568925.1
Archaea Thermoproteati Nitrososphaerota Nitrosopumilus sp. MCV0401811.1
Archaea Thermoproteati Nitrososphaerota Nitrosopumilus sp. WP 300327117.1
Archaea Thermoproteati Nitrososphaerota Nitrosopumilus sp. MDH3490256.1

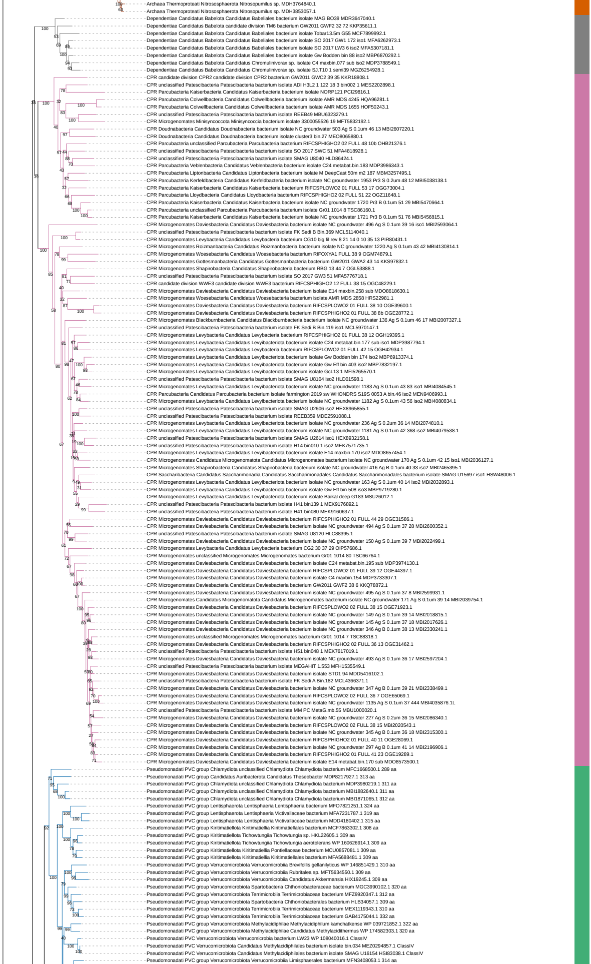
Archaea Thermoproteati Nitrososphaerota Nitrosopumilus sp. MDH3764840.1
Archaea Thermoproteati Nitrososphaerota Nitrosopumilus sp. MDH3853057.1
Dependentiae Candidatus Babelota Candidatus Babeliales bacterium isolate MAG BO39 MDR3647040.1
Dependentiae Candidatus Babelota candidate division TM6 bacterium GW2011 GWF2 32 72 KKP35611.1
Dependentiae Candidatus Babelota Candidatus Babeliales bacterium isolate Tobar13.5m G55 MCF7899992.1
Dependentiae Candidatus Babelota Candidatus Babeliales bacterium isolate SO 2017 GW1 172 iso1 MFA6262973.1
Dependentiae Candidatus Babelota Candidatus Babeliales bacterium isolate SO 2017 LW3 6 iso2 MFA5307181.1
Dependentiae Candidatus Babelota Candidatus Babeliales bacterium isolate Gw Bodden bin 88 iso2 MBP6870292.1
Dependentiae Candidatus Babelota Candidatus Chromulinivorax sp. isolate C4 maxbin.077 sub iso2 MDP3788549.1
Dependentiae Candidatus Babelota Candidatus Chromulinivorax sp. isolate SJ.T10 1 semi39 MGZ6254928.1
CPR candidate division CPR2 candidate division CPR2 bacterium GW2011 GWC2 39 35 KKR18808.1
CPR unclassified Patescibacteria Patescibacteria bacterium isolate ADI H3L2 1 122 18 3 bin002 1 MES2202898.1
CPR Parcubacteria Kaiserbacteria Candidatus Kaiserbacteria bacterium isolate NORP121 PCI29816.1
CPR Parcubacteria Colwellbacteria Candidatus Colwellbacteria bacterium isolate AMR MDS 4245 HQA96281.1
CPR Parcubacteria Colwellbacteria Candidatus Colwellbacteria bacterium isolate AMR MDS 1655 HOF50243.1
CPR unclassified Patescibacteria Patescibacteria bacterium isolate REEB49 MBU6323279.1
CPR Microgenomates Minisyncoccota Minisyncoccia bacterium isolate 3300055526 19 MFT5832192.1
CPR Doudnabacteria Candidatus Doudnabacteria bacterium isolate NC groundwater 503 Ag S 0.1um 46 13 MBI2607220.1
CPR Doudnabacteria Candidatus Doudnabacteria bacterium isolate cluster3 bin.27 MEO8065880.1
CPR Parcubacteria unclassified Parcubacteria Parcubacteria bacterium RIFCSPHIGHO2 02 FULL 48 10b OHB21376.1
CPR unclassified Patescibacteria Patescibacteria bacterium isolate SO 2017 SWC 51 MFA4818928.1
CPR unclassified Patescibacteria Patescibacteria bacterium isolate SMAG U8040 HLD86424.1
CPR Parcubacteria Veblenbacteria Candidatus Veblenbacteria bacterium isolate C24 metabat.bin.183 MDP3986343.1
CPR Parcubacteria Liptonbacteria Candidatus Liptonbacteria bacterium isolate M DeepCast 50m m2 187 MBM3257495.1
CPR Parcubacteria Kerfeldbacteria Candidatus Kerfeldbacteria bacterium isolate NC groundwater 1953 Pr3 S 0.2um 48 12 MBI5038138.1
CPR Parcubacteria Kaiserbacteria Candidatus Kaiserbacteria bacterium RIFCSPLOWO2 01 FULL 53 17 OGG73004.1
CPR Parcubacteria Lloydbacteria Candidatus Lloydbacteria bacterium RIFCSPHIGHO2 02 FULL 51 22 OGZ11648.1
CPR Parcubacteria Kaiserbacteria Candidatus Kaiserbacteria bacterium isolate NC groundwater 1720 Pr3 B 0.1um 51 29 MBI5470664.1
CPR Parcubacteria unclassified Parcubacteria Parcubacteria bacterium isolate Gr01 1014 8 TSC86160.1
CPR Parcubacteria Kaiserbacteria Candidatus Kaiserbacteria bacterium isolate NC groundwater 1721 Pr3 B 0.1um 51 76 MBI5456815.1
CPR Microgenomates Daviesbacteria Candidatus Daviesbacteria bacterium isolate NC groundwater 496 Ag S 0.1um 39 16 iso1 MBI2593064.1
CPR unclassified Patescibacteria Patescibacteria bacterium isolate FK Sedi B Bin.369 MCL5114040.1
CPR Microgenomates Levybacteria Candidatus Levybacteria bacterium CG10 big fil rev 8 21 14 0 10 35 13 PIR80431.1
CPR Microgenomates Roizmanbacteria Candidatus Roizmanbacteria bacterium isolate NC groundwater 1220 Ag S 0.1um 43 42 MBI4130814.1
CPR Microgenomates Woesebacteria Candidatus Woesebacteria bacterium RIFOXYA1 FULL 38 9 OGM74879.1
CPR Microgenomates Gottesmanbacteria Candidatus Gottesmanbacteria bacterium GW2011 GWA2 43 14 KKS97832.1
CPR Microgenomates Shapirobacteria Candidatus Shapirobacteria bacterium RBG 13 44 7 OGL53888.1
CPR unclassified Patescibacteria Patescibacteria bacterium isolate SO 2017 GW3 51 MFA5776718.1
CPR candidate division WWE3 candidate division WWE3 bacterium RIFCSPHIGHO2 12 FULL 38 15 OGC48229.1
CPR Microgenomates Daviesbacteria Candidatus Daviesbacteria bacterium isolate E14 maxbin.258 sub MDO8618630.1
CPR Microgenomates Woesebacteria Candidatus Woesebacteria bacterium isolate AMR MDS 2858 HRS22981.1
CPR Microgenomates Daviesbacteria Candidatus Daviesbacteria bacterium RIFCSPLOWO2 01 FULL 38 10 OGE39600.1
CPR Microgenomates Daviesbacteria Candidatus Daviesbacteria bacterium RIFCSPHIGHO2 01 FULL 38 8b OGE28772.1
CPR Microgenomates Blackburnbacteria Candidatus Blackburnbacteria bacterium isolate NC groundwater 136 Ag S 0.1um 46 17 MBI2007327.1
CPR unclassified Patescibacteria Patescibacteria bacterium isolate FK Sedi B Bin.119 iso1 MCL5970147.1
CPR Microgenomates Levybacteria Candidatus Levybacteria bacterium RIFCSPHIGHO2 01 FULL 38 12 OGH19395.1
CPR Microgenomates Levybacteria Candidatus Levybacteriota bacterium isolate C24 metabat.bin.177 sub iso1 MDP3987794.1
CPR Microgenomates Levybacteria Candidatus Levybacteria bacterium RIFCSPLOWO2 01 FULL 42 15 OGH42934.1
CPR Microgenomates Levybacteria Candidatus Levyibacteriota bacterium isolate Gw Bodden bin 174 iso2 MBP6913374.1
CPR Microgenomates Levybacteria Candidatus Levyibacteriota bacterium isolate Gw Eff bin 403 iso2 MBP7832197.1
CPR Microgenomates Levybacteria Candidatus Levyibacteriota bacterium isolate GcL13 1 MFI5265570.1
CPR unclassified Patescibacteria Patescibacteria bacterium isolate SMAG U8104 iso2 HLD01598.1
CPR Microgenomates Levybacteria Candidatus Levyibacteriota bacterium isolate NC groundwater 1183 Ag S 0.1um 43 83 iso1 MBI4084545.1
CPR Parcubacteria Candidatus Parcubacteria bacterium isolate farmington 2019 sw WHONDRS S19S 0053 A bin.46 iso2 MEN9406993.1
CPR Microgenomates Levybacteria Candidatus Levyibacteriota bacterium isolate NC groundwater 1182 Ag S 0.1um 43 56 iso2 MBI4080834.1
CPR unclassified Patescibacteria Patescibacteria bacterium isolate SMAG U2606 iso2 HEX8965855.1
CPR unclassified Patescibacteria Patescibacteria bacterium isolate REEB359 MDE2591088.1
CPR Microgenomates Levybacteria Candidatus Levyibacteriota bacterium isolate NC groundwater 236 Ag S 0.2um 36 14 MBI2074810.1
CPR Microgenomates Levybacteria Candidatus Levyibacteriota bacterium isolate NC groundwater 1181 Ag S 0.1um 42 368 iso2 MBI4079538.1
CPR unclassified Patescibacteria Patescibacteria bacterium isolate SMAG U2614 iso1 HEX8932158.1
CPR unclassified Patescibacteria Patescibacteria bacterium isolate H14 bin010 1 iso2 MEK7571735.1
CPR Microgenomates Levybacteria Candidatus Levyibacteriota bacterium isolate E14 maxbin.170 iso2 MDO8657454.1
CPR Microgenomates Candidatus Microgenomatota Candidatus Microgenomates bacterium isolate NC groundwater 170 Ag S 0.1um 42 15 iso1 MBI2036127.1
CPR Microgenomates Shapirobacteria Candidatus Shapirobacteria bacterium isolate NC groundwater 416 Ag B 0.1um 40 33 iso2 MBI2465395.1
CPR Saccharibacteria Candidatus Saccharimonadia Candidatus Saccharimonadales Candidatus Saccharimonadales bacterium isolate SMAG U15697 iso1 HSW48006.1
CPR Microgenomates Levybacteria Candidatus Levyibacteriota bacterium isolate NC groundwater 163 Ag S 0.1um 40 14 iso2 MBI2032893.1
CPR Microgenomates Levybacteria Candidatus Levyibacteriota bacterium isolate Gw Eff bin 508 iso3 MBP9719280.1
CPR Microgenomates Levybacteria Candidatus Levyibacteriota bacterium isolate Baikal deep G183 MSU26012.1
CPR unclassified Patescibacteria Patescibacteria bacterium isolate H41 bin139 1 MEK9176892.1
CPR unclassified Patescibacteria Patescibacteria bacterium isolate H41 bin080 MEK9160637.1
CPR Microgenomates Daviesbacteria Candidatus Daviesbacteria bacterium RIFCSPHIGHO2 01 FULL 44 29 OGE31586.1
CPR Microgenomates Daviesbacteria Candidatus Daviesbacteria bacterium isolate NC groundwater 494 Ag S 0.1um 37 28 MBI2600352.1
CPR unclassified Patescibacteria Patescibacteria bacterium isolate SMAG U8120 HLC88395.1
CPR Microgenomates Daviesbacteria Candidatus Daviesbacteria bacterium isolate NC groundwater 150 Ag S 0.1um 39 7 MBI2022499.1
CPR Microgenomates Levybacteria Candidatus Levybacteria bacterium CG2 30 37 29 OIP57686.1
CPR Microgenomates unclassified Microgenomates Microgenomates bacterium Gr01 1014 80 TSC66764.1
CPR Microgenomates Daviesbacteria Candidatus Daviesbacteria bacterium isolate C24 metabat.bin.195 sub MDP3974130.1
CPR Microgenomates Daviesbacteria Candidatus Daviesbacteria bacterium RIFCSPLOWO2 01 FULL 39 12 OGE44397.1
CPR Microgenomates Daviesbacteria Candidatus Daviesbacteria bacterium isolate C4 maxbin.154 MDP3733307.1
CPR Microgenomates Daviesbacteria Candidatus Daviesbacteria bacterium GW2011 GWF2 38 6 KKQ78872.1
CPR Microgenomates Daviesbacteria Candidatus Daviesbacteria bacterium isolate NC groundwater 495 Ag S 0.1um 37 8 MBI2599931.1
CPR Microgenomates Candidatus Microgenomatota Candidatus Microgenomates bacterium isolate NC groundwater 171 Ag S 0.1um 39 14 MBI2039754.1
CPR Microgenomates Daviesbacteria Candidatus Daviesbacteria bacterium RIFCSPLOWO2 02 FULL 38 15 OGE71923.1
CPR Microgenomates Daviesbacteria Candidatus Daviesbacteria bacterium isolate NC groundwater 149 Ag S 0.1um 39 14 MBI2018815.1
CPR Microgenomates Daviesbacteria Candidatus Daviesbacteria bacterium isolate NC groundwater 145 Ag S 0.1um 37 18 MBI2017626.1
CPR Microgenomates Daviesbacteria Candidatus Daviesbacteria bacterium isolate NC groundwater 346 Ag B 0.1um 38 13 MBI2330241.1
CPR Microgenomates unclassified Microgenomates Microgenomates bacterium Gr01 1014 7 TSC88318.1
CPR Microgenomates Daviesbacteria Candidatus Daviesbacteria bacterium RIFCSPHIGHO2 02 FULL 36 13 OGE31462.1
CPR unclassified Patescibacteria Patescibacteria bacterium isolate H51 bin048 1 MEK7617019.1
CPR Microgenomates Daviesbacteria Candidatus Daviesbacteria bacterium isolate NC groundwater 493 Ag S 0.1um 36 17 MBI2597204.1
CPR unclassified Patescibacteria Patescibacteria bacterium isolate MEGAHIT 1.553 MFH1535549.1
CPR Microgenomates Daviesbacteria Candidatus Daviesbacteria bacterium isolate STD1 94 MDD5416102.1
CPR unclassified Patescibacteria Patescibacteria bacterium isolate FK Sedi A Bin.182 MCL4366371.1
CPR Microgenomates Daviesbacteria Candidatus Daviesbacteria bacterium isolate NC groundwater 347 Ag B 0.1um 39 21 MBI2338499.1
CPR Microgenomates Daviesbacteria Candidatus Daviesbacteria bacterium RIFCSPLOWO2 02 FULL 36 7 OGE65069.1
CPR Microgenomates Daviesbacteria Candidatus Daviesbacteria bacterium isolate NC groundwater 1135 Ag S 0.1um 37 444 MBI4035876.1L
CPR unclassified Patescibacteria Patescibacteria bacterium isolate MM PC MetaG.mb.55 MBU1000020.1
CPR Microgenomates Daviesbacteria Candidatus Daviesbacteria bacterium isolate NC groundwater 227 Ag S 0.2um 36 15 MBI2086340.1
CPR Microgenomates Daviesbacteria Candidatus Daviesbacteria bacterium RIFCSPLOWO2 02 FULL 38 15 MBI2020543.1
CPR Microgenomates Daviesbacteria Candidatus Daviesbacteria bacterium isolate NC groundwater 345 Ag B 0.1um 36 18 MBI2315300.1
CPR Microgenomates Daviesbacteria Candidatus Daviesbacteria bacterium RIFCSPHIGHO2 01 FULL 40 11 OGE28069.1
CPR Microgenomates Daviesbacteria Candidatus Daviesbacteria bacterium isolate NC groundwater 297 Ag B 0.1um 41 14 MBI2196906.1
CPR Microgenomates Daviesbacteria Candidatus Daviesbacteria bacterium RIFCSPHIGHO2 01 FULL 41 23 OGE19289.1
CPR Microgenomates Daviesbacteria Candidatus Daviesbacteria bacterium isolate E14 metabat.bin.170 sub MDO8573500.1
Pseudomonadati PVC group Chlamydiota unclassified Chlamydiota Chlamydiota bacterium MFC1668500.1 289 aa
Pseudomonadati PVC group Candidatus Auribacterota Candidatus Theseobacter MDP8217927.1 313 aa
Pseudomonadati PVC group Chlamydiota unclassified Chlamydiota Chlamydiota bacterium MDP3980219.1 311 aa
Pseudomonadati PVC group Chlamydiota unclassified Chlamydiota Chlamydiota bacterium MBI1882640.1 311 aa
Pseudomonadati PVC group Chlamydiota unclassified Chlamydiota Chlamydiota bacterium MBI1871065.1 312 aa
Pseudomonadati PVC group Lentisphaerota Lentisphaeria Lentisphaeria bacterium MFO7821251.1 324 aa
Pseudomonadati PVC group Lentisphaerota Lentisphaeria Victivallaceae bacterium MFA7231787.1 319 aa
Pseudomonadati PVC group Lentisphaerota Lentisphaeria Victivallaceae bacterium MDD4180402.1 315 aa
Pseudomonadati PVC group Kiritimatiellota Kiritimatiellia Kiritimatiellales bacterium MCF7863302.1 308 aa
Pseudomonadati PVC group Kiritimatiellota Tichowtungiia Tichowtungia sp. HKL22605.1 309 aa
Pseudomonadati PVC group Kiritimatiellota Tichowtungiia Tichowtungia aerotolerans WP 160626914.1 309 aa
Pseudomonadati PVC group Kiritimatiellota Kiritimatiellia Pontiellaceae bacterium MCU0857081.1 309 aa
Pseudomonadati PVC group Kiritimatiellota Kiritimatiellia Kiritimatiellales bacterium MFA5688481.1 309 aa
Pseudomonadati PVC group Verrucomicrobiota Verrucomicrobiia Brevifollis gellanilyticus WP 146851429.1 310 aa
Pseudomonadati PVC group Verrucomicrobiota Verrucomicrobiia Rubritalea sp. MFT5634550.1 309 aa
Pseudomonadati PVC group Verrucomicrobiota Verrucomicrobiia Candidatus Akkermansia HIX19245.1 309 aa
Pseudomonadati PVC group Verrucomicrobiota Spartobacteria Chthoniobacteraceae bacterium MGC3990102.1 320 aa
Pseudomonadati PVC group Verrucomicrobiota Terrimicrobiia Terrimicrobiaceae bacterium MFZ9920347.1 312 aa
Pseudomonadati PVC group Verrucomicrobiota Spartobacteria Chthoniobacterales bacterium HLB34057.1 309 aa
Pseudomonadati PVC group Verrucomicrobiota Terrimicrobiia Terrimicrobiaceae bacterium MEX1119343.1 310 aa
Pseudomonadati PVC group Verrucomicrobiota Terrimicrobiia Terrimicrobiaceae bacterium GAB4175044.1 332 aa
Pseudomonadati PVC group Verrucomicrobiota Methylacidiphilae Methylacidiphilum kamchatkense WP 039721852.1 322 aa
Pseudomonadati PVC group Verrucomicrobiota Methylacidiphilae Candidatus Methylacidithermus WP 174582303.1 320 aa
Pseudomonadati PVC Verrucomicrobiota Verrucomicrobia bacterium LW23 WP 108040016.1 ClassIV
Pseudomonadati PVC Verrucomicrobiota Candidatus Methylacidiphilales bacterium isolate bin.034 MEZ0294857.1 ClassIV
Pseudomonadati PVC Verrucomicrobiota Candidatus Methylacidiphilales bacterium isolate SMAG U16154 HSI83038.1 ClassIV
Pseudomonadati PVC group Verrucomicrobiota Verrucomicrobiia Limisphaerales bacterium MFN3408053.1 314 aa

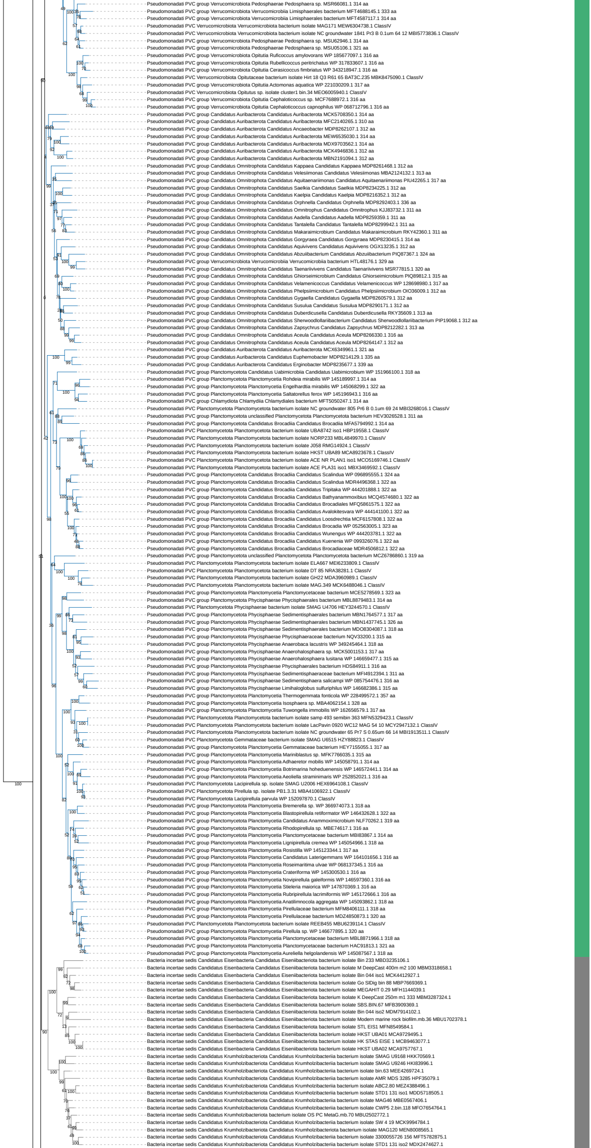

Pseudomonadati PVC group Verrucomicrobiota Pedosphaerae Pedosphaera sp. MSR66081.1 314 aa
Pseudomonadati PVC group Verrucomicrobiota Verrucomicrobiia Limisphaerales bacterium MFT4688145.1 333 aa
Pseudomonadati PVC group Verrucomicrobiota Verrucomicrobiia Limisphaerales bacterium MFT4587117.1 314 aa
Pseudomonadati PVC Verrucomicrobiota Verrucomicrobiota bacterium isolate MAG171 MEW6304738.1 ClassIV
Pseudomonadati PVC Verrucomicrobiota Verrucomicrobiota bacterium isolate NC groundwater 1841 Pr3 B 0.1um 64 12 MBI5773836.1 ClassIV
Pseudomonadati PVC group Verrucomicrobiota Pedosphaerae Pedosphaera sp. MSU62946.1 314 aa
Pseudomonadati PVC group Verrucomicrobiota Pedosphaerae Pedosphaera sp. MSU05106.1 321 aa
Pseudomonadati PVC group Verrucomicrobiota Opitutia Ruficoccus amylovorans WP 185677097.1 316 aa
Pseudomonadati PVC group Verrucomicrobiota Opitutia Rubellicoccus peritrichatus WP 317833607.1 316 aa
Pseudomonadati PVC group Verrucomicrobiota Opitutia Cerasicoccus fimbriatus WP 343218947.1 316 aa
Pseudomonadati PVC Verrucomicrobiota Opitutaceae bacterium isolate Hirt 18 Q3 R61 65 BAT3C.235 MBK8475090.1 ClassIV
Pseudomonadati PVC group Verrucomicrobiota Opitutia Actomonas aquatica WP 221030209.1 317 aa
Pseudomonadati PVC Verrucomicrobiota Opitutus sp. isolate cluster1 bin.34 MEO6005940.1 ClassIV
Pseudomonadati PVC group Verrucomicrobiota Opitutia Cephaloticoccus sp. MCF7688972.1 316 aa
Pseudomonadati PVC group Verrucomicrobiota Opitutia Cephaloticoccus capnophilus WP 068712796.1 316 aa
Pseudomonadati PVC group Candidatus Auribacterota Candidatus Auribacterota MCK5708350.1 314 aa
Pseudomonadati PVC group Candidatus Auribacterota Candidatus Auribacterota MFC2140265.1 310 aa
Pseudomonadati PVC group Candidatus Auribacterota Candidatus Ancaeobacter MDP8262107.1 312 aa
Pseudomonadati PVC group Candidatus Auribacterota Candidatus Auribacterota MEW6535030.1 314 aa
Pseudomonadati PVC group Candidatus Auribacterota Candidatus Auribacterota MDX9703562.1 314 aa
Pseudomonadati PVC group Candidatus Auribacterota Candidatus Auribacterota MCK4946836.1 312 aa
Pseudomonadati PVC group Candidatus Auribacterota Candidatus Auribacterota MBN2191094.1 312 aa
Pseudomonadati PVC group Candidatus Omnitrophota Candidatus Kappaea Candidatus Kappaea MDP8261468.1 312 aa
Pseudomonadati PVC group Candidatus Omnitrophota Candidatus Velesiimonas Candidatus Velesiimonas MBA2124132.1 313 aa
Pseudomonadati PVC group Candidatus Omnitrophota Candidatus Aquitaenariimonas Candidatus Aquitaenariimonas PIU42265.1 317 aa
Pseudomonadati PVC group Candidatus Omnitrophota Candidatus Saelkia Candidatus Saelkia MDP8234225.1 312 aa
Pseudomonadati PVC group Candidatus Omnitrophota Candidatus Kaelpia Candidatus Kaelpia MDP8216352.1 312 aa
Pseudomonadati PVC group Candidatus Omnitrophota Candidatus Orphnella Candidatus Orphnella MDP8292403.1 336 aa
Pseudomonadati PVC group Candidatus Omnitrophota Candidatus Omnitrophus Candidatus Omnitrophus KJJ83732.1 311 aa
Pseudomonadati PVC group Candidatus Omnitrophota Candidatus Aadella Candidatus Aadella MDP8259359.1 311 aa
Pseudomonadati PVC group Candidatus Omnitrophota Candidatus Tantalella Candidatus Tantalella MDP8299942.1 311 aa
Pseudomonadati PVC group Candidatus Omnitrophota Candidatus Makaraimicrobium Candidatus Makaraimicrobium RKY42360.1 311 aa
Pseudomonadati PVC group Candidatus Omnitrophota Candidatus Gorgyraea Candidatus Gorgyraea MDP8230415.1 314 aa
Pseudomonadati PVC group Candidatus Omnitrophota Candidatus Aquivivens Candidatus Aquivivens OGX13235.1 312 aa
Pseudomonadati PVC group Candidatus Omnitrophota Candidatus Abzuiibacterium Candidatus Abzuiibacterium PIQ87367.1 324 aa
Pseudomonadati PVC group Verrucomicrobiota Verrucomicrobiia Verrucomicrobiia bacterium HTL48176.1 329 aa
Pseudomonadati PVC group Candidatus Omnitrophota Candidatus Taenariivivens Candidatus Taenariivivens MSR77815.1 320 aa
Pseudomonadati PVC group Candidatus Omnitrophota Candidatus Ghiorseimicrobium Candidatus Ghiorseimicrobium PIQ89812.1 315 aa
Pseudomonadati PVC group Candidatus Omnitrophota Candidatus Velamenicoccus Candidatus Velamenicoccus WP 128698980.1 317 aa
Pseudomonadati PVC group Candidatus Omnitrophota Candidatus Phelpsiimicrobium Candidatus Phelpsiimicrobium OIO36009.1 312 aa
Pseudomonadati PVC group Candidatus Omnitrophota Candidatus Gygaella Candidatus Gygaella MDP8260579.1 312 aa
Pseudomonadati PVC group Candidatus Omnitrophota Candidatus Susulua Candidatus Susulua MDP8290171.1 312 aa
Pseudomonadati PVC group Candidatus Omnitrophota Candidatus Duberdicusella Candidatus Duberdicusella RKY35609.1 313 aa
Pseudomonadati PVC group Candidatus Omnitrophota Candidatus Sherwoodlollariibacterium Candidatus Sherwoodlollariibacterium PIP19068.1 312 aa
Pseudomonadati PVC group Candidatus Omnitrophota Candidatus Zapsychrus Candidatus Zapsychrus MDP8212282.1 313 aa
Pseudomonadati PVC group Candidatus Omnitrophota Candidatus Aceula Candidatus Aceula MDP8266330.1 316 aa
Pseudomonadati PVC group Candidatus Omnitrophota Candidatus Aceula Candidatus Aceula MDP8264147.1 312 aa
Pseudomonadati PVC group Candidatus Auribacterota Candidatus Auribacterota MCX6349961.1 321 aa
Pseudomonadati PVC group Candidatus Auribacterota Candidatus Euphemobacter MDP8214129.1 335 aa
Pseudomonadati PVC group Candidatus Auribacterota Candidatus Erginobacter MDP8235677.1 339 aa
Pseudomonadati PVC group Planctomycetota Candidatus Uabimicrobiia Candidatus Uabimicrobium WP 151966100.1 318 aa
Pseudomonadati PVC group Planctomycetota Planctomycetia Rohdeia mirabilis WP 145189997.1 314 aa
Pseudomonadati PVC group Planctomycetota Planctomycetia Engelhardtia mirabilis WP 145068299.1 322 aa
Pseudomonadati PVC group Planctomycetota Planctomycetia Saltatorellus ferox WP 145196943.1 316 aa
Pseudomonadati PVC group Chlamydiota Chlamydiia Chlamydiales bacterium MFT5050247.1 314 aa
Pseudomonadati PVC Planctomycetota Planctomycetota bacterium isolate NC groundwater 805 Pr6 B 0.1um 69 24 MBI3268016.1 ClassIV
Pseudomonadati PVC group Planctomycetota unclassified Planctomycetota Planctomycetota bacterium HEV3026528.1 311 aa
Pseudomonadati PVC group Planctomycetota Candidatus Brocadiia Candidatus Brocadiia MFA5794992.1 314 aa
Pseudomonadati PVC Planctomycetota Planctomycetota bacterium isolate UBA8742 iso1 HBP19558.1 ClassIV
Pseudomonadati PVC Planctomycetota Planctomycetota bacterium isolate NORP233 MBL4849970.1 ClassIV
Pseudomonadati PVC Planctomycetota Planctomycetota bacterium isolate J058 RMG14924.1 ClassIV
Pseudomonadati PVC Planctomycetota Planctomycetota bacterium isolate HKST UBA89 MCA8923678.1 ClassIV
Pseudomonadati PVC Planctomycetota Planctomycetota bacterium isolate ACE NR PLAN1 iso1 MCO5169746.1 ClassIV
Pseudomonadati PVC Planctomycetota Planctomycetota bacterium isolate ACE PLA31 iso1 MBX3469592.1 ClassIV
Pseudomonadati PVC group Planctomycetota Candidatus Brocadiia Candidatus Scalindua WP 096895555.1 324 aa
Pseudomonadati PVC group Planctomycetota Candidatus Brocadiia Candidatus Scalindua MDR4496368.1 322 aa
Pseudomonadati PVC group Planctomycetota Candidatus Brocadiia Candidatus Tripitaka WP 444201888.1 322 aa
Pseudomonadati PVC group Planctomycetota Candidatus Brocadiia Candidatus Bathyanammoxibius MCQ4574680.1 322 aa
Pseudomonadati PVC group Planctomycetota Candidatus Brocadiia Candidatus Brocadiales MFQ5861575.1 322 aa
Pseudomonadati PVC group Planctomycetota Candidatus Brocadiia Candidatus Avalokitesvara WP 444141100.1 322 aa
Pseudomonadati PVC group Planctomycetota Candidatus Brocadiia Candidatus Loosdrechtia MCF6157808.1 322 aa
Pseudomonadati PVC group Planctomycetota Candidatus Brocadiia Candidatus Brocadia WP 052563005.1 323 aa
Pseudomonadati PVC group Planctomycetota Candidatus Brocadiia Candidatus Wunengus WP 444203781.1 322 aa
Pseudomonadati PVC group Planctomycetota Candidatus Brocadiia Candidatus Kuenenia WP 099326076.1 322 aa
Pseudomonadati PVC group Planctomycetota Candidatus Brocadiia Candidatus Brocadiaceae MDR4506812.1 322 aa
Pseudomonadati PVC group Planctomycetota unclassified Planctomycetota Planctomycetota bacterium MCZ6786860.1 319 aa
Pseudomonadati PVC Planctomycetota Planctomycetota bacterium isolate ELA667 MEI6233809.1 ClassIV
Pseudomonadati PVC Planctomycetota Planctomycetota bacterium isolate DT 85 NRA38281.1 ClassIV
Pseudomonadati PVC Planctomycetota Planctomycetota bacterium isolate GH22 MDA3960989.1 ClassIV
Pseudomonadati PVC Planctomycetota Planctomycetota bacterium isolate MAG.349 MCK6488046.1 ClassIV
Pseudomonadati PVC group Planctomycetota Planctomycetia Planctomycetaceae bacterium MCE5278569.1 323 aa
Pseudomonadati PVC group Planctomycetota Phycisphaerae Phycisphaerales bacterium MBL8879483.1 314 aa
Pseudomonadati PVC Planctomycetota Phycisphaerae bacterium isolate SMAG U4706 HEY3244570.1 ClassIV
Pseudomonadati PVC group Planctomycetota Phycisphaerae Sedimentisphaerales bacterium MBN1764577.1 317 aa
Pseudomonadati PVC group Planctomycetota Phycisphaerae Sedimentisphaerales bacterium MBN1437745.1 326 aa
Pseudomonadati PVC group Planctomycetota Phycisphaerae Sedimentisphaerales bacterium MDO8304087.1 318 aa
Pseudomonadati PVC group Planctomycetota Phycisphaerae Phycisphaeraceae bacterium NQV33200.1 315 aa
Pseudomonadati PVC group Planctomycetota Phycisphaerae Anaerobaca lacustris WP 349245464.1 318 aa
Pseudomonadati PVC group Planctomycetota Phycisphaerae Anaerohalosphaera sp. MCK5001153.1 317 aa
Pseudomonadati PVC group Planctomycetota Phycisphaerae Anaerohalosphaera lusitana WP 146659477.1 315 aa
Pseudomonadati PVC group Planctomycetota Phycisphaerae Phycisphaerales bacterium HDS84911.1 316 aa
Pseudomonadati PVC group Planctomycetota Phycisphaerae Sedimentisphaeraceae bacterium MFI4912394.1 311 aa
Pseudomonadati PVC group Planctomycetota Phycisphaerae Sedimentisphaera salicampi WP 085754476.1 316 aa
Pseudomonadati PVC group Planctomycetota Phycisphaerae Limihaloglobus sulfuriphilus WP 146682386.1 315 aa
Pseudomonadati PVC group Planctomycetota Planctomycetia Thermogemmata fonticola WP 228499572.1 357 aa
Pseudomonadati PVC group Planctomycetota Planctomycetia Isosphaera sp. MBA4062154.1 328 aa
Pseudomonadati PVC group Planctomycetota Planctomycetia Tuwongella immobilis WP 162656579.1 317 aa
Pseudomonadati PVC Planctomycetota Planctomycetota bacterium isolate samp 493 semibin 363 MFN5329423.1 ClassIV
Pseudomonadati PVC Planctomycetota Planctomycetota bacterium isolate LacPavin 0920 WC12 MAG 54 10 MCY2947132.1 ClassIV
Pseudomonadati PVC Planctomycetota Planctomycetota bacterium isolate NC groundwater 65 Pr7 S 0.65um 66 14 MBI1913511.1 ClassIV
Pseudomonadati PVC Planctomycetota Gemmataceae bacterium isolate SMAG U6515 HZY88823.1 ClassIV
Pseudomonadati PVC group Planctomycetota Planctomycetia Gemmataceae bacterium HEY7155055.1 317 aa
Pseudomonadati PVC group Planctomycetota Planctomycetia Mariniblastus sp. MFK7766035.1 315 aa
Pseudomonadati PVC group Planctomycetota Planctomycetia Adhaeretor mobilis WP 145058791.1 314 aa
Pseudomonadati PVC group Planctomycetota Planctomycetia Botrimarina hoheduenensis WP 146572441.1 314 aa
Pseudomonadati PVC group Planctomycetota Planctomycetia Aeoliella straminimaris WP 252852021.1 316 aa
Pseudomonadati PVC Planctomycetota Lacipirellula sp. isolate SMAG U2006 HEX6964108.1 ClassIV
Pseudomonadati PVC Planctomycetota Pirellula sp. isolate PB1.3.31 MBA4106922.1 ClassIV
Pseudomonadati PVC Planctomycetota Lacipirellula parvula WP 152097870.1 ClassIV
Pseudomonadati PVC group Planctomycetota Planctomycetia Bremerella sp. WP 366974073.1 318 aa
Pseudomonadati PVC group Planctomycetota Planctomycetia Blastopirellula retiformator WP 146432628.1 322 aa
Pseudomonadati PVC group Planctomycetota Planctomycetia Candidatus Anammoximicrobium NLF70262.1 319 aa
Pseudomonadati PVC group Planctomycetota Planctomycetia Rhodopirellula sp. MBE74617.1 316 aa
Pseudomonadati PVC group Planctomycetota Planctomycetia Planctomycetaceae bacterium MBI83867.1 314 aa
Pseudomonadati PVC group Planctomycetota Planctomycetia Lignipirellula cremea WP 145054966.1 318 aa
Pseudomonadati PVC group Planctomycetota Planctomycetia Rosistilla WP 145123344.1 317 aa
Pseudomonadati PVC group Planctomycetota Planctomycetia Candidatus Laterigemmans WP 164101656.1 316 aa
Pseudomonadati PVC group Planctomycetota Planctomycetia Roseimaritima ulvae WP 068137345.1 316 aa
Pseudomonadati PVC group Planctomycetota Planctomycetia Crateriforma WP 145300530.1 316 aa
Pseudomonadati PVC group Planctomycetota Planctomycetia Novipirellula galeiformis WP 146597360.1 316 aa
Pseudomonadati PVC group Planctomycetota Planctomycetia Stieleria maiorica WP 147870369.1 316 aa
Pseudomonadati PVC group Planctomycetota Planctomycetia Rubripirellula lacrimiformis WP 145172666.1 316 aa
Pseudomonadati PVC group Planctomycetota Planctomycetia Anatilimnocola aggregata WP 145093862.1 318 aa
Pseudomonadati PVC group Planctomycetota Planctomycetia Pirellulaceae bacterium MFM8406111.1 318 aa
Pseudomonadati PVC group Planctomycetota Planctomycetia Pirellulaceae bacterium MDZ4850873.1 320 aa
Pseudomonadati PVC Planctomycetota Planctomycetota bacterium isolate REEB455 MBU6239114.1 ClassIV
Pseudomonadati PVC group Planctomycetota Planctomycetia Pirellula sp. WP 146677895.1 320 aa
Pseudomonadati PVC group Planctomycetota Planctomycetia Planctomycetaceae bacterium MBL8871966.1 318 aa
Pseudomonadati PVC group Planctomycetota Planctomycetia Planctomycetaceae bacterium HAC91813.1 321 aa
Pseudomonadati PVC group Planctomycetota Planctomycetia Aureliella helgolandensis WP 145087567.1 318 aa
Bacteria incertae sedis Candidatus Eisenbacteria Candidatus Eiseniibacteriota bacterium isolate Bin 233 MBD3235106.1
Bacteria incertae sedis Candidatus Eisenbacteria Candidatus Eiseniibacteriota bacterium isolate M DeepCast 400m m2 100 MBM3318658.1
Bacteria incertae sedis Candidatus Eisenbacteria Candidatus Eiseniibacteriota bacterium isolate Bin 044 iso1 MCK4412927.1
Bacteria incertae sedis Candidatus Eisenbacteria Candidatus Eiseniibacteriota bacterium isolate Go SlDig bin 88 MBP7669369.1
Bacteria incertae sedis Candidatus Eisenbacteria Candidatus Eiseniibacteriota bacterium isolate MEGAHIT 0.29 MFH1144039.1
Bacteria incertae sedis Candidatus Eisenbacteria Candidatus Eiseniibacteriota bacterium isolate K DeepCast 250m m1 333 MBM3287324.1
Bacteria incertae sedis Candidatus Eisenbacteria Candidatus Eiseniibacteriota bacterium isolate SBS.BIN.67 MFB3909369.1
Bacteria incertae sedis Candidatus Eisenbacteria Candidatus Eiseniibacteriota bacterium isolate Bin 044 iso2 MDM7914102.1
Bacteria incertae sedis Candidatus Eisenbacteria Candidatus Eiseniibacteriota bacterium isolate Modern marine rock biofilm.mb.36 MBU1702378.1
Bacteria incertae sedis Candidatus Eisenbacteria Candidatus Eiseniibacteriota bacterium isolate STL EIS1 MFN8549584.1
Bacteria incertae sedis Candidatus Eisenbacteria Candidatus Eiseniibacteriota bacterium isolate HKST UBA01 MCA9729495.1
Bacteria incertae sedis Candidatus Eisenbacteria Candidatus Eiseniibacteriota bacterium isolate HK STAS EISE 1 MCB9463077.1
Bacteria incertae sedis Candidatus Eisenbacteria Candidatus Eiseniibacteriota bacterium isolate HKST UBA02 MCA9757767.1
Bacteria incertae sedis Candidatus Krumholzibacteriota Candidatus Krumholzibacteriia bacterium isolate SMAG U9168 HKK70569.1
Bacteria incertae sedis Candidatus Krumholzibacteriota Candidatus Krumholzibacteriia bacterium isolate SMAG U9246 HKI83996.1
Bacteria incertae sedis Candidatus Krumholzibacteriota Candidatus Krumholzibacteriia bacterium isolate bin.63 MEE4269724.1
Bacteria incertae sedis Candidatus Krumholzibacteriota Candidatus Krumholzibacteriia bacterium isolate AMR MDS 3285 HPF35079.1
Bacteria incertae sedis Candidatus Krumholzibacteriota Candidatus Krumholzibacteriia bacterium isolate ABC2.80 MEZ4388496.1
Bacteria incertae sedis Candidatus Krumholzibacteriota Candidatus Krumholzibacteriia bacterium isolate STD1 131 iso1 MDD5718505.1
Bacteria incertae sedis Candidatus Krumholzibacteriota Candidatus Krumholzibacteriia bacterium isolate MAG46 MBE0567406.1
Bacteria incertae sedis Candidatus Krumholzibacteriota Candidatus Krumholzibacteriia bacterium isolate CWP5 2.bin.118 MFO7654764.1
Bacteria incertae sedis Candidatus Krumholzibacteriota bacterium isolate OS PC MetaG.mb.70 MBU2502772.1
Bacteria incertae sedis Candidatus Krumholzibacteriota Candidatus Krumholzibacteriia bacterium isolate SW 4 19 MCK9994784.1
Bacteria incertae sedis Candidatus Krumholzibacteriota Candidatus Krumholzibacteriota bacterium isolate MAG120 MEN8008565.1
Bacteria incertae sedis Candidatus Krumholzibacteriota Candidatus Krumholzibacteriia bacterium isolate 3300055726 156 MFT5782875.1
Bacteria incertae sedis Candidatus Krumholzibacteriota Candidatus Krumholzibacteriia bacterium isolate STD1 131 iso2 MDX2474627.1

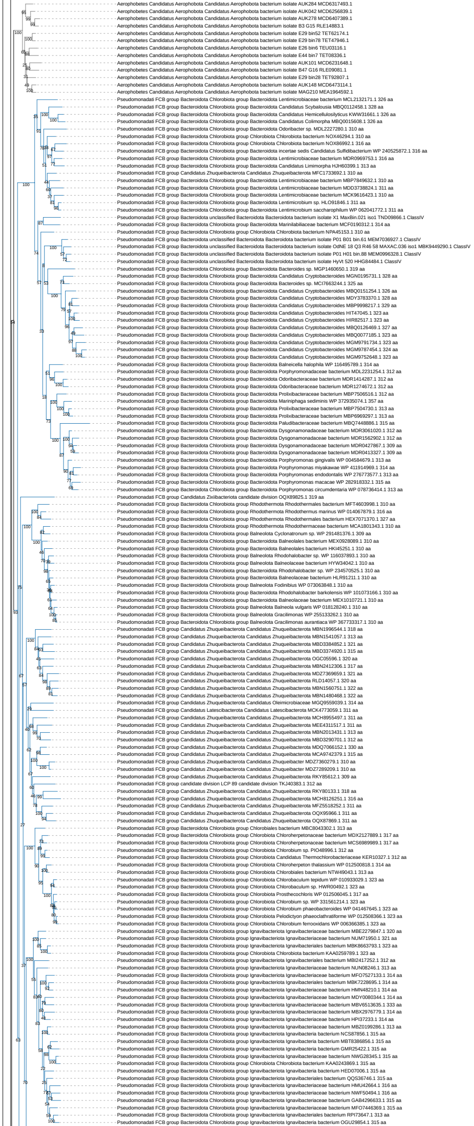

Aerophobetes Candidatus Aerophobota Candidatus Aerophobota bacterium isolate AUK284 MCD6317493.1
Aerophobetes Candidatus Aerophobota Candidatus Aerophobota bacterium isolate AUK042 MCD6256839.1
Aerophobetes Candidatus Aerophobota Candidatus Aerophobota bacterium isolate AUK278 MCD6407389.1
Aerophobetes Candidatus Aerophobota Candidatus Aerophobota bacterium isolate B3 G15 RLE14883.1
Aerophobetes Candidatus Aerophobota Candidatus Aerophobota bacterium isolate E29 bin52 TET62174.1
Aerophobetes Candidatus Aerophobota Candidatus Aerophobota bacterium isolate E29 bin78 TET47946.1
Aerophobetes Candidatus Aerophobota Candidatus Aerophobota bacterium isolate E26 bin6 TEU03116.1
Aerophobetes Candidatus Aerophobota Candidatus Aerophobota bacterium isolate E44 bin7 TET08336.1
Aerophobetes Candidatus Aerophobota Candidatus Aerophobota bacterium isolate AUK101 MCD6231648.1
Aerophobetes Candidatus Aerophobota Candidatus Aerophobota bacterium isolate B47 G16 RLE09081.1
Aerophobetes Candidatus Aerophobota Candidatus Aerophobota bacterium isolate E29 bin28 TET92807.1
Aerophobetes Candidatus Aerophobota Candidatus Aerophobota bacterium isolate AUK148 MCD6473114.1
Aerophobetes Candidatus Aerophobota Candidatus Aerophobota bacterium isolate MAG210 MEA1964592.1
Pseudomonadati FCB group Bacteroidota Chlorobiota group Bacteroidota Lentimicrobiaceae bacterium MCL2132171.1 326 aa
Pseudomonadati FCB group Bacteroidota Chlorobiota group Bacteroidota Candidatus Scybalousia MBQ0112458.1 328 aa
Pseudomonadati FCB group Bacteroidota Chlorobiota group Bacteroidota Candidatus Hemicellulosilyticus KWW31661.1 326 aa
Pseudomonadati FCB group Bacteroidota Chlorobiota group Bacteroidota Candidatus Colimorpha MBQ0015608.1 326 aa
Pseudomonadati FCB group Bacteroidota Chlorobiota group Bacteroidota Odoribacter sp. MDL2227280.1 310 aa
Pseudomonadati FCB group Bacteroidota Chlorobiota group Chlorobiota Chlorobiota bacterium NOX46294.1 310 aa
Pseudomonadati FCB group Bacteroidota Chlorobiota group Chlorobiota Chlorobiota bacterium NOX86992.1 316 aa
Pseudomonadati FCB group Bacteroidota Chlorobiota group Bacteroidota incertae sedis Candidatus Sulfidibacterium WP 240525872.1 316 aa
Pseudomonadati FCB group Bacteroidota Chlorobiota group Bacteroidota Lentimicrobiaceae bacterium MDR0969753.1 316 aa
Pseudomonadati FCB group Bacteroidota Chlorobiota group Bacteroidota Candidatus Limimorpha HJH60399.1 313 aa
Pseudomonadati FCB group Candidatus Zhuqueibacterota Candidatus Zhuqueibacterota MFC1733692.1 310 aa
Pseudomonadati FCB group Bacteroidota Chlorobiota group Bacteroidota Lentimicrobiaceae bacterium MBP7849632.1 310 aa
Pseudomonadati FCB group Bacteroidota Chlorobiota group Bacteroidota Lentimicrobiaceae bacterium MDD3738824.1 311 aa
Pseudomonadati FCB group Bacteroidota Chlorobiota group Bacteroidota Lentimicrobiaceae bacterium MCK9616423.1 310 aa
Pseudomonadati FCB group Bacteroidota Chlorobiota group Bacteroidota Lentimicrobium sp. HLO91846.1 311 aa
Pseudomonadati FCB group Bacteroidota Chlorobiota group Bacteroidota Lentimicrobium saccharophilum WP 062041772.1 311 aa
Pseudomonadati FCB group Bacteroidota unclassified Bacteroidota Bacteroidota bacterium isolate X1 MaxBin.021 iso1 TND09866.1 ClassIV
Pseudomonadati FCB group Bacteroidota Chlorobiota group Bacteroidota Marinilabiliaceae bacterium MCF0190312.1 314 aa
Pseudomonadati FCB group Bacteroidota Chlorobiota group Chlorobiota Chlorobiota bacterium NPA45153.1 310 aa
Pseudomonadati FCB group Bacteroidota unclassified Bacteroidota Bacteroidota bacterium isolate P01 B01 bin.61 MEM7036927.1 ClassIV
Pseudomonadati FCB group Bacteroidota unclassified Bacteroidota Bacteroidota bacterium isolate OdNE 18 Q3 R46 58 MAXAC.036 iso1 MBK9449290.1 ClassIV
Pseudomonadati FCB group Bacteroidota unclassified Bacteroidota Bacteroidota bacterium isolate P01 H01 bin.88 MEM0996328.1 ClassIV
Pseudomonadati FCB group Bacteroidota unclassified Bacteroidota Bacteroidota bacterium isolate HyVt 520 HHG84484.1 ClassIV
Pseudomonadati FCB group Bacteroidota Chlorobiota group Bacteroidota Bacteroides sp. MGP1460650.1 319 aa
Pseudomonadati FCB group Bacteroidota Chlorobiota group Bacteroidota Candidatus Cryptobacteroides MGN0195731.1 328 aa
Pseudomonadati FCB group Bacteroidota Chlorobiota group Bacteroidota Bacteroides sp. MCI7663244.1 325 aa
Pseudomonadati FCB group Bacteroidota Chlorobiota group Bacteroidota Candidatus Cryptobacteroides MBQ0151254.1 326 aa
Pseudomonadati FCB group Bacteroidota Chlorobiota group Bacteroidota Candidatus Cryptobacteroides MDY3783370.1 328 aa
Pseudomonadati FCB group Bacteroidota Chlorobiota group Bacteroidota Candidatus Cryptobacteroides MBP9998217.1 329 aa
Pseudomonadati FCB group Bacteroidota Chlorobiota group Bacteroidota Candidatus Cryptobacteroides HIT47045.1 323 aa
Pseudomonadati FCB group Bacteroidota Chlorobiota group Bacteroidota Candidatus Cryptobacteroides HIR82517.1 323 aa
Pseudomonadati FCB group Bacteroidota Chlorobiota group Bacteroidota Candidatus Cryptobacteroides MBQ0126469.1 327 aa
Pseudomonadati FCB group Bacteroidota Chlorobiota group Bacteroidota Candidatus Cryptobacteroides MBQ0077185.1 323 aa
Pseudomonadati FCB group Bacteroidota Chlorobiota group Bacteroidota Candidatus Cryptobacteroides MGM9791734.1 323 aa
Pseudomonadati FCB group Bacteroidota Chlorobiota group Bacteroidota Candidatus Cryptobacteroides MGM9787454.1 324 aa
Pseudomonadati FCB group Bacteroidota Chlorobiota group Bacteroidota Candidatus Cryptobacteroides MGM9752648.1 323 aa
Pseudomonadati FCB group Bacteroidota Chlorobiota group Bacteroidota Balneicella halophila WP 116495789.1 314 aa
Pseudomonadati FCB group Bacteroidota Chlorobiota group Bacteroidota Porphyromonadaceae bacterium MDL2231254.1 312 aa
Pseudomonadati FCB group Bacteroidota Chlorobiota group Bacteroidota Odoribacteraceae bacterium MDR1414287.1 312 aa
Pseudomonadati FCB group Bacteroidota Chlorobiota group Bacteroidota Odoribacteraceae bacterium MDR1274672.1 312 aa
Pseudomonadati FCB group Bacteroidota Chlorobiota group Bacteroidota Prolixibacteraceae bacterium MBP7506516.1 312 aa
Pseudomonadati FCB group Bacteroidota Chlorobiota group Bacteroidota Mariniphaga sediminis WP 372935074.1 357 aa
Pseudomonadati FCB group Bacteroidota Chlorobiota group Bacteroidota Prolixibacteraceae bacterium MBP7504730.1 313 aa
Pseudomonadati FCB group Bacteroidota Chlorobiota group Bacteroidota Prolixibacteraceae bacterium MBP6969297.1 313 aa
Pseudomonadati FCB group Bacteroidota Chlorobiota group Bacteroidota Paludibacteraceae bacterium MBQ7448886.1 315 aa
Pseudomonadati FCB group Bacteroidota Chlorobiota group Bacteroidota Dysgonamonadaceae bacterium MDR3061020.1 312 aa
Pseudomonadati FCB group Bacteroidota Chlorobiota group Bacteroidota Dysgonamonadaceae bacterium MDR1562902.1 312 aa
Pseudomonadati FCB group Bacteroidota Chlorobiota group Bacteroidota Dysgonamonadaceae bacterium MDR0427867.1 309 aa
Pseudomonadati FCB group Bacteroidota Chlorobiota group Bacteroidota Dysgonamonadaceae bacterium MDR0413327.1 309 aa
Pseudomonadati FCB group Bacteroidota Chlorobiota group Bacteroidota Porphyromonas gingivalis WP 004584679.1 313 aa
Pseudomonadati FCB group Bacteroidota Chlorobiota group Bacteroidota Porphyromonas miyakawae WP 411914969.1 314 aa
Pseudomonadati FCB group Bacteroidota Chlorobiota group Bacteroidota Porphyromonas endodontalis WP 276773577.1 313 aa
Pseudomonadati FCB group Bacteroidota Chlorobiota group Bacteroidota Porphyromonas macacae WP 282918332.1 315 aa
Pseudomonadati FCB group Bacteroidota Chlorobiota group Bacteroidota Porphyromonas circumdentaria WP 078736414.1 313 aa
Pseudomonadati FCB group Candidatus Zixiibacteriota candidate division OQX89825.1 319 aa
Pseudomonadati FCB group Bacteroidota Chlorobiota group Rhodothermota Rhodothermales bacterium MFT4603998.1 310 aa
Pseudomonadati FCB group Bacteroidota Chlorobiota group Rhodothermota Rhodothermus marinus WP 014067879.1 316 aa
Pseudomonadati FCB group Bacteroidota Chlorobiota group Rhodothermota Rhodothermales bacterium HEX7071370.1 327 aa
Pseudomonadati FCB group Bacteroidota Chlorobiota group Rhodothermota Rhodothermaceae bacterium MCA1801343.1 310 aa
Pseudomonadati FCB group Bacteroidota Chlorobiota group Balneolota Cyclonatronum sp. WP 291481376.1 309 aa
Pseudomonadati FCB group Bacteroidota Chlorobiota group Bacteroidota Balneolales bacterium MEX0928089.1 310 aa
Pseudomonadati FCB group Bacteroidota Chlorobiota group Bacteroidota Balneolales bacterium HKI45251.1 310 aa
Pseudomonadati FCB group Bacteroidota Chlorobiota group Balneolota Rhodohalobacter sp. WP 116037893.1 310 aa
Pseudomonadati FCB group Bacteroidota Chlorobiota group Balneolota Balneolaceae bacterium HYW34042.1 310 aa
Pseudomonadati FCB group Bacteroidota Chlorobiota group Bacteroidota Rhodohalobacter sp. WP 234570525.1 310 aa
Pseudomonadati FCB group Bacteroidota Chlorobiota group Bacteroidota Balneolaceae bacterium HLR91211.1 310 aa
Pseudomonadati FCB group Bacteroidota Chlorobiota group Balneolota Fodinibius WP 073063848.1 310 aa
Pseudomonadati FCB group Bacteroidota Chlorobiota group Bacteroidota Rhodohalobacter barkolensis WP 101073166.1 310 aa
Pseudomonadati FCB group Bacteroidota Chlorobiota group Bacteroidota Balneolaceae bacterium MEX1010721.1 310 aa
Pseudomonadati FCB group Bacteroidota Chlorobiota group Balneolota Balneola vulgaris WP 018128240.1 310 aa
Pseudomonadati FCB group Bacteroidota Chlorobiota group Balneolota Gracilimonas WP 255133262.1 310 aa
Pseudomonadati FCB group Bacteroidota Chlorobiota group Balneolota Gracilimonas aurantiaca WP 367733317.1 310 aa
Pseudomonadati FCB group Candidatus Zhuqueibacterota Candidatus Zhuqueibacterota MBN1996544.1 318 aa
Pseudomonadati FCB group Candidatus Zhuqueibacterota Candidatus Zhuqueibacterota MBN1541057.1 313 aa
Pseudomonadati FCB group Candidatus Zhuqueibacterota Candidatus Zhuqueibacterota MBD3384852.1 321 aa
Pseudomonadati FCB group Candidatus Zhuqueibacterota Candidatus Zhuqueibacterota MBD3374920.1 315 aa
Pseudomonadati FCB group Candidatus Zhuqueibacterota Candidatus Zhuqueibacterota OGC05596.1 320 aa
Pseudomonadati FCB group Candidatus Zhuqueibacterota Candidatus Zhuqueibacterota MBN2412306.1 317 aa
Pseudomonadati FCB group Candidatus Zhuqueibacterota Candidatus Zhuqueibacterota MDZ7369659.1 321 aa
Pseudomonadati FCB group Candidatus Zhuqueibacterota Candidatus Zhuqueibacterota RLD14057.1 320 aa
Pseudomonadati FCB group Candidatus Zhuqueibacterota Candidatus Zhuqueibacterota MBN1560751.1 322 aa
Pseudomonadati FCB group Candidatus Zhuqueibacterota Candidatus Zhuqueibacterota MBN1480468.1 322 aa
Pseudomonadati FCB group Candidatus Zhuqueibacterota Candidatus Oleimicrobiaceae MGQ9559039.1 314 aa
Pseudomonadati FCB group Candidatus Latescibacterota Candidatus Latescibacterota MCK4773059.1 311 aa
Pseudomonadati FCB group Candidatus Zhuqueibacterota Candidatus Zhuqueibacterota MCH8955497.1 311 aa
Pseudomonadati FCB group Candidatus Zhuqueibacterota Candidatus Zhuqueibacterota MEE4311517.1 311 aa
Pseudomonadati FCB group Candidatus Zhuqueibacterota Candidatus Zhuqueibacterota MBN2013431.1 313 aa
Pseudomonadati FCB group Candidatus Zhuqueibacterota Candidatus Zhuqueibacterota MBD3290701.1 312 aa
Pseudomonadati FCB group Candidatus Zhuqueibacterota Candidatus Zhuqueibacterota MDQ7066152.1 330 aa
Pseudomonadati FCB group Candidatus Zhuqueibacterota Candidatus Zhuqueibacterota MCA9742379.1 315 aa
Pseudomonadati FCB group Candidatus Zhuqueibacterota Candidatus Zhuqueibacter MDZ7360279.1 310 aa
Pseudomonadati FCB group Candidatus Zhuqueibacterota Candidatus Zhuqueibacter MDZ7289209.1 310 aa
Pseudomonadati FCB group Candidatus Zhuqueibacterota Candidatus Zhuqueibacterota RKY85612.1 309 aa
Pseudomonadati FCB group candidate division LCP 89 candidate division TKJ40383.1 312 aa
Pseudomonadati FCB group Candidatus Zhuqueibacterota Candidatus Zhuqueibacterota RKY80133.1 318 aa
Pseudomonadati FCB group Candidatus Zhuqueibacterota Candidatus Zhuqueibacterota MCH8126251.1 316 aa
Pseudomonadati FCB group Candidatus Zhuqueibacterota Candidatus Zhuqueibacterota MFZ5518252.1 311 aa
Pseudomonadati FCB group Candidatus Zhuqueibacterota Candidatus Zhuqueibacterota OQX95966.1 311 aa
Pseudomonadati FCB group Candidatus Zhuqueibacterota Candidatus Zhuqueibacterota OQX87869.1 311 aa
Pseudomonadati FCB group Bacteroidota Chlorobiota group Chlorobiales bacterium MBC8043302.1 313 aa
Pseudomonadati FCB group Bacteroidota Chlorobiota group Chlorobiota Chloroherpetonaceae bacterium MDX2127889.1 317 aa
Pseudomonadati FCB group Bacteroidota Chlorobiota group Chlorobiota Chloroherpetonaceae bacterium MCS6989989.1 317 aa
Pseudomonadati FCB group Bacteroidota Chlorobiota group Chlorobiota Chlorobium sp. PIO48996.1 312 aa
Pseudomonadati FCB group Bacteroidota Chlorobiota group Chlorobiota Candidatus Thermochlorobacteriaceae KER10327.1 312 aa
Pseudomonadati FCB group Bacteroidota Chlorobiota group Chlorobiota Chloroherpeton thalassium WP 012500818.1 314 aa
Pseudomonadati FCB group Bacteroidota Chlorobiota group Chlorobiota Chlorobiales bacterium NTW49043.1 313 aa
Pseudomonadati FCB group Bacteroidota Chlorobiota group Chlorobiota Chlorobaculum tepidum WP 010933029.1 323 aa
Pseudomonadati FCB group Bacteroidota Chlorobiota group Chlorobiota Chlorobaculum sp. HWR00492.1 323 aa
Pseudomonadati FCB group Bacteroidota Chlorobiota group Chlorobiota Prosthecochloris WP 012506045.1 317 aa
Pseudomonadati FCB group Bacteroidota Chlorobiota group Chlorobiota Chlorobium sp. WP 331561214.1 323 aa
Pseudomonadati FCB group Bacteroidota Chlorobiota group Chlorobiota Chlorobium phaeobacteroides WP 041467645.1 323 aa
Pseudomonadati FCB group Bacteroidota Chlorobiota group Chlorobiota Pelodictyon phaeoclathratiforme WP 012508366.1 323 aa
Pseudomonadati FCB group Bacteroidota Chlorobiota group Chlorobiota Chlorobium ferrooxidans WP 006366385.1 323 aa
Pseudomonadati FCB group Bacteroidota Chlorobiota group Ignavibacteriota Ignavibacteriaceae bacterium MBE2279847.1 320 aa
Pseudomonadati FCB group Bacteroidota Chlorobiota group Ignavibacteriota Ignavibacteriaceae bacterium NUM71950.1 321 aa
Pseudomonadati FCB group Bacteroidota Chlorobiota group Ignavibacteriota Ignavibacteriales bacterium MBK8663793.1 323 aa
Pseudomonadati FCB group Bacteroidota Chlorobiota group Chlorobiota Chlorobiota bacterium KAA0259789.1 323 aa
Pseudomonadati FCB group Bacteroidota Chlorobiota group Ignavibacteriota Ignavibacteriales bacterium MBI2417252.1 312 aa
Pseudomonadati FCB group Bacteroidota Chlorobiota group Ignavibacteriota Ignavibacteriaceae bacterium NUN08246.1 313 aa
Pseudomonadati FCB group Bacteroidota Chlorobiota group Ignavibacteriota Ignavibacteriaceae bacterium MFO7527133.1 314 aa
Pseudomonadati FCB group Bacteroidota Chlorobiota group Ignavibacteriota Ignavibacteriales bacterium MBK7228695.1 314 aa
Pseudomonadati FCB group Bacteroidota Chlorobiota group Ignavibacteriota Ignavibacteriaceae bacterium HMN48210.1 314 aa
Pseudomonadati FCB group Bacteroidota Chlorobiota group Ignavibacteriota Ignavibacteriaceae bacterium MDY0080344.1 314 aa
Pseudomonadati FCB group Bacteroidota Chlorobiota group Ignavibacteriota Ignavibacteriaceae bacterium MBV6513635.1 333 aa
Pseudomonadati FCB group Bacteroidota Chlorobiota group Ignavibacteriota Ignavibacteriaceae bacterium MBX2976779.1 314 aa
Pseudomonadati FCB group Bacteroidota Chlorobiota group Ignavibacteriota Ignavibacteriaceae bacterium HPI37233.1 314 aa
Pseudomonadati FCB group Bacteroidota Chlorobiota group Ignavibacteriota Ignavibacteriaceae bacterium MBZ0199286.1 313 aa
Pseudomonadati FCB group Bacteroidota Chlorobiota group Ignavibacteriota Ignavibacteria bacterium NCS87856.1 315 aa
Pseudomonadati FCB group Bacteroidota Chlorobiota group Ignavibacteriota Ignavibacteria bacterium MBT8386856.1 315 aa
Pseudomonadati FCB group Bacteroidota Chlorobiota group Ignavibacteriota Ignavibacteria bacterium GMR25422.1 315 aa
Pseudomonadati FCB group Bacteroidota Chlorobiota group Ignavibacteriota Ignavibacteriaceae bacterium NWG28345.1 315 aa
Pseudomonadati FCB group Bacteroidota Chlorobiota group Chlorobiota Chlorobiota bacterium KAA0243869.1 315 aa
Pseudomonadati FCB group Bacteroidota Chlorobiota group Ignavibacteriota Ignavibacteria bacterium HED07006.1 315 aa
Pseudomonadati FCB group Bacteroidota Chlorobiota group Ignavibacteriota Ignavibacteriales bacterium QQS36746.1 315 aa
Pseudomonadati FCB group Bacteroidota Chlorobiota group Ignavibacteriota Ignavibacteriaceae bacterium HMU42664.1 316 aa
Pseudomonadati FCB group Bacteroidota Chlorobiota group Ignavibacteriota Ignavibacteriaceae bacterium NWF50494.1 316 aa
Pseudomonadati FCB group Bacteroidota Chlorobiota group Ignavibacteriota Ignavibacteriaceae bacterium GAB4296633.1 315 aa
Pseudomonadati FCB group Bacteroidota Chlorobiota group Ignavibacteriota Ignavibacteriaceae bacterium MFO7446369.1 315 aa
Pseudomonadati FCB group Bacteroidota Chlorobiota group Ignavibacteriota Ignavibacteriales bacterium RPI73647.1 313 aa
Pseudomonadati FCB group Bacteroidota Chlorobiota group Ignavibacteriota Ignavibacteria bacterium OGU29854.1 315 aa

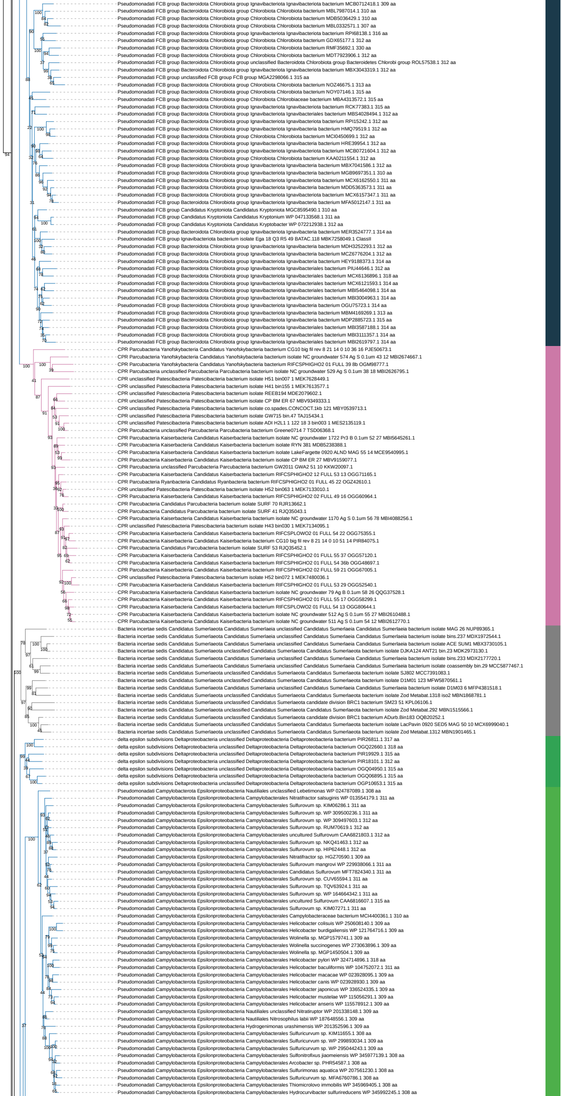

Pseudomonadati FCB group Bacteroidota Chlorobiota group Ignavibacteriota Ignavibacteriota bacterium MCB0712418.1 309 aa
Pseudomonadati FCB group Bacteroidota Chlorobiota group Chlorobiota Chlorobiota bacterium MBL7987014.1 310 aa
Pseudomonadati FCB group Bacteroidota Chlorobiota group Chlorobiota Chlorobiota bacterium MDB5036429.1 310 aa
Pseudomonadati FCB group Bacteroidota Chlorobiota group Chlorobiota Chlorobiota bacterium MBL0332571.1 307 aa
Pseudomonadati FCB group Bacteroidota Chlorobiota group Ignavibacteriota Ignavibacteriota bacterium RPI68138.1 316 aa
Pseudomonadati FCB group Bacteroidota Chlorobiota group Chlorobiota Chlorobiota bacterium GDX65177.1 312 aa
Pseudomonadati FCB group Bacteroidota Chlorobiota group Chlorobiota Chlorobiota bacterium RMF35692.1 330 aa
Pseudomonadati FCB group Bacteroidota Chlorobiota group Chlorobiota Chlorobiota bacterium MDT7923906.1 312 aa
Pseudomonadati FCB group Bacteroidota Chlorobiota group unclassified Bacteroidota Chlorobiota group Bacteroidetes Chlorobi group ROL57538.1 312 aa
Pseudomonadati FCB group Bacteroidota Chlorobiota group Ignavibacteriota Ignavibacteriota bacterium MBX3043319.1 312 aa
Pseudomonadati FCB group unclassified FCB group FCB group MGA2298066.1 315 aa
Pseudomonadati FCB group Bacteroidota Chlorobiota group Chlorobiota Chlorobiota bacterium NOZ46675.1 313 aa
Pseudomonadati FCB group Bacteroidota Chlorobiota group Chlorobiota Chlorobiota bacterium NOY07146.1 315 aa
Pseudomonadati FCB group Bacteroidota Chlorobiota group Chlorobiota Chlorobiaceae bacterium MBA4313572.1 315 aa
Pseudomonadati FCB group Bacteroidota Chlorobiota group Ignavibacteriota Ignavibacteriota bacterium RCK77383.1 315 aa
Pseudomonadati FCB group Bacteroidota Chlorobiota group Ignavibacteriota Ignavibacteriales bacterium MBS4028494.1 312 aa
Pseudomonadati FCB group Bacteroidota Chlorobiota group Ignavibacteriota Ignavibacteriota bacterium RPI15242.1 312 aa
Pseudomonadati FCB group Bacteroidota Chlorobiota group Ignavibacteriota Ignavibacteria bacterium HMQ79519.1 312 aa
Pseudomonadati FCB group Bacteroidota Chlorobiota group Chlorobiota Chlorobiota bacterium MCI0450699.1 312 aa
Pseudomonadati FCB group Bacteroidota Chlorobiota group Ignavibacteriota Ignavibacteria bacterium HRE39954.1 312 aa
Pseudomonadati FCB group Bacteroidota Chlorobiota group Ignavibacteriota Ignavibacteria bacterium MCB0721604.1 312 aa
Pseudomonadati FCB group Bacteroidota Chlorobiota group Chlorobiota Chlorobiota bacterium KAA0211554.1 312 aa
Pseudomonadati FCB group Bacteroidota Chlorobiota group Ignavibacteriota Ignavibacteria bacterium MBX7041586.1 312 aa
Pseudomonadati FCB group Bacteroidota Chlorobiota group Ignavibacteriota Ignavibacteria bacterium MGB9697351.1 310 aa
Pseudomonadati FCB group Bacteroidota Chlorobiota group Ignavibacteriota Ignavibacteriota bacterium MCX6162550.1 311 aa
Pseudomonadati FCB group Bacteroidota Chlorobiota group Ignavibacteriota Ignavibacteria bacterium MDD5363573.1 311 aa
Pseudomonadati FCB group Bacteroidota Chlorobiota group Ignavibacteriota Ignavibacteriota bacterium MCX6157347.1 311 aa
Pseudomonadati FCB group Bacteroidota Chlorobiota group Ignavibacteriota Ignavibacteria bacterium MFA5012147.1 311 aa
Pseudomonadati FCB group Candidatus Kryptoniota Candidatus Kryptoniota MGC8595490.1 310 aa
Pseudomonadati FCB group Candidatus Kryptoniota Candidatus Kryptonium WP 047133568.1 311 aa
Pseudomonadati FCB group Candidatus Kryptoniota Candidatus Kryptobacter WP 072212938.1 312 aa
Pseudomonadati FCB group Bacteroidota Chlorobiota group Ignavibacteriota Ignavibacteria bacterium MER3524777.1 314 aa
Pseudomonadati FCB group Ignavibacteriota bacterium isolate Ega 18 Q3 R5 49 BATAC.118 MBK7258049.1 ClassII
Pseudomonadati FCB group Bacteroidota Chlorobiota group Ignavibacteriota Ignavibacteria bacterium MDH3252293.1 312 aa
Pseudomonadati FCB group Bacteroidota Chlorobiota group Ignavibacteriota Ignavibacteria bacterium MCZ6776204.1 312 aa
Pseudomonadati FCB group Bacteroidota Chlorobiota group Ignavibacteriota Ignavibacteria bacterium HEY9188373.1 314 aa
Pseudomonadati FCB group Bacteroidota Chlorobiota group Ignavibacteriota Ignavibacteriales bacterium PIU44646.1 312 aa
Pseudomonadati FCB group Bacteroidota Chlorobiota group Ignavibacteriota Ignavibacteriales bacterium MCX6136896.1 318 aa
Pseudomonadati FCB group Bacteroidota Chlorobiota group Ignavibacteriota Ignavibacteriales bacterium MCX6121593.1 314 aa
Pseudomonadati FCB group Bacteroidota Chlorobiota group Ignavibacteriota Ignavibacteriales bacterium MBI5464098.1 314 aa
Pseudomonadati FCB group Bacteroidota Chlorobiota group Ignavibacteriota Ignavibacteriales bacterium MBI3004963.1 314 aa
Pseudomonadati FCB group Bacteroidota Chlorobiota group Ignavibacteriota Ignavibacteria bacterium OGU75723.1 314 aa
Pseudomonadati FCB group Bacteroidota Chlorobiota group Ignavibacteriota Ignavibacteria bacterium MBM4169269.1 313 aa
Pseudomonadati FCB group Bacteroidota Chlorobiota group Ignavibacteriota Ignavibacteria bacterium MDP2885723.1 315 aa
Pseudomonadati FCB group Bacteroidota Chlorobiota group Ignavibacteriota Ignavibacteriales bacterium MBI3587188.1 314 aa
Pseudomonadati FCB group Bacteroidota Chlorobiota group Ignavibacteriota Ignavibacteriales bacterium MBI3111357.1 314 aa
Pseudomonadati FCB group Bacteroidota Chlorobiota group Ignavibacteriota Ignavibacteriales bacterium MBI2619797.1 314 aa
CPR Parcubacteria Yanofskybacteria Candidatus Yanofskybacteria bacterium CG10 big fil rev 8 21 14 0 10 36 16 PJE50673.1
CPR Parcubacteria Yanofskybacteria Candidatus Yanofskybacteria bacterium isolate NC groundwater 574 Ag S 0.1um 43 12 MBI2674667.1
CPR Parcubacteria Yanofskybacteria Candidatus Yanofskybacteria bacterium RIFCSPHIGHO2 01 FULL 39 8b OGM98777.1
CPR Parcubacteria unclassified Parcubacteria Parcubacteria bacterium isolate NC groundwater 529 Ag S 0.1um 38 18 MBI2626795.1
CPR unclassified Patescibacteria Patescibacteria bacterium isolate H51 bin007 1 MEK7628449.1
CPR unclassified Patescibacteria Patescibacteria bacterium isolate H41 bin155 1 MEK7613577.1
CPR unclassified Patescibacteria Patescibacteria bacterium isolate REEB194 MDE2079602.1
CPR unclassified Patescibacteria Patescibacteria bacterium isolate CP BM ER 67 MBV9349333.1
CPR unclassified Patescibacteria Patescibacteria bacterium isolate co.spades.CONCOCT.1kb 121 MBY0539713.1
CPR unclassified Patescibacteria Patescibacteria bacterium isolate GW715 bin.47 TAJ15434.1
CPR unclassified Patescibacteria Patescibacteria bacterium isolate ADI H2L1 1 122 18 3 bin003 1 MES2135119.1
CPR Parcubacteria unclassified Parcubacteria Parcubacteria bacterium Greene0714 7 TSD06368.1
CPR Parcubacteria Kaiserbacteria Candidatus Kaiserbacteria bacterium isolate NC groundwater 1722 Pr3 B 0.1um 52 27 MBI5645261.1
CPR Parcubacteria Kaiserbacteria Candidatus Kaiserbacteria bacterium isolate RYN 381 MDB5238388.1
CPR Parcubacteria Kaiserbacteria Candidatus Kaiserbacteria bacterium isolate LakeFargette 0920 ALND MAG 55 14 MCE9540995.1
CPR Parcubacteria Kaiserbacteria Candidatus Kaiserbacteria bacterium isolate CP BM ER 27 MBV9159077.1
CPR Parcubacteria unclassified Parcubacteria Parcubacteria bacterium GW2011 GWA2 51 10 KKW20097.1
CPR Parcubacteria Kaiserbacteria Candidatus Kaiserbacteria bacterium RIFCSPHIGHO2 12 FULL 53 13 OGG71165.1
CPR Parcubacteria Ryanbacteria Candidatus Ryanbacteria bacterium RIFCSPHIGHO2 01 FULL 45 22 OGZ42610.1
CPR unclassified Patescibacteria Patescibacteria bacterium isolate H52 bin063 1 MEK7133010.1
CPR Parcubacteria Kaiserbacteria Candidatus Kaiserbacteria bacterium RIFCSPHIGHO2 02 FULL 49 16 OGG60964.1
CPR Parcubacteria Candidatus Parcubacteria bacterium isolate SURF 70 RJR13662.1
CPR Parcubacteria Candidatus Parcubacteria bacterium isolate SURF 41 RJQ35043.1
CPR Parcubacteria Kaiserbacteria Candidatus Kaiserbacteria bacterium isolate NC groundwater 1170 Ag S 0.1um 56 78 MBI4088256.1
CPR unclassified Patescibacteria Patescibacteria bacterium isolate H43 bin030 1 MEK7134095.1
CPR Parcubacteria Kaiserbacteria Candidatus Kaiserbacteria bacterium RIFCSPLOWO2 01 FULL 54 22 OGG75355.1
CPR Parcubacteria Kaiserbacteria Candidatus Kaiserbacteria bacterium CG10 big fil rev 8 21 14 0 10 51 14 PIR84075.1
CPR Parcubacteria Candidatus Parcubacteria bacterium isolate SURF 53 RJQ35452.1
CPR Parcubacteria Kaiserbacteria Candidatus Kaiserbacteria bacterium RIFCSPHIGHO2 01 FULL 55 37 OGG57120.1
CPR Parcubacteria Kaiserbacteria Candidatus Kaiserbacteria bacterium RIFCSPHIGHO2 01 FULL 54 36b OGG48697.1
CPR Parcubacteria Kaiserbacteria Candidatus Kaiserbacteria bacterium RIFCSPHIGHO2 02 FULL 59 21 OGG67005.1
CPR unclassified Patescibacteria Patescibacteria bacterium isolate H52 bin072 1 MEK7480036.1
CPR Parcubacteria Kaiserbacteria Candidatus Kaiserbacteria bacterium RIFCSPHIGHO2 01 FULL 53 29 OGG52540.1
CPR Parcubacteria Kaiserbacteria Candidatus Kaiserbacteria bacterium isolate NC groundwater 79 Ag B 0.1um 58 26 QQG37528.1
CPR Parcubacteria Kaiserbacteria Candidatus Kaiserbacteria bacterium RIFCSPHIGHO2 01 FULL 55 17 OGG58299.1
CPR Parcubacteria Kaiserbacteria Candidatus Kaiserbacteria bacterium RIFCSPLOWO2 01 FULL 54 13 OGG80644.1
CPR Parcubacteria Kaiserbacteria Candidatus Kaiserbacteria bacterium isolate NC groundwater 512 Ag S 0.1um 55 27 MBI2610488.1
CPR Parcubacteria Kaiserbacteria Candidatus Kaiserbacteria bacterium isolate NC groundwater 511 Ag S 0.1um 54 12 MBI2612770.1
Bacteria incertae sedis Candidatus Sumerlaeota Candidatus Sumerlaeia unclassified Candidatus Sumerlaeia Candidatus Sumerlaeia bacterium isolate MAG 26 NUP89365.1
Bacteria incertae sedis Candidatus Sumerlaeota Candidatus Sumerlaeia unclassified Candidatus Sumerlaeia Candidatus Sumerlaeia bacterium isolate bins.237 MDX1972544.1
Bacteria incertae sedis Candidatus Sumerlaeota Candidatus Sumerlaeia unclassified Candidatus Sumerlaeia Candidatus Sumerlaeia bacterium isolate ACE SUM1 MBX3730105.1
Bacteria incertae sedis Candidatus Sumerlaeota unclassified Candidatus Sumerlaeota Candidatus Sumerlaeota bacterium isolate DJKA124 ANT21 bin.23 MDK2973130.1
Bacteria incertae sedis Candidatus Sumerlaeota Candidatus Sumerlaeia unclassified Candidatus Sumerlaeia Candidatus Sumerlaeia bacterium isolate bins.233 MDX2177720.1
Bacteria incertae sedis Candidatus Sumerlaeota Candidatus Sumerlaeia unclassified Candidatus Sumerlaeia Candidatus Sumerlaeia bacterium isolate coassembly bin.29 MCC5877467.1
Bacteria incertae sedis Candidatus Sumerlaeota unclassified Candidatus Sumerlaeota Candidatus Sumerlaeota bacterium isolate SJ802 MCC7391083.1
Bacteria incertae sedis Candidatus Sumerlaeota unclassified Candidatus Sumerlaeota Candidatus Sumerlaeota bacterium isolate D1M01 123 MFW5870561.1
Bacteria incertae sedis Candidatus Sumerlaeota Candidatus Sumerlaeia unclassified Candidatus Sumerlaeia Candidatus Sumerlaeia bacterium isolate D1M03 6 MFP4381518.1
Bacteria incertae sedis Candidatus Sumerlaeota unclassified Candidatus Sumerlaeota Candidatus Sumerlaeota bacterium isolate Zod Metabat.1318 iso2 MBN1868781.1
Bacteria incertae sedis Candidatus Sumerlaeota unclassified Candidatus Sumerlaeota candidate division BRC1 bacterium SM23 51 KPL06106.1
Bacteria incertae sedis Candidatus Sumerlaeota unclassified Candidatus Sumerlaeota Candidatus Sumerlaeota bacterium isolate Zod Metabat.292 MBN1515566.1
Bacteria incertae sedis Candidatus Sumerlaeota unclassified Candidatus Sumerlaeota candidate division BRC1 bacterium ADurb.Bin183 OQB20252.1
Bacteria incertae sedis Candidatus Sumerlaeota unclassified Candidatus Sumerlaeota Candidatus Sumerlaeota bacterium isolate LacPavin 0920 SED5 MAG 50 10 MCX6999040.1
Bacteria incertae sedis Candidatus Sumerlaeota unclassified Candidatus Sumerlaeota Candidatus Sumerlaeota bacterium isolate Zod Metabat.1312 MBN1901465.1
delta epsilon subdivisions Deltaproteobacteria unclassified Deltaproteobacteria Deltaproteobacteria bacterium PIR26811.1 317 aa
delta epsilon subdivisions Deltaproteobacteria unclassified Deltaproteobacteria Deltaproteobacteria bacterium OGQ22660.1 318 aa
delta epsilon subdivisions Deltaproteobacteria unclassified Deltaproteobacteria Deltaproteobacteria bacterium PIR19929.1 315 aa
delta epsilon subdivisions Deltaproteobacteria unclassified Deltaproteobacteria Deltaproteobacteria bacterium PIR18101.1 312 aa
delta epsilon subdivisions Deltaproteobacteria unclassified Deltaproteobacteria Deltaproteobacteria bacterium OGQ04950.1 315 aa
delta epsilon subdivisions Deltaproteobacteria unclassified Deltaproteobacteria Deltaproteobacteria bacterium OGQ06895.1 315 aa
delta epsilon subdivisions Deltaproteobacteria unclassified Deltaproteobacteria Deltaproteobacteria bacterium OGP10653.1 315 aa
Pseudomonadati Campylobacterota Epsilonproteobacteria Nautiliales unclassified Lebetimonas WP 024787089.1 308 aa
Pseudomonadati Campylobacterota Epsilonproteobacteria Campylobacterales Nitratifractor salsuginis WP 013554179.1 311 aa
Pseudomonadati Campylobacterota Epsilonproteobacteria Campylobacterales Sulfurovum sp. KIM06286.1 311 aa
Pseudomonadati Campylobacterota Epsilonproteobacteria Campylobacterales Sulfurovum sp. WP 309500236.1 311 aa
Pseudomonadati Campylobacterota Epsilonproteobacteria Campylobacterales Sulfurovum sp. WP 309497603.1 312 aa
Pseudomonadati Campylobacterota Epsilonproteobacteria Campylobacterales Sulfurovum sp. RUM70619.1 312 aa
Pseudomonadati Campylobacterota Epsilonproteobacteria Campylobacterales uncultured Sulfurovum CAA6821803.1 312 aa
Pseudomonadati Campylobacterota Epsilonproteobacteria Campylobacterales Sulfurovum sp. NKQ41463.1 312 aa
Pseudomonadati Campylobacterota Epsilonproteobacteria Campylobacterales Sulfurovum sp. HIP62448.1 312 aa
Pseudomonadati Campylobacterota Epsilonproteobacteria Campylobacterales Nitratifractor sp. HGZ70590.1 309 aa
Pseudomonadati Campylobacterota Epsilonproteobacteria Campylobacterales Sulfurovum mangrovi WP 229938066.1 311 aa
Pseudomonadati Campylobacterota Epsilonproteobacteria Campylobacterales Candidatus Sulfurovum MFT7824340.1 311 aa
Pseudomonadati Campylobacterota Epsilonproteobacteria Campylobacterales Sulfurovum sp. CUV65594.1 311 aa
Pseudomonadati Campylobacterota Epsilonproteobacteria Campylobacterales Sulfurovum sp. TQV63924.1 311 aa
Pseudomonadati Campylobacterota Epsilonproteobacteria Campylobacterales Sulfurovum sp. WP 164664342.1 311 aa
Pseudomonadati Campylobacterota Epsilonproteobacteria Campylobacterales uncultured Sulfurovum CAA6816607.1 315 aa
Pseudomonadati Campylobacterota Epsilonproteobacteria Campylobacterales Sulfurovum sp. KIM07271.1 311 aa
Pseudomonadati Campylobacterota Epsilonproteobacteria Campylobacterales Campylobacteraceae bacterium MCI4400361.1 310 aa
Pseudomonadati Campylobacterota Epsilonproteobacteria Campylobacterales Helicobacter colisuis WP 250608140.1 309 aa
Pseudomonadati Campylobacterota Epsilonproteobacteria Campylobacterales Helicobacter burdigaliensis WP 121764716.1 309 aa
Pseudomonadati Campylobacterota Epsilonproteobacteria Campylobacterales Wolinella sp. MGP1579741.1 309 aa
Pseudomonadati Campylobacterota Epsilonproteobacteria Campylobacterales Wolinella succinogenes WP 273063896.1 309 aa
Pseudomonadati Campylobacterota Epsilonproteobacteria Campylobacterales Wolinella sp. MGP1450504.1 309 aa
Pseudomonadati Campylobacterota Epsilonproteobacteria Campylobacterales Helicobacter pylori WP 324714896.1 318 aa
Pseudomonadati Campylobacterota Epsilonproteobacteria Campylobacterales Helicobacter baculiformis WP 104752072.1 311 aa
Pseudomonadati Campylobacterota Epsilonproteobacteria Campylobacterales Helicobacter macacae WP 023928095.1 309 aa
Pseudomonadati Campylobacterota Epsilonproteobacteria Campylobacterales Helicobacter canis WP 023928930.1 309 aa
Pseudomonadati Campylobacterota Epsilonproteobacteria Campylobacterales Helicobacter japonicus WP 336524335.1 309 aa
Pseudomonadati Campylobacterota Epsilonproteobacteria Campylobacterales Helicobacter mustelae WP 115056291.1 309 aa
Pseudomonadati Campylobacterota Epsilonproteobacteria Campylobacterales Helicobacter anseris WP 115578912.1 309 aa
Pseudomonadati Campylobacterota Epsilonproteobacteria Nautiliales unclassified Nitratiruptor WP 201338148.1 309 aa
Pseudomonadati Campylobacterota Epsilonproteobacteria Nautiliales Nitrosophilus labii WP 187648556.1 309 aa
Pseudomonadati Campylobacterota Epsilonproteobacteria Hydrogenimonas urashimensis WP 201352596.1 309 aa
Pseudomonadati Campylobacterota Epsilonproteobacteria Campylobacterales Sulfuricurvum sp. KIM11655.1 308 aa
Pseudomonadati Campylobacterota Epsilonproteobacteria Campylobacterales Sulfuricurvum sp. WP 299893034.1 309 aa
Pseudomonadati Campylobacterota Epsilonproteobacteria Campylobacterales Sulfuricurvum sp. WP 295044243.1 309 aa
Pseudomonadati Campylobacterota Epsilonproteobacteria Campylobacterales Sulfonitrofixus jiaomeiensis WP 345977139.1 308 aa
Pseudomonadati Campylobacterota Epsilonproteobacteria Campylobacterales Arcobacter sp. PHR54587.1 308 aa
Pseudomonadati Campylobacterota Epsilonproteobacteria Campylobacterales Sulfurimonas aquatica WP 207561230.1 308 aa
Pseudomonadati Campylobacterota Epsilonproteobacteria Campylobacterales Sulfuricurvum sp. MFA6760786.1 308 aa
Pseudomonadati Campylobacterota Epsilonproteobacteria Campylobacterales Thiomicrolovo immobilis WP 345969405.1 308 aa
Pseudomonadati Campylobacterota Epsilonproteobacteria Campylobacterales Hydrocurvibacter sulfurireducens WP 345992245.1 308 aa

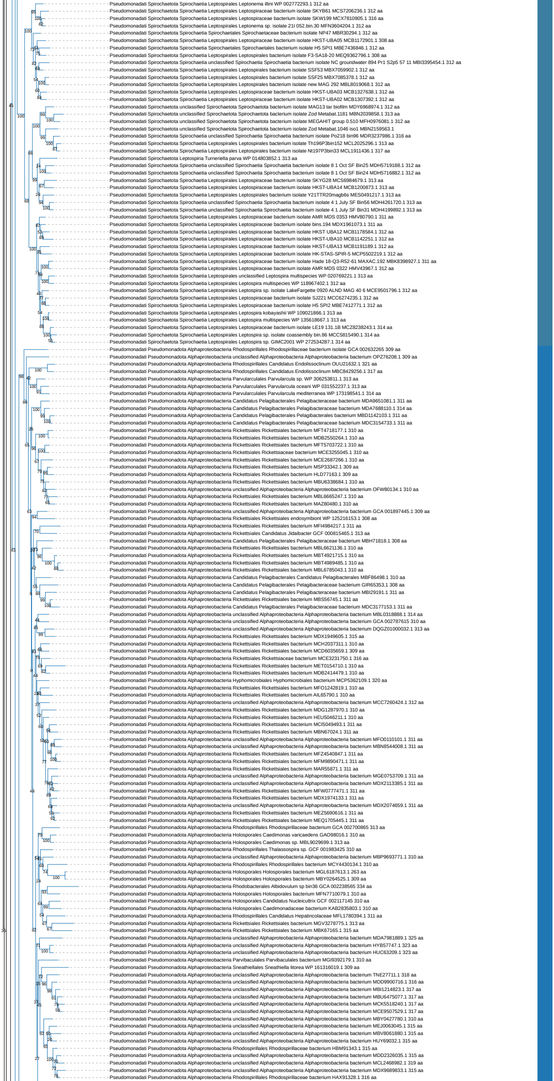

Pseudomonadati Spirochaetota Spirochaetia Leptospirales Leptonema illini WP 002772293.1 312 aa
Pseudomonadati Spirochaetota Spirochaetia Leptospirales Leptospiraceae bacterium isolate SKYB61 MCS7206236.1 312 aa
Pseudomonadati Spirochaetota Spirochaetia Leptospirales Leptospiraceae bacterium isolate SKW199 MCX7810905.1 316 aa
Pseudomonadati Spirochaetota Spirochaetia Leptospirales Leptonema sp. isolate 21I 052.bin.30 MFN3604204.1 312 aa
Pseudomonadati Spirochaetota Spirochaetia Spirochaetales Spirochaetaceae bacterium isolate NP47 MBR30294.1 312 aa
Pseudomonadati Spirochaetota Spirochaetia Leptospirales Leptospiraceae bacterium isolate HKST-UBA05 MCB1172901.1 308 aa
Pseudomonadati Spirochaetota Spirochaetia Spirochaetales Spirochaetales bacterium isolate H5 SPI1 MBE7436846.1 312 aa
Pseudomonadati Spirochaetota Spirochaetia Leptospirales Leptospirales bacterium isolate F3-SA18-20 MEQ9362796.1 308 aa
Pseudomonadati Spirochaetota Spirochaetia unclassified Spirochaetia Spirochaetia bacterium isolate NC groundwater 894 Pr1 S2p5 57 11 MBI3395454.1 312 aa
Pseudomonadati Spirochaetota Spirochaetia Leptospirales Leptospirales bacterium isolate SSF53 MBX7059902.1 312 aa
Pseudomonadati Spirochaetota Spirochaetia Leptospirales Leptospirales bacterium isolate SSF25 MBX7085378.1 312 aa
Pseudomonadati Spirochaetota Spirochaetia Leptospirales Leptospirales bacterium isolate new MAG 292 MBL8019068.1 312 aa
Pseudomonadati Spirochaetota Spirochaetia Leptospirales Leptospiraceae bacterium isolate HKST-UBA03 MCB1327638.1 312 aa
Pseudomonadati Spirochaetota Spirochaetia Leptospirales Leptospiraceae bacterium isolate HKST-UBA02 MCB1307392.1 312 aa
Pseudomonadati Spirochaetota unclassified Spirochaetota Spirochaetota bacterium isolate MAG13 tar biofilm MDY6968974.1 312 aa
Pseudomonadati Spirochaetota unclassified Spirochaetota Spirochaetota bacterium isolate Zod Metabat.1181 MBN2039858.1 313 aa
Pseudomonadati Spirochaetota unclassified Spirochaetota Spirochaetota bacterium isolate MEGAHIT group 0.510 MFH0976081.1 312 aa
Pseudomonadati Spirochaetota unclassified Spirochaetota Spirochaetota bacterium isolate Zod Metabat.1046 iso1 MBN2159563.1
Pseudomonadati Spirochaetota Spirochaetia unclassified Spirochaetia Spirochaetia bacterium isolate Po218 bin96 MDR3237986.1 316 aa
Pseudomonadati Spirochaetota Spirochaetia Leptospirales Leptospirales bacterium isolate Th196P3bin152 MCL2025296.1 313 aa
Pseudomonadati Spirochaetota Spirochaetia Leptospirales Leptospirales bacterium isolate Nt197P3bin33 MCL1911436.1 317 aa
Pseudomonadati Spirochaetota Leptospiria Turneriella parva WP 014803852.1 313 aa
Pseudomonadati Spirochaetota Spirochaetia unclassified Spirochaetia Spirochaetia bacterium isolate 8 1 Oct SF Bin25 MDH5719188.1 312 aa
Pseudomonadati Spirochaetota Spirochaetia unclassified Spirochaetia Spirochaetia bacterium isolate 8 1 Oct SF Bin24 MDH5716882.1 312 aa
Pseudomonadati Spirochaetota Spirochaetia Leptospirales Leptospiraceae bacterium isolate SKYG28 MCS6984679.1 313 aa
Pseudomonadati Spirochaetota Spirochaetia Leptospirales Leptospiraceae bacterium isolate HKST-UBA14 MCB1200873.1 313 aa
Pseudomonadati Spirochaetota Spirochaetia Leptospirales Leptospiraceae bacterium isolate Y21TTR20magb6s MES0491217.1 313 aa
Pseudomonadati Spirochaetota Spirochaetia unclassified Spirochaetia Spirochaetia bacterium isolate 4 1 July SF Bin56 MDH4261720.1 313 aa
Pseudomonadati Spirochaetota Spirochaetia unclassified Spirochaetia Spirochaetia bacterium isolate 4 1 July SF Bin31 MDH4199892.1 313 aa
Pseudomonadati Spirochaetota Spirochaetia Leptospirales Leptospiraceae bacterium isolate AMR MDS 0353 HMV80790.1 311 aa
Pseudomonadati Spirochaetota Spirochaetia Leptospirales Leptospiraceae bacterium isolate bins.194 MDX1961073.1 311 aa
Pseudomonadati Spirochaetota Spirochaetia Leptospirales Leptospiraceae bacterium isolate HKST UBA12 MCB1178584.1 312 aa
Pseudomonadati Spirochaetota Spirochaetia Leptospirales Leptospiraceae bacterium isolate HKST-UBA10 MCB1142251.1 312 aa
Pseudomonadati Spirochaetota Spirochaetia Leptospirales Leptospiraceae bacterium isolate HKST-UBA13 MCB1191189.1 312 aa
Pseudomonadati Spirochaetota Spirochaetia Leptospirales Leptospiraceae bacterium isolate HK-STAS-SPIR-5 MCP5502219.1 312 aa
Pseudomonadati Spirochaetota Spirochaetia Leptospirales Leptospiraceae bacterium isolate Hade 18-Q3-R52-61 MAXAC.192 MBK8398927.1 311 aa
Pseudomonadati Spirochaetota Spirochaetia Leptospirales Leptospiraceae bacterium isolate AMR MDS 0322 HMV43967.1 312 aa
Pseudomonadati Spirochaetota Spirochaetia Leptospirales unclassified Leptospira multispecies WP 020769221.1 313 aa
Pseudomonadati Spirochaetota Spirochaetia Leptospirales Leptospira multispecies WP 118967402.1 312 aa
Pseudomonadati Spirochaetota Spirochaetia Leptospirales Leptospira sp. isolate LakeFargette 0920 ALND MAG 40 6 MCE9501796.1 312 aa
Pseudomonadati Spirochaetota Spirochaetia Leptospirales Leptospiraceae bacterium isolate SJ221 MCC6274235.1 312 aa
Pseudomonadati Spirochaetota Spirochaetia Leptospirales Leptospiraceae bacterium isolate H5 SPI2 MBE7412771.1 312 aa
Pseudomonadati Spirochaetota Spirochaetia Leptospirales Leptospira kobayashii WP 109021866.1 313 aa
Pseudomonadati Spirochaetota Spirochaetia Leptospirales Leptospira multispecies WP 135618667.1 313 aa
Pseudomonadati Spirochaetota Spirochaetia Leptospirales Leptospiraceae bacterium isolate LE19 131.1B MCZ8238243.1 314 aa
Pseudomonadati Spirochaetota Spirochaetia Leptospirales Leptospira sp. isolate coassembly bin.86 MCC5815490.1 314 aa
Pseudomonadati Spirochaetota Spirochaetia Leptospirales Leptospira sp. GIMC2001 WP 272534287.1 314 aa
Pseudomonadati Pseudomonadota Alphaproteobacteria Rhodospirillales Rhodospirillaceae bacterium isolate GCA 002632265 309 aa
Pseudomonadati Pseudomonadota Alphaproteobacteria unclassified Alphaproteobacteria Alphaproteobacteria bacterium OPZ76208.1 309 aa
Pseudomonadati Pseudomonadota Alphaproteobacteria Rhodospirillales Candidatus Endolissoclinum OUU21632.1 321 aa
Pseudomonadati Pseudomonadota Alphaproteobacteria Rhodospirillales Candidatus Endolissoclinum MBC8429256.1 317 aa
Pseudomonadati Pseudomonadota Alphaproteobacteria Parvularculales Parvularcula sp. WP 306253811.1 313 aa
Pseudomonadati Pseudomonadota Alphaproteobacteria Parvularculales Parvularcula oceani WP 031552237.1 313 aa
Pseudomonadati Pseudomonadota Alphaproteobacteria Parvularculales Parvularcula mediterranea WP 173198541.1 314 aa
Pseudomonadati Pseudomonadota Alphaproteobacteria Candidatus Pelagibacterales Pelagibacteraceae bacterium MDA9651081.1 311 aa
Pseudomonadati Pseudomonadota Alphaproteobacteria Candidatus Pelagibacterales Pelagibacteraceae bacterium MDA7688110.1 314 aa
Pseudomonadati Pseudomonadota Alphaproteobacteria Candidatus Pelagibacterales Pelagibacterales bacterium MBD1142103.1 311 aa
Pseudomonadati Pseudomonadota Alphaproteobacteria Candidatus Pelagibacterales Pelagibacteraceae bacterium MDC3154733.1 311 aa
Pseudomonadati Pseudomonadota Alphaproteobacteria Rickettsiales Rickettsiales bacterium MFT4718177.1 310 aa
Pseudomonadati Pseudomonadota Alphaproteobacteria Rickettsiales Rickettsiales bacterium MDB2550264.1 310 aa
Pseudomonadati Pseudomonadota Alphaproteobacteria Rickettsiales Rickettsiales bacterium MFT5703722.1 310 aa
Pseudomonadati Pseudomonadota Alphaproteobacteria Rickettsiales Rickettsiaceae bacterium MCE3255045.1 310 aa
Pseudomonadati Pseudomonadota Alphaproteobacteria Rickettsiales Rickettsiales bacterium MCE2687266.1 310 aa
Pseudomonadati Pseudomonadota Alphaproteobacteria Rickettsiales Rickettsiales bacterium MSP33342.1 309 aa
Pseudomonadati Pseudomonadota Alphaproteobacteria Rickettsiales Rickettsiales bacterium HLD77163.1 309 aa
Pseudomonadati Pseudomonadota Alphaproteobacteria Rickettsiales Rickettsiales bacterium MBU6338684.1 310 aa
Pseudomonadati Pseudomonadota Alphaproteobacteria unclassified Alphaproteobacteria Alphaproteobacteria bacterium OFW80134.1 310 aa
Pseudomonadati Pseudomonadota Alphaproteobacteria Rickettsiales Rickettsiales bacterium MBL6665247.1 310 aa
Pseudomonadati Pseudomonadota Alphaproteobacteria Rickettsiales Rickettsiales bacterium MAZ80480.1 310 aa
Pseudomonadati Pseudomonadota Alphaproteobacteria unclassified Alphaproteobacteria Alphaproteobacteria bacterium GCA 001897445.1 309 aa
Pseudomonadati Pseudomonadota Alphaproteobacteria Rickettsiales Rickettsiales endosymbiont WP 125216153.1 308 aa
Pseudomonadati Pseudomonadota Alphaproteobacteria Rickettsiales Rickettsiales bacterium MFI4984217.1 311 aa
Pseudomonadati Pseudomonadota Alphaproteobacteria Rickettsiales Candidatus Jidaibacter GCF 000815465.1 313 aa
Pseudomonadati Pseudomonadota Alphaproteobacteria Candidatus Pelagibacterales Pelagibacteraceae bacterium MBH71818.1 308 aa
Pseudomonadati Pseudomonadota Alphaproteobacteria Rickettsiales Rickettsiales bacterium MBL6621136.1 310 aa
Pseudomonadati Pseudomonadota Alphaproteobacteria Rickettsiales Rickettsiales bacterium MBT4921715.1 310 aa
Pseudomonadati Pseudomonadota Alphaproteobacteria Rickettsiales Rickettsiales bacterium MBT4989485.1 310 aa
Pseudomonadati Pseudomonadota Alphaproteobacteria Rickettsiales Rickettsiales bacterium MBL6785043.1 310 aa
Pseudomonadati Pseudomonadota Alphaproteobacteria Candidatus Pelagibacterales Candidatus Pelagibacterales MBF86498.1 310 aa
Pseudomonadati Pseudomonadota Alphaproteobacteria Candidatus Pelagibacterales Pelagibacteraceae bacterium GIR65353.1 308 aa
Pseudomonadati Pseudomonadota Alphaproteobacteria Candidatus Pelagibacterales Pelagibacteraceae bacterium MBI29191.1 311 aa
Pseudomonadati Pseudomonadota Alphaproteobacteria Rickettsiales Rickettsiales bacterium MBS56745.1 311 aa
Pseudomonadati Pseudomonadota Alphaproteobacteria Candidatus Pelagibacterales Pelagibacteraceae bacterium MDC3177153.1 311 aa
Pseudomonadati Pseudomonadota Alphaproteobacteria unclassified Alphaproteobacteria Alphaproteobacteria bacterium MBL0318888.1 314 aa
Pseudomonadati Pseudomonadota Alphaproteobacteria unclassified Alphaproteobacteria Alphaproteobacteria bacterium GCA 002787615 310 aa
Pseudomonadati Pseudomonadota Alphaproteobacteria unclassified Alphaproteobacteria Alphaproteobacteria bacterium DQGZ01000032.1 313 aa
Pseudomonadati Pseudomonadota Alphaproteobacteria Rickettsiales Rickettsiales bacterium MDX1949605.1 315 aa
Pseudomonadati Pseudomonadota Alphaproteobacteria Rickettsiales Rickettsiales bacterium MCH2037311.1 310 aa
Pseudomonadati Pseudomonadota Alphaproteobacteria Rickettsiales Rickettsiales bacterium MCD6035659.1 309 aa
Pseudomonadati Pseudomonadota Alphaproteobacteria Rickettsiales Rickettsiaceae bacterium MCE3231750.1 316 aa
Pseudomonadati Pseudomonadota Alphaproteobacteria Rickettsiales Rickettsiales bacterium MET0154710.1 310 aa
Pseudomonadati Pseudomonadota Alphaproteobacteria Rickettsiales Rickettsiales bacterium MDB2414479.1 310 aa
Pseudomonadati Pseudomonadota Alphaproteobacteria Hyphomicrobiales Hyphomicrobiales bacterium MCP5362109.1 320 aa
Pseudomonadati Pseudomonadota Alphaproteobacteria Rickettsiales Rickettsiales bacterium MFO1242819.1 310 aa
Pseudomonadati Pseudomonadota Alphaproteobacteria Rickettsiales Rickettsiales bacterium AIL65790.1 310 aa
Pseudomonadati Pseudomonadota Alphaproteobacteria unclassified Alphaproteobacteria Alphaproteobacteria bacterium MCC7260424.1 312 aa
Pseudomonadati Pseudomonadota Alphaproteobacteria Rickettsiales Rickettsiales bacterium MDG1287970.1 310 aa
Pseudomonadati Pseudomonadota Alphaproteobacteria Rickettsiales Rickettsiales bacterium HEU5046211.1 310 aa
Pseudomonadati Pseudomonadota Alphaproteobacteria Rickettsiales Rickettsiales bacterium MCI5049493.1 311 aa
Pseudomonadati Pseudomonadota Alphaproteobacteria Rickettsiales Rickettsiales bacterium MBN67024.1 311 aa
Pseudomonadati Pseudomonadota Alphaproteobacteria unclassified Alphaproteobacteria Alphaproteobacteria bacterium MFO0110101.1 311 aa
Pseudomonadati Pseudomonadota Alphaproteobacteria unclassified Alphaproteobacteria Alphaproteobacteria bacterium MBN8544008.1 311 aa
Pseudomonadati Pseudomonadota Alphaproteobacteria Rickettsiales Rickettsiales bacterium MFZ4540847.1 311 aa
Pseudomonadati Pseudomonadota Alphaproteobacteria Rickettsiales Rickettsiales bacterium MFM9890471.1 311 aa
Pseudomonadati Pseudomonadota Alphaproteobacteria Rickettsiales Rickettsiales bacterium MAR55871.1 311 aa
Pseudomonadati Pseudomonadota Alphaproteobacteria unclassified Alphaproteobacteria Alphaproteobacteria bacterium MGE0753709.1 311 aa
Pseudomonadati Pseudomonadota Alphaproteobacteria unclassified Alphaproteobacteria Alphaproteobacteria bacterium MDX2113385.1 311 aa
Pseudomonadati Pseudomonadota Alphaproteobacteria Rickettsiales Rickettsiales bacterium MFW0777471.1 311 aa
Pseudomonadati Pseudomonadota Alphaproteobacteria Rickettsiales Rickettsiales bacterium MDX1974133.1 311 aa
Pseudomonadati Pseudomonadota Alphaproteobacteria unclassified Alphaproteobacteria Alphaproteobacteria bacterium MDX2074659.1 311 aa
Pseudomonadati Pseudomonadota Alphaproteobacteria Rickettsiales Rickettsiales bacterium MEZ5690616.1 311 aa
Pseudomonadati Pseudomonadota Alphaproteobacteria Rickettsiales Rickettsiales bacterium MEQ1705445.1 311 aa
Pseudomonadati Pseudomonadota Alphaproteobacteria Rhodospirillales Rhodospirillaceae bacterium GCA 002700865 313 aa
Pseudomonadati Pseudomonadota Alphaproteobacteria Holosporales Caedimonas varicaedens GAO98016.1 310 aa
Pseudomonadati Pseudomonadota Alphaproteobacteria Holosporales Caedimonas sp. MBL9029699.1 313 aa
Pseudomonadati Pseudomonadota Alphaproteobacteria Rhodospirillales Thalassospira sp. GCF 001983425 310 aa
Pseudomonadati Pseudomonadota Alphaproteobacteria unclassified Alphaproteobacteria Alphaproteobacteria bacterium MBP9693771.1 310 aa
Pseudomonadati Pseudomonadota Alphaproteobacteria Rhodospirillales Rhodospirillales bacterium MCY4430134.1 310 aa
Pseudomonadati Pseudomonadota Alphaproteobacteria Holosporales Holosporales bacterium MGL6187613.1 263 aa
Pseudomonadati Pseudomonadota Alphaproteobacteria Holosporales Holosporales bacterium MBY0264525.1 309 aa
Pseudomonadati Pseudomonadota Alphaproteobacteria Rhodobacterales Albidovulum sp bin36 GCA 002238565 334 aa
Pseudomonadati Pseudomonadota Alphaproteobacteria Holosporales Holosporales bacterium MFN7710079.1 310 aa
Pseudomonadati Pseudomonadota Alphaproteobacteria Holosporales Candidatus Nucleicultrix GCF 002117145 310 aa
Pseudomonadati Pseudomonadota Alphaproteobacteria Holosporales Caedimonadaceae bacterium KAB2835803.1 310 aa
Pseudomonadati Pseudomonadota Alphaproteobacteria Rhodospirillales Candidatus Hepatincolaceae MFL1780394.1 311 aa
Pseudomonadati Pseudomonadota Alphaproteobacteria Rickettsiales Rickettsiales bacterium MGV3278775.1 313 aa
Pseudomonadati Pseudomonadota Alphaproteobacteria Rickettsiales Rickettsiales bacterium MBK67165.1 315 aa
Pseudomonadati Pseudomonadota Alphaproteobacteria unclassified Alphaproteobacteria Alphaproteobacteria bacterium MDA7981889.1 325 aa
Pseudomonadati Pseudomonadota Alphaproteobacteria unclassified Alphaproteobacteria Alphaproteobacteria bacterium HYB57747.1 323 aa
Pseudomonadati Pseudomonadota Alphaproteobacteria unclassified Alphaproteobacteria Alphaproteobacteria bacterium HUC63209.1 323 aa
Pseudomonadati Pseudomonadota Alphaproteobacteria Parvibaculales Parvibaculales bacterium MGI9392179.1 310 aa
Pseudomonadati Pseudomonadota Alphaproteobacteria Sneathiellales Sneathiella litorea WP 161316019.1 309 aa
Pseudomonadati Pseudomonadota Alphaproteobacteria unclassified Alphaproteobacteria Alphaproteobacteria bacterium TNE27711.1 318 aa
Pseudomonadati Pseudomonadota Alphaproteobacteria unclassified Alphaproteobacteria Alphaproteobacteria bacterium MDD9900716.1 316 aa
Pseudomonadati Pseudomonadota Alphaproteobacteria unclassified Alphaproteobacteria Alphaproteobacteria bacterium MBI1214823.1 317 aa
Pseudomonadati Pseudomonadota Alphaproteobacteria unclassified Alphaproteobacteria Alphaproteobacteria bacterium MBU6475077.1 317 aa
Pseudomonadati Pseudomonadota Alphaproteobacteria unclassified Alphaproteobacteria Alphaproteobacteria bacterium MCK5518240.1 317 aa
Pseudomonadati Pseudomonadota Alphaproteobacteria unclassified Alphaproteobacteria Alphaproteobacteria bacterium MCE9507529.1 317 aa
Pseudomonadati Pseudomonadota Alphaproteobacteria unclassified Alphaproteobacteria Alphaproteobacteria bacterium MBY0427780.1 310 aa
Pseudomonadati Pseudomonadota Alphaproteobacteria unclassified Alphaproteobacteria Alphaproteobacteria bacterium MEJ0063045.1 315 aa
Pseudomonadati Pseudomonadota Alphaproteobacteria unclassified Alphaproteobacteria Alphaproteobacteria bacterium MBV8061880.1 315 aa
Pseudomonadati Pseudomonadota Alphaproteobacteria unclassified Alphaproteobacteria Alphaproteobacteria bacterium HUY69032.1 315 aa
Pseudomonadati Pseudomonadota Alphaproteobacteria Rhodospirillales Rhodospirillaceae bacterium HBM91343.1 315 aa
Pseudomonadati Pseudomonadota Alphaproteobacteria unclassified Alphaproteobacteria Alphaproteobacteria bacterium MDD2326035.1 315 aa
Pseudomonadati Pseudomonadota Alphaproteobacteria unclassified Alphaproteobacteria Alphaproteobacteria bacterium MCL2468982.1 319 aa
Pseudomonadati Pseudomonadota Alphaproteobacteria unclassified Alphaproteobacteria Alphaproteobacteria bacterium MDX9689833.1 315 aa
Pseudomonadati Pseudomonadota Alphaproteobacteria Rhodospirillales Rhodospirillaceae bacterium HAX91328.1 316 aa

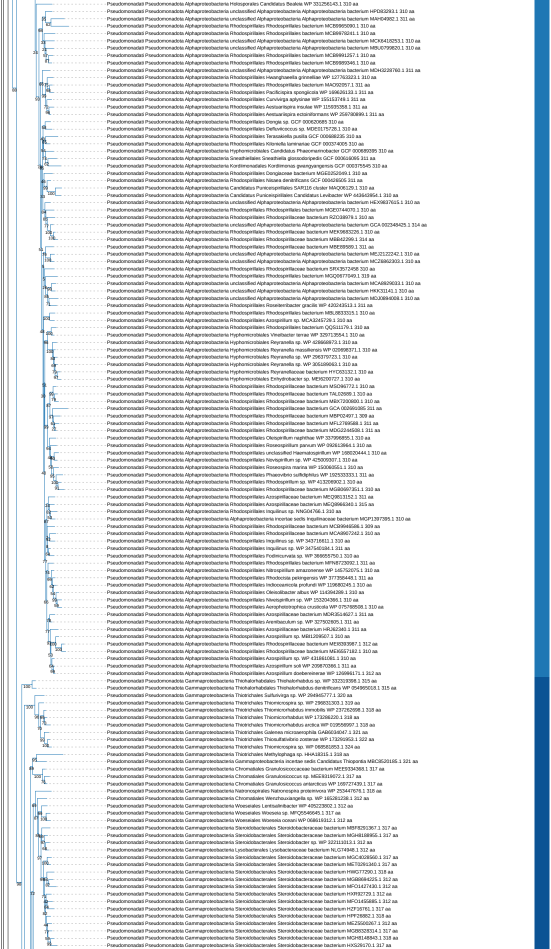
Pseudomonadati Pseudomonadota Alphaproteobacteria Holosporales Candidatus Bealeia WP 331256143.1 310 aa
Pseudomonadati Pseudomonadota Alphaproteobacteria unclassified Alphaproteobacteria Alphaproteobacteria bacterium HPD83293.1 310 aa
Pseudomonadati Pseudomonadota Alphaproteobacteria unclassified Alphaproteobacteria Alphaproteobacteria bacterium MAH04982.1 311 aa
Pseudomonadati Pseudomonadota Alphaproteobacteria Rhodospirillales Rhodospirillales bacterium MCB9965090.1 310 aa
Pseudomonadati Pseudomonadota Alphaproteobacteria Rhodospirillales Rhodospirillales bacterium MCB9978241.1 310 aa
Pseudomonadati Pseudomonadota Alphaproteobacteria unclassified Alphaproteobacteria Alphaproteobacteria bacterium MCK6418253.1 310 aa
Pseudomonadati Pseudomonadota Alphaproteobacteria unclassified Alphaproteobacteria Alphaproteobacteria bacterium MBU0799820.1 310 aa
Pseudomonadati Pseudomonadota Alphaproteobacteria Rhodospirillales Rhodospirillales bacterium MCB9991257.1 310 aa
Pseudomonadati Pseudomonadota Alphaproteobacteria Rhodospirillales Rhodospirillales bacterium MCB9989346.1 310 aa
Pseudomonadati Pseudomonadota Alphaproteobacteria unclassified Alphaproteobacteria Alphaproteobacteria bacterium MDH3228760.1 311 aa
Pseudomonadati Pseudomonadota Alphaproteobacteria Rhodospirillales Hwanghaeella grinnelliae WP 127763323.1 310 aa
Pseudomonadati Pseudomonadota Alphaproteobacteria Rhodospirillales Rhodospirillales bacterium MAO92057.1 311 aa
Pseudomonadati Pseudomonadota Alphaproteobacteria Rhodospirillales Pacificispira spongiicola WP 169626133.1 311 aa
Pseudomonadati Pseudomonadota Alphaproteobacteria Rhodospirillales Curvivirga aplysinae WP 155153749.1 311 aa
Pseudomonadati Pseudomonadota Alphaproteobacteria Rhodospirillales Aestuariispira insulae WP 115935358.1 311 aa
Pseudomonadati Pseudomonadota Alphaproteobacteria Rhodospirillales Aestuariispira ectoiniformans WP 259780899.1 311 aa
Pseudomonadati Pseudomonadota Alphaproteobacteria Rhodospirillales Dongia sp. GCF 000620685 310 aa
Pseudomonadati Pseudomonadota Alphaproteobacteria Rhodospirillales Defluviicoccus sp. MDE0175728.1 310 aa
Pseudomonadati Pseudomonadota Alphaproteobacteria Rhodospirillales Terasakiella pusilla GCF 000688235 310 aa
Pseudomonadati Pseudomonadota Alphaproteobacteria Rhodospirillales Kiloniella laminariae GCF 000374005 310 aa
Pseudomonadati Pseudomonadota Alphaproteobacteria Hyphomicrobiales Candidatus Phaeomarinobacter GCF 000689395 310 aa
Pseudomonadati Pseudomonadota Alphaproteobacteria Sneathiellales Sneathiella glossodoripedis GCF 000616095 311 aa
Pseudomonadati Pseudomonadota Alphaproteobacteria Kordiimonadales Kordiimonas gwangyangensis GCF 000375545 310 aa
Pseudomonadati Pseudomonadota Alphaproteobacteria Rhodospirillales Dongiaceae bacterium MGE0252049.1 310 aa
Pseudomonadati Pseudomonadota Alphaproteobacteria Rhodospirillales Nisaea denitrificans GCF 000426505 311 aa
Pseudomonadati Pseudomonadota Alphaproteobacteria Candidatus Puniceispirillales SAR116 cluster MAQ06129.1 310 aa
Pseudomonadati Pseudomonadota Alphaproteobacteria Candidatus Puniceispirillales Candidatus Levibacter WP 443643954.1 310 aa
Pseudomonadati Pseudomonadota Alphaproteobacteria unclassified Alphaproteobacteria Alphaproteobacteria bacterium HEX9837615.1 310 aa
Pseudomonadati Pseudomonadota Alphaproteobacteria Rhodospirillales Rhodospirillales bacterium MGE0744070.1 310 aa
Pseudomonadati Pseudomonadota Alphaproteobacteria Rhodospirillales Rhodospirillaceae bacterium RZO38979.1 310 aa
Pseudomonadati Pseudomonadota Alphaproteobacteria unclassified Alphaproteobacteria Alphaproteobacteria bacterium GCA 002348425.1 314 aa
Pseudomonadati Pseudomonadota Alphaproteobacteria Rhodospirillales Rhodospirillaceae bacterium MEK9683226.1 310 aa
Pseudomonadati Pseudomonadota Alphaproteobacteria Rhodospirillales Rhodospirillaceae bacterium MBB42299.1 314 aa
Pseudomonadati Pseudomonadota Alphaproteobacteria Rhodospirillales Rhodospirillaceae bacterium MBE89589.1 311 aa
Pseudomonadati Pseudomonadota Alphaproteobacteria unclassified Alphaproteobacteria Alphaproteobacteria bacterium MEJ2122242.1 310 aa
Pseudomonadati Pseudomonadota Alphaproteobacteria unclassified Alphaproteobacteria Alphaproteobacteria bacterium MCZ6862303.1 310 aa
Pseudomonadati Pseudomonadota Alphaproteobacteria Rhodospirillales Rhodospirillaceae bacterium SRX3572458 310 aa
Pseudomonadati Pseudomonadota Alphaproteobacteria Rhodospirillales Rhodospirillales bacterium MGQ0677049.1 319 aa
Pseudomonadati Pseudomonadota Alphaproteobacteria unclassified Alphaproteobacteria Alphaproteobacteria bacterium MCA8929033.1 310 aa
Pseudomonadati Pseudomonadota Alphaproteobacteria unclassified Alphaproteobacteria Alphaproteobacteria bacterium HKK31141.1 310 aa
Pseudomonadati Pseudomonadota Alphaproteobacteria unclassified Alphaproteobacteria Alphaproteobacteria bacterium MDJ0894008.1 310 aa
Pseudomonadati Pseudomonadota Alphaproteobacteria Rhodospirillales Roseiterribacter gracilis WP 420243513.1 311 aa
Pseudomonadati Pseudomonadota Alphaproteobacteria Rhodospirillales Rhodospirillales bacterium MBL8833315.1 310 aa
Pseudomonadati Pseudomonadota Alphaproteobacteria Rhodospirillales Azospirillum sp. MCA3245729.1 310 aa
Pseudomonadati Pseudomonadota Alphaproteobacteria Rhodospirillales Rhodospirillales bacterium QQS11179.1 310 aa
Pseudomonadati Pseudomonadota Alphaproteobacteria Hyphomicrobiales Vineibacter terrae WP 329713554.1 310 aa
Pseudomonadati Pseudomonadota Alphaproteobacteria Hyphomicrobiales Reyranella sp. WP 428668973.1 310 aa
Pseudomonadati Pseudomonadota Alphaproteobacteria Hyphomicrobiales Reyranella massiliensis WP 020698371.1 310 aa
Pseudomonadati Pseudomonadota Alphaproteobacteria Hyphomicrobiales Reyranella sp. WP 296379723.1 310 aa
Pseudomonadati Pseudomonadota Alphaproteobacteria Hyphomicrobiales Reyranella sp. WP 305189063.1 310 aa
Pseudomonadati Pseudomonadota Alphaproteobacteria Hyphomicrobiales Reyranellaceae bacterium HYC63132.1 310 aa
Pseudomonadati Pseudomonadota Alphaproteobacteria Hyphomicrobiales Enhydrobacter sp. MEI6200727.1 310 aa
Pseudomonadati Pseudomonadota Alphaproteobacteria Rhodospirillales Rhodospirillaceae bacterium MSO96772.1 310 aa
Pseudomonadati Pseudomonadota Alphaproteobacteria Rhodospirillales Rhodospirillaceae bacterium TAL02689.1 310 aa
Pseudomonadati Pseudomonadota Alphaproteobacteria Rhodospirillales Rhodospirillaceae bacterium MBX7200800.1 310 aa
Pseudomonadati Pseudomonadota Alphaproteobacteria Rhodospirillales Rhodospirillaceae bacterium GCA 002691085 311 aa
Pseudomonadati Pseudomonadota Alphaproteobacteria Rhodospirillales Rhodospirillaceae bacterium MBP02497.1 309 aa
Pseudomonadati Pseudomonadota Alphaproteobacteria Rhodospirillales Rhodospirillaceae bacterium MFL2769588.1 311 aa
Pseudomonadati Pseudomonadota Alphaproteobacteria Rhodospirillales Rhodospirillaceae bacterium MDG2244508.1 311 aa
Pseudomonadati Pseudomonadota Alphaproteobacteria Rhodospirillales Oleispirillum naphthae WP 337996855.1 310 aa
Pseudomonadati Pseudomonadota Alphaproteobacteria Rhodospirillales Roseospirillum parvum WP 092613964.1 310 aa
Pseudomonadati Pseudomonadota Alphaproteobacteria Rhodospirillales unclassified Haematospirillum WP 168020444.1 310 aa
Pseudomonadati Pseudomonadota Alphaproteobacteria Rhodospirillales Novispirillum sp. WP 425009307.1 310 aa
Pseudomonadati Pseudomonadota Alphaproteobacteria Rhodospirillales Roseospira marina WP 150060551.1 310 aa
Pseudomonadati Pseudomonadota Alphaproteobacteria Rhodospirillales Phaeovibrio sulfidiphilus WP 192533333.1 311 aa
Pseudomonadati Pseudomonadota Alphaproteobacteria Rhodospirillales Rhodospirillum sp. WP 413206902.1 310 aa
Pseudomonadati Pseudomonadota Alphaproteobacteria Rhodospirillales Rhodospirillaceae bacterium MGB0697351.1 310 aa
Pseudomonadati Pseudomonadota Alphaproteobacteria Rhodospirillales Azospirillaceae bacterium MEQ9813152.1 311 aa
Pseudomonadati Pseudomonadota Alphaproteobacteria Rhodospirillales Azospirillaceae bacterium MEQ8966340.1 315 aa
Pseudomonadati Pseudomonadota Alphaproteobacteria Rhodospirillales Inquilinus sp. NNG04766.1 310 aa
Pseudomonadati Pseudomonadota Alphaproteobacteria Alphaproteobacteria incertae sedis Inquilinaceae bacterium MGP1397395.1 310 aa
Pseudomonadati Pseudomonadota Alphaproteobacteria Rhodospirillales Rhodospirillaceae bacterium MCB9946586.1 309 aa
Pseudomonadati Pseudomonadota Alphaproteobacteria Rhodospirillales Rhodospirillaceae bacterium MCA8907242.1 310 aa
Pseudomonadati Pseudomonadota Alphaproteobacteria Rhodospirillales Inquilinus sp. WP 343716611.1 310 aa
Pseudomonadati Pseudomonadota Alphaproteobacteria Rhodospirillales Inquilinus sp. WP 347540184.1 311 aa
Pseudomonadati Pseudomonadota Alphaproteobacteria Rhodospirillales Fodinicurvata sp. WP 366655750.1 310 aa
Pseudomonadati Pseudomonadota Alphaproteobacteria Rhodospirillales Rhodospirillales bacterium MFN8723092.1 311 aa
Pseudomonadati Pseudomonadota Alphaproteobacteria Rhodospirillales Nitrospirillum amazonense WP 145752075.1 310 aa
Pseudomonadati Pseudomonadota Alphaproteobacteria Rhodospirillales Rhodocista pekingensis WP 377358448.1 311 aa
Pseudomonadati Pseudomonadota Alphaproteobacteria Rhodospirillales Indioceanicola profundi WP 119680245.1 310 aa
Pseudomonadati Pseudomonadota Alphaproteobacteria Rhodospirillales Oleisolibacter albus WP 114394289.1 310 aa
Pseudomonadati Pseudomonadota Alphaproteobacteria Rhodospirillales Niveispirillum sp. WP 153204366.1 310 aa
Pseudomonadati Pseudomonadota Alphaproteobacteria Rhodospirillales Aerophototrophica crusticola WP 075768508.1 310 aa
Pseudomonadati Pseudomonadota Alphaproteobacteria Rhodospirillales Azospirillaceae bacterium MDR3514627.1 311 aa
Pseudomonadati Pseudomonadota Alphaproteobacteria Rhodospirillales Arenibaculum sp. WP 327502605.1 311 aa
Pseudomonadati Pseudomonadota Alphaproteobacteria Rhodospirillales Azospirillaceae bacterium HRJ62340.1 311 aa
Pseudomonadati Pseudomonadota Alphaproteobacteria Rhodospirillales Azospirillum sp. MBI1209507.1 310 aa
Pseudomonadati Pseudomonadota Alphaproteobacteria Rhodospirillales Rhodospirillaceae bacterium MEI8393987.1 312 aa
Pseudomonadati Pseudomonadota Alphaproteobacteria Rhodospirillales Rhodospirillaceae bacterium MEI6557182.1 310 aa
Pseudomonadati Pseudomonadota Alphaproteobacteria Rhodospirillales Azospirillum sp. WP 431861081.1 310 aa
Pseudomonadati Pseudomonadota Alphaproteobacteria Rhodospirillales Azospirillum soli WP 209870366.1 311 aa
Pseudomonadati Pseudomonadota Alphaproteobacteria Rhodospirillales Azospirillum doebereinerae WP 126996171.1 312 aa
Pseudomonadati Pseudomonadota Gammaproteobacteria Thiohalorhabdales Thiohalorhabdus sp. WP 332319398.1 315 aa
Pseudomonadati Pseudomonadota Gammaproteobacteria Thiohalorhabdales Thiohalorhabdus denitrificans WP 054965018.1 315 aa
Pseudomonadati Pseudomonadota Gammaproteobacteria Thiotrichales Sulfurivirga sp. WP 294945777.1 320 aa
Pseudomonadati Pseudomonadota Gammaproteobacteria Thiotrichales Thiomicrospira sp. WP 296831303.1 319 aa
Pseudomonadati Pseudomonadota Gammaproteobacteria Thiotrichales Thiomicrorhabdus immobilis WP 237262698.1 318 aa
Pseudomonadati Pseudomonadota Gammaproteobacteria Thiotrichales Thiomicrorhabdus WP 173286220.1 318 aa
Pseudomonadati Pseudomonadota Gammaproteobacteria Thiotrichales Thiomicrorhabdus arctica WP 019556997.1 318 aa
Pseudomonadati Pseudomonadota Gammaproteobacteria Thiotrichales Galenea microaerophila GAB6034047.1 321 aa
Pseudomonadati Pseudomonadota Gammaproteobacteria Thiotrichales Thiosulfativibrio zosterae WP 173291953.1 322 aa
Pseudomonadati Pseudomonadota Gammaproteobacteria Thiotrichales Thiomicrospira sp. WP 068581853.1 324 aa
Pseudomonadati Pseudomonadota Gammaproteobacteria Thiotrichales Methylophaga sp. HHA18315.1 318 aa
Pseudomonadati Pseudomonadota Gammaproteobacteria Gammaproteobacteria incertae sedis Candidatus Thiopontia MBC8520185.1 321 aa
Pseudomonadati Pseudomonadota Gammaproteobacteria Chromatiales Granulosicoccaceae bacterium MEE9334368.1 317 aa
Pseudomonadati Pseudomonadota Gammaproteobacteria Chromatiales Granulosicoccus sp. MEE9319072.1 317 aa
Pseudomonadati Pseudomonadota Gammaproteobacteria Chromatiales Granulosicoccus antarcticus WP 169727439.1 317 aa
Pseudomonadati Pseudomonadota Gammaproteobacteria Natronospirales Natronospira proteinivora WP 253447676.1 318 aa
Pseudomonadati Pseudomonadota Gammaproteobacteria Chromatiales Wenzhouxiangella sp. WP 165281238.1 312 aa
Pseudomonadati Pseudomonadota Gammaproteobacteria Woeseiales Lentisalinibacter WP 405223802.1 312 aa
Pseudomonadati Pseudomonadota Gammaproteobacteria Woeseiales Woeseia sp. MFQ5546645.1 317 aa
Pseudomonadati Pseudomonadota Gammaproteobacteria Woeseiales Woeseia oceani WP 068619312.1 312 aa
Pseudomonadati Pseudomonadota Gammaproteobacteria Steroidobacterales Steroidobacteraceae bacterium MBF8291367.1 317 aa
Pseudomonadati Pseudomonadota Gammaproteobacteria Steroidobacterales Steroidobacteraceae bacterium MGH8188955.1 317 aa
Pseudomonadati Pseudomonadota Gammaproteobacteria Steroidobacterales Steroidobacter sp. WP 322111013.1 312 aa
Pseudomonadati Pseudomonadota Gammaproteobacteria Lysobacterales Lysobacteraceae bacterium NLG74948.1 312 aa
Pseudomonadati Pseudomonadota Gammaproteobacteria Steroidobacterales Steroidobacteraceae bacterium MGC4028560.1 317 aa
Pseudomonadati Pseudomonadota Gammaproteobacteria Steroidobacterales Steroidobacteraceae bacterium MET0291340.1 317 aa
Pseudomonadati Pseudomonadota Gammaproteobacteria Steroidobacterales Steroidobacteraceae bacterium HWG77290.1 318 aa
Pseudomonadati Pseudomonadota Gammaproteobacteria Steroidobacterales Steroidobacteraceae bacterium MGB8694225.1 312 aa
Pseudomonadati Pseudomonadota Gammaproteobacteria Steroidobacterales Steroidobacteraceae bacterium MFO1427430.1 312 aa
Pseudomonadati Pseudomonadota Gammaproteobacteria Steroidobacterales Steroidobacteraceae bacterium HXR92729.1 312 aa
Pseudomonadati Pseudomonadota Gammaproteobacteria Steroidobacterales Steroidobacteraceae bacterium MFO1455885.1 312 aa
Pseudomonadati Pseudomonadota Gammaproteobacteria Steroidobacterales Steroidobacteraceae bacterium HZF16761.1 317 aa
Pseudomonadati Pseudomonadota Gammaproteobacteria Steroidobacterales Steroidobacteraceae bacterium HPF26882.1 318 aa
Pseudomonadati Pseudomonadota Gammaproteobacteria Steroidobacterales Steroidobacteraceae bacterium MEZ5500267.1 312 aa
Pseudomonadati Pseudomonadota Gammaproteobacteria Steroidobacterales Steroidobacteraceae bacterium MGB8328314.1 317 aa
Pseudomonadati Pseudomonadota Gammaproteobacteria Steroidobacterales Steroidobacteraceae bacterium MGH8148843.1 318 aa
Pseudomonadati Pseudomonadota Gammaproteobacteria Steroidobacterales Steroidobacteraceae bacterium HXS29170.1 317 aa

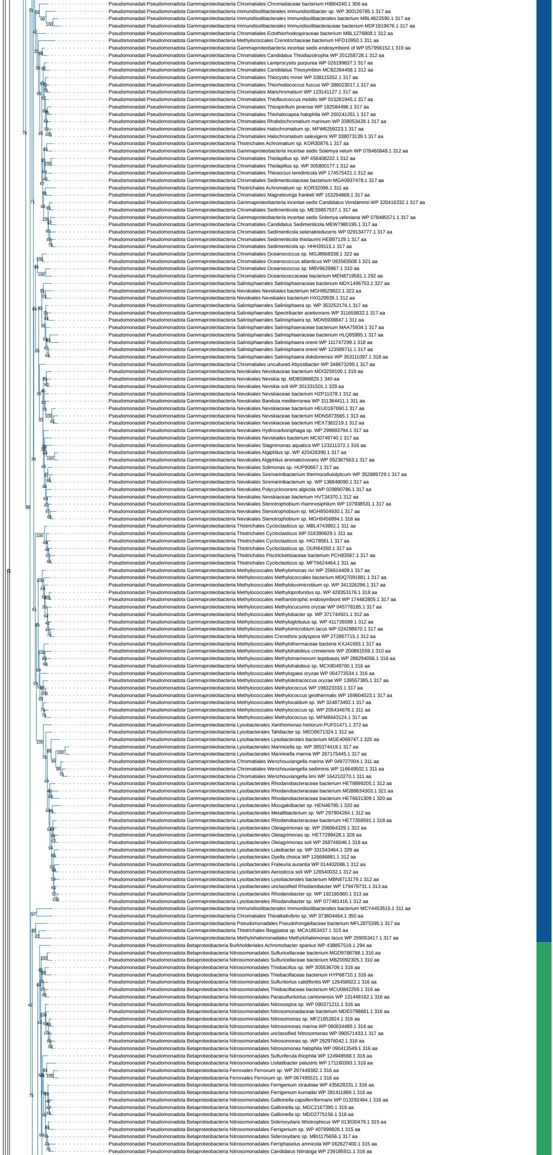

Pseudomonadati Pseudomonadota Gammaproteobacteria Chromatiales Chromatiaceae bacterium HIB84240.1 306 aa
Pseudomonadati Pseudomonadota Gammaproteobacteria Immundisolibacterales Immundisolibacter sp. WP 300126785.1 317 aa
Pseudomonadati Pseudomonadota Gammaproteobacteria Immundisolibacterales Immundisolibacterales bacterium MBL4622590.1 317 aa
Pseudomonadati Pseudomonadota Gammaproteobacteria Immundisolibacterales Immundisolibacteraceae bacterium MDF1819676.1 317 aa
Pseudomonadati Pseudomonadota Gammaproteobacteria Chromatiales Ectothiorhodospiraceae bacterium MBL1276808.1 312 aa
Pseudomonadati Pseudomonadota Gammaproteobacteria Methylococcales Crenotrichaceae bacterium HFD10950.1 311 aa
Pseudomonadati Pseudomonadota Gammaproteobacteria Gammaproteobacteria incertae sedis endosymbiont of WP 057956152.1 319 aa
Pseudomonadati Pseudomonadota Gammaproteobacteria Chromatiales Candidatus Thiodiazotropha WP 201258728.1 312 aa
Pseudomonadati Pseudomonadota Gammaproteobacteria Chromatiales Lamprocystis purpurea WP 026199837.1 317 aa
Pseudomonadati Pseudomonadota Gammaproteobacteria Chromatiales Candidatus Thiosymbion MCB2264458.1 312 aa
Pseudomonadati Pseudomonadota Gammaproteobacteria Chromatiales Thiocystis minor WP 338115352.1 317 aa
Pseudomonadati Pseudomonadota Gammaproteobacteria Chromatiales Thiorhodococcus fuscus WP 386023017.1 317 aa
Pseudomonadati Pseudomonadota Gammaproteobacteria Chromatiales Marichromatium WP 123141127.1 317 aa
Pseudomonadati Pseudomonadota Gammaproteobacteria Chromatiales Thioflavicoccus mobilis WP 015281945.1 317 aa
Pseudomonadati Pseudomonadota Gammaproteobacteria Chromatiales Thiospirillum jenense WP 182584496.1 317 aa
Pseudomonadati Pseudomonadota Gammaproteobacteria Chromatiales Thiohalocapsa halophila WP 200241251.1 317 aa
Pseudomonadati Pseudomonadota Gammaproteobacteria Chromatiales Rhabdochromatium marinum WP 338053428.1 317 aa
Pseudomonadati Pseudomonadota Gammaproteobacteria Chromatiales Halochromatium sp. MFW6259223.1 317 aa
Pseudomonadati Pseudomonadota Gammaproteobacteria Chromatiales Halochromatium salexigens WP 338073139.1 317 aa
Pseudomonadati Pseudomonadota Gammaproteobacteria Thiotrichales Achromatium sp. KOR30876.1 317 aa
Pseudomonadati Pseudomonadota Gammaproteobacteria Gammaproteobacteria incertae sedis Solemya velum WP 078460848.1 312 aa
Pseudomonadati Pseudomonadota Gammaproteobacteria Chromatiales Thiolapillus sp. WP 456408222.1 312 aa
Pseudomonadati Pseudomonadota Gammaproteobacteria Chromatiales Thiolapillus sp. WP 305800177.1 312 aa
Pseudomonadati Pseudomonadota Gammaproteobacteria Chromatiales Thiosocius teredinicola WP 174575421.1 312 aa
Pseudomonadati Pseudomonadota Gammaproteobacteria Chromatiales Sedimenticolaceae bacterium MGA0937478.1 317 aa
Pseudomonadati Pseudomonadota Gammaproteobacteria Thiotrichales Achromatium sp. KOR32096.1 311 aa
Pseudomonadati Pseudomonadota Gammaproteobacteria Chromatiales Magnetovirga frankeli WP 153294868.1 317 aa
Pseudomonadati Pseudomonadota Gammaproteobacteria Gammaproteobacteria incertae sedis Candidatus Vondammii WP 320416332.1 317 aa
Pseudomonadati Pseudomonadota Gammaproteobacteria Chromatiales Sedimenticola sp. MES9857537.1 317 aa
Pseudomonadati Pseudomonadota Gammaproteobacteria Gammaproteobacteria incertae sedis Solemya velesiana WP 078485571.1 317 aa
Pseudomonadati Pseudomonadota Gammaproteobacteria Chromatiales Candidatus Sedimenticola MEW7980195.1 317 aa
Pseudomonadati Pseudomonadota Gammaproteobacteria Chromatiales Sedimenticola selenatireducens WP 029134777.1 317 aa
Pseudomonadati Pseudomonadota Gammaproteobacteria Chromatiales Sedimenticola thiotaurini HEB97129.1 317 aa
Pseudomonadati Pseudomonadota Gammaproteobacteria Chromatiales Sedimenticola sp. HHH39115.1 317 aa
Pseudomonadati Pseudomonadota Gammaproteobacteria Chromatiales Oceanococcus sp. MGJ8668338.1 322 aa
Pseudomonadati Pseudomonadota Gammaproteobacteria Chromatiales Oceanococcus atlanticus WP 083563508.1 321 aa
Pseudomonadati Pseudomonadota Gammaproteobacteria Chromatiales Oceanococcus sp. MBV6629867.1 310 aa
Pseudomonadati Pseudomonadota Gammaproteobacteria Chromatiales Oceanococcaceae bacterium MEN8719581.1 292 aa
Pseudomonadati Pseudomonadota Gammaproteobacteria Salinisphaerales Salinisphaeraceae bacterium MDX1496753.1 327 aa
Pseudomonadati Pseudomonadota Gammaproteobacteria Nevskiales Nevskiales bacterium MGH8529822.1 322 aa
Pseudomonadati Pseudomonadota Gammaproteobacteria Nevskiales Nevskiales bacterium HXG29939.1 312 aa
Pseudomonadati Pseudomonadota Gammaproteobacteria Salinisphaerales Salinisphaera sp. WP 353252176.1 317 aa
Pseudomonadati Pseudomonadota Gammaproteobacteria Salinisphaerales Spectribacter acetivorans WP 311659832.1 317 aa
Pseudomonadati Pseudomonadota Gammaproteobacteria Salinisphaerales Salinisphaera sp. MDN5938847.1 311 aa
Pseudomonadati Pseudomonadota Gammaproteobacteria Salinisphaerales Salinisphaeraceae bacterium MAA75934.1 317 aa
Pseudomonadati Pseudomonadota Gammaproteobacteria Salinisphaerales Salinisphaeraceae bacterium HLQ85995.1 317 aa
Pseudomonadati Pseudomonadota Gammaproteobacteria Salinisphaerales Salinisphaera orenii WP 111747299.1 318 aa
Pseudomonadati Pseudomonadota Gammaproteobacteria Salinisphaerales Salinisphaera orenii WP 123589711.1 317 aa
Pseudomonadati Pseudomonadota Gammaproteobacteria Salinisphaerales Salinisphaera dokdonensis WP 353111097.1 318 aa
Pseudomonadati Pseudomonadota Gammaproteobacteria Chromatiales uncultured Abyssibacter WP 348673299.1 317 aa
Pseudomonadati Pseudomonadota Gammaproteobacteria Nevskiales Nevskiaceae bacterium MDI3259100.1 319 aa
Pseudomonadati Pseudomonadota Gammaproteobacteria Nevskiales Nevskia sp. MDB5986829.1 340 aa
Pseudomonadati Pseudomonadota Gammaproteobacteria Nevskiales Nevskia soli WP 301331501.1 329 aa
Pseudomonadati Pseudomonadota Gammaproteobacteria Nevskiales Nevskiaceae bacterium HZP11078.1 312 aa
Pseudomonadati Pseudomonadota Gammaproteobacteria Nevskiales Banduia mediterranea WP 311364411.1 311 aa
Pseudomonadati Pseudomonadota Gammaproteobacteria Nevskiales Nevskiaceae bacterium HEU0197690.1 317 aa
Pseudomonadati Pseudomonadota Gammaproteobacteria Nevskiales Nevskiaceae bacterium MDN5873565.1 313 aa
Pseudomonadati Pseudomonadota Gammaproteobacteria Nevskiales Nevskiaceae bacterium HEX7382219.1 312 aa
Pseudomonadati Pseudomonadota Gammaproteobacteria Nevskiales Hydrocarboniphaga sp. WP 299693794.1 317 aa
Pseudomonadati Pseudomonadota Gammaproteobacteria Nevskiales Nevskiales bacterium MCI0749740.1 317 aa
Pseudomonadati Pseudomonadota Gammaproteobacteria Nevskiales Stagnimonas aquatica WP 123211372.1 316 aa
Pseudomonadati Pseudomonadota Gammaproteobacteria Nevskiales Algiphilus sp. WP 420426390.1 317 aa
Pseudomonadati Pseudomonadota Gammaproteobacteria Nevskiales Algiphilus aromaticivorans WP 052367563.1 317 aa
Pseudomonadati Pseudomonadota Gammaproteobacteria Nevskiales Solimonas sp. HUP90667.1 317 aa
Pseudomonadati Pseudomonadota Gammaproteobacteria Nevskiales Sinimarinibacterium thermocellulolyticum WP 352889729.1 317 aa
Pseudomonadati Pseudomonadota Gammaproteobacteria Nevskiales Sinimarinibacterium sp. WP 136848090.1 317 aa
Pseudomonadati Pseudomonadota Gammaproteobacteria Nevskiales Polycyclovorans algicola WP 029890786.1 317 aa
Pseudomonadati Pseudomonadota Gammaproteobacteria Nevskiales Nevskiaceae bacterium HVT34370.1 312 aa
Pseudomonadati Pseudomonadota Gammaproteobacteria Nevskiales Stenotrophobium rhamnosiphilum WP 107938531.1 317 aa
Pseudomonadati Pseudomonadota Gammaproteobacteria Nevskiales Stenotrophobium sp. MGH8504930.1 317 aa
Pseudomonadati Pseudomonadota Gammaproteobacteria Nevskiales Stenotrophobium sp. MGH8456894.1 318 aa
Pseudomonadati Pseudomonadota Gammaproteobacteria Thiotrichales Cycloclasticus sp. MBL4743992.1 311 aa
Pseudomonadati Pseudomonadota Gammaproteobacteria Thiotrichales Cycloclasticus WP 016390629.1 311 aa
Pseudomonadati Pseudomonadota Gammaproteobacteria Thiotrichales Cycloclasticus sp. HIG78581.1 317 aa
Pseudomonadati Pseudomonadota Gammaproteobacteria Thiotrichales Cycloclasticus sp. OUR84350.1 317 aa
Pseudomonadati Pseudomonadota Gammaproteobacteria Thiotrichales Piscirickettsiaceae bacterium PCH83567.1 317 aa
Pseudomonadati Pseudomonadota Gammaproteobacteria Thiotrichales Cycloclasticus sp. MFT6624464.1 311 aa
Pseudomonadati Pseudomonadota Gammaproteobacteria Methylococcales Methylomonas rivi WP 256614409.1 317 aa
Pseudomonadati Pseudomonadota Gammaproteobacteria Methylococcales Methylococcales bacterium MDQ7091881.1 317 aa
Pseudomonadati Pseudomonadota Gammaproteobacteria Methylococcales Methylotuvimicrobium sp. WP 341326296.1 317 aa
Pseudomonadati Pseudomonadota Gammaproteobacteria Methylococcales Methyloprofundus sp. WP 428353176.1 318 aa
Pseudomonadati Pseudomonadota Gammaproteobacteria Methylococcales methanotrophic endosymbiont WP 174482805.1 317 aa
Pseudomonadati Pseudomonadota Gammaproteobacteria Methylococcales Methylocucumis oryzae WP 045778185.1 317 aa
Pseudomonadati Pseudomonadota Gammaproteobacteria Methylococcales Methylobacter sp. WP 371744921.1 312 aa
Pseudomonadati Pseudomonadota Gammaproteobacteria Methylococcales Methyloglobulus sp. WP 411726599.1 312 aa
Pseudomonadati Pseudomonadota Gammaproteobacteria Methylococcales Methylomicrobium lacus WP 024298670.1 317 aa
Pseudomonadati Pseudomonadota Gammaproteobacteria Methylococcales Crenothrix polyspora WP 272867715.1 312 aa
Pseudomonadati Pseudomonadota Gammaproteobacteria Methylococcales Methylothermaceae bacteria KXJ41693.1 317 aa
Pseudomonadati Pseudomonadota Gammaproteobacteria Methylococcales Methylohalobius crimeensis WP 200861559.1 310 aa
Pseudomonadati Pseudomonadota Gammaproteobacteria Methylococcales Methylomarinovum tepidoasis WP 286294056.1 316 aa
Pseudomonadati Pseudomonadota Gammaproteobacteria Methylococcales Methylohalobius sp. MCX8049700.1 316 aa
Pseudomonadati Pseudomonadota Gammaproteobacteria Methylococcales Methylogaea oryzae WP 054773534.1 316 aa
Pseudomonadati Pseudomonadota Gammaproteobacteria Methylococcales Methylotetracoccus oryzae WP 139557385.1 317 aa
Pseudomonadati Pseudomonadota Gammaproteobacteria Methylococcales Methylococcus WP 198323333.1 317 aa
Pseudomonadati Pseudomonadota Gammaproteobacteria Methylococcales Methylococcus geothermalis WP 169604023.1 317 aa
Pseudomonadati Pseudomonadota Gammaproteobacteria Methylococcales Methylocaldum sp. WP 324873492.1 317 aa
Pseudomonadati Pseudomonadota Gammaproteobacteria Methylococcales Methylococcus sp. WP 205434676.1 311 aa
Pseudomonadati Pseudomonadota Gammaproteobacteria Methylococcales Methylococcus sp. MFM8443124.1 317 aa
Pseudomonadati Pseudomonadota Gammaproteobacteria Lysobacterales Xanthomonas hortorum PUF01471.1 372 aa
Pseudomonadati Pseudomonadota Gammaproteobacteria Lysobacterales Tahibacter sp. MEO8671324.1 312 aa
Pseudomonadati Pseudomonadota Gammaproteobacteria Lysobacterales Lysobacterales bacterium MGE4069747.1 325 aa
Pseudomonadati Pseudomonadota Gammaproteobacteria Lysobacterales Marinicella sp. WP 395374419.1 317 aa
Pseudomonadati Pseudomonadota Gammaproteobacteria Lysobacterales Marinicella marina WP 267175445.1 317 aa
Pseudomonadati Pseudomonadota Gammaproteobacteria Chromatiales Wenzhouxiangella marina WP 049727004.1 311 aa
Pseudomonadati Pseudomonadota Gammaproteobacteria Chromatiales Wenzhouxiangella sediminis WP 116649502.1 311 aa
Pseudomonadati Pseudomonadota Gammaproteobacteria Chromatiales Wenzhouxiangella limi WP 164210270.1 311 aa
Pseudomonadati Pseudomonadota Gammaproteobacteria Lysobacterales Rhodanobacteraceae bacterium HET8899205.1 312 aa
Pseudomonadati Pseudomonadota Gammaproteobacteria Lysobacterales Rhodanobacteraceae bacterium MGB8634303.1 321 aa
Pseudomonadati Pseudomonadota Gammaproteobacteria Lysobacterales Rhodanobacteraceae bacterium HET6631309.1 320 aa
Pseudomonadati Pseudomonadota Gammaproteobacteria Lysobacterales Mizugakiibacter sp. HEN46795.1 320 aa
Pseudomonadati Pseudomonadota Gammaproteobacteria Lysobacterales Metallibacterium sp. WP 297904264.1 312 aa
Pseudomonadati Pseudomonadota Gammaproteobacteria Lysobacterales Rhodanobacteraceae bacterium HET7358591.1 318 aa
Pseudomonadati Pseudomonadota Gammaproteobacteria Lysobacterales Oleiagrimonas sp. WP 206064329.1 322 aa
Pseudomonadati Pseudomonadota Gammaproteobacteria Lysobacterales Oleiagrimonas sp. HET7299428.1 328 aa
Pseudomonadati Pseudomonadota Gammaproteobacteria Lysobacterales Oleiagrimonas soli WP 268746046.1 318 aa
Pseudomonadati Pseudomonadota Gammaproteobacteria Lysobacterales Luteibacter sp. WP 331543464.1 329 aa
Pseudomonadati Pseudomonadota Gammaproteobacteria Lysobacterales Dyella choica WP 126686881.1 312 aa
Pseudomonadati Pseudomonadota Gammaproteobacteria Lysobacterales Frateuria aurantia WP 014402086.1 312 aa
Pseudomonadati Pseudomonadota Gammaproteobacteria Lysobacterales Aerosticca soli WP 126540032.1 312 aa
Pseudomonadati Pseudomonadota Gammaproteobacteria Lysobacterales Lysobacterales bacterium MBN8713179.1 312 aa
Pseudomonadati Pseudomonadota Gammaproteobacteria Lysobacterales unclassified Rhodanobacter WP 179479731.1 313 aa
Pseudomonadati Pseudomonadota Gammaproteobacteria Lysobacterales Rhodanobacter sp. WP 192165960.1 313 aa
Pseudomonadati Pseudomonadota Gammaproteobacteria Lysobacterales Rhodanobacter sp. WP 077481416.1 312 aa
Pseudomonadati Pseudomonadota Gammaproteobacteria Immundisolibacterales Immundisolibacterales bacterium MCY4453515.1 311 aa
Pseudomonadati Pseudomonadota Gammaproteobacteria Chromatiales Thioalkalivibrio sp. WP 373604464.1 350 aa
Pseudomonadati Pseudomonadota Gammaproteobacteria Pseudomonadales Pseudohongiellaceae bacterium MFL2875395.1 317 aa
Pseudomonadati Pseudomonadota Gammaproteobacteria Thiotrichales Beggiatoa sp. MCA1853437.1 315 aa
Pseudomonadati Pseudomonadota Gammaproteobacteria Methylohalomonadales Methylohalomonas lacus WP 259053417.1 317 aa
Pseudomonadati Pseudomonadota Betaproteobacteria Burkholderiales Achromobacter spanius WP 438857519.1 294 aa
Pseudomonadati Pseudomonadota Betaproteobacteria Nitrosomonadales Sulfuricellaceae bacterium MGD9788788.1 316 aa
Pseudomonadati Pseudomonadota Betaproteobacteria Nitrosomonadales Sulfuricellaceae bacterium MBZ0092305.1 310 aa
Pseudomonadati Pseudomonadota Betaproteobacteria Nitrosomonadales Thiobacillus sp. WP 305536709.1 316 aa
Pseudomonadati Pseudomonadota Betaproteobacteria Nitrosomonadales Thiobacillaceae bacterium HYP68710.1 316 aa
Pseudomonadati Pseudomonadota Betaproteobacteria Nitrosomonadales Sulfuritortus calidifontis WP 126458922.1 316 aa
Pseudomonadati Pseudomonadota Betaproteobacteria Nitrosomonadales Thiobacillaceae bacterium MCU0842259.1 316 aa
Pseudomonadati Pseudomonadota Betaproteobacteria Nitrosomonadales Parasulfuritortus cantonensis WP 131448162.1 316 aa
Pseudomonadati Pseudomonadota Betaproteobacteria Nitrosomonadales Nitrosospira sp. WP 090371211.1 316 aa
Pseudomonadati Pseudomonadota Betaproteobacteria Nitrosomonadales Nitrosomonadaceae bacterium MDE0798681.1 316 aa
Pseudomonadati Pseudomonadota Betaproteobacteria Nitrosomonadales Nitrosomonas sp. MFZ1852824.1 316 aa
Pseudomonadati Pseudomonadota Betaproteobacteria Nitrosomonadales Nitrosomonas marina WP 090634489.1 316 aa
Pseudomonadati Pseudomonadota Betaproteobacteria Nitrosomonadales unclassified Nitrosomonas WP 090571433.1 317 aa
Pseudomonadati Pseudomonadota Betaproteobacteria Nitrosomonadales Nitrosomonas sp. WP 292976042.1 316 aa
Pseudomonadati Pseudomonadota Betaproteobacteria Nitrosomonadales Nitrosomonas halophila WP 090413549.1 316 aa
Pseudomonadati Pseudomonadota Betaproteobacteria Nitrosomonadales Sulfuriferula thiophila WP 124948568.1 316 aa
Pseudomonadati Pseudomonadota Betaproteobacteria Nitrosomonadales Usitatibacter palustris WP 171160393.1 316 aa
Pseudomonadati Pseudomonadota Betaproteobacteria Ferrovales Ferrovum sp. WP 297449382.1 316 aa
Pseudomonadati Pseudomonadota Betaproteobacteria Ferrovales Ferrovum sp. WP 067495521.1 316 aa
Pseudomonadati Pseudomonadota Betaproteobacteria Nitrosomonadales Ferrigenium straubiae WP 435628231.1 316 aa
Pseudomonadati Pseudomonadota Betaproteobacteria Nitrosomonadales Ferrigenium kumadai WP 281411869.1 316 aa
Pseudomonadati Pseudomonadota Betaproteobacteria Nitrosomonadales Gallionella capsiferriformans WP 013292464.1 316 aa
Pseudomonadati Pseudomonadota Betaproteobacteria Nitrosomonadales Gallionella sp. MGC2167390.1 316 aa
Pseudomonadati Pseudomonadota Betaproteobacteria Nitrosomonadales Gallionella sp. MDD2775156.1 316 aa
Pseudomonadati Pseudomonadota Betaproteobacteria Nitrosomonadales Sideroxydans lithotrophicus WP 013030479.1 315 aa
Pseudomonadati Pseudomonadota Betaproteobacteria Nitrosomonadales Ferrigenium sp. WP 407899826.1 315 aa
Pseudomonadati Pseudomonadota Betaproteobacteria Nitrosomonadales Sideroxydans sp. MBI1175656.1 317 aa
Pseudomonadati Pseudomonadota Betaproteobacteria Nitrosomonadales Ferriphaselus amnicola WP 062627400.1 315 aa
Pseudomonadati Pseudomonadota Betaproteobacteria Nitrosomonadales Candidatus Nitrotoga WP 239185911.1 316 aa

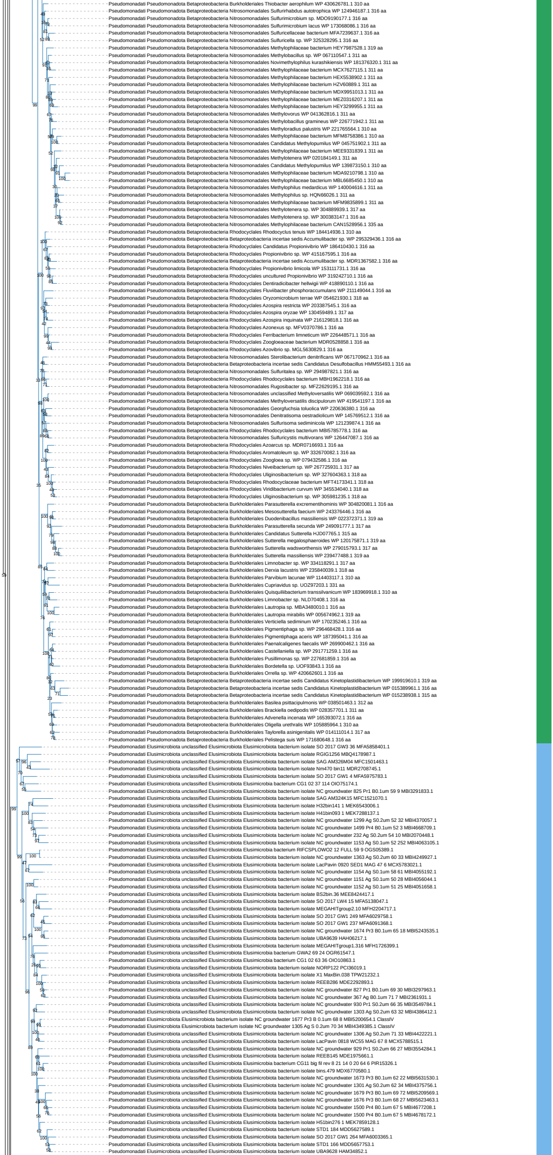

Pseudomonadati Pseudomonadota Betaproteobacteria Burkholderiales Thiobacter aerophilum WP 430626781.1 310 aa
Pseudomonadati Pseudomonadota Betaproteobacteria Nitrosomonadales Sulfurirhabdus autotrophica WP 124946187.1 316 aa
Pseudomonadati Pseudomonadota Betaproteobacteria Nitrosomonadales Sulfurimicrobium sp. MDO9190177.1 316 aa
Pseudomonadati Pseudomonadota Betaproteobacteria Nitrosomonadales Sulfurimicrobium lacus WP 173068086.1 316 aa
Pseudomonadati Pseudomonadota Betaproteobacteria Nitrosomonadales Sulfuricellaceae bacterium MFA7239637.1 316 aa
Pseudomonadati Pseudomonadota Betaproteobacteria Nitrosomonadales Sulfuricella sp. WP 325328295.1 316 aa
Pseudomonadati Pseudomonadota Betaproteobacteria Nitrosomonadales Methylophilaceae bacterium HEY7987528.1 319 aa
Pseudomonadati Pseudomonadota Betaproteobacteria Nitrosomonadales Methylobacillus sp. WP 067110547.1 311 aa
Pseudomonadati Pseudomonadota Betaproteobacteria Nitrosomonadales Novimethylophilus kurashikiensis WP 181376320.1 311 aa
Pseudomonadati Pseudomonadota Betaproteobacteria Nitrosomonadales Methylophilaceae bacterium MCX7627115.1 311 aa
Pseudomonadati Pseudomonadota Betaproteobacteria Nitrosomonadales Methylophilaceae bacterium HEX5538902.1 311 aa
Pseudomonadati Pseudomonadota Betaproteobacteria Nitrosomonadales Methylophilaceae bacterium HZV60889.1 311 aa
Pseudomonadati Pseudomonadota Betaproteobacteria Nitrosomonadales Methylophilaceae bacterium MDX9951013.1 311 aa
Pseudomonadati Pseudomonadota Betaproteobacteria Nitrosomonadales Methylophilaceae bacterium MEZ0316207.1 311 aa
Pseudomonadati Pseudomonadota Betaproteobacteria Nitrosomonadales Methylophilaceae bacterium HEY3299955.1 311 aa
Pseudomonadati Pseudomonadota Betaproteobacteria Nitrosomonadales Methylovorus WP 041362816.1 311 aa
Pseudomonadati Pseudomonadota Betaproteobacteria Nitrosomonadales Methylobacillus gramineus WP 226771942.1 311 aa
Pseudomonadati Pseudomonadota Betaproteobacteria Nitrosomonadales Methyloradius palustris WP 221765564.1 310 aa
Pseudomonadati Pseudomonadota Betaproteobacteria Nitrosomonadales Methylophilaceae bacterium MFM8758386.1 310 aa
Pseudomonadati Pseudomonadota Betaproteobacteria Nitrosomonadales Candidatus Methylopumilus WP 045751902.1 311 aa
Pseudomonadati Pseudomonadota Betaproteobacteria Nitrosomonadales Methylophilaceae bacterium MEE9331839.1 311 aa
Pseudomonadati Pseudomonadota Betaproteobacteria Nitrosomonadales Methylotenera WP 020184149.1 311 aa
Pseudomonadati Pseudomonadota Betaproteobacteria Nitrosomonadales Candidatus Methylopumilus WP 139873150.1 310 aa
Pseudomonadati Pseudomonadota Betaproteobacteria Nitrosomonadales Methylophilaceae bacterium MDA9210798.1 310 aa
Pseudomonadati Pseudomonadota Betaproteobacteria Nitrosomonadales Methylophilaceae bacterium MBL6685450.1 310 aa
Pseudomonadati Pseudomonadota Betaproteobacteria Nitrosomonadales Methylophilus medardicus WP 140004616.1 311 aa
Pseudomonadati Pseudomonadota Betaproteobacteria Nitrosomonadales Methylophilus sp. HQN66026.1 311 aa
Pseudomonadati Pseudomonadota Betaproteobacteria Nitrosomonadales Methylophilaceae bacterium MFM9835899.1 311 aa
Pseudomonadati Pseudomonadota Betaproteobacteria Nitrosomonadales Methylotenera sp. WP 304889939.1 317 aa
Pseudomonadati Pseudomonadota Betaproteobacteria Nitrosomonadales Methylotenera sp. WP 300383147.1 316 aa
Pseudomonadati Pseudomonadota Betaproteobacteria Nitrosomonadales Methylophilaceae bacterium CAN1528956.1 335 aa
Pseudomonadati Pseudomonadota Betaproteobacteria Rhodocyclales Rhodocyclus tenuis WP 184414936.1 310 aa
Pseudomonadati Pseudomonadota Betaproteobacteria Betaproteobacteria incertae sedis Accumulibacter sp. WP 295329436.1 316 aa
Pseudomonadati Pseudomonadota Betaproteobacteria Rhodocyclales Candidatus Propionivibrio WP 186410430.1 316 aa
Pseudomonadati Pseudomonadota Betaproteobacteria Rhodocyclales Propionivibrio sp. WP 415167595.1 316 aa
Pseudomonadati Pseudomonadota Betaproteobacteria Betaproteobacteria incertae sedis Accumulibacter sp. MDR1367582.1 316 aa
Pseudomonadati Pseudomonadota Betaproteobacteria Rhodocyclales Propionivibrio limicola WP 153111731.1 316 aa
Pseudomonadati Pseudomonadota Betaproteobacteria Rhodocyclales uncultured Propionivibrio WP 319242710.1 316 aa
Pseudomonadati Pseudomonadota Betaproteobacteria Rhodocyclales Dentiradicibacter hellwigii WP 418890110.1 316 aa
Pseudomonadati Pseudomonadota Betaproteobacteria Rhodocyclales Fluviibacter phosphoraccumulans WP 211149044.1 316 aa
Pseudomonadati Pseudomonadota Betaproteobacteria Rhodocyclales Oryzomicrobium terrae WP 054621930.1 318 aa
Pseudomonadati Pseudomonadota Betaproteobacteria Rhodocyclales Azospira restricta WP 203387545.1 316 aa
Pseudomonadati Pseudomonadota Betaproteobacteria Rhodocyclales Azospira oryzae WP 130459489.1 317 aa
Pseudomonadati Pseudomonadota Betaproteobacteria Rhodocyclales Azospira inquinata WP 216129818.1 316 aa
Pseudomonadati Pseudomonadota Betaproteobacteria Rhodocyclales Azonexus sp. MFV0370786.1 316 aa
Pseudomonadati Pseudomonadota Betaproteobacteria Rhodocyclales Ferribacterium limneticum WP 226448571.1 316 aa
Pseudomonadati Pseudomonadota Betaproteobacteria Rhodocyclales Zoogloeaceae bacterium MDR0528858.1 316 aa
Pseudomonadati Pseudomonadota Betaproteobacteria Rhodocyclales Azovibrio sp. MGL5630829.1 316 aa
Pseudomonadati Pseudomonadota Betaproteobacteria Nitrosomonadales Sterolibacterium denitrificans WP 067170962.1 316 aa
Pseudomonadati Pseudomonadota Betaproteobacteria Betaproteobacteria incertae sedis Candidatus Desulfobacillus HMM55493.1 316 aa
Pseudomonadati Pseudomonadota Betaproteobacteria Nitrosomonadales Sulfuritalea sp. WP 294987821.1 316 aa
Pseudomonadati Pseudomonadota Betaproteobacteria Rhodocyclales Rhodocyclales bacterium MBH1962218.1 316 aa
Pseudomonadati Pseudomonadota Betaproteobacteria Nitrosomonadales Rugosibacter sp. MFZ2629195.1 316 aa
Pseudomonadati Pseudomonadota Betaproteobacteria Nitrosomonadales unclassified Methyloversatilis WP 069039592.1 316 aa
Pseudomonadati Pseudomonadota Betaproteobacteria Nitrosomonadales Methyloversatilis discipulorum WP 419541197.1 316 aa
Pseudomonadati Pseudomonadota Betaproteobacteria Nitrosomonadales Georgfuchsia toluolica WP 220636380.1 316 aa
Pseudomonadati Pseudomonadota Betaproteobacteria Nitrosomonadales Denitratisoma oestradiolicum WP 145769512.1 316 aa
Pseudomonadati Pseudomonadota Betaproteobacteria Nitrosomonadales Sulfurisoma sediminicola WP 121239874.1 316 aa
Pseudomonadati Pseudomonadota Betaproteobacteria Rhodocyclales Rhodocyclales bacterium MBI5785778.1 316 aa
Pseudomonadati Pseudomonadota Betaproteobacteria Nitrosomonadales Sulfuricystis multivorans WP 126447087.1 316 aa
Pseudomonadati Pseudomonadota Betaproteobacteria Rhodocyclales Azoarcus sp. MDR0716693.1 316 aa
Pseudomonadati Pseudomonadota Betaproteobacteria Rhodocyclales Aromatoleum sp. WP 332670082.1 316 aa
Pseudomonadati Pseudomonadota Betaproteobacteria Rhodocyclales Zoogloea sp. WP 079432586.1 316 aa
Pseudomonadati Pseudomonadota Betaproteobacteria Rhodocyclales Niveibacterium sp. WP 267725931.1 317 aa
Pseudomonadati Pseudomonadota Betaproteobacteria Rhodocyclales Uliginosibacterium sp. WP 327604363.1 318 aa
Pseudomonadati Pseudomonadota Betaproteobacteria Rhodocyclales Rhodocyclaceae bacterium MFT4173341.1 318 aa
Pseudomonadati Pseudomonadota Betaproteobacteria Rhodocyclales Viridibacterium curvum WP 345534040.1 318 aa
Pseudomonadati Pseudomonadota Betaproteobacteria Rhodocyclales Uliginosibacterium sp. WP 305981235.1 318 aa
Pseudomonadati Pseudomonadota Betaproteobacteria Burkholderiales Parasutterella excrementihominis WP 304820081.1 316 aa
Pseudomonadati Pseudomonadota Betaproteobacteria Burkholderiales Mesosutterella faecium WP 243376446.1 316 aa
Pseudomonadati Pseudomonadota Betaproteobacteria Burkholderiales Duodenibacillus massiliensis WP 022372371.1 319 aa
Pseudomonadati Pseudomonadota Betaproteobacteria Burkholderiales Parasutterella secunda WP 249091777.1 317 aa
Pseudomonadati Pseudomonadota Betaproteobacteria Burkholderiales Candidatus Sutterella HJD07765.1 315 aa
Pseudomonadati Pseudomonadota Betaproteobacteria Burkholderiales Sutterella megalosphaeroides WP 120175871.1 319 aa
Pseudomonadati Pseudomonadota Betaproteobacteria Burkholderiales Sutterella wadsworthensis WP 279015793.1 317 aa
Pseudomonadati Pseudomonadota Betaproteobacteria Burkholderiales Sutterella massiliensis WP 239477488.1 319 aa
Pseudomonadati Pseudomonadota Betaproteobacteria Burkholderiales Limnobacter sp. WP 334118291.1 317 aa
Pseudomonadati Pseudomonadota Betaproteobacteria Burkholderiales Derxia lacustris WP 235840039.1 318 aa
Pseudomonadati Pseudomonadota Betaproteobacteria Burkholderiales Parvibium lacunae WP 114403117.1 310 aa
Pseudomonadati Pseudomonadota Betaproteobacteria Burkholderiales Cupriavidus sp. UOZ97203.1 331 aa
Pseudomonadati Pseudomonadota Betaproteobacteria Burkholderiales Quisquiliibacterium transsilvanicum WP 183969918.1 310 aa
Pseudomonadati Pseudomonadota Betaproteobacteria Burkholderiales Limnobacter sp. NLD70408.1 316 aa
Pseudomonadati Pseudomonadota Betaproteobacteria Burkholderiales Lautropia sp. MBA3480010.1 316 aa
Pseudomonadati Pseudomonadota Betaproteobacteria Burkholderiales Lautropia mirabilis WP 005674962.1 319 aa
Pseudomonadati Pseudomonadota Betaproteobacteria Burkholderiales Verticiella sediminum WP 170235246.1 316 aa
Pseudomonadati Pseudomonadota Betaproteobacteria Burkholderiales Pigmentiphaga sp. WP 296468428.1 316 aa
Pseudomonadati Pseudomonadota Betaproteobacteria Burkholderiales Pigmentiphaga aceris WP 187395041.1 316 aa
Pseudomonadati Pseudomonadota Betaproteobacteria Burkholderiales Paenalcaligenes faecalis WP 269900462.1 316 aa
Pseudomonadati Pseudomonadota Betaproteobacteria Burkholderiales Castellaniella sp. WP 291771259.1 316 aa
Pseudomonadati Pseudomonadota Betaproteobacteria Burkholderiales Pusillimonas sp. WP 227681859.1 316 aa
Pseudomonadati Pseudomonadota Betaproteobacteria Burkholderiales Bordetella sp. UOF93843.1 316 aa
Pseudomonadati Pseudomonadota Betaproteobacteria Burkholderiales Orrella sp. WP 420662601.1 316 aa
Pseudomonadati Pseudomonadota Betaproteobacteria Betaproteobacteria incertae sedis Candidatus Kinetoplastidibacterium WP 199919610.1 319 aa
Pseudomonadati Pseudomonadota Betaproteobacteria Betaproteobacteria incertae sedis Candidatus Kinetoplastidibacterium WP 015389961.1 316 aa
Pseudomonadati Pseudomonadota Betaproteobacteria Betaproteobacteria incertae sedis Candidatus Kinetoplastidibacterium WP 015238938.1 315 aa
Pseudomonadati Pseudomonadota Betaproteobacteria Burkholderiales Basilea psittacipulmonis WP 038501463.1 312 aa
Pseudomonadati Pseudomonadota Betaproteobacteria Burkholderiales Brackiella oedipodis WP 028357701.1 311 aa
Pseudomonadati Pseudomonadota Betaproteobacteria Burkholderiales Advenella incenata WP 165393072.1 316 aa
Pseudomonadati Pseudomonadota Betaproteobacteria Burkholderiales Oligella urethralis WP 105885964.1 310 aa
Pseudomonadati Pseudomonadota Betaproteobacteria Burkholderiales Taylorella asinigenitalis WP 014111014.1 317 aa
Pseudomonadati Pseudomonadota Betaproteobacteria Burkholderiales Pelistega suis WP 171680648.1 316 aa
Pseudomonadati Elusimicrobiota unclassified Elusimicrobiota Elusimicrobiota bacterium isolate SO 2017 GW3 36 MFA5858401.1
Pseudomonadati Elusimicrobiota unclassified Elusimicrobiota Elusimicrobiota bacterium isolate RGIG1256 MBQ4178987.1
Pseudomonadati Elusimicrobiota unclassified Elusimicrobiota Elusimicrobiota bacterium isolate SAG AM326M04 MFC1501463.1
Pseudomonadati Elusimicrobiota unclassified Elusimicrobiota Elusimicrobiota bacterium isolate Nm470 bin11 MDR2708745.1
Pseudomonadati Elusimicrobiota unclassified Elusimicrobiota Elusimicrobiota bacterium isolate SO 2017 GW1 4 MFA5975783.1
Pseudomonadati Elusimicrobiota unclassified Elusimicrobiota Elusimicrobia bacterium CG1 02 37 114 OIO75174.1
Pseudomonadati Elusimicrobiota unclassified Elusimicrobiota Elusimicrobiota bacterium isolate NC groundwater 825 Pr1 B0.1um 59 9 MBI3291833.1
Pseudomonadati Elusimicrobiota unclassified Elusimicrobiota Elusimicrobiota bacterium isolate SAG AM324K15 MFC1521070.1
Pseudomonadati Elusimicrobiota unclassified Elusimicrobiota Elusimicrobiota bacterium isolate H32bin141 1 MEK6543006.1
Pseudomonadati Elusimicrobiota unclassified Elusimicrobiota Elusimicrobiota bacterium isolate H41bin093 1 MEK7288137.1
Pseudomonadati Elusimicrobiota unclassified Elusimicrobiota Elusimicrobiota bacterium isolate NC groundwater 1299 Ag S0.2um 52 32 MBI4370057.1
Pseudomonadati Elusimicrobiota unclassified Elusimicrobiota Elusimicrobiota bacterium isolate NC groundwater 1499 Pr4 B0.1um 52 3 MBI4668709.1
Pseudomonadati Elusimicrobiota unclassified Elusimicrobiota Elusimicrobiota bacterium isolate NC groundwater 232 Ag S0.2um 54 10 MBI2070448.1
Pseudomonadati Elusimicrobiota unclassified Elusimicrobiota Elusimicrobiota bacterium isolate NC groundwater 1153 Ag S0.1um 52 252 MBI4063105.1
Pseudomonadati Elusimicrobiota unclassified Elusimicrobiota Elusimicrobia bacterium RIFCSPLOWO2 12 FULL 59 9 OGS05389.1
Pseudomonadati Elusimicrobiota unclassified Elusimicrobiota Elusimicrobiota bacterium isolate NC groundwater 1363 Ag S0.2um 60 33 MBI4249927.1
Pseudomonadati Elusimicrobiota unclassified Elusimicrobiota Elusimicrobiota bacterium isolate LacPavin 0920 SED1 MAG 47 6 MCX5783021.1
Pseudomonadati Elusimicrobiota unclassified Elusimicrobiota Elusimicrobiota bacterium isolate NC groundwater 1154 Ag S0.1um 58 61 MBI4055192.1
Pseudomonadati Elusimicrobiota unclassified Elusimicrobiota Elusimicrobiota bacterium isolate NC groundwater 1151 Ag S0.1um 50 28 MBI4056044.1
Pseudomonadati Elusimicrobiota unclassified Elusimicrobiota Elusimicrobiota bacterium isolate NC groundwater 1152 Ag S0.1um 51 25 MBI4051658.1
Pseudomonadati Elusimicrobiota unclassified Elusimicrobiota Elusimicrobiota bacterium isolate BS2bin.36 MEE8424417.1
Pseudomonadati Elusimicrobiota unclassified Elusimicrobiota Elusimicrobiota bacterium isolate SO 2017 LW4 15 MFA5138047.1
Pseudomonadati Elusimicrobiota unclassified Elusimicrobiota Elusimicrobiota bacterium isolate MEGAHITgroup2.10 MFH2204717.1
Pseudomonadati Elusimicrobiota unclassified Elusimicrobiota Elusimicrobiota bacterium isolate SO 2017 GW1 249 MFA6029758.1
Pseudomonadati Elusimicrobiota unclassified Elusimicrobiota Elusimicrobiota bacterium isolate SO 2017 GW1 237 MFA6091368.1
Pseudomonadati Elusimicrobiota unclassified Elusimicrobiota Elusimicrobiota bacterium isolate NC groundwater 1674 Pr3 B0.1um 65 18 MBI5243535.1
Pseudomonadati Elusimicrobiota unclassified Elusimicrobiota Elusimicrobiota bacterium isolate UBA9639 HAH06217.1
Pseudomonadati Elusimicrobiota unclassified Elusimicrobiota Elusimicrobiota bacterium isolate MEGAHITgroup1.316 MFH1726399.1
Pseudomonadati Elusimicrobiota unclassified Elusimicrobiota Elusimicrobia bacterium GWA2 69 24 OGR61547.1
Pseudomonadati Elusimicrobiota unclassified Elusimicrobiota Elusimicrobia bacterium CG1 02 63 36 OIO10863.1
Pseudomonadati Elusimicrobiota unclassified Elusimicrobiota Elusimicrobiota bacterium isolate NORP122 PCI36019.1
Pseudomonadati Elusimicrobiota unclassified Elusimicrobiota Elusimicrobiota bacterium isolate X1 MaxBin.038 TPW21232.1
Pseudomonadati Elusimicrobiota unclassified Elusimicrobiota Elusimicrobiota bacterium isolate REEB286 MDE2292893.1
Pseudomonadati Elusimicrobiota unclassified Elusimicrobiota Elusimicrobiota bacterium isolate NC groundwater 827 Pr1 B0.1um 69 30 MBI3297963.1
Pseudomonadati Elusimicrobiota unclassified Elusimicrobiota Elusimicrobiota bacterium isolate NC groundwater 367 Ag B0.1um 71 7 MBI2361931.1
Pseudomonadati Elusimicrobiota unclassified Elusimicrobiota Elusimicrobiota bacterium isolate NC groundwater 930 Pr1 S0.2um 66 35 MBI3549784.1
Pseudomonadati Elusimicrobiota unclassified Elusimicrobiota Elusimicrobiota bacterium isolate NC groundwater 1303 Ag S0.2um 63 32 MBI4386412.1
Pseudomonadati Elusimicrobiota Elusimicrobiota bacterium isolate NC groundwater 1677 Pr3 B 0.1um 68 8 MBI5200654.1 ClassIV
Pseudomonadati Elusimicrobiota Elusimicrobiota bacterium isolate NC groundwater 1305 Ag S 0.2um 70 34 MBI4349385.1 ClassIV
Pseudomonadati Elusimicrobiota unclassified Elusimicrobiota Elusimicrobiota bacterium isolate NC groundwater 1306 Ag S0.2um 71 33 MBI4422221.1
Pseudomonadati Elusimicrobiota unclassified Elusimicrobiota Elusimicrobiota bacterium isolate LacPavin 0818 WC55 MAG 67 8 MCX5788515.1
Pseudomonadati Elusimicrobiota unclassified Elusimicrobiota Elusimicrobiota bacterium isolate NC groundwater 929 Pr1 S0.2um 66 27 MBI3554284.1
Pseudomonadati Elusimicrobiota unclassified Elusimicrobiota Elusimicrobiota bacterium isolate REEB145 MDE1975661.1
Pseudomonadati Elusimicrobiota unclassified Elusimicrobiota Elusimicrobia bacterium CG11 big fil rev 8 21 14 0 20 64 6 PIR15326.1
Pseudomonadati Elusimicrobiota unclassified Elusimicrobiota Elusimicrobiota bacterium isolate bins.479 MDX6770580.1
Pseudomonadati Elusimicrobiota unclassified Elusimicrobiota Elusimicrobiota bacterium isolate NC groundwater 1673 Pr3 B0.1um 62 22 MBI5631530.1
Pseudomonadati Elusimicrobiota unclassified Elusimicrobiota Elusimicrobiota bacterium isolate NC groundwater 1301 Ag S0.2um 62 34 MBI4375756.1
Pseudomonadati Elusimicrobiota unclassified Elusimicrobiota Elusimicrobiota bacterium isolate NC groundwater 1679 Pr3 B0.1um 69 72 MBI5209569.1
Pseudomonadati Elusimicrobiota unclassified Elusimicrobiota Elusimicrobiota bacterium isolate NC groundwater 1676 Pr3 B0.1um 68 27 MBI5623463.1
Pseudomonadati Elusimicrobiota unclassified Elusimicrobiota Elusimicrobiota bacterium isolate NC groundwater 1500 Pr4 B0.1um 67 5 MBI4677208.1
Pseudomonadati Elusimicrobiota unclassified Elusimicrobiota Elusimicrobiota bacterium isolate NC groundwater 1500 Pr4 B0.1um 67 5 MBI4678172.1
Pseudomonadati Elusimicrobiota unclassified Elusimicrobiota Elusimicrobiota bacterium isolate H51bin276 1 MEK7859128.1
Pseudomonadati Elusimicrobiota unclassified Elusimicrobiota Elusimicrobiota bacterium isolate STD1 184 MDD5627589.1
Pseudomonadati Elusimicrobiota unclassified Elusimicrobiota Elusimicrobiota bacterium isolate SO 2017 GW1 264 MFA6003365.1
Pseudomonadati Elusimicrobiota unclassified Elusimicrobiota Elusimicrobiota bacterium isolate STD1 166 MDD5657753.1
Pseudomonadati Elusimicrobiota unclassified Elusimicrobiota Elusimicrobiota bacterium isolate UBA9628 HAM34852.1

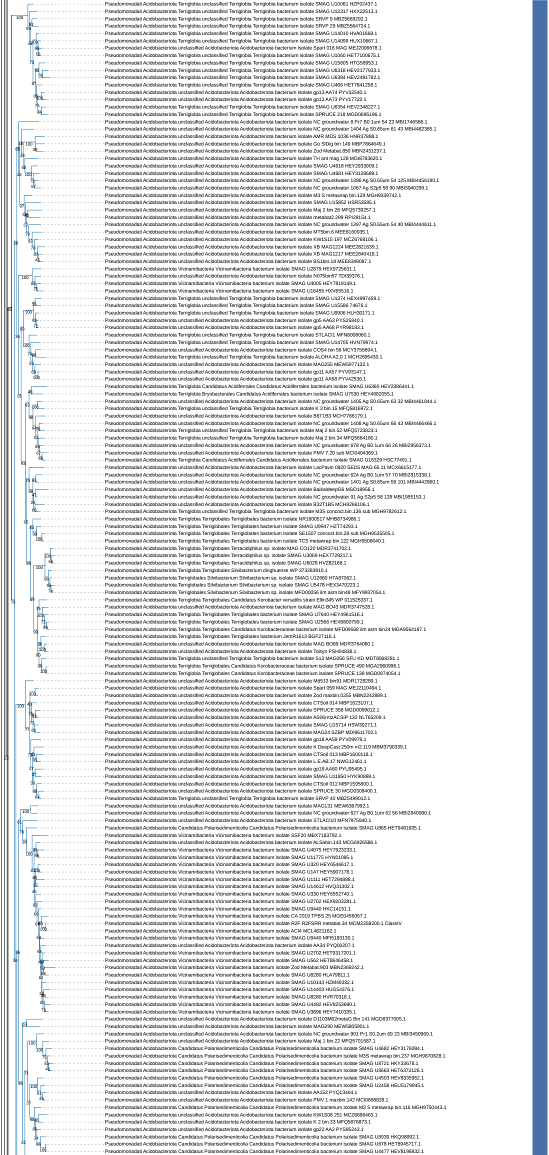

Pseudomonadati Acidobacteriota Terriglobia unclassified Terriglobia Terriglobia bacterium isolate SMAG U10061 HZP02437.1
Pseudomonadati Acidobacteriota Terriglobia unclassified Terriglobia Terriglobia bacterium isolate SMAG U12317 HXX22513.1
Pseudomonadati Acidobacteriota Terriglobia unclassified Terriglobia Terriglobia bacterium isolate SRVP 9 MBZ5669292.1
Pseudomonadati Acidobacteriota Terriglobia unclassified Terriglobia Terriglobia bacterium isolate SRVP 29 MBZ5564724.1
Pseudomonadati Acidobacteriota Terriglobia unclassified Terriglobia Terriglobia bacterium isolate SMAG U14010 HVA01669.1
Pseudomonadati Acidobacteriota Terriglobia unclassified Terriglobia Terriglobia bacterium isolate SMAG U14099 HUX10667.1
Pseudomonadati Acidobacteriota unclassified Acidobacteriota Acidobacteriota bacterium isolate Spart 016 MAG MEJ2006678.1
Pseudomonadati Acidobacteriota Terriglobia unclassified Terriglobia Terriglobia bacterium isolate SMAG U1060 HET7100675.1
Pseudomonadati Acidobacteriota Terriglobia unclassified Terriglobia Terriglobia bacterium isolate SMAG U15605 HTG58953.1
Pseudomonadati Acidobacteriota Terriglobia unclassified Terriglobia Terriglobia bacterium isolate SMAG U6318 HEV2177933.1
Pseudomonadati Acidobacteriota Terriglobia unclassified Terriglobia Terriglobia bacterium isolate SMAG U6384 HEV2491782.1
Pseudomonadati Acidobacteriota Terriglobia unclassified Terriglobia Terriglobia bacterium isolate SMAG U466 HET7841258.1
Pseudomonadati Acidobacteriota unclassified Acidobacteriota Acidobacteriota bacterium isolate gp13 AA74 PYV32540.1
Pseudomonadati Acidobacteriota unclassified Acidobacteriota Acidobacteriota bacterium isolate gp13 AA73 PYV17722.1
Pseudomonadati Acidobacteriota Terriglobia unclassified Terriglobia Terriglobia bacterium isolate SMAG U6354 HEV2348227.1
Pseudomonadati Acidobacteriota Terriglobia unclassified Terriglobia Terriglobia bacterium isolate SPRUCE 218 MGD0695196.1
Pseudomonadati Acidobacteriota unclassified Acidobacteriota Acidobacteriota bacterium isolate NC groundwater 8 Pr7 B0.1um 54 23 MBI1746566.1
Pseudomonadati Acidobacteriota unclassified Acidobacteriota Acidobacteriota bacterium isolate NC groundwater 1404 Ag S0.65um 61 43 MBI4482365.1
Pseudomonadati Acidobacteriota unclassified Acidobacteriota Acidobacteriota bacterium isolate AMR MDS 1036 HNR37888.1
Pseudomonadati Acidobacteriota unclassified Acidobacteriota Acidobacteriota bacterium isolate Go SlDig bin 149 MBP7864649.1
Pseudomonadati Acidobacteriota unclassified Acidobacteriota Acidobacteriota bacterium isolate Zod Metabat.850 MBN2431237.1
Pseudomonadati Acidobacteriota unclassified Acidobacteriota Acidobacteriota bacterium isolate TH ant mag 128 MGI8783620.1
Pseudomonadati Acidobacteriota unclassified Acidobacteriota Acidobacteriota bacterium isolate SMAG U4618 HEY2933909.1
Pseudomonadati Acidobacteriota unclassified Acidobacteriota Acidobacteriota bacterium isolate SMAG U4681 HEY3128689.1
Pseudomonadati Acidobacteriota unclassified Acidobacteriota Acidobacteriota bacterium isolate NC groundwater 1396 Ag S0.65um 54 125 MBI4456190.1
Pseudomonadati Acidobacteriota unclassified Acidobacteriota Acidobacteriota bacterium isolate NC groundwater 1067 Ag S2p5 58 90 MBI3940299.1
Pseudomonadati Acidobacteriota unclassified Acidobacteriota Acidobacteriota bacterium isolate M3 S metawrap bin.129 MGH9339742.1
Pseudomonadati Acidobacteriota unclassified Acidobacteriota Acidobacteriota bacterium isolate SMAG U15852 HSR53585.1
Pseudomonadati Acidobacteriota unclassified Acidobacteriota Acidobacteriota bacterium isolate Maj 2 bin.26 MFQ5739257.1
Pseudomonadati Acidobacteriota unclassified Acidobacteriota Acidobacteriota bacterium isolate metabat2.299 RPI29154.1
Pseudomonadati Acidobacteriota unclassified Acidobacteriota Acidobacteriota bacterium isolate NC groundwater 1397 Ag S0.65um 54 40 MBI4444611.1
Pseudomonadati Acidobacteriota unclassified Acidobacteriota Acidobacteriota bacterium isolate MT5bin.6 MEE8160935.1
Pseudomonadati Acidobacteriota unclassified Acidobacteriota Acidobacteriota bacterium isolate KW1S15 197 MCZ6768106.1
Pseudomonadati Acidobacteriota unclassified Acidobacteriota Acidobacteriota bacterium isolate XB MAG1224 MEE2821639.1
Pseudomonadati Acidobacteriota unclassified Acidobacteriota Acidobacteriota bacterium isolate XB MAG1217 MEE2840418.1
Pseudomonadati Acidobacteriota unclassified Acidobacteriota Acidobacteriota bacterium isolate BS1bin.18 MEE8349087.1
Pseudomonadati Acidobacteriota Vicinamibacteria Vicinamibacteria bacterium isolate SMAG U2879 HEX9725631.1
Pseudomonadati Acidobacteriota unclassified Acidobacteriota Acidobacteriota bacterium isolate N075bin57 TDI39376.1
Pseudomonadati Acidobacteriota Vicinamibacteria Vicinamibacteria bacterium isolate SMAG U4005 HEY7819149.1
Pseudomonadati Acidobacteriota Vicinamibacteria Vicinamibacteria bacterium isolate SMAG U16455 HXV65516.1
Pseudomonadati Acidobacteriota Terriglobia unclassified Terriglobia Terriglobia bacterium isolate SMAG U1374 HEX4997459.1
Pseudomonadati Acidobacteriota Terriglobia unclassified Terriglobia Terriglobia bacterium isolate SMAG U15586 74676.1
Pseudomonadati Acidobacteriota Terriglobia unclassified Terriglobia Terriglobia bacterium isolate SMAG U9906 HLH30171.1
Pseudomonadati Acidobacteriota unclassified Acidobacteriota Acidobacteriota bacterium isolate gp5 AA63 PYS25840.1
Pseudomonadati Acidobacteriota unclassified Acidobacteriota Acidobacteriota bacterium isolate gp5 AA68 PYR98183.1
Pseudomonadati Acidobacteriota Terriglobia unclassified Terriglobia Terriglobia bacterium isolate STLACI1 MFN8008060.1
Pseudomonadati Acidobacteriota Terriglobia unclassified Terriglobia Terriglobia bacterium isolate SMAG U14705 HVN79974.1
Pseudomonadati Acidobacteriota unclassified Acidobacteriota Acidobacteriota bacterium isolate COS4 bin 56 MCY3756694.1
Pseudomonadati Acidobacteriota Terriglobia unclassified Terriglobia Terriglobia bacterium isolate ALOHA A2.0 1 MCH2695430.1
Pseudomonadati Acidobacteriota unclassified Acidobacteriota Acidobacteriota bacterium isolate MAG255 MEW5977132.1
Pseudomonadati Acidobacteriota unclassified Acidobacteriota Acidobacteriota bacterium isolate gp11 AA57 PYV93147.1
Pseudomonadati Acidobacteriota unclassified Acidobacteriota Acidobacteriota bacterium isolate gp11 AA58 PYV42536.1
Pseudomonadati Acidobacteriota Terriglobia Candidatus Acidiferrales Candidatus Acidiferrales bacterium isolate SMAG U6360 HEV2386441.1
Pseudomonadati Acidobacteriota Terriglobia Bryobacterales Candidatus Acidiferrales bacterium isolate SMAG U7530 HEY4682055.1
Pseudomonadati Acidobacteriota unclassified Acidobacteriota Acidobacteriota bacterium isolate NC groundwater 1405 Ag S0.65um 63 32 MBI4461844.1
Pseudomonadati Acidobacteriota Terriglobia unclassified Terriglobia Terriglobia bacterium isolate K 3 bin.15 MFQ5816972.1
Pseudomonadati Acidobacteriota unclassified Acidobacteriota Acidobacteriota bacterium isolate B6T1B3 MCH7766179.1
Pseudomonadati Acidobacteriota unclassified Acidobacteriota Acidobacteriota bacterium isolate NC groundwater 1408 Ag S0.65um 66 43 MBI4466466.1
Pseudomonadati Acidobacteriota Terriglobia unclassified Terriglobia Terriglobia bacterium isolate Maj 2 bin.52 MFQ5723823.1
Pseudomonadati Acidobacteriota Terriglobia unclassified Terriglobia Terriglobia bacterium isolate Maj 2 bin.34 MFQ5664180.1
Pseudomonadati Acidobacteriota unclassified Acidobacteriota Acidobacteriota bacterium isolate NC groundwater 678 Ag B0.1um 66 26 MBI2956373.1
Pseudomonadati Acidobacteriota unclassified Acidobacteriota Acidobacteriota bacterium isolate PMV 7.20 sub MCI0404369.1
Pseudomonadati Acidobacteriota Terriglobia Candidatus Acidiferrales Candidatus Acidiferrales bacterium isolate SMAG U16329 HSC77491.1
Pseudomonadati Acidobacteriota unclassified Acidobacteriota Acidobacteriota bacterium isolate LacPavin 0920 SED5 MAG 65 11 MCX6615177.1
Pseudomonadati Acidobacteriota unclassified Acidobacteriota Acidobacteriota bacterium isolate NC groundwater 624 Ag B0.1um 57 70 MBI2815338.1
Pseudomonadati Acidobacteriota unclassified Acidobacteriota Acidobacteriota bacterium isolate NC groundwater 1401 Ag S0.65um 59 101 MBI4442960.1
Pseudomonadati Acidobacteriota unclassified Acidobacteriota Acidobacteriota bacterium isolate BaikaldeepG6 MSO18956.1
Pseudomonadati Acidobacteriota unclassified Acidobacteriota Acidobacteriota bacterium isolate NC groundwater 91 Ag S2p5 58 128 MBI1955153.1
Pseudomonadati Acidobacteriota unclassified Acidobacteriota Acidobacteriota bacterium isolate B32T1B5 MCH8266106.1
Pseudomonadati Acidobacteriota Terriglobia unclassified Terriglobia Terriglobia bacterium isolate M3S concoct.bin.136 sub MGH9782612.1
Pseudomonadati Acidobacteriota Terriglobia Terriglobales Terriglobales bacterium isolate NR1800517 MHB8734986.1
Pseudomonadati Acidobacteriota Terriglobia Terriglobales Terriglobales bacterium isolate SMAG U9947 HZT74293.1
Pseudomonadati Acidobacteriota Terriglobia Terriglobales Terriglobales bacterium isolate SE1507 concoct.bin.28 sub MGH9535505.1
Pseudomonadati Acidobacteriota Terriglobia Terriglobales Terriglobales bacterium isolate TCS metawrap bin.122 MGH9506040.1
Pseudomonadati Acidobacteriota Terriglobia Terriglobales Terracidiphilus sp. isolate MAG CO120 MDR3741702.1
Pseudomonadati Acidobacteriota Terriglobia Terriglobales Terracidiphilus sp. isolate SMAG U3069 HEX7729217.1
Pseudomonadati Acidobacteriota Terriglobia Terriglobales Terracidiphilus sp. isolate SMAG U6028 HVZ82168.1
Pseudomonadati Acidobacteriota Terriglobia Terriglobales Silvibacterium dinghuense WP 373283910.1
Pseudomonadati Acidobacteriota Terriglobales Silvibacterium Silvibacterium sp. isolate SMAG U12660 HTA87062.1
Pseudomonadati Acidobacteriota Terriglobales Silvibacterium Silvibacterium sp. isolate SMAG U5478 HEX3470223.1
Pseudomonadati Acidobacteriota Terriglobales Silvibacterium Silvibacterium sp. isolate MFD00056 ilm asm bin48 MFY9937054.1
Pseudomonadati Acidobacteriota Terriglobia Terriglobales Candidatus Korobacter versatilis strain Ellin345 WP 011525337.1
Pseudomonadati Acidobacteriota unclassified Acidobacteriota Acidobacteriota bacterium isolate MAG BO43 MDR3747528.1
Pseudomonadati Acidobacteriota Terriglobia Terriglobales Terriglobales bacterium isolate SMAG U7640 HEY4961516.1
Pseudomonadati Acidobacteriota Terriglobia Terriglobales Terriglobales bacterium isolate SMAG U2566 HEX8800799.1
Pseudomonadati Acidobacteriota Terriglobia Terriglobales Candidatus Korobacteraceae bacterium isolate MFD09568 ilm asm bin24 MGA9564187.1
Pseudomonadati Acidobacteriota Terriglobia Terriglobales Terriglobales bacterium JemR1613 BGF27116.1
Pseudomonadati Acidobacteriota unclassified Acidobacteriota Acidobacteriota bacterium isolate MAG BO89 MDR3764060.1
Pseudomonadati Acidobacteriota unclassified Acidobacteriota Acidobacteriota bacterium isolate Tolsyn PSH04838.1
Pseudomonadati Acidobacteriota Terriglobia unclassified Terriglobia Terriglobia bacterium isolate S13 MAG056 SFU KD MDT8068281.1
Pseudomonadati Acidobacteriota Terriglobia Terriglobales Candidatus Korobacteraceae bacterium isolate SPRUCE 490 MGA2960996.1
Pseudomonadati Acidobacteriota Terriglobia Terriglobales Candidatus Korobacteraceae bacterium isolate SPRUCE 138 MGD0974054.1
Pseudomonadati Acidobacteriota unclassified Acidobacteriota Acidobacteriota bacterium isolate Md513 bin91 MDR1728289.1
Pseudomonadati Acidobacteriota unclassified Acidobacteriota Acidobacteriota bacterium isolate Spart 059 MAG MEJ2110494.1
Pseudomonadati Acidobacteriota unclassified Acidobacteriota Acidobacteriota bacterium isolate Zod maxbin.0255 MBN2242889.1
Pseudomonadati Acidobacteriota unclassified Acidobacteriota Acidobacteriota bacterium isolate CTSoil 014 MBP1623107.1
Pseudomonadati Acidobacteriota unclassified Acidobacteriota Acidobacteriota bacterium isolate SPRUCE 358 MGD0099012.1
Pseudomonadati Acidobacteriota unclassified Acidobacteriota Acidobacteriota bacterium isolate AS06rmzACSIP 132 NLT65209.1
Pseudomonadati Acidobacteriota unclassified Acidobacteriota Acidobacteriota bacterium isolate SMAG U15714 HSW39271.1
Pseudomonadati Acidobacteriota unclassified Acidobacteriota Acidobacteriota bacterium isolate MAG24 SZBP MDI9611702.1
Pseudomonadati Acidobacteriota unclassified Acidobacteriota Acidobacteriota bacterium isolate gp18 AA59 PYV09979.1
Pseudomonadati Acidobacteriota unclassified Acidobacteriota Acidobacteriota bacterium isolate K DeepCast 250m m2 119 MBM3790339.1
Pseudomonadati Acidobacteriota unclassified Acidobacteriota Acidobacteriota bacterium isolate CTSoil 013 MBP1600118.1
Pseudomonadati Acidobacteriota unclassified Acidobacteriota Acidobacteriota bacterium isolate L.E.AB.17 NWG12461.1
Pseudomonadati Acidobacteriota unclassified Acidobacteriota Acidobacteriota bacterium isolate gp18 AA60 PYU95495.1
Pseudomonadati Acidobacteriota unclassified Acidobacteriota Acidobacteriota bacterium isolate SMAG U11850 HYK90898.1
Pseudomonadati Acidobacteriota unclassified Acidobacteriota Acidobacteriota bacterium isolate CTSoil 012 MBP1595800.1
Pseudomonadati Acidobacteriota unclassified Acidobacteriota Acidobacteriota bacterium isolate SPRUCE 30 MGD0308400.1
Pseudomonadati Acidobacteriota Terriglobia unclassified Terriglobia Terriglobia bacterium isolate SRVP 40 MBZ5499012.1
Pseudomonadati Acidobacteriota unclassified Acidobacteriota Acidobacteriota bacterium isolate MAG131 MEW6367992.1
Pseudomonadati Acidobacteriota unclassified Acidobacteriota Acidobacteriota bacterium isolate NC groundwater 627 Ag B0.1um 62 56 MBI2840080.1
Pseudomonadati Acidobacteriota unclassified Acidobacteriota Acidobacteriota bacterium isolate STLACI10 MFN7975940.1
Pseudomonadati Acidobacteriota Candidatus Polarisedimenticolia Candidatus Polarisedimenticolia bacterium isolate SMAG U865 HET9481935.1
Pseudomonadati Acidobacteriota Vicinamibacteria Vicinamibacteria bacterium isolate SSF20 MBX7183792.1
Pseudomonadati Acidobacteriota unclassified Acidobacteriota Acidobacteriota bacterium isolate ALSabin.143 MCG6926586.1
Pseudomonadati Acidobacteriota Vicinamibacteria Vicinamibacteria bacterium isolate SMAG U4075 HEY7923233.1
Pseudomonadati Acidobacteriota Vicinamibacteria Vicinamibacteria bacterium isolate SMAG U11775 HYN01095.1
Pseudomonadati Acidobacteriota Vicinamibacteria Vicinamibacteria bacterium isolate SMAG U320 HEY6546617.1
Pseudomonadati Acidobacteriota Vicinamibacteria Vicinamibacteria bacterium isolate SMAG U147 HEY5907178.1
Pseudomonadati Acidobacteriota Vicinamibacteria Vicinamibacteria bacterium isolate SMAG U1111 HET7294888.1
Pseudomonadati Acidobacteriota Vicinamibacteria Vicinamibacteria bacterium isolate SMAG U14612 HVQ31302.1
Pseudomonadati Acidobacteriota Vicinamibacteria Vicinamibacteria bacterium isolate SMAG U330 HEY6552740.1
Pseudomonadati Acidobacteriota Vicinamibacteria Vicinamibacteria bacterium isolate SMAG U2702 HEX9203281.1
Pseudomonadati Acidobacteriota Vicinamibacteria Vicinamibacteria bacterium isolate SMAG U9440 HKC14151.1
Pseudomonadati Acidobacteriota Vicinamibacteria Vicinamibacteria bacterium isolate CA 2019 TPBS 25 MGE0456067.1
Pseudomonadati Acidobacteriota Vicinamibacteria Vicinamibacteria bacterium isolate R2F R2FSRR metabat.34 MCM2258200.1 ClassIV
Pseudomonadati Acidobacteriota Vicinamibacteria Vicinamibacteria bacterium isolate ACI4 MCL4821162.1
Pseudomonadati Acidobacteriota Vicinamibacteria Vicinamibacteria bacterium isolate SMAG U9440 MFI5183130.1
Pseudomonadati Acidobacteriota unclassified Acidobacteriota Acidobacteriota bacterium isolate AA34 PYQ00207.1
Pseudomonadati Acidobacteriota Vicinamibacteria Vicinamibacteria bacterium isolate SMAG U2702 HET9317201.1
Pseudomonadati Acidobacteriota Vicinamibacteria Vicinamibacteria bacterium isolate SMAG U562 HET8646458.1
Pseudomonadati Acidobacteriota Vicinamibacteria Vicinamibacteria bacterium isolate Zod Metabat.903 MBN2369242.1
Pseudomonadati Acidobacteriota Vicinamibacteria Vicinamibacteria bacterium isolate SMAG U8280 HLA79811.1
Pseudomonadati Acidobacteriota Vicinamibacteria Vicinamibacteria bacterium isolate SMAG U10143 HZM49332.1
Pseudomonadati Acidobacteriota Vicinamibacteria Vicinamibacteria bacterium isolate SMAG U14403 HUG54376.1
Pseudomonadati Acidobacteriota Vicinamibacteria Vicinamibacteria bacterium isolate SMAG U8280 HVR70318.1
Pseudomonadati Acidobacteriota Vicinamibacteria Vicinamibacteria bacterium isolate SMAG U4492 HEV8253690.1
Pseudomonadati Acidobacteriota Vicinamibacteria Vicinamibacteria bacterium isolate SMAG U3896 HEY7410335.1
Pseudomonadati Acidobacteriota unclassified Acidobacteriota Acidobacteriota bacterium isolate D1103M02metaG Bin 141 MGD8377005.1
Pseudomonadati Acidobacteriota unclassified Acidobacteriota Acidobacteriota bacterium isolate MAG290 MEW5805901.1
Pseudomonadati Acidobacteriota unclassified Acidobacteriota Acidobacteriota bacterium isolate NC groundwater 901 Pr1 S0.2um 69 23 MBI3450969.1
Pseudomonadati Acidobacteriota unclassified Acidobacteriota Acidobacteriota bacterium isolate Maj 1 bin.22 MFQ5701687.1
Pseudomonadati Acidobacteriota Candidatus Polarisedimenticolia Candidatus Polarisedimenticolia bacterium isolate SMAG U4682 HEY3176084.1
Pseudomonadati Acidobacteriota Candidatus Polarisedimenticolia Candidatus Polarisedimenticolia bacterium isolate M3S metawrap bin.237 MGH9870628.1
Pseudomonadati Acidobacteriota Candidatus Polarisedimenticolia Candidatus Polarisedimenticolia bacterium isolate SMAG U8721 HKY33678.1
Pseudomonadati Acidobacteriota Candidatus Polarisedimenticolia Candidatus Polarisedimenticolia bacterium isolate SMAG U8663 HET6372126.1
Pseudomonadati Acidobacteriota Candidatus Polarisedimenticolia Candidatus Polarisedimenticolia bacterium isolate SMAG U4503 HEV8335952.1
Pseudomonadati Acidobacteriota Candidatus Polarisedimenticolia Candidatus Polarisedimenticolia bacterium isolate SMAG U2458 HEU5179945.1
Pseudomonadati Acidobacteriota unclassified Acidobacteriota Acidobacteriota bacterium isolate AA152 PYQ13464.1
Pseudomonadati Acidobacteriota unclassified Acidobacteriota Acidobacteriota bacterium isolate PMV 1 maxbin.142 MCI0658828.1
Pseudomonadati Acidobacteriota Candidatus Polarisedimenticolia Candidatus Polarisedimenticolia bacterium isolate M3 S metawrap bin.116 MGH9750443.1
Pseudomonadati Acidobacteriota unclassified Acidobacteriota Acidobacteriota bacterium isolate KW1S08 251 MCZ6696463.1
Pseudomonadati Acidobacteriota unclassified Acidobacteriota Acidobacteriota bacterium isolate K 2 bin.33 MFQ5876873.1
Pseudomonadati Acidobacteriota unclassified Acidobacteriota Acidobacteriota bacterium isolate gp22 AA2 PYS95243.1
Pseudomonadati Acidobacteriota Candidatus Polarisedimenticolia Candidatus Polarisedimenticolia bacterium isolate SMAG U8939 HKQ98992.1
Pseudomonadati Acidobacteriota Candidatus Polarisedimenticolia Candidatus Polarisedimenticolia bacterium isolate SMAG U678 HET8945717.1
Pseudomonadati Acidobacteriota Candidatus Polarisedimenticolia Candidatus Polarisedimenticolia bacterium isolate SMAG U4477 HEV8198832.1

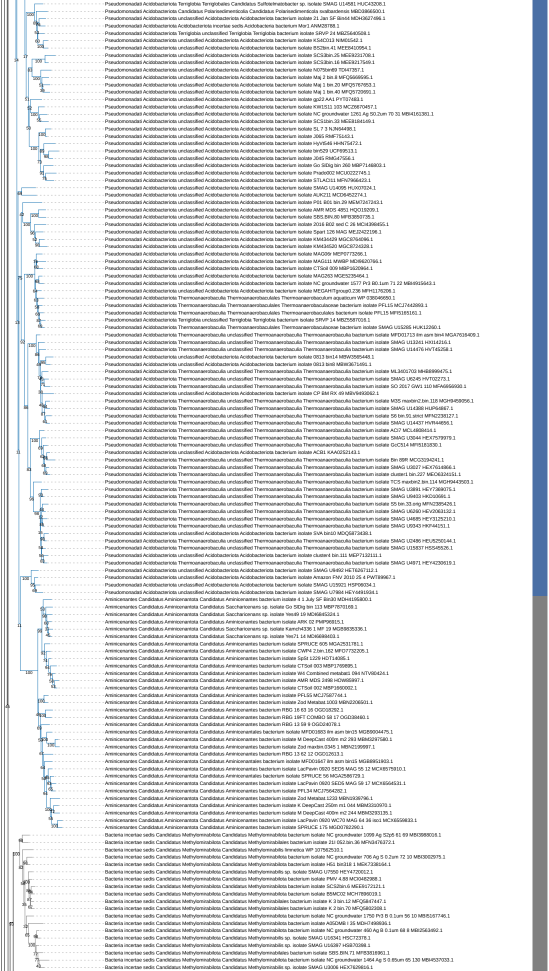
Pseudomonadati Acidobacteriota Terriglobia Terriglobales Candidatus Sulfotelmatobacter sp. isolate SMAG U14581 HUC43208.1
Pseudomonadati Acidobacteriota Candidatus Polarisedimenticolia Candidatus Polarisedimenticola svalbardensis MBD3866500.1
Pseudomonadati Acidobacteriota unclassified Acidobacteriota Acidobacteriota bacterium isolate 21 Jan SF Bin44 MDH3627496.1
Pseudomonadati Acidobacteriota Acidobacteriota incertae sedis Acidobacteria bacterium Mor1 ANM28788.1
Pseudomonadati Acidobacteriota Terriglobia unclassified Terriglobia Terriglobia bacterium isolate SRVP 24 MBZ5640508.1
Pseudomonadati Acidobacteriota unclassified Acidobacteriota Acidobacteriota bacterium isolate KS4C013 NIM01542.1
Pseudomonadati Acidobacteriota unclassified Acidobacteriota Acidobacteriota bacterium isolate BS2bin.41 MEE8410954.1
Pseudomonadati Acidobacteriota unclassified Acidobacteriota Acidobacteriota bacterium isolate SCS3bin.25 MEE9231708.1
Pseudomonadati Acidobacteriota unclassified Acidobacteriota Acidobacteriota bacterium isolate SCS3bin.16 MEE9217549.1
Pseudomonadati Acidobacteriota unclassified Acidobacteriota Acidobacteriota bacterium isolate N075bin69 TDI47357.1
Pseudomonadati Acidobacteriota unclassified Acidobacteriota Acidobacteriota bacterium isolate Maj 2 bin.8 MFQ5669595.1
Pseudomonadati Acidobacteriota unclassified Acidobacteriota Acidobacteriota bacterium isolate Maj 1 bin.20 MFQ5767653.1
Pseudomonadati Acidobacteriota unclassified Acidobacteriota Acidobacteriota bacterium isolate Maj 1 bin.40 MFQ5720691.1
Pseudomonadati Acidobacteriota unclassified Acidobacteriota Acidobacteriota bacterium isolate gp22 AA1 PYT07483.1
Pseudomonadati Acidobacteriota unclassified Acidobacteriota Acidobacteriota bacterium isolate KW1S11 103 MCZ6670457.1
Pseudomonadati Acidobacteriota unclassified Acidobacteriota Acidobacteriota bacterium isolate NC groundwater 1261 Ag S0.2um 70 31 MBI4161381.1
Pseudomonadati Acidobacteriota unclassified Acidobacteriota Acidobacteriota bacterium isolate SCS1bin.33 MEE8184149.1
Pseudomonadati Acidobacteriota unclassified Acidobacteriota Acidobacteriota bacterium isolate SL 7 3 NJN64498.1
Pseudomonadati Acidobacteriota unclassified Acidobacteriota Acidobacteriota bacterium isolate J065 RMF75143.1
Pseudomonadati Acidobacteriota unclassified Acidobacteriota Acidobacteriota bacterium isolate HyVt546 HHN75472.1
Pseudomonadati Acidobacteriota unclassified Acidobacteriota Acidobacteriota bacterium isolate bin529 UCF69513.1
Pseudomonadati Acidobacteriota unclassified Acidobacteriota Acidobacteriota bacterium isolate J045 RMG47556.1
Pseudomonadati Acidobacteriota unclassified Acidobacteriota Acidobacteriota bacterium isolate Go SlDig bin 260 MBP7146803.1
Pseudomonadati Acidobacteriota unclassified Acidobacteriota Acidobacteriota bacterium isolate Prado002 MCU0222745.1
Pseudomonadati Acidobacteriota unclassified Acidobacteriota Acidobacteriota bacterium isolate STLACI11 MFN7966423.1
Pseudomonadati Acidobacteriota unclassified Acidobacteriota Acidobacteriota bacterium isolate SMAG U14095 HUX07024.1
Pseudomonadati Acidobacteriota unclassified Acidobacteriota Acidobacteriota bacterium isolate AUK211 MCD6452274.1
Pseudomonadati Acidobacteriota unclassified Acidobacteriota Acidobacteriota bacterium isolate P01 B01 bin.29 MEM7247243.1
Pseudomonadati Acidobacteriota unclassified Acidobacteriota Acidobacteriota bacterium isolate AMR MDS 4851 HQO19209.1
Pseudomonadati Acidobacteriota unclassified Acidobacteriota Acidobacteriota bacterium isolate SBS.BIN.80 MFB3850735.1
Pseudomonadati Acidobacteriota unclassified Acidobacteriota Acidobacteriota bacterium isolate 2016 B02 sed C 26 MCI4398455.1
Pseudomonadati Acidobacteriota unclassified Acidobacteriota Acidobacteriota bacterium isolate Spart 126 MAG MEJ2422196.1
Pseudomonadati Acidobacteriota unclassified Acidobacteriota Acidobacteriota bacterium isolate KM434429 MGC8764096.1
Pseudomonadati Acidobacteriota unclassified Acidobacteriota Acidobacteriota bacterium isolate KM434520 MGC8724328.1
Pseudomonadati Acidobacteriota unclassified Acidobacteriota Acidobacteriota bacterium isolate MAG06r MEP0773266.1
Pseudomonadati Acidobacteriota unclassified Acidobacteriota Acidobacteriota bacterium isolate MAG111 MWBP MDI9620766.1
Pseudomonadati Acidobacteriota unclassified Acidobacteriota Acidobacteriota bacterium isolate CTSoil 009 MBP1620964.1
Pseudomonadati Acidobacteriota unclassified Acidobacteriota Acidobacteriota bacterium isolate MAG263 MGE5235464.1
Pseudomonadati Acidobacteriota unclassified Acidobacteriota Acidobacteriota bacterium isolate NC groundwater 1577 Pr3 B0.1um 71 22 MBI4915643.1
Pseudomonadati Acidobacteriota unclassified Acidobacteriota Acidobacteriota bacterium isolate MEGAHITgroup0.236 MFH1176206.1
Pseudomonadati Acidobacteriota Thermoanaerobaculia Thermoanaerobaculales Thermoanaerobaculum aquaticum WP 038046650.1
Pseudomonadati Acidobacteriota Thermoanaerobaculia Thermoanaerobaculales Thermoanaerobaculaceae bacterium isolate PFL15 MCJ7442893.1
Pseudomonadati Acidobacteriota Thermoanaerobaculia Thermoanaerobaculales Thermoanaerobaculales bacterium isolate PFL15 MFI5165161.1
Pseudomonadati Acidobacteriota Terriglobia unclassified Terriglobia Terriglobia bacterium isolate SRVP 14 MBZ5587016.1
Pseudomonadati Acidobacteriota Thermoanaerobaculia Thermoanaerobaculales Thermoanaerobaculaceae bacterium isolate SMAG U15285 HUK12260.1
Pseudomonadati Acidobacteriota Thermoanaerobaculia unclassified Thermoanaerobaculia Thermoanaerobaculia bacterium isolate MFD01713 ilm asm bin4 MGA7616409.1
Pseudomonadati Acidobacteriota Thermoanaerobaculia unclassified Thermoanaerobaculia Thermoanaerobaculia bacterium isolate SMAG U13241 HXI14216.1
Pseudomonadati Acidobacteriota Thermoanaerobaculia unclassified Thermoanaerobaculia Thermoanaerobaculia bacterium isolate SMAG U14476 HVT45258.1
Pseudomonadati Acidobacteriota unclassified Acidobacteriota Acidobacteriota bacterium isolate 0813 bin14 MBW3565448.1
Pseudomonadati Acidobacteriota unclassified Acidobacteriota Acidobacteriota bacterium isolate 0813 bin8 MBW3671491.1
Pseudomonadati Acidobacteriota Thermoanaerobaculia unclassified Thermoanaerobaculia Thermoanaerobaculia bacterium isolate ML3401703 MHB8999475.1
Pseudomonadati Acidobacteriota Thermoanaerobaculia unclassified Thermoanaerobaculia Thermoanaerobaculia bacterium isolate SMAG U6245 HVT02273.1
Pseudomonadati Acidobacteriota Thermoanaerobaculia unclassified Thermoanaerobaculia Thermoanaerobaculia bacterium isolate SO 2017 GW1 110 MFA6956930.1
Pseudomonadati Acidobacteriota unclassified Acidobacteriota Acidobacteriota bacterium isolate CP BM RX 49 MBV9493062.1
Pseudomonadati Acidobacteriota Thermoanaerobaculia unclassified Thermoanaerobaculia Thermoanaerobaculia bacterium isolate M3S maxbin2.bin.118 MGH9459056.1
Pseudomonadati Acidobacteriota Thermoanaerobaculia unclassified Thermoanaerobaculia Thermoanaerobaculia bacterium isolate SMAG U14388 HUP64867.1
Pseudomonadati Acidobacteriota Thermoanaerobaculia unclassified Thermoanaerobaculia Thermoanaerobaculia bacterium isolate S6 bin.91.strict MFN2238127.1
Pseudomonadati Acidobacteriota Thermoanaerobaculia unclassified Thermoanaerobaculia Thermoanaerobaculia bacterium isolate SMAG U14437 HVR44656.1
Pseudomonadati Acidobacteriota Thermoanaerobaculia unclassified Thermoanaerobaculia Thermoanaerobaculia bacterium isolate ACI7 MCL4808414.1
Pseudomonadati Acidobacteriota Thermoanaerobaculia unclassified Thermoanaerobaculia Thermoanaerobaculia bacterium isolate SMAG U3044 HEX7579979.1
Pseudomonadati Acidobacteriota Thermoanaerobaculia unclassified Thermoanaerobaculia Thermoanaerobaculia bacterium isolate GcC514 MFI5181830.1
Pseudomonadati Acidobacteriota unclassified Acidobacteriota Acidobacteriota bacterium isolate ACB1 KAA0252143.1
Pseudomonadati Acidobacteriota Thermoanaerobaculia unclassified Thermoanaerobaculia Thermoanaerobaculia bacterium isolate Bin 89R MCG3194241.1
Pseudomonadati Acidobacteriota Thermoanaerobaculia unclassified Thermoanaerobaculia Thermoanaerobaculia bacterium isolate SMAG U3027 HEX7614866.1
Pseudomonadati Acidobacteriota Thermoanaerobaculia unclassified Thermoanaerobaculia Thermoanaerobaculia bacterium isolate cluster1 bin.227 MEO6324151.1
Pseudomonadati Acidobacteriota Thermoanaerobaculia unclassified Thermoanaerobaculia Thermoanaerobaculia bacterium isolate TCS maxbin2.bin.114 MGH9443503.1
Pseudomonadati Acidobacteriota Thermoanaerobaculia unclassified Thermoanaerobaculia Thermoanaerobaculia bacterium isolate SMAG U3891 HEY7369075.1
Pseudomonadati Acidobacteriota Thermoanaerobaculia unclassified Thermoanaerobaculia Thermoanaerobaculia bacterium isolate SMAG U9403 HKD10691.1
Pseudomonadati Acidobacteriota Thermoanaerobaculia unclassified Thermoanaerobaculia Thermoanaerobaculia bacterium isolate S5 bin.33.orig MFN2385426.1
Pseudomonadati Acidobacteriota Thermoanaerobaculia unclassified Thermoanaerobaculia Thermoanaerobaculia bacterium isolate SMAG U6260 HEV2063132.1
Pseudomonadati Acidobacteriota Thermoanaerobaculia unclassified Thermoanaerobaculia Thermoanaerobaculia bacterium isolate SMAG U4685 HEY3125210.1
Pseudomonadati Acidobacteriota Thermoanaerobaculia unclassified Thermoanaerobaculia Thermoanaerobaculia bacterium isolate SMAG U9343 HKF44151.1
Pseudomonadati Acidobacteriota unclassified Acidobacteriota Acidobacteriota bacterium isolate SVA bin10 MDQ5873438.1
Pseudomonadati Acidobacteriota Thermoanaerobaculia unclassified Thermoanaerobaculia Thermoanaerobaculia bacterium isolate SMAG U2486 HEU5250144.1
Pseudomonadati Acidobacteriota Thermoanaerobaculia unclassified Thermoanaerobaculia Thermoanaerobaculia bacterium isolate SMAG U15837 HSS45526.1
Pseudomonadati Acidobacteriota unclassified Acidobacteriota Acidobacteriota bacterium isolate cluster4 bin.111 MEP7132111.1
Pseudomonadati Acidobacteriota Thermoanaerobaculia unclassified Thermoanaerobaculia Thermoanaerobaculia bacterium isolate SMAG U4971 HEY4230619.1
Pseudomonadati Acidobacteriota unclassified Acidobacteriota Acidobacteriota bacterium isolate SMAG U9492 HET6267112.1
Pseudomonadati Acidobacteriota unclassified Acidobacteriota Acidobacteriota bacterium isolate Amazon FNV 2010 25 4 PWT89967.1
Pseudomonadati Acidobacteriota unclassified Acidobacteriota Acidobacteriota bacterium isolate SMAG U15921 HSP06034.1
Pseudomonadati Acidobacteriota unclassified Acidobacteriota Acidobacteriota bacterium isolate SMAG U7984 HEY4491934.1
Aminicenantes Candidatus Aminicenantota Candidatus Aminicenantes bacterium isolate 4 1 July SF Bin30 MDH4195800.1
Aminicenantes Candidatus Aminicenantota Candidatus Saccharicenans sp. isolate Go SlDig bin 113 MBP7870169.1
Aminicenantes Candidatus Aminicenantota Candidatus Saccharicenans sp. isolate Yes49 19 MDI6845324.1
Aminicenantes Candidatus Aminicenantota Candidatus Aminicenantes bacterium isolate ARK 02 PMP96915.1
Aminicenantes Candidatus Aminicenantota Candidatus Saccharicenans sp. isolate Kamch4336 1 MF 19 MGB9835336.1
Aminicenantes Candidatus Aminicenantota Candidatus Saccharicenans sp. isolate Yes71 14 MDI6698403.1
Aminicenantes Candidatus Aminicenantota Candidatus Aminicenantes bacterium isolate SPRUCE 605 MGA2531781.1
Aminicenantes Candidatus Aminicenantota Candidatus Aminicenantes bacterium isolate CWP4 2.bin.162 MFO7732205.1
Aminicenantes Candidatus Aminicenantota Candidatus Aminicenantes bacterium isolate SpSt 1229 HDT14085.1
Aminicenantes Candidatus Aminicenantota Candidatus Aminicenantes bacterium isolate CTSoil 003 MBP1769895.1
Aminicenantes Candidatus Aminicenantota Candidatus Aminicenantes bacterium isolate W4 Combined metabat1 094 NTV80424.1
Aminicenantes Candidatus Aminicenantota Candidatus Aminicenantes bacterium isolate AMR MDS 2498 HOW85997.1
Aminicenantes Candidatus Aminicenantota Candidatus Aminicenantes bacterium isolate CTSoil 002 MBP1660002.1
Aminicenantes Candidatus Aminicenantota Candidatus Aminicenantes bacterium isolate PFL55 MCJ7587744.1
Aminicenantes Candidatus Aminicenantota Candidatus Aminicenantes bacterium isolate Zod Metabat.1003 MBN2206501.1
Aminicenantes Candidatus Aminicenantota Candidatus Aminicenantes bacterium RBG 16 63 16 OGD18292.1
Aminicenantes Candidatus Aminicenantota Candidatus Aminicenantes bacterium RBG 19FT COMBO 58 17 OGD38460.1
Aminicenantes Candidatus Aminicenantota Candidatus Aminicenantes bacterium RBG 13 59 9 OGD24078.1
Aminicenantes Candidatus Aminicenantota Candidatus Aminicenantales bacterium isolate MFD01683 ilm asm bin15 MGB9004475.1
Aminicenantes Candidatus Aminicenantota Candidatus Aminicenantes bacterium isolate M DeepCast 400m m2 293 MBM3297580.1
Aminicenantes Candidatus Aminicenantota Candidatus Aminicenantes bacterium isolate Zod maxbin.0345 1 MBN2199997.1
Aminicenantes Candidatus Aminicenantota Candidatus Aminicenantes bacterium RBG 13 62 12 OGD12613.1
Aminicenantes Candidatus Aminicenantota Candidatus Aminicenantales bacterium isolate MFD01647 ilm asm bin15 MGB8951903.1
Aminicenantes Candidatus Aminicenantota Candidatus Aminicenantes bacterium isolate LacPavin 0920 SED5 MAG 55 12 MCX6575910.1
Aminicenantes Candidatus Aminicenantota Candidatus Aminicenantales bacterium isolate SPRUCE 56 MGA2586729.1
Aminicenantes Candidatus Aminicenantota Candidatus Aminicenantes bacterium isolate LacPavin 0920 SED5 MAG 59 17 MCX6564531.1
Aminicenantes Candidatus Aminicenantota Candidatus Aminicenantes bacterium isolate PFL34 MCJ7564282.1
Aminicenantes Candidatus Aminicenantota Candidatus Aminicenantes bacterium isolate Zod Metabat.1233 MBN1939796.1
Aminicenantes Candidatus Aminicenantota Candidatus Aminicenantes bacterium isolate K DeepCast 250m m1 044 MBM3310970.1
Aminicenantes Candidatus Aminicenantota Candidatus Aminicenantes bacterium isolate M DeepCast 400m m2 244 MBM3293135.1
Aminicenantes Candidatus Aminicenantota Candidatus Aminicenantes bacterium isolate LacPavin 0920 WC70 MAG 64 36 iso1 MCX6559833.1
Aminicenantes Candidatus Aminicenantota Candidatus Aminicenantes bacterium isolate SPRUCE 175 MGD0782290.1
Bacteria incertae sedis Candidatus Methylomirabilota Candidatus Methylomirabilota bacterium isolate NC groundwater 1099 Ag S2p5 61 69 MBI3988016.1
Bacteria incertae sedis Candidatus Methylomirabilota Candidatus Methylomirabilales bacterium isolate 21I 052.bin.36 MFN3476372.1
Bacteria incertae sedis Candidatus Methylomirabilota Candidatus Methylomirabilis limnetica WP 107562510.1
Bacteria incertae sedis Candidatus Methylomirabilota Candidatus Methylomirabilota bacterium isolate NC groundwater 706 Ag S 0.2um 72 10 MBI3002975.1
Bacteria incertae sedis Candidatus Methylomirabilota Candidatus Methylomirabilota bacterium isolate H51 bin318 1 MEK7338164.1
Bacteria incertae sedis Candidatus Methylomirabilota Candidatus Methylomirabilis sp. isolate SMAG U7550 HEY4720012.1
Bacteria incertae sedis Candidatus Methylomirabilota Candidatus Methylomirabilota bacterium isolate PMV 4.88 MCI0482988.1
Bacteria incertae sedis Candidatus Methylomirabilota Candidatus Methylomirabilota bacterium isolate SCS2bin.6 MEE9172121.1
Bacteria incertae sedis Candidatus Methylomirabilota Candidatus Methylomirabilota bacterium isolate B5MC02 MCH7896019.1
Bacteria incertae sedis Candidatus Methylomirabilota Candidatus Methylomirabilales bacterium isolate K 3 bin.12 MFQ5847447.1
Bacteria incertae sedis Candidatus Methylomirabilota Candidatus Methylomirabilales bacterium isolate K 2 bin.70 MFQ5802308.1
Bacteria incertae sedis Candidatus Methylomirabilota Candidatus Methylomirabilota bacterium isolate NC groundwater 1750 Pr3 B 0.1um 56 10 MBI5167746.1
Bacteria incertae sedis Candidatus Methylomirabilota Candidatus Methylomirabilota bacterium isolate A05DMB I 35 MDH7498936.1
Bacteria incertae sedis Candidatus Methylomirabilota Candidatus Methylomirabilota bacterium isolate NC groundwater 460 Ag B 0.1um 68 8 MBI2563492.1
Bacteria incertae sedis Candidatus Methylomirabilota Candidatus Methylomirabilis sp. isolate SMAG U16341 HSC72378.1
Bacteria incertae sedis Candidatus Methylomirabilota Candidatus Methylomirabilis sp. isolate SMAG U16397 HSB70398.1
Bacteria incertae sedis Candidatus Methylomirabilota Candidatus Methylomirabilales bacterium isolate SBS.BIN.71 MFB3816961.1
Bacteria incertae sedis Candidatus Methylomirabilota Candidatus Methylomirabilota bacterium isolate NC groundwater 1464 Ag S 0.65um 65 130 MBI4537033.1
Bacteria incertae sedis Candidatus Methylomirabilota Candidatus Methylomirabilis sp. isolate SMAG U3006 HEX7629816.1

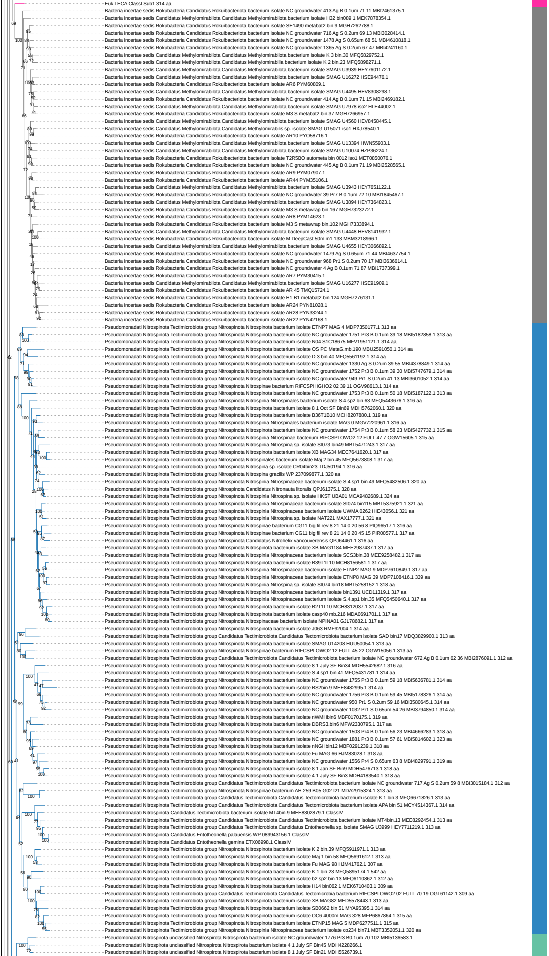

Euk LECA ClassI Sub1 314 aa
Bacteria incertae sedis Rokubacteria Candidatus Rokuibacteriota bacterium isolate NC groundwater 413 Ag B 0.1um 71 11 MBI2461375.1
Bacteria incertae sedis Candidatus Methylomirabilota Candidatus Methylomirabilota bacterium isolate H32 bin089 1 MEK7878354.1
Bacteria incertae sedis Candidatus Rokuibacteriota Candidatus Rokuibacteriota bacterium isolate SE1490 metabat2.bin.9 MGH7262788.1
Bacteria incertae sedis Rokubacteria Candidatus Rokuibacteriota bacterium isolate NC groundwater 716 Ag S 0.2um 69 13 MBI3028414.1
Bacteria incertae sedis Rokubacteria Candidatus Rokuibacteriota bacterium isolate NC groundwater 1478 Ag S 0.65um 68 51 MBI4610818.1
Bacteria incertae sedis Rokubacteria Candidatus Rokuibacteriota bacterium isolate NC groundwater 1365 Ag S 0.2um 67 47 MBI4241160.1
Bacteria incertae sedis Candidatus Methylomirabilota Candidatus Methylomirabilota bacterium isolate K 3 bin.30 MFQ5829752.1
Bacteria incertae sedis Candidatus Methylomirabilota Candidatus Methylomirabilia bacterium isolate K 2 bin.23 MFQ5898271.1
Bacteria incertae sedis Candidatus Methylomirabilota Candidatus Methylomirabilota bacterium isolate SMAG U3939 HEY7601172.1
Bacteria incertae sedis Candidatus Methylomirabilota Candidatus Methylomirabilota bacterium isolate SMAG U16272 HSE94476.1
Bacteria incertae sedis Rokubacteria Candidatus Rokuibacteriota bacterium isolate AR6 PYM60809.1
Bacteria incertae sedis Candidatus Methylomirabilota Candidatus Methylomirabilota bacterium isolate SMAG U4495 HEV8308298.1
Bacteria incertae sedis Rokubacteria Candidatus Rokuibacteriota bacterium isolate NC groundwater 414 Ag B 0.1um 71 15 MBI2469182.1
Bacteria incertae sedis Candidatus Methylomirabilota Candidatus Methylomirabilota bacterium isolate SMAG U7978 iso2 HLE44002.1
Bacteria incertae sedis Rokubacteria Candidatus Rokuibacteriota bacterium isolate M3 S metabat2.bin.37 MGH7266957.1
Bacteria incertae sedis Candidatus Methylomirabilota Candidatus Methylomirabilota bacterium isolate SMAG U4560 HEV8458445.1
Bacteria incertae sedis Candidatus Methylomirabilota Candidatus Methylomirabilis sp. isolate SMAG U15071 iso1 HXJ78540.1
Bacteria incertae sedis Rokubacteria Candidatus Rokuibacteriota bacterium isolate AR10 PYO58716.1
Bacteria incertae sedis Candidatus Methylomirabilota Candidatus Methylomirabilota bacterium isolate SMAG U13394 HWN55903.1
Bacteria incertae sedis Candidatus Methylomirabilota Candidatus Methylomirabilota bacterium isolate SMAG U10074 HZP36224.1
Bacteria incertae sedis Rokubacteria Candidatus Rokuibacteriota bacterium isolate T2R5BO autometa bin 0012 iso1 MET0850076.1
Bacteria incertae sedis Rokubacteria Candidatus Rokuibacteriota bacterium isolate NC groundwater 445 Ag B 0.1um 71 19 MBI2528565.1
Bacteria incertae sedis Rokubacteria Candidatus Rokuibacteriota bacterium isolate AR9 PYM07907.1
Bacteria incertae sedis Rokubacteria Candidatus Rokuibacteriota bacterium isolate AR44 PYM35106.1
Bacteria incertae sedis Candidatus Methylomirabilota Candidatus Methylomirabilota bacterium isolate SMAG U3943 HEY7651122.1
Bacteria incertae sedis Rokubacteria Candidatus Rokuibacteriota bacterium isolate NC groundwater 39 Pr7 B 0.1um 72 10 MBI1845467.1
Bacteria incertae sedis Candidatus Methylomirabilota Candidatus Methylomirabilota bacterium isolate SMAG U3894 HEY7364823.1
Bacteria incertae sedis Rokubacteria Candidatus Rokuibacteriota bacterium isolate M3 S metawrap bin.167 MGH7323272.1
Bacteria incertae sedis Rokubacteria Candidatus Rokuibacteriota bacterium isolate AR8 PYM14623.1
Bacteria incertae sedis Rokubacteria Candidatus Rokuibacteriota bacterium isolate M3 S metawrap bin.102 MGH7333894.1
Bacteria incertae sedis Candidatus Methylomirabilota Candidatus Methylomirabilota bacterium isolate SMAG U4448 HEV8141932.1
Bacteria incertae sedis Rokubacteria Candidatus Rokuibacteriota bacterium isolate M DeepCast 50m m1 133 MBM3218966.1
Bacteria incertae sedis Candidatus Methylomirabilota Candidatus Methylomirabilota bacterium isolate SMAG U4655 HEY3066892.1
Bacteria incertae sedis Rokubacteria Candidatus Rokuibacteriota bacterium isolate NC groundwater 1479 Ag S 0.65um 71 44 MBI4637754.1
Bacteria incertae sedis Rokubacteria Candidatus Rokuibacteriota bacterium isolate NC groundwater 968 Pr1 S 0.2um 70 17 MBI3636614.1
Bacteria incertae sedis Rokubacteria Candidatus Rokuibacteriota bacterium isolate NC groundwater 4 Ag B 0.1um 71 87 MBI1737399.1
Bacteria incertae sedis Rokubacteria Candidatus Rokuibacteriota bacterium isolate AR7 PYM30415.1
Bacteria incertae sedis Candidatus Methylomirabilota Candidatus Methylomirabilota bacterium isolate SMAG U16277 HSE91909.1
Bacteria incertae sedis Rokubacteria Candidatus Rokuibacteriota bacterium isolate AR 45 TMQ15724.1
Bacteria incertae sedis Rokubacteria Candidatus Rokuibacteriota bacterium isolate H1 B1 metabat2.bin.124 MGH7276131.1
Bacteria incertae sedis Rokubacteria Candidatus Rokuibacteriota bacterium isolate AR24 PYN81028.1
Bacteria incertae sedis Rokubacteria Candidatus Rokuibacteriota bacterium isolate AR28 PYN33244.1
Bacteria incertae sedis Rokubacteria Candidatus Rokuibacteriota bacterium isolate AR22 PYN42168.1
Pseudomonadati Nitrospinota Tectimicrobiota group Nitrospinota Nitrospinota bacterium isolate ETNP7 MAG 4 MDP7350177.1 313 aa
Pseudomonadati Nitrospinota Tectimicrobiota group Nitrospinota Nitrospinota bacterium isolate NC groundwater 1751 Pr3 B 0.1um 39 18 MBI5182858.1 313 aa
Pseudomonadati Nitrospinota Tectimicrobiota group Nitrospinota Nitrospinota bacterium isolate N04 S1C18675 MFV1951121.1 314 aa
Pseudomonadati Nitrospinota Tectimicrobiota group Nitrospinota Nitrospinota bacterium isolate OS PC MetaG.mb.190 MBU2591050.1 314 aa
Pseudomonadati Nitrospinota Tectimicrobiota group Nitrospinota Nitrospinota bacterium isolate D 3 bin.40 MFQ5561192.1 314 aa
Pseudomonadati Nitrospinota Tectimicrobiota group Nitrospinota Nitrospinota bacterium isolate NC groundwater 1330 Ag S 0.2um 39 55 MBI4378849.1 314 aa
Pseudomonadati Nitrospinota Tectimicrobiota group Nitrospinota Nitrospinota bacterium isolate NC groundwater 1752 Pr3 B 0.1um 39 30 MBI5747679.1 314 aa
Pseudomonadati Nitrospinota Tectimicrobiota group Nitrospinota Nitrospinota bacterium isolate NC groundwater 949 Pr1 S 0.2um 41 13 MBI3601052.1 314 aa
Pseudomonadati Nitrospinota Tectimicrobiota group Nitrospinota Nitrospinae bacterium RIFCSPHIGHO2 02 39 11 OGV98613.1 314 aa
Pseudomonadati Nitrospinota Tectimicrobiota group Nitrospinota Nitrospinota bacterium isolate NC groundwater 1753 Pr3 B 0.1um 50 18 MBI5187122.1 313 aa
Pseudomonadati Nitrospinota Tectimicrobiota group Nitrospinota Nitrospinia Nitrospinales bacterium isolate S.4.sp2 bin.63 MFQ5443676.1 316 aa
Pseudomonadati Nitrospinota Tectimicrobiota group Nitrospinota Nitrospinota bacterium isolate 8 1 Oct SF Bin69 MDH5762060.1 320 aa
Pseudomonadati Nitrospinota Tectimicrobiota group Nitrospinota Nitrospinota bacterium isolate B36T1B10 MCH8207880.1 319 aa
Pseudomonadati Nitrospinota Tectimicrobiota group Nitrospinota Nitrospinia Nitrospinales bacterium isolate MAG 0 MGV7220961.1 316 aa
Pseudomonadati Nitrospinota Tectimicrobiota group Nitrospinota Nitrospinota bacterium isolate NC groundwater 1754 Pr3 B 0.1um 58 23 MBI5427732.1 315 aa
Pseudomonadati Nitrospinota Tectimicrobiota group Nitrospinota Nitrospinia Nitrospinae bacterium RIFCSPLOWO2 12 FULL 47 7 OGW15605.1 315 aa
Pseudomonadati Nitrospinota Tectimicrobiota group Nitrospinota Nitrospinia Nitrospina sp. isolate SI073 bin49 MBT5471243.1 317 aa
Pseudomonadati Nitrospinota Tectimicrobiota group Nitrospinota Nitrospinota bacterium isolate XB MAG34 MEC7641620.1 317 aa
Pseudomonadati Nitrospinota Tectimicrobiota group Nitrospinota Nitrospinales bacterium isolate Maj 2 bin.45 MFQ5673808.1 317 aa
Pseudomonadati Nitrospinota Tectimicrobiota group Nitrospinota Nitrospina sp. isolate CR04bin23 TDJ50194.1 316 aa
Pseudomonadati Nitrospinota Tectimicrobiota group Nitrospinota Nitrospina gracilis WP 237099877.1 320 aa
Pseudomonadati Nitrospinota Tectimicrobiota group Nitrospinota Nitrospinia Nitrospinaceae bacterium isolate S.4.sp1 bin.49 MFQ5482506.1 320 aa
Pseudomonadati Nitrospinota Tectimicrobiota group Nitrospinota Candidatus Nitronauta litoralis QPJ61375.1 328 aa
Pseudomonadati Nitrospinota Tectimicrobiota group Nitrospinota Nitrospinia Nitrospina sp. isolate HKST UBA01 MCA9482689.1 324 aa
Pseudomonadati Nitrospinota Tectimicrobiota group Nitrospinota Nitrospinia Nitrospinaceae bacterium isolate SI074 bin115 MBT5375921.1 321 aa
Pseudomonadati Nitrospinota Tectimicrobiota group Nitrospinota Nitrospinia Nitrospinaceae bacterium isolate UWMA 0262 HIE43056.1 321 aa
Pseudomonadati Nitrospinota Tectimicrobiota group Nitrospinota Nitrospinia Nitrospina sp. isolate NAT221 MAX17777.1 321 aa
Pseudomonadati Nitrospinota Tectimicrobiota group Nitrospinota Nitrospinae bacterium CG11 big fil rev 8 21 14 0 20 56 8 PIQ96517.1 316 aa
Pseudomonadati Nitrospinota Tectimicrobiota group Nitrospinota Nitrospinae bacterium CG11 big fil rev 8 21 14 0 20 45 15 PIR00577.1 317 aa
Pseudomonadati Nitrospinota Tectimicrobiota group Nitrospinota Candidatus Nitrohelix vancouverensis QPJ64461.1 316 aa
Pseudomonadati Nitrospinota Tectimicrobiota group Nitrospinota Nitrospinota bacterium isolate XB MAG1184 MEE2987437.1 317 aa
Pseudomonadati Nitrospinota Tectimicrobiota group Nitrospinota Nitrospinia Nitrospinaceae bacterium isolate SCS3bin.38 MEE9258482.1 317 aa
Pseudomonadati Nitrospinota Tectimicrobiota group Nitrospinota Nitrospinota bacterium isolate B39T1L10 MCH8156581.1 317 aa
Pseudomonadati Nitrospinota Tectimicrobiota group Nitrospinota Nitrospinia Nitrospinaceae bacterium isolate ETNP2 MAG 9 MDP7610849.1 317 aa
Pseudomonadati Nitrospinota Tectimicrobiota group Nitrospinota Nitrospinia Nitrospinaceae bacterium isolate ETNP8 MAG 39 MDP7108416.1 339 aa
Pseudomonadati Nitrospinota Tectimicrobiota group Nitrospinota Nitrospinia Nitrospina sp. isolate SI074 bin18 MBT5258152.1 318 aa
Pseudomonadati Nitrospinota Tectimicrobiota group Nitrospinota Nitrospinia Nitrospinaceae bacterium isolate bin1391 UCD11319.1 317 aa
Pseudomonadati Nitrospinota Tectimicrobiota group Nitrospinota Nitrospinia Nitrospinaceae bacterium isolate S.4.sp1 bin.35 MFQ5450640.1 317 aa
Pseudomonadati Nitrospinota Tectimicrobiota group Nitrospinota Nitrospinota bacterium isolate B2T1L10 MCH8312037.1 317 aa
Pseudomonadati Nitrospinota Tectimicrobiota group Nitrospinota Nitrospinota bacterium isolate casp40 mb.216 MDA0691701.1 317 aa
Pseudomonadati Nitrospinota Tectimicrobiota group Nitrospinota Nitrospinia Nitrospinaceae bacterium isolate NPINA01 GJL78682.1 317 aa
Pseudomonadati Nitrospinota Tectimicrobiota group Nitrospinota Nitrospinota bacterium isolate J063 RMF92004.1 314 aa
Pseudomonadati Nitrospinota Tectimicrobiota group Candidatus Tectimicrobiota Candidatus Tectomicrobiota bacterium isolate SAD bin17 MDQ3829900.1 313 aa
Pseudomonadati Nitrospinota Tectimicrobiota group Nitrospinota Nitrospinota bacterium isolate SMAG U14208 HUU50054.1 313 aa
Pseudomonadati Nitrospinota Tectimicrobiota group Nitrospinota Nitrospinae bacterium RIFCSPLOWO2 12 FULL 45 22 OGW15056.1 313 aa
Pseudomonadati Nitrospinota Tectimicrobiota group Candidatus Tectimicrobiota Candidatus Tectimicrobiota bacterium isolate NC groundwater 672 Ag B 0.1um 62 36 MBI2876091.1 312 aa
Pseudomonadati Nitrospinota Tectimicrobiota group Nitrospinota Nitrospinota bacterium isolate 8 1 July SF Bin34 MDH5542682.1 316 aa
Pseudomonadati Nitrospinota Tectimicrobiota group Nitrospinota Nitrospinota bacterium isolate S.4.sp1 bin.41 MFQ5431781.1 314 aa
Pseudomonadati Nitrospinota Tectimicrobiota group Nitrospinota Nitrospinota bacterium isolate NC groundwater 1755 Pr3 B 0.1um 59 18 MBI5636781.1 314 aa
Pseudomonadati Nitrospinota Tectimicrobiota group Nitrospinota Nitrospinota bacterium isolate BS2bin.9 MEE8482995.1 314 aa
Pseudomonadati Nitrospinota Tectimicrobiota group Nitrospinota Nitrospinota bacterium isolate NC groundwater 1756 Pr3 B 0.1um 59 45 MBI5178326.1 314 aa
Pseudomonadati Nitrospinota Tectimicrobiota group Nitrospinota Nitrospinota bacterium isolate NC groundwater 950 Pr1 S 0.2um 59 16 MBI3580645.1 314 aa
Pseudomonadati Nitrospinota Tectimicrobiota group Nitrospinota Nitrospinota bacterium isolate NC groundwater 1032 Pr1 S 0.65um 54 26 MBI3794850.1 314 aa
Pseudomonadati Nitrospinota Tectimicrobiota group Nitrospinota Nitrospinota bacterium isolate nWMHbin6 MBF0170175.1 319 aa
Pseudomonadati Nitrospinota Tectimicrobiota group Nitrospinota Nitrospinota bacterium isolate DBRS3.bin6 MFW2330795.1 317 aa
Pseudomonadati Nitrospinota Tectimicrobiota group Nitrospinota Nitrospinota bacterium isolate NC groundwater 1503 Pr4 B 0.1um 56 23 MBI4666283.1 318 aa
Pseudomonadati Nitrospinota Tectimicrobiota group Nitrospinota Nitrospinota bacterium isolate NC groundwater 1881 Pr3 B 0.1um 57 61 MBI5814602.1 323 aa
Pseudomonadati Nitrospinota Tectimicrobiota group Nitrospinota Nitrospinota bacterium isolate nNGHbin12 MBF0291239.1 318 aa
Pseudomonadati Nitrospinota Tectimicrobiota group Nitrospinota Nitrospinota bacterium isolate Fu MAG 66 HJM83028.1 318 aa
Pseudomonadati Nitrospinota Tectimicrobiota group Nitrospinota Nitrospinota bacterium isolate NC groundwater 1556 Pr4 S 0.65um 63 8 MBI4829791.1 319 aa
Pseudomonadati Nitrospinota Tectimicrobiota group Nitrospinota Nitrospinota bacterium isolate 8 1 Jan SF Bin9 MDH5476713.1 318 aa
Pseudomonadati Nitrospinota Tectimicrobiota group Nitrospinota Nitrospinota bacterium isolate 4 1 July SF Bin3 MDH4183540.1 318 aa
Pseudomonadati Nitrospinota Tectimicrobiota group Candidatus Tectimicrobiota Candidatus Tectimicrobiota bacterium isolate NC groundwater 717 Ag S 0.2um 59 8 MBI3015184.1 312 aa
Pseudomonadati Nitrospinota Tectimicrobiota group Nitrospinota Nitrospinae bacterium AH 259 B05 G02 I21 MDA2915324.1 313 aa
Pseudomonadati Nitrospinota Tectimicrobiota group Candidatus Tectimicrobiota Candidatus Tectomicrobiota bacterium isolate K 1 bin.3 MFQ6671826.1 313 aa
Pseudomonadati Nitrospinota Tectimicrobiota group Candidatus Tectimicrobiota Candidatus Tectomicrobiota bacterium isolate APA bin 51 MCY4514367.1 314 aa
Pseudomonadati Nitrospinota Candidatus Tectimicrobiota bacterium isolate MT4bin.9 MEE8302879.1 ClassIV
Pseudomonadati Nitrospinota Tectimicrobiota group Candidatus Tectimicrobiota Candidatus Tectimicrobiota bacterium isolate MT4bin.13 MEE8292454.1 313 aa
Pseudomonadati Nitrospinota Tectimicrobiota group Candidatus Tectimicrobiota Candidatus Entotheonella sp. isolate SMAG U3999 HEY7711219.1 313 aa
Pseudomonadati Nitrospinota Candidatus Entotheonella palauensis WP 089943156.1 ClassIV
Pseudomonadati Nitrospinota Candidatus Entotheonella gemina ETX06998.1 ClassIV
Pseudomonadati Nitrospinota Tectimicrobiota group Nitrospinota Nitrospinota bacterium isolate K 2 bin.39 MFQ5911971.1 313 aa
Pseudomonadati Nitrospinota Tectimicrobiota group Nitrospinota Nitrospinota bacterium isolate Maj 1 bin.58 MFQ5691612.1 313 aa
Pseudomonadati Nitrospinota Tectimicrobiota group Nitrospinota Nitrospinota bacterium isolate Fu MAG 98 HJM41762.1 307 aa
Pseudomonadati Nitrospinota Tectimicrobiota group Nitrospinota Nitrospinota bacterium isolate K 1 bin.23 MFQ5895174.1 542 aa
Pseudomonadati Nitrospinota Tectimicrobiota group Nitrospinota Nitrospinota bacterium isolate b2.sp2 bin.13 MFQ6110862.1 312 aa
Pseudomonadati Nitrospinota Tectimicrobiota group Nitrospinota Nitrospinota bacterium isolate H14 bin062 1 MEK6710403.1 309 aa
Pseudomonadati Nitrospinota Tectimicrobiota group Candidatus Tectimicrobiota Candidatus Tectomicrobia bacterium RIFCSPLOWO2 02 FULL 70 19 OGL61142.1 309 aa
Pseudomonadati Nitrospinota Tectimicrobiota group Nitrospinota Nitrospinota bacterium isolate XB MAG82 MED5578443.1 313 aa
Pseudomonadati Nitrospinota Tectimicrobiota group Nitrospinota Nitrospinota bacterium isolate SB0662 bin 51 MYA95395.1 314 aa
Pseudomonadati Nitrospinota Tectimicrobiota group Nitrospinota Nitrospinota bacterium isolate OC6 4000m MAG 328 MFP6867864.1 315 aa
Pseudomonadati Nitrospinota Tectimicrobiota group Nitrospinota Nitrospinota bacterium isolate ETNP15 MAG 5 MDP6277511.1 315 aa
Pseudomonadati Nitrospinota Tectimicrobiota group Nitrospinota Nitrospinia Nitrospinaceae bacterium isolate co234 bin71 MBT3352051.1 320 aa
Pseudomonadati Nitrospirota unclassified Nitrospirota Nitrospirota bacterium isolate NC groundwater 1776 Pr3 B0.1um 70 102 MBI5136583.1
Pseudomonadati Nitrospirota unclassified Nitrospirota Nitrospirota bacterium isolate 4 1 July SF Bin45 MDH4228266.1
Pseudomonadati Nitrospirota unclassified Nitrospirota Nitrospirota bacterium isolate 8 1 July SF Bin21 MDH5526739.1

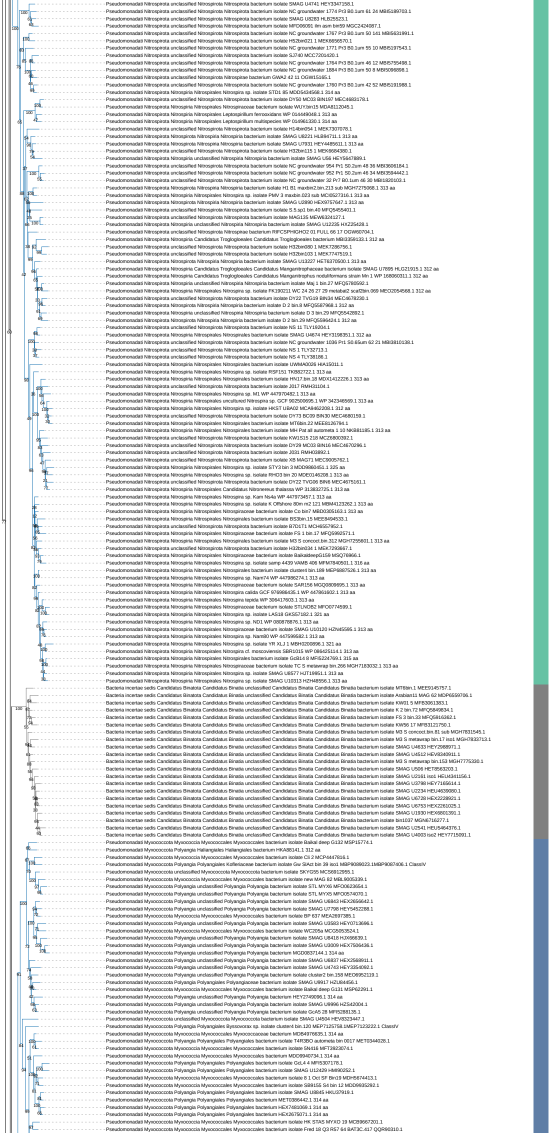
Pseudomonadati Nitrospirota unclassified Nitrospirota Nitrospirota bacterium isolate SMAG U4741 HEY3347158.1
Pseudomonadati Nitrospirota unclassified Nitrospirota Nitrospirota bacterium isolate NC groundwater 1774 Pr3 B0.1um 61 24 MBI5189703.1
Pseudomonadati Nitrospirota unclassified Nitrospirota Nitrospirota bacterium isolate SMAG U8283 HLB25523.1
Pseudomonadati Nitrospirota unclassified Nitrospirota Nitrospirota bacterium isolate MFD06091 ilm asm bin59 MGC2424087.1
Pseudomonadati Nitrospirota unclassified Nitrospirota Nitrospirota bacterium isolate NC groundwater 1767 Pr3 B0.1um 50 141 MBI5631991.1
Pseudomonadati Nitrospirota unclassified Nitrospirota Nitrospirota bacterium isolate H52bin021 1 MEK6656570.1
Pseudomonadati Nitrospirota unclassified Nitrospirota Nitrospirota bacterium isolate NC groundwater 1771 Pr3 B0.1um 55 10 MBI5197543.1
Pseudomonadati Nitrospirota unclassified Nitrospirota Nitrospirota bacterium isolate SJ740 MCC7201420.1
Pseudomonadati Nitrospirota unclassified Nitrospirota Nitrospirota bacterium isolate NC groundwater 1764 Pr3 B0.1um 46 12 MBI5755498.1
Pseudomonadati Nitrospirota unclassified Nitrospirota Nitrospirota bacterium isolate NC groundwater 1884 Pr3 B0.1um 50 8 MBI5096898.1
Pseudomonadati Nitrospirota unclassified Nitrospirota Nitrospirae bacterium GWA2 42 11 OGW15165.1
Pseudomonadati Nitrospirota unclassified Nitrospirota Nitrospirota bacterium isolate NC groundwater 1760 Pr3 B0.1um 42 52 MBI5191988.1
Pseudomonadati Nitrospirota Nitrospiria Nitrospirales Nitrospira sp. isolate STD1 85 MDD5434568.1 314 aa
Pseudomonadati Nitrospirota unclassified Nitrospirota Nitrospirota bacterium isolate DY50 MC03 BIN197 MEC4683178.1
Pseudomonadati Nitrospirota Nitrospiria Nitrospirales Nitrospiraceae bacterium isolate WUY.bin15 MDA8112045.1
Pseudomonadati Nitrospirota Nitrospiria Nitrospirales Leptospirillum ferrooxidans WP 014449048.1 313 aa
Pseudomonadati Nitrospirota Nitrospiria Nitrospirales Leptospirillum multispecies WP 014961330.1 314 aa
Pseudomonadati Nitrospirota unclassified Nitrospirota Nitrospirota bacterium isolate H14bin054 1 MEK7307078.1
Pseudomonadati Nitrospirota Nitrospirota Nitrospiria Nitrospiria bacterium isolate SMAG U8221 HLB94711.1 313 aa
Pseudomonadati Nitrospirota Nitrospirota Nitrospiria Nitrospiria bacterium isolate SMAG U7931 HEY4485611.1 313 aa
Pseudomonadati Nitrospirota unclassified Nitrospirota Nitrospirota bacterium isolate H32bin115 1 MEK6684380.1
Pseudomonadati Nitrospirota Nitrospiria unclassified Nitrospiria Nitrospiria bacterium isolate SMAG U56 HEY5647889.1
Pseudomonadati Nitrospirota unclassified Nitrospirota Nitrospirota bacterium isolate NC groundwater 954 Pr1 S0.2um 48 36 MBI3606184.1
Pseudomonadati Nitrospirota unclassified Nitrospirota Nitrospirota bacterium isolate NC groundwater 952 Pr1 S0.2um 46 34 MBI3594442.1
Pseudomonadati Nitrospirota unclassified Nitrospirota Nitrospirota bacterium isolate NC groundwater 32 Pr7 B0.1um 46 30 MBI1820103.1
Pseudomonadati Nitrospirota Nitrospirota Nitrospiria Nitrospiria bacterium isolate H1 B1 maxbin2.bin.213 sub MGH7275068.1 313 aa
Pseudomonadati Nitrospirota Nitrospiria Nitrospirales Nitrospira sp. isolate PMV 3 maxbin.023 sub MCI0527316.1 313 aa
Pseudomonadati Nitrospirota Nitrospirota Nitrospiria Nitrospiria bacterium isolate SMAG U2890 HEX9757647.1 313 aa
Pseudomonadati Nitrospirota unclassified Nitrospirota Nitrospirota bacterium isolate S.5.sp1 bin.40 MFQ5455401.1
Pseudomonadati Nitrospirota unclassified Nitrospirota Nitrospirota bacterium isolate MAG135 MEW6324127.1
Pseudomonadati Nitrospirota Nitrospiria unclassified Nitrospiria Nitrospiria bacterium isolate SMAG U12235 HXZ25428.1
Pseudomonadati Nitrospirota unclassified Nitrospirota Nitrospirae bacterium RIFCSPHIGHO2 01 FULL 66 17 OGW60704.1
Pseudomonadati Nitrospirota Nitrospiria Candidatus Troglogloeales Candidatus Troglogloeales bacterium MBI3359133.1 312 aa
Pseudomonadati Nitrospirota unclassified Nitrospirota Nitrospirota bacterium isolate H32bin080 1 MEK7286756.1
Pseudomonadati Nitrospirota unclassified Nitrospirota Nitrospirota bacterium isolate H32bin103 1 MEK7747519.1
Pseudomonadati Nitrospirota Nitrospirota Nitrospiria Nitrospiria bacterium isolate SMAG U13227 HET6370500.1 313 aa
Pseudomonadati Nitrospirota Nitrospiria Candidatus Troglogloeales Candidatus Manganitrophaceae bacterium isolate SMAG U7895 HLG21915.1 312 aa
Pseudomonadati Nitrospirota Nitrospiria Candidatus Troglogloeales Candidatus Manganitrophus noduliformans strain Mn 1 WP 168060311.1 312 aa
Pseudomonadati Nitrospirota Nitrospiria unclassified Nitrospiria Nitrospiria bacterium isolate Maj 1 bin.27 MFQ5780592.1
Pseudomonadati Nitrospirota Nitrospiria Nitrospirales Nitrospira sp. isolate FK190211 WC 24 26 27 29 metabat2 scaf2bin.069 MEO2054568.1 312 aa
Pseudomonadati Nitrospirota unclassified Nitrospirota Nitrospirota bacterium isolate DY22 TVG19 BIN34 MEC4678230.1
Pseudomonadati Nitrospirota Nitrospirota Nitrospiria Nitrospiria bacterium isolate D 2 bin.8 MFQ5587968.1 312 aa
Pseudomonadati Nitrospirota Nitrospiria unclassified Nitrospiria Nitrospiria bacterium isolate D 3 bin.29 MFQ5542892.1
Pseudomonadati Nitrospirota Nitrospirota Nitrospiria Nitrospiria bacterium isolate D 2 bin.29 MFQ5596424.1 312 aa
Pseudomonadati Nitrospirota unclassified Nitrospirota Nitrospirota bacterium isolate NS 11 TLY19204.1
Pseudomonadati Nitrospirota Nitrospiria Nitrospirales Nitrospirales bacterium isolate SMAG U4674 HEY3198351.1 312 aa
Pseudomonadati Nitrospirota unclassified Nitrospirota Nitrospirota bacterium isolate NC groundwater 1036 Pr1 S0.65um 62 21 MBI3810138.1
Pseudomonadati Nitrospirota unclassified Nitrospirota Nitrospirota bacterium isolate NS 1 TLY32713.1
Pseudomonadati Nitrospirota unclassified Nitrospirota Nitrospirota bacterium isolate NS 4 TLY38186.1
Pseudomonadati Nitrospirota Nitrospiria Nitrospirales Nitrospirales bacterium isolate UWMA0026 HIA15011.1
Pseudomonadati Nitrospirota Nitrospiria Nitrospirales Nitrospira sp. isolate RSF151 TKB82722.1 313 aa
Pseudomonadati Nitrospirota Nitrospiria Nitrospirales Nitrospirales bacterium isolate HN17.bin.18 MDX1412226.1 313 aa
Pseudomonadati Nitrospirota unclassified Nitrospirota Nitrospirota bacterium isolate J017 RMH31104.1
Pseudomonadati Nitrospirota Nitrospiria Nitrospirales Nitrospira sp. M1 WP 447970482.1 313 aa
Pseudomonadati Nitrospirota Nitrospiria Nitrospirales uncultured Nitrospira sp. GCF 902500695.1 WP 342346569.1 313 aa
Pseudomonadati Nitrospirota Nitrospiria Nitrospirales Nitrospira sp. isolate HKST UBA02 MCA9462208.1 312 aa
Pseudomonadati Nitrospirota unclassified Nitrospirota Nitrospirota bacterium isolate DY73 BC09 BIN30 MEC4680159.1
Pseudomonadati Nitrospirota Nitrospiria Nitrospirales Nitrospirales bacterium isolate MT6bin.22 MEE8126794.1
Pseudomonadati Nitrospirota Nitrospiria Nitrospirales Nitrospirales bacterium isolate MH Pat all autometa 1 10 NKB81185.1 313 aa
Pseudomonadati Nitrospirota unclassified Nitrospirota Nitrospirota bacterium isolate KW1S15 218 MCZ6800392.1
Pseudomonadati Nitrospirota unclassified Nitrospirota Nitrospirota bacterium isolate DY29 MC03 BIN16 MEC4670296.1
Pseudomonadati Nitrospirota unclassified Nitrospirota Nitrospirota bacterium isolate J031 RMH03892.1
Pseudomonadati Nitrospirota unclassified Nitrospirota Nitrospirota bacterium isolate XB MAG71 MEC9005762.1
Pseudomonadati Nitrospirota Nitrospiria Nitrospirales Nitrospira sp. isolate STY3 bin 3 MDD9860451.1 325 aa
Pseudomonadati Nitrospirota Nitrospiria Nitrospirales Nitrospira sp. isolate RHO3 bin 20 MDE0146208.1 313 aa
Pseudomonadati Nitrospirota unclassified Nitrospirota Nitrospirota bacterium isolate DY22 TVG06 BIN6 MEC4675161.1
Pseudomonadati Nitrospirota Nitrospiria Nitrospirales Candidatus Nitronereus thalassa WP 313832725.1 313 aa
Pseudomonadati Nitrospirota Nitrospiria Nitrospirales Nitrospira sp. Kam Ns4a WP 447973457.1 313 aa
Pseudomonadati Nitrospirota Nitrospiria Nitrospirales Nitrospira sp. isolate K Offshore 80m m2 121 MBM4123262.1 313 aa
Pseudomonadati Nitrospirota Nitrospiria Nitrospirales Nitrospiraceae bacterium isolate Co bin7 MBD0305163.1 313 aa
Pseudomonadati Nitrospirota Nitrospiria Nitrospirales Nitrospirales bacterium isolate BS3bin.15 MEE8494533.1
Pseudomonadati Nitrospirota unclassified Nitrospirota Nitrospirota bacterium isolate B7D1T1 MCH6557952.1
Pseudomonadati Nitrospirota Nitrospiria Nitrospirales Nitrospiraceae bacterium isolate FS 1 bin.17 MFQ5992571.1
Pseudomonadati Nitrospirota Nitrospiria Nitrospirales Nitrospirales bacterium isolate M3 S concoct.bin.312 MGH7255601.1 313 aa
Pseudomonadati Nitrospirota unclassified Nitrospirota Nitrospirota bacterium isolate H32bin034 1 MEK7293667.1
Pseudomonadati Nitrospirota Nitrospiria Nitrospirales Nitrospiraceae bacterium isolate BaikaldeepG159 MSQ76966.1
Pseudomonadati Nitrospirota Nitrospiria Nitrospirales Nitrospira sp. isolate samp 4439 VAMB 406 MFM7840501.1 316 aa
Pseudomonadati Nitrospirota Nitrospiria Nitrospirales Nitrospirales bacterium isolate cluster4 bin.189 MEP6887526.1 313 aa
Pseudomonadati Nitrospirota Nitrospiria Nitrospirales Nitrospira sp. Nam74 WP 447986274.1 313 aa
Pseudomonadati Nitrospirota Nitrospiria Nitrospirales Nitrospiraceae bacterium isolate SAR156 MGQ0809695.1 313 aa
Pseudomonadati Nitrospirota Nitrospiria Nitrospirales Nitrospira calida GCF 976986435.1 WP 447861602.1 313 aa
Pseudomonadati Nitrospirota Nitrospiria Nitrospirales Nitrospira tepida WP 306417603.1 313 aa
Pseudomonadati Nitrospirota Nitrospiria Nitrospirales Nitrospiraceae bacterium isolate STLNOB2 MFO0774599.1
Pseudomonadati Nitrospirota Nitrospiria Nitrospirales Nitrospira sp. isolate LAS18 GKS57182.1 321 aa
Pseudomonadati Nitrospirota Nitrospiria Nitrospirales Nitrospira sp. ND1 WP 080878876.1 313 aa
Pseudomonadati Nitrospirota Nitrospiria Nitrospirales Nitrospiraceae bacterium isolate SMAG U10120 HZN45595.1 313 aa
Pseudomonadati Nitrospirota Nitrospiria Nitrospirales Nitrospira sp. Nam80 WP 447599582.1 313 aa
Pseudomonadati Nitrospirota Nitrospiria Nitrospirales Nitrospira sp. isolate YR XLJ 1 MBH0200896.1 321 aa
Pseudomonadati Nitrospirota Nitrospiria Nitrospirales Nitrospira cf. moscoviensis SBR1015 WP 086425114.1 313 aa
Pseudomonadati Nitrospirota Nitrospiria Nitrospirales Nitrospirales bacterium isolate GcB14 8 MFI5224769.1 315 aa
Pseudomonadati Nitrospirota Nitrospiria Nitrospirales Nitrospiraceae bacterium isolate TC S metawrap bin.266 MGH7183032.1 313 aa
Pseudomonadati Nitrospirota Nitrospiria Nitrospirales Nitrospira sp. isolate SMAG U8577 HJT19951.1 313 aa
Pseudomonadati Nitrospirota Nitrospiria Nitrospirales Nitrospira sp. isolate SMAG U10313 HZH48556.1 313 aa
Bacteria incertae sedis Candidatus Binatota Candidatus Binatia unclassified Candidatus Binatia Candidatus Binatia bacterium isolate MT6bin.1 MEE9145757.1
Bacteria incertae sedis Candidatus Binatota Candidatus Binatia unclassified Candidatus Binatia Candidatus Binatia bacterium isolate Arabian11 MAG 62 MDP6559706.1
Bacteria incertae sedis Candidatus Binatota Candidatus Binatia unclassified Candidatus Binatia Candidatus Binatia bacterium isolate KW01 5 MFB3061383.1
Bacteria incertae sedis Candidatus Binatota Candidatus Binatia unclassified Candidatus Binatia Candidatus Binatia bacterium isolate K 2 bin.72 MFQ5849834.1
Bacteria incertae sedis Candidatus Binatota Candidatus Binatia unclassified Candidatus Binatia Candidatus Binatia bacterium isolate FS 3 bin.33 MFQ5916362.1
Bacteria incertae sedis Candidatus Binatota Candidatus Binatia unclassified Candidatus Binatia Candidatus Binatia bacterium isolate KW56 17 MFB3121750.1
Bacteria incertae sedis Candidatus Binatota Candidatus Binatia unclassified Candidatus Binatia Candidatus Binatia bacterium isolate M3 S concoct.bin.81 sub MGH7831545.1
Bacteria incertae sedis Candidatus Binatota Candidatus Binatia unclassified Candidatus Binatia Candidatus Binatia bacterium isolate M3 S metawrap bin.17 iso1 MGH7833713.1
Bacteria incertae sedis Candidatus Binatota Candidatus Binatia unclassified Candidatus Binatia Candidatus Binatia bacterium isolate SMAG U4633 HEY2988971.1
Bacteria incertae sedis Candidatus Binatota Candidatus Binatia unclassified Candidatus Binatia Candidatus Binatia bacterium isolate SMAG U4512 HEV8340911.1
Bacteria incertae sedis Candidatus Binatota Candidatus Binatia unclassified Candidatus Binatia Candidatus Binatia bacterium isolate M3 S metawrap bin.153 MGH7775330.1
Bacteria incertae sedis Candidatus Binatota Candidatus Binatia unclassified Candidatus Binatia Candidatus Binatia bacterium isolate SMAG U506 HET8563203.1
Bacteria incertae sedis Candidatus Binatota Candidatus Binatia unclassified Candidatus Binatia Candidatus Binatia bacterium isolate SMAG U2161 iso1 HEU4341156.1
Bacteria incertae sedis Candidatus Binatota Candidatus Binatia unclassified Candidatus Binatia Candidatus Binatia bacterium isolate SMAG U3798 HEY7165614.1
Bacteria incertae sedis Candidatus Binatota Candidatus Binatia unclassified Candidatus Binatia Candidatus Binatia bacterium isolate SMAG U2234 HEU4639080.1
Bacteria incertae sedis Candidatus Binatota Candidatus Binatia unclassified Candidatus Binatia Candidatus Binatia bacterium isolate SMAG U6728 HEX2228921.1
Bacteria incertae sedis Candidatus Binatota Candidatus Binatia unclassified Candidatus Binatia Candidatus Binatia bacterium isolate SMAG U6753 HEX2261025.1
Bacteria incertae sedis Candidatus Binatota Candidatus Binatia unclassified Candidatus Binatia Candidatus Binatia bacterium isolate SMAG U1930 HEX6801391.1
Bacteria incertae sedis Candidatus Binatota Candidatus Binatia unclassified Candidatus Binatia Candidatus Binatia bacterium isolate bin1037 MGN6716277.1
Bacteria incertae sedis Candidatus Binatota Candidatus Binatia unclassified Candidatus Binatia Candidatus Binatia bacterium isolate SMAG U2541 HEU5464376.1
Bacteria incertae sedis Candidatus Binatota Candidatus Binatia unclassified Candidatus Binatia Candidatus Binatia bacterium isolate SMAG U4003 iso2 HEY7715091.1
Pseudomonadati Myxococcota Myxococcia Myxococcales Myxococcales bacterium isolate Baikal deep G132 MSP15774.1
Pseudomonadati Myxococcota Polyangia Haliangiales Haliangiales bacterium HKA88141.1 312 aa
Pseudomonadati Myxococcota Myxococcia Myxococcales Myxococcales bacterium isolate Cli 2 MCP4447816.1
Pseudomonadati Myxococcota Polyangia Polyangiales Kofleriaceae bacterium isolate Gw SlAct bin 39 iso1 MBP9089023.1MBP9087406.1 ClassIV
Pseudomonadati Myxococcota unclassified Myxococcota Myxococcota bacterium isolate SKYG55 MCS6912955.1
Pseudomonadati Myxococcota Myxococcia Myxococcales Myxococcales bacterium isolate new MAG 82 MBL9005339.1
Pseudomonadati Myxococcota Polyangia unclassified Polyangia Polyangia bacterium isolate STL MYX6 MFO0623654.1
Pseudomonadati Myxococcota Polyangia unclassified Polyangia Polyangia bacterium isolate STL MYX5 MFO0574070.1
Pseudomonadati Myxococcota Polyangia unclassified Polyangia Polyangia bacterium isolate SMAG U6843 HEX2656642.1
Pseudomonadati Myxococcota Polyangia unclassified Polyangia Polyangia bacterium isolate SMAG U7798 HEY5452288.1
Pseudomonadati Myxococcota Myxococcia Myxococcales Myxococcales bacterium isolate BP 637 MEA2697385.1
Pseudomonadati Myxococcota Polyangia unclassified Polyangia Polyangia bacterium isolate SMAG U3583 HEY0713696.1
Pseudomonadati Myxococcota Myxococcia Myxococcales Myxococcales bacterium isolate WC205a MCG5053524.1
Pseudomonadati Myxococcota Polyangia unclassified Polyangia Polyangia bacterium isolate SMAG U8418 HJX66639.1
Pseudomonadati Myxococcota Polyangia unclassified Polyangia Polyangia bacterium isolate SMAG U3009 HEX7506436.1
Pseudomonadati Myxococcota Polyangia unclassified Polyangia Polyangia bacterium MGD0837144.1 314 aa
Pseudomonadati Myxococcota Polyangia unclassified Polyangia Polyangia bacterium isolate SMAG U6837 HEX2568911.1
Pseudomonadati Myxococcota Polyangia unclassified Polyangia Polyangia bacterium isolate SMAG U4743 HEY3354092.1
Pseudomonadati Myxococcota Polyangia unclassified Polyangia Polyangia bacterium isolate cluster2 bin.158 MEO6952119.1
Pseudomonadati Myxococcota Polyangia Polyangiales Polyangiaceae bacterium isolate SMAG U9917 HZU84456.1
Pseudomonadati Myxococcota Myxococcia Myxococcales Myxococcales bacterium isolate Baikal deep G131 MSP62291.1
Pseudomonadati Myxococcota Polyangia unclassified Polyangia Polyangia bacterium HEY2749096.1 314 aa
Pseudomonadati Myxococcota Polyangia unclassified Polyangia Polyangia bacterium isolate SMAG U9996 HZS42004.1
Pseudomonadati Myxococcota Polyangia unclassified Polyangia Polyangia bacterium isolate GcA5 28 MFI5288135.1
Pseudomonadati Myxococcota unclassified Myxococcota Myxococcota bacterium isolate SMAG U4504 HEV8323447.1
Pseudomonadati Myxococcota Polyangia Polyangiales Byssovorax sp. isolate cluster4 bin.120 MEP7125758.1MEP7123222.1 ClassIV
Pseudomonadati Myxococcota Myxococcia Myxococcales Myxococcaceae bacterium MDB4976635.1 314 aa
Pseudomonadati Myxococcota Polyangia Polyangiales Polyangiales bacterium isolate T4R3BO autometa bin 0017 MET0344028.1
Pseudomonadati Myxococcota Myxococcia Myxococcales Myxococcales bacterium isolate 5N416 MFT3923074.1
Pseudomonadati Myxococcota Myxococcia Myxococcales Myxococcales bacterium MDD9940734.1 314 aa
Pseudomonadati Myxococcota Polyangia Polyangiales Polyangiales bacterium isolate GcL4 4 MFI5307178.1
Pseudomonadati Myxococcota Polyangia Polyangiales Polyangiales bacterium isolate SMAG U12429 HMI90252.1
Pseudomonadati Myxococcota Myxococcia Myxococcales Myxococcales bacterium isolate 8 1 Oct SF Bin19 MDH5674413.1
Pseudomonadati Myxococcota Myxococcia Myxococcales Myxococcales bacterium isolate SB9155 S4 bin 12 MDD9935292.1
Pseudomonadati Myxococcota Polyangia Polyangiales Polyangiales bacterium isolate SMAG U8845 HKU37919.1
Pseudomonadati Myxococcota Polyangia Polyangiales Polyangiales bacterium MET0386442.1 314 aa
Pseudomonadati Myxococcota Polyangia Polyangiales Polyangiales bacterium HEX7481069.1 314 aa
Pseudomonadati Myxococcota Polyangia Polyangiales Polyangiales bacterium HEX2675071.1 314 aa
Pseudomonadati Myxococcota Myxococcia Myxococcales Myxococcales bacterium isolate HK STAS MYXO 19 MCB9667201.1
Pseudomonadati Myxococcota Myxococcia Myxococcales Myxococcales bacterium isolate Fred 18 Q3 R57 64 BAT3C.417 QQR90310.1

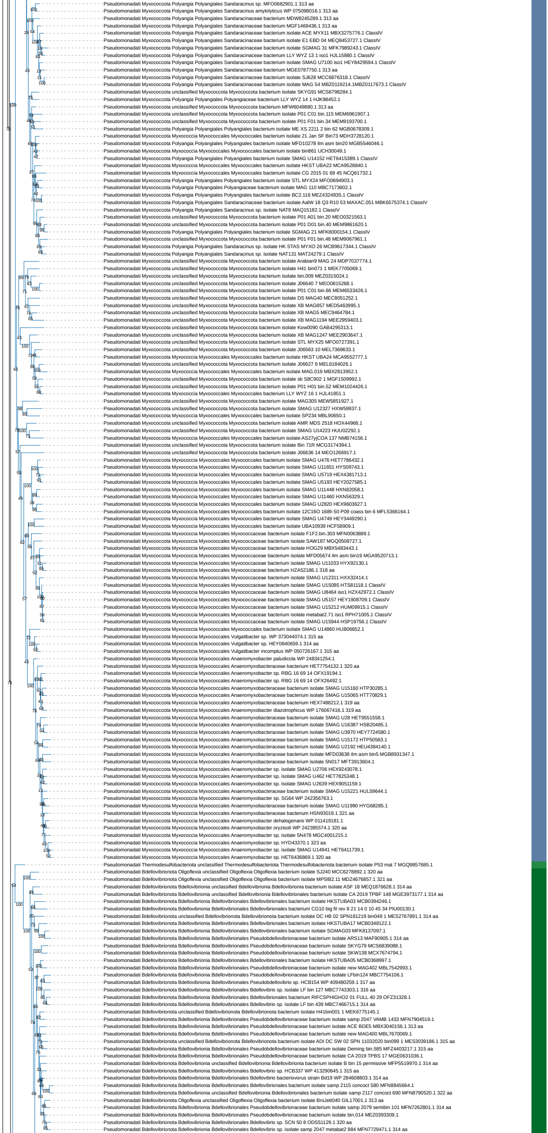
Pseudomonadati Myxococcota Polyangia Polyangiales Sandaracinus sp. MFO0682901.1 313 aa
Pseudomonadati Myxococcota Polyangia Polyangiales Sandaracinus amylolyticus WP 075098016.1 313 aa
Pseudomonadati Myxococcota Polyangia Polyangiales Sandaracinaceae bacterium MDW8245289.1 313 aa
Pseudomonadati Myxococcota Polyangia Polyangiales Sandaracinaceae bacterium MGF1469436.1 313 aa
Pseudomonadati Myxococcota Polyangia Polyangiales Sandaracinaceae bacterium isolate ACE MYX11 MBX3275776.1 ClassIV
Pseudomonadati Myxococcota Polyangia Polyangiales Sandaracinaceae bacterium isolate E1 EBD 04 MEQ8453727.1 ClassIV
Pseudomonadati Myxococcota Polyangia Polyangiales Sandaracinaceae bacterium isolate SGMAG 31 MFK7989243.1 ClassIV
Pseudomonadati Myxococcota Polyangia Polyangiales Sandaracinaceae bacterium LLY WYZ 13 1 iso1 HJL15880.1 ClassIV
Pseudomonadati Myxococcota Polyangia Polyangiales Sandaracinaceae bacterium isolate SMAG U7100 iso1 HEY8429594.1 ClassIV
Pseudomonadati Myxococcota Polyangia Polyangiales Sandaracinaceae bacterium MGE0787750.1 313 aa
Pseudomonadati Myxococcota Polyangia Polyangiales Sandaracinaceae bacterium isolate SJ628 MCC6876318.1 ClassIV
Pseudomonadati Myxococcota Polyangia Polyangiales Sandaracinaceae bacterium isolate MAG 54 MBZ0119214.1MBZ0117673.1 ClassIV
Pseudomonadati Myxococcota unclassified Myxococcota Myxococcota bacterium isolate SKYG91 MCS6798284.1
Pseudomonadati Myxococcota Polyangia Polyangiales Polyangiaceae bacterium LLY WYZ 14 1 HJK98452.1
Pseudomonadati Myxococcota unclassified Myxococcota Myxococcota bacterium MFW6049880.1 313 aa
Pseudomonadati Myxococcota unclassified Myxococcota Myxococcota bacterium isolate P01 C01 bin.115 MEM6961907.1
Pseudomonadati Myxococcota unclassified Myxococcota Myxococcota bacterium isolate P01 F01 bin.34 MEM9193700.1
Pseudomonadati Myxococcota Polyangia Polyangiales Polyangiales bacterium isolate ME XS 2211 2 bin 62 MGB0678309.1
Pseudomonadati Myxococcota Myxococcia Myxococcales Myxococcales bacterium isolate 21 Jan SF Bin73 MDH3728120.1
Pseudomonadati Myxococcota Polyangia Polyangiales Polyangiales bacterium isolate MFD10278 ilm asm bin20 MGB5546046.1
Pseudomonadati Myxococcota Myxococcia Myxococcales Myxococcales bacterium isolate bin861 UCH30049.1
Pseudomonadati Myxococcota Polyangia Polyangiales Polyangiales bacterium isolate SMAG U14152 HET6415389.1 ClassIV
Pseudomonadati Myxococcota Myxococcia Myxococcales Myxococcales bacterium isolate HKST UBA22 MCA9528840.1
Pseudomonadati Myxococcota Myxococcia Myxococcales Myxococcales bacterium isolate CG 2015 01 69 45 NCQ61732.1
Pseudomonadati Myxococcota Polyangia Polyangiales Polyangiales bacterium isolate STL MYX24 MFO0694903.1
Pseudomonadati Myxococcota Polyangia Polyangiales Polyangiaceae bacterium isolate MAG 110 MBC7173602.1
Pseudomonadati Myxococcota Polyangia Polyangiales Polyangiales bacterium isolate BC2.116 MEZ4324835.1 ClassIV
Pseudomonadati Myxococcota Polyangia Polyangiales Sandaracinaceae bacterium isolate AalW 18 Q3 R10 53 MAXAC.051 MBK6575374.1 ClassIV
Pseudomonadati Myxococcota Polyangia Polyangiales Sandaracinus sp. isolate NAT8 MAQ15182.1 ClassIV
Pseudomonadati Myxococcota unclassified Myxococcota Myxococcota bacterium isolate P01 A01 bin.20 MEO0321563.1
Pseudomonadati Myxococcota unclassified Myxococcota Myxococcota bacterium isolate P01 D01 bin.40 MEM9861620.1
Pseudomonadati Myxococcota Polyangia Polyangiales Polyangiales bacterium isolate SGMAG 21 MFK8000154.1 ClassIV
Pseudomonadati Myxococcota unclassified Myxococcota Myxococcota bacterium isolate P01 F01 bin.48 MEM9067961.1
Pseudomonadati Myxococcota Polyangia Polyangiales Sandaracinus sp. isolate HK STAS MYXO 26 MCB9617344.1 ClassIV
Pseudomonadati Myxococcota Polyangia Polyangiales Sandaracinus sp. isolate NAT131 MAT24279.1 ClassIV
Pseudomonadati Myxococcota unclassified Myxococcota Myxococcota bacterium isolate Arabian9 MAG 24 MDP7037774.1
Pseudomonadati Myxococcota unclassified Myxococcota Myxococcota bacterium isolate H41 bin071 1 MEK7705069.1
Pseudomonadati Myxococcota unclassified Myxococcota Myxococcota bacterium isolate bin.009 MEZ0315024.1
Pseudomonadati Myxococcota unclassified Myxococcota Myxococcota bacterium isolate J06640 7 MEO0815268.1
Pseudomonadati Myxococcota unclassified Myxococcota Myxococcota bacterium isolate P01 C01 bin.66 MEM6533426.1
Pseudomonadati Myxococcota unclassified Myxococcota Myxococcota bacterium isolate DS MAG40 MEC8051252.1
Pseudomonadati Myxococcota unclassified Myxococcota Myxococcota bacterium isolate XB MAG857 MED5463995.1
Pseudomonadati Myxococcota unclassified Myxococcota Myxococcota bacterium isolate XB MAG5 MEC9464784.1
Pseudomonadati Myxococcota unclassified Myxococcota Myxococcota bacterium isolate XB MAG1194 MEE2959403.1
Pseudomonadati Myxococcota unclassified Myxococcota Myxococcota bacterium isolate Kow0090 GAB4295313.1
Pseudomonadati Myxococcota unclassified Myxococcota Myxococcota bacterium isolate XB MAG1247 MEE2903647.1
Pseudomonadati Myxococcota unclassified Myxococcota Myxococcota bacterium isolate STL MYX25 MFO0727391.1
Pseudomonadati Myxococcota unclassified Myxococcota Myxococcota bacterium isolate J06563 10 MEL7369633.1
Pseudomonadati Myxococcota Myxococcia Myxococcales Myxococcales bacterium isolate HKST UBA24 MCA9552777.1
Pseudomonadati Myxococcota unclassified Myxococcota Myxococcota bacterium isolate J06627 6 MEL6184026.1
Pseudomonadati Myxococcota Myxococcia Myxococcales Myxococcales bacterium isolate MAG.019 MBX2813952.1
Pseudomonadati Myxococcota unclassified Myxococcota Myxococcota bacterium isolate sb S8C902 1 MGF1509992.1
Pseudomonadati Myxococcota unclassified Myxococcota Myxococcota bacterium isolate P01 H01 bin.52 MEM1024426.1
Pseudomonadati Myxococcota Myxococcia Myxococcales Myxococcales bacterium LLY WYZ 16 1 HJL41851.1
Pseudomonadati Myxococcota unclassified Myxococcota Myxococcota bacterium isolate MAG305 MEW5851927.1
Pseudomonadati Myxococcota unclassified Myxococcota Myxococcota bacterium isolate SMAG U12327 HXW59937.1
Pseudomonadati Myxococcota Myxococcia Myxococcales Myxococcales bacterium isolate SP234 MBL90650.1
Pseudomonadati Myxococcota unclassified Myxococcota Myxococcota bacterium isolate AMR MDS 2518 HOX44968.1
Pseudomonadati Myxococcota unclassified Myxococcota Myxococcota bacterium isolate SMAG U14223 HUU02292.1
Pseudomonadati Myxococcota Myxococcia Myxococcales Myxococcales bacterium isolate AS27yjCOA 137 NMB74156.1
Pseudomonadati Myxococcota unclassified Myxococcota Myxococcota bacterium isolate Bin 71R MCG3174394.1
Pseudomonadati Myxococcota unclassified Myxococcota Myxococcota bacterium isolate J06636 14 MEO1266917.1
Pseudomonadati Myxococcota Myxococcia Myxococcales Myxococcales bacterium isolate SMAG U476 HET7786432.1
Pseudomonadati Myxococcota Myxococcia Myxococcales Myxococcales bacterium isolate SMAG U11651 HYS09743.1
Pseudomonadati Myxococcota Myxococcia Myxococcales Myxococcales bacterium isolate SMAG U5719 HEX4381713.1
Pseudomonadati Myxococcota Myxococcia Myxococcales Myxococcales bacterium isolate SMAG U5193 HEY2027585.1
Pseudomonadati Myxococcota Myxococcia Myxococcales Myxococcales bacterium isolate SMAG U11448 HXN82058.1
Pseudomonadati Myxococcota Myxococcia Myxococcales Myxococcales bacterium isolate SMAG U11460 HXN56329.1
Pseudomonadati Myxococcota Myxococcia Myxococcales Myxococcales bacterium isolate SMAG U2820 HEX9603627.1
Pseudomonadati Myxococcota Myxococcia Myxococcales Myxococcales bacterium isolate 12C16O 168h 50 P09 coass bin 6 MFL5366164.1
Pseudomonadati Myxococcota Myxococcia Myxococcales Myxococcales bacterium isolate SMAG U4749 HEY3449290.1
Pseudomonadati Myxococcota Myxococcia Myxococcales Myxococcales bacterium isolate UBA10939 HCF58909.1
Pseudomonadati Myxococcota Myxococcia Myxococcales Myxococcaceae bacterium isolate F1F2.bin.303 MFN0063889.1
Pseudomonadati Myxococcota Myxococcia Myxococcales Myxococcaceae bacterium isolate SAW187 MGQ0508727.1
Pseudomonadati Myxococcota Myxococcia Myxococcales Myxococcaceae bacterium isolate HOG29 MBX5483443.1
Pseudomonadati Myxococcota Myxococcia Myxococcales Myxococcaceae bacterium isolate MFD05674 ilm asm bin19 MGA9520713.1
Pseudomonadati Myxococcota Myxococcia Myxococcales Myxococcaceae bacterium isolate SMAG U11033 HYX92130.1
Pseudomonadati Myxococcota Myxococcia Myxococcales Myxococcaceae bacterium HZA52186.1 318 aa
Pseudomonadati Myxococcota Myxococcia Myxococcales Myxococcaceae bacterium isolate SMAG U12311 HXX32414.1
Pseudomonadati Myxococcota Myxococcia Myxococcales Myxococcaceae bacterium isolate SMAG U15085 HTS81118.1 ClassIV
Pseudomonadati Myxococcota Myxococcia Myxococcales Myxococcaceae bacterium isolate SMAG U8464 iso1 HZX42972.1 ClassIV
Pseudomonadati Myxococcota Myxococcia Myxococcales Myxococcaceae bacterium isolate SMAG U5157 HEY1908709.1 ClassIV
Pseudomonadati Myxococcota Myxococcia Myxococcales Myxococcaceae bacterium isolate SMAG U15212 HUM09915.1 ClassIV
Pseudomonadati Myxococcota Myxococcia Myxococcales Myxococcaceae bacterium isolate metabat2.71 iso1 RPH71005.1 ClassIV
Pseudomonadati Myxococcota Myxococcia Myxococcales Myxococcaceae bacterium isolate SMAG U15944 HSP19756.1 ClassIV
Pseudomonadati Myxococcota Myxococcia Myxococcales Myxococcales bacterium isolate SMAG U14860 HUB06652.1
Pseudomonadati Myxococcota Myxococcia Myxococcales Vulgatibacter sp. WP 373044074.1 315 aa
Pseudomonadati Myxococcota Myxococcia Myxococcales Vulgatibacter sp. HEY0840659.1 314 aa
Pseudomonadati Myxococcota Myxococcia Myxococcales Vulgatibacter incomptus WP 050726167.1 315 aa
Pseudomonadati Myxococcota Myxococcia Myxococcales Anaeromyxobacter paludicola WP 248341254.1
Pseudomonadati Myxococcota Myxococcia Myxococcales Anaeromyxobacteraceae bacterium HET7754132.1 320 aa
Pseudomonadati Myxococcota Myxococcia Myxococcales Anaeromyxobacter sp. RBG 16 69 14 OFX19194.1
Pseudomonadati Myxococcota Myxococcia Myxococcales Anaeromyxobacter sp. RBG 16 69 14 OFX26492.1
Pseudomonadati Myxococcota Myxococcia Myxococcales Anaeromyxobacteraceae bacterium isolate SMAG U15160 HTP30285.1
Pseudomonadati Myxococcota Myxococcia Myxococcales Anaeromyxobacteraceae bacterium isolate SMAG U15065 HTT70829.1
Pseudomonadati Myxococcota Myxococcia Myxococcales Anaeromyxobacteraceae bacterium HEX7488212.1 319 aa
Pseudomonadati Myxococcota Myxococcia Myxococcales Anaeromyxobacter diazotrophicus WP 176067416.1 319 aa
Pseudomonadati Myxococcota Myxococcia Myxococcales Anaeromyxobacteraceae bacterium isolate SMAG U28 HET9551558.1
Pseudomonadati Myxococcota Myxococcia Myxococcales Anaeromyxobacteraceae bacterium isolate SMAG U16387 HSB20485.1
Pseudomonadati Myxococcota Myxococcia Myxococcales Anaeromyxobacteraceae bacterium isolate SMAG U3970 HEY7724580.1
Pseudomonadati Myxococcota Myxococcia Myxococcales Anaeromyxobacteraceae bacterium isolate SMAG U15172 HTP50563.1
Pseudomonadati Myxococcota Myxococcia Myxococcales Anaeromyxobacteraceae bacterium isolate SMAG U2192 HEU4384140.1
Pseudomonadati Myxococcota Myxococcia Myxococcales Anaeromyxobacteraceae bacterium isolate MFD03638 ilm asm bin5 MGB8931347.1
Pseudomonadati Myxococcota Myxococcia Myxococcales Anaeromyxobacteraceae bacterium isolate 5N017 MFT3913604.1
Pseudomonadati Myxococcota Myxococcia Myxococcales Anaeromyxobacter sp. isolate SMAG U2706 HEX9243078.1
Pseudomonadati Myxococcota Myxococcia Myxococcales Anaeromyxobacter sp. isolate SMAG U462 HET7825348.1
Pseudomonadati Myxococcota Myxococcia Myxococcales Anaeromyxobacter sp. isolate SMAG U2639 HEX9051159.1
Pseudomonadati Myxococcota Myxococcia Myxococcales Anaeromyxobacteraceae bacterium isolate SMAG U15221 HUL59644.1
Pseudomonadati Myxococcota Myxococcia Myxococcales Anaeromyxobacter sp. SG64 WP 242356763.1
Pseudomonadati Myxococcota Myxococcia Myxococcales Anaeromyxobacteraceae bacterium isolate SMAG U11990 HYG68285.1
Pseudomonadati Myxococcota Myxococcia Myxococcales Anaeromyxobacteraceae bacterium HSN93019.1 321 aa
Pseudomonadati Myxococcota Myxococcia Myxococcales Anaeromyxobacter dehalogenans WP 011419181.1
Pseudomonadati Myxococcota Myxococcia Myxococcales Anaeromyxobacter oryzisoli WP 242395574.1 320 aa
Pseudomonadati Myxococcota Myxococcia Myxococcales Anaeromyxobacter sp. isolate 5N478 MGC4001215.1
Pseudomonadati Myxococcota Myxococcia Myxococcales Anaeromyxobacter sp. HYD43370.1 323 aa
Pseudomonadati Myxococcota Myxococcia Myxococcales Anaeromyxobacter sp. isolate SMAG U14941 HET6411739.1
Pseudomonadati Myxococcota Myxococcia Myxococcales Anaeromyxobacter sp. HET6436869.1 320 aa
Pseudomonadati Thermodesulfobacteriota unclassified Thermodesulfobacteriota Thermodesulfobacteriota bacterium isolate P53 mat 7 MGQ9857685.1
Pseudomonadati Bdellovibrionota Oligoflexia unclassified Oligoflexia Oligoflexia bacterium isolate SJ240 MCC6278892.1 320 aa
Pseudomonadati Bdellovibrionota Oligoflexia unclassified Oligoflexia Oligoflexia bacterium isolate MP5IB2.11 MDZ4676857.1 322 aa
Pseudomonadati Bdellovibrionota Bdellovibrionia unclassified Bdellovibrionia Bdellovibrionia bacterium isolate ASF 18 MEQ1876628.1 314 aa
Pseudomonadati Bdellovibrionota Bdellovibrionia unclassified Bdellovibrionia Bdellovibrionales bacterium isolate CA 2019 TPBF 148 MGE3973177.1 314 aa
Pseudomonadati Bdellovibrionota Bdellovibrionia Bdellovibrionales Bdellovibrionales bacterium isolate HKSTUBA03 MCB0394246.1
Pseudomonadati Bdellovibrionota Bdellovibrionia Bdellovibrionales Bdellovibrionales bacterium CG10 big fil rev 8 21 14 0 10 45 34 PIU00130.1
Pseudomonadati Bdellovibrionota unclassified Bdellovibrionota Bdellovibrionota bacterium isolate DC HB 02 SPN181219 bin049 1 MES2767891.1 314 aa
Pseudomonadati Bdellovibrionota Bdellovibrionia Bdellovibrionales Bdellovibrionales bacterium isolate HKSTUBA17 MCB0348122.1
Pseudomonadati Bdellovibrionota Bdellovibrionia Bdellovibrionales Bdellovibrionales bacterium isolate SGMAG03 MFK8137097.1
Pseudomonadati Bdellovibrionota Bdellovibrionia Bdellovibrionales Pseudobdellovibrionaceae bacterium isolate ARS13 MAF90905.1 314 aa
Pseudomonadati Bdellovibrionota Bdellovibrionia Bdellovibrionales Pseudobdellovibrionaceae bacterium isolate SKYG79 MCS6839088.1
Pseudomonadati Bdellovibrionota Bdellovibrionia Bdellovibrionales Pseudobdellovibrionaceae bacterium isolate SKW138 MCX7674794.1
Pseudomonadati Bdellovibrionota Bdellovibrionia Bdellovibrionales Bdellovibrionales bacterium isolate HKSTUBA05 MCB0368997.1
Pseudomonadati Bdellovibrionota Bdellovibrionia Bdellovibrionales Pseudobdellovibrionaceae bacterium isolate new MAG402 MBL7542993.1
Pseudomonadati Bdellovibrionota Bdellovibrionia Bdellovibrionales Pseudobdellovibrionaceae bacterium isolate LFbin124 MBC7754106.1
Pseudomonadati Bdellovibrionota Bdellovibrionia Bdellovibrionales Pseudobdellovibrio sp. HCB154 WP 409480259.1 317 aa
Pseudomonadati Bdellovibrionota Bdellovibrionia Bdellovibrionales Bdellovibrio sp. isolate LF bin 127 MBC7743303.1 316 aa
Pseudomonadati Bdellovibrionota Bdellovibrionia Bdellovibrionales Bdellovibrionales bacterium RIFCSPHIGHO2 01 FULL 40 29 OFZ31328.1
Pseudomonadati Bdellovibrionota Bdellovibrionia Bdellovibrionales Bdellovibrio sp. isolate LF bin 439 MBC7466715.1 314 aa
Pseudomonadati Bdellovibrionota unclassified Bdellovibrionota Bdellovibrionota bacterium isolate H41bin001 1 MEZ6775145.1
Pseudomonadati Bdellovibrionota Bdellovibrionia Bdellovibrionales Pseudobdellovibrionaceae bacterium isolate samp 2047 VAMB 1433 MFN7904519.1
Pseudomonadati Bdellovibrionota Bdellovibrionia Bdellovibrionales Pseudobdellovibrionaceae bacterium isolate ACE BDE5 MBX3040156.1 313 aa
Pseudomonadati Bdellovibrionota Bdellovibrionia Bdellovibrionales Pseudobdellovibrionaceae bacterium isolate new MAG400 MBL7670069.1
Pseudomonadati Bdellovibrionota unclassified Bdellovibrionota Bdellovibrionota bacterium isolate ADI DC SW 02 SPN 11032020 bin099 1 MES3039186.1 315 aa
Pseudomonadati Bdellovibrionota Bdellovibrionia Bdellovibrionales Pseudobdellovibrionaceae bacterium isolate Deming bin.585 MFZ4403217.1 315 aa
Pseudomonadati Bdellovibrionota Bdellovibrionia Bdellovibrionales Pseudobdellovibrionaceae bacterium isolate CA 2019 TPBS 17 MGE0631036.1
Pseudomonadati Bdellovibrionota Bdellovibrionia unclassified Bdellovibrionia Bdellovibrionia bacterium isolate B bin 15 permissive MFP5519970.1 314 aa
Pseudomonadati Bdellovibrionota Bdellovibrionia Bdellovibrionales Bdellovibrio sp. HCB337 WP 413290645.1 315 aa
Pseudomonadati Bdellovibrionota Bdellovibrionia Bdellovibrionales Bdellovibrio bacteriovorus strain Bd19 WP 284608803.1 314 aa
Pseudomonadati Bdellovibrionota Bdellovibrionia Bdellovibrionales Bdellovibrionales bacterium isolate samp 2115 concoct 580 MFN8845664.1
Pseudomonadati Bdellovibrionota Bdellovibrionia unclassified Bdellovibrionia Bdellovibrionales bacterium isolate samp 2117 concoct 690 MFN8790520.1 322 aa
Pseudomonadati Bdellovibrionota Oligoflexia unclassified Oligoflexia Oligoflexia bacterium isolate BroJett040 GIL17001.1 313 aa
Pseudomonadati Bdellovibrionota Bdellovibrionia Bdellovibrionales Pseudobdellovibrionaceae bacterium isolate samp 2079 semibin 101 MFN7262801.1 314 aa
Pseudomonadati Bdellovibrionota Bdellovibrionia Bdellovibrionales Pseudobdellovibrionaceae bacterium isolate bin.014 MEZ0393309.1
Pseudomonadati Bdellovibrionota Bdellovibrionia Bdellovibrionales Bdellovibrio sp. SCN 50 8 ODS51129.1 320 aa
Pseudomonadati Bdellovibrionota Bdellovibrionia Bdellovibrionales Bdellovibrio sp. isolate samp 2047 metabat2 884 MFN7729471.1 314 aa

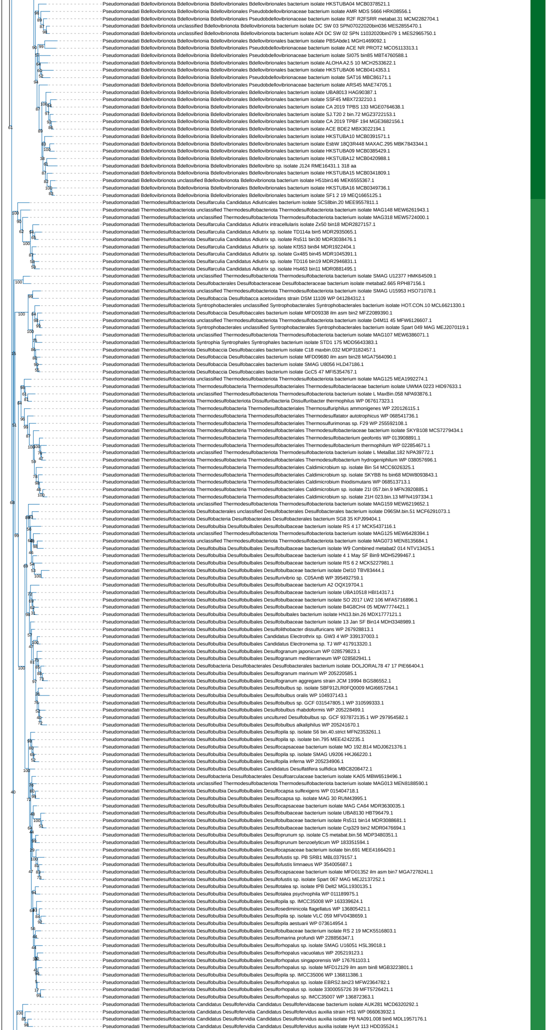
Pseudomonadati Bdellovibrionota Bdellovibrionia Bdellovibrionales Bdellovibrionales bacterium isolate HKSTUBA04 MCB0378521.1
Pseudomonadati Bdellovibrionota Bdellovibrionia Bdellovibrionales Pseudobdellovibrionaceae bacterium isolate AMR MDS 5666 HRK08556.1
Pseudomonadati Bdellovibrionota Bdellovibrionia Bdellovibrionales Pseudobdellovibrionaceae bacterium isolate R2F R2FSRR metabat.31 MCM2282704.1
Pseudomonadati Bdellovibrionota unclassified Bdellovibrionota Bdellovibrionota bacterium isolate DC SW 03 SPN07022020bin036 MES2855470.1
Pseudomonadati Bdellovibrionota unclassified Bdellovibrionota Bdellovibrionota bacterium isolate ADI DC SW 02 SPN 11032020bin079 1 MES2965750.1
Pseudomonadati Bdellovibrionota Bdellovibrionia Bdellovibrionales Bdellovibrionales bacterium isolate PBSAbde1 MGH1469092.1
Pseudomonadati Bdellovibrionota Bdellovibrionia Bdellovibrionales Pseudobdellovibrionaceae bacterium isolate ACE NR PROT2 MCO5113313.1
Pseudomonadati Bdellovibrionota Bdellovibrionia Bdellovibrionales Pseudobdellovibrionaceae bacterium isolate SI075 bin85 MBT4760588.1
Pseudomonadati Bdellovibrionota Bdellovibrionia Bdellovibrionales Bdellovibrionales bacterium isolate ALOHA A2.5 10 MCH2533622.1
Pseudomonadati Bdellovibrionota Bdellovibrionia Bdellovibrionales Bdellovibrionales bacterium isolate HKSTUBA06 MCB0414353.1
Pseudomonadati Bdellovibrionota Bdellovibrionia Bdellovibrionales Pseudobdellovibrionaceae bacterium isolate SAT16 MBC86171.1
Pseudomonadati Bdellovibrionota Bdellovibrionia Bdellovibrionales Pseudobdellovibrionaceae bacterium isolate ARS45 MAE74705.1
Pseudomonadati Bdellovibrionota Bdellovibrionia Bdellovibrionales Bdellovibrionales bacterium isolate UBA8013 HAG90387.1
Pseudomonadati Bdellovibrionota Bdellovibrionia Bdellovibrionales Bdellovibrionales bacterium isolate SSF45 MBX7232210.1
Pseudomonadati Bdellovibrionota Bdellovibrionia Bdellovibrionales Bdellovibrionales bacterium isolate CA 2019 TPBS 133 MGE0764638.1
Pseudomonadati Bdellovibrionota Bdellovibrionia Bdellovibrionales Bdellovibrionales bacterium isolate SJ.T20 2 bin.72 MGZ3722153.1
Pseudomonadati Bdellovibrionota Bdellovibrionia Bdellovibrionales Bdellovibrionales bacterium isolate CA 2019 TPBF 194 MGE3682156.1
Pseudomonadati Bdellovibrionota Bdellovibrionia Bdellovibrionales Bdellovibrionales bacterium isolate ACE BDE2 MBX3022194.1
Pseudomonadati Bdellovibrionota Bdellovibrionia Bdellovibrionales Bdellovibrionales bacterium isolate HKSTUBA10 MCB0391571.1
Pseudomonadati Bdellovibrionota Bdellovibrionia Bdellovibrionales Bdellovibrionales bacterium isolate EsbW 18Q3R448 MAXAC.295 MBK7843344.1
Pseudomonadati Bdellovibrionota Bdellovibrionia Bdellovibrionales Bdellovibrionales bacterium isolate HKSTUBA09 MCB0385429.1
Pseudomonadati Bdellovibrionota Bdellovibrionia Bdellovibrionales Bdellovibrionales bacterium isolate HKSTUBA12 MCB0420988.1
Pseudomonadati Bdellovibrionota Bdellovibrionia Bdellovibrionales Bdellovibrio sp. isolate J124 RME16431.1 318 aa
Pseudomonadati Bdellovibrionota Bdellovibrionia Bdellovibrionales Bdellovibrionales bacterium isolate HKSTUBA15 MCB0341809.1
Pseudomonadati Bdellovibrionota unclassified Bdellovibrionota Bdellovibrionota bacterium isolate H51bin146 MEK6555367.1
Pseudomonadati Bdellovibrionota Bdellovibrionia Bdellovibrionales Bdellovibrionales bacterium isolate HKSTUBA16 MCB0349736.1
Pseudomonadati Bdellovibrionota Bdellovibrionia Bdellovibrionales Bdellovibrionales bacterium isolate SF1 2 19 MEQ1665125.1
Pseudomonadati Thermodesulfobacteriota Desulfarculia Candidatus Adiutricales bacterium isolate SCS8bin.20 MEE9557811.1
Pseudomonadati Thermodesulfobacteriota unclassified Thermodesulfobacteriota Thermodesulfobacteriota bacterium isolate MAG148 MEW6261943.1
Pseudomonadati Thermodesulfobacteriota unclassified Thermodesulfobacteriota Thermodesulfobacteriota bacterium isolate MAG318 MEW5724000.1
Pseudomonadati Thermodesulfobacteriota Desulfarculia Candidatus Adiutrix intracellularis isolate Zx50 bin18 MDR2827157.1
Pseudomonadati Thermodesulfobacteriota Desulfarculia Candidatus Adiutrix sp. isolate TD114a bin5 MDR2935065.1
Pseudomonadati Thermodesulfobacteriota Desulfarculia Candidatus Adiutrix sp. isolate Rs511 bin30 MDR3038476.1
Pseudomonadati Thermodesulfobacteriota Desulfarculia Candidatus Adiutrix sp. isolate Kf353 bin84 MDR1922404.1
Pseudomonadati Thermodesulfobacteriota Desulfarculia Candidatus Adiutrix sp. isolate Gx485 bin45 MDR1045391.1
Pseudomonadati Thermodesulfobacteriota Desulfarculia Candidatus Adiutrix sp. isolate TD116 bin19 MDR2946831.1
Pseudomonadati Thermodesulfobacteriota Desulfarculia Candidatus Adiutrix sp. isolate Hs463 bin11 MDR0881495.1
Pseudomonadati Thermodesulfobacteriota unclassified Thermodesulfobacteriota Thermodesulfobacteriota bacterium isolate SMAG U12377 HMK64509.1
Pseudomonadati Thermodesulfobacteriota Desulfobacterales Desulfobacteraceae Desulfobacteraceae bacterium isolate metabat2.665 RPH87156.1
Pseudomonadati Thermodesulfobacteriota unclassified Thermodesulfobacteriota Thermodesulfobacteriota bacterium isolate SMAG U15953 HSO71078.1
Pseudomonadati Thermodesulfobacteriota Desulfobaccia Desulfobacca acetoxidans strain DSM 11109 WP 041284312.1
Pseudomonadati Thermodesulfobacteriota Syntrophobacterales unclassified Syntrophobacterales Syntrophobacterales bacterium isolate HOT.CON.10 MCL6621330.1
Pseudomonadati Thermodesulfobacteriota Desulfobaccia Desulfobaccales Desulfobaccales bacterium isolate MFD09338 ilm asm bin2 MFZ2089390.1
Pseudomonadati Thermodesulfobacteriota unclassified Thermodesulfobacteriota Thermodesulfobacteriota bacterium isolate D4M11 45 MFW6126607.1
Pseudomonadati Thermodesulfobacteriota Syntrophobacterales unclassified Syntrophobacterales Syntrophobacterales bacterium isolate Spart 049 MAG MEJ2070119.1
Pseudomonadati Thermodesulfobacteriota unclassified Thermodesulfobacteriota Thermodesulfobacteriota bacterium isolate MAG107 MEW6386071.1
Pseudomonadati Thermodesulfobacteriota Syntrophia Syntrophales Syntrophales bacterium isolate STD1 175 MDD5643383.1
Pseudomonadati Thermodesulfobacteriota Desulfobaccia Desulfobaccales Desulfobaccales bacterium isolate C18 maxbin.032 MDP3182457.1
Pseudomonadati Thermodesulfobacteriota Desulfobaccia Desulfobaccales Desulfobaccales bacterium isolate MFD09680 ilm asm bin28 MGA7564090.1
Pseudomonadati Thermodesulfobacteriota Desulfobaccia Desulfobaccales Desulfobaccales bacterium isolate SMAG U8056 HLD47186.1
Pseudomonadati Thermodesulfobacteriota Desulfobaccia Desulfobaccales Desulfobaccales bacterium isolate GcC5 47 MFI5354767.1
Pseudomonadati Thermodesulfobacteriota unclassified Thermodesulfobacteriota Thermodesulfobacteriota bacterium isolate MAG125 MEA1992274.1
Pseudomonadati Thermodesulfobacteriota Thermodesulfobacteria Thermodesulfobacteriales Thermodesulfobacteriaceae bacterium isolate UWMA 0223 HID97633.1
Pseudomonadati Thermodesulfobacteriota unclassified Thermodesulfobacteriota Thermodesulfobacteriota bacterium isolate L MaxBin.058 NPA93876.1
Pseudomonadati Thermodesulfobacteriota Thermodesulfobacteriota Dissulfuribacteria Dissulfuribacter thermophilus WP 067617323.1
Pseudomonadati Thermodesulfobacteriota Thermodesulfobacteria Thermodesulfobacteriales Thermosulfuriphilus ammonigenes WP 220126115.1
Pseudomonadati Thermodesulfobacteriota Thermodesulfobacteria Thermodesulfobacteriales Thermodesulfatator autotrophicus WP 068541736.1
Pseudomonadati Thermodesulfobacteriota Thermodesulfobacteria Thermodesulfobacteriales Thermosulfurimonas sp. F29 WP 255592108.1
Pseudomonadati Thermodesulfobacteriota Thermodesulfobacteria Thermodesulfobacteriales Thermodesulfobacteriaceae bacterium isolate SKYB108 MCS7279434.1
Pseudomonadati Thermodesulfobacteriota Thermodesulfobacteria Thermodesulfobacteriales Thermodesulfobacterium geofontis WP 013908891.1
Pseudomonadati Thermodesulfobacteriota Thermodesulfobacteria Thermodesulfobacteriales Thermodesulfobacterium thermophilum WP 022854671.1
Pseudomonadati Thermodesulfobacteriota unclassified Thermodesulfobacteriota Thermodesulfobacteriota bacterium isolate L MetaBat.182 NPA39772.1
Pseudomonadati Thermodesulfobacteriota Thermodesulfobacteria Thermodesulfobacteriales Thermodesulfobacterium hydrogeniphilum WP 038057696.1
Pseudomonadati Thermodesulfobacteriota Thermodesulfobacteria Thermodesulfobacteriales Caldimicrobium sp. isolate Bin S4 MCC6026325.1
Pseudomonadati Thermodesulfobacteriota Thermodesulfobacteria Thermodesulfobacteriales Caldimicrobium sp. isolate SKYBB hs bin68 MDW8093843.1
Pseudomonadati Thermodesulfobacteriota Thermodesulfobacteria Thermodesulfobacteriales Caldimicrobium thiodismutans WP 068513713.1
Pseudomonadati Thermodesulfobacteriota Thermodesulfobacteria Thermodesulfobacteriales Caldimicrobium sp. isolate 21I 057.bin.9 MFN3920885.1
Pseudomonadati Thermodesulfobacteriota Thermodesulfobacteria Thermodesulfobacteriales Caldimicrobium sp. isolate 21H 023.bin.13 MFN4197334.1
Pseudomonadati Thermodesulfobacteriota unclassified Thermodesulfobacteriota Thermodesulfobacteriota bacterium isolate MAG159 MEW6219652.1
Pseudomonadati Thermodesulfobacteriota Desulfobacterales unclassified Desulfobacterales Desulfobacterales bacterium isolate D96SM.bin.51 MCF6291073.1
Pseudomonadati Thermodesulfobacteriota Desulfobacteria Desulfobacterales Desulfobacterales bacterium SG8 35 KPJ99404.1
Pseudomonadati Thermodesulfobacteriota Desulfobulbia Desulfobulbales Desulfobulbaceae bacterium isolate RS 4 17 MCK5437116.1
Pseudomonadati Thermodesulfobacteriota unclassified Thermodesulfobacteriota Thermodesulfobacteriota bacterium isolate MAG125 MEW6428394.1
Pseudomonadati Thermodesulfobacteriota unclassified Thermodesulfobacteriota Thermodesulfobacteriota bacterium isolate MAG073 MEN8135684.1
Pseudomonadati Thermodesulfobacteriota Desulfobulbia Desulfobulbales Desulfobulbaceae bacterium isolate W9 Combined metabat2 014 NTV13425.1
Pseudomonadati Thermodesulfobacteriota Desulfobulbia Desulfobulbales Desulfobulbaceae bacterium isolate 4 1 May SF Bin9 MDH5299467.1
Pseudomonadati Thermodesulfobacteriota Desulfobulbia Desulfobulbales Desulfobulbaceae bacterium isolate RS 6 2 MCK5227981.1
Pseudomonadati Thermodesulfobacteriota Desulfobulbia Desulfobulbales Desulfobulbaceae bacterium isolate Del10 TBV83444.1
Pseudomonadati Thermodesulfobacteriota Desulfobulbia Desulfobulbales Desulfurivibrio sp. C05AmB WP 395492759.1
Pseudomonadati Thermodesulfobacteriota Desulfobulbia Desulfobulbales Desulfobulbaceae bacterium A2 OQX19704.1
Pseudomonadati Thermodesulfobacteriota Desulfobulbia Desulfobulbales Desulfobulbaceae bacterium isolate UBA10518 HBI14317.1
Pseudomonadati Thermodesulfobacteriota Desulfobulbia Desulfobulbales Desulfobulbaceae bacterium isolate SO 2017 LW2 106 MFA5716896.1
Pseudomonadati Thermodesulfobacteriota Desulfobulbia Desulfobulbales Desulfobulbaceae bacterium isolate B4G8CH4 05 MDW7774421.1
Pseudomonadati Thermodesulfobacteriota Desulfobulbia Desulfobulbales Desulfobulbales bacterium isolate HN13.bin.26 MDX1777121.1
Pseudomonadati Thermodesulfobacteriota Desulfobulbia Desulfobulbales Desulfobulbaceae bacterium isolate 13 Jan SF Bin14 MDH3348989.1
Pseudomonadati Thermodesulfobacteriota Desulfobulbia Desulfobulbales Desulfolithobacter dissulfuricans WP 267928813.1
Pseudomonadati Thermodesulfobacteriota Desulfobulbia Desulfobulbales Candidatus Electrothrix sp. GW3 4 WP 339137003.1
Pseudomonadati Thermodesulfobacteriota Desulfobulbia Desulfobulbales Candidatus Electronema sp. TJ WP 417913320.1
Pseudomonadati Thermodesulfobacteriota Desulfobulbia Desulfobulbales Desulfogranum japonicum WP 028579823.1
Pseudomonadati Thermodesulfobacteriota Desulfobulbia Desulfobulbales Desulfogranum mediterraneum WP 028582941.1
Pseudomonadati Thermodesulfobacteriota Desulfobacteria Desulfobacterales Desulfobacterales bacterium isolate DOLJORAL78 47 17 PIE66404.1
Pseudomonadati Thermodesulfobacteriota Desulfobulbia Desulfobulbales Desulfogranum marinum WP 205220585.1
Pseudomonadati Thermodesulfobacteriota Desulfobulbia Desulfobulbales Desulfogranum aggregans strain JCM 19994 BGS86552.1
Pseudomonadati Thermodesulfobacteriota Desulfobulbia Desulfobulbales Desulfobulbus sp. isolate SBF912LR0FQ0009 MGI6657264.1
Pseudomonadati Thermodesulfobacteriota Desulfobulbia Desulfobulbales Desulfobulbus oralis WP 104937143.1
Pseudomonadati Thermodesulfobacteriota Desulfobulbia Desulfobulbales Desulfobulbus sp. GCF 031547805.1 WP 310599333.1
Pseudomonadati Thermodesulfobacteriota Desulfobulbia Desulfobulbales Desulfobulbus rhabdoformis WP 205228499.1
Pseudomonadati Thermodesulfobacteriota Desulfobulbia Desulfobulbales uncultured Desulfobulbus sp. GCF 937872135.1 WP 297954582.1
Pseudomonadati Thermodesulfobacteriota Desulfobulbia Desulfobulbales Desulfobulbus alkaliphilus WP 205241670.1
Pseudomonadati Thermodesulfobacteriota Desulfobulbia Desulfobulbales Desulfopila sp. isolate S6 bin.40.strict MFN2353261.1
Pseudomonadati Thermodesulfobacteriota Desulfobulbia Desulfobulbales Desulfopila sp. isolate bin.795 MEE4242235.1
Pseudomonadati Thermodesulfobacteriota Desulfobulbia Desulfobulbales Desulfocapsaceae bacterium isolate MO 192.B14 MDJ0621376.1
Pseudomonadati Thermodesulfobacteriota Desulfobulbia Desulfobulbales Desulfopila sp. isolate SMAG U9206 HKJ66220.1
Pseudomonadati Thermodesulfobacteriota Desulfobulbia Desulfobulbales Desulfopila inferna WP 205234906.1
Pseudomonadati Thermodesulfobacteriota Desulfobulbia Desulfobulbales Candidatus Desulfatifera sulfidica MBC8208472.1
Pseudomonadati Thermodesulfobacteriota Desulfobacteria Desulfobacterales Desulfoarculaceae bacterium isolate KA05 MBW6519496.1
Pseudomonadati Thermodesulfobacteriota unclassified Thermodesulfobacteriota Thermodesulfobacteriota bacterium isolate MAG013 MEN8188590.1
Pseudomonadati Thermodesulfobacteriota Desulfobulbia Desulfobulbales Desulfocapsa sulfexigens WP 015404718.1
Pseudomonadati Thermodesulfobacteriota Desulfobulbia Desulfobulbales Desulfocapsa sp. isolate MAG 30 RUM43995.1
Pseudomonadati Thermodesulfobacteriota Desulfobulbia Desulfobulbales Desulfocapsaceae bacterium isolate MAG CA64 MDR3630035.1
Pseudomonadati Thermodesulfobacteriota Desulfobulbia Desulfobulbales Desulfobulbaceae bacterium isolate UBA8130 HBT96479.1
Pseudomonadati Thermodesulfobacteriota Desulfobulbia Desulfobulbales Desulfobulbaceae bacterium isolate Rs511 bin14 MDR3088681.1
Pseudomonadati Thermodesulfobacteriota Desulfobulbia Desulfobulbales Desulfobulbaceae bacterium isolate Crp329 bin2 MDR0476694.1
Pseudomonadati Thermodesulfobacteriota Desulfobulbia Desulfobulbales Desulfoprunum sp. isolate C5 metabat.bin.56 MDP3480351.1
Pseudomonadati Thermodesulfobacteriota Desulfobulbia Desulfobulbales Desulfoprunum benzoelyticum WP 183351594.1
Pseudomonadati Thermodesulfobacteriota Desulfobulbia Desulfobulbales Desulfocapsaceae bacterium isolate bin.691 MEE4166420.1
Pseudomonadati Thermodesulfobacteriota Desulfobulbia Desulfobulbales Desulfofustis sp. PB SRB1 MBL0379157.1
Pseudomonadati Thermodesulfobacteriota Desulfobulbia Desulfobulbales Desulfofustis limnaeus WP 354005687.1
Pseudomonadati Thermodesulfobacteriota Desulfobulbia Desulfobulbales Desulfocapsaceae bacterium isolate MFD01352 ilm asm bin7 MGA7278241.1
Pseudomonadati Thermodesulfobacteriota Desulfobulbia Desulfobulbales Desulfofustis sp. isolate Spart 067 MAG MEJ2137252.1
Pseudomonadati Thermodesulfobacteriota Desulfobulbia Desulfobulbales Desulfotalea sp. isolate tPB Delt2 MGL1930135.1
Pseudomonadati Thermodesulfobacteriota Desulfobulbia Desulfobulbales Desulfotalea psychrophila WP 011189975.1
Pseudomonadati Thermodesulfobacteriota Desulfobulbia Desulfobulbales Desulfopila sp. IMCC35008 WP 163339624.1
Pseudomonadati Thermodesulfobacteriota Desulfobulbia Desulfobulbales Desulfosediminicola flagellatus WP 136805421.1
Pseudomonadati Thermodesulfobacteriota Desulfobulbia Desulfobulbales Desulfopila sp. isolate VLC 059 MFV0438659.1
Pseudomonadati Thermodesulfobacteriota Desulfobulbia Desulfobulbales Desulfopila aestuarii WP 073614954.1
Pseudomonadati Thermodesulfobacteriota Desulfobulbia Desulfobulbales Desulfobulbaceae bacterium isolate RS 2 19 MCK5516803.1
Pseudomonadati Thermodesulfobacteriota Desulfobulbia Desulfobulbales Desulfomarina profundi WP 228856347.1
Pseudomonadati Thermodesulfobacteriota Desulfobulbia Desulfobulbales Desulforhopalus sp. isolate SMAG U16051 HSL39018.1
Pseudomonadati Thermodesulfobacteriota Desulfobulbia Desulfobulbales Desulforhopalus vacuolatus WP 205219123.1
Pseudomonadati Thermodesulfobacteriota Desulfobulbia Desulfobulbales Desulforhopalus singaporensis WP 176761103.1
Pseudomonadati Thermodesulfobacteriota Desulfobulbia Desulfobulbales Desulforhopalus sp. isolate MFD12129 ilm asm bin8 MGB3223801.1
Pseudomonadati Thermodesulfobacteriota Desulfobulbia Desulfobulbales Desulfopila sp. IMCC35006 WP 136811386.1
Pseudomonadati Thermodesulfobacteriota Desulfobulbia Desulfobulbales Desulforhopalus sp. isolate EBRS2.bin23 MFW2364782.1
Pseudomonadati Thermodesulfobacteriota Desulfobulbia Desulfobulbales Desulforhopalus sp. isolate 3300055726 39 MFT5726421.1
Pseudomonadati Thermodesulfobacteriota Desulfobulbia Desulfobulbales Desulforhopalus sp. IMCC35007 WP 136872363.1
Pseudomonadati Thermodesulfobacteriota Candidatus Desulfofervidia Candidatus Desulfofervidaceae bacterium isolate AUK281 MCD6320292.1
Pseudomonadati Thermodesulfobacteriota Candidatus Desulfofervidia Candidatus Desulfofervidus auxilia strain HS1 WP 066063932.1
Pseudomonadati Thermodesulfobacteriota Candidatus Desulfofervidia Candidatus Desulfofervidus auxilia isolate PB NA091.008 bin6 MDL1957176.1
Pseudomonadati Thermodesulfobacteriota Candidatus Desulfofervidia Candidatus Desulfofervidus auxilia isolate HyVt 113 HDD35524.1

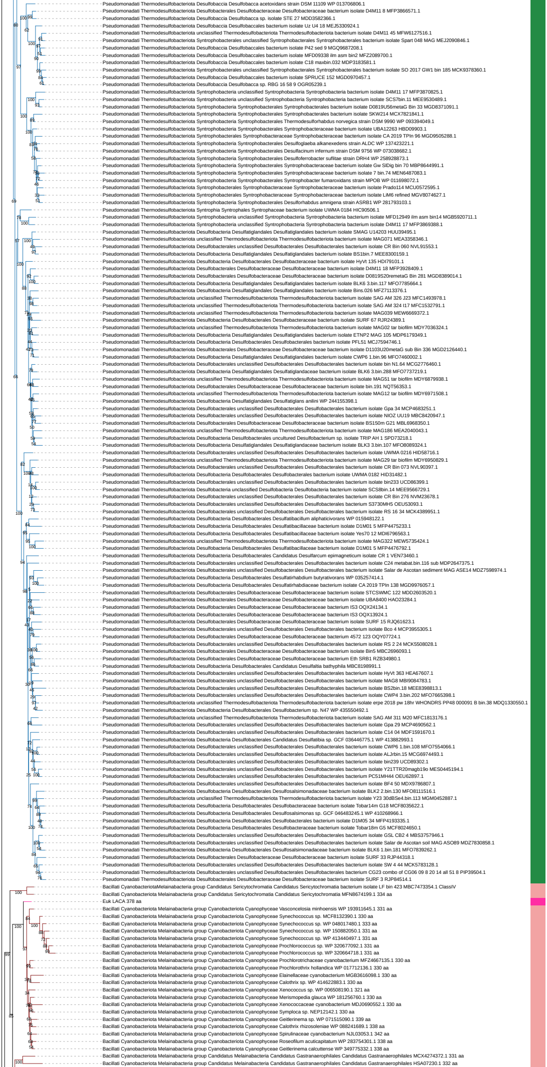

Pseudomonadati Thermodesulfobacteriota Desulfobaccia Desulfobacca acetoxidans strain DSM 11109 WP 013706806.1
Pseudomonadati Thermodesulfobacteriota Desulfobacterales Desulfobacteraceae Desulfobacteraceae bacterium isolate D4M11 8 MFP3866571.1
Pseudomonadati Thermodesulfobacteriota Desulfobaccia Desulfobacca sp. isolate STE 27 MDD3582366.1
Pseudomonadati Thermodesulfobacteriota Desulfobaccia Desulfobaccales bacterium isolate Uz U4 18 MEJ5330924.1
Pseudomonadati Thermodesulfobacteriota unclassified Thermodesulfobacteriota Thermodesulfobacteriota bacterium isolate D4M11 45 MFW6127516.1
Pseudomonadati Thermodesulfobacteriota Syntrophobacterales unclassified Syntrophobacterales Syntrophobacterales bacterium isolate Spart 048 MAG MEJ2090846.1
Pseudomonadati Thermodesulfobacteriota Desulfobaccia Desulfobaccales Desulfobaccales bacterium isolate P42 sed 9 MGQ9687208.1
Pseudomonadati Thermodesulfobacteriota Desulfobaccia Desulfobaccales Desulfobaccales bacterium isolate MFD09338 ilm asm bin2 MFZ2089700.1
Pseudomonadati Thermodesulfobacteriota Desulfobaccia Desulfobaccales Desulfobaccales bacterium isolate C18 maxbin.032 MDP3183581.1
Pseudomonadati Thermodesulfobacteriota Syntrophobacterales unclassified Syntrophobacterales Syntrophobacterales bacterium isolate SO 2017 GW1 bin 185 MCK9378360.1
Pseudomonadati Thermodesulfobacteriota Desulfobaccia Desulfobaccales Desulfobaccales bacterium isolate SPRUCE 152 MGD0970457.1
Pseudomonadati Thermodesulfobacteriota Desulfobaccia Desulfobacca Desulfobacca sp. RBG 16 58 9 OGR05239.1
Pseudomonadati Thermodesulfobacteriota Syntrophobacteria unclassified Syntrophobacteria Syntrophobacteria bacterium isolate D4M11 17 MFP3870825.1
Pseudomonadati Thermodesulfobacteriota Syntrophobacteria unclassified Syntrophobacteria Syntrophobacteria bacterium isolate SCS7bin.11 MEE9530489.1
Pseudomonadati Thermodesulfobacteriota Syntrophobacteria Syntrophobacterales Syntrophobacterales bacterium isolate D0819U56metaG Bin 33 MGD8371091.1
Pseudomonadati Thermodesulfobacteriota Syntrophobacteria Syntrophobacterales Syntrophobacterales bacterium isolate SKW214 MCX7821841.1
Pseudomonadati Thermodesulfobacteriota Syntrophobacteria Syntrophobacterales Thermodesulforhabdus norvegica strain DSM 9990 WP 093394049.1
Pseudomonadati Thermodesulfobacteriota Syntrophobacteria Syntrophobacterales Syntrophobacteraceae bacterium isolate UBA12263 HBD09903.1
Pseudomonadati Thermodesulfobacteriota Syntrophobacterales Syntrophobacteraceae Syntrophobacteraceae bacterium isolate CA 2019 TPIn 96 MGD9505288.1
Pseudomonadati Thermodesulfobacteriota Syntrophobacteria Syntrophobacterales Desulfoglaeba alkanexedens strain ALDC WP 137423221.1
Pseudomonadati Thermodesulfobacteriota Syntrophobacteria Syntrophobacterales Desulfacinum infernum strain DSM 9756 WP 073038682.1
Pseudomonadati Thermodesulfobacteriota Syntrophobacteria Syntrophobacterales Desulfoferrobacter suflitae strain DRH4 WP 258928873.1
Pseudomonadati Thermodesulfobacteriota Syntrophobacteria Syntrophobacterales Syntrophobacteraceae bacterium isolate Gw SIDig bin 70 MBP8644991.1
Pseudomonadati Thermodesulfobacteriota Syntrophobacteria Syntrophobacterales Syntrophobacteraceae bacterium isolate 7 bin.74 MEN6487083.1
Pseudomonadati Thermodesulfobacteriota Syntrophobacteria Syntrophobacterales Syntrophobacter fumaroxidans strain MPOB WP 011698072.1
Pseudomonadati Thermodesulfobacteriota Syntrophobacterales Syntrophobacteraceae Syntrophobacteraceae bacterium isolate Prado114 MCU0572595.1
Pseudomonadati Thermodesulfobacteriota Syntrophobacterales Syntrophobacteraceae Syntrophobacteraceae bacterium isolate LiM6 refined MGV8074627.1
Pseudomonadati Thermodesulfobacteriota Syntrophobacteria Syntrophobacterales Desulforhabdus amnigena strain ASRB1 WP 281793103.1
Pseudomonadati Thermodesulfobacteriota Syntrophia Syntrophales Syntrophaceae bacterium isolate UWMA 0184 HIC90506.1
Pseudomonadati Thermodesulfobacteriota Syntrophobacteria unclassified Syntrophobacteria Syntrophobacteria bacterium isolate MFD12949 ilm asm bin14 MGB5920711.1
Pseudomonadati Thermodesulfobacteriota Syntrophobacteria unclassified Syntrophobacteria Syntrophobacteria bacterium isolate D4M11 17 MFP3869388.1
Pseudomonadati Thermodesulfobacteriota Desulfobacteria Desulfatiglandales Desulfatiglandales bacterium isolate SMAG U14203 HUU39495.1
Pseudomonadati Thermodesulfobacteriota unclassified Thermodesulfobacteriota Thermodesulfobacteriota bacterium isolate MAG071 MEA3358346.1
Pseudomonadati Thermodesulfobacteriota Desulfobacterales unclassified Desulfobacterales Desulfobacterales bacterium isolate CR Bin 060 NVL91553.1
Pseudomonadati Thermodesulfobacteriota Desulfobacteria Desulfatiglandales Desulfatiglandales bacterium isolate BS1bin.7 MEE8300159.1
Pseudomonadati Thermodesulfobacteriota Desulfobacteria Desulfobacterales Desulfobacteraceae bacterium isolate HyVt 135 HDI79101.1
Pseudomonadati Thermodesulfobacteriota Desulfobacteria Desulfobacterales Desulfobacteraceae bacterium isolate D4M11 18 MFP3928409.1
Pseudomonadati Thermodesulfobacteriota Desulfobacterales Desulfobacteraceae Desulfobacteraceae bacterium isolate D0819S20remetaG Bin 281 MGD8389014.1
Pseudomonadati Thermodesulfobacteriota Desulfobacteria Desulfatiglandales Desulfatiglandales bacterium isolate BLK6 3.bin.117 MFO7785664.1
Pseudomonadati Thermodesulfobacteriota Desulfobacteria Desulfatiglandales Desulfatiglandales bacterium isolate Bins.026 MFZ7113376.1
Pseudomonadati Thermodesulfobacteriota unclassified Thermodesulfobacteriota Thermodesulfobacteriota bacterium isolate SAG AM 326 J23 MFC1493978.1
Pseudomonadati Thermodesulfobacteriota unclassified Thermodesulfobacteriota Thermodesulfobacteriota bacterium isolate SAG AM 324 I17 MFC1532791.1
Pseudomonadati Thermodesulfobacteriota unclassified Thermodesulfobacteriota Thermodesulfobacteriota bacterium isolate MAG039 MEW6669372.1
Pseudomonadati Thermodesulfobacteriota Desulfobacteria Desulfobacterales Desulfobacteraceae bacterium isolate SURF 67 RJR24389.1
Pseudomonadati Thermodesulfobacteriota unclassified Thermodesulfobacteriota Thermodesulfobacteriota bacterium isolate MAG02 tar biofilm MDY7036324.1
Pseudomonadati Thermodesulfobacteriota Desulfobacteria Desulfatiglandales Desulfatiglandales bacterium isolate ETNP2 MAG 105 MDP6179349.1
Pseudomonadati Thermodesulfobacteriota Desulfobacteria Desulfobacterales Desulfobacteraceae bacterium isolate PFL51 MCJ7594746.1
Pseudomonadati Thermodesulfobacteriota Desulfobacterales Desulfobacteraceae Desulfobacteraceae bacterium isolate D1103U20metaG sub Bin 336 MGD2126440.1
Pseudomonadati Thermodesulfobacteriota Desulfobacteria Desulfatiglandales Desulfatiglandales bacterium isolate CWP6 1.bin.96 MFO7460002.1
Pseudomonadati Thermodesulfobacteriota Desulfobacterales unclassified Desulfobacterales Desulfobacterales bacterium isolate bin N1.64 MCG2776460.1
Pseudomonadati Thermodesulfobacteriota Desulfobacteria Desulfatiglandales Desulfatiglandaceae bacterium isolate BLK6 3.bin.288 MFO7737219.1
Pseudomonadati Thermodesulfobacteriota unclassified Thermodesulfobacteriota Thermodesulfobacteriota bacterium isolate MAG51 tar biofilm MDY6879938.1
Pseudomonadati Thermodesulfobacteriota Desulfobacterales Desulfobacteraceae Desulfobacteraceae bacterium isolate bin.191 NQT56353.1
Pseudomonadati Thermodesulfobacteriota unclassified Thermodesulfobacteriota Thermodesulfobacteriota bacterium isolate MAG12 tar biofilm MDY6971508.1
Pseudomonadati Thermodesulfobacteriota Desulfobacteria Desulfatiglandales Desulfatiglans anilini WP 244155398.1
Pseudomonadati Thermodesulfobacteriota Desulfobacterales unclassified Desulfobacterales Desulfobacterales bacterium isolate Gpa 34 MCP4683251.1
Pseudomonadati Thermodesulfobacteriota Desulfobacterales unclassified Desulfobacterales Desulfobacterales bacterium isolate NIOZ UU19 MBC8420947.1
Pseudomonadati Thermodesulfobacteriota Desulfobacterales Desulfobacteraceae Desulfobacteraceae bacterium isolate BS150m G21 MBL6968350.1
Pseudomonadati Thermodesulfobacteriota unclassified Thermodesulfobacteriota Thermodesulfobacteriota bacterium isolate MAG186 MEA2040043.1
Pseudomonadati Thermodesulfobacteriota Desulfobacteria Desulfobacterales uncultured Desulfobacterium sp. isolate TRIP AH 1 SPD73218.1
Pseudomonadati Thermodesulfobacteriota Desulfobacteria Desulfatiglandales Desulfatiglandaceae bacterium isolate BLK3 3.bin.107 MFO8089324.1
Pseudomonadati Thermodesulfobacteriota Desulfobacterales unclassified Desulfobacterales Desulfobacterales bacterium isolate UWMA 0216 HID58716.1
Pseudomonadati Thermodesulfobacteriota unclassified Thermodesulfobacteriota Thermodesulfobacteriota bacterium isolate MAG29 tar biofilm MDY6950829.1
Pseudomonadati Thermodesulfobacteriota Desulfobacterales unclassified Desulfobacterales Desulfobacterales bacterium isolate CR Bin 073 NVL90397.1
Pseudomonadati Thermodesulfobacteriota Desulfobacteria Desulfobacterales Desulfobacterales bacterium isolate UWMA 0182 HID31482.1
Pseudomonadati Thermodesulfobacteriota Desulfobacterales unclassified Desulfobacterales Desulfobacterales bacterium isolate bin233 UCD86399.1
Pseudomonadati Thermodesulfobacteriota Desulfobacteria unclassified Desulfobacteria Desulfobacteria bacterium isolate SCS8bin.14 MEE9566729.1
Pseudomonadati Thermodesulfobacteriota Desulfobacterales unclassified Desulfobacterales Desulfobacterales bacterium isolate CR Bin 276 NVM23678.1
Pseudomonadati Thermodesulfobacteriota Desulfobacterales unclassified Desulfobacterales Desulfobacterales bacterium S3730MH5 OEU53093.1
Pseudomonadati Thermodesulfobacteriota Desulfobacterales unclassified Desulfobacterales Desulfobacterales bacterium isolate RS 16 34 MCK4389951.1
Pseudomonadati Thermodesulfobacteriota Desulfobacteria Desulfobacterales Desulfatibacillum aliphaticivorans WP 015948122.1
Pseudomonadati Thermodesulfobacteriota Desulfobacteria Desulfobacterales Desulfatibacillaceae bacterium isolate D1M01 5 MFP4475233.1
Pseudomonadati Thermodesulfobacteriota Desulfobacteria Desulfobacterales Desulfatibacillaceae bacterium isolate Yes70 12 MDI6796563.1
Pseudomonadati Thermodesulfobacteriota unclassified Thermodesulfobacteriota Thermodesulfobacteriota bacterium isolate MAG322 MEW5735424.1
Pseudomonadati Thermodesulfobacteriota Desulfobacteria Desulfobacterales Desulfatibacillaceae bacterium isolate D1M01 5 MFP4476792.1
Pseudomonadati Thermodesulfobacteriota Desulfobacteria Desulfobacterales Candidatus Desulfarcum epimagneticum isolate CR 1 VEN73460.1
Pseudomonadati Thermodesulfobacteriota Desulfobacterales unclassified Desulfobacterales Desulfobacterales bacterium isolate C24 metabat.bin.116 sub MDP2647375.1
Pseudomonadati Thermodesulfobacteriota Desulfobacterales unclassified Desulfobacterales Desulfobacterales bacterium isolate Salar de Ascotan sediment MAG ASE14 MDZ7598974.1
Pseudomonadati Thermodesulfobacteriota Desulfobacteria Desulfobacterales Desulfatirhabdium butyrativorans WP 035257414.1
Pseudomonadati Thermodesulfobacteriota Desulfobacteria Desulfobacterales Desulfatirhabdiaceae bacterium isolate CA 2019 TPIn 138 MGD9976057.1
Pseudomonadati Thermodesulfobacteriota Desulfobacteria Desulfobacterales Desulfobacteraceae bacterium isolate STCSWMC 122 MDD2603520.1
Pseudomonadati Thermodesulfobacteriota Desulfobacterales Desulfobacteraceae Desulfobacteraceae bacterium isolate UBA8400 HAO23284.1
Pseudomonadati Thermodesulfobacteriota Desulfobacterales Desulfobacteraceae Desulfobacteraceae bacterium IS3 OQX24134.1
Pseudomonadati Thermodesulfobacteriota Desulfobacterales Desulfobacteraceae Desulfobacteraceae bacterium IS3 OQX13924.1
Pseudomonadati Thermodesulfobacteriota Desulfobacteria Desulfobacterales Desulfobacteraceae bacterium isolate SURF 15 RJQ61623.1
Pseudomonadati Thermodesulfobacteriota Desulfobacterales unclassified Desulfobacterales Desulfobacterales bacterium isolate Bco 4 MCP3955305.1
Pseudomonadati Thermodesulfobacteriota Desulfobacterales Desulfobacteraceae Desulfobacteraceae bacterium 4572 123 OQY07724.1
Pseudomonadati Thermodesulfobacteriota Desulfobacterales unclassified Desulfobacterales Desulfobacterales bacterium isolate RS 2 24 MCK5508028.1
Pseudomonadati Thermodesulfobacteriota Desulfobacteria Desulfobacterales Desulfobacteraceae bacterium isolate Bin5 MBC2696093.1
Pseudomonadati Thermodesulfobacteriota Desulfobacterales Desulfobacteraceae Desulfobacteraceae bacterium Eth SRB1 RZB34980.1
Pseudomonadati Thermodesulfobacteriota Desulfobacteria Desulfobacterales Candidatus Desulfaltia bathyphila MBC8198991.1
Pseudomonadati Thermodesulfobacteriota Desulfobacterales unclassified Desulfobacterales Desulfobacterales bacterium isolate HyVt 363 HEA67607.1
Pseudomonadati Thermodesulfobacteriota Desulfobacterales unclassified Desulfobacterales Desulfobacterales bacterium isolate MAG8 MBI9084783.1
Pseudomonadati Thermodesulfobacteriota Desulfobacterales unclassified Desulfobacterales Desulfobacterales bacterium isolate BS2bin.18 MEE8398813.1
Pseudomonadati Thermodesulfobacteriota Desulfobacterales unclassified Desulfobacterales Desulfobacterales bacterium isolate CWP4 3.bin.202 MFO7665398.1
Pseudomonadati Thermodesulfobacteriota unclassified Thermodesulfobacteriota Thermodesulfobacteriota bacterium isolate erpe 2018 pw 18hr WHONDRS PP48 000091 B bin.38 MDQ1330550.1
Pseudomonadati Thermodesulfobacteriota Desulfobacteria Desulfobacterales Desulfobacterium sp. N47 WP 435550492.1
Pseudomonadati Thermodesulfobacteriota unclassified Thermodesulfobacteriota Thermodesulfobacteriota bacterium isolate SAG AM 311 M20 MFC1813176.1
Pseudomonadati Thermodesulfobacteriota Desulfobacterales unclassified Desulfobacterales Desulfobacterales bacterium isolate Gpa 29 MCP4690562.1
Pseudomonadati Thermodesulfobacteriota Desulfobacterales unclassified Desulfobacterales Desulfobacterales bacterium isolate C14 04 MDF1591670.1
Pseudomonadati Thermodesulfobacteriota Desulfobacteria Desulfobacterales Candidatus Desulfatibia sp. GCF 036446775.1 WP 413882993.1
Pseudomonadati Thermodesulfobacteriota Desulfobacterales unclassified Desulfobacterales Desulfobacterales bacterium isolate CWP6 1.bin.108 MFO7554066.1
Pseudomonadati Thermodesulfobacteriota Desulfobacterales unclassified Desulfobacterales Desulfobacterales bacterium isolate ALJrbin.15 MCG6974493.1
Pseudomonadati Thermodesulfobacteriota Desulfobacterales unclassified Desulfobacterales Desulfobacterales bacterium isolate bin239 UCD89302.1
Pseudomonadati Thermodesulfobacteriota Desulfobacterales unclassified Desulfobacterales Desulfobacterales bacterium isolate Y21TTR20magb19o MES0445194.1
Pseudomonadati Thermodesulfobacteriota Desulfobacterales unclassified Desulfobacterales Desulfobacterales bacterium PC51MH44 OEU62897.1
Pseudomonadati Thermodesulfobacteriota Desulfobacterales unclassified Desulfobacterales Desulfobacterales bacterium isolate BF4 50 MDX9786807.1
Pseudomonadati Thermodesulfobacteriota Desulfobacteria Desulfobacterales Desulfosalsimonadaceae bacterium isolate BLK2 2.bin.130 MFO8111516.1
Pseudomonadati Thermodesulfobacteriota unclassified Thermodesulfobacteriota Thermodesulfobacteriota bacterium isolate Y23 30dBSe4.bin.113 MGM0452887.1
Pseudomonadati Thermodesulfobacteriota Desulfobacteria Desulfobacterales Desulfobacteraceae bacterium isolate Tobar14m G18 MCF8035622.1
Pseudomonadati Thermodesulfobacteriota Desulfobacteria Desulfobacterales Desulfosalsimonas sp. GCF 046483245.1 WP 410268966.1
Pseudomonadati Thermodesulfobacteriota Desulfobacteria Desulfobacterales Desulfobacterales bacterium isolate D1M05 34 MFP4193335.1
Pseudomonadati Thermodesulfobacteriota Desulfobacteria Desulfobacterales Desulfobacteraceae bacterium isolate Tobar18m G5 MCF8024650.1
Pseudomonadati Thermodesulfobacteriota Desulfobacterales unclassified Desulfobacterales Desulfobacterales bacterium isolate GSL CB2 4 MBS3757946.1
Pseudomonadati Thermodesulfobacteriota Desulfobacterales unclassified Desulfobacterales Desulfobacterales bacterium isolate Salar de Ascotan soil MAG ASO89 MDZ7830858.1
Pseudomonadati Thermodesulfobacteriota Desulfobacteria Desulfobacterales Desulfosalsimonadaceae bacterium isolate BLK6 1.bin.181 MFO7839262.1
Pseudomonadati Thermodesulfobacteriota Desulfobacteria Desulfobacterales Desulfobacteraceae bacterium isolate SURF 33 RJP44318.1
Pseudomonadati Thermodesulfobacteriota Desulfobacterales unclassified Desulfobacterales Desulfobacterales bacterium isolate SW 4 44 MCK5783128.1
Pseudomonadati Thermodesulfobacteriota Desulfobacterales unclassified Desulfobacterales Desulfobacterales bacterium isolate CG23 combo of CG06 09 8 20 14 all 51 8 PIP39504.1
Pseudomonadati Thermodesulfobacteriota Desulfobacteria Desulfobacterales Desulfobacteraceae bacterium isolate SURF 3 RJP84514.1
Bacillati CyanobacteriotaMelainabacteria group Candidatus Sericytochromatia Candidatus Sericytochromatia bacterium isolate LF bin 423 MBC7473354.1 ClassIV
Bacillati Cyanobacteriota Melainabacteria group Candidatus Sericytochromatia Candidatus Sericytochromatia MFN8674199.1 334 aa
Euk LACA 378 aa
Bacillati Cyanobacteriota Melainabacteria group Cyanobacteriota Cyanophyceae Vasconcelosia minhoensis WP 193911645.1 331 aa
Bacillati Cyanobacteriota Melainabacteria group Cyanobacteriota Cyanophyceae Synechococcus sp. MCF8132390.1 330 aa
Bacillati Cyanobacteriota Melainabacteria group Cyanobacteriota Cyanophyceae Synechococcus sp. WP 048017480.1 333 aa
Bacillati Cyanobacteriota Melainabacteria group Cyanobacteriota Cyanophyceae Synechococcus sp. WP 150882050.1 331 aa
Bacillati Cyanobacteriota Melainabacteria group Cyanobacteriota Cyanophyceae Synechococcus sp. WP 413440497.1 331 aa
Bacillati Cyanobacteriota Melainabacteria group Cyanobacteriota Cyanophyceae Prochlorococcus sp. WP 320677092.1 331 aa
Bacillati Cyanobacteriota Melainabacteria group Cyanobacteriota Cyanophyceae Prochlorococcus sp. WP 320664718.1 331 aa
Bacillati Cyanobacteriota Melainabacteria group Cyanobacteriota Cyanophyceae Prochlorotrichaceae cyanobacterium MFZ4667135.1 330 aa
Bacillati Cyanobacteriota Melainabacteria group Cyanobacteriota Cyanophyceae Prochlorothrix hollandica WP 017712136.1 330 aa
Bacillati Cyanobacteriota Melainabacteria group Cyanobacteriota Cyanophyceae Elainellaceae cyanobacterium MGB3616098.1 330 aa
Bacillati Cyanobacteriota Melainabacteria group Cyanobacteriota Cyanophyceae Calothrix sp. WP 414622883.1 330 aa
Bacillati Cyanobacteriota Melainabacteria group Cyanobacteriota Cyanophyceae Xenococcus sp. WP 006508190.1 321 aa
Bacillati Cyanobacteriota Melainabacteria group Cyanobacteriota Cyanophyceae Merismopedia glauca WP 181256760.1 330 aa
Bacillati Cyanobacteriota Melainabacteria group Cyanobacteriota Cyanophyceae Xenococcaceae cyanobacterium MDJ0690552.1 330 aa
Bacillati Cyanobacteriota Melainabacteria group Cyanobacteriota Cyanophyceae Symploca sp. NEP12142.1 330 aa
Bacillati Cyanobacteriota Melainabacteria group Cyanobacteriota Cyanophyceae Geitlerinema sp. WP 071515090.1 339 aa
Bacillati Cyanobacteriota Melainabacteria group Cyanobacteriota Cyanophyceae Calothrix rhizosoleniae WP 088241689.1 338 aa
Bacillati Cyanobacteriota Melainabacteria group Cyanobacteriota Cyanophyceae Spirulinaceae cyanobacterium NJL03053.1 342 aa
Bacillati Cyanobacteriota Melainabacteria group Cyanobacteriota Cyanophyceae Roseofilum acuticapitatum WP 283754301.1 338 aa
Bacillati Cyanobacteriota Melainabacteria group Cyanobacteriota Cyanophyceae Geitlerinema calcuttense WP 349775332.1 338 aa
Bacillati Cyanobacteriota Melainabacteria group Candidatus Melainabacteria Candidatus Gastranaerophilales Candidatus Gastranaerophilales MCX4274372.1 331 aa
Bacillati Cyanobacteriota Melainabacteria group Candidatus Melainabacteria Candidatus Gastranaerophilales Candidatus Gastranaerophilales HSA07230.1 332 aa

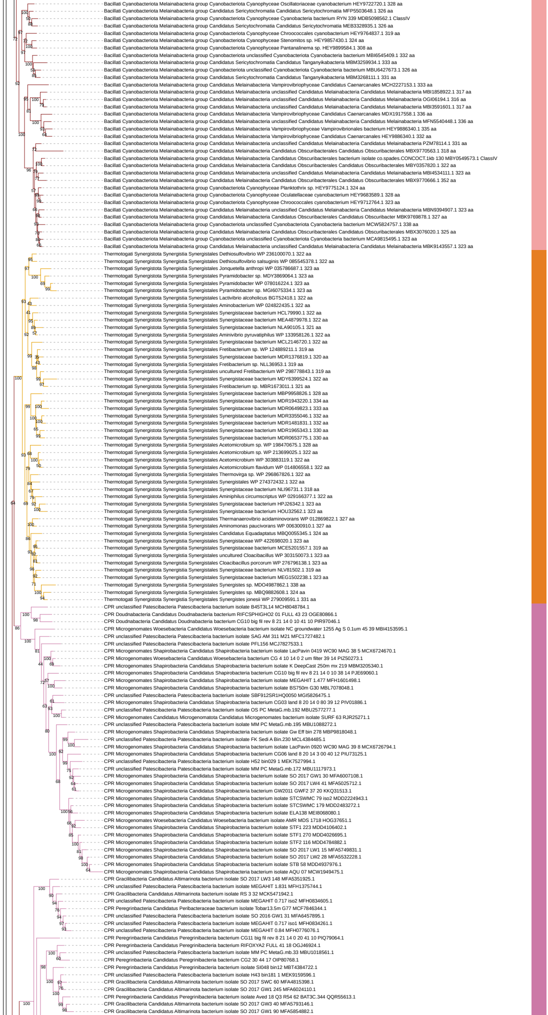

Bacillati Cyanobacteriota Melainabacteria group Cyanobacteriota Cyanophyceae Oscillatoriaceae cyanobacterium HEY9722720.1 328 aa
Bacillati Cyanobacteriota Melainabacteria group Candidatus Sericytochromatia Candidatus Sericytochromatia MFP5503648.1 326 aa
Bacillati Cyanobacteriota Melainabacteria group Cyanobacteriota Cyanophyceae Cyanobacteria bacterium RYN 339 MDB5098562.1 ClassIV
Bacillati Cyanobacteriota Melainabacteria group Candidatus Sericytochromatia Candidatus Sericytochromatia MEB3328935.1 326 aa
Bacillati Cyanobacteriota Melainabacteria group Cyanobacteriota Cyanophyceae Chroococcales cyanobacterium HEY9764837.1 319 aa
Bacillati Cyanobacteriota Melainabacteria group Cyanobacteriota Cyanophyceae Stenomitos sp. HEY9857430.1 324 aa
Bacillati Cyanobacteriota Melainabacteria group Cyanobacteriota Cyanophyceae Pantanalinema sp. HEY9899584.1 308 aa
Bacillati Cyanobacteriota Melainabacteria group Cyanobacteriota unclassified Cyanobacteriota Cyanobacteria bacterium MBI6545409.1 332 aa
Bacillati Cyanobacteriota Melainabacteria group Candidatus Sericytochromatia Candidatus Tanganyikabacteria MBM3259934.1 333 aa
Bacillati Cyanobacteriota Melainabacteria group Cyanobacteriota unclassified Cyanobacteriota Cyanobacteria bacterium MBU6427673.1 326 aa
Bacillati Cyanobacteriota Melainabacteria group Candidatus Sericytochromatia Candidatus Tanganyikabacteria MBM3268111.1 331 aa
Bacillati Cyanobacteriota Melainabacteria group Candidatus Melainabacteria Vampirovibriophyceae Candidatus Caenarcanales MCH2227153.1 333 aa
Bacillati Cyanobacteriota Melainabacteria group Candidatus Melainabacteria unclassified Candidatus Melainabacteria Candidatus Melainabacteria MBI1858922.1 317 aa
Bacillati Cyanobacteriota Melainabacteria group Candidatus Melainabacteria unclassified Candidatus Melainabacteria Candidatus Melainabacteria OGI06194.1 316 aa
Bacillati Cyanobacteriota Melainabacteria group Candidatus Melainabacteria unclassified Candidatus Melainabacteria Candidatus Melainabacteria MBI3591601.1 317 aa
Bacillati Cyanobacteriota Melainabacteria group Candidatus Melainabacteria Vampirovibriophyceae Candidatus Caenarcanales MDX1917558.1 336 aa
Bacillati Cyanobacteriota Melainabacteria group Candidatus Melainabacteria unclassified Candidatus Melainabacteria Candidatus Melainabacteria MFN5540448.1 336 aa
Bacillati Cyanobacteriota Melainabacteria group Candidatus Melainabacteria Vampirovibriophyceae Vampirovibrionales bacterium HEY9886340.1 335 aa
Bacillati Cyanobacteriota Melainabacteria group Candidatus Melainabacteria Vampirovibriophyceae Candidatus Caenarcanales HEY9886340.1 332 aa
Bacillati Cyanobacteriota Melainabacteria group Candidatus Melainabacteria unclassified Candidatus Melainabacteria Candidatus Melainabacteria PZM78114.1 331 aa
Bacillati Cyanobacteriota Melainabacteria group Candidatus Melainabacteria Candidatus Obscuribacterales Candidatus Obscuribacterales MBX9770563.1 318 aa
Bacillati Cyanobacteriota Melainabacteria group Candidatus Melainabacteria Candidatus Obscuribacterales Candidatus Obscuribacterales bacterium isolate co.spades.CONCOCT.1kb 130 MBY0549573.1 ClassIV
Bacillati Cyanobacteriota Melainabacteria group Candidatus Melainabacteria Candidatus Obscuribacterales Candidatus Obscuribacterales MBY0357820.1 322 aa
Bacillati Cyanobacteriota Melainabacteria group Candidatus Melainabacteria unclassified Candidatus Melainabacteria Candidatus Melainabacteria MBI4534111.1 323 aa
Bacillati Cyanobacteriota Melainabacteria group Candidatus Melainabacteria Candidatus Obscuribacterales Candidatus Obscuribacterales MBX9770666.1 352 aa
Bacillati Cyanobacteriota Melainabacteria group Cyanobacteriota Cyanophyceae Planktothrix sp. HEY9775124.1 324 aa
Bacillati Cyanobacteriota Melainabacteria group Cyanobacteriota Cyanophyceae Oculatellaceae cyanobacterium HEY9683589.1 328 aa
Bacillati Cyanobacteriota Melainabacteria group Cyanobacteriota Cyanophyceae Chroococcales cyanobacterium HEY9712764.1 323 aa
Bacillati Cyanobacteriota Melainabacteria group Candidatus Melainabacteria unclassified Candidatus Melainabacteria Candidatus Melainabacteria MBN9394907.1 323 aa
Bacillati Cyanobacteriota Melainabacteria group Candidatus Melainabacteria Candidatus Obscuribacterales Candidatus Obscuribacter MBK9769878.1 327 aa
Bacillati Cyanobacteriota Melainabacteria group Cyanobacteriota unclassified Cyanobacteriota Cyanobacteria bacterium MCW5824757.1 338 aa
Bacillati Cyanobacteriota Melainabacteria group Candidatus Melainabacteria Candidatus Obscuribacterales Candidatus Obscuribacterales MBX3076020.1 325 aa
Bacillati Cyanobacteriota Melainabacteria group Cyanobacteriota unclassified Cyanobacteriota Cyanobacteria bacterium MCA9815495.1 323 aa
Bacillati Cyanobacteriota Melainabacteria group Candidatus Melainabacteria unclassified Candidatus Melainabacteria Candidatus Melainabacteria MBK9143557.1 323 aa
Thermotogati Synergistota Synergistia Synergistales Dethiosulfovibrio WP 236100070.1 322 aa
Thermotogati Synergistota Synergistia Synergistales Dethiosulfovibrio salsuginis WP 085545378.1 322 aa
Thermotogati Synergistota Synergistia Synergistales Jonquetella anthropi WP 035786687.1 323 aa
Thermotogati Synergistota Synergistia Synergistales Pyramidobacter sp. MDY3869064.1 323 aa
Thermotogati Synergistota Synergistia Synergistales Pyramidobacter WP 078016224.1 323 aa
Thermotogati Synergistota Synergistia Synergistales Pyramidobacter sp. MGI6075334.1 323 aa
Thermotogati Synergistota Synergistia Synergistales Lactivibrio alcoholicus BGT52418.1 322 aa
Thermotogati Synergistota Synergistia Synergistales Aminobacterium WP 024822435.1 322 aa
Thermotogati Synergistota Synergistia Synergistales Synergistaceae bacterium HCL79990.1 322 aa
Thermotogati Synergistota Synergistia Synergistales Synergistaceae bacterium MEA4879978.1 322 aa
Thermotogati Synergistota Synergistia Synergistales Synergistaceae bacterium NLA90105.1 321 aa
Thermotogati Synergistota Synergistia Synergistales Aminivibrio pyruvatiphilus WP 133958126.1 322 aa
Thermotogati Synergistota Synergistia Synergistales Synergistaceae bacterium MCL2146720.1 322 aa
Thermotogati Synergistota Synergistia Synergistales Fretibacterium sp. WP 124889211.1 319 aa
Thermotogati Synergistota Synergistia Synergistales Synergistaceae bacterium MDR1376819.1 320 aa
Thermotogati Synergistota Synergistia Synergistales Fretibacterium sp. NLL36953.1 319 aa
Thermotogati Synergistota Synergistia Synergistales uncultured Fretibacterium WP 298778843.1 319 aa
Thermotogati Synergistota Synergistia Synergistales Synergistaceae bacterium MDY6399524.1 322 aa
Thermotogati Synergistota Synergistia Synergistales Fretibacterium sp. MBR1673011.1 321 aa
Thermotogati Synergistota Synergistia Synergistales Synergistaceae bacterium MBP9958826.1 328 aa
Thermotogati Synergistota Synergistia Synergistales Synergistaceae bacterium MDR1943220.1 334 aa
Thermotogati Synergistota Synergistia Synergistales Synergistaceae bacterium MDR0649823.1 333 aa
Thermotogati Synergistota Synergistia Synergistales Synergistaceae bacterium MDR3355046.1 332 aa
Thermotogati Synergistota Synergistia Synergistales Synergistaceae bacterium MDR1481831.1 332 aa
Thermotogati Synergistota Synergistia Synergistales Synergistaceae bacterium MDR1965343.1 330 aa
Thermotogati Synergistota Synergistia Synergistales Synergistaceae bacterium MDR0653775.1 330 aa
Thermotogati Synergistota Synergistia Synergistales Acetomicrobium sp. WP 198470675.1 328 aa
Thermotogati Synergistota Synergistia Synergistales Acetomicrobium sp. WP 213699025.1 322 aa
Thermotogati Synergistota Synergistia Synergistales Acetomicrobium WP 303883119.1 322 aa
Thermotogati Synergistota Synergistia Synergistales Acetomicrobium flavidum WP 014806558.1 322 aa
Thermotogati Synergistota Synergistia Synergistales Thermovirga sp. WP 296867826.1 322 aa
Thermotogati Synergistota Synergistia Synergistales Synergistales WP 274372432.1 322 aa
Thermotogati Synergistota Synergistia Synergistales Synergistaceae bacterium NLI96731.1 318 aa
Thermotogati Synergistota Synergistia Synergistales Aminiphilus circumscriptus WP 029166377.1 322 aa
Thermotogati Synergistota Synergistia Synergistales Synergistaceae bacterium HPJ26342.1 323 aa
Thermotogati Synergistota Synergistia Synergistales Synergistaceae bacterium HOU32562.1 323 aa
Thermotogati Synergistota Synergistia Synergistales Thermanaerovibrio acidaminovorans WP 012869822.1 327 aa
Thermotogati Synergistota Synergistia Synergistales Aminomonas paucivorans WP 006300910.1 327 aa
Thermotogati Synergistota Synergistia Synergistales Candidatus Equadaptatus MBQ0055345.1 324 aa
Thermotogati Synergistota Synergistia Synergistales Synergistaceae WP 422698020.1 323 aa
Thermotogati Synergistota Synergistia Synergistales Synergistaceae bacterium MCE5201557.1 319 aa
Thermotogati Synergistota Synergistia Synergistales uncultured Cloacibacillus WP 303150073.1 323 aa
Thermotogati Synergistota Synergistia Synergistales Cloacibacillus porcorum WP 276796138.1 323 aa
Thermotogati Synergistota Synergistia Synergistales Synergistaceae bacterium NLV81502.1 319 aa
Thermotogati Synergistota Synergistia Synergistales Synergistaceae bacterium MEG1502238.1 323 aa
Thermotogati Synergistota Synergistia Synergistales Synergistes sp. MDO4987862.1 338 aa
Thermotogati Synergistota Synergistia Synergistales Synergistes sp. MBQ9882608.1 324 aa
Thermotogati Synergistota Synergistia Synergistales Synergistes jonesii WP 279009591.1 331 aa
CPR unclassified Patescibacteria Patescibacteria bacterium isolate B45T3L14 MCH8048784.1
CPR Doudnabacteria Candidatus Doudnabacteria bacterium RIFCSPHIGHO2 01 FULL 43 23 OGE80866.1
CPR Doudnabacteria Candidatus Doudnabacteria bacterium CG10 big fil rev 8 21 14 0 10 41 10 PIR97046.1
CPR Microgenomates Woesebacteria Candidatus Woesebacteria bacterium isolate NC groundwater 1255 Ag S 0.1um 45 39 MBI4153595.1
CPR unclassified Patescibacteria Patescibacteria bacterium isolate SAG AM 311 M21 MFC1727482.1
CPR unclassified Patescibacteria Patescibacteria bacterium isolate PFL156 MCJ7827533.1
CPR Microgenomates Shapirobacteria Candidatus Shapirobacteria bacterium isolate LacPavin 0419 WC90 MAG 38 5 MCX6724670.1
CPR Microgenomates Woesebacteria Candidatus Woesebacteria bacterium CG 4 10 14 0 2 um filter 39 14 PIZ50273.1
CPR Microgenomates Shapirobacteria Candidatus Shapirobacteria bacterium isolate K DeepCast 250m mx 219 MBM3205340.1
CPR Microgenomates Shapirobacteria Candidatus Shapirobacteria bacterium CG10 big fil rev 8 21 14 0 10 38 14 PJE69060.1
CPR Microgenomates Shapirobacteria Candidatus Shapirobacteria bacterium isolate MEGAHIT 1.477 MFH1601498.1
CPR Microgenomates Shapirobacteria Candidatus Shapirobacteria bacterium isolate BS750m G30 MBL7078048.1
CPR unclassified Patescibacteria Patescibacteria bacterium isolate SBF912SR1HQ0050 MGI5826475.1
CPR Microgenomates Shapirobacteria Candidatus Shapirobacteria bacterium CG03 land 8 20 14 0 80 39 12 PIV01886.1
CPR unclassified Patescibacteria Patescibacteria bacterium isolate OS PC MetaG.mb.192 MBU2577277.1
CPR Microgenomates Candidatus Microgenomatota Candidatus Microgenomates bacterium isolate SURF 63 RJR25271.1
CPR unclassified Patescibacteria Patescibacteria bacterium isolate MM PC MetaG.mb.195 MBU1088272.1
CPR Microgenomates Shapirobacteria Candidatus Shapirobacteria bacterium isolate Gw Eff bin 278 MBP9818048.1
CPR unclassified Patescibacteria Patescibacteria bacterium isolate FK Sedi A Bin.230 MCL4384485.1
CPR Microgenomates Shapirobacteria Candidatus Shapirobacteria bacterium isolate LacPavin 0920 WC90 MAG 39 8 MCX6726794.1
CPR Microgenomates Shapirobacteria Candidatus Shapirobacteria bacterium CG06 land 8 20 14 3 00 40 12 PIU73125.1
CPR unclassified Patescibacteria Patescibacteria bacterium isolate H52 bin029 1 MEK7527994.1
CPR unclassified Patescibacteria Patescibacteria bacterium isolate MM PC MetaG.mb.172 MBU1117973.1
CPR Microgenomates Shapirobacteria Candidatus Shapirobacteria bacterium isolate SO 2017 GW1 30 MFA6007108.1
CPR Microgenomates Shapirobacteria Candidatus Shapirobacteria bacterium isolate SO 2017 LW4 41 MFA5025712.1
CPR Microgenomates Shapirobacteria Candidatus Shapirobacteria bacterium GW2011 GWF2 37 20 KKQ31513.1
CPR Microgenomates Shapirobacteria Candidatus Shapirobacteria bacterium isolate STCSWMC 79 iso2 MDD2224943.1
CPR Microgenomates Shapirobacteria Candidatus Shapirobacteria bacterium isolate STCSWMC 179 MDD2483272.1
CPR Microgenomates Shapirobacteria Candidatus Shapirobacteria bacterium isolate ELA138 MEI8068080.1
CPR Microgenomates Woesebacteria Candidatus Woesebacteria bacterium isolate AMR MDS 1718 HOG37651.1
CPR Microgenomates Shapirobacteria Candidatus Shapirobacteria bacterium isolate STF1 223 MDD4106402.1
CPR Microgenomates Shapirobacteria Candidatus Shapirobacteria bacterium isolate STF1 270 MDD4026695.1
CPR Microgenomates Shapirobacteria Candidatus Shapirobacteria bacterium isolate STF2 116 MDD4784882.1
CPR Microgenomates Shapirobacteria Candidatus Shapirobacteria bacterium isolate SO 2017 LW1 15 MFA5749831.1
CPR Microgenomates Shapirobacteria Candidatus Shapirobacteria bacterium isolate SO 2017 LW2 28 MFA5532228.1
CPR Microgenomates Shapirobacteria Candidatus Shapirobacteria bacterium isolate STB 58 MDD4937976.1
CPR Microgenomates Shapirobacteria Candidatus Shapirobacteria bacterium isolate AQU 07 MCW1949475.1
CPR Gracilibacteria Candidatus Altimarinota bacterium isolate SO 2017 LW3 148 MFA5351925.1
CPR unclassified Patescibacteria Patescibacteria bacterium isolate MEGAHIT 1.831 MFH1375744.1
CPR Gracilibacteria Candidatus Altimarinota bacterium isolate RS 3 32 MCK5471942.1
CPR unclassified Patescibacteria Patescibacteria bacterium isolate MEGAHIT 0.717 iso2 MFH0834605.1
CPR Peregrinibacteria Candidatus Peribacteraceae bacterium isolate Tobar13.5m G77 MCF7846344.1
CPR unclassified Patescibacteria Patescibacteria bacterium isolate SO 2016 GW1 31 MFA6457895.1
CPR unclassified Patescibacteria Patescibacteria bacterium isolate MEGAHIT 0.717 iso1 MFH0834261.1
CPR unclassified Patescibacteria Patescibacteria bacterium isolate MEGAHIT 0.84 MFH0776076.1
CPR Peregrinibacteria Candidatus Peregrinibacteria bacterium CG11 big fil rev 8 21 14 0 20 41 10 PIQ79064.1
CPR Peregrinibacteria Candidatus Peregrinibacteria bacterium RIFOXYA2 FULL 41 18 OGJ46924.1
CPR unclassified Patescibacteria Patescibacteria bacterium isolate MM PC MetaG.mb.33 MBU1018561.1
CPR Peregrinibacteria Candidatus Peregrinibacteria bacterium CG2 30 44 17 OIP80768.1
CPR Peregrinibacteria Candidatus Peregrinibacteria bacterium isolate SI048 bin12 MBT4384722.1
CPR unclassified Patescibacteria Patescibacteria bacterium isolate H43 bin181 1 MEK9159596.1
CPR Gracilibacteria Candidatus Altimarinota bacterium isolate SO 2017 SWC 60 MFA4815398.1
CPR Gracilibacteria Candidatus Altimarinota bacterium isolate SO 2017 GW1 245 MFA6024110.1
CPR Peregrinibacteria Candidatus Peregrinibacteria bacterium isolate Aved 18 Q3 R54 62 BAT3C.344 QQR55613.1
CPR Gracilibacteria Candidatus Altimarinota bacterium isolate SO 2017 GW3 40 MFA5793146.1
CPR Gracilibacteria Candidatus Altimarinota bacterium isolate SO 2017 GW1 90 MFA5854882.1

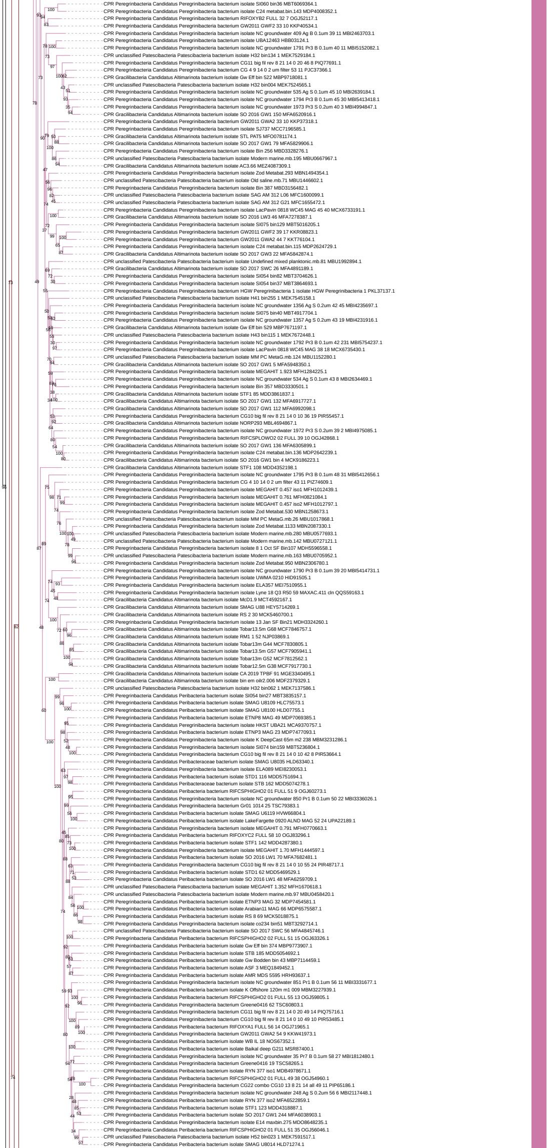
CPR Peregrinbacteria Candidatus Peregrinibacteria bacterium isolate SI060 bin36 MBT6069364.1
CPR Peregrinbacteria Candidatus Peregrinibacteria bacterium isolate C24 metabat.bin.143 MDP4008352.1
CPR Peregrinbacteria Candidatus Peregrinibacteria bacterium RIFOXYB2 FULL 32 7 OGJ52117.1
CPR Peregrinbacteria Candidatus Peregrinibacteria bacterium GW2011 GWF2 33 10 KKP40534.1
CPR Peregrinbacteria Candidatus Peregrinibacteria bacterium isolate NC groundwater 409 Ag B 0.1um 39 11 MBI2463703.1
CPR Peregrinbacteria Candidatus Peregrinibacteria bacterium isolate UBA12463 HBB03124.1
CPR Peregrinbacteria Candidatus Peregrinibacteria bacterium isolate NC groundwater 1791 Pr3 B 0.1um 40 11 MBI5152082.1
CPR unclassified Patescibacteria Patescibacteria bacterium isolate H32 bin134 1 MEK7529184.1
CPR Peregrinbacteria Candidatus Peregrinibacteria bacterium CG11 big fil rev 8 21 14 0 20 46 8 PIQ77691.1
CPR Peregrinbacteria Candidatus Peregrinibacteria bacterium CG 4 9 14 0 2 um filter 53 11 PJC37366.1
CPR Gracilibacteria Candidatus Altimarinota bacterium isolate Gw Eff bin 522 MBP9718081.1
CPR unclassified Patescibacteria Patescibacteria bacterium isolate H32 bin004 MEK7524565.1
CPR Peregrinbacteria Candidatus Peregrinibacteria bacterium isolate NC groundwater 535 Ag S 0.1um 45 10 MBI2639184.1
CPR Peregrinbacteria Candidatus Peregrinibacteria bacterium isolate NC groundwater 1794 Pr3 B 0.1um 45 30 MBI5413418.1
CPR Peregrinbacteria Candidatus Peregrinibacteria bacterium isolate NC groundwater 1973 Pr3 S 0.2um 40 3 MBI4994847.1
CPR Gracilibacteria Candidatus Altimarinota bacterium isolate SO 2016 GW1 150 MFA6520916.1
CPR Peregrinbacteria Candidatus Peregrinibacteria bacterium GW2011 GWA2 33 10 KKP37318.1
CPR Peregrinbacteria Candidatus Peregrinibacteria bacterium isolate SJ737 MCC7196585.1
CPR Gracilibacteria Candidatus Altimarinota bacterium isolate STL PAT5 MFO0781174.1
CPR Gracilibacteria Candidatus Altimarinota bacterium isolate SO 2017 GW1 79 MFA5829906.1
CPR Peregrinbacteria Candidatus Peregrinibacteria bacterium isolate Bin 256 MBD3328276.1
CPR unclassified Patescibacteria Patescibacteria bacterium isolate Modern marine.mb.195 MBU0667967.1
CPR Gracilibacteria Candidatus Altimarinota bacterium isolate AC3.66 MEZ4087309.1
CPR Peregrinbacteria Candidatus Peregrinibacteria bacterium isolate Zod Metabat.293 MBN1494354.1
CPR unclassified Patescibacteria Patescibacteria bacterium isolate Old saline.mb.71 MBU1446602.1
CPR Peregrinbacteria Candidatus Peregrinibacteria bacterium isolate Bin 387 MBD3156482.1
CPR unclassified Patescibacteria Patescibacteria bacterium isolate SAG AM 312 L06 MFC1600099.1
CPR unclassified Patescibacteria Patescibacteria bacterium isolate SAG AM 312 G21 MFC1655472.1
CPR Peregrinbacteria Candidatus Peregrinibacteria bacterium isolate LacPavin 0818 WC45 MAG 45 40 MCX6733191.1
CPR Gracilibacteria Candidatus Altimarinota bacterium isolate SO 2016 LW3 46 MFA7278387.1
CPR Peregrinbacteria Candidatus Peregrinibacteria bacterium isolate SI075 bin129 MBT5016205.1
CPR Peregrinbacteria Candidatus Peregrinibacteria bacterium GW2011 GWF2 39 17 KKR08823.1
CPR Peregrinbacteria Candidatus Peregrinibacteria bacterium GW2011 GWA2 44 7 KKT76104.1
CPR Peregrinbacteria Candidatus Peregrinibacteria bacterium isolate C24 metabat.bin.115 MDP2624729.1
CPR Gracilibacteria Candidatus Altimarinota bacterium isolate SO 2017 GW3 22 MFA5842874.1
CPR unclassified Patescibacteria Patescibacteria bacterium isolate Undefined mixed planktonic.mb.81 MBU1992894.1
CPR Gracilibacteria Candidatus Altimarinota bacterium isolate SO 2017 SWC 26 MFA4891189.1
CPR Peregrinbacteria Candidatus Peregrinibacteria bacterium isolate SI054 bin82 MBT3704626.1
CPR Peregrinbacteria Candidatus Peregrinibacteria bacterium isolate SI054 bin37 MBT3864693.1
CPR Peregrinbacteria Candidatus Peregrinibacteria bacterium HGW Peregrinibacteria 1 isolate HGW Peregrinibacteria 1 PKL37137.1
CPR unclassified Patescibacteria Patescibacteria bacterium isolate H41 bin255 1 MEK7545158.1
CPR Peregrinbacteria Candidatus Peregrinibacteria bacterium isolate NC groundwater 1356 Ag S 0.2um 42 45 MBI4235697.1
CPR Peregrinbacteria Candidatus Peregrinibacteria bacterium isolate SI075 bin40 MBT4917704.1
CPR Peregrinbacteria Candidatus Peregrinibacteria bacterium isolate NC groundwater 1357 Ag S 0.2um 43 19 MBI4231916.1
CPR Gracilibacteria Candidatus Altimarinota bacterium isolate Gw Eff bin 529 MBP7671197.1
CPR unclassified Patescibacteria Patescibacteria bacterium isolate H43 bin115 1 MEK7672448.1
CPR Peregrinbacteria Candidatus Peregrinibacteria bacterium isolate NC groundwater 1792 Pr3 B 0.1um 42 231 MBI5754237.1
CPR Peregrinbacteria Candidatus Peregrinibacteria bacterium isolate LacPavin 0818 WC45 MAG 38 18 MCX6735430.1
CPR unclassified Patescibacteria Patescibacteria bacterium isolate MM PC MetaG.mb.124 MBU1152280.1
CPR Gracilibacteria Candidatus Altimarinota bacterium isolate SO 2017 GW1 5 MFA5948350.1
CPR Peregrinbacteria Candidatus Peregrinibacteria bacterium isolate MEGAHIT 1.923 MFH1284225.1
CPR Peregrinbacteria Candidatus Peregrinibacteria bacterium isolate NC groundwater 534 Ag S 0.1um 43 8 MBI2634469.1
CPR Peregrinbacteria Candidatus Peregrinibacteria bacterium isolate Bin 357 MBD3330501.1
CPR Gracilibacteria Candidatus Altimarinota bacterium isolate STF1 85 MDD3861837.1
CPR Gracilibacteria Candidatus Altimarinota bacterium isolate SO 2017 GW1 132 MFA6917727.1
CPR Gracilibacteria Candidatus Altimarinota bacterium isolate SO 2017 GW1 112 MFA6992098.1
CPR Peregrinbacteria Candidatus Peregrinibacteria bacterium CG10 big fil rev 8 21 14 0 10 36 19 PIR55457.1
CPR Gracilibacteria Candidatus Altimarinota bacterium isolate NORP293 MBL4694867.1
CPR Peregrinbacteria Candidatus Peregrinibacteria bacterium isolate NC groundwater 1972 Pr3 S 0.2um 39 2 MBI4975085.1
CPR Peregrinbacteria Candidatus Peregrinibacteria bacterium RIFCSPLOWO2 02 FULL 39 10 OGJ42868.1
CPR Gracilibacteria Candidatus Altimarinota bacterium isolate SO 2017 GW1 136 MFA6305899.1
CPR Peregrinbacteria Candidatus Peregrinibacteria bacterium isolate C24 metabat.bin.136 MDP2642239.1
CPR Gracilibacteria Candidatus Altimarinota bacterium isolate SO 2016 GW1 bin 4 MCK9186223.1
CPR Gracilibacteria Candidatus Altimarinota bacterium isolate STF1 108 MDD4352198.1
CPR Peregrinbacteria Candidatus Peregrinibacteria bacterium isolate NC groundwater 1795 Pr3 B 0.1um 48 31 MBI5412656.1
CPR Peregrinbacteria Candidatus Peregrinibacteria bacterium CG 4 10 14 0 2 um filter 43 11 PIZ74609.1
CPR Peregrinbacteria Candidatus Peregrinibacteria bacterium isolate MEGAHIT 0.457 iso1 MFH1012439.1
CPR Peregrinbacteria Candidatus Peregrinibacteria bacterium isolate MEGAHIT 0.761 MFH0821084.1
CPR Peregrinbacteria Candidatus Peregrinibacteria bacterium isolate MEGAHIT 0.457 iso2 MFH1012797.1
CPR Peregrinbacteria Candidatus Peregrinibacteria bacterium isolate Zod Metabat.530 MBN1258673.1
CPR unclassified Patescibacteria Patescibacteria bacterium isolate MM PC MetaG.mb.26 MBU1017868.1
CPR Peregrinbacteria Candidatus Peregrinibacteria bacterium isolate Zod Metabat.1133 MBN2087330.1
CPR unclassified Patescibacteria Patescibacteria bacterium isolate Modern marine.mb.280 MBU0577693.1
CPR unclassified Patescibacteria Patescibacteria bacterium isolate Modern marine.mb.142 MBU0727121.1
CPR Peregrinbacteria Candidatus Peregrinibacteria bacterium isolate 8 1 Oct SF Bin107 MDH5596558.1
CPR unclassified Patescibacteria Patescibacteria bacterium isolate Modern marine.mb.163 MBU0705952.1
CPR Peregrinbacteria Candidatus Peregrinibacteria bacterium isolate Zod Metabat.950 MBN2306780.1
CPR Peregrinbacteria Candidatus Peregrinibacteria bacterium isolate NC groundwater 1790 Pr3 B 0.1um 39 20 MBI5414731.1
CPR Peregrinbacteria Candidatus Peregrinibacteria bacterium isolate UWMA 0210 HID91505.1
CPR Peregrinbacteria Candidatus Peregrinibacteria bacterium isolate ELA357 MEI7510955.1
CPR Peregrinbacteria Candidatus Peregrinibacteria bacterium isolate Lyne 18 Q3 R50 59 MAXAC.411 cln QQS59163.1
CPR Gracilibacteria Candidatus Altimarinota bacterium isolate McD1.9 MCT4592167.1
CPR Gracilibacteria Candidatus Altimarinota bacterium isolate SMAG U88 HEY5714269.1
CPR Gracilibacteria Candidatus Altimarinota bacterium isolate RS 2 30 MCK5460700.1
CPR Peregrinbacteria Candidatus Peregrinibacteria bacterium isolate 13 Jan SF Bin21 MDH3324260.1
CPR Gracilibacteria Candidatus Altimarinota bacterium isolate Tobar13.5m G68 MCF7846757.1
CPR Gracilibacteria Candidatus Altimarinota bacterium isolate RM1 1 52 NJP03869.1
CPR Gracilibacteria Candidatus Altimarinota bacterium isolate Tobar13m G44 MCF7830805.1
CPR Gracilibacteria Candidatus Altimarinota bacterium isolate Tobar13.5m G57 MCF7905941.1
CPR Gracilibacteria Candidatus Altimarinota bacterium isolate Tobar13m G52 MCF7812562.1
CPR Gracilibacteria Candidatus Altimarinota bacterium isolate Tobar12.5m G38 MCF7917730.1
CPR Gracilibacteria Candidatus Altimarinota bacterium isolate CA 2019 TPBF 91 MGE3340495.1
CPR Gracilibacteria Candidatus Altimarinota bacterium isolate bin em oilr2.006 MDF2379329.1
CPR unclassified Patescibacteria Patescibacteria bacterium isolate H32 bin062 1 MEK7137586.1
CPR Peregrinbacteria Candidatus Peribacteria bacterium isolate SI054 bin27 MBT3835157.1
CPR Peregrinbacteria Candidatus Peribacteria bacterium isolate SMAG U8109 HLC75573.1
CPR Peregrinbacteria Candidatus Peribacteria bacterium isolate SMAG U8100 HLD07755.1
CPR Peregrinbacteria Candidatus Peribacteria bacterium isolate ETNP8 MAG 49 MDP7069385.1
CPR Peregrinbacteria Candidatus Peribacteria bacterium isolate HKST UBA21 MCA9370757.1
CPR Peregrinbacteria Candidatus Peribacteria bacterium isolate ETNP3 MAG 23 MDP7477093.1
CPR Peregrinbacteria Candidatus Peregrinibacteria bacterium isolate K DeepCast 65m m2 238 MBM3231286.1
CPR Peregrinbacteria Candidatus Peregrinibacteria bacterium isolate SI074 bin159 MBT5236804.1
CPR Peregrinbacteria Candidatus Peregrinibacteria bacterium CG10 big fil rev 8 21 14 0 10 42 8 PIR53664.1
CPR Peregrinbacteria Candidatus Peribacteraceae bacterium isolate SMAG U8035 HLD63340.1
CPR Peregrinbacteria Candidatus Peregrinibacteria bacterium isolate ELA089 MEI8230053.1
CPR Peregrinbacteria Candidatus Peribacteria bacterium isolate STD1 116 MDD5751694.1
CPR Peregrinbacteria Candidatus Peribacteraceae bacterium isolate STB 162 MDD5074278.1
CPR Peregrinbacteria Candidatus Peribacteria bacterium RIFCSPHIGHO2 01 FULL 51 9 OGJ60273.1
CPR Peregrinbacteria Candidatus Peregrinibacteria bacterium isolate NC groundwater 850 Pr1 B 0.1um 50 22 MBI3336026.1
CPR Peregrinbacteria Candidatus Peribacteria bacterium Gr01 1014 25 TSC79383.1
CPR Peregrinbacteria Candidatus Peribacteria bacterium isolate SMAG U6119 HVW66804.1
CPR Peregrinbacteria Candidatus Peribacteria bacterium isolate LakeFargette 0920 ALND MAG 52 24 UPA22189.1
CPR Peregrinbacteria Candidatus Peregrinibacteria bacterium isolate MEGAHIT 0.791 MFH0770663.1
CPR Peregrinbacteria Candidatus Peribacteria bacterium RIFOXYC2 FULL 58 10 OGJ83296.1
CPR Peregrinbacteria Candidatus Peribacteria bacterium isolate STF1 142 MDD4287380.1
CPR Peregrinbacteria Candidatus Peregrinibacteria bacterium isolate MEGAHIT 1.70 MFH1444597.1
CPR Peregrinbacteria Candidatus Peribacteria bacterium isolate SO 2016 LW1 70 MFA7682481.1
CPR Peregrinbacteria Candidatus Peregrinibacteria bacterium CG10 big fil rev 8 21 14 0 10 55 24 PIR48717.1
CPR Peregrinbacteria Candidatus Peribacteria bacterium isolate STD1 62 MDD5469529.1
CPR Peregrinbacteria Candidatus Peribacteria bacterium isolate SO 2016 LW1 48 MFA6259709.1
CPR unclassified Patescibacteria Patescibacteria bacterium isolate MEGAHIT 1.352 MFH1670618.1
CPR unclassified Patescibacteria Patescibacteria bacterium isolate Modern marine.mb.97 MBU0458420.1
CPR Peregrinbacteria Candidatus Peribacteria bacterium isolate ETNP3 MAG 32 MDP7454581.1
CPR Peregrinbacteria Candidatus Peribacteria bacterium isolate Arabian11 MAG 66 MDP6575587.1
CPR Peregrinbacteria Candidatus Peribacteria bacterium isolate RS 8 69 MCK5018875.1
CPR Peregrinbacteria Candidatus Peregrinibacteria bacterium isolate co234 bin51 MBT3292714.1
CPR unclassified Patescibacteria Patescibacteria bacterium isolate SO 2017 SWC 56 MFA4845746.1
CPR Peregrinbacteria Candidatus Peribacteria bacterium RIFCSPHIGHO2 02 FULL 51 15 OGJ63326.1
CPR Peregrinbacteria Candidatus Peribacteria bacterium isolate Gw Eff bin 374 MBP9773907.1
CPR Peregrinbacteria Candidatus Peribacteria bacterium isolate STB 185 MDD5054692.1
CPR Peregrinbacteria Candidatus Peribacteria bacterium isolate Gw Bodden bin 43 MBP7114459.1
CPR Peregrinbacteria Candidatus Peribacteria bacterium isolate ASF 3 MEQ1849452.1
CPR Peregrinbacteria Candidatus Peribacteria bacterium isolate AMR MDS 5595 HRH93637.1
CPR Peregrinbacteria Candidatus Peregrinibacteria bacterium isolate NC groundwater 851 Pr1 B 0.1um 56 11 MBI3331677.1
CPR Peregrinbacteria Candidatus Peribacteria bacterium isolate K Offshore 120m m1 009 MBM3227939.1
CPR Peregrinbacteria Candidatus Peribacteria bacterium RIFCSPHIGHO2 01 FULL 55 13 OGJ59805.1
CPR Peregrinbacteria Candidatus Peregrinibacteria bacterium Greene0416 62 TSC60803.1
CPR Peregrinbacteria Candidatus Peregrinibacteria bacterium CG11 big fil rev 8 21 14 0 20 49 14 PIQ75716.1
CPR Peregrinbacteria Candidatus Peregrinibacteria bacterium CG10 big fil rev 8 21 14 0 10 49 10 PIR53485.1
CPR Peregrinbacteria Candidatus Peribacteria bacterium RIFOXYA1 FULL 56 14 OGJ71965.1
CPR Peregrinbacteria Candidatus Peribacteria bacterium GW2011 GWA2 54 9 KKW41973.1
CPR Peregrinbacteria Candidatus Peribacteria bacterium isolate WB IL 18 NOS67352.1
CPR Peregrinbacteria Candidatus Peribacteria bacterium isolate Baikal deep G211 MSR87400.1
CPR Peregrinbacteria Candidatus Peregrinibacteria bacterium isolate NC groundwater 35 Pr7 B 0.1um 58 27 MBI1812480.1
CPR Peregrinbacteria Candidatus Peregrinibacteria bacterium Greene0416 19 TSC58265.1
CPR Peregrinbacteria Candidatus Peribacteria bacterium isolate RYN 377 iso1 MDB4978671.1
CPR Peregrinbacteria Candidatus Peribacteria bacterium RIFCSPHIGHO2 01 FULL 49 38 OGJ54960.1
CPR Peregrinbacteria Candidatus Peregrinibacteria bacterium CG22 combo CG10 13 8 21 14 all 49 11 PIP65186.1
CPR Peregrinbacteria Candidatus Peregrinibacteria bacterium isolate NC groundwater 248 Ag S 0.2um 56 6 MBI2117448.1
CPR Peregrinbacteria Candidatus Peribacteria bacterium isolate RYN 377 iso2 MFA6522859.1
CPR Peregrinbacteria Candidatus Peribacteria bacterium isolate STF1 123 MDD4318887.1
CPR Peregrinbacteria Candidatus Peribacteria bacterium isolate SO 2017 GW1 244 MFA6038903.1
CPR Peregrinbacteria Candidatus Peribacteria bacterium isolate E14 maxbin.275 MDO8648235.1
CPR Peregrinbacteria Candidatus Peribacteria bacterium RIFCSPHIGHO2 01 FULL 51 35 OGJ56046.1
CPR unclassified Patescibacteria Patescibacteria bacterium isolate H52 bin023 1 MEK7591517.1
CPR Peregrinbacteria Candidatus Peribacteria bacterium isolate SMAG U8014 HLD71274.1

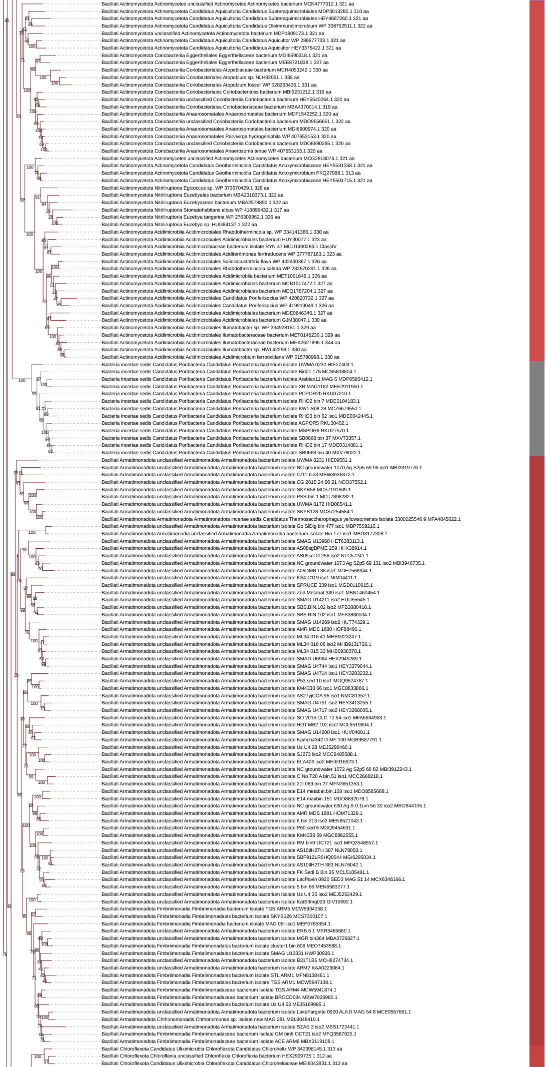
Bacillati Actinomycetota Actinomycetes unclassified Actinomycetes Actinomycetes bacterium MCK4777012.1 321 aa
Bacillati Actinomycetota Actinomycetota Candidatus Aquicultoria Candidatus Subteraquimicrobiales MDP3013280.1 310 aa
Bacillati Actinomycetota Actinomycetota Candidatus Aquicultoria Candidatus Subteraquimicrobiales HEY4687260.1 321 aa
Bacillati Actinomycetota Actinomycetota Candidatus Aquicultoria Candidatus Oleimmundimicrobium WP 308752511.1 322 aa
Bacillati Actinomycetota unclassified Actinomycetota Actinomycetota bacterium MDP1809173.1 321 aa
Bacillati Actinomycetota Actinomycetota Candidatus Aquicultoria Candidatus Aquicultor WP 286677733.1 321 aa
Bacillati Actinomycetota Actinomycetota Candidatus Aquicultoria Candidatus Aquicultor HEY3375422.1 321 aa
Bacillati Actinomycetota Coriobacteriia Eggerthellales Eggerthellaceae bacterium MGI6590318.1 321 aa
Bacillati Actinomycetota Coriobacteriia Eggerthellales Eggerthellaceae bacterium MEE8721838.1 327 aa
Bacillati Actinomycetota Coriobacteriia Coriobacteriales Atopobiaceae bacterium MCH4053242.1 330 aa
Bacillati Actinomycetota Coriobacteriia Coriobacteriales Atopobium sp. NLH92051.1 335 aa
Bacillati Actinomycetota Coriobacteriia Coriobacteriales Atopobium fossor WP 028263426.1 331 aa
Bacillati Actinomycetota Coriobacteriia Coriobacteriales Coriobacteriales bacterium MBI5231212.1 319 aa
Bacillati Actinomycetota Coriobacteriia unclassified Coriobacteriia Coriobacteriia bacterium HEY5540064.1 320 aa
Bacillati Actinomycetota Coriobacteriia Coriobacteriales Coriobacteriaceae bacterium MBA4370014.1 319 aa
Bacillati Actinomycetota Coriobacteriia Anaerosomatales Anaerosomatales bacterium MDF1542252.1 320 aa
Bacillati Actinomycetota Coriobacteriia unclassified Coriobacteriia Coriobacteriia bacterium MDO9556651.1 322 aa
Bacillati Actinomycetota Coriobacteriia Anaerosomatales Anaerosomatales bacterium MDI6900974.1 320 aa
Bacillati Actinomycetota Coriobacteriia Anaerosomatales Parvivirga hydrogeniphila WP 407653153.1 320 aa
Bacillati Actinomycetota Coriobacteriia unclassified Coriobacteriia Coriobacteriia bacterium MDO8880265.1 320 aa
Bacillati Actinomycetota Coriobacteriia Anaerosomatales Anaerosoma tenue WP 407653153.1 320 aa
Bacillati Actinomycetota Actinomycetes unclassified Actinomycetes Actinomycetes bacterium MCG2818076.1 321 aa
Bacillati Actinomycetota Actinomycetota Candidatus Geothermincolia Candidatus Anoxymicrobiaceae HEY5531358.1 321 aa
Bacillati Actinomycetota Actinomycetota Candidatus Geothermincolia Candidatus Anoxymicrobium PKQ27998.1 313 aa
Bacillati Actinomycetota Actinomycetota Candidatus Geothermincolia Candidatus Anoxymicrobiaceae HEY5501715.1 321 aa
Bacillati Actinomycetota Nitriliruptoria Egicoccus sp. WP 373670429.1 328 aa
Bacillati Actinomycetota Nitriliruptoria Euzebyales bacterium MBA2318373.1 322 aa
Bacillati Actinomycetota Nitriliruptoria Euzebyaceae bacterium MBA2578890.1 322 aa
Bacillati Actinomycetota Nitriliruptoria Stomatohabitans albus WP 418896432.1 317 aa
Bacillati Actinomycetota Nitriliruptoria Euzebya tangerina WP 276309962.1 326 aa
Bacillati Actinomycetota Nitriliruptoria Euzebya sp. HUG84137.1 322 aa
Bacillati Actinomycetota Acidimicrobiia Acidimicrobiales Rhabdothermincola sp. WP 334141386.1 330 aa
Bacillati Actinomycetota Acidimicrobiia Acidimicrobiales Acidimicrobiales bacterium HUY30077.1 323 aa
Bacillati Actinomycetota Acidimicrobiia Acidimicrobiales Acidimicrobiaceae bacterium isolate RYN 47 MCU1490266.1 ClassIV
Bacillati Actinomycetota Acidimicrobiia Acidimicrobiales Aciditerrimonas ferrireducens WP 377787183.1 323 aa
Bacillati Actinomycetota Acidimicrobiia Acidimicrobiales Salinilacustrithrix flava WP 432430367.1 326 aa
Bacillati Actinomycetota Acidimicrobiia Acidimicrobiales Rhabdothermincola salaria WP 232670291.1 326 aa
Bacillati Actinomycetota Acidimicrobiia Acidimicrobiales Acidimicrobiia bacterium MET1001646.1 326 aa
Bacillati Actinomycetota Acidimicrobiia Acidimicrobiales Acidimicrobiales bacterium MCB1017472.1 327 aa
Bacillati Actinomycetota Acidimicrobiia Acidimicrobiales Acidimicrobiales bacterium MEQ1787204.1 327 aa
Bacillati Actinomycetota Acidimicrobiia Acidimicrobiales Candidatus Poriferisocius WP 420620732.1 327 aa
Bacillati Actinomycetota Acidimicrobiia Acidimicrobiales Candidatus Poriferisocius WP 419919049.1 326 aa
Bacillati Actinomycetota Acidimicrobiia Acidimicrobiales Acidimicrobiales bacterium MDE0846346.1 327 aa
Bacillati Actinomycetota Acidimicrobiia Acidimicrobiales Acidimicrobiales bacterium GJM38047.1 330 aa
Bacillati Actinomycetota Acidimicrobiia Acidimicrobiales Ilumatobacter sp. WP 394928151.1 329 aa
Bacillati Actinomycetota Acidimicrobiia Acidimicrobiales Ilumatobacteraceae bacterium MET0146230.1 329 aa
Bacillati Actinomycetota Acidimicrobiia Acidimicrobiales Ilumatobacteraceae bacterium MEX2627696.1 344 aa
Bacillati Actinomycetota Acidimicrobiia Acidimicrobiales Ilumatobacter sp. HWL42298.1 330 aa
Bacillati Actinomycetota Acidimicrobiia Acidimicrobiales Acidimicrobium ferrooxidans WP 015798998.1 330 aa
Bacteria incertae sedis Candidatus Poribacteria Candidatus Poribacteria bacterium isolate UWMA 0232 HIE27409.1
Bacteria incertae sedis Candidatus Poribacteria Candidatus Poribacteria bacterium isolate Bin01 175 MCS5608804.1
Bacteria incertae sedis Candidatus Poribacteria Candidatus Poribacteria bacterium isolate Arabian11 MAG 5 MDP6595412.1
Bacteria incertae sedis Candidatus Poribacteria Candidatus Poribacteria bacterium isolate XB MAG1182 MEE2911950.1
Bacteria incertae sedis Candidatus Poribacteria Candidatus Poribacteria bacterium isolate PCPOR2b RKU07210.1
Bacteria incertae sedis Candidatus Poribacteria Candidatus Poribacteria bacterium isolate RHO2 bin 7 MDE0184183.1
Bacteria incertae sedis Candidatus Poribacteria Candidatus Poribacteria bacterium isolate KW1 S08 28 MCZ6679550.1
Bacteria incertae sedis Candidatus Poribacteria Candidatus Poribacteria bacterium isolate RHO3 bin 62 iso1 MDE0042445.1
Bacteria incertae sedis Candidatus Poribacteria Candidatus Poribacteria bacterium isolate AGPOR5 RKU30402.1
Bacteria incertae sedis Candidatus Poribacteria Candidatus Poribacteria bacterium isolate MSPOR6 RKU27570.1
Bacteria incertae sedis Candidatus Poribacteria Candidatus Poribacteria bacterium isolate SB0668 bin 37 MXV73357.1
Bacteria incertae sedis Candidatus Poribacteria Candidatus Poribacteria bacterium isolate RHO2 bin 17 MDE0314881.1
Bacteria incertae sedis Candidatus Poribacteria Candidatus Poribacteria bacterium isolate SB0668 bin 40 MXV78022.1
Bacillati Armatimonadota unclassified Armatimonadota Armatimonadota bacterium isolate UWMA 0231 HIE09551.1
Bacillati Armatimonadota unclassified Armatimonadota Armatimonadota bacterium isolate NC groundwater 1070 Ag S2p5 59 96 iso1 MBI3919776.1
Bacillati Armatimonadota unclassified Armatimonadota Armatimonadota bacterium isolate 0711 bin3 MBW3636872.1
Bacillati Armatimonadota unclassified Armatimonadota Armatimonadota bacterium isolate CG 2015 24 66 21 NCO37552.1
Bacillati Armatimonadota unclassified Armatimonadota Armatimonadota bacterium isolate SKYB58 MCS7191609.1
Bacillati Armatimonadota unclassified Armatimonadota Armatimonadota bacterium isolate PSS.bin.1 MDT7898282.1
Bacillati Armatimonadota unclassified Armatimonadota Armatimonadota bacterium isolate UWMA 0172 HID08541.1
Bacillati Armatimonadota unclassified Armatimonadota Armatimonadota bacterium isolate SKYB128 MCS7254584.1
Bacillati Armatimonadota Armatimonadota Armatimonadota incertae sedis Candidatus Thermosaccharophagus yellowstonensis isolate 3300025546 9 MFA4045022.1
Bacillati Armatimonadota unclassified Armatimonadota Armatimonadota bacterium isolate Go SlDig bin 477 iso1 MBP7559210.1
Bacillati Armatimonadota Armatimonadia unclassified Armatimonadia Armatimonadia bacterium isolate Bin 177 iso1 MBD3177308.1
Bacillati Armatimonadota unclassified Armatimonadota Armatimonadota bacterium isolate SMAG U13960 HET6383113.1
Bacillati Armatimonadota unclassified Armatimonadota Armatimonadota bacterium isolate AS08sgBPME 259 HHX38914.1
Bacillati Armatimonadota unclassified Armatimonadota Armatimonadota bacterium isolate AS09scLD 256 iso2 NLC57241.1
Bacillati Armatimonadota unclassified Armatimonadota Armatimonadota bacterium isolate NC groundwater 1073 Ag S2p5 69 131 iso2 MBI3946735.1
Bacillati Armatimonadota unclassified Armatimonadota Armatimonadota bacterium isolate A05DMB I 38 iso1 MDH7568344.1
Bacillati Armatimonadota unclassified Armatimonadota Armatimonadota bacterium isolate KS4 C119 iso1 NIM04411.1
Bacillati Armatimonadota unclassified Armatimonadota Armatimonadota bacterium isolate SPRUCE 339 iso1 MGD0110615.1
Bacillati Armatimonadota unclassified Armatimonadota Armatimonadota bacterium isolate Zod Metabat.349 iso1 MBN1460454.1
Bacillati Armatimonadota unclassified Armatimonadota Armatimonadota bacterium isolate SMAG U14211 iso2 HUU55545.1
Bacillati Armatimonadota unclassified Armatimonadota Armatimonadota bacterium isolate SBS.BIN.102 iso2 MFB3880410.1
Bacillati Armatimonadota unclassified Armatimonadota Armatimonadota bacterium isolate SBS.BIN.102 iso1 MFB3880004.1
Bacillati Armatimonadota unclassified Armatimonadota Armatimonadota bacterium isolate SMAG U14269 iso2 HUT74329.1
Bacillati Armatimonadota unclassified Armatimonadota Armatimonadota bacterium isolate AMR MDS 1680 HOF88490.1
Bacillati Armatimonadota unclassified Armatimonadota Armatimonadota bacterium isolate ML34 016 41 MHB9023247.1
Bacillati Armatimonadota unclassified Armatimonadota Armatimonadota bacterium isolate ML34 016 08 iso2 MHB9131726.1
Bacillati Armatimonadota unclassified Armatimonadota Armatimonadota bacterium isolate ML34 015 33 MHB0938378.1
Bacillati Armatimonadota unclassified Armatimonadota Armatimonadota bacterium isolate SMAG U6964 HEX2949289.1
Bacillati Armatimonadota unclassified Armatimonadota Armatimonadota bacterium isolate SMAG U4744 iso1 HEY3379044.1
Bacillati Armatimonadota unclassified Armatimonadota Armatimonadota bacterium isolate SMAG U4714 iso1 HEY3283232.1
Bacillati Armatimonadota unclassified Armatimonadota Armatimonadota bacterium isolate P53 sed 10 iso1 MGQ9524787.1
Bacillati Armatimonadota unclassified Armatimonadota Armatimonadota bacterium isolate KM4338 66 iso1 MGC8833866.1
Bacillati Armatimonadota unclassified Armatimonadota Armatimonadota bacterium isolate AS27yjCOA 96 iso1 NMC81352.1
Bacillati Armatimonadota unclassified Armatimonadota Armatimonadota bacterium isolate SMAG U4751 iso2 HEY3413255.1
Bacillati Armatimonadota unclassified Armatimonadota Armatimonadota bacterium isolate SMAG U4717 iso2 HEY3268005.1
Bacillati Armatimonadota unclassified Armatimonadota Armatimonadota bacterium isolate SO 2016 CLC T2 64 iso1 MFA6664963.1
Bacillati Armatimonadota unclassified Armatimonadota Armatimonadota bacterium isolate HOT.MB2.102 iso2 MCL6519604.1
Bacillati Armatimonadota unclassified Armatimonadota Armatimonadota bacterium isolate SMAG U14200 iso1 HUV04601.1
Bacillati Armatimonadota unclassified Armatimonadota Armatimonadota bacterium isolate Kamch4342 D MF 100 MGB9587791.1
Bacillati Armatimonadota unclassified Armatimonadota Armatimonadota bacterium isolate Uz U4 28 MEJ5296460.1
Bacillati Armatimonadota unclassified Armatimonadota Armatimonadota bacterium isolate SJ273 iso2 MCC6485588.1
Bacillati Armatimonadota unclassified Armatimonadota Armatimonadota bacterium isolate ELA409 iso2 MEI6916623.1
Bacillati Armatimonadota unclassified Armatimonadota Armatimonadota bacterium isolate NC groundwater 1072 Ag S2p5 66 82 MBI3912243.1
Bacillati Armatimonadota unclassified Armatimonadota Armatimonadota bacterium isolate C No T20 A bin.51 iso1 MCC2668218.1
Bacillati Armatimonadota unclassified Armatimonadota Armatimonadota bacterium isolate 21l 069.bin.27 MFN3651353.1
Bacillati Armatimonadota unclassified Armatimonadota Armatimonadota bacterium isolate E14 metabat.bin.108 iso1 MDO8585688.1
Bacillati Armatimonadota unclassified Armatimonadota Armatimonadota bacterium isolate E14 maxbin.151 MDO8682078.1
Bacillati Armatimonadota unclassified Armatimonadota Armatimonadota bacterium isolate NC groundwater 630 Ag B 0.1um 56 30 iso2 MBI2844105.1
Bacillati Armatimonadota unclassified Armatimonadota Armatimonadota bacterium isolate AMR MDS 1991 HOM71329.1
Bacillati Armatimonadota unclassified Armatimonadota Armatimonadota bacterium isolate 6 bin.213 iso2 MEN6521043.1
Bacillati Armatimonadota unclassified Armatimonadota Armatimonadota bacterium isolate P60 sed 5 MGQ9454631.1
Bacillati Armatimonadota unclassified Armatimonadota Armatimonadota bacterium isolate KM4338 59 MGC8862555.1
Bacillati Armatimonadota unclassified Armatimonadota Armatimonadota bacterium isolate RM bin9 OCT21 iso1 MFQ3548557.1
Bacillati Armatimonadota unclassified Armatimonadota Armatimonadota bacterium isolate AS10tlH2TH 387 NLN78050.1
Bacillati Armatimonadota unclassified Armatimonadota Armatimonadota bacterium isolate SBF912LR0HQ0044 MGI6295034.1
Bacillati Armatimonadota unclassified Armatimonadota Armatimonadota bacterium isolate AS10tlH2TH 383 NLN76042.1
Bacillati Armatimonadota unclassified Armatimonadota Armatimonadota bacterium isolate FK Sedi B Bin.35 MCL5105481.1
Bacillati Armatimonadota unclassified Armatimonadota Armatimonadota bacterium isolate LacPavin 0920 SED3 MAG 51 14 MCX6346166.1
Bacillati Armatimonadota unclassified Armatimonadota Armatimonadota bacterium isolate 5 bin.66 MEN6583277.1
Bacillati Armatimonadota unclassified Armatimonadota Armatimonadota bacterium isolate Uz U4 35 iso2 MEJ5253429.1
Bacillati Armatimonadota unclassified Armatimonadota Armatimonadota bacterium isolate KatS3mg023 GIV19692.1
Bacillati Armatimonadota Fimbriimonadia Fimbriimonadia bacterium isolate TGS ARM5 MCW5934258.1
Bacillati Armatimonadota Fimbriimonadia Fimbriimonadales bacterium isolate SKYB126 MCS7300107.1
Bacillati Armatimonadota Fimbriimonadia Fimbriimonadia bacterium isolate MAG 05r iso1 MEP0765354.1
Bacillati Armatimonadota unclassified Armatimonadota Armatimonadota bacterium isolate ERB 6 1 MER3466680.1
Bacillati Armatimonadota unclassified Armatimonadota Armatimonadota bacterium isolate MGR bin364 MBA3726827.1
Bacillati Armatimonadota Fimbriimonadia Fimbriimonadales bacterium isolate cluster1 bin.608 MEO7453588.1
Bacillati Armatimonadota Fimbriimonadia Fimbriimonadales bacterium isolate SMAG U13331 HWP30926.1
Bacillati Armatimonadota unclassified Armatimonadota Armatimonadota bacterium isolate B31T1B5 MCH8274734.1
Bacillati Armatimonadota unclassified Armatimonadota Armatimonadota bacterium isolate ARM2 KAA0229084.1
Bacillati Armatimonadota Fimbriimonadia Fimbriimonadales bacterium isolate STL ARM1 MFN8138461.1
Bacillati Armatimonadota Fimbriimonadia Fimbriimonadales bacterium isolate TGS ARM1 MCW5947138.1
Bacillati Armatimonadota Fimbriimonadia Fimbriimonadaceae bacterium isolate TGS ARM4 MCW5941874.1
Bacillati Armatimonadota Fimbriimonadia Fimbriimonadaceae bacterium isolate BROCD034 MBW7926980.1
Bacillati Armatimonadota Fimbriimonadia Fimbriimonadales bacterium isolate Uz U4 53 MEJ5169985.1
Bacillati Armatimonadota unclassified Armatimonadota Armatimonadota bacterium isolate LakeFargette 0920 ALND MAG 54 8 MCE9557661.1
Bacillati Armatimonadota Chthonomonadia Chthonomonas sp. isolate new MAG 281 MBL8048410.1
Bacillati Armatimonadota unclassified Armatimonadota Armatimonadota bacterium isolate SZAS 3 iso2 MBS1722441.1
Bacillati Armatimonadota Fimbriimonadia Fimbriimonadaceae bacterium isolate GM bin6 OCT21 iso2 MFQ3587025.1
Bacillati Armatimonadota Fimbriimonadia Fimbriimonadaceae bacterium isolate ACE ARM6 MBX3119108.1
Bacillati Chloroflexota Candidatus Uliximicrobia Chloroflexota Candidatus Chlorohelix WP 342398145.1 313 aa
Bacillati Chloroflexota Chloroflexia unclassified Chloroflexia Chloroflexia bacterium HEX2909735.1 312 aa
Bacillati Chloroflexota Candidatus Uliximicrobia Chloroflexota Candidatus Chloroheliaceae MEI6043931.1 313 aa

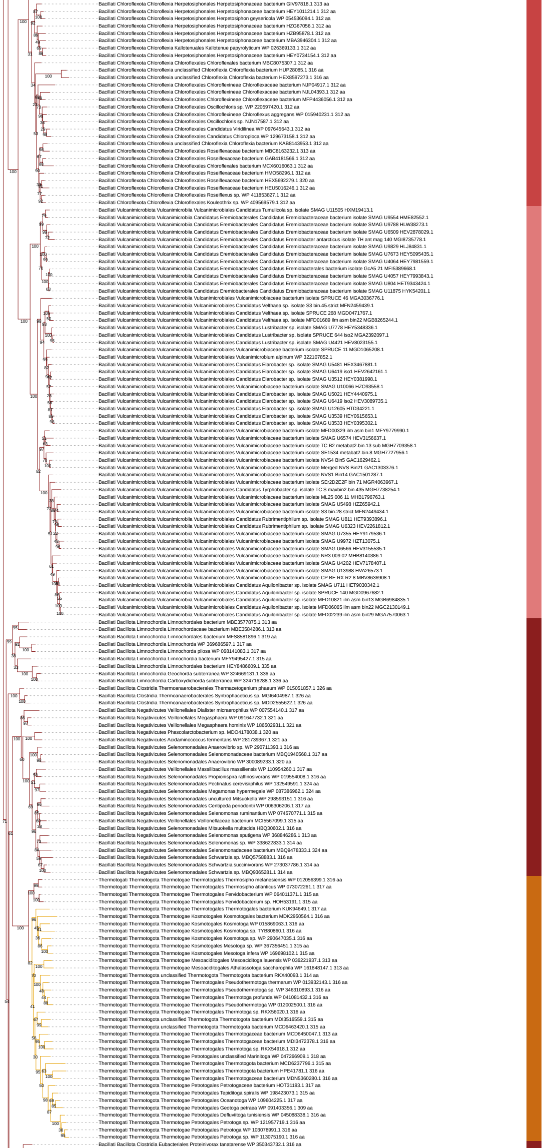
Bacillati Chloroflexota Chloroflexia Herpetosiphonales Herpetosiphonaceae bacterium GIV97818.1 313 aa
Bacillati Chloroflexota Chloroflexia Herpetosiphonales Herpetosiphonaceae bacterium HEY1011214.1 312 aa
Bacillati Chloroflexota Chloroflexia Herpetosiphonales Herpetosiphonaceae Herpetosiphon geysericola WP 054536094.1 312 aa
Bacillati Chloroflexota Chloroflexia Herpetosiphonales Herpetosiphonaceae bacterium HZG67056.1 312 aa
Bacillati Chloroflexota Chloroflexia Herpetosiphonales Herpetosiphonaceae bacterium HZB95878.1 312 aa
Bacillati Chloroflexota Chloroflexia Herpetosiphonales Herpetosiphonaceae bacterium MBA3946304.1 312 aa
Bacillati Chloroflexota Chloroflexia Kallotenuales Kallotenue papyrolyticum WP 026369133.1 312 aa
Bacillati Chloroflexota Chloroflexia Herpetosiphonales Herpetosiphonaceae bacterium HEY0734154.1 312 aa
Bacillati Chloroflexota Chloroflexia Chloroflexales Chloroflexales bacterium MBC8075307.1 312 aa
Bacillati Chloroflexota Chloroflexia unclassified Chloroflexia Chloroflexia bacterium HUP28085.1 316 aa
Bacillati Chloroflexota Chloroflexia unclassified Chloroflexia Chloroflexia bacterium HEX8597273.1 316 aa
Bacillati Chloroflexota Chloroflexia Chloroflexales Chloroflexineae Chloroflexaceae bacterium NJP04917.1 312 aa
Bacillati Chloroflexota Chloroflexia Chloroflexales Chloroflexineae Chloroflexaceae bacterium NJL04393.1 312 aa
Bacillati Chloroflexota Chloroflexia Chloroflexales Chloroflexineae Chloroflexaceae bacterium MFP4436056.1 312 aa
Bacillati Chloroflexota Chloroflexia Chloroflexales Oscillochloris sp. WP 220597420.1 312 aa
Bacillati Chloroflexota Chloroflexia Chloroflexales Chloroflexineae Chloroflexus aggregans WP 015940231.1 312 aa
Bacillati Chloroflexota Chloroflexia Chloroflexales Oscillochloris sp. NJN17587.1 312 aa
Bacillati Chloroflexota Chloroflexia Chloroflexales Candidatus Viridilinea WP 097645643.1 312 aa
Bacillati Chloroflexota Chloroflexia Chloroflexales Candidatus Chloroploca WP 129673158.1 312 aa
Bacillati Chloroflexota Chloroflexia unclassified Chloroflexia Chloroflexia bacterium KAB8143953.1 312 aa
Bacillati Chloroflexota Chloroflexia Chloroflexales Roseiflexaceae bacterium MBC8163232.1 313 aa
Bacillati Chloroflexota Chloroflexia Chloroflexales Roseiflexaceae bacterium GAB4181566.1 312 aa
Bacillati Chloroflexota Chloroflexia Chloroflexales Chloroflexales bacterium MCX6016063.1 312 aa
Bacillati Chloroflexota Chloroflexia Chloroflexales Roseiflexaceae bacterium HMO58296.1 312 aa
Bacillati Chloroflexota Chloroflexia Chloroflexales Roseiflexaceae bacterium HEX5692279.1 320 aa
Bacillati Chloroflexota Chloroflexia Chloroflexales Roseiflexaceae bacterium HEU5016246.1 312 aa
Bacillati Chloroflexota Chloroflexia Chloroflexales Roseiflexus sp. WP 411853827.1 312 aa
Bacillati Chloroflexota Chloroflexia Chloroflexales Kouleothrix sp. WP 409569579.1 312 aa
Bacillati Vulcanimicrobiota Vulcanimicrobiia Vulcanimicrobiales Candidatus Tumulicola sp. isolate SMAG U11505 HXM19413.1
Bacillati Vulcanimicrobiota Vulcanimicrobiia Candidatus Eremiobacterales Candidatus Eremiobacteraceae bacterium isolate SMAG U9554 HME82552.1
Bacillati Vulcanimicrobiota Vulcanimicrobiia Candidatus Eremiobacterales Candidatus Eremiobacteraceae bacterium isolate SMAG U9788 HLW38273.1
Bacillati Vulcanimicrobiota Vulcanimicrobiia Candidatus Eremiobacterales Candidatus Eremiobacteraceae bacterium isolate SMAG U6509 HEV2878029.1
Bacillati Vulcanimicrobiota Vulcanimicrobiia Candidatus Eremiobacterales Candidatus Eremiobacter antarcticus isolate TH ant mag 140 MGI8735778.1
Bacillati Vulcanimicrobiota Vulcanimicrobiia Candidatus Eremiobacterales Candidatus Eremiobacteraceae bacterium isolate SMAG U9829 HLJ84831.1
Bacillati Vulcanimicrobiota Vulcanimicrobiia Candidatus Eremiobacterales Candidatus Eremiobacteraceae bacterium isolate SMAG U7673 HEY5095435.1
Bacillati Vulcanimicrobiota Vulcanimicrobiia Candidatus Eremiobacterales Candidatus Eremiobacteraceae bacterium isolate SMAG U4064 HEY7981559.1
Bacillati Vulcanimicrobiota Vulcanimicrobiia Candidatus Eremiobacterales Candidatus Eremiobacterales bacterium isolate GcA5 21 MFI5389668.1
Bacillati Vulcanimicrobiota Vulcanimicrobiia Candidatus Eremiobacterales Candidatus Eremiobacteraceae bacterium isolate SMAG U4057 HEY7993843.1
Bacillati Vulcanimicrobiota Vulcanimicrobiia Candidatus Eremiobacterales Candidatus Eremiobacteraceae bacterium isolate SMAG U804 HET9343424.1
Bacillati Vulcanimicrobiota Vulcanimicrobiia Candidatus Eremiobacterales Candidatus Eremiobacteraceae bacterium isolate SMAG U11875 HYK54201.1
Bacillati Vulcanimicrobiota Vulcanimicrobiia Vulcanimicrobiales Vulcanimicrobiaceae bacterium isolate SPRUCE 46 MGA3036776.1
Bacillati Vulcanimicrobiota Vulcanimicrobiia Vulcanimicrobiales Candidatus Velthaea sp. isolate S3 bin.45.strict MFN2459439.1
Bacillati Vulcanimicrobiota Vulcanimicrobiia Vulcanimicrobiales Candidatus Velthaea sp. isolate SPRUCE 268 MGD0471767.1
Bacillati Vulcanimicrobiota Vulcanimicrobiia Vulcanimicrobiales Candidatus Velthaea sp. isolate MFD01689 ilm asm bin22 MGB8265244.1
Bacillati Vulcanimicrobiota Vulcanimicrobiia Vulcanimicrobiales Candidatus Lustribacter sp. isolate SMAG U7778 HEY5348336.1
Bacillati Vulcanimicrobiota Vulcanimicrobiia Vulcanimicrobiales Candidatus Lustribacter sp. isolate SPRUCE 644 iso2 MGA2392097.1
Bacillati Vulcanimicrobiota Vulcanimicrobiia Vulcanimicrobiales Candidatus Lustribacter sp. isolate SMAG U4421 HEV8023155.1
Bacillati Vulcanimicrobiota Vulcanimicrobiia Vulcanimicrobiales Vulcanimicrobiaceae bacterium isolate SPRUCE 11 MGD1065208.1
Bacillati Vulcanimicrobiota Vulcanimicrobiia Vulcanimicrobiales Vulcanimicrobium alpinum WP 322107852.1
Bacillati Vulcanimicrobiota Vulcanimicrobiia Vulcanimicrobiales Candidatus Elarobacter sp. isolate SMAG U5481 HEX3467881.1
Bacillati Vulcanimicrobiota Vulcanimicrobiia Vulcanimicrobiales Candidatus Elarobacter sp. isolate SMAG U6419 iso1 HEV2642161.1
Bacillati Vulcanimicrobiota Vulcanimicrobiia Vulcanimicrobiales Candidatus Elarobacter sp. isolate SMAG U3512 HEY0381998.1
Bacillati Vulcanimicrobiota Vulcanimicrobiia Vulcanimicrobiales Vulcanimicrobiaceae bacterium isolate SMAG U10066 HZO93558.1
Bacillati Vulcanimicrobiota Vulcanimicrobiia Vulcanimicrobiales Candidatus Elarobacter sp. isolate SMAG U5021 HEY4440975.1
Bacillati Vulcanimicrobiota Vulcanimicrobiia Vulcanimicrobiales Candidatus Elarobacter sp. isolate SMAG U6419 iso2 HEV3089735.1
Bacillati Vulcanimicrobiota Vulcanimicrobiia Vulcanimicrobiales Candidatus Elarobacter sp. isolate SMAG U12605 HTD34221.1
Bacillati Vulcanimicrobiota Vulcanimicrobiia Vulcanimicrobiales Candidatus Elarobacter sp. isolate SMAG U3539 HEY0615653.1
Bacillati Vulcanimicrobiota Vulcanimicrobiia Vulcanimicrobiales Candidatus Elarobacter sp. isolate SMAG U3533 HEY0395302.1
Bacillati Vulcanimicrobiota Vulcanimicrobiia Vulcanimicrobiales Vulcanimicrobiaceae bacterium isolate MFD00329 ilm asm bin1 MFY9779990.1
Bacillati Vulcanimicrobiota Vulcanimicrobiia Vulcanimicrobiales Vulcanimicrobiaceae bacterium isolate SMAG U6574 HEV3156637.1
Bacillati Vulcanimicrobiota Vulcanimicrobiia Vulcanimicrobiales Vulcanimicrobiaceae bacterium isolate TC B2 metabat2.bin.13 sub MGH7709358.1
Bacillati Vulcanimicrobiota Vulcanimicrobiia Vulcanimicrobiales Vulcanimicrobiaceae bacterium isolate SE1534 metabat2.bin.8 MGH7727956.1
Bacillati Vulcanimicrobiota Vulcanimicrobiia Vulcanimicrobiales Vulcanimicrobiaceae bacterium isolate NVS4 Bin5 GAC1629462.1
Bacillati Vulcanimicrobiota Vulcanimicrobiia Vulcanimicrobiales Vulcanimicrobiaceae bacterium isolate Merged NVS Bin21 GAC1303376.1
Bacillati Vulcanimicrobiota Vulcanimicrobiia Vulcanimicrobiales Vulcanimicrobiaceae bacterium isolate NVS1 Bin14 GAC1501287.1
Bacillati Vulcanimicrobiota Vulcanimicrobiia Vulcanimicrobiales Vulcanimicrobiaceae bacterium isolate SEr2D2E2F bin 71 MGR4063967.1
Bacillati Vulcanimicrobiota Vulcanimicrobiia Vulcanimicrobiales Candidatus Tyrphobacter sp. isolate TC S maxbin2.bin.435 MGH7738254.1
Bacillati Vulcanimicrobiota Vulcanimicrobiia Vulcanimicrobiales Vulcanimicrobiaceae bacterium isolate ML25 006 11 MHB1796763.1
Bacillati Vulcanimicrobiota Vulcanimicrobiia Vulcanimicrobiales Vulcanimicrobiaceae bacterium isolate SMAG U5498 HZZ65942.1
Bacillati Vulcanimicrobiota Vulcanimicrobiia Vulcanimicrobiales Vulcanimicrobiaceae bacterium isolate S3 bin.28.strict MFN2449434.1
Bacillati Vulcanimicrobiota Vulcanimicrobiia Vulcanimicrobiales Candidatus Rubrimentiphilum sp. isolate SMAG U811 HET9393896.1
Bacillati Vulcanimicrobiota Vulcanimicrobiia Vulcanimicrobiales Candidatus Rubrimentiphilum sp. isolate SMAG U6323 HEV2261812.1
Bacillati Vulcanimicrobiota Vulcanimicrobiia Vulcanimicrobiales Vulcanimicrobiaceae bacterium isolate SMAG U7355 HEY9179536.1
Bacillati Vulcanimicrobiota Vulcanimicrobiia Vulcanimicrobiales Vulcanimicrobiaceae bacterium isolate SMAG U9972 HZT13075.1
Bacillati Vulcanimicrobiota Vulcanimicrobiia Vulcanimicrobiales Vulcanimicrobiaceae bacterium isolate SMAG U6566 HEV3155535.1
Bacillati Vulcanimicrobiota Vulcanimicrobiia Vulcanimicrobiales Vulcanimicrobiaceae bacterium isolate NR3 009 02 MHB8140386.1
Bacillati Vulcanimicrobiota Vulcanimicrobiia Vulcanimicrobiales Vulcanimicrobiaceae bacterium isolate SMAG U4202 HEV7178407.1
Bacillati Vulcanimicrobiota Vulcanimicrobiia Vulcanimicrobiales Vulcanimicrobiaceae bacterium isolate SMAG U13988 HVA26573.1
Bacillati Vulcanimicrobiota Vulcanimicrobiia Vulcanimicrobiales Vulcanimicrobiaceae bacterium isolate CP BE RX R2 8 MBV8636908.1
Bacillati Vulcanimicrobiota Vulcanimicrobiia Vulcanimicrobiales Candidatus Aquilonibacter sp. isolate SMAG U711 HET9030342.1
Bacillati Vulcanimicrobiota Vulcanimicrobiia Vulcanimicrobiales Candidatus Aquilonibacter sp. isolate SPRUCE 140 MGD0967682.1
Bacillati Vulcanimicrobiota Vulcanimicrobiia Vulcanimicrobiales Candidatus Aquilonibacter sp. isolate MFD10821 ilm asm bin13 MGB6984835.1
Bacillati Vulcanimicrobiota Vulcanimicrobiia Vulcanimicrobiales Candidatus Aquilonibacter sp. isolate MFD06065 ilm asm bin22 MGC2130149.1
Bacillati Vulcanimicrobiota Vulcanimicrobiia Vulcanimicrobiales Candidatus Aquilonibacter sp. isolate MFD02239 ilm asm bin29 MGA7570063.1
Bacillati Bacillota Limnochordia Limnochordales bacterium MBE3577875.1 313 aa
Bacillati Bacillota Limnochordia Limnochordaceae bacterium MBE3584286.1 313 aa
Bacillati Bacillota Limnochordia Limnochordales bacterium MFS8581896.1 319 aa
Bacillati Bacillota Limnochordia Limnochorda WP 369686597.1 317 aa
Bacillati Bacillota Limnochordia Limnochorda pilosa WP 068141083.1 317 aa
Bacillati Bacillota Limnochordia Limnochordia bacterium MFY9495427.1 315 aa
Bacillati Bacillota Limnochordia Limnochordales bacterium HEY8486609.1 335 aa
Bacillati Bacillota Limnochordia Geochorda subterranea WP 324669131.1 336 aa
Bacillati Bacillota Limnochordia Carboxydichorda subterranea WP 324716288.1 336 aa
Bacillati Bacillota Clostridia Thermoanaerobacterales Thermacetogenium phaeum WP 015051857.1 326 aa
Bacillati Bacillota Clostridia Thermoanaerobacterales Syntrophaceticus sp. MGI6404987.1 326 aa
Bacillati Bacillota Clostridia Thermoanaerobacterales Syntrophaceticus sp. MDD2555622.1 326 aa
Bacillati Bacillota Negativicutes Veillonellales Dialister micraerophilus WP 007554140.1 317 aa
Bacillati Bacillota Negativicutes Veillonellales Megasphaera WP 091647732.1 321 aa
Bacillati Bacillota Negativicutes Veillonellales Megasphaera hominis WP 186502931.1 321 aa
Bacillati Bacillota Negativicutes Phascolarctobacterium sp. MDO4178038.1 320 aa
Bacillati Bacillota Negativicutes Acidaminococcus fermentans WP 281739367.1 321 aa
Bacillati Bacillota Negativicutes Selenomonadales Anaerovibrio sp. WP 290711393.1 316 aa
Bacillati Bacillota Negativicutes Selenomonadales Selenomonadaceae bacterium MBQ1940568.1 317 aa
Bacillati Bacillota Negativicutes Selenomonadales Anaerovibrio WP 300089233.1 320 aa
Bacillati Bacillota Negativicutes Veillonellales Massilibacillus massiliensis WP 110954260.1 317 aa
Bacillati Bacillota Negativicutes Selenomonadales Propionispira raffinosivorans WP 019554008.1 316 aa
Bacillati Bacillota Negativicutes Selenomonadales Pectinatus cerevisiiphilus WP 132549591.1 324 aa
Bacillati Bacillota Negativicutes Selenomonadales Megamonas hypermegale WP 087386962.1 324 aa
Bacillati Bacillota Negativicutes Selenomonadales uncultured Mitsuokella WP 298593151.1 316 aa
Bacillati Bacillota Negativicutes Selenomonadales Centipeda periodontii WP 006306206.1 317 aa
Bacillati Bacillota Negativicutes Selenomonadales Selenomonas ruminantium WP 074570771.1 315 aa
Bacillati Bacillota Negativicutes Veillonellales Veillonellaceae bacterium MCI5567099.1 315 aa
Bacillati Bacillota Negativicutes Selenomonadales Mitsuokella multacida HBQ30602.1 316 aa
Bacillati Bacillota Negativicutes Selenomonadales Selenomonas sputigena WP 368846286.1 313 aa
Bacillati Bacillota Negativicutes Selenomonadales Selenomonas sp. WP 338622833.1 314 aa
Bacillati Bacillota Negativicutes Selenomonadales Selenomonadaceae bacterium MBQ9478333.1 324 aa
Bacillati Bacillota Negativicutes Selenomonadales Schwartzia sp. MBQ5758883.1 316 aa
Bacillati Bacillota Negativicutes Selenomonadales Schwartzia succinivorans WP 273037786.1 314 aa
Bacillati Bacillota Negativicutes Selenomonadales Schwartzia sp. MBQ9365281.1 314 aa
Thermotogati Thermotogota Thermotogae Thermotogales Thermosipho melanesiensis WP 012056399.1 316 aa
Thermotogati Thermotogota Thermotogae Thermotogales Thermosipho atlanticus WP 073072261.1 317 aa
Thermotogati Thermotogota Thermotogae Thermotogales Fervidobacterium WP 064011371.1 315 aa
Thermotogati Thermotogota Thermotogae Thermotogales Fervidobacterium sp. HOH53191.1 315 aa
Thermotogati Thermotogota Thermotogae Thermotogales Thermotogales bacterium KUK94649.1 317 aa
Thermotogati Thermotogota Thermotogae Kosmotogales Kosmotogales bacterium MDK2950564.1 316 aa
Thermotogati Thermotogota Thermotogae Kosmotogales Kosmotoga WP 015869063.1 316 aa
Thermotogati Thermotogota Thermotogae Kosmotogales Kosmotoga sp. TYB80860.1 316 aa
Thermotogati Thermotogota Thermotogae Kosmotogales Kosmotoga sp. WP 290647035.1 316 aa
Thermotogati Thermotogota Thermotogae Kosmotogales Mesotoga sp. WP 367356451.1 315 aa
Thermotogati Thermotogota Thermotogae Kosmotogales Mesotoga infera WP 169698102.1 315 aa
Thermotogati Thermotogota Thermotogae Mesoaciditogales Mesoaciditoga lauensis WP 036221937.1 313 aa
Thermotogati Thermotogota Thermotogae Mesoaciditogales Athalassotoga saccharophila WP 161848147.1 313 aa
Thermotogati Thermotogota unclassified Thermotogota Thermotogota bacterium RKX40093.1 314 aa
Thermotogati Thermotogota Thermotogae Thermotogales Pseudothermotoga thermarum WP 013932143.1 316 aa
Thermotogati Thermotogota Thermotogae Thermotogales Pseudothermotoga sp. WP 346310893.1 316 aa
Thermotogati Thermotogota Thermotogae Thermotogales Thermotoga profunda WP 041081432.1 316 aa
Thermotogati Thermotogota Thermotogae Thermotogales Pseudothermotoga WP 012002500.1 316 aa
Thermotogati Thermotogota Thermotogae Thermotogales Thermotoga sp. RKX56020.1 316 aa
Thermotogati Thermotogota unclassified Thermotogota Thermotogota bacterium MDI3516559.1 315 aa
Thermotogati Thermotogota unclassified Thermotogota Thermotogota bacterium MCD6463420.1 315 aa
Thermotogati Thermotogota Thermotogae Thermotogales Thermotogaceae bacterium MCD6450047.1 313 aa
Thermotogati Thermotogota Thermotogae Thermotogales Thermotogaceae bacterium MDI3472378.1 316 aa
Thermotogati Thermotogota Thermotogae Thermotogales Thermotoga sp. RKX54918.1 312 aa
Thermotogati Thermotogota Thermotogae Petrotogales unclassified Marinitoga WP 047266909.1 318 aa
Thermotogati Thermotogota Thermotogae Thermotogales Thermotogota bacterium MCD6237796.1 315 aa
Thermotogati Thermotogota Thermotogae Thermotogales Thermotogota bacterium HPE41781.1 316 aa
Thermotogati Thermotogota Thermotogae Thermotogales Thermotogaceae bacterium MDN5360280.1 316 aa
Thermotogati Thermotogota Thermotogae Petrotogales Petrotogaceae bacterium HOT31193.1 317 aa
Thermotogati Thermotogota Thermotogae Petrotogales Tepiditoga spiralis WP 198423073.1 315 aa
Thermotogati Thermotogota Thermotogae Petrotogales Oceanotoga WP 109604225.1 317 aa
Thermotogati Thermotogota Thermotogae Petrotogales Geotoga petraea WP 091403356.1 309 aa
Thermotogati Thermotogota Thermotogae Petrotogales Defluviitoga tunisiensis WP 045088338.1 316 aa
Thermotogati Thermotogota Thermotogae Petrotogales Petrotoga sp. WP 121957719.1 316 aa
Thermotogati Thermotogota Thermotogae Petrotogales Petrotoga WP 103078991.1 316 aa
Thermotogati Thermotogota Thermotogae Petrotogales Petrotoga sp. WP 113075190.1 316 aa
Bacillati Bacillota Clostridia Eubacteriales Proteinivorax tanatarense WP 350343732.1 316 aa

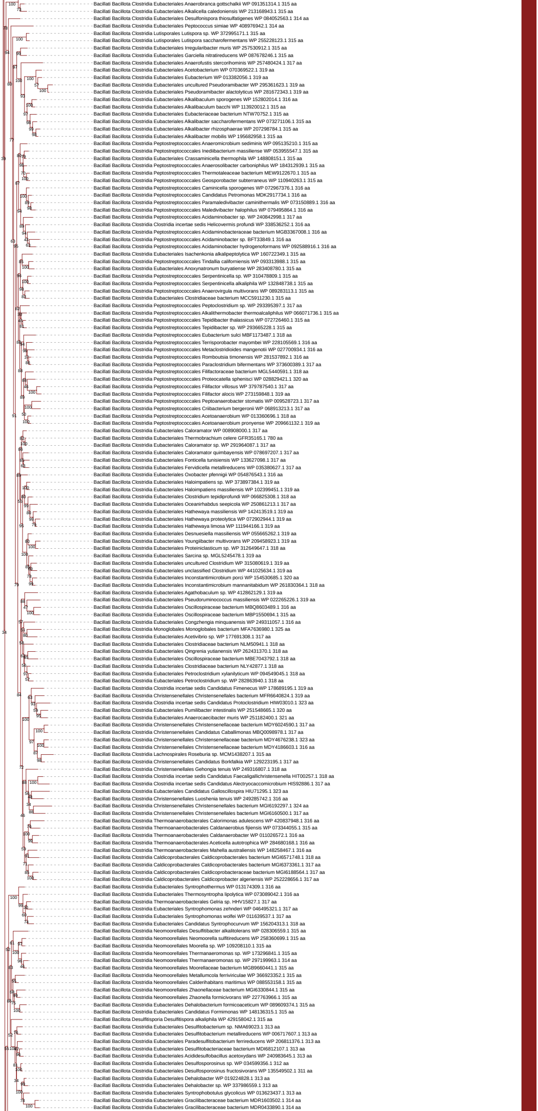

Bacillati Bacillota Clostridia Eubacteriales Anaerobranca gottschalkii WP 091351314.1 315 aa
Bacillati Bacillota Clostridia Eubacteriales Alkalicella caledoniensis WP 213168943.1 315 aa
Bacillati Bacillota Clostridia Eubacteriales Desulfonispora thiosulfatigenes WP 084052563.1 314 aa
Bacillati Bacillota Clostridia Eubacteriales Peptococcus simiae WP 408976942.1 314 aa
Bacillati Bacillota Clostridia Lutisporales Lutispora sp. WP 372995171.1 315 aa
Bacillati Bacillota Clostridia Lutisporales Lutispora saccharofermentans WP 255228123.1 315 aa
Bacillati Bacillota Clostridia Eubacteriales Irregularibacter muris WP 257530912.1 315 aa
Bacillati Bacillota Clostridia Eubacteriales Garciella nitratireducens WP 087678246.1 315 aa
Bacillati Bacillota Clostridia Eubacteriales Anaerofustis stercorihominis WP 257480424.1 317 aa
Bacillati Bacillota Clostridia Eubacteriales Acetobacterium WP 070369522.1 319 aa
Bacillati Bacillota Clostridia Eubacteriales Eubacterium WP 013382056.1 319 aa
Bacillati Bacillota Clostridia Eubacteriales uncultured Pseudoramibacter WP 295361623.1 319 aa
Bacillati Bacillota Clostridia Eubacteriales Pseudoramibacter alactolyticus WP 281672343.1 319 aa
Bacillati Bacillota Clostridia Eubacteriales Alkalibaculum sporogenes WP 152802014.1 316 aa
Bacillati Bacillota Clostridia Eubacteriales Alkalibaculum bacchi WP 113920012.1 315 aa
Bacillati Bacillota Clostridia Eubacteriales Eubacteriaceae bacterium NTW70752.1 315 aa
Bacillati Bacillota Clostridia Eubacteriales Alkalibacter saccharofermentans WP 073271106.1 315 aa
Bacillati Bacillota Clostridia Eubacteriales Alkalibacter rhizosphaerae WP 207298784.1 315 aa
Bacillati Bacillota Clostridia Eubacteriales Alkalibacter mobilis WP 195682958.1 315 aa
Bacillati Bacillota Clostridia Peptostreptococcales Anaeromicrobium sediminis WP 095135210.1 315 aa
Bacillati Bacillota Clostridia Peptostreptococcales Inediibacterium massiliense WP 053955547.1 315 aa
Bacillati Bacillota Clostridia Eubacteriales Crassaminicella thermophila WP 148808151.1 315 aa
Bacillati Bacillota Clostridia Peptostreptococcales Anaerosolibacter carboniphilus WP 184312939.1 315 aa
Bacillati Bacillota Clostridia Peptostreptococcales Thermotaleaceae bacterium MEW9122670.1 315 aa
Bacillati Bacillota Clostridia Peptostreptococcales Geosporobacter subterraneus WP 110940263.1 315 aa
Bacillati Bacillota Clostridia Peptostreptococcales Caminicella sporogenes WP 072967376.1 316 aa
Bacillati Bacillota Clostridia Peptostreptococcales Candidatus Petromonas MDK2917734.1 316 aa
Bacillati Bacillota Clostridia Peptostreptococcales Paramaledivibacter caminithermalis WP 073150889.1 316 aa
Bacillati Bacillota Clostridia Peptostreptococcales Maledivibacter halophilus WP 079495864.1 316 aa
Bacillati Bacillota Clostridia Peptostreptococcales Acidaminobacter sp. WP 240842998.1 317 aa
Bacillati Bacillota Clostridia Clostridia incertae sedis Helicovermis profundi WP 338536252.1 316 aa
Bacillati Bacillota Clostridia Peptostreptococcales Acidaminobacteraceae bacterium MGB3367008.1 316 aa
Bacillati Bacillota Clostridia Peptostreptococcales Acidaminobacter sp. BFT33849.1 316 aa
Bacillati Bacillota Clostridia Peptostreptococcales Acidaminobacter hydrogenoformans WP 092588916.1 316 aa
Bacillati Bacillota Clostridia Eubacteriales Isachenkonia alkalipeptolytica WP 160722349.1 315 aa
Bacillati Bacillota Clostridia Peptostreptococcales Tindallia californiensis WP 093313988.1 315 aa
Bacillati Bacillota Clostridia Eubacteriales Anoxynatronum buryatiense WP 283408780.1 315 aa
Bacillati Bacillota Clostridia Peptostreptococcales Serpentinicella sp. WP 310478809.1 315 aa
Bacillati Bacillota Clostridia Peptostreptococcales Serpentinicella alkaliphila WP 132848738.1 315 aa
Bacillati Bacillota Clostridia Peptostreptococcales Anaerovirgula multivorans WP 089283113.1 315 aa
Bacillati Bacillota Clostridia Eubacteriales Clostridiaceae bacterium MCC5911230.1 315 aa
Bacillati Bacillota Clostridia Peptostreptococcales Peptoclostridium sp. WP 293395397.1 317 aa
Bacillati Bacillota Clostridia Peptostreptococcales Alkalithermobacter thermoalcaliphilus WP 066071736.1 315 aa
Bacillati Bacillota Clostridia Peptostreptococcales Tepidibacter thalassicus WP 072726460.1 315 aa
Bacillati Bacillota Clostridia Peptostreptococcales Tepidibacter sp. WP 293665228.1 315 aa
Bacillati Bacillota Clostridia Peptostreptococcales Eubacterium sulci MBF1173487.1 318 aa
Bacillati Bacillota Clostridia Peptostreptococcales Terrisporobacter mayombei WP 228105569.1 316 aa
Bacillati Bacillota Clostridia Peptostreptococcales Metaclostridioides mangenotii WP 027700934.1 316 aa
Bacillati Bacillota Clostridia Peptostreptococcales Romboutsia timonensis WP 281537892.1 316 aa
Bacillati Bacillota Clostridia Peptostreptococcales Paraclostridium bifermentans WP 373600389.1 317 aa
Bacillati Bacillota Clostridia Peptostreptococcales Filifactoraceae bacterium MGL5440591.1 318 aa
Bacillati Bacillota Clostridia Peptostreptococcales Proteocatella sphenisci WP 028829421.1 320 aa
Bacillati Bacillota Clostridia Peptostreptococcales Filifactor villosus WP 379787540.1 317 aa
Bacillati Bacillota Clostridia Peptostreptococcales Filifactor alocis WP 273159848.1 319 aa
Bacillati Bacillota Clostridia Peptostreptococcales Peptoanaerobacter stomatis WP 009528723.1 317 aa
Bacillati Bacillota Clostridia Peptostreptococcales Criibacterium bergeronii WP 068913213.1 317 aa
Bacillati Bacillota Clostridia Peptostreptococcales Acetoanaerobium WP 013360696.1 318 aa
Bacillati Bacillota Clostridia Peptostreptococcales Acetoanaerobium pronyense WP 209661132.1 319 aa
Bacillati Bacillota Clostridia Eubacteriales Caloramator WP 008908000.1 317 aa
Bacillati Bacillota Clostridia Eubacteriales Thermobrachium celere GFR35165.1 780 aa
Bacillati Bacillota Clostridia Eubacteriales Caloramator sp. WP 291964087.1 317 aa
Bacillati Bacillota Clostridia Eubacteriales Caloramator quimbayensis WP 078697207.1 317 aa
Bacillati Bacillota Clostridia Eubacteriales Fonticella tunisiensis WP 133627098.1 317 aa
Bacillati Bacillota Clostridia Eubacteriales Fervidicella metallireducens WP 035380627.1 317 aa
Bacillati Bacillota Clostridia Eubacteriales Oxobacter pfennigii WP 054876543.1 316 aa
Bacillati Bacillota Clostridia Eubacteriales Haloimpatiens sp. WP 373897384.1 319 aa
Bacillati Bacillota Clostridia Eubacteriales Haloimpatiens massiliensis WP 102399451.1 319 aa
Bacillati Bacillota Clostridia Eubacteriales Clostridium tepidiprofundi WP 066825308.1 318 aa
Bacillati Bacillota Clostridia Eubacteriales Oceanirhabdus seepicola WP 250861213.1 317 aa
Bacillati Bacillota Clostridia Eubacteriales Hathewaya massiliensis WP 142413519.1 319 aa
Bacillati Bacillota Clostridia Eubacteriales Hathewaya proteolytica WP 072902944.1 319 aa
Bacillati Bacillota Clostridia Eubacteriales Hathewaya limosa WP 111944166.1 319 aa
Bacillati Bacillota Clostridia Eubacteriales Desnuesiella massiliensis WP 055665262.1 319 aa
Bacillati Bacillota Clostridia Eubacteriales Youngiibacter multivorans WP 209458923.1 319 aa
Bacillati Bacillota Clostridia Eubacteriales Proteiniclasticum sp. WP 312649647.1 318 aa
Bacillati Bacillota Clostridia Eubacteriales Sarcina sp. MGL5245478.1 319 aa
Bacillati Bacillota Clostridia Eubacteriales uncultured Clostridium WP 315080619.1 319 aa
Bacillati Bacillota Clostridia Eubacteriales unclassified Clostridium WP 441025634.1 319 aa
Bacillati Bacillota Clostridia Eubacteriales Inconstantimicrobium porci WP 154530685.1 320 aa
Bacillati Bacillota Clostridia Eubacteriales Inconstantimicrobium mannanitabidum WP 261830364.1 318 aa
Bacillati Bacillota Clostridia Eubacteriales Agathobaculum sp. WP 412862129.1 319 aa
Bacillati Bacillota Clostridia Eubacteriales Pseudoruminococcus massiliensis WP 022265226.1 319 aa
Bacillati Bacillota Clostridia Eubacteriales Oscillospiraceae bacterium MBQ8603489.1 316 aa
Bacillati Bacillota Clostridia Eubacteriales Oscillospiraceae bacterium MBP1550694.1 315 aa
Bacillati Bacillota Clostridia Eubacteriales Congzhengia minquanensis WP 249311057.1 316 aa
Bacillati Bacillota Clostridia Monoglobales Monoglobales bacterium MFA7636980.1 325 aa
Bacillati Bacillota Clostridia Eubacteriales Acetivibrio sp. WP 177691308.1 317 aa
Bacillati Bacillota Clostridia Eubacteriales Clostridiaceae bacterium NLM50941.1 318 aa
Bacillati Bacillota Clostridia Eubacteriales Qingrenia yutianensis WP 262431370.1 318 aa
Bacillati Bacillota Clostridia Eubacteriales Oscillospiraceae bacterium MBE7043792.1 318 aa
Bacillati Bacillota Clostridia Eubacteriales Clostridiaceae bacterium NLY42877.1 318 aa
Bacillati Bacillota Clostridia Eubacteriales Petroclostridium xylanilyticum WP 094549045.1 318 aa
Bacillati Bacillota Clostridia Eubacteriales Petroclostridium sp. WP 282863940.1 318 aa
Bacillati Bacillota Clostridia Clostridia incertae sedis Candidatus Fimenecus WP 178689195.1 319 aa
Bacillati Bacillota Clostridia Christensenellales Christensenellales bacterium MFR6640824.1 319 aa
Bacillati Bacillota Clostridia Clostridia incertae sedis Candidatus Protoclostridium HIW03010.1 323 aa
Bacillati Bacillota Clostridia Eubacteriales Pumilibacter intestinalis WP 251548665.1 320 aa
Bacillati Bacillota Clostridia Eubacteriales Anaerocaecibacter muris WP 251182400.1 321 aa
Bacillati Bacillota Clostridia Christensenellales Christensenellaceae bacterium MDY6024590.1 317 aa
Bacillati Bacillota Clostridia Christensenellales Candidatus Caballimonas MBQ0098978.1 317 aa
Bacillati Bacillota Clostridia Christensenellales Christensenellaceae bacterium MDY4676238.1 323 aa
Bacillati Bacillota Clostridia Christensenellales Christensenellaceae bacterium MDY4186603.1 316 aa
Bacillati Bacillota Clostridia Lachnospirales Roseburia sp. MCM1438207.1 315 aa
Bacillati Bacillota Clostridia Christensenellales Candidatus Borkfalkia WP 129223195.1 317 aa
Bacillati Bacillota Clostridia Christensenellales Gehongia tenuis WP 249316807.1 318 aa
Bacillati Bacillota Clostridia Clostridia incertae sedis Candidatus Faecaligallichristensenella HIT00257.1 318 aa
Bacillati Bacillota Clostridia Clostridia incertae sedis Candidatus Alectryocaccomicrobium HIS92886.1 317 aa
Bacillati Bacillota Clostridia Eubacteriales Candidatus Galloscillospira HIU71295.1 323 aa
Bacillati Bacillota Clostridia Christensenellales Luoshenia tenuis WP 249285742.1 316 aa
Bacillati Bacillota Clostridia Christensenellales Christensenellales bacterium MGI6192297.1 324 aa
Bacillati Bacillota Clostridia Christensenellales Christensenellales bacterium MGI6160500.1 317 aa
Bacillati Bacillota Clostridia Thermoanaerobacterales Calorimonas adulescens WP 420837948.1 316 aa
Bacillati Bacillota Clostridia Thermoanaerobacterales Caldanaerobius fijiensis WP 073344055.1 315 aa
Bacillati Bacillota Clostridia Thermoanaerobacterales Caldanaerobacter WP 011026572.1 316 aa
Bacillati Bacillota Clostridia Thermoanaerobacterales Aceticella autotrophica WP 284680168.1 316 aa
Bacillati Bacillota Clostridia Thermoanaerobacterales Mahella australiensis WP 148258467.1 316 aa
Bacillati Bacillota Clostridia Caldicoprobacterales Caldicoprobacterales bacterium MGI6571748.1 318 aa
Bacillati Bacillota Clostridia Caldicoprobacterales Caldicoprobacterales bacterium MGI6373361.1 317 aa
Bacillati Bacillota Clostridia Caldicoprobacterales Caldicoprobacteraceae bacterium MGI6188564.1 317 aa
Bacillati Bacillota Clostridia Caldicoprobacterales Caldicoprobacter algeriensis WP 252228656.1 317 aa
Bacillati Bacillota Clostridia Eubacteriales Syntrophothermus WP 013174309.1 316 aa
Bacillati Bacillota Clostridia Eubacteriales Thermosyntropha lipolytica WP 073089042.1 316 aa
Bacillati Bacillota Clostridia Thermoanaerobacterales Gelria sp. HHV15827.1 317 aa
Bacillati Bacillota Clostridia Eubacteriales Syntrophomonas zehnderi WP 046495321.1 317 aa
Bacillati Bacillota Clostridia Eubacteriales Syntrophomonas wolfei WP 011639537.1 317 aa
Bacillati Bacillota Clostridia Eubacteriales Candidatus Syntrophocurvum WP 156204313.1 318 aa
Bacillati Bacillota Clostridia Neomoorellales Desulfitibacter alkalitolerans WP 028306559.1 315 aa
Bacillati Bacillota Clostridia Neomoorellales Neomoorella sulfitireducens WP 258360699.1 315 aa
Bacillati Bacillota Clostridia Neomoorellales Moorella sp. WP 109208110.1 315 aa
Bacillati Bacillota Clostridia Neomoorellales Thermanaeromonas sp. WP 173296841.1 315 aa
Bacillati Bacillota Clostridia Neomoorellales Thermanaeromonas sp. WP 297199963.1 314 aa
Bacillati Bacillota Clostridia Neomoorellales Moorellaceae bacterium MGB9660441.1 315 aa
Bacillati Bacillota Clostridia Neomoorellales Metallumcola ferriviriculae WP 366923352.1 315 aa
Bacillati Bacillota Clostridia Neomoorellales Calderihabitans maritimus WP 088553158.1 315 aa
Bacillati Bacillota Clostridia Neomoorellales Zhaonellaceae bacterium MGI6330844.1 315 aa
Bacillati Bacillota Clostridia Neomoorellales Zhaonella formicivorans WP 227763966.1 315 aa
Bacillati Bacillota Clostridia Eubacteriales Dehalobacterium formicoaceticum WP 089609374.1 315 aa
Bacillati Bacillota Clostridia Eubacteriales Candidatus Formimonas WP 148136315.1 315 aa
Bacillati Bacillota Desulfitisporia Desulfitispora alkaliphila WP 429158042.1 315 aa
Bacillati Bacillota Clostridia Eubacteriales Desulfitobacterium sp. NMA69023.1 313 aa
Bacillati Bacillota Clostridia Eubacteriales Desulfitobacterium metallireducens WP 006717607.1 313 aa
Bacillati Bacillota Clostridia Eubacteriales Paradesulfitobacterium ferrireducens WP 206811376.1 313 aa
Bacillati Bacillota Clostridia Eubacteriales Desulfitobacteriaceae bacterium MDI6812107.1 313 aa
Bacillati Bacillota Clostridia Eubacteriales Acididesulfobacillus acetoxydans WP 240983645.1 313 aa
Bacillati Bacillota Clostridia Eubacteriales Desulfosporosinus sp. WP 034599356.1 312 aa
Bacillati Bacillota Clostridia Eubacteriales Desulfosporosinus fructosivorans WP 135549502.1 311 aa
Bacillati Bacillota Clostridia Eubacteriales Dehalobacter WP 019224828.1 313 aa
Bacillati Bacillota Clostridia Eubacteriales Dehalobacter sp. WP 337986559.1 313 aa
Bacillati Bacillota Clostridia Eubacteriales Syntrophobotulus glycolicus WP 013623437.1 313 aa
Bacillati Bacillota Clostridia Eubacteriales Gracilibacteraceae bacterium MDR1603502.1 314 aa
Bacillati Bacillota Clostridia Eubacteriales Gracilibacteraceae bacterium MDR0433890.1 314 aa

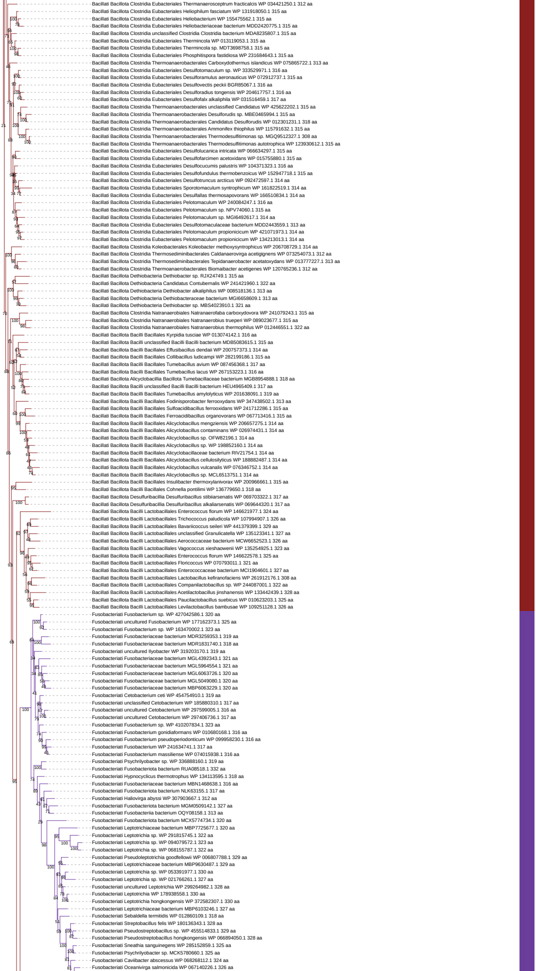
Bacillati Bacillota Clostridia Eubacteriales Thermanaerosceptrum fracticalcis WP 034421250.1 312 aa
Bacillati Bacillota Clostridia Eubacteriales Heliophilum fasciatum WP 131918050.1 315 aa
Bacillati Bacillota Clostridia Eubacteriales Heliobacterium WP 155475562.1 315 aa
Bacillati Bacillota Clostridia Eubacteriales Heliobacteriaceae bacterium MDD2420775.1 315 aa
Bacillati Bacillota Clostridia unclassified Clostridia Clostridia bacterium MDA8235807.1 315 aa
Bacillati Bacillota Clostridia Eubacteriales Thermincola WP 013119053.1 315 aa
Bacillati Bacillota Clostridia Eubacteriales Thermincola sp. MDT3698758.1 315 aa
Bacillati Bacillota Clostridia Eubacteriales Phosphitispora fastidiosa WP 231684643.1 315 aa
Bacillati Bacillota Clostridia Thermoanaerobacterales Carboxydothermus islandicus WP 075865722.1 313 aa
Bacillati Bacillota Clostridia Eubacteriales Desulfotomaculum sp. WP 333529971.1 316 aa
Bacillati Bacillota Clostridia Eubacteriales Desulforamulus aeronauticus WP 072912737.1 315 aa
Bacillati Bacillota Clostridia Eubacteriales Desulfovectis peckii BGR85067.1 316 aa
Bacillati Bacillota Clostridia Eubacteriales Desulforadius tongensis WP 204617757.1 316 aa
Bacillati Bacillota Clostridia Eubacteriales Desulfofalx alkaliphila WP 031516459.1 317 aa
Bacillati Bacillota Clostridia Thermoanaerobacterales unclassified Candidatus WP 425622202.1 315 aa
Bacillati Bacillota Clostridia Thermoanaerobacterales Desulforudis sp. MBE0465994.1 315 aa
Bacillati Bacillota Clostridia Thermoanaerobacterales Candidatus Desulforudis WP 012301231.1 318 aa
Bacillati Bacillota Clostridia Thermoanaerobacterales Ammonifex thiophilus WP 115791632.1 315 aa
Bacillati Bacillota Clostridia Thermoanaerobacterales Thermodesulfitimonas sp. MGQ9512327.1 308 aa
Bacillati Bacillota Clostridia Thermoanaerobacterales Thermodesulfitimonas autotrophica WP 123930612.1 315 aa
Bacillati Bacillota Clostridia Eubacteriales Desulfolucanica intricata WP 066634297.1 315 aa
Bacillati Bacillota Clostridia Eubacteriales Desulfofarcimen acetoxidans WP 015755880.1 315 aa
Bacillati Bacillota Clostridia Eubacteriales Desulfocucumis palustris WP 104371323.1 316 aa
Bacillati Bacillota Clostridia Eubacteriales Desulfofundulus thermobenzoicus WP 152947718.1 315 aa
Bacillati Bacillota Clostridia Eubacteriales Desulfotruncus arcticus WP 092472597.1 314 aa
Bacillati Bacillota Clostridia Eubacteriales Sporotomaculum syntrophicum WP 161822519.1 314 aa
Bacillati Bacillota Clostridia Eubacteriales Desulfallas thermosapovorans WP 166510834.1 314 aa
Bacillati Bacillota Clostridia Eubacteriales Pelotomaculum WP 240084247.1 316 aa
Bacillati Bacillota Clostridia Eubacteriales Pelotomaculum sp. NPV74060.1 315 aa
Bacillati Bacillota Clostridia Eubacteriales Pelotomaculum sp. MGI6492617.1 314 aa
Bacillati Bacillota Clostridia Eubacteriales Desulfotomaculaceae bacterium MDD2443559.1 313 aa
Bacillati Bacillota Clostridia Eubacteriales Pelotomaculum propionicicum WP 421071973.1 314 aa
Bacillati Bacillota Clostridia Eubacteriales Pelotomaculum propionicicum WP 134213013.1 314 aa
Bacillati Bacillota Clostridia Koleobacterales Koleobacter methoxysyntrophicus WP 206708729.1 314 aa
Bacillati Bacillota Clostridia Thermosediminibacterales Caldanaerovirga acetigignens WP 073254073.1 312 aa
Bacillati Bacillota Clostridia Thermosediminibacterales Tepidanaerobacter acetatoxydans WP 013777227.1 313 aa
Bacillati Bacillota Clostridia Thermoanaerobacterales Biomaibacter acetigenes WP 120765236.1 312 aa
Bacillati Bacillota Dethiobacteria Dethiobacter sp. RJX24749.1 315 aa
Bacillati Bacillota Dethiobacteria Candidatus Contubernalis WP 241421960.1 322 aa
Bacillati Bacillota Dethiobacteria Dethiobacter alkaliphilus WP 008518136.1 313 aa
Bacillati Bacillota Dethiobacteria Dethiobacteraceae bacterium MGI6658609.1 313 aa
Bacillati Bacillota Dethiobacteria Dethiobacter sp. MBS4023910.1 321 aa
Bacillati Bacillota Clostridia Natranaerobiales Natranaerofaba carboxydovora WP 241079243.1 315 aa
Bacillati Bacillota Clostridia Natranaerobiales Natranaerobius trueperi WP 089023677.1 315 aa
Bacillati Bacillota Clostridia Natranaerobiales Natranaerobius thermophilus WP 012446551.1 322 aa
Bacillati Bacillota Bacilli Bacillales Kyrpidia tusciae WP 013074142.1 316 aa
Bacillati Bacillota Bacilli unclassified Bacilli Bacilli bacterium MDB5083615.1 315 aa
Bacillati Bacillota Bacilli Bacillales Effusibacillus dendaii WP 200757373.1 314 aa
Bacillati Bacillota Bacilli Bacillales Collibacillus ludicampi WP 282199186.1 315 aa
Bacillati Bacillota Bacilli Bacillales Tumebacillus avium WP 087456368.1 317 aa
Bacillati Bacillota Bacilli Bacillales Tumebacillus lacus WP 267153223.1 316 aa
Bacillati Bacillota Alicyclobacillia Bacillota Tumebacillaceae bacterium MGB8954888.1 318 aa
Bacillati Bacillota Bacilli unclassified Bacilli Bacilli bacterium HEU4965409.1 317 aa
Bacillati Bacillota Bacilli Bacillales Tumebacillus amylolyticus WP 201638091.1 319 aa
Bacillati Bacillota Bacilli Bacillales Fodinisporobacter ferrooxydans WP 347438502.1 313 aa
Bacillati Bacillota Bacilli Bacillales Sulfoacidibacillus ferrooxidans WP 241712286.1 315 aa
Bacillati Bacillota Bacilli Bacillales Ferroacidibacillus organovorans WP 067713416.1 315 aa
Bacillati Bacillota Bacilli Bacillales Alicyclobacillus mengziensis WP 206657275.1 314 aa
Bacillati Bacillota Bacilli Bacillales Alicyclobacillus contaminans WP 026974431.1 314 aa
Bacillati Bacillota Bacilli Bacillales Alicyclobacillus sp. OFW82196.1 314 aa
Bacillati Bacillota Bacilli Bacillales Alicyclobacillus sp. WP 198852160.1 314 aa
Bacillati Bacillota Bacilli Bacillales Alicyclobacillaceae bacterium RIV21754.1 314 aa
Bacillati Bacillota Bacilli Bacillales Alicyclobacillus cellulosilyticus WP 188882487.1 314 aa
Bacillati Bacillota Bacilli Bacillales Alicyclobacillus vulcanalis WP 076346752.1 314 aa
Bacillati Bacillota Bacilli Bacillales Alicyclobacillus sp. MCL6513751.1 314 aa
Bacillati Bacillota Bacilli Bacillales Insulibacter thermoxylanivorax WP 200966661.1 315 aa
Bacillati Bacillota Bacilli Bacillales Cohnella pontilimi WP 136779650.1 318 aa
Bacillati Bacillota Desulfuribacillia Desulfuribacillus stibiiarsenatis WP 069703322.1 317 aa
Bacillati Bacillota Desulfuribacillia Desulfuribacillus alkaliarsenatis WP 069644320.1 317 aa
Bacillati Bacillota Bacilli Lactobacillales Enterococcus florum WP 146621977.1 324 aa
Bacillati Bacillota Bacilli Lactobacillales Trichococcus paludicola WP 107994907.1 326 aa
Bacillati Bacillota Bacilli Lactobacillales Bavariicoccus seileri WP 441379399.1 329 aa
Bacillati Bacillota Bacilli Lactobacillales unclassified Granulicatella WP 135123341.1 327 aa
Bacillati Bacillota Bacilli Lactobacillales Aerococcaceae bacterium MCW6652523.1 326 aa
Bacillati Bacillota Bacilli Lactobacillales Vagococcus xieshaowenii WP 135254925.1 323 aa
Bacillati Bacillota Bacilli Lactobacillales Enterococcus florum WP 146622578.1 325 aa
Bacillati Bacillota Bacilli Lactobacillales Floricoccus WP 070793011.1 321 aa
Bacillati Bacillota Bacilli Lactobacillales Enterococcaceae bacterium MCI1904601.1 327 aa
Bacillati Bacillota Bacilli Lactobacillales Lactobacillus kefiranofaciens WP 261912176.1 308 aa
Bacillati Bacillota Bacilli Lactobacillales Companilactobacillus sp. WP 244087001.1 322 aa
Bacillati Bacillota Bacilli Lactobacillales Acetilactobacillus jinshanensis WP 133442439.1 328 aa
Bacillati Bacillota Bacilli Lactobacillales Paucilactobacillus suebicus WP 010623203.1 325 aa
Bacillati Bacillota Bacilli Lactobacillales Levilactobacillus bambusae WP 109251128.1 326 aa
Fusobacteriati Fusobacterium sp. WP 427042586.1 320 aa
Fusobacteriati uncultured Fusobacterium WP 177162373.1 325 aa
Fusobacteriati Fusobacterium sp. WP 163470002.1 323 aa
Fusobacteriati Fusobacteriaceae bacterium MDR3259353.1 319 aa
Fusobacteriati Fusobacteriaceae bacterium MDR1831740.1 318 aa
Fusobacteriati uncultured Ilyobacter WP 319203170.1 319 aa
Fusobacteriati Fusobacteriaceae bacterium MGL4392343.1 321 aa
Fusobacteriati Fusobacteriaceae bacterium MGL5964554.1 321 aa
Fusobacteriati Fusobacteriaceae bacterium MGL6063726.1 320 aa
Fusobacteriati Fusobacteriaceae bacterium MGL5049080.1 320 aa
Fusobacteriati Fusobacteriaceae bacterium MBP6063229.1 320 aa
Fusobacteriati Cetobacterium ceti WP 454754910.1 319 aa
Fusobacteriati unclassified Cetobacterium WP 185880310.1 317 aa
Fusobacteriati uncultured Cetobacterium WP 297599005.1 316 aa
Fusobacteriati uncultured Cetobacterium WP 297406736.1 317 aa
Fusobacteriati Fusobacterium sp. WP 410207834.1 323 aa
Fusobacteriati Fusobacterium gonidiaformans WP 010680168.1 316 aa
Fusobacteriati Fusobacterium pseudoperiodonticum WP 099958230.1 316 aa
Fusobacteriati Fusobacterium WP 241634741.1 317 aa
Fusobacteriati Fusobacterium massiliense WP 074015938.1 316 aa
Fusobacteriati Psychrilyobacter sp. WP 336888160.1 319 aa
Fusobacteriati Fusobacteriota bacterium RUA08518.1 332 aa
Fusobacteriati Hypnocyclicus thermotrophus WP 134113595.1 318 aa
Fusobacteriati Fusobacteriaceae bacterium MBN1468638.1 316 aa
Fusobacteriati Fusobacteriota bacterium NLK63155.1 317 aa
Fusobacteriati Haliovirga abyssi WP 307903667.1 312 aa
Fusobacteriati Fusobacteriota bacterium MGM0509142.1 327 aa
Fusobacteriati Fusobacteriia bacterium OQY08158.1 313 aa
Fusobacteriati Fusobacteriota bacterium MCX5774734.1 320 aa
Fusobacteriati Leptotrichiaceae bacterium MBP7725677.1 320 aa
Fusobacteriati Leptotrichia sp. WP 291815745.1 322 aa
Fusobacteriati Leptotrichia sp. WP 094079572.1 323 aa
Fusobacteriati Leptotrichia sp. WP 068155787.1 322 aa
Fusobacteriati Pseudoleptotrichia goodfellowii WP 006807788.1 329 aa
Fusobacteriati Leptotrichiaceae bacterium MBP9630487.1 329 aa
Fusobacteriati Leptotrichia sp. WP 053391977.1 330 aa
Fusobacteriati Leptotrichia sp. WP 021766261.1 327 aa
Fusobacteriati uncultured Leptotrichia WP 299264982.1 328 aa
Fusobacteriati Leptotrichia WP 178938558.1 330 aa
Fusobacteriati Leptotrichia hongkongensis WP 372582307.1 330 aa
Fusobacteriati Leptotrichiaceae bacterium MBP6103246.1 327 aa
Fusobacteriati Sebaldella termitidis WP 012860109.1 318 aa
Fusobacteriati Streptobacillus felis WP 180136343.1 328 aa
Fusobacteriati Pseudostreptobacillus sp. WP 455514833.1 329 aa
Fusobacteriati Pseudostreptobacillus hongkongensis WP 066894050.1 328 aa
Fusobacteriati Sneathia sanguinegens WP 285152859.1 325 aa
Fusobacteriati Psychrilyobacter sp. MCK5780660.1 325 aa
Fusobacteriati Caviibacter abscessus WP 068268112.1 324 aa
Fusobacteriati Oceanivirga salmonicida WP 067140226.1 326 aa

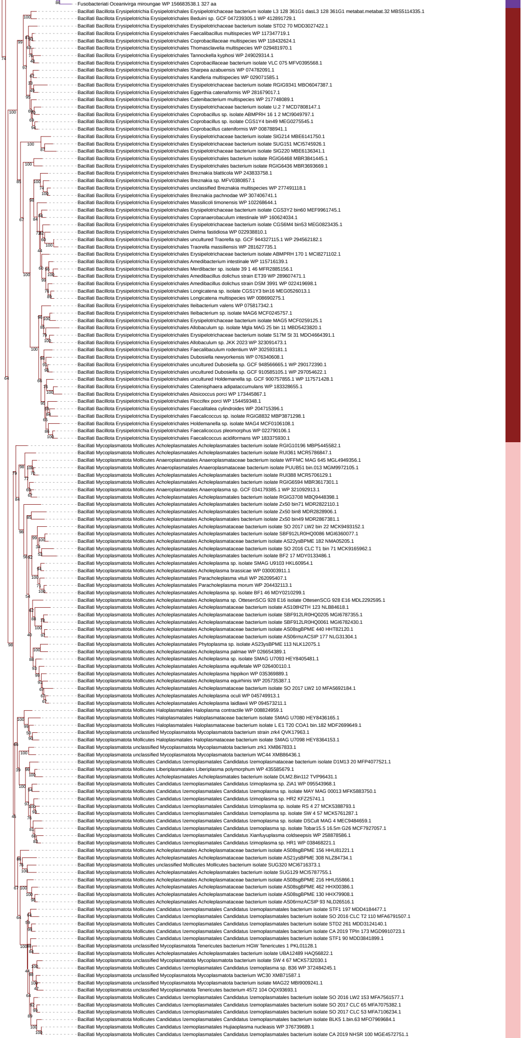

Fusobacteriati Oceanivirga miroungae WP 156683538.1 327 aa
Bacillati Bacillota Erysipelotrichia Erysipelotrichales Erysipelotrichaceae bacterium isolate L3 128 361G1 dasL3 128 361G1 metabat.metabat.32 MBS5114335.1
Bacillati Bacillota Erysipelotrichia Erysipelotrichales Beduini sp. GCF 047239305.1 WP 412891729.1
Bacillati Bacillota Erysipelotrichia Erysipelotrichales Erysipelotrichaceae bacterium isolate STD2 70 MDD3027422.1
Bacillati Bacillota Erysipelotrichia Erysipelotrichales Faecalibacillus multispecies WP 117347719.1
Bacillati Bacillota Erysipelotrichia Erysipelotrichales Coprobacillaceae multispecies WP 118432624.1
Bacillati Bacillota Erysipelotrichia Erysipelotrichales Thomasclavelia multispecies WP 029481970.1
Bacillati Bacillota Erysipelotrichia Erysipelotrichales Tannockella kyphosi WP 249029314.1
Bacillati Bacillota Erysipelotrichia Erysipelotrichales Coprobacillaceae bacterium isolate VLC 075 MFV0395568.1
Bacillati Bacillota Erysipelotrichia Erysipelotrichales Sharpea azabuensis WP 074782091.1
Bacillati Bacillota Erysipelotrichia Erysipelotrichales Kandleria multispecies WP 029071585.1
Bacillati Bacillota Erysipelotrichia Erysipelotrichales Erysipelotrichaceae bacterium isolate RGIG9341 MBO6047387.1
Bacillati Bacillota Erysipelotrichia Erysipelotrichales Eggerthia catenaformis WP 281679017.1
Bacillati Bacillota Erysipelotrichia Erysipelotrichales Catenibacterium multispecies WP 217748089.1
Bacillati Bacillota Erysipelotrichia Erysipelotrichales Erysipelotrichaceae bacterium isolate U.2 7 MCD7808147.1
Bacillati Bacillota Erysipelotrichia Erysipelotrichales Coprobacillus sp. isolate ABMPRH 16 1 2 MCI9049797.1
Bacillati Bacillota Erysipelotrichia Erysipelotrichales Coprobacillus sp. isolate CGS1Y4 bin49 MEG0275545.1
Bacillati Bacillota Erysipelotrichia Erysipelotrichales Coprobacillus cateniformis WP 008788941.1
Bacillati Bacillota Erysipelotrichia Erysipelotrichales Erysipelotrichaceae bacterium isolate SIG214 MBE6141750.1
Bacillati Bacillota Erysipelotrichia Erysipelotrichales Erysipelotrichaceae bacterium isolate SUG151 MCI5745926.1
Bacillati Bacillota Erysipelotrichia Erysipelotrichales Erysipelotrichaceae bacterium isolate SIG220 MBE6136341.1
Bacillati Bacillota Erysipelotrichia Erysipelotrichales Erysipelotrichales bacterium isolate RGIG6468 MBR3841445.1
Bacillati Bacillota Erysipelotrichia Erysipelotrichales Erysipelotrichales bacterium isolate RGIG6436 MBR3693669.1
Bacillati Bacillota Erysipelotrichia Erysipelotrichales Breznakia blatticola WP 243833758.1
Bacillati Bacillota Erysipelotrichia Erysipelotrichales Breznakia sp. MFV0380857.1
Bacillati Bacillota Erysipelotrichia Erysipelotrichales unclassified Breznakia multispecies WP 277491118.1
Bacillati Bacillota Erysipelotrichia Erysipelotrichales Breznakia pachnodae WP 307406741.1
Bacillati Bacillota Erysipelotrichia Erysipelotrichales Massilicoli timonensis WP 102268644.1
Bacillati Bacillota Erysipelotrichia Erysipelotrichales Erysipelotrichaceae bacterium isolate CGS3Y2 bin60 MEF9961745.1
Bacillati Bacillota Erysipelotrichia Erysipelotrichales Copranaerobaculum intestinale WP 160624034.1
Bacillati Bacillota Erysipelotrichia Erysipelotrichales Erysipelotrichaceae bacterium isolate CGS6M4 bin53 MEG0823435.1
Bacillati Bacillota Erysipelotrichia Erysipelotrichales Dielma fastidiosa WP 022938810.1
Bacillati Bacillota Erysipelotrichia Erysipelotrichales uncultured Traorella sp. GCF 944327115.1 WP 294562182.1
Bacillati Bacillota Erysipelotrichia Erysipelotrichales Traorella massiliensis WP 281627735.1
Bacillati Bacillota Erysipelotrichia Erysipelotrichales Erysipelotrichaceae bacterium isolate ABMPRH 170 1 MCI8271102.1
Bacillati Bacillota Erysipelotrichia Erysipelotrichales Amedibacterium intestinale WP 115716139.1
Bacillati Bacillota Erysipelotrichia Erysipelotrichales Merdibacter sp. isolate 39 1 46 MFR2885156.1
Bacillati Bacillota Erysipelotrichia Erysipelotrichales Amedibacillus dolichus strain ET39 WP 289607471.1
Bacillati Bacillota Erysipelotrichia Erysipelotrichales Amedibacillus dolichus strain DSM 3991 WP 022419698.1
Bacillati Bacillota Erysipelotrichia Erysipelotrichales Longicatena sp. isolate CGS1Y3 bin16 MEG0526013.1
Bacillati Bacillota Erysipelotrichia Erysipelotrichales Longicatena multispecies WP 008690275.1
Bacillati Bacillota Erysipelotrichia Erysipelotrichales Ileibacterium valens WP 075817342.1
Bacillati Bacillota Erysipelotrichia Erysipelotrichales Ileibacterium sp. isolate MAG6 MCF0245757.1
Bacillati Bacillota Erysipelotrichia Erysipelotrichales Erysipelotrichaceae bacterium isolate MAG5 MCF0259125.1
Bacillati Bacillota Erysipelotrichia Erysipelotrichales Allobaculum sp. isolate Mgla MAG 25 bin 11 MBD5423820.1
Bacillati Bacillota Erysipelotrichia Erysipelotrichales Erysipelotrichaceae bacterium isolate S17M St 31 MDO4664391.1
Bacillati Bacillota Erysipelotrichia Erysipelotrichales Allobaculum sp. JKK 2023 WP 323091473.1
Bacillati Bacillota Erysipelotrichia Erysipelotrichales Faecalibaculum rodentium WP 302593181.1
Bacillati Bacillota Erysipelotrichia Erysipelotrichales Dubosiella newyorkensis WP 076340608.1
Bacillati Bacillota Erysipelotrichia Erysipelotrichales uncultured Dubosiella sp. GCF 948566665.1 WP 290172390.1
Bacillati Bacillota Erysipelotrichia Erysipelotrichales uncultured Dubosiella sp. GCF 910585105.1 WP 297054622.1
Bacillati Bacillota Erysipelotrichia Erysipelotrichales uncultured Holdemanella sp. GCF 900757855.1 WP 117571428.1
Bacillati Bacillota Erysipelotrichia Erysipelotrichales Catenisphaera adipataccumulans WP 183328655.1
Bacillati Bacillota Erysipelotrichia Erysipelotrichales Absicoccus porci WP 173445867.1
Bacillati Bacillota Erysipelotrichia Erysipelotrichales Floccifex porci WP 154459348.1
Bacillati Bacillota Erysipelotrichia Erysipelotrichales Faecalitalea cylindroides WP 204715396.1
Bacillati Bacillota Erysipelotrichia Erysipelotrichales Faecalicoccus sp. isolate RGIG8832 MBP3871298.1
Bacillati Bacillota Erysipelotrichia Erysipelotrichales Holdemanella sp. isolate MAG4 MCF0106108.1
Bacillati Bacillota Erysipelotrichia Erysipelotrichales Faecalicoccus pleomorphus WP 022790106.1
Bacillati Bacillota Erysipelotrichia Erysipelotrichales Faecalicoccus acidiformans WP 183375930.1
Bacillati Mycoplasmatota Mollicutes Acholeplasmatales Acholeplasmatales bacterium isolate RGIG10196 MBP5445582.1
Bacillati Mycoplasmatota Mollicutes Acholeplasmatales Acholeplasmatales bacterium isolate RUI361 MCR5786847.1
Bacillati Mycoplasmatota Mollicutes Anaeroplasmatales Anaeroplasmataceae bacterium isolate WFFMC MAG 645 MGL4949356.1
Bacillati Mycoplasmatota Mollicutes Anaeroplasmatales Anaeroplasmataceae bacterium isolate PUUB51 bin.013 MGM9972105.1
Bacillati Mycoplasmatota Mollicutes Acholeplasmatales Acholeplasmatales bacterium isolate RUI388 MCR5706129.1
Bacillati Mycoplasmatota Mollicutes Acholeplasmatales Acholeplasmatales bacterium isolate RGIG6594 MBR3617301.1
Bacillati Mycoplasmatota Mollicutes Anaeroplasmatales Anaeroplasma sp. GCF 034179385.1 WP 321092913.1
Bacillati Mycoplasmatota Mollicutes Acholeplasmatales Acholeplasmatales bacterium isolate RGIG3708 MBQ9448398.1
Bacillati Mycoplasmatota Mollicutes Acholeplasmatales Acholeplasmatales bacterium isolate Zx50 bin71 MDR2822110.1
Bacillati Mycoplasmatota Mollicutes Acholeplasmatales Acholeplasmatales bacterium isolate Zx50 bin8 MDR2828906.1
Bacillati Mycoplasmatota Mollicutes Acholeplasmatales Acholeplasmatales bacterium isolate Zx50 bin49 MDR2867381.1
Bacillati Mycoplasmatota Mollicutes Acholeplasmatales Acholeplasmataceae bacterium isolate SO 2017 LW2 bin 22 MCK9493152.1
Bacillati Mycoplasmatota Mollicutes Acholeplasmatales Acholeplasmataceae bacterium isolate SBF912LR0HQ0086 MGI6360077.1
Bacillati Mycoplasmatota Mollicutes Acholeplasmatales Acholeplasmataceae bacterium isolate AS22ysBPME 182 NMA05205.1
Bacillati Mycoplasmatota Mollicutes Acholeplasmatales Acholeplasmataceae bacterium isolate SO 2016 CLC T1 bin 71 MCK9165962.1
Bacillati Mycoplasmatota Mollicutes Acholeplasmatales Acholeplasmatales bacterium isolate BF2 17 MDY0133486.1
Bacillati Mycoplasmatota Mollicutes Acholeplasmatales Acholeplasma sp. isolate SMAG U9103 HKL60954.1
Bacillati Mycoplasmatota Mollicutes Acholeplasmatales Acholeplasma brassicae WP 030003911.1
Bacillati Mycoplasmatota Mollicutes Acholeplasmatales Paracholeplasma vituli WP 262095407.1
Bacillati Mycoplasmatota Mollicutes Acholeplasmatales Paracholeplasma morum WP 204432113.1
Bacillati Mycoplasmatota Mollicutes Acholeplasmatales Acholeplasma sp. isolate BF1 46 MDY0210299.1
Bacillati Mycoplasmatota Mollicutes Acholeplasmatales Acholeplasma sp. OttesenSCG 928 E16 isolate OttesenSCG 928 E16 MDL2292595.1
Bacillati Mycoplasmatota Mollicutes Acholeplasmatales Acholeplasmataceae bacterium isolate AS10tlH2TH 123 NLB84618.1
Bacillati Mycoplasmatota Mollicutes Acholeplasmatales Acholeplasmataceae bacterium isolate SBF912LR0HQ0205 MGI6787355.1
Bacillati Mycoplasmatota Mollicutes Acholeplasmatales Acholeplasmataceae bacterium isolate SBF912LR0HQ0061 MGI6782430.1
Bacillati Mycoplasmatota Mollicutes Acholeplasmatales Acholeplasmataceae bacterium isolate AS08sgBPME 440 HHT82120.1
Bacillati Mycoplasmatota Mollicutes Acholeplasmatales Acholeplasmataceae bacterium isolate AS06rmzACSIP 177 NLG31304.1
Bacillati Mycoplasmatota Mollicutes Acholeplasmatales Phytoplasma sp. isolate AS23ysBPME 113 NLK12075.1
Bacillati Mycoplasmatota Mollicutes Acholeplasmatales Acholeplasma palmae WP 026654389.1
Bacillati Mycoplasmatota Mollicutes Acholeplasmatales Acholeplasma sp. isolate SMAG U7093 HEY8405481.1
Bacillati Mycoplasmatota Mollicutes Acholeplasmatales Acholeplasma equifetale WP 026400110.1
Bacillati Mycoplasmatota Mollicutes Acholeplasmatales Acholeplasma hippikon WP 035369889.1
Bacillati Mycoplasmatota Mollicutes Acholeplasmatales Acholeplasma equirhinis WP 205735387.1
Bacillati Mycoplasmatota Mollicutes Acholeplasmatales Acholeplasmataceae bacterium isolate SO 2017 LW2 10 MFA5692184.1
Bacillati Mycoplasmatota Mollicutes Acholeplasmatales Acholeplasma oculi WP 045749913.1
Bacillati Mycoplasmatota Mollicutes Acholeplasmatales Acholeplasma laidlawii WP 094573211.1
Bacillati Mycoplasmatota Mollicutes Haloplasmatales Haloplasma contractile WP 008824959.1
Bacillati Mycoplasmatota Mollicutes Haloplasmatales Haloplasmataceae bacterium isolate SMAG U7080 HEY8436165.1
Bacillati Mycoplasmatota Mollicutes Haloplasmatales Haloplasmataceae bacterium isolate L E1 T20 COA1 bin.182 MDF2699649.1
Bacillati Mycoplasmatota unclassified Mycoplasmatota Mycoplasmatota bacterium strain zrk4 QVK17963.1
Bacillati Mycoplasmatota Mollicutes Haloplasmatales Haloplasmataceae bacterium isolate SMAG U7098 HEY8364153.1
Bacillati Mycoplasmatota unclassified Mycoplasmatota Mycoplasmatota bacterium zrk1 XMB67833.1
Bacillati Mycoplasmatota unclassified Mycoplasmatota Mycoplasmatota bacterium WC44 XMB86436.1
Bacillati Mycoplasmatota Mollicutes Candidatus Izemoplasmatales Candidatus Izemoplasmataceae bacterium isolate D1M13 20 MFP4077521.1
Bacillati Mycoplasmatota Mollicutes Liberiplasmatales Liberiplasma polymorphum WP 435585679.1
Bacillati Mycoplasmatota Mollicutes Acholeplasmatales Acholeplasmatales bacterium isolate DLM2.Bin112 TVP96431.1
Bacillati Mycoplasmatota Mollicutes Candidatus Izemoplasmatales Candidatus Izimoplasma sp. ZiA1 WP 095543968.1
Bacillati Mycoplasmatota Mollicutes Candidatus Izemoplasmatales Candidatus Izemoplasma sp. isolate MAY MAG 00013 MFK5883750.1
Bacillati Mycoplasmatota Mollicutes Candidatus Izemoplasmatales Candidatus Izimoplasma sp. HR2 KFZ25741.1
Bacillati Mycoplasmatota Mollicutes Candidatus Izemoplasmatales Candidatus Izimoplasma sp. isolate RS 4 27 MCK5388793.1
Bacillati Mycoplasmatota Mollicutes Candidatus Izemoplasmatales Candidatus Izimoplasma sp. isolate SW 4 57 MCK5761287.1
Bacillati Mycoplasmatota Mollicutes Candidatus Izemoplasmatales Candidatus Izemoplasma sp. isolate DSCult MAG 4 MEC9484659.1
Bacillati Mycoplasmatota Mollicutes Candidatus Izemoplasmatales Candidatus Izemoplasma sp. isolate Tobar15.5 16.5m G26 MCF7927057.1
Bacillati Mycoplasmatota Mollicutes Candidatus Izemoplasmatales Candidatus Xianfuyuplasma coldseepsis WP 258878586.1
Bacillati Mycoplasmatota Mollicutes Candidatus Izemoplasmatales Candidatus Izimoplasma sp. HR1 WP 038468221.1
Bacillati Mycoplasmatota Mollicutes Acholeplasmatales Acholeplasmataceae bacterium isolate AS08sgBPME 156 HHU81221.1
Bacillati Mycoplasmatota Mollicutes Acholeplasmatales Acholeplasmataceae bacterium isolate AS21ysBPME 308 NLZ84734.1
Bacillati Mycoplasmatota Mollicutes unclassified Mollicutes Mollicutes bacterium isolate SUG320 MCI6716373.1
Bacillati Mycoplasmatota Mollicutes Acholeplasmatales Acholeplasmatales bacterium isolate SUG129 MCI5787755.1
Bacillati Mycoplasmatota Mollicutes Acholeplasmatales Acholeplasmataceae bacterium isolate AS08sgBPME 216 HHU55866.1
Bacillati Mycoplasmatota Mollicutes Acholeplasmatales Acholeplasmataceae bacterium isolate AS08sgBPME 462 HHX00386.1
Bacillati Mycoplasmatota Mollicutes Acholeplasmatales Acholeplasmataceae bacterium isolate AS08sgBPME 130 HHX79908.1
Bacillati Mycoplasmatota Mollicutes Acholeplasmatales Acholeplasmataceae bacterium isolate AS06rmzACSIP 93 NLD26516.1
Bacillati Mycoplasmatota Mollicutes Candidatus Izemoplasmatales Candidatus Izemoplasmatales bacterium isolate STF1 197 MDD4184477.1
Bacillati Mycoplasmatota Mollicutes Candidatus Izemoplasmatales Candidatus Izemoplasmatales bacterium isolate SO 2016 CLC T2 110 MFA6791507.1
Bacillati Mycoplasmatota Mollicutes Candidatus Izemoplasmatales Candidatus Izemoplasmatales bacterium isolate STD2 261 MDD3124140.1
Bacillati Mycoplasmatota Mollicutes Candidatus Izemoplasmatales Candidatus Izemoplasmatales bacterium isolate CA 2019 TPIn 173 MGD9910723.1
Bacillati Mycoplasmatota Mollicutes Candidatus Izemoplasmatales Candidatus Izemoplasmatales bacterium isolate STF1 90 MDD3841899.1
Bacillati Mycoplasmatota unclassified Mycoplasmatota Tenericutes bacterium HGW Tenericutes 1 PKL01128.1
Bacillati Mycoplasmatota Mollicutes Acholeplasmatales Acholeplasmatales bacterium isolate UBA12489 HAQ56822.1
Bacillati Mycoplasmatota unclassified Mycoplasmatota Mycoplasmatota bacterium isolate SW 4 67 MCK5732030.1
Bacillati Mycoplasmatota Mollicutes Candidatus Izemoplasmatales Candidatus Izemoplasma sp. B36 WP 372484245.1
Bacillati Mycoplasmatota unclassified Mycoplasmatota Mycoplasmatota bacterium WC30 XMB71587.1
Bacillati Mycoplasmatota unclassified Mycoplasmatota Mycoplasmatota bacterium isolate MAG22 MBI9009241.1
Bacillati Mycoplasmatota unclassified Mycoplasmatota Tenericutes bacterium 4572 104 OQX93693.1
Bacillati Mycoplasmatota Mollicutes Candidatus Izemoplasmatales Candidatus Izemoplasmatales bacterium isolate SO 2016 LW2 153 MFA7561577.1
Bacillati Mycoplasmatota Mollicutes Candidatus Izemoplasmatales Candidatus Izemoplasmatales bacterium isolate SO 2017 CLC 65 MFA7075382.1
Bacillati Mycoplasmatota Mollicutes Candidatus Izemoplasmatales Candidatus Izemoplasmatales bacterium isolate SO 2017 CLC 53 MFA7106234.1
Bacillati Mycoplasmatota Mollicutes Candidatus Izemoplasmatales Candidatus Izemoplasmatales bacterium isolate BLK5 1.bin.63 MFO7969684.1
Bacillati Mycoplasmatota Mollicutes Candidatus Izemoplasmatales Hujiaoplasma nucleasis WP 376739689.1
Bacillati Mycoplasmatota Mollicutes Candidatus Izemoplasmatales Candidatus Izemoplasmatales bacterium isolate CA 2019 NHSR 100 MGE4572751.1
