# Supplementary material for: Divergent evolution of the PRPS enzymes across the tree of life
Source: bioRxiv. 2026 Jun 3:2026.06.01.728777. Preprint. [Version 1] doi: 10.64898/2026.06.01.728777 (PMC13252144; doi:10.64898/2026.06.01.728777)
Supplement: Supplement 3 [file media-3.pdf]

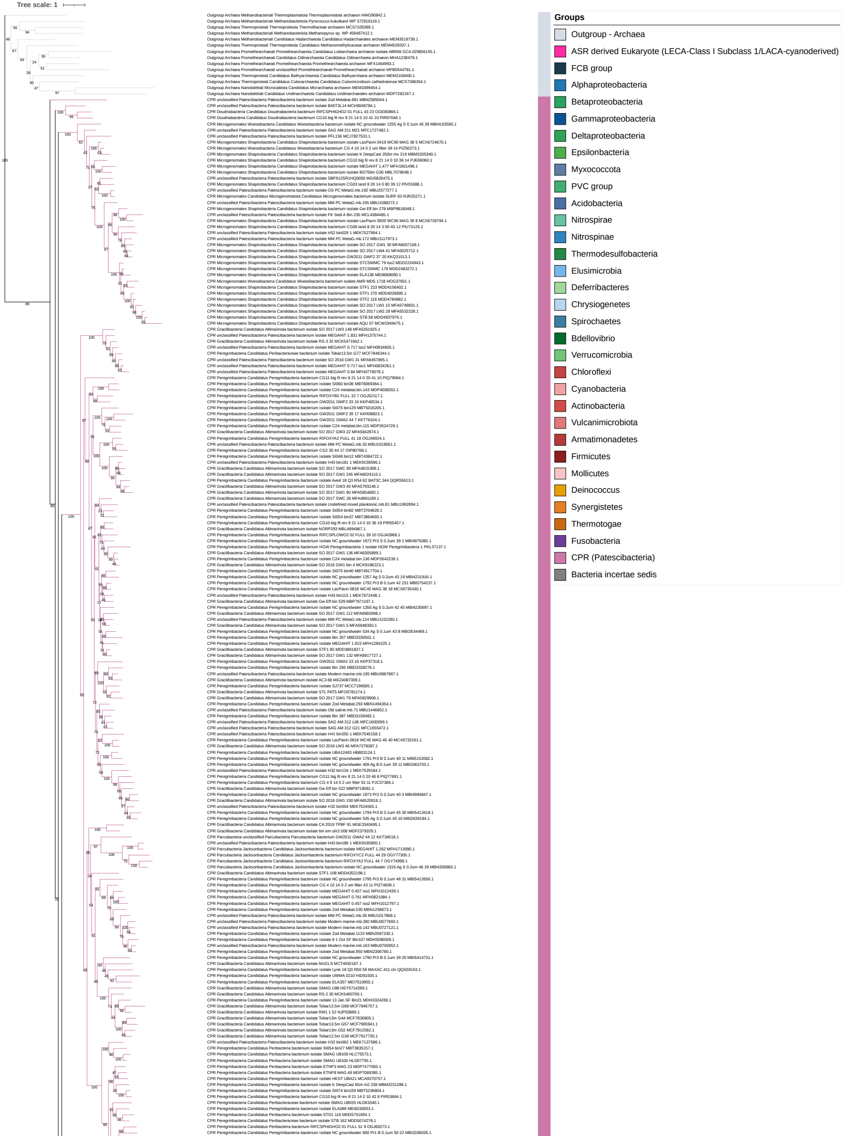
Tree scale: 1
Groups
Outgroup - Archaea
ASR derived Eukaryote (LECA-Class I Subclass 1/LACA-cyanoderived)
FCB group
Alphaproteobacteria
Betaproteobacteria
Gammaproteobacteria
Deltaproteobacteria
Epsilonbacteria
Myxococcota
PVC group
Acidobacteria
Nitrospirae
Nitrospinae
Thermodesulfobacteria
Elusimicrobia
Deferribacteres
Chrysiogenetes
Spirochaetes
Bdellovibrio
Verrucomicrobia
Chloroflexi
Cyanobacteria
Actinobacteria
Vulcanimicrobiota
Armatimonadetes
Firmicutes
Mollicutes
Deinococcus
Synergistetes
Thermotogae
Fusobacteria
CPR (Patescibacteria)
Bacteria incertae sedis

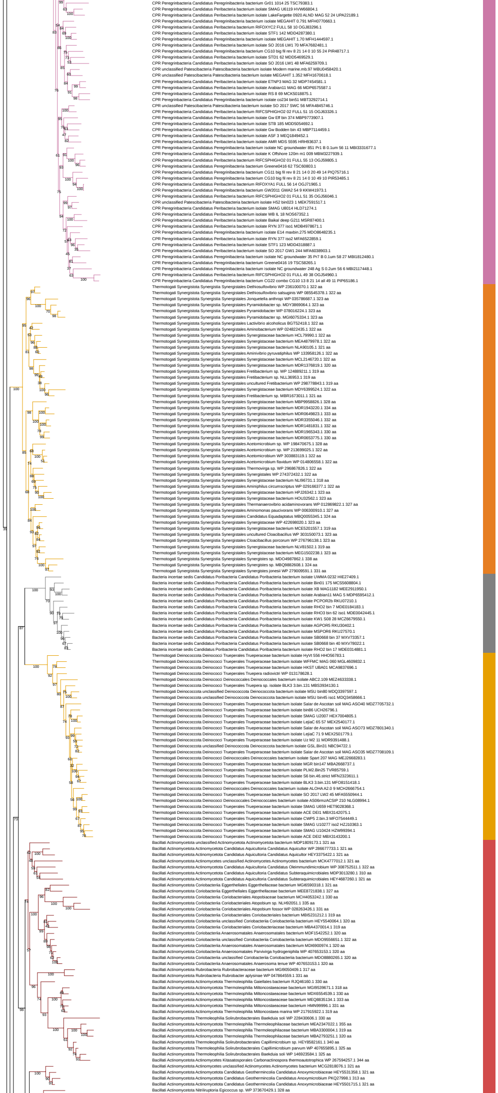

CPR Peregrinbacteria Candidatus Peregrinibacteria bacterium Gr01 1014 25 TSC79383.1
CPR Peregrinbacteria Candidatus Peribacteria bacterium isolate SMAG U6119 HVW66804.1
CPR Peregrinbacteria Candidatus Peribacteria bacterium isolate LakeFargette 0920 ALND MAG 52 24 UPA22189.1
CPR Peregrinbacteria Candidatus Peregrinibacteria bacterium isolate MEGAHIT 0.791 MFH0770663.1
CPR Peregrinbacteria Candidatus Peribacteria bacterium RIFOXYC2 FULL 58 10 OGJ83296.1
CPR Peregrinbacteria Candidatus Peribacteria bacterium isolate STF1 142 MDD4287380.1
CPR Peregrinbacteria Candidatus Peregrinibacteria bacterium isolate MEGAHIT 1.70 MFH1444597.1
CPR Peregrinbacteria Candidatus Peribacteria bacterium isolate SO 2016 LW1 70 MFA7682481.1
CPR Peregrinbacteria Candidatus Peregrinibacteria bacterium CG10 big fil rev 8 21 14 0 10 55 24 PIR48717.1
CPR Peregrinbacteria Candidatus Peribacteria bacterium isolate STD1 62 MDD5469529.1
CPR Peregrinbacteria Candidatus Peribacteria bacterium isolate SO 2016 LW1 48 MFA6259709.1
CPR unclassified Patescibacteria Patescibacteria bacterium isolate Modern marine.mb.97 MBU0458420.1
CPR unclassified Patescibacteria Patescibacteria bacterium isolate MEGAHIT 1.352 MFH1670618.1
CPR Peregrinbacteria Candidatus Peribacteria bacterium isolate ETNP3 MAG 32 MDP7454581.1
CPR Peregrinbacteria Candidatus Peribacteria bacterium isolate Arabian11 MAG 66 MDP6575587.1
CPR Peregrinbacteria Candidatus Peribacteria bacterium isolate RS 8 69 MCK5018875.1
CPR Peregrinbacteria Candidatus Peregrinibacteria bacterium isolate co234 bin51 MBT3292714.1
CPR unclassified Patescibacteria Patescibacteria bacterium isolate SO 2017 SWC 56 MFA4845746.1
CPR Peregrinbacteria Candidatus Peribacteria bacterium RIFCSPHIGHO2 02 FULL 51 15 OGJ63326.1
CPR Peregrinbacteria Candidatus Peribacteria bacterium isolate Gw Eff bin 374 MBP9773907.1
CPR Peregrinbacteria Candidatus Peribacteria bacterium isolate STB 185 MDD5054692.1
CPR Peregrinbacteria Candidatus Peribacteria bacterium isolate Gw Bodden bin 43 MBP7114459.1
CPR Peregrinbacteria Candidatus Peribacteria bacterium isolate ASF 3 MEQ1849452.1
CPR Peregrinbacteria Candidatus Peribacteria bacterium isolate AMR MDS 5595 HRH93637.1
CPR Peregrinbacteria Candidatus Peregrinibacteria bacterium isolate NC groundwater 851 Pr1 B 0.1um 56 11 MBI3331677.1
CPR Peregrinbacteria Candidatus Peribacteria bacterium isolate K Offshore 120m m1 009 MBM3227939.1
CPR Peregrinbacteria Candidatus Peribacteria bacterium RIFCSPHIGHO2 01 FULL 55 13 OGJ59805.1
CPR Peregrinbacteria Candidatus Peregrinibacteria bacterium Greene0416 62 TSC60803.1
CPR Peregrinbacteria Candidatus Peregrinibacteria bacterium CG11 big fil rev 8 21 14 0 20 49 14 PIQ75716.1
CPR Peregrinbacteria Candidatus Peregrinibacteria bacterium CG10 big fil rev 8 21 14 0 10 49 10 PIR53485.1
CPR Peregrinbacteria Candidatus Peribacteria bacterium RIFOXYA1 FULL 56 14 OGJ71965.1
CPR Peregrinbacteria Candidatus Peregrinibacteria bacterium GW2011 GWA2 54 9 KKW41973.1
CPR Peregrinbacteria Candidatus Peribacteria bacterium RIFCSPHIGHO2 01 FULL 51 35 OGJ56046.1
CPR unclassified Patescibacteria Patescibacteria bacterium isolate H52 bin023 1 MEK7591517.1
CPR Peregrinbacteria Candidatus Peribacteria bacterium isolate SMAG U8014 HLD71274.1
CPR Peregrinbacteria Candidatus Peribacteria bacterium isolate WB IL 18 NOS67352.1
CPR Peregrinbacteria Candidatus Peribacteria bacterium isolate Baikal deep G211 MSR87400.1
CPR Peregrinbacteria Candidatus Peribacteria bacterium isolate RYN 377 iso1 MDB4978671.1
CPR Peregrinbacteria Candidatus Peregrinibacteria bacterium isolate E14 maxbin.275 MDO8648235.1
CPR Peregrinbacteria Candidatus Peribacteria bacterium isolate RYN 377 iso2 MFA6522859.1
CPR Peregrinbacteria Candidatus Peribacteria bacterium isolate STF1 123 MDD4318887.1
CPR Peregrinbacteria Candidatus Peribacteria bacterium isolate SO 2017 GW1 244 MFA6038903.1
CPR Peregrinbacteria Candidatus Peregrinibacteria bacterium isolate NC groundwater 35 Pr7 B 0.1um 58 27 MBI1812480.1
CPR Peregrinbacteria Candidatus Peregrinibacteria bacterium Greene0416 19 TSC58265.1
CPR Peregrinbacteria Candidatus Peregrinibacteria bacterium isolate NC groundwater 248 Ag S 0.2um 56 6 MBI2117448.1
CPR Peregrinbacteria Candidatus Peribacteria bacterium RIFCSPHIGHO2 01 FULL 49 38 OGJ54960.1
CPR Peregrinbacteria Candidatus Peregrinibacteria bacterium CG22 combo CG10 13 8 21 14 all 49 11 PIP65186.1
Thermotogati Synergistota Synergistia Synergistales Dethiosulfovibrio WP 236100070.1 322 aa
Thermotogati Synergistota Synergistia Synergistales Dethiosulfovibrio salsuginis WP 085545378.1 322 aa
Thermotogati Synergistota Synergistia Synergistales Jonquetella anthropi WP 035786687.1 323 aa
Thermotogati Synergistota Synergistia Synergistales Pyramidobacter sp. MDY3869064.1 323 aa
Thermotogati Synergistota Synergistia Synergistales Pyramidobacter WP 078016224.1 323 aa
Thermotogati Synergistota Synergistia Synergistales Pyramidobacter sp. MGI6075334.1 323 aa
Thermotogati Synergistota Synergistia Synergistales Lactivibrio alcoholicus BGT52418.1 322 aa
Thermotogati Synergistota Synergistia Synergistales Aminobacterium WP 024822435.1 322 aa
Thermotogati Synergistota Synergistia Synergistales Synergistaceae bacterium HCL79990.1 322 aa
Thermotogati Synergistota Synergistia Synergistales Synergistaceae bacterium MEA4879978.1 322 aa
Thermotogati Synergistota Synergistia Synergistales Synergistaceae bacterium NLA90105.1 321 aa
Thermotogati Synergistota Synergistia Synergistales Aminivibrio pyruvatiphilus WP 133958126.1 322 aa
Thermotogati Synergistota Synergistia Synergistales Synergistaceae bacterium MCL2146720.1 322 aa
Thermotogati Synergistota Synergistia Synergistales Synergistaceae bacterium MDR1376819.1 320 aa
Thermotogati Synergistota Synergistia Synergistales Fretibacterium sp. WP 124889211.1 319 aa
Thermotogati Synergistota Synergistia Synergistales Fretibacterium sp. NLL36953.1 319 aa
Thermotogati Synergistota Synergistia Synergistales uncultured Fretibacterium WP 298778843.1 319 aa
Thermotogati Synergistota Synergistia Synergistales Synergistaceae bacterium MDY6399524.1 322 aa
Thermotogati Synergistota Synergistia Synergistales Fretibacterium sp. MBR1673011.1 321 aa
Thermotogati Synergistota Synergistia Synergistales Synergistaceae bacterium MBP9958826.1 328 aa
Thermotogati Synergistota Synergistia Synergistales Synergistaceae bacterium MDR1943220.1 334 aa
Thermotogati Synergistota Synergistia Synergistales Synergistaceae bacterium MDR0649823.1 333 aa
Thermotogati Synergistota Synergistia Synergistales Synergistaceae bacterium MDR3355046.1 332 aa
Thermotogati Synergistota Synergistia Synergistales Synergistaceae bacterium MDR1481831.1 332 aa
Thermotogati Synergistota Synergistia Synergistales Synergistaceae bacterium MDR1965343.1 330 aa
Thermotogati Synergistota Synergistia Synergistales Synergistaceae bacterium MDR0653775.1 330 aa
Thermotogati Synergistota Synergistia Synergistales Acetomicrobium sp. WP 198470675.1 328 aa
Thermotogati Synergistota Synergistia Synergistales Acetomicrobium sp. WP 213699025.1 322 aa
Thermotogati Synergistota Synergistia Synergistales Acetomicrobium WP 303883119.1 322 aa
Thermotogati Synergistota Synergistia Synergistales Acetomicrobium flavidum WP 014806558.1 322 aa
Thermotogati Synergistota Synergistia Synergistales Thermovirga sp. WP 296867826.1 322 aa
Thermotogati Synergistota Synergistia Synergistales Synergistales WP 274372432.1 322 aa
Thermotogati Synergistota Synergistia Synergistales Synergistaceae bacterium NLI96731.1 318 aa
Thermotogati Synergistota Synergistia Synergistales Aminiphilus circumscriptus WP 029166377.1 322 aa
Thermotogati Synergistota Synergistia Synergistales Synergistaceae bacterium HPJ26342.1 323 aa
Thermotogati Synergistota Synergistia Synergistales Synergistaceae bacterium HOU32562.1 323 aa
Thermotogati Synergistota Synergistia Synergistales Thermanaerovibrio acidaminovorans WP 012869822.1 327 aa
Thermotogati Synergistota Synergistia Synergistales Aminomonas paucivorans WP 006300910.1 327 aa
Thermotogati Synergistota Synergistia Synergistales Candidatus Equadaptatus MBQ0055345.1 324 aa
Thermotogati Synergistota Synergistia Synergistales Synergistaceae WP 422698020.1 323 aa
Thermotogati Synergistota Synergistia Synergistales Synergistaceae bacterium MCE5201557.1 319 aa
Thermotogati Synergistota Synergistia Synergistales uncultured Cloacibacillus WP 303150073.1 323 aa
Thermotogati Synergistota Synergistia Synergistales Cloacibacillus porcorum WP 276796138.1 323 aa
Thermotogati Synergistota Synergistia Synergistales Synergistaceae bacterium NLV81502.1 319 aa
Thermotogati Synergistota Synergistia Synergistales Synergistaceae bacterium MEG1502238.1 323 aa
Thermotogati Synergistota Synergistia Synergistales Synergistes sp. MDO4987862.1 338 aa
Thermotogati Synergistota Synergistia Synergistales Synergistes sp. MBQ9882608.1 324 aa
Thermotogati Synergistota Synergistia Synergistales Synergistes jonesii WP 279009591.1 331 aa
Bacteria incertae sedis Candidatus Poribacteria Candidatus Poribacteria bacterium isolate UWMA 0232 HIE27409.1
Bacteria incertae sedis Candidatus Poribacteria Candidatus Poribacteria bacterium isolate Bin01 175 MCS5608804.1
Bacteria incertae sedis Candidatus Poribacteria Candidatus Poribacteria bacterium isolate XB MAG1182 MEE2911950.1
Bacteria incertae sedis Candidatus Poribacteria Candidatus Poribacteria bacterium isolate Arabian11 MAG 5 MDP6595412.1
Bacteria incertae sedis Candidatus Poribacteria Candidatus Poribacteria bacterium isolate PCPOR2b RKU07210.1
Bacteria incertae sedis Candidatus Poribacteria Candidatus Poribacteria bacterium isolate RHO2 bin 7 MDE0184183.1
Bacteria incertae sedis Candidatus Poribacteria Candidatus Poribacteria bacterium isolate RHO3 bin 62 iso1 MDE0042445.1
Bacteria incertae sedis Candidatus Poribacteria Candidatus Poribacteria bacterium isolate KW1 S08 28 MCZ6679550.1
Bacteria incertae sedis Candidatus Poribacteria Candidatus Poribacteria bacterium isolate AGPOR5 RKU30402.1
Bacteria incertae sedis Candidatus Poribacteria Candidatus Poribacteria bacterium isolate MSPOR6 RKU27570.1
Bacteria incertae sedis Candidatus Poribacteria Candidatus Poribacteria bacterium isolate SB0668 bin 37 MXV73357.1
Bacteria incertae sedis Candidatus Poribacteria Candidatus Poribacteria bacterium isolate SB0668 bin 40 MXV78022.1
Bacteria incertae sedis Candidatus Poribacteria Candidatus Poribacteria bacterium isolate RHO2 bin 17 MDE0314881.1
Thermotogati Deinococcota Deinococci Trueperales Trueperaceae bacterium isolate HyVt 556 HHO56783.1
Thermotogati Deinococcota Deinococci Trueperales Trueperaceae bacterium isolate WFFMC MAG 060 MGL4609832.1
Thermotogati Deinococcota Deinococci Trueperales Trueperaceae bacterium isolate HKST UBA01 MCA9837696.1
Thermotogati Deinococcota Deinococci Trueperales Truepera radiovictri WP 013178628.1
Thermotogati Deinococcota Deinococci Deinococcales Deinococcales bacterium isolate ABC2.109 MEZ4633338.1
Thermotogati Deinococcota Deinococci Trueperales Truepera sp. isolate BLK3 3.bin.131 MBS3934130.1
Thermotogati Deinococcota unclassified Deinococcota Deinococcota bacterium isolate MSU bin80 MDQ3397597.1
Thermotogati Deinococcota unclassified Deinococcota Deinococcota bacterium isolate MSU bin45 iso1 MDQ3458666.1
Thermotogati Deinococcota Deinococci Trueperales Trueperaceae bacterium isolate Salar de Ascotan soil MAG ASO40 MDZ7705732.1
Thermotogati Deinococcota Deinococci Trueperales Trueperaceae bacterium isolate bin86 UCH26796.1
Thermotogati Deinococcota Deinococci Trueperales Trueperaceae bacterium isolate SMAG U2007 HEX7004805.1
Thermotogati Deinococcota Deinococci Trueperales Trueperaceae bacterium isolate LejiaC 65 57 MEX2540177.1
Thermotogati Deinococcota Deinococci Trueperales Trueperaceae bacterium isolate Salar de Ascotan soil MAG ASO73 MDZ7801340.1
Thermotogati Deinococcota Deinococci Trueperales Trueperaceae bacterium isolate LejiaC 71 9 MEX2501779.1
Thermotogati Deinococcota Deinococci Trueperales Trueperaceae bacterium isolate Uz M2 11 MDR9391488.1
Thermotogati Deinococcota unclassified Deinococcota Deinococcota bacterium isolate GSL.Bin31 NBC94722.1
Thermotogati Deinococcota Deinococci Trueperales Trueperaceae bacterium isolate Salar de Ascotan soil MAG ASO35 MDZ7708109.1
Thermotogati Deinococcota Deinococci Deinococcales Deinococcales bacterium isolate Spart 207 MAG MEJ2668283.1
Thermotogati Deinococcota Deinococci Trueperales Trueperaceae bacterium isolate MGR bin147 MBA2668737.1
Thermotogati Deinococcota Deinococci Trueperales Trueperaceae bacterium isolate PLM2.Bin25 TVR85759.1
Thermotogati Deinococcota Deinococci Trueperales Trueperaceae bacterium isolate S6 bin.46.strict MFN2323611.1
Thermotogati Deinococcota Deinococci Trueperales Trueperaceae bacterium isolate BLK3 3.bin.131 MFO8151418.1
Thermotogati Deinococcota Deinococci Deinococcales Deinococcales bacterium isolate ALOHA A2.0 9 MCH2666754.1
Thermotogati Deinococcota Deinococci Trueperales Trueperaceae bacterium isolate SO 2017 LW2 45 MFA5550944.1
Thermotogati Deinococcota Deinococci Deinococcales Deinococcales bacterium isolate AS06rmzACSIP 210 NLG08994.1
Thermotogati Deinococcota Deinococci Trueperales Trueperaceae bacterium isolate SMAG U659 HET9028368.1
Thermotogati Deinococcota Deinococci Trueperales Trueperaceae bacterium isolate ACE DEI1 MBX3142075.1
Thermotogati Deinococcota Deinococci Trueperales Trueperaceae bacterium isolate CWP5 2.bin.3 MFO7544449.1
Thermotogati Deinococcota Deinococci Trueperales Trueperaceae bacterium isolate SMAG U10277 iso2 HZJ10363.1
Thermotogati Deinococcota Deinococci Trueperales Trueperaceae bacterium isolate SMAG U10424 HZW99394.1
Thermotogati Deinococcota Deinococci Trueperales Trueperaceae bacterium isolate ACE DEI2 MBX3143200.1
Bacillati Actinomycetota unclassified Actinomycetota Actinomycetota bacterium MDP1809173.1 321 aa
Bacillati Actinomycetota Actinomycetota Candidatus Aquicultoria Candidatus Aquicultor WP 286677733.1 321 aa
Bacillati Actinomycetota Actinomycetota Candidatus Aquicultoria Candidatus Aquicultor HEY3375422.1 321 aa
Bacillati Actinomycetota Actinomycetes unclassified Actinomycetes Actinomycetes bacterium MCK4777012.1 321 aa
Bacillati Actinomycetota Actinomycetota Candidatus Aquicultoria Candidatus Oleimmundimicrobium WP 308752511.1 322 aa
Bacillati Actinomycetota Actinomycetota Candidatus Aquicultoria Candidatus Subteraquimicrobiales MDP3013280.1 310 aa
Bacillati Actinomycetota Actinomycetota Candidatus Aquicultoria Candidatus Subteraquimicrobiales HEY4687260.1 321 aa
Bacillati Actinomycetota Coriobacteriia Eggerthellales Eggerthellaceae bacterium MGI6590318.1 321 aa
Bacillati Actinomycetota Coriobacteriia Eggerthellales Eggerthellaceae bacterium MEE8721838.1 327 aa
Bacillati Actinomycetota Coriobacteriia Coriobacteriales Atopobiaceae bacterium MCH4053242.1 330 aa
Bacillati Actinomycetota Coriobacteriia Coriobacteriales Atopobium sp. NLH92051.1 335 aa
Bacillati Actinomycetota Coriobacteriia Coriobacteriales Atopobium fossor WP 028263426.1 331 aa
Bacillati Actinomycetota Coriobacteriia Coriobacteriales Coriobacteriales bacterium MBI5231212.1 319 aa
Bacillati Actinomycetota Coriobacteriia unclassified Coriobacteriia Coriobacteriia bacterium HEY5540064.1 320 aa
Bacillati Actinomycetota Coriobacteriia Coriobacteriales Coriobacteriaceae bacterium MBA4370014.1 319 aa
Bacillati Actinomycetota Coriobacteriia Anaerosomatales Anaerosomatales bacterium MDF1542252.1 320 aa
Bacillati Actinomycetota Coriobacteriia unclassified Coriobacteriia Coriobacteriia bacterium MDO9556651.1 322 aa
Bacillati Actinomycetota Coriobacteriia Anaerosomatales Anaerosomatales bacterium MDI6900974.1 320 aa
Bacillati Actinomycetota Coriobacteriia Anaerosomatales Parvivirga hydrogeniphila WP 407653153.1 320 aa
Bacillati Actinomycetota Coriobacteriia unclassified Coriobacteriia Coriobacteriia bacterium MDO8880265.1 320 aa
Bacillati Actinomycetota Coriobacteriia Anaerosomatales Anaerosoma tenue WP 407653153.1 320 aa
Bacillati Actinomycetota Rubrobacteria Rubrobacteraceae bacterium MGI9050409.1 317 aa
Bacillati Actinomycetota Rubrobacteria Rubrobacter aplysinae WP 047864559.1 331 aa
Bacillati Actinomycetota Actinomycetota Thermoleophilia Gaiellales bacterium RJQ46160.1 330 aa
Bacillati Actinomycetota Actinomycetota Thermoleophilia Miltoncostaeaceae bacterium MGI9539671.1 318 aa
Bacillati Actinomycetota Actinomycetota Thermoleophilia Miltoncostaeaceae bacterium MDX6554539.1 330 aa
Bacillati Actinomycetota Actinomycetota Thermoleophilia Miltoncostaeaceae bacterium MEQ8835134.1 333 aa
Bacillati Actinomycetota Actinomycetota Thermoleophilia Miltoncostaeaceae bacterium HMN99996.1 331 aa
Bacillati Actinomycetota Actinomycetota Thermoleophilia Miltoncostaea marina WP 217915922.1 319 aa
Bacillati Actinomycetota Thermoleophilia Solirubrobacterales Baekduia soli WP 228430606.1 330 aa
Bacillati Actinomycetota Actinomycetota Thermoleophilia Thermoleophilaceae bacterium MEA2347022.1 355 aa
Bacillati Actinomycetota Actinomycetota Thermoleophilia Thermoleophilaceae bacterium MBA3300004.1 319 aa
Bacillati Actinomycetota Actinomycetota Thermoleophilia Thermoleophilaceae bacterium MBA2793251.1 320 aa
Bacillati Actinomycetota Thermoleophilia Solirubrobacterales Capillimicrobium sp. HEY8582161.1 340 aa
Bacillati Actinomycetota Thermoleophilia Solirubrobacterales Capillimicrobium parvum WP 407655895.1 325 aa
Bacillati Actinomycetota Thermoleophilia Solirubrobacterales Baekduia soli WP 146923584.1 325 aa
Bacillati Actinomycetota Actinomycetes Kitasatosporales Carbonactinospora thermoautotrophica WP 267594257.1 344 aa
Bacillati Actinomycetota Actinomycetes unclassified Actinomycetes Actinomycetes bacterium MCG2818076.1 321 aa
Bacillati Actinomycetota Actinomycetota Candidatus Geothermincolia Candidatus Anoxymicrobiaceae HEY5531358.1 321 aa
Bacillati Actinomycetota Actinomycetota Candidatus Geothermincolia Candidatus Anoxymicrobium PKQ27998.1 313 aa
Bacillati Actinomycetota Actinomycetota Candidatus Geothermincolia Candidatus Anoxymicrobiaceae HEY5501715.1 321 aa
Bacillati Actinomycetota Nitriliruptoria Egicoccus sp. WP 373670429.1 328 aa

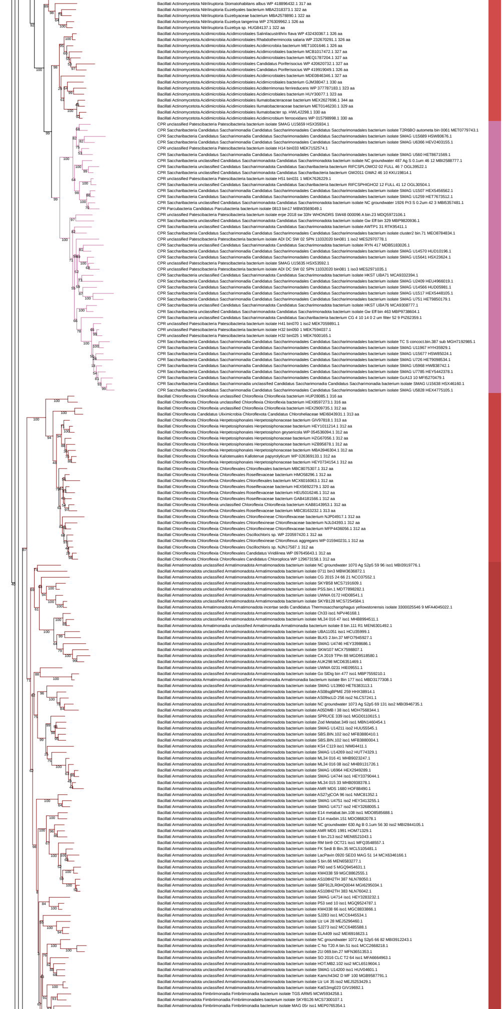

Bacillati Actinomycetota Nitriliruptoria Stomatohabitans albus WP 418896432.1 317 aa
Bacillati Actinomycetota Nitriliruptoria Euzebyales bacterium MBA2318373.1 322 aa
Bacillati Actinomycetota Nitriliruptoria Euzebyaceae bacterium MBA2578890.1 322 aa
Bacillati Actinomycetota Nitriliruptoria Euzebya tangerina WP 276309962.1 326 aa
Bacillati Actinomycetota Nitriliruptoria Euzebya sp. HUG84137.1 322 aa
Bacillati Actinomycetota Acidimicrobiia Acidimicrobiales Salinilacustrithrix flava WP 432430367.1 326 aa
Bacillati Actinomycetota Acidimicrobiia Acidimicrobiales Rhabdothermincola salaria WP 232670291.1 326 aa
Bacillati Actinomycetota Acidimicrobiia Acidimicrobiales Acidimicrobiia bacterium MET1001646.1 326 aa
Bacillati Actinomycetota Acidimicrobiia Acidimicrobiales Acidimicrobiales bacterium MCB1017472.1 327 aa
Bacillati Actinomycetota Acidimicrobiia Acidimicrobiales Acidimicrobiales bacterium MEQ1787204.1 327 aa
Bacillati Actinomycetota Acidimicrobiia Acidimicrobiales Candidatus Poriferisocius WP 420620732.1 327 aa
Bacillati Actinomycetota Acidimicrobiia Acidimicrobiales Candidatus Poriferisocius WP 419919049.1 326 aa
Bacillati Actinomycetota Acidimicrobiia Acidimicrobiales Acidimicrobiales bacterium MDE0846346.1 327 aa
Bacillati Actinomycetota Acidimicrobiia Acidimicrobiales Acidimicrobiales bacterium GJM38047.1 330 aa
Bacillati Actinomycetota Acidimicrobiia Acidimicrobiales Aciditerrimonas ferrireducens WP 377787183.1 323 aa
Bacillati Actinomycetota Acidimicrobiia Acidimicrobiales Acidimicrobiales bacterium HUY30077.1 323 aa
Bacillati Actinomycetota Acidimicrobiia Acidimicrobiales Ilumatobacteraceae bacterium MEX2627696.1 344 aa
Bacillati Actinomycetota Acidimicrobiia Acidimicrobiales Ilumatobacteraceae bacterium MET0146230.1 329 aa
Bacillati Actinomycetota Acidimicrobiia Acidimicrobiales Ilumatobacter sp. HWL42298.1 330 aa
Bacillati Actinomycetota Acidimicrobiia Acidimicrobiales Acidimicrobium ferrooxidans WP 015798998.1 330 aa
CPR unclassified Patescibacteria Patescibacteria bacterium isolate SMAG U15659 HSX35934.1
CPR Saccharibacteria Candidatus Saccharimonadia Candidatus Saccharimonadales Candidatus Saccharimonadales bacterium isolate T2R6BO autometa bin 0061 MET0779743.1
CPR Saccharibacteria Candidatus Saccharimonadia Candidatus Saccharimonadales Candidatus Saccharimonadales bacterium isolate SMAG U15689 HSW80676.1
CPR Saccharibacteria Candidatus Saccharimonadia Candidatus Saccharimonadales Candidatus Saccharimonadales bacterium isolate SMAG U6366 HEV2403155.1
CPR unclassified Patescibacteria Patescibacteria bacterium isolate H14 bin033 MEK7152574.1
CPR Saccharibacteria Candidatus Saccharimonadia Candidatus Saccharimonadales Candidatus Saccharimonadales bacterium isolate SMAG U560 HET8671569.1
CPR Saccharibacteria unclassified Candidatus Saccharimonadota Candidatus Saccharimonadota bacterium isolate NC groundwater 487 Ag S 0.1um 46 12 MBI2588777.1
CPR Saccharibacteria unclassified Candidatus Saccharimonadota Candidatus Saccharibacteria bacterium RIFCSPLOWO2 02 FULL 46 7 OGL39522.1
CPR Saccharibacteria unclassified Candidatus Saccharimonadota Candidatus Saccharibacteria bacterium GW2011 GWA2 46 10 KKU19814.1
CPR unclassified Patescibacteria Patescibacteria bacterium isolate H51 bin031 1 MEK7626229.1
CPR Saccharibacteria unclassified Candidatus Saccharimonadota Candidatus Saccharibacteria bacterium RIFCSPHIGHO2 12 FULL 41 12 OGL30504.1
CPR Saccharibacteria Candidatus Saccharimonadia Candidatus Saccharimonadales Candidatus Saccharimonadales bacterium isolate SMAG U1507 HEX5456562.1
CPR Saccharibacteria Candidatus Saccharimonadia Candidatus Saccharimonadales Candidatus Saccharimonadales bacterium isolate SMAG U1259 HET7673512.1
CPR Saccharibacteria unclassified Candidatus Saccharimonadota Candidatus Saccharimonadota bacterium isolate NC groundwater 1926 Pr3 S 0.2um 42 3 MBI5357481.1
CPR Parcubacteria Candidatus Parcubacteria bacterium isolate 0813 bin17 MBW3569049.1
CPR unclassified Patescibacteria Patescibacteria bacterium isolate erpe 2018 sw 33hr WHONDRS SW48 000096 A bin.23 MDQ5972106.1
CPR Saccharibacteria unclassified Candidatus Saccharimonadota Candidatus Saccharimonadota bacterium isolate Gw Eff bin 329 MBP9820936.1
CPR Saccharibacteria unclassified Candidatus Saccharimonadota Candidatus Saccharimonadota bacterium isolate AWTP1 31 RTK95411.1
CPR Saccharibacteria Candidatus Saccharimonadia Candidatus Saccharimonadales Candidatus Saccharimonadales bacterium isolate cluster2 bin.71 MEO8784834.1
CPR unclassified Patescibacteria Patescibacteria bacterium isolate ADI DC SW 02 SPN 11032020 bin081 1 iso2 MES2970778.1
CPR Saccharibacteria unclassified Candidatus Saccharimonadota Candidatus Saccharimonadota bacterium isolate RYN 417 MDB5183026.1
CPR Saccharibacteria Candidatus Saccharimonadia Candidatus Saccharimonadales Candidatus Saccharimonadales bacterium isolate SMAG U14570 HUD10196.1
CPR Saccharibacteria Candidatus Saccharimonadia Candidatus Saccharimonadales Candidatus Saccharimonadales bacterium isolate SMAG U15641 HSX23624.1
CPR unclassified Patescibacteria Patescibacteria bacterium isolate SMAG U15635 HSX53592.1
CPR unclassified Patescibacteria Patescibacteria bacterium isolate ADI DC SW 02 SPN 11032020 bin081 1 iso3 MES2971035.1
CPR Saccharibacteria unclassified Candidatus Saccharimonadota Candidatus Saccharimonadota bacterium isolate HKST UBA71 MCA9332394.1
CPR Saccharibacteria Candidatus Saccharimonadia Candidatus Saccharimonadales Candidatus Saccharimonadales bacterium isolate SMAG U2409 HEU4966019.1
CPR Saccharibacteria Candidatus Saccharimonadia Candidatus Saccharimonadales Candidatus Saccharimonadales bacterium isolate SMAG U14566 HUD05981.1
CPR Saccharibacteria Candidatus Saccharimonadia Candidatus Saccharimonadales Candidatus Saccharimonadales bacterium isolate SMAG U1517 HEX5448105.1
CPR Saccharibacteria Candidatus Saccharimonadia Candidatus Saccharimonadales Candidatus Saccharimonadales bacterium isolate SMAG U751 HET9850179.1
CPR Saccharibacteria unclassified Candidatus Saccharimonadota Candidatus Saccharimonadota bacterium isolate HKST UBA76 MCA9308777.1
CPR Saccharibacteria unclassified Candidatus Saccharimonadota Candidatus Saccharimonadota bacterium isolate Gw Eff bin 463 MBP9738604.1
CPR Saccharibacteria unclassified Candidatus Saccharimonadota Candidatus Saccharibacteria bacterium CG 4 10 14 0 2 um filter 52 9 PIZ62359.1
CPR unclassified Patescibacteria Patescibacteria bacterium isolate H41 bin070 1 iso2 MEK7059891.1
CPR unclassified Patescibacteria Patescibacteria bacterium isolate H32 bin050 1 MEK7594037.1
CPR unclassified Patescibacteria Patescibacteria bacterium isolate H32 bin025 1 MEK7600165.1
CPR Saccharibacteria Candidatus Saccharimonadia Candidatus Saccharimonadales Candidatus Saccharimonadales bacterium isolate TC S concoct.bin.387 sub MGH7192985.1
CPR Saccharibacteria Candidatus Saccharimonadia Candidatus Saccharimonadales Candidatus Saccharimonadales bacterium isolate SMAG U11967 HYH35929.1
CPR Saccharibacteria Candidatus Saccharimonadia Candidatus Saccharimonadales Candidatus Saccharimonadales bacterium isolate SMAG U15677 HSW85024.1
CPR Saccharibacteria Candidatus Saccharimonadia Candidatus Saccharimonadales Candidatus Saccharimonadales bacterium isolate SMAG U726 HET9098534.1
CPR Saccharibacteria Candidatus Saccharimonadia Candidatus Saccharimonadales Candidatus Saccharimonadales bacterium isolate SMAG U5968 HWB38742.1
CPR Saccharibacteria Candidatus Saccharimonadia Candidatus Saccharimonadales Candidatus Saccharimonadales bacterium isolate SMAG U7785 HEY5442378.1
CPR Saccharibacteria Candidatus Saccharimonadia Candidatus Saccharimonadales Candidatus Saccharimonadales bacterium isolate GcA13 10 MFI5270479.1
CPR Saccharibacteria Candidatus Saccharimonadia unclassified Candidatus Saccharimonadia Candidatus Saccharimonadia bacterium isolate SMAG U15638 HSX46160.1
CPR Saccharibacteria Candidatus Saccharimonadia Candidatus Saccharimonadales Candidatus Saccharimonadales bacterium isolate SMAG U5828 HEX4775105.1
Bacillati Chloroflexota Chloroflexia unclassified Chloroflexia Chloroflexia bacterium HUP28085.1 316 aa
Bacillati Chloroflexota Chloroflexia unclassified Chloroflexia Chloroflexia bacterium HEX8597273.1 316 aa
Bacillati Chloroflexota Chloroflexia unclassified Chloroflexia Chloroflexia bacterium HEX2909735.1 312 aa
Bacillati Chloroflexota Candidatus Uliximicrobia Chloroflexota Candidatus Chloroheliaceae MEI6043931.1 313 aa
Bacillati Chloroflexota Chloroflexia Herpetosiphonales Herpetosiphonaceae bacterium GIV97818.1 313 aa
Bacillati Chloroflexota Chloroflexia Herpetosiphonales Herpetosiphonaceae bacterium HEY1011214.1 312 aa
Bacillati Chloroflexota Chloroflexia Herpetosiphonales Herpetosiphon geysericola WP 054536094.1 312 aa
Bacillati Chloroflexota Chloroflexia Herpetosiphonales Herpetosiphonaceae bacterium HZG67056.1 312 aa
Bacillati Chloroflexota Chloroflexia Herpetosiphonales Herpetosiphonaceae bacterium HZB95878.1 312 aa
Bacillati Chloroflexota Chloroflexia Herpetosiphonales Herpetosiphonaceae bacterium MBA3946304.1 312 aa
Bacillati Chloroflexota Chloroflexia Kallotenuales Kallotenue papyrolyticum WP 026369133.1 312 aa
Bacillati Chloroflexota Chloroflexia Herpetosiphonales Herpetosiphonaceae bacterium HEY0734154.1 312 aa
Bacillati Chloroflexota Chloroflexia Chloroflexales Chloroflexales bacterium MBC8075307.1 312 aa
Bacillati Chloroflexota Chloroflexia Chloroflexales Roseiflexaceae bacterium HMO58296.1 312 aa
Bacillati Chloroflexota Chloroflexia Chloroflexales Chloroflexales bacterium MCX6016063.1 312 aa
Bacillati Chloroflexota Chloroflexia Chloroflexales Roseiflexaceae bacterium HEX5692279.1 320 aa
Bacillati Chloroflexota Chloroflexia Chloroflexales Roseiflexaceae bacterium HEU5016246.1 312 aa
Bacillati Chloroflexota Chloroflexia Chloroflexales Roseiflexaceae bacterium GAB4181566.1 312 aa
Bacillati Chloroflexota Chloroflexia unclassified Chloroflexia Chloroflexia bacterium KAB8143953.1 312 aa
Bacillati Chloroflexota Chloroflexia Chloroflexales Roseiflexaceae bacterium MBC8163232.1 313 aa
Bacillati Chloroflexota Chloroflexia Chloroflexales Chloroflexineae Chloroflexaceae bacterium NJP04917.1 312 aa
Bacillati Chloroflexota Chloroflexia Chloroflexales Chloroflexineae Chloroflexaceae bacterium NJL04393.1 312 aa
Bacillati Chloroflexota Chloroflexia Chloroflexales Chloroflexineae Chloroflexaceae bacterium MFP4436056.1 312 aa
Bacillati Chloroflexota Chloroflexia Chloroflexales Oscillochloris sp. WP 220597420.1 312 aa
Bacillati Chloroflexota Chloroflexia Chloroflexales Chloroflexineae Chloroflexus aggregans WP 015940231.1 312 aa
Bacillati Chloroflexota Chloroflexia Chloroflexales Oscillochloris sp. NJN17587.1 312 aa
Bacillati Chloroflexota Chloroflexia Chloroflexales Candidatus Viridilinea WP 097645643.1 312 aa
Bacillati Chloroflexota Chloroflexia Chloroflexales Candidatus Chloroploca WP 129673158.1 312 aa
Bacillati Armatimonadota unclassified Armatimonadota Armatimonadota bacterium isolate NC groundwater 1070 Ag S2p5 59 96 iso1 MBI3919776.1
Bacillati Armatimonadota unclassified Armatimonadota Armatimonadota bacterium isolate 0711 bin3 MBW3636872.1
Bacillati Armatimonadota unclassified Armatimonadota Armatimonadota bacterium isolate CG 2015 24 66 21 NCO37552.1
Bacillati Armatimonadota unclassified Armatimonadota Armatimonadota bacterium isolate SKYB58 MCS7191609.1
Bacillati Armatimonadota unclassified Armatimonadota Armatimonadota bacterium isolate PSS.bin.1 MDT7898282.1
Bacillati Armatimonadota unclassified Armatimonadota Armatimonadota bacterium isolate UWMA 0172 HID08541.1
Bacillati Armatimonadota unclassified Armatimonadota Armatimonadota bacterium isolate SKYB128 MCS7254584.1
Bacillati Armatimonadota Armatimonadota Armatimonadota incertae sedis Candidatus Thermosaccharophagus yellowstonensis isolate 3300025546 9 MFA4045022.1
Bacillati Armatimonadota unclassified Armatimonadota Armatimonadota bacterium isolate Ch33 iso1 NPV46168.1
Bacillati Armatimonadota unclassified Armatimonadota Armatimonadota bacterium isolate ML34 016 47 iso1 MHB8994511.1
Bacillati Armatimonadota Armatimonadia unclassified Armatimonadia Armatimonadia bacterium isolate 8 bin.111 R1 MEN6301492.1
Bacillati Armatimonadota unclassified Armatimonadota Armatimonadota bacterium isolate UBA11051 iso1 HCU35999.1
Bacillati Armatimonadota unclassified Armatimonadota Armatimonadota bacterium isolate BLK5 2.bin.37 MFO7945927.1
Bacillati Armatimonadota unclassified Armatimonadota Armatimonadota bacterium isolate SMAG U4746 HEY3398686.1
Bacillati Armatimonadota unclassified Armatimonadota Armatimonadota bacterium isolate SKW107 MCX7598807.1
Bacillati Armatimonadota unclassified Armatimonadota Armatimonadota bacterium isolate CA 2019 TPln 88 MGD9518580.1
Bacillati Armatimonadota unclassified Armatimonadota Armatimonadota bacterium isolate AUK298 MCD6351469.1
Bacillati Armatimonadota unclassified Armatimonadota Armatimonadota bacterium isolate UWMA 0231 HIE09551.1
Bacillati Armatimonadota unclassified Armatimonadota Armatimonadota bacterium isolate Go SlDig bin 477 iso1 MBP7559210.1
Bacillati Armatimonadota Armatimonadia unclassified Armatimonadia Armatimonadia bacterium isolate Bin 177 iso1 MBD3177308.1
Bacillati Armatimonadota unclassified Armatimonadota Armatimonadota bacterium isolate SMAG U13960 HET6383113.1
Bacillati Armatimonadota unclassified Armatimonadota Armatimonadota bacterium isolate AS08sgBPME 259 HHX38914.1
Bacillati Armatimonadota unclassified Armatimonadota Armatimonadota bacterium isolate AS09scLD 256 iso2 NLC57241.1
Bacillati Armatimonadota unclassified Armatimonadota Armatimonadota bacterium isolate NC groundwater 1073 Ag S2p5 69 131 iso2 MBI3946735.1
Bacillati Armatimonadota unclassified Armatimonadota Armatimonadota bacterium isolate A05DMB I 38 iso1 MDH7568344.1
Bacillati Armatimonadota unclassified Armatimonadota Armatimonadota bacterium isolate SPRUCE 339 iso1 MGD0110615.1
Bacillati Armatimonadota unclassified Armatimonadota Armatimonadota bacterium isolate Zod Metabat.349 iso1 MBN1460454.1
Bacillati Armatimonadota unclassified Armatimonadota Armatimonadota bacterium isolate SMAG U14211 iso2 HUU55545.1
Bacillati Armatimonadota unclassified Armatimonadota Armatimonadota bacterium isolate SBS.BIN.102 iso2 MFB3880410.1
Bacillati Armatimonadota unclassified Armatimonadota Armatimonadota bacterium isolate SBS.BIN.102 iso1 MFB3880004.1
Bacillati Armatimonadota unclassified Armatimonadota Armatimonadota bacterium isolate KS4 C119 iso1 NIM04411.1
Bacillati Armatimonadota unclassified Armatimonadota Armatimonadota bacterium isolate SMAG U14269 iso2 HUT74329.1
Bacillati Armatimonadota unclassified Armatimonadota Armatimonadota bacterium isolate ML34 016 41 MHB9023247.1
Bacillati Armatimonadota unclassified Armatimonadota Armatimonadota bacterium isolate ML34 016 08 iso2 MHB9131726.1
Bacillati Armatimonadota unclassified Armatimonadota Armatimonadota bacterium isolate SMAG U6964 HEX2949289.1
Bacillati Armatimonadota unclassified Armatimonadota Armatimonadota bacterium isolate SMAG U4744 iso1 HEY3379044.1
Bacillati Armatimonadota unclassified Armatimonadota Armatimonadota bacterium isolate ML34 015 33 MHB0938378.1
Bacillati Armatimonadota unclassified Armatimonadota Armatimonadota bacterium isolate AMR MDS 1680 HOF88490.1
Bacillati Armatimonadota unclassified Armatimonadota Armatimonadota bacterium isolate AS27yjCOA 96 iso1 NMC81352.1
Bacillati Armatimonadota unclassified Armatimonadota Armatimonadota bacterium isolate SMAG U4751 iso2 HEY3413255.1
Bacillati Armatimonadota unclassified Armatimonadota Armatimonadota bacterium isolate SMAG U4717 iso2 HEY3268005.1
Bacillati Armatimonadota unclassified Armatimonadota Armatimonadota bacterium isolate E14 metabat.bin.108 iso1 MDO8585688.1
Bacillati Armatimonadota unclassified Armatimonadota Armatimonadota bacterium isolate E14 maxbin.151 MDO8682078.1
Bacillati Armatimonadota unclassified Armatimonadota Armatimonadota bacterium isolate NC groundwater 630 Ag B 0.1um 56 30 iso2 MBI2844105.1
Bacillati Armatimonadota unclassified Armatimonadota Armatimonadota bacterium isolate AMR MDS 1991 HOM71329.1
Bacillati Armatimonadota unclassified Armatimonadota Armatimonadota bacterium isolate 6 bin.213 iso2 MEN6521043.1
Bacillati Armatimonadota unclassified Armatimonadota Armatimonadota bacterium isolate RM bin9 OCT21 iso1 MFQ3548557.1
Bacillati Armatimonadota unclassified Armatimonadota Armatimonadota bacterium isolate FK Sedi B Bin.35 MCL5105481.1
Bacillati Armatimonadota unclassified Armatimonadota Armatimonadota bacterium isolate LacPavin 0920 SED3 MAG 51 14 MCX6346166.1
Bacillati Armatimonadota unclassified Armatimonadota Armatimonadota bacterium isolate 5 bin.66 MEN6583277.1
Bacillati Armatimonadota unclassified Armatimonadota Armatimonadota bacterium isolate P60 sed 5 MGQ9454631.1
Bacillati Armatimonadota unclassified Armatimonadota Armatimonadota bacterium isolate KM4338 59 MGC8862555.1
Bacillati Armatimonadota unclassified Armatimonadota Armatimonadota bacterium isolate AS10tlH2TH 387 NLN78050.1
Bacillati Armatimonadota unclassified Armatimonadota Armatimonadota bacterium isolate SBF912LR0HQ0044 MGI6295034.1
Bacillati Armatimonadota unclassified Armatimonadota Armatimonadota bacterium isolate AS10tlH2TH 383 NLN76042.1
Bacillati Armatimonadota unclassified Armatimonadota Armatimonadota bacterium isolate SMAG U4714 iso1 HEY3283232.1
Bacillati Armatimonadota unclassified Armatimonadota Armatimonadota bacterium isolate P53 sed 10 iso1 MGQ9524787.1
Bacillati Armatimonadota unclassified Armatimonadota Armatimonadota bacterium isolate KM4338 66 iso1 MGC8833866.1
Bacillati Armatimonadota unclassified Armatimonadota Armatimonadota bacterium isolate SJ283 iso1 MCC6445534.1
Bacillati Armatimonadota unclassified Armatimonadota Armatimonadota bacterium isolate Uz U4 28 MEJ5296460.1
Bacillati Armatimonadota unclassified Armatimonadota Armatimonadota bacterium isolate SJ273 iso2 MCC6485588.1
Bacillati Armatimonadota unclassified Armatimonadota Armatimonadota bacterium isolate ELA409 iso2 MEI6916623.1
Bacillati Armatimonadota unclassified Armatimonadota Armatimonadota bacterium isolate NC groundwater 1072 Ag S2p5 66 82 MBI3912243.1
Bacillati Armatimonadota unclassified Armatimonadota Armatimonadota bacterium isolate C No T20 A bin.51 iso1 MCC2668218.1
Bacillati Armatimonadota unclassified Armatimonadota Armatimonadota bacterium isolate 21I 069.bin.27 MFN3651353.1
Bacillati Armatimonadota unclassified Armatimonadota Armatimonadota bacterium isolate SO 2016 CLC T2 64 iso1 MFA6664963.1
Bacillati Armatimonadota unclassified Armatimonadota Armatimonadota bacterium isolate HOT.MB2.102 iso2 MCL6519604.1
Bacillati Armatimonadota unclassified Armatimonadota Armatimonadota bacterium isolate SMAG U14200 iso1 HUV04601.1
Bacillati Armatimonadota unclassified Armatimonadota Armatimonadota bacterium isolate Kamch4342 D MF 100 MGB9587791.1
Bacillati Armatimonadota unclassified Armatimonadota Armatimonadota bacterium isolate Uz U4 35 iso2 MEJ5253429.1
Bacillati Armatimonadota unclassified Armatimonadota Armatimonadota bacterium isolate KatS3mg023 GIV19692.1
Bacillati Armatimonadota Fimbriimonadia Fimbriimonadia bacterium isolate TGS ARM5 MCW5934258.1
Bacillati Armatimonadota Fimbriimonadia Fimbriimonadales bacterium isolate SKYB126 MCS7300107.1
Bacillati Armatimonadota Fimbriimonadia Fimbriimonadia bacterium isolate MAG 05r iso1 MEP0765354.1

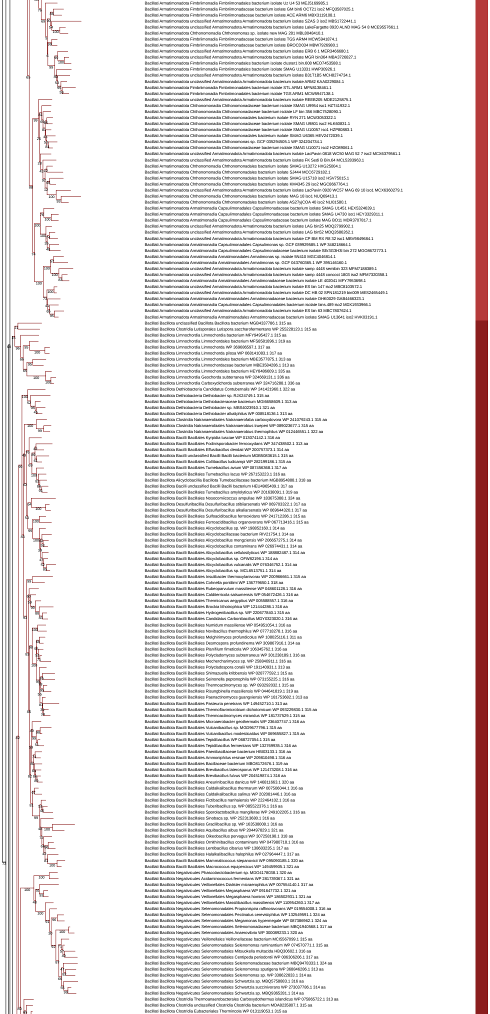

Bacillati Armatimonadota Fimbriimonadia Fimbriimonadales bacterium isolate Uz U4 53 MEJ5169985.1
Bacillati Armatimonadota Fimbriimonadia Fimbriimonadaceae bacterium isolate GM bin6 OCT21 iso2 MFQ3587025.1
Bacillati Armatimonadota Fimbriimonadia Fimbriimonadaceae bacterium isolate ACE ARM6 MBX3119108.1
Bacillati Armatimonadota unclassified Armatimonadota Armatimonadota bacterium isolate SZAS 3 iso2 MBS1722441.1
Bacillati Armatimonadota unclassified Armatimonadota Armatimonadota bacterium isolate LakeFargette 0920 ALND MAG 54 8 MCE9557661.1
Bacillati Armatimonadota Chthonomonadia Chthonomonas sp. isolate new MAG 281 MBL8048410.1
Bacillati Armatimonadota Fimbriimonadia Fimbriimonadaceae bacterium isolate TGS ARM4 MCW5941874.1
Bacillati Armatimonadota Fimbriimonadia Fimbriimonadaceae bacterium isolate BROCD034 MBW7926980.1
Bacillati Armatimonadota unclassified Armatimonadota Armatimonadota bacterium isolate ERB 6 1 MER3466680.1
Bacillati Armatimonadota unclassified Armatimonadota Armatimonadota bacterium isolate MGR bin364 MBA3726827.1
Bacillati Armatimonadota Fimbriimonadia Fimbriimonadales bacterium isolate cluster1 bin.608 MEO7453588.1
Bacillati Armatimonadota Fimbriimonadia Fimbriimonadales bacterium isolate SMAG U13331 HWP30926.1
Bacillati Armatimonadota unclassified Armatimonadota Armatimonadota bacterium isolate B31T1B5 MCH8274734.1
Bacillati Armatimonadota unclassified Armatimonadota Armatimonadota bacterium isolate ARM2 KAA0229084.1
Bacillati Armatimonadota Fimbriimonadia Fimbriimonadales bacterium isolate STL ARM1 MFN8138461.1
Bacillati Armatimonadota Fimbriimonadia Fimbriimonadales bacterium isolate TGS ARM1 MCW5947138.1
Bacillati Armatimonadota unclassified Armatimonadota Armatimonadota bacterium isolate REEB205 MDE2125875.1
Bacillati Armatimonadota Chthonomonadia Chthonomonadaceae bacterium isolate SMAG U9954 iso1 HZT41932.1
Bacillati Armatimonadota Chthonomonadia Chthonomonadaceae bacterium isolate LF bin 356 MBC7528090.1
Bacillati Armatimonadota Chthonomonadia Chthonomonadales bacterium isolate RYN 271 MCW3053322.1
Bacillati Armatimonadota Chthonomonadia Chthonomonadaceae bacterium isolate SMAG U9801 iso2 HLK60831.1
Bacillati Armatimonadota Chthonomonadia Chthonomonadaceae bacterium isolate SMAG U10057 iso1 HZP80883.1
Bacillati Armatimonadota Chthonomonadia Chthonomonadales bacterium isolate SMAG U6365 HEV2472039.1
Bacillati Armatimonadota Chthonomonadia Chthonomonas sp. GCF 035294505.1 WP 324204734.1
Bacillati Armatimonadota Chthonomonadia Chthonomonadaceae bacterium isolate SMAG U10071 iso2 HZO89061.1
Bacillati Armatimonadota unclassified Armatimonadota Armatimonadota bacterium isolate LacPavin 0818 WC50 MAG 52 7 iso2 MCX6379561.1
Bacillati Armatimonadota unclassified Armatimonadota Armatimonadota bacterium isolate FK Sedi B Bin.64 MCL5283963.1
Bacillati Armatimonadota Chthonomonadia Chthonomonadales bacterium isolate SMAG U13272 HXG25004.1
Bacillati Armatimonadota Chthonomonadia Chthonomonadales bacterium isolate SJ444 MCC6729182.1
Bacillati Armatimonadota Chthonomonadia Chthonomonadales bacterium isolate SMAG U15718 iso2 HSV75015.1
Bacillati Armatimonadota Chthonomonadia Chthonomonadales bacterium isolate KM4345 29 iso2 MGC8667764.1
Bacillati Armatimonadota unclassified Armatimonadota Armatimonadota bacterium isolate LacPavin 0920 WC57 MAG 69 10 iso1 MCX6360279.1
Bacillati Armatimonadota Chthonomonadia Chthonomonadales bacterium isolate MAG 18 iso1 NUQ69413.1
Bacillati Armatimonadota Chthonomonadia Chthonomonadales bacterium isolate AS27yjCOA 40 iso2 NLI01580.1
Bacillati Armatimonadota Armatimonadia Capsulimonadales Capsulimonadaceae bacterium isolate SMAG U1451 HEX5324639.1
Bacillati Armatimonadota Armatimonadia Capsulimonadales Capsulimonadaceae bacterium isolate SMAG U4730 iso1 HEY3329311.1
Bacillati Armatimonadota Armatimonadia Capsulimonadales Capsulimonadaceae bacterium isolate MAG BO11 MDR3707817.1
Bacillati Armatimonadota unclassified Armatimonadota Armatimonadota bacterium isolate LAG bin25 MDQ2799902.1
Bacillati Armatimonadota unclassified Armatimonadota Armatimonadota bacterium isolate LAG bin52 MDQ2686262.1
Bacillati Armatimonadota unclassified Armatimonadota Armatimonadota bacterium isolate CP BM RX R8 32 iso1 MBV9849684.1
Bacillati Armatimonadota Armatimonadia Capsulimonadales Capsulimonas sp. GCF 039929585.1 WP 348218664.1
Bacillati Armatimonadota Armatimonadia Capsulimonadales Capsulimonadaceae bacterium isolate SEr3G3H3I bin 272 MGO8672773.1
Bacillati Armatimonadota Armatimonadia Armatimonadales Armatimonas sp. isolate 5N410 MGC4046814.1
Bacillati Armatimonadota Armatimonadia Armatimonadales Armatimonas sp. GCF 043760365.1 WP 395146160.1
Bacillati Armatimonadota unclassified Armatimonadota Armatimonadota bacterium isolate samp 4448 semibin 323 MFM7188389.1
Bacillati Armatimonadota unclassified Armatimonadota Armatimonadota bacterium isolate samp 4448 concoct 1803 iso2 MFM7320358.1
Bacillati Armatimonadota Armatimonadia Armatimonadales Armatimonadaceae bacterium isolate LE 402041 MFY7953698.1
Bacillati Armatimonadota unclassified Armatimonadota Armatimonadota bacterium isolate ES bin 147 iso2 MBC8103572.1
Bacillati Armatimonadota unclassified Armatimonadota Armatimonadota bacterium isolate DC HB 02 SPN181219 bin009 MES2465449.1
Bacillati Armatimonadota Armatimonadia Armatimonadales Armatimonadaceae bacterium isolate OHK0029 GAB4466323.1
Bacillati Armatimonadota Armatimonadia Capsulimonadales Capsulimonadales bacterium isolate bins.489 iso2 MDX1933966.1
Bacillati Armatimonadota unclassified Armatimonadota Armatimonadota bacterium isolate ES bin 63 MBC7807624.1
Bacillati Armatimonadota Armatimonadia Armatimonadales Armatimonadaceae bacterium isolate SMAG U13641 iso2 HVK03191.1
Bacillati Bacillota unclassified Bacillota Bacillota bacterium MGB4337786.1 315 aa
Bacillati Bacillota Clostridia Lutisporales Lutispora saccharofermentans WP 255228123.1 315 aa
Bacillati Bacillota Limnochordia Limnochordia bacterium MFY9495427.1 315 aa
Bacillati Bacillota Limnochordia Limnochordales bacterium MFS8581896.1 319 aa
Bacillati Bacillota Limnochordia Limnochorda WP 369686597.1 317 aa
Bacillati Bacillota Limnochordia Limnochorda pilosa WP 068141083.1 317 aa
Bacillati Bacillota Limnochordia Limnochordales bacterium MBE3577875.1 313 aa
Bacillati Bacillota Limnochordia Limnochordaceae bacterium MBE3584286.1 313 aa
Bacillati Bacillota Limnochordia Limnochordales bacterium HEY8486609.1 335 aa
Bacillati Bacillota Limnochordia Geochorda subterranea WP 324669131.1 336 aa
Bacillati Bacillota Limnochordia Carboxydichorda subterranea WP 324716288.1 336 aa
Bacillati Bacillota Dethiobacteria Candidatus Contubernalis WP 241421960.1 322 aa
Bacillati Bacillota Dethiobacteria Dethiobacter sp. RJX24749.1 315 aa
Bacillati Bacillota Dethiobacteria Dethiobacteraceae bacterium MGI6658609.1 313 aa
Bacillati Bacillota Dethiobacteria Dethiobacter sp. MBS4023910.1 321 aa
Bacillati Bacillota Dethiobacteria Dethiobacter alkaliphilus WP 008518136.1 313 aa
Bacillati Bacillota Clostridia Natranaerobiales Natranaerofaba carboxydovora WP 241079243.1 315 aa
Bacillati Bacillota Clostridia Natranaerobiales Natranaerobius trueperi WP 089023677.1 315 aa
Bacillati Bacillota Clostridia Natranaerobiales Natranaerobius thermophilus WP 012446551.1 322 aa
Bacillati Bacillota Bacilli Bacillales Kyrpidia tusciae WP 013074142.1 316 aa
Bacillati Bacillota Bacilli Bacillales Fodinisporobacter ferrooxydans WP 347438502.1 313 aa
Bacillati Bacillota Bacilli Bacillales Effusibacillus dendaii WP 200757373.1 314 aa
Bacillati Bacillota Bacilli unclassified Bacilli Bacilli bacterium MDB5083615.1 315 aa
Bacillati Bacillota Bacilli Bacillales Collibacillus ludicampi WP 282199186.1 315 aa
Bacillati Bacillota Bacilli Bacillales Tumebacillus avium WP 087456368.1 317 aa
Bacillati Bacillota Bacilli Bacillales Tumebacillus lacus WP 267153223.1 316 aa
Bacillati Bacillota Alicyclobacillia Bacillota Tumebacillaceae bacterium MGB8954888.1 318 aa
Bacillati Bacillota Bacilli unclassified Bacilli Bacilli bacterium HEU4965409.1 317 aa
Bacillati Bacillota Bacilli Bacillales Tumebacillus amylolyticus WP 201638091.1 319 aa
Bacillati Bacillota Bacilli Bacillales Nosocomiicoccus ampullae WP 183675388.1 324 aa
Bacillati Bacillota Desulfuribacillia Desulfuribacillus stibiiarsenatis WP 069703322.1 317 aa
Bacillati Bacillota Desulfuribacillia Desulfuribacillus alkaliarsenatis WP 069644320.1 317 aa
Bacillati Bacillota Bacilli Bacillales Sulfoacidibacillus ferrooxidans WP 241712286.1 315 aa
Bacillati Bacillota Bacilli Bacillales Ferroacidibacillus organovorans WP 067713416.1 315 aa
Bacillati Bacillota Bacilli Bacillales Alicyclobacillus sp. WP 198852160.1 314 aa
Bacillati Bacillota Bacilli Bacillales Alicyclobacillaceae bacterium RIV21754.1 314 aa
Bacillati Bacillota Bacilli Bacillales Alicyclobacillus mengziensis WP 206657275.1 314 aa
Bacillati Bacillota Bacilli Bacillales Alicyclobacillus contaminans WP 026974431.1 314 aa
Bacillati Bacillota Bacilli Bacillales Alicyclobacillus cellulosilyticus WP 188882487.1 314 aa
Bacillati Bacillota Bacilli Bacillales Alicyclobacillus sp. OFW82196.1 314 aa
Bacillati Bacillota Bacilli Bacillales Alicyclobacillus vulcanalis WP 076346752.1 314 aa
Bacillati Bacillota Bacilli Bacillales Alicyclobacillus sp. MCL6513751.1 314 aa
Bacillati Bacillota Bacilli Bacillales Insulibacter thermoxylanivorax WP 200966661.1 315 aa
Bacillati Bacillota Bacilli Bacillales Cohnella pontilimi WP 136779650.1 318 aa
Bacillati Bacillota Bacilli Bacillales Rubeoparvulum massiliense WP 048601128.1 316 aa
Bacillati Bacillota Bacilli Bacillales Calditerricola satsumensis WP 054672426.1 316 aa
Bacillati Bacillota Bacilli Bacillales Thermicanus aegyptius WP 005588557.1 316 aa
Bacillati Bacillota Bacilli Bacillales Brockia lithotrophica WP 121444286.1 316 aa
Bacillati Bacillota Bacilli Bacillales Hydrogenibacillus sp. WP 220677840.1 315 aa
Bacillati Bacillota Bacilli Bacillales Candidatus Carbonibacillus MDY0323020.1 316 aa
Bacillati Bacillota Bacilli Bacillales Numidum massiliense WP 054951054.1 316 aa
Bacillati Bacillota Bacilli Bacillales Novibacillus thermophilus WP 077718278.1 316 aa
Bacillati Bacillota Bacilli Bacillales Melghirimyces profundicolus WP 108025116.1 311 aa
Bacillati Bacillota Bacilli Bacillales Desmospora profundinema WP 309867916.1 314 aa
Bacillati Bacillota Bacilli Bacillales Planifilum fimeticola WP 106345762.1 316 aa
Bacillati Bacillota Bacilli Bacillales Polycladomyces subterraneus WP 301238189.1 316 aa
Bacillati Bacillota Bacilli Bacillales Mechercharimyces sp. WP 258840911.1 316 aa
Bacillati Bacillota Bacilli Bacillales Polycladospora coralii WP 191140931.1 313 aa
Bacillati Bacillota Bacilli Bacillales Shimazuella kribbensis WP 028777592.1 315 aa
Bacillati Bacillota Bacilli Bacillales Seinonella peptonophila WP 073155225.1 316 aa
Bacillati Bacillota Bacilli Bacillales Thermoactinomyces sp. WP 093292032.1 315 aa
Bacillati Bacillota Bacilli Bacillales Risungbinella massiliensis WP 044641819.1 319 aa
Bacillati Bacillota Bacilli Bacillales Paenactinomyces guangxiensis WP 181753682.1 313 aa
Bacillati Bacillota Bacilli Bacillales Pasteuria penetrans WP 149452710.1 313 aa
Bacillati Bacillota Bacilli Bacillales Thermoflavimicrobium dichotomicum WP 093229830.1 315 aa
Bacillati Bacillota Bacilli Bacillales Thermoactinomyces mirandus WP 181737529.1 315 aa
Bacillati Bacillota Bacilli Bacillales Microaerobacter geothermalis WP 236407747.1 316 aa
Bacillati Bacillota Bacilli Bacillales Vulcanibacillus sp. MGD9677796.1 315 aa
Bacillati Bacillota Bacilli Bacillales Vulcanibacillus modesticaldus WP 069655827.1 315 aa
Bacillati Bacillota Bacilli Bacillales Tepidibacillus WP 068727054.1 315 aa
Bacillati Bacillota Bacilli Bacillales Tepidibacillus fermentans WP 132769935.1 316 aa
Bacillati Bacillota Bacilli Bacillales Paenibacillaceae bacterium HBI03133.1 316 aa
Bacillati Bacillota Bacilli Bacillales Ammoniphilus resinae WP 209810498.1 316 aa
Bacillati Bacillota Bacilli Bacillales Bacillaceae bacterium MBO8172676.1 319 aa
Bacillati Bacillota Bacilli Bacillales Brevibacillus laterosporus WP 121473208.1 316 aa
Bacillati Bacillota Bacilli Bacillales Brevibacillus fulvus WP 204519874.1 316 aa
Bacillati Bacillota Bacilli Bacillales Aneurinibacillus danicus WP 146811663.1 320 aa
Bacillati Bacillota Bacilli Bacillales Caldalkalibacillus thermarum WP 007506044.1 316 aa
Bacillati Bacillota Bacilli Bacillales Caldalkalibacillus salinus WP 202081446.1 316 aa
Bacillati Bacillota Bacilli Bacillales Fictibacillus nanhaiensis WP 222464102.1 316 aa
Bacillati Bacillota Bacilli Bacillales Tuberibacillus sp. WP 085522376.1 316 aa
Bacillati Bacillota Bacilli Bacillales Sporolactobacillus mangiferae WP 249102205.1 316 aa
Bacillati Bacillota Bacilli Bacillales Sinobaca sp. WP 252313680.1 316 aa
Bacillati Bacillota Bacilli Bacillales Gracilibacillus sp. WP 163538008.1 316 aa
Bacillati Bacillota Bacilli Bacillales Aquibacillus albus WP 204497829.1 321 aa
Bacillati Bacillota Bacilli Bacillales Oikeobacillus pervagus WP 307258198.1 318 aa
Bacillati Bacillota Bacilli Bacillales Ornithinibacillus contaminans WP 047980718.1 316 aa
Bacillati Bacillota Bacilli Bacillales Lentibacillus cibarius WP 138603235.1 317 aa
Bacillati Bacillota Bacilli Bacillales Halalkalibacillus halophilus WP 027964447.1 317 aa
Bacillati Bacillota Bacilli Bacillales Mammaliicoccus stepanovicii WP 095090185.1 320 aa
Bacillati Bacillota Bacilli Bacillales Macrococcus equipercicus WP 149459905.1 321 aa
Bacillati Bacillota Negativicutes Phascolarctobacterium sp. MDO4178038.1 320 aa
Bacillati Bacillota Negativicutes Acidaminococcus fermentans WP 281739367.1 321 aa
Bacillati Bacillota Negativicutes Veillonellales Dialister micraerophilus WP 007554140.1 317 aa
Bacillati Bacillota Negativicutes Veillonellales Megasphaera WP 091647732.1 321 aa
Bacillati Bacillota Negativicutes Veillonellales Megasphaera hominis WP 186502931.1 321 aa
Bacillati Bacillota Negativicutes Veillonellales Massilibacillus massiliensis WP 110954260.1 317 aa
Bacillati Bacillota Negativicutes Selenomonadales Propionispira raffinosivorans WP 019554008.1 316 aa
Bacillati Bacillota Negativicutes Selenomonadales Pectinatus cerevisiiphilus WP 132549591.1 324 aa
Bacillati Bacillota Negativicutes Selenomonadales Megamonas hypermegale WP 087386962.1 324 aa
Bacillati Bacillota Negativicutes Selenomonadales Selenomonadaceae bacterium MBQ1940568.1 317 aa
Bacillati Bacillota Negativicutes Selenomonadales Anaerovibrio WP 300089233.1 320 aa
Bacillati Bacillota Negativicutes Veillonellales Veillonellaceae bacterium MCI5567099.1 315 aa
Bacillati Bacillota Negativicutes Selenomonadales Selenomonas ruminantium WP 074570771.1 315 aa
Bacillati Bacillota Negativicutes Selenomonadales Mitsuokella multacida HBQ30602.1 316 aa
Bacillati Bacillota Negativicutes Selenomonadales Centipeda periodontii WP 006306206.1 317 aa
Bacillati Bacillota Negativicutes Selenomonadales Selenomonadaceae bacterium MBQ9478333.1 324 aa
Bacillati Bacillota Negativicutes Selenomonadales Selenomonas sputigena WP 368846286.1 313 aa
Bacillati Bacillota Negativicutes Selenomonadales Selenomonas sp. WP 338622833.1 314 aa
Bacillati Bacillota Negativicutes Selenomonadales Schwartzia sp. MBQ5758883.1 316 aa
Bacillati Bacillota Negativicutes Selenomonadales Schwartzia succinivorans WP 273037786.1 314 aa
Bacillati Bacillota Negativicutes Selenomonadales Schwartzia sp. MBQ9365281.1 314 aa
Bacillati Bacillota Clostridia Thermoanaerobacterales Carboxydothermus islandicus WP 075865722.1 313 aa
Bacillati Bacillota Clostridia unclassified Clostridia Clostridia bacterium MDA8235807.1 315 aa
Bacillati Bacillota Clostridia Eubacteriales Thermincola WP 013119053.1 315 aa

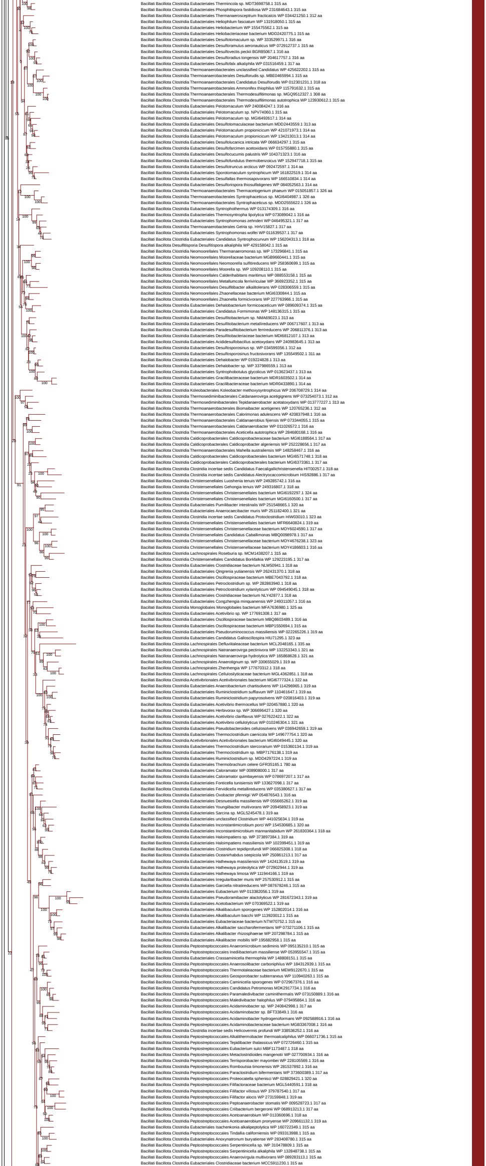

Bacillati Bacillota Clostridia Eubacteriales Thermincola sp. MDT3698758.1 315 aa
Bacillati Bacillota Clostridia Eubacteriales Phosphitispora fastidiosa WP 231684643.1 315 aa
Bacillati Bacillota Clostridia Eubacteriales Thermanaerosceptrum fracticalcis WP 034421250.1 312 aa
Bacillati Bacillota Clostridia Eubacteriales Heliophilum fasciatum WP 131918050.1 315 aa
Bacillati Bacillota Clostridia Eubacteriales Heliobacterium WP 155475562.1 315 aa
Bacillati Bacillota Clostridia Eubacteriales Heliobacteriaceae bacterium MDD2420775.1 315 aa
Bacillati Bacillota Clostridia Eubacteriales Desulfotomaculum sp. WP 333529971.1 316 aa
Bacillati Bacillota Clostridia Eubacteriales Desulforamulus aeronauticus WP 072912737.1 315 aa
Bacillati Bacillota Clostridia Eubacteriales Desulfovectis peckii BGR85067.1 316 aa
Bacillati Bacillota Clostridia Eubacteriales Desulforadius tongensis WP 204617757.1 316 aa
Bacillati Bacillota Clostridia Eubacteriales Desulfofalx alkaliphila WP 031516459.1 317 aa
Bacillati Bacillota Clostridia Thermoanaerobacterales unclassified Candidatus WP 425622202.1 315 aa
Bacillati Bacillota Clostridia Thermoanaerobacterales Desulforudis sp. MBE0465994.1 315 aa
Bacillati Bacillota Clostridia Thermoanaerobacterales Candidatus Desulforudis WP 012301231.1 318 aa
Bacillati Bacillota Clostridia Thermoanaerobacterales Ammonifex thiophilus WP 115791632.1 315 aa
Bacillati Bacillota Clostridia Thermoanaerobacterales Thermodesulfitimonas sp. MGQ9512327.1 308 aa
Bacillati Bacillota Clostridia Thermoanaerobacterales Thermodesulfitimonas autotrophica WP 123930612.1 315 aa
Bacillati Bacillota Clostridia Eubacteriales Pelotomaculum WP 240084247.1 316 aa
Bacillati Bacillota Clostridia Eubacteriales Pelotomaculum sp. NPV74060.1 315 aa
Bacillati Bacillota Clostridia Eubacteriales Pelotomaculum sp. MGI6492617.1 314 aa
Bacillati Bacillota Clostridia Eubacteriales Desulfotomaculaceae bacterium MDD2443559.1 313 aa
Bacillati Bacillota Clostridia Eubacteriales Pelotomaculum propionicicum WP 421071973.1 314 aa
Bacillati Bacillota Clostridia Eubacteriales Pelotomaculum propionicicum WP 134213013.1 314 aa
Bacillati Bacillota Clostridia Eubacteriales Desulfolucanica intricata WP 066634297.1 315 aa
Bacillati Bacillota Clostridia Eubacteriales Desulfofarcimen acetoxidans WP 015755880.1 315 aa
Bacillati Bacillota Clostridia Eubacteriales Desulfocucumis palustris WP 104371323.1 316 aa
Bacillati Bacillota Clostridia Eubacteriales Desulfofundulus thermobenzoicus WP 152947718.1 315 aa
Bacillati Bacillota Clostridia Eubacteriales Desulfotruncus arcticus WP 092472597.1 314 aa
Bacillati Bacillota Clostridia Eubacteriales Sporotomaculum syntrophicum WP 161822519.1 314 aa
Bacillati Bacillota Clostridia Eubacteriales Desulfallas thermosapovorans WP 166510834.1 314 aa
Bacillati Bacillota Clostridia Eubacteriales Desulfonispora thiosulfatigenes WP 084052563.1 314 aa
Bacillati Bacillota Clostridia Thermoanaerobacterales Thermacetogenium phaeum WP 015051857.1 326 aa
Bacillati Bacillota Clostridia Thermoanaerobacterales Syntrophaceticus sp. MGI6404987.1 326 aa
Bacillati Bacillota Clostridia Thermoanaerobacterales Syntrophaceticus sp. MDD2555622.1 326 aa
Bacillati Bacillota Clostridia Eubacteriales Syntrophothermus WP 013174309.1 316 aa
Bacillati Bacillota Clostridia Eubacteriales Thermosyntropha lipolytica WP 073089042.1 316 aa
Bacillati Bacillota Clostridia Eubacteriales Syntrophomonas zehnderi WP 046495321.1 317 aa
Bacillati Bacillota Clostridia Thermoanaerobacterales Gelria sp. HHV15827.1 317 aa
Bacillati Bacillota Clostridia Eubacteriales Syntrophomonas wolfei WP 011639537.1 317 aa
Bacillati Bacillota Clostridia Eubacteriales Candidatus Syntrophocurvum WP 156204313.1 318 aa
Bacillati Bacillota Desulfitisporia Desulfitispora alkaliphila WP 429158042.1 315 aa
Bacillati Bacillota Clostridia Neomoorellales Thermanaeromonas sp. WP 173296841.1 315 aa
Bacillati Bacillota Clostridia Neomoorellales Moorellaceae bacterium MGB9660441.1 315 aa
Bacillati Bacillota Clostridia Neomoorellales Neomoorella sulfitireducens WP 258360699.1 315 aa
Bacillati Bacillota Clostridia Neomoorellales Moorella sp. WP 109208110.1 315 aa
Bacillati Bacillota Clostridia Neomoorellales Calderihabitans maritimus WP 088553158.1 315 aa
Bacillati Bacillota Clostridia Neomoorellales Metallumcola ferriviriculae WP 366923352.1 315 aa
Bacillati Bacillota Clostridia Neomoorellales Desulfitibacter alkalitolerans WP 028306559.1 315 aa
Bacillati Bacillota Clostridia Neomoorellales Zhaonellaceae bacterium MGI6330844.1 315 aa
Bacillati Bacillota Clostridia Neomoorellales Zhaonella formicivorans WP 227763966.1 315 aa
Bacillati Bacillota Clostridia Eubacteriales Dehalobacterium formicoaceticum WP 089609374.1 315 aa
Bacillati Bacillota Clostridia Eubacteriales Candidatus Formimonas WP 148136315.1 315 aa
Bacillati Bacillota Clostridia Eubacteriales Desulfitobacterium sp. NMA69023.1 313 aa
Bacillati Bacillota Clostridia Eubacteriales Desulfitobacterium metallireducens WP 006717607.1 313 aa
Bacillati Bacillota Clostridia Eubacteriales Paradesulfitobacterium ferrireducens WP 206811376.1 313 aa
Bacillati Bacillota Clostridia Eubacteriales Desulfitobacteriaceae bacterium MDI6812107.1 313 aa
Bacillati Bacillota Clostridia Eubacteriales Acididesulfobacillus acetoxydans WP 240983645.1 313 aa
Bacillati Bacillota Clostridia Eubacteriales Desulfosporosinus sp. WP 034599356.1 312 aa
Bacillati Bacillota Clostridia Eubacteriales Desulfosporosinus fructosivorans WP 135549502.1 311 aa
Bacillati Bacillota Clostridia Eubacteriales Dehalobacter WP 019224828.1 313 aa
Bacillati Bacillota Clostridia Eubacteriales Dehalobacter sp. WP 337986559.1 313 aa
Bacillati Bacillota Clostridia Eubacteriales Syntrophobotulus glycolicus WP 013623437.1 313 aa
Bacillati Bacillota Clostridia Eubacteriales Gracilibacteraceae bacterium MDR1603502.1 314 aa
Bacillati Bacillota Clostridia Eubacteriales Gracilibacteraceae bacterium MDR0433890.1 314 aa
Bacillati Bacillota Clostridia Koleobacterales Koleobacter methoxysyntrophicus WP 206708729.1 314 aa
Bacillati Bacillota Clostridia Thermosediminibacterales Caldanaerovirga acetigignens WP 073254073.1 312 aa
Bacillati Bacillota Clostridia Thermosediminibacterales Tepidanaerobacter acetatoxydans WP 013777227.1 313 aa
Bacillati Bacillota Clostridia Thermoanaerobacterales Biomaibacter acetigenes WP 120765236.1 312 aa
Bacillati Bacillota Clostridia Thermoanaerobacterales Calorimonas adulescens WP 420837948.1 316 aa
Bacillati Bacillota Clostridia Thermoanaerobacterales Caldanaerobius fijiensis WP 073344055.1 315 aa
Bacillati Bacillota Clostridia Thermoanaerobacterales Caldanaerobacter WP 011026572.1 316 aa
Bacillati Bacillota Clostridia Thermoanaerobacterales Aceticella autotrophica WP 284680168.1 316 aa
Bacillati Bacillota Clostridia Caldicoprobacterales Caldicoprobacteraceae bacterium MGI6188564.1 317 aa
Bacillati Bacillota Clostridia Caldicoprobacterales Caldicoprobacter algeriensis WP 252228656.1 317 aa
Bacillati Bacillota Clostridia Thermoanaerobacterales Mahella australiensis WP 148258467.1 316 aa
Bacillati Bacillota Clostridia Caldicoprobacterales Caldicoprobacterales bacterium MGI6571748.1 318 aa
Bacillati Bacillota Clostridia Caldicoprobacterales Caldicoprobacterales bacterium MGI6373361.1 317 aa
Bacillati Bacillota Clostridia Clostridia incertae sedis Candidatus Faecaligallichristensenella HIT00257.1 318 aa
Bacillati Bacillota Clostridia Clostridia incertae sedis Candidatus Alectryocaccomicrobium HIS92886.1 317 aa
Bacillati Bacillota Clostridia Christensenellales Luoshenia tenuis WP 249285742.1 316 aa
Bacillati Bacillota Clostridia Christensenellales Gehongia tenuis WP 249316807.1 318 aa
Bacillati Bacillota Clostridia Christensenellales Christensenellales bacterium MGI6192297.1 324 aa
Bacillati Bacillota Clostridia Christensenellales Christensenellales bacterium MGI6160500.1 317 aa
Bacillati Bacillota Clostridia Eubacteriales Pumilibacter intestinalis WP 251548665.1 320 aa
Bacillati Bacillota Clostridia Eubacteriales Anaerocaecibacter muris WP 251182400.1 321 aa
Bacillati Bacillota Clostridia Clostridia incertae sedis Candidatus Protoclostridium HIW03010.1 323 aa
Bacillati Bacillota Clostridia Christensenellales Christensenellales bacterium MFR6640824.1 319 aa
Bacillati Bacillota Clostridia Christensenellales Christensenellaceae bacterium MDY6024590.1 317 aa
Bacillati Bacillota Clostridia Christensenellales Candidatus Caballimonas MBQ0098978.1 317 aa
Bacillati Bacillota Clostridia Christensenellales Christensenellaceae bacterium MDY4676238.1 323 aa
Bacillati Bacillota Clostridia Christensenellales Christensenellaceae bacterium MDY4186603.1 316 aa
Bacillati Bacillota Clostridia Lachnospirales Roseburia sp. MCM1438207.1 315 aa
Bacillati Bacillota Clostridia Christensenellales Candidatus Borkfalkia WP 129223195.1 317 aa
Bacillati Bacillota Clostridia Eubacteriales Clostridiaceae bacterium NLM50941.1 318 aa
Bacillati Bacillota Clostridia Eubacteriales Qingrenia yutianensis WP 262431370.1 318 aa
Bacillati Bacillota Clostridia Eubacteriales Oscillospiraceae bacterium MBE7043792.1 318 aa
Bacillati Bacillota Clostridia Eubacteriales Petroclostridium sp. WP 282863940.1 318 aa
Bacillati Bacillota Clostridia Eubacteriales Petroclostridium xylanilyticum WP 094549045.1 318 aa
Bacillati Bacillota Clostridia Eubacteriales Clostridiaceae bacterium NLY42877.1 318 aa
Bacillati Bacillota Clostridia Eubacteriales Congzhengia minquanensis WP 249311057.1 316 aa
Bacillati Bacillota Clostridia Monoglobales Monoglobales bacterium MFA7636980.1 325 aa
Bacillati Bacillota Clostridia Eubacteriales Acetivibrio sp. WP 177691308.1 317 aa
Bacillati Bacillota Clostridia Eubacteriales Oscillospiraceae bacterium MBQ8603489.1 316 aa
Bacillati Bacillota Clostridia Eubacteriales Oscillospiraceae bacterium MBP1550694.1 315 aa
Bacillati Bacillota Clostridia Eubacteriales Pseudoruminococcus massiliensis WP 022265226.1 319 aa
Bacillati Bacillota Clostridia Eubacteriales Candidatus Galloscillospira HIU71295.1 323 aa
Bacillati Bacillota Clostridia Lachnospirales Defluviitaleaceae bacterium MCL2048165.1 335 aa
Bacillati Bacillota Clostridia Lachnospirales Natranaerovirga pectinivora WP 132253343.1 321 aa
Bacillati Bacillota Clostridia Lachnospirales Natranaerovirga hydrolytica WP 165868628.1 321 aa
Bacillati Bacillota Clostridia Lachnospirales Anaerotignum sp. WP 330655029.1 319 aa
Bacillati Bacillota Clostridia Lachnospirales Zhenhengia WP 177670312.1 318 aa
Bacillati Bacillota Clostridia Lachnospirales Cellulosilyticaceae bacterium MGL4362851.1 318 aa
Bacillati Bacillota Clostridia Acetivibrionales Acetivibrionales bacterium MGI6777324.1 322 aa
Bacillati Bacillota Clostridia Eubacteriales Anaerobacterium chartisolvens WP 114296965.1 319 aa
Bacillati Bacillota Clostridia Eubacteriales Ruminiclostridium sufflavum WP 110461647.1 319 aa
Bacillati Bacillota Clostridia Eubacteriales Ruminiclostridium papyrosolvens WP 020816403.1 319 aa
Bacillati Bacillota Clostridia Eubacteriales Acetivibrio thermocellus WP 020457880.1 320 aa
Bacillati Bacillota Clostridia Eubacteriales Herbivorax sp. WP 306696427.1 320 aa
Bacillati Bacillota Clostridia Eubacteriales Acetivibrio clariflavus WP 027622422.1 322 aa
Bacillati Bacillota Clostridia Eubacteriales Acetivibrio cellulolyticus WP 010246304.1 321 aa
Bacillati Bacillota Clostridia Eubacteriales Pseudobacteroides cellulosolvens WP 036942659.1 319 aa
Bacillati Bacillota Clostridia Eubacteriales Thermoclostridium caenicola WP 149677754.1 320 aa
Bacillati Bacillota Clostridia Acetivibrionales Acetivibrionales bacterium MGI6049445.1 320 aa
Bacillati Bacillota Clostridia Eubacteriales Thermoclostridium stercorarium WP 015360134.1 319 aa
Bacillati Bacillota Clostridia Eubacteriales Thermoclostridium sp. MBP7176138.1 319 aa
Bacillati Bacillota Clostridia Eubacteriales Ruminiclostridium sp. MDD4297224.1 319 aa
Bacillati Bacillota Clostridia Eubacteriales Thermobrachium celere GFR35165.1 780 aa
Bacillati Bacillota Clostridia Eubacteriales Caloramator WP 008908000.1 317 aa
Bacillati Bacillota Clostridia Eubacteriales Caloramator quimbayensis WP 078697207.1 317 aa
Bacillati Bacillota Clostridia Eubacteriales Fonticella tunisiensis WP 133627098.1 317 aa
Bacillati Bacillota Clostridia Eubacteriales Fervidicella metallireducens WP 035380627.1 317 aa
Bacillati Bacillota Clostridia Eubacteriales Oxobacter pfennigii WP 054876543.1 316 aa
Bacillati Bacillota Clostridia Eubacteriales Desnuesiella massiliensis WP 055665262.1 319 aa
Bacillati Bacillota Clostridia Eubacteriales Youngiibacter multivorans WP 209458923.1 319 aa
Bacillati Bacillota Clostridia Eubacteriales Sarcina sp. MGL5245478.1 319 aa
Bacillati Bacillota Clostridia Eubacteriales unclassified Clostridium WP 441025634.1 319 aa
Bacillati Bacillota Clostridia Eubacteriales Inconstantimicrobium porci WP 154530685.1 320 aa
Bacillati Bacillota Clostridia Eubacteriales Inconstantimicrobium mannanitabidum WP 261830364.1 318 aa
Bacillati Bacillota Clostridia Eubacteriales Haloimpatiens sp. WP 373897384.1 319 aa
Bacillati Bacillota Clostridia Eubacteriales Haloimpatiens massiliensis WP 102399451.1 319 aa
Bacillati Bacillota Clostridia Eubacteriales Clostridium tepidiprofundi WP 066825308.1 318 aa
Bacillati Bacillota Clostridia Eubacteriales Oceanirhabdus seepicola WP 250861213.1 317 aa
Bacillati Bacillota Clostridia Eubacteriales Hathewaya massiliensis WP 142413519.1 319 aa
Bacillati Bacillota Clostridia Eubacteriales Hathewaya proteolytica WP 072902944.1 319 aa
Bacillati Bacillota Clostridia Eubacteriales Hathewaya limosa WP 111944166.1 319 aa
Bacillati Bacillota Clostridia Eubacteriales Irregularibacter muris WP 257530912.1 315 aa
Bacillati Bacillota Clostridia Eubacteriales Garciella nitratireducens WP 087678246.1 315 aa
Bacillati Bacillota Clostridia Eubacteriales Eubacterium WP 013382056.1 319 aa
Bacillati Bacillota Clostridia Eubacteriales Pseudoramibacter alactolyticus WP 281672343.1 319 aa
Bacillati Bacillota Clostridia Eubacteriales Acetobacterium WP 070369522.1 319 aa
Bacillati Bacillota Clostridia Eubacteriales Alkalibaculum sporogenes WP 152802014.1 316 aa
Bacillati Bacillota Clostridia Eubacteriales Alkalibaculum bacchi WP 113920012.1 315 aa
Bacillati Bacillota Clostridia Eubacteriales Eubacteriaceae bacterium NTW70752.1 315 aa
Bacillati Bacillota Clostridia Eubacteriales Alkalibacter saccharofermentans WP 073271106.1 315 aa
Bacillati Bacillota Clostridia Eubacteriales Alkalibacter rhizosphaerae WP 207298784.1 315 aa
Bacillati Bacillota Clostridia Eubacteriales Alkalibacter mobilis WP 195682958.1 315 aa
Bacillati Bacillota Clostridia Peptostreptococcales Anaeromicrobium sediminis WP 095135210.1 315 aa
Bacillati Bacillota Clostridia Peptostreptococcales Inediibacterium massiliense WP 053955547.1 315 aa
Bacillati Bacillota Clostridia Eubacteriales Crassaminicella thermophila WP 148808151.1 315 aa
Bacillati Bacillota Clostridia Peptostreptococcales Anaerosolibacter carboniphilus WP 184312939.1 315 aa
Bacillati Bacillota Clostridia Peptostreptococcales Thermotaleaceae bacterium MEW9122670.1 315 aa
Bacillati Bacillota Clostridia Peptostreptococcales Geosporobacter subterraneus WP 110940263.1 315 aa
Bacillati Bacillota Clostridia Peptostreptococcales Caminicella sporogenes WP 072967376.1 316 aa
Bacillati Bacillota Clostridia Peptostreptococcales Candidatus Petromonas MDK2917734.1 316 aa
Bacillati Bacillota Clostridia Peptostreptococcales Paramaledivibacter caminithermalis WP 073150889.1 316 aa
Bacillati Bacillota Clostridia Peptostreptococcales Maledivibacter halophilus WP 079495864.1 316 aa
Bacillati Bacillota Clostridia Peptostreptococcales Acidaminobacter sp. WP 240842998.1 317 aa
Bacillati Bacillota Clostridia Peptostreptococcales Acidaminobacter sp. BFT33849.1 316 aa
Bacillati Bacillota Clostridia Peptostreptococcales Acidaminobacter hydrogenoformans WP 092588916.1 316 aa
Bacillati Bacillota Clostridia Peptostreptococcales Acidaminobacteraceae bacterium MGB3367008.1 316 aa
Bacillati Bacillota Clostridia Clostridia incertae sedis Helicovermis profundi WP 338536252.1 316 aa
Bacillati Bacillota Clostridia Peptostreptococcales Alkalithermobacter thermoalcaliphilus WP 066071736.1 315 aa
Bacillati Bacillota Clostridia Peptostreptococcales Tepidibacter thalassicus WP 072726460.1 315 aa
Bacillati Bacillota Clostridia Peptostreptococcales Eubacterium sulci MBF1173487.1 318 aa
Bacillati Bacillota Clostridia Peptostreptococcales Metaclostridioides mangenotii WP 027700934.1 316 aa
Bacillati Bacillota Clostridia Peptostreptococcales Terrisporobacter mayombei WP 228105569.1 316 aa
Bacillati Bacillota Clostridia Peptostreptococcales Romboutsia timonensis WP 281537892.1 316 aa
Bacillati Bacillota Clostridia Peptostreptococcales Paraclostridium bifermentans WP 373600389.1 317 aa
Bacillati Bacillota Clostridia Peptostreptococcales Proteocatella sphenisci WP 028829421.1 320 aa
Bacillati Bacillota Clostridia Peptostreptococcales Filifactoraceae bacterium MGL5440591.1 318 aa
Bacillati Bacillota Clostridia Peptostreptococcales Filifactor villosus WP 379787540.1 317 aa
Bacillati Bacillota Clostridia Peptostreptococcales Filifactor alocis WP 273159848.1 319 aa
Bacillati Bacillota Clostridia Peptostreptococcales Peptoanaerobacter stomatis WP 009528723.1 317 aa
Bacillati Bacillota Clostridia Peptostreptococcales Criibacterium bergeronii WP 068913213.1 317 aa
Bacillati Bacillota Clostridia Peptostreptococcales Acetoanaerobium WP 013360696.1 318 aa
Bacillati Bacillota Clostridia Peptostreptococcales Acetoanaerobium pronyense WP 209661132.1 319 aa
Bacillati Bacillota Clostridia Eubacteriales Isachenkonia alkalipeptolytica WP 160722349.1 315 aa
Bacillati Bacillota Clostridia Peptostreptococcales Tindallia californiensis WP 093313988.1 315 aa
Bacillati Bacillota Clostridia Eubacteriales Anoxynatronum buryatiense WP 283408780.1 315 aa
Bacillati Bacillota Clostridia Peptostreptococcales Serpentinicella sp. WP 310478809.1 315 aa
Bacillati Bacillota Clostridia Peptostreptococcales Serpentinicella alkaliphila WP 132848738.1 315 aa
Bacillati Bacillota Clostridia Peptostreptococcales Anaerovirgula multivorans WP 089283113.1 315 aa
Bacillati Bacillota Clostridia Eubacteriales Clostridiaceae bacterium MCC5911230.1 315 aa

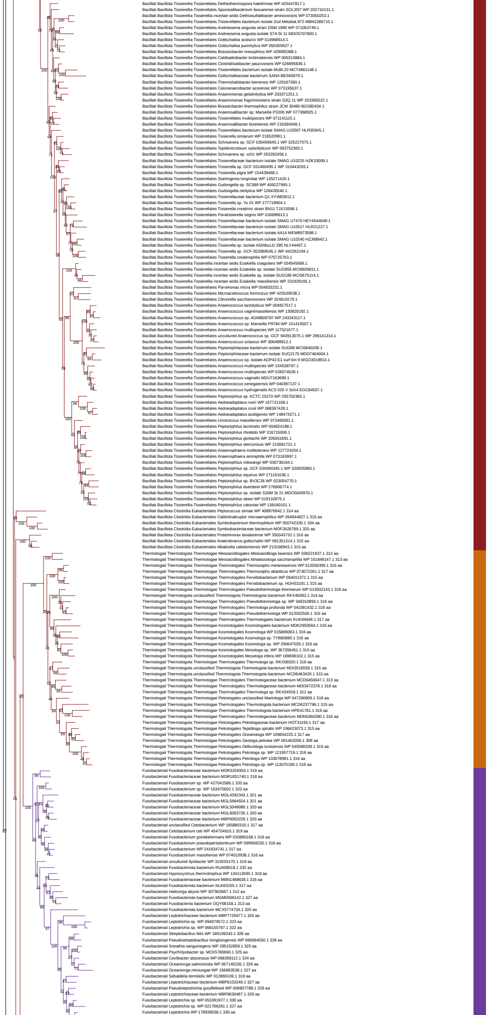
Bacillati Bacillota Tissierellia Tissierellales Dethiothermospora halolimnae WP 425447817.1
Bacillati Bacillota Tissierellia Tissierellales Sporosalibacterium faouarense strain SOL3f37 WP 202710131.1
Bacillati Bacillota Tissierellia Tissierellia incertae sedis Dethiosulfatibacter aminovorans WP 073050253.1
Bacillati Bacillota Tissierellia Tissierellales Tissierellales bacterium isolate Zod Metabat.972 MBN2286710.1
Bacillati Bacillota Tissierellia Tissierellales Andreesenia angusta strain DSM 1989 WP 071063749.1
Bacillati Bacillota Tissierellia Tissierellales Andreesenia angusta isolate S7A St 11 MDO5707800.1
Bacillati Bacillota Tissierellia Tissierellales Gottschalkia acidurici WP 014968514.1
Bacillati Bacillota Tissierellia Tissierellales Gottschalkia purinilytica WP 050355927.1
Bacillati Bacillota Tissierellia Tissierellales Brassicibacter mesophilus WP 429085388.1
Bacillati Bacillota Tissierellia Tissierellales Caldisalinibacter kiritimatiensis WP 006313884.1
Bacillati Bacillota Tissierellia Tissierellales Clostridiisalibacter paucivorans WP 026895836.1
Bacillati Bacillota Tissierellia Tissierellales Tissierellales bacterium isolate McBI.20 MCT4661148.1
Bacillati Bacillota Tissierellia Tissierellales Gottschalkiaceae bacterium SANA BES65979.1
Bacillati Bacillota Tissierellia Tissierellales Thermohalobacter berrensis WP 120167360.1
Bacillati Bacillota Tissierellia Tissierellales Caloranaerobacter azorensis WP 073195637.1
Bacillati Bacillota Tissierellia Tissierellales Anaeromonas gelatinilytica WP 203371251.1
Bacillati Bacillota Tissierellia Tissierellales Anaeromonas frigoriresistens strain D2Q 11 WP 203365522.1
Bacillati Bacillota Tissierellia Tissierellales Brassicibacter thermophilus strain JCM 30480 BGS80434.1
Bacillati Bacillota Tissierellia Tissierellales Anaerosalibacter sp. Marseille P3206 WP 077368925.1
Bacillati Bacillota Tissierellia Tissierellales Tissierellales multispecies WP 071141115.1
Bacillati Bacillota Tissierellia Tissierellales Anaerosalibacter bizertensis WP 216384048.1
Bacillati Bacillota Tissierellia Tissierellales Tissierellales bacterium isolate SMAG U10507 HLR35945.1
Bacillati Bacillota Tissierellia Tissierellales Tissierella simiarum WP 216520991.1
Bacillati Bacillota Tissierellia Tissierellales Schnuerera sp. GCF 035459645.1 WP 325227075.1
Bacillati Bacillota Tissierellia Tissierellales Tepidimicrobium xylanilyticum WP 093752363.1
Bacillati Bacillota Tissierellia Tissierellales Schnuerera sp. xch1 WP 263282456.1
Bacillati Bacillota Tissierellia Tissierellales Tissierellaceae bacterium isolate SMAG U10225 HZK33009.1
Bacillati Bacillota Tissierellia Tissierellales Tissierella sp. GCF 031460495.1 WP 310443293.1
Bacillati Bacillota Tissierellia Tissierellales Tissierella pigra WP 154439468.1
Bacillati Bacillota Tissierellia Tissierellales Soehngenia longivitae WP 135271420.1
Bacillati Bacillota Tissierellia Tissierellales Gudongella sp. SC589 WP 409227995.1
Bacillati Bacillota Tissierellia Tissierellales Gudongella oleilytica WP 128426540.1
Bacillati Bacillota Tissierellia Tissierellales Tissierellaceae bacterium Q1 XYW83912.1
Bacillati Bacillota Tissierellia Tissierellales Tissierella sp. Yu 01 WP 277718904.1
Bacillati Bacillota Tissierellia Tissierellales Tissierella creatinini strain BN11 TJX15596.1
Bacillati Bacillota Tissierellia Tissierellales Paratissierella segnis WP 316699613.1
Bacillati Bacillota Tissierellia Tissierellales Tissierellaceae bacterium isolate SMAG U7470 HEY4544049.1
Bacillati Bacillota Tissierellia Tissierellales Tissierellaceae bacterium isolate SMAG U10517 HLR21227.1
Bacillati Bacillota Tissierellia Tissierellales Tissierellaceae bacterium isolate AA14 MEW8973588.1
Bacillati Bacillota Tissierellia Tissierellales Tissierellaceae bacterium isolate SMAG U10240 HZJ98942.1
Bacillati Bacillota Tissierellia Tissierellales Tissierella sp. isolate AS09scLD 285 NLY44467.1
Bacillati Bacillota Tissierellia Tissierellales Tissierella sp. GCF 052089545.1 WP 442262194.1
Bacillati Bacillota Tissierellia Tissierellales Tissierella creatinophila WP 075725763.1
Bacillati Bacillota Tissierellia Tissierellia incertae sedis Ezakiella coagulans WP 034545668.1
Bacillati Bacillota Tissierellia Tissierellia incertae sedis Ezakiella sp. isolate SUG955 MCI6609811.1
Bacillati Bacillota Tissierellia Tissierellia incertae sedis Ezakiella sp. isolate SUG186 MCI5675114.1
Bacillati Bacillota Tissierellia Tissierellia incertae sedis Ezakiella massiliensis WP 231929165.1
Bacillati Bacillota Tissierellia Tissierellales Parvimonas micra WP 004833231.1
Bacillati Bacillota Tissierellia Tissierellales Microaceticoccus formicicus WP 425539538.1
Bacillati Bacillota Tissierellia Tissierellales Citroniella saccharovorans WP 324619170.1
Bacillati Bacillota Tissierellia Tissierellales Anaerococcus lactolyticus WP 004827517.1
Bacillati Bacillota Tissierellia Tissierellales Anaerococcus vaginimassiliensis WP 130820192.1
Bacillati Bacillota Tissierellia Tissierellales Anaerococcus sp. AGMB09787 WP 243343117.1
Bacillati Bacillota Tissierellia Tissierellales Anaerococcus sp. Marseille P9784 WP 151410507.1
Bacillati Bacillota Tissierellia Tissierellales Anaerococcus multispecies WP 117521677.1
Bacillati Bacillota Tissierellia Tissierellales uncultured Anaerococcus sp. GCF 943913075.1 WP 296141314.1
Bacillati Bacillota Tissierellia Tissierellales Anaerococcus octavius WP 306489912.1
Bacillati Bacillota Tissierellia Tissierellales Peptoniphilaceae bacterium isolate SUG89 MCI5840208.1
Bacillati Bacillota Tissierellia Tissierellales Peptoniphilaceae bacterium isolate SUG2175 MDD7464004.1
Bacillati Bacillota Tissierellia Tissierellales Anaerococcus sp. isolate AOP43 E1 surf bin 9 MGO3018910.1
Bacillati Bacillota Tissierellia Tissierellales Anaerococcus multispecies WP 154538797.1
Bacillati Bacillota Tissierellia Tissierellales Anaerococcus multispecies WP 026074028.1
Bacillati Bacillota Tissierellia Tissierellales Anaerococcus vaginalis MDU7163699.1
Bacillati Bacillota Tissierellia Tissierellales Anaerococcus senegalensis WP 040397137.1
Bacillati Bacillota Tissierellia Tissierellales Anaerococcus hydrogenalis ACS 025 V Sch4 EGC84507.1
Bacillati Bacillota Tissierellia Tissierellales Peptoniphilus sp. KCTC 25270 WP 255702365.1
Bacillati Bacillota Tissierellia Tissierellales Aedoeadaptatus ivorii WP 197721108.1
Bacillati Bacillota Tissierellia Tissierellales Aedoeadaptatus coxii WP 068367426.1
Bacillati Bacillota Tissierellia Tissierellales Aedoeadaptatus acetigenes WP 148474271.1
Bacillati Bacillota Tissierellia Tissierellales Urinicoccus massiliensis WP 072469381.1
Bacillati Bacillota Tissierellia Tissierellales Peptoniphilus lacrimalis WP 004824188.1
Bacillati Bacillota Tissierellia Tissierellales Peptoniphilus rhinitidis WP 316715006.1
Bacillati Bacillota Tissierellia Tissierellales Peptoniphilus gorbachii WP 205051691.1
Bacillati Bacillota Tissierellia Tissierellales Peptoniphilus stercorisuis WP 210061721.1
Bacillati Bacillota Tissierellia Tissierellales Anaerosphaera multitolerans WP 127724204.1
Bacillati Bacillota Tissierellia Tissierellales Anaerosphaera aminiphila WP 073183997.1
Bacillati Bacillota Tissierellia Tissierellales Peptoniphilus mikwangii WP 036730164.1
Bacillati Bacillota Tissierellia Tissierellales Peptoniphilus sp. GCF 034090345.1 WP 320635960.1
Bacillati Bacillota Tissierellia Tissierellales Peptoniphilus equinus WP 271191036.1
Bacillati Bacillota Tissierellia Tissierellales Peptoniphilus sp. BV3C26 WP 023054770.1
Bacillati Bacillota Tissierellia Tissierellales Peptoniphilus duerdenii WP 276906774.1
Bacillati Bacillota Tissierellia Tissierellales Peptoniphilus sp. isolate S30M St 21 MDO5040970.1
Bacillati Bacillota Tissierellia Tissierellales Peptoniphilus obesi WP 019132875.1
Bacillati Bacillota Tissierellia Tissierellales Peptoniphilus catoniae WP 138160101.1
Bacillati Bacillota Clostridia Eubacteriales Peptococcus simiae WP 408976942.1 314 aa
Bacillati Bacillota Clostridia Eubacteriales Caldinitratiruptor microaerophilus WP 264844827.1 315 aa
Bacillati Bacillota Clostridia Eubacteriales Symbiobacterium thermophilum WP 050742330.1 334 aa
Bacillati Bacillota Clostridia Eubacteriales Symbiobacteriaceae bacterium MDF2626789.1 325 aa
Bacillati Bacillota Clostridia Eubacteriales Proteinivorax tanatarense WP 350343732.1 316 aa
Bacillati Bacillota Clostridia Eubacteriales Anaerobranca gottschalkii WP 091351314.1 315 aa
Bacillati Bacillota Clostridia Eubacteriales Alkalicella caledoniensis WP 213168943.1 315 aa
Thermotogati Thermotogota Thermotogae Mesoaciditogales Mesoaciditoga lauensis WP 036221937.1 313 aa
Thermotogati Thermotogota Thermotogae Mesoaciditogales Athalassotoga saccharophila WP 161848147.1 313 aa
Thermotogati Thermotogota Thermotogae Thermotogales Thermosipho melanesiensis WP 012056399.1 316 aa
Thermotogati Thermotogota Thermotogae Thermotogales Thermosipho atlanticus WP 073072261.1 317 aa
Thermotogati Thermotogota Thermotogae Thermotogales Fervidobacterium WP 064011371.1 315 aa
Thermotogati Thermotogota Thermotogae Thermotogales Fervidobacterium sp. HOH53191.1 315 aa
Thermotogati Thermotogota Thermotogae Thermotogales Pseudothermotoga thermarum WP 013932143.1 316 aa
Thermotogati Thermotogota unclassified Thermotogota Thermotogota bacterium RKX40093.1 314 aa
Thermotogati Thermotogota Thermotogae Thermotogales Pseudothermotoga sp. WP 346310893.1 316 aa
Thermotogati Thermotogota Thermotogae Thermotogales Thermotoga profunda WP 041081432.1 316 aa
Thermotogati Thermotogota Thermotogae Thermotogales Pseudothermotoga WP 012002500.1 316 aa
Thermotogati Thermotogota Thermotogae Thermotogales Thermotogales bacterium KUK94649.1 317 aa
Thermotogati Thermotogota Thermotogae Kosmotogales Kosmotogales bacterium MDK2950564.1 316 aa
Thermotogati Thermotogota Thermotogae Kosmotogales Kosmotoga WP 015869063.1 316 aa
Thermotogati Thermotogota Thermotogae Kosmotogales Kosmotoga sp. TYB80860.1 316 aa
Thermotogati Thermotogota Thermotogae Kosmotogales Kosmotoga sp. WP 290647035.1 316 aa
Thermotogati Thermotogota Thermotogae Kosmotogales Mesotoga sp. WP 367356451.1 315 aa
Thermotogati Thermotogota Thermotogae Kosmotogales Mesotoga infera WP 169698102.1 315 aa
Thermotogati Thermotogota Thermotogae Thermotogales Thermotoga sp. RKX56020.1 316 aa
Thermotogati Thermotogota unclassified Thermotogota Thermotogota bacterium MDI3516559.1 315 aa
Thermotogati Thermotogota unclassified Thermotogota Thermotogota bacterium MCD6463420.1 315 aa
Thermotogati Thermotogota Thermotogae Thermotogales Thermotogaceae bacterium MCD6450047.1 313 aa
Thermotogati Thermotogota Thermotogae Thermotogales Thermotogaceae bacterium MDI3472378.1 316 aa
Thermotogati Thermotogota Thermotogae Thermotogales Thermotoga sp. RKX54918.1 312 aa
Thermotogati Thermotogota Thermotogae Petrotogales unclassified Marinitoga WP 047266909.1 318 aa
Thermotogati Thermotogota Thermotogae Thermotogales Thermotogota bacterium MCD6237796.1 315 aa
Thermotogati Thermotogota Thermotogae Thermotogales Thermotogota bacterium HPE41781.1 316 aa
Thermotogati Thermotogota Thermotogae Thermotogales Thermotogaceae bacterium MDN5360280.1 316 aa
Thermotogati Thermotogota Thermotogae Petrotogales Petrotogaceae bacterium HOT31193.1 317 aa
Thermotogati Thermotogota Thermotogae Petrotogales Tepiditoga spiralis WP 198423073.1 315 aa
Thermotogati Thermotogota Thermotogae Petrotogales Oceanotoga WP 109604225.1 317 aa
Thermotogati Thermotogota Thermotogae Petrotogales Geotoga petraea WP 091403356.1 309 aa
Thermotogati Thermotogota Thermotogae Petrotogales Defluviitoga tunisiensis WP 045088338.1 316 aa
Thermotogati Thermotogota Thermotogae Petrotogales Petrotoga sp. WP 121957719.1 316 aa
Thermotogati Thermotogota Thermotogae Petrotogales Petrotoga WP 103078991.1 316 aa
Thermotogati Thermotogota Thermotogae Petrotogales Petrotoga sp. WP 113075190.1 316 aa
Fusobacteriati Fusobacteriaceae bacterium MDR3259353.1 319 aa
Fusobacteriati Fusobacteriaceae bacterium MDR1831740.1 318 aa
Fusobacteriati Fusobacterium sp. WP 427042586.1 320 aa
Fusobacteriati Fusobacterium sp. WP 163470002.1 323 aa
Fusobacteriati Fusobacteriaceae bacterium MGL4392343.1 321 aa
Fusobacteriati Fusobacteriaceae bacterium MGL5964554.1 321 aa
Fusobacteriati Fusobacteriaceae bacterium MGL5049080.1 320 aa
Fusobacteriati Fusobacteriaceae bacterium MGL6063726.1 320 aa
Fusobacteriati Fusobacteriaceae bacterium MBP6063229.1 320 aa
Fusobacteriati unclassified Cetobacterium WP 185880310.1 317 aa
Fusobacteriati Cetobacterium ceti WP 454754910.1 319 aa
Fusobacteriati Fusobacterium gonidiaformans WP 010680168.1 316 aa
Fusobacteriati Fusobacterium pseudoperiodonticum WP 099958230.1 316 aa
Fusobacteriati Fusobacterium WP 241634741.1 317 aa
Fusobacteriati Fusobacterium massiliense WP 074015938.1 316 aa
Fusobacteriati uncultured Ilyobacter WP 319203170.1 319 aa
Fusobacteriati Fusobacteriota bacterium RUA08518.1 332 aa
Fusobacteriati Hypnocyclicus thermotrophus WP 134113595.1 318 aa
Fusobacteriati Fusobacteriaceae bacterium MBN1468638.1 316 aa
Fusobacteriati Fusobacteriota bacterium NLK63155.1 317 aa
Fusobacteriati Haliovirga abyssi WP 307903667.1 312 aa
Fusobacteriati Fusobacteriota bacterium MGM0509142.1 327 aa
Fusobacteriati Fusobacteriia bacterium OQY08158.1 313 aa
Fusobacteriati Fusobacteriota bacterium MCX5774734.1 320 aa
Fusobacteriati Leptotrichiaceae bacterium MBP7725677.1 320 aa
Fusobacteriati Leptotrichia sp. WP 094079572.1 323 aa
Fusobacteriati Leptotrichia sp. WP 068155787.1 322 aa
Fusobacteriati Streptobacillus felis WP 180136343.1 328 aa
Fusobacteriati Pseudostreptobacillus hongkongensis WP 066894050.1 328 aa
Fusobacteriati Sneathia sanguinegens WP 285152859.1 325 aa
Fusobacteriati Psychrilyobacter sp. MCK5780660.1 325 aa
Fusobacteriati Cavibacter abscessus WP 068268112.1 324 aa
Fusobacteriati Oceanivirga salmonicida WP 067140226.1 326 aa
Fusobacteriati Oceanivirga miroungae WP 156683538.1 327 aa
Fusobacteriati Sebaldella termitidis WP 012860109.1 318 aa
Fusobacteriati Leptotrichiaceae bacterium MBP6103246.1 327 aa
Fusobacteriati Pseudoleptotrichia goodfellowii WP 006807788.1 329 aa
Fusobacteriati Leptotrichiaceae bacterium MBP9630487.1 329 aa
Fusobacteriati Leptotrichia sp. WP 053391977.1 330 aa
Fusobacteriati Leptotrichia sp. WP 021766261.1 327 aa
Fusobacteriati Leptotrichia WP 178938558.1 330 aa

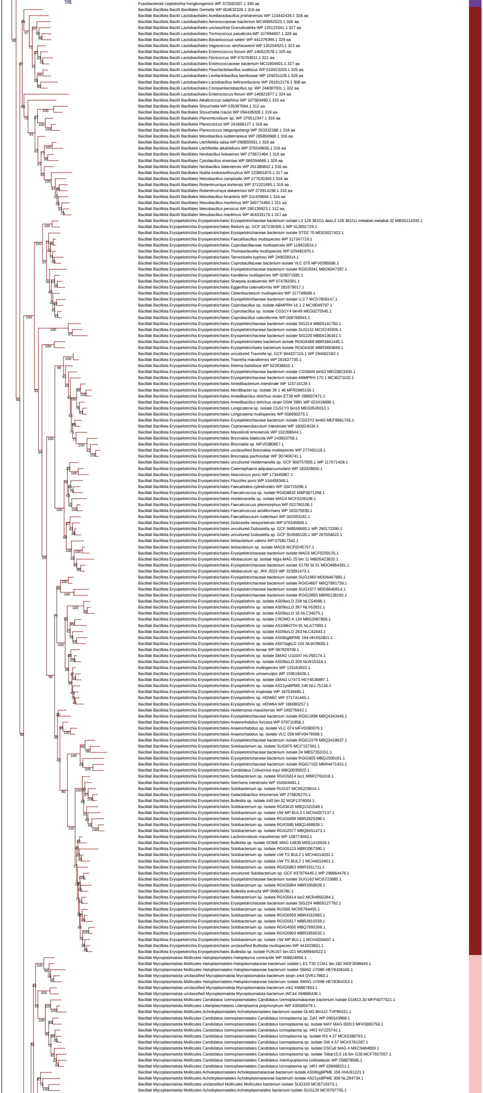
Fusobacteriati Leptotrichia hongkongensis WP 372582307.1 330 aa
Bacillati Bacillota Bacilli Bacillales Gemella WP 004632328.1 316 aa
Bacillati Bacillota Bacilli Lactobacillales Acetilactobacillus jinshanensis WP 133442439.1 328 aa
Bacillati Bacillota Bacilli Lactobacillales Aerococcaceae bacterium MCW6652523.1 326 aa
Bacillati Bacillota Bacilli Lactobacillales unclassified Granulicatella WP 135123341.1 327 aa
Bacillati Bacillota Bacilli Lactobacillales Trichococcus paludicola WP 107994907.1 326 aa
Bacillati Bacillota Bacilli Lactobacillales Bavariicoccus seileri WP 441379399.1 329 aa
Bacillati Bacillota Bacilli Lactobacillales Vagococcus xieshaowenii WP 135254925.1 323 aa
Bacillati Bacillota Bacilli Lactobacillales Enterococcus florum WP 146622578.1 325 aa
Bacillati Bacillota Bacilli Lactobacillales Floricoccus WP 070793011.1 321 aa
Bacillati Bacillota Bacilli Lactobacillales Enterococcaceae bacterium MCI1904601.1 327 aa
Bacillati Bacillota Bacilli Lactobacillales Paucilactobacillus suebicus WP 010623203.1 325 aa
Bacillati Bacillota Bacilli Lactobacillales Levilactobacillus bambusae WP 109251128.1 326 aa
Bacillati Bacillota Bacilli Lactobacillales Lactobacillus kefiranofaciens WP 261912176.1 308 aa
Bacillati Bacillota Bacilli Lactobacillales Companilactobacillus sp. WP 244087001.1 322 aa
Bacillati Bacillota Bacilli Lactobacillales Enterococcus florum WP 146621977.1 324 aa
Bacillati Bacillota Bacilli Bacillales Alkalicoccus saliphilus WP 107583490.1 315 aa
Bacillati Bacillota Bacilli Bacillales Shouchella WP 035397664.1 312 aa
Bacillati Bacillota Bacilli Bacillales Shouchella clausii WP 094426008.1 319 aa
Bacillati Bacillota Bacilli Bacillales Planomicrobium sp. WP 370512347.1 316 aa
Bacillati Bacillota Bacilli Bacillales Planococcus WP 241666127.1 316 aa
Bacillati Bacillota Bacilli Bacillales Planococcus beigongshangi WP 203332288.1 316 aa
Bacillati Bacillota Bacilli Bacillales Mesobacillus subterraneus WP 285856988.1 316 aa
Bacillati Bacillota Bacilli Bacillales Litchfieldia salsa WP 090855931.1 316 aa
Bacillati Bacillota Bacilli Bacillales Litchfieldia alkalitelluris WP 078549656.1 316 aa
Bacillati Bacillota Bacilli Bacillales Neobacillus kokaensis WP 275671464.1 316 aa
Bacillati Bacillota Bacilli Bacillales Cytobacillus eiseniae WP 066394669.1 326 aa
Bacillati Bacillota Bacilli Bacillales Neobacillus bataviensis WP 261380642.1 316 aa
Bacillati Bacillota Bacilli Bacillales Niallia endozanthoxylica WP 223881875.1 317 aa
Bacillati Bacillota Bacilli Bacillales Mesobacillus campisalis WP 277620300.1 316 aa
Bacillati Bacillota Bacilli Bacillales Robertmurraya korlensis WP 371321695.1 316 aa
Bacillati Bacillota Bacilli Bacillales Robertmurraya dakarensis WP 272914198.1 316 aa
Bacillati Bacillota Bacilli Bacillales Mesobacillus foraminis WP 311439604.1 316 aa
Bacillati Bacillota Bacilli Bacillales Mesobacillus maritimus WP 349774460.1 311 aa
Bacillati Bacillota Bacilli Bacillales Mesobacillus persicus WP 280139923.1 312 aa
Bacillati Bacillota Bacilli Bacillales Mesobacillus maritimus WP 404333176.1 317 aa
Bacillati Bacillota Erysipelotrichia Erysipelotrichales Erysipelotrichaceae bacterium isolate L3 128 361G1 dasL3 128 361G1 metabat.metabat.32 MBS5114335.1
Bacillati Bacillota Erysipelotrichia Erysipelotrichales Beduini sp. GCF 047239305.1 WP 412891729.1
Bacillati Bacillota Erysipelotrichia Erysipelotrichales Erysipelotrichaceae bacterium isolate STD2 70 MDD3027422.1
Bacillati Bacillota Erysipelotrichia Erysipelotrichales Faecalibacillus multispecies WP 117347719.1
Bacillati Bacillota Erysipelotrichia Erysipelotrichales Coprobacillaceae multispecies WP 118432624.1
Bacillati Bacillota Erysipelotrichia Erysipelotrichales Thomasclavelia multispecies WP 029481970.1
Bacillati Bacillota Erysipelotrichia Erysipelotrichales Tannockella kyphosi WP 249029314.1
Bacillati Bacillota Erysipelotrichia Erysipelotrichales Coprobacillaceae bacterium isolate VLC 075 MFV0395568.1
Bacillati Bacillota Erysipelotrichia Erysipelotrichales Erysipelotrichaceae bacterium isolate RGIG9341 MBO6047387.1
Bacillati Bacillota Erysipelotrichia Erysipelotrichales Kandleria multispecies WP 029071585.1
Bacillati Bacillota Erysipelotrichia Erysipelotrichales Sharpea azabuensis WP 074782091.1
Bacillati Bacillota Erysipelotrichia Erysipelotrichales Eggerthia catenaformis WP 281679017.1
Bacillati Bacillota Erysipelotrichia Erysipelotrichales Catenibacterium multispecies WP 217748089.1
Bacillati Bacillota Erysipelotrichia Erysipelotrichales Erysipelotrichaceae bacterium isolate U.2 7 MCD7808147.1
Bacillati Bacillota Erysipelotrichia Erysipelotrichales Coprobacillus sp. isolate ABMPRH 16 1 2 MCI9049797.1
Bacillati Bacillota Erysipelotrichia Erysipelotrichales Coprobacillus sp. isolate CGS1Y4 bin49 MEG0275545.1
Bacillati Bacillota Erysipelotrichia Erysipelotrichales Coprobacillus cateniformis WP 008788941.1
Bacillati Bacillota Erysipelotrichia Erysipelotrichales Erysipelotrichaceae bacterium isolate SIG214 MBE6141750.1
Bacillati Bacillota Erysipelotrichia Erysipelotrichales Erysipelotrichaceae bacterium isolate SUG151 MCI5745926.1
Bacillati Bacillota Erysipelotrichia Erysipelotrichales Erysipelotrichaceae bacterium isolate SIG220 MBE6136341.1
Bacillati Bacillota Erysipelotrichia Erysipelotrichales Erysipelotrichales bacterium isolate RGIG6468 MBR3841445.1
Bacillati Bacillota Erysipelotrichia Erysipelotrichales Erysipelotrichales bacterium isolate RGIG6436 MBR3693669.1
Bacillati Bacillota Erysipelotrichia Erysipelotrichales uncultured Traorella sp. GCF 944327115.1 WP 294562182.1
Bacillati Bacillota Erysipelotrichia Erysipelotrichales Traorella massiliensis WP 281627735.1
Bacillati Bacillota Erysipelotrichia Erysipelotrichales Dielma fastidiosa WP 022938810.1
Bacillati Bacillota Erysipelotrichia Erysipelotrichales Erysipelotrichaceae bacterium isolate CGS6M4 bin53 MEG0823435.1
Bacillati Bacillota Erysipelotrichia Erysipelotrichales Erysipelotrichaceae bacterium isolate ABMPRH 170 1 MCI8271102.1
Bacillati Bacillota Erysipelotrichia Erysipelotrichales Amedibacterium intestinale WP 115716139.1
Bacillati Bacillota Erysipelotrichia Erysipelotrichales Merdibacter sp. isolate 39 1 46 MFR2885156.1
Bacillati Bacillota Erysipelotrichia Erysipelotrichales Amedibacillus dolichus strain ET39 WP 289607471.1
Bacillati Bacillota Erysipelotrichia Erysipelotrichales Amedibacillus dolichus strain DSM 3991 WP 022419698.1
Bacillati Bacillota Erysipelotrichia Erysipelotrichales Longicatena sp. isolate CGS1Y3 bin16 MEG0526013.1
Bacillati Bacillota Erysipelotrichia Erysipelotrichales Longicatena multispecies WP 008690275.1
Bacillati Bacillota Erysipelotrichia Erysipelotrichales Erysipelotrichaceae bacterium isolate CGS3Y2 bin60 MEF9961745.1
Bacillati Bacillota Erysipelotrichia Erysipelotrichales Copranaerobaculum intestinale WP 160624034.1
Bacillati Bacillota Erysipelotrichia Erysipelotrichales Massilicoli timonensis WP 102268644.1
Bacillati Bacillota Erysipelotrichia Erysipelotrichales Breznakia blatticola WP 243833758.1
Bacillati Bacillota Erysipelotrichia Erysipelotrichales Breznakia sp. MFV0380857.1
Bacillati Bacillota Erysipelotrichia Erysipelotrichales unclassified Breznakia multispecies WP 277491118.1
Bacillati Bacillota Erysipelotrichia Erysipelotrichales Breznakia pachnodae WP 307406741.1
Bacillati Bacillota Erysipelotrichia Erysipelotrichales uncultured Holdemanella sp. GCF 900757855.1 WP 117571428.1
Bacillati Bacillota Erysipelotrichia Erysipelotrichales Catenisphaera adipataccumulans WP 183328655.1
Bacillati Bacillota Erysipelotrichia Erysipelotrichales Absicoccus porci WP 173445867.1
Bacillati Bacillota Erysipelotrichia Erysipelotrichales Floccifex porci WP 154459348.1
Bacillati Bacillota Erysipelotrichia Erysipelotrichales Faecalitalea cylindroides WP 204715396.1
Bacillati Bacillota Erysipelotrichia Erysipelotrichales Faecalicoccus sp. isolate RGIG8832 MBP3871298.1
Bacillati Bacillota Erysipelotrichia Erysipelotrichales Holdemanella sp. isolate MAG4 MCF0106108.1
Bacillati Bacillota Erysipelotrichia Erysipelotrichales Faecalicoccus pleomorphus WP 022790106.1
Bacillati Bacillota Erysipelotrichia Erysipelotrichales Faecalicoccus acidiformans WP 183375930.1
Bacillati Bacillota Erysipelotrichia Erysipelotrichales Faecalibaculum rodentium WP 302593181.1
Bacillati Bacillota Erysipelotrichia Erysipelotrichales Dubosiella newyorkensis WP 076340608.1
Bacillati Bacillota Erysipelotrichia Erysipelotrichales uncultured Dubosiella sp. GCF 948566665.1 WP 290172390.1
Bacillati Bacillota Erysipelotrichia Erysipelotrichales uncultured Dubosiella sp. GCF 910585105.1 WP 297054622.1
Bacillati Bacillota Erysipelotrichia Erysipelotrichales Ileibacterium valens WP 075817342.1
Bacillati Bacillota Erysipelotrichia Erysipelotrichales Ileibacterium sp. isolate MAG6 MCF0245757.1
Bacillati Bacillota Erysipelotrichia Erysipelotrichales Erysipelotrichaceae bacterium isolate MAG5 MCF0259125.1
Bacillati Bacillota Erysipelotrichia Erysipelotrichales Allobaculum sp. isolate Mgla MAG 25 bin 11 MBD5423820.1
Bacillati Bacillota Erysipelotrichia Erysipelotrichales Erysipelotrichaceae bacterium isolate S17M St 31 MDO4664391.1
Bacillati Bacillota Erysipelotrichia Erysipelotrichales Allobaculum sp. JKK 2023 WP 323091473.1
Bacillati Bacillota Erysipelotrichia Erysipelotrichales Erysipelotrichaceae bacterium isolate SUG1660 MDD6467685.1
Bacillati Bacillota Erysipelotrichia Erysipelotrichales Erysipelotrichaceae bacterium isolate RGIG4607 MBQ7891759.1
Bacillati Bacillota Erysipelotrichia Erysipelotrichales Erysipelotrichaceae bacterium isolate SUG1577 MDD6640814.1
Bacillati Bacillota Erysipelotrichia Erysipelotrichales Erysipelotrichaceae bacterium isolate RGIG2863 MBR0138192.1
Bacillati Bacillota Erysipelotrichia Erysipelotrichales Erysipelothrix sp. isolate AS09scLD 238 NLC54996.1
Bacillati Bacillota Erysipelotrichia Erysipelotrichales Erysipelothrix sp. isolate AS09scLD 397 NLY62831.1
Bacillati Bacillota Erysipelotrichia Erysipelotrichales Erysipelothrix sp. isolate AS09scLD 16 NLC34075.1
Bacillati Bacillota Erysipelotrichia Erysipelotrichales Erysipelothrix sp. isolate CROMO A 134 MBS3987806.1
Bacillati Bacillota Erysipelotrichia Erysipelotrichales Erysipelothrix sp. isolate AS10tlH2TH 91 NLA77993.1
Bacillati Bacillota Erysipelotrichia Erysipelotrichales Erysipelothrix sp. isolate AS09scLD 263 NLC42443.1
Bacillati Bacillota Erysipelotrichia Erysipelotrichales Erysipelothrix sp. isolate AS08sgBPME 194 HHX52801.1
Bacillati Bacillota Erysipelotrichia Erysipelotrichales Erysipelothrix sp. isolate AS07pgkLD 216 NLW29650.1
Bacillati Bacillota Erysipelotrichia Erysipelotrichales Erysipelothrix larvae WP 067629708.1
Bacillati Bacillota Erysipelotrichia Erysipelotrichales Erysipelothrix sp. isolate SMAG U10347 HLV50174.1
Bacillati Bacillota Erysipelotrichia Erysipelotrichales Erysipelothrix sp. isolate AS09scLD 209 NLW15324.1
Bacillati Bacillota Erysipelotrichia Erysipelotrichales Erysipelothrix multispecies WP 125163922.1
Bacillati Bacillota Erysipelotrichia Erysipelotrichales Erysipelothrix urinaevulpis WP 159518426.1
Bacillati Bacillota Erysipelotrichia Erysipelotrichales Erysipelothrix sp. isolate SMAG U7473 HEY4536897.1
Bacillati Bacillota Erysipelotrichia Erysipelotrichales Erysipelothrix sp. isolate AS21ysBPME 246 NLL75136.1
Bacillati Bacillota Erysipelotrichia Erysipelotrichales Erysipelothrix inopinata WP 187534485.1
Bacillati Bacillota Erysipelotrichia Erysipelotrichales Erysipelothrix sp. HDW6C WP 371741445.1
Bacillati Bacillota Erysipelotrichia Erysipelotrichales Erysipelothrix sp. HDW6A WP 166080257.1
Bacillati Bacillota Erysipelotrichia Erysipelotrichales Holdemania massiliensis WP 240276642.1
Bacillati Bacillota Erysipelotrichia Erysipelotrichales Erysipelotrichaceae bacterium isolate RGIG1896 MBQ4343445.1
Bacillati Bacillota Erysipelotrichia Erysipelotrichales Anaerorhabdus furcosa WP 078711858.1
Bacillati Bacillota Erysipelotrichia Erysipelotrichales Anaerorhabdus sp. isolate VLC 074 MFV0380079.1
Bacillati Bacillota Erysipelotrichia Erysipelotrichales Anaerorhabdus sp. isolate VLC 039 MFV0479598.1
Bacillati Bacillota Erysipelotrichia Erysipelotrichales Erysipelotrichaceae bacterium isolate RGIG2379 MBQ3418637.1
Bacillati Bacillota Erysipelotrichia Erysipelotrichales Solobacterium sp. isolate SUG975 MCI7157361.1
Bacillati Bacillota Erysipelotrichia Erysipelotrichales Erysipelotrichaceae bacterium isolate 24 MBS7353151.1
Bacillati Bacillota Erysipelotrichia Erysipelotrichales Erysipelotrichaceae bacterium isolate RGIG605 MBQ1509191.1
Bacillati Bacillota Erysipelotrichia Erysipelotrichales Erysipelotrichaceae bacterium isolate RGIG7102 MBR4471415.1
Bacillati Bacillota Erysipelotrichia Erysipelotrichales Candidatus Colivicinus equi MBQ0035922.1
Bacillati Bacillota Erysipelotrichia Erysipelotrichales Solobacterium sp. isolate RGIG5614 iso1 MBR2761018.1
Bacillati Bacillota Erysipelotrichia Erysipelotrichales Stecheria intestinalis WP 154504481.1
Bacillati Bacillota Erysipelotrichia Erysipelotrichales Solobacterium sp. isolate RUI107 MCR5229014.1
Bacillati Bacillota Erysipelotrichia Erysipelotrichales Galactobacillus timonensis WP 276826270.1
Bacillati Bacillota Erysipelotrichia Erysipelotrichales Bulleidia sp. isolate A40 bin.52 MGP1376004.1
Bacillati Bacillota Erysipelotrichia Erysipelotrichales Solobacterium sp. isolate RGIG615 MBQ1531549.1
Bacillati Bacillota Erysipelotrichia Erysipelotrichales Solobacterium sp. isolate UW MP BUL3 1 MCH4207137.1
Bacillati Bacillota Erysipelotrichia Erysipelotrichales Solobacterium sp. isolate RGIG5699 MBR2825396.1
Bacillati Bacillota Erysipelotrichia Erysipelotrichales Solobacterium sp. isolate RGIG585 MBQ1468926.1
Bacillati Bacillota Erysipelotrichia Erysipelotrichales Solobacterium sp. isolate RGIG2577 MBQ6451473.1
Bacillati Bacillota Erysipelotrichia Erysipelotrichales Lactimicrobium massiliense WP 108774563.1
Bacillati Bacillota Erysipelotrichia Erysipelotrichales Bulleidia sp. isolate DOME MAG 14839 MEE1410618.1
Bacillati Bacillota Erysipelotrichia Erysipelotrichales Solobacterium sp. isolate RGIG5123 MBR2067380.1
Bacillati Bacillota Erysipelotrichia Erysipelotrichales Solobacterium sp. isolate UW TS BUL2 1 MCH4014032.1
Bacillati Bacillota Erysipelotrichia Erysipelotrichales Solobacterium sp. isolate UW TS BUL2 1 MCH4013401.1
Bacillati Bacillota Erysipelotrichia Erysipelotrichales Solobacterium sp. isolate RGIG5863 MBR3311711.1
Bacillati Bacillota Erysipelotrichia Erysipelotrichales uncultured Solobacterium sp. GCF 937876445.1 WP 298664476.1
Bacillati Bacillota Erysipelotrichia Erysipelotrichales Erysipelotrichaceae bacterium isolate SUG162 MCI5723685.1
Bacillati Bacillota Erysipelotrichia Erysipelotrichales Solobacterium sp. isolate RGIG5894 MBR3358928.1
Bacillati Bacillota Erysipelotrichia Erysipelotrichales Bulleidia extructa WP 006626780.1
Bacillati Bacillota Erysipelotrichia Erysipelotrichales Solobacterium sp. isolate RGIG5614 iso2 MCR4950284.1
Bacillati Bacillota Erysipelotrichia Erysipelotrichales Erysipelotrichaceae bacterium isolate SIG224 MBE6127792.1
Bacillati Bacillota Erysipelotrichia Erysipelotrichales Solobacterium sp. isolate RUI365 MCR5794455.1
Bacillati Bacillota Erysipelotrichia Erysipelotrichales Solobacterium sp. isolate RGIG6959 MBR4162983.1
Bacillati Bacillota Erysipelotrichia Erysipelotrichales Solobacterium sp. isolate RGIG5617 MBR2810259.1
Bacillati Bacillota Erysipelotrichia Erysipelotrichales Solobacterium sp. isolate RGIG4500 MBQ7993266.1
Bacillati Bacillota Erysipelotrichia Erysipelotrichales Solobacterium sp. isolate RGIG5903 MBR3350032.1
Bacillati Bacillota Erysipelotrichia Erysipelotrichales Solobacterium sp. isolate UW MP BUL1 1 MCH4206407.1
Bacillati Bacillota Erysipelotrichia Erysipelotrichales unclassified Bulleidia multispecies WP 441023831.1
Bacillati Bacillota Erysipelotrichia Erysipelotrichales Bulleidia sp. isolate FUKU07 bin.021 MGM9940522.1
Bacillati Mycoplasmatota Mollicutes Haloplasmatales Haloplasma contractile WP 008824959.1
Bacillati Mycoplasmatota Mollicutes Haloplasmatales Haloplasmataceae bacterium isolate L E1 T20 COA1 bin.182 MDF2699649.1
Bacillati Mycoplasmatota Mollicutes Haloplasmatales Haloplasmataceae bacterium isolate SMAG U7080 HEY8436165.1
Bacillati Mycoplasmatota unclassified Mycoplasmatota Mycoplasmatota bacterium strain zrk4 QVK17963.1
Bacillati Mycoplasmatota Mollicutes Haloplasmatales Haloplasmataceae bacterium isolate SMAG U7098 HEY8364153.1
Bacillati Mycoplasmatota unclassified Mycoplasmatota Mycoplasmatota bacterium zrk1 XMB67833.1
Bacillati Mycoplasmatota unclassified Mycoplasmatota Mycoplasmatota bacterium WC44 XMB86436.1
Bacillati Mycoplasmatota Mollicutes Candidatus Izemoplasmatales Candidatus Izemoplasmataceae bacterium isolate D1M13 20 MFP4077521.1
Bacillati Mycoplasmatota Mollicutes Liberiplasmatales Liberiplasma polymorphum WP 435585679.1
Bacillati Mycoplasmatota Mollicutes Acholeplasmatales Acholeplasmatales bacterium isolate DLM2.Bin112 TVP96431.1
Bacillati Mycoplasmatota Mollicutes Candidatus Izemoplasmatales Candidatus Izimoplasma sp. ZiA1 WP 095543968.1
Bacillati Mycoplasmatota Mollicutes Candidatus Izemoplasmatales Candidatus Izemoplasma sp. isolate MAY MAG 00013 MFK5883750.1
Bacillati Mycoplasmatota Mollicutes Candidatus Izemoplasmatales Candidatus Izimoplasma sp. HR2 KFZ25741.1
Bacillati Mycoplasmatota Mollicutes Candidatus Izemoplasmatales Candidatus Izimoplasma sp. isolate RS 4 27 MCK5388793.1
Bacillati Mycoplasmatota Mollicutes Candidatus Izemoplasmatales Candidatus Izemoplasma sp. isolate SW 4 57 MCK5761287.1
Bacillati Mycoplasmatota Mollicutes Candidatus Izemoplasmatales Candidatus Izemoplasma sp. isolate DSCult MAG 4 MEC9484659.1
Bacillati Mycoplasmatota Mollicutes Candidatus Izemoplasmatales Candidatus Izemoplasma sp. isolate Tobar15.5 16.5m G26 MCF7927057.1
Bacillati Mycoplasmatota Mollicutes Candidatus Izemoplasmatales Candidatus Xianfuyuplasma coldseepsis WP 258878586.1
Bacillati Mycoplasmatota Mollicutes Candidatus Izemoplasmatales Candidatus Izimoplasma sp. HR1 WP 038468221.1
Bacillati Mycoplasmatota Mollicutes Acholeplasmatales Acholeplasmataceae bacterium isolate AS08sgBPME 156 HHU81221.1
Bacillati Mycoplasmatota Mollicutes Acholeplasmatales Acholeplasmataceae bacterium isolate AS21ysBPME 308 NLZ84734.1
Bacillati Mycoplasmatota Mollicutes unclassified Mollicutes Mollicutes bacterium isolate SUG320 MCI6716373.1
Bacillati Mycoplasmatota Mollicutes Acholeplasmatales Acholeplasmatales bacterium isolate SUG129 MCI5787755.1

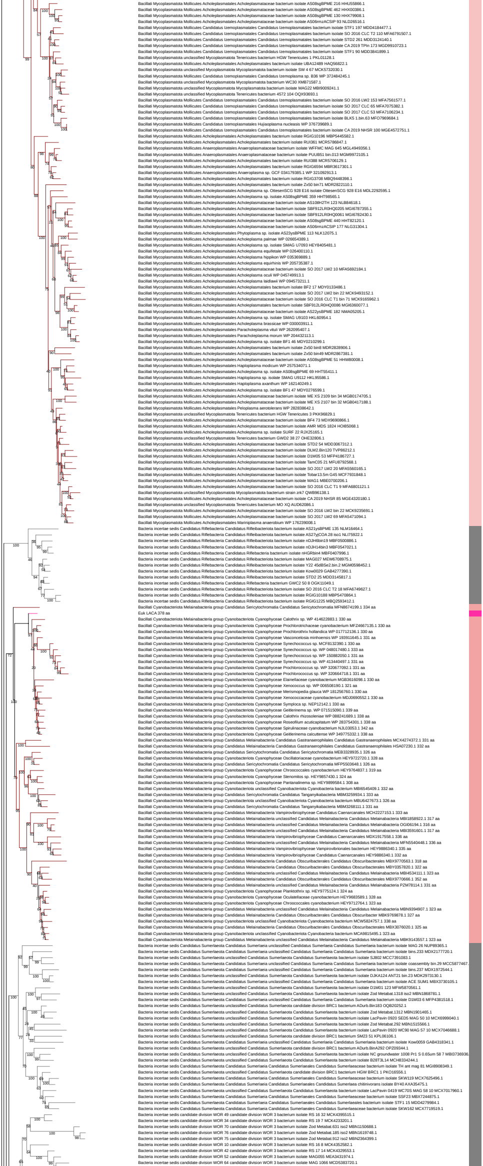
Bacillati Mycoplasmatota Mollicutes Acholeplasmatales Acholeplasmataceae bacterium isolate AS08sgBPME 216 HHU55866.1
Bacillati Mycoplasmatota Mollicutes Acholeplasmatales Acholeplasmataceae bacterium isolate AS08sgBPME 462 HHX00386.1
Bacillati Mycoplasmatota Mollicutes Acholeplasmatales Acholeplasmataceae bacterium isolate AS08sgBPME 130 HHX79908.1
Bacillati Mycoplasmatota Mollicutes Acholeplasmatales Acholeplasmataceae bacterium isolate AS06rmzACSIP 93 NLD26516.1
Bacillati Mycoplasmatota Mollicutes Candidatus Izemoplasmatales Candidatus Izemoplasmatales bacterium isolate STF1 197 MDD4184477.1
Bacillati Mycoplasmatota Mollicutes Candidatus Izemoplasmatales Candidatus Izemoplasmatales bacterium isolate SO 2016 CLC T2 110 MFA6791507.1
Bacillati Mycoplasmatota Mollicutes Candidatus Izemoplasmatales Candidatus Izemoplasmatales bacterium isolate STD2 261 MDD3124140.1
Bacillati Mycoplasmatota Mollicutes Candidatus Izemoplasmatales Candidatus Izemoplasmatales bacterium isolate CA 2019 TPIn 173 MGD9910723.1
Bacillati Mycoplasmatota Mollicutes Candidatus Izemoplasmatales Candidatus Izemoplasmatales bacterium isolate STF1 90 MDD3841899.1
Bacillati Mycoplasmatota unclassified Mycoplasmatota Tenericutes bacterium HGW Tenericutes 1 PKL01128.1
Bacillati Mycoplasmatota Mollicutes Acholeplasmatales Acholeplasmatales bacterium isolate UBA12489 HAQ56822.1
Bacillati Mycoplasmatota unclassified Mycoplasmatota Mycoplasmatota bacterium isolate SW 4 67 MCK5732030.1
Bacillati Mycoplasmatota Mollicutes Candidatus Izemoplasmatales Candidatus Izemoplasma sp. B36 WP 372484245.1
Bacillati Mycoplasmatota unclassified Mycoplasmatota Mycoplasmatota bacterium WC30 XMB71587.1
Bacillati Mycoplasmatota unclassified Mycoplasmatota Mycoplasmatota bacterium isolate MAG22 MBI9009241.1
Bacillati Mycoplasmatota unclassified Mycoplasmatota Tenericutes bacterium 4572 104 OQX93693.1
Bacillati Mycoplasmatota Mollicutes Candidatus Izemoplasmatales Candidatus Izemoplasmatales bacterium isolate SO 2016 LW2 153 MFA7561577.1
Bacillati Mycoplasmatota Mollicutes Candidatus Izemoplasmatales Candidatus Izemoplasmatales bacterium isolate SO 2017 CLC 65 MFA7075382.1
Bacillati Mycoplasmatota Mollicutes Candidatus Izemoplasmatales Candidatus Izemoplasmatales bacterium isolate SO 2017 CLC 53 MFA7106234.1
Bacillati Mycoplasmatota Mollicutes Candidatus Izemoplasmatales Candidatus Izemoplasmatales bacterium isolate BLK5 1.bin.63 MFO7969684.1
Bacillati Mycoplasmatota Mollicutes Candidatus Izemoplasmatales Hujiaoplasma nucleasis WP 376739689.1
Bacillati Mycoplasmatota Mollicutes Candidatus Izemoplasmatales Candidatus Izemoplasmatales bacterium isolate CA 2019 NHSR 100 MGE4572751.1
Bacillati Mycoplasmatota Mollicutes Acholeplasmatales Acholeplasmatales bacterium isolate RGIG10196 MBP5445582.1
Bacillati Mycoplasmatota Mollicutes Acholeplasmatales Acholeplasmatales bacterium isolate RUI361 MCR5786847.1
Bacillati Mycoplasmatota Mollicutes Anaeroplasmatales Anaeroplasmataceae bacterium isolate WFFMC MAG 645 MGL4949356.1
Bacillati Mycoplasmatota Mollicutes Anaeroplasmatales Anaeroplasmataceae bacterium isolate PUUB51 bin.013 MGM9972105.1
Bacillati Mycoplasmatota Mollicutes Acholeplasmatales Acholeplasmatales bacterium isolate RUI388 MCR5706129.1
Bacillati Mycoplasmatota Mollicutes Acholeplasmatales Acholeplasmatales bacterium isolate RGIG6594 MBR3617301.1
Bacillati Mycoplasmatota Mollicutes Anaeroplasmatales Anaeroplasma sp. GCF 034179385.1 WP 321092913.1
Bacillati Mycoplasmatota Mollicutes Acholeplasmatales Acholeplasmatales bacterium isolate RGIG3708 MBQ9448398.1
Bacillati Mycoplasmatota Mollicutes Acholeplasmatales Acholeplasmatales bacterium isolate Zx50 bin71 MDR2822110.1
Bacillati Mycoplasmatota Mollicutes Acholeplasmatales Acholeplasma sp. OttesenSCG 928 E16 isolate OttesenSCG 928 E16 MDL2292595.1
Bacillati Mycoplasmatota Mollicutes Acholeplasmatales Acholeplasma sp. isolate AS08sgBPME 359 HHT98565.1
Bacillati Mycoplasmatota Mollicutes Acholeplasmatales Acholeplasmataceae bacterium isolate AS10tlH2TH 123 NLB84618.1
Bacillati Mycoplasmatota Mollicutes Acholeplasmatales Acholeplasmataceae bacterium isolate SBF912LR0HQ0205 MGI6787355.1
Bacillati Mycoplasmatota Mollicutes Acholeplasmatales Acholeplasmataceae bacterium isolate SBF912LR0HQ0061 MGI6782430.1
Bacillati Mycoplasmatota Mollicutes Acholeplasmatales Acholeplasmataceae bacterium isolate AS08sgBPME 440 HHT82120.1
Bacillati Mycoplasmatota Mollicutes Acholeplasmatales Acholeplasmataceae bacterium isolate AS06rmzACSIP 177 NLG31304.1
Bacillati Mycoplasmatota Mollicutes Acholeplasmatales Phytoplasma sp. isolate AS23ysBPME 113 NLK12075.1
Bacillati Mycoplasmatota Mollicutes Acholeplasmatales Acholeplasma palmae WP 026654389.1
Bacillati Mycoplasmatota Mollicutes Acholeplasmatales Acholeplasma sp. isolate SMAG U7093 HEY8405481.1
Bacillati Mycoplasmatota Mollicutes Acholeplasmatales Acholeplasma equifetale WP 026400110.1
Bacillati Mycoplasmatota Mollicutes Acholeplasmatales Acholeplasma hippikon WP 035369889.1
Bacillati Mycoplasmatota Mollicutes Acholeplasmatales Acholeplasma equirhinis WP 205735387.1
Bacillati Mycoplasmatota Mollicutes Acholeplasmatales Acholeplasmataceae bacterium isolate SO 2017 LW2 10 MFA5692184.1
Bacillati Mycoplasmatota Mollicutes Acholeplasmatales Acholeplasma oculi WP 045749913.1
Bacillati Mycoplasmatota Mollicutes Acholeplasmatales Acholeplasma laidlawii WP 094573211.1
Bacillati Mycoplasmatota Mollicutes Acholeplasmatales Acholeplasmatales bacterium isolate BF2 17 MDY0133486.1
Bacillati Mycoplasmatota Mollicutes Acholeplasmatales Acholeplasmataceae bacterium isolate SO 2017 LW2 bin 22 MCK9493152.1
Bacillati Mycoplasmatota Mollicutes Acholeplasmatales Acholeplasmataceae bacterium isolate SO 2016 CLC T1 bin 71 MCK9165962.1
Bacillati Mycoplasmatota Mollicutes Acholeplasmatales Acholeplasmatales bacterium isolate SBF912LR0HQ0086 MGI6360077.1
Bacillati Mycoplasmatota Mollicutes Acholeplasmatales Acholeplasmataceae bacterium isolate AS22ysBPME 182 NMA05205.1
Bacillati Mycoplasmatota Mollicutes Acholeplasmatales Acholeplasma sp. isolate SMAG U9103 HKL60954.1
Bacillati Mycoplasmatota Mollicutes Acholeplasmatales Acholeplasma brassicae WP 030003911.1
Bacillati Mycoplasmatota Mollicutes Acholeplasmatales Paracholeplasma vituli WP 262095407.1
Bacillati Mycoplasmatota Mollicutes Acholeplasmatales Paracholeplasma morum WP 204432113.1
Bacillati Mycoplasmatota Mollicutes Acholeplasmatales Acholeplasma sp. isolate BF1 46 MDY0210299.1
Bacillati Mycoplasmatota Mollicutes Acholeplasmatales Acholeplasmatales bacterium isolate Zx50 bin8 MDR2828906.1
Bacillati Mycoplasmatota Mollicutes Acholeplasmatales Acholeplasmatales bacterium isolate Zx50 bin49 MDR2867381.1
Bacillati Mycoplasmatota Mollicutes Acholeplasmatales Acholeplasmataceae bacterium isolate AS08sgBPME 51 HHW80008.1
Bacillati Mycoplasmatota Mollicutes Acholeplasmatales Haploplasma modicum WP 257534071.1
Bacillati Mycoplasmatota Mollicutes Acholeplasmatales Acholeplasma sp. isolate AS08sgBPME 69 HHT55411.1
Bacillati Mycoplasmatota Mollicutes Acholeplasmatales Haploplasma sp. isolate SMAG U9112 HKL95586.1
Bacillati Mycoplasmatota Mollicutes Acholeplasmatales Haploplasma axanthum WP 162140249.1
Bacillati Mycoplasmatota Mollicutes Acholeplasmatales Acholeplasma sp. isolate BF1 47 MDY0276599.1
Bacillati Mycoplasmatota Mollicutes Acholeplasmatales Acholeplasmataceae bacterium isolate ME XS 2109 bin 34 MGB0174705.1
Bacillati Mycoplasmatota Mollicutes Acholeplasmatales Acholeplasmataceae bacterium isolate ME XS 2107 bin 32 MGB0417188.1
Bacillati Mycoplasmatota Mollicutes Acholeplasmatales Peloplasma aerotolerans WP 282838642.1
Bacillati Mycoplasmatota unclassified Mycoplasmatota Tenericutes bacterium HGW Tenericutes 3 PKK96829.1
Bacillati Mycoplasmatota Mollicutes Acholeplasmatales Acholeplasmataceae bacterium isolate BF4 73 MDX9690866.1
Bacillati Mycoplasmatota Mollicutes Acholeplasmatales Acholeplasmataceae bacterium isolate AMR MDS 1824 HOI85068.1
Bacillati Mycoplasmatota Mollicutes Acholeplasmatales Acholeplasma sp. isolate SURF 22 RJX25165.1
Bacillati Mycoplasmatota unclassified Mycoplasmatota Tenericutes bacterium GWD2 38 27 OHE32806.1
Bacillati Mycoplasmatota Mollicutes Acholeplasmatales Acholeplasmataceae bacterium isolate STD2 54 MDD3067312.1
Bacillati Mycoplasmatota Mollicutes Acholeplasmatales Acholeplasmataceae bacterium isolate DLM2.Bin120 TVP86212.1
Bacillati Mycoplasmatota Mollicutes Acholeplasmatales Acholeplasmataceae bacterium isolate D1M05 53 MFP4186727.1
Bacillati Mycoplasmatota Mollicutes Acholeplasmatales Acholeplasmataceae bacterium isolate TamC05 21 MFU8792568.1
Bacillati Mycoplasmatota Mollicutes Acholeplasmatales Acholeplasmataceae bacterium isolate SO 2017 LW2 20 MFA5560165.1
Bacillati Mycoplasmatota Mollicutes Acholeplasmatales Acholeplasmataceae bacterium isolate Tobar13.5m G45 MCF7931848.1
Bacillati Mycoplasmatota Mollicutes Acholeplasmatales Acholeplasmataceae bacterium isolate MAG1 MBE0700206.1
Bacillati Mycoplasmatota Mollicutes Acholeplasmatales Acholeplasmataceae bacterium isolate SO 2016 CLC T1 9 MFA6801121.1
Bacillati Mycoplasmatota unclassified Mycoplasmatota Mycoplasmatota bacterium strain zrk7 QWB96138.1
Bacillati Mycoplasmatota Mollicutes Acholeplasmatales Acholeplasmataceae bacterium isolate CA 2019 NHSR 85 MGE4320180.1
Bacillati Mycoplasmatota unclassified Mycoplasmatota Tenericutes bacterium MO XQ AUD62086.1
Bacillati Mycoplasmatota Mollicutes Acholeplasmatales Acholeplasmataceae bacterium isolate SO 2016 LW2 bin 22 MCK9235691.1
Bacillati Mycoplasmatota Mollicutes Acholeplasmatales Acholeplasmataceae bacterium isolate SO 2017 LW2 69 MFA5471094.1
Bacillati Mycoplasmatota Mollicutes Acholeplasmatales Mariniplasma anaerobium WP 176239008.1
Bacteria incertae sedis Candidatus Riflebacteria Candidatus Rifleibacteriota bacterium isolate AS21ysBPME 135 NLM16464.1
Bacteria incertae sedis Candidatus Riflebacteria Candidatus Rifleibacteriota bacterium isolate AS27yjCOA 28 iso1 NLI75922.1
Bacteria incertae sedis Candidatus Riflebacteria Candidatus Rifleibacteriota bacterium isolate nDJH6bin19 MBF0500886.1
Bacteria incertae sedis Candidatus Riflebacteria Candidatus Rifleibacteriota bacterium isolate nDJH14bin3 MBF0547021.1
Bacteria incertae sedis Candidatus Riflebacteria Candidatus Riflebacteria bacterium isolate nHGRbin4 MBF0407996.1
Bacteria incertae sedis Candidatus Riflebacteria Candidatus Rifleibacteriota bacterium isolate MAG027 MEW6708975.1
Bacteria incertae sedis Candidatus Riflebacteria Candidatus Rifleibacteriota bacterium isolate Y22 45dBSe2.bin.2 MGM0598452.1
Bacteria incertae sedis Candidatus Riflebacteria Candidatus Rifleibacteriota bacterium isolate Kow0029 GAB4277390.1
Bacteria incertae sedis Candidatus Riflebacteria Candidatus Rifleibacteriota bacterium isolate STD2 25 MDD3145817.1
Bacteria incertae sedis Candidatus Riflebacteria Candidatus Riflebacteria bacterium GWC2 50 8 OGK11049.1
Bacteria incertae sedis Candidatus Riflebacteria Candidatus Rifleibacteriota bacterium isolate SO 2016 CLC T2 18 MFA6749627.1
Bacteria incertae sedis Candidatus Riflebacteria Candidatus Rifleibacteriota bacterium isolate RGIG10188 MBP5470864.1
Bacteria incertae sedis Candidatus Riflebacteria Candidatus Rifleibacteriota bacterium isolate RGIG1225 MBQ2593412.1
Bacillati Cyanobacteriota Melainabacteria group Candidatus Sericytochromatia Candidatus Sericytochromatia MFN8674199.1 334 aa
Euk LACA 378 aa
Bacillati Cyanobacteriota Melainabacteria group Cyanobacteriota Cyanophyceae Calothrix sp. WP 414622883.1 330 aa
Bacillati Cyanobacteriota Melainabacteria group Cyanobacteriota Cyanophyceae Prochlorotrichaceae cyanobacterium MFZ4667135.1 330 aa
Bacillati Cyanobacteriota Melainabacteria group Cyanobacteriota Cyanophyceae Prochlorothrix hollandica WP 017712136.1 330 aa
Bacillati Cyanobacteriota Melainabacteria group Cyanobacteriota Cyanophyceae Vasconcelosia minhoensis WP 193911645.1 331 aa
Bacillati Cyanobacteriota Melainabacteria group Cyanobacteriota Cyanophyceae Synechococcus sp. MCF8132390.1 330 aa
Bacillati Cyanobacteriota Melainabacteria group Cyanobacteriota Cyanophyceae Synechococcus sp. WP 048017480.1 333 aa
Bacillati Cyanobacteriota Melainabacteria group Cyanobacteriota Cyanophyceae Synechococcus sp. WP 150882050.1 331 aa
Bacillati Cyanobacteriota Melainabacteria group Cyanobacteriota Cyanophyceae Synechococcus sp. WP 413440497.1 331 aa
Bacillati Cyanobacteriota Melainabacteria group Cyanobacteriota Cyanophyceae Prochlorococcus sp. WP 320677092.1 331 aa
Bacillati Cyanobacteriota Melainabacteria group Cyanobacteriota Cyanophyceae Prochlorococcus sp. WP 320664718.1 331 aa
Bacillati Cyanobacteriota Melainabacteria group Cyanobacteriota Cyanophyceae Elainellaceae cyanobacterium MGB3616098.1 330 aa
Bacillati Cyanobacteriota Melainabacteria group Cyanobacteriota Cyanophyceae Xenococcus sp. WP 006508190.1 321 aa
Bacillati Cyanobacteriota Melainabacteria group Cyanobacteriota Cyanophyceae Merismopedia glauca WP 181256760.1 330 aa
Bacillati Cyanobacteriota Melainabacteria group Cyanobacteriota Cyanophyceae Xenococcaceae cyanobacterium MDJ0690552.1 330 aa
Bacillati Cyanobacteriota Melainabacteria group Cyanobacteriota Cyanophyceae Symploca sp. NEP12142.1 330 aa
Bacillati Cyanobacteriota Melainabacteria group Cyanobacteriota Cyanophyceae Geitlerinema sp. WP 071515090.1 339 aa
Bacillati Cyanobacteriota Melainabacteria group Cyanobacteriota Cyanophyceae Calothrix rhizosoleniae WP 088241689.1 338 aa
Bacillati Cyanobacteriota Melainabacteria group Cyanobacteriota Cyanophyceae Roseofilum acuticapitatum WP 283754301.1 338 aa
Bacillati Cyanobacteriota Melainabacteria group Cyanobacteriota Cyanophyceae Spirulinaceae cyanobacterium NJL03053.1 342 aa
Bacillati Cyanobacteriota Melainabacteria group Cyanobacteriota Cyanophyceae Geitlerinema calcuttense WP 349775332.1 338 aa
Bacillati Cyanobacteriota Melainabacteria group Candidatus Melainabacteria Candidatus Gastranaerophilales Candidatus Gastranaerophilales MCX4274372.1 331 aa
Bacillati Cyanobacteriota Melainabacteria group Candidatus Melainabacteria Candidatus Gastranaerophilales Candidatus Gastranaerophilales HSA07230.1 332 aa
Bacillati Cyanobacteriota Melainabacteria group Candidatus Sericytochromatia Candidatus Sericytochromatia MEB3328935.1 326 aa
Bacillati Cyanobacteriota Melainabacteria group Cyanobacteriota Cyanophyceae Oscillatoriaceae cyanobacterium HEY9722720.1 328 aa
Bacillati Cyanobacteriota Melainabacteria group Candidatus Sericytochromatia Candidatus Sericytochromatia MFP5503648.1 326 aa
Bacillati Cyanobacteriota Melainabacteria group Cyanobacteriota Cyanophyceae Chroococcales cyanobacterium HEY9764837.1 319 aa
Bacillati Cyanobacteriota Melainabacteria group Cyanobacteriota Cyanophyceae Stenomitos sp. HEY9857430.1 324 aa
Bacillati Cyanobacteriota Melainabacteria group Cyanobacteriota Cyanophyceae Pantanalinema sp. HEY9899584.1 308 aa
Bacillati Cyanobacteriota Melainabacteria group Cyanobacteriota unclassified Cyanobacteriota Cyanobacteria bacterium MBI6545409.1 332 aa
Bacillati Cyanobacteriota Melainabacteria group Candidatus Sericytochromatia Candidatus Tanganyikabacteria MBM3259934.1 333 aa
Bacillati Cyanobacteriota Melainabacteria group Cyanobacteriota unclassified Cyanobacteriota Cyanobacteria bacterium MBU6427673.1 326 aa
Bacillati Cyanobacteriota Melainabacteria group Candidatus Sericytochromatia Candidatus Tanganyikabacteria MBM3268111.1 331 aa
Bacillati Cyanobacteriota Melainabacteria group Candidatus Melainabacteria Vampirovibriophyceae Candidatus Caenarcanales MCH2227153.1 333 aa
Bacillati Cyanobacteriota Melainabacteria group Candidatus Melainabacteria unclassified Candidatus Melainabacteria Candidatus Melainabacteria MBI1858922.1 317 aa
Bacillati Cyanobacteriota Melainabacteria group Candidatus Melainabacteria unclassified Candidatus Melainabacteria Candidatus Melainabacteria OGI06194.1 316 aa
Bacillati Cyanobacteriota Melainabacteria group Candidatus Melainabacteria unclassified Candidatus Melainabacteria Candidatus Melainabacteria MBI3591601.1 317 aa
Bacillati Cyanobacteriota Melainabacteria group Candidatus Melainabacteria Vampirovibriophyceae Candidatus Caenarcanales MDX1917558.1 336 aa
Bacillati Cyanobacteriota Melainabacteria group Candidatus Melainabacteria unclassified Candidatus Melainabacteria Candidatus Melainabacteria MFN5540448.1 336 aa
Bacillati Cyanobacteriota Melainabacteria group Candidatus Melainabacteria Vampirovibriophyceae Vampirovibrionales bacterium HEY9886340.1 335 aa
Bacillati Cyanobacteriota Melainabacteria group Candidatus Melainabacteria Vampirovibriophyceae Candidatus Caenarcanales HEY9886340.1 332 aa
Bacillati Cyanobacteriota Melainabacteria group Candidatus Melainabacteria Candidatus Obscuribacterales Candidatus Obscuribacterales MBX9770563.1 318 aa
Bacillati Cyanobacteriota Melainabacteria group Candidatus Melainabacteria Candidatus Obscuribacterales Candidatus Obscuribacterales MBY0357820.1 322 aa
Bacillati Cyanobacteriota Melainabacteria group Candidatus Melainabacteria unclassified Candidatus Melainabacteria Candidatus Melainabacteria MBI4534111.1 323 aa
Bacillati Cyanobacteriota Melainabacteria group Candidatus Melainabacteria Candidatus Obscuribacterales Candidatus Obscuribacterales MBX9770666.1 352 aa
Bacillati Cyanobacteriota Melainabacteria group Candidatus Melainabacteria unclassified Candidatus Melainabacteria Candidatus Melainabacteria PZM78114.1 331 aa
Bacillati Cyanobacteriota Melainabacteria group Cyanobacteriota Cyanophyceae Planktothrix sp. HEY9775124.1 324 aa
Bacillati Cyanobacteriota Melainabacteria group Cyanobacteriota Cyanophyceae Oculatellaceae cyanobacterium HEY9683589.1 328 aa
Bacillati Cyanobacteriota Melainabacteria group Cyanobacteriota Cyanophyceae Chroococcales cyanobacterium HEY9712764.1 323 aa
Bacillati Cyanobacteriota Melainabacteria group Candidatus Melainabacteria unclassified Candidatus Melainabacteria Candidatus Melainabacteria MBN9394907.1 323 aa
Bacillati Cyanobacteriota Melainabacteria group Candidatus Melainabacteria Candidatus Obscuribacterales Candidatus Obscuribacter MBK9769878.1 327 aa
Bacillati Cyanobacteriota Melainabacteria group Cyanobacteriota unclassified Cyanobacteriota Cyanobacteria bacterium MCW5824757.1 338 aa
Bacillati Cyanobacteriota Melainabacteria group Candidatus Melainabacteria Candidatus Obscuribacterales Candidatus Obscuribacterales MBX3076020.1 325 aa
Bacillati Cyanobacteriota Melainabacteria group Cyanobacteriota unclassified Cyanobacteriota Cyanobacteria bacterium MCA9815495.1 323 aa
Bacillati Cyanobacteriota Melainabacteria group Candidatus Melainabacteria unclassified Candidatus Melainabacteria Candidatus Melainabacteria MBK9143557.1 323 aa
Bacteria incertae sedis Candidatus Sumerlaeota Candidatus Sumerlaeia unclassified Candidatus Sumerlaeia Candidatus Sumerlaeia bacterium isolate MAG 26 NUP89365.1
Bacteria incertae sedis Candidatus Sumerlaeota Candidatus Sumerlaeia unclassified Candidatus Sumerlaeia Candidatus Sumerlaeia bacterium isolate bins.233 MDX2177720.1
Bacteria incertae sedis Candidatus Sumerlaeota unclassified Candidatus Sumerlaeota Candidatus Sumerlaeota bacterium isolate SJ802 MCC7391083.1
Bacteria incertae sedis Candidatus Sumerlaeota Candidatus Sumerlaeia unclassified Candidatus Sumerlaeia Candidatus Sumerlaeia bacterium isolate coassembly bin.29 MCC5877467.
Bacteria incertae sedis Candidatus Sumerlaeota Candidatus Sumerlaeia unclassified Candidatus Sumerlaeia Candidatus Sumerlaeia bacterium isolate bins.237 MDX1972544.1
Bacteria incertae sedis Candidatus Sumerlaeota unclassified Candidatus Sumerlaeota Candidatus Sumerlaeota bacterium isolate DJKA124 ANT21 bin.23 MDK2973130.1
Bacteria incertae sedis Candidatus Sumerlaeota Candidatus Sumerlaeia unclassified Candidatus Sumerlaeia Candidatus Sumerlaeia bacterium isolate ACE SUM1 MBX3730105.1
Bacteria incertae sedis Candidatus Sumerlaeota unclassified Candidatus Sumerlaeota Candidatus Sumerlaeota bacterium isolate D1M01 123 MFW5870561.1
Bacteria incertae sedis Candidatus Sumerlaeota unclassified Candidatus Sumerlaeota Candidatus Sumerlaeota bacterium isolate Zod Metabat.1318 iso2 MBN1868781.1
Bacteria incertae sedis Candidatus Sumerlaeota Candidatus Sumerlaeia unclassified Candidatus Sumerlaeia Candidatus Sumerlaeia bacterium isolate D1M03 6 MFP4381518.1
Bacteria incertae sedis Candidatus Sumerlaeota unclassified Candidatus Sumerlaeota candidate division BRC1 bacterium ADurb.Bin183 OQB20252.1
Bacteria incertae sedis Candidatus Sumerlaeota unclassified Candidatus Sumerlaeota Candidatus Sumerlaeota bacterium isolate Zod Metabat.1312 MBN1901465.1
Bacteria incertae sedis Candidatus Sumerlaeota unclassified Candidatus Sumerlaeota Candidatus Sumerlaeota bacterium isolate LacPavin 0920 SED5 MAG 50 10 MCX6999040.1
Bacteria incertae sedis Candidatus Sumerlaeota unclassified Candidatus Sumerlaeota Candidatus Sumerlaeota bacterium isolate Zod Metabat.292 MBN1515566.1
Bacteria incertae sedis Candidatus Sumerlaeota unclassified Candidatus Sumerlaeota Candidatus Sumerlaeota bacterium isolate LacPavin 0920 WC90 MAG 57 10 MCX7046688.1
Bacteria incertae sedis Candidatus Sumerlaeota unclassified Candidatus Sumerlaeota candidate division BRC1 bacterium SM23 51 KPL06106.1
Bacteria incertae sedis Candidatus Sumerlaeota Candidatus Sumerlaeia unclassified Candidatus Sumerlaeia Candidatus Sumerlaeia bacterium isolate Kow0059 GAB4318341.1
Bacteria incertae sedis Candidatus Sumerlaeota unclassified Candidatus Sumerlaeota candidate division BRC1 bacterium ADurb.BinA292 OPZ09344.1
Bacteria incertae sedis Candidatus Sumerlaeota unclassified Candidatus Sumerlaeota Candidatus Sumerlaeota bacterium isolate NC groundwater 1008 Pr1 S 0.65um 58 7 MBI3736936.
Bacteria incertae sedis Candidatus Sumerlaeota unclassified Candidatus Sumerlaeota Candidatus Sumerlaeota bacterium isolate B28T3L14 MCH8334244.1
Bacteria incertae sedis Candidatus Sumerlaeota Candidatus Sumerlaeia Candidatus Sumerlaeales Candidatus Sumerlaeaceae bacterium isolate TH ant mag 81 MGI8908349.1
Bacteria incertae sedis Candidatus Sumerlaeota unclassified Candidatus Sumerlaeota candidate division BRC1 bacterium HGW BRC1 1 PKO16556.1
Bacteria incertae sedis Candidatus Sumerlaeota Candidatus Sumerlaeia Candidatus Sumerlaeales Candidatus Sumerlaeaceae bacterium isolate SKW119 MCX7625496.1
Bacteria incertae sedis Candidatus Sumerlaeota Candidatus Sumerlaeia Candidatus Sumerlaeales Candidatus Sumerlaea chitinivorans isolate BY40 AXA35475.1
Bacteria incertae sedis Candidatus Sumerlaeota unclassified Candidatus Sumerlaeota Candidatus Sumerlaeota bacterium isolate LacPavin 0419 WC70S MAG 58 10 MCX7017960.1
Bacteria incertae sedis Candidatus Sumerlaeota Candidatus Sumerlaeia Candidatus Sumerlaeales Candidatus Sumerlaeaceae bacterium isolate SSF23 MBX7244875.1
Bacteria incertae sedis Candidatus Sumerlaeota Candidatus Sumerlaeia Candidatus Sumerlaeales Candidatus Sumerlaeales bacterium isolate STF1 15 MDD4279984.1
Bacteria incertae sedis Candidatus Sumerlaeota Candidatus Sumerlaeia Candidatus Sumerlaeales Candidatus Sumerlaeaceae bacterium isolate SKW162 MCX7719519.1
Bacteria incertae sedis candidate division WOR 49 candidate division WOR 3 bacterium isolate RS 16 32 MCK4395515.1
Bacteria incertae sedis candidate division WOR 34 candidate division WOR 3 bacterium isolate RS 19 7 MCK4233201.1
Bacteria incertae sedis candidate division WOR 70 candidate division WOR 3 bacterium isolate Zod Metabat.631 iso2 MBN1150688.1
Bacteria incertae sedis candidate division WOR 76 candidate division WOR 3 bacterium isolate Zod Metabat.185 iso2 MBN1619748.1
Bacteria incertae sedis candidate division WOR 75 candidate division WOR 3 bacterium isolate Zod Metabat.912 iso2 MBN2364399.1
Bacteria incertae sedis candidate division WOR 10 candidate division WOR 3 bacterium isolate RS 16 8 MCK4352582.1
Bacteria incertae sedis candidate division WOR 42 candidate division WOR 3 bacterium isolate RS 17 14 MCK4329553.1
Bacteria incertae sedis candidate division WOR 52 candidate division WOR 3 bacterium isolate MAG055 MEA3431974.1
Bacteria incertae sedis candidate division WOR 64 candidate division WOR 3 bacterium isolate MAG 1066 MCD5383720.1

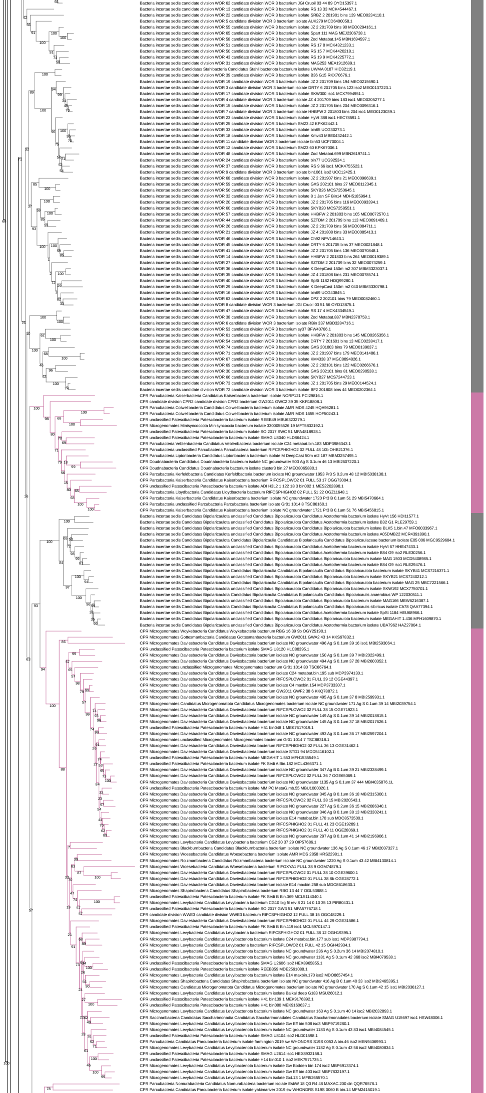

Bacteria incertae sedis candidate division WOR 62 candidate division WOR 3 bacterium JGI Cruoil 03 44 89 OYD15397.1
Bacteria incertae sedis candidate division WOR 13 candidate division WOR 3 bacterium isolate RS 13 33 MCK4544467.1
Bacteria incertae sedis candidate division WOR 22 candidate division WOR 3 bacterium isolate SRBZ 2 201901 bins 139 MEO0234110.1
Bacteria incertae sedis candidate division WOR 5 candidate division WOR 3 bacterium isolate AUK279 MCD6400058.1
Bacteria incertae sedis candidate division WOR 55 candidate division WOR 3 bacterium isolate JZ 2 201709 bins 90 MEO0294161.1
Bacteria incertae sedis candidate division WOR 65 candidate division WOR 3 bacterium isolate Spart 111 MAG MEJ2306738.1
Bacteria incertae sedis candidate division WOR 58 candidate division WOR 3 bacterium isolate Zod Metabat.145 MBN1694597.1
Bacteria incertae sedis candidate division WOR 51 candidate division WOR 3 bacterium isolate RS 17 8 MCK4321233.1
Bacteria incertae sedis candidate division WOR 50 candidate division WOR 3 bacterium isolate RS 15 7 MCK4420218.1
Bacteria incertae sedis candidate division WOR 43 candidate division WOR 3 bacterium isolate RS 19 9 MCK4225772.1
Bacteria incertae sedis candidate division WOR 31 candidate division WOR 3 bacterium isolate MAG253 MEA1912689.1
Bacteria incertae sedis Candidatus Stahlbacteria Candidatus Stahlbacteriota bacterium isolate UWMA 0187 HID32119.1
Bacteria incertae sedis candidate division WOR 39 candidate division WOR 3 bacterium isolate B36 G15 RKX70676.1
Bacteria incertae sedis candidate division WOR 19 candidate division WOR 3 bacterium isolate JZ 2 201709 bins 194 MEO0215690.1
Bacteria incertae sedis candidate division WOR 3 candidate division WOR 3 bacterium isolate DRTY 6 201705 bins 123 iso2 MEO0137223.1
Bacteria incertae sedis candidate division WOR 17 candidate division WOR 3 bacterium isolate SKW300 iso1 MCX7994951.1
Bacteria incertae sedis candidate division WOR 4 candidate division WOR 3 bacterium isolate JZ 4 201709 bins 183 iso1 MEO0205277.1
Bacteria incertae sedis candidate division WOR 15 candidate division WOR 3 bacterium isolate JZ 2 201705 bins 204 MEO0096316.1
Bacteria incertae sedis candidate division WOR 7 candidate division WOR 3 bacterium isolate HHBFW 2 201803 bins 204 iso1 MEO0123039.1
Bacteria incertae sedis candidate division WOR 23 candidate division WOR 3 bacterium isolate HyVt 388 iso1 HEC78591.1
Bacteria incertae sedis candidate division WOR 25 candidate division WOR 3 bacterium isolate SM23 42 KPK62442.1
Bacteria incertae sedis candidate division WOR 33 candidate division WOR 3 bacterium isolate bin65 UCG30273.1
Bacteria incertae sedis candidate division WOR 18 candidate division WOR 3 bacterium isolate Kmv43 MBE0432442.1
Bacteria incertae sedis candidate division WOR 11 candidate division WOR 3 bacterium isolate bin53 UCF70004.1
Bacteria incertae sedis candidate division WOR 12 candidate division WOR 3 bacterium isolate SM23 60 KPK67006.1
Bacteria incertae sedis candidate division WOR 48 candidate division WOR 3 bacterium isolate Zod Metabat.699 MBN2619741.1
Bacteria incertae sedis candidate division WOR 24 candidate division WOR 3 bacterium isolate bin77 UCG92534.1
Bacteria incertae sedis candidate division WOR 37 candidate division WOR 3 bacterium isolate RS 9 66 iso1 MCK4755523.1
Bacteria incertae sedis candidate division WOR 9 candidate division WOR 3 bacterium isolate bin1061 iso2 UCC12425.1
Bacteria incertae sedis candidate division WOR 68 candidate division WOR 3 bacterium isolate JZ 2 201907 bins 21 MEO0098639.1
Bacteria incertae sedis candidate division WOR 59 candidate division WOR 3 bacterium isolate GXS 202101 bins 27 MEO0112345.1
Bacteria incertae sedis candidate division WOR 56 candidate division WOR 3 bacterium isolate SKYB26 MCS7250645.1
Bacteria incertae sedis candidate division WOR 32 candidate division WOR 3 bacterium isolate 8 1 Jan SF Bin14 MDH5185994.1
Bacteria incertae sedis candidate division WOR 20 candidate division WOR 3 bacterium isolate JZ 2 201705 bins 116 MEO0093394.1
Bacteria incertae sedis candidate division WOR 60 candidate division WOR 3 bacterium isolate SKYB20 MCS7258551.1
Bacteria incertae sedis candidate division WOR 57 candidate division WOR 3 bacterium isolate HHBFW 2 201803 bins 105 MEO0072570.1
Bacteria incertae sedis candidate division WOR 44 candidate division WOR 3 bacterium isolate SZTDM 2 201709 bins 113 MEO0091409.1
Bacteria incertae sedis candidate division WOR 26 candidate division WOR 3 bacterium isolate JZ 2 201709 bins 56 MEO0084711.1
Bacteria incertae sedis candidate division WOR 21 candidate division WOR 3 bacterium isolate JZ 4 201808 bins 33 MEO0085413.1
Bacteria incertae sedis candidate division WOR 46 candidate division WOR 3 bacterium isolate Ch92 NPV14643.1
Bacteria incertae sedis candidate division WOR 45 candidate division WOR 3 bacterium isolate DRTY 6 201705 bins 37 MEO0021848.1
Bacteria incertae sedis candidate division WOR 41 candidate division WOR 3 bacterium isolate JZ 2 201705 bins 136 MEO0070848.1
Bacteria incertae sedis candidate division WOR 14 candidate division WOR 3 bacterium isolate HHBFW 2 201803 bins 264 MEO0019389.1
Bacteria incertae sedis candidate division WOR 27 candidate division WOR 3 bacterium isolate SZTDM 2 201709 bins 32 MEO0073259.1
Bacteria incertae sedis candidate division WOR 36 candidate division WOR 3 bacterium isolate K DeepCast 150m m2 307 MBM3323037.1
Bacteria incertae sedis candidate division WOR 35 candidate division WOR 3 bacterium isolate JZ 4 201808 bins 231 MEO0078574.1
Bacteria incertae sedis candidate division WOR 40 candidate division WOR 3 bacterium isolate SpSt 1182 HDQ99280.1
Bacteria incertae sedis candidate division WOR 29 candidate division WOR 3 bacterium isolate K DeepCast 150m m2 040 MBM3330798.1
Bacteria incertae sedis candidate division WOR 16 candidate division WOR 3 bacterium isolate bin69 UCG43845.1
Bacteria incertae sedis candidate division WOR 63 candidate division WOR 3 bacterium isolate DPZ 2 202101 bins 79 MEO0082460.1
Bacteria incertae sedis candidate division WOR 8 candidate division WOR 3 bacterium JGI Cruoil 03 51 56 OYD13875.1
Bacteria incertae sedis candidate division WOR 47 candidate division WOR 3 bacterium isolate RS 17 4 MCK4334549.1
Bacteria incertae sedis candidate division WOR 38 candidate division WOR 3 bacterium isolate Zod Metabat.887 MBN2378758.1
Bacteria incertae sedis candidate division WOR 6 candidate division WOR 3 bacterium isolate RBin 337 MBD3284716.1
Bacteria incertae sedis candidate division WOR 53 candidate division WOR 3 bacterium sy37 BFW40786.1
Bacteria incertae sedis candidate division WOR 61 candidate division WOR 3 bacterium isolate HHBFW 2 201803 bins 145 MEO0265356.1
Bacteria incertae sedis candidate division WOR 54 candidate division WOR 3 bacterium isolate DRTY 7 201601 bins 13 MEO0238417.1
Bacteria incertae sedis candidate division WOR 74 candidate division WOR 3 bacterium isolate GXS 201803 bins 79 MEO0139037.1
Bacteria incertae sedis candidate division WOR 71 candidate division WOR 3 bacterium isolate JZ 2 201907 bins 179 MEO0141486.1
Bacteria incertae sedis candidate division WOR 67 candidate division WOR 3 bacterium isolate KM4338 37 MGC8894826.1
Bacteria incertae sedis candidate division WOR 69 candidate division WOR 3 bacterium isolate JZ 2 202101 bins 122 MEO0266676.1
Bacteria incertae sedis candidate division WOR 30 candidate division WOR 3 bacterium isolate GXS 202101 bins 81 MEO0290538.1
Bacteria incertae sedis candidate division WOR 66 candidate division WOR 3 bacterium isolate SKYB27 MCS7244723.1
Bacteria incertae sedis candidate division WOR 73 candidate division WOR 3 bacterium isolate JZ 1 201705 bins 29 MEO0144524.1
Bacteria incertae sedis candidate division WOR 72 candidate division WOR 3 bacterium isolate BF2 201808 bins 44 MEO0202364.1
CPR Parcubacteria Kaiserbacteria Candidatus Kaiserbacteria bacterium isolate NORP121 PCI29816.1
CPR candidate division CPR2 candidate division CPR2 bacterium GW2011 GWC2 39 35 KKR18808.1
CPR Parcubacteria Colwellbacteria Candidatus Colwellbacteria bacterium isolate AMR MDS 4245 HQA96281.1
CPR Parcubacteria Colwellbacteria Candidatus Colwellbacteria bacterium isolate AMR MDS 1655 HOF50243.1
CPR unclassified Patescibacteria Patescibacteria bacterium isolate REEB49 MBU6323279.1
CPR Microgenomates Minisyncoccota Minisyncoccia bacterium isolate 3300055526 19 MFT5832192.1
CPR unclassified Patescibacteria Patescibacteria bacterium isolate SO 2017 SWC 51 MFA4818928.1
CPR unclassified Patescibacteria Patescibacteria bacterium isolate SMAG U8040 HLD86424.1
CPR Parcubacteria Veblenbacteria Candidatus Veblenbacteria bacterium isolate C24 metabat.bin.183 MDP3986343.1
CPR Parcubacteria unclassified Parcubacteria Parcubacteria bacterium RIFCSPHIGHO2 02 FULL 48 10b OHB21376.1
CPR Parcubacteria Liptonbacteria Candidatus Liptonbacteria bacterium isolate M DeepCast 50m m2 187 MBM3257495.1
CPR Doudnabacteria Candidatus Doudnabacteria bacterium isolate NC groundwater 503 Ag S 0.1um 46 13 MBI2607220.1
CPR Doudnabacteria Candidatus Doudnabacteria bacterium isolate cluster3 bin.27 MEO8065880.1
CPR Parcubacteria Kerfeldbacteria Candidatus Kerfeldbacteria bacterium isolate NC groundwater 1953 Pr3 S 0.2um 48 12 MBI5038138.1
CPR Parcubacteria Kaiserbacteria Candidatus Kaiserbacteria bacterium RIFCSPLOWO2 01 FULL 53 17 OGG73004.1
CPR unclassified Patescibacteria Patescibacteria bacterium isolate ADI H3L2 1 122 18 3 bin002 1 MES2202898.1
CPR Parcubacteria Lloydbacteria Candidatus Lloydbacteria bacterium RIFCSPHIGHO2 02 FULL 51 22 OGZ11648.1
CPR Parcubacteria Kaiserbacteria Candidatus Kaiserbacteria bacterium isolate NC groundwater 1720 Pr3 B 0.1um 51 29 MBI5470664.1
CPR Parcubacteria unclassified Parcubacteria Parcubacteria bacterium isolate Gr01 1014 8 TSC86160.1
CPR Parcubacteria Kaiserbacteria Candidatus Kaiserbacteria bacterium isolate NC groundwater 1721 Pr3 B 0.1um 51 76 MBI5456815.1
Bacteria incertae sedis Candidatus Bipolaricaulota unclassified Candidatus Bipolaricaulota Candidatus Acetothermia bacterium isolate HyVt 156 HDI11577.1
Bacteria incertae sedis Candidatus Bipolaricaulota unclassified Candidatus Bipolaricaulota Candidatus Acetothermia bacterium isolate B32 G1 RLE29759.1
Bacteria incertae sedis Candidatus Bipolaricaulota unclassified Candidatus Bipolaricaulota Candidatus Bipolaricaulota bacterium isolate BLK5 1.bin.47 MFO8033967.1
Bacteria incertae sedis Candidatus Bipolaricaulota unclassified Candidatus Bipolaricaulota Candidatus Acetothermia bacterium isolate A05DMB22 MCR4391890.1
Bacteria incertae sedis Candidatus Bipolaricaulota Candidatus Bipolaricaulia Candidatus Bipolaricaulia Candidatus Bipolaricaulaceae bacterium isolate E05 006 MGC9529684.1
Bacteria incertae sedis Candidatus Bipolaricaulota unclassified Candidatus Bipolaricaulota Candidatus Acetothermia bacterium isolate HyVt 67 HHE47433.1
Bacteria incertae sedis Candidatus Bipolaricaulota unclassified Candidatus Bipolaricaulota Candidatus Acetothermia bacterium isolate B84 G9 iso2 RLE30256.1
Bacteria incertae sedis Candidatus Bipolaricaulota unclassified Candidatus Bipolaricaulota Candidatus Bipolaricaulota bacterium isolate MAG 1503 MCD5408985.1
Bacteria incertae sedis Candidatus Bipolaricaulota unclassified Candidatus Bipolaricaulota Candidatus Acetothermia bacterium isolate B84 G9 iso1 RLE29476.1
Bacteria incertae sedis Candidatus Bipolaricaulota Candidatus Bipolaricaulia Candidatus Bipolaricaulia Candidatus Bipolaricaulota bacterium isolate SKYB41 MCS7216371.1
Bacteria incertae sedis Candidatus Bipolaricaulota unclassified Candidatus Bipolaricaulota Candidatus Bipolaricaulota bacterium isolate SKYB21 MCS7240212.1
Bacteria incertae sedis Candidatus Bipolaricaulota Candidatus Bipolaricaulia Candidatus Bipolaricaulia Candidatus Bipolaricaulota bacterium isolate MAG 25 MBC7221566.1
Bacteria incertae sedis Candidatus Bipolaricaulota unclassified Candidatus Bipolaricaulota Candidatus Bipolaricaulota bacterium isolate SKW192 MCX7750701.1
Bacteria incertae sedis Candidatus Bipolaricaulota Candidatus Bipolaricaulia Candidatus Bipolaricaulia Candidatus Bipolaricaulis anaerobius WP 122030511.1
Bacteria incertae sedis Candidatus Bipolaricaulota unclassified Candidatus Bipolaricaulota Candidatus Bipolaricaulota bacterium isolate MAG166 MEW6216387.1
Bacteria incertae sedis Candidatus Bipolaricaulota Candidatus Bipolaricaulia Candidatus Bipolaricaulia Candidatus Bipolaricaulis sibiricus isolate Ch78 QAA77394.1
Bacteria incertae sedis Candidatus Bipolaricaulota unclassified Candidatus Bipolaricaulota Candidatus Acetothermia bacterium isolate SpSt 1184 HEU68966.1
Bacteria incertae sedis Candidatus Bipolaricaulota unclassified Candidatus Bipolaricaulota Candidatus Bipolaricaulota bacterium isolate MEGAHIT 1.436 MFH1609870.1
Bacteria incertae sedis Candidatus Bipolaricaulota unclassified Candidatus Bipolaricaulota Candidatus Acetothermia bacterium isolate UBA7962 HAZ27804.1
CPR Microgenomates Woykebacteria Candidatus Woykebacteria bacterium RBG 16 39 9b OGY25190.1
CPR Microgenomates Gottesmanbacteria Candidatus Gottesmanbacteria bacterium GW2011 GWA2 43 14 KKS97832.1
CPR Microgenomates Daviesbacteria Candidatus Daviesbacteria bacterium isolate NC groundwater 496 Ag S 0.1um 39 16 iso1 MBI2593064.1
CPR unclassified Patescibacteria Patescibacteria bacterium isolate SMAG U8120 HLC88395.1
CPR Microgenomates Daviesbacteria Candidatus Daviesbacteria bacterium isolate NC groundwater 150 Ag S 0.1um 39 7 MBI2022499.1
CPR Microgenomates Daviesbacteria Candidatus Daviesbacteria bacterium isolate NC groundwater 494 Ag S 0.1um 37 28 MBI2600352.1
CPR Microgenomates unclassified Microgenomates Microgenomates bacterium Gr01 1014 80 TSC66764.1
CPR Microgenomates Daviesbacteria Candidatus Daviesbacteria bacterium isolate C24 metabat.bin.195 sub MDP3974130.1
CPR Microgenomates Daviesbacteria Candidatus Daviesbacteria bacterium RIFCSPLOWO2 01 FULL 39 12 OGE43497.1
CPR Microgenomates Daviesbacteria Candidatus Daviesbacteria bacterium isolate C4 maxbin.154 MDP3733307.1
CPR Microgenomates Daviesbacteria Candidatus Daviesbacteria bacterium GW2011 GWF2 38 6 KKQ78872.1
CPR Microgenomates Daviesbacteria Candidatus Daviesbacteria bacterium isolate NC groundwater 495 Ag S 0.1um 37 8 MBI2599931.1
CPR Microgenomates Candidatus Microgenomatota Candidatus Microgenomates bacterium isolate NC groundwater 171 Ag S 0.1um 39 14 MBI2039754.1
CPR Microgenomates Daviesbacteria Candidatus Daviesbacteria bacterium RIFCSPLOWO2 02 FULL 38 15 OGE71923.1
CPR Microgenomates Daviesbacteria Candidatus Daviesbacteria bacterium isolate NC groundwater 149 Ag S 0.1um 39 14 MBI2018815.1
CPR Microgenomates Daviesbacteria Candidatus Daviesbacteria bacterium isolate NC groundwater 145 Ag S 0.1um 37 18 MBI2017626.1
CPR unclassified Patescibacteria Patescibacteria bacterium isolate H51 bin048 1 MEK7617019.1
CPR Microgenomates Daviesbacteria Candidatus Daviesbacteria bacterium isolate NC groundwater 493 Ag S 0.1um 36 17 MBI2597204.1
CPR Microgenomates unclassified Microgenomates Microgenomates bacterium Gr01 1014 7 TSC88318.1
CPR Microgenomates Daviesbacteria Candidatus Daviesbacteria bacterium RIFCSPHIGHO2 02 FULL 36 13 OGE31462.1
CPR Microgenomates Daviesbacteria Candidatus Daviesbacteria bacterium isolate STD1 94 MDD5416102.1
CPR unclassified Patescibacteria Patescibacteria bacterium isolate MEGAHIT 1.553 MFH1535549.1
CPR unclassified Patescibacteria Patescibacteria bacterium isolate FK Sedi A Bin.182 MCL4366371.1
CPR Microgenomates Daviesbacteria Candidatus Daviesbacteria bacterium isolate NC groundwater 347 Ag B 0.1um 39 21 MBI2338499.1
CPR Microgenomates Daviesbacteria Candidatus Daviesbacteria bacterium RIFCSPLOWO2 02 FULL 36 7 OGE65069.1
CPR Microgenomates Daviesbacteria Candidatus Daviesbacteria bacterium isolate NC groundwater 1135 Ag S 0.1um 37 444 MBI4035876.1L
CPR unclassified Patescibacteria Patescibacteria bacterium isolate MM PC MetaG.mb.55 MBU1000020.1
CPR Microgenomates Daviesbacteria Candidatus Daviesbacteria bacterium isolate NC groundwater 345 Ag B 0.1um 36 18 MBI2315300.1
CPR Microgenomates Daviesbacteria Candidatus Daviesbacteria bacterium RIFCSPLOWO2 02 FULL 38 15 MBI2020543.1
CPR Microgenomates Daviesbacteria Candidatus Daviesbacteria bacterium isolate NC groundwater 227 Ag S 0.2um 36 15 MBI2086340.1
CPR Microgenomates Daviesbacteria Candidatus Daviesbacteria bacterium isolate NC groundwater 346 Ag B 0.1um 38 13 MBI2330241.1
CPR Microgenomates Daviesbacteria Candidatus Daviesbacteria bacterium isolate E14 metabat.bin.170 sub MDO8573500.1
CPR Microgenomates Daviesbacteria Candidatus Daviesbacteria bacterium RIFCSPHIGHO2 01 FULL 41 23 OGE19289.1
CPR Microgenomates Daviesbacteria Candidatus Daviesbacteria bacterium RIFCSPHIGHO2 01 FULL 40 11 OGE28069.1
CPR Microgenomates Daviesbacteria Candidatus Daviesbacteria bacterium isolate NC groundwater 297 Ag B 0.1um 41 14 MBI2196906.1
CPR Microgenomates Levybacteria Candidatus Levybacteria bacterium CG2 30 37 29 OIP57686.1
CPR Microgenomates Blackburnbacteria Candidatus Blackburnbacteria bacterium isolate NC groundwater 136 Ag S 0.1um 46 17 MBI2007327.1
CPR Microgenomates Woesebacteria Candidatus Woesebacteria bacterium isolate AMR MDS 2858 HRS22981.1
CPR Microgenomates Roizmanbacteria Candidatus Roizmanbacteria bacterium isolate NC groundwater 1220 Ag S 0.1um 43 42 MBI4130814.1
CPR Microgenomates Woesebacteria Candidatus Woesebacteria bacterium RIFOXYA1 FULL 38 9 OGM74879.1
CPR Microgenomates Daviesbacteria Candidatus Daviesbacteria bacterium RIFCSPLOWO2 01 FULL 38 10 OGE39600.1
CPR Microgenomates Daviesbacteria Candidatus Daviesbacteria bacterium RIFCSPHIGHO2 01 FULL 38 8b OGE28772.1
CPR Microgenomates Daviesbacteria Candidatus Daviesbacteria bacterium isolate E14 maxbin.258 sub MDO8618630.1
CPR Microgenomates Shapirobacteria Candidatus Shapirobacteria bacterium RBG 13 44 7 OGL53888.1
CPR unclassified Patescibacteria Patescibacteria bacterium isolate FK Sedi B Bin.369 MCL5114040.1
CPR Microgenomates Levybacteria Candidatus Levybacteria bacterium CG10 big fil rev 8 21 14 0 10 35 13 PIR80431.1
CPR unclassified Patescibacteria Patescibacteria bacterium isolate SO 2017 GW3 51 MFA5776718.1
CPR candidate division WWE3 candidate division WWE3 bacterium RIFCSPHIGHO2 12 FULL 38 15 OGC48229.1
CPR Microgenomates Daviesbacteria Candidatus Daviesbacteria bacterium RIFCSPHIGHO2 01 FULL 44 29 OGE31586.1
CPR unclassified Patescibacteria Patescibacteria bacterium isolate FK Sedi B Bin.119 iso1 MCL5970147.1
CPR Microgenomates Levybacteria Candidatus Levybacteria bacterium RIFCSPHIGHO2 01 FULL 38 12 OGH19395.1
CPR Microgenomates Levybacteria Candidatus Levyibacteriota bacterium isolate C24 metabat.bin.177 sub iso1 MDP3987794.1
CPR Microgenomates Levybacteria Candidatus Levybacteria bacterium RIFCSPLOWO2 01 FULL 42 15 OGH42934.1
CPR Microgenomates Levybacteria Candidatus Levyibacteriota bacterium isolate NC groundwater 236 Ag S 0.2um 36 14 MBI2074810.1
CPR Microgenomates Levybacteria Candidatus Levyibacteriota bacterium isolate NC groundwater 1181 Ag S 0.1um 42 368 iso2 MBI4079538.1
CPR unclassified Patescibacteria Patescibacteria bacterium isolate SMAG U2606 iso2 HEX8965855.1
CPR unclassified Patescibacteria Patescibacteria bacterium isolate REEB359 MDE2591088.1
CPR Microgenomates Levybacteria Candidatus Levyibacteriota bacterium isolate E14 maxbin.170 iso2 MDO8657454.1
CPR Microgenomates Shapirobacteria Candidatus Shapirobacteria bacterium isolate NC groundwater 416 Ag B 0.1um 40 33 iso2 MBI2465395.1
CPR Microgenomates Candidatus Microgenomatota Candidatus Microgenomates bacterium isolate NC groundwater 170 Ag S 0.1um 42 15 iso1 MBI2036127.1
CPR Microgenomates Levybacteria Candidatus Levyibacteriota bacterium isolate Baikal deep G183 MSU26012.1
CPR unclassified Patescibacteria Patescibacteria bacterium isolate H41 bin139 1 MEK9176892.1
CPR unclassified Patescibacteria Patescibacteria bacterium isolate H41 bin080 MEK9160637.1
CPR Microgenomates Levybacteria Candidatus Levyibacteriota bacterium isolate NC groundwater 163 Ag S 0.1um 40 14 iso2 MBI2032893.1
CPR Saccharibacteria Candidatus Saccharimonadia Candidatus Saccharimonadales Candidatus Saccharimonadales bacterium isolate SMAG U15697 iso1 HSW48006.1
CPR Microgenomates Levybacteria Candidatus Levyibacteriota bacterium isolate Gw Eff bin 508 iso3 MBP9719280.1
CPR Microgenomates Levybacteria Candidatus Levyibacteriota bacterium isolate NC groundwater 1183 Ag S 0.1um 43 83 iso1 MBI4084545.1
CPR unclassified Patescibacteria Patescibacteria bacterium isolate SMAG U8104 iso2 HLD01598.1
CPR Parcubacteria Candidatus Parcubacteria bacterium isolate farmington 2019 sw WHONDRS S19S 0053 A bin.46 iso2 MEN9406993.1
CPR Microgenomates Levybacteria Candidatus Levyibacteriota bacterium isolate NC groundwater 1182 Ag S 0.1um 43 56 iso2 MBI4080834.1
CPR unclassified Patescibacteria Patescibacteria bacterium isolate SMAG U2614 iso1 HEX8932158.1
CPR unclassified Patescibacteria Patescibacteria bacterium isolate H14 bin010 1 iso2 MEK7571735.1
CPR Microgenomates Levybacteria Candidatus Levyibacteriota bacterium isolate Gw Bodden bin 174 iso2 MBP6913374.1
CPR Microgenomates Levybacteria Candidatus Levyibacteriota bacterium isolate Gw Eff bin 403 iso2 MBP7832197.1
CPR Microgenomates Levybacteria Candidatus Levyibacteriota bacterium isolate GcL13 1 MFI5265570.1
CPR Parcubacteria Nomurabacteria Candidatus Nomurabacteria bacterium isolate EsbW 18 Q3 R4 48 MAXAC.200 cln QQR76578.1
CPR Parcubacteria Candidatus Parcubacteria bacterium isolate yakimariver 2019 sw WHONDRS S19S 0060 B bin.14 MFM2415019.1

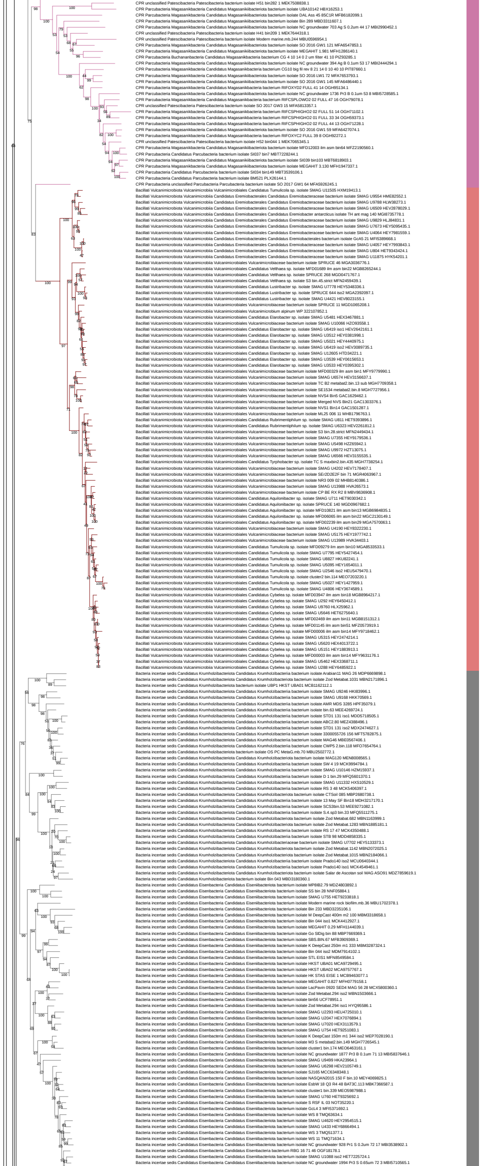

CPR unclassified Patescibacteria Patescibacteria bacterium isolate H51 bin282 1 MEK7508838.1
CPR Parcubacteria Magasanikbacteria Candidatus Magasanikiibacteriota bacterium isolate UBA10142 HBX16253.1
CPR Parcubacteria Magasanikbacteria Candidatus Magasanikiibacteriota bacterium isolate DAL Ass 45 85C1R MFB6182099.1
CPR Parcubacteria Magasanikbacteria Candidatus Magasanikiibacteriota bacterium isolate Bin 289 MBD3311607.1
CPR Parcubacteria Magasanikbacteria Candidatus Magasanikiibacteriota bacterium isolate NC groundwater 703 Ag S 0.2um 44 17 MBI2990452.1
CPR unclassified Patescibacteria Patescibacteria bacterium isolate H41 bin209 1 MEK7644318.1
CPR unclassified Patescibacteria Patescibacteria bacterium isolate Modern marine.mb.244 MBU0596954.1
CPR Parcubacteria Magasanikbacteria Candidatus Magasanikiibacteriota bacterium isolate SO 2016 GW1 121 MFA6547853.1
CPR Parcubacteria Magasanikbacteria Candidatus Magasanikiibacteriota bacterium isolate MEGAHIT 1.981 MFH1286140.1
CPR Parcubacteria Buchananbacteria Candidatus Magasanikbacteria bacterium CG 4 10 14 0 2 um filter 41 10 PIZ93285.1
CPR Parcubacteria Magasanikbacteria Candidatus Magasanikiibacteriota bacterium isolate NC groundwater 394 Ag B 0.1um 53 17 MBI2444294.1
CPR Parcubacteria Magasanikbacteria Candidatus Magasanikbacteria bacterium CG10 big fil rev 8 21 14 0 10 40 10 PIT87660.1
CPR Parcubacteria Magasanikbacteria Candidatus Magasanikiibacteriota bacterium isolate SO 2016 LW1 72 MFA7653793.1
CPR Parcubacteria Magasanikbacteria Candidatus Magasanikiibacteriota bacterium isolate SO 2016 GW1 145 MFA6486440.1
CPR Parcubacteria Magasanikbacteria Candidatus Magasanikbacteria bacterium RIFOXYD2 FULL 41 14 OGH95134.1
CPR Parcubacteria Magasanikbacteria Candidatus Magasanikiibacteriota bacterium isolate NC groundwater 1736 Pr3 B 0.1um 53 8 MBI5728585.1
CPR Parcubacteria Magasanikbacteria Candidatus Magasanikbacteria bacterium RIFCSPLOWO2 02 FULL 47 16 OGH79078.1
CPR unclassified Patescibacteria Patescibacteria bacterium isolate SO 2017 GW3 15 MFA5813357.1
CPR Parcubacteria Magasanikbacteria Candidatus Magasanikbacteria bacterium RIFCSPHIGHO2 02 FULL 51 14 OGH71102.1
CPR Parcubacteria Magasanikbacteria Candidatus Magasanikbacteria bacterium RIFCSPHIGHO2 01 FULL 33 34 OGH59373.1
CPR Parcubacteria Magasanikbacteria Candidatus Magasanikbacteria bacterium RIFCSPHIGHO2 02 FULL 44 13 OGH71228.1
CPR Parcubacteria Magasanikbacteria Candidatus Magasanikiibacteriota bacterium isolate SO 2016 GW1 59 MFA6427074.1
CPR Parcubacteria Magasanikbacteria Candidatus Magasanikbacteria bacterium RIFOXYC2 FULL 39 8 OGH92272.1
CPR unclassified Patescibacteria Patescibacteria bacterium isolate H52 bin044 1 MEK7065345.1
CPR Parcubacteria Magasanikbacteria Candidatus Magasanikiibacteriota bacterium isolate MFD12003 ilm asm bin64 MFZ2190560.1
CPR Parcubacteria Candidatus Parcubacteria bacterium isolate SI037 bin7 MBT7228244.1
CPR Parcubacteria Magasanikbacteria Candidatus Magasanikiibacteriota bacterium isolate SI039 bin103 MBT6818903.1
CPR Parcubacteria Magasanikbacteria Candidatus Magasanikiibacteriota bacterium isolate MEGAHIT 3.130 MFH1947337.1
CPR Parcubacteria Candidatus Parcubacteria bacterium isolate SI034 bin149 MBT3539106.1
CPR Parcubacteria Candidatus Parcubacteria bacterium isolate BM521 PLX26144.1
CPR Parcubacteria unclassified Parcubacteria Parcubacteria bacterium isolate SO 2017 GW1 64 MFA5926245.1
Bacillati Vulcanimicrobiota Vulcanimicrobiia Vulcanimicrobiales Candidatus Tumulicola sp. isolate SMAG U11505 HXM19413.1
Bacillati Vulcanimicrobiota Vulcanimicrobiia Candidatus Eremiobacterales Candidatus Eremiobacteraceae bacterium isolate SMAG U9554 HME82552.1
Bacillati Vulcanimicrobiota Vulcanimicrobiia Candidatus Eremiobacterales Candidatus Eremiobacteraceae bacterium isolate SMAG U9788 HLW38273.1
Bacillati Vulcanimicrobiota Vulcanimicrobiia Candidatus Eremiobacterales Candidatus Eremiobacteraceae bacterium isolate SMAG U6509 HEV2878029.1
Bacillati Vulcanimicrobiota Vulcanimicrobiia Candidatus Eremiobacterales Candidatus Eremiobacter antarcticus isolate TH ant mag 140 MGI8735778.1
Bacillati Vulcanimicrobiota Vulcanimicrobiia Candidatus Eremiobacterales Candidatus Eremiobacteraceae bacterium isolate SMAG U9829 HLJ84831.1
Bacillati Vulcanimicrobiota Vulcanimicrobiia Candidatus Eremiobacterales Candidatus Eremiobacteraceae bacterium isolate SMAG U7673 HEY5095435.1
Bacillati Vulcanimicrobiota Vulcanimicrobiia Candidatus Eremiobacterales Candidatus Eremiobacteraceae bacterium isolate SMAG U4064 HEY7981559.1
Bacillati Vulcanimicrobiota Vulcanimicrobiia Candidatus Eremiobacterales Candidatus Eremiobacterales bacterium isolate GcA5 21 MFI5389668.1
Bacillati Vulcanimicrobiota Vulcanimicrobiia Candidatus Eremiobacterales Candidatus Eremiobacteraceae bacterium isolate SMAG U4057 HEY7993843.1
Bacillati Vulcanimicrobiota Vulcanimicrobiia Candidatus Eremiobacterales Candidatus Eremiobacteraceae bacterium isolate SMAG U804 HET9343424.1
Bacillati Vulcanimicrobiota Vulcanimicrobiia Candidatus Eremiobacterales Candidatus Eremiobacteraceae bacterium isolate SMAG U11875 HYK54201.1
Bacillati Vulcanimicrobiota Vulcanimicrobiia Vulcanimicrobiales Vulcanimicrobiaceae bacterium isolate SPRUCE 46 MGA3036776.1
Bacillati Vulcanimicrobiota Vulcanimicrobiia Vulcanimicrobiales Candidatus Velthaea sp. isolate MFD01689 ilm asm bin22 MGB8265244.1
Bacillati Vulcanimicrobiota Vulcanimicrobiia Vulcanimicrobiales Candidatus Velthaea sp. isolate SPRUCE 268 MGD0471767.1
Bacillati Vulcanimicrobiota Vulcanimicrobiia Vulcanimicrobiales Candidatus Velthaea sp. isolate S3 bin.45.strict MFN2459439.1
Bacillati Vulcanimicrobiota Vulcanimicrobiia Vulcanimicrobiales Candidatus Lustribacter sp. isolate SMAG U7778 HEY5348336.1
Bacillati Vulcanimicrobiota Vulcanimicrobiia Vulcanimicrobiales Candidatus Lustribacter sp. isolate SPRUCE 644 iso2 MGA2392097.1
Bacillati Vulcanimicrobiota Vulcanimicrobiia Vulcanimicrobiales Candidatus Lustribacter sp. isolate SMAG U4421 HEV8023155.1
Bacillati Vulcanimicrobiota Vulcanimicrobiia Vulcanimicrobiales Vulcanimicrobiaceae bacterium isolate SPRUCE 11 MGD1065208.1
Bacillati Vulcanimicrobiota Vulcanimicrobiia Vulcanimicrobiales Vulcanimicrobium alpinum WP 322107852.1
Bacillati Vulcanimicrobiota Vulcanimicrobiia Vulcanimicrobiales Candidatus Elarobacter sp. isolate SMAG U5481 HEX3467881.1
Bacillati Vulcanimicrobiota Vulcanimicrobiia Vulcanimicrobiales Vulcanimicrobiaceae bacterium isolate SMAG U10066 HZO93558.1
Bacillati Vulcanimicrobiota Vulcanimicrobiia Vulcanimicrobiales Candidatus Elarobacter sp. isolate SMAG U6419 iso1 HEV2642161.1
Bacillati Vulcanimicrobiota Vulcanimicrobiia Vulcanimicrobiales Candidatus Elarobacter sp. isolate SMAG U3512 HEY0381998.1
Bacillati Vulcanimicrobiota Vulcanimicrobiia Vulcanimicrobiales Candidatus Elarobacter sp. isolate SMAG U5021 HEY4440975.1
Bacillati Vulcanimicrobiota Vulcanimicrobiia Vulcanimicrobiales Candidatus Elarobacter sp. isolate SMAG U6419 iso2 HEV3089735.1
Bacillati Vulcanimicrobiota Vulcanimicrobiia Vulcanimicrobiales Candidatus Elarobacter sp. isolate SMAG U12605 HTD34221.1
Bacillati Vulcanimicrobiota Vulcanimicrobiia Vulcanimicrobiales Candidatus Elarobacter sp. isolate SMAG U3539 HEY0615653.1
Bacillati Vulcanimicrobiota Vulcanimicrobiia Vulcanimicrobiales Candidatus Elarobacter sp. isolate SMAG U3533 HEY0395302.1
Bacillati Vulcanimicrobiota Vulcanimicrobiia Vulcanimicrobiales Vulcanimicrobiaceae bacterium isolate MFD00329 ilm asm bin1 MFY9779990.1
Bacillati Vulcanimicrobiota Vulcanimicrobiia Vulcanimicrobiales Vulcanimicrobiaceae bacterium isolate SMAG U6574 HEV3156637.1
Bacillati Vulcanimicrobiota Vulcanimicrobiia Vulcanimicrobiales Vulcanimicrobiaceae bacterium isolate TC B2 metabat2.bin.13 sub MGH7709358.1
Bacillati Vulcanimicrobiota Vulcanimicrobiia Vulcanimicrobiales Vulcanimicrobiaceae bacterium isolate SE1534 metabat2.bin.8 MGH7727956.1
Bacillati Vulcanimicrobiota Vulcanimicrobiia Vulcanimicrobiales Vulcanimicrobiaceae bacterium isolate NVS4 Bin5 GAC1629462.1
Bacillati Vulcanimicrobiota Vulcanimicrobiia Vulcanimicrobiales Vulcanimicrobiaceae bacterium isolate Merged NVS Bin21 GAC1303376.1
Bacillati Vulcanimicrobiota Vulcanimicrobiia Vulcanimicrobiales Vulcanimicrobiaceae bacterium isolate NVS1 Bin14 GAC1501287.1
Bacillati Vulcanimicrobiota Vulcanimicrobiia Vulcanimicrobiales Vulcanimicrobiaceae bacterium isolate ML25 006 11 MHB1796763.1
Bacillati Vulcanimicrobiota Vulcanimicrobiia Vulcanimicrobiales Candidatus Rubrimentiphilum sp. isolate SMAG U811 HET9393896.1
Bacillati Vulcanimicrobiota Vulcanimicrobiia Vulcanimicrobiales Candidatus Rubrimentiphilum sp. isolate SMAG U6323 HEV2261812.1
Bacillati Vulcanimicrobiota Vulcanimicrobiia Vulcanimicrobiales Vulcanimicrobiaceae bacterium isolate S3 bin.28.strict MFN2449434.1
Bacillati Vulcanimicrobiota Vulcanimicrobiia Vulcanimicrobiales Vulcanimicrobiaceae bacterium isolate SMAG U7355 HEY9179536.1
Bacillati Vulcanimicrobiota Vulcanimicrobiia Vulcanimicrobiales Vulcanimicrobiaceae bacterium isolate SMAG U5498 HZZ65942.1
Bacillati Vulcanimicrobiota Vulcanimicrobiia Vulcanimicrobiales Vulcanimicrobiaceae bacterium isolate SMAG U9972 HZT13075.1
Bacillati Vulcanimicrobiota Vulcanimicrobiia Vulcanimicrobiales Vulcanimicrobiaceae bacterium isolate SMAG U6566 HEV3155535.1
Bacillati Vulcanimicrobiota Vulcanimicrobiia Vulcanimicrobiales Candidatus Tyrphobacter sp. isolate TC S maxbin2.bin.435 MGH7738254.1
Bacillati Vulcanimicrobiota Vulcanimicrobiia Vulcanimicrobiales Vulcanimicrobiaceae bacterium isolate SMAG U4202 HEV7178407.1
Bacillati Vulcanimicrobiota Vulcanimicrobiia Vulcanimicrobiales Vulcanimicrobiaceae bacterium isolate SEr2D2E2F bin 71 MGR4063967.1
Bacillati Vulcanimicrobiota Vulcanimicrobiia Vulcanimicrobiales Vulcanimicrobiaceae bacterium isolate NR3 009 02 MHB8140386.1
Bacillati Vulcanimicrobiota Vulcanimicrobiia Vulcanimicrobiales Vulcanimicrobiaceae bacterium isolate SMAG U13988 HVA26573.1
Bacillati Vulcanimicrobiota Vulcanimicrobiia Vulcanimicrobiales Vulcanimicrobiaceae bacterium isolate CP BE RX R2 8 MBV8636908.1
Bacillati Vulcanimicrobiota Vulcanimicrobiia Vulcanimicrobiales Candidatus Aquilonibacter sp. isolate SMAG U711 HET9030342.1
Bacillati Vulcanimicrobiota Vulcanimicrobiia Vulcanimicrobiales Candidatus Aquilonibacter sp. isolate SPRUCE 140 MGD0967682.1
Bacillati Vulcanimicrobiota Vulcanimicrobiia Vulcanimicrobiales Candidatus Aquilonibacter sp. isolate MFD10821 ilm asm bin13 MGB6984835.1
Bacillati Vulcanimicrobiota Vulcanimicrobiia Vulcanimicrobiales Candidatus Aquilonibacter sp. isolate MFD06065 ilm asm bin22 MGC2130149.1
Bacillati Vulcanimicrobiota Vulcanimicrobiia Vulcanimicrobiales Candidatus Aquilonibacter sp. isolate MFD02239 ilm asm bin29 MGA7570063.1
Bacillati Vulcanimicrobiota Vulcanimicrobiia Vulcanimicrobiales Vulcanimicrobiaceae bacterium isolate SMAG U4190 HEY8322230.1
Bacillati Vulcanimicrobiota Vulcanimicrobiia Vulcanimicrobiales Vulcanimicrobiaceae bacterium isolate SMAG U5175 HEY1977742.1
Bacillati Vulcanimicrobiota Vulcanimicrobiia Vulcanimicrobiales Vulcanimicrobiaceae bacterium isolate SMAG U13989 HVA34403.1
Bacillati Vulcanimicrobiota Vulcanimicrobiia Vulcanimicrobiales Candidatus Tumulicola sp. isolate MFD09279 ilm asm bin10 MGA8533533.1
Bacillati Vulcanimicrobiota Vulcanimicrobiia Vulcanimicrobiales Candidatus Tumulicola sp. isolate SMAG U7795 HEY5427454.1
Bacillati Vulcanimicrobiota Vulcanimicrobiia Vulcanimicrobiales Candidatus Tumulicola sp. isolate SMAG U8827 HKU82241.1
Bacillati Vulcanimicrobiota Vulcanimicrobiia Vulcanimicrobiales Candidatus Tumulicola sp. isolate SMAG U5095 HEY1654011.1
Bacillati Vulcanimicrobiota Vulcanimicrobiia Vulcanimicrobiales Candidatus Tumulicola sp. isolate SMAG U2546 iso2 HEU5479470.1
Bacillati Vulcanimicrobiota Vulcanimicrobiia Vulcanimicrobiales Candidatus Tumulicola sp. isolate cluster2 bin.114 MEO7203220.1
Bacillati Vulcanimicrobiota Vulcanimicrobiia Vulcanimicrobiales Candidatus Tumulicola sp. isolate SMAG U5027 HEY1427959.1
Bacillati Vulcanimicrobiota Vulcanimicrobiia Vulcanimicrobiales Candidatus Tumulicola sp. isolate SMAG U4806 HEY3674589.1
Bacillati Vulcanimicrobiota Vulcanimicrobiia Vulcanimicrobiales Candidatus Cybelea sp. isolate MFD03947 ilm asm bin18 MGB8964217.1
Bacillati Vulcanimicrobiota Vulcanimicrobiia Vulcanimicrobiales Candidatus Cybelea sp. isolate SMAG U292 HEY6450412.1
Bacillati Vulcanimicrobiota Vulcanimicrobiia Vulcanimicrobiales Candidatus Cybelea sp. isolate SMAG U9760 HLX25962.1
Bacillati Vulcanimicrobiota Vulcanimicrobiia Vulcanimicrobiales Candidatus Cybelea sp. isolate SMAG U5646 HET6275640.1
Bacillati Vulcanimicrobiota Vulcanimicrobiia Vulcanimicrobiales Candidatus Cybelea sp. isolate MFD02469 ilm asm bin11 MGB8151312.1
Bacillati Vulcanimicrobiota Vulcanimicrobiia Vulcanimicrobiales Candidatus Cybelea sp. isolate MFD01145 ilm asm bin51 MFZ0573919.1
Bacillati Vulcanimicrobiota Vulcanimicrobiia Vulcanimicrobiales Candidatus Cybelea sp. isolate MFD00006 ilm asm bin14 MFY9718462.1
Bacillati Vulcanimicrobiota Vulcanimicrobiia Vulcanimicrobiales Candidatus Cybelea sp. isolate SMAG U5315 HEY2474214.1
Bacillati Vulcanimicrobiota Vulcanimicrobiia Vulcanimicrobiales Candidatus Cybelea sp. isolate SMAG U5620 HEX4013722.1
Bacillati Vulcanimicrobiota Vulcanimicrobiia Vulcanimicrobiales Candidatus Cybelea sp. isolate SMAG U5151 HEY1883913.1
Bacillati Vulcanimicrobiota Vulcanimicrobiia Vulcanimicrobiales Candidatus Cybelea sp. isolate MFD00003 ilm asm bin14 MFY9631176.1
Bacillati Vulcanimicrobiota Vulcanimicrobiia Vulcanimicrobiales Candidatus Cybelea sp. isolate SMAG U5462 HEX3368711.1
Bacillati Vulcanimicrobiota Vulcanimicrobiia Vulcanimicrobiales Candidatus Cybelea sp. isolate SMAG U288 HEY6485922.1
Bacteria incertae sedis Candidatus Krumholzibacteriota Candidatus Krumholzibacteriia bacterium isolate Arabian11 MAG 26 MDP6669898.1
Bacteria incertae sedis Candidatus Krumholzibacteriota Candidatus Krumholzibacteriota bacterium isolate Zod Metabat.1031 MBN2171896.1
Bacteria incertae sedis Candidatus Krumholzibacteriota bacterium isolate UBP1 HKST UBA01 MCB1162112.1
Bacteria incertae sedis Candidatus Krumholzibacteriota Candidatus Krumholzibacteriia bacterium isolate SMAG U9246 HKI83996.1
Bacteria incertae sedis Candidatus Krumholzibacteriota Candidatus Krumholzibacteriia bacterium isolate SMAG U9168 HKK70569.1
Bacteria incertae sedis Candidatus Krumholzibacteriota Candidatus Krumholzibacteriia bacterium isolate AMR MDS 3285 HPF35079.1
Bacteria incertae sedis Candidatus Krumholzibacteriota Candidatus Krumholzibacteriia bacterium isolate bin.63 MEE4269724.1
Bacteria incertae sedis Candidatus Krumholzibacteriota Candidatus Krumholzibacteriia bacterium isolate STD1 131 iso1 MDD5718505.1
Bacteria incertae sedis Candidatus Krumholzibacteriota Candidatus Krumholzibacteriia bacterium isolate ABC2.80 MEZ4388496.1
Bacteria incertae sedis Candidatus Krumholzibacteriota Candidatus Krumholzibacteriia bacterium isolate STD1 131 iso2 MDX2474627.1
Bacteria incertae sedis Candidatus Krumholzibacteriota Candidatus Krumholzibacteriia bacterium isolate 3300055726 156 MFT5782875.1
Bacteria incertae sedis Candidatus Krumholzibacteriota Candidatus Krumholzibacteriia bacterium isolate MAG46 MBE0567406.1
Bacteria incertae sedis Candidatus Krumholzibacteriota Candidatus Krumholzibacteriia bacterium isolate CWP5 2.bin.118 MFO7654764.1
Bacteria incertae sedis Candidatus Krumholzibacteriota bacterium isolate OS PC MetaG.mb.70 MBU2502772.1
Bacteria incertae sedis Candidatus Krumholzibacteriota Candidatus Krumholzibacteriota bacterium isolate MAG120 MEN8008565.1
Bacteria incertae sedis Candidatus Krumholzibacteriota Candidatus Krumholzibacteriia bacterium isolate SW 4 19 MCK9994784.1
Bacteria incertae sedis Candidatus Krumholzibacteriota Candidatus Krumholzibacteriia bacterium isolate SMAG U10146 HZM15937.1
Bacteria incertae sedis Candidatus Krumholzibacteriota Candidatus Krumholzibacteriia bacterium isolate D 1 bin.29 MFQ5601370.1
Bacteria incertae sedis Candidatus Krumholzibacteriota Candidatus Krumholzibacteriia bacterium isolate SMAG U11332 HXS10529.1
Bacteria incertae sedis Candidatus Krumholzibacteriota Candidatus Krumholzibacteriia bacterium isolate RS 3 48 MCK5406397.1
Bacteria incertae sedis Candidatus Krumholzibacteriota Candidatus Krumholzibacteriota bacterium isolate CTSoil 085 MBP2680738.1
Bacteria incertae sedis Candidatus Krumholzibacteriota Candidatus Krumholzibacteriia bacterium isolate 13 May SF Bin18 MDH3217170.1
Bacteria incertae sedis Candidatus Krumholzibacteriota Candidatus Krumholzibacteriia bacterium isolate SCS3bin.53 MEE9271082.1
Bacteria incertae sedis Candidatus Krumholzibacteriota Candidatus Krumholzibacteriia bacterium isolate S.4.sp3 bin.33 MFQ5511275.1
Bacteria incertae sedis Candidatus Krumholzibacteriota Candidatus Krumholzibacteriota bacterium isolate Zod Metabat.682 MBN1163999.1
Bacteria incertae sedis Candidatus Krumholzibacteriota Candidatus Krumholzibacteriota bacterium isolate Zod Metabat.1283 MBN1885181.1
Bacteria incertae sedis Candidatus Krumholzibacteriota Candidatus Krumholzibacteriia bacterium isolate RS 17 47 MCK4350488.1
Bacteria incertae sedis Candidatus Krumholzibacteriota Candidatus Krumholzibacteriia bacterium isolate STB 98 MDD4858335.1
Bacteria incertae sedis Candidatus Krumholzibacteriota Candidatus Krumholzibacteriaceae bacterium isolate SMAG U7702 HEY5133373.1
Bacteria incertae sedis Candidatus Krumholzibacteriota Candidatus Krumholzibacteriota bacterium isolate Zod Metabat.1142 MBN2072025.1
Bacteria incertae sedis Candidatus Krumholzibacteriota Candidatus Krumholzibacteriota bacterium isolate Zod Metabat.1015 MBN2184066.1
Bacteria incertae sedis Candidatus Krumholzibacteriota Candidatus Krumholzibacteriia bacterium isolate Prado140 iso2 MCU0640344.1
Bacteria incertae sedis Candidatus Krumholzibacteriota Candidatus Krumholzibacteriia bacterium isolate Prado140 iso1 MCK4549461.1
Bacteria incertae sedis Candidatus Krumholzibacteriota Candidatus Krumholzibacteriota bacterium isolate Salar de Ascotan soil MAG ASO91 MDZ7859619.1
Bacteria incertae sedis Candidatus Krumholzibacteriota bacterium isolate Bin 043 MBD3180360.1
Bacteria incertae sedis Candidatus Eisenbacteria Candidatus Eiseniibacteriota bacterium isolate MP8IB2.79 MDZ4803892.1
Bacteria incertae sedis Candidatus Eisenbacteria Candidatus Eiseniibacteriota bacterium isolate SS bin 28 NNF05884.1
Bacteria incertae sedis Candidatus Eisenbacteria Candidatus Eiseniibacteriota bacterium isolate SMAG U755 HET9233818.1
Bacteria incertae sedis Candidatus Eisenbacteria Candidatus Eiseniibacteriota bacterium isolate Modern marine rock biofilm.mb.36 MBU1702378.1
Bacteria incertae sedis Candidatus Eisenbacteria Candidatus Eiseniibacteriota bacterium isolate Bin 233 MBD3235106.1
Bacteria incertae sedis Candidatus Eisenbacteria Candidatus Eiseniibacteriota bacterium isolate M DeepCast 400m m2 100 MBM3318658.1
Bacteria incertae sedis Candidatus Eisenbacteria Candidatus Eiseniibacteriota bacterium isolate Bin 044 iso1 MCK4412927.1
Bacteria incertae sedis Candidatus Eisenbacteria Candidatus Eiseniibacteriota bacterium isolate MEGAHIT 0.29 MFH1144039.1
Bacteria incertae sedis Candidatus Eisenbacteria Candidatus Eiseniibacteriota bacterium isolate Go SIDig bin 88 MBP7669369.1
Bacteria incertae sedis Candidatus Eisenbacteria Candidatus Eiseniibacteriota bacterium isolate SBS.BIN.67 MFB3909369.1
Bacteria incertae sedis Candidatus Eisenbacteria Candidatus Eiseniibacteriota bacterium isolate K DeepCast 250m m1 333 MBM3287324.1
Bacteria incertae sedis Candidatus Eisenbacteria Candidatus Eiseniibacteriota bacterium isolate Bin 044 iso2 MDM7914102.1
Bacteria incertae sedis Candidatus Eisenbacteria Candidatus Eiseniibacteriota bacterium isolate STL EIS1 MFN8549584.1
Bacteria incertae sedis Candidatus Eisenbacteria Candidatus Eiseniibacteriota bacterium isolate HKST UBA01 MCA9729495.1
Bacteria incertae sedis Candidatus Eisenbacteria Candidatus Eiseniibacteriota bacterium isolate HKST UBA02 MCA9757767.1
Bacteria incertae sedis Candidatus Eisenbacteria Candidatus Eiseniibacteriota bacterium isolate HK STAS EISE 1 MCB9463077.1
Bacteria incertae sedis Candidatus Eisenbacteria Candidatus Eiseniibacteriota bacterium isolate MEGAHIT 0.827 MFH0779158.1
Bacteria incertae sedis Candidatus Eisenbacteria Candidatus Eiseniibacteriota bacterium isolate LacPavin 0920 SED4 MAG 56 28 MCX5800360.1
Bacteria incertae sedis Candidatus Eisenbacteria Candidatus Eiseniibacteriota bacterium isolate Zod Metabat.294 iso2 MBN1503666.1
Bacteria incertae sedis Candidatus Eisenbacteria Candidatus Eiseniibacteriota bacterium isolate bin56 UCF78951.1
Bacteria incertae sedis Candidatus Eisenbacteria Candidatus Eiseniibacteriota bacterium isolate Zod Metabat.294 iso1 HYQ95586.1
Bacteria incertae sedis Candidatus Eisenbacteria Candidatus Eiseniibacteriota bacterium isolate SMAG U2293 HEU4725010.1
Bacteria incertae sedis Candidatus Eisenbacteria Candidatus Eiseniibacteriota bacterium isolate SMAG U2047 HEX7076894.1
Bacteria incertae sedis Candidatus Eisenbacteria Candidatus Eiseniibacteriota bacterium isolate SMAG U7020 HEX3113579.1
Bacteria incertae sedis Candidatus Eisenbacteria Candidatus Eiseniibacteriota bacterium isolate SMAG U754 HET9251083.1
Bacteria incertae sedis Candidatus Eisenbacteria Candidatus Eiseniibacteriota bacterium isolate K DeepCast 150m m1 344 iso2 MEP7028190.1
Bacteria incertae sedis Candidatus Eisenbacteria Candidatus Eiseniibacteriota bacterium isolate M3 S metabat2.bin.149 MGH7726545.1
Bacteria incertae sedis Candidatus Eisenbacteria Candidatus Eiseniibacteriota bacterium isolate cluster1 bin.174 MEO6463161.1
Bacteria incertae sedis Candidatus Eisenbacteria Candidatus Eiseniibacteriota bacterium isolate NC groundwater 1877 Pr3 B 0.1um 71 13 MBI5837646.1
Bacteria incertae sedis Candidatus Eisenbacteria Candidatus Eiseniibacteriota bacterium isolate SMAG U9499 HKA23964.1
Bacteria incertae sedis Candidatus Eisenbacteria Candidatus Eiseniibacteriota bacterium isolate SMAG U6298 HEV2105749.1
Bacteria incertae sedis Candidatus Eisenbacteria Candidatus Eiseniibacteriota bacterium isolate SJ165 MCC6348348.1
Bacteria incertae sedis Candidatus Eisenbacteria Candidatus Eiseniibacteriota bacterium isolate NASQAN2015 150 F bin.10 MEY4069825.1
Bacteria incertae sedis Candidatus Eisenbacteria Candidatus Eiseniibacteriota bacterium isolate EsbW 18 Q3 R4 48 BAT3C.113 MBK7366587.1
Bacteria incertae sedis Candidatus Eisenbacteria Candidatus Eiseniibacteriota bacterium isolate cluster1 bin.339 MEO5987988.1
Bacteria incertae sedis Candidatus Eisenbacteria Candidatus Eiseniibacteriota bacterium isolate SMAG U760 HET9325692.1
Bacteria incertae sedis Candidatus Eisenbacteria Candidatus Eiseniibacteriota bacterium isolate S RSF IL 03 NOT35220.1
Bacteria incertae sedis Candidatus Eisenbacteria Candidatus Eiseniibacteriota bacterium isolate GcL4 3 MFI5371692.1
Bacteria incertae sedis Candidatus Eisenbacteria Candidatus Eiseniibacteriota bacterium isolate WS 8 TMQ63634.1
Bacteria incertae sedis Candidatus Eisenbacteria Candidatus Eiseniibacteriota bacterium isolate SMAG U4620 HEY2954515.1
Bacteria incertae sedis Candidatus Eisenbacteria Candidatus Eiseniibacteriota bacterium isolate SMAG U433 HEY6866494.1
Bacteria incertae sedis Candidatus Eisenbacteria Candidatus Eiseniibacteriota bacterium isolate WS 3 TMQ51377.1
Bacteria incertae sedis Candidatus Eisenbacteria Candidatus Eiseniibacteriota bacterium isolate WS 11 TMQ71634.1
Bacteria incertae sedis Candidatus Eisenbacteria Candidatus Eiseniibacteriota bacterium isolate NC groundwater 928 Pr1 S 0.2um 72 17 MBI3538902.1
Bacteria incertae sedis Candidatus Eisenbacteria Candidatus Eisenbacteria bacterium RBG 16 71 46 OGF18178.1
Bacteria incertae sedis Candidatus Eisenbacteria Candidatus Eiseniibacteriota bacterium isolate SMAG U1088 iso2 HET7225724.1
Bacteria incertae sedis Candidatus Eisenbacteria Candidatus Eiseniibacteriota bacterium isolate NC groundwater 1994 Pr3 S 0.65um 72 3 MBI5710565.1

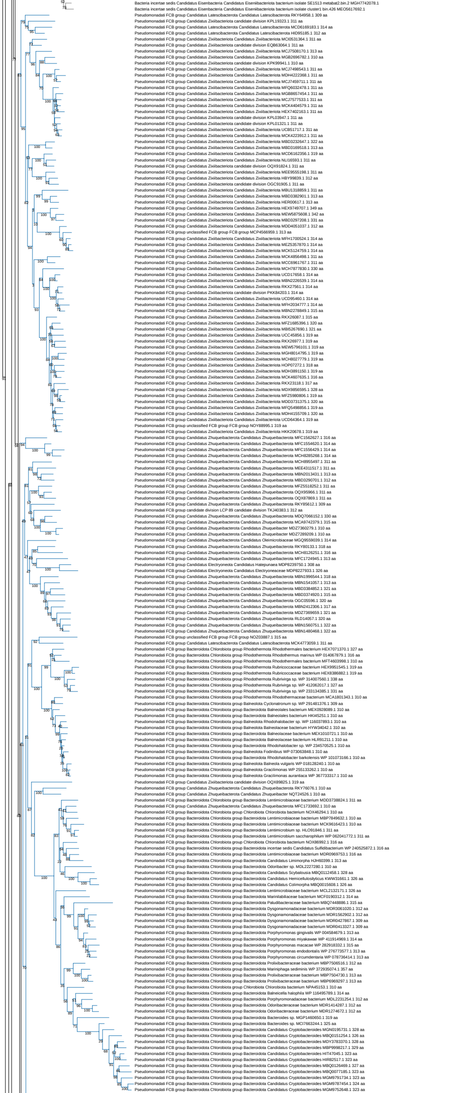
Bacteria incertae sedis Candidatus Eisenbacteria Candidatus Eiseniibacteriota bacterium isolate SE1513 metabat2.bin.2 MGH7742078.1
Bacteria incertae sedis Candidatus Eisenbacteria Candidatus Eiseniibacteriota bacterium isolate cluster1 bin.426 MEO5617692.1
Pseudomonadati FCB group Candidatus Latescibacterota Candidatus Latescibacterota RKY64958.1 309 aa
Pseudomonadati FCB group Candidatus Zixiibacteriota candidate division KPL19323.1 311 aa
Pseudomonadati FCB group Candidatus Latescibacterota Candidatus Latescibacterota MCD6169183.1 314 aa
Pseudomonadati FCB group Candidatus Latescibacterota Candidatus Latescibacterota HID95185.1 312 aa
Pseudomonadati FCB group Candidatus Zixiibacteriota Candidatus Zixiibacteriota MCI0531364.1 311 aa
Pseudomonadati FCB group Candidatus Zixiibacteriota candidate division EQB63064.1 311 aa
Pseudomonadati FCB group Candidatus Zixiibacteriota Candidatus Zixiibacteriota MCJ7508170.1 313 aa
Pseudomonadati FCB group Candidatus Zixiibacteriota Candidatus Zixiibacteriota MGB2696782.1 310 aa
Pseudomonadati FCB group Candidatus Zixiibacteriota candidate division KPK99941.1 310 aa
Pseudomonadati FCB group Candidatus Zixiibacteriota Candidatus Zixiibacteriota MCJ7498543.1 311 aa
Pseudomonadati FCB group Candidatus Zixiibacteriota Candidatus Zixiibacteriota MDH4222368.1 311 aa
Pseudomonadati FCB group Candidatus Zixiibacteriota Candidatus Zixiibacteriota MCJ7459711.1 311 aa
Pseudomonadati FCB group Candidatus Zixiibacteriota Candidatus Zixiibacteriota MFQ6032478.1 311 aa
Pseudomonadati FCB group Candidatus Zixiibacteriota Candidatus Zixiibacteriota MGB8657454.1 311 aa
Pseudomonadati FCB group Candidatus Zixiibacteriota Candidatus Zixiibacteriota MCJ7577533.1 311 aa
Pseudomonadati FCB group Candidatus Zixiibacteriota Candidatus Zixiibacteriota MCK4404579.1 311 aa
Pseudomonadati FCB group Candidatus Zixiibacteriota Candidatus Zixiibacteriota HEX7402163.1 311 aa
Pseudomonadati FCB group Candidatus Zixiibacteriota candidate division KPL03947.1 311 aa
Pseudomonadati FCB group Candidatus Zixiibacteriota candidate division KPL01321.1 311 aa
Pseudomonadati FCB group Candidatus Zixiibacteriota Candidatus Zixiibacteriota UCB51717.1 311 aa
Pseudomonadati FCB group Candidatus Zixiibacteriota Candidatus Zixiibacteriota MCK4223912.1 311 aa
Pseudomonadati FCB group Candidatus Zixiibacteriota Candidatus Zixiibacteriota MBD3232647.1 322 aa
Pseudomonadati FCB group Candidatus Zixiibacteriota Candidatus Zixiibacteriota MBD3169518.1 313 aa
Pseudomonadati FCB group Candidatus Zixiibacteriota Candidatus Zixiibacteriota MCD6162356.1 319 aa
Pseudomonadati FCB group Candidatus Zixiibacteriota Candidatus Zixiibacteriota NLI16593.1 311 aa
Pseudomonadati FCB group Candidatus Zixiibacteriota candidate division OQX91824.1 311 aa
Pseudomonadati FCB group Candidatus Zixiibacteriota Candidatus Zixiibacteriota MEE9555198.1 311 aa
Pseudomonadati FCB group Candidatus Zixiibacteriota Candidatus Zixiibacteriota HBY99839.1 312 aa
Pseudomonadati FCB group Candidatus Zixiibacteriota candidate division OGC91905.1 311 aa
Pseudomonadati FCB group Candidatus Zixiibacteriota Candidatus Zixiibacteriota MBU1318859.1 311 aa
Pseudomonadati FCB group Candidatus Zixiibacteriota Candidatus Zixiibacteriota MBD3382901.1 313 aa
Pseudomonadati FCB group Candidatus Zixiibacteriota Candidatus Zixiibacteriota HER00617.1 313 aa
Pseudomonadati FCB group Candidatus Zixiibacteriota Candidatus Zixiibacteriota HEX9749707.1 349 aa
Pseudomonadati FCB group Candidatus Zixiibacteriota Candidatus Zixiibacteriota MEW5875608.1 342 aa
Pseudomonadati FCB group Candidatus Zixiibacteriota Candidatus Zixiibacteriota MBD3297208.1 331 aa
Pseudomonadati FCB group Candidatus Zixiibacteriota Candidatus Zixiibacteriota MDD4051037.1 312 aa
Pseudomonadati FCB group unclassified FCB group FCB group MCP4566959.1 313 aa
Pseudomonadati FCB group Candidatus Zixiibacteriota Candidatus Zixiibacteriota MFH1700524.1 314 aa
Pseudomonadati FCB group Candidatus Zixiibacteriota Candidatus Zixiibacteriota MEZ5357870.1 314 aa
Pseudomonadati FCB group Candidatus Zixiibacteriota Candidatus Zixiibacteriota MCK5124759.1 314 aa
Pseudomonadati FCB group Candidatus Zixiibacteriota Candidatus Zixiibacteriota MCK4856498.1 311 aa
Pseudomonadati FCB group Candidatus Zixiibacteriota Candidatus Zixiibacteriota MCC6961767.1 311 aa
Pseudomonadati FCB group Candidatus Zixiibacteriota Candidatus Zixiibacteriota MCH7877830.1 330 aa
Pseudomonadati FCB group Candidatus Zixiibacteriota Candidatus Zixiibacteriota UCD17658.1 314 aa
Pseudomonadati FCB group Candidatus Zixiibacteriota Candidatus Zixiibacteriota MBN2226539.1 314 aa
Pseudomonadati FCB group Candidatus Zixiibacteriota Candidatus Zixiibacteriota RKX27561.1 314 aa
Pseudomonadati FCB group Candidatus Zixiibacteriota candidate division PKK84203.1 314 aa
Pseudomonadati FCB group Candidatus Zixiibacteriota Candidatus Zixiibacteriota UCD95460.1 314 aa
Pseudomonadati FCB group Candidatus Zixiibacteriota Candidatus Zixiibacteriota MFH2034777.1 314 aa
Pseudomonadati FCB group Candidatus Zixiibacteriota Candidatus Zixiibacteriota MBN2278849.1 315 aa
Pseudomonadati FCB group Candidatus Zixiibacteriota Candidatus Zixiibacteriota RKX26087.1 315 aa
Pseudomonadati FCB group Candidatus Zixiibacteriota Candidatus Zixiibacteriota MFZ1685396.1 320 aa
Pseudomonadati FCB group Candidatus Zixiibacteriota Candidatus Zixiibacteriota MBI5267690.1 321 aa
Pseudomonadati FCB group Candidatus Zixiibacteriota Candidatus Zixiibacteriota UCC45856.1 319 aa
Pseudomonadati FCB group Candidatus Zixiibacteriota Candidatus Zixiibacteriota RKX26977.1 319 aa
Pseudomonadati FCB group Candidatus Zixiibacteriota Candidatus Zixiibacteriota MEW5796101.1 319 aa
Pseudomonadati FCB group Candidatus Zixiibacteriota Candidatus Zixiibacteriota MGH8014795.1 319 aa
Pseudomonadati FCB group Candidatus Zixiibacteriota Candidatus Zixiibacteriota MCH8027779.1 319 aa
Pseudomonadati FCB group Candidatus Zixiibacteriota Candidatus Zixiibacteriota HOP07272.1 318 aa
Pseudomonadati FCB group Candidatus Zixiibacteriota Candidatus Zixiibacteriota MDH3891150.1 319 aa
Pseudomonadati FCB group Candidatus Zixiibacteriota Candidatus Zixiibacteriota MCK4607635.1 316 aa
Pseudomonadati FCB group Candidatus Zixiibacteriota Candidatus Zixiibacteriota RKX23118.1 317 aa
Pseudomonadati FCB group Candidatus Zixiibacteriota Candidatus Zixiibacteriota MDX9856595.1 328 aa
Pseudomonadati FCB group Candidatus Zixiibacteriota Candidatus Zixiibacteriota MFZ5980806.1 319 aa
Pseudomonadati FCB group Candidatus Zixiibacteriota Candidatus Zixiibacteriota MDD3731375.1 320 aa
Pseudomonadati FCB group Candidatus Zixiibacteriota Candidatus Zixiibacteriota MFQ5498856.1 319 aa
Pseudomonadati FCB group Candidatus Zixiibacteriota Candidatus Zixiibacteriota MDH4155709.1 320 aa
Pseudomonadati FCB group Candidatus Zixiibacteriota Candidatus Zixiibacteriota UCD64364.1 319 aa
Pseudomonadati FCB group unclassified FCB group FCB group NOY88995.1 319 aa
Pseudomonadati FCB group Candidatus Zixiibacteriota Candidatus Zixiibacteriota HKK20678.1 319 aa
Pseudomonadati FCB group Candidatus Zhuqueibacterota Candidatus Zhuqueibacterota MFC1562627.1 316 aa
Pseudomonadati FCB group Candidatus Zhuqueibacterota Candidatus Zhuqueibacterota MFC1554620.1 314 aa
Pseudomonadati FCB group Candidatus Zhuqueibacterota Candidatus Zhuqueibacterota MFC1556429.1 314 aa
Pseudomonadati FCB group Candidatus Zhuqueibacterota Candidatus Zhuqueibacterota MCH8285268.1 314 aa
Pseudomonadati FCB group Candidatus Zhuqueibacterota Candidatus Zhuqueibacterota MCH8955497.1 311 aa
Pseudomonadati FCB group Candidatus Zhuqueibacterota Candidatus Zhuqueibacterota MEE4311517.1 311 aa
Pseudomonadati FCB group Candidatus Zhuqueibacterota Candidatus Zhuqueibacterota MBN2013431.1 313 aa
Pseudomonadati FCB group Candidatus Zhuqueibacterota Candidatus Zhuqueibacterota MBD3290701.1 312 aa
Pseudomonadati FCB group Candidatus Zhuqueibacterota Candidatus Zhuqueibacterota MFZ5518252.1 311 aa
Pseudomonadati FCB group Candidatus Zhuqueibacterota Candidatus Zhuqueibacterota OQX95966.1 311 aa
Pseudomonadati FCB group Candidatus Zhuqueibacterota Candidatus Zhuqueibacterota OQX87869.1 311 aa
Pseudomonadati FCB group Candidatus Zhuqueibacterota Candidatus Zhuqueibacterota RKY85612.1 309 aa
Pseudomonadati FCB group candidate division LCP 89 candidate division TKJ40383.1 312 aa
Pseudomonadati FCB group Candidatus Zhuqueibacterota Candidatus Zhuqueibacterota MDQ7066152.1 330 aa
Pseudomonadati FCB group Candidatus Zhuqueibacterota Candidatus Zhuqueibacterota MCA9742379.1 315 aa
Pseudomonadati FCB group Candidatus Zhuqueibacterota Candidatus Zhuqueibacter MDZ7360279.1 310 aa
Pseudomonadati FCB group Candidatus Zhuqueibacterota Candidatus Zhuqueibacter MDZ7289209.1 310 aa
Pseudomonadati FCB group Candidatus Zhuqueibacterota Candidatus Oleimicrobiaceae MGQ9559039.1 314 aa
Pseudomonadati FCB group Candidatus Zhuqueibacterota Candidatus Zhuqueibacterota RKY80133.1 318 aa
Pseudomonadati FCB group Candidatus Zhuqueibacterota Candidatus Zhuqueibacterota MCH8126251.1 316 aa
Pseudomonadati FCB group Candidatus Zhuqueibacterota Candidatus Zhuqueibacterota MFC1724945.1 313 aa
Pseudomonadati FCB group Candidatus Electryoneota Candidatus Hatepunaea MDP8239750.1 308 aa
Pseudomonadati FCB group Candidatus Electryoneota Candidatus Electryoneaceae MDP8227933.1 326 aa
Pseudomonadati FCB group Candidatus Zhuqueibacterota Candidatus Zhuqueibacterota MBN1996544.1 318 aa
Pseudomonadati FCB group Candidatus Zhuqueibacterota Candidatus Zhuqueibacterota MBN1541057.1 313 aa
Pseudomonadati FCB group Candidatus Zhuqueibacterota Candidatus Zhuqueibacterota MBD3384852.1 321 aa
Pseudomonadati FCB group Candidatus Zhuqueibacterota Candidatus Zhuqueibacterota MBD3374920.1 315 aa
Pseudomonadati FCB group Candidatus Zhuqueibacterota Candidatus Zhuqueibacterota OGC05596.1 320 aa
Pseudomonadati FCB group Candidatus Zhuqueibacterota Candidatus Zhuqueibacterota MBN2412306.1 317 aa
Pseudomonadati FCB group Candidatus Zhuqueibacterota Candidatus Zhuqueibacterota MDZ7369659.1 321 aa
Pseudomonadati FCB group Candidatus Zhuqueibacterota Candidatus Zhuqueibacterota RLD14057.1 320 aa
Pseudomonadati FCB group Candidatus Zhuqueibacterota Candidatus Zhuqueibacterota MBN1560751.1 322 aa
Pseudomonadati FCB group Candidatus Zhuqueibacterota Candidatus Zhuqueibacterota MBN1480468.1 322 aa
Pseudomonadati FCB group unclassified FCB group FCB group NOZ03887.1 315 aa
Pseudomonadati FCB group Candidatus Latescibacterota Candidatus Latescibacterota MCK4773059.1 311 aa
Pseudomonadati FCB group Bacteroidota Chlorobiota group Rhodothermota Rhodothermales bacterium HEX7071370.1 327 aa
Pseudomonadati FCB group Bacteroidota Chlorobiota group Rhodothermota Rhodothermus marinus WP 014067879.1 316 aa
Pseudomonadati FCB group Bacteroidota Chlorobiota group Rhodothermota Rhodothermales bacterium MFT4603998.1 310 aa
Pseudomonadati FCB group Bacteroidota Chlorobiota group Rhodothermota Rubricoccaceae bacterium HEX9951545.1 319 aa
Pseudomonadati FCB group Bacteroidota Chlorobiota group Rhodothermota Rubricoccaceae bacterium HEX8386882.1 319 aa
Pseudomonadati FCB group Bacteroidota Chlorobiota group Rhodothermota Rubrivirga sp. WP 314007560.1 338 aa
Pseudomonadati FCB group Bacteroidota Chlorobiota group Rhodothermota Rubrivirga sp. WP 412062017.1 327 aa
Pseudomonadati FCB group Bacteroidota Chlorobiota group Rhodothermota Rubrivirga sp. WP 233134385.1 331 aa
Pseudomonadati FCB group Bacteroidota Chlorobiota group Rhodothermota Rhodothermaceae bacterium MCA1801343.1 310 aa
Pseudomonadati FCB group Bacteroidota Chlorobiota group Balneolota Cyclonatronum sp. WP 291481376.1 309 aa
Pseudomonadati FCB group Bacteroidota Chlorobiota group Bacteroidota Balneolales bacterium MEX0928089.1 310 aa
Pseudomonadati FCB group Bacteroidota Chlorobiota group Bacteroidota Balneolales bacterium HKI45251.1 310 aa
Pseudomonadati FCB group Bacteroidota Chlorobiota group Balneolota Rhodohalobacter sp. WP 116037893.1 310 aa
Pseudomonadati FCB group Bacteroidota Chlorobiota group Balneolota Balneolaceae bacterium HYW34042.1 310 aa
Pseudomonadati FCB group Bacteroidota Chlorobiota group Bacteroidota Balneolaceae bacterium MEX1010721.1 310 aa
Pseudomonadati FCB group Bacteroidota Chlorobiota group Bacteroidota Balneolaceae bacterium HLR91211.1 310 aa
Pseudomonadati FCB group Bacteroidota Chlorobiota group Bacteroidota Rhodohalobacter sp. WP 234570525.1 310 aa
Pseudomonadati FCB group Bacteroidota Chlorobiota group Balneolota Fodinibius WP 073063848.1 310 aa
Pseudomonadati FCB group Bacteroidota Chlorobiota group Bacteroidota Rhodohalobacter barkolensis WP 101073166.1 310 aa
Pseudomonadati FCB group Bacteroidota Chlorobiota group Balneolota Balneola vulgaris WP 018128240.1 310 aa
Pseudomonadati FCB group Bacteroidota Chlorobiota group Balneolota Gracilimonas WP 255133262.1 310 aa
Pseudomonadati FCB group Bacteroidota Chlorobiota group Balneolota Gracilimonas aurantiaca WP 367733317.1 310 aa
Pseudomonadati FCB group Candidatus Zixiibacteriota candidate division OQX89825.1 319 aa
Pseudomonadati FCB group Candidatus Zhuqueibacterota Candidatus Zhuqueibacterota RKY76076.1 310 aa
Pseudomonadati FCB group Candidatus Zhuqueibacterota Candidatus Zhuqueibacter NQT24526.1 310 aa
Pseudomonadati FCB group Bacteroidota Chlorobiota group Bacteroidota Lentimicrobiaceae bacterium MDD3738824.1 311 aa
Pseudomonadati FCB group Candidatus Zhuqueibacterota Candidatus Zhuqueibacterota MFC1733692.1 310 aa
Pseudomonadati FCB group Bacteroidota Chlorobiota group Chlorobiota Chlorobiota bacterium NOX46294.1 310 aa
Pseudomonadati FCB group Bacteroidota Chlorobiota group Bacteroidota Lentimicrobiaceae bacterium MBP7849632.1 310 aa
Pseudomonadati FCB group Bacteroidota Chlorobiota group Bacteroidota Lentimicrobiaceae bacterium MCK9616423.1 310 aa
Pseudomonadati FCB group Bacteroidota Chlorobiota group Bacteroidota Lentimicrobium sp. HLO91846.1 311 aa
Pseudomonadati FCB group Bacteroidota Chlorobiota group Bacteroidota Lentimicrobium saccharophilum WP 062041772.1 311 aa
Pseudomonadati FCB group Bacteroidota Chlorobiota group Chlorobiota Chlorobiota bacterium NOX86992.1 316 aa
Pseudomonadati FCB group Bacteroidota Chlorobiota group Bacteroidota incertae sedis Candidatus Sulfidibacterium WP 240525872.1 316 aa
Pseudomonadati FCB group Bacteroidota Chlorobiota group Bacteroidota Lentimicrobiaceae bacterium MDR0969753.1 316 aa
Pseudomonadati FCB group Bacteroidota Chlorobiota group Bacteroidota Candidatus Limimorpha HJH60399.1 313 aa
Pseudomonadati FCB group Bacteroidota Chlorobiota group Bacteroidota Odoribacter sp. MDL2227280.1 310 aa
Pseudomonadati FCB group Bacteroidota Chlorobiota group Bacteroidota Candidatus Scybalousia MBQ0112458.1 328 aa
Pseudomonadati FCB group Bacteroidota Chlorobiota group Bacteroidota Candidatus Hemicellulosilyticus KWW31661.1 326 aa
Pseudomonadati FCB group Bacteroidota Chlorobiota group Bacteroidota Candidatus Colimorpha MBQ0015608.1 326 aa
Pseudomonadati FCB group Bacteroidota Chlorobiota group Bacteroidota Lentimicrobiaceae bacterium MCL2132171.1 326 aa
Pseudomonadati FCB group Bacteroidota Chlorobiota group Bacteroidota Marinilabiliaceae bacterium MCF0190312.1 314 aa
Pseudomonadati FCB group Bacteroidota Chlorobiota group Bacteroidota Paludibacteraceae bacterium MBQ7448886.1 315 aa
Pseudomonadati FCB group Bacteroidota Chlorobiota group Bacteroidota Dysgonamonadaceae bacterium MDR3061020.1 312 aa
Pseudomonadati FCB group Bacteroidota Chlorobiota group Bacteroidota Dysgonamonadaceae bacterium MDR1562902.1 312 aa
Pseudomonadati FCB group Bacteroidota Chlorobiota group Bacteroidota Dysgonamonadaceae bacterium MDR0427867.1 309 aa
Pseudomonadati FCB group Bacteroidota Chlorobiota group Bacteroidota Dysgonamonadaceae bacterium MDR0413327.1 309 aa
Pseudomonadati FCB group Bacteroidota Chlorobiota group Bacteroidota Porphyromonas gingivalis WP 004584679.1 313 aa
Pseudomonadati FCB group Bacteroidota Chlorobiota group Bacteroidota Porphyromonas miyakawae WP 411914969.1 314 aa
Pseudomonadati FCB group Bacteroidota Chlorobiota group Bacteroidota Porphyromonas macacae WP 282918332.1 315 aa
Pseudomonadati FCB group Bacteroidota Chlorobiota group Bacteroidota Porphyromonas endodontalis WP 276773577.1 313 aa
Pseudomonadati FCB group Bacteroidota Chlorobiota group Bacteroidota Porphyromonas circumdentaria WP 078736414.1 313 aa
Pseudomonadati FCB group Bacteroidota Chlorobiota group Bacteroidota Prolixibacteraceae bacterium MBP7506516.1 312 aa
Pseudomonadati FCB group Bacteroidota Chlorobiota group Bacteroidota Mariniphaga sediminis WP 372935074.1 357 aa
Pseudomonadati FCB group Bacteroidota Chlorobiota group Bacteroidota Prolixibacteraceae bacterium MBP7504730.1 313 aa
Pseudomonadati FCB group Bacteroidota Chlorobiota group Bacteroidota Prolixibacteraceae bacterium MBP6969297.1 313 aa
Pseudomonadati FCB group Bacteroidota Chlorobiota group Chlorobiota Chlorobiota bacterium NPA45153.1 310 aa
Pseudomonadati FCB group Bacteroidota Chlorobiota group Bacteroidota Balneicella halophila WP 116495789.1 314 aa
Pseudomonadati FCB group Bacteroidota Chlorobiota group Bacteroidota Porphyromonadaceae bacterium MDL2231254.1 312 aa
Pseudomonadati FCB group Bacteroidota Chlorobiota group Bacteroidota Odoribacteraceae bacterium MDR1414287.1 312 aa
Pseudomonadati FCB group Bacteroidota Chlorobiota group Bacteroidota Odoribacteraceae bacterium MDR1274672.1 312 aa
Pseudomonadati FCB group Bacteroidota Chlorobiota group Bacteroidota Bacteroides sp. MGP1460650.1 319 aa
Pseudomonadati FCB group Bacteroidota Chlorobiota group Bacteroidota Bacteroides sp. MCI7663244.1 325 aa
Pseudomonadati FCB group Bacteroidota Chlorobiota group Bacteroidota Candidatus Cryptobacteroides MGN0195731.1 328 aa
Pseudomonadati FCB group Bacteroidota Chlorobiota group Bacteroidota Candidatus Cryptobacteroides MBQ0151254.1 326 aa
Pseudomonadati FCB group Bacteroidota Chlorobiota group Bacteroidota Candidatus Cryptobacteroides MDY3783370.1 328 aa
Pseudomonadati FCB group Bacteroidota Chlorobiota group Bacteroidota Candidatus Cryptobacteroides MBP9998217.1 329 aa
Pseudomonadati FCB group Bacteroidota Chlorobiota group Bacteroidota Candidatus Cryptobacteroides HIT47045.1 323 aa
Pseudomonadati FCB group Bacteroidota Chlorobiota group Bacteroidota Candidatus Cryptobacteroides HIR82517.1 323 aa
Pseudomonadati FCB group Bacteroidota Chlorobiota group Bacteroidota Candidatus Cryptobacteroides MBQ0126469.1 327 aa
Pseudomonadati FCB group Bacteroidota Chlorobiota group Bacteroidota Candidatus Cryptobacteroides MBQ0077185.1 323 aa
Pseudomonadati FCB group Bacteroidota Chlorobiota group Bacteroidota Candidatus Cryptobacteroides MGM9791734.1 323 aa
Pseudomonadati FCB group Bacteroidota Chlorobiota group Bacteroidota Candidatus Cryptobacteroides MGM9787454.1 324 aa
Pseudomonadati FCB group Bacteroidota Chlorobiota group Bacteroidota Candidatus Cryptobacteroides MGM9752648.1 323 aa

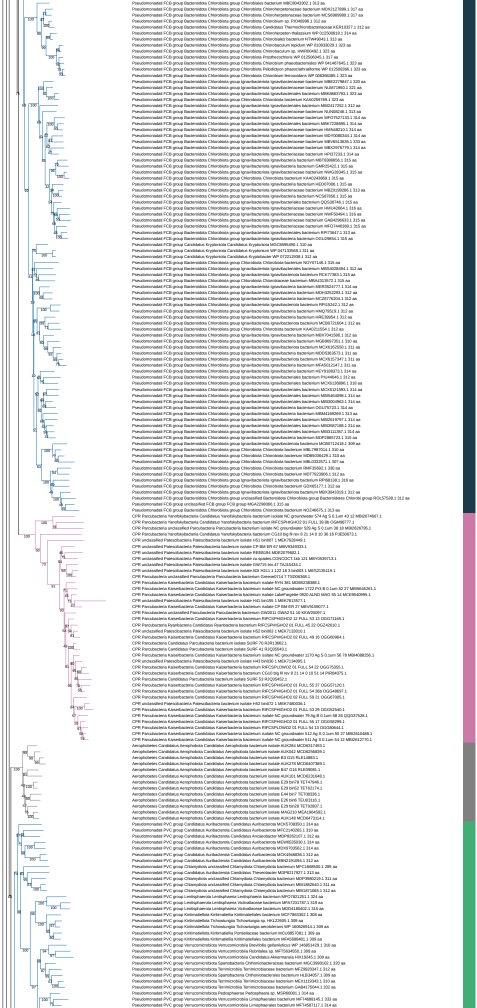
Pseudomonadati FCB group Bacteroidota Chlorobiota group Chlorobiales bacterium MBC8043302.1 313 aa
Pseudomonadati FCB group Bacteroidota Chlorobiota group Chlorobiota Chloroherpetonaceae bacterium MDX2127889.1 317 aa
Pseudomonadati FCB group Bacteroidota Chlorobiota group Chlorobiota Chloroherpetonaceae bacterium MCS6989989.1 317 aa
Pseudomonadati FCB group Bacteroidota Chlorobiota group Chlorobiota Chlorobium sp. PIO48996.1 312 aa
Pseudomonadati FCB group Bacteroidota Chlorobiota group Chlorobiota Candidatus Thermochlorobacteriaceae KER10327.1 312 aa
Pseudomonadati FCB group Bacteroidota Chlorobiota group Chlorobiota Chloroherpeton thalassium WP 012500818.1 314 aa
Pseudomonadati FCB group Bacteroidota Chlorobiota group Chlorobiota Chlorobiales bacterium NTW49043.1 313 aa
Pseudomonadati FCB group Bacteroidota Chlorobiota group Chlorobiota Chlorobaculum tepidum WP 010933029.1 323 aa
Pseudomonadati FCB group Bacteroidota Chlorobiota group Chlorobiota Chlorobaculum sp. HWR00492.1 323 aa
Pseudomonadati FCB group Bacteroidota Chlorobiota group Chlorobiota Prosthecochloris WP 012506045.1 317 aa
Pseudomonadati FCB group Bacteroidota Chlorobiota group Chlorobiota Chlorobium phaeobacteroides WP 041467645.1 323 aa
Pseudomonadati FCB group Bacteroidota Chlorobiota group Chlorobiota Pelodictyon phaeoclathratiforme WP 012508366.1 323 aa
Pseudomonadati FCB group Bacteroidota Chlorobiota group Chlorobiota Chlorobium ferrooxidans WP 006366385.1 323 aa
Pseudomonadati FCB group Bacteroidota Chlorobiota group Ignavibacteriota Ignavibacteriaceae bacterium MBE2279847.1 320 aa
Pseudomonadati FCB group Bacteroidota Chlorobiota group Ignavibacteriota Ignavibacteriaceae bacterium NUM71950.1 321 aa
Pseudomonadati FCB group Bacteroidota Chlorobiota group Ignavibacteriota Ignavibacteriales bacterium MBK8663793.1 323 aa
Pseudomonadati FCB group Bacteroidota Chlorobiota group Chlorobiota Chlorobiota bacterium KAA0259789.1 323 aa
Pseudomonadati FCB group Bacteroidota Chlorobiota group Ignavibacteriota Ignavibacteriales bacterium MBI2417252.1 312 aa
Pseudomonadati FCB group Bacteroidota Chlorobiota group Ignavibacteriota Ignavibacteriaceae bacterium NUN08246.1 313 aa
Pseudomonadati FCB group Bacteroidota Chlorobiota group Ignavibacteriota Ignavibacteriaceae bacterium MFO7527133.1 314 aa
Pseudomonadati FCB group Bacteroidota Chlorobiota group Ignavibacteriota Ignavibacteriales bacterium MBK7228695.1 314 aa
Pseudomonadati FCB group Bacteroidota Chlorobiota group Ignavibacteriota Ignavibacteriaceae bacterium HMN48210.1 314 aa
Pseudomonadati FCB group Bacteroidota Chlorobiota group Ignavibacteriota Ignavibacteriaceae bacterium MDY0080344.1 314 aa
Pseudomonadati FCB group Bacteroidota Chlorobiota group Ignavibacteriota Ignavibacteriaceae bacterium MBV6513635.1 333 aa
Pseudomonadati FCB group Bacteroidota Chlorobiota group Ignavibacteriota Ignavibacteriaceae bacterium MBX2976779.1 314 aa
Pseudomonadati FCB group Bacteroidota Chlorobiota group Ignavibacteriota Ignavibacteriaceae bacterium HPI37233.1 314 aa
Pseudomonadati FCB group Bacteroidota Chlorobiota group Ignavibacteriota Ignavibacteria bacterium MBT8386856.1 315 aa
Pseudomonadati FCB group Bacteroidota Chlorobiota group Ignavibacteriota Ignavibacteria bacterium GMR25422.1 315 aa
Pseudomonadati FCB group Bacteroidota Chlorobiota group Ignavibacteriota Ignavibacteriaceae bacterium NWG28345.1 315 aa
Pseudomonadati FCB group Bacteroidota Chlorobiota group Chlorobiota Chlorobiota bacterium KAA0243869.1 315 aa
Pseudomonadati FCB group Bacteroidota Chlorobiota group Ignavibacteriota Ignavibacteria bacterium HED07006.1 315 aa
Pseudomonadati FCB group Bacteroidota Chlorobiota group Ignavibacteriota Ignavibacteriaceae bacterium MBZ0199286.1 313 aa
Pseudomonadati FCB group Bacteroidota Chlorobiota group Ignavibacteriota Ignavibacteria bacterium NCS87856.1 315 aa
Pseudomonadati FCB group Bacteroidota Chlorobiota group Ignavibacteriota Ignavibacteriales bacterium QQS36746.1 315 aa
Pseudomonadati FCB group Bacteroidota Chlorobiota group Ignavibacteriota Ignavibacteriaceae bacterium HMU42664.1 316 aa
Pseudomonadati FCB group Bacteroidota Chlorobiota group Ignavibacteriota Ignavibacteriaceae bacterium NWF50494.1 316 aa
Pseudomonadati FCB group Bacteroidota Chlorobiota group Ignavibacteriota Ignavibacteriaceae bacterium GAB4296633.1 315 aa
Pseudomonadati FCB group Bacteroidota Chlorobiota group Ignavibacteriota Ignavibacteriaceae bacterium MFO7446369.1 315 aa
Pseudomonadati FCB group Bacteroidota Chlorobiota group Ignavibacteriota Ignavibacteriales bacterium RPI73647.1 313 aa
Pseudomonadati FCB group Bacteroidota Chlorobiota group Ignavibacteriota Ignavibacteria bacterium OGU29854.1 315 aa
Pseudomonadati FCB group Candidatus Kryptoniota Candidatus Kryptoniota MGC8595490.1 310 aa
Pseudomonadati FCB group Candidatus Kryptoniota Candidatus Kryptonium WP 047133568.1 311 aa
Pseudomonadati FCB group Candidatus Kryptoniota Candidatus Kryptobacter WP 072212938.1 312 aa
Pseudomonadati FCB group Bacteroidota Chlorobiota group Chlorobiota Chlorobiota bacterium NOY07146.1 315 aa
Pseudomonadati FCB group Bacteroidota Chlorobiota group Ignavibacteriota Ignavibacteriales bacterium MBS4028494.1 312 aa
Pseudomonadati FCB group Bacteroidota Chlorobiota group Ignavibacteriota Ignavibacteriota bacterium RCK77383.1 315 aa
Pseudomonadati FCB group Bacteroidota Chlorobiota group Chlorobiota Chlorobiaceae bacterium MBA4313572.1 315 aa
Pseudomonadati FCB group Bacteroidota Chlorobiota group Ignavibacteriota Ignavibacteria bacterium MER3524777.1 314 aa
Pseudomonadati FCB group Bacteroidota Chlorobiota group Ignavibacteriota Ignavibacteria bacterium MDH3252293.1 312 aa
Pseudomonadati FCB group Bacteroidota Chlorobiota group Ignavibacteriota Ignavibacteria bacterium MCZ6776204.1 312 aa
Pseudomonadati FCB group Bacteroidota Chlorobiota group Ignavibacteriota Ignavibacteriota bacterium RPI15242.1 312 aa
Pseudomonadati FCB group Bacteroidota Chlorobiota group Ignavibacteriota Ignavibacteria bacterium HMQ79519.1 312 aa
Pseudomonadati FCB group Bacteroidota Chlorobiota group Ignavibacteriota Ignavibacteria bacterium HRE39954.1 312 aa
Pseudomonadati FCB group Bacteroidota Chlorobiota group Ignavibacteriota Ignavibacteriota bacterium MCB0721604.1 312 aa
Pseudomonadati FCB group Bacteroidota Chlorobiota group Chlorobiota Chlorobiota bacterium KAA0211554.1 312 aa
Pseudomonadati FCB group Bacteroidota Chlorobiota group Ignavibacteriota Ignavibacteria bacterium MBX7041586.1 312 aa
Pseudomonadati FCB group Bacteroidota Chlorobiota group Ignavibacteriota Ignavibacteria bacterium MGB9697351.1 310 aa
Pseudomonadati FCB group Bacteroidota Chlorobiota group Ignavibacteriota Ignavibacteriota bacterium MCX6162550.1 311 aa
Pseudomonadati FCB group Bacteroidota Chlorobiota group Ignavibacteriota Ignavibacteria bacterium MDD5363573.1 311 aa
Pseudomonadati FCB group Bacteroidota Chlorobiota group Ignavibacteriota Ignavibacteriota bacterium MCX6157347.1 311 aa
Pseudomonadati FCB group Bacteroidota Chlorobiota group Ignavibacteriota Ignavibacteria bacterium MFA5012147.1 311 aa
Pseudomonadati FCB group Bacteroidota Chlorobiota group Ignavibacteriota Ignavibacteria bacterium HEY9188373.1 314 aa
Pseudomonadati FCB group Bacteroidota Chlorobiota group Ignavibacteriota Ignavibacteriales bacterium PIU44646.1 312 aa
Pseudomonadati FCB group Bacteroidota Chlorobiota group Ignavibacteriota Ignavibacteriales bacterium MCX6136896.1 318 aa
Pseudomonadati FCB group Bacteroidota Chlorobiota group Ignavibacteriota Ignavibacteriales bacterium MCX6121593.1 314 aa
Pseudomonadati FCB group Bacteroidota Chlorobiota group Ignavibacteriota Ignavibacteriales bacterium MBI5464098.1 314 aa
Pseudomonadati FCB group Bacteroidota Chlorobiota group Ignavibacteriota Ignavibacteriales bacterium MBI3004963.1 314 aa
Pseudomonadati FCB group Bacteroidota Chlorobiota group Ignavibacteriota Ignavibacteria bacterium OGU75723.1 314 aa
Pseudomonadati FCB group Bacteroidota Chlorobiota group Ignavibacteriota Ignavibacteria bacterium MBM4169269.1 313 aa
Pseudomonadati FCB group Bacteroidota Chlorobiota group Ignavibacteriota Ignavibacteriales bacterium MBI2619797.1 314 aa
Pseudomonadati FCB group Bacteroidota Chlorobiota group Ignavibacteriota Ignavibacteriales bacterium MBI3587188.1 314 aa
Pseudomonadati FCB group Bacteroidota Chlorobiota group Ignavibacteriota Ignavibacteriales bacterium MBI3111357.1 314 aa
Pseudomonadati FCB group Bacteroidota Chlorobiota group Ignavibacteriota Ignavibacteria bacterium MDP2885723.1 315 aa
Pseudomonadati FCB group Bacteroidota Chlorobiota group Ignavibacteriota Ignavibacteriota bacterium MCB0712418.1 309 aa
Pseudomonadati FCB group Bacteroidota Chlorobiota group Chlorobiota Chlorobiota bacterium MBL7987014.1 310 aa
Pseudomonadati FCB group Bacteroidota Chlorobiota group Chlorobiota Chlorobiota bacterium MDB5036429.1 310 aa
Pseudomonadati FCB group Bacteroidota Chlorobiota group Chlorobiota Chlorobiota bacterium MBL0332571.1 307 aa
Pseudomonadati FCB group Bacteroidota Chlorobiota group Chlorobiota Chlorobiota bacterium RMF35692.1 330 aa
Pseudomonadati FCB group Bacteroidota Chlorobiota group Chlorobiota Chlorobiota bacterium MDT7923906.1 312 aa
Pseudomonadati FCB group Bacteroidota Chlorobiota group Ignavibacteriota Ignavibacteriota bacterium RPI68138.1 316 aa
Pseudomonadati FCB group Bacteroidota Chlorobiota group Chlorobiota Chlorobiota bacterium GDX65177.1 312 aa
Pseudomonadati FCB group Bacteroidota Chlorobiota group Ignavibacteriota Ignavibacteriota bacterium MBX3043319.1 312 aa
Pseudomonadati FCB group Bacteroidota Chlorobiota group unclassified Bacteroidota Chlorobiota group Bacteroidetes Chlorobi group ROL57538.1 312 aa
Pseudomonadati FCB group unclassified FCB group FCB group MGA2298066.1 315 aa
Pseudomonadati FCB group Bacteroidota Chlorobiota group Chlorobiota Chlorobiota bacterium NOZ46675.1 313 aa
CPR Parcubacteria Yanofskybacteria Candidatus Yanofskybacteria bacterium isolate NC groundwater 574 Ag S 0.1um 43 12 MBI2674667.1
CPR Parcubacteria Yanofskybacteria Candidatus Yanofskybacteria bacterium RIFCSPHIGHO2 01 FULL 39 8b OGM98777.1
CPR Parcubacteria unclassified Parcubacteria Parcubacteria bacterium isolate NC groundwater 529 Ag S 0.1um 38 18 MBI2626795.1
CPR Parcubacteria Yanofskybacteria Candidatus Yanofskybacteria bacterium CG10 big fil rev 8 21 14 0 10 36 16 PJE50673.1
CPR unclassified Patescibacteria Patescibacteria bacterium isolate H51 bin007 1 MEK7628449.1
CPR unclassified Patescibacteria Patescibacteria bacterium isolate CP BM ER 67 MBV9349333.1
CPR unclassified Patescibacteria Patescibacteria bacterium isolate REEB194 MDE2079602.1
CPR unclassified Patescibacteria Patescibacteria bacterium isolate co.spades.CONCOCT.1kb 121 MBY0539713.1
CPR unclassified Patescibacteria Patescibacteria bacterium isolate GW715 bin.47 TAJ15434.1
CPR unclassified Patescibacteria Patescibacteria bacterium isolate ADI H2L1 1 122 18 3 bin003 1 MES2135119.1
CPR Parcubacteria unclassified Parcubacteria Parcubacteria bacterium Greene0714 7 TSD06368.1
CPR Parcubacteria Kaiserbacteria Candidatus Kaiserbacteria bacterium isolate RYN 381 MDB5238388.1
CPR Parcubacteria Kaiserbacteria Candidatus Kaiserbacteria bacterium isolate NC groundwater 1722 Pr3 B 0.1um 52 27 MBI5645261.1
CPR Parcubacteria Kaiserbacteria Candidatus Kaiserbacteria bacterium isolate LakeFargette 0920 ALND MAG 55 14 MCE9540995.1
CPR unclassified Patescibacteria Patescibacteria bacterium isolate H41 bin155 1 MEK7613577.1
CPR Parcubacteria Kaiserbacteria Candidatus Kaiserbacteria bacterium isolate CP BM ER 27 MBV9159077.1
CPR Parcubacteria unclassified Parcubacteria Parcubacteria bacterium GW2011 GWA2 51 10 KKW20097.1
CPR Parcubacteria Kaiserbacteria Candidatus Kaiserbacteria bacterium RIFCSPHIGHO2 12 FULL 53 13 OGG71165.1
CPR Parcubacteria Ryanbacteria Candidatus Ryanbacteria bacterium RIFCSPHIGHO2 01 FULL 45 22 OGZ42610.1
CPR unclassified Patescibacteria Patescibacteria bacterium isolate H52 bin063 1 MEK7133010.1
CPR Parcubacteria Kaiserbacteria Candidatus Kaiserbacteria bacterium RIFCSPHIGHO2 02 FULL 49 16 OGG60964.1
CPR Parcubacteria Candidatus Parcubacteria bacterium isolate SURF 70 RJR13662.1
CPR Parcubacteria Candidatus Parcubacteria bacterium isolate SURF 41 RJQ35043.1
CPR Parcubacteria Kaiserbacteria Candidatus Kaiserbacteria bacterium isolate NC groundwater 1170 Ag S 0.1um 56 78 MBI4088256.1
CPR unclassified Patescibacteria Patescibacteria bacterium isolate H43 bin030 1 MEK7134095.1
CPR Parcubacteria Kaiserbacteria Candidatus Kaiserbacteria bacterium RIFCSPLOWO2 01 FULL 54 22 OGG75355.1
CPR Parcubacteria Kaiserbacteria Candidatus Kaiserbacteria bacterium CG10 big fil rev 8 21 14 0 10 51 14 PIR84075.1
CPR Parcubacteria Candidatus Parcubacteria bacterium isolate SURF 53 RJQ35452.1
CPR Parcubacteria Kaiserbacteria Candidatus Kaiserbacteria bacterium RIFCSPHIGHO2 01 FULL 55 37 OGG57120.1
CPR Parcubacteria Kaiserbacteria Candidatus Kaiserbacteria bacterium RIFCSPHIGHO2 01 FULL 54 36b OGG48697.1
CPR Parcubacteria Kaiserbacteria Candidatus Kaiserbacteria bacterium RIFCSPHIGHO2 02 FULL 59 21 OGG67005.1
CPR unclassified Patescibacteria Patescibacteria bacterium isolate H52 bin072 1 MEK7480036.1
CPR Parcubacteria Kaiserbacteria Candidatus Kaiserbacteria bacterium RIFCSPHIGHO2 01 FULL 53 29 OGG52540.1
CPR Parcubacteria Kaiserbacteria Candidatus Kaiserbacteria bacterium isolate NC groundwater 79 Ag B 0.1um 58 26 QQG37528.1
CPR Parcubacteria Kaiserbacteria Candidatus Kaiserbacteria bacterium RIFCSPHIGHO2 01 FULL 55 17 OGG58299.1
CPR Parcubacteria Kaiserbacteria Candidatus Kaiserbacteria bacterium RIFCSPLOWO2 01 FULL 54 13 OGG80644.1
CPR Parcubacteria Kaiserbacteria Candidatus Kaiserbacteria bacterium isolate NC groundwater 512 Ag S 0.1um 55 27 MBI2610488.1
CPR Parcubacteria Kaiserbacteria Candidatus Kaiserbacteria bacterium isolate NC groundwater 511 Ag S 0.1um 54 12 MBI2612770.1
Aerophobetes Candidatus Aerophobota Candidatus Aerophobota bacterium isolate AUK284 MCD6317493.1
Aerophobetes Candidatus Aerophobota Candidatus Aerophobota bacterium isolate AUK042 MCD6256839.1
Aerophobetes Candidatus Aerophobota Candidatus Aerophobota bacterium isolate B3 G15 RLE14883.1
Aerophobetes Candidatus Aerophobota Candidatus Aerophobota bacterium isolate AUK278 MCD6407389.1
Aerophobetes Candidatus Aerophobota Candidatus Aerophobota bacterium isolate B47 G16 RLE09081.1
Aerophobetes Candidatus Aerophobota Candidatus Aerophobota bacterium isolate AUK101 MCD6231648.1
Aerophobetes Candidatus Aerophobota Candidatus Aerophobota bacterium isolate E29 bin78 TET47946.1
Aerophobetes Candidatus Aerophobota Candidatus Aerophobota bacterium isolate E29 bin52 TET62174.1
Aerophobetes Candidatus Aerophobota Candidatus Aerophobota bacterium isolate E44 bin7 TET08336.1
Aerophobetes Candidatus Aerophobota Candidatus Aerophobota bacterium isolate E26 bin6 TEU03116.1
Aerophobetes Candidatus Aerophobota Candidatus Aerophobota bacterium isolate E29 bin28 TET92807.1
Aerophobetes Candidatus Aerophobota Candidatus Aerophobota bacterium isolate MAG210 MEA1964592.1
Aerophobetes Candidatus Aerophobota Candidatus Aerophobota bacterium isolate AUK148 MCD6473114.1
Pseudomonadati PVC group Candidatus Auribacterota Candidatus Auribacterota MCK5708350.1 314 aa
Pseudomonadati PVC group Candidatus Auribacterota Candidatus Auribacterota MFC2140265.1 310 aa
Pseudomonadati PVC group Candidatus Auribacterota Candidatus Ancaeobacter MDP8262107.1 312 aa
Pseudomonadati PVC group Candidatus Auribacterota Candidatus Auribacterota MEW6535030.1 314 aa
Pseudomonadati PVC group Candidatus Auribacterota Candidatus Auribacterota MDX9703562.1 314 aa
Pseudomonadati PVC group Candidatus Auribacterota Candidatus Auribacterota MCK4946836.1 312 aa
Pseudomonadati PVC group Candidatus Auribacterota Candidatus Auribacterota MBN2191094.1 312 aa
Pseudomonadati PVC group Chlamydiota unclassified Chlamydiota Chlamydiota bacterium MFC1668500.1 289 aa
Pseudomonadati PVC group Candidatus Auribacterota Candidatus Theseobacter MDP8217927.1 313 aa
Pseudomonadati PVC group Chlamydiota unclassified Chlamydiota Chlamydiota bacterium MDP3980219.1 311 aa
Pseudomonadati PVC group Chlamydiota unclassified Chlamydiota Chlamydiota bacterium MBI1882640.1 311 aa
Pseudomonadati PVC group Chlamydiota unclassified Chlamydiota Chlamydiota bacterium MBI1871065.1 312 aa
Pseudomonadati PVC group Lentisphaerota Lentisphaeria Lentisphaeria bacterium MFO7821251.1 324 aa
Pseudomonadati PVC group Lentisphaerota Lentisphaeria Victivallaceae bacterium MFA7231787.1 319 aa
Pseudomonadati PVC group Lentisphaerota Lentisphaeria Victivallaceae bacterium MDD4180402.1 315 aa
Pseudomonadati PVC group Kiritimatiellota Kiritimatiellia Kiritimatiellales bacterium MCF7863302.1 308 aa
Pseudomonadati PVC group Kiritimatiellota Tichowtungiia Tichowtungia sp. HKL22605.1 309 aa
Pseudomonadati PVC group Kiritimatiellota Tichowtungiia Tichowtungia aerotolerans WP 160626914.1 309 aa
Pseudomonadati PVC group Kiritimatiellota Kiritimatiellia Pontiellaceae bacterium MCU0857081.1 309 aa
Pseudomonadati PVC group Kiritimatiellota Kiritimatiellia Kiritimatiellales bacterium MFA5688481.1 309 aa
Pseudomonadati PVC group Verrucomicrobiota Verrucomicrobiia Brevifollis gellanilyticus WP 146851429.1 310 aa
Pseudomonadati PVC group Verrucomicrobiota Verrucomicrobiia Rubritalea sp. MFT5634550.1 309 aa
Pseudomonadati PVC group Verrucomicrobiota Verrucomicrobiia Candidatus Akkermansia HIX19245.1 309 aa
Pseudomonadati PVC group Verrucomicrobiota Spartobacteria Chthoniobacteraceae bacterium MGC3990102.1 320 aa
Pseudomonadati PVC group Verrucomicrobiota Terrimicrobiia Terrimicrobiaceae bacterium MFZ9920347.1 312 aa
Pseudomonadati PVC group Verrucomicrobiota Spartobacteria Chthoniobacterales bacterium HLB34057.1 309 aa
Pseudomonadati PVC group Verrucomicrobiota Terrimicrobiia Terrimicrobiaceae bacterium MEX1119343.1 310 aa
Pseudomonadati PVC group Verrucomicrobiota Terrimicrobiia Terrimicrobiaceae bacterium GAB4175044.1 332 aa
Pseudomonadati PVC group Verrucomicrobiota Pedosphaerae Pedosphaera sp. MSR66081.1 314 aa
Pseudomonadati PVC group Verrucomicrobiota Verrucomicrobiia Limisphaerales bacterium MFT4688145.1 333 aa
Pseudomonadati PVC group Verrucomicrobiota Verrucomicrobiia Limisphaerales bacterium MFT4587117.1 314 aa

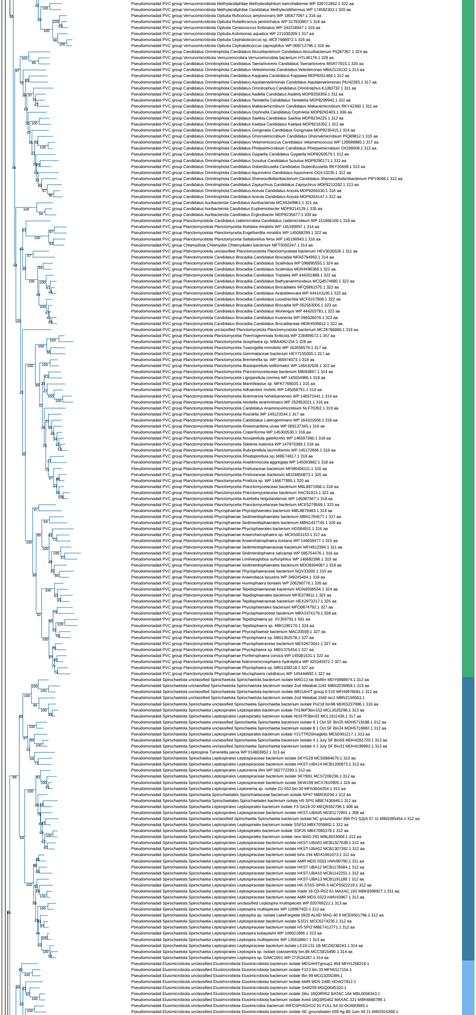

Pseudomonadati PVC group Verrucomicrobiota Methylacidiphilae Methylacidiphilum kamchatkense WP 039721852.1 322 aa
Pseudomonadati PVC group Verrucomicrobiota Methylacidiphilae Candidatus Methylacidithermus WP 174582303.1 320 aa
Pseudomonadati PVC group Verrucomicrobiota Opitutia Ruficoccus amylovorans WP 185677097.1 316 aa
Pseudomonadati PVC group Verrucomicrobiota Opitutia Rubellicoccus peritrichatus WP 317833607.1 316 aa
Pseudomonadati PVC group Verrucomicrobiota Opitutia Cerasicoccus fimbriatus WP 343218947.1 316 aa
Pseudomonadati PVC group Verrucomicrobiota Opitutia Actomonas aquatica WP 221030209.1 317 aa
Pseudomonadati PVC group Verrucomicrobiota Opitutia Cephaloticoccus sp. MCF7688972.1 316 aa
Pseudomonadati PVC group Verrucomicrobiota Opitutia Cephaloticoccus capnophilus WP 068712796.1 316 aa
Pseudomonadati PVC group Candidatus Omnitrophota Candidatus Abzuiibacterium Candidatus Abzuiibacterium PIQ87367.1 324 aa
Pseudomonadati PVC group Verrucomicrobiota Verrucomicrobiia Verrucomicrobiia bacterium HTL48176.1 329 aa
Pseudomonadati PVC group Candidatus Omnitrophota Candidatus Taenariivivens Candidatus Taenariivivens MSR77815.1 320 aa
Pseudomonadati PVC group Candidatus Omnitrophota Candidatus Velesiimonas Candidatus Velesiimonas MBA2124132.1 313 aa
Pseudomonadati PVC group Candidatus Omnitrophota Candidatus Kappaea Candidatus Kappaea MDP8261468.1 312 aa
Pseudomonadati PVC group Candidatus Omnitrophota Candidatus Aquitaenariimonas Candidatus Aquitaenariimonas PIU42265.1 317 aa
Pseudomonadati PVC group Candidatus Omnitrophota Candidatus Omnitrophus Candidatus Omnitrophus KJJ83732.1 311 aa
Pseudomonadati PVC group Candidatus Omnitrophota Candidatus Aadella Candidatus Aadella MDP8259359.1 311 aa
Pseudomonadati PVC group Candidatus Omnitrophota Candidatus Tantalella Candidatus Tantalella MDP8299942.1 311 aa
Pseudomonadati PVC group Candidatus Omnitrophota Candidatus Makaraimicrobium Candidatus Makaraimicrobium RKY42360.1 311 aa
Pseudomonadati PVC group Candidatus Omnitrophota Candidatus Orphnella Candidatus Orphnella MDP8292403.1 336 aa
Pseudomonadati PVC group Candidatus Omnitrophota Candidatus Saelkia Candidatus Saelkia MDP8234225.1 312 aa
Pseudomonadati PVC group Candidatus Omnitrophota Candidatus Kaelpia Candidatus Kaelpia MDP8216352.1 312 aa
Pseudomonadati PVC group Candidatus Omnitrophota Candidatus Gorgyraea Candidatus Gorgyraea MDP8230415.1 314 aa
Pseudomonadati PVC group Candidatus Omnitrophota Candidatus Ghiorseimicrobium Candidatus Ghiorseimicrobium PIQ89812.1 315 aa
Pseudomonadati PVC group Candidatus Omnitrophota Candidatus Velamenicoccus Candidatus Velamenicoccus WP 128698980.1 317 aa
Pseudomonadati PVC group Candidatus Omnitrophota Candidatus Phelpsiimicrobium Candidatus Phelpsiimicrobium OIO36009.1 312 aa
Pseudomonadati PVC group Candidatus Omnitrophota Candidatus Gygaella Candidatus Gygaella MDP8260579.1 312 aa
Pseudomonadati PVC group Candidatus Omnitrophota Candidatus Susulua Candidatus Susulua MDP8290171.1 312 aa
Pseudomonadati PVC group Candidatus Omnitrophota Candidatus Duberdicusella Candidatus Duberdicusella RKY35609.1 313 aa
Pseudomonadati PVC group Candidatus Omnitrophota Candidatus Aquivivens Candidatus Aquivivens OGX13235.1 312 aa
Pseudomonadati PVC group Candidatus Omnitrophota Candidatus Sherwoodlollariibacterium Candidatus Sherwoodlollariibacterium PIP19068.1 312 aa
Pseudomonadati PVC group Candidatus Omnitrophota Candidatus Zapsychrus Candidatus Zapsychrus MDP8212282.1 313 aa
Pseudomonadati PVC group Candidatus Omnitrophota Candidatus Aceula Candidatus Aceula MDP8266330.1 316 aa
Pseudomonadati PVC group Candidatus Omnitrophota Candidatus Aceula Candidatus Aceula MDP8264147.1 312 aa
Pseudomonadati PVC group Candidatus Auribacterota Candidatus Auribacterota MCX6349961.1 321 aa
Pseudomonadati PVC group Candidatus Auribacterota Candidatus Euphemobacter MDP8214129.1 335 aa
Pseudomonadati PVC group Candidatus Auribacterota Candidatus Erginobacter MDP8235677.1 339 aa
Pseudomonadati PVC group Planctomycetota Candidatus Uabimicrobiia Candidatus Uabimicrobium WP 151966100.1 318 aa
Pseudomonadati PVC group Planctomycetota Planctomycetia Rohdeia mirabilis WP 145189997.1 314 aa
Pseudomonadati PVC group Planctomycetota Planctomycetia Engelhardtia mirabilis WP 145068299.1 322 aa
Pseudomonadati PVC group Planctomycetota Planctomycetia Saltatorellus ferox WP 145196943.1 316 aa
Pseudomonadati PVC group Chlamydiota Chlamydiia Chlamydiales bacterium MFT5050247.1 314 aa
Pseudomonadati PVC group Planctomycetota unclassified Planctomycetota Planctomycetota bacterium HEV3026528.1 311 aa
Pseudomonadati PVC group Planctomycetota Candidatus Brocadiia Candidatus Brocadiia MFA5794992.1 314 aa
Pseudomonadati PVC group Planctomycetota Candidatus Brocadiia Candidatus Scalindua WP 096895555.1 324 aa
Pseudomonadati PVC group Planctomycetota Candidatus Brocadiia Candidatus Scalindua MDR4496368.1 322 aa
Pseudomonadati PVC group Planctomycetota Candidatus Brocadiia Candidatus Tripitaka WP 444201888.1 322 aa
Pseudomonadati PVC group Planctomycetota Candidatus Brocadiia Candidatus Bathyanammoxibius MCQ4574680.1 322 aa
Pseudomonadati PVC group Planctomycetota Candidatus Brocadiia Candidatus Brocadiales MFQ5861575.1 322 aa
Pseudomonadati PVC group Planctomycetota Candidatus Brocadiia Candidatus Avalokitesvara WP 444141100.1 322 aa
Pseudomonadati PVC group Planctomycetota Candidatus Brocadiia Candidatus Loosdrechtia MCF6157808.1 322 aa
Pseudomonadati PVC group Planctomycetota Candidatus Brocadiia Candidatus Brocadia WP 052563005.1 323 aa
Pseudomonadati PVC group Planctomycetota Candidatus Brocadiia Candidatus Wunengus WP 444203781.1 322 aa
Pseudomonadati PVC group Planctomycetota Candidatus Brocadiia Candidatus Kuenenia WP 099326076.1 322 aa
Pseudomonadati PVC group Planctomycetota Candidatus Brocadiia Candidatus Brocadiaceae MDR4506812.1 322 aa
Pseudomonadati PVC group Planctomycetota unclassified Planctomycetota Planctomycetota bacterium MCZ6786860.1 319 aa
Pseudomonadati PVC group Planctomycetota Planctomycetia Thermogemmata fonticola WP 228499572.1 357 aa
Pseudomonadati PVC group Planctomycetota Planctomycetia Isosphaera sp. MBA4062154.1 328 aa
Pseudomonadati PVC group Planctomycetota Planctomycetia Tuwongella immobilis WP 162656579.1 317 aa
Pseudomonadati PVC group Planctomycetota Planctomycetia Gemmataceae bacterium HEY7155055.1 317 aa
Pseudomonadati PVC group Planctomycetota Planctomycetia Bremerella sp. WP 366974073.1 318 aa
Pseudomonadati PVC group Planctomycetota Planctomycetia Blastopirellula retiformator WP 146432628.1 322 aa
Pseudomonadati PVC group Planctomycetota Planctomycetia Planctomycetaceae bacterium MBI83867.1 314 aa
Pseudomonadati PVC group Planctomycetota Planctomycetia Lignipirellula cremea WP 145054966.1 318 aa
Pseudomonadati PVC group Planctomycetota Planctomycetia Mariniblastus sp. MFK7766035.1 315 aa
Pseudomonadati PVC group Planctomycetota Planctomycetia Adhaeretor mobilis WP 145058791.1 314 aa
Pseudomonadati PVC group Planctomycetota Planctomycetia Botrimarina hoheduenensis WP 146572441.1 314 aa
Pseudomonadati PVC group Planctomycetota Planctomycetia Aeoliella straminimaris WP 252852021.1 316 aa
Pseudomonadati PVC group Planctomycetota Planctomycetia Candidatus Anammoximicrobium NLF70262.1 319 aa
Pseudomonadati PVC group Planctomycetota Planctomycetia Rosistilla WP 145123344.1 317 aa
Pseudomonadati PVC group Planctomycetota Planctomycetia Candidatus Laterigemmans WP 164101656.1 316 aa
Pseudomonadati PVC group Planctomycetota Planctomycetia Roseimaritima ulvae WP 068137345.1 316 aa
Pseudomonadati PVC group Planctomycetota Planctomycetia Crateriforma WP 145300530.1 316 aa
Pseudomonadati PVC group Planctomycetota Planctomycetia Novipirellula galeiformis WP 146597360.1 316 aa
Pseudomonadati PVC group Planctomycetota Planctomycetia Stieleria maiorica WP 147870369.1 316 aa
Pseudomonadati PVC group Planctomycetota Planctomycetia Rubripirellula lacrimiformis WP 145172666.1 316 aa
Pseudomonadati PVC group Planctomycetota Planctomycetia Rhodopirellula sp. MBE74617.1 316 aa
Pseudomonadati PVC group Planctomycetota Planctomycetia Anatilimnocola aggregata WP 145093862.1 318 aa
Pseudomonadati PVC group Planctomycetota Planctomycetia Pirellulaceae bacterium MFM8406111.1 318 aa
Pseudomonadati PVC group Planctomycetota Planctomycetia Pirellulaceae bacterium MDZ4850873.1 320 aa
Pseudomonadati PVC group Planctomycetota Planctomycetia Pirellula sp. WP 146677895.1 320 aa
Pseudomonadati PVC group Planctomycetota Planctomycetia Planctomycetaceae bacterium MBL8871966.1 318 aa
Pseudomonadati PVC group Planctomycetota Planctomycetia Planctomycetaceae bacterium HAC91813.1 321 aa
Pseudomonadati PVC group Planctomycetota Planctomycetia Aureliella helgolandensis WP 145087567.1 318 aa
Pseudomonadati PVC group Planctomycetota Planctomycetia Planctomycetaceae bacterium MCE5278569.1 323 aa
Pseudomonadati PVC group Planctomycetota Phycisphaerae Phycisphaerales bacterium MBL8879483.1 314 aa
Pseudomonadati PVC group Planctomycetota Phycisphaerae Sedimentisphaerales bacterium MBN1764577.1 317 aa
Pseudomonadati PVC group Planctomycetota Phycisphaerae Sedimentisphaerales bacterium MBN1437745.1 326 aa
Pseudomonadati PVC group Planctomycetota Phycisphaerae Phycisphaerales bacterium HDS84911.1 316 aa
Pseudomonadati PVC group Planctomycetota Phycisphaerae Anaerohalosphaera sp. MCK5001153.1 317 aa
Pseudomonadati PVC group Planctomycetota Phycisphaerae Anaerohalosphaera lusitana WP 146659477.1 315 aa
Pseudomonadati PVC group Planctomycetota Phycisphaerae Sedimentisphaeraceae bacterium MFI4912394.1 311 aa
Pseudomonadati PVC group Planctomycetota Phycisphaerae Sedimentisphaera salicampi WP 085754476.1 316 aa
Pseudomonadati PVC group Planctomycetota Phycisphaerae Limihaloglobus sulfuriphilus WP 146682386.1 315 aa
Pseudomonadati PVC group Planctomycetota Phycisphaerae Sedimentisphaerales bacterium MDO8304087.1 318 aa
Pseudomonadati PVC group Planctomycetota Phycisphaerae Phycisphaeraceae bacterium NQV33200.1 315 aa
Pseudomonadati PVC group Planctomycetota Phycisphaerae Anaerobaca lacustris WP 349245464.1 318 aa
Pseudomonadati PVC group Planctomycetota Phycisphaerae Humisphaera borealis WP 206290776.1 326 aa
Pseudomonadati PVC group Planctomycetota Phycisphaerae Tepidisphaeraceae bacterium MGN6506554.1 324 aa
Pseudomonadati PVC group Planctomycetota Phycisphaerae Tepidisphaerales bacterium MFI5379811.1 322 aa
Pseudomonadati PVC group Planctomycetota Phycisphaerae Tepidisphaeraceae bacterium HEX2973217.1 325 aa
Pseudomonadati PVC group Planctomycetota Phycisphaerae Phycisphaerales bacterium MFO0874792.1 327 aa
Pseudomonadati PVC group Planctomycetota Phycisphaerae Phycisphaeraceae bacterium MBX3374179.1 328 aa
Pseudomonadati PVC group Planctomycetota Phycisphaerae Tepidisphaera sp. XVJ59791.1 581 aa
Pseudomonadati PVC group Planctomycetota Phycisphaerae Tepidisphaera sp. MBI1190170.1 324 aa
Pseudomonadati PVC group Planctomycetota Phycisphaerae Phycisphaerae bacterium MAC20559.1 327 aa
Pseudomonadati PVC group Planctomycetota Phycisphaerae Phycisphaera sp. MBI1302578.1 327 aa
Pseudomonadati PVC group Planctomycetota Phycisphaerae Phycisphaeraceae bacterium MEX2673041.1 327 aa
Pseudomonadati PVC group Planctomycetota Phycisphaerae Phycisphaera sp. MBI1375454.1 327 aa
Pseudomonadati PVC group Planctomycetota Phycisphaerae Poriferisphaera corsica WP 145081531.1 322 aa
Pseudomonadati PVC group Planctomycetota Phycisphaerae Natronomicrosphaera hydrolytica WP 425345972.1 327 aa
Pseudomonadati PVC group Planctomycetota Phycisphaerae Phycisphaera sp. MBI1338218.1 327 aa
Pseudomonadati PVC group Planctomycetota Phycisphaerae Mucisphaera calidilacus WP 145444992.1 327 aa
Pseudomonadati Spirochaetota unclassified Spirochaetota Spirochaetota bacterium isolate MAG13 tar biofilm MDY6968974.1 312 aa
Pseudomonadati Spirochaetota unclassified Spirochaetota Spirochaetota bacterium isolate Zod Metabat.1181 MBN2039858.1 313 aa
Pseudomonadati Spirochaetota unclassified Spirochaetota Spirochaetota bacterium isolate MEGAHIT group 0.510 MFH0976081.1 312 aa
Pseudomonadati Spirochaetota unclassified Spirochaetota Spirochaetota bacterium isolate Zod Metabat.1046 iso1 MBN2159563.1
Pseudomonadati Spirochaetota Spirochaetia unclassified Spirochaetia Spirochaetia bacterium isolate Po218 bin96 MDR3237986.1 316 aa
Pseudomonadati Spirochaetota Spirochaetia Leptospirales Leptospirales bacterium isolate Th196P3bin152 MCL2025296.1 313 aa
Pseudomonadati Spirochaetota Spirochaetia Leptospirales Leptospirales bacterium isolate Nt197P3bin33 MCL1911436.1 317 aa
Pseudomonadati Spirochaetota Spirochaetia unclassified Spirochaetia Spirochaetia bacterium isolate 8 1 Oct SF Bin25 MDH5719188.1 312 aa
Pseudomonadati Spirochaetota Spirochaetia unclassified Spirochaetia Spirochaetia bacterium isolate 8 1 Oct SF Bin24 MDH5716882.1 312 aa
Pseudomonadati Spirochaetota Spirochaetia Leptospirales Leptospirales bacterium isolate Y21TTR20magb6s MES0491217.1 313 aa
Pseudomonadati Spirochaetota Spirochaetia unclassified Spirochaetia Spirochaetia bacterium isolate 4 1 July SF Bin56 MDH4261720.1 313 aa
Pseudomonadati Spirochaetota Spirochaetia unclassified Spirochaetia Spirochaetia bacterium isolate 4 1 July SF Bin31 MDH4199892.1 313 aa
Pseudomonadati Spirochaetota Leptospiria Turneriella parva WP 014803852.1 313 aa
Pseudomonadati Spirochaetota Spirochaetia Leptospirales Leptospiraceae bacterium isolate SKYG28 MCS6984679.1 313 aa
Pseudomonadati Spirochaetota Spirochaetia Leptospirales Leptospiraceae bacterium isolate HKST-UBA14 MCB1200873.1 313 aa
Pseudomonadati Spirochaetota Spirochaetia Leptospirales Leptonema illini WP 002772293.1 312 aa
Pseudomonadati Spirochaetota Spirochaetia Leptospirales Leptospiraceae bacterium isolate SKYB61 MCS7206236.1 312 aa
Pseudomonadati Spirochaetota Spirochaetia Leptospirales Leptospiraceae bacterium isolate SKW199 MCX7810905.1 316 aa
Pseudomonadati Spirochaetota Spirochaetia Leptospirales Leptonema sp. isolate 21I 052.bin.30 MFN3604204.1 312 aa
Pseudomonadati Spirochaetota Spirochaetia Spirochaetales Spirochaetaceae bacterium isolate NP47 MBR30294.1 312 aa
Pseudomonadati Spirochaetota Spirochaetia Spirochaetales Spirochaetales bacterium isolate H5 SPI1 MBE7436846.1 312 aa
Pseudomonadati Spirochaetota Spirochaetia Leptospirales Leptospirales bacterium isolate F3-SA18-20 MEQ9362796.1 308 aa
Pseudomonadati Spirochaetota Spirochaetia Leptospirales Leptospiraceae bacterium isolate HKST-UBA05 MCB1172901.1 308 aa
Pseudomonadati Spirochaetota Spirochaetia unclassified Spirochaetia Spirochaetia bacterium isolate NC groundwater 894 Pr1 S2p5 57 11 MBI3395454.1 312 aa
Pseudomonadati Spirochaetota Spirochaetia Leptospirales Leptospirales bacterium isolate SSF53 MBX7059902.1 312 aa
Pseudomonadati Spirochaetota Spirochaetia Leptospirales Leptospirales bacterium isolate SSF25 MBX7085378.1 312 aa
Pseudomonadati Spirochaetota Spirochaetia Leptospirales Leptospirales bacterium isolate new MAG 292 MBL8019068.1 312 aa
Pseudomonadati Spirochaetota Spirochaetia Leptospirales Leptospiraceae bacterium isolate HKST-UBA03 MCB1327638.1 312 aa
Pseudomonadati Spirochaetota Spirochaetia Leptospirales Leptospiraceae bacterium isolate HKST-UBA02 MCB1307392.1 312 aa
Pseudomonadati Spirochaetota Spirochaetia Leptospirales Leptospiraceae bacterium isolate bins.194 MDX1961073.1 311 aa
Pseudomonadati Spirochaetota Spirochaetia Leptospirales Leptospiraceae bacterium isolate AMR MDS 0353 HMV80790.1 311 aa
Pseudomonadati Spirochaetota Spirochaetia Leptospirales Leptospiraceae bacterium isolate HKST UBA12 MCB1178584.1 312 aa
Pseudomonadati Spirochaetota Spirochaetia Leptospirales Leptospiraceae bacterium isolate HKST-UBA10 MCB1142251.1 312 aa
Pseudomonadati Spirochaetota Spirochaetia Leptospirales Leptospiraceae bacterium isolate HKST-UBA13 MCB1191189.1 312 aa
Pseudomonadati Spirochaetota Spirochaetia Leptospirales Leptospiraceae bacterium isolate HK-STAS-SPIR-5 MCP5502219.1 312 aa
Pseudomonadati Spirochaetota Spirochaetia Leptospirales Leptospiraceae bacterium isolate Hade 18-Q3-R52-61 MAXAC.192 MBK8398927.1 311 aa
Pseudomonadati Spirochaetota Spirochaetia Leptospirales Leptospiraceae bacterium isolate AMR MDS 0322 HMV43967.1 312 aa
Pseudomonadati Spirochaetota Spirochaetia Leptospirales unclassified Leptospira multispecies WP 020769221.1 313 aa
Pseudomonadati Spirochaetota Spirochaetia Leptospirales Leptospira multispecies WP 118967402.1 312 aa
Pseudomonadati Spirochaetota Spirochaetia Leptospirales Leptospira sp. isolate LakeFargette 0920 ALND MAG 40 6 MCE9501796.1 312 aa
Pseudomonadati Spirochaetota Spirochaetia Leptospirales Leptospiraceae bacterium isolate SJ221 MCC6274235.1 312 aa
Pseudomonadati Spirochaetota Spirochaetia Leptospirales Leptospiraceae bacterium isolate H5 SPI2 MBE7412771.1 312 aa
Pseudomonadati Spirochaetota Spirochaetia Leptospirales Leptospira kobayashii WP 109021866.1 313 aa
Pseudomonadati Spirochaetota Spirochaetia Leptospirales Leptospira multispecies WP 135618667.1 313 aa
Pseudomonadati Spirochaetota Spirochaetia Leptospirales Leptospiraceae bacterium isolate LE19 131.1B MCZ8238243.1 314 aa
Pseudomonadati Spirochaetota Spirochaetia Leptospirales Leptospira sp. isolate coassembly bin.86 MCC5815490.1 314 aa
Pseudomonadati Spirochaetota Spirochaetia Leptospirales Leptospira sp. GIMC2001 WP 272534287.1 314 aa
Pseudomonadati Elusimicrobiota unclassified Elusimicrobiota Elusimicrobiota bacterium isolate MEGAHITgroup1.958 MFH1258218.1
Pseudomonadati Elusimicrobiota unclassified Elusimicrobiota Elusimicrobiota bacterium isolate F1F2.bin.33 MFN0117154.1
Pseudomonadati Elusimicrobiota unclassified Elusimicrobiota Elusimicrobiota bacterium isolate Bin 99 MCG3205399.1
Pseudomonadati Elusimicrobiota unclassified Elusimicrobiota Elusimicrobiota bacterium isolate AMR MDS 2485 HOW27812.1
Pseudomonadati Elusimicrobiota unclassified Elusimicrobiota Elusimicrobiota bacterium isolate SAR259 MGQ0645320.1
Pseudomonadati Elusimicrobiota unclassified Elusimicrobiota Elusimicrobiota bacterium isolate Skiv 18Q3R952 BATAC.164 MBL0058343.1
Pseudomonadati Elusimicrobiota unclassified Elusimicrobiota Elusimicrobiota bacterium isolate Aved 18Q3R5462 MAXAC.321 MBK6880799.1
Pseudomonadati Elusimicrobiota unclassified Elusimicrobiota Elusimicrobia bacterium RIFCSPHIGHO2 01 FULL 64 10 OGR83965.1
Pseudomonadati Elusimicrobiota unclassified Elusimicrobiota Elusimicrobiota bacterium isolate NC groundwater 659 Ag B0.1um 49 21 MBI2915368.1

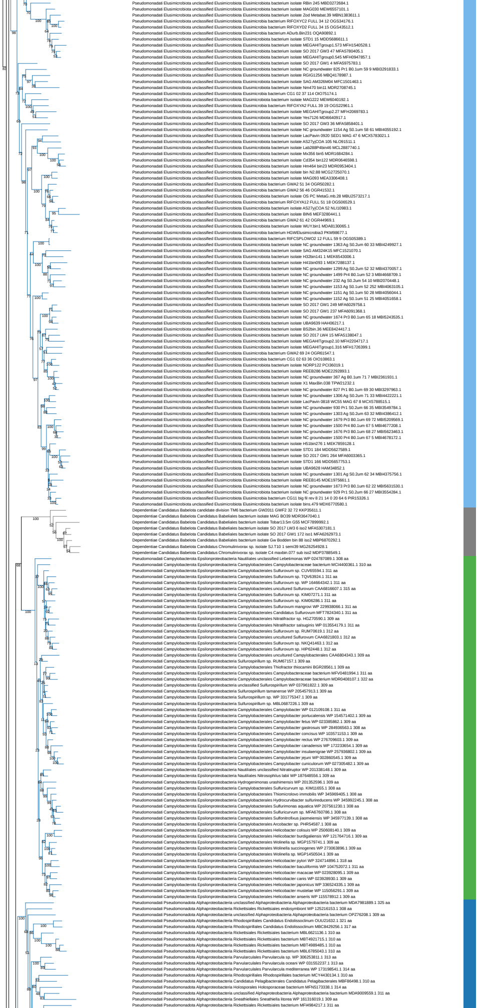
Pseudomonadati Elusimicrobiota unclassified Elusimicrobiota Elusimicrobiota bacterium isolate RBin 245 MBD3272684.1
Pseudomonadati Elusimicrobiota unclassified Elusimicrobiota Elusimicrobiota bacterium isolate MAG030 MEW6557101.1
Pseudomonadati Elusimicrobiota unclassified Elusimicrobiota Elusimicrobiota bacterium isolate Zod Metabat.39 MBN1383611.1
Pseudomonadati Elusimicrobiota unclassified Elusimicrobiota Elusimicrobia bacterium RIFOXYC2 FULL 34 12 OGS34176.1
Pseudomonadati Elusimicrobiota unclassified Elusimicrobiota Elusimicrobia bacterium RIFOXYD2 FULL 34 15 OGS43512.1
Pseudomonadati Elusimicrobiota unclassified Elusimicrobiota Elusimicrobia bacterium ADurb.Bin231 OQA90892.1
Pseudomonadati Elusimicrobiota unclassified Elusimicrobiota Elusimicrobiota bacterium isolate STD1 15 MDD5686611.1
Pseudomonadati Elusimicrobiota unclassified Elusimicrobiota Elusimicrobiota bacterium isolate MEGAHITgroup1.573 MFH1540528.1
Pseudomonadati Elusimicrobiota unclassified Elusimicrobiota Elusimicrobiota bacterium isolate SO 2017 GW3 47 MFA5780405.1
Pseudomonadati Elusimicrobiota unclassified Elusimicrobiota Elusimicrobiota bacterium isolate MEGAHITgroup0.545 MFH0947857.1
Pseudomonadati Elusimicrobiota unclassified Elusimicrobiota Elusimicrobiota bacterium isolate SO 2017 GW1 4 MFA5975783.1
Pseudomonadati Elusimicrobiota unclassified Elusimicrobiota Elusimicrobiota bacterium isolate NC groundwater 825 Pr1 B0.1um 59 9 MBI3291833.1
Pseudomonadati Elusimicrobiota unclassified Elusimicrobiota Elusimicrobiota bacterium isolate RGIG1256 MBQ4178987.1
Pseudomonadati Elusimicrobiota unclassified Elusimicrobiota Elusimicrobiota bacterium isolate SAG AM326M04 MFC1501463.1
Pseudomonadati Elusimicrobiota unclassified Elusimicrobiota Elusimicrobiota bacterium isolate Nm470 bin11 MDR2708745.1
Pseudomonadati Elusimicrobiota unclassified Elusimicrobiota Elusimicrobia bacterium CG1 02 37 114 OIO75174.1
Pseudomonadati Elusimicrobiota unclassified Elusimicrobiota Elusimicrobiota bacterium isolate MAG222 MEW6040192.1
Pseudomonadati Elusimicrobiota unclassified Elusimicrobiota Elusimicrobia bacterium RIFOXYA2 FULL 39 19 OGS22961.1
Pseudomonadati Elusimicrobiota unclassified Elusimicrobiota Elusimicrobiota bacterium isolate MEGAHITgroup2.27 MFH2069783.1
Pseudomonadati Elusimicrobiota unclassified Elusimicrobiota Elusimicrobiota bacterium isolate Yes7126 MDI6640917.1
Pseudomonadati Elusimicrobiota unclassified Elusimicrobiota Elusimicrobiota bacterium isolate SO 2017 GW3 36 MFA5858401.1
Pseudomonadati Elusimicrobiota unclassified Elusimicrobiota Elusimicrobiota bacterium isolate NC groundwater 1154 Ag S0.1um 58 61 MBI4055192.1
Pseudomonadati Elusimicrobiota unclassified Elusimicrobiota Elusimicrobiota bacterium isolate LacPavin 0920 SED1 MAG 47 6 MCX5783021.1
Pseudomonadati Elusimicrobiota unclassified Elusimicrobiota Elusimicrobiota bacterium isolate AS27yjCOA 105 NLO91511.1
Pseudomonadati Elusimicrobiota unclassified Elusimicrobiota Elusimicrobiota bacterium isolate Lab288P4bin46 MCL2887740.1
Pseudomonadati Elusimicrobiota unclassified Elusimicrobiota Elusimicrobiota bacterium isolate Mx356 bin5 MDR1684284.1
Pseudomonadati Elusimicrobiota unclassified Elusimicrobiota Elusimicrobiota bacterium isolate Cd354 bin122 MDR0646598.1
Pseudomonadati Elusimicrobiota unclassified Elusimicrobiota Elusimicrobiota bacterium isolate Hm464 bin23 MDR0953404.1
Pseudomonadati Elusimicrobiota unclassified Elusimicrobiota Elusimicrobiota bacterium isolate bin N2.88 MCG2725070.1
Pseudomonadati Elusimicrobiota unclassified Elusimicrobiota Elusimicrobiota bacterium isolate MAG093 MEA3306408.1
Pseudomonadati Elusimicrobiota unclassified Elusimicrobiota Elusimicrobia bacterium GWA2 51 34 OGR50282.1
Pseudomonadati Elusimicrobiota unclassified Elusimicrobiota Elusimicrobia bacterium GWA2 56 46 OGR41532.1
Pseudomonadati Elusimicrobiota unclassified Elusimicrobiota Elusimicrobiota bacterium isolate OS PC MetaG.mb.28 MBU2573217.1
Pseudomonadati Elusimicrobiota unclassified Elusimicrobiota Elusimicrobia bacterium RIFOXYA12 FULL 51 18 OGS06529.1
Pseudomonadati Elusimicrobiota unclassified Elusimicrobiota Elusimicrobiota bacterium isolate AS27yjCOA 52 NLI10983.1
Pseudomonadati Elusimicrobiota unclassified Elusimicrobiota Elusimicrobiota bacterium isolate BIN6 MEF3280441.1
Pseudomonadati Elusimicrobiota unclassified Elusimicrobiota Elusimicrobia bacterium GWA2 61 42 OGR44969.1
Pseudomonadati Elusimicrobiota unclassified Elusimicrobiota Elusimicrobiota bacterium isolate WUY.bin1 MDA8130065.1
Pseudomonadati Elusimicrobiota unclassified Elusimicrobiota Elusimicrobia bacterium HGWElusimicrobia3 PKM98677.1
Pseudomonadati Elusimicrobiota unclassified Elusimicrobiota Elusimicrobia bacterium RIFCSPLOWO2 12 FULL 59 9 OGS05389.1
Pseudomonadati Elusimicrobiota unclassified Elusimicrobiota Elusimicrobiota bacterium isolate NC groundwater 1363 Ag S0.2um 60 33 MBI4249927.1
Pseudomonadati Elusimicrobiota unclassified Elusimicrobiota Elusimicrobiota bacterium isolate SAG AM324K15 MFC1521070.1
Pseudomonadati Elusimicrobiota unclassified Elusimicrobiota Elusimicrobiota bacterium isolate H32bin141 1 MEK6543006.1
Pseudomonadati Elusimicrobiota unclassified Elusimicrobiota Elusimicrobiota bacterium isolate H41bin093 1 MEK7288137.1
Pseudomonadati Elusimicrobiota unclassified Elusimicrobiota Elusimicrobiota bacterium isolate NC groundwater 1299 Ag S0.2um 52 32 MBI4370057.1
Pseudomonadati Elusimicrobiota unclassified Elusimicrobiota Elusimicrobiota bacterium isolate NC groundwater 1499 Pr4 B0.1um 52 3 MBI4668709.1
Pseudomonadati Elusimicrobiota unclassified Elusimicrobiota Elusimicrobiota bacterium isolate NC groundwater 232 Ag S0.2um 54 10 MBI2070448.1
Pseudomonadati Elusimicrobiota unclassified Elusimicrobiota Elusimicrobiota bacterium isolate NC groundwater 1153 Ag S0.1um 52 252 MBI4063105.1
Pseudomonadati Elusimicrobiota unclassified Elusimicrobiota Elusimicrobiota bacterium isolate NC groundwater 1151 Ag S0.1um 50 28 MBI4056044.1
Pseudomonadati Elusimicrobiota unclassified Elusimicrobiota Elusimicrobiota bacterium isolate NC groundwater 1152 Ag S0.1um 51 25 MBI4051658.1
Pseudomonadati Elusimicrobiota unclassified Elusimicrobiota Elusimicrobiota bacterium isolate SO 2017 GW1 249 MFA6029758.1
Pseudomonadati Elusimicrobiota unclassified Elusimicrobiota Elusimicrobiota bacterium isolate SO 2017 GW1 237 MFA6091368.1
Pseudomonadati Elusimicrobiota unclassified Elusimicrobiota Elusimicrobiota bacterium isolate NC groundwater 1674 Pr3 B0.1um 65 18 MBI5243535.1
Pseudomonadati Elusimicrobiota unclassified Elusimicrobiota Elusimicrobiota bacterium isolate UBA9639 HAH06217.1
Pseudomonadati Elusimicrobiota unclassified Elusimicrobiota Elusimicrobiota bacterium isolate BS2bin.36 MEE8424417.1
Pseudomonadati Elusimicrobiota unclassified Elusimicrobiota Elusimicrobiota bacterium isolate SO 2017 LW4 15 MFA5138047.1
Pseudomonadati Elusimicrobiota unclassified Elusimicrobiota Elusimicrobiota bacterium isolate MEGAHITgroup2.10 MFH2204717.1
Pseudomonadati Elusimicrobiota unclassified Elusimicrobiota Elusimicrobiota bacterium isolate MEGAHITgroup1.316 MFH1726399.1
Pseudomonadati Elusimicrobiota unclassified Elusimicrobiota Elusimicrobia bacterium GWA2 69 24 OGR61547.1
Pseudomonadati Elusimicrobiota unclassified Elusimicrobiota Elusimicrobia bacterium CG1 02 63 36 OIO10863.1
Pseudomonadati Elusimicrobiota unclassified Elusimicrobiota Elusimicrobiota bacterium isolate NORP122 PCI36019.1
Pseudomonadati Elusimicrobiota unclassified Elusimicrobiota Elusimicrobiota bacterium isolate REEB286 MDE2292893.1
Pseudomonadati Elusimicrobiota unclassified Elusimicrobiota Elusimicrobiota bacterium isolate NC groundwater 367 Ag B0.1um 71 7 MBI2361931.1
Pseudomonadati Elusimicrobiota unclassified Elusimicrobiota Elusimicrobiota bacterium isolate X1 MaxBin.038 TPW21232.1
Pseudomonadati Elusimicrobiota unclassified Elusimicrobiota Elusimicrobiota bacterium isolate NC groundwater 827 Pr1 B0.1um 69 30 MBI3297963.1
Pseudomonadati Elusimicrobiota unclassified Elusimicrobiota Elusimicrobiota bacterium isolate NC groundwater 1306 Ag S0.2um 71 33 MBI4422221.1
Pseudomonadati Elusimicrobiota unclassified Elusimicrobiota Elusimicrobiota bacterium isolate LacPavin 0818 WC55 MAG 67 8 MCX5788515.1
Pseudomonadati Elusimicrobiota unclassified Elusimicrobiota Elusimicrobiota bacterium isolate NC groundwater 930 Pr1 S0.2um 66 35 MBI3549784.1
Pseudomonadati Elusimicrobiota unclassified Elusimicrobiota Elusimicrobiota bacterium isolate NC groundwater 1303 Ag S0.2um 63 32 MBI4386412.1
Pseudomonadati Elusimicrobiota unclassified Elusimicrobiota Elusimicrobiota bacterium isolate NC groundwater 1679 Pr3 B0.1um 69 72 MBI5209569.1
Pseudomonadati Elusimicrobiota unclassified Elusimicrobiota Elusimicrobiota bacterium isolate NC groundwater 1500 Pr4 B0.1um 67 5 MBI4677208.1
Pseudomonadati Elusimicrobiota unclassified Elusimicrobiota Elusimicrobiota bacterium isolate NC groundwater 1676 Pr3 B0.1um 68 27 MBI5623463.1
Pseudomonadati Elusimicrobiota unclassified Elusimicrobiota Elusimicrobiota bacterium isolate NC groundwater 1500 Pr4 B0.1um 67 5 MBI4678172.1
Pseudomonadati Elusimicrobiota unclassified Elusimicrobiota Elusimicrobiota bacterium isolate H51bin276 1 MEK7859128.1
Pseudomonadati Elusimicrobiota unclassified Elusimicrobiota Elusimicrobiota bacterium isolate STD1 184 MDD5627589.1
Pseudomonadati Elusimicrobiota unclassified Elusimicrobiota Elusimicrobiota bacterium isolate SO 2017 GW1 264 MFA6003365.1
Pseudomonadati Elusimicrobiota unclassified Elusimicrobiota Elusimicrobiota bacterium isolate STD1 166 MDD5657753.1
Pseudomonadati Elusimicrobiota unclassified Elusimicrobiota Elusimicrobiota bacterium isolate UBA9628 HAM34852.1
Pseudomonadati Elusimicrobiota unclassified Elusimicrobiota Elusimicrobiota bacterium isolate NC groundwater 1301 Ag S0.2um 62 34 MBI4375756.1
Pseudomonadati Elusimicrobiota unclassified Elusimicrobiota Elusimicrobiota bacterium isolate REEB145 MDE1975661.1
Pseudomonadati Elusimicrobiota unclassified Elusimicrobiota Elusimicrobiota bacterium isolate NC groundwater 1673 Pr3 B0.1um 62 22 MBI5631530.1
Pseudomonadati Elusimicrobiota unclassified Elusimicrobiota Elusimicrobiota bacterium isolate NC groundwater 929 Pr1 S0.2um 66 27 MBI3554284.1
Pseudomonadati Elusimicrobiota unclassified Elusimicrobiota Elusimicrobia bacterium CG11 big fil rev 8 21 14 0 20 64 6 PIR15326.1
Pseudomonadati Elusimicrobiota unclassified Elusimicrobiota Elusimicrobiota bacterium isolate bins.479 MDX6770580.1
Dependentiae Candidatus Babelota candidate division TM6 bacterium GW2011 GWF2 32 72 KKP35611.1
Dependentiae Candidatus Babelota Candidatus Babeliales bacterium isolate MAG BO39 MDR3647040.1
Dependentiae Candidatus Babelota Candidatus Babeliales bacterium isolate Tobar13.5m G55 MCF7899992.1
Dependentiae Candidatus Babelota Candidatus Babeliales bacterium isolate SO 2017 LW3 6 iso2 MFA5307181.1
Dependentiae Candidatus Babelota Candidatus Babeliales bacterium isolate SO 2017 GW1 172 iso1 MFA6262973.1
Dependentiae Candidatus Babelota Candidatus Babeliales bacterium isolate Gw Bodden bin 88 iso2 MBP6870292.1
Dependentiae Candidatus Babelota Candidatus Chromulinivorax sp. isolate SJ.T10 1 semi39 MGZ6254928.1
Dependentiae Candidatus Babelota Candidatus Chromulinivorax sp. isolate C4 maxbin.077 sub iso2 MDP3788549.1
Pseudomonadati Campylobacterota Epsilonproteobacteria Nautiliales unclassified Lebetimonas WP 024787089.1 308 aa
Pseudomonadati Campylobacterota Epsilonproteobacteria Campylobacterales Campylobacteraceae bacterium MCI4400361.1 310 aa
Pseudomonadati Campylobacterota Epsilonproteobacteria Campylobacterales Sulfurovum sp. CUV65594.1 311 aa
Pseudomonadati Campylobacterota Epsilonproteobacteria Campylobacterales Sulfurovum sp. TQV63924.1 311 aa
Pseudomonadati Campylobacterota Epsilonproteobacteria Campylobacterales Sulfurovum sp. WP 164664342.1 311 aa
Pseudomonadati Campylobacterota Epsilonproteobacteria Campylobacterales uncultured Sulfurovum CAA6816607.1 315 aa
Pseudomonadati Campylobacterota Epsilonproteobacteria Campylobacterales Sulfurovum sp. KIM07271.1 311 aa
Pseudomonadati Campylobacterota Epsilonproteobacteria Campylobacterales Sulfurovum sp. KIM06286.1 311 aa
Pseudomonadati Campylobacterota Epsilonproteobacteria Campylobacterales Sulfurovum mangrovi WP 229938066.1 311 aa
Pseudomonadati Campylobacterota Epsilonproteobacteria Campylobacterales Candidatus Sulfurovum MFT7824340.1 311 aa
Pseudomonadati Campylobacterota Epsilonproteobacteria Campylobacterales Nitratifractor sp. HGZ70590.1 309 aa
Pseudomonadati Campylobacterota Epsilonproteobacteria Campylobacterales Nitratifractor salsuginis WP 013554179.1 311 aa
Pseudomonadati Campylobacterota Epsilonproteobacteria Campylobacterales Sulfurovum sp. RUM70619.1 312 aa
Pseudomonadati Campylobacterota Epsilonproteobacteria Campylobacterales uncultured Sulfurovum CAA6821803.1 312 aa
Pseudomonadati Campylobacterota Epsilonproteobacteria Campylobacterales Sulfurovum sp. NKQ41463.1 312 aa
Pseudomonadati Campylobacterota Epsilonproteobacteria Campylobacterales Sulfurovum sp. HIP62448.1 312 aa
Pseudomonadati Campylobacterota Epsilonproteobacteria Campylobacterales uncultured Campylobacterales CAA6804343.1 309 aa
Pseudomonadati Campylobacterota Epsilonproteobacteria Sulfurospirillum sp. RUM67157.1 309 aa
Pseudomonadati Campylobacterota Epsilonproteobacteria Campylobacterales Thiofractor thiocamini BGR28561.1 309 aa
Pseudomonadati Campylobacterota Epsilonproteobacteria Campylobacterales Campylobacteraceae bacterium MFV0481994.1 311 aa
Pseudomonadati Campylobacterota Epsilonproteobacteria Campylobacterales Campylobacteraceae bacterium MDR0408107.1 322 aa
Pseudomonadati Campylobacterota Epsilonproteobacteria unclassified Sulfurospirillum WP 037961822.1 309 aa
Pseudomonadati Campylobacterota Epsilonproteobacteria Sulfurospirillum tamanense WP 205457913.1 309 aa
Pseudomonadati Campylobacterota Epsilonproteobacteria Sulfurospirillum sp. WP 331775347.1 309 aa
Pseudomonadati Campylobacterota Epsilonproteobacteria Sulfurospirillum sp. MBL0687226.1 309 aa
Pseudomonadati Campylobacterota Epsilonproteobacteria Campylobacterales Campylobacter WP 012109108.1 311 aa
Pseudomonadati Campylobacterota Epsilonproteobacteria Campylobacterales Campylobacter portucalensis WP 154571402.1 309 aa
Pseudomonadati Campylobacterota Epsilonproteobacteria Campylobacterales Campylobacter fetus WP 023385862.1 309 aa
Pseudomonadati Campylobacterota Epsilonproteobacteria Campylobacterales Campylobacter gastrosuis WP 284936563.1 308 aa
Pseudomonadati Campylobacterota Epsilonproteobacteria Campylobacterales Campylobacter concisus WP 103571153.1 309 aa
Pseudomonadati Campylobacterota Epsilonproteobacteria Campylobacterales Campylobacter rectus WP 276709603.1 309 aa
Pseudomonadati Campylobacterota Epsilonproteobacteria Campylobacterales Campylobacter canadensis WP 172233654.1 309 aa
Pseudomonadati Campylobacterota Epsilonproteobacteria Campylobacterales Campylobacter insulaenigrae WP 257936802.1 309 aa
Pseudomonadati Campylobacterota Epsilonproteobacteria Campylobacterales Campylobacter jejuni WP 002860545.1 309 aa
Pseudomonadati Campylobacterota Epsilonproteobacteria Campylobacterales Campylobacter cuniculorum WP 027305482.1 309 aa
Pseudomonadati Campylobacterota Epsilonproteobacteria Nautiliales unclassified Nitratiruptor WP 201338148.1 309 aa
Pseudomonadati Campylobacterota Epsilonproteobacteria Nautiliales Nitrosophilus labii WP 187648556.1 309 aa
Pseudomonadati Campylobacterota Epsilonproteobacteria Hydrogenimonas urashimensis WP 201352596.1 309 aa
Pseudomonadati Campylobacterota Epsilonproteobacteria Campylobacterales Sulfuricurvum sp. KIM11655.1 308 aa
Pseudomonadati Campylobacterota Epsilonproteobacteria Campylobacterales Thiomicrolovo immobilis WP 345969405.1 308 aa
Pseudomonadati Campylobacterota Epsilonproteobacteria Campylobacterales Hydrocurvibacter sulfurireducens WP 345992245.1 308 aa
Pseudomonadati Campylobacterota Epsilonproteobacteria Campylobacterales Sulfurimonas aquatica WP 207561230.1 308 aa
Pseudomonadati Campylobacterota Epsilonproteobacteria Campylobacterales Sulfuricurvum sp. MFA6760786.1 308 aa
Pseudomonadati Campylobacterota Epsilonproteobacteria Campylobacterales Sulfonitrofixus jiaomeiensis WP 345977139.1 308 aa
Pseudomonadati Campylobacterota Epsilonproteobacteria Campylobacterales Arcobacter sp. PHR54587.1 308 aa
Pseudomonadati Campylobacterota Epsilonproteobacteria Campylobacterales Helicobacter colisuis WP 250608140.1 309 aa
Pseudomonadati Campylobacterota Epsilonproteobacteria Campylobacterales Helicobacter burdigaliensis WP 121764716.1 309 aa
Pseudomonadati Campylobacterota Epsilonproteobacteria Campylobacterales Wolinella sp. MGP1579741.1 309 aa
Pseudomonadati Campylobacterota Epsilonproteobacteria Campylobacterales Wolinella succinogenes WP 273063896.1 309 aa
Pseudomonadati Campylobacterota Epsilonproteobacteria Campylobacterales Wolinella sp. MGP1450504.1 309 aa
Pseudomonadati Campylobacterota Epsilonproteobacteria Campylobacterales Helicobacter pylori WP 324714896.1 318 aa
Pseudomonadati Campylobacterota Epsilonproteobacteria Campylobacterales Helicobacter baculiformis WP 104752072.1 311 aa
Pseudomonadati Campylobacterota Epsilonproteobacteria Campylobacterales Helicobacter macacae WP 023928095.1 309 aa
Pseudomonadati Campylobacterota Epsilonproteobacteria Campylobacterales Helicobacter canis WP 023928930.1 309 aa
Pseudomonadati Campylobacterota Epsilonproteobacteria Campylobacterales Helicobacter japonicus WP 336524335.1 309 aa
Pseudomonadati Campylobacterota Epsilonproteobacteria Campylobacterales Helicobacter mustelae WP 115056291.1 309 aa
Pseudomonadati Campylobacterota Epsilonproteobacteria Campylobacterales Helicobacter anseris WP 115578912.1 309 aa
Pseudomonadati Pseudomonadota Alphaproteobacteria unclassified Alphaproteobacteria Alphaproteobacteria bacterium MDA7981889.1 325 aa
Pseudomonadati Pseudomonadota Alphaproteobacteria Rickettsiales Rickettsiales endosymbiont WP 125216153.1 308 aa
Pseudomonadati Pseudomonadota Alphaproteobacteria unclassified Alphaproteobacteria Alphaproteobacteria bacterium OPZ76208.1 309 aa
Pseudomonadati Pseudomonadota Alphaproteobacteria Rhodospirillales Candidatus Endolissoclinum OUU21632.1 321 aa
Pseudomonadati Pseudomonadota Alphaproteobacteria Rhodospirillales Candidatus Endolissoclinum MBC8429256.1 317 aa
Pseudomonadati Pseudomonadota Alphaproteobacteria Rickettsiales Rickettsiales bacterium MBL6621136.1 310 aa
Pseudomonadati Pseudomonadota Alphaproteobacteria Rickettsiales Rickettsiales bacterium MBT4921715.1 310 aa
Pseudomonadati Pseudomonadota Alphaproteobacteria Rickettsiales Rickettsiales bacterium MBT4989485.1 310 aa
Pseudomonadati Pseudomonadota Alphaproteobacteria Rickettsiales Rickettsiales bacterium MBL6785043.1 310 aa
Pseudomonadati Pseudomonadota Alphaproteobacteria Parvularculales Parvularcula sp. WP 306253811.1 313 aa
Pseudomonadati Pseudomonadota Alphaproteobacteria Parvularculales Parvularcula oceani WP 031552237.1 313 aa
Pseudomonadati Pseudomonadota Alphaproteobacteria Parvularculales Parvularcula mediterranea WP 173198541.1 314 aa
Pseudomonadati Pseudomonadota Alphaproteobacteria Rhodospirillales Rhodospirillales bacterium MCY4430134.1 310 aa
Pseudomonadati Pseudomonadota Alphaproteobacteria Candidatus Pelagibacterales Candidatus Pelagibacterales MBF86498.1 310 aa
Pseudomonadati Pseudomonadota Alphaproteobacteria Holosporales Holosporaceae bacterium MFN5173338.1 314 aa
Pseudomonadati Pseudomonadota Alphaproteobacteria unclassified Alphaproteobacteria Alphaproteobacteria bacterium MDA9009559.1 311 aa
Pseudomonadati Pseudomonadota Alphaproteobacteria Sneathiellales Sneathiella litorea WP 161316019.1 309 aa
Pseudomonadati Pseudomonadota Alphaproteobacteria Rickettsiales Rickettsiales bacterium MFI4984217.1 311 aa

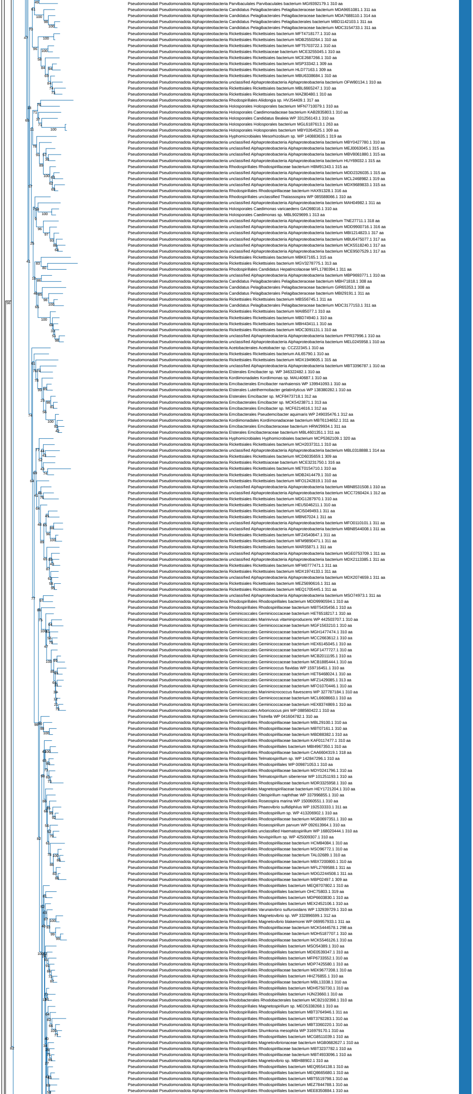

Pseudomonadati Pseudomonadota Alphaproteobacteria Parvibaculales Parvibaculales bacterium MGI9392179.1 310 aa
Pseudomonadati Pseudomonadota Alphaproteobacteria Candidatus Pelagibacterales Pelagibacteraceae bacterium MDA9651081.1 311 aa
Pseudomonadati Pseudomonadota Alphaproteobacteria Candidatus Pelagibacterales Pelagibacteraceae bacterium MDA7688110.1 314 aa
Pseudomonadati Pseudomonadota Alphaproteobacteria Candidatus Pelagibacterales Pelagibacterales bacterium MBD1142103.1 311 aa
Pseudomonadati Pseudomonadota Alphaproteobacteria Candidatus Pelagibacterales Pelagibacteraceae bacterium MDC3154733.1 311 aa
Pseudomonadati Pseudomonadota Alphaproteobacteria Rickettsiales Rickettsiales bacterium MFT4718177.1 310 aa
Pseudomonadati Pseudomonadota Alphaproteobacteria Rickettsiales Rickettsiales bacterium MDB2550264.1 310 aa
Pseudomonadati Pseudomonadota Alphaproteobacteria Rickettsiales Rickettsiales bacterium MFT5703722.1 310 aa
Pseudomonadati Pseudomonadota Alphaproteobacteria Rickettsiales Rickettsiaceae bacterium MCE3255045.1 310 aa
Pseudomonadati Pseudomonadota Alphaproteobacteria Rickettsiales Rickettsiales bacterium MCE2687266.1 310 aa
Pseudomonadati Pseudomonadota Alphaproteobacteria Rickettsiales Rickettsiales bacterium MSP33342.1 309 aa
Pseudomonadati Pseudomonadota Alphaproteobacteria Rickettsiales Rickettsiales bacterium HLD77163.1 309 aa
Pseudomonadati Pseudomonadota Alphaproteobacteria Rickettsiales Rickettsiales bacterium MBU6338684.1 310 aa
Pseudomonadati Pseudomonadota Alphaproteobacteria unclassified Alphaproteobacteria Alphaproteobacteria bacterium OFW80134.1 310 aa
Pseudomonadati Pseudomonadota Alphaproteobacteria Rickettsiales Rickettsiales bacterium MBL6665247.1 310 aa
Pseudomonadati Pseudomonadota Alphaproteobacteria Rickettsiales Rickettsiales bacterium MAZ80480.1 310 aa
Pseudomonadati Pseudomonadota Alphaproteobacteria Rhodospirillales Aliidongia sp. HVJ54409.1 317 aa
Pseudomonadati Pseudomonadota Alphaproteobacteria Holosporales Holosporales bacterium MFN7710079.1 310 aa
Pseudomonadati Pseudomonadota Alphaproteobacteria Holosporales Caedimonadaceae bacterium KAB2835803.1 310 aa
Pseudomonadati Pseudomonadota Alphaproteobacteria Holosporales Candidatus Bealeia WP 331256143.1 310 aa
Pseudomonadati Pseudomonadota Alphaproteobacteria Holosporales Holosporales bacterium MGL6187613.1 263 aa
Pseudomonadati Pseudomonadota Alphaproteobacteria Holosporales Holosporales bacterium MBY0264525.1 309 aa
Pseudomonadati Pseudomonadota Alphaproteobacteria Hyphomicrobiales Mesorhizobium sp. WP 140883635.1 319 aa
Pseudomonadati Pseudomonadota Alphaproteobacteria unclassified Alphaproteobacteria Alphaproteobacteria bacterium MBY0427780.1 310 aa
Pseudomonadati Pseudomonadota Alphaproteobacteria unclassified Alphaproteobacteria Alphaproteobacteria bacterium MEJ0063045.1 315 aa
Pseudomonadati Pseudomonadota Alphaproteobacteria unclassified Alphaproteobacteria Alphaproteobacteria bacterium MBV8061880.1 315 aa
Pseudomonadati Pseudomonadota Alphaproteobacteria unclassified Alphaproteobacteria Alphaproteobacteria bacterium HUY69032.1 315 aa
Pseudomonadati Pseudomonadota Alphaproteobacteria Rhodospirillales Rhodospirillaceae bacterium HBM91343.1 315 aa
Pseudomonadati Pseudomonadota Alphaproteobacteria unclassified Alphaproteobacteria Alphaproteobacteria bacterium MDD2326035.1 315 aa
Pseudomonadati Pseudomonadota Alphaproteobacteria unclassified Alphaproteobacteria Alphaproteobacteria bacterium MCL2468982.1 319 aa
Pseudomonadati Pseudomonadota Alphaproteobacteria unclassified Alphaproteobacteria Alphaproteobacteria bacterium MDX9689833.1 315 aa
Pseudomonadati Pseudomonadota Alphaproteobacteria Rhodospirillales Rhodospirillaceae bacterium HAX91328.1 316 aa
Pseudomonadati Pseudomonadota Alphaproteobacteria Rhodospirillales unclassified Thalassospira WP 085588066.1 310 aa
Pseudomonadati Pseudomonadota Alphaproteobacteria unclassified Alphaproteobacteria Alphaproteobacteria bacterium MAH04982.1 311 aa
Pseudomonadati Pseudomonadota Alphaproteobacteria Holosporales Caedimonas varicaedens GAO98016.1 310 aa
Pseudomonadati Pseudomonadota Alphaproteobacteria Holosporales Caedimonas sp. MBL9029699.1 313 aa
Pseudomonadati Pseudomonadota Alphaproteobacteria unclassified Alphaproteobacteria Alphaproteobacteria bacterium TNE27711.1 318 aa
Pseudomonadati Pseudomonadota Alphaproteobacteria unclassified Alphaproteobacteria Alphaproteobacteria bacterium MDD9900716.1 316 aa
Pseudomonadati Pseudomonadota Alphaproteobacteria unclassified Alphaproteobacteria Alphaproteobacteria bacterium MBI1214823.1 317 aa
Pseudomonadati Pseudomonadota Alphaproteobacteria unclassified Alphaproteobacteria Alphaproteobacteria bacterium MBU6475077.1 317 aa
Pseudomonadati Pseudomonadota Alphaproteobacteria unclassified Alphaproteobacteria Alphaproteobacteria bacterium MCK5518240.1 317 aa
Pseudomonadati Pseudomonadota Alphaproteobacteria unclassified Alphaproteobacteria Alphaproteobacteria bacterium MCE9507529.1 317 aa
Pseudomonadati Pseudomonadota Alphaproteobacteria Rickettsiales Rickettsiales bacterium MBK67165.1 315 aa
Pseudomonadati Pseudomonadota Alphaproteobacteria Rickettsiales Rickettsiales bacterium MGV3278775.1 313 aa
Pseudomonadati Pseudomonadota Alphaproteobacteria Rhodospirillales Candidatus Hepatincolaceae MFL1780394.1 311 aa
Pseudomonadati Pseudomonadota Alphaproteobacteria unclassified Alphaproteobacteria Alphaproteobacteria bacterium MBP9693771.1 310 aa
Pseudomonadati Pseudomonadota Alphaproteobacteria Candidatus Pelagibacterales Pelagibacteraceae bacterium MBH71818.1 308 aa
Pseudomonadati Pseudomonadota Alphaproteobacteria Candidatus Pelagibacterales Pelagibacteraceae bacterium GIR65353.1 308 aa
Pseudomonadati Pseudomonadota Alphaproteobacteria Candidatus Pelagibacterales Pelagibacteraceae bacterium MBI29191.1 311 aa
Pseudomonadati Pseudomonadota Alphaproteobacteria Rickettsiales Rickettsiales bacterium MBS56745.1 311 aa
Pseudomonadati Pseudomonadota Alphaproteobacteria Candidatus Pelagibacterales Pelagibacteraceae bacterium MDC3177153.1 311 aa
Pseudomonadati Pseudomonadota Alphaproteobacteria Rickettsiales Rickettsiales bacterium MAI85077.1 310 aa
Pseudomonadati Pseudomonadota Alphaproteobacteria Rickettsiales Rickettsiales bacterium MBD74940.1 310 aa
Pseudomonadati Pseudomonadota Alphaproteobacteria Rickettsiales Rickettsiales bacterium MBH43411.1 310 aa
Pseudomonadati Pseudomonadota Alphaproteobacteria Rickettsiales Rickettsiales bacterium MDC3091131.1 310 aa
Pseudomonadati Pseudomonadota Alphaproteobacteria unclassified Alphaproteobacteria Alphaproteobacteria bacterium PPR37996.1 310 aa
Pseudomonadati Pseudomonadota Alphaproteobacteria unclassified Alphaproteobacteria Alphaproteobacteria bacterium MEL0245958.1 310 aa
Pseudomonadati Pseudomonadota Alphaproteobacteria Acetobacterales Acetobacter sp. CCZ22345.1 310 aa
Pseudomonadati Pseudomonadota Alphaproteobacteria Rickettsiales Rickettsiales bacterium AIL65790.1 310 aa
Pseudomonadati Pseudomonadota Alphaproteobacteria Rickettsiales Rickettsiales bacterium MDX1949605.1 315 aa
Pseudomonadati Pseudomonadota Alphaproteobacteria unclassified Alphaproteobacteria Alphaproteobacteria bacterium MBT3396787.1 310 aa
Pseudomonadati Pseudomonadota Alphaproteobacteria Elsterales Emcibacter sp. WP 346322482.1 310 aa
Pseudomonadati Pseudomonadota Alphaproteobacteria Kordiimonadales Kordiimonas sp. MAU40687.1 310 aa
Pseudomonadati Pseudomonadota Alphaproteobacteria Emcibacterales Emcibacter nanhaiensis WP 139941093.1 310 aa
Pseudomonadati Pseudomonadota Alphaproteobacteria Elsterales Luteithermobacter gelatinilyticus WP 138380282.1 310 aa
Pseudomonadati Pseudomonadota Alphaproteobacteria Elsterales Emcibacter sp. MCF8473718.1 312 aa
Pseudomonadati Pseudomonadota Alphaproteobacteria Emcibacterales Emcibacter sp. MCK5423871.1 313 aa
Pseudomonadati Pseudomonadota Alphaproteobacteria Emcibacterales Emcibacter sp. MCF6214616.1 312 aa
Pseudomonadati Pseudomonadota Alphaproteobacteria Emcibacterales Pseudemcibacter aquimaris WP 249035476.1 312 aa
Pseudomonadati Pseudomonadota Alphaproteobacteria Kordiimonadales Kordiimonadaceae bacterium MBT6134652.1 311 aa
Pseudomonadati Pseudomonadota Alphaproteobacteria Emcibacterales Emcibacteraceae bacterium HRW29934.1 311 aa
Pseudomonadati Pseudomonadota Alphaproteobacteria Elsterales Emcibacteraceae bacterium MBL4601351.1 311 aa
Pseudomonadati Pseudomonadota Alphaproteobacteria Hyphomicrobiales Hyphomicrobiales bacterium MCP5362109.1 320 aa
Pseudomonadati Pseudomonadota Alphaproteobacteria Rickettsiales Rickettsiales bacterium MCH2037311.1 310 aa
Pseudomonadati Pseudomonadota Alphaproteobacteria unclassified Alphaproteobacteria Alphaproteobacteria bacterium MBL0318888.1 314 aa
Pseudomonadati Pseudomonadota Alphaproteobacteria Rickettsiales Rickettsiales bacterium MCD6035659.1 309 aa
Pseudomonadati Pseudomonadota Alphaproteobacteria Rickettsiales Rickettsiaceae bacterium MCE3231750.1 316 aa
Pseudomonadati Pseudomonadota Alphaproteobacteria Rickettsiales Rickettsiales bacterium MET0154710.1 310 aa
Pseudomonadati Pseudomonadota Alphaproteobacteria Rickettsiales Rickettsiales bacterium MDB2414479.1 310 aa
Pseudomonadati Pseudomonadota Alphaproteobacteria Rickettsiales Rickettsiales bacterium MFO1242819.1 310 aa
Pseudomonadati Pseudomonadota Alphaproteobacteria unclassified Alphaproteobacteria Alphaproteobacteria bacterium MBN8531508.1 310 aa
Pseudomonadati Pseudomonadota Alphaproteobacteria unclassified Alphaproteobacteria Alphaproteobacteria bacterium MCC7260424.1 312 aa
Pseudomonadati Pseudomonadota Alphaproteobacteria Rickettsiales Rickettsiales bacterium MDG1287970.1 310 aa
Pseudomonadati Pseudomonadota Alphaproteobacteria Rickettsiales Rickettsiales bacterium HEU5046211.1 310 aa
Pseudomonadati Pseudomonadota Alphaproteobacteria Rickettsiales Rickettsiales bacterium MCI5049493.1 311 aa
Pseudomonadati Pseudomonadota Alphaproteobacteria Rickettsiales Rickettsiales bacterium MBN67024.1 311 aa
Pseudomonadati Pseudomonadota Alphaproteobacteria unclassified Alphaproteobacteria Alphaproteobacteria bacterium MFO0110101.1 311 aa
Pseudomonadati Pseudomonadota Alphaproteobacteria unclassified Alphaproteobacteria Alphaproteobacteria bacterium MBN8544008.1 311 aa
Pseudomonadati Pseudomonadota Alphaproteobacteria Rickettsiales Rickettsiales bacterium MFZ4540847.1 311 aa
Pseudomonadati Pseudomonadota Alphaproteobacteria Rickettsiales Rickettsiales bacterium MFM9890471.1 311 aa
Pseudomonadati Pseudomonadota Alphaproteobacteria Rickettsiales Rickettsiales bacterium MAR55871.1 311 aa
Pseudomonadati Pseudomonadota Alphaproteobacteria unclassified Alphaproteobacteria Alphaproteobacteria bacterium MGE0753709.1 311 aa
Pseudomonadati Pseudomonadota Alphaproteobacteria unclassified Alphaproteobacteria Alphaproteobacteria bacterium MDX2113385.1 311 aa
Pseudomonadati Pseudomonadota Alphaproteobacteria Rickettsiales Rickettsiales bacterium MFW0777471.1 311 aa
Pseudomonadati Pseudomonadota Alphaproteobacteria Rickettsiales Rickettsiales bacterium MDX1974133.1 311 aa
Pseudomonadati Pseudomonadota Alphaproteobacteria unclassified Alphaproteobacteria Alphaproteobacteria bacterium MDX2074659.1 311 aa
Pseudomonadati Pseudomonadota Alphaproteobacteria Rickettsiales Rickettsiales bacterium MEZ5690616.1 311 aa
Pseudomonadati Pseudomonadota Alphaproteobacteria Rickettsiales Rickettsiales bacterium MEQ1705445.1 311 aa
Pseudomonadati Pseudomonadota Alphaproteobacteria unclassified Alphaproteobacteria Alphaproteobacteria bacterium MSO74973.1 311 aa
Pseudomonadati Pseudomonadota Alphaproteobacteria Rhodospirillales Rhodospirillales bacterium MDD9990594.1 310 aa
Pseudomonadati Pseudomonadota Alphaproteobacteria Rhodospirillales Rhodospirillaceae bacterium MBT5435456.1 310 aa
Pseudomonadati Pseudomonadota Alphaproteobacteria Geminicoccales Geminicoccaceae bacterium HET6518217.1 310 aa
Pseudomonadati Pseudomonadota Alphaproteobacteria Geminicoccales Marinivivus vitaminiproducens WP 442503707.1 310 aa
Pseudomonadati Pseudomonadota Alphaproteobacteria Geminicoccales Geminicoccaceae bacterium MGF1563210.1 310 aa
Pseudomonadati Pseudomonadota Alphaproteobacteria Geminicoccales Geminicoccaceae bacterium MGH1477474.1 310 aa
Pseudomonadati Pseudomonadota Alphaproteobacteria Geminicoccales Geminicoccaceae bacterium MCC2663612.1 310 aa
Pseudomonadati Pseudomonadota Alphaproteobacteria Geminicoccales Geminicoccaceae bacterium HEX6145045.1 310 aa
Pseudomonadati Pseudomonadota Alphaproteobacteria Geminicoccales Geminicoccaceae bacterium MGF1477727.1 310 aa
Pseudomonadati Pseudomonadota Alphaproteobacteria Geminicoccales Geminicoccaceae bacterium MCB2011195.1 310 aa
Pseudomonadati Pseudomonadota Alphaproteobacteria Geminicoccales Geminicoccaceae bacterium MCB1885444.1 310 aa
Pseudomonadati Pseudomonadota Alphaproteobacteria Geminicoccales Geminicoccus flavidas WP 159716451.1 310 aa
Pseudomonadati Pseudomonadota Alphaproteobacteria Geminicoccales Geminicoccaceae bacterium HET6468024.1 310 aa
Pseudomonadati Pseudomonadota Alphaproteobacteria Geminicoccales Geminicoccaceae bacterium MFZ1429085.1 313 aa
Pseudomonadati Pseudomonadota Alphaproteobacteria Geminicoccales Geminicoccaceae bacterium MFO1070446.1 310 aa
Pseudomonadati Pseudomonadota Alphaproteobacteria Geminicoccales Marinimicrococcus flavescens WP 327787184.1 310 aa
Pseudomonadati Pseudomonadota Alphaproteobacteria Geminicoccales Geminicoccaceae bacterium MCL6608663.1 310 aa
Pseudomonadati Pseudomonadota Alphaproteobacteria Geminicoccales Geminicoccaceae bacterium HEX8374869.1 310 aa
Pseudomonadati Pseudomonadota Alphaproteobacteria Geminicoccales Arboricoccus pini WP 088560422.1 310 aa
Pseudomonadati Pseudomonadota Alphaproteobacteria Geminicoccales Tistrella WP 041604782.1 310 aa
Pseudomonadati Pseudomonadota Alphaproteobacteria Rhodospirillales Rhodospirillaceae bacterium MBL29100.1 310 aa
Pseudomonadati Pseudomonadota Alphaproteobacteria Rhodospirillales Rhodospirillaceae bacterium MBT07161.1 310 aa
Pseudomonadati Pseudomonadota Alphaproteobacteria Rhodospirillales Rhodospirillaceae bacterium MBD88382.1 310 aa
Pseudomonadati Pseudomonadota Alphaproteobacteria Rhodospirillales Rhodospirillaceae bacterium KAF0117477.1 310 aa
Pseudomonadati Pseudomonadota Alphaproteobacteria Rhodospirillales Rhodospirillales bacterium MBI4967350.1 310 aa
Pseudomonadati Pseudomonadota Alphaproteobacteria Rhodospirillales Rhodospirillaceae bacterium CAA6604319.1 318 aa
Pseudomonadati Pseudomonadota Alphaproteobacteria Rhodospirillales Telmatospirillum sp. WP 142847296.1 310 aa
Pseudomonadati Pseudomonadota Alphaproteobacteria Rhodospirillales Rhodospirillales WP 009871053.1 310 aa
Pseudomonadati Pseudomonadota Alphaproteobacteria Rhodospirillales Rhodospirillaceae bacterium MDY0241796.1 310 aa
Pseudomonadati Pseudomonadota Alphaproteobacteria Rhodospirillales Telmatospirillum siberiense WP 101251193.1 310 aa
Pseudomonadati Pseudomonadota Alphaproteobacteria Rhodospirillales Rhodospirillaceae bacterium MDR3325958.1 310 aa
Pseudomonadati Pseudomonadota Alphaproteobacteria Rhodospirillales Magnetospirillaceae bacterium HEY1721204.1 310 aa
Pseudomonadati Pseudomonadota Alphaproteobacteria Rhodospirillales Oleispirillum naphthae WP 337996855.1 310 aa
Pseudomonadati Pseudomonadota Alphaproteobacteria Rhodospirillales Roseospira marina WP 150060551.1 310 aa
Pseudomonadati Pseudomonadota Alphaproteobacteria Rhodospirillales Phaeovibrio sulfidiphilus WP 192533333.1 311 aa
Pseudomonadati Pseudomonadota Alphaproteobacteria Rhodospirillales Rhodospirillum sp. WP 413206902.1 310 aa
Pseudomonadati Pseudomonadota Alphaproteobacteria Rhodospirillales Rhodospirillaceae bacterium MGB0697351.1 310 aa
Pseudomonadati Pseudomonadota Alphaproteobacteria Rhodospirillales Roseospirillum parvum WP 092613964.1 310 aa
Pseudomonadati Pseudomonadota Alphaproteobacteria Rhodospirillales unclassified Haematospirillum WP 168020444.1 310 aa
Pseudomonadati Pseudomonadota Alphaproteobacteria Rhodospirillales Novispirillum sp. WP 425009307.1 310 aa
Pseudomonadati Pseudomonadota Alphaproteobacteria Rhodospirillales Rhodospirillaceae bacterium HCM84084.1 310 aa
Pseudomonadati Pseudomonadota Alphaproteobacteria Rhodospirillales Rhodospirillaceae bacterium MSO96772.1 310 aa
Pseudomonadati Pseudomonadota Alphaproteobacteria Rhodospirillales Rhodospirillaceae bacterium TAL02689.1 310 aa
Pseudomonadati Pseudomonadota Alphaproteobacteria Rhodospirillales Rhodospirillaceae bacterium MBX7200800.1 310 aa
Pseudomonadati Pseudomonadota Alphaproteobacteria Rhodospirillales Rhodospirillaceae bacterium MFL2769588.1 311 aa
Pseudomonadati Pseudomonadota Alphaproteobacteria Rhodospirillales Rhodospirillaceae bacterium MDG2244508.1 311 aa
Pseudomonadati Pseudomonadota Alphaproteobacteria Rhodospirillales Rhodospirillaceae bacterium MBP02497.1 309 aa
Pseudomonadati Pseudomonadota Alphaproteobacteria Rhodospirillales Rhodospirillales bacterium MEQ8707802.1 310 aa
Pseudomonadati Pseudomonadota Alphaproteobacteria Rhodospirillales Rhodospirillales bacterium OHC75803.1 319 aa
Pseudomonadati Pseudomonadota Alphaproteobacteria Rhodospirillales Rhodospirillales bacterium MDP6603830.1 310 aa
Pseudomonadati Pseudomonadota Alphaproteobacteria Rhodospirillales Rhodospirillales bacterium MEX2452106.1 310 aa
Pseudomonadati Pseudomonadota Alphaproteobacteria Rhodospirillales Varunaivibrio sulfuroxidans WP 132939729.1 310 aa
Pseudomonadati Pseudomonadota Alphaproteobacteria Rhodospirillales Magnetovibrio sp. WP 332896599.1 312 aa
Pseudomonadati Pseudomonadota Alphaproteobacteria Rhodospirillales Magnetovibrio blakemorei WP 069957933.1 311 aa
Pseudomonadati Pseudomonadota Alphaproteobacteria Rhodospirillales Rhodospirillaceae bacterium MCK5444578.1 298 aa
Pseudomonadati Pseudomonadota Alphaproteobacteria Rhodospirillales Rhodospirillaceae bacterium MDH5187707.1 310 aa
Pseudomonadati Pseudomonadota Alphaproteobacteria Rhodospirillales Rhodospirillaceae bacterium MCK5546126.1 310 aa
Pseudomonadati Pseudomonadota Alphaproteobacteria Rhodospirillales Rhodospirillales bacterium MSO54389.1 310 aa
Pseudomonadati Pseudomonadota Alphaproteobacteria Rhodospirillales Rhodospirillales bacterium MDE0539347.1 310 aa
Pseudomonadati Pseudomonadota Alphaproteobacteria Rhodospirillales Rhodospirillales bacterium MFP6733552.1 310 aa
Pseudomonadati Pseudomonadota Alphaproteobacteria Rhodospirillales Rhodospirillales bacterium MDP7425580.1 310 aa
Pseudomonadati Pseudomonadota Alphaproteobacteria Rhodospirillales Rhodospirillaceae bacterium MEK9677208.1 310 aa
Pseudomonadati Pseudomonadota Alphaproteobacteria Rhodospirillales Rhodospirillales bacterium HHZ76855.1 310 aa
Pseudomonadati Pseudomonadota Alphaproteobacteria Rhodospirillales Rhodospirillaceae bacterium MBL13338.1 310 aa
Pseudomonadati Pseudomonadota Alphaproteobacteria Rhodospirillales Rhodospirillales bacterium MDH5750730.1 310 aa
Pseudomonadati Pseudomonadota Alphaproteobacteria Rhodospirillales Rhodospirillales bacterium HJN23660.1 310 aa
Pseudomonadati Pseudomonadota Alphaproteobacteria Rhodobacterales Rhodobacterales bacterium MCB2102398.1 310 aa
Pseudomonadati Pseudomonadota Alphaproteobacteria Rhodospirillales Magnetospirillum sp. MEO5338268.1 310 aa
Pseudomonadati Pseudomonadota Alphaproteobacteria Rhodospirillales Rhodospirillales bacterium MBT3764946.1 311 aa
Pseudomonadati Pseudomonadota Alphaproteobacteria Rhodospirillales Rhodospirillales bacterium MBT3792283.1 310 aa
Pseudomonadati Pseudomonadota Alphaproteobacteria Rhodospirillales Rhodospirillales bacterium MBT3360220.1 310 aa
Pseudomonadati Pseudomonadota Alphaproteobacteria Rhodospirillales Shumkonia mesophila WP 316979170.1 310 aa
Pseudomonadati Pseudomonadota Alphaproteobacteria Rhodospirillales Rhodospirillales bacterium MCG8511039.1 310 aa
Pseudomonadati Pseudomonadota Alphaproteobacteria Rhodospirillales Magnetovibrionaceae bacterium MGB0682627.1 310 aa
Pseudomonadati Pseudomonadota Alphaproteobacteria Rhodospirillales Rhodospirillaceae bacterium MBT3237782.1 310 aa
Pseudomonadati Pseudomonadota Alphaproteobacteria Rhodospirillales Rhodospirillaceae bacterium MBT4933096.1 310 aa
Pseudomonadati Pseudomonadota Alphaproteobacteria Rhodospirillales Magnetovibrio sp. MBH88902.1 310 aa
Pseudomonadati Pseudomonadota Alphaproteobacteria Rhodospirillales Rhodospirillales bacterium MEQ9554138.1 310 aa
Pseudomonadati Pseudomonadota Alphaproteobacteria Rhodospirillales Rhodospirillales bacterium MEQ8665680.1 310 aa
Pseudomonadati Pseudomonadota Alphaproteobacteria Rhodospirillales Rhodospirillales bacterium MBT5519798.1 310 aa
Pseudomonadati Pseudomonadota Alphaproteobacteria Rhodospirillales Rhodospirillales bacterium MEZ7844788.1 310 aa
Pseudomonadati Pseudomonadota Alphaproteobacteria Rhodospirillales Rhodospirillales bacterium MEE8350884.1 310 aa

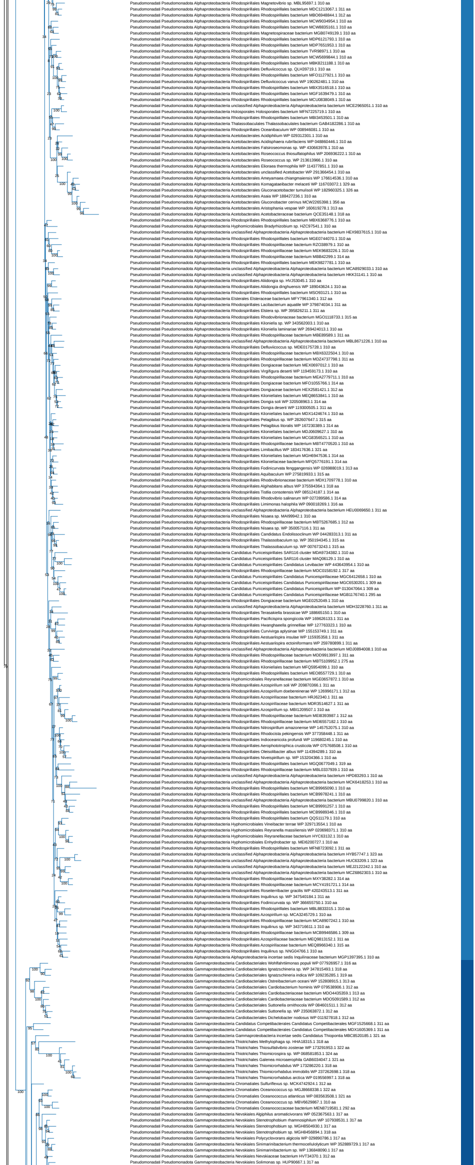
Pseudomonadati Pseudomonadota Alphaproteobacteria Rhodospirillales Magnetovibrio sp. MBL95697.1 310 aa
Pseudomonadati Pseudomonadota Alphaproteobacteria Rhodospirillales Rhodospirillales bacterium MDC1213067.1 311 aa
Pseudomonadati Pseudomonadota Alphaproteobacteria Rhodospirillales Rhodospirillales bacterium MBO6948844.1 312 aa
Pseudomonadati Pseudomonadota Alphaproteobacteria Rhodospirillales Rhodospirillales bacterium MCW9034954.1 310 aa
Pseudomonadati Pseudomonadota Alphaproteobacteria Rhodospirillales Rhodospirillales bacterium MCW8835161.1 310 aa
Pseudomonadati Pseudomonadota Alphaproteobacteria Rhodospirillales Magnetospiraceae bacterium MGB0749139.1 310 aa
Pseudomonadati Pseudomonadota Alphaproteobacteria Rhodospirillales Rhodospirillales bacterium MDP6121793.1 310 aa
Pseudomonadati Pseudomonadota Alphaproteobacteria Rhodospirillales Rhodospirillales bacterium MDP7651953.1 310 aa
Pseudomonadati Pseudomonadota Alphaproteobacteria Rhodospirillales Rhodospirillales bacterium TVR98971.1 310 aa
Pseudomonadati Pseudomonadota Alphaproteobacteria Rhodospirillales Rhodospirillales bacterium MCW5699844.1 310 aa
Pseudomonadati Pseudomonadota Alphaproteobacteria Rhodospirillales Rhodospirillales bacterium MBK8211188.1 310 aa
Pseudomonadati Pseudomonadota Alphaproteobacteria Rhodospirillales Defluviicoccus sp. QLH39719.1 310 aa
Pseudomonadati Pseudomonadota Alphaproteobacteria Rhodospirillales Rhodospirillales bacterium MFO1127921.1 310 aa
Pseudomonadati Pseudomonadota Alphaproteobacteria Rhodospirillales Defluviicoccus vanus WP 190262481.1 310 aa
Pseudomonadati Pseudomonadota Alphaproteobacteria Rhodospirillales Rhodospirillales bacterium MBX3516518.1 310 aa
Pseudomonadati Pseudomonadota Alphaproteobacteria Rhodospirillales Rhodospirillales bacterium MGF1639479.1 310 aa
Pseudomonadati Pseudomonadota Alphaproteobacteria Rhodospirillales Rhodospirillales bacterium MCU0838049.1 310 aa
Pseudomonadati Pseudomonadota Alphaproteobacteria unclassified Alphaproteobacteria Alphaproteobacteria bacterium MCE2965051.1 310 aa
Pseudomonadati Pseudomonadota Alphaproteobacteria Holosporales Holosporales bacterium MFN7225719.1 310 aa
Pseudomonadati Pseudomonadota Alphaproteobacteria Rhodospirillales Rhodospirillales bacterium MBI3453501.1 310 aa
Pseudomonadati Pseudomonadota Alphaproteobacteria Thalassobaculales Thalassobaculales bacterium GAB4182286.1 310 aa
Pseudomonadati Pseudomonadota Alphaproteobacteria Rhodospirillales Oceanibaculum WP 008946081.1 310 aa
Pseudomonadati Pseudomonadota Alphaproteobacteria Acetobacterales Acidiphilium WP 029312301.1 310 aa
Pseudomonadati Pseudomonadota Alphaproteobacteria Acetobacterales Acidisphaera rubrifaciens WP 048860446.1 310 aa
Pseudomonadati Pseudomonadota Alphaproteobacteria Acetobacterales Falsiroseomonas sp. WP 430663978.1 310 aa
Pseudomonadati Pseudomonadota Alphaproteobacteria Acetobacterales Roseococcus thiosulfatophilus WP 206936222.1 310 aa
Pseudomonadati Pseudomonadota Alphaproteobacteria Acetobacterales Roseococcus sp. WP 213613966.1 310 aa
Pseudomonadati Pseudomonadota Alphaproteobacteria Acetobacterales Elioraea thermophila WP 114377851.1 310 aa
Pseudomonadati Pseudomonadota Alphaproteobacteria Acetobacterales unclassified Acetobacter WP 291366454.1 310 aa
Pseudomonadati Pseudomonadota Alphaproteobacteria Acetobacterales Ameyamaea chiangmaiensis WP 176614536.1 310 aa
Pseudomonadati Pseudomonadota Alphaproteobacteria Acetobacterales Komagataeibacter melaceti WP 116703072.1 329 aa
Pseudomonadati Pseudomonadota Alphaproteobacteria Acetobacterales Gluconacetobacter tumulisoli WP 182960325.1 326 aa
Pseudomonadati Pseudomonadota Alphaproteobacteria Acetobacterales Asaia WP 188427236.1 310 aa
Pseudomonadati Pseudomonadota Alphaproteobacteria Acetobacterales Gluconobacter cerinus MCW2265398.1 356 aa
Pseudomonadati Pseudomonadota Alphaproteobacteria Acetobacterales Aristophania vespae WP 160619278.1 313 aa
Pseudomonadati Pseudomonadota Alphaproteobacteria Acetobacterales Acetobacteraceae bacterium QCE35148.1 318 aa
Pseudomonadati Pseudomonadota Alphaproteobacteria Rhodospirillales Rhodospirillales bacterium MBX6368776.1 310 aa
Pseudomonadati Pseudomonadota Alphaproteobacteria Hyphomicrobiales Bradyrhizobium sp. HZC97541.1 310 aa
Pseudomonadati Pseudomonadota Alphaproteobacteria unclassified Alphaproteobacteria Alphaproteobacteria bacterium HEX9837615.1 310 aa
Pseudomonadati Pseudomonadota Alphaproteobacteria Rhodospirillales Rhodospirillales bacterium MGE0744070.1 310 aa
Pseudomonadati Pseudomonadota Alphaproteobacteria Rhodospirillales Rhodospirillaceae bacterium RZO38979.1 310 aa
Pseudomonadati Pseudomonadota Alphaproteobacteria Rhodospirillales Rhodospirillales bacterium MEK9683226.1 310 aa
Pseudomonadati Pseudomonadota Alphaproteobacteria Rhodospirillales Rhodospirillaceae bacterium MBB42299.1 314 aa
Pseudomonadati Pseudomonadota Alphaproteobacteria Rhodospirillales Rhodospirillales bacterium MEK9827781.1 310 aa
Pseudomonadati Pseudomonadota Alphaproteobacteria unclassified Alphaproteobacteria Alphaproteobacteria bacterium MCA8929033.1 310 aa
Pseudomonadati Pseudomonadota Alphaproteobacteria unclassified Alphaproteobacteria Alphaproteobacteria bacterium HKK31141.1 310 aa
Pseudomonadati Pseudomonadota Alphaproteobacteria Rhodospirillales Aliidongia sp. HVJ53045.1 310 aa
Pseudomonadati Pseudomonadota Alphaproteobacteria Rhodospirillales Aliidongia dinghuensis WP 189043624.1 310 aa
Pseudomonadati Pseudomonadota Alphaproteobacteria Rhodospirillales Rhodospirillales bacterium MSO93121.1 310 aa
Pseudomonadati Pseudomonadota Alphaproteobacteria Elsterales Elsteraceae bacterium MFY7961340.1 312 aa
Pseudomonadati Pseudomonadota Alphaproteobacteria Rhodospirillales Lacibacterium aquatile WP 379874034.1 311 aa
Pseudomonadati Pseudomonadota Alphaproteobacteria Rhodospirillales Elstera sp. WP 395826211.1 311 aa
Pseudomonadati Pseudomonadota Alphaproteobacteria Rhodospirillales Rhodovibrionaceae bacterium MGO1118733.1 315 aa
Pseudomonadati Pseudomonadota Alphaproteobacteria Rhodospirillales Kiloniella sp. WP 343562003.1 310 aa
Pseudomonadati Pseudomonadota Alphaproteobacteria Rhodospirillales Kiloniella laminariae WP 269424013.1 310 aa
Pseudomonadati Pseudomonadota Alphaproteobacteria Rhodospirillales Rhodospirillaceae bacterium MBE89589.1 311 aa
Pseudomonadati Pseudomonadota Alphaproteobacteria unclassified Alphaproteobacteria Alphaproteobacteria bacterium MBL8671226.1 310 aa
Pseudomonadati Pseudomonadota Alphaproteobacteria Rhodospirillales Defluviicoccus sp. MDE0175728.1 310 aa
Pseudomonadati Pseudomonadota Alphaproteobacteria Rhodospirillales Rhodospirillaceae bacterium MBX6322504.1 310 aa
Pseudomonadati Pseudomonadota Alphaproteobacteria Rhodospirillales Rhodospirillaceae bacterium MDZ4737798.1 311 aa
Pseudomonadati Pseudomonadota Alphaproteobacteria Rhodospirillales Dongiaceae bacterium MEX0697012.1 310 aa
Pseudomonadati Pseudomonadota Alphaproteobacteria Rhodospirillales Virgifigura deserti WP 119459173.1 310 aa
Pseudomonadati Pseudomonadota Alphaproteobacteria Rhodospirillales Rhodospirillaceae bacterium MEA2779711.1 310 aa
Pseudomonadati Pseudomonadota Alphaproteobacteria Rhodospirillales Dongiaceae bacterium MFO1055766.1 314 aa
Pseudomonadati Pseudomonadota Alphaproteobacteria Rhodospirillales Dongiaceae bacterium HEX2581421.1 312 aa
Pseudomonadati Pseudomonadota Alphaproteobacteria Rhodospirillales Kiloniellales bacterium MEQ8653841.1 310 aa
Pseudomonadati Pseudomonadota Alphaproteobacteria Rhodospirillales Dongia soli WP 320508963.1 314 aa
Pseudomonadati Pseudomonadota Alphaproteobacteria Rhodospirillales Dongia deserti WP 119300505.1 311 aa
Pseudomonadati Pseudomonadota Alphaproteobacteria Rhodospirillales Kiloniellales bacterium MDX1424674.1 310 aa
Pseudomonadati Pseudomonadota Alphaproteobacteria Rhodospirillales Pelagibius sp. WP 282607647.1 315 aa
Pseudomonadati Pseudomonadota Alphaproteobacteria Rhodospirillales Pelagibius litoralis WP 167230389.1 314 aa
Pseudomonadati Pseudomonadota Alphaproteobacteria Rhodospirillales Kiloniellales bacterium MDJ0609627.1 310 aa
Pseudomonadati Pseudomonadota Alphaproteobacteria Rhodospirillales Kiloniellales bacterium MCG8356521.1 310 aa
Pseudomonadati Pseudomonadota Alphaproteobacteria Rhodospirillales Rhodospirillaceae bacterium MBT4770520.1 310 aa
Pseudomonadati Pseudomonadota Alphaproteobacteria Rhodospirillales Limibacillus WP 183417636.1 321 aa
Pseudomonadati Pseudomonadota Alphaproteobacteria Rhodospirillales Kiloniellales bacterium MGH6947536.1 314 aa
Pseudomonadati Pseudomonadota Alphaproteobacteria Rhodospirillales Kiloniellaceae bacterium MFQ5776191.1 314 aa
Pseudomonadati Pseudomonadota Alphaproteobacteria Rhodospirillales Fodinicurvata fenggangensis WP 026988019.1 313 aa
Pseudomonadati Pseudomonadota Alphaproteobacteria Rhodospirillales Aquibaculum WP 275819933.1 315 aa
Pseudomonadati Pseudomonadota Alphaproteobacteria Rhodospirillales Rhodovibrionaceae bacterium MDX1709778.1 310 aa
Pseudomonadati Pseudomonadota Alphaproteobacteria Rhodospirillales Algihabitans albus WP 375594364.1 318 aa
Pseudomonadati Pseudomonadota Alphaproteobacteria Rhodospirillales Tistlia consotensis WP 085124187.1 314 aa
Pseudomonadati Pseudomonadota Alphaproteobacteria Rhodospirillales Rhodovibrio salinarum WP 027289586.1 314 aa
Pseudomonadati Pseudomonadota Alphaproteobacteria Rhodospirillales Limimonas halophila WP 090018269.1 316 aa
Pseudomonadati Pseudomonadota Alphaproteobacteria unclassified Alphaproteobacteria Alphaproteobacteria bacterium HEU0069650.1 311 aa
Pseudomonadati Pseudomonadota Alphaproteobacteria Rhodospirillales Nisaea sp. MAI99942.1 310 aa
Pseudomonadati Pseudomonadota Alphaproteobacteria Rhodospirillales Rhodospirillaceae bacterium MBT5267685.1 312 aa
Pseudomonadati Pseudomonadota Alphaproteobacteria Rhodospirillales Nisaea sp. WP 350057116.1 311 aa
Pseudomonadati Pseudomonadota Alphaproteobacteria Rhodospirillales Candidatus Endolissoclinum WP 044283313.1 311 aa
Pseudomonadati Pseudomonadota Alphaproteobacteria Rhodospirillales Thalassobaculum sp. WP 350194345.1 315 aa
Pseudomonadati Pseudomonadota Alphaproteobacteria Rhodospirillales Thalassobaculum sp. WP 007673243.1 315 aa
Pseudomonadati Pseudomonadota Alphaproteobacteria Candidatus Puniceispirillales SAR116 cluster MDA9734382.1 310 aa
Pseudomonadati Pseudomonadota Alphaproteobacteria Candidatus Puniceispirillales SAR116 cluster MAQ06129.1 310 aa
Pseudomonadati Pseudomonadota Alphaproteobacteria Candidatus Puniceispirillales Candidatus Levibacter WP 443643954.1 310 aa
Pseudomonadati Pseudomonadota Alphaproteobacteria Rhodospirillales Rhodospirillaceae bacterium MDC0158192.1 317 aa
Pseudomonadati Pseudomonadota Alphaproteobacteria Candidatus Puniceispirillales Candidatus Puniceispirillaceae MGC6412658.1 310 aa
Pseudomonadati Pseudomonadota Alphaproteobacteria Candidatus Puniceispirillales Candidatus Puniceispirillaceae MGC6530201.1 309 aa
Pseudomonadati Pseudomonadota Alphaproteobacteria Candidatus Puniceispirillales Candidatus Puniceispirillum WP 013047064.1 309 aa
Pseudomonadati Pseudomonadota Alphaproteobacteria Candidatus Puniceispirillales Candidatus Puniceispirillaceae MGB1176740.1 295 aa
Pseudomonadati Pseudomonadota Alphaproteobacteria Rhodospirillales Dongiaceae bacterium MGE0252049.1 310 aa
Pseudomonadati Pseudomonadota Alphaproteobacteria unclassified Alphaproteobacteria Alphaproteobacteria bacterium MDH3228760.1 311 aa
Pseudomonadati Pseudomonadota Alphaproteobacteria Rhodospirillales Terasakiella brassicae WP 188665150.1 310 aa
Pseudomonadati Pseudomonadota Alphaproteobacteria Rhodospirillales Pacificispira spongiicola WP 169626133.1 311 aa
Pseudomonadati Pseudomonadota Alphaproteobacteria Rhodospirillales Hwanghaeella grinnelliae WP 127763323.1 310 aa
Pseudomonadati Pseudomonadota Alphaproteobacteria Rhodospirillales Curvivirga aplysinae WP 155153749.1 311 aa
Pseudomonadati Pseudomonadota Alphaproteobacteria Rhodospirillales Aestuariispira insulae WP 115935358.1 311 aa
Pseudomonadati Pseudomonadota Alphaproteobacteria Rhodospirillales Aestuariispira ectoiniformans WP 259780899.1 311 aa
Pseudomonadati Pseudomonadota Alphaproteobacteria unclassified Alphaproteobacteria Alphaproteobacteria bacterium MDJ0894008.1 310 aa
Pseudomonadati Pseudomonadota Alphaproteobacteria Rhodospirillales Rhodospirillaceae bacterium MDD9913997.1 311 aa
Pseudomonadati Pseudomonadota Alphaproteobacteria Rhodospirillales Rhodospirillaceae bacterium MBT5109952.1 275 aa
Pseudomonadati Pseudomonadota Alphaproteobacteria Rhodospirillales Kiloniellales bacterium MFQ5954099.1 310 aa
Pseudomonadati Pseudomonadota Alphaproteobacteria Rhodospirillales Rhodospirillaceae bacterium MEO8557729.1 310 aa
Pseudomonadati Pseudomonadota Alphaproteobacteria Hyphomicrobiales Reyranellaceae bacterium MGE0657872.1 310 aa
Pseudomonadati Pseudomonadota Alphaproteobacteria Rhodospirillales Azospirillum soli WP 209870366.1 311 aa
Pseudomonadati Pseudomonadota Alphaproteobacteria Rhodospirillales Azospirillum doebereinerae WP 126996171.1 312 aa
Pseudomonadati Pseudomonadota Alphaproteobacteria Rhodospirillales Azospirillaceae bacterium HRJ62340.1 311 aa
Pseudomonadati Pseudomonadota Alphaproteobacteria Rhodospirillales Azospirillaceae bacterium MDR3514627.1 311 aa
Pseudomonadati Pseudomonadota Alphaproteobacteria Rhodospirillales Azospirillum sp. MBI1209507.1 310 aa
Pseudomonadati Pseudomonadota Alphaproteobacteria Rhodospirillales Rhodospirillaceae bacterium MEI8393987.1 312 aa
Pseudomonadati Pseudomonadota Alphaproteobacteria Rhodospirillales Rhodospirillaceae bacterium MEI6557182.1 310 aa
Pseudomonadati Pseudomonadota Alphaproteobacteria Rhodospirillales Nitrospirillum amazonense WP 145752075.1 310 aa
Pseudomonadati Pseudomonadota Alphaproteobacteria Rhodospirillales Rhodocista pekingensis WP 377358448.1 311 aa
Pseudomonadati Pseudomonadota Alphaproteobacteria Rhodospirillales Indioceanicola profundi WP 119680245.1 310 aa
Pseudomonadati Pseudomonadota Alphaproteobacteria Rhodospirillales Aerophototrophica crusticola WP 075768508.1 310 aa
Pseudomonadati Pseudomonadota Alphaproteobacteria Rhodospirillales Oleisolibacter albus WP 114394289.1 310 aa
Pseudomonadati Pseudomonadota Alphaproteobacteria Rhodospirillales Niveispirillum sp. WP 153204366.1 310 aa
Pseudomonadati Pseudomonadota Alphaproteobacteria Rhodospirillales Rhodospirillaceae bacterium MGQ0677049.1 319 aa
Pseudomonadati Pseudomonadota Alphaproteobacteria Rhodospirillales Rhodospirillaceae bacterium MBL0337939.1 310 aa
Pseudomonadati Pseudomonadota Alphaproteobacteria unclassified Alphaproteobacteria Alphaproteobacteria bacterium HPD83293.1 310 aa
Pseudomonadati Pseudomonadota Alphaproteobacteria unclassified Alphaproteobacteria Alphaproteobacteria bacterium MCK6418253.1 310 aa
Pseudomonadati Pseudomonadota Alphaproteobacteria Rhodospirillales Rhodospirillales bacterium MCB9965090.1 310 aa
Pseudomonadati Pseudomonadota Alphaproteobacteria Rhodospirillales Rhodospirillales bacterium MCB9978241.1 310 aa
Pseudomonadati Pseudomonadota Alphaproteobacteria unclassified Alphaproteobacteria Alphaproteobacteria bacterium MBU0799820.1 310 aa
Pseudomonadati Pseudomonadota Alphaproteobacteria Rhodospirillales Rhodospirillales bacterium MCB9991257.1 310 aa
Pseudomonadati Pseudomonadota Alphaproteobacteria Rhodospirillales Rhodospirillales bacterium MCB9989346.1 310 aa
Pseudomonadati Pseudomonadota Alphaproteobacteria Rhodospirillales Rhodospirillales bacterium QQS11179.1 310 aa
Pseudomonadati Pseudomonadota Alphaproteobacteria Hyphomicrobiales Vineibacter terrae WP 329713554.1 310 aa
Pseudomonadati Pseudomonadota Alphaproteobacteria Hyphomicrobiales Reyranella massiliensis WP 020698371.1 310 aa
Pseudomonadati Pseudomonadota Alphaproteobacteria Hyphomicrobiales Reyranellaceae bacterium HYC63132.1 310 aa
Pseudomonadati Pseudomonadota Alphaproteobacteria Hyphomicrobiales Enhydrobacter sp. MEI6200727.1 310 aa
Pseudomonadati Pseudomonadota Alphaproteobacteria Rhodospirillales Rhodospirillales bacterium MFN8723092.1 311 aa
Pseudomonadati Pseudomonadota Alphaproteobacteria unclassified Alphaproteobacteria Alphaproteobacteria bacterium HYB57747.1 323 aa
Pseudomonadati Pseudomonadota Alphaproteobacteria unclassified Alphaproteobacteria Alphaproteobacteria bacterium HUC63209.1 323 aa
Pseudomonadati Pseudomonadota Alphaproteobacteria unclassified Alphaproteobacteria Alphaproteobacteria bacterium MEJ2122242.1 310 aa
Pseudomonadati Pseudomonadota Alphaproteobacteria unclassified Alphaproteobacteria Alphaproteobacteria bacterium MCZ6862303.1 310 aa
Pseudomonadati Pseudomonadota Alphaproteobacteria Rhodospirillales Rhodospirillaceae bacterium MXY38282.1 314 aa
Pseudomonadati Pseudomonadota Alphaproteobacteria Rhodospirillales Rhodospirillaceae bacterium MCY4191721.1 314 aa
Pseudomonadati Pseudomonadota Alphaproteobacteria Rhodospirillales Roseiterribacter gracilis WP 420243513.1 311 aa
Pseudomonadati Pseudomonadota Alphaproteobacteria Rhodospirillales Inquilinus sp. WP 347540184.1 311 aa
Pseudomonadati Pseudomonadota Alphaproteobacteria Rhodospirillales Fodinicurvata sp. WP 366655750.1 310 aa
Pseudomonadati Pseudomonadota Alphaproteobacteria Rhodospirillales Rhodospirillales bacterium MBL8833315.1 310 aa
Pseudomonadati Pseudomonadota Alphaproteobacteria Rhodospirillales Azospirillum sp. MCA3245729.1 310 aa
Pseudomonadati Pseudomonadota Alphaproteobacteria Rhodospirillales Rhodospirillaceae bacterium MCA8907242.1 310 aa
Pseudomonadati Pseudomonadota Alphaproteobacteria Rhodospirillales Inquilinus sp. WP 343716611.1 310 aa
Pseudomonadati Pseudomonadota Alphaproteobacteria Rhodospirillales Rhodospirillaceae bacterium MCB9946586.1 309 aa
Pseudomonadati Pseudomonadota Alphaproteobacteria Rhodospirillales Azospirillaceae bacterium MEQ9813152.1 311 aa
Pseudomonadati Pseudomonadota Alphaproteobacteria Rhodospirillales Azospirillaceae bacterium MEQ8966340.1 315 aa
Pseudomonadati Pseudomonadota Alphaproteobacteria Rhodospirillales Inquilinus sp. NNG04766.1 310 aa
Pseudomonadati Pseudomonadota Alphaproteobacteria Alphaproteobacteria incertae sedis Inquilinaceae bacterium MGP1397395.1 310 aa
Pseudomonadati Pseudomonadota Gammaproteobacteria Cardiobacteriales Wohlfahrtiimonas populi WP 077926957.1 316 aa
Pseudomonadati Pseudomonadota Gammaproteobacteria Cardiobacteriales Ignatzschineria sp. WP 347815493.1 318 aa
Pseudomonadati Pseudomonadota Gammaproteobacteria Cardiobacteriales Ignatzschineria indica WP 109235285.1 319 aa
Pseudomonadati Pseudomonadota Gammaproteobacteria Cardiobacteriales Ostreibacterium oceani WP 152808915.1 313 aa
Pseudomonadati Pseudomonadota Gammaproteobacteria Cardiobacteriales Cardiobacterium hominis WP 079538906.1 312 aa
Pseudomonadati Pseudomonadota Gammaproteobacteria Cardiobacteriales Cardiobacteriaceae bacterium MDO4435659.1 313 aa
Pseudomonadati Pseudomonadota Gammaproteobacteria Cardiobacteriales Cardiobacteriaceae bacterium MDO5091589.1 312 aa
Pseudomonadati Pseudomonadota Gammaproteobacteria Cardiobacteriales Suttonella ornithocola WP 084601511.1 312 aa
Pseudomonadati Pseudomonadota Gammaproteobacteria Cardiobacteriales Suttonella sp. WP 235063872.1 312 aa
Pseudomonadati Pseudomonadota Gammaproteobacteria Cardiobacteriales Dichelobacter nodosus WP 011927818.1 312 aa
Pseudomonadati Pseudomonadota Gammaproteobacteria Candidatus Competibacterales Candidatus Competibacterales MGF1525668.1 311 aa
Pseudomonadati Pseudomonadota Gammaproteobacteria Candidatus Competibacterales Candidatus Competibacterales MDX1605369.1 311 aa
Pseudomonadati Pseudomonadota Gammaproteobacteria Gammaproteobacteria incertae sedis Candidatus Thiopontia MBC8520185.1 321 aa
Pseudomonadati Pseudomonadota Gammaproteobacteria Thiotrichales Methylophaga sp. HHA18315.1 318 aa
Pseudomonadati Pseudomonadota Gammaproteobacteria Thiotrichales Thiosulfativibrio zosterae WP 173291953.1 322 aa
Pseudomonadati Pseudomonadota Gammaproteobacteria Thiotrichales Thiomicrospira sp. WP 068581853.1 324 aa
Pseudomonadati Pseudomonadota Gammaproteobacteria Thiotrichales Galenea microaerophila GAB6034047.1 321 aa
Pseudomonadati Pseudomonadota Gammaproteobacteria Thiotrichales Thiomicrorhabdus WP 173286220.1 318 aa
Pseudomonadati Pseudomonadota Gammaproteobacteria Thiotrichales Thiomicrorhabdus immobilis WP 237262698.1 318 aa
Pseudomonadati Pseudomonadota Gammaproteobacteria Thiotrichales Thiomicrorhabdus arctica WP 019556997.1 318 aa
Pseudomonadati Pseudomonadota Gammaproteobacteria Chromatiales Sulfuriflexus sp. MCK4742924.1 312 aa
Pseudomonadati Pseudomonadota Gammaproteobacteria Chromatiales Oceanococcus sp. MGJ8668338.1 322 aa
Pseudomonadati Pseudomonadota Gammaproteobacteria Chromatiales Oceanococcus atlanticus WP 083563508.1 321 aa
Pseudomonadati Pseudomonadota Gammaproteobacteria Chromatiales Oceanococcus sp. MBV6629867.1 310 aa
Pseudomonadati Pseudomonadota Gammaproteobacteria Chromatiales Oceanococcaceae bacterium MEN8719581.1 292 aa
Pseudomonadati Pseudomonadota Gammaproteobacteria Nevskiales Algiphilus aromaticivorans WP 052367563.1 317 aa
Pseudomonadati Pseudomonadota Gammaproteobacteria Nevskiales Stenotrophobium rhamnosiphilum WP 107938531.1 317 aa
Pseudomonadati Pseudomonadota Gammaproteobacteria Nevskiales Stenotrophobium sp. MGH8504930.1 317 aa
Pseudomonadati Pseudomonadota Gammaproteobacteria Nevskiales Stenotrophobium sp. MGH8456894.1 318 aa
Pseudomonadati Pseudomonadota Gammaproteobacteria Nevskiales Polycyclovorans algicola WP 029890786.1 317 aa
Pseudomonadati Pseudomonadota Gammaproteobacteria Nevskiales Sinimarinibacterium thermocellulolyticum WP 352889729.1 317 aa
Pseudomonadati Pseudomonadota Gammaproteobacteria Nevskiales Sinimarinibacterium sp. WP 136848090.1 317 aa
Pseudomonadati Pseudomonadota Gammaproteobacteria Nevskiales Nevskiaceae bacterium HVT34370.1 312 aa
Pseudomonadati Pseudomonadota Gammaproteobacteria Nevskiales Solimonas sp. HUP90667.1 317 aa

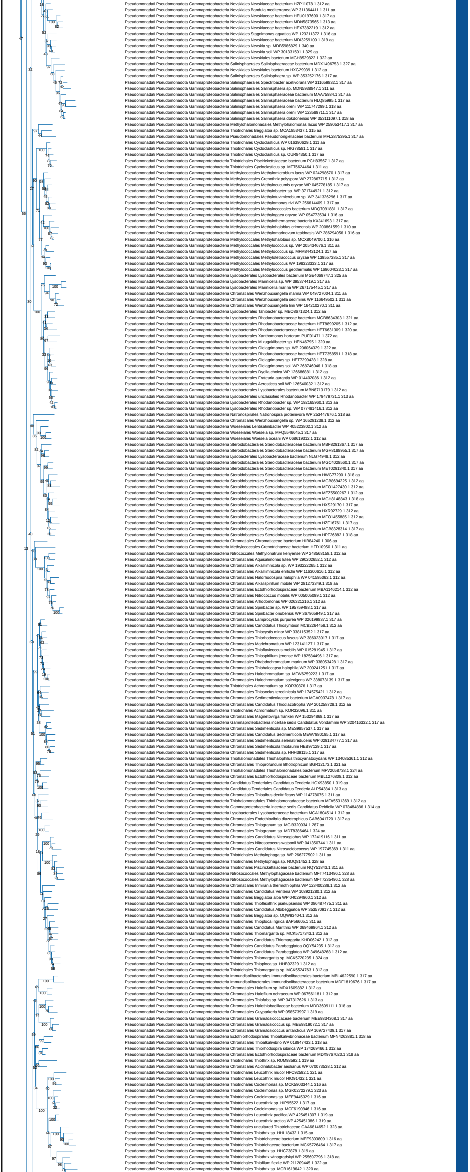
Pseudomonadati Pseudomonadota Gammaproteobacteria Nevskiales Nevskiaceae bacterium HZP11078.1 312 aa
Pseudomonadati Pseudomonadota Gammaproteobacteria Nevskiales Banduia mediterranea WP 311364411.1 311 aa
Pseudomonadati Pseudomonadota Gammaproteobacteria Nevskiales Nevskiaceae bacterium HEU0197690.1 317 aa
Pseudomonadati Pseudomonadota Gammaproteobacteria Nevskiales Nevskiaceae bacterium MDN5873565.1 313 aa
Pseudomonadati Pseudomonadota Gammaproteobacteria Nevskiales Nevskiaceae bacterium HEX7382219.1 312 aa
Pseudomonadati Pseudomonadota Gammaproteobacteria Nevskiales Stagnimonas aquatica WP 123211372.1 316 aa
Pseudomonadati Pseudomonadota Gammaproteobacteria Nevskiales Nevskiaceae bacterium MDI3259100.1 319 aa
Pseudomonadati Pseudomonadota Gammaproteobacteria Nevskiales Nevskia sp. MDB5986829.1 340 aa
Pseudomonadati Pseudomonadota Gammaproteobacteria Nevskiales Nevskia soli WP 301331501.1 329 aa
Pseudomonadati Pseudomonadota Gammaproteobacteria Nevskiales Nevskiales bacterium MGH8529822.1 322 aa
Pseudomonadati Pseudomonadota Gammaproteobacteria Salinisphaerales Salinisphaeraceae bacterium MDX1496753.1 327 aa
Pseudomonadati Pseudomonadota Gammaproteobacteria Nevskiales Nevskiales bacterium HXG29939.1 312 aa
Pseudomonadati Pseudomonadota Gammaproteobacteria Salinisphaerales Salinisphaera sp. WP 353252176.1 317 aa
Pseudomonadati Pseudomonadota Gammaproteobacteria Salinisphaerales Spectribacter acetivorans WP 311659832.1 317 aa
Pseudomonadati Pseudomonadota Gammaproteobacteria Salinisphaerales Salinisphaera sp. MDN5938847.1 311 aa
Pseudomonadati Pseudomonadota Gammaproteobacteria Salinisphaerales Salinisphaeraceae bacterium MAA75934.1 317 aa
Pseudomonadati Pseudomonadota Gammaproteobacteria Salinisphaerales Salinisphaeraceae bacterium HLQ85995.1 317 aa
Pseudomonadati Pseudomonadota Gammaproteobacteria Salinisphaerales Salinisphaera orenii WP 111747299.1 318 aa
Pseudomonadati Pseudomonadota Gammaproteobacteria Salinisphaerales Salinisphaera orenii WP 123589711.1 317 aa
Pseudomonadati Pseudomonadota Gammaproteobacteria Salinisphaerales Salinisphaera dokdonensis WP 353111097.1 318 aa
Pseudomonadati Pseudomonadota Gammaproteobacteria Methylohalomonadales Methylohalomonas lacus WP 259053417.1 317 aa
Pseudomonadati Pseudomonadota Gammaproteobacteria Thiotrichales Beggiatoa sp. MCA1853437.1 315 aa
Pseudomonadati Pseudomonadota Gammaproteobacteria Pseudomonadales Pseudohongiellaceae bacterium MFL2875395.1 317 aa
Pseudomonadati Pseudomonadota Gammaproteobacteria Thiotrichales Cycloclasticus WP 016390629.1 311 aa
Pseudomonadati Pseudomonadota Gammaproteobacteria Thiotrichales Cycloclasticus sp. HIG78581.1 317 aa
Pseudomonadati Pseudomonadota Gammaproteobacteria Thiotrichales Cycloclasticus sp. OUR84350.1 317 aa
Pseudomonadati Pseudomonadota Gammaproteobacteria Thiotrichales Piscirickettsiaceae bacterium PCH83567.1 317 aa
Pseudomonadati Pseudomonadota Gammaproteobacteria Thiotrichales Cycloclasticus sp. MFT6624464.1 311 aa
Pseudomonadati Pseudomonadota Gammaproteobacteria Methylococcales Methylomicrobium lacus WP 024298670.1 317 aa
Pseudomonadati Pseudomonadota Gammaproteobacteria Methylococcales Crenothrix polyspora WP 272867715.1 312 aa
Pseudomonadati Pseudomonadota Gammaproteobacteria Methylococcales Methylocucumis oryzae WP 045778185.1 317 aa
Pseudomonadati Pseudomonadota Gammaproteobacteria Methylococcales Methylobacter sp. WP 371744921.1 312 aa
Pseudomonadati Pseudomonadota Gammaproteobacteria Methylococcales Methylotuvimicrobium sp. WP 341326296.1 317 aa
Pseudomonadati Pseudomonadota Gammaproteobacteria Methylococcales Methylomonas rivi WP 256614409.1 317 aa
Pseudomonadati Pseudomonadota Gammaproteobacteria Methylococcales Methylococcales bacterium MDQ7091881.1 317 aa
Pseudomonadati Pseudomonadota Gammaproteobacteria Methylococcales Methylogaea oryzae WP 054773534.1 316 aa
Pseudomonadati Pseudomonadota Gammaproteobacteria Methylococcales Methylothermaceae bacteria KXJ41693.1 317 aa
Pseudomonadati Pseudomonadota Gammaproteobacteria Methylococcales Methylohalobius crimeensis WP 200861559.1 310 aa
Pseudomonadati Pseudomonadota Gammaproteobacteria Methylococcales Methylomarinovum tepidoasis WP 286294056.1 316 aa
Pseudomonadati Pseudomonadota Gammaproteobacteria Methylococcales Methylohalobius sp. MCX8049700.1 316 aa
Pseudomonadati Pseudomonadota Gammaproteobacteria Methylococcales Methylococcus sp. WP 205434676.1 311 aa
Pseudomonadati Pseudomonadota Gammaproteobacteria Methylococcales Methylococcus sp. MFM8443124.1 317 aa
Pseudomonadati Pseudomonadota Gammaproteobacteria Methylococcales Methylotetracoccus oryzae WP 139557385.1 317 aa
Pseudomonadati Pseudomonadota Gammaproteobacteria Methylococcales Methylococcus WP 198323333.1 317 aa
Pseudomonadati Pseudomonadota Gammaproteobacteria Methylococcales Methylococcus geothermalis WP 169604023.1 317 aa
Pseudomonadati Pseudomonadota Gammaproteobacteria Lysobacterales Lysobacterales bacterium MGE4069747.1 325 aa
Pseudomonadati Pseudomonadota Gammaproteobacteria Lysobacterales Marinicella sp. WP 395374419.1 317 aa
Pseudomonadati Pseudomonadota Gammaproteobacteria Lysobacterales Marinicella marina WP 267175445.1 317 aa
Pseudomonadati Pseudomonadota Gammaproteobacteria Chromatiales Wenzhouxiangella marina WP 049727004.1 311 aa
Pseudomonadati Pseudomonadota Gammaproteobacteria Chromatiales Wenzhouxiangella sediminis WP 116649502.1 311 aa
Pseudomonadati Pseudomonadota Gammaproteobacteria Chromatiales Wenzhouxiangella limi WP 164210270.1 311 aa
Pseudomonadati Pseudomonadota Gammaproteobacteria Lysobacterales Tahibacter sp. MEO8671324.1 312 aa
Pseudomonadati Pseudomonadota Gammaproteobacteria Lysobacterales Rhodanobacteraceae bacterium MGB8634303.1 321 aa
Pseudomonadati Pseudomonadota Gammaproteobacteria Lysobacterales Rhodanobacteraceae bacterium HET8899205.1 312 aa
Pseudomonadati Pseudomonadota Gammaproteobacteria Lysobacterales Rhodanobacteraceae bacterium HET6631309.1 320 aa
Pseudomonadati Pseudomonadota Gammaproteobacteria Lysobacterales Xanthomonas hortorum PUF01471.1 372 aa
Pseudomonadati Pseudomonadota Gammaproteobacteria Lysobacterales Mizugakiibacter sp. HEN46795.1 320 aa
Pseudomonadati Pseudomonadota Gammaproteobacteria Lysobacterales Oleiagrimonas sp. WP 206064329.1 322 aa
Pseudomonadati Pseudomonadota Gammaproteobacteria Lysobacterales Rhodanobacteraceae bacterium HET7358591.1 318 aa
Pseudomonadati Pseudomonadota Gammaproteobacteria Lysobacterales Oleiagrimonas sp. HET7299428.1 328 aa
Pseudomonadati Pseudomonadota Gammaproteobacteria Lysobacterales Oleiagrimonas soli WP 268746046.1 318 aa
Pseudomonadati Pseudomonadota Gammaproteobacteria Lysobacterales Dyella choica WP 126686881.1 312 aa
Pseudomonadati Pseudomonadota Gammaproteobacteria Lysobacterales Frateuria aurantia WP 014402086.1 312 aa
Pseudomonadati Pseudomonadota Gammaproteobacteria Lysobacterales Aerosticca soli WP 126540032.1 312 aa
Pseudomonadati Pseudomonadota Gammaproteobacteria Lysobacterales Lysobacterales bacterium MBN8713179.1 312 aa
Pseudomonadati Pseudomonadota Gammaproteobacteria Lysobacterales unclassified Rhodanobacter WP 179479731.1 313 aa
Pseudomonadati Pseudomonadota Gammaproteobacteria Lysobacterales Rhodanobacter sp. WP 192165960.1 313 aa
Pseudomonadati Pseudomonadota Gammaproteobacteria Lysobacterales Rhodanobacter sp. WP 077481416.1 312 aa
Pseudomonadati Pseudomonadota Gammaproteobacteria Natronospirales Natronospira proteinivora WP 253447676.1 318 aa
Pseudomonadati Pseudomonadota Gammaproteobacteria Chromatiales Wenzhouxiangella sp. WP 165281238.1 312 aa
Pseudomonadati Pseudomonadota Gammaproteobacteria Woeseiales Lentisalinibacter WP 405223802.1 312 aa
Pseudomonadati Pseudomonadota Gammaproteobacteria Woeseiales Woeseia sp. MFQ5546645.1 317 aa
Pseudomonadati Pseudomonadota Gammaproteobacteria Woeseiales Woeseia oceani WP 068619312.1 312 aa
Pseudomonadati Pseudomonadota Gammaproteobacteria Steroidobacterales Steroidobacteraceae bacterium MBF8291367.1 317 aa
Pseudomonadati Pseudomonadota Gammaproteobacteria Steroidobacterales Steroidobacteraceae bacterium MGH8188955.1 317 aa
Pseudomonadati Pseudomonadota Gammaproteobacteria Lysobacterales Lysobacteraceae bacterium NLG74948.1 312 aa
Pseudomonadati Pseudomonadota Gammaproteobacteria Steroidobacterales Steroidobacteraceae bacterium MGC4028560.1 317 aa
Pseudomonadati Pseudomonadota Gammaproteobacteria Steroidobacterales Steroidobacteraceae bacterium MET0291340.1 317 aa
Pseudomonadati Pseudomonadota Gammaproteobacteria Steroidobacterales Steroidobacteraceae bacterium HWG77290.1 318 aa
Pseudomonadati Pseudomonadota Gammaproteobacteria Steroidobacterales Steroidobacteraceae bacterium MGB8694225.1 312 aa
Pseudomonadati Pseudomonadota Gammaproteobacteria Steroidobacterales Steroidobacteraceae bacterium MFO1427430.1 312 aa
Pseudomonadati Pseudomonadota Gammaproteobacteria Steroidobacterales Steroidobacteraceae bacterium MEZ5500267.1 312 aa
Pseudomonadati Pseudomonadota Gammaproteobacteria Steroidobacterales Steroidobacteraceae bacterium MGH8148843.1 318 aa
Pseudomonadati Pseudomonadota Gammaproteobacteria Steroidobacterales Steroidobacteraceae bacterium HXS29170.1 317 aa
Pseudomonadati Pseudomonadota Gammaproteobacteria Steroidobacterales Steroidobacteraceae bacterium HXR92729.1 312 aa
Pseudomonadati Pseudomonadota Gammaproteobacteria Steroidobacterales Steroidobacteraceae bacterium MFO1455885.1 312 aa
Pseudomonadati Pseudomonadota Gammaproteobacteria Steroidobacterales Steroidobacteraceae bacterium HZF16761.1 317 aa
Pseudomonadati Pseudomonadota Gammaproteobacteria Steroidobacterales Steroidobacteraceae bacterium MGB8328314.1 317 aa
Pseudomonadati Pseudomonadota Gammaproteobacteria Steroidobacterales Steroidobacteraceae bacterium HPF26882.1 318 aa
Pseudomonadati Pseudomonadota Gammaproteobacteria Chromatiales Chromatiaceae bacterium HIB84240.1 306 aa
Pseudomonadati Pseudomonadota Gammaproteobacteria Methylococcales Crenotrichaceae bacterium HFD10950.1 311 aa
Pseudomonadati Pseudomonadota Gammaproteobacteria Nitrococcales Methylonatrum kenyense WP 248568158.1 312 aa
Pseudomonadati Pseudomonadota Gammaproteobacteria Chromatiales Aquisalimonas lutea WP 290202652.1 312 aa
Pseudomonadati Pseudomonadota Gammaproteobacteria Chromatiales Alkalilimnicola sp. WP 193222265.1 312 aa
Pseudomonadati Pseudomonadota Gammaproteobacteria Chromatiales Alkalilimnicola ehrlichii WP 116300616.1 312 aa
Pseudomonadati Pseudomonadota Gammaproteobacteria Chromatiales Halorhodospira halophila WP 041595063.1 312 aa
Pseudomonadati Pseudomonadota Gammaproteobacteria Chromatiales Alkalispirillum mobile WP 281273349.1 318 aa
Pseudomonadati Pseudomonadota Gammaproteobacteria Chromatiales Ectothiorhodospiraceae bacterium MBA1146214.1 312 aa
Pseudomonadati Pseudomonadota Gammaproteobacteria Chromatiales Nitrococcus mobilis WP 005005099.1 312 aa
Pseudomonadati Pseudomonadota Gammaproteobacteria Chromatiales Arhodomonas WP 026321216.1 312 aa
Pseudomonadati Pseudomonadota Gammaproteobacteria Chromatiales Spiribacter sp. WP 195759488.1 317 aa
Pseudomonadati Pseudomonadota Gammaproteobacteria Chromatiales Spiribacter onubensis WP 367965949.1 317 aa
Pseudomonadati Pseudomonadota Gammaproteobacteria Chromatiales Lamprocystis purpurea WP 026199837.1 317 aa
Pseudomonadati Pseudomonadota Gammaproteobacteria Chromatiales Candidatus Thiosymbion MCB2264458.1 312 aa
Pseudomonadati Pseudomonadota Gammaproteobacteria Chromatiales Thiocystis minor WP 338115352.1 317 aa
Pseudomonadati Pseudomonadota Gammaproteobacteria Chromatiales Thiorhodococcus fuscus WP 386023017.1 317 aa
Pseudomonadati Pseudomonadota Gammaproteobacteria Chromatiales Marichromatium WP 123141127.1 317 aa
Pseudomonadati Pseudomonadota Gammaproteobacteria Chromatiales Thioflavicoccus mobilis WP 015281945.1 317 aa
Pseudomonadati Pseudomonadota Gammaproteobacteria Chromatiales Thiospirillum jenense WP 182584496.1 317 aa
Pseudomonadati Pseudomonadota Gammaproteobacteria Chromatiales Rhabdochromatium marinum WP 338053428.1 317 aa
Pseudomonadati Pseudomonadota Gammaproteobacteria Chromatiales Thiohalocapsa halophila WP 200241251.1 317 aa
Pseudomonadati Pseudomonadota Gammaproteobacteria Chromatiales Halochromatium sp. MFW6259223.1 317 aa
Pseudomonadati Pseudomonadota Gammaproteobacteria Chromatiales Halochromatium salexigens WP 338073139.1 317 aa
Pseudomonadati Pseudomonadota Gammaproteobacteria Thiotrichales Achromatium sp. KOR30876.1 317 aa
Pseudomonadati Pseudomonadota Gammaproteobacteria Chromatiales Thiosocius teredinicola WP 174575421.1 312 aa
Pseudomonadati Pseudomonadota Gammaproteobacteria Chromatiales Sedimenticolaceae bacterium MGA0937478.1 317 aa
Pseudomonadati Pseudomonadota Gammaproteobacteria Chromatiales Candidatus Thiodiazotropha WP 201258728.1 312 aa
Pseudomonadati Pseudomonadota Gammaproteobacteria Thiotrichales Achromatium sp. KOR32096.1 311 aa
Pseudomonadati Pseudomonadota Gammaproteobacteria Chromatiales Magnetovirga frankeli WP 153294868.1 317 aa
Pseudomonadati Pseudomonadota Gammaproteobacteria Gammaproteobacteria incertae sedis Candidatus Vondammii WP 320416332.1 317 aa
Pseudomonadati Pseudomonadota Gammaproteobacteria Chromatiales Sedimenticola sp. MES9857537.1 317 aa
Pseudomonadati Pseudomonadota Gammaproteobacteria Chromatiales Candidatus Sedimenticola MEW7980195.1 317 aa
Pseudomonadati Pseudomonadota Gammaproteobacteria Chromatiales Sedimenticola selenatireducens WP 029134777.1 317 aa
Pseudomonadati Pseudomonadota Gammaproteobacteria Chromatiales Sedimenticola thiotaurini HEB97129.1 317 aa
Pseudomonadati Pseudomonadota Gammaproteobacteria Chromatiales Sedimenticola sp. HHH39115.1 317 aa
Pseudomonadati Pseudomonadota Gammaproteobacteria Thiohalomonadales Thiohalophilus thiocyanatoxydans WP 134085361.1 312 aa
Pseudomonadati Pseudomonadota Gammaproteobacteria Chromatiales Thioprofundum lithotrophicum BGR12173.1 321 aa
Pseudomonadati Pseudomonadota Gammaproteobacteria Thiohalomonadales Thiohalomonadales bacterium MFV2058738.1 324 aa
Pseudomonadati Pseudomonadota Gammaproteobacteria Chromatiales Ectothiorhodospiraceae bacterium MBL1276808.1 312 aa
Pseudomonadati Pseudomonadota Gammaproteobacteria Candidatus Tenderiales Candidatus Tenderia HGX93850.1 319 aa
Pseudomonadati Pseudomonadota Gammaproteobacteria Candidatus Tenderiales Candidatus Tenderia ALP54384.1 313 aa
Pseudomonadati Pseudomonadota Gammaproteobacteria Chromatiales Thioalbus denitrificans WP 114278075.1 311 aa
Pseudomonadati Pseudomonadota Gammaproteobacteria Thiohalomonadales Thiohalomonadaceae bacterium MFA5531369.1 312 aa
Pseudomonadati Pseudomonadota Gammaproteobacteria Gammaproteobacteria incertae sedis Candidatus Reidiella WP 078484886.1 314 aa
Pseudomonadati Pseudomonadota Gammaproteobacteria Lysobacterales Lysobacteraceae bacterium MCA1804514.1 312 aa
Pseudomonadati Pseudomonadota Gammaproteobacteria Chromatiales Endothiovibrio diazotrophicus GAB6041720.1 317 aa
Pseudomonadati Pseudomonadota Gammaproteobacteria Chromatiales Thiogranum sp. MGI9320034.1 287 aa
Pseudomonadati Pseudomonadota Gammaproteobacteria Chromatiales Thiogranum sp. MDT8386464.1 324 aa
Pseudomonadati Pseudomonadota Gammaproteobacteria Chromatiales Candidatus Nitrosoglobus WP 172419116.1 311 aa
Pseudomonadati Pseudomonadota Gammaproteobacteria Chromatiales Nitrosococcus watsonii WP 041350744.1 311 aa
Pseudomonadati Pseudomonadota Gammaproteobacteria Chromatiales Candidatus Nitrosacidococcus WP 197745369.1 311 aa
Pseudomonadati Pseudomonadota Gammaproteobacteria Thiotrichales Methylophaga sp. WP 266277502.1 311 aa
Pseudomonadati Pseudomonadota Gammaproteobacteria Thiotrichales Methylophaga sp. NOQ81452.1 328 aa
Pseudomonadati Pseudomonadota Gammaproteobacteria Thiotrichales Piscirickettsiaceae bacterium NQY51843.1 311 aa
Pseudomonadati Pseudomonadota Gammaproteobacteria Nitrosococcales Methylophagaceae bacterium MFT7413496.1 328 aa
Pseudomonadati Pseudomonadota Gammaproteobacteria Nitrosococcales Methylophagaceae bacterium MFT7235496.1 328 aa
Pseudomonadati Pseudomonadota Gammaproteobacteria Chromatiales Inmirania thermothiophila WP 123400288.1 312 aa
Pseudomonadati Pseudomonadota Gammaproteobacteria Thiotrichales Candidatus Venteria WP 103921280.1 312 aa
Pseudomonadati Pseudomonadota Gammaproteobacteria Thiotrichales Beggiatoa alba WP 040294960.1 312 aa
Pseudomonadati Pseudomonadota Gammaproteobacteria Thiotrichales Thioflexithrix psekupsensis WP 086487475.1 311 aa
Pseudomonadati Pseudomonadota Gammaproteobacteria Thiotrichales Candidatus Albibeggiatoa WP 353570917.1 312 aa
Pseudomonadati Pseudomonadota Gammaproteobacteria Thiotrichales Beggiatoa sp. OQW93404.1 312 aa
Pseudomonadati Pseudomonadota Gammaproteobacteria Thiotrichales Thioploca ingrica BAP56605.1 311 aa
Pseudomonadati Pseudomonadota Gammaproteobacteria Thiotrichales Candidatus Marithrix WP 069469964.1 312 aa
Pseudomonadati Pseudomonadota Gammaproteobacteria Thiotrichales Thiomargarita sp. MCK5717343.1 312 aa
Pseudomonadati Pseudomonadota Gammaproteobacteria Thiotrichales Candidatus Thiomargarita KHD06242.1 312 aa
Pseudomonadati Pseudomonadota Gammaproteobacteria Thiotrichales Candidatus Parabeggiatoa OQY54235.1 312 aa
Pseudomonadati Pseudomonadota Gammaproteobacteria Thiotrichales Candidatus Parabeggiatoa WP 349648268.1 312 aa
Pseudomonadati Pseudomonadota Gammaproteobacteria Thiotrichales Thiomargarita sp. MCK5720235.1 324 aa
Pseudomonadati Pseudomonadota Gammaproteobacteria Thiotrichales Thioploca sp. HHB92329.1 312 aa
Pseudomonadati Pseudomonadota Gammaproteobacteria Thiotrichales Thiomargarita sp. MCK5524763.1 312 aa
Pseudomonadati Pseudomonadota Gammaproteobacteria Immundisolibacterales Immundisolibacterales bacterium MBL4622590.1 317 aa
Pseudomonadati Pseudomonadota Gammaproteobacteria Immundisolibacterales Immundisolibacteraceae bacterium MDF1819676.1 317 aa
Pseudomonadati Pseudomonadota Gammaproteobacteria Chromatiales Halofilum sp. MDX1609882.1 312 aa
Pseudomonadati Pseudomonadota Gammaproteobacteria Chromatiales Halofilum ochraceum WP 067561181.1 312 aa
Pseudomonadati Pseudomonadota Gammaproteobacteria Chromatiales Thiofaba sp. WP 347317626.1 313 aa
Pseudomonadati Pseudomonadota Gammaproteobacteria Chromatiales Halothiobacillaceae bacterium MDD3609111.1 318 aa
Pseudomonadati Pseudomonadota Gammaproteobacteria Chromatiales Guyparkeria WP 058573997.1 319 aa
Pseudomonadati Pseudomonadota Gammaproteobacteria Chromatiales Granulosicoccaceae bacterium MEE9334368.1 317 aa
Pseudomonadati Pseudomonadota Gammaproteobacteria Chromatiales Granulosicoccus sp. MEE9319072.1 317 aa
Pseudomonadati Pseudomonadota Gammaproteobacteria Chromatiales Granulosicoccus antarcticus WP 169727439.1 317 aa
Pseudomonadati Pseudomonadota Gammaproteobacteria Ectothiorhodospirales Thioalkalivibrionaceae bacterium MFN4263881.1 318 aa
Pseudomonadati Pseudomonadota Gammaproteobacteria Chromatiales Thioalkalivibrio WP 018947433.1 318 aa
Pseudomonadati Pseudomonadota Gammaproteobacteria Chromatiales Thiorhodospira sibirica WP 174269466.1 312 aa
Pseudomonadati Pseudomonadota Gammaproteobacteria Chromatiales Ectothiorhodospiraceae bacterium MDX9767020.1 318 aa
Pseudomonadati Pseudomonadota Gammaproteobacteria Thiotrichales Thiothrix sp. RUM93592.1 319 aa
Pseudomonadati Pseudomonadota Gammaproteobacteria Chromatiales Acidihalobacter aeolianus WP 070073538.1 312 aa
Pseudomonadati Pseudomonadota Gammaproteobacteria Thiotrichales Leucothrix mucor HFC92592.1 321 aa
Pseudomonadati Pseudomonadota Gammaproteobacteria Thiotrichales Leucothrix mucor HIO91432.1 321 aa
Pseudomonadati Pseudomonadota Gammaproteobacteria Thiotrichales Cocleimonas sp. MCK5903344.1 316 aa
Pseudomonadati Pseudomonadota Gammaproteobacteria Thiotrichales Cocleimonas sp. MGK0272279.1 323 aa
Pseudomonadati Pseudomonadota Gammaproteobacteria Thiotrichales Cocleimonas sp. MEE9445329.1 316 aa
Pseudomonadati Pseudomonadota Gammaproteobacteria Thiotrichales Leucothrix sp. HIP95522.1 317 aa
Pseudomonadati Pseudomonadota Gammaproteobacteria Thiotrichales Cocleimonas sp. MCF6190946.1 316 aa
Pseudomonadati Pseudomonadota Gammaproteobacteria Thiotrichales Leucothrix pacifica WP 425451307.1 319 aa
Pseudomonadati Pseudomonadota Gammaproteobacteria Thiotrichales Leucothrix arctica WP 425451386.1 319 aa
Pseudomonadati Pseudomonadota Gammaproteobacteria Thiotrichales uncultured Thiotrichaceae CAA6814652.1 323 aa
Pseudomonadati Pseudomonadota Gammaproteobacteria Thiotrichales Thiothrix sp. HHL18432.1 315 aa
Pseudomonadati Pseudomonadota Gammaproteobacteria Thiotrichales Thiotrichaceae bacterium MEE9303809.1 316 aa
Pseudomonadati Pseudomonadota Gammaproteobacteria Thiotrichales Thiotrichaceae bacterium MCK5726464.1 317 aa
Pseudomonadati Pseudomonadota Gammaproteobacteria Thiotrichales Thiothrix sp. HHC73878.1 319 aa
Pseudomonadati Pseudomonadota Gammaproteobacteria Thiotrichales Thiothrix winogradskyi WP 255697796.1 318 aa
Pseudomonadati Pseudomonadota Gammaproteobacteria Thiotrichales Thiofilum flexile WP 211209445.1 322 aa
Pseudomonadati Pseudomonadota Gammaproteobacteria Thiotrichales Thiothrix sp. MCB1619642.1 320 aa

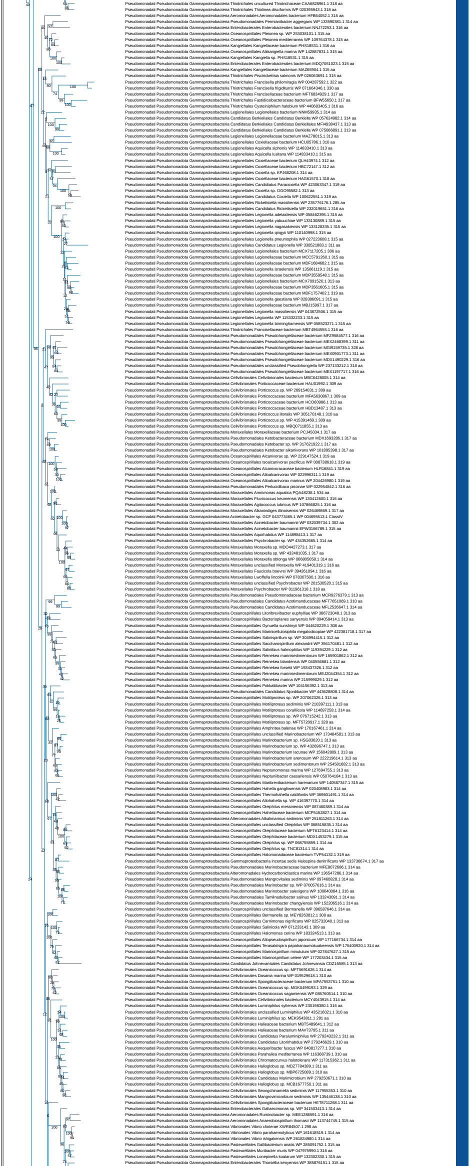

Pseudomonadati Pseudomonadota Gammaproteobacteria Thiotrichales uncultured Thiotrichaceae CAA6828961.1 318 aa
Pseudomonadati Pseudomonadota Gammaproteobacteria Thiotrichales Thiolinea disciformis WP 020395943.1 318 aa
Pseudomonadati Pseudomonadota Gammaproteobacteria Aeromonadales Aeromonadales bacterium HFB64052.1 315 aa
Pseudomonadati Pseudomonadota Gammaproteobacteria Pseudomonadales Permianibacter aggregans WP 133590381.1 314 aa
Pseudomonadati Pseudomonadota Gammaproteobacteria Enterobacterales Enterobacterales bacterium NNJ72253.1 316 aa
Pseudomonadati Pseudomonadota Gammaproteobacteria Oceanospirillales Pleionea sp. WP 253038101.1 315 aa
Pseudomonadati Pseudomonadota Gammaproteobacteria Oceanospirillales Pleionea mediterranea WP 109764378.1 315 aa
Pseudomonadati Pseudomonadota Gammaproteobacteria Kangiellales Kangiellaceae bacterium PHS18531.1 316 aa
Pseudomonadati Pseudomonadota Gammaproteobacteria Oceanospirillales Aliikangiella marina WP 142887831.1 315 aa
Pseudomonadati Pseudomonadota Gammaproteobacteria Kangiellales Kangiella sp. PHS18531.1 315 aa
Pseudomonadati Pseudomonadota Gammaproteobacteria Enterobacterales Enterobacterales bacterium MDQ7051023.1 315 aa
Pseudomonadati Pseudomonadota Gammaproteobacteria Kangiellales Kangiellaceae bacterium MAZ65904.1 315 aa
Pseudomonadati Pseudomonadota Gammaproteobacteria Thiotrichales Piscirickettsia salmonis WP 026063691.1 315 aa
Pseudomonadati Pseudomonadota Gammaproteobacteria Thiotrichales Francisella philomiragia WP 004287592.1 322 aa
Pseudomonadati Pseudomonadota Gammaproteobacteria Thiotrichales Francisella frigiditurris WP 071664346.1 330 aa
Pseudomonadati Pseudomonadota Gammaproteobacteria Thiotrichales Francisellaceae bacterium MFT6834929.1 317 aa
Pseudomonadati Pseudomonadota Gammaproteobacteria Thiotrichales Fastidiosibacteraceae bacterium BFW55650.1 317 aa
Pseudomonadati Pseudomonadota Gammaproteobacteria Thiotrichales Cysteiniphilum halobium WP 440683405.1 316 aa
Pseudomonadati Pseudomonadota Gammaproteobacteria Legionellales Legionellales bacterium NNM59935.1 314 aa
Pseudomonadati Pseudomonadota Gammaproteobacteria Candidatus Berkiellales Candidatus Berkiella WP 057624982.1 314 aa
Pseudomonadati Pseudomonadota Gammaproteobacteria Candidatus Berkiellales Candidatus Berkiellales MFI4938437.1 313 aa
Pseudomonadati Pseudomonadota Gammaproteobacteria Candidatus Berkiellales Candidatus Berkiella WP 075066891.1 313 aa
Pseudomonadati Pseudomonadota Gammaproteobacteria Legionellales Legionellaceae bacterium MAZ78015.1 313 aa
Pseudomonadati Pseudomonadota Gammaproteobacteria Legionellales Coxiellaceae bacterium HCU05786.1 310 aa
Pseudomonadati Pseudomonadota Gammaproteobacteria Legionellales Aquicella siphonis WP 114833410.1 313 aa
Pseudomonadati Pseudomonadota Gammaproteobacteria Legionellales Aquicella lusitana WP 114833410.1 315 aa
Pseudomonadati Pseudomonadota Gammaproteobacteria Legionellales Coxiellaceae bacterium QLH43974.1 312 aa
Pseudomonadati Pseudomonadota Gammaproteobacteria Legionellales Coxiellaceae bacterium HBC72147.1 312 aa
Pseudomonadati Pseudomonadota Gammaproteobacteria Legionellales Coxiella sp. KPJ68208.1 314 aa
Pseudomonadati Pseudomonadota Gammaproteobacteria Legionellales Coxiellaceae bacterium HAG61570.1 318 aa
Pseudomonadati Pseudomonadota Gammaproteobacteria Legionellales Candidiatus Paracoxiella WP 423063347.1 319 aa
Pseudomonadati Pseudomonadota Gammaproteobacteria Legionellales Coxiella sp. OGO95582.1 313 aa
Pseudomonadati Pseudomonadota Gammaproteobacteria Legionellales Candidatus Coxiella WP 100622551.1 319 aa
Pseudomonadati Pseudomonadota Gammaproteobacteria Legionellales Rickettsiella massiliensis WP 235776176.1 285 aa
Pseudomonadati Pseudomonadota Gammaproteobacteria Legionellales Candidatus Rickettsiella WP 232019651.1 316 aa
Pseudomonadati Pseudomonadota Gammaproteobacteria Legionellales Legionella adelaidensis WP 058462395.1 315 aa
Pseudomonadati Pseudomonadota Gammaproteobacteria Legionellales Legionella yabuuchiae WP 133130889.1 315 aa
Pseudomonadati Pseudomonadota Gammaproteobacteria Legionellales Legionella nagasakiensis WP 133128335.1 315 aa
Pseudomonadati Pseudomonadota Gammaproteobacteria Legionellales Legionella qingyii WP 110140998.1 315 aa
Pseudomonadati Pseudomonadota Gammaproteobacteria Legionellales Legionella pneumophila WP 027223608.1 315 aa
Pseudomonadati Pseudomonadota Gammaproteobacteria Legionellales Candidatus Legionella WP 338521883.1 311 aa
Pseudomonadati Pseudomonadota Gammaproteobacteria Legionellales Legionellales bacterium MCX7117205.1 306 aa
Pseudomonadati Pseudomonadota Gammaproteobacteria Legionellales Legionellaceae bacterium MCC5791260.1 315 aa
Pseudomonadati Pseudomonadota Gammaproteobacteria Legionellales Legionellaceae bacterium MDF1684682.1 315 aa
Pseudomonadati Pseudomonadota Gammaproteobacteria Legionellales Legionella israelensis WP 135061119.1 315 aa
Pseudomonadati Pseudomonadota Gammaproteobacteria Legionellales Legionellaceae bacterium MDP3559548.1 315 aa
Pseudomonadati Pseudomonadota Gammaproteobacteria Legionellales Legionellales bacterium MCX7091520.1 313 aa
Pseudomonadati Pseudomonadota Gammaproteobacteria Legionellales Legionellaceae bacterium MDP3561605.1 315 aa
Pseudomonadati Pseudomonadota Gammaproteobacteria Legionellales Legionellaceae bacterium MDF1757402.1 319 aa
Pseudomonadati Pseudomonadota Gammaproteobacteria Legionellales Legionella geestiana WP 028386091.1 315 aa
Pseudomonadati Pseudomonadota Gammaproteobacteria Legionellales Legionellaceae bacterium MBJ15997.1 317 aa
Pseudomonadati Pseudomonadota Gammaproteobacteria Legionellales Legionella massiliensis WP 043872506.1 315 aa
Pseudomonadati Pseudomonadota Gammaproteobacteria Legionellales Legionella WP 115332233.1 315 aa
Pseudomonadati Pseudomonadota Gammaproteobacteria Legionellales Legionella birminghamensis WP 058523271.1 315 aa
Pseudomonadati Pseudomonadota Gammaproteobacteria Thiotrichales Francisellaceae bacterium MBT4964555.1 316 aa
Pseudomonadati Pseudomonadota Gammaproteobacteria Pseudomonadales Pseudohongiellaceae bacterium MFZ9584577.1 316 aa
Pseudomonadati Pseudomonadota Gammaproteobacteria Pseudomonadales Pseudohongiellaceae bacterium MEX2468399.1 311 aa
Pseudomonadati Pseudomonadota Gammaproteobacteria Pseudomonadales Pseudohongiellaceae bacterium MGI9249735.1 328 aa
Pseudomonadati Pseudomonadota Gammaproteobacteria Pseudomonadales Pseudohongiellaceae bacterium MEX0901773.1 311 aa
Pseudomonadati Pseudomonadota Gammaproteobacteria Pseudomonadales Pseudohongiellaceae bacterium MDX1490229.1 316 aa
Pseudomonadati Pseudomonadota Gammaproteobacteria Pseudomonadales unclassified Pseudohongiella WP 237133212.1 318 aa
Pseudomonadati Pseudomonadota Gammaproteobacteria Pseudomonadales Pseudohongiellaceae bacterium MEX1197717.1 316 aa
Pseudomonadati Pseudomonadota Gammaproteobacteria Cellvibrionales Cellvibrionales bacterium MBC6428005.1 314 aa
Pseudomonadati Pseudomonadota Gammaproteobacteria Cellvibrionales Porticoccaceae bacterium HAU01992.1 309 aa
Pseudomonadati Pseudomonadota Gammaproteobacteria Cellvibrionales Porticoccus sp. WP 289154031.1 309 aa
Pseudomonadati Pseudomonadota Gammaproteobacteria Cellvibrionales Porticoccaceae bacterium MFA5630867.1 309 aa
Pseudomonadati Pseudomonadota Gammaproteobacteria Cellvibrionales Porticoccaceae bacterium HCO60986.1 313 aa
Pseudomonadati Pseudomonadota Gammaproteobacteria Cellvibrionales Porticoccaceae bacterium HBD13487.1 313 aa
Pseudomonadati Pseudomonadota Gammaproteobacteria Cellvibrionales Porticoccus litoralis WP 305170148.1 310 aa
Pseudomonadati Pseudomonadota Gammaproteobacteria Cellvibrionales Porticoccus sp. WP 415391469.1 309 aa
Pseudomonadati Pseudomonadota Gammaproteobacteria Cellvibrionales Porticoccus sp. MBQ0711855.1 313 aa
Pseudomonadati Pseudomonadota Gammaproteobacteria Moraxellales Moraxellaceae bacterium PCJ45034.1 317 aa
Pseudomonadati Pseudomonadota Gammaproteobacteria Pseudomonadales Ketobacteraceae bacterium MDX1693286.1 317 aa
Pseudomonadati Pseudomonadota Gammaproteobacteria Pseudomonadales Ketobacter sp. WP 317621922.1 317 aa
Pseudomonadati Pseudomonadota Gammaproteobacteria Pseudomonadales Ketobacter alkanivorans WP 101895398.1 317 aa
Pseudomonadati Pseudomonadota Gammaproteobacteria Oceanospirillales Alcanivorax sp. WP 229147524.1 319 aa
Pseudomonadati Pseudomonadota Gammaproteobacteria Oceanospirillales Isoalcanivorax pacificus WP 008738618.1 319 aa
Pseudomonadati Pseudomonadota Gammaproteobacteria Oceanospirillales Alcanivoracaceae bacterium HLR16841.1 319 aa
Pseudomonadati Pseudomonadota Gammaproteobacteria Oceanospirillales Alloalcanivorax WP 022996311.1 319 aa
Pseudomonadati Pseudomonadota Gammaproteobacteria Oceanospirillales Alloalcanivorax marinus WP 204426980.1 319 aa
Pseudomonadati Pseudomonadota Gammaproteobacteria Pseudomonadales Perlucidibaca piscinae WP 022954842.1 316 aa
Pseudomonadati Pseudomonadota Gammaproteobacteria Moraxellales Amnimonas aquatica PQA48238.1 534 aa
Pseudomonadati Pseudomonadota Gammaproteobacteria Moraxellales Fluviicoccus keumensis WP 130412600.1 316 aa
Pseudomonadati Pseudomonadota Gammaproteobacteria Moraxellales Agitococcus lubricus WP 107866825.1 316 aa
Pseudomonadati Pseudomonadota Gammaproteobacteria Moraxellales Alkanindiges illinoisensis WP 026469869.1 317 aa
Pseudomonadati Pseudomonadota Gammaproteobacteria Acinetobacter sp. GCF 043773465.1 WP 004695513.1 ClassIV
Pseudomonadati Pseudomonadota Gammaproteobacteria Moraxellales Acinetobacter baumannii WP 032039734.1 302 aa
Pseudomonadati Pseudomonadota Gammaproteobacteria Moraxellales Acinetobacter baumannii EPW3166789.1 315 aa
Pseudomonadati Pseudomonadota Gammaproteobacteria Moraxellales Aquirhabdus WP 114898413.1 317 aa
Pseudomonadati Pseudomonadota Gammaproteobacteria Moraxellales Psychrobacter sp. WP 434352665.1 314 aa
Pseudomonadati Pseudomonadota Gammaproteobacteria Moraxellales Moraxella sp. MDO4427273.1 317 aa
Pseudomonadati Pseudomonadota Gammaproteobacteria Moraxellales Moraxella sp. WP 432481035.1 317 aa
Pseudomonadati Pseudomonadota Gammaproteobacteria Moraxellales Moraxella oblonga WP 066805058.1 314 aa
Pseudomonadati Pseudomonadota Gammaproteobacteria Moraxellales unclassified Moraxella WP 419401319.1 316 aa
Pseudomonadati Pseudomonadota Gammaproteobacteria Moraxellales Faucicola boevrei WP 394261094.1 316 aa
Pseudomonadati Pseudomonadota Gammaproteobacteria Moraxellales Lwoffella lincolnii WP 078307500.1 316 aa
Pseudomonadati Pseudomonadota Gammaproteobacteria Moraxellales unclassified Psychrobacter WP 201530520.1 315 aa
Pseudomonadati Pseudomonadota Gammaproteobacteria Moraxellales Psychrobacter WP 011961318.1 318 aa
Pseudomonadati Pseudomonadota Gammaproteobacteria Pseudomonadales Pseudomonadaceae bacterium MCR9276379.1 313 aa
Pseudomonadati Pseudomonadota Gammaproteobacteria Pseudomonadales Candidatus Azotimanducaceae MFT7651069.1 310 aa
Pseudomonadati Pseudomonadota Gammaproteobacteria Pseudomonadales Candidatus Azotimanducaceae MFL2526647.1 314 aa
Pseudomonadati Pseudomonadota Gammaproteobacteria Oceanospirillales Litoribrevibacter euphylliae WP 386723048.1 313 aa
Pseudomonadati Pseudomonadota Gammaproteobacteria Oceanospirillales Bacterioplanes sanyensis WP 094058414.1 313 aa
Pseudomonadati Pseudomonadota Gammaproteobacteria Oceanospirillales Gynuella sunshinyii WP 044620229.1 308 aa
Pseudomonadati Pseudomonadota Gammaproteobacteria Oceanospirillales Marinicellulosiphila megalodicopiae WP 422381718.1 317 aa
Pseudomonadati Pseudomonadota Gammaproteobacteria Oceanospirillales Salinispirillum sp. WP 304994415.1 312 aa
Pseudomonadati Pseudomonadota Gammaproteobacteria Oceanospirillales Saccharospirillum alexandrii WP 394170481.1 312 aa
Pseudomonadati Pseudomonadota Gammaproteobacteria Oceanospirillales Salinibius halmophilus WP 119394229.1 312 aa
Pseudomonadati Pseudomonadota Gammaproteobacteria Oceanospirillales Reinekea marinisedimentorum WP 165901862.1 312 aa
Pseudomonadati Pseudomonadota Gammaproteobacteria Oceanospirillales Reinekea blandensis WP 040556681.1 312 aa
Pseudomonadati Pseudomonadota Gammaproteobacteria Oceanospirillales Reinekea forsetii WP 193437326.1 312 aa
Pseudomonadati Pseudomonadota Gammaproteobacteria Oceanospirillales Reinekea marinisedimentorum MEJ2044354.1 312 aa
Pseudomonadati Pseudomonadota Gammaproteobacteria Oceanospirillales Reinekea marina WP 215999029.1 312 aa
Pseudomonadati Pseudomonadota Gammaproteobacteria Oceanospirillales Pokkaliibacter WP 104156392.1 313 aa
Pseudomonadati Pseudomonadota Gammaproteobacteria Pseudomonadales Candidatus Njordibacter WP 443628808.1 314 aa
Pseudomonadati Pseudomonadota Gammaproteobacteria Oceanospirillales Motiliproteus sp. WP 207062326.1 313 aa
Pseudomonadati Pseudomonadota Gammaproteobacteria Oceanospirillales Motiliproteus sediminis WP 210397111.1 313 aa
Pseudomonadati Pseudomonadota Gammaproteobacteria Oceanospirillales Motiliproteus coralliicola WP 114697258.1 314 aa
Pseudomonadati Pseudomonadota Gammaproteobacteria Oceanospirillales Motiliproteus sp. WP 076715242.1 313 aa
Pseudomonadati Pseudomonadota Gammaproteobacteria Oceanospirillales Motiliproteus sp. MFT5720917.1 328 aa
Pseudomonadati Pseudomonadota Gammaproteobacteria Oceanospirillales Amphritea balenae WP 170167461.1 314 aa
Pseudomonadati Pseudomonadota Gammaproteobacteria Oceanospirillales unclassified Marinobacterium WP 173484581.1 313 aa
Pseudomonadati Pseudomonadota Gammaproteobacteria Oceanospirillales Marinobacterium sp. HSG03620.1 313 aa
Pseudomonadati Pseudomonadota Gammaproteobacteria Oceanospirillales Marinobacterium sp. WP 432696747.1 313 aa
Pseudomonadati Pseudomonadota Gammaproteobacteria Oceanospirillales Marinobacterium lacunae WP 156042809.1 313 aa
Pseudomonadati Pseudomonadota Gammaproteobacteria Oceanospirillales Marinobacterium arenosum WP 222219614.1 313 aa
Pseudomonadati Pseudomonadota Gammaproteobacteria Oceanospirillales Marinobacterium sedimentorum WP 254581682.1 313 aa
Pseudomonadati Pseudomonadota Gammaproteobacteria Oceanospirillales Neptunomonas marina WP 127694755.1 313 aa
Pseudomonadati Pseudomonadota Gammaproteobacteria Oceanospirillales Neptuniibacter caesariensis WP 050764184.1 313 aa
Pseudomonadati Pseudomonadota Gammaproteobacteria Oceanospirillales Maribrevibacterium harenarium WP 140587347.1 315 aa
Pseudomonadati Pseudomonadota Gammaproteobacteria Oceanospirillales Hahella ganghwensis WP 020408983.1 314 aa
Pseudomonadati Pseudomonadota Gammaproteobacteria Oceanospirillales Thermohahella caldifontis WP 369601491.1 314 aa
Pseudomonadati Pseudomonadota Gammaproteobacteria Oceanospirillales Allohahella sp. WP 416397770.1 314 aa
Pseudomonadati Pseudomonadota Gammaproteobacteria Oceanospirillales Oleiphilus messinensis WP 087460389.1 314 aa
Pseudomonadati Pseudomonadota Gammaproteobacteria Oceanospirillales Hahellaceae bacterium MCP5162827.1 314 aa
Pseudomonadati Pseudomonadota Gammaproteobacteria Alteromonadales Alkalimarinus sediminis WP 251811263.1 314 aa
Pseudomonadati Pseudomonadota Gammaproteobacteria Oceanospirillales unclassified Oleiphilus WP 068515835.1 314 aa
Pseudomonadati Pseudomonadota Gammaproteobacteria Oceanospirillales Oleiphilaceae bacterium MFT6123414.1 314 aa
Pseudomonadati Pseudomonadota Gammaproteobacteria Oceanospirillales Oleiphilaceae bacterium MDX1453279.1 315 aa
Pseudomonadati Pseudomonadota Gammaproteobacteria Oceanospirillales Oleiphilus sp. WP 068755859.1 314 aa
Pseudomonadati Pseudomonadota Gammaproteobacteria Oceanospirillales Oleiphilus sp. TNC81314.1 314 aa
Pseudomonadati Pseudomonadota Gammaproteobacteria Oceanospirillales Halomonadaceae bacterium TVP54132.1 319 aa
Pseudomonadati Pseudomonadota Gammaproteobacteria Gammaproteobacteria incertae sedis Halospina denitrificans WP 133736674.1 317 aa
Pseudomonadati Pseudomonadota Gammaproteobacteria Pseudomonadales Marinobacteraceae bacterium MFE8072686.1 314 aa
Pseudomonadati Pseudomonadota Gammaproteobacteria Alteromonadales Hydrocarboniclastica marina WP 136547286.1 314 aa
Pseudomonadati Pseudomonadota Gammaproteobacteria Pseudomonadales Mangrovitalea sediminis WP 097460828.1 314 aa
Pseudomonadati Pseudomonadota Gammaproteobacteria Pseudomonadales Marinobacter sp. WP 070057616.1 314 aa
Pseudomonadati Pseudomonadota Gammaproteobacteria Pseudomonadales Marinobacter salexigens WP 100640084.1 316 aa
Pseudomonadati Pseudomonadota Gammaproteobacteria Pseudomonadales Tamilnaduibacter salinus WP 133243091.1 314 aa
Pseudomonadati Pseudomonadota Gammaproteobacteria Pseudomonadales Marinobacter changyiensis WP 152206516.1 314 aa
Pseudomonadati Pseudomonadota Gammaproteobacteria Oceanospirillales unclassified Bermanella WP 396587646.1 314 aa
Pseudomonadati Pseudomonadota Gammaproteobacteria Oceanospirillales Bermanella sp. MEY8263812.1 308 aa
Pseudomonadati Pseudomonadota Gammaproteobacteria Oceanospirillales Carnimonas nigrificans WP 025732040.1 313 aa
Pseudomonadati Pseudomonadota Gammaproteobacteria Oceanospirillales Salinicola WP 071233143.1 309 aa
Pseudomonadati Pseudomonadota Gammaproteobacteria Oceanospirillales Halomonas cerina WP 183324513.1 313 aa
Pseudomonadati Pseudomonadota Gammaproteobacteria Oceanospirillales Allopseudospirillum japonicum WP 177166734.1 314 aa
Pseudomonadati Pseudomonadota Gammaproteobacteria Oceanospirillales Terasakiispira papahanaumokuakeensis WP 175400920.1 314 aa
Pseudomonadati Pseudomonadota Gammaproteobacteria Oceanospirillales Marinospirillum minutulum WP 027847627.1 315 aa
Pseudomonadati Pseudomonadota Gammaproteobacteria Oceanospirillales Marinospirillum celere WP 177203434.1 315 aa
Pseudomonadati Pseudomonadota Gammaproteobacteria Candidatus Johnevansiales Candidatus Johnevansia CDZ16585.1 313 aa
Pseudomonadati Pseudomonadota Gammaproteobacteria Cellvibrionales Oceanicoccus sp. MFT5691626.1 314 aa
Pseudomonadati Pseudomonadota Gammaproteobacteria Cellvibrionales Dasania marina WP 019529618.1 310 aa
Pseudomonadati Pseudomonadota Gammaproteobacteria Cellvibrionales Spongiibacteraceae bacterium MFA7553751.1 310 aa
Pseudomonadati Pseudomonadota Gammaproteobacteria Cellvibrionales Oceanicoccus sp. MGK0499193.1 329 aa
Pseudomonadati Pseudomonadota Gammaproteobacteria Cellvibrionales Oceanicoccus sagamiensis WP 085760514.1 310 aa
Pseudomonadati Pseudomonadota Gammaproteobacteria Cellvibrionales Cellvibrionales bacterium MCY4043915.1 314 aa
Pseudomonadati Pseudomonadota Gammaproteobacteria Cellvibrionales Luminiphilus syltensis WP 230198380.1 316 aa
Pseudomonadati Pseudomonadota Gammaproteobacteria Cellvibrionales unclassified Luminiphilus WP 435216021.1 310 aa
Pseudomonadati Pseudomonadota Gammaproteobacteria Cellvibrionales Luminiphilus sp. MEK9543811.1 281 aa
Pseudomonadati Pseudomonadota Gammaproteobacteria Cellvibrionales Halieaceae bacterium MBT5489641.1 312 aa
Pseudomonadati Pseudomonadota Gammaproteobacteria Cellvibrionales Halieaceae bacterium MAV73795.1 311 aa
Pseudomonadati Pseudomonadota Gammaproteobacteria Cellvibrionales Candidatus Paraluminiphilus WP 279243232.1 311 aa
Pseudomonadati Pseudomonadota Gammaproteobacteria Cellvibrionales Candidatus Litorirhabdus WP 279246629.1 310 aa
Pseudomonadati Pseudomonadota Gammaproteobacteria Cellvibrionales Aequoribacter fuscus WP 040817277.1 310 aa
Pseudomonadati Pseudomonadota Gammaproteobacteria Cellvibrionales Parahaliea mediterranea WP 116368739.1 310 aa
Pseudomonadati Pseudomonadota Gammaproteobacteria Cellvibrionales Chromatocurvus halotolerans WP 117315362.1 311 aa
Pseudomonadati Pseudomonadota Gammaproteobacteria Cellvibrionales Halioglobus sp. MDZ7784389.1 311 aa
Pseudomonadati Pseudomonadota Gammaproteobacteria Cellvibrionales Halioglobus sp. MBP6725089.1 310 aa
Pseudomonadati Pseudomonadota Gammaproteobacteria Cellvibrionales Candidatus Marimicrobium WP 279250671.1 310 aa
Pseudomonadati Pseudomonadota Gammaproteobacteria Cellvibrionales Halioglobus sp. MCB1677750.1 311 aa
Pseudomonadati Pseudomonadota Gammaproteobacteria Cellvibrionales Seongchinamella sediminis WP 117955353.1 310 aa
Pseudomonadati Pseudomonadota Gammaproteobacteria Cellvibrionales Mangrovimicrobium sediminis WP 135446138.1 310 aa
Pseudomonadati Pseudomonadota Gammaproteobacteria Cellvibrionales Spongiibacteraceae bacterium HET8711268.1 311 aa
Pseudomonadati Pseudomonadota Gammaproteobacteria Enterobacterales Gallaecimonas sp. WP 341503413.1 314 aa
Pseudomonadati Pseudomonadota Gammaproteobacteria Aeromonadales Ruminobacter sp. MEE1286591.1 316 aa
Pseudomonadati Pseudomonadota Gammaproteobacteria Aeromonadales Anaerobiospirillum thomasii WP 113744745.1 315 aa
Pseudomonadati Pseudomonadota Gammaproteobacteria Vibrionales Vibrio cholerae XWR84507.1 298 aa
Pseudomonadati Pseudomonadota Gammaproteobacteria Vibrionales Vibrio parahaemolyticus WP 161618519.1 314 aa
Pseudomonadati Pseudomonadota Gammaproteobacteria Vibrionales Vibrio ishigakensis WP 261834980.1 314 aa
Pseudomonadati Pseudomonadota Gammaproteobacteria Pasteurellales Gallibacterium anatis WP 285091752.1 315 aa
Pseudomonadati Pseudomonadota Gammaproteobacteria Pasteurellales Muribacter muris WP 047975990.1 316 aa
Pseudomonadati Pseudomonadota Gammaproteobacteria Pasteurellales Lonepinella koalarum WP 132302330.1 315 aa
Pseudomonadati Pseudomonadota Gammaproteobacteria Enterobacterales Thorsellia kenyensis WP 385876151.1 315 aa

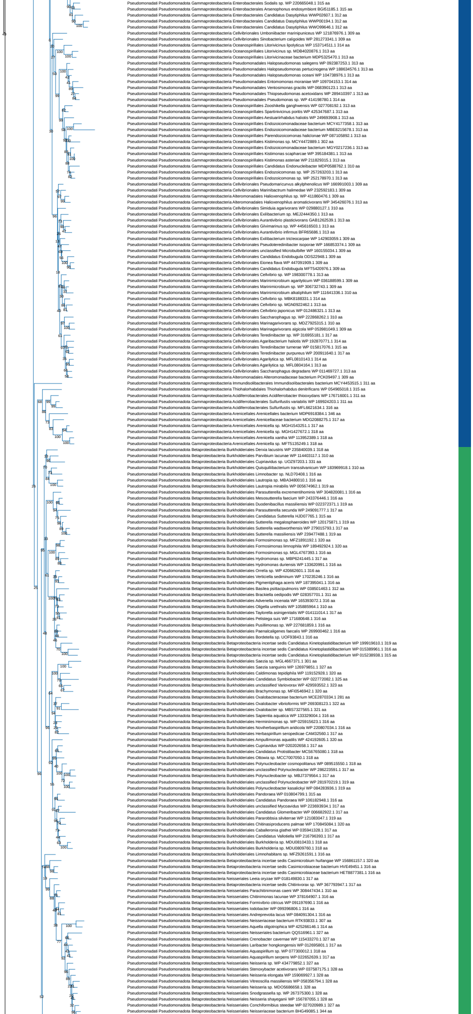

Pseudomonadati Pseudomonadota Gammaproteobacteria Enterobacterales Sodalis sp. WP 220665048.1 315 aa
Pseudomonadati Pseudomonadota Gammaproteobacteria Enterobacterales Arsenophonus endosymbiont BGI51185.1 315 aa
Pseudomonadati Pseudomonadota Gammaproteobacteria Enterobacterales Candidatus Dasytiphilus WWP02607.1 312 aa
Pseudomonadati Pseudomonadota Gammaproteobacteria Enterobacterales Candidatus Dasytiphilus WWP00194.1 312 aa
Pseudomonadati Pseudomonadota Gammaproteobacteria Enterobacterales Candidatus Dasytiphilus WWO99646.1 312 aa
Pseudomonadati Pseudomonadota Gammaproteobacteria Cellvibrionales Umboniibacter marinipuniceus WP 121876976.1 309 aa
Pseudomonadati Pseudomonadota Gammaproteobacteria Cellvibrionales Sinobacterium caligoides WP 281273341.1 309 aa
Pseudomonadati Pseudomonadota Gammaproteobacteria Oceanospirillales Litorivicinus lipolyticus WP 153714511.1 314 aa
Pseudomonadati Pseudomonadota Gammaproteobacteria Oceanospirillales Litorivicinus sp. MDB4020876.1 313 aa
Pseudomonadati Pseudomonadota Gammaproteobacteria Oceanospirillales Litorivicinaceae bacterium MDP5325470.1 313 aa
Pseudomonadati Pseudomonadota Gammaproteobacteria Pseudomonadales Halopseudomonas salegens WP 092387253.1 313 aa
Pseudomonadati Pseudomonadota Gammaproteobacteria Pseudomonadales Halopseudomonas pertucinogena WP 188634576.1 313 aa
Pseudomonadati Pseudomonadota Gammaproteobacteria Pseudomonadales Halopseudomonas oceani WP 104738976.1 313 aa
Pseudomonadati Pseudomonadota Gammaproteobacteria Pseudomonadales Entomomonas moraniae WP 109704153.1 314 aa
Pseudomonadati Pseudomonadota Gammaproteobacteria Pseudomonadales Ventosimonas gracilis WP 068390123.1 313 aa
Pseudomonadati Pseudomonadota Gammaproteobacteria Pseudomonadales Thiopseudomonas acetoxidans WP 289410397.1 313 aa
Pseudomonadati Pseudomonadota Gammaproteobacteria Pseudomonadales Pseudomonas sp. WP 414198780.1 314 aa
Pseudomonadati Pseudomonadota Gammaproteobacteria Oceanospirillales Zooshikella ganghwensis WP 027708192.1 313 aa
Pseudomonadati Pseudomonadota Gammaproteobacteria Oceanospirillales Spartinivicinus poritis WP 425347687.1 313 aa
Pseudomonadati Pseudomonadota Gammaproteobacteria Oceanospirillales Aestuariirhabdus haliotis WP 249693908.1 313 aa
Pseudomonadati Pseudomonadota Gammaproteobacteria Oceanospirillales Endozoicomonadaceae bacterium MCY4177358.1 313 aa
Pseudomonadati Pseudomonadota Gammaproteobacteria Oceanospirillales Endozoicomonadaceae bacterium MBE8215678.1 313 aa
Pseudomonadati Pseudomonadota Gammaproteobacteria Oceanospirillales Parendozoicomonas haliclonae WP 087105892.1 313 aa
Pseudomonadati Pseudomonadota Gammaproteobacteria Oceanospirillales Kistimonas sp. MCY4472889.1 302 aa
Pseudomonadati Pseudomonadota Gammaproteobacteria Oceanospirillales Endozoicomonadaceae bacterium MGY0217236.1 313 aa
Pseudomonadati Pseudomonadota Gammaproteobacteria Oceanospirillales Kistimonas scapharcae WP 395184381.1 313 aa
Pseudomonadati Pseudomonadota Gammaproteobacteria Oceanospirillales Kistimonas asteriae WP 211829315.1 313 aa
Pseudomonadati Pseudomonadota Gammaproteobacteria Oceanospirillales Candidatus Endonucleibacter MDP0588762.1 310 aa
Pseudomonadati Pseudomonadota Gammaproteobacteria Oceanospirillales Endozoicomonas sp. WP 257263203.1 313 aa
Pseudomonadati Pseudomonadota Gammaproteobacteria Oceanospirillales Endozoicomonas sp. WP 252178970.1 313 aa
Pseudomonadati Pseudomonadota Gammaproteobacteria Cellvibrionales Pseudomaricurvus alkylphenolicus WP 166991003.1 309 aa
Pseudomonadati Pseudomonadota Gammaproteobacteria Cellvibrionales Marinibactrum halimedae WP 232592183.1 309 aa
Pseudomonadati Pseudomonadota Gammaproteobacteria Alteromonadales Halioxenophilus sp. WP 411860476.1 309 aa
Pseudomonadati Pseudomonadota Gammaproteobacteria Alteromonadales Halioxenophilus aromaticivorans WP 345426076.1 313 aa
Pseudomonadati Pseudomonadota Gammaproteobacteria Cellvibrionales Simiduia agarivorans WP 029880127.1 310 aa
Pseudomonadati Pseudomonadota Gammaproteobacteria Cellvibrionales Exilibacterium sp. MEJ2444350.1 313 aa
Pseudomonadati Pseudomonadota Gammaproteobacteria Cellvibrionales Aurantivibrio plasticivorans GAB1262539.1 313 aa
Pseudomonadati Pseudomonadota Gammaproteobacteria Cellvibrionales Gilvimarinus sp. WP 445616503.1 313 aa
Pseudomonadati Pseudomonadota Gammaproteobacteria Cellvibrionales Aurantivibrio infirmus BFR65686.1 313 aa
Pseudomonadati Pseudomonadota Gammaproteobacteria Cellvibrionales Exilibacterium tricleocarpae WP 142903059.1 309 aa
Pseudomonadati Pseudomonadota Gammaproteobacteria Cellvibrionales Pseudoteredinibacter isoporae WP 166853374.1 309 aa
Pseudomonadati Pseudomonadota Gammaproteobacteria Cellvibrionales unclassified Microbulbifer WP 160155034.1 309 aa
Pseudomonadati Pseudomonadota Gammaproteobacteria Cellvibrionales Candidatus Endobugula ODS22948.1 309 aa
Pseudomonadati Pseudomonadota Gammaproteobacteria Cellvibrionales Eionea flava WP 447091909.1 309 aa
Pseudomonadati Pseudomonadota Gammaproteobacteria Cellvibrionales Candidatus Endobugula MFT5420976.1 309 aa
Pseudomonadati Pseudomonadota Gammaproteobacteria Cellvibrionales Cellvibrio sp. WP 198300778.1 313 aa
Pseudomonadati Pseudomonadota Gammaproteobacteria Cellvibrionales Marinimicrobium agarilyticum WP 036188599.1 309 aa
Pseudomonadati Pseudomonadota Gammaproteobacteria Cellvibrionales Marinimicrobium sp. WP 306732743.1 309 aa
Pseudomonadati Pseudomonadota Gammaproteobacteria Cellvibrionales Marinimicrobium alkaliphilum WP 111641336.1 310 aa
Pseudomonadati Pseudomonadota Gammaproteobacteria Cellvibrionales Cellvibrio sp. MBK8188331.1 314 aa
Pseudomonadati Pseudomonadota Gammaproteobacteria Cellvibrionales Cellvibrio sp. MGN0922462.1 313 aa
Pseudomonadati Pseudomonadota Gammaproteobacteria Cellvibrionales Cellvibrio japonicus WP 012486321.1 313 aa
Pseudomonadati Pseudomonadota Gammaproteobacteria Cellvibrionales Saccharophagus sp. WP 222868262.1 310 aa
Pseudomonadati Pseudomonadota Gammaproteobacteria Cellvibrionales Marinagarivorans sp. MDZ7925315.1 310 aa
Pseudomonadati Pseudomonadota Gammaproteobacteria Cellvibrionales Marinagarivorans algicola WP 053981049.1 309 aa
Pseudomonadati Pseudomonadota Gammaproteobacteria Cellvibrionales Teredinibacter sp. WP 316955181.1 317 aa
Pseudomonadati Pseudomonadota Gammaproteobacteria Cellvibrionales Agaribacterium haliotis WP 192870771.1 314 aa
Pseudomonadati Pseudomonadota Gammaproteobacteria Cellvibrionales Teredinibacter turnerae WP 015817076.1 315 aa
Pseudomonadati Pseudomonadota Gammaproteobacteria Cellvibrionales Teredinibacter purpureus WP 200911640.1 317 aa
Pseudomonadati Pseudomonadota Gammaproteobacteria Cellvibrionales Agarilytica sp. MFL0810143.1 314 aa
Pseudomonadati Pseudomonadota Gammaproteobacteria Cellvibrionales Agarilytica sp. MFL0804164.1 313 aa
Pseudomonadati Pseudomonadota Gammaproteobacteria Cellvibrionales Saccharophagus degradans WP 011469727.1 313 aa
Pseudomonadati Pseudomonadota Gammaproteobacteria Alteromonadales Alteromonadaceae bacterium PCK09497.1 309 aa
Pseudomonadati Pseudomonadota Gammaproteobacteria Immundisolibacterales Immundisolibacterales bacterium MCY4453515.1 311 aa
Pseudomonadati Pseudomonadota Gammaproteobacteria Thiohalorhabdales Thiohalorhabdus denitrificans WP 054965018.1 315 aa
Pseudomonadati Pseudomonadota Gammaproteobacteria Acidiferrobacterales Acidiferrobacter thiooxydans WP 176716001.1 311 aa
Pseudomonadati Pseudomonadota Gammaproteobacteria Acidiferrobacterales Sulfurifustis variabilis WP 169924203.1 311 aa
Pseudomonadati Pseudomonadota Gammaproteobacteria Acidiferrobacterales Sulfurifustis sp. MFL6621634.1 316 aa
Pseudomonadati Pseudomonadota Gammaproteobacteria Arenicellales Arenicellales bacterium MDP6918384.1 346 aa
Pseudomonadati Pseudomonadota Gammaproteobacteria Arenicellales Arenicellaceae bacterium MDG2088275.1 317 aa
Pseudomonadati Pseudomonadota Gammaproteobacteria Arenicellales Arenicella sp. MGH1543251.1 317 aa
Pseudomonadati Pseudomonadota Gammaproteobacteria Arenicellales Arenicella sp. MGH1427672.1 318 aa
Pseudomonadati Pseudomonadota Gammaproteobacteria Arenicellales Arenicella xantha WP 113952389.1 318 aa
Pseudomonadati Pseudomonadota Gammaproteobacteria Arenicellales Arenicella sp. MFT5135249.1 318 aa
Pseudomonadati Pseudomonadota Betaproteobacteria Burkholderiales Derxia lacustris WP 235840039.1 318 aa
Pseudomonadati Pseudomonadota Betaproteobacteria Burkholderiales Parvibium lacunae WP 114403117.1 310 aa
Pseudomonadati Pseudomonadota Betaproteobacteria Burkholderiales Cupriavidus sp. UOZ97203.1 331 aa
Pseudomonadati Pseudomonadota Betaproteobacteria Burkholderiales Quisquiliibacterium transsilvanicum WP 183969918.1 310 aa
Pseudomonadati Pseudomonadota Betaproteobacteria Burkholderiales Limnobacter sp. NLD70408.1 316 aa
Pseudomonadati Pseudomonadota Betaproteobacteria Burkholderiales Lautropia sp. MBA3480010.1 316 aa
Pseudomonadati Pseudomonadota Betaproteobacteria Burkholderiales Lautropia mirabilis WP 005674962.1 319 aa
Pseudomonadati Pseudomonadota Betaproteobacteria Burkholderiales Parasutterella excrementihominis WP 304820081.1 316 aa
Pseudomonadati Pseudomonadota Betaproteobacteria Burkholderiales Mesosutterella faecium WP 243376446.1 316 aa
Pseudomonadati Pseudomonadota Betaproteobacteria Burkholderiales Duodenibacillus massiliensis WP 022372371.1 319 aa
Pseudomonadati Pseudomonadota Betaproteobacteria Burkholderiales Parasutterella secunda WP 249091777.1 317 aa
Pseudomonadati Pseudomonadota Betaproteobacteria Burkholderiales Candidatus Sutterella HJD07765.1 315 aa
Pseudomonadati Pseudomonadota Betaproteobacteria Burkholderiales Sutterella megalosphaeroides WP 120175871.1 319 aa
Pseudomonadati Pseudomonadota Betaproteobacteria Burkholderiales Sutterella wadsworthensis WP 279015793.1 317 aa
Pseudomonadati Pseudomonadota Betaproteobacteria Burkholderiales Sutterella massiliensis WP 239477488.1 319 aa
Pseudomonadati Pseudomonadota Betaproteobacteria Burkholderiales Formosimonas sp. MFZ1891192.1 320 aa
Pseudomonadati Pseudomonadota Betaproteobacteria Burkholderiales Formosimonas limnophila WP 189492924.1 320 aa
Pseudomonadati Pseudomonadota Betaproteobacteria Burkholderiales Formosimonas sp. MGL4767393.1 316 aa
Pseudomonadati Pseudomonadota Betaproteobacteria Burkholderiales Hydromonas sp. MBP6241445.1 317 aa
Pseudomonadati Pseudomonadota Betaproteobacteria Burkholderiales Hydromonas duriensis WP 133620991.1 316 aa
Pseudomonadati Pseudomonadota Betaproteobacteria Burkholderiales Orrella sp. WP 420662601.1 316 aa
Pseudomonadati Pseudomonadota Betaproteobacteria Burkholderiales Verticiella sediminum WP 170235246.1 316 aa
Pseudomonadati Pseudomonadota Betaproteobacteria Burkholderiales Pigmentiphaga aceris WP 187395041.1 316 aa
Pseudomonadati Pseudomonadota Betaproteobacteria Burkholderiales Basilea psittacipulmonis WP 038501463.1 312 aa
Pseudomonadati Pseudomonadota Betaproteobacteria Burkholderiales Brackiella oedipodis WP 028357701.1 311 aa
Pseudomonadati Pseudomonadota Betaproteobacteria Burkholderiales Advenella incenata WP 165393072.1 316 aa
Pseudomonadati Pseudomonadota Betaproteobacteria Burkholderiales Oligella urethralis WP 105885964.1 310 aa
Pseudomonadati Pseudomonadota Betaproteobacteria Burkholderiales Taylorella asinigenitalis WP 014111014.1 317 aa
Pseudomonadati Pseudomonadota Betaproteobacteria Burkholderiales Pelistega suis WP 171680648.1 316 aa
Pseudomonadati Pseudomonadota Betaproteobacteria Burkholderiales Pusillimonas sp. WP 227681859.1 316 aa
Pseudomonadati Pseudomonadota Betaproteobacteria Burkholderiales Paenalcaligenes faecalis WP 269900462.1 316 aa
Pseudomonadati Pseudomonadota Betaproteobacteria Burkholderiales Bordetella sp. UOF93843.1 316 aa
Pseudomonadati Pseudomonadota Betaproteobacteria Betaproteobacteria incertae sedis Candidatus Kinetoplastidibacterium WP 199919610.1 319 aa
Pseudomonadati Pseudomonadota Betaproteobacteria Betaproteobacteria incertae sedis Candidatus Kinetoplastidibacterium WP 015389961.1 316 aa
Pseudomonadati Pseudomonadota Betaproteobacteria Betaproteobacteria incertae sedis Candidatus Kinetoplastidibacterium WP 015238938.1 315 aa
Pseudomonadati Pseudomonadota Betaproteobacteria Burkholderiales Saezia sp. MGL4667371.1 301 aa
Pseudomonadati Pseudomonadota Betaproteobacteria Burkholderiales Saezia sanguinis WP 126979851.1 327 aa
Pseudomonadati Pseudomonadota Betaproteobacteria Burkholderiales Caldimonas tepidiphila WP 119152928.1 320 aa
Pseudomonadati Pseudomonadota Betaproteobacteria Burkholderiales Candidatus Symbiobacter WP 022772082.1 325 aa
Pseudomonadati Pseudomonadota Betaproteobacteria Burkholderiales unclassified Variovorax WP 429593552.1 323 aa
Pseudomonadati Pseudomonadota Betaproteobacteria Burkholderiales Brachymonas sp. MFI0546942.1 320 aa
Pseudomonadati Pseudomonadota Betaproteobacteria Burkholderiales Oxalobacteraceae bacterium MCE2870334.1 281 aa
Pseudomonadati Pseudomonadota Betaproteobacteria Burkholderiales Oxalobacter vibrioformis WP 269308123.1 322 aa
Pseudomonadati Pseudomonadota Betaproteobacteria Burkholderiales Oxalobacter sp. MBS7327565.1 321 aa
Pseudomonadati Pseudomonadota Betaproteobacteria Burkholderiales Sapientia aquatica WP 133329004.1 316 aa
Pseudomonadati Pseudomonadota Betaproteobacteria Burkholderiales Herminiimonas sp. WP 025915623.1 316 aa
Pseudomonadati Pseudomonadota Betaproteobacteria Burkholderiales Noviherbaspirillum aridicola WP 220807034.1 316 aa
Pseudomonadati Pseudomonadota Betaproteobacteria Burkholderiales Herbaspirillum seropedicae CAM32560.1 317 aa
Pseudomonadati Pseudomonadota Betaproteobacteria Burkholderiales Ampullimonas aquatilis WP 424192605.1 320 aa
Pseudomonadati Pseudomonadota Betaproteobacteria Burkholderiales Cupriavidus WP 020202658.1 317 aa
Pseudomonadati Pseudomonadota Betaproteobacteria Burkholderiales Candidatus Protistibacter MCS6765080.1 318 aa
Pseudomonadati Pseudomonadota Betaproteobacteria Burkholderiales Ottowia sp. MCC7007050.1 318 aa
Pseudomonadati Pseudomonadota Betaproteobacteria Burkholderiales Polynucleobacter cosmopolitanus WP 089515550.1 318 aa
Pseudomonadati Pseudomonadota Betaproteobacteria Burkholderiales unclassified Polynucleobacter WP 286223591.1 317 aa
Pseudomonadati Pseudomonadota Betaproteobacteria Burkholderiales Polynucleobacter sp. MBJ7379564.1 317 aa
Pseudomonadati Pseudomonadota Betaproteobacteria Burkholderiales unclassified Polynucleobacter WP 281970219.1 319 aa
Pseudomonadati Pseudomonadota Betaproteobacteria Burkholderiales Polynucleobacter kasalickyi WP 084283936.1 319 aa
Pseudomonadati Pseudomonadota Betaproteobacteria Burkholderiales Pandoraea WP 010804799.1 315 aa
Pseudomonadati Pseudomonadota Betaproteobacteria Burkholderiales Candidatus Pandoraea WP 106182948.1 316 aa
Pseudomonadati Pseudomonadota Betaproteobacteria Burkholderiales unclassified Mycoavidus WP 223693934.1 317 aa
Pseudomonadati Pseudomonadota Betaproteobacteria Burkholderiales Candidatus Glomeribacter WP 006682922.1 317 aa
Pseudomonadati Pseudomonadota Betaproteobacteria Burkholderiales Pararobbsia silviterrae WP 121083047.1 319 aa
Pseudomonadati Pseudomonadota Betaproteobacteria Burkholderiales Chitinasiproducens palmae WP 170845084.1 320 aa
Pseudomonadati Pseudomonadota Betaproteobacteria Burkholderiales Caballeronia glathei WP 035941328.1 317 aa
Pseudomonadati Pseudomonadota Betaproteobacteria Burkholderiales Candidatus Vallotiella WP 216796393.1 317 aa
Pseudomonadati Pseudomonadota Betaproteobacteria Burkholderiales Burkholderia sp. MDU0810433.1 318 aa
Pseudomonadati Pseudomonadota Betaproteobacteria Burkholderiales Burkholderia sp. MDU0809760.1 318 aa
Pseudomonadati Pseudomonadota Betaproteobacteria Burkholderiales Limnohabitans sp. MFZ9261591.1 316 aa
Pseudomonadati Pseudomonadota Betaproteobacteria Betaproteobacteria incertae sedis Casimicrobium huifangae WP 156861157.1 320 aa
Pseudomonadati Pseudomonadota Betaproteobacteria Betaproteobacteria incertae sedis Casimicrobiaceae bacterium HVE49451.1 316 aa
Pseudomonadati Pseudomonadota Betaproteobacteria Betaproteobacteria incertae sedis Casimicrobiaceae bacterium HET8877381.1 316 aa
Pseudomonadati Pseudomonadota Betaproteobacteria Neisseriales Leeia oryzae WP 018149830.1 317 aa
Pseudomonadati Pseudomonadota Betaproteobacteria Betaproteobacteria incertae sedis Chitinivorax sp. WP 367793947.1 317 aa
Pseudomonadati Pseudomonadota Betaproteobacteria Neisseriales Parachitinimonas caeni WP 308447434.1 310 aa
Pseudomonadati Pseudomonadota Betaproteobacteria Neisseriales Chitinimonas lacunae WP 378164907.1 316 aa
Pseudomonadati Pseudomonadota Betaproteobacteria Neisseriales Formivibrio citricus WP 091197690.1 316 aa
Pseudomonadati Pseudomonadota Betaproteobacteria Neisseriales Iodobacter WP 099396806.1 316 aa
Pseudomonadati Pseudomonadota Betaproteobacteria Neisseriales Andreprevotia lacus WP 084091304.1 316 aa
Pseudomonadati Pseudomonadota Betaproteobacteria Neisseriales Neisseriaceae bacterium RTK93833.1 307 aa
Pseudomonadati Pseudomonadota Betaproteobacteria Neisseriales Aquella oligotrophica WP 425266146.1 314 aa
Pseudomonadati Pseudomonadota Betaproteobacteria Neisseriales Neisseriales bacterium QQS16961.1 327 aa
Pseudomonadati Pseudomonadota Betaproteobacteria Neisseriales Crenobacter cavernae WP 115433270.1 327 aa
Pseudomonadati Pseudomonadota Betaproteobacteria Neisseriales Laribacter hongkongensis WP 012695801.1 317 aa
Pseudomonadati Pseudomonadota Betaproteobacteria Neisseriales Aquaspirillum sp. WP 077300012.1 318 aa
Pseudomonadati Pseudomonadota Betaproteobacteria Neisseriales Aquaspirillum serpens WP 022652639.1 317 aa
Pseudomonadati Pseudomonadota Betaproteobacteria Neisseriales Neisseria sp. WP 434779852.1 327 aa
Pseudomonadati Pseudomonadota Betaproteobacteria Neisseriales Stenoxybacter acetivorans WP 037587175.1 328 aa
Pseudomonadati Pseudomonadota Betaproteobacteria Neisseriales Neisseria elongata WP 159069927.1 328 aa
Pseudomonadati Pseudomonadota Betaproteobacteria Neisseriales Vitreoscilla massiliensis WP 058356794.1 328 aa
Pseudomonadati Pseudomonadota Betaproteobacteria Neisseriales Neisseria sp. MDO5686658.1 328 aa
Pseudomonadati Pseudomonadota Betaproteobacteria Neisseriales Snodgrassella sp. WP 267375300.1 328 aa
Pseudomonadati Pseudomonadota Betaproteobacteria Neisseriales Neisseria shayeganii WP 156787055.1 328 aa
Pseudomonadati Pseudomonadota Betaproteobacteria Neisseriales Conchiformibius steedae WP 027020989.1 327 aa
Pseudomonadati Pseudomonadota Betaproteobacteria Neisseriales Neisseriaceae bacterium BHG49085.1 344 aa

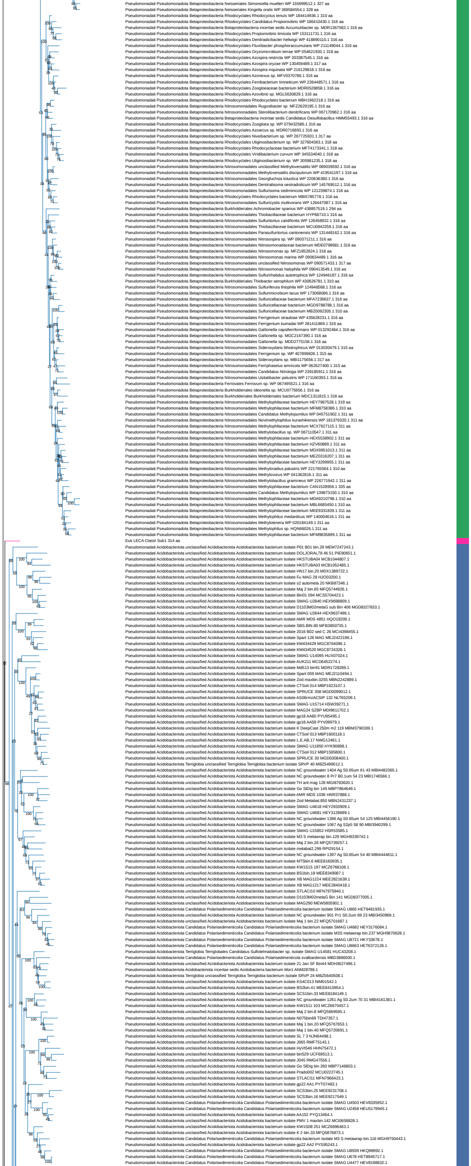
Pseudomonadati Pseudomonadota Betaproteobacteria Neisseriales Simonsiella muelleri WP 155999512.1 327 aa
Pseudomonadati Pseudomonadota Betaproteobacteria Neisseriales Kingella oralis WP 369584554.1 328 aa
Pseudomonadati Pseudomonadota Betaproteobacteria Rhodocyclales Rhodocyclus tenuis WP 184414936.1 310 aa
Pseudomonadati Pseudomonadota Betaproteobacteria Rhodocyclales Candidatus Propionivibrio WP 186410430.1 316 aa
Pseudomonadati Pseudomonadota Betaproteobacteria Betaproteobacteria incertae sedis Accumulibacter sp. MDR1367582.1 316 aa
Pseudomonadati Pseudomonadota Betaproteobacteria Rhodocyclales Propionivibrio limicola WP 153111731.1 316 aa
Pseudomonadati Pseudomonadota Betaproteobacteria Rhodocyclales Dentiradicibacter hellwigii WP 418890110.1 316 aa
Pseudomonadati Pseudomonadota Betaproteobacteria Rhodocyclales Fluviibacter phosphoraccumulans WP 211149044.1 316 aa
Pseudomonadati Pseudomonadota Betaproteobacteria Rhodocyclales Oryzomicrobium terrae WP 054621930.1 318 aa
Pseudomonadati Pseudomonadota Betaproteobacteria Rhodocyclales Azospira restricta WP 203387545.1 316 aa
Pseudomonadati Pseudomonadota Betaproteobacteria Rhodocyclales Azospira oryzae WP 130459489.1 317 aa
Pseudomonadati Pseudomonadota Betaproteobacteria Rhodocyclales Azospira inquinata WP 216129818.1 316 aa
Pseudomonadati Pseudomonadota Betaproteobacteria Rhodocyclales Azonexus sp. MFV0370786.1 316 aa
Pseudomonadati Pseudomonadota Betaproteobacteria Rhodocyclales Ferribacterium limneticum WP 226448571.1 316 aa
Pseudomonadati Pseudomonadota Betaproteobacteria Rhodocyclales Zoogloeaceae bacterium MDR0528858.1 316 aa
Pseudomonadati Pseudomonadota Betaproteobacteria Rhodocyclales Azovibrio sp. MGL5630829.1 316 aa
Pseudomonadati Pseudomonadota Betaproteobacteria Rhodocyclales Rhodocyclales bacterium MBH1962218.1 316 aa
Pseudomonadati Pseudomonadota Betaproteobacteria Nitrosomonadales Rugosibacter sp. MFZ2629195.1 316 aa
Pseudomonadati Pseudomonadota Betaproteobacteria Nitrosomonadales Sterolibacterium denitrificans WP 067170962.1 316 aa
Pseudomonadati Pseudomonadota Betaproteobacteria Betaproteobacteria incertae sedis Candidatus Desulfobacillus HMM55493.1 316 aa
Pseudomonadati Pseudomonadota Betaproteobacteria Rhodocyclales Zoogloea sp. WP 079432586.1 316 aa
Pseudomonadati Pseudomonadota Betaproteobacteria Rhodocyclales Azoarcus sp. MDR0716693.1 316 aa
Pseudomonadati Pseudomonadota Betaproteobacteria Rhodocyclales Niveibacterium sp. WP 267725931.1 317 aa
Pseudomonadati Pseudomonadota Betaproteobacteria Rhodocyclales Uliginosibacterium sp. WP 327604363.1 318 aa
Pseudomonadati Pseudomonadota Betaproteobacteria Rhodocyclales Rhodocyclaceae bacterium MFT4173341.1 318 aa
Pseudomonadati Pseudomonadota Betaproteobacteria Rhodocyclales Viridibacterium curvum WP 345534040.1 318 aa
Pseudomonadati Pseudomonadota Betaproteobacteria Rhodocyclales Uliginosibacterium sp. WP 305981235.1 318 aa
Pseudomonadati Pseudomonadota Betaproteobacteria Nitrosomonadales unclassified Methyloversatilis WP 069039592.1 316 aa
Pseudomonadati Pseudomonadota Betaproteobacteria Nitrosomonadales Methyloversatilis discipulorum WP 419541197.1 316 aa
Pseudomonadati Pseudomonadota Betaproteobacteria Nitrosomonadales Georgfuchsia toluolica WP 220636380.1 316 aa
Pseudomonadati Pseudomonadota Betaproteobacteria Nitrosomonadales Denitratisoma oestradiolicum WP 145769512.1 316 aa
Pseudomonadati Pseudomonadota Betaproteobacteria Nitrosomonadales Sulfurisoma sediminicola WP 121239874.1 316 aa
Pseudomonadati Pseudomonadota Betaproteobacteria Rhodocyclales Rhodocyclales bacterium MBI5785778.1 316 aa
Pseudomonadati Pseudomonadota Betaproteobacteria Nitrosomonadales Sulfuricystis multivorans WP 126447087.1 316 aa
Pseudomonadati Pseudomonadota Betaproteobacteria Burkholderiales Achromobacter spanius WP 438857519.1 294 aa
Pseudomonadati Pseudomonadota Betaproteobacteria Nitrosomonadales Thiobacillaceae bacterium HYP68710.1 316 aa
Pseudomonadati Pseudomonadota Betaproteobacteria Nitrosomonadales Sulfuritortus calidifontis WP 126458922.1 316 aa
Pseudomonadati Pseudomonadota Betaproteobacteria Nitrosomonadales Thiobacillaceae bacterium MCU0842259.1 316 aa
Pseudomonadati Pseudomonadota Betaproteobacteria Nitrosomonadales Parasulfuritortus cantonensis WP 131448162.1 316 aa
Pseudomonadati Pseudomonadota Betaproteobacteria Nitrosomonadales Nitrosospira sp. WP 090371211.1 316 aa
Pseudomonadati Pseudomonadota Betaproteobacteria Nitrosomonadales Nitrosomonadaceae bacterium MDE0798681.1 316 aa
Pseudomonadati Pseudomonadota Betaproteobacteria Nitrosomonadales Nitrosomonas sp. MFZ1852824.1 316 aa
Pseudomonadati Pseudomonadota Betaproteobacteria Nitrosomonadales Nitrosomonas marina WP 090634489.1 316 aa
Pseudomonadati Pseudomonadota Betaproteobacteria Nitrosomonadales unclassified Nitrosomonas WP 090571433.1 317 aa
Pseudomonadati Pseudomonadota Betaproteobacteria Nitrosomonadales Nitrosomonas halophila WP 090413549.1 316 aa
Pseudomonadati Pseudomonadota Betaproteobacteria Nitrosomonadales Sulfurirhabdus autotrophica WP 124946187.1 316 aa
Pseudomonadati Pseudomonadota Betaproteobacteria Burkholderiales Thiobacter aerophilum WP 430626781.1 310 aa
Pseudomonadati Pseudomonadota Betaproteobacteria Nitrosomonadales Sulfuriferula thiophila WP 124948568.1 316 aa
Pseudomonadati Pseudomonadota Betaproteobacteria Nitrosomonadales Sulfurimicrobium lacus WP 173068086.1 316 aa
Pseudomonadati Pseudomonadota Betaproteobacteria Nitrosomonadales Sulfuricellaceae bacterium MFA7239637.1 316 aa
Pseudomonadati Pseudomonadota Betaproteobacteria Nitrosomonadales Sulfuricellaceae bacterium MGD9788788.1 316 aa
Pseudomonadati Pseudomonadota Betaproteobacteria Nitrosomonadales Sulfuricellaceae bacterium MBZ0092305.1 310 aa
Pseudomonadati Pseudomonadota Betaproteobacteria Nitrosomonadales Ferrigenium straubiae WP 435628231.1 316 aa
Pseudomonadati Pseudomonadota Betaproteobacteria Nitrosomonadales Ferrigenium kumadai WP 281411869.1 316 aa
Pseudomonadati Pseudomonadota Betaproteobacteria Nitrosomonadales Gallionella capsiferriformans WP 013292464.1 316 aa
Pseudomonadati Pseudomonadota Betaproteobacteria Nitrosomonadales Gallionella sp. MGC2167390.1 316 aa
Pseudomonadati Pseudomonadota Betaproteobacteria Nitrosomonadales Gallionella sp. MDD2775156.1 316 aa
Pseudomonadati Pseudomonadota Betaproteobacteria Nitrosomonadales Sideroxydans lithotrophicus WP 013030479.1 315 aa
Pseudomonadati Pseudomonadota Betaproteobacteria Nitrosomonadales Ferrigenium sp. WP 407899826.1 315 aa
Pseudomonadati Pseudomonadota Betaproteobacteria Nitrosomonadales Sideroxydans sp. MBI1175656.1 317 aa
Pseudomonadati Pseudomonadota Betaproteobacteria Nitrosomonadales Ferriphaselus amnicola WP 062627400.1 315 aa
Pseudomonadati Pseudomonadota Betaproteobacteria Nitrosomonadales Candidatus Nitrotoga WP 239185911.1 316 aa
Pseudomonadati Pseudomonadota Betaproteobacteria Nitrosomonadales Usitatibacter palustris WP 171160393.1 316 aa
Pseudomonadati Pseudomonadota Betaproteobacteria Ferrovales Ferrovum sp. WP 067495521.1 316 aa
Pseudomonadati Pseudomonadota Betaproteobacteria Burkholderiales Ideonella sp. MCU0775656.1 316 aa
Pseudomonadati Pseudomonadota Betaproteobacteria Burkholderiales Burkholderiales bacterium MDC1311815.1 316 aa
Pseudomonadati Pseudomonadota Betaproteobacteria Nitrosomonadales Methylophilaceae bacterium HEY7987528.1 319 aa
Pseudomonadati Pseudomonadota Betaproteobacteria Nitrosomonadales Methylophilaceae bacterium MFM8758386.1 310 aa
Pseudomonadati Pseudomonadota Betaproteobacteria Nitrosomonadales Candidatus Methylopumilus WP 045751902.1 311 aa
Pseudomonadati Pseudomonadota Betaproteobacteria Nitrosomonadales Novimethylophilus kurashikiensis WP 181376320.1 311 aa
Pseudomonadati Pseudomonadota Betaproteobacteria Nitrosomonadales Methylophilaceae bacterium MCX7627115.1 311 aa
Pseudomonadati Pseudomonadota Betaproteobacteria Nitrosomonadales Methylobacillus sp. WP 067110547.1 311 aa
Pseudomonadati Pseudomonadota Betaproteobacteria Nitrosomonadales Methylophilaceae bacterium HEX5538902.1 311 aa
Pseudomonadati Pseudomonadota Betaproteobacteria Nitrosomonadales Methylophilaceae bacterium HZV60889.1 311 aa
Pseudomonadati Pseudomonadota Betaproteobacteria Nitrosomonadales Methylophilaceae bacterium MDX9951013.1 311 aa
Pseudomonadati Pseudomonadota Betaproteobacteria Nitrosomonadales Methylophilaceae bacterium MEZ0316207.1 311 aa
Pseudomonadati Pseudomonadota Betaproteobacteria Nitrosomonadales Methylophilaceae bacterium HEY3299955.1 311 aa
Pseudomonadati Pseudomonadota Betaproteobacteria Nitrosomonadales Methyloradius palustris WP 221765564.1 310 aa
Pseudomonadati Pseudomonadota Betaproteobacteria Nitrosomonadales Methylovorus WP 041362816.1 311 aa
Pseudomonadati Pseudomonadota Betaproteobacteria Nitrosomonadales Methylobacillus gramineus WP 226771942.1 311 aa
Pseudomonadati Pseudomonadota Betaproteobacteria Nitrosomonadales Methylophilaceae bacterium CAN1528956.1 335 aa
Pseudomonadati Pseudomonadota Betaproteobacteria Nitrosomonadales Candidatus Methylopumilus WP 139873150.1 310 aa
Pseudomonadati Pseudomonadota Betaproteobacteria Nitrosomonadales Methylophilaceae bacterium MDA9210798.1 310 aa
Pseudomonadati Pseudomonadota Betaproteobacteria Nitrosomonadales Methylophilaceae bacterium MBL6685450.1 310 aa
Pseudomonadati Pseudomonadota Betaproteobacteria Nitrosomonadales Methylophilaceae bacterium MEE9331839.1 311 aa
Pseudomonadati Pseudomonadota Betaproteobacteria Nitrosomonadales Methylophilus medardicus WP 140004616.1 311 aa
Pseudomonadati Pseudomonadota Betaproteobacteria Nitrosomonadales Methylotenera WP 020184149.1 311 aa
Pseudomonadati Pseudomonadota Betaproteobacteria Nitrosomonadales Methylophilus sp. HQN66026.1 311 aa
Pseudomonadati Pseudomonadota Betaproteobacteria Nitrosomonadales Methylophilaceae bacterium MFM9835899.1 311 aa
Euk LECA ClassI Sub1 314 aa
Pseudomonadati Acidobacteriota unclassified Acidobacteriota Acidobacteriota bacterium isolate P01 B01 bin.29 MEM7247243.1
Pseudomonadati Acidobacteriota unclassified Acidobacteriota Acidobacteriota bacterium isolate DOLJORAL78 46 51 PIE90651.1
Pseudomonadati Acidobacteriota unclassified Acidobacteriota Acidobacteriota bacterium isolate HKSTUBA04 MCB1044807.1
Pseudomonadati Acidobacteriota unclassified Acidobacteriota Acidobacteriota bacterium isolate HKSTUBA03 MCB1052485.1
Pseudomonadati Acidobacteriota unclassified Acidobacteriota Acidobacteriota bacterium isolate HN17.bin.20 MDX1389722.1
Pseudomonadati Acidobacteriota unclassified Acidobacteriota Acidobacteriota bacterium isolate Fu MAG 28 HJO03200.1
Pseudomonadati Acidobacteriota unclassified Acidobacteriota Acidobacteriota bacterium isolate s2 autometa 20 NKB87346.1
Pseudomonadati Acidobacteriota unclassified Acidobacteriota Acidobacteriota bacterium isolate Maj 2 bin.65 MFQ5744926.1
Pseudomonadati Acidobacteriota unclassified Acidobacteriota Acidobacteriota bacterium isolate Bin01 094 MCS5704423.1
Pseudomonadati Acidobacteriota unclassified Acidobacteriota Acidobacteriota bacterium isolate SMAG U2840 HEX9698809.1
Pseudomonadati Acidobacteriota unclassified Acidobacteriota Acidobacteriota bacterium isolate D1103M02metaG sub Bin 406 MGD8327833.1
Pseudomonadati Acidobacteriota unclassified Acidobacteriota Acidobacteriota bacterium isolate SMAG U2844 HEX9637496.1
Pseudomonadati Acidobacteriota unclassified Acidobacteriota Acidobacteriota bacterium isolate AMR MDS 4851 HQO19209.1
Pseudomonadati Acidobacteriota unclassified Acidobacteriota Acidobacteriota bacterium isolate SBS.BIN.80 MFB3850735.1
Pseudomonadati Acidobacteriota unclassified Acidobacteriota Acidobacteriota bacterium isolate 2016 B02 sed C 26 MCI4398455.1
Pseudomonadati Acidobacteriota unclassified Acidobacteriota Acidobacteriota bacterium isolate Spart 126 MAG MEJ2422196.1
Pseudomonadati Acidobacteriota unclassified Acidobacteriota Acidobacteriota bacterium isolate KM434429 MGC8764096.1
Pseudomonadati Acidobacteriota unclassified Acidobacteriota Acidobacteriota bacterium isolate KM434520 MGC8724328.1
Pseudomonadati Acidobacteriota unclassified Acidobacteriota Acidobacteriota bacterium isolate SMAG U14095 HUX07024.1
Pseudomonadati Acidobacteriota unclassified Acidobacteriota Acidobacteriota bacterium isolate AUK211 MCD6452274.1
Pseudomonadati Acidobacteriota unclassified Acidobacteriota Acidobacteriota bacterium isolate Md513 bin91 MDR1728289.1
Pseudomonadati Acidobacteriota unclassified Acidobacteriota Acidobacteriota bacterium isolate Spart 059 MAG MEJ2110494.1
Pseudomonadati Acidobacteriota unclassified Acidobacteriota Acidobacteriota bacterium isolate Zod maxbin.0255 MBN2242889.1
Pseudomonadati Acidobacteriota unclassified Acidobacteriota Acidobacteriota bacterium isolate CTSoil 014 MBP1623107.1
Pseudomonadati Acidobacteriota unclassified Acidobacteriota Acidobacteriota bacterium isolate SPRUCE 358 MGD0099012.1
Pseudomonadati Acidobacteriota unclassified Acidobacteriota Acidobacteriota bacterium isolate AS06rmzACSIP 132 NLT65209.1
Pseudomonadati Acidobacteriota unclassified Acidobacteriota Acidobacteriota bacterium isolate SMAG U15714 HSW39271.1
Pseudomonadati Acidobacteriota unclassified Acidobacteriota Acidobacteriota bacterium isolate MAG24 SZBP MDI9611702.1
Pseudomonadati Acidobacteriota unclassified Acidobacteriota Acidobacteriota bacterium isolate gp18 AA60 PYU95495.1
Pseudomonadati Acidobacteriota unclassified Acidobacteriota Acidobacteriota bacterium isolate gp18 AA59 PYV09979.1
Pseudomonadati Acidobacteriota unclassified Acidobacteriota Acidobacteriota bacterium isolate K DeepCast 250m m2 119 MBM3790339.1
Pseudomonadati Acidobacteriota unclassified Acidobacteriota Acidobacteriota bacterium isolate CTSoil 013 MBP1600118.1
Pseudomonadati Acidobacteriota unclassified Acidobacteriota Acidobacteriota bacterium isolate L.E.AB.17 NWG12461.1
Pseudomonadati Acidobacteriota unclassified Acidobacteriota Acidobacteriota bacterium isolate SMAG U11850 HYK90898.1
Pseudomonadati Acidobacteriota unclassified Acidobacteriota Acidobacteriota bacterium isolate CTSoil 012 MBP1595800.1
Pseudomonadati Acidobacteriota unclassified Acidobacteriota Acidobacteriota bacterium isolate SPRUCE 30 MGD0308400.1
Pseudomonadati Acidobacteriota Terriglobia unclassified Terriglobia Terriglobia bacterium isolate SRVP 40 MBZ5499012.1
Pseudomonadati Acidobacteriota unclassified Acidobacteriota Acidobacteriota bacterium isolate NC groundwater 1404 Ag S0.65um 61 43 MBI4482365.1
Pseudomonadati Acidobacteriota unclassified Acidobacteriota Acidobacteriota bacterium isolate NC groundwater 8 Pr7 B0.1um 54 23 MBI1746566.1
Pseudomonadati Acidobacteriota unclassified Acidobacteriota Acidobacteriota bacterium isolate TH ant mag 128 MGI8783620.1
Pseudomonadati Acidobacteriota unclassified Acidobacteriota Acidobacteriota bacterium isolate Go SlDig bin 149 MBP7864649.1
Pseudomonadati Acidobacteriota unclassified Acidobacteriota Acidobacteriota bacterium isolate AMR MDS 1036 HNR37888.1
Pseudomonadati Acidobacteriota unclassified Acidobacteriota Acidobacteriota bacterium isolate Zod Metabat.850 MBN2431237.1
Pseudomonadati Acidobacteriota unclassified Acidobacteriota Acidobacteriota bacterium isolate SMAG U4618 HEY2933909.1
Pseudomonadati Acidobacteriota unclassified Acidobacteriota Acidobacteriota bacterium isolate SMAG U4681 HEY3128689.1
Pseudomonadati Acidobacteriota unclassified Acidobacteriota Acidobacteriota bacterium isolate NC groundwater 1396 Ag S0.65um 54 125 MBI4456190.1
Pseudomonadati Acidobacteriota unclassified Acidobacteriota Acidobacteriota bacterium isolate NC groundwater 1067 Ag S2p5 58 90 MBI3940299.1
Pseudomonadati Acidobacteriota unclassified Acidobacteriota Acidobacteriota bacterium isolate SMAG U15852 HSR53585.1
Pseudomonadati Acidobacteriota unclassified Acidobacteriota Acidobacteriota bacterium isolate M3 S metawrap bin.129 MGH9339742.1
Pseudomonadati Acidobacteriota unclassified Acidobacteriota Acidobacteriota bacterium isolate Maj 2 bin.26 MFQ5739257.1
Pseudomonadati Acidobacteriota unclassified Acidobacteriota Acidobacteriota bacterium isolate metabat2.299 RPI29154.1
Pseudomonadati Acidobacteriota unclassified Acidobacteriota Acidobacteriota bacterium isolate NC groundwater 1397 Ag S0.65um 54 40 MBI4444611.1
Pseudomonadati Acidobacteriota unclassified Acidobacteriota Acidobacteriota bacterium isolate MT5bin.6 MEE8160935.1
Pseudomonadati Acidobacteriota unclassified Acidobacteriota Acidobacteriota bacterium isolate KW1S15 197 MCZ6768106.1
Pseudomonadati Acidobacteriota unclassified Acidobacteriota Acidobacteriota bacterium isolate BS1bin.18 MEE8349087.1
Pseudomonadati Acidobacteriota unclassified Acidobacteriota Acidobacteriota bacterium isolate XB MAG1224 MEE2821639.1
Pseudomonadati Acidobacteriota unclassified Acidobacteriota Acidobacteriota bacterium isolate XB MAG1217 MEE2840418.1
Pseudomonadati Acidobacteriota unclassified Acidobacteriota Acidobacteriota bacterium isolate STLACI10 MFN7975940.1
Pseudomonadati Acidobacteriota unclassified Acidobacteriota Acidobacteriota bacterium isolate D1103M02metaG Bin 141 MGD8377005.1
Pseudomonadati Acidobacteriota unclassified Acidobacteriota Acidobacteriota bacterium isolate MAG290 MEW5805901.1
Pseudomonadati Acidobacteriota Candidatus Polarisedimenticolia Candidatus Polarisedimenticolia bacterium isolate SMAG U865 HET9481935.1
Pseudomonadati Acidobacteriota unclassified Acidobacteriota Acidobacteriota bacterium isolate NC groundwater 901 Pr1 S0.2um 69 23 MBI3450969.1
Pseudomonadati Acidobacteriota unclassified Acidobacteriota Acidobacteriota bacterium isolate Maj 1 bin.22 MFQ5701687.1
Pseudomonadati Acidobacteriota Candidatus Polarisedimenticolia Candidatus Polarisedimenticolia bacterium isolate SMAG U4682 HEY3176084.1
Pseudomonadati Acidobacteriota Candidatus Polarisedimenticolia Candidatus Polarisedimenticolia bacterium isolate M3S metawrap bin.237 MGH9870628.1
Pseudomonadati Acidobacteriota Candidatus Polarisedimenticolia Candidatus Polarisedimenticolia bacterium isolate SMAG U8721 HKY33678.1
Pseudomonadati Acidobacteriota Candidatus Polarisedimenticolia Candidatus Polarisedimenticolia bacterium isolate SMAG U8663 HET6372126.1
Pseudomonadati Acidobacteriota Terriglobia Terriglobales Candidatus Sulfotelmatobacter sp. isolate SMAG U14581 HUC43208.1
Pseudomonadati Acidobacteriota Candidatus Polarisedimenticolia Candidatus Polarisedimenticola svalbardensis MBD3866500.1
Pseudomonadati Acidobacteriota unclassified Acidobacteriota Acidobacteriota bacterium isolate 21 Jan SF Bin44 MDH3627496.1
Pseudomonadati Acidobacteriota Acidobacteriota incertae sedis Acidobacteria bacterium Mor1 ANM28788.1
Pseudomonadati Acidobacteriota Terriglobia unclassified Terriglobia Terriglobia bacterium isolate SRVP 24 MBZ5640508.1
Pseudomonadati Acidobacteriota unclassified Acidobacteriota Acidobacteriota bacterium isolate KS4C013 NIM01542.1
Pseudomonadati Acidobacteriota unclassified Acidobacteriota Acidobacteriota bacterium isolate BS2bin.41 MEE8410954.1
Pseudomonadati Acidobacteriota unclassified Acidobacteriota Acidobacteriota bacterium isolate SCS1bin.33 MEE8184149.1
Pseudomonadati Acidobacteriota unclassified Acidobacteriota Acidobacteriota bacterium isolate NC groundwater 1261 Ag S0.2um 70 31 MBI4161381.1
Pseudomonadati Acidobacteriota unclassified Acidobacteriota Acidobacteriota bacterium isolate KW1S11 103 MCZ6670457.1
Pseudomonadati Acidobacteriota unclassified Acidobacteriota Acidobacteriota bacterium isolate Maj 2 bin.8 MFQ5669595.1
Pseudomonadati Acidobacteriota unclassified Acidobacteriota Acidobacteriota bacterium isolate N075bin69 TDI47357.1
Pseudomonadati Acidobacteriota unclassified Acidobacteriota Acidobacteriota bacterium isolate Maj 1 bin.20 MFQ5767653.1
Pseudomonadati Acidobacteriota unclassified Acidobacteriota Acidobacteriota bacterium isolate Maj 1 bin.40 MFQ5720691.1
Pseudomonadati Acidobacteriota unclassified Acidobacteriota Acidobacteriota bacterium isolate SL 7 3 NJN64498.1
Pseudomonadati Acidobacteriota unclassified Acidobacteriota Acidobacteriota bacterium isolate J065 RMF75143.1
Pseudomonadati Acidobacteriota unclassified Acidobacteriota Acidobacteriota bacterium isolate HyVt546 HHN75472.1
Pseudomonadati Acidobacteriota unclassified Acidobacteriota Acidobacteriota bacterium isolate bin529 UCF69513.1
Pseudomonadati Acidobacteriota unclassified Acidobacteriota Acidobacteriota bacterium isolate J045 RMG47556.1
Pseudomonadati Acidobacteriota unclassified Acidobacteriota Acidobacteriota bacterium isolate Go SlDig bin 260 MBP7146803.1
Pseudomonadati Acidobacteriota unclassified Acidobacteriota Acidobacteriota bacterium isolate Prado002 MCU0222745.1
Pseudomonadati Acidobacteriota unclassified Acidobacteriota Acidobacteriota bacterium isolate STLACI11 MFN7966423.1
Pseudomonadati Acidobacteriota unclassified Acidobacteriota Acidobacteriota bacterium isolate gp22 AA1 PYT07483.1
Pseudomonadati Acidobacteriota unclassified Acidobacteriota Acidobacteriota bacterium isolate SCS3bin.25 MEE9231708.1
Pseudomonadati Acidobacteriota unclassified Acidobacteriota Acidobacteriota bacterium isolate SCS3bin.16 MEE9217549.1
Pseudomonadati Acidobacteriota Candidatus Polarisedimenticolia Candidatus Polarisedimenticolia bacterium isolate SMAG U4503 HEV8335952.1
Pseudomonadati Acidobacteriota Candidatus Polarisedimenticolia Candidatus Polarisedimenticolia bacterium isolate SMAG U2458 HEU5179945.1
Pseudomonadati Acidobacteriota unclassified Acidobacteriota Acidobacteriota bacterium isolate AA152 PYQ13464.1
Pseudomonadati Acidobacteriota unclassified Acidobacteriota Acidobacteriota bacterium isolate PMV 1 maxbin.142 MCI0658828.1
Pseudomonadati Acidobacteriota unclassified Acidobacteriota Acidobacteriota bacterium isolate KW1S08 251 MCZ6696463.1
Pseudomonadati Acidobacteriota unclassified Acidobacteriota Acidobacteriota bacterium isolate K 2 bin.33 MFQ5876873.1
Pseudomonadati Acidobacteriota Candidatus Polarisedimenticolia Candidatus Polarisedimenticolia bacterium isolate M3 S metawrap bin.116 MGH9750443.1
Pseudomonadati Acidobacteriota unclassified Acidobacteriota Acidobacteriota bacterium isolate gp22 AA2 PYS95243.1
Pseudomonadati Acidobacteriota Candidatus Polarisedimenticolia Candidatus Polarisedimenticolia bacterium isolate SMAG U8939 HKQ98992.1
Pseudomonadati Acidobacteriota Candidatus Polarisedimenticolia Candidatus Polarisedimenticolia bacterium isolate SMAG U678 HET8945717.1
Pseudomonadati Acidobacteriota Candidatus Polarisedimenticolia Candidatus Polarisedimenticolia bacterium isolate SMAG U4477 HEV8198832.1

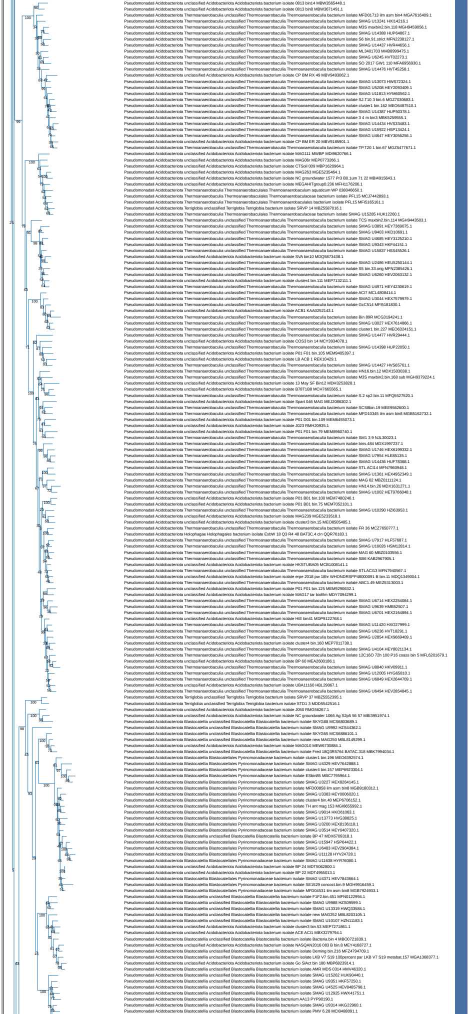
Pseudomonadati Acidobacteriota unclassified Acidobacteriota Acidobacteriota bacterium isolate 0813 bin14 MBW3565448.1
Pseudomonadati Acidobacteriota unclassified Acidobacteriota Acidobacteriota bacterium isolate 0813 bin8 MBW3671491.1
Pseudomonadati Acidobacteriota Thermoanaerobaculia unclassified Thermoanaerobaculia Thermoanaerobaculia bacterium isolate MFD01713 ilm asm bin4 MGA7616409.1
Pseudomonadati Acidobacteriota Thermoanaerobaculia unclassified Thermoanaerobaculia Thermoanaerobaculia bacterium isolate SMAG U13241 HXI14216.1
Pseudomonadati Acidobacteriota Thermoanaerobaculia unclassified Thermoanaerobaculia Thermoanaerobaculia bacterium isolate M3S maxbin2.bin.118 MGH9459056.1
Pseudomonadati Acidobacteriota Thermoanaerobaculia unclassified Thermoanaerobaculia Thermoanaerobaculia bacterium isolate SMAG U14388 HUP64867.1
Pseudomonadati Acidobacteriota Thermoanaerobaculia unclassified Thermoanaerobaculia Thermoanaerobaculia bacterium isolate S6 bin.91.strict MFN2238127.1
Pseudomonadati Acidobacteriota Thermoanaerobaculia unclassified Thermoanaerobaculia Thermoanaerobaculia bacterium isolate SMAG U14437 HVR44656.1
Pseudomonadati Acidobacteriota Thermoanaerobaculia unclassified Thermoanaerobaculia Thermoanaerobaculia bacterium isolate ML3401703 MHB8999475.1
Pseudomonadati Acidobacteriota Thermoanaerobaculia unclassified Thermoanaerobaculia Thermoanaerobaculia bacterium isolate SMAG U6245 HVT02273.1
Pseudomonadati Acidobacteriota Thermoanaerobaculia unclassified Thermoanaerobaculia Thermoanaerobaculia bacterium isolate SO 2017 GW1 110 MFA6956930.1
Pseudomonadati Acidobacteriota Thermoanaerobaculia unclassified Thermoanaerobaculia Thermoanaerobaculia bacterium isolate SMAG U14476 HVT45258.1
Pseudomonadati Acidobacteriota unclassified Acidobacteriota Acidobacteriota bacterium isolate CP BM RX 49 MBV9493062.1
Pseudomonadati Acidobacteriota Thermoanaerobaculia unclassified Thermoanaerobaculia Thermoanaerobaculia bacterium isolate SMAG U13073 HWS72324.1
Pseudomonadati Acidobacteriota Thermoanaerobaculia unclassified Thermoanaerobaculia Thermoanaerobaculia bacterium isolate SMAG U5208 HEY2093409.1
Pseudomonadati Acidobacteriota Thermoanaerobaculia unclassified Thermoanaerobaculia Thermoanaerobaculia bacterium isolate SMAG U11813 HYM60562.1
Pseudomonadati Acidobacteriota Thermoanaerobaculia unclassified Thermoanaerobaculia Thermoanaerobaculia bacterium isolate SJ.T10 3 bin.6 MGZ7030683.1
Pseudomonadati Acidobacteriota Thermoanaerobaculia unclassified Thermoanaerobaculia Thermoanaerobaculia bacterium isolate cluster1 bin.162 MEO6487510.1
Pseudomonadati Acidobacteriota Thermoanaerobaculia unclassified Thermoanaerobaculia Thermoanaerobaculia bacterium isolate SMAG U14387 HUP50378.1
Pseudomonadati Acidobacteriota Thermoanaerobaculia unclassified Thermoanaerobaculia Thermoanaerobaculia bacterium isolate 3 4 m bin3 MBK5259555.1
Pseudomonadati Acidobacteriota Thermoanaerobaculia unclassified Thermoanaerobaculia Thermoanaerobaculia bacterium isolate SMAG U14434 HVS33483.1
Pseudomonadati Acidobacteriota Thermoanaerobaculia unclassified Thermoanaerobaculia Thermoanaerobaculia bacterium isolate SMAG U15922 HSP13424.1
Pseudomonadati Acidobacteriota Thermoanaerobaculia unclassified Thermoanaerobaculia Thermoanaerobaculia bacterium isolate SMAG U4647 HEY3056256.1
Pseudomonadati Acidobacteriota unclassified Acidobacteriota Acidobacteriota bacterium isolate CP BM ER 20 MBV9185901.1
Pseudomonadati Acidobacteriota Thermoanaerobaculia unclassified Thermoanaerobaculia Thermoanaerobaculia bacterium isolate TP.T20 1 bin.67 MGZ5477671.1
Pseudomonadati Acidobacteriota unclassified Acidobacteriota Acidobacteriota bacterium isolate MAG111 MWBP MDI9620766.1
Pseudomonadati Acidobacteriota unclassified Acidobacteriota Acidobacteriota bacterium isolate MAG06r MEP0773266.1
Pseudomonadati Acidobacteriota unclassified Acidobacteriota Acidobacteriota bacterium isolate CTSoil 009 MBP1620964.1
Pseudomonadati Acidobacteriota unclassified Acidobacteriota Acidobacteriota bacterium isolate MAG263 MGE5235464.1
Pseudomonadati Acidobacteriota unclassified Acidobacteriota Acidobacteriota bacterium isolate NC groundwater 1577 Pr3 B0.1um 71 22 MBI4915643.1
Pseudomonadati Acidobacteriota unclassified Acidobacteriota Acidobacteriota bacterium isolate MEGAHITgroup0.236 MFH1176206.1
Pseudomonadati Acidobacteriota Thermoanaerobaculia Thermoanaerobaculales Thermoanaerobaculum aquaticum WP 038046650.1
Pseudomonadati Acidobacteriota Thermoanaerobaculia Thermoanaerobaculales Thermoanaerobaculaceae bacterium isolate PFL15 MCJ7442893.1
Pseudomonadati Acidobacteriota Thermoanaerobaculia Thermoanaerobaculales Thermoanaerobaculales bacterium isolate PFL15 MFI5165161.1
Pseudomonadati Acidobacteriota Terriglobia unclassified Terriglobia Terriglobia bacterium isolate SRVP 14 MBZ5587016.1
Pseudomonadati Acidobacteriota Thermoanaerobaculia Thermoanaerobaculales Thermoanaerobaculaceae bacterium isolate SMAG U15285 HUK12260.1
Pseudomonadati Acidobacteriota Thermoanaerobaculia unclassified Thermoanaerobaculia Thermoanaerobaculia bacterium isolate TCS maxbin2.bin.114 MGH9443503.1
Pseudomonadati Acidobacteriota Thermoanaerobaculia unclassified Thermoanaerobaculia Thermoanaerobaculia bacterium isolate SMAG U3891 HEY7369075.1
Pseudomonadati Acidobacteriota Thermoanaerobaculia unclassified Thermoanaerobaculia Thermoanaerobaculia bacterium isolate SMAG U9403 HKD10691.1
Pseudomonadati Acidobacteriota Thermoanaerobaculia unclassified Thermoanaerobaculia Thermoanaerobaculia bacterium isolate SMAG U4685 HEY3125210.1
Pseudomonadati Acidobacteriota Thermoanaerobaculia unclassified Thermoanaerobaculia Thermoanaerobaculia bacterium isolate SMAG U9343 HKF44151.1
Pseudomonadati Acidobacteriota Thermoanaerobaculia unclassified Thermoanaerobaculia Thermoanaerobaculia bacterium isolate SMAG U15837 HSS45526.1
Pseudomonadati Acidobacteriota unclassified Acidobacteriota Acidobacteriota bacterium isolate SVA bin10 MDQ5873438.1
Pseudomonadati Acidobacteriota Thermoanaerobaculia unclassified Thermoanaerobaculia Thermoanaerobaculia bacterium isolate SMAG U2486 HEU5250144.1
Pseudomonadati Acidobacteriota Thermoanaerobaculia unclassified Thermoanaerobaculia Thermoanaerobaculia bacterium isolate S5 bin.33.orig MFN2385426.1
Pseudomonadati Acidobacteriota Thermoanaerobaculia unclassified Thermoanaerobaculia Thermoanaerobaculia bacterium isolate SMAG U6260 HEV2063132.1
Pseudomonadati Acidobacteriota unclassified Acidobacteriota Acidobacteriota bacterium isolate cluster4 bin.111 MEP7132111.1
Pseudomonadati Acidobacteriota Thermoanaerobaculia unclassified Thermoanaerobaculia Thermoanaerobaculia bacterium isolate SMAG U4971 HEY4230619.1
Pseudomonadati Acidobacteriota Thermoanaerobaculia unclassified Thermoanaerobaculia Thermoanaerobaculia bacterium isolate ACI7 MCL4808414.1
Pseudomonadati Acidobacteriota Thermoanaerobaculia unclassified Thermoanaerobaculia Thermoanaerobaculia bacterium isolate SMAG U3044 HEX7579979.1
Pseudomonadati Acidobacteriota Thermoanaerobaculia unclassified Thermoanaerobaculia Thermoanaerobaculia bacterium isolate GcC514 MFI5181830.1
Pseudomonadati Acidobacteriota unclassified Acidobacteriota Acidobacteriota bacterium isolate ACB1 KAA0252143.1
Pseudomonadati Acidobacteriota Thermoanaerobaculia unclassified Thermoanaerobaculia Thermoanaerobaculia bacterium isolate Bin 89R MCG3194241.1
Pseudomonadati Acidobacteriota Thermoanaerobaculia unclassified Thermoanaerobaculia Thermoanaerobaculia bacterium isolate SMAG U3027 HEX7614866.1
Pseudomonadati Acidobacteriota Thermoanaerobaculia unclassified Thermoanaerobaculia Thermoanaerobaculia bacterium isolate cluster1 bin.227 MEO6324151.1
Pseudomonadati Acidobacteriota Thermoanaerobaculia unclassified Thermoanaerobaculia Thermoanaerobaculia bacterium isolate SMAG U14477 HVR29444.1
Pseudomonadati Acidobacteriota unclassified Acidobacteriota Acidobacteriota bacterium isolate COS3 bin 14 MCY3934078.1
Pseudomonadati Acidobacteriota Thermoanaerobaculia unclassified Thermoanaerobaculia Thermoanaerobaculia bacterium isolate SMAG U14398 HUP22050.1
Pseudomonadati Acidobacteriota unclassified Acidobacteriota Acidobacteriota bacterium isolate P01 F01 bin.105 MEM9405397.1
Pseudomonadati Acidobacteriota unclassified Acidobacteriota Acidobacteriota bacterium isolate LB ACB 1 REK10429.1
Pseudomonadati Acidobacteriota Thermoanaerobaculia unclassified Thermoanaerobaculia Thermoanaerobaculia bacterium isolate SMAG U14427 HVS65761.1
Pseudomonadati Acidobacteriota Thermoanaerobaculia unclassified Thermoanaerobaculia Thermoanaerobaculia bacterium isolate HN16.bin.12 MDX1503038.1
Pseudomonadati Acidobacteriota Thermoanaerobaculia unclassified Thermoanaerobaculia Thermoanaerobaculia bacterium isolate M3S maxbin2.bin.168 sub MGH9379224.1
Pseudomonadati Acidobacteriota unclassified Acidobacteriota Acidobacteriota bacterium isolate 13 May SF Bin12 MDH3253828.1
Pseudomonadati Acidobacteriota unclassified Acidobacteriota Acidobacteriota bacterium isolate B78T1B8 MCH7665565.1
Pseudomonadati Acidobacteriota Thermoanaerobaculia unclassified Thermoanaerobaculia Thermoanaerobaculia bacterium isolate S.2 sp2 bin.11 MFQ5527520.1
Pseudomonadati Acidobacteriota unclassified Acidobacteriota Acidobacteriota bacterium isolate Spart 046 MAG MEJ2086302.1
Pseudomonadati Acidobacteriota Thermoanaerobaculia unclassified Thermoanaerobaculia Thermoanaerobaculia bacterium isolate SCS8bin.19 MEE9562600.1
Pseudomonadati Acidobacteriota Thermoanaerobaculia unclassified Thermoanaerobaculia Thermoanaerobaculia bacterium isolate MFD10345 ilm asm bin8 MGB5162732.1
Pseudomonadati Acidobacteriota unclassified Acidobacteriota Acidobacteriota bacterium isolate P01 D01 bin.109 MEM6455073.1
Pseudomonadati Acidobacteriota unclassified Acidobacteriota Acidobacteriota bacterium isolate J023 RMH20935.1
Pseudomonadati Acidobacteriota unclassified Acidobacteriota Acidobacteriota bacterium isolate P01 F01 bin.79 MEM8960740.1
Pseudomonadati Acidobacteriota Thermoanaerobaculia unclassified Thermoanaerobaculia Thermoanaerobaculia bacterium isolate SM1 3 9 NJL30023.1
Pseudomonadati Acidobacteriota Thermoanaerobaculia unclassified Thermoanaerobaculia Thermoanaerobaculia bacterium isolate bins.484 MDX1997237.1
Pseudomonadati Acidobacteriota Thermoanaerobaculia unclassified Thermoanaerobaculia Thermoanaerobaculia bacterium isolate SMAG U1746 HEX6199332.1
Pseudomonadati Acidobacteriota Thermoanaerobaculia unclassified Thermoanaerobaculia Thermoanaerobaculia bacterium isolate SMAG U7954 HLE85135.1
Pseudomonadati Acidobacteriota Thermoanaerobaculia unclassified Thermoanaerobaculia Thermoanaerobaculia bacterium isolate SMAG U14436 HUF78368.1
Pseudomonadati Acidobacteriota Thermoanaerobaculia unclassified Thermoanaerobaculia Thermoanaerobaculia bacterium isolate STL ACI14 MFN7960948.1
Pseudomonadati Acidobacteriota Thermoanaerobaculia unclassified Thermoanaerobaculia Thermoanaerobaculia bacterium isolate SMAG U1361 HEX4952349.1
Pseudomonadati Acidobacteriota Thermoanaerobaculia unclassified Thermoanaerobaculia Thermoanaerobaculia bacterium isolate MAG 62 MBZ0111124.1
Pseudomonadati Acidobacteriota Thermoanaerobaculia unclassified Thermoanaerobaculia Thermoanaerobaculia bacterium isolate HN14.bin.26 MDX1631271.1
Pseudomonadati Acidobacteriota Thermoanaerobaculia unclassified Thermoanaerobaculia Thermoanaerobaculia bacterium isolate SMAG U1002 HET9766048.1
Pseudomonadati Acidobacteriota unclassified Acidobacteriota Acidobacteriota bacterium isolate P01 B01 bin.100 MEM7480246.1
Pseudomonadati Acidobacteriota unclassified Acidobacteriota Acidobacteriota bacterium isolate P01 B01 bin.75 MEM7052101.1
Pseudomonadati Acidobacteriota Thermoanaerobaculia unclassified Thermoanaerobaculia Thermoanaerobaculia bacterium isolate SMAG U10290 HZI63953.1
Pseudomonadati Acidobacteriota unclassified Acidobacteriota Acidobacteriota bacterium isolate MAG239 MGE5233518.1
Pseudomonadati Acidobacteriota unclassified Acidobacteriota Acidobacteriota bacterium isolate cluster3 bin.15 MEO8505485.1
Pseudomonadati Acidobacteriota Thermoanaerobaculia unclassified Thermoanaerobaculia Thermoanaerobaculia bacterium isolate FR 36 MCZ7650777.1
Pseudomonadati Acidobacteriota Holophagae Holophagales Holophagales bacterium isolate EsbW 18 Q3 R4 48 BAT3C.4 cln QQR76183.1
Pseudomonadati Acidobacteriota Thermoanaerobaculia unclassified Thermoanaerobaculia Thermoanaerobaculia bacterium isolate SMAG U7917 HLF57687.1
Pseudomonadati Acidobacteriota Thermoanaerobaculia unclassified Thermoanaerobaculia Thermoanaerobaculia bacterium isolate SMAG U16026 HSM12814.1
Pseudomonadati Acidobacteriota Thermoanaerobaculia unclassified Thermoanaerobaculia Thermoanaerobaculia bacterium isolate MAG 60 MBZ0103556.1
Pseudomonadati Acidobacteriota Thermoanaerobaculia unclassified Thermoanaerobaculia Thermoanaerobaculia bacterium isolate SB6 KAB2967905.1
Pseudomonadati Acidobacteriota unclassified Acidobacteriota Acidobacteriota bacterium isolate HKSTUBA05 MCB1008141.1
Pseudomonadati Acidobacteriota Thermoanaerobaculia unclassified Thermoanaerobaculia Thermoanaerobaculia bacterium isolate STLACI13 MFN7940567.1
Pseudomonadati Acidobacteriota unclassified Acidobacteriota Acidobacteriota bacterium isolate erpe 2018 pw 18hr WHONDRSPP48000091 B bin.11 MDQ1349004.1
Pseudomonadati Acidobacteriota Thermoanaerobaculia unclassified Thermoanaerobaculia Thermoanaerobaculia bacterium isolate ABC1.49 MEZ5313003.1
Pseudomonadati Acidobacteriota unclassified Acidobacteriota Acidobacteriota bacterium isolate P01 F01 bin.125 MEM9290632.1
Pseudomonadati Acidobacteriota unclassified Acidobacteriota Acidobacteriota bacterium isolate MAG17 tar biofilm MDY7094299.1
Pseudomonadati Acidobacteriota Thermoanaerobaculia unclassified Thermoanaerobaculia Thermoanaerobaculia bacterium isolate SMAG U6714 HEX2254084.1
Pseudomonadati Acidobacteriota Thermoanaerobaculia unclassified Thermoanaerobaculia Thermoanaerobaculia bacterium isolate SMAG U9639 HMB52507.1
Pseudomonadati Acidobacteriota Thermoanaerobaculia unclassified Thermoanaerobaculia Thermoanaerobaculia bacterium isolate SMAG U6701 HEX2164894.1
Pseudomonadati Acidobacteriota unclassified Acidobacteriota Acidobacteriota bacterium isolate HIE bin41 MDP9122768.1
Pseudomonadati Acidobacteriota Thermoanaerobaculia unclassified Thermoanaerobaculia Thermoanaerobaculia bacterium isolate SMAG U11420 HXO27999.1
Pseudomonadati Acidobacteriota Thermoanaerobaculia unclassified Thermoanaerobaculia Thermoanaerobaculia bacterium isolate SMAG U6236 HVT18291.1
Pseudomonadati Acidobacteriota Thermoanaerobaculia unclassified Thermoanaerobaculia Thermoanaerobaculia bacterium isolate SMAG U2854 HEX9669409.1
Pseudomonadati Acidobacteriota unclassified Acidobacteriota Acidobacteriota bacterium isolate cluster4 bin.160 MEP7011738.1
Pseudomonadati Acidobacteriota Thermoanaerobaculia unclassified Thermoanaerobaculia Thermoanaerobaculia bacterium isolate SMAG U4104 HEY8021134.1
Pseudomonadati Acidobacteriota Thermoanaerobaculia unclassified Thermoanaerobaculia Thermoanaerobaculia bacterium isolate 12C16O 72h 100 P16 coass bin 5 MFL6201679.1
Pseudomonadati Acidobacteriota unclassified Acidobacteriota Acidobacteriota bacterium isolate BP 60 MEA2600186.1
Pseudomonadati Acidobacteriota Thermoanaerobaculia unclassified Thermoanaerobaculia Thermoanaerobaculia bacterium isolate SMAG U8840 HKV09911.1
Pseudomonadati Acidobacteriota Thermoanaerobaculia unclassified Thermoanaerobaculia Thermoanaerobaculia bacterium isolate SMAG U12005 HYG65810.1
Pseudomonadati Acidobacteriota Thermoanaerobaculia unclassified Thermoanaerobaculia Thermoanaerobaculia bacterium isolate SMAG U6849 HEX2644709.1
Pseudomonadati Acidobacteriota unclassified Acidobacteriota Acidobacteriota bacterium isolate UBA11160 HBL29067.1
Pseudomonadati Acidobacteriota Thermoanaerobaculia unclassified Thermoanaerobaculia Thermoanaerobaculia bacterium isolate SMAG U6494 HEV2854845.1
Pseudomonadati Acidobacteriota Terriglobia unclassified Terriglobia Terriglobia bacterium isolate SRVP 37 MBZ5552395.1
Pseudomonadati Acidobacteriota Terriglobia unclassified Terriglobia Terriglobia bacterium isolate STD1 3 MDD5542516.1
Pseudomonadati Acidobacteriota unclassified Acidobacteriota Acidobacteriota bacterium isolate J050 RMG56267.1
Pseudomonadati Acidobacteriota unclassified Acidobacteriota Acidobacteriota bacterium isolate NC groundwater 1066 Ag S2p5 56 57 MBI3951974.1
Pseudomonadati Acidobacteriota Blastocatellia unclassified Blastocatellia Blastocatellia bacterium isolate SKYG88 MCS6803689.1
Pseudomonadati Acidobacteriota Blastocatellia unclassified Blastocatellia Blastocatellia bacterium isolate SMAG U9992 HZS44362.1
Pseudomonadati Acidobacteriota Blastocatellia unclassified Blastocatellia Blastocatellia bacterium isolate SKYG65 MCS6886101.1
Pseudomonadati Acidobacteriota Blastocatellia unclassified Blastocatellia Blastocatellia bacterium isolate new MAG250 MBL8149299.1
Pseudomonadati Acidobacteriota unclassified Acidobacteriota Acidobacteriota bacterium isolate MAG010 MEW6730884.1
Pseudomonadati Acidobacteriota Blastocatellia unclassified Blastocatellia Blastocatellia bacterium isolate Fred 18Q3R5764 BATAC.318 MBK7994034.1
Pseudomonadati Acidobacteriota Blastocatellia Blastocatellales Pyrinomonadaceae bacterium isolate cluster1 bin.196 MEO6392574.1
Pseudomonadati Acidobacteriota Blastocatellia Blastocatellales Pyrinomonadaceae bacterium isolate SMAG U4329 HEV7642888.1
Pseudomonadati Acidobacteriota Blastocatellia Blastocatellales Pyrinomonadaceae bacterium isolate cluster4 bin.157 MEP6923304.1
Pseudomonadati Acidobacteriota Blastocatellia Blastocatellales Pyrinomonadaceae bacterium isolate ESbin85 MBC7795964.1
Pseudomonadati Acidobacteriota Blastocatellia Blastocatellales Pyrinomonadaceae bacterium isolate SMAG U3227 HEX8264145.1
Pseudomonadati Acidobacteriota Blastocatellia Blastocatellales Pyrinomonadaceae bacterium isolate MFD00858 ilm asm bin8 MGB9180312.1
Pseudomonadati Acidobacteriota Blastocatellia Blastocatellales Pyrinomonadaceae bacterium isolate SMAG U3383 HEY0006020.1
Pseudomonadati Acidobacteriota Blastocatellia Blastocatellales Pyrinomonadaceae bacterium isolate cluster4 bin.40 MEP6706152.1
Pseudomonadati Acidobacteriota Blastocatellia Blastocatellales Pyrinomonadaceae bacterium isolate TH ant mag 153 MGI8655992.1
Pseudomonadati Acidobacteriota Blastocatellia Blastocatellales Pyrinomonadaceae bacterium isolate SMAG U9014 HKO61063.1
Pseudomonadati Acidobacteriota Blastocatellia Blastocatellales Pyrinomonadaceae bacterium isolate SMAG U13773 HVG38825.1
Pseudomonadati Acidobacteriota Blastocatellia Blastocatellales Pyrinomonadaceae bacterium isolate SMAG U3200 HEX8136118.1
Pseudomonadati Acidobacteriota Blastocatellia Blastocatellales Pyrinomonadaceae bacterium isolate SMAG U3514 HEY0407320.1
Pseudomonadati Acidobacteriota Blastocatellia unclassified Blastocatellia Blastocatellia bacterium isolate BP 47 MDX6709318.1
Pseudomonadati Acidobacteriota Blastocatellia Blastocatellales Pyrinomonadaceae bacterium isolate SMAG U15947 HSP64422.1
Pseudomonadati Acidobacteriota Blastocatellia Blastocatellales Pyrinomonadaceae bacterium isolate SMAG U6483 HEV2904384.1
Pseudomonadati Acidobacteriota Blastocatellia Blastocatellales Pyrinomonadaceae bacterium isolate SMAG U11128 HYV24728.1
Pseudomonadati Acidobacteriota Blastocatellia Blastocatellales Pyrinomonadaceae bacterium isolate SMAG U11638 HYR76080.1
Pseudomonadati Acidobacteriota unclassified Acidobacteriota Acidobacteriota bacterium isolate BP 24 MDT5062800.1
Pseudomonadati Acidobacteriota unclassified Acidobacteriota Acidobacteriota bacterium isolate BP 22 MDT4955013.1
Pseudomonadati Acidobacteriota Blastocatellia Blastocatellales Pyrinomonadaceae bacterium isolate SMAG U4371 HEV7843664.1
Pseudomonadati Acidobacteriota Blastocatellia Blastocatellales Pyrinomonadaceae bacterium isolate SE1529 concoct.bin.9 MGH9916459.1
Pseudomonadati Acidobacteriota Blastocatellia Blastocatellales Pyrinomonadaceae bacterium isolate MFD04531 ilm asm bin8 MGB7924933.1
Pseudomonadati Acidobacteriota Blastocatellia unclassified Blastocatellia Blastocatellia bacterium isolate F1F2.bin.451 MFN0122994.1
Pseudomonadati Acidobacteriota Blastocatellia unclassified Blastocatellia Blastocatellia bacterium isolate SMAG U9988 HZS09599.1
Pseudomonadati Acidobacteriota Blastocatellia unclassified Blastocatellia Blastocatellia bacterium isolate SMAG U13319 HWQ33584.1
Pseudomonadati Acidobacteriota Blastocatellia unclassified Blastocatellia Blastocatellia bacterium isolate new MAG252 MBL8203105.1
Pseudomonadati Acidobacteriota Blastocatellia unclassified Blastocatellia Blastocatellia bacterium isolate SMAG U10107 HZN11183.1
Pseudomonadati Acidobacteriota unclassified Acidobacteriota Acidobacteriota bacterium isolate cluster3 bin.53 MEP7271861.1
Pseudomonadati Acidobacteriota unclassified Acidobacteriota Acidobacteriota bacterium isolate ACE ACI1 MBK3279794.1
Pseudomonadati Acidobacteriota Blastocatellia unclassified Blastocatellia Blastocatellia bacterium isolate Bacteria.bin 4 MBO0721839.1
Pseudomonadati Acidobacteriota unclassified Acidobacteriota Acidobacteriota bacterium isolate NASQAN2016 083 B bin.8 MEY4168727.1
Pseudomonadati Acidobacteriota Blastocatellia unclassified Blastocatellia Blastocatellia bacterium isolate Deming bin.216 MFZ4794709.1
Pseudomonadati Acidobacteriota Blastocatellia unclassified Blastocatellia Blastocatellia bacterium isolate LKB V7 S19 100percent par LKB V7 S19 metabat.157 MGA1368377.1
Pseudomonadati Acidobacteriota unclassified Acidobacteriota Acidobacteriota bacterium isolate Go SlAct bin 180 MBP6823914.1
Pseudomonadati Acidobacteriota Blastocatellia unclassified Blastocatellia Blastocatellia bacterium isolate AMR MDS 0314 HMV46320.1
Pseudomonadati Acidobacteriota Blastocatellia unclassified Blastocatellia Blastocatellia bacterium isolate SMAG U15262 HUK90440.1
Pseudomonadati Acidobacteriota Blastocatellia unclassified Blastocatellia Blastocatellia bacterium isolate SMAG U9351 HKF57250.1
Pseudomonadati Acidobacteriota Blastocatellia unclassified Blastocatellia Blastocatellia bacterium isolate SMAG U4525 HEV8485798.1
Pseudomonadati Acidobacteriota Blastocatellia unclassified Blastocatellia Blastocatellia bacterium isolate SMAG U12925 HWX41751.1
Pseudomonadati Acidobacteriota Blastocatellia unclassified Blastocatellia Blastocatellia bacterium AA13 PYP90190.1
Pseudomonadati Acidobacteriota Blastocatellia unclassified Blastocatellia Blastocatellia bacterium isolate SMAG U9314 HKG22960.1
Pseudomonadati Acidobacteriota Blastocatellia unclassified Blastocatellia Blastocatellia bacterium isolate PMV 6.28 MCI0488091.1

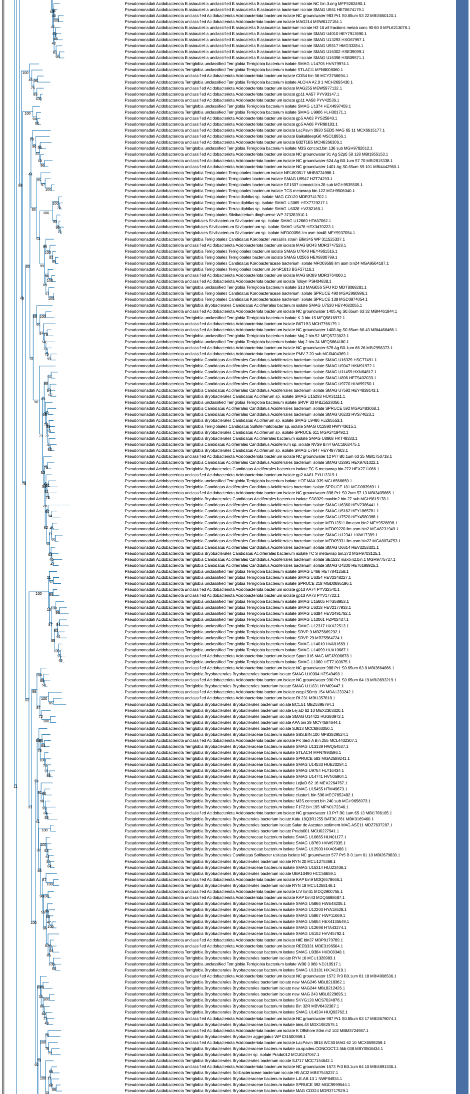
Pseudomonadati Acidobacteriota Blastocatellia unclassified Blastocatellia Blastocatellia bacterium isolate NC bin.3.orig MFP5263490.1
Pseudomonadati Acidobacteriota Blastocatellia unclassified Blastocatellia Blastocatellia bacterium isolate SMAG U561 HET8674179.1
Pseudomonadati Acidobacteriota unclassified Acidobacteriota Acidobacteriota bacterium isolate NC groundwater 983 Pr1 S0.65um 53 22 MBI3650120.1
Pseudomonadati Acidobacteriota unclassified Acidobacteriota Acidobacteriota bacterium isolate MAG214 MEW6127154.1
Pseudomonadati Acidobacteriota Blastocatellia unclassified Blastocatellia Blastocatellia bacterium isolate H3 16 all fractions metab conc 90 60 0 MFL6213078.1
Pseudomonadati Acidobacteriota Blastocatellia unclassified Blastocatellia Blastocatellia bacterium isolate SMAG U4010 HEY7913690.1
Pseudomonadati Acidobacteriota Blastocatellia unclassified Blastocatellia Blastocatellia bacterium isolate SMAG U13293 HXG67957.1
Pseudomonadati Acidobacteriota Blastocatellia unclassified Blastocatellia Blastocatellia bacterium isolate SMAG U9517 HMG33284.1
Pseudomonadati Acidobacteriota Blastocatellia unclassified Blastocatellia Blastocatellia bacterium isolate SMAG U16302 HSE39099.1
Pseudomonadati Acidobacteriota Blastocatellia unclassified Blastocatellia Blastocatellia bacterium isolate SMAG U16398 HSB09571.1
Pseudomonadati Acidobacteriota Terriglobia unclassified Terriglobia Terriglobia bacterium isolate SMAG U14705 HVN79974.1
Pseudomonadati Acidobacteriota Terriglobia unclassified Terriglobia Terriglobia bacterium isolate STLACI1 MFN8008060.1
Pseudomonadati Acidobacteriota unclassified Acidobacteriota Acidobacteriota bacterium isolate COS4 bin 56 MCY3756694.1
Pseudomonadati Acidobacteriota Terriglobia unclassified Terriglobia Terriglobia bacterium isolate ALOHA A2.0 1 MCH2695430.1
Pseudomonadati Acidobacteriota unclassified Acidobacteriota Acidobacteriota bacterium isolate MAG255 MEW5977132.1
Pseudomonadati Acidobacteriota unclassified Acidobacteriota Acidobacteriota bacterium isolate gp11 AA57 PYV93147.1
Pseudomonadati Acidobacteriota unclassified Acidobacteriota Acidobacteriota bacterium isolate gp11 AA58 PYV42536.1
Pseudomonadati Acidobacteriota Terriglobia unclassified Terriglobia Terriglobia bacterium isolate SMAG U1374 HEX4997459.1
Pseudomonadati Acidobacteriota Terriglobia unclassified Terriglobia Terriglobia bacterium isolate SMAG U9906 HLH30171.1
Pseudomonadati Acidobacteriota unclassified Acidobacteriota Acidobacteriota bacterium isolate gp5 AA63 PYS25840.1
Pseudomonadati Acidobacteriota unclassified Acidobacteriota Acidobacteriota bacterium isolate gp5 AA68 PYR98183.1
Pseudomonadati Acidobacteriota unclassified Acidobacteriota Acidobacteriota bacterium isolate LacPavin 0920 SED5 MAG 65 11 MCX6615177.1
Pseudomonadati Acidobacteriota unclassified Acidobacteriota Acidobacteriota bacterium isolate BaikaldeepG6 MSO18956.1
Pseudomonadati Acidobacteriota unclassified Acidobacteriota Acidobacteriota bacterium isolate B32T1B5 MCH8266106.1
Pseudomonadati Acidobacteriota Terriglobia unclassified Terriglobia Terriglobia bacterium isolate M3S concoct.bin.136 sub MGH9782612.1
Pseudomonadati Acidobacteriota unclassified Acidobacteriota Acidobacteriota bacterium isolate NC groundwater 91 Ag S2p5 58 128 MBI1955153.1
Pseudomonadati Acidobacteriota unclassified Acidobacteriota Acidobacteriota bacterium isolate NC groundwater 624 Ag B0.1um 57 70 MBI2815338.1
Pseudomonadati Acidobacteriota unclassified Acidobacteriota Acidobacteriota bacterium isolate NC groundwater 1401 Ag S0.65um 59 101 MBI4442960.1
Pseudomonadati Acidobacteriota Terriglobia Terriglobales Terriglobales bacterium isolate NR1800517 MHB8734986.1
Pseudomonadati Acidobacteriota Terriglobia Terriglobales Terriglobales bacterium isolate SMAG U9947 HZT74293.1
Pseudomonadati Acidobacteriota Terriglobia Terriglobales Terriglobales bacterium isolate SE1507 concoct.bin.28 sub MGH9535505.1
Pseudomonadati Acidobacteriota Terriglobia Terriglobales Terriglobales bacterium isolate TCS metawrap bin.122 MGH9506040.1
Pseudomonadati Acidobacteriota Terriglobia Terriglobales Terracidiphilus sp. isolate MAG CO120 MDR3741702.1
Pseudomonadati Acidobacteriota Terriglobia Terriglobales Terracidiphilus sp. isolate SMAG U3069 HEX7729217.1
Pseudomonadati Acidobacteriota Terriglobia Terriglobales Terracidiphilus sp. isolate SMAG U6028 HVZ82168.1
Pseudomonadati Acidobacteriota Terriglobia Terriglobales Silvibacterium dinghuense WP 373283910.1
Pseudomonadati Acidobacteriota Terriglobales Silvibacterium Silvibacterium sp. isolate SMAG U12660 HTA87062.1
Pseudomonadati Acidobacteriota Terriglobales Silvibacterium Silvibacterium sp. isolate SMAG U5478 HEX3470223.1
Pseudomonadati Acidobacteriota Terriglobales Silvibacterium Silvibacterium sp. isolate MFD00056 ilm asm bin48 MFY9937054.1
Pseudomonadati Acidobacteriota Terriglobia Terriglobales Candidatus Korobacter versatilis strain Ellin345 WP 011525337.1
Pseudomonadati Acidobacteriota unclassified Acidobacteriota Acidobacteriota bacterium isolate MAG BO43 MDR3747528.1
Pseudomonadati Acidobacteriota Terriglobia Terriglobales Terriglobales bacterium isolate SMAG U7640 HEY4961516.1
Pseudomonadati Acidobacteriota Terriglobia Terriglobales Terriglobales bacterium isolate SMAG U2566 HEX8800799.1
Pseudomonadati Acidobacteriota Terriglobia Terriglobales Candidatus Korobacteraceae bacterium isolate MFD09568 ilm asm bin24 MGA9564187.1
Pseudomonadati Acidobacteriota Terriglobia Terriglobales Terriglobales bacterium JemR1613 BGF27116.1
Pseudomonadati Acidobacteriota unclassified Acidobacteriota Acidobacteriota bacterium isolate MAG BO89 MDR3764060.1
Pseudomonadati Acidobacteriota unclassified Acidobacteriota Acidobacteriota bacterium isolate Tolsyn PSH04838.1
Pseudomonadati Acidobacteriota Terriglobia unclassified Terriglobia Terriglobia bacterium isolate S13 MAG056 SFU KD MDT8068281.1
Pseudomonadati Acidobacteriota Terriglobia Terriglobales Candidatus Korobacteraceae bacterium isolate SPRUCE 490 MGA2960996.1
Pseudomonadati Acidobacteriota Terriglobia Terriglobales Candidatus Korobacteraceae bacterium isolate SPRUCE 138 MGD0974054.1
Pseudomonadati Acidobacteriota Terriglobia Bryobacterales Candidatus Acidiferrales bacterium isolate SMAG U7530 HEY4682055.1
Pseudomonadati Acidobacteriota unclassified Acidobacteriota Acidobacteriota bacterium isolate NC groundwater 1405 Ag S0.65um 63 32 MBI4461844.1
Pseudomonadati Acidobacteriota Terriglobia unclassified Terriglobia Terriglobia bacterium isolate K 3 bin.15 MFQ5816972.1
Pseudomonadati Acidobacteriota unclassified Acidobacteriota Acidobacteriota bacterium isolate B6T1B3 MCH7766179.1
Pseudomonadati Acidobacteriota unclassified Acidobacteriota Acidobacteriota bacterium isolate NC groundwater 1408 Ag S0.65um 66 43 MBI4466466.1
Pseudomonadati Acidobacteriota Terriglobia unclassified Terriglobia Terriglobia bacterium isolate Maj 2 bin.52 MFQ5723823.1
Pseudomonadati Acidobacteriota Terriglobia unclassified Terriglobia Terriglobia bacterium isolate Maj 2 bin.34 MFQ5664180.1
Pseudomonadati Acidobacteriota unclassified Acidobacteriota Acidobacteriota bacterium isolate NC groundwater 678 Ag B0.1um 66 26 MBI2956373.1
Pseudomonadati Acidobacteriota unclassified Acidobacteriota Acidobacteriota bacterium isolate PMV 7.20 sub MCI0404369.1
Pseudomonadati Acidobacteriota Terriglobia Candidatus Acidiferrales Candidatus Acidiferrales bacterium isolate SMAG U16329 HSC77491.1
Pseudomonadati Acidobacteriota Terriglobia Candidatus Acidiferrales Candidatus Acidiferrales bacterium isolate SMAG U9047 HKM91972.1
Pseudomonadati Acidobacteriota Terriglobia Candidatus Acidiferrales Candidatus Acidiferrales bacterium isolate SMAG U11459 HXN64817.1
Pseudomonadati Acidobacteriota Terriglobia Candidatus Acidiferrales Candidatus Acidiferrales bacterium isolate SMAG U806 HET9402030.1
Pseudomonadati Acidobacteriota Terriglobia Candidatus Acidiferrales Candidatus Acidiferrales bacterium isolate SMAG U9770 HLW99750.1
Pseudomonadati Acidobacteriota Terriglobia Candidatus Acidiferrales Candidatus Acidiferrales bacterium isolate SMAG U7592 HEY4839143.1
Pseudomonadati Acidobacteriota Terriglobia Bryobacterales Candidatus Acidiferrum sp. isolate SMAG U15283 HUK31111.1
Pseudomonadati Acidobacteriota Terriglobia unclassified Terriglobia Terriglobia bacterium isolate SRVP 33 MBZ5528056.1
Pseudomonadati Acidobacteriota Terriglobia Candidatus Acidiferrales Candidatus Acidiferrales bacterium isolate SPRUCE 592 MGA2483068.1
Pseudomonadati Acidobacteriota Terriglobia Candidatus Acidiferrales Candidatus Acidiferrales bacterium isolate SMAG U6233 HVS74623.1
Pseudomonadati Acidobacteriota Terriglobia Bryobacterales Candidatus Acidiferrum sp. isolate SMAG U9486 HJZ65553.1
Pseudomonadati Acidobacteriota Terriglobia Terriglobales Candidatus Sulfotelmatobacter sp. isolate SMAG U12890 HWY43615.1
Pseudomonadati Acidobacteriota Terriglobia Bryobacterales Candidatus Acidiferrum sp. isolate SPRUCE 611 MGA2419492.1
Pseudomonadati Acidobacteriota Terriglobia Bryobacterales Candidatus Acidiferrales bacterium isolate SMAG U8868 HKT48333.1
Pseudomonadati Acidobacteriota Terriglobia Candidatus Acidiferrales Candidatus Acidiferrum sp. isolate NVS9 Bin4 GAC1663475.1
Pseudomonadati Acidobacteriota Terriglobia Bryobacterales Candidatus Acidiferrum sp. isolate SMAG U7647 HEY4977603.1
Pseudomonadati Acidobacteriota unclassified Acidobacteriota Acidobacteriota bacterium isolate NC groundwater 12 Pr7 B0.1um 63 25 MBI1750718.1
Pseudomonadati Acidobacteriota Terriglobia Candidatus Acidiferrales Candidatus Acidiferrales bacterium isolate SMAG U2891 HEX9761022.1
Pseudomonadati Acidobacteriota Terriglobia Bryobacterales Candidatus Acidiferrales bacterium isolate TC S metawrap bin.272 HEX2711066.1
Pseudomonadati Acidobacteriota unclassified Acidobacteriota Acidobacteriota bacterium isolate gp2 AA81 PYU13319.1
Pseudomonadati Acidobacteriota Terriglobia unclassified Terriglobia Terriglobia bacterium isolate HOT.MAX.039 MCL6566650.1
Pseudomonadati Acidobacteriota Terriglobia Candidatus Acidiferrales Candidatus Acidiferrales bacterium isolate SPRUCE 181 MGD0839691.1
Pseudomonadati Acidobacteriota unclassified Acidobacteriota Acidobacteriota bacterium isolate NC groundwater 898 Pr1 S0.2um 57 13 MBI3405665.1
Pseudomonadati Acidobacteriota Terriglobia Bryobacterales Candidatus Acidiferrales bacterium isolate SD8029 maxbin2.bin.27 sub MGH9815178.1
Pseudomonadati Acidobacteriota Terriglobia Candidatus Acidiferrales Candidatus Acidiferrales bacterium isolate SMAG U6360 HEV2386441.1
Pseudomonadati Acidobacteriota Terriglobia Candidatus Acidiferrales Candidatus Acidiferrales bacterium isolate SMAG U5162 HEY1865781.1
Pseudomonadati Acidobacteriota Terriglobia Candidatus Acidiferrales Candidatus Acidiferrales bacterium isolate SMAG U7520 HEY4580388.1
Pseudomonadati Acidobacteriota Terriglobia Candidatus Acidiferrales Candidatus Acidiferrales bacterium isolate MFD13511 ilm asm bin2 MFY9528898.1
Pseudomonadati Acidobacteriota Terriglobia Candidatus Acidiferrales Candidatus Acidiferrales bacterium isolate MFD09220 ilm asm bin2 MGA8231949.1
Pseudomonadati Acidobacteriota Terriglobia Candidatus Acidiferrales Candidatus Acidiferrales bacterium isolate SMAG U12341 HXW17389.1
Pseudomonadati Acidobacteriota Terriglobia Candidatus Acidiferrales Candidatus Acidiferrales bacterium isolate MFD05931 ilm asm bin22 MGA8074753.1
Pseudomonadati Acidobacteriota Terriglobia Candidatus Acidiferrales Candidatus Acidiferrales bacterium isolate SMAG U6614 HEV3253301.1
Pseudomonadati Acidobacteriota Terriglobia Bryobacterales Candidatus Acidiferrales bacterium isolate TC S metawrap bin.272 MGH9703125.1
Pseudomonadati Acidobacteriota Terriglobia Candidatus Acidiferrales Candidatus Acidiferrales bacterium isolate SE1532 maxbin2.bin.1 MGH9775727.1
Pseudomonadati Acidobacteriota Terriglobia Candidatus Acidiferrales Candidatus Acidiferrales bacterium isolate SMAG U4200 HET6198925.1
Pseudomonadati Acidobacteriota Terriglobia unclassified Terriglobia Terriglobia bacterium isolate SMAG U466 HET7841258.1
Pseudomonadati Acidobacteriota Terriglobia unclassified Terriglobia Terriglobia bacterium isolate SMAG U6354 HEV2348227.1
Pseudomonadati Acidobacteriota Terriglobia unclassified Terriglobia Terriglobia bacterium isolate SPRUCE 218 MGD0695196.1
Pseudomonadati Acidobacteriota unclassified Acidobacteriota Acidobacteriota bacterium isolate gp13 AA74 PYV32540.1
Pseudomonadati Acidobacteriota unclassified Acidobacteriota Acidobacteriota bacterium isolate gp13 AA73 PYV17722.1
Pseudomonadati Acidobacteriota Terriglobia unclassified Terriglobia Terriglobia bacterium isolate SMAG U15605 HTG58953.1
Pseudomonadati Acidobacteriota Terriglobia unclassified Terriglobia Terriglobia bacterium isolate SMAG U6318 HEV2177933.1
Pseudomonadati Acidobacteriota Terriglobia unclassified Terriglobia Terriglobia bacterium isolate SMAG U6384 HEV2491782.1
Pseudomonadati Acidobacteriota Terriglobia unclassified Terriglobia Terriglobia bacterium isolate SMAG U10061 HZP02437.1
Pseudomonadati Acidobacteriota Terriglobia unclassified Terriglobia Terriglobia bacterium isolate SMAG U12317 HXX22513.1
Pseudomonadati Acidobacteriota Terriglobia unclassified Terriglobia Terriglobia bacterium isolate SRVP 9 MBZ5669292.1
Pseudomonadati Acidobacteriota Terriglobia unclassified Terriglobia Terriglobia bacterium isolate SRVP 29 MBZ5564724.1
Pseudomonadati Acidobacteriota Terriglobia unclassified Terriglobia Terriglobia bacterium isolate SMAG U14010 HVA01669.1
Pseudomonadati Acidobacteriota Terriglobia unclassified Terriglobia Terriglobia bacterium isolate SMAG U14099 HUX10667.1
Pseudomonadati Acidobacteriota unclassified Acidobacteriota Acidobacteriota bacterium isolate Spart 016 MAG MEJ2006678.1
Pseudomonadati Acidobacteriota Terriglobia unclassified Terriglobia Terriglobia bacterium isolate SMAG U1060 HET7100675.1
Pseudomonadati Acidobacteriota unclassified Acidobacteriota Acidobacteriota bacterium isolate NC groundwater 988 Pr1 S0.65um 63 8 MBI3664866.1
Pseudomonadati Acidobacteriota Terriglobia Bryobacterales Bryobacterales bacterium isolate SMAG U10004 HZS49498.1
Pseudomonadati Acidobacteriota unclassified Acidobacteriota Acidobacteriota bacterium isolate NC groundwater 990 Pr1 S0.65um 64 19 MBI3693219.1
Pseudomonadati Acidobacteriota Terriglobia Bryobacterales Bryobacterales bacterium isolate SMAG U11831 HYM09447.1
Pseudomonadati Acidobacteriota unclassified Acidobacteriota Acidobacteriota bacterium isolate casp150mb.154 MDA1233242.1
Pseudomonadati Acidobacteriota unclassified Acidobacteriota Acidobacteriota bacterium isolate RI 231 MBI1357818.1
Pseudomonadati Acidobacteriota Terriglobia Bryobacterales Bryobacterales bacterium isolate BC1.51 MEZ5395794.1
Pseudomonadati Acidobacteriota Terriglobia Bryobacterales Bryobacterales bacterium isolate LejiaD 62 10 MEX2303320.1
Pseudomonadati Acidobacteriota Terriglobia Bryobacterales Bryobacterales bacterium isolate SMAG U14422 HUG80972.1
Pseudomonadati Acidobacteriota Terriglobia Bryobacterales Bryobacterales bacterium isolate APA bin 29 MCY4584644.1
Pseudomonadati Acidobacteriota Terriglobia Bryobacterales Bryobacterales bacterium isolate SJ613 MCC6863050.1
Pseudomonadati Acidobacteriota Terriglobia Bryobacterales Bryobacteraceae bacterium isolate SBS.BIN.100 MFB3829524.1
Pseudomonadati Acidobacteriota unclassified Acidobacteriota Acidobacteriota bacterium isolate FK Sedi A Bin.255 MCL4402307.1
Pseudomonadati Acidobacteriota Terriglobia Bryobacterales Bryobacteraceae bacterium isolate SMAG U13139 HWQ54537.1
Pseudomonadati Acidobacteriota Terriglobia Bryobacterales Bryobacteraceae bacterium isolate STLACI4 MFN7993596.1
Pseudomonadati Acidobacteriota Terriglobia Bryobacterales Bryobacteraceae bacterium isolate SPRUCE 583 MGA2589241.1
Pseudomonadati Acidobacteriota Terriglobia Bryobacterales Bryobacteraceae bacterium isolate SMAG U14533 HUE20284.1
Pseudomonadati Acidobacteriota Terriglobia Bryobacterales Bryobacteraceae bacterium isolate SMAG U9754 HLY16434.1
Pseudomonadati Acidobacteriota Terriglobia Bryobacterales Bryobacteraceae bacterium isolate SMAG U14741 HVN05904.1
Pseudomonadati Acidobacteriota Terriglobia Bryobacterales Bryobacteraceae bacterium isolate LejiaD 62 16 MEX2264767.1
Pseudomonadati Acidobacteriota Terriglobia Bryobacterales Bryobacteraceae bacterium isolate SMAG U15455 HTM49673.1
Pseudomonadati Acidobacteriota Terriglobia Bryobacterales Bryobacteraceae bacterium isolate cluster1 bin.596 MEO7652482.1
Pseudomonadati Acidobacteriota Terriglobia Bryobacterales Bryobacteraceae bacterium isolate M3S concoct.bin.240 sub MGH9656973.1
Pseudomonadati Acidobacteriota Terriglobia Bryobacterales Bryobacteraceae bacterium isolate F1F2.bin.195 MFN0172346.1
Pseudomonadati Acidobacteriota unclassified Acidobacteriota Acidobacteriota bacterium isolate NC groundwater 13 Pr7 B0.1um 65 13 MBI1786185.1
Pseudomonadati Acidobacteriota Terriglobia Bryobacterales Bryobacterales bacterium isolate Kalu 18Q3R1255 BAT3C.261 MBK9169460.1
Pseudomonadati Acidobacteriota Terriglobia Bryobacterales Bryobacterales bacterium isolate Salar de Ascotan sediment MAG ASE11 MDZ7637287.1
Pseudomonadati Acidobacteriota Terriglobia Bryobacterales Bryobacterales bacterium isolate Prado001 MCU0227941.1
Pseudomonadati Acidobacteriota Terriglobia Bryobacterales Bryobacteraceae bacterium isolate SMAG U10665 HLN01177.1
Pseudomonadati Acidobacteriota Terriglobia Bryobacterales Bryobacteraceae bacterium isolate SMAG U8769 HKW97935.1
Pseudomonadati Acidobacteriota Terriglobia Bryobacterales Bryobacteraceae bacterium isolate SMAG U12900 HXA06488.1
Pseudomonadati Acidobacteriota Terriglobia Bryobacterales Candidatus Solibacter usitatus isolate NC groundwater 577 Pr5 B 0.1um 61 10 MBI2679830.1
Pseudomonadati Acidobacteriota Terriglobia Bryobacterales Bryobacterales bacterium isolate RYN 20 MCU1275369.1
Pseudomonadati Acidobacteriota Terriglobia Bryobacterales Bryobacteraceae bacterium isolate SMAG U15314 HUJ23406.1
Pseudomonadati Acidobacteriota Terriglobia Bryobacterales Bryobacterales bacterium isolate UBA10490 HCC56659.1
Pseudomonadati Acidobacteriota unclassified Acidobacteriota Acidobacteriota bacterium isolate KAP bin9 MDQ6678666.1
Pseudomonadati Acidobacteriota Terriglobia Bryobacterales Bryobacterales bacterium isolate RYN 18 MCU1258146.1
Pseudomonadati Acidobacteriota unclassified Acidobacteriota Acidobacteriota bacterium isolate LIV bin31 MDQ2900755.1
Pseudomonadati Acidobacteriota unclassified Acidobacteriota Acidobacteriota bacterium isolate KAP bin43 MDQ6698687.1
Pseudomonadati Acidobacteriota Terriglobia Bryobacterales Bryobacteraceae bacterium isolate SMAG U5866 HWE48205.1
Pseudomonadati Acidobacteriota Terriglobia Bryobacterales Bryobacteraceae bacterium isolate SMAG U12203 HYA18528.1
Pseudomonadati Acidobacteriota Terriglobia Bryobacterales Bryobacteraceae bacterium isolate SMAG U5867 HWF11669.1
Pseudomonadati Acidobacteriota Terriglobia Bryobacterales Bryobacteraceae bacterium isolate SMAG U5654 HEX4135549.1
Pseudomonadati Acidobacteriota Terriglobia Bryobacterales Bryobacteraceae bacterium isolate SMAG U12698 HTA43274.1
Pseudomonadati Acidobacteriota Terriglobia Bryobacterales Bryobacteraceae bacterium isolate SMAG U6152 HVV45792.1
Pseudomonadati Acidobacteriota unclassified Acidobacteriota Acidobacteriota bacterium isolate HIE bin37 MDP9170789.1
Pseudomonadati Acidobacteriota unclassified Acidobacteriota Acidobacteriota bacterium isolate REEB331 MDE3196564.1
Pseudomonadati Acidobacteriota Terriglobia Bryobacterales Bryobacteraceae bacterium isolate SMAG U9384 HKD08348.1
Pseudomonadati Acidobacteriota Terriglobia Bryobacterales Bryobacterales bacterium isolate RYN 16 MCU1328983.1
Pseudomonadati Acidobacteriota Terriglobia unclassified Terriglobia Terriglobia bacterium isolate WB8 3 068 NDJ10517.1
Pseudomonadati Acidobacteriota Terriglobia Bryobacterales Bryobacteraceae bacterium isolate SMAG U13181 HXJ41218.1
Pseudomonadati Acidobacteriota unclassified Acidobacteriota Acidobacteriota bacterium isolate NC groundwater 1572 Pr3 B0.1um 61 18 MBI4906536.1
Pseudomonadati Acidobacteriota Terriglobia Bryobacterales Bryobacterales bacterium isolate new MAG246 MBL8218362.1
Pseudomonadati Acidobacteriota Terriglobia Bryobacterales Bryobacterales bacterium isolate new MAG244 MBL8212426.1
Pseudomonadati Acidobacteriota Terriglobia Bryobacterales Bryobacterales bacterium isolate new MAG 243 MBL8229695.1
Pseudomonadati Acidobacteriota Terriglobia Bryobacterales Bryobacteraceae bacterium isolate SKYG128 MCS7024876.1
Pseudomonadati Acidobacteriota Terriglobia Bryobacterales Bryobacteraceae bacterium isolate Bin 32R MBV6432387.1
Pseudomonadati Acidobacteriota Terriglobia Bryobacterales Bryobacteraceae bacterium isolate SMAG U14334 HUQ93762.1
Pseudomonadati Acidobacteriota unclassified Acidobacteriota Acidobacteriota bacterium isolate NC groundwater 987 Pr1 S0.65um 63 17 MBI3679074.1
Pseudomonadati Acidobacteriota Terriglobia Bryobacterales Bryobacteraceae bacterium isolate bins.48 MDX1982575.1
Pseudomonadati Acidobacteriota unclassified Acidobacteriota Acidobacteriota bacterium isolate K Offshore 80m m2 102 MBM3724987.1
Pseudomonadati Acidobacteriota Terriglobia Bryobacterales Bryobacter aggregatus WP 031500959.1
Pseudomonadati Acidobacteriota unclassified Acidobacteriota Acidobacteriota bacterium isolate LacPavin 0818 WC50 MAG 62 10 MCX6598259.1
Pseudomonadati Acidobacteriota Terriglobia Bryobacterales Bryobacteraceae bacterium isolate co.spades.CONCOCT.2.5kb 038 MBY0508434.1
Pseudomonadati Acidobacteriota Terriglobia Bryobacterales Bryobacter sp. isolate Prado012 MCU0247067.1
Pseudomonadati Acidobacteriota Terriglobia Bryobacterales Bryobacterales bacterium isolate SJ717 MCC7154642.1
Pseudomonadati Acidobacteriota unclassified Acidobacteriota Acidobacteriota bacterium isolate NC groundwater 1573 Pr3 B0.1um 64 10 MBI4891336.1
Pseudomonadati Acidobacteriota Terriglobia Bryobacterales Solibacteraceae bacterium isolate H5 ACI2 MBE7545237.1
Pseudomonadati Acidobacteriota Terriglobia Bryobacterales Bryobacteraceae bacterium isolate L.E.AB.13 1 NWF84934.1
Pseudomonadati Acidobacteriota Terriglobia Bryobacterales Bryobacteraceae bacterium isolate SPRUCE 392 MGC9999544.1
Pseudomonadati Acidobacteriota Terriglobia Bryobacterales Bryobacteraceae bacterium isolate MAG CO324 MDR3717929.1

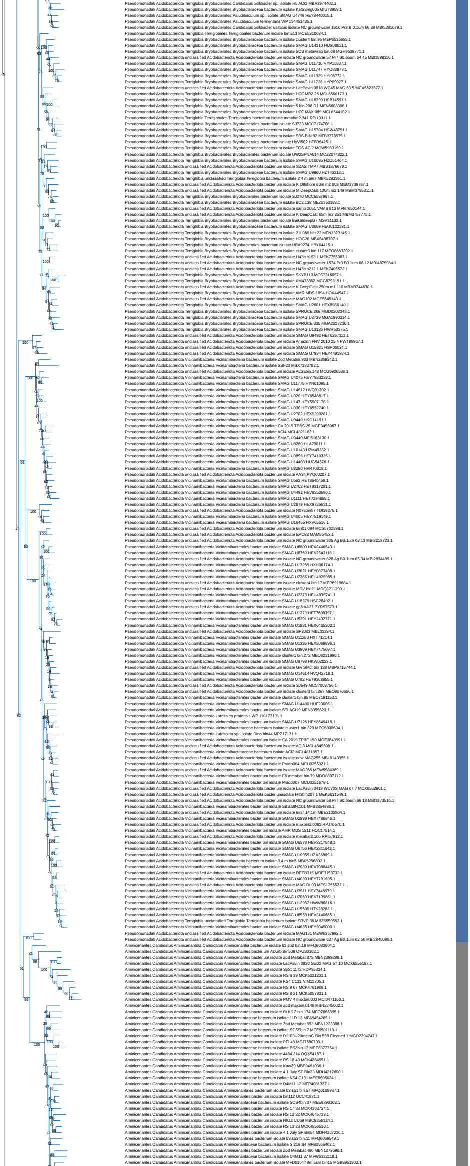
Pseudomonadati Acidobacteriota Terriglobia Bryobacterales Candidatus Solibacter sp. isolate H5 ACI2 MBA3974402.1
Pseudomonadati Acidobacteriota Terriglobia Bryobacterales Bryobacteraceae bacterium isolate KatS3mg005 GIU78959.1
Pseudomonadati Acidobacteriota Terriglobia Bryobacterales Paludibaculum sp. isolate SMAG U4748 HEY3440015.1
Pseudomonadati Acidobacteriota Terriglobia Bryobacterales Paludibaculum fermentans WP 194451435.1
Pseudomonadati Acidobacteriota Terriglobia Bryobacterales Candidatus Solibacter usitatus isolate NC groundwater 1610 Pr3 B 0.1um 66 38 MBI5281079.1
Pseudomonadati Acidobacteriota Terriglobia Terriglobales Terriglobales bacterium isolate bin.513 MCE5310034.1
Pseudomonadati Acidobacteriota Terriglobia Bryobacterales Bryobacteraceae bacterium isolate cluster4 bin.95 MEP6535655.1
Pseudomonadati Acidobacteriota Terriglobia Bryobacterales Bryobacteraceae bacterium isolate SMAG U14310 HUS08621.1
Pseudomonadati Acidobacteriota Terriglobia Bryobacterales Bryobacteraceae bacterium isolate SCS metawrap bin.68 MGH9628771.1
Pseudomonadati Acidobacteriota unclassified Acidobacteriota Acidobacteriota bacterium isolate NC groundwater 57 Pr7 S0.65um 64 45 MBI1898110.1
Pseudomonadati Acidobacteriota Terriglobia Bryobacterales Bryobacteraceae bacterium isolate SMAG U11716 HYP15537.1
Pseudomonadati Acidobacteriota Terriglobia Bryobacterales Bryobacteraceae bacterium isolate SMAG U11747 HYO83973.1
Pseudomonadati Acidobacteriota Terriglobia Bryobacterales Bryobacteraceae bacterium isolate SMAG U11929 HYI96772.1
Pseudomonadati Acidobacteriota Terriglobia Bryobacterales Bryobacteraceae bacterium isolate SMAG U11728 HYP09027.1
Pseudomonadati Acidobacteriota unclassified Acidobacteriota Acidobacteriota bacterium isolate LacPavin 0818 WC45 MAG 63 5 MCX6623377.1
Pseudomonadati Acidobacteriota Terriglobia Bryobacterales Bryobacteraceae bacterium isolate HOT.MB2.26 MCL6506173.1
Pseudomonadati Acidobacteriota Terriglobia Bryobacterales Bryobacteraceae bacterium isolate SMAG U16399 HSB14551.1
Pseudomonadati Acidobacteriota Terriglobia Bryobacterales Bryobacteraceae bacterium isolate 5 bin.208 R1 MEN6608398.1
Pseudomonadati Acidobacteriota Terriglobia Bryobacterales Bryobacteraceae bacterium isolate HOT.MAX.089 MCL6544182.1
Pseudomonadati Acidobacteriota Terriglobia Terriglobales Terriglobales bacterium isolate metabat2.341 RPI13311.1
Pseudomonadati Acidobacteriota Terriglobia Bryobacterales Bryobacterales bacterium isolate SJ723 MCC7174708.1
Pseudomonadati Acidobacteriota Terriglobia Bryobacterales Bryobacteraceae bacterium isolate SMAG U15704 HSW48751.1
Pseudomonadati Acidobacteriota Terriglobia Bryobacterales Bryobacteraceae bacterium isolate SBS.BIN.82 MFB3778575.1
Pseudomonadati Acidobacteriota Terriglobia Bryobacterales Bryobacterales bacterium isolate HyVt502 HFB98425.1
Pseudomonadati Acidobacteriota Terriglobia Bryobacterales Bryobacteraceae bacterium isolate TGS ACI2 MCW5983169.1
Pseudomonadati Acidobacteriota Terriglobia Bryobacterales Bryobacterales bacterium isolate UW2SPNA014 MCZ2074822.1
Pseudomonadati Acidobacteriota Terriglobia Bryobacterales Bryobacteraceae bacterium isolate SMAG U10095 HZO51464.1
Pseudomonadati Acidobacteriota unclassified Acidobacteriota Acidobacteriota bacterium isolate SZAS TMP7 MBS1876679.1
Pseudomonadati Acidobacteriota Terriglobia Bryobacterales Bryobacteraceae bacterium isolate SMAG U9960 HZT40213.1
Pseudomonadati Acidobacteriota Terriglobia unclassified Terriglobia Terriglobia bacterium isolate 3 4 m bin7 MBK5293361.1
Pseudomonadati Acidobacteriota unclassified Acidobacteriota Acidobacteriota bacterium isolate K Offshore 80m m2 003 MBM3739787.1
Pseudomonadati Acidobacteriota unclassified Acidobacteriota Acidobacteriota bacterium isolate M DeepCast 100m m2 149 MBM3795311.1
Pseudomonadati Acidobacteriota Terriglobia Bryobacterales Bryobacterales bacterium isolate SJ270 MCC6587987.1
Pseudomonadati Acidobacteriota Terriglobia Bryobacterales Bryobacteraceae bacterium isolate BC2.138 MEZ5353193.1
Pseudomonadati Acidobacteriota unclassified Acidobacteriota Acidobacteriota bacterium isolate samp 2051 VAMB 810 MFN7650144.1
Pseudomonadati Acidobacteriota unclassified Acidobacteriota Acidobacteriota bacterium isolate K DeepCast 65m m2 251 MBM3757775.1
Pseudomonadati Acidobacteriota Terriglobia Bryobacterales Bryobacterales bacterium isolate BaikaldeepG7 MSV31133.1
Pseudomonadati Acidobacteriota Terriglobia Bryobacterales Bryobacteraceae bacterium isolate SMAG U3669 HEU0122231.1
Pseudomonadati Acidobacteriota Terriglobia Bryobacterales Bryobacteraceae bacterium isolate 21l 069.bin.23 MFN3323145.1
Pseudomonadati Acidobacteriota Terriglobia Bryobacterales Bryobacteraceae bacterium isolate HOG28 MBX5496707.1
Pseudomonadati Acidobacteriota Terriglobia Bryobacterales Bryobacterales bacterium isolate UBA9274 HBY64415.1
Pseudomonadati Acidobacteriota Terriglobia Bryobacterales Bryobacteraceae bacterium isolate cluster3 bin.117 MEO8663292.1
Pseudomonadati Acidobacteriota unclassified Acidobacteriota Acidobacteriota bacterium isolate H43bin153 1 MEK7755387.1
Pseudomonadati Acidobacteriota unclassified Acidobacteriota Acidobacteriota bacterium isolate NC groundwater 1574 Pr3 B0.1um 66 12 MBI4875984.1
Pseudomonadati Acidobacteriota unclassified Acidobacteriota Acidobacteriota bacterium isolate H43bin212 1 MEK7405522.1
Pseudomonadati Acidobacteriota Terriglobia Bryobacterales Bryobacteraceae bacterium isolate SKYB110 MCS7316057.1
Pseudomonadati Acidobacteriota Terriglobia Bryobacterales Bryobacteraceae bacterium isolate KM433862 MGC8793151.1
Pseudomonadati Acidobacteriota unclassified Acidobacteriota Acidobacteriota bacterium isolate K DeepCast 250m m1 110 MBM3744630.1
Pseudomonadati Acidobacteriota Terriglobia Bryobacterales Bryobacteraceae bacterium isolate AMR MDS 1894 HOK44547.1
Pseudomonadati Acidobacteriota unclassified Acidobacteriota Acidobacteriota bacterium isolate MAG102 MGE5645143.1
Pseudomonadati Acidobacteriota Terriglobia Bryobacterales Bryobacteraceae bacterium isolate SMAG U2601 HEX8986140.1
Pseudomonadati Acidobacteriota Terriglobia Bryobacterales Bryobacteraceae bacterium isolate SPRUCE 368 MGD0202248.1
Pseudomonadati Acidobacteriota Terriglobia Bryobacterales Bryobacteraceae bacterium isolate SMAG U3739 MGA1990314.1
Pseudomonadati Acidobacteriota Terriglobia Bryobacterales Bryobacteraceae bacterium isolate SPRUCE 635 MGA2327236.1
Pseudomonadati Acidobacteriota Terriglobia Bryobacterales Bryobacteraceae bacterium isolate SMAG U13126 HWR53375.1
Pseudomonadati Acidobacteriota unclassified Acidobacteriota Acidobacteriota bacterium isolate SMAG U9492 HET6267112.1
Pseudomonadati Acidobacteriota unclassified Acidobacteriota Acidobacteriota bacterium isolate Amazon FNV 2010 25 4 PWT89967.1
Pseudomonadati Acidobacteriota unclassified Acidobacteriota Acidobacteriota bacterium isolate SMAG U15921 HSP06034.1
Pseudomonadati Acidobacteriota unclassified Acidobacteriota Acidobacteriota bacterium isolate SMAG U7984 HEY4491934.1
Pseudomonadati Acidobacteriota Vicinamibacteria Vicinamibacteria bacterium isolate Zod Metabat.903 MBN2369242.1
Pseudomonadati Acidobacteriota Vicinamibacteria Vicinamibacteria bacterium isolate SSF20 MBX7183792.1
Pseudomonadati Acidobacteriota unclassified Acidobacteriota Acidobacteriota bacterium isolate ALSabin.143 MCG6926586.1
Pseudomonadati Acidobacteriota Vicinamibacteria Vicinamibacteria bacterium isolate SMAG U4075 HEY7923233.1
Pseudomonadati Acidobacteriota Vicinamibacteria Vicinamibacteria bacterium isolate SMAG U11775 HYN01095.1
Pseudomonadati Acidobacteriota Vicinamibacteria Vicinamibacteria bacterium isolate SMAG U14612 HVQ31302.1
Pseudomonadati Acidobacteriota Vicinamibacteria Vicinamibacteria bacterium isolate SMAG U320 HEY6546617.1
Pseudomonadati Acidobacteriota Vicinamibacteria Vicinamibacteria bacterium isolate SMAG U147 HEY5907178.1
Pseudomonadati Acidobacteriota Vicinamibacteria Vicinamibacteria bacterium isolate SMAG U330 HEY6552740.1
Pseudomonadati Acidobacteriota Vicinamibacteria Vicinamibacteria bacterium isolate SMAG U2702 HEX9203281.1
Pseudomonadati Acidobacteriota Vicinamibacteria Vicinamibacteria bacterium isolate SMAG U9440 HKC14151.1
Pseudomonadati Acidobacteriota Vicinamibacteria Vicinamibacteria bacterium isolate CA 2019 TPBS 25 MGE0456067.1
Pseudomonadati Acidobacteriota Vicinamibacteria Vicinamibacteria bacterium isolate ACI4 MCL4821162.1
Pseudomonadati Acidobacteriota Vicinamibacteria Vicinamibacteria bacterium isolate SMAG U9440 MFI5183130.1
Pseudomonadati Acidobacteriota Vicinamibacteria Vicinamibacteria bacterium isolate SMAG U8280 HLA79811.1
Pseudomonadati Acidobacteriota Vicinamibacteria Vicinamibacteria bacterium isolate SMAG U10143 HZM49332.1
Pseudomonadati Acidobacteriota Vicinamibacteria Vicinamibacteria bacterium isolate SMAG U3896 HEY7410335.1
Pseudomonadati Acidobacteriota Vicinamibacteria Vicinamibacteria bacterium isolate SMAG U14403 HUG54376.1
Pseudomonadati Acidobacteriota Vicinamibacteria Vicinamibacteria bacterium isolate SMAG U8280 HVR70318.1
Pseudomonadati Acidobacteriota unclassified Acidobacteriota Acidobacteriota bacterium isolate AA34 PYQ00207.1
Pseudomonadati Acidobacteriota Vicinamibacteria Vicinamibacteria bacterium isolate SMAG U562 HET8646458.1
Pseudomonadati Acidobacteriota Vicinamibacteria Vicinamibacteria bacterium isolate SMAG U2702 HET9317201.1
Pseudomonadati Acidobacteriota Vicinamibacteria Vicinamibacteria bacterium isolate SMAG U4492 HEV8253690.1
Pseudomonadati Acidobacteriota Vicinamibacteria Vicinamibacteria bacterium isolate SMAG U1111 HET7294888.1
Pseudomonadati Acidobacteriota Vicinamibacteria Vicinamibacteria bacterium isolate SMAG U2879 HEX9725631.1
Pseudomonadati Acidobacteriota unclassified Acidobacteriota Acidobacteriota bacterium isolate N075bin57 TDI39376.1
Pseudomonadati Acidobacteriota Vicinamibacteria Vicinamibacteria bacterium isolate SMAG U4005 HEY7819149.1
Pseudomonadati Acidobacteriota Vicinamibacteria Vicinamibacteria bacterium isolate SMAG U16455 HXV65516.1
Pseudomonadati Acidobacteriota unclassified Acidobacteriota Acidobacteriota bacterium isolate Bin01 094 MCS5702398.1
Pseudomonadati Acidobacteriota unclassified Acidobacteriota Acidobacteriota bacterium isolate EAC88 MAM85452.1
Pseudomonadati Acidobacteriota unclassified Acidobacteriota Acidobacteriota bacterium isolate NC groundwater 305 Ag B0.1um 68 13 MBI2219723.1
Pseudomonadati Acidobacteriota Vicinamibacteria Vicinamibacterales bacterium isolate SMAG U6800 HEX2446543.1
Pseudomonadati Acidobacteriota Vicinamibacteria Vicinamibacterales bacterium isolate SMAG U6769 HEX2343118.1
Pseudomonadati Acidobacteriota unclassified Acidobacteriota Acidobacteriota bacterium isolate NC groundwater 628 Ag B0.1um 65 34 MBI2834499.1
Pseudomonadati Acidobacteriota Vicinamibacteria Vicinamibacterales bacterium isolate SMAG U13259 HXH06174.1
Pseudomonadati Acidobacteriota Vicinamibacteria Vicinamibacterales bacterium isolate SMAG U3631 HEY0873498.1
Pseudomonadati Acidobacteriota Vicinamibacteria Vicinamibacterales bacterium isolate SMAG U2365 HEU4925985.1
Pseudomonadati Acidobacteriota unclassified Acidobacteriota Acidobacteriota bacterium isolate cluster4 bin.17 MEP6918684.1
Pseudomonadati Acidobacteriota unclassified Acidobacteriota Acidobacteriota bacterium isolate MDV bin21 MDQ3211290.1
Pseudomonadati Acidobacteriota Vicinamibacteria Vicinamibacterales bacterium isolate SMAG U2373 HEU4935741.1
Pseudomonadati Acidobacteriota Vicinamibacteria Vicinamibacterales bacterium isolate SMAG U16379 HSC26492.1
Pseudomonadati Acidobacteriota unclassified Acidobacteriota Acidobacteriota bacterium isolate gp6 AA37 PYR57573.1
Pseudomonadati Acidobacteriota Vicinamibacteria Vicinamibacterales bacterium isolate SMAG U1273 HET7698397.1
Pseudomonadati Acidobacteriota Vicinamibacteria Vicinamibacterales bacterium isolate SMAG U5291 HEY2432771.1
Pseudomonadati Acidobacteriota Vicinamibacteria Vicinamibacterales bacterium isolate SMAG U1831 HEX6465203.1
Pseudomonadati Acidobacteriota unclassified Acidobacteriota Acidobacteriota bacterium isolate SP3003 MBL02384.1
Pseudomonadati Acidobacteriota Vicinamibacteria Vicinamibacterales bacterium isolate SMAG U11280 HXT71214.1
Pseudomonadati Acidobacteriota Vicinamibacteria Vicinamibacterales bacterium isolate SMAG U1395 HEX5069896.1
Pseudomonadati Acidobacteriota Vicinamibacteria Vicinamibacterales bacterium isolate SMAG U3909 HEY7475697.1
Pseudomonadati Acidobacteriota Vicinamibacteria Vicinamibacterales bacterium isolate cluster1 bin.272 MEO6221990.1
Pseudomonadati Acidobacteriota Vicinamibacteria Vicinamibacterales bacterium isolate SMAG U8798 HKW02023.1
Pseudomonadati Acidobacteriota unclassified Acidobacteriota Acidobacteriota bacterium isolate Gw SlAct bin 138 MBP6715744.1
Pseudomonadati Acidobacteriota Vicinamibacteria Vicinamibacterales bacterium isolate SMAG U14614 HVQ42718.1
Pseudomonadati Acidobacteriota Vicinamibacteria Vicinamibacterales bacterium isolate SMAG U782 HET9368855.1
Pseudomonadati Acidobacteriota unclassified Acidobacteriota Acidobacteriota bacterium isolate SJ549 MCC7008756.1
Pseudomonadati Acidobacteriota unclassified Acidobacteriota Acidobacteriota bacterium isolate cluster3 bin.267 MEO8070656.1
Pseudomonadati Acidobacteriota Vicinamibacteria Vicinamibacterales bacterium isolate cluster1 bin.85 MEO7191153.1
Pseudomonadati Acidobacteriota Vicinamibacteria Vicinamibacterales bacterium isolate SMAG U14480 HUF23005.1
Pseudomonadati Acidobacteriota Vicinamibacteria Vicinamibacterales bacterium isolate STLACI19 MFN8059623.1
Pseudomonadati Acidobacteriota Vicinamibacteria Luteitalea pratensis WP 110173191.1
Pseudomonadati Acidobacteriota Vicinamibacteria Vicinamibacterales bacterium isolate SMAG U7126 HEY8549418.1
Pseudomonadati Acidobacteriota Vicinamibacteria Vicinamibacteraceae bacterium isolate cluster1 bin.329 MEO6008604.1
Pseudomonadati Acidobacteriota Vicinamibacteria Luteitalea sp. isolate Dino bin44 MPZ17131.1
Pseudomonadati Acidobacteriota Vicinamibacteria Vicinamibacterales bacterium isolate CA 2019 TPBF 190 MGE3843991.1
Pseudomonadati Acidobacteriota unclassified Acidobacteriota Acidobacteriota bacterium isolate ACI3 MCL4845608.1
Pseudomonadati Acidobacteriota Vicinamibacteria Vicinamibacteraceae bacterium isolate ACI2 MCL4811857.1
Pseudomonadati Acidobacteriota unclassified Acidobacteriota Acidobacteriota bacterium isolate new MAG255 MBL8143955.1
Pseudomonadati Acidobacteriota Vicinamibacteria Vicinamibacterales bacterium isolate Prado004 MCU0255321.1
Pseudomonadati Acidobacteriota unclassified Acidobacteriota Acidobacteriota bacterium isolate MAG266 MEW5984389.1
Pseudomonadati Acidobacteriota Vicinamibacteria Vicinamibacterales bacterium isolate E6 metabat.bin.79 MDO8837112.1
Pseudomonadati Acidobacteriota Vicinamibacteria Vicinamibacterales bacterium isolate Prado007 MCU0251678.1
Pseudomonadati Acidobacteriota unclassified Acidobacteriota Acidobacteriota bacterium isolate LacPavin 0419 WC70S MAG 67 7 MCX6552861.1
Pseudomonadati Acidobacteriota unclassified Acidobacteriota Acidobacteriota bacteriumisolate H43bin207 1 MEK6631549.1
Pseudomonadati Acidobacteriota unclassified Acidobacteriota Acidobacteriota bacterium isolate NC groundwater 58 Pr7 S0.65um 66 18 MBI1873516.1
Pseudomonadati Acidobacteriota Vicinamibacteria Vicinamibacterales bacterium isolate SBS.BIN.101 MFB3854986.1
Pseudomonadati Acidobacteriota unclassified Acidobacteriota Acidobacteriota bacterium isolate Bin7 14.1m MBE3132804.1
Pseudomonadati Acidobacteriota Vicinamibacteria Vicinamibacterales bacterium isolate SMAG U2999 HEX7486846.1
Pseudomonadati Acidobacteriota unclassified Acidobacteriota Acidobacteriota bacterium isolate maxbin2.0082 RPJ70670.1
Pseudomonadati Acidobacteriota Vicinamibacteria Vicinamibacterales bacterium isolate AMR MDS 1511 HOC17514.1
Pseudomonadati Acidobacteriota unclassified Acidobacteriota Acidobacteriota bacterium isolate metabat2.166 RPI57912.1
Pseudomonadati Acidobacteriota Vicinamibacteria Vicinamibacterales bacterium isolate SMAG U6578 HEV3217848.1
Pseudomonadati Acidobacteriota Vicinamibacteria Vicinamibacterales bacterium isolate SMAG U6756 HEX2311643.1
Pseudomonadati Acidobacteriota Vicinamibacteria Vicinamibacterales bacterium isolate SMAG U10955 HZA36869.1
Pseudomonadati Acidobacteriota Vicinamibacteria Vicinamibacteria bacterium isolate 3 4 m bin5 MBK5296802.1
Pseudomonadati Acidobacteriota Vicinamibacteria Vicinamibacterales bacterium isolate SMAG U2030 HEX7088445.1
Pseudomonadati Acidobacteriota unclassified Acidobacteriota Acidobacteriota bacterium isolate REEB315 MDE3153732.1
Pseudomonadati Acidobacteriota Vicinamibacteria Vicinamibacterales bacterium isolate SMAG U4039 HEY7791695.1
Pseudomonadati Acidobacteriota unclassified Acidobacteriota Acidobacteriota bacterium isolate MAG Dt 03 MES1256522.1
Pseudomonadati Acidobacteriota Vicinamibacteria Vicinamibacterales bacterium isolate SMAG U3911 HEY7445979.1
Pseudomonadati Acidobacteriota Vicinamibacteria Vicinamibacterales bacterium isolate SMAG U2059 HEX7139851.1
Pseudomonadati Acidobacteriota Vicinamibacteria Vicinamibacterales bacterium isolate SMAG U12952 HWW86816.1
Pseudomonadati Acidobacteriota Vicinamibacteria Vicinamibacterales bacterium isolate SMAG U15500 HTK28263.1
Pseudomonadati Acidobacteriota Vicinamibacteria Vicinamibacterales bacterium isolate SMAG U6558 HEV3140665.1
Pseudomonadati Acidobacteriota Terriglobia unclassified Terriglobia Terriglobia bacterium isolate SRVP 36 MBZ5559553.1
Pseudomonadati Acidobacteriota Vicinamibacteria Vicinamibacterales bacterium isolate SMAG U4635 HEY3045000.1
Pseudomonadati Acidobacteriota unclassified Acidobacteriota Acidobacteriota bacterium isolate MAG131 MEW6367992.1
Pseudomonadati Acidobacteriota unclassified Acidobacteriota Acidobacteriota bacterium isolate NC groundwater 627 Ag B0.1um 62 56 MBI2840080.1
Aminicenantes Candidatus Aminicenantota Candidatus Aminicenantia bacterium isolate b3.sp2 bin.19 MFQ6083604.1
Aminicenantes Candidatus Aminicenantota Candidatus Aminicenantes bacterium ADurb.Bin508 OPZ63162.1
Aminicenantes Candidatus Aminicenantota Candidatus Aminicenantes bacterium isolate Zod Metabat.875 MBN2399288.1
Aminicenantes Candidatus Aminicenantota Candidatus Aminicenantes bacterium isolate LacPavin 0920 SED2 MAG 57 10 MCX6556187.1
Aminicenantes Candidatus Aminicenantota Candidatus Aminicenantes bacterium isolate SpSt 1172 HDP95324.1
Aminicenantes Candidatus Aminicenantota Candidatus Aminicenantes bacterium isolate RS 6 39 MCK5221231.1
Aminicenantes Candidatus Aminicenantota Candidatus Aminicenantes bacterium isolate KS4 C131 NIM12705.1
Aminicenantes Candidatus Aminicenantota Candidatus Aminicenantes bacterium isolate RS 9 67 MCK4761909.1
Aminicenantes Candidatus Aminicenantota Candidatus Aminicenantes bacterium isolate RS 8 31 MCK5057831.1
Aminicenantes Candidatus Aminicenantota Candidatus Aminicenantes bacterium isolate PMV 4 maxbin.003 MCI0471160.1
Aminicenantes Candidatus Aminicenantota Candidatus Aminicenantes bacterium isolate Zod maxbin.0146 MBN2245002.1
Aminicenantes Candidatus Aminicenantota Candidatus Aminicenantes bacterium isolate BLK5 2.bin.174 MFO7866395.1
Aminicenantes Candidatus Aminicenantota Candidatus Aminicenantaceae bacterium isolate 11D 13 MFA9454285.1
Aminicenantes Candidatus Aminicenantota Candidatus Aminicenantes bacterium isolate Zod Metabat.553 MBN1223388.1
Aminicenantes Candidatus Aminicenantota Candidatus Aminicenantaceae bacterium isolate SCS5bin.7 MEE9501113.1
Aminicenantes Candidatus Aminicenantota Candidatus Aminicenantes bacterium isolate D1103U20metaG Bin 558 Cleaned 1 MGD2294247.1
Aminicenantes Candidatus Aminicenantota Candidatus Aminicenantes bacterium isolate PFL48 MCJ7580709.1
Aminicenantes Candidatus Aminicenantota Candidatus Aminicenantaceae bacterium isolate BS2bin.13 MEE8377754.1
Aminicenantes Candidatus Aminicenantota Candidatus Aminicenantes bacterium isolate 4484 214 OQX54187.1
Aminicenantes Candidatus Aminicenantota Candidatus Aminicenantes bacterium isolate RS 18 43 MCK4264551.1
Aminicenantes Candidatus Aminicenantota Candidatus Aminicenantes bacterium isolate Kmv29 MBE0461036.1
Aminicenantes Candidatus Aminicenantota Candidatus Aminicenantes bacterium isolate 4 1 July SF Bin33 MDH4217600.1
Aminicenantes Candidatus Aminicenantota Candidatus Aminicenantaceae bacterium isolate KS4 C131 MEE8605034.1
Aminicenantes Candidatus Aminicenantota Candidatus Aminicenantes bacterium isolate D4M11 12 MFP4081337.1
Aminicenantes Candidatus Aminicenantota Candidatus Aminicenantales bacterium isolate b2.sp1 bin.57 MFQ6108937.1
Aminicenantes Candidatus Aminicenantota Candidatus Aminicenantes bacterium isolate bin112 UCC41871.1
Aminicenantes Candidatus Aminicenantota Candidatus Aminicenantaceae bacterium isolate SCS4bin.37 MEE9390102.1
Aminicenantes Candidatus Aminicenantota Candidatus Aminicenantes bacterium isolate RS 17 38 MCK4363734.1
Aminicenantes Candidatus Aminicenantota Candidatus Aminicenantes bacterium isolate RS 12 32 MCK4645739.1
Aminicenantes Candidatus Aminicenantota Candidatus Aminicenantes bacterium isolate NIOZ UU59 MBC8358124.1
Aminicenantes Candidatus Aminicenantota Candidatus Aminicenantes bacterium isolate RS 13 23 MCK4556510.1
Aminicenantes Candidatus Aminicenantota Candidatus Aminicenantes bacterium isolate 4 1 July SF Bin54 MDH4257226.1
Aminicenantes Candidatus Aminicenantota Candidatus Aminicenantales bacterium isolate b3.sp3 bin.11 MFQ6069549.1
Aminicenantes Candidatus Aminicenantota Candidatus Aminicenantaceae bacterium isolate S 218 B4 MFB0566462.1
Aminicenantes Candidatus Aminicenantota Candidatus Aminicenantes bacterium isolate Zod Metabat.480 MBN1273696.1
Aminicenantes Candidatus Aminicenantota Candidatus Aminicenantaceae bacterium isolate D4M11 37 MFW6132118.1
Aminicenantes Candidatus Aminicenantota Candidatus Aminicenantales bacterium isolate MFD01647 ilm asm bin15 MGB8951903.1

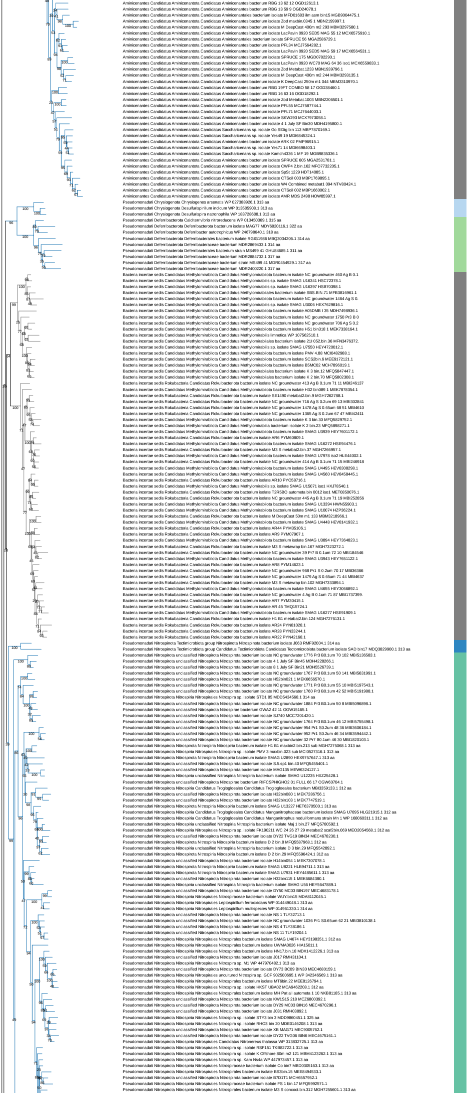
Aminicenantes Candidatus Aminicenantota Candidatus Aminicenantes bacterium RBG 13 62 12 OGD12613.1
Aminicenantes Candidatus Aminicenantota Candidatus Aminicenantes bacterium RBG 13 59 9 OGD24078.1
Aminicenantes Candidatus Aminicenantota Candidatus Aminicenantales bacterium isolate MFD01683 ilm asm bin15 MGB9004475.1
Aminicenantes Candidatus Aminicenantota Candidatus Aminicenantes bacterium isolate Zod maxbin.0345 1 MBN2199997.1
Aminicenantes Candidatus Aminicenantota Candidatus Aminicenantes bacterium isolate M DeepCast 400m m2 293 MBM3297580.1
Aminicenantes Candidatus Aminicenantota Candidatus Aminicenantes bacterium isolate LacPavin 0920 SED5 MAG 55 12 MCX6575910.1
Aminicenantes Candidatus Aminicenantota Candidatus Aminicenantales bacterium isolate SPRUCE 56 MGA2586729.1
Aminicenantes Candidatus Aminicenantota Candidatus Aminicenantes bacterium isolate PFL34 MCJ7564282.1
Aminicenantes Candidatus Aminicenantota Candidatus Aminicenantes bacterium isolate LacPavin 0920 SED5 MAG 59 17 MCX6564531.1
Aminicenantes Candidatus Aminicenantota Candidatus Aminicenantes bacterium isolate SPRUCE 175 MGD0782290.1
Aminicenantes Candidatus Aminicenantota Candidatus Aminicenantes bacterium isolate LacPavin 0920 WC70 MAG 64 36 iso1 MCX6559833.1
Aminicenantes Candidatus Aminicenantota Candidatus Aminicenantes bacterium isolate Zod Metabat.1233 MBN1939796.1
Aminicenantes Candidatus Aminicenantota Candidatus Aminicenantes bacterium isolate M DeepCast 400m m2 244 MBM3293135.1
Aminicenantes Candidatus Aminicenantota Candidatus Aminicenantes bacterium isolate K DeepCast 250m m1 044 MBM3310970.1
Aminicenantes Candidatus Aminicenantota Candidatus Aminicenantes bacterium RBG 19FT COMBO 58 17 OGD38460.1
Aminicenantes Candidatus Aminicenantota Candidatus Aminicenantes bacterium RBG 16 63 16 OGD18292.1
Aminicenantes Candidatus Aminicenantota Candidatus Aminicenantes bacterium isolate Zod Metabat.1003 MBN2206501.1
Aminicenantes Candidatus Aminicenantota Candidatus Aminicenantes bacterium isolate PFL55 MCJ7587744.1
Aminicenantes Candidatus Aminicenantota Candidatus Aminicenantes bacterium isolate PFL71 MCJ7644003.1
Aminicenantes Candidatus Aminicenantota Candidatus Aminicenantes bacterium isolate SKW293 MCX7973058.1
Aminicenantes Candidatus Aminicenantota Candidatus Aminicenantes bacterium isolate 4 1 July SF Bin30 MDH4195800.1
Aminicenantes Candidatus Aminicenantota Candidatus Saccharicenans sp. isolate Go SIDig bin 113 MBP7870169.1
Aminicenantes Candidatus Aminicenantota Candidatus Saccharicenans sp. isolate Yes49 19 MDI6845324.1
Aminicenantes Candidatus Aminicenantota Candidatus Aminicenantes bacterium isolate ARK 02 PMP96915.1
Aminicenantes Candidatus Aminicenantota Candidatus Saccharicenans sp. isolate Yes71 14 MDI6698403.1
Aminicenantes Candidatus Aminicenantota Candidatus Saccharicenans sp. isolate Kamch4336 1 MF 19 MGB9835336.1
Aminicenantes Candidatus Aminicenantota Candidatus Aminicenantes bacterium isolate SPRUCE 605 MGA2531781.1
Aminicenantes Candidatus Aminicenantota Candidatus Aminicenantes bacterium isolate CWP4 2.bin.162 MFO7732205.1
Aminicenantes Candidatus Aminicenantota Candidatus Aminicenantes bacterium isolate SpSt 1229 HDT14085.1
Aminicenantes Candidatus Aminicenantota Candidatus Aminicenantes bacterium isolate CTSoil 003 MBP1769895.1
Aminicenantes Candidatus Aminicenantota Candidatus Aminicenantes bacterium isolate W4 Combined metabat1 094 NTV80424.1
Aminicenantes Candidatus Aminicenantota Candidatus Aminicenantes bacterium isolate CTSoil 002 MBP1660002.1
Aminicenantes Candidatus Aminicenantota Candidatus Aminicenantes bacterium isolate AMR MDS 2498 HOW85997.1
Pseudomonadati Chrysiogenota Chrysiogenes arsenatis WP 027388926.1 313 aa
Pseudomonadati Chrysiogenota Desulfurispirillum indicum WP 013505908.1 313 aa
Pseudomonadati Chrysiogenota Desulfurispira natronophila WP 183728608.1 313 aa
Pseudomonadati Deferribacterota Calditerrivibrio nitroreducens WP 013450369.1 315 aa
Pseudomonadati Deferribacterota Deferribacterota bacterium isolate MAG77 MDY6820116.1 322 aa
Pseudomonadati Deferribacterota Deferribacter autotrophicus WP 246798640.1 318 aa
Pseudomonadati Deferribacterota Deferribacterales bacterium isolate RGIG1986 MBQ3034206.1 314 aa
Pseudomonadati Deferribacterota Deferribacteraceae bacterium MDR2869433.1 314 aa
Pseudomonadati Deferribacterota Deferribacterales bacterium strain MS499 41 GHU84685.1 311 aa
Pseudomonadati Deferribacterota Deferribacteraceae bacterium MDR2884732.1 317 aa
Pseudomonadati Deferribacterota Deferribacteraceae bacterium strain MS499 41 MDR0454929.1 317 aa
Pseudomonadati Deferribacterota Deferribacteraceae bacterium MDR2400220.1 317 aa
Bacteria incertae sedis Candidatus Methylomirabilota Candidatus Methylomirabilota bacterium isolate NC groundwater 460 Ag B 0.1
Bacteria incertae sedis Candidatus Methylomirabilota Candidatus Methylomirabilis sp. isolate SMAG U16341 HSC72378.1
Bacteria incertae sedis Candidatus Methylomirabilota Candidatus Methylomirabilis sp. isolate SMAG U16397 HSB70398.1
Bacteria incertae sedis Candidatus Methylomirabilota Candidatus Methylomirabilales bacterium isolate SBS.BIN.71 MFB3816961.1
Bacteria incertae sedis Candidatus Methylomirabilota Candidatus Methylomirabilota bacterium isolate NC groundwater 1464 Ag S 0.
Bacteria incertae sedis Candidatus Methylomirabilota Candidatus Methylomirabilis sp. isolate SMAG U3006 HEX7629816.1
Bacteria incertae sedis Candidatus Methylomirabilota Candidatus Methylomirabilota bacterium isolate A05DMB I 35 MDH7498936.1
Bacteria incertae sedis Candidatus Methylomirabilota Candidatus Methylomirabilota bacterium isolate NC groundwater 1750 Pr3 B 0
Bacteria incertae sedis Candidatus Methylomirabilota Candidatus Methylomirabilota bacterium isolate NC groundwater 706 Ag S 0.2
Bacteria incertae sedis Candidatus Methylomirabilota Candidatus Methylomirabilota bacterium isolate H51 bin318 1 MEK7338164.1
Bacteria incertae sedis Candidatus Methylomirabilota Candidatus Methylomirabilis limnetica WP 107562510.1
Bacteria incertae sedis Candidatus Methylomirabilota Candidatus Methylomirabilales bacterium isolate 21I 052.bin.36 MFN3476372.
Bacteria incertae sedis Candidatus Methylomirabilota Candidatus Methylomirabilis sp. isolate SMAG U7550 HEY4720012.1
Bacteria incertae sedis Candidatus Methylomirabilota Candidatus Methylomirabilota bacterium isolate PMV 4.88 MCI0482988.1
Bacteria incertae sedis Candidatus Methylomirabilota Candidatus Methylomirabilota bacterium isolate SCS2bin.6 MEE9172121.1
Bacteria incertae sedis Candidatus Methylomirabilota Candidatus Methylomirabilota bacterium isolate B5MC02 MCH7896019.1
Bacteria incertae sedis Candidatus Methylomirabilota Candidatus Methylomirabilales bacterium isolate K 3 bin.12 MFQ5847447.1
Bacteria incertae sedis Candidatus Methylomirabilota Candidatus Methylomirabilales bacterium isolate K 2 bin.70 MFQ5802308.1
Bacteria incertae sedis Rokubacteria Candidatus Rokuibacteriota bacterium isolate NC groundwater 413 Ag B 0.1um 71 11 MBI246137
Bacteria incertae sedis Candidatus Methylomirabilota Candidatus Methylomirabilota bacterium isolate H32 bin089 1 MEK7878354.1
Bacteria incertae sedis Rokubacteria Candidatus Rokuibacteriota bacterium isolate SE1490 metabat2.bin.9 MGH7262788.1
Bacteria incertae sedis Rokubacteria Candidatus Rokuibacteriota bacterium isolate NC groundwater 716 Ag S 0.2um 69 13 MBI302841
Bacteria incertae sedis Rokubacteria Candidatus Rokuibacteriota bacterium isolate NC groundwater 1478 Ag S 0.65um 68 51 MBI4610
Bacteria incertae sedis Rokubacteria Candidatus Rokuibacteriota bacterium isolate NC groundwater 1365 Ag S 0.2um 67 47 MBI42411
Bacteria incertae sedis Candidatus Methylomirabilota Candidatus Methylomirabilota bacterium isolate K 3 bin.30 MFQ5829752.1
Bacteria incertae sedis Candidatus Methylomirabilota Candidatus Methylomirabilia bacterium isolate K 2 bin.23 MFQ5898271.1
Bacteria incertae sedis Candidatus Methylomirabilota Candidatus Methylomirabilota bacterium isolate SMAG U3939 HEY7601172.1
Bacteria incertae sedis Rokubacteria Candidatus Rokuibacteriota bacterium isolate AR6 PYM60809.1
Bacteria incertae sedis Candidatus Methylomirabilota Candidatus Methylomirabilota bacterium isolate SMAG U16272 HSE94476.1
Bacteria incertae sedis Rokubacteria Candidatus Rokuibacteriota bacterium isolate M3 S metabat2.bin.37 MGH7266957.1
Bacteria incertae sedis Candidatus Methylomirabilota Candidatus Methylomirabilota bacterium isolate SMAG U7978 iso2 HLE44002.1
Bacteria incertae sedis Rokubacteria Candidatus Rokuibacteriota bacterium isolate NC groundwater 414 Ag B 0.1um 71 15 MBI246918
Bacteria incertae sedis Candidatus Methylomirabilota Candidatus Methylomirabilota bacterium isolate SMAG U4495 HEV8308298.1
Bacteria incertae sedis Candidatus Methylomirabilota Candidatus Methylomirabilota bacterium isolate SMAG U4560 HEV8458445.1
Bacteria incertae sedis Rokubacteria Candidatus Rokuibacteriota bacterium isolate AR10 PYO58716.1
Bacteria incertae sedis Candidatus Methylomirabilota Candidatus Methylomirabilis sp. isolate SMAG U15071 iso1 HXJ78540.1
Bacteria incertae sedis Rokubacteria Candidatus Rokuibacteriota bacterium isolate T2R5BO autometa bin 0012 iso1 MET0850076.1
Bacteria incertae sedis Rokubacteria Candidatus Rokuibacteriota bacterium isolate NC groundwater 445 Ag B 0.1um 71 19 MBI252856
Bacteria incertae sedis Candidatus Methylomirabilota Candidatus Methylomirabilota bacterium isolate SMAG U13394 HWN55903.1
Bacteria incertae sedis Candidatus Methylomirabilota Candidatus Methylomirabilota bacterium isolate SMAG U10074 HZP36224.1
Bacteria incertae sedis Rokubacteria Candidatus Rokuibacteriota bacterium isolate M DeepCast 50m m1 133 MBM3218966.1
Bacteria incertae sedis Candidatus Methylomirabilota Candidatus Methylomirabilota bacterium isolate SMAG U4448 HEV8141932.1
Bacteria incertae sedis Rokubacteria Candidatus Rokuibacteriota bacterium isolate AR44 PYM35106.1
Bacteria incertae sedis Rokubacteria Candidatus Rokuibacteriota bacterium isolate AR9 PYM07907.1
Bacteria incertae sedis Candidatus Methylomirabilota Candidatus Methylomirabilota bacterium isolate SMAG U3894 HEY7364823.1
Bacteria incertae sedis Rokubacteria Candidatus Rokuibacteriota bacterium isolate M3 S metawrap bin.167 MGH7323272.1
Bacteria incertae sedis Rokubacteria Candidatus Rokuibacteriota bacterium isolate NC groundwater 39 Pr7 B 0.1um 72 10 MBI184546
Bacteria incertae sedis Candidatus Methylomirabilota Candidatus Methylomirabilota bacterium isolate SMAG U3943 HEY7651122.1
Bacteria incertae sedis Rokubacteria Candidatus Rokuibacteriota bacterium isolate AR8 PYM14623.1
Bacteria incertae sedis Rokubacteria Candidatus Rokuibacteriota bacterium isolate NC groundwater 968 Pr1 S 0.2um 70 17 MBI36366
Bacteria incertae sedis Rokubacteria Candidatus Rokuibacteriota bacterium isolate NC groundwater 1479 Ag S 0.65um 71 44 MBI4637
Bacteria incertae sedis Rokubacteria Candidatus Rokuibacteriota bacterium isolate M3 S metawrap bin.102 MGH7333894.1
Bacteria incertae sedis Candidatus Methylomirabilota Candidatus Methylomirabilota bacterium isolate SMAG U4655 HEY3066892.1
Bacteria incertae sedis Rokubacteria Candidatus Rokuibacteriota bacterium isolate NC groundwater 4 Ag B 0.1um 71 87 MBI1737399.
Bacteria incertae sedis Rokubacteria Candidatus Rokuibacteriota bacterium isolate AR7 PYM30415.1
Bacteria incertae sedis Rokubacteria Candidatus Rokuibacteriota bacterium isolate AR 45 TMQ15724.1
Bacteria incertae sedis Candidatus Methylomirabilota Candidatus Methylomirabilota bacterium isolate SMAG U16277 HSE91909.1
Bacteria incertae sedis Rokubacteria Candidatus Rokuibacteriota bacterium isolate H1 B1 metabat2.bin.124 MGH7276131.1
Bacteria incertae sedis Rokubacteria Candidatus Rokuibacteriota bacterium isolate AR24 PYN81028.1
Bacteria incertae sedis Rokubacteria Candidatus Rokuibacteriota bacterium isolate AR28 PYN33244.1
Bacteria incertae sedis Rokubacteria Candidatus Rokuibacteriota bacterium isolate AR22 PYN42168.1
Pseudomonadati Nitrospinota Tectimicrobiota group Nitrospinota Nitrospinota bacterium isolate J063 RMF92004.1 314 aa
Pseudomonadati Nitrospinota Tectimicrobiota group Candidatus Tectimicrobiota Candidatus Tectomicrobiota bacterium isolate SAD bin17 MDQ3829900.1 313 aa
Pseudomonadati Nitrospirota unclassified Nitrospirota Nitrospirota bacterium isolate NC groundwater 1776 Pr3 B0.1um 70 102 MBI5136583.1
Pseudomonadati Nitrospirota unclassified Nitrospirota Nitrospirota bacterium isolate 4 1 July SF Bin45 MDH4228266.1
Pseudomonadati Nitrospirota unclassified Nitrospirota Nitrospirota bacterium isolate 8 1 July SF Bin21 MDH5526739.1
Pseudomonadati Nitrospirota unclassified Nitrospirota Nitrospirota bacterium isolate NC groundwater 1767 Pr3 B0.1um 50 141 MBI5631991.1
Pseudomonadati Nitrospirota unclassified Nitrospirota Nitrospirota bacterium isolate H52bin021 1 MEK6656570.1
Pseudomonadati Nitrospirota unclassified Nitrospirota Nitrospirota bacterium isolate NC groundwater 1771 Pr3 B0.1um 55 10 MBI5197543.1
Pseudomonadati Nitrospirota unclassified Nitrospirota Nitrospirota bacterium isolate NC groundwater 1760 Pr3 B0.1um 42 52 MBI5191988.1
Pseudomonadati Nitrospirota Nitrospiria Nitrospirales Nitrospira sp. isolate STD1 85 MDD5434568.1 314 aa
Pseudomonadati Nitrospirota unclassified Nitrospirota Nitrospirota bacterium isolate NC groundwater 1884 Pr3 B0.1um 50 8 MBI5096898.1
Pseudomonadati Nitrospirota unclassified Nitrospirota Nitrospirae bacterium GWA2 42 11 OGW15165.1
Pseudomonadati Nitrospirota unclassified Nitrospirota Nitrospirota bacterium isolate SJ740 MCC7201420.1
Pseudomonadati Nitrospirota unclassified Nitrospirota Nitrospirota bacterium isolate NC groundwater 1764 Pr3 B0.1um 46 12 MBI5755498.1
Pseudomonadati Nitrospirota unclassified Nitrospirota Nitrospirota bacterium isolate NC groundwater 954 Pr1 S0.2um 48 36 MBI3606184.1
Pseudomonadati Nitrospirota unclassified Nitrospirota Nitrospirota bacterium isolate NC groundwater 952 Pr1 S0.2um 46 34 MBI3594442.1
Pseudomonadati Nitrospirota unclassified Nitrospirota Nitrospirota bacterium isolate NC groundwater 32 Pr7 B0.1um 46 30 MBI1820103.1
Pseudomonadati Nitrospirota Nitrospirota Nitrospiria Nitrospiria bacterium isolate H1 B1 maxbin2.bin.213 sub MGH7275068.1 313 aa
Pseudomonadati Nitrospirota Nitrospiria Nitrospirales Nitrospira sp. isolate PMV 3 maxbin.023 sub MCI0527316.1 313 aa
Pseudomonadati Nitrospirota Nitrospirota Nitrospiria Nitrospiria bacterium isolate SMAG U2890 HEX9757647.1 313 aa
Pseudomonadati Nitrospirota unclassified Nitrospirota Nitrospirota bacterium isolate S.5.sp1 bin.40 MFQ5455401.1
Pseudomonadati Nitrospirota unclassified Nitrospirota Nitrospirota bacterium isolate MAG135 MEW6324127.1
Pseudomonadati Nitrospirota Nitrospiria unclassified Nitrospiria Nitrospiria bacterium isolate SMAG U12235 HXZ25428.1
Pseudomonadati Nitrospirota unclassified Nitrospirota Nitrospirae bacterium RIFCSPHIGHO2 01 FULL 66 17 OGW60704.1
Pseudomonadati Nitrospirota Nitrospiria Candidatus Troglogloeales Candidatus Troglogloeales bacterium MBI3359133.1 312 aa
Pseudomonadati Nitrospirota unclassified Nitrospirota Nitrospirota bacterium isolate H32bin080 1 MEK7286756.1
Pseudomonadati Nitrospirota unclassified Nitrospirota Nitrospirota bacterium isolate H32bin103 1 MEK7747519.1
Pseudomonadati Nitrospirota Nitrospirota Nitrospiria Nitrospiria bacterium isolate SMAG U13227 HET6370500.1 313 aa
Pseudomonadati Nitrospirota Nitrospiria Candidatus Troglogloeales Candidatus Manganitrophaceae bacterium isolate SMAG U7895 HLG21915.1 312 aa
Pseudomonadati Nitrospirota Nitrospiria Candidatus Troglogloeales Candidatus Manganitrophus noduliformans strain Mn 1 WP 168060311.1 312 aa
Pseudomonadati Nitrospirota Nitrospiria unclassified Nitrospiria Nitrospiria bacterium isolate Maj 1 bin.27 MFQ5780592.1
Pseudomonadati Nitrospirota Nitrospiria Nitrospirales Nitrospira sp. isolate FK190211 WC 24 26 27 29 metabat2 scaf2bin.069 MEO2054568.1 312 aa
Pseudomonadati Nitrospirota unclassified Nitrospirota Nitrospirota bacterium isolate DY22 TVG19 BIN34 MEC4678230.1
Pseudomonadati Nitrospirota Nitrospirota Nitrospiria Nitrospiria bacterium isolate D 2 bin.8 MFQ5587968.1 312 aa
Pseudomonadati Nitrospirota Nitrospiria unclassified Nitrospiria Nitrospiria bacterium isolate D 3 bin.29 MFQ5542892.1
Pseudomonadati Nitrospirota Nitrospirota Nitrospiria Nitrospiria bacterium isolate D 2 bin.29 MFQ5596424.1 312 aa
Pseudomonadati Nitrospirota unclassified Nitrospirota Nitrospirota bacterium isolate H14bin054 1 MEK7307078.1
Pseudomonadati Nitrospirota Nitrospirota Nitrospiria Nitrospiria bacterium isolate SMAG U8221 HLB94711.1 313 aa
Pseudomonadati Nitrospirota Nitrospirota Nitrospiria Nitrospiria bacterium isolate SMAG U7931 HEY4485611.1 313 aa
Pseudomonadati Nitrospirota unclassified Nitrospirota Nitrospirota bacterium isolate H32bin115 1 MEK6684380.1
Pseudomonadati Nitrospirota Nitrospiria unclassified Nitrospiria Nitrospiria bacterium isolate SMAG U56 HEY5647889.1
Pseudomonadati Nitrospirota unclassified Nitrospirota Nitrospirota bacterium isolate DY50 MC03 BIN197 MEC4683178.1
Pseudomonadati Nitrospirota Nitrospiria Nitrospirales Nitrospiraceae bacterium isolate WUY.bin15 MDA8112045.1
Pseudomonadati Nitrospirota Nitrospiria Nitrospirales Leptospirillum ferrooxidans WP 014449048.1 313 aa
Pseudomonadati Nitrospirota Nitrospiria Nitrospirales Leptospirillum multispecies WP 014961330.1 314 aa
Pseudomonadati Nitrospirota unclassified Nitrospirota Nitrospirota bacterium isolate NS 1 TLY32713.1
Pseudomonadati Nitrospirota unclassified Nitrospirota Nitrospirota bacterium isolate NC groundwater 1036 Pr1 S0.65um 62 21 MBI3810138.1
Pseudomonadati Nitrospirota unclassified Nitrospirota Nitrospirota bacterium isolate NS 4 TLY38186.1
Pseudomonadati Nitrospirota unclassified Nitrospirota Nitrospirota bacterium isolate NS 11 TLY19204.1
Pseudomonadati Nitrospirota Nitrospiria Nitrospirales Nitrospirales bacterium isolate SMAG U4674 HEY3198351.1 312 aa
Pseudomonadati Nitrospirota Nitrospiria Nitrospirales Nitrospirales bacterium isolate UWMA0026 HIA15011.1
Pseudomonadati Nitrospirota Nitrospiria Nitrospirales Nitrospirales bacterium isolate HN17.bin.18 MDX1412226.1 313 aa
Pseudomonadati Nitrospirota unclassified Nitrospirota Nitrospirota bacterium isolate J017 RMH31104.1
Pseudomonadati Nitrospirota Nitrospiria Nitrospirales Nitrospira sp. M1 WP 447970482.1 313 aa
Pseudomonadati Nitrospirota unclassified Nitrospirota Nitrospirota bacterium isolate DY73 BC09 BIN30 MEC4680159.1
Pseudomonadati Nitrospirota Nitrospiria Nitrospirales uncultured Nitrospira sp. GCF 902500695.1 WP 342346569.1 313 aa
Pseudomonadati Nitrospirota Nitrospiria Nitrospirales Nitrospirales bacterium isolate MT6bin.22 MEE8126794.1
Pseudomonadati Nitrospirota Nitrospiria Nitrospirales Nitrospira sp. isolate HKST UBA02 MCA9462208.1 312 aa
Pseudomonadati Nitrospirota Nitrospiria Nitrospirales Nitrospirales bacterium isolate MH Pat all autometa 1 10 NKB81185.1 313 aa
Pseudomonadati Nitrospirota unclassified Nitrospirota Nitrospirota bacterium isolate KW1S15 218 MCZ6800392.1
Pseudomonadati Nitrospirota unclassified Nitrospirota Nitrospirota bacterium isolate DY29 MC03 BIN16 MEC4670296.1
Pseudomonadati Nitrospirota unclassified Nitrospirota Nitrospirota bacterium isolate J031 RMH03892.1
Pseudomonadati Nitrospirota Nitrospiria Nitrospirales Nitrospira sp. isolate STY3 bin 3 MDD9860451.1 325 aa
Pseudomonadati Nitrospirota Nitrospiria Nitrospirales Nitrospira sp. isolate RHO3 bin 20 MDE0146208.1 313 aa
Pseudomonadati Nitrospirota unclassified Nitrospirota Nitrospirota bacterium isolate XB MAG71 MEC9005762.1
Pseudomonadati Nitrospirota unclassified Nitrospirota Nitrospirota bacterium isolate DY22 TVG06 BIN6 MEC4675161.1
Pseudomonadati Nitrospirota Nitrospiria Nitrospirales Candidatus Nitronereus thalassa WP 313832725.1 313 aa
Pseudomonadati Nitrospirota Nitrospiria Nitrospirales Nitrospira sp. isolate RSF151 TKB82722.1 313 aa
Pseudomonadati Nitrospirota Nitrospiria Nitrospirales Nitrospira sp. isolate K Offshore 80m m2 121 MBM4123262.1 313 aa
Pseudomonadati Nitrospirota Nitrospiria Nitrospirales Nitrospira sp. Kam Ns4a WP 447973457.1 313 aa
Pseudomonadati Nitrospirota Nitrospiria Nitrospirales Nitrospiraceae bacterium isolate Co bin7 MBD0305163.1 313 aa
Pseudomonadati Nitrospirota Nitrospiria Nitrospirales Nitrospirales bacterium isolate BS3bin.15 MEE8494533.1
Pseudomonadati Nitrospirota unclassified Nitrospirota Nitrospirota bacterium isolate B7D1T1 MCH6557952.1
Pseudomonadati Nitrospirota Nitrospiria Nitrospirales Nitrospiraceae bacterium isolate FS 1 bin.17 MFQ5992571.1
Pseudomonadati Nitrospirota Nitrospiria Nitrospirales Nitrospirales bacterium isolate M3 S concoct.bin.312 MGH7255601.1 313 aa

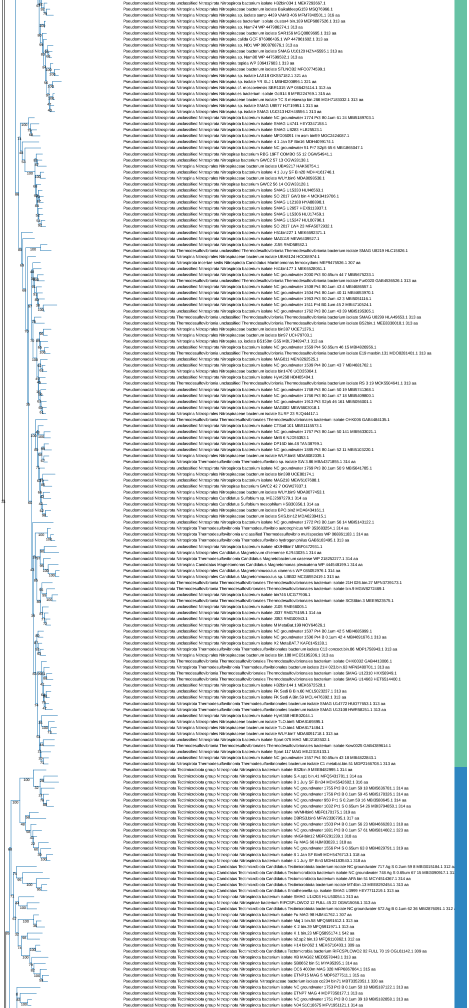

Pseudomonadati Nitrospirota unclassified Nitrospirota Nitrospirota bacterium isolate H32bin034 1 MEK7293667.1
Pseudomonadati Nitrospirota Nitrospiria Nitrospirales Nitrospiraceae bacterium isolate BaikaldeepG159 MSQ76966.1
Pseudomonadati Nitrospirota Nitrospiria Nitrospirales Nitrospira sp. isolate samp 4439 VAMB 406 MFM7840501.1 316 aa
Pseudomonadati Nitrospirota Nitrospiria Nitrospirales Nitrospirales bacterium isolate cluster4 bin.189 MEP6887526.1 313 aa
Pseudomonadati Nitrospirota Nitrospiria Nitrospirales Nitrospira sp. Nam74 WP 447986274.1 313 aa
Pseudomonadati Nitrospirota Nitrospiria Nitrospirales Nitrospiraceae bacterium isolate SAR156 MGQ0809695.1 313 aa
Pseudomonadati Nitrospirota Nitrospiria Nitrospirales Nitrospira calida GCF 976986435.1 WP 447861602.1 313 aa
Pseudomonadati Nitrospirota Nitrospiria Nitrospirales Nitrospira sp. ND1 WP 080878876.1 313 aa
Pseudomonadati Nitrospirota Nitrospiria Nitrospirales Nitrospiraceae bacterium isolate SMAG U10120 HZN45595.1 313 aa
Pseudomonadati Nitrospirota Nitrospiria Nitrospirales Nitrospira sp. Nam80 WP 447599582.1 313 aa
Pseudomonadati Nitrospirota Nitrospiria Nitrospirales Nitrospira tepida WP 306417603.1 313 aa
Pseudomonadati Nitrospirota Nitrospiria Nitrospirales Nitrospiraceae bacterium isolate STLNOB2 MFO0774599.1
Pseudomonadati Nitrospirota Nitrospiria Nitrospirales Nitrospira sp. isolate LAS18 GKS57182.1 321 aa
Pseudomonadati Nitrospirota Nitrospiria Nitrospirales Nitrospira sp. isolate YR XLJ 1 MBH0200896.1 321 aa
Pseudomonadati Nitrospirota Nitrospiria Nitrospirales Nitrospira cf. moscoviensis SBR1015 WP 086425114.1 313 aa
Pseudomonadati Nitrospirota Nitrospiria Nitrospirales Nitrospirales bacterium isolate GcB14 8 MFI5224769.1 315 aa
Pseudomonadati Nitrospirota Nitrospiria Nitrospirales Nitrospiraceae bacterium isolate TC S metawrap bin.266 MGH7183032.1 313 aa
Pseudomonadati Nitrospirota Nitrospiria Nitrospirales Nitrospira sp. isolate SMAG U8577 HJT19951.1 313 aa
Pseudomonadati Nitrospirota Nitrospiria Nitrospirales Nitrospira sp. isolate SMAG U10313 HZH48556.1 313 aa
Pseudomonadati Nitrospirota unclassified Nitrospirota Nitrospirota bacterium isolate NC groundwater 1774 Pr3 B0.1um 61 24 MBI5189703.1
Pseudomonadati Nitrospirota unclassified Nitrospirota Nitrospirota bacterium isolate SMAG U4741 HEY3347158.1
Pseudomonadati Nitrospirota unclassified Nitrospirota Nitrospirota bacterium isolate SMAG U8283 HLB25523.1
Pseudomonadati Nitrospirota unclassified Nitrospirota Nitrospirota bacterium isolate MFD06091 ilm asm bin59 MGC2424087.1
Pseudomonadati Nitrospirota unclassified Nitrospirota Nitrospirota bacterium isolate 4 1 Jan SF Bin16 MDH4099174.1
Pseudomonadati Nitrospirota unclassified Nitrospirota Nitrospirota bacterium isolate NC groundwater 51 Pr7 S2p5 65 6 MBI1865047.1
Pseudomonadati Nitrospirota unclassified Nitrospirota Nitrospirae bacterium RBG 19FT COMBO 55 12 OGW54941.1
Pseudomonadati Nitrospirota unclassified Nitrospirota Nitrospirae bacterium GWC2 57 13 OGW28138.1
Pseudomonadati Nitrospirota Nitrospiria Nitrospirales Nitrospiraceae bacterium isolate UBA9217 HAK60754.1
Pseudomonadati Nitrospirota unclassified Nitrospirota Nitrospirota bacterium isolate 4 1 July SF Bin20 MDH4161746.1
Pseudomonadati Nitrospirota Nitrospiria Nitrospirales Nitrospiraceae bacterium isolate WUY.bin6 MDA8098538.1
Pseudomonadati Nitrospirota unclassified Nitrospirota Nitrospirae bacterium GWC2 56 14 OGW33128.1
Pseudomonadati Nitrospirota unclassified Nitrospirota Nitrospirota bacterium isolate SMAG U15330 HUI46563.1
Pseudomonadati Nitrospirota unclassified Nitrospirota Nitrospirota bacterium isolate SO 2017 GW3 bin 4 MCK9419706.1
Pseudomonadati Nitrospirota unclassified Nitrospirota Nitrospirota bacterium isolate SMAG U12188 HYA88898.1
Pseudomonadati Nitrospirota unclassified Nitrospirota Nitrospirota bacterium isolate SMAG U2657 HEX9113937.1
Pseudomonadati Nitrospirota unclassified Nitrospirota Nitrospirota bacterium isolate SMAG U15306 HUJ17459.1
Pseudomonadati Nitrospirota unclassified Nitrospirota Nitrospirota bacterium isolate SMAG U15247 HUL00796.1
Pseudomonadati Nitrospirota unclassified Nitrospirota Nitrospirota bacterium isolate SO 2017 LW4 23 MFA5072932.1
Pseudomonadati Nitrospirota unclassified Nitrospirota Nitrospirota bacterium isolate H51bin227 1 MEK6692371.1
Pseudomonadati Nitrospirota unclassified Nitrospirota Nitrospirota bacterium isolate MAG119 MEW6409527.1
Pseudomonadati Nitrospirota unclassified Nitrospirota Nitrospirota bacterium isolate J155 RMD58582.1
Pseudomonadati Nitrospirota Thermodesulfovibrionia unclassified Thermodesulfovibrionia Thermodesulfovibrionia bacterium isolate SMAG U8219 HLC15826.1
Pseudomonadati Nitrospirota Nitrospiria Nitrospirales Nitrospiraceae bacterium isolate UBA8124 HCC68974.1
Pseudomonadati Nitrospirota Nitrospirota incertae sedis Nitrospirota Candidatus Mariimomonas ferrooxydans MEF9475536.1 307 aa
Pseudomonadati Nitrospirota unclassified Nitrospirota Nitrospirota bacterium isolate H41bin177 1 MEK6528051.1
Pseudomonadati Nitrospirota unclassified Nitrospirota Nitrospirota bacterium isolate NC groundwater 2000 Pr3 S0.65um 44 7 MBI5675233.1
Pseudomonadati Nitrospirota Thermodesulfovibrionia unclassified Thermodesulfovibrionia Thermodesulfovibrionia bacterium isolate Fur0020 GAB4536526.1 313 aa
Pseudomonadati Nitrospirota unclassified Nitrospirota Nitrospirota bacterium isolate NC groundwater 1508 Pr4 B0.1um 43 4 MBI4686557.1
Pseudomonadati Nitrospirota unclassified Nitrospirota Nitrospirota bacterium isolate NC groundwater 1504 Pr4 B0.1um 40 11 MBI4653970.1
Pseudomonadati Nitrospirota unclassified Nitrospirota Nitrospirota bacterium isolate NC groundwater 1963 Pr3 S0.2um 42 3 MBI5051116.1
Pseudomonadati Nitrospirota unclassified Nitrospirota Nitrospirota bacterium isolate NC groundwater 1511 Pr4 B0.1um 45 2 MBI4710524.1
Pseudomonadati Nitrospirota unclassified Nitrospirota Nitrospirota bacterium isolate NC groundwater 1762 Pr3 B0.1um 43 39 MBI5195305.1
Pseudomonadati Nitrospirota Thermodesulfovibrionia unclassified Thermodesulfovibrionia Thermodesulfovibrionia bacterium isolate SMAG U8299 HLA49653.1 313 aa
Pseudomonadati Nitrospirota Thermodesulfovibrionia unclassified Thermodesulfovibrionia Thermodesulfovibrionia bacterium isolate BS2bin.1 MEE8330018.1 313 aa
Pseudomonadati Nitrospirota Nitrospiria Nitrospirales Nitrospiraceae bacterium isolate bin387 UCE71376.1
Pseudomonadati Nitrospirota Nitrospiria Nitrospirales Nitrospiraceae bacterium isolate bin97 UCH79703.1
Pseudomonadati Nitrospirota Nitrospiria Nitrospirales Nitrospira sp. isolate BS150m G55 MBL7048947.1 313 aa
Pseudomonadati Nitrospirota unclassified Nitrospirota Nitrospirota bacterium isolate NC groundwater 1559 Pr4 S0.65um 46 15 MBI4826956.1
Pseudomonadati Nitrospirota Thermodesulfovibrionia unclassified Thermodesulfovibrionia Thermodesulfovibrionia bacterium isolate E19 maxbin.131 MDO8281401.1 313 aa
Pseudomonadati Nitrospirota unclassified Nitrospirota Nitrospirota bacterium isolate MAG011 MEN8262525.1
Pseudomonadati Nitrospirota unclassified Nitrospirota Nitrospirota bacterium isolate NC groundwater 1509 Pr4 B0.1um 43 7 MBI4681762.1
Pseudomonadati Nitrospirota Nitrospiria Nitrospirales Nitrospiraceae bacterium isolate bin1476 UCD35004.1
Pseudomonadati Nitrospirota unclassified Nitrospirota Nitrospirota bacterium isolate HyVt268 HDH05404.1
Pseudomonadati Nitrospirota Thermodesulfovibrionia unclassified Thermodesulfovibrionia Thermodesulfovibrionia bacterium isolate RS 3 19 MCK5504641.1 313 aa
Pseudomonadati Nitrospirota unclassified Nitrospirota Nitrospirota bacterium isolate NC groundwater 1768 Pr3 B0.1um 50 19 MBI5741368.1
Pseudomonadati Nitrospirota unclassified Nitrospirota Nitrospirota bacterium isolate NC groundwater 1766 Pr3 B0.1um 47 18 MBI5409800.1
Pseudomonadati Nitrospirota unclassified Nitrospirota Nitrospirota bacterium isolate NC groundwater 1913 Pr3 S2p5 46 161 MBI5056001.1
Pseudomonadati Nitrospirota unclassified Nitrospirota Nitrospirota bacterium isolate MAG082 MEW6603018.1
Pseudomonadati Nitrospirota Nitrospiria Nitrospirales Nitrospiraceae bacterium isolate SURF 23 RJQ44417.1
Pseudomonadati Nitrospirota Thermodesulfovibrionia Thermodesulfovibrionales Thermodesulfovibrionales bacterium isolate OHK006 GAB4484135.1
Pseudomonadati Nitrospirota unclassified Nitrospirota Nitrospirota bacterium isolate CTSoil 101 MBS1115573.1
Pseudomonadati Nitrospirota unclassified Nitrospirota Nitrospirota bacterium isolate NC groundwater 1767 Pr3 B0.1um 50 141 MBI5633021.1
Pseudomonadati Nitrospirota unclassified Nitrospirota Nitrospirota bacterium isolate MnB 6 NJD56353.1
Pseudomonadati Nitrospirota unclassified Nitrospirota Nitrospirota bacterium isolate DP16D bin.48 TAN38799.1
Pseudomonadati Nitrospirota unclassified Nitrospirota Nitrospirota bacterium isolate NC groundwater 1885 Pr3 B0.1um 52 11 MBI5103220.1
Pseudomonadati Nitrospirota Nitrospiria Nitrospirales Nitrospiraceae bacterium isolate WUY.bin8 MDA8082035.1
Pseudomonadati Nitrospirota Nitrospirota Thermodesulfovibrionia Thermodesulfovibrio sp. isolate SW.3.86 MBA4371855.1 314 aa
Pseudomonadati Nitrospirota unclassified Nitrospirota Nitrospirota bacterium isolate NC groundwater 1769 Pr3 B0.1um 50 9 MBI5641785.1
Pseudomonadati Nitrospirota Nitrospiria Nitrospirales Nitrospiraceae bacterium isolate bin398 UCE80174.1
Pseudomonadati Nitrospirota unclassified Nitrospirota Nitrospirota bacterium isolate MAG218 MEW6107688.1
Pseudomonadati Nitrospirota unclassified Nitrospirota Nitrospirae bacterium GWC2 42 7 OGW27837.1
Pseudomonadati Nitrospirota Nitrospiria Nitrospirales Nitrospiraceae bacterium isolate WUY.bin9 MDA8077453.1
Pseudomonadati Nitrospirota Nitrospiria Nitrospirales Candidatus Sulfobium sp. MEJ2697279.1 314 aa
Pseudomonadati Nitrospirota Nitrospiria Nitrospirales Candidatus Sulfobium mesophilum HSB30356.1 314 aa
Pseudomonadati Nitrospirota Nitrospiria Nitrospirales Nitrospiraceae bacterium isolate BPO.bin2 MDA8434161.1
Pseudomonadati Nitrospirota Nitrospiria Nitrospirales Nitrospiraceae bacterium isolate SKS.bin12 MDA8239415.1
Pseudomonadati Nitrospirota unclassified Nitrospirota Nitrospirota bacterium isolate NC groundwater 1772 Pr3 B0.1um 56 14 MBI5143122.1
Pseudomonadati Nitrospirota Nitrospirota Thermodesulfovibrionia Thermodesulfovibrio autotrophicus WP 353683254.1 314 aa
Pseudomonadati Nitrospirota Nitrospirota Thermodesulfovibrionia unclassified Thermodesulfovibrio multispecies WP 068861183.1 314 aa
Pseudomonadati Nitrospirota Nitrospirota Thermodesulfovibrionia Thermodesulfovibrio hydrogeniphilus GAB6183495.1 313 aa
Pseudomonadati Nitrospirota unclassified Nitrospirota Nitrospirota bacterium isolate nDJH8bin7 MBF0472931.1
Pseudomonadati Nitrospirota Nitrospiria Nitrospirales Candidatus Magnetovum chiemense KJR43035.1 314 aa
Pseudomonadati Nitrospirota Nitrospirota Thermodesulfovibrionia Candidatus Magnetobacterium casense WP 218252277.1 314 aa
Pseudomonadati Nitrospirota Nitrospiria Candidatus Magnetomonas Candidatus Magnetomonas plexicatena WP 444548199.1 314 aa
Pseudomonadati Nitrospirota Nitrospiria Nitrospirales Candidatus Magnetominusculus xianensis WP 085052976.1 314 aa
Pseudomonadati Nitrospirota Nitrospiria Nitrospirales Candidatus Magnetominusculus sp. LBB02 MCG6552419.1 313 aa
Pseudomonadati Nitrospirota Thermodesulfovibrionia Thermodesulfovibrionales Thermodesulfovibrionales bacterium isolate 21H 026.bin.27 MFN3739173.1
Pseudomonadati Nitrospirota Thermodesulfovibrionia Thermodesulfovibrionales Thermodesulfovibrionales bacterium isolate bin.9 MGW8272469.1
Pseudomonadati Nitrospirota unclassified Nitrospirota Nitrospirota bacterium isolate bin746 UCG77906.1
Pseudomonadati Nitrospirota Thermodesulfovibrionia Thermodesulfovibrionales Thermodesulfovibrionales bacterium isolate SCS6bin.3 MEE9523575.1
Pseudomonadati Nitrospirota unclassified Nitrospirota Nitrospirota bacterium isolate J105 RME66005.1
Pseudomonadati Nitrospirota unclassified Nitrospirota Nitrospirota bacterium isolate J037 RMG75159.1 314 aa
Pseudomonadati Nitrospirota unclassified Nitrospirota Nitrospirota bacterium isolate J053 RMG00943.1
Pseudomonadati Nitrospirota unclassified Nitrospirota Nitrospirota bacterium isolate M MetaBat.199 NOY64626.1
Pseudomonadati Nitrospirota unclassified Nitrospirota Nitrospirota bacterium isolate NC groundwater 1507 Pr4 B0.1um 42 5 MBI4685999.1
Pseudomonadati Nitrospirota unclassified Nitrospirota Nitrospirota bacterium isolate NC groundwater 1506 Pr4 B 0.1um 42 4 MBI4691676.1 313 aa
Pseudomonadati Nitrospirota unclassified Nitrospirota Nitrospirota bacterium isolate X2 MetaBAT.7 KAF0145138.1
Pseudomonadati Nitrospirota Nitrospirota Thermodesulfovibrionia Thermodesulfovibrionales bacterium isolate C13 concoct.bin.86 MDP1758943.1 313 aa
Pseudomonadati Nitrospirota Nitrospiria Nitrospirales Nitrospiraceae bacterium isolate bin.188 MCE5195206.1 313 aa
Pseudomonadati Nitrospirota Thermodesulfovibrionia Thermodesulfovibrionales Thermodesulfovibrionales bacterium isolate OHK0032 GAB4413006.1
Pseudomonadati Nitrospirota Nitrospirota Thermodesulfovibrionia Thermodesulfovibrionales bacterium isolate 21H 023.bin.63 MFN3480701.1 313 aa
Pseudomonadati Nitrospirota Thermodesulfovibrionia Thermodesulfovibrionales Thermodesulfovibrionales bacterium isolate SMAG U12310 HXX58949.1
Pseudomonadati Nitrospirota Thermodesulfovibrionia Thermodesulfovibrionales Thermodesulfovibrionales bacterium isolate SMAG U14663 HET6514400.1
Pseudomonadati Nitrospirota unclassified Nitrospirota Nitrospirota bacterium isolate H32bin144 1 MEK6672528.1
Pseudomonadati Nitrospirota unclassified Nitrospirota Nitrospirota bacterium isolate FK Sedi B Bin.60 MCL5023237.1 313 aa
Pseudomonadati Nitrospirota unclassified Nitrospirota Nitrospirota bacterium isolate FK Sedi A Bin.59 MCL4476392.1 313 aa
Pseudomonadati Nitrospirota Nitrospirota Thermodesulfovibrionia Thermodesulfovibrionales bacterium isolate SMAG U14772 HUO77653.1 313 aa
Pseudomonadati Nitrospirota Nitrospirota Thermodesulfovibrionia Thermodesulfovibrionales bacterium isolate SMAG U13108 HWR58251.1 313 aa
Pseudomonadati Nitrospirota unclassified Nitrospirota Nitrospirota bacterium isolate HyVt368 HEB02044.1
Pseudomonadati Nitrospirota Nitrospiria Nitrospirales Nitrospiraceae bacterium isolate TLO.bin5 MDA8169895.1
Pseudomonadati Nitrospirota Nitrospiria Nitrospirales Nitrospiraceae bacterium isolate TLO.bin4 MDA8171484.1
Pseudomonadati Nitrospirota Nitrospiria Nitrospirales Nitrospiraceae bacterium isolate WUY.bin7 MDA8091718.1 313 aa
Pseudomonadati Nitrospirota unclassified Nitrospirota Nitrospirota bacterium isolate Spart 075 MAG MEJ2183502.1
Pseudomonadati Nitrospirota Thermodesulfovibrionia Thermodesulfovibrionales Thermodesulfovibrionales bacterium isolate Kow0025 GAB4389614.1
Pseudomonadati Nitrospirota unclassified Nitrospirota Nitrospirota bacterium isolate Spart 117 MAG MEJ2315133.1
Pseudomonadati Nitrospirota unclassified Nitrospirota Nitrospirota bacterium isolate NC groundwater 1557 Pr4 S0.65um 43 18 MBI4822843.1
Pseudomonadati Nitrospirota Nitrospirota Thermodesulfovibrionia Thermodesulfovibrionales bacterium isolate C1 metabat.bin.51 MDP2166708.1 313 aa
Pseudomonadati Nitrospinota Tectimicrobiota group Nitrospinota Nitrospinota bacterium isolate BS2bin.9 MEE8482995.1 314 aa
Pseudomonadati Nitrospinota Tectimicrobiota group Nitrospinota Nitrospinota bacterium isolate S.4.sp1 bin.41 MFQ5431781.1 314 aa
Pseudomonadati Nitrospinota Tectimicrobiota group Nitrospinota Nitrospinota bacterium isolate 8 1 July SF Bin34 MDH5542682.1 316 aa
Pseudomonadati Nitrospinota Tectimicrobiota group Nitrospinota Nitrospinota bacterium isolate NC groundwater 1755 Pr3 B 0.1um 59 18 MBI5636781.1 314 aa
Pseudomonadati Nitrospinota Tectimicrobiota group Nitrospinota Nitrospinota bacterium isolate NC groundwater 1756 Pr3 B 0.1um 59 45 MBI5178326.1 314 aa
Pseudomonadati Nitrospinota Tectimicrobiota group Nitrospinota Nitrospinota bacterium isolate NC groundwater 950 Pr1 S 0.2um 59 16 MBI3580645.1 314 aa
Pseudomonadati Nitrospinota Tectimicrobiota group Nitrospinota Nitrospinota bacterium isolate NC groundwater 1032 Pr1 S 0.65um 54 26 MBI3794850.1 314 aa
Pseudomonadati Nitrospinota Tectimicrobiota group Nitrospinota Nitrospinota bacterium isolate nWMHbin6 MBF0170175.1 319 aa
Pseudomonadati Nitrospinota Tectimicrobiota group Nitrospinota Nitrospinota bacterium isolate DBRS3.bin6 MFW2330795.1 317 aa
Pseudomonadati Nitrospinota Tectimicrobiota group Nitrospinota Nitrospinota bacterium isolate NC groundwater 1503 Pr4 B 0.1um 56 23 MBI4666283.1 318 aa
Pseudomonadati Nitrospinota Tectimicrobiota group Nitrospinota Nitrospinota bacterium isolate NC groundwater 1881 Pr3 B 0.1um 57 61 MBI5814602.1 323 aa
Pseudomonadati Nitrospinota Tectimicrobiota group Nitrospinota Nitrospinota bacterium isolate nNGHbin12 MBF0291239.1 318 aa
Pseudomonadati Nitrospinota Tectimicrobiota group Nitrospinota Nitrospinota bacterium isolate Fu MAG 66 HJM83028.1 318 aa
Pseudomonadati Nitrospinota Tectimicrobiota group Nitrospinota Nitrospinota bacterium isolate NC groundwater 1556 Pr4 S 0.65um 63 8 MBI4829791.1 319 aa
Pseudomonadati Nitrospinota Tectimicrobiota group Nitrospinota Nitrospinota bacterium isolate 8 1 Jan SF Bin9 MDH5476713.1 318 aa
Pseudomonadati Nitrospinota Tectimicrobiota group Nitrospinota Nitrospinota bacterium isolate 4 1 July SF Bin3 MDH4183540.1 318 aa
Pseudomonadati Nitrospinota Tectimicrobiota group Candidatus Tectimicrobiota Candidatus Tectimicrobiota bacterium isolate NC groundwater 717 Ag S 0.2um 59 8 MBI3015184.1 312 a
Pseudomonadati Nitrospinota Tectimicrobiota group Candidatus Tectimicrobiota Candidatus Tectomicrobiota bacterium isolate NC groundwater 748 Ag S 0.65um 67 15 MBI3090917.1 31
Pseudomonadati Nitrospinota Tectimicrobiota group Candidatus Tectimicrobiota Candidatus Tectomicrobiota bacterium isolate APA bin 51 MCY4514367.1 314 aa
Pseudomonadati Nitrospinota Tectimicrobiota group Candidatus Tectimicrobiota Candidatus Tectimicrobiota bacterium isolate MT4bin.13 MEE8292454.1 313 aa
Pseudomonadati Nitrospinota Tectimicrobiota group Candidatus Tectimicrobiota Candidatus Entotheonella sp. isolate SMAG U3999 HEY7711219.1 313 aa
Pseudomonadati Nitrospinota Tectimicrobiota group Nitrospinota Nitrospinota bacterium isolate SMAG U14208 HUU50054.1 313 aa
Pseudomonadati Nitrospinota Tectimicrobiota group Nitrospinota Nitrospinae bacterium RIFCSPLOWO2 12 FULL 45 22 OGW15056.1 313 aa
Pseudomonadati Nitrospinota Tectimicrobiota group Candidatus Tectimicrobiota Candidatus Tectimicrobiota bacterium isolate NC groundwater 672 Ag B 0.1um 62 36 MBI2876091.1 312
Pseudomonadati Nitrospinota Tectimicrobiota group Nitrospinota Nitrospinota bacterium isolate Fu MAG 98 HJM41762.1 307 aa
Pseudomonadati Nitrospinota Tectimicrobiota group Nitrospinota Nitrospinota bacterium isolate Maj 1 bin.58 MFQ5691612.1 313 aa
Pseudomonadati Nitrospinota Tectimicrobiota group Nitrospinota Nitrospinota bacterium isolate K 2 bin.39 MFQ5911971.1 313 aa
Pseudomonadati Nitrospinota Tectimicrobiota group Nitrospinota Nitrospinota bacterium isolate K 1 bin.23 MFQ5895174.1 542 aa
Pseudomonadati Nitrospinota Tectimicrobiota group Nitrospinota Nitrospinota bacterium isolate b2.sp2 bin.13 MFQ6110862.1 312 aa
Pseudomonadati Nitrospinota Tectimicrobiota group Nitrospinota Nitrospinota bacterium isolate H14 bin062 1 MEK6710403.1 309 aa
Pseudomonadati Nitrospinota Tectimicrobiota group Candidatus Tectimicrobiota Candidatus Tectomicrobia bacterium RIFCSPLOWO2 02 FULL 70 19 OGL61142.1 309 aa
Pseudomonadati Nitrospinota Tectimicrobiota group Nitrospinota Nitrospinota bacterium isolate XB MAG82 MED5578443.1 313 aa
Pseudomonadati Nitrospinota Tectimicrobiota group Nitrospinota Nitrospinota bacterium isolate SB0662 bin 51 MYA95395.1 314 aa
Pseudomonadati Nitrospinota Tectimicrobiota group Nitrospinota Nitrospinota bacterium isolate OC6 4000m MAG 328 MFP6867864.1 315 aa
Pseudomonadati Nitrospinota Tectimicrobiota group Nitrospinota Nitrospinota bacterium isolate ETNP15 MAG 5 MDP6277511.1 315 aa
Pseudomonadati Nitrospinota Tectimicrobiota group Nitrospinota Nitrospinia Nitrospinaceae bacterium isolate co234 bin71 MBT3352051.1 320 aa
Pseudomonadati Nitrospinota Tectimicrobiota group Nitrospinota Nitrospinota bacterium isolate NC groundwater 1753 Pr3 B 0.1um 50 18 MBI5187122.1 313 aa
Pseudomonadati Nitrospinota Tectimicrobiota group Nitrospinota Nitrospinota bacterium isolate ETNP7 MAG 4 MDP7350177.1 313 aa
Pseudomonadati Nitrospinota Tectimicrobiota group Nitrospinota Nitrospinota bacterium isolate NC groundwater 1751 Pr3 B 0.1um 39 18 MBI5182858.1 313 aa
Pseudomonadati Nitrospinota Tectimicrobiota group Nitrospinota Nitrospinota bacterium isolate N04 S1C18675 MFV1951121.1 314 aa

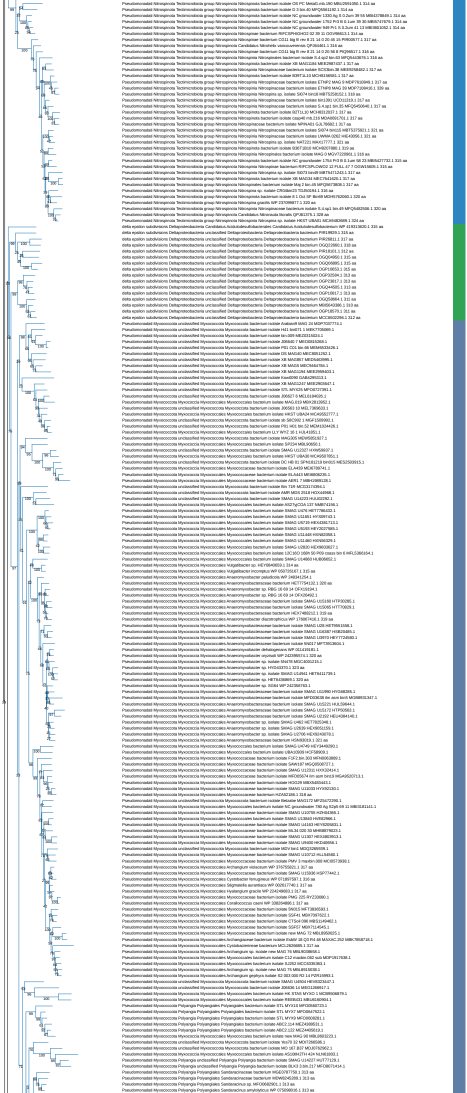

Pseudomonadati Nitrospinota Tectimicrobiota group Nitrospinota Nitrospinota bacterium isolate OS PC MetaG.mb.190 MBU2591050.1 314 aa
Pseudomonadati Nitrospinota Tectimicrobiota group Nitrospinota Nitrospinota bacterium isolate D 3 bin.40 MFQ5561192.1 314 aa
Pseudomonadati Nitrospinota Tectimicrobiota group Nitrospinota Nitrospinota bacterium isolate NC groundwater 1330 Ag S 0.2um 39 55 MBI4378849.1 314 aa
Pseudomonadati Nitrospinota Tectimicrobiota group Nitrospinota Nitrospinota bacterium isolate NC groundwater 1752 Pr3 B 0.1um 39 30 MBI5747679.1 314 aa
Pseudomonadati Nitrospinota Tectimicrobiota group Nitrospinota Nitrospinota bacterium isolate NC groundwater 949 Pr1 S 0.2um 41 13 MBI3601052.1 314 aa
Pseudomonadati Nitrospinota Tectimicrobiota group Nitrospinota Nitrospinae bacterium RIFCSPHIGHO2 02 39 11 OGV98613.1 314 aa
Pseudomonadati Nitrospinota Tectimicrobiota group Nitrospinota Nitrospinae bacterium CG11 big fil rev 8 21 14 0 20 45 15 PIR00577.1 317 aa
Pseudomonadati Nitrospinota Tectimicrobiota group Nitrospinota Candidatus Nitrohelix vancouverensis QPJ64461.1 316 aa
Pseudomonadati Nitrospinota Tectimicrobiota group Nitrospinota Nitrospinae bacterium CG11 big fil rev 8 21 14 0 20 56 8 PIQ96517.1 316 aa
Pseudomonadati Nitrospinota Tectimicrobiota group Nitrospinota Nitrospinia Nitrospinales bacterium isolate S.4.sp2 bin.63 MFQ5443676.1 316 aa
Pseudomonadati Nitrospinota Tectimicrobiota group Nitrospinota Nitrospinota bacterium isolate XB MAG1184 MEE2987437.1 317 aa
Pseudomonadati Nitrospinota Tectimicrobiota group Nitrospinota Nitrospinia Nitrospinaceae bacterium isolate SCS3bin.38 MEE9258482.1 317 aa
Pseudomonadati Nitrospinota Tectimicrobiota group Nitrospinota Nitrospinota bacterium isolate B39T1L10 MCH8156581.1 317 aa
Pseudomonadati Nitrospinota Tectimicrobiota group Nitrospinota Nitrospinia Nitrospinaceae bacterium isolate ETNP2 MAG 9 MDP7610849.1 317 aa
Pseudomonadati Nitrospinota Tectimicrobiota group Nitrospinota Nitrospinia Nitrospinaceae bacterium isolate ETNP8 MAG 39 MDP7108416.1 339 aa
Pseudomonadati Nitrospinota Tectimicrobiota group Nitrospinota Nitrospinia Nitrospina sp. isolate SI074 bin18 MBT5258152.1 318 aa
Pseudomonadati Nitrospinota Tectimicrobiota group Nitrospinota Nitrospinia Nitrospinaceae bacterium isolate bin1391 UCD11319.1 317 aa
Pseudomonadati Nitrospinota Tectimicrobiota group Nitrospinota Nitrospinia Nitrospinaceae bacterium isolate S.4.sp1 bin.35 MFQ5450640.1 317 aa
Pseudomonadati Nitrospinota Tectimicrobiota group Nitrospinota Nitrospinota bacterium isolate B2T1L10 MCH8312037.1 317 aa
Pseudomonadati Nitrospinota Tectimicrobiota group Nitrospinota Nitrospinota bacterium isolate casp40 mb.216 MDA0691701.1 317 aa
Pseudomonadati Nitrospinota Tectimicrobiota group Nitrospinota Nitrospinaceae bacterium isolate NPINA01 GJL78682.1 317 aa
Pseudomonadati Nitrospinota Tectimicrobiota group Nitrospinota Nitrospinia Nitrospinaceae bacterium isolate SI074 bin115 MBT5375921.1 321 aa
Pseudomonadati Nitrospinota Tectimicrobiota group Nitrospinota Nitrospinia Nitrospinaceae bacterium isolate UWMA 0262 HIE43056.1 321 aa
Pseudomonadati Nitrospinota Tectimicrobiota group Nitrospinota Nitrospinia Nitrospina sp. isolate NAT221 MAX17777.1 321 aa
Pseudomonadati Nitrospinota Tectimicrobiota group Nitrospinota Nitrospinota bacterium isolate B36T1B10 MCH8207880.1 319 aa
Pseudomonadati Nitrospinota Tectimicrobiota group Nitrospinota Nitrospinia Nitrospinales bacterium isolate MAG 0 MGV7220961.1 316 aa
Pseudomonadati Nitrospinota Tectimicrobiota group Nitrospinota Nitrospinota bacterium isolate NC groundwater 1754 Pr3 B 0.1um 58 23 MBI5427732.1 315 aa
Pseudomonadati Nitrospinota Tectimicrobiota group Nitrospinota Nitrospinia Nitrospinae bacterium RIFCSPLOWO2 12 FULL 47 7 OGW15605.1 315 aa
Pseudomonadati Nitrospinota Tectimicrobiota group Nitrospinota Nitrospinia Nitrospina sp. isolate SI073 bin49 MBT5471243.1 317 aa
Pseudomonadati Nitrospinota Tectimicrobiota group Nitrospinota Nitrospinota bacterium isolate XB MAG34 MEC7641620.1 317 aa
Pseudomonadati Nitrospinota Tectimicrobiota group Nitrospinota Nitrospinales bacterium isolate Maj 2 bin.45 MFQ5673808.1 317 aa
Pseudomonadati Nitrospinota Tectimicrobiota group Nitrospinota Nitrospina sp. isolate CR04bin23 TDJ50194.1 316 aa
Pseudomonadati Nitrospinota Tectimicrobiota group Nitrospinota Nitrospinota bacterium isolate 8 1 Oct SF Bin69 MDH5762060.1 320 aa
Pseudomonadati Nitrospinota Tectimicrobiota group Nitrospinota Nitrospina gracilis WP 237099877.1 320 aa
Pseudomonadati Nitrospinota Tectimicrobiota group Nitrospinota Nitrospinia Nitrospinaceae bacterium isolate S.4.sp1 bin.49 MFQ5482506.1 320 aa
Pseudomonadati Nitrospinota Tectimicrobiota group Nitrospinota Candidatus Nitronauta litoralis QPJ61375.1 328 aa
Pseudomonadati Nitrospinota Tectimicrobiota group Nitrospinota Nitrospinia Nitrospina sp. isolate HKST UBA01 MCA9482689.1 324 aa
delta epsilon subdivisions Deltaproteobacteria Candidatus Acidulodesulfobacterales Candidatus Acidulodesulfobacterium WP 419313620.1 315 aa
delta epsilon subdivisions Deltaproteobacteria unclassified Deltaproteobacteria Deltaproteobacteria bacterium PIR19929.1 315 aa
delta epsilon subdivisions Deltaproteobacteria unclassified Deltaproteobacteria Deltaproteobacteria bacterium PIR26811.1 317 aa
delta epsilon subdivisions Deltaproteobacteria unclassified Deltaproteobacteria Deltaproteobacteria bacterium OGQ22660.1 318 aa
delta epsilon subdivisions Deltaproteobacteria unclassified Deltaproteobacteria Deltaproteobacteria bacterium PIR18101.1 312 aa
delta epsilon subdivisions Deltaproteobacteria unclassified Deltaproteobacteria Deltaproteobacteria bacterium OGQ04950.1 315 aa
delta epsilon subdivisions Deltaproteobacteria unclassified Deltaproteobacteria Deltaproteobacteria bacterium OGQ06895.1 315 aa
delta epsilon subdivisions Deltaproteobacteria unclassified Deltaproteobacteria Deltaproteobacteria bacterium OGP10653.1 315 aa
delta epsilon subdivisions Deltaproteobacteria unclassified Deltaproteobacteria Deltaproteobacteria bacterium OGP32584.1 313 aa
delta epsilon subdivisions Deltaproteobacteria unclassified Deltaproteobacteria Deltaproteobacteria bacterium OGP23817.1 313 aa
delta epsilon subdivisions Deltaproteobacteria unclassified Deltaproteobacteria Deltaproteobacteria bacterium OGQ44505.1 313 aa
delta epsilon subdivisions Deltaproteobacteria unclassified Deltaproteobacteria Deltaproteobacteria bacterium OGP10817.1 313 aa
delta epsilon subdivisions Deltaproteobacteria unclassified Deltaproteobacteria Deltaproteobacteria bacterium OGQ58664.1 311 aa
delta epsilon subdivisions Deltaproteobacteria unclassified Deltaproteobacteria Deltaproteobacteria bacterium MBI5643386.1 310 aa
delta epsilon subdivisions Deltaproteobacteria unclassified Deltaproteobacteria Deltaproteobacteria bacterium OGP18570.1 311 aa
delta epsilon subdivisions Deltaproteobacteria unclassified Deltaproteobacteria Deltaproteobacteria bacterium MCC6502296.1 312 aa
Pseudomonadati Myxococcota unclassified Myxococcota Myxococcota bacterium isolate Arabian9 MAG 24 MDP7037774.1
Pseudomonadati Myxococcota unclassified Myxococcota Myxococcota bacterium isolate H41 bin071 1 MEK7705069.1
Pseudomonadati Myxococcota unclassified Myxococcota Myxococcota bacterium isolate bin.009 MEZ0315024.1
Pseudomonadati Myxococcota unclassified Myxococcota Myxococcota bacterium isolate J06640 7 MEO0815268.1
Pseudomonadati Myxococcota unclassified Myxococcota Myxococcota bacterium isolate P01 C01 bin.66 MEM6533426.1
Pseudomonadati Myxococcota unclassified Myxococcota Myxococcota bacterium isolate DS MAG40 MEC8051252.1
Pseudomonadati Myxococcota unclassified Myxococcota Myxococcota bacterium isolate XB MAG857 MED5463995.1
Pseudomonadati Myxococcota unclassified Myxococcota Myxococcota bacterium isolate XB MAG5 MEC9464784.1
Pseudomonadati Myxococcota unclassified Myxococcota Myxococcota bacterium isolate XB MAG1194 MEE2959403.1
Pseudomonadati Myxococcota unclassified Myxococcota Myxococcota bacterium isolate Kow0090 GAB4295313.1
Pseudomonadati Myxococcota unclassified Myxococcota Myxococcota bacterium isolate XB MAG1247 MEE2903647.1
Pseudomonadati Myxococcota unclassified Myxococcota Myxococcota bacterium isolate STL MYX25 MFO0727391.1
Pseudomonadati Myxococcota unclassified Myxococcota Myxococcota bacterium isolate J06627 6 MEL6184026.1
Pseudomonadati Myxococcota Myxococcia Myxococcales Myxococcales bacterium isolate MAG.019 MBX2813952.1
Pseudomonadati Myxococcota unclassified Myxococcota Myxococcota bacterium isolate J06563 10 MEL7369633.1
Pseudomonadati Myxococcota Myxococcia Myxococcales Myxococcales bacterium isolate HKST UBA24 MCA9552777.1
Pseudomonadati Myxococcota unclassified Myxococcota Myxococcota bacterium isolate sb S8C902 1 MGF1509992.1
Pseudomonadati Myxococcota unclassified Myxococcota Myxococcota bacterium isolate P01 H01 bin.52 MEM1024426.1
Pseudomonadati Myxococcota Myxococcia Myxococcales Myxococcales bacterium LLY WYZ 16 1 HJL41851.1
Pseudomonadati Myxococcota unclassified Myxococcota Myxococcota bacterium isolate MAG305 MEW5851927.1
Pseudomonadati Myxococcota Myxococcia Myxococcales Myxococcales bacterium isolate SP234 MBL90650.1
Pseudomonadati Myxococcota unclassified Myxococcota Myxococcota bacterium isolate SMAG U12327 HXW59937.1
Pseudomonadati Myxococcota Myxococcia Myxococcales Myxococcales bacterium isolate HKST UBA30 MCA9507851.1
Pseudomonadati Myxococcota unclassified Myxococcota Myxococcota bacterium isolate DC HB 01 SPN181219 bin015 MES2503915.1
Pseudomonadati Myxococcota Myxococcia Myxococcales Myxococcaceae bacterium isolate ELA439 MEI6789741.1
Pseudomonadati Myxococcota Myxococcia Myxococcales Myxococcaceae bacterium isolate ELA443 MEI6806235.1
Pseudomonadati Myxococcota Myxococcia Myxococcales Myxococcaceae bacterium isolate AER1 7 MBH1989128.1
Pseudomonadati Myxococcota unclassified Myxococcota Myxococcota bacterium isolate Bin 71R MCG3174394.1
Pseudomonadati Myxococcota unclassified Myxococcota Myxococcota bacterium isolate AMR MDS 2518 HOX44968.1
Pseudomonadati Myxococcota unclassified Myxococcota Myxococcota bacterium isolate SMAG U14223 HUU02292.1
Pseudomonadati Myxococcota Myxococcia Myxococcales Myxococcales bacterium isolate AS27yjCOA 137 NMB74156.1
Pseudomonadati Myxococcota Myxococcia Myxococcales Myxococcales bacterium isolate SMAG U476 HET7786432.1
Pseudomonadati Myxococcota Myxococcia Myxococcales Myxococcales bacterium isolate SMAG U11651 HYS09743.1
Pseudomonadati Myxococcota Myxococcia Myxococcales Myxococcales bacterium isolate SMAG U5719 HEX4381713.1
Pseudomonadati Myxococcota Myxococcia Myxococcales Myxococcales bacterium isolate SMAG U5193 HEY2027585.1
Pseudomonadati Myxococcota Myxococcia Myxococcales Myxococcales bacterium isolate SMAG U11448 HXN82058.1
Pseudomonadati Myxococcota Myxococcia Myxococcales Myxococcales bacterium isolate SMAG U11460 HXN56329.1
Pseudomonadati Myxococcota Myxococcia Myxococcales Myxococcales bacterium isolate SMAG U2820 HEX9603627.1
Pseudomonadati Myxococcota Myxococcia Myxococcales Myxococcales bacterium isolate 12C16O 168h 50 P09 coass bin 6 MFL5366164.1
Pseudomonadati Myxococcota Myxococcia Myxococcales Myxococcales bacterium isolate SMAG U14860 HUB06652.1
Pseudomonadati Myxococcota Myxococcia Myxococcales Vulgatibacter sp. HEY0840659.1 314 aa
Pseudomonadati Myxococcota Myxococcia Myxococcales Vulgatibacter incomptus WP 050726167.1 315 aa
Pseudomonadati Myxococcota Myxococcia Myxococcales Anaeromyxobacter paludicola WP 248341254.1
Pseudomonadati Myxococcota Myxococcia Myxococcales Anaeromyxobacteraceae bacterium HET7754132.1 320 aa
Pseudomonadati Myxococcota Myxococcia Myxococcales Anaeromyxobacter sp. RBG 16 69 14 OFX19194.1
Pseudomonadati Myxococcota Myxococcia Myxococcales Anaeromyxobacter sp. RBG 16 69 14 OFX26492.1
Pseudomonadati Myxococcota Myxococcia Myxococcales Anaeromyxobacteraceae bacterium isolate SMAG U15160 HTP30285.1
Pseudomonadati Myxococcota Myxococcia Myxococcales Anaeromyxobacteraceae bacterium isolate SMAG U15065 HTT70829.1
Pseudomonadati Myxococcota Myxococcia Myxococcales Anaeromyxobacteraceae bacterium HEX7488212.1 319 aa
Pseudomonadati Myxococcota Myxococcia Myxococcales Anaeromyxobacter diazotrophicus WP 176067416.1 319 aa
Pseudomonadati Myxococcota Myxococcia Myxococcales Anaeromyxobacteraceae bacterium isolate SMAG U28 HET9551558.1
Pseudomonadati Myxococcota Myxococcia Myxococcales Anaeromyxobacteraceae bacterium isolate SMAG U16387 HSB20485.1
Pseudomonadati Myxococcota Myxococcia Myxococcales Anaeromyxobacteraceae bacterium isolate SMAG U3970 HEY7724580.1
Pseudomonadati Myxococcota Myxococcia Myxococcales Anaeromyxobacteraceae bacterium isolate 5N017 MFT3913604.1
Pseudomonadati Myxococcota Myxococcia Myxococcales Anaeromyxobacter dehalogenans WP 011419181.1
Pseudomonadati Myxococcota Myxococcia Myxococcales Anaeromyxobacter oryzisoli WP 242395574.1 320 aa
Pseudomonadati Myxococcota Myxococcia Myxococcales Anaeromyxobacter sp. isolate 5N478 MGC4001215.1
Pseudomonadati Myxococcota Myxococcia Myxococcales Anaeromyxobacter sp. HYD43370.1 323 aa
Pseudomonadati Myxococcota Myxococcia Myxococcales Anaeromyxobacter sp. isolate SMAG U14941 HET6411739.1
Pseudomonadati Myxococcota Myxococcia Myxococcales Anaeromyxobacter sp. HET6436869.1 320 aa
Pseudomonadati Myxococcota Myxococcia Myxococcales Anaeromyxobacter sp. SG64 WP 242356763.1
Pseudomonadati Myxococcota Myxococcia Myxococcales Anaeromyxobacteraceae bacterium isolate SMAG U11990 HYG68285.1
Pseudomonadati Myxococcota Myxococcia Myxococcales Anaeromyxobacteraceae bacterium isolate MFD03638 ilm asm bin5 MGB8931347.1
Pseudomonadati Myxococcota Myxococcia Myxococcales Anaeromyxobacteraceae bacterium isolate SMAG U15221 HUL59644.1
Pseudomonadati Myxococcota Myxococcia Myxococcales Anaeromyxobacteraceae bacterium isolate SMAG U15172 HTP50563.1
Pseudomonadati Myxococcota Myxococcia Myxococcales Anaeromyxobacteraceae bacterium isolate SMAG U2192 HEU4384140.1
Pseudomonadati Myxococcota Myxococcia Myxococcales Anaeromyxobacter sp. isolate SMAG U462 HET7825348.1
Pseudomonadati Myxococcota Myxococcia Myxococcales Anaeromyxobacter sp. isolate SMAG U2639 HEX9051159.1
Pseudomonadati Myxococcota Myxococcia Myxococcales Anaeromyxobacter sp. isolate SMAG U2706 HEX9243078.1
Pseudomonadati Myxococcota Myxococcia Myxococcales Anaeromyxobacteraceae bacterium HSN93019.1 321 aa
Pseudomonadati Myxococcota Myxococcia Myxococcales Myxococcales bacterium isolate SMAG U4749 HEY3449290.1
Pseudomonadati Myxococcota Myxococcia Myxococcales Myxococcales bacterium isolate UBA10939 HCF58909.1
Pseudomonadati Myxococcota Myxococcia Myxococcales Myxococcaceae bacterium isolate F1F2.bin.303 MFN0063889.1
Pseudomonadati Myxococcota Myxococcia Myxococcales Myxococcaceae bacterium isolate SAW187 MGQ0508727.1
Pseudomonadati Myxococcota Myxococcia Myxococcales Myxococcaceae bacterium isolate SMAG U12311 HXX32414.1
Pseudomonadati Myxococcota Myxococcia Myxococcales Myxococcaceae bacterium isolate MFD05674 ilm asm bin19 MGA9520713.1
Pseudomonadati Myxococcota Myxococcia Myxococcales Myxococcaceae bacterium isolate HOG29 MBX5483443.1
Pseudomonadati Myxococcota Myxococcia Myxococcales Myxococcaceae bacterium isolate SMAG U11033 HYX92130.1
Pseudomonadati Myxococcota Myxococcia Myxococcales Myxococcaceae bacterium HZA52186.1 318 aa
Pseudomonadati Myxococcota unclassified Myxococcota Myxococcota bacterium isolate Betzabe MAG172 MFZ5472290.1
Pseudomonadati Myxococcota Myxococcia Myxococcales Myxococcales bacterium isolate NC groundwater 780 Ag S2p5 69 11 MBI3181141.1
Pseudomonadati Myxococcota Myxococcia Myxococcales Myxococcaceae bacterium isolate SMAG U10755 HZH04365.1
Pseudomonadati Myxococcota Myxococcia Myxococcales Myxococcales bacterium isolate SMAG U13840 HVE82966.1
Pseudomonadati Myxococcota Myxococcia Myxococcales Myxococcaceae bacterium isolate SMAG U4163 HEY8205831.1
Pseudomonadati Myxococcota Myxococcia Myxococcales Myxococcaceae bacterium isolate ML34 020 30 MHB8879023.1
Pseudomonadati Myxococcota Myxococcia Myxococcales Myxococcaceae bacterium isolate SMAG U1307 HEX4803913.1
Pseudomonadati Myxococcota Myxococcia Myxococcales Myxococcaceae bacterium isolate SMAG U9400 HKD40656.1
Pseudomonadati Myxococcota unclassified Myxococcota Myxococcota bacterium isolate MDV bin1 MDQ3265939.1
Pseudomonadati Myxococcota Myxococcia Myxococcales Myxococcaceae bacterium isolate SMAG U10712 HLL54560.1
Pseudomonadati Myxococcota Myxococcia Myxococcales Myxococcaceae bacterium isolate PMV 3 maxbin.008 MCI0573938.1
Pseudomonadati Myxococcota Myxococcia Myxococcales Archangium violaceum WP 376755821.1 317 aa
Pseudomonadati Myxococcota Myxococcia Myxococcales Myxococcaceae bacterium isolate SMAG U15936 HSP77442.1
Pseudomonadati Myxococcota Myxococcia Myxococcales Cystobacter ferrugineus WP 071897597.1 316 aa
Pseudomonadati Myxococcota Myxococcia Myxococcales Stigmatella aurantiaca WP 002617740.1 317 aa
Pseudomonadati Myxococcota Myxococcia Myxococcales Hyalangium gracile WP 224249683.1 317 aa
Pseudomonadati Myxococcota Myxococcia Myxococcales Myxococcaceae bacterium isolate PMG 225 RYZ33080.1
Pseudomonadati Myxococcota Myxococcia Myxococcales Corallococcus caeni WP 338264886.1 317 aa
Pseudomonadati Myxococcota Myxococcia Myxococcales Myxococcaceae bacterium isolate 5N015 MFT3836593.1
Pseudomonadati Myxococcota Myxococcia Myxococcales Myxococcaceae bacterium isolate SSF41 MBX7097622.1
Pseudomonadati Myxococcota Myxococcia Myxococcales Myxococcaceae bacterium isolate CTSoil 096 MBS1149462.1
Pseudomonadati Myxococcota Myxococcia Myxococcales Myxococcaceae bacterium isolate SSF57 MBX7114545.1
Pseudomonadati Myxococcota Myxococcia Myxococcales Myxococcaceae bacterium isolate new MAG 72 MBL8950025.1
Pseudomonadati Myxococcota Myxococcia Myxococcales Archangiaceae bacterium isolate EsbW 18 Q3 R4 48 MAXAC.252 MBK7858718.1
Pseudomonadati Myxococcota Myxococcia Myxococcales Cystobacterineae bacterium MCL2626685.1 317 aa
Pseudomonadati Myxococcota Myxococcia Myxococcales Archangium sp. isolate new MAG 76 MBL9038658.1
Pseudomonadati Myxococcota Myxococcia Myxococcales Myxococcales bacterium isolate C12 maxbin.062 sub MDP1917638.1
Pseudomonadati Myxococcota Myxococcia Myxococcales Myxococcales bacterium isolate SJ252 MCC6335363.1
Pseudomonadati Myxococcota Myxococcia Myxococcales Archangium sp. isolate new MAG 75 MBL8915038.1
Pseudomonadati Myxococcota Myxococcia Myxococcales Archangium gephyra isolate S2 003 000 R2 14 PZR15993.1
Pseudomonadati Myxococcota unclassified Myxococcota Myxococcota bacterium isolate SMAG U4504 HEV8323447.1
Pseudomonadati Myxococcota unclassified Myxococcota Myxococcota bacterium isolate J06636 14 MEO1266917.1
Pseudomonadati Myxococcota Myxococcia Myxococcales Myxococcales bacterium isolate HK STAS MYXO 1 MCB9506879.1
Pseudomonadati Myxococcota Myxococcia Myxococcales Myxococcales bacterium isolate REEB431 MBU6160904.1
Pseudomonadati Myxococcota Polyangia Polyangiales Polyangiales bacterium isolate STL MYX10 MFO0560723.1
Pseudomonadati Myxococcota Polyangia Polyangiales Polyangiales bacterium isolate STL MYX7 MFO0647522.1
Pseudomonadati Myxococcota Polyangia Polyangiales Polyangiales bacterium isolate STL MYX9 MFO0608281.1
Pseudomonadati Myxococcota Polyangia Polyangiales Polyangiales bacterium isolate ABC2.114 MEZ4389531.1
Pseudomonadati Myxococcota Polyangia Polyangiales Polyangiales bacterium isolate ABC2.122 MEZ4405619.1
Pseudomonadati Myxococcota Myxococcia Myxococcales Myxococcales bacterium isolate new MAG 90 MBL8603223.1
Pseudomonadati Myxococcota unclassified Myxococcota Myxococcota bacterium isolate Yes70 32 MDI7266586.1
Pseudomonadati Myxococcota unclassified Myxococcota Myxococcota bacterium isolate MO 167.B37 MDJ0762962.1
Pseudomonadati Myxococcota Myxococcia Myxococcales Myxococcales bacterium isolate AS10tlH2TH 424 NLN61803.1
Pseudomonadati Myxococcota Polyangia unclassified Polyangia Polyangia bacterium isolate SMAG U14227 HUT77129.1
Pseudomonadati Myxococcota Polyangia unclassified Polyangia Polyangia bacterium isolate BLK3 3.bin.217 MFO8071414.1
Pseudomonadati Myxococcota Polyangia Polyangiales Sandaracinaceae bacterium MGE0787750.1 313 aa
Pseudomonadati Myxococcota Polyangia Polyangiales Sandaracinaceae bacterium MDW8245289.1 313 aa
Pseudomonadati Myxococcota Polyangia Polyangiales Sandaracinus sp. MFO0682901.1 313 aa
Pseudomonadati Myxococcota Polyangia Polyangiales Sandaracinus amylolyticus WP 075098016.1 313 aa

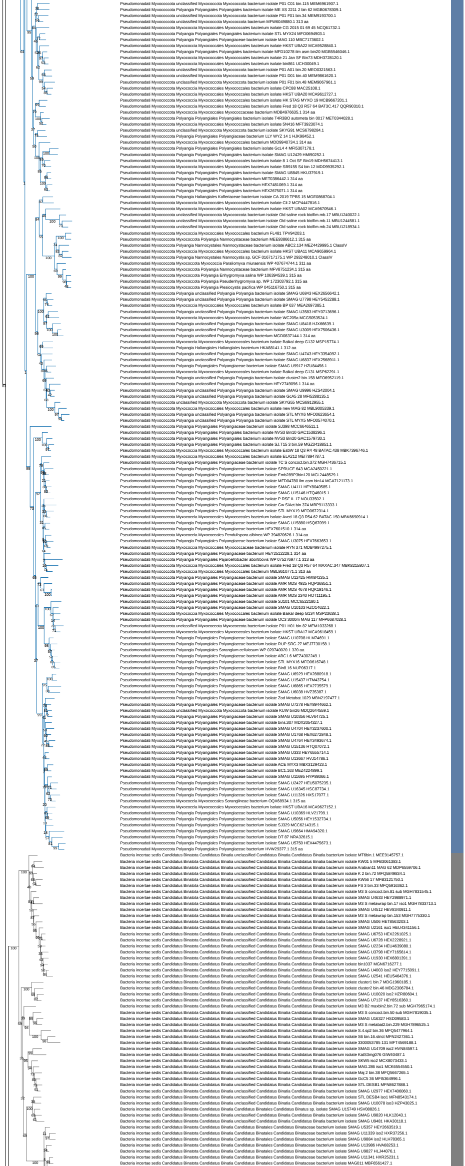
Pseudomonadati Myxococcota unclassified Myxococcota Myxococcota bacterium isolate P01 C01 bin.115 MEM6961907.1
Pseudomonadati Myxococcota Polyangia Polyangiales Polyangiales bacterium isolate ME XS 2211 2 bin 62 MGB0678309.1
Pseudomonadati Myxococcota unclassified Myxococcota Myxococcota bacterium isolate P01 F01 bin.34 MEM9193700.1
Pseudomonadati Myxococcota unclassified Myxococcota Myxococcota bacterium MFW6049880.1 313 aa
Pseudomonadati Myxococcota Myxococcia Myxococcales Myxococcales bacterium isolate CG 2015 01 69 45 NCQ61732.1
Pseudomonadati Myxococcota Polyangia Polyangiales Polyangiales bacterium isolate STL MYX24 MFO0694903.1
Pseudomonadati Myxococcota Polyangia Polyangiales Polyangiaceae bacterium isolate MAG 110 MBC7173602.1
Pseudomonadati Myxococcota Myxococcia Myxococcales Myxococcales bacterium isolate HKST UBA22 MCA9528840.1
Pseudomonadati Myxococcota Polyangia Polyangiales Polyangiales bacterium isolate MFD10278 ilm asm bin20 MGB5546046.1
Pseudomonadati Myxococcota Myxococcia Myxococcales Myxococcales bacterium isolate 21 Jan SF Bin73 MDH3728120.1
Pseudomonadati Myxococcota Myxococcia Myxococcales Myxococcales bacterium isolate bin861 UCH30049.1
Pseudomonadati Myxococcota unclassified Myxococcota Myxococcota bacterium isolate P01 A01 bin.20 MEO0321563.1
Pseudomonadati Myxococcota unclassified Myxococcota Myxococcota bacterium isolate P01 D01 bin.40 MEM9861620.1
Pseudomonadati Myxococcota unclassified Myxococcota Myxococcota bacterium isolate P01 F01 bin.48 MEM9067961.1
Pseudomonadati Myxococcota Myxococcia Myxococcales Myxococcales bacterium isolate CPC88 MAC25108.1
Pseudomonadati Myxococcota Myxococcia Myxococcales Myxococcales bacterium isolate HKST UBA20 MCA9612727.1
Pseudomonadati Myxococcota Myxococcia Myxococcales Myxococcales bacterium isolate HK STAS MYXO 19 MCB9667201.1
Pseudomonadati Myxococcota Myxococcia Myxococcales Myxococcales bacterium isolate Fred 18 Q3 R57 64 BAT3C.417 QQR90310.1
Pseudomonadati Myxococcota Myxococcia Myxococcales Myxococcaceae bacterium MDB4976635.1 314 aa
Pseudomonadati Myxococcota Polyangia Polyangiales Polyangiales bacterium isolate T4R3BO autometa bin 0017 MET0344028.1
Pseudomonadati Myxococcota Myxococcia Myxococcales Myxococcales bacterium isolate 5N416 MFT3923074.1
Pseudomonadati Myxococcota unclassified Myxococcota Myxococcota bacterium isolate SKYG91 MCS6798284.1
Pseudomonadati Myxococcota Polyangia Polyangiales Polyangiaceae bacterium LLY WYZ 14 1 HJK98452.1
Pseudomonadati Myxococcota Myxococcia Myxococcales Myxococcales bacterium MDD9940734.1 314 aa
Pseudomonadati Myxococcota Polyangia Polyangiales Polyangiales bacterium isolate GcL4 4 MFI5307178.1
Pseudomonadati Myxococcota Polyangia Polyangiales Polyangiales bacterium isolate SMAG U12429 HMI90252.1
Pseudomonadati Myxococcota Myxococcia Myxococcales Myxococcales bacterium isolate 8 1 Oct SF Bin19 MDH5674413.1
Pseudomonadati Myxococcota Myxococcia Myxococcales Myxococcales bacterium isolate SB9155 S4 bin 12 MDD9935292.1
Pseudomonadati Myxococcota Polyangia Polyangiales Polyangiales bacterium isolate SMAG U8845 HKU37919.1
Pseudomonadati Myxococcota Polyangia Polyangiales Polyangiales bacterium MET0386442.1 314 aa
Pseudomonadati Myxococcota Polyangia Polyangiales Polyangiales bacterium HEX7481069.1 314 aa
Pseudomonadati Myxococcota Polyangia Polyangiales Polyangiales bacterium HEX2675071.1 314 aa
Pseudomonadati Myxococcota Polyangia Haliangiales Kofleriaceae bacterium isolate CA 2019 TPBS 15 MGE0868704.1
Pseudomonadati Myxococcota Myxococcia Myxococcales Myxococcales bacterium isolate Cli 2 MCP4447816.1
Pseudomonadati Myxococcota Myxococcia Myxococcales Myxococcales bacterium isolate HKST UBA02 MCA9670546.1
Pseudomonadati Myxococcota unclassified Myxococcota Myxococcota bacterium isolate Old saline rock biofilm.mb.17 MBU1240022.1
Pseudomonadati Myxococcota unclassified Myxococcota Myxococcota bacterium isolate Old saline rock biofilm.mb.11 MBU1244581.1
Pseudomonadati Myxococcota unclassified Myxococcota Myxococcota bacterium isolate Old saline rock biofilm.mb.24 MBU1218934.1
Pseudomonadati Myxococcota Myxococcia Myxococcales Myxococcales bacterium FL481 TPV94203.1
Pseudomonadati Myxococcota Myxococcota Polyangia Nannocystaceae bacterium MEE9386612.1 315 aa
Pseudomonadati Myxococcota Polyangia Nannocystales Nannocystaceae bacterium isolate ABC2.134 MEZ4429995.1 ClassIV
Pseudomonadati Myxococcota Myxococcia Myxococcales Myxococcales bacterium isolate HKST UBA11 MCA9659964.1
Pseudomonadati Myxococcota Polyangia Nannocystales Nannocystis sp. GCF 016717175.1 WP 293248010.1 ClassIV
Pseudomonadati Myxococcota Myxococcota Myxococcia Paraliomyxa miuraensis WP 407674744.1 311 aa
Pseudomonadati Myxococcota Myxococcota Polyangia Nannocystaceae bacterium MFV8751234.1 315 aa
Pseudomonadati Myxococcota Myxococcota Polyangia Enhygromyxa salina WP 106394539.1 315 aa
Pseudomonadati Myxococcota Myxococcota Polyangia Pseudenhygromyxa sp. WP 172303792.1 315 aa
Pseudomonadati Myxococcota Myxococcota Polyangia Plesiocystis pacifica WP 045116750.1 315 aa
Pseudomonadati Myxococcota Polyangia unclassified Polyangia Polyangia bacterium isolate SMAG U6843 HEX2656642.1
Pseudomonadati Myxococcota Polyangia unclassified Polyangia Polyangia bacterium isolate SMAG U7798 HEY5452288.1
Pseudomonadati Myxococcota Myxococcia Myxococcales Myxococcales bacterium isolate BP 637 MEA2697385.1
Pseudomonadati Myxococcota Polyangia unclassified Polyangia Polyangia bacterium isolate SMAG U3583 HEY0713696.1
Pseudomonadati Myxococcota Myxococcia Myxococcales Myxococcales bacterium isolate WC205a MCG5053524.1
Pseudomonadati Myxococcota Polyangia unclassified Polyangia Polyangia bacterium isolate SMAG U8418 HJX66639.1
Pseudomonadati Myxococcota Polyangia unclassified Polyangia Polyangia bacterium isolate SMAG U3009 HEX7506436.1
Pseudomonadati Myxococcota Polyangia unclassified Polyangia Polyangia bacterium MGD0837144.1 314 aa
Pseudomonadati Myxococcota Myxococcia Myxococcales Myxococcales bacterium isolate Baikal deep G132 MSP15774.1
Pseudomonadati Myxococcota Polyangia Haliangiales Haliangiales bacterium HKA88141.1 312 aa
Pseudomonadati Myxococcota Polyangia unclassified Polyangia Polyangia bacterium isolate SMAG U4743 HEY3354092.1
Pseudomonadati Myxococcota Polyangia unclassified Polyangia Polyangia bacterium isolate SMAG U6837 HEX2568911.1
Pseudomonadati Myxococcota Polyangia Polyangiales Polyangiaceae bacterium isolate SMAG U9917 HZU84456.1
Pseudomonadati Myxococcota Myxococcia Myxococcales Myxococcales bacterium isolate Baikal deep G131 MSP62291.1
Pseudomonadati Myxococcota Polyangia unclassified Polyangia Polyangia bacterium isolate cluster2 bin.158 MEO6952119.1
Pseudomonadati Myxococcota Polyangia unclassified Polyangia Polyangia bacterium HEY2749096.1 314 aa
Pseudomonadati Myxococcota Polyangia unclassified Polyangia Polyangia bacterium isolate SMAG U9996 HZS42004.1
Pseudomonadati Myxococcota Polyangia unclassified Polyangia Polyangia bacterium isolate GcA5 28 MFI5288135.1
Pseudomonadati Myxococcota unclassified Myxococcota Myxococcota bacterium isolate SKYG55 MCS6912955.1
Pseudomonadati Myxococcota Myxococcia Myxococcales Myxococcales bacterium isolate new MAG 82 MBL9005339.1
Pseudomonadati Myxococcota Polyangia unclassified Polyangia Polyangia bacterium isolate STL MYX6 MFO0623654.1
Pseudomonadati Myxococcota Polyangia unclassified Polyangia Polyangia bacterium isolate STL MYX5 MFO0574070.1
Pseudomonadati Myxococcota Polyangia Polyangiales Polyangiaceae bacterium isolate SJ398 MCC6646511.1
Pseudomonadati Myxococcota Polyangia Polyangiales Polyangiales bacterium isolate NVS3 Bin10 GAC1538296.1
Pseudomonadati Myxococcota Polyangia Polyangiales Polyangiales bacterium isolate NVS3 Bin20 GAC1579730.1
Pseudomonadati Myxococcota Polyangia Polyangiales Polyangiales bacterium isolate SJ.T15 3 bin.59 MGZ3418851.1
Pseudomonadati Myxococcota Myxococcia Myxococcales Myxococcales bacterium isolate EsbW 18 Q3 R4 48 BATAC.438 MBK7396746.1
Pseudomonadati Myxococcota Myxococcia Myxococcales Myxococcales bacterium isolate ELA212 MEI7894787.1
Pseudomonadati Myxococcota Polyangia Polyangiales Polyangiaceae bacterium isolate TC S concoct.bin.372 MGH7436715.1
Pseudomonadati Myxococcota Polyangia Polyangiales Polyangiaceae bacterium isolate SPRUCE 643 MGA2450221.1
Pseudomonadati Myxococcota Polyangia Polyangiales Polyangiaceae bacterium isolate Emb289P3bin120 MCL2448529.1
Pseudomonadati Myxococcota Polyangia Polyangiales Polyangiaceae bacterium isolate MFD04780 ilm asm bin14 MGA7121173.1
Pseudomonadati Myxococcota Polyangia Polyangiales Polyangiaceae bacterium isolate SMAG U4111 HEY8040585.1
Pseudomonadati Myxococcota Polyangia Polyangiales Polyangiaceae bacterium isolate SMAG U15146 HTQ46015.1
Pseudomonadati Myxococcota Polyangia Polyangiales Polyangiaceae bacterium isolate P RSF IL 17 NOU33502.1
Pseudomonadati Myxococcota Polyangia Polyangiales Polyangiaceae bacterium isolate Gw SIAct bin 374 MBP9113333.1
Pseudomonadati Myxococcota Polyangia Polyangiales Polyangiaceae bacterium isolate STL MYX19 MFO0672314.1
Pseudomonadati Myxococcota Myxococcia Myxococcales Myxococcales bacterium isolate Aved 18 Q3 R54 62 BATAC.150 MBK6690914.1
Pseudomonadati Myxococcota Polyangia Polyangiales Polyangiaceae bacterium isolate SMAG U15880 HSQ67099.1
Pseudomonadati Myxococcota Polyangia Polyangiales Polyangiaceae bacterium HEX7601510.1 314 aa
Pseudomonadati Myxococcota Myxococcia Myxococcales Pendulispora albinea WP 394820626.1 314 aa
Pseudomonadati Myxococcota Polyangia Polyangiales Polyangiaceae bacterium isolate SMAG U3075 HEX7663653.1
Pseudomonadati Myxococcota Myxococcia Myxococcales Myxococcaceae bacterium isolate RYN 371 MDB4997275.1
Pseudomonadati Myxococcota Polyangia Polyangiales Polyangiaceae bacterium HEY2512228.1 314 aa
Pseudomonadati Myxococcota Polyangia Polyangiales Pajaroellobacter abortibovis WP 075276977.1 313 aa
Pseudomonadati Myxococcota Myxococcia Myxococcales Myxococcales bacterium isolate Fred 18 Q3 R57 64 MAXAC.347 MBK8215807.1
Pseudomonadati Myxococcota Myxococcia Myxococcales Myxococcales bacterium MBL8610771.1 313 aa
Pseudomonadati Myxococcota Polyangia Polyangiales Polyangiaceae bacterium isolate SMAG U12425 HMI84235.1
Pseudomonadati Myxococcota Polyangia Polyangiales Polyangiaceae bacterium isolate AMR MDS 4925 HQP36851.1
Pseudomonadati Myxococcota Polyangia Polyangiales Polyangiaceae bacterium isolate AMR MDS 4678 HQK19146.1
Pseudomonadati Myxococcota Polyangia Polyangiales Polyangiaceae bacterium isolate AMR MDS 2340 HOT11195.1
Pseudomonadati Myxococcota Polyangia Polyangiales Polyangiaceae bacterium isolate SJ101 MCC6522180.1
Pseudomonadati Myxococcota Polyangia Polyangiales Polyangiaceae bacterium isolate SMAG U10103 HZO14622.1
Pseudomonadati Myxococcota Myxococcia Myxococcales Myxococcales bacterium isolate Baikal deep G134 MSP23638.1
Pseudomonadati Myxococcota Polyangia Polyangiales Polyangiaceae bacterium isolate OC3 3000m MAG 117 MFP6687028.1
Pseudomonadati Myxococcota unclassified Myxococcota Myxococcota bacterium isolate P01 H01 bin.82 MEM1033268.1
Pseudomonadati Myxococcota Myxococcia Myxococcales Myxococcales bacterium isolate HKST UBA17 MCA9618459.1
Pseudomonadati Myxococcota Polyangia Polyangiales Polyangiaceae bacterium isolate SMAG U10708 HLM74691.1
Pseudomonadati Myxococcota Polyangia Polyangiales Polyangiaceae bacterium isolate RUP SRG 27 MEJ7730158.1
Pseudomonadati Myxococcota Polyangia Polyangiales Sorangium cellulosum WP 020740020.1 320 aa
Pseudomonadati Myxococcota Polyangia Polyangiales Polyangiaceae bacterium isolate ABC1.6 MEZ4302249.1
Pseudomonadati Myxococcota Polyangia Polyangiales Polyangiaceae bacterium isolate STL MYX16 MFO0616748.1
Pseudomonadati Myxococcota Polyangia Polyangiales Polyangiaceae bacterium isolate Bin8.16 NUP06317.1
Pseudomonadati Myxococcota Polyangia Polyangiales Polyangiaceae bacterium isolate SMAG U6929 HEX2880918.1
Pseudomonadati Myxococcota Polyangia Polyangiales Polyangiaceae bacterium isolate SMAG U15437 HTM43754.1
Pseudomonadati Myxococcota Polyangia Polyangiales Polyangiaceae bacterium isolate SMAG U6865 HEX2735579.1
Pseudomonadati Myxococcota Polyangia Polyangiales Polyangiaceae bacterium isolate SMAG U6038 HVZ35387.1
Pseudomonadati Myxococcota Polyangia Polyangiales Polyangiaceae bacterium isolate Zod Metabat.1029 MBN2197477.1
Pseudomonadati Myxococcota Polyangia Polyangiales Polyangiaceae bacterium isolate SMAG U7278 HEY8944662.1
Pseudomonadati Myxococcota unclassified Myxococcota Myxococcota bacterium isolate KUW bin26 MDQ2644559.1
Pseudomonadati Myxococcota Polyangia Polyangiales Polyangiaceae bacterium isolate SMAG U10356 HLV64725.1
Pseudomonadati Myxococcota Polyangia Polyangiales Polyangiaceae bacterium isolate bins.307 MDX2054327.1
Pseudomonadati Myxococcota Polyangia Polyangiales Polyangiaceae bacterium isolate SMAG U4704 HEY3237600.1
Pseudomonadati Myxococcota Polyangia Polyangiales Polyangiaceae bacterium isolate SMAG U1768 HEX6272848.1
Pseudomonadati Myxococcota Polyangia Polyangiales Polyangiaceae bacterium isolate SMAG U4764 HEY3493674.1
Pseudomonadati Myxococcota Polyangia Polyangiales Polyangiaceae bacterium isolate SMAG U15136 HTQ07072.1
Pseudomonadati Myxococcota Polyangia Polyangiales Polyangiaceae bacterium isolate SMAG U333 HEY6555714.1
Pseudomonadati Myxococcota Polyangia Polyangiales Polyangiaceae bacterium isolate SMAG U13667 HVJ14786.1
Pseudomonadati Myxococcota Polyangia Polyangiales Polyangiaceae bacterium isolate ACE MYX3 MBX3129423.1
Pseudomonadati Myxococcota Polyangia Polyangiales Polyangiaceae bacterium isolate BC1.163 MEZ4224899.1
Pseudomonadati Myxococcota Polyangia Polyangiales Polyangiaceae bacterium isolate SMAG U11695 HYP89366.1
Pseudomonadati Myxococcota Polyangia Polyangiales Polyangiaceae bacterium isolate SMAG U2427 HEU5075235.1
Pseudomonadati Myxococcota Polyangia Polyangiales Polyangiaceae bacterium isolate SMAG U16345 HSC87734.1
Pseudomonadati Myxococcota Polyangia Polyangiales Polyangiaceae bacterium isolate SMAG U11326 HXS17077.1
Pseudomonadati Myxococcota Myxococcia Myxococcales Sorangiineae bacterium OQX68934.1 315 aa
Pseudomonadati Myxococcota Myxococcia Myxococcales Myxococcales bacterium isolate HKST UBA16 MCA9627152.1
Pseudomonadati Myxococcota Polyangia Polyangiales Polyangiaceae bacterium isolate SMAG U10369 HLV21799.1
Pseudomonadati Myxococcota Polyangia Polyangiales Polyangiaceae bacterium isolate SMAG U5056 HEY1532734.1
Pseudomonadati Myxococcota Polyangia Polyangiales Polyangiaceae bacterium isolate SJ329 MCC6214315.1
Pseudomonadati Myxococcota Polyangia Polyangiales Polyangiaceae bacterium isolate SMAG U9664 HMA94320.1
Pseudomonadati Myxococcota Polyangia Polyangiales Polyangiaceae bacterium isolate DT 87 NRA32615.1
Pseudomonadati Myxococcota Polyangia Polyangiales Polyangiaceae bacterium isolate SMAG U5750 HEX4475673.1
Pseudomonadati Myxococcota Polyangia Polyangiales Polyangiaceae bacterium HVW29377.1 315 aa
Bacteria incertae sedis Candidatus Binatota Candidatus Binatia unclassified Candidatus Binatia Candidatus Binatia bacterium isolate MT6bin.1 MEE9145757.1
Bacteria incertae sedis Candidatus Binatota Candidatus Binatia unclassified Candidatus Binatia Candidatus Binatia bacterium isolate KW01 5 MFB3061383.1
Bacteria incertae sedis Candidatus Binatota Candidatus Binatia unclassified Candidatus Binatia Candidatus Binatia bacterium isolate Arabian11 MAG 62 MDP6559706.1
Bacteria incertae sedis Candidatus Binatota Candidatus Binatia unclassified Candidatus Binatia Candidatus Binatia bacterium isolate K 2 bin.72 MFQ5849834.1
Bacteria incertae sedis Candidatus Binatota Candidatus Binatia unclassified Candidatus Binatia Candidatus Binatia bacterium isolate KW56 17 MFB3121750.1
Bacteria incertae sedis Candidatus Binatota Candidatus Binatia unclassified Candidatus Binatia Candidatus Binatia bacterium isolate FS 3 bin.33 MFQ5916362.1
Bacteria incertae sedis Candidatus Binatota Candidatus Binatia unclassified Candidatus Binatia Candidatus Binatia bacterium isolate M3 S concoct.bin.81 sub MGH7831545.1
Bacteria incertae sedis Candidatus Binatota Candidatus Binatia unclassified Candidatus Binatia Candidatus Binatia bacterium isolate SMAG U4633 HEY2988971.1
Bacteria incertae sedis Candidatus Binatota Candidatus Binatia unclassified Candidatus Binatia Candidatus Binatia bacterium isolate M3 S metawrap bin.17 iso1 MGH7833713.1
Bacteria incertae sedis Candidatus Binatota Candidatus Binatia unclassified Candidatus Binatia Candidatus Binatia bacterium isolate SMAG U4512 HEV8340911.1
Bacteria incertae sedis Candidatus Binatota Candidatus Binatia unclassified Candidatus Binatia Candidatus Binatia bacterium isolate M3 S metawrap bin.153 MGH7775330.1
Bacteria incertae sedis Candidatus Binatota Candidatus Binatia unclassified Candidatus Binatia Candidatus Binatia bacterium isolate SMAG U506 HET8563203.1
Bacteria incertae sedis Candidatus Binatota Candidatus Binatia unclassified Candidatus Binatia Candidatus Binatia bacterium isolate SMAG U2161 iso1 HEU4341156.1
Bacteria incertae sedis Candidatus Binatota Candidatus Binatia unclassified Candidatus Binatia Candidatus Binatia bacterium isolate SMAG U6753 HEX2261025.1
Bacteria incertae sedis Candidatus Binatota Candidatus Binatia unclassified Candidatus Binatia Candidatus Binatia bacterium isolate SMAG U6728 HEX2228921.1
Bacteria incertae sedis Candidatus Binatota Candidatus Binatia unclassified Candidatus Binatia Candidatus Binatia bacterium isolate SMAG U2234 HEU4639080.1
Bacteria incertae sedis Candidatus Binatota Candidatus Binatia unclassified Candidatus Binatia Candidatus Binatia bacterium isolate SMAG U3798 HEY7165614.1
Bacteria incertae sedis Candidatus Binatota Candidatus Binatia unclassified Candidatus Binatia Candidatus Binatia bacterium isolate SMAG U1930 HEX6801391.1
Bacteria incertae sedis Candidatus Binatota Candidatus Binatia unclassified Candidatus Binatia Candidatus Binatia bacterium isolate bin1037 MGN6716277.1
Bacteria incertae sedis Candidatus Binatota Candidatus Binatia unclassified Candidatus Binatia Candidatus Binatia bacterium isolate SMAG U4003 iso2 HEY7715091.1
Bacteria incertae sedis Candidatus Binatota Candidatus Binatia unclassified Candidatus Binatia Candidatus Binatia bacterium isolate SMAG U2541 HEU5464376.1
Bacteria incertae sedis Candidatus Binatota Candidatus Binatia unclassified Candidatus Binatia Candidatus Binatia bacterium isolate cluster1 bin.7 MDG1960185.1
Bacteria incertae sedis Candidatus Binatota Candidatus Binatia unclassified Candidatus Binatia Candidatus Binatia bacterium isolate cluster2 bin.46 MDG2306784.1
Bacteria incertae sedis Candidatus Binatota Candidatus Binatia unclassified Candidatus Binatia Candidatus Binatia bacterium isolate SMAG U10020 iso2 HZR80604.1
Bacteria incertae sedis Candidatus Binatota Candidatus Binatia unclassified Candidatus Binatia Candidatus Binatia bacterium isolate SMAG U7137 HEY8516360.1
Bacteria incertae sedis Candidatus Binatota Candidatus Binatia unclassified Candidatus Binatia Candidatus Binatia bacterium isolate M3 B2 maxbin2.bin.72 sub MGH7965174.1
Bacteria incertae sedis Candidatus Binatota Candidatus Binatia unclassified Candidatus Binatia Candidatus Binatia bacterium isolate M3 S concoct.bin.50 sub MGH7819035.1
Bacteria incertae sedis Candidatus Binatota Candidatus Binatia unclassified Candidatus Binatia Candidatus Binatia bacterium isolate SMAG U16327 HSD09583.1
Bacteria incertae sedis Candidatus Binatota Candidatus Binatia unclassified Candidatus Binatia Candidatus Binatia bacterium isolate M3 S metabat2.bin.229 MGH7896525.1
Bacteria incertae sedis Candidatus Binatota Candidatus Binatia unclassified Candidatus Binatia Candidatus Binatia bacterium isolate S.4.sp2 bin.36 MFQ5477964.1
Bacteria incertae sedis Candidatus Binatota Candidatus Binatia unclassified Candidatus Binatia Candidatus Binatia bacterium isolate S6 bin.16.strict MFN2427361.1
Bacteria incertae sedis Candidatus Binatota Candidatus Binatia unclassified Candidatus Binatia Candidatus Binatia bacterium isolate 3300053785 131 MFT4569188.1
Bacteria incertae sedis Candidatus Binatota Candidatus Binatia unclassified Candidatus Binatia Candidatus Binatia bacterium isolate SMAG U14709 iso2 HVN84597.1
Bacteria incertae sedis Candidatus Binatota Candidatus Binatia unclassified Candidatus Binatia Candidatus Binatia bacterium isolate KatS3mg076 GIW40487.1
Bacteria incertae sedis Candidatus Binatota Candidatus Binatia unclassified Candidatus Binatia Candidatus Binatia bacterium isolate SKW5 iso2 MCX8073433.1
Bacteria incertae sedis Candidatus Binatota Candidatus Binatia unclassified Candidatus Binatia Candidatus Binatia bacterium isolate MAG.286 iso1 MCK6554550.1
Bacteria incertae sedis Candidatus Binatota Candidatus Binatia unclassified Candidatus Binatia Candidatus Binatia bacterium isolate Maj 2 bin.38 MFQ5667265.1
Bacteria incertae sedis Candidatus Binatota Candidatus Binatia unclassified Candidatus Binatia Candidatus Binatia bacterium isolate GcC5 36 MFI5364996.1
Bacteria incertae sedis Candidatus Binatota Candidatus Binatia unclassified Candidatus Binatia Candidatus Binatia bacterium isolate STL DESB1 MFN8627888.1
Bacteria incertae sedis Candidatus Binatota Candidatus Binatia unclassified Candidatus Binatia Candidatus Binatia bacterium isolate SMAG U2977 HEX7406060.1
Bacteria incertae sedis Candidatus Binatota Candidatus Binatia unclassified Candidatus Binatia Candidatus Binatia bacterium isolate STL DESB4 iso1 MFN8543174.1
Bacteria incertae sedis Candidatus Binatota Candidatus Binatia unclassified Candidatus Binatia Candidatus Binatia bacterium isolate SMAG U10078 iso3 HZP43025.1
Bacteria incertae sedis Candidatus Binatota Candidatus Binatia Candidatus Binatales Candidatus Binatus sp. isolate SMAG U15749 HSV08826.1
Bacteria incertae sedis Candidatus Binatota Candidatus Binatia unclassified Candidatus Binatia Candidatus Binatia bacterium isolate SMAG U9820 HLK12043.1
Bacteria incertae sedis Candidatus Binatota Candidatus Binatia unclassified Candidatus Binatia Candidatus Binatia bacterium isolate SMAG U9481 HKA30118.1
Bacteria incertae sedis Candidatus Binatota Candidatus Binatia Candidatus Binatales Candidatus Binataceae bacterium isolate SMAG U5357 HEY2663519.1
Bacteria incertae sedis Candidatus Binatota Candidatus Binatia Candidatus Binatales Candidatus Binataceae bacterium isolate SMAG U11339 iso2 HXR37256.1
Bacteria incertae sedis Candidatus Binatota Candidatus Binatia Candidatus Binatales Candidatus Binataceae bacterium isolate SMAG U9884 iso2 HLH78365.1
Bacteria incertae sedis Candidatus Binatota Candidatus Binatia Candidatus Binatales Candidatus Binataceae bacterium isolate SMAG U13986 HVA68253.1
Bacteria incertae sedis Candidatus Binatota Candidatus Binatia Candidatus Binatales Candidatus Binataceae bacterium isolate SMAG U9827 HLJ44076.1
Bacteria incertae sedis Candidatus Binatota Candidatus Binatia Candidatus Binatales Candidatus Binataceae bacterium isolate SMAG U11341 HXR25231.1
Bacteria incertae sedis Candidatus Binatota Candidatus Binatia Candidatus Binatales Candidatus Binataceae bacterium isolate MAG011 MBF6561427.1

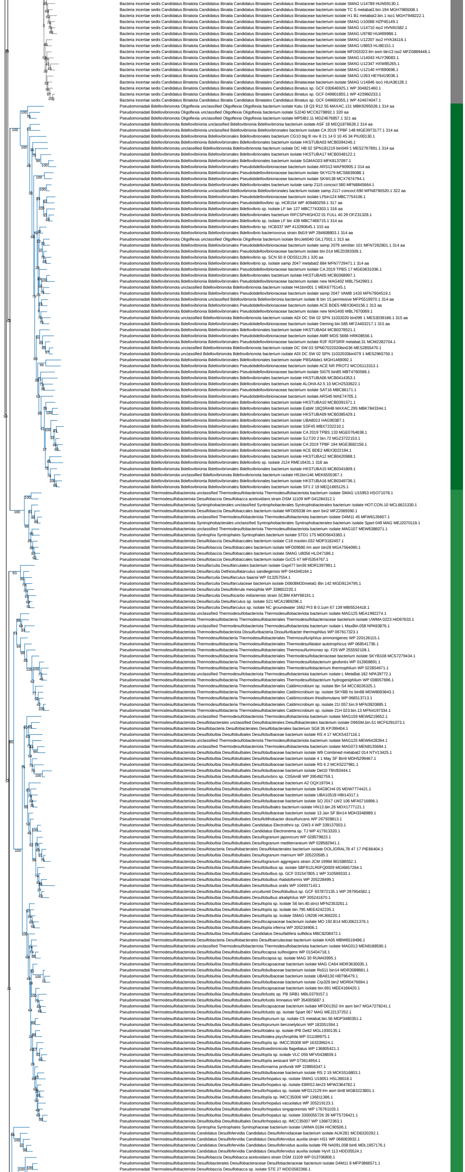

Bacteria incertae sedis Candidatus Binatota Candidatus Binatia Candidatus Binatales Candidatus Binataceae bacterium isolate SMAG U14789 HUN59130.1
Bacteria incertae sedis Candidatus Binatota Candidatus Binatia Candidatus Binatales Candidatus Binataceae bacterium isolate TC S metabat2.bin.194 MGH7985008.1
Bacteria incertae sedis Candidatus Binatota Candidatus Binatia Candidatus Binatales Candidatus Binataceae bacterium isolate H1 B1 metabat2.bin.1 iso1 MGH7948222.1
Bacteria incertae sedis Candidatus Binatota Candidatus Binatia Candidatus Binatales Candidatus Binataceae bacterium isolate SMAG U10068 HZP45149.1
Bacteria incertae sedis Candidatus Binatota Candidatus Binatia Candidatus Binatales Candidatus Binataceae bacterium isolate SMAG U14710 iso2 HVN91582.1
Bacteria incertae sedis Candidatus Binatota Candidatus Binatia Candidatus Binatales Candidatus Binataceae bacterium isolate SMAG U9780 HLW69986.1
Bacteria incertae sedis Candidatus Binatota Candidatus Binatia Candidatus Binatales Candidatus Binataceae bacterium isolate SMAG U12207 iso2 HYA34116.1
Bacteria incertae sedis Candidatus Binatota Candidatus Binatia Candidatus Binatales Candidatus Binataceae bacterium isolate SMAG U9853 HLI80151.1
Bacteria incertae sedis Candidatus Binatota Candidatus Binatia Candidatus Binatales Candidatus Binataceae bacterium isolate MFD03323 ilm asm bin13 iso2 MFZ0889448.1
Bacteria incertae sedis Candidatus Binatota Candidatus Binatia Candidatus Binatales Candidatus Binataceae bacterium isolate SMAG U14043 HUY39083.1
Bacteria incertae sedis Candidatus Binatota Candidatus Binatia Candidatus Binatales Candidatus Binataceae bacterium isolate SMAG U12347 HXW85265.1
Bacteria incertae sedis Candidatus Binatota Candidatus Binatia Candidatus Binatales Candidatus Binataceae bacterium isolate SMAG U12140 HYB90696.1
Bacteria incertae sedis Candidatus Binatota Candidatus Binatia Candidatus Binatales Candidatus Binataceae bacterium isolate SMAG U263 HEY6419036.1
Bacteria incertae sedis Candidatus Binatota Candidatus Binatia Candidatus Binatales Candidatus Binataceae bacterium isolate SMAG U14846 iso1 HUA36128.1
Bacteria incertae sedis Candidatus Binatota Candidatus Binatia Candidatus Binatales Candidatus Binatus sp. GCF 030646925.1 WP 304821460.1
Bacteria incertae sedis Candidatus Binatota Candidatus Binatia Candidatus Binatales Candidatus Binatus sp. GCF 049801855.1 WP 423960233.1
Bacteria incertae sedis Candidatus Binatota Candidatus Binatia Candidatus Binatales Candidatus Binatus sp. GCF 049692055.1 WP 424674047.1
Pseudomonadati Bdellovibrionota Oligoflexia unclassified Oligoflexia Oligoflexia bacterium isolate Kalu 18 Q3 R12 55 MAXAC.131 MBK9295028.1 314 aa
Pseudomonadati Bdellovibrionota Oligoflexia unclassified Oligoflexia Oligoflexia bacterium isolate SJ240 MCC6278892.1 320 aa
Pseudomonadati Bdellovibrionota Oligoflexia unclassified Oligoflexia Oligoflexia bacterium isolate MP5IB2.11 MDZ4676857.1 321 aa
Pseudomonadati Bdellovibrionota Bdellovibrionia unclassified Bdellovibrionia Bdellovibrionia bacterium isolate ASF 18 MEQ1876628.1 314 aa
Pseudomonadati Bdellovibrionota Bdellovibrionia Bdellovibrionales unclassified Bdellovibrionales Bdellovibrionales bacterium isolate CA 2019 TPBF 148 MGE3973177.1 314 aa
Pseudomonadati Bdellovibrionota Bdellovibrionia Bdellovibrionales Bdellovibrionales bacterium CG10 big fil rev 8 21 14 0 10 45 34 PIU00130.1
Pseudomonadati Bdellovibrionota Bdellovibrionia Bdellovibrionales Bdellovibrionales bacterium isolate HKSTUBA03 MCB0394246.1
Pseudomonadati Bdellovibrionota unclassified Bdellovibrionota Bdellovibrionota bacterium isolate DC HB 02 SPN181219 bin049 1 MES2767891.1 314 aa
Pseudomonadati Bdellovibrionota Bdellovibrionia Bdellovibrionales Bdellovibrionales bacterium isolate HKSTUBA17 MCB0348122.1
Pseudomonadati Bdellovibrionota Bdellovibrionia Bdellovibrionales Bdellovibrionales bacterium isolate SGMAG03 MFK8137097.1
Pseudomonadati Bdellovibrionota Bdellovibrionia Bdellovibrionales Pseudobdellovibrionaceae bacterium isolate ARS13 MAF90905.1 314 aa
Pseudomonadati Bdellovibrionota Bdellovibrionia Bdellovibrionales Pseudobdellovibrionaceae bacterium isolate SKYG79 MCS6839088.1
Pseudomonadati Bdellovibrionota Bdellovibrionia Bdellovibrionales Pseudobdellovibrionaceae bacterium isolate SKW138 MCX7674794.1
Pseudomonadati Bdellovibrionota Bdellovibrionia Bdellovibrionales Bdellovibrionales bacterium isolate samp 2115 concoct 580 MFN8845664.1
Pseudomonadati Bdellovibrionota Bdellovibrionia unclassified Bdellovibrionia Bdellovibrionales bacterium isolate samp 2117 concoct 690 MFN8790520.1 322 aa
Pseudomonadati Bdellovibrionota Bdellovibrionia Bdellovibrionales Pseudobdellovibrionaceae bacterium isolate LFbin124 MBC7754106.1
Pseudomonadati Bdellovibrionota Bdellovibrionia Bdellovibrionales Pseudobdellovibrio sp. HCB154 WP 409480259.1 317 aa
Pseudomonadati Bdellovibrionota Bdellovibrionia Bdellovibrionales Bdellovibrio sp. isolate LF bin 127 MBC7743303.1 316 aa
Pseudomonadati Bdellovibrionota Bdellovibrionia Bdellovibrionales Bdellovibrionales bacterium RIFCSPHIGHO2 01 FULL 40 29 OFZ31328.1
Pseudomonadati Bdellovibrionota Bdellovibrionia Bdellovibrionales Bdellovibrio sp. isolate LF bin 439 MBC7466715.1 314 aa
Pseudomonadati Bdellovibrionota Bdellovibrionia Bdellovibrionales Bdellovibrio sp. HCB337 WP 413290645.1 315 aa
Pseudomonadati Bdellovibrionota Bdellovibrionia Bdellovibrionales Bdellovibrio bacteriovorus strain Bd19 WP 284608803.1 314 aa
Pseudomonadati Bdellovibrionota Oligoflexia unclassified Oligoflexia Oligoflexia bacterium isolate BroJett040 GIL17001.1 313 aa
Pseudomonadati Bdellovibrionota Bdellovibrionia Bdellovibrionales Pseudobdellovibrionaceae bacterium isolate samp 2079 semibin 101 MFN7262801.1 314 aa
Pseudomonadati Bdellovibrionota Bdellovibrionia Bdellovibrionales Pseudobdellovibrionaceae bacterium isolate bin.014 MEZ0393309.1
Pseudomonadati Bdellovibrionota Bdellovibrionia Bdellovibrionales Bdellovibrio sp. SCN 50 8 ODS51129.1 320 aa
Pseudomonadati Bdellovibrionota Bdellovibrionia Bdellovibrionales Bdellovibrio sp. isolate samp 2047 metabat2 884 MFN7729471.1 314 aa
Pseudomonadati Bdellovibrionota Bdellovibrionia Bdellovibrionales Pseudobdellovibrionaceae bacterium isolate CA 2019 TPBS 17 MGE0631036.1
Pseudomonadati Bdellovibrionota Bdellovibrionia Bdellovibrionales Bdellovibrionales bacterium isolate HKSTUBA05 MCB0368997.1
Pseudomonadati Bdellovibrionota Bdellovibrionia Bdellovibrionales Pseudobdellovibrionaceae bacterium isolate new MAG402 MBL7542993.1
Pseudomonadati Bdellovibrionota unclassified Bdellovibrionota Bdellovibrionota bacterium isolate H41bin001 1 MEK6775145.1
Pseudomonadati Bdellovibrionota Bdellovibrionia Bdellovibrionales Pseudobdellovibrionaceae bacterium isolate samp 2047 VAMB 1433 MFN7904519.1
Pseudomonadati Bdellovibrionota Bdellovibrionia unclassified Bdellovibrionia Bdellovibrionia bacterium isolate B bin 15 permissive MFP5519970.1 314 aa
Pseudomonadati Bdellovibrionota Bdellovibrionia Bdellovibrionales Pseudobdellovibrionaceae bacterium isolate ACE BDE5 MBX3040156.1 313 aa
Pseudomonadati Bdellovibrionota Bdellovibrionia Bdellovibrionales Pseudobdellovibrionaceae bacterium isolate new MAG400 MBL7670069.1
Pseudomonadati Bdellovibrionota unclassified Bdellovibrionota Bdellovibrionota bacterium isolate ADI DC SW 02 SPN 11032020 bin099 1 MES3039186.1 315 aa
Pseudomonadati Bdellovibrionota Bdellovibrionia Bdellovibrionales Pseudobdellovibrionaceae bacterium isolate Deming bin.585 MFZ4403217.1 315 aa
Pseudomonadati Bdellovibrionota Bdellovibrionia Bdellovibrionales Bdellovibrionales bacterium isolate HKSTUBA04 MCB0378521.1
Pseudomonadati Bdellovibrionota Bdellovibrionia Bdellovibrionales Pseudobdellovibrionaceae bacterium isolate AMR MDS 5666 HRK08556.1
Pseudomonadati Bdellovibrionota Bdellovibrionia Bdellovibrionales Pseudobdellovibrionaceae bacterium isolate R2F R2FSRR metabat.31 MCM2282704.1
Pseudomonadati Bdellovibrionota unclassified Bdellovibrionota Bdellovibrionota bacterium isolate DC SW 03 SPN07022020bin036 MES2855470.1
Pseudomonadati Bdellovibrionota unclassified Bdellovibrionota Bdellovibrionota bacterium isolate ADI DC SW 02 SPN 11032020bin079 1 MES2965750.1
Pseudomonadati Bdellovibrionota Bdellovibrionia Bdellovibrionales Bdellovibrionales bacterium isolate PBSAbde1 MGH1469092.1
Pseudomonadati Bdellovibrionota Bdellovibrionia Bdellovibrionales Pseudobdellovibrionaceae bacterium isolate ACE NR PROT2 MCO5113313.1
Pseudomonadati Bdellovibrionota Bdellovibrionia Bdellovibrionales Pseudobdellovibrionaceae bacterium isolate SI075 bin85 MBT4760588.1
Pseudomonadati Bdellovibrionota Bdellovibrionia Bdellovibrionales Bdellovibrionales bacterium isolate HKSTUBA06 MCB0414353.1
Pseudomonadati Bdellovibrionota Bdellovibrionia Bdellovibrionales Bdellovibrionales bacterium isolate ALOHA A2.5 10 MCH2533622.1
Pseudomonadati Bdellovibrionota Bdellovibrionia Bdellovibrionales Pseudobdellovibrionaceae bacterium isolate SAT16 MBC86171.1
Pseudomonadati Bdellovibrionota Bdellovibrionia Bdellovibrionales Pseudobdellovibrionaceae bacterium isolate ARS45 MAE74705.1
Pseudomonadati Bdellovibrionota Bdellovibrionia Bdellovibrionales Bdellovibrionales bacterium isolate HKSTUBA10 MCB0391571.1
Pseudomonadati Bdellovibrionota Bdellovibrionia Bdellovibrionales Bdellovibrionales bacterium isolate EsbW 18Q3R448 MAXAC.295 MBK7843344.1
Pseudomonadati Bdellovibrionota Bdellovibrionia Bdellovibrionales Bdellovibrionales bacterium isolate HKSTUBA09 MCB0385429.1
Pseudomonadati Bdellovibrionota Bdellovibrionia Bdellovibrionales Bdellovibrionales bacterium isolate UBA8013 HAG90387.1
Pseudomonadati Bdellovibrionota Bdellovibrionia Bdellovibrionales Bdellovibrionales bacterium isolate SSF45 MBX7232210.1
Pseudomonadati Bdellovibrionota Bdellovibrionia Bdellovibrionales Bdellovibrionales bacterium isolate CA 2019 TPBS 133 MGE0764638.1
Pseudomonadati Bdellovibrionota Bdellovibrionia Bdellovibrionales Bdellovibrionales bacterium isolate SJ.T20 2 bin.72 MGZ3722153.1
Pseudomonadati Bdellovibrionota Bdellovibrionia Bdellovibrionales Bdellovibrionales bacterium isolate CA 2019 TPBF 194 MGE3682156.1
Pseudomonadati Bdellovibrionota Bdellovibrionia Bdellovibrionales Bdellovibrionales bacterium isolate ACE BDE2 MBX3022194.1
Pseudomonadati Bdellovibrionota Bdellovibrionia Bdellovibrionales Bdellovibrionales bacterium isolate HKSTUBA12 MCB0420988.1
Pseudomonadati Bdellovibrionota Bdellovibrionia Bdellovibrionales Bdellovibrio sp. isolate J124 RME16431.1 318 aa
Pseudomonadati Bdellovibrionota Bdellovibrionia Bdellovibrionales Bdellovibrionales bacterium isolate HKSTUBA15 MCB0341809.1
Pseudomonadati Bdellovibrionota unclassified Bdellovibrionota Bdellovibrionota bacterium isolate H51bin146 MEK6555367.1
Pseudomonadati Bdellovibrionota Bdellovibrionia Bdellovibrionales Bdellovibrionales bacterium isolate HKSTUBA16 MCB0349736.1
Pseudomonadati Bdellovibrionota Bdellovibrionia Bdellovibrionales Bdellovibrionales bacterium isolate SF1 2 19 MEQ1665125.1
Pseudomonadati Thermodesulfobacteriota unclassified Thermodesulfobacteriota Thermodesulfobacteriota bacterium isolate SMAG U15953 HSO71078.1
Pseudomonadati Thermodesulfobacteriota Desulfobaccia Desulfobaccales Desulfobacca acetoxidans strain DSM 11109 WP 041284312.1
Pseudomonadati Thermodesulfobacteriota Syntrophobacteria Syntrophobacterales unclassified Syntrophobacterales Syntrophobacterales bacterium isolate HOT.CON.10 MCL6621330.1
Pseudomonadati Thermodesulfobacteriota Desulfobaccia Desulfobaccales Desulfobaccales bacterium isolate MFD09338 ilm asm bin2 MFZ2089390.1
Pseudomonadati Thermodesulfobacteriota unclassified Thermodesulfobacteriota Thermodesulfobacteriota bacterium isolate D4M11 45 MFW6126607.1
Pseudomonadati Thermodesulfobacteriota Syntrophobacteria Syntrophobacterales unclassified Syntrophobacterales Syntrophobacterales bacterium isolate Spart 049 MAG MEJ2070119.1
Pseudomonadati Thermodesulfobacteriota unclassified Thermodesulfobacteriota Thermodesulfobacteriota bacterium isolate MAG107 MEW6386071.1
Pseudomonadati Thermodesulfobacteriota Syntrophia Syntrophales Syntrophales bacterium isolate STD1 175 MDD5643383.1
Pseudomonadati Thermodesulfobacteriota Desulfobaccia Desulfobaccales Desulfobaccales bacterium isolate C18 maxbin.032 MDP3182457.1
Pseudomonadati Thermodesulfobacteriota Desulfobaccia Desulfobaccales Desulfobaccales bacterium isolate MFD09680 ilm asm bin28 MGA7564090.1
Pseudomonadati Thermodesulfobacteriota Desulfobaccia Desulfobaccales Desulfobaccales bacterium isolate SMAG U8056 HLD47186.1
Pseudomonadati Thermodesulfobacteriota Desulfobaccia Desulfobaccales Desulfobaccales bacterium isolate GcC5 47 MFI5354767.1
Pseudomonadati Thermodesulfobacteriota Desulfarculia Desulfarculales Desulfarculales bacterium isolate Gsp477 bin36 MDR1397991.1
Pseudomonadati Thermodesulfobacteriota Desulfarculia Desulfarculales Dethiosulfatarculus sandiegensis WP 044346164.1
Pseudomonadati Thermodesulfobacteriota Desulfarculia Desulfarculales Desulfarculus baarsii WP 013257554.1
Pseudomonadati Thermodesulfobacteriota Desulfarculia Desulfarculales Desulfarculaceae bacterium isolate D0608M20metaG Bin 142 MGD9124795.1
Pseudomonadati Thermodesulfobacteriota Desulfarculia Desulfarculales Desulfoferula mesophila WP 338602220.1
Pseudomonadati Thermodesulfobacteriota Desulfarculia Desulfarculales Desulfocarbo indianensis strain SCBM KMY68191.1
Pseudomonadati Thermodesulfobacteriota Desulfarculia Desulfarculales Desulfarculus sp. isolate S21 MCA1989296.1
Pseudomonadati Thermodesulfobacteriota Desulfarculia Desulfarculales Desulfarculus sp. isolate NC groundwater 1662 Pr3 B 0.1um 67 139 MBI5524418.1
Pseudomonadati Thermodesulfobacteriota unclassified Thermodesulfobacteriota Thermodesulfobacteriota bacterium isolate MAG125 MEA1992274.1
Pseudomonadati Thermodesulfobacteriota Thermodesulfobacteria Thermodesulfobacteriales Thermodesulfobacteriaceae bacterium isolate UWMA 0223 HID97633.1
Pseudomonadati Thermodesulfobacteriota unclassified Thermodesulfobacteriota Thermodesulfobacteriota bacterium isolate L MaxBin.058 NPA93876.1
Pseudomonadati Thermodesulfobacteriota Thermodesulfobacteriota Dissulfuribacteria Dissulfuribacter thermophilus WP 067617323.1
Pseudomonadati Thermodesulfobacteriota Thermodesulfobacteria Thermodesulfobacteriales Thermosulfuriphilus ammonigenes WP 220126115.1
Pseudomonadati Thermodesulfobacteriota Thermodesulfobacteria Thermodesulfobacteriales Thermodesulfatator autotrophicus WP 068541736.1
Pseudomonadati Thermodesulfobacteriota Thermodesulfobacteria Thermodesulfobacteriales Thermosulfurimonas sp. F29 WP 255592108.1
Pseudomonadati Thermodesulfobacteriota Thermodesulfobacteria Thermodesulfobacteriales Thermodesulfobacteriaceae bacterium isolate SKYB108 MCS7279434.1
Pseudomonadati Thermodesulfobacteriota Thermodesulfobacteria Thermodesulfobacteriales Thermodesulfobacterium geofontis WP 013908891.1
Pseudomonadati Thermodesulfobacteriota Thermodesulfobacteria Thermodesulfobacteriales Thermodesulfobacterium thermophilum WP 022854671.1
Pseudomonadati Thermodesulfobacteriota unclassified Thermodesulfobacteriota Thermodesulfobacteriota bacterium isolate L MetaBat.182 NPA39772.1
Pseudomonadati Thermodesulfobacteriota Thermodesulfobacteria Thermodesulfobacteriales Thermodesulfobacterium hydrogeniphilum WP 038057696.1
Pseudomonadati Thermodesulfobacteriota Thermodesulfobacteria Thermodesulfobacteriales Caldimicrobium sp. isolate Bin S4 MCC6026325.1
Pseudomonadati Thermodesulfobacteriota Thermodesulfobacteria Thermodesulfobacteriales Caldimicrobium sp. isolate SKYBB hs bin68 MDW8093843.1
Pseudomonadati Thermodesulfobacteriota Thermodesulfobacteria Thermodesulfobacteriales Caldimicrobium thiodismutans WP 068513713.1
Pseudomonadati Thermodesulfobacteriota Thermodesulfobacteria Thermodesulfobacteriales Caldimicrobium sp. isolate 21I 057.bin.9 MFN3920885.1
Pseudomonadati Thermodesulfobacteriota Thermodesulfobacteria Thermodesulfobacteriales Caldimicrobium sp. isolate 21H 023.bin.13 MFN4197334.1
Pseudomonadati Thermodesulfobacteriota unclassified Thermodesulfobacteriota Thermodesulfobacteriota bacterium isolate MAG159 MEW6219652.1
Pseudomonadati Thermodesulfobacteriota Desulfobacteria Desulfobacterales unclassified Desulfobacterales Desulfobacterales bacterium isolate D96SM.bin.51 MCF6291073.1
Pseudomonadati Thermodesulfobacteriota Desulfobacteria Desulfobacterales Desulfobacterales bacterium SG8 35 KPJ99404.1
Pseudomonadati Thermodesulfobacteriota Desulfobulbia Desulfobulbales Desulfobulbaceae bacterium isolate RS 4 17 MCK5437116.1
Pseudomonadati Thermodesulfobacteriota unclassified Thermodesulfobacteriota Thermodesulfobacteriota bacterium isolate MAG125 MEW6428394.1
Pseudomonadati Thermodesulfobacteriota unclassified Thermodesulfobacteriota Thermodesulfobacteriota bacterium isolate MAG073 MEN8135684.1
Pseudomonadati Thermodesulfobacteriota Desulfobulbia Desulfobulbales Desulfobulbaceae bacterium isolate W9 Combined metabat2 014 NTV13425.1
Pseudomonadati Thermodesulfobacteriota Desulfobulbia Desulfobulbales Desulfobulbaceae bacterium isolate 4 1 May SF Bin9 MDH5299467.1
Pseudomonadati Thermodesulfobacteriota Desulfobulbia Desulfobulbales Desulfobulbaceae bacterium isolate RS 6 2 MCK5227981.1
Pseudomonadati Thermodesulfobacteriota Desulfobulbia Desulfobulbales Desulfobulbaceae bacterium isolate Del10 TBV83444.1
Pseudomonadati Thermodesulfobacteriota Desulfobulbia Desulfobulbales Desulfurivibrio sp. C05AmB WP 395492759.1
Pseudomonadati Thermodesulfobacteriota Desulfobulbia Desulfobulbales Desulfobulbaceae bacterium A2 OQX19704.1
Pseudomonadati Thermodesulfobacteriota Desulfobulbia Desulfobulbales Desulfobulbaceae bacterium isolate B4G8CH4 05 MDW7774421.1
Pseudomonadati Thermodesulfobacteriota Desulfobulbia Desulfobulbales Desulfobulbaceae bacterium isolate UBA10518 HBI14317.1
Pseudomonadati Thermodesulfobacteriota Desulfobulbia Desulfobulbales Desulfobulbaceae bacterium isolate SO 2017 LW2 106 MFA5716896.1
Pseudomonadati Thermodesulfobacteriota Desulfobulbia Desulfobulbales Desulfobulbales bacterium isolate HN13.bin.26 MDX1777121.1
Pseudomonadati Thermodesulfobacteriota Desulfobulbia Desulfobulbales Desulfobulbaceae bacterium isolate 13 Jan SF Bin14 MDH3348989.1
Pseudomonadati Thermodesulfobacteriota Desulfobulbia Desulfobulbales Desulfolithobacter dissulfuricans WP 267928813.1
Pseudomonadati Thermodesulfobacteriota Desulfobulbia Desulfobulbales Candidatus Electrothrix sp. GW3 4 WP 339137003.1
Pseudomonadati Thermodesulfobacteriota Desulfobulbia Desulfobulbales Candidatus Electronema sp. TJ WP 417913320.1
Pseudomonadati Thermodesulfobacteriota Desulfobulbia Desulfobulbales Desulfogranum japonicum WP 028579823.1
Pseudomonadati Thermodesulfobacteriota Desulfobulbia Desulfobulbales Desulfogranum mediterraneum WP 028582941.1
Pseudomonadati Thermodesulfobacteriota Desulfobacteria Desulfobacterales Desulfobacterales bacterium isolate DOLJORAL78 47 17 PIE66404.1
Pseudomonadati Thermodesulfobacteriota Desulfobulbia Desulfobulbales Desulfogranum marinum WP 205220585.1
Pseudomonadati Thermodesulfobacteriota Desulfobulbia Desulfobulbales Desulfogranum aggregans strain JCM 19994 BGS86552.1
Pseudomonadati Thermodesulfobacteriota Desulfobulbia Desulfobulbales Desulfobulbus sp. isolate SBF912LR0FQ0009 MGI6657264.1
Pseudomonadati Thermodesulfobacteriota Desulfobulbia Desulfobulbales Desulfobulbus sp. GCF 031547805.1 WP 310599333.1
Pseudomonadati Thermodesulfobacteriota Desulfobulbia Desulfobulbales Desulfobulbus rhabdoformis WP 205228499.1
Pseudomonadati Thermodesulfobacteriota Desulfobulbia Desulfobulbales Desulfobulbus oralis WP 104937143.1
Pseudomonadati Thermodesulfobacteriota Desulfobulbia Desulfobulbales uncultured Desulfobulbus sp. GCF 937872135.1 WP 297954582.1
Pseudomonadati Thermodesulfobacteriota Desulfobulbia Desulfobulbales Desulfobulbus alkaliphilus WP 205241670.1
Pseudomonadati Thermodesulfobacteriota Desulfobulbia Desulfobulbales Desulfopila sp. isolate S6 bin.40 strict MFN2353261.1
Pseudomonadati Thermodesulfobacteriota Desulfobulbia Desulfobulbales Desulfopila sp. isolate bin.795 MEE4242235.1
Pseudomonadati Thermodesulfobacteriota Desulfobulbia Desulfobulbales Desulfopila sp. isolate SMAG U9206 HKJ66220.1
Pseudomonadati Thermodesulfobacteriota Desulfobulbia Desulfobulbales Desulfocapsaceae bacterium isolate MO 192.B14 MDJ0621376.1
Pseudomonadati Thermodesulfobacteriota Desulfobulbia Desulfobulbales Desulfopila inferna WP 205234906.1
Pseudomonadati Thermodesulfobacteriota Desulfobulbia Desulfobulbales Candidatus Desulfatifera sulfidica MBC8208472.1
Pseudomonadati Thermodesulfobacteriota Desulfobacteria Desulfobacterales Desulfosarcinaceae bacterium isolate KA05 MBW6519496.1
Pseudomonadati Thermodesulfobacteriota unclassified Thermodesulfobacteriota Thermodesulfobacteriota bacterium isolate MAG013 MEN8188590.1
Pseudomonadati Thermodesulfobacteriota Desulfobulbia Desulfobulbales Desulfocapsa sulfexigens WP 015404718.1
Pseudomonadati Thermodesulfobacteriota Desulfobulbia Desulfobulbales Desulfocapsa sp. isolate MAG 30 RUM43995.1
Pseudomonadati Thermodesulfobacteriota Desulfobulbia Desulfobulbales Desulfocapsaceae bacterium isolate MAG CA64 MDR3630035.1
Pseudomonadati Thermodesulfobacteriota Desulfobulbia Desulfobulbales Desulfobulbaceae bacterium isolate Rs511 bin14 MDR3088681.1
Pseudomonadati Thermodesulfobacteriota Desulfobulbia Desulfobulbales Desulfobulbaceae bacterium isolate UBA8130 HBT96479.1
Pseudomonadati Thermodesulfobacteriota Desulfobulbia Desulfobulbales Desulfobulbaceae bacterium isolate Crp329 bin2 MDR0476694.1
Pseudomonadati Thermodesulfobacteriota Desulfobulbia Desulfobulbales Desulfocapsaceae bacterium isolate bin.691 MEE4166420.1
Pseudomonadati Thermodesulfobacteriota Desulfobulbia Desulfobulbales Desulfofustis sp. PB SRB1 MBL0379157.1
Pseudomonadati Thermodesulfobacteriota Desulfobulbia Desulfobulbales Desulfofustis limnaeus WP 354005687.1
Pseudomonadati Thermodesulfobacteriota Desulfobulbia Desulfobulbales Desulfocapsaceae bacterium isolate MFD01352 ilm asm bin7 MGA7278241.1
Pseudomonadati Thermodesulfobacteriota Desulfobulbia Desulfobulbales Desulfofustis sp. isolate Spart 067 MAG MEJ2137252.1
Pseudomonadati Thermodesulfobacteriota Desulfobulbia Desulfobulbales Desulfoprunum sp. isolate C5 metabat.bin.56 MDP3480351.1
Pseudomonadati Thermodesulfobacteriota Desulfobulbia Desulfobulbales Desulfoprunum benzoelyticum WP 183351594.1
Pseudomonadati Thermodesulfobacteriota Desulfobulbia Desulfobulbales Desulfotalea sp. isolate tPB Delt2 MGL1930135.1
Pseudomonadati Thermodesulfobacteriota Desulfobulbia Desulfobulbales Desulfotalea psychrophila WP 011189975.1
Pseudomonadati Thermodesulfobacteriota Desulfobulbia Desulfobulbales Desulfopila sp. IMCC35008 WP 163339624.1
Pseudomonadati Thermodesulfobacteriota Desulfobulbia Desulfobulbales Desulfosediminicola flagellatus WP 136805421.1
Pseudomonadati Thermodesulfobacteriota Desulfobulbia Desulfobulbales Desulfopila sp. isolate VLC 059 MFV0438659.1
Pseudomonadati Thermodesulfobacteriota Desulfobulbia Desulfobulbales Desulfopila aestuarii WP 073614954.1
Pseudomonadati Thermodesulfobacteriota Desulfobulbia Desulfobulbales Desulfomarina profundi WP 228856347.1
Pseudomonadati Thermodesulfobacteriota Desulfobulbia Desulfobulbales Desulfobulbaceae bacterium isolate RS 2 19 MCK5516803.1
Pseudomonadati Thermodesulfobacteriota Desulfobulbia Desulfobulbales Desulforhopalus sp. isolate SMAG U16051 HSL39018.1
Pseudomonadati Thermodesulfobacteriota Desulfobulbia Desulfobulbales Desulforhopalus sp. isolate EBRS2.bin23 MFW2364782.1
Pseudomonadati Thermodesulfobacteriota Desulfobulbia Desulfobulbales Desulforhopalus sp. isolate MFD12129 ilm asm bin8 MGB3223801.1
Pseudomonadati Thermodesulfobacteriota Desulfobulbia Desulfobulbales Desulfopila sp. IMCC35006 WP 136811386.1
Pseudomonadati Thermodesulfobacteriota Desulfobulbia Desulfobulbales Desulforhopalus vacuolatus WP 205219123.1
Pseudomonadati Thermodesulfobacteriota Desulfobulbia Desulfobulbales Desulforhopalus singaporensis WP 176761103.1
Pseudomonadati Thermodesulfobacteriota Desulfobulbia Desulfobulbales Desulforhopalus sp. isolate 3300055726 39 MFT5726421.1
Pseudomonadati Thermodesulfobacteriota Desulfobulbia Desulfobulbales Desulforhopalus sp. IMCC35007 WP 136872363.1
Pseudomonadati Thermodesulfobacteriota Syntrophia Syntrophales Syntrophaceae bacterium isolate UWMA 0184 HIC90506.1
Pseudomonadati Thermodesulfobacteriota Candidatus Desulfofervidia Candidatus Desulfofervidaceae bacterium isolate AUK281 MCD6320292.1
Pseudomonadati Thermodesulfobacteriota Candidatus Desulfofervidia Candidatus Desulfofervidus auxilia strain HS1 WP 066063932.1
Pseudomonadati Thermodesulfobacteriota Candidatus Desulfofervidia Candidatus Desulfofervidus auxilia isolate PB NA091.008 bin6 MDL1957176.1
Pseudomonadati Thermodesulfobacteriota Candidatus Desulfofervidia Candidatus Desulfofervidus auxilia isolate HyVt 113 MDD35524.1
Pseudomonadati Thermodesulfobacteriota Desulfobaccia Desulfobaccales Desulfobacca acetoxidans strain DSM 11109 WP 013706806.1
Pseudomonadati Thermodesulfobacteriota Desulfobacteria Desulfobacterales Desulfobacteraceae bacterium isolate D4M11 8 MFP3866571.1
Pseudomonadati Thermodesulfobacteriota Desulfobaccia Desulfobaccales Desulfobacca sp. isolate STE 27 MDD3582366.1

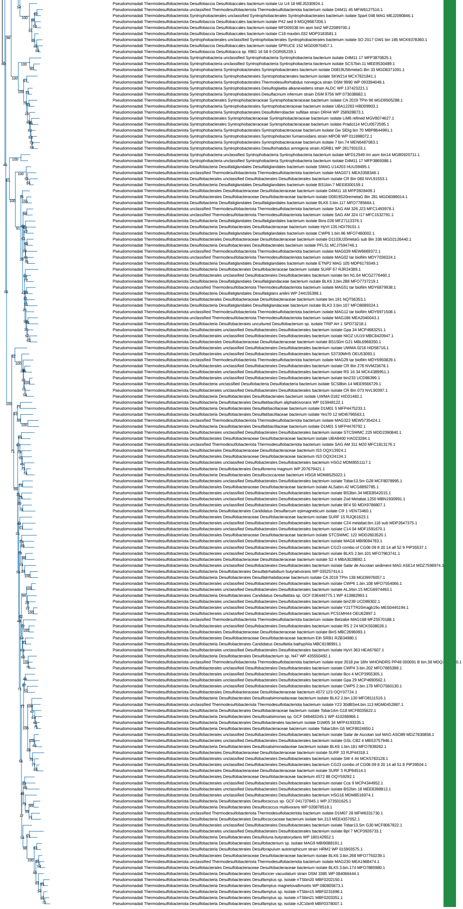
Pseudomonadati Thermodesulfobacteriota Desulfobaccia Desulfobaccales bacterium isolate Uz U4 18 MEJ5330924.1
Pseudomonadati Thermodesulfobacteriota unclassified Thermodesulfobacteriota Thermodesulfobacteriota bacterium isolate D4M11 45 MFW6127516.1
Pseudomonadati Thermodesulfobacteriota Syntrophobacterales unclassified Syntrophobacterales Syntrophobacterales bacterium isolate Spart 048 MAG MEJ2090846.1
Pseudomonadati Thermodesulfobacteriota Desulfobaccia Desulfobaccales bacterium isolate P42 sed 9 MGQ9687208.1
Pseudomonadati Thermodesulfobacteriota Desulfobaccia Desulfobaccales bacterium isolate MFD09338 ilm asm bin2 MFZ2089700.1
Pseudomonadati Thermodesulfobacteriota Desulfobaccia Desulfobaccales bacterium isolate C18 maxbin.032 MDP3183581.1
Pseudomonadati Thermodesulfobacteriota Syntrophobacterales unclassified Syntrophobacterales Syntrophobacterales bacterium isolate SO 2017 GW1 bin 185 MCK9378360.1
Pseudomonadati Thermodesulfobacteriota Desulfobaccia Desulfobaccales bacterium isolate SPRUCE 152 MGD0970457.1
Pseudomonadati Thermodesulfobacteriota Desulfobaccia Desulfobacca sp. RBG 16 58 9 OGR05239.1
Pseudomonadati Thermodesulfobacteriota Syntrophobacteria unclassified Syntrophobacteria Syntrophobacteria bacterium isolate D4M11 17 MFP3870825.1
Pseudomonadati Thermodesulfobacteriota Syntrophobacteria unclassified Syntrophobacteria Syntrophobacteria bacterium isolate SCS7bin.11 MEE9530489.1
Pseudomonadati Thermodesulfobacteriota Syntrophobacteria Syntrophobacterales Syntrophobacterales bacterium isolate D0819U56metaG Bin 33 MGD8371091.1
Pseudomonadati Thermodesulfobacteriota Syntrophobacteria Syntrophobacterales Syntrophobacterales bacterium isolate SKW214 MCX7821841.1
Pseudomonadati Thermodesulfobacteriota Syntrophobacteria Syntrophobacterales Thermodesulforhabdus norvegica strain DSM 9990 WP 093394049.1
Pseudomonadati Thermodesulfobacteriota Syntrophobacteria Syntrophobacterales Desulfoglaeba alkanexedens strain ALDC WP 137423221.1
Pseudomonadati Thermodesulfobacteriota Syntrophobacteria Syntrophobacterales Desulfacinum infernum strain DSM 9756 WP 073038682.1
Pseudomonadati Thermodesulfobacteriota Syntrophobacterales Syntrophobacteraceae Syntrophobacteraceae bacterium isolate CA 2019 TPIn 96 MGD9505288.1
Pseudomonadati Thermodesulfobacteriota Syntrophobacteria Syntrophobacterales Syntrophobacteraceae bacterium isolate UBA12263 HBD09903.1
Pseudomonadati Thermodesulfobacteriota Syntrophobacteria Syntrophobacterales Desulfoferrobacter suflitae strain DRH4 WP 258928873.1
Pseudomonadati Thermodesulfobacteriota Syntrophobacterales Syntrophobacteraceae Syntrophobacteraceae bacterium isolate LiM6 refined MGV8074627.1
Pseudomonadati Thermodesulfobacteriota Syntrophobacterales Syntrophobacteraceae Syntrophobacteraceae bacterium isolate Prado114 MCU0572595.1
Pseudomonadati Thermodesulfobacteriota Syntrophobacteria Syntrophobacterales Syntrophobacteraceae bacterium isolate Gw SlDig bin 70 MBP8644991.1
Pseudomonadati Thermodesulfobacteriota Syntrophobacteria Syntrophobacterales Syntrophobacter fumaroxidans strain MPOB WP 011698072.1
Pseudomonadati Thermodesulfobacteriota Syntrophobacteria Syntrophobacterales Syntrophobacteraceae bacterium isolate 7 bin.74 MEN6487083.1
Pseudomonadati Thermodesulfobacteriota Syntrophobacteria Syntrophobacterales Desulforhabdus amnigena strain ASRB1 WP 281793103.1
Pseudomonadati Thermodesulfobacteriota Syntrophobacteria unclassified Syntrophobacteria Syntrophobacteria bacterium isolate MFD12949 ilm asm bin14 MGB5920711.1
Pseudomonadati Thermodesulfobacteriota Syntrophobacteria unclassified Syntrophobacteria Syntrophobacteria bacterium isolate D4M11 17 MFP3869388.1
Pseudomonadati Thermodesulfobacteriota Desulfobacteria Desulfatiglandales Desulfatiglandales bacterium isolate SMAG U14203 HUU39495.1
Pseudomonadati Thermodesulfobacteriota unclassified Thermodesulfobacteriota Thermodesulfobacteriota bacterium isolate MAG071 MEA3358346.1
Pseudomonadati Thermodesulfobacteriota Desulfobacterales unclassified Desulfobacterales Desulfobacterales bacterium isolate CR Bin 060 NVL91553.1
Pseudomonadati Thermodesulfobacteriota Desulfobacteria Desulfatiglandales Desulfatiglandales bacterium isolate BS1bin.7 MEE8300159.1
Pseudomonadati Thermodesulfobacteriota Desulfobacterales Desulfobacteraceae Desulfobacteraceae bacterium isolate D4M11 18 MFP3928409.1
Pseudomonadati Thermodesulfobacteriota Desulfobacterales Desulfobacteraceae Desulfobacteraceae bacterium isolate D0819S20remetaG Bin 281 MGD8389014.1
Pseudomonadati Thermodesulfobacteriota Desulfobacteria Desulfatiglandales Desulfatiglandales bacterium isolate BLK6 3.bin.117 MFO7785664.1
Pseudomonadati Thermodesulfobacteriota unclassified Thermodesulfobacteriota Thermodesulfobacteriota bacterium isolate SAG AM 326 J23 MFC1493978.1
Pseudomonadati Thermodesulfobacteriota unclassified Thermodesulfobacteriota Thermodesulfobacteriota bacterium isolate SAG AM 324 I17 MFC1532791.1
Pseudomonadati Thermodesulfobacteriota Desulfobacteria Desulfatiglandales Desulfatiglandales bacterium isolate Bins.026 MFZ7113376.1
Pseudomonadati Thermodesulfobacteriota Desulfobacteria Desulfobacterales Desulfobacteraceae bacterium isolate HyVt 135 HDI79101.1
Pseudomonadati Thermodesulfobacteriota Desulfobacteria Desulfatiglandales Desulfatiglandales bacterium isolate CWP6 1.bin.96 MFO7460002.1
Pseudomonadati Thermodesulfobacteriota Desulfobacterales Desulfobacteraceae Desulfobacteraceae bacterium isolate D1103U20metaG sub Bin 336 MGD2126440.1
Pseudomonadati Thermodesulfobacteriota Desulfobacteria Desulfobacterales Desulfobacterales bacterium isolate PFL51 MCJ7594746.1
Pseudomonadati Thermodesulfobacteriota unclassified Thermodesulfobacteriota Thermodesulfobacteriota bacterium isolate MAG039 MEW6669372.1
Pseudomonadati Thermodesulfobacteriota unclassified Thermodesulfobacteriota Thermodesulfobacteriota bacterium isolate MAG02 tar biofilm MDY7036324.1
Pseudomonadati Thermodesulfobacteriota Desulfobacteria Desulfatiglandales Desulfatiglandales bacterium isolate ETNP2 MAG 105 MDP6179349.1
Pseudomonadati Thermodesulfobacteriota Desulfobacteria Desulfobacterales Desulfobacteraceae bacterium isolate SURF 67 RJR24389.1
Pseudomonadati Thermodesulfobacteriota Desulfobacterales unclassified Desulfobacterales Desulfobacterales bacterium isolate bin N1.64 MCG2776460.1
Pseudomonadati Thermodesulfobacteriota Desulfobacteria Desulfatiglandales Desulfatiglandaceae bacterium isolate BLK6 3.bin.288 MFO7737219.1
Pseudomonadati Thermodesulfobacteriota unclassified Thermodesulfobacteriota Thermodesulfobacteriota bacterium isolate MAG51 tar biofilm MDY6879938.1
Pseudomonadati Thermodesulfobacteriota Desulfobacteria Desulfatiglandales Desulfatiglans anilini WP 244155398.1
Pseudomonadati Thermodesulfobacteriota Desulfobacterales Desulfobacteraceae Desulfobacteraceae bacterium isolate bin.191 NQT56353.1
Pseudomonadati Thermodesulfobacteriota Desulfobacteria Desulfatiglandales Desulfatiglandaceae bacterium isolate BLK3 3.bin.107 MFO8089324.1
Pseudomonadati Thermodesulfobacteriota unclassified Thermodesulfobacteriota Thermodesulfobacteriota bacterium isolate MAG12 tar biofilm MDY6971508.1
Pseudomonadati Thermodesulfobacteriota unclassified Thermodesulfobacteriota Thermodesulfobacteriota bacterium isolate MAG186 MEA2040043.1
Pseudomonadati Thermodesulfobacteriota Desulfobacteria Desulfobacterales uncultured Desulfobacterium sp. isolate TRIP AH 1 SPD73218.1
Pseudomonadati Thermodesulfobacteriota Desulfobacterales unclassified Desulfobacterales Desulfobacterales bacterium isolate Gpa 34 MCP4683251.1
Pseudomonadati Thermodesulfobacteriota Desulfobacterales unclassified Desulfobacterales Desulfobacterales bacterium isolate NIOZ UU19 MBC8420947.1
Pseudomonadati Thermodesulfobacteriota Desulfobacterales Desulfobacteraceae Desulfobacteraceae bacterium isolate BS150m G21 MBL6968350.1
Pseudomonadati Thermodesulfobacteriota Desulfobacterales unclassified Desulfobacterales Desulfobacterales bacterium isolate UWMA 0216 HID58716.1
Pseudomonadati Thermodesulfobacteriota Desulfobacterales unclassified Desulfobacterales Desulfobacterales bacterium S3730MH5 OEU53093.1
Pseudomonadati Thermodesulfobacteriota unclassified Thermodesulfobacteriota Thermodesulfobacteriota bacterium isolate MAG29 tar biofilm MDY6950829.1
Pseudomonadati Thermodesulfobacteriota Desulfobacterales unclassified Desulfobacterales Desulfobacterales bacterium isolate CR Bin 276 NVM23678.1
Pseudomonadati Thermodesulfobacteriota Desulfobacterales unclassified Desulfobacterales Desulfobacterales bacterium isolate RS 16 34 MCK4389951.1
Pseudomonadati Thermodesulfobacteriota Desulfobacterales unclassified Desulfobacterales Desulfobacterales bacterium isolate bin233 UCD86399.1
Pseudomonadati Thermodesulfobacteriota Desulfobacteria unclassified Desulfobacteria Desulfobacteria bacterium isolate SCS8bin.14 MEE9566729.1
Pseudomonadati Thermodesulfobacteriota Desulfobacterales unclassified Desulfobacterales Desulfobacterales bacterium isolate CR Bin 073 NVL90397.1
Pseudomonadati Thermodesulfobacteriota Desulfobacteria Desulfobacterales Desulfobacterales bacterium isolate UWMA 0182 HID31482.1
Pseudomonadati Thermodesulfobacteriota Desulfobacteria Desulfobacterales Desulfatibacillum aliphaticivorans WP 015948122.1
Pseudomonadati Thermodesulfobacteriota Desulfobacteria Desulfobacterales Desulfatibacillaceae bacterium isolate D1M01 5 MFP4475233.1
Pseudomonadati Thermodesulfobacteriota Desulfobacteria Desulfobacterales Desulfatibacillaceae bacterium isolate Yes70 12 MDI6796563.1
Pseudomonadati Thermodesulfobacteriota unclassified Thermodesulfobacteriota Thermodesulfobacteriota bacterium isolate MAG322 MEW5735424.1
Pseudomonadati Thermodesulfobacteriota Desulfobacteria Desulfobacterales Desulfatibacillaceae bacterium isolate D1M01 5 MFP4476792.1
Pseudomonadati Thermodesulfobacteriota Desulfobacterales unclassified Desulfobacterales Desulfobacterales bacterium isolate STCSWMC 225 MDD2390840.1
Pseudomonadati Thermodesulfobacteriota Desulfobacterales Desulfobacteraceae Desulfobacteraceae bacterium isolate UBA8400 HAO23284.1
Pseudomonadati Thermodesulfobacteriota unclassified Thermodesulfobacteriota Thermodesulfobacteriota bacterium isolate SAG AM 311 M20 MFC1813176.1
Pseudomonadati Thermodesulfobacteriota Desulfobacterales Desulfobacteraceae Desulfobacteraceae bacterium IS3 OQX13924.1
Pseudomonadati Thermodesulfobacteriota Desulfobacterales Desulfobacteraceae Desulfobacteraceae bacterium IS3 OQX24134.1
Pseudomonadati Thermodesulfobacteriota Desulfobacterales unclassified Desulfobacterales Desulfobacterales bacterium HSG2 MDM8551117.1
Pseudomonadati Thermodesulfobacteriota Desulfobacteria Desulfobacterales Desulfonema magnum WP 207679421.1
Pseudomonadati Thermodesulfobacteriota Desulfobacteria Desulfobacterales Desulfococcaceae bacterium HSG8 MDM8525022.1
Pseudomonadati Thermodesulfobacteriota Desulfobacterales unclassified Desulfobacterales Desulfobacterales bacterium isolate Tobar13.5m G28 MCF8078995.1
Pseudomonadati Thermodesulfobacteriota Desulfobacterales Desulfobacteraceae Desulfobacteraceae bacterium isolate ALSabin.42 MCG6892785.1
Pseudomonadati Thermodesulfobacteriota Desulfobacterales unclassified Desulfobacterales Desulfobacterales bacterium isolate BS3bin.34 MEE8542015.1
Pseudomonadati Thermodesulfobacteriota Desulfobacterales unclassified Desulfobacterales Desulfobacterales bacterium isolate Zod Metabat.1250 MBN1930991.1
Pseudomonadati Thermodesulfobacteriota Desulfobacterales unclassified Desulfobacterales Desulfobacterales bacterium isolate BF4 50 MDX9786807.1
Pseudomonadati Thermodesulfobacteriota Desulfobacteria Desulfobacterales Candidatus Desulfarcum epimagneticum isolate CR 1 VEN73460.1
Pseudomonadati Thermodesulfobacteriota Desulfobacterales Desulfobacteraceae Desulfobacteraceae bacterium isolate SURF 15 RJQ61623.1
Pseudomonadati Thermodesulfobacteriota Desulfobacterales unclassified Desulfobacterales Desulfobacterales bacterium isolate C24 metabat.bin.116 sub MDP2647375.1
Pseudomonadati Thermodesulfobacteriota Desulfobacterales unclassified Desulfobacterales Desulfobacterales bacterium isolate C14 04 MDF1591670.1
Pseudomonadati Thermodesulfobacteriota Desulfobacterales Desulfobacteraceae Desulfobacteraceae bacterium isolate STCSWMC 122 MDD2603520.1
Pseudomonadati Thermodesulfobacteriota Desulfobacterales unclassified Desulfobacterales Desulfobacterales bacterium isolate MAG8 MBI9084783.1
Pseudomonadati Thermodesulfobacteriota Desulfobacterales unclassified Desulfobacterales Desulfobacterales bacterium CG23 combo of CG06 09 8 20 14 all 52 9 PIP35537.1
Pseudomonadati Thermodesulfobacteriota Desulfobacterales unclassified Desulfobacterales Desulfobacterales bacterium isolate BLK5 2.bin.101 MFO7963741.1
Pseudomonadati Thermodesulfobacteriota Desulfobacterales Desulfobacteraceae Desulfobacteraceae bacterium isolate S2 4 MBA3028892.1
Pseudomonadati Thermodesulfobacteriota Desulfobacterales unclassified Desulfobacterales Desulfobacterales bacterium isolate Salar de Ascotan sediment MAG ASE14 MDZ7598974.1
Pseudomonadati Thermodesulfobacteriota Desulfobacteria Desulfobacterales Desulfatirhabdium butyrativorans WP 035257414.1
Pseudomonadati Thermodesulfobacteriota Desulfobacteria Desulfobacterales Desulfatirhabdiaceae bacterium isolate CA 2019 TPIn 138 MGD9976057.1
Pseudomonadati Thermodesulfobacteriota Desulfobacterales unclassified Desulfobacterales Desulfobacterales bacterium isolate CWP6 1.bin.108 MFO7554066.1
Pseudomonadati Thermodesulfobacteriota Desulfobacterales unclassified Desulfobacterales Desulfobacterales bacterium isolate ALJrbin.15 MCG6974493.1
Pseudomonadati Thermodesulfobacteriota Desulfobacteria Desulfobacterales Candidatus Desulfatibia sp. GCF 036446775.1 WP 413882993.1
Pseudomonadati Thermodesulfobacteriota Desulfobacterales unclassified Desulfobacterales Desulfobacterales bacterium isolate bin239 UCD89302.1
Pseudomonadati Thermodesulfobacteriota Desulfobacterales unclassified Desulfobacterales Desulfobacterales bacterium isolate Y21TTR20magb19o MES0445194.1
Pseudomonadati Thermodesulfobacteriota Desulfobacterales unclassified Desulfobacterales Desulfobacterales bacterium PC51MH44 OEU62897.1
Pseudomonadati Thermodesulfobacteriota unclassified Thermodesulfobacteriota Thermodesulfobacteriota bacterium isolate Betzabe MAG168 MFZ5570188.1
Pseudomonadati Thermodesulfobacteriota Desulfobacterales unclassified Desulfobacterales Desulfobacterales bacterium isolate RS 2 24 MCK5508028.1
Pseudomonadati Thermodesulfobacteriota Desulfobacterales Desulfobacteraceae Desulfobacteraceae bacterium isolate Bin5 MBC2696093.1
Pseudomonadati Thermodesulfobacteriota Desulfobacterales Desulfobacteraceae Desulfobacteraceae bacterium Eth SRB1 RZB34980.1
Pseudomonadati Thermodesulfobacteriota Desulfobacteria Desulfobacterales Candidatus Desulfaltia bathyphila MBC8198991.1
Pseudomonadati Thermodesulfobacteriota Desulfobacterales unclassified Desulfobacterales Desulfobacterales bacterium isolate HyVt 363 HEA67607.1
Pseudomonadati Thermodesulfobacteriota Desulfobacteria Desulfobacterales Desulfobacterium sp. N47 WP 435550492.1
Pseudomonadati Thermodesulfobacteriota unclassified Thermodesulfobacteriota Thermodesulfobacteriota bacterium isolate erpe 2018 pw 18hr WHONDRS PP48 000091 B bin.38 MDQ1
Pseudomonadati Thermodesulfobacteriota Desulfobacterales unclassified Desulfobacterales Desulfobacterales bacterium isolate CWP4 3.bin.202 MFO7665398.1
Pseudomonadati Thermodesulfobacteriota Desulfobacterales unclassified Desulfobacterales Desulfobacterales bacterium isolate Bco 4 MCP3955305.1
Pseudomonadati Thermodesulfobacteriota Desulfobacterales unclassified Desulfobacterales Desulfobacterales bacterium isolate Gpa 29 MCP4690562.1
Pseudomonadati Thermodesulfobacteriota Desulfobacterales unclassified Desulfobacterales Desulfobacterales bacterium isolate CWP5 2.bin.178 MFO7560130.1
Pseudomonadati Thermodesulfobacteriota Desulfobacterales Desulfobacteraceae Desulfobacteraceae bacterium 4572 123 OQY07724.1
Pseudomonadati Thermodesulfobacteriota Desulfobacteria Desulfobacterales Desulfosalsimonadaceae bacterium isolate BLK2 2.bin.130 MFO8111516.1
Pseudomonadati Thermodesulfobacteriota unclassified Thermodesulfobacteriota Thermodesulfobacteriota bacterium isolate Y23 30dBSe4.bin.113 MGM0452887.1
Pseudomonadati Thermodesulfobacteriota Desulfobacteria Desulfobacterales Desulfobacteraceae bacterium isolate Tobar14m G18 MCF8035622.1
Pseudomonadati Thermodesulfobacteriota Desulfobacteria Desulfobacterales Desulfosalsimonas sp. GCF 046483245.1 WP 410268966.1
Pseudomonadati Thermodesulfobacteriota Desulfobacteria Desulfobacterales Desulfobacterales bacterium isolate D1M05 34 MFP4193335.1
Pseudomonadati Thermodesulfobacteriota Desulfobacteria Desulfobacterales Desulfobacteraceae bacterium isolate Tobar18m G5 MCF8024650.1
Pseudomonadati Thermodesulfobacteriota Desulfobacterales unclassified Desulfobacterales Desulfobacterales bacterium isolate Salar de Ascotan soil MAG ASO89 MDZ7830858.1
Pseudomonadati Thermodesulfobacteriota Desulfobacterales unclassified Desulfobacterales Desulfobacterales bacterium isolate GSL CB2 4 MBS3757946.1
Pseudomonadati Thermodesulfobacteriota Desulfobacteria Desulfobacterales Desulfosalsimonadaceae bacterium isolate BLK6 1.bin.181 MFO7839262.1
Pseudomonadati Thermodesulfobacteriota Desulfobacterales Desulfobacteraceae Desulfobacteraceae bacterium isolate SURF 33 RJP44318.1
Pseudomonadati Thermodesulfobacteriota Desulfobacterales unclassified Desulfobacterales Desulfobacterales bacterium isolate SW 4 44 MCK5783128.1
Pseudomonadati Thermodesulfobacteriota Desulfobacterales unclassified Desulfobacterales Desulfobacterales bacterium CG23 combo of CG06 09 8 20 14 all 51 8 PIP39504.1
Pseudomonadati Thermodesulfobacteriota Desulfobacterales Desulfobacteraceae Desulfobacteraceae bacterium isolate SURF 3 RJP84514.1
Pseudomonadati Thermodesulfobacteriota Desulfobacterales Desulfobacteraceae Desulfobacteraceae bacterium 4572 88 OQY59292.1
Pseudomonadati Thermodesulfobacteriota Desulfobacterales unclassified Desulfobacterales Desulfobacterales bacterium isolate Cca 9 MCP4344952.1
Pseudomonadati Thermodesulfobacteriota Desulfobacterales unclassified Desulfobacterales Desulfobacterales bacterium isolate BS2bin.18 MEE8398813.1
Pseudomonadati Thermodesulfobacteriota Desulfobacterales unclassified Desulfobacterales Desulfobacterales bacterium HSG16 MDM8516974.1
Pseudomonadati Thermodesulfobacteriota Desulfobacteria Desulfobacterales Desulfococcus sp. GCF 041737845.1 WP 373501625.1
Pseudomonadati Thermodesulfobacteriota Desulfobacteria Desulfobacterales Desulfococcus multivorans WP 020878518.1
Pseudomonadati Thermodesulfobacteriota unclassified Thermodesulfobacteriota Thermodesulfobacteriota bacterium isolate D1M07 28 MFW6331730.1
Pseudomonadati Thermodesulfobacteriota Desulfobacteria Desulfobacterales Desulfococcaceae bacterium isolate bin.313 MEE4357052.1
Pseudomonadati Thermodesulfobacteriota Desulfobacterales unclassified Desulfobacterales Desulfobacterales bacterium isolate Tobar13.5m G30 MCF8067822.1
Pseudomonadati Thermodesulfobacteriota Desulfobacterales unclassified Desulfobacterales Desulfobacterales bacterium isolate Bpl 7 MCP3926733.1
Pseudomonadati Thermodesulfobacteriota Desulfobacteria Desulfobacterales Desulfoluna butyratoxydans WP 180142652.1
Pseudomonadati Thermodesulfobacteriota Desulfobacteria Desulfobacterales Desulfobacterium sp. isolate MAG9 MBI9088191.1
Pseudomonadati Thermodesulfobacteriota Desulfobacteria Desulfobacterales Desulforapulum autotrophicum strain HRM2 WP 015903575.1
Pseudomonadati Thermodesulfobacteriota Desulfobacterales Desulfobacteraceae Desulfobacteraceae bacterium isolate BLK6 3.bin.268 MFO7750239.1
Pseudomonadati Thermodesulfobacteriota unclassified Thermodesulfobacteriota Thermodesulfobacteriota bacterium isolate MAG230 MEA1968474.1
Pseudomonadati Thermodesulfobacteriota Desulfobacterales Desulfobacteraceae Desulfobacteraceae bacterium isolate BLK5 3.bin.174 MFO7885980.1
Pseudomonadati Thermodesulfobacteriota Desulfobacteria Desulfobacterales Desulfocicer vacuolatum strain DSM 3385 WP 084066444.1
Pseudomonadati Thermodesulfobacteriota Desulfobacteria Desulfobacterales Desulfamplus sp. isolate nTSbin20 MBF0202150.1
Pseudomonadati Thermodesulfobacteriota Desulfobacteria Desulfobacterales Desulfamplus magnetovallimortis WP 080805673.1
Pseudomonadati Thermodesulfobacteriota Desulfobacteria Desulfobacterales Desulfamplus sp. isolate nTSbin15 MBF0231696.1
Pseudomonadati Thermodesulfobacteriota Desulfobacteria Desulfobacterales Desulfamplus sp. isolate nTSbin21 MBF0203351.1
Pseudomonadati Thermodesulfobacteriota Desulfobacteria Desulfobacterales Desulfamplus sp. isolate nJC1bin9 MBF0378007.1
