# Supplementary material for: Divergent evolution of the PRPS enzymes across the tree of life
Source: bioRxiv. 2026 Jun 3:2026.06.01.728777. Preprint. [Version 1] doi: 10.64898/2026.06.01.728777 (PMC13252144; doi:10.64898/2026.06.01.728777)
Supplement: Supplement 4 [file media-4.pdf]

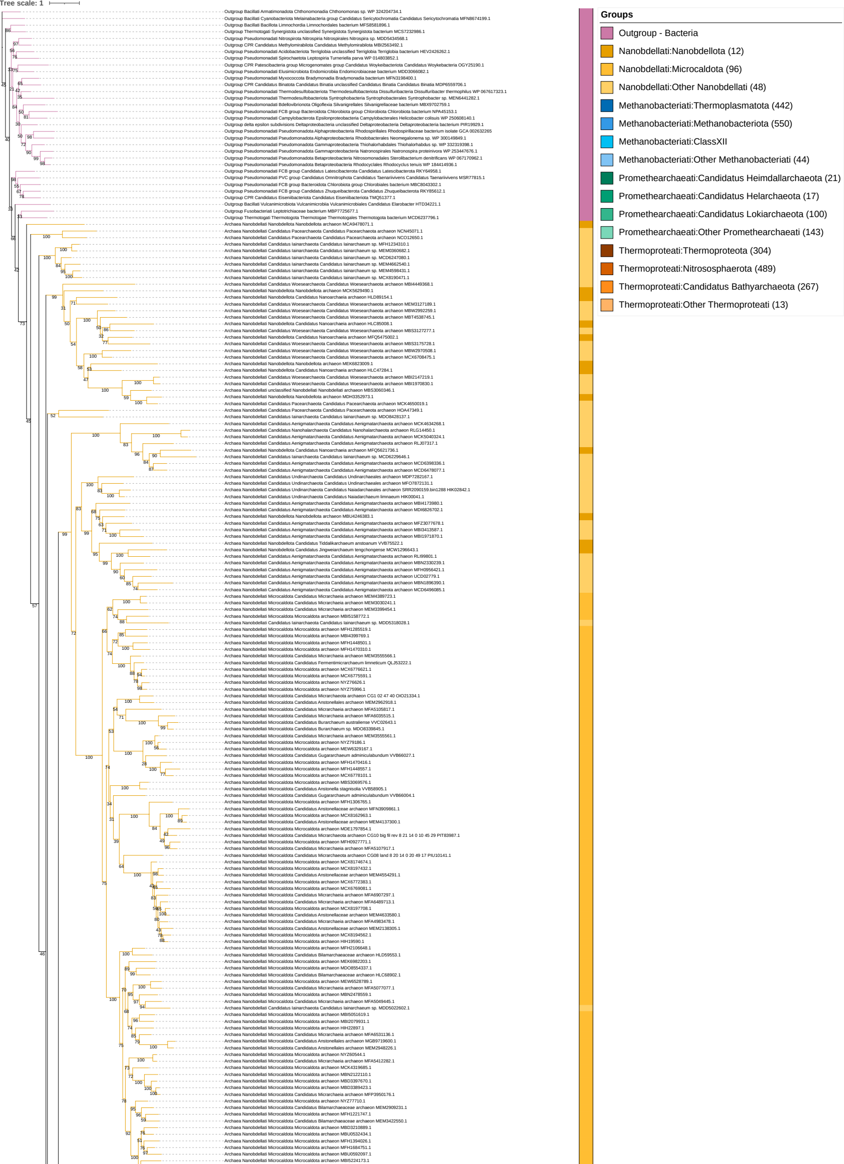
Tree scale: 1
Outgroup Bacillati Armatimonadota Chthonomonadia Chthonomonas sp. WP 324204734.1
Outgroup Bacillati Cyanobacteriota Melainabacteria group Candidatus Sericytochromatia Candidatus Sericytochromatia MFN8674199.1
Outgroup Bacillati Bacillota Limnochordia Limnochordales bacterium MFS8581896.1
Outgroup Thermotogati Synergistota unclassified Synergistota Synergistota bacterium MCS7232986.1
Outgroup Pseudomonadati Nitrospirota Nitrospiria Nitrospirales Nitrospira sp. MDD5434568.1
Outgroup CPR Candidatus Methylomirabilota Candidatus Methylomirabilota MBI2563492.1
Outgroup Pseudomonadati Acidobacteriota Terriglobia unclassified Terriglobia Terriglobia bacterium HEV2426262.1
Outgroup Pseudomonadati Spirochaetota Leptospiria Turneriella parva WP 014803852.1
Outgroup CPR Patescibacteria group Microgenomates group Candidatus Woykeibacteriota Candidatus Woykebacteria OGY25190.1
Outgroup Pseudomonadati Elusimicrobiota Endomicrobiia Endomicrobiaceae bacterium MDD3066082.1
Outgroup Pseudomonadati Myxococcota Bradymonadia Bradymonadia bacterium MFN3198400.1
Outgroup CPR Candidatus Binatota Candidatus Binatia unclassified Candidatus Binatia Candidatus Binatia MDP6559706.1
Outgroup Pseudomonadati Thermodesulfobacteriota Thermodesulfobacteriota Dissulfuribacteria Dissulfuribacter thermophilus WP 067617323.1
Outgroup Pseudomonadati Thermodesulfobacteriota Syntrophobacteria Syntrophobacterales Syntrophobacter sp. MEN6441282.1
Outgroup Pseudomonadati Bdellovibrionota Oligoflexia Silvanigrellales Silvanigrellaceae bacterium MBX9702759.1
Outgroup Pseudomonadati FCB group Bacteroidota Chlorobiota group Chlorobiota Chlorobiota bacterium NPA45153.1
Outgroup Pseudomonadati Campylobacterota Epsilonproteobacteria Campylobacterales Helicobacter colisuis WP 250608140.1
Outgroup delta epsilon subdivisions Deltaproteobacteria unclassified Deltaproteobacteria Deltaproteobacteria bacterium PIR19929.1
Outgroup Pseudomonadati Pseudomonadota Alphaproteobacteria Rhodospirillales Rhodospirillaceae bacterium isolate GCA 002632265
Outgroup Pseudomonadati Pseudomonadota Alphaproteobacteria Rhodobacterales Neomegalonema sp. WP 300149849.1
Outgroup Pseudomonadati Pseudomonadota Gammaproteobacteria Thiohalorhabdales Thiohalorhabdus sp. WP 332319398.1
Outgroup Pseudomonadati Pseudomonadota Gammaproteobacteria Natronospirales Natronospira proteinivora WP 253447676.1
Outgroup Pseudomonadati Pseudomonadota Betaproteobacteria Nitrosomonadales Sterolibacterium denitrificans WP 067170962.1
Outgroup Pseudomonadati Pseudomonadota Betaproteobacteria Rhodocyclales Rhodocyclus tenuis WP 184414936.1
Outgroup Pseudomonadati FCB group Candidatus Latescibacterota Candidatus Latescibacterota RKY64958.1
Outgroup Pseudomonadati PVC group Candidatus Omnitrophota Candidatus Taenariivivens Candidatus Taenariivivens MSR77815.1
Outgroup Pseudomonadati FCB group Bacteroidota Chlorobiota group Chlorobiales bacterium MBC8043302.1
Outgroup Pseudomonadati FCB group Candidatus Zhuqueibacterota Candidatus Zhuqueibacterota RKY85612.1
Outgroup CPR Candidatus Eiseniibacteriota Candidatus Eiseniibacteriota TMQ51377.1
Outgroup Bacillati Vulcanimicrobiota Vulcanimicrobiia Vulcanimicrobiales Candidatus Elarobacter HTD34221.1
Outgroup Fusobacteriati Leptotrichiaceae bacterium MBP7725677.1
Outgroup Thermotogati Thermotogota Thermotogae Thermotogales Thermotogota bacterium MCD6237796.1
Archaea Nanobdellati Nanobdellota Nanobdellota archaeon MCA9478071.1
Archaea Nanobdellati Candidatus Pacearchaeota Candidatus Pacearchaeota archaeon NCN45071.1
Archaea Nanobdellati Candidatus Pacearchaeota Candidatus Pacearchaeota archaeon NCO12650.1
Archaea Nanobdellati Candidatus Iainarchaeota Candidatus Iainarchaeum sp. MFH1234310.1
Archaea Nanobdellati Candidatus Iainarchaeota Candidatus Iainarchaeum sp. MEM0360682.1
Archaea Nanobdellati Candidatus Iainarchaeota Candidatus Iainarchaeum sp. MCD6247080.1
Archaea Nanobdellati Candidatus Iainarchaeota Candidatus Iainarchaeum sp. MEM4662540.1
Archaea Nanobdellati Candidatus Iainarchaeota Candidatus Iainarchaeum sp. MEM4598431.1
Archaea Nanobdellati Candidatus Iainarchaeota Candidatus Iainarchaeum sp. MCX8190471.1
Archaea Nanobdellati Candidatus Woesearchaeota Candidatus Woesearchaeota archaeon MBI4449368.1
Archaea Nanobdellati Nanobdellota Nanobdellota archaeon MCK5629490.1
Archaea Nanobdellati Nanobdellota Candidatus Nanoarchaeia archaeon HLD89154.1
Archaea Nanobdellati Candidatus Woesearchaeota Candidatus Woesearchaeota archaeon MEM3127189.1
Archaea Nanobdellati Candidatus Woesearchaeota Candidatus Woesearchaeota archaeon MBW2992259.1
Archaea Nanobdellati Candidatus Woesearchaeota Candidatus Woesearchaeota archaeon MBT4538745.1
Archaea Nanobdellati Nanobdellota Candidatus Nanoarchaeia archaeon HLC85008.1
Archaea Nanobdellati Candidatus Woesearchaeota Candidatus Woesearchaeota archaeon MBS3127277.1
Archaea Nanobdellati Nanobdellota Candidatus Nanoarchaeia archaeon MFQ5475002.1
Archaea Nanobdellati Candidatus Woesearchaeota Candidatus Woesearchaeota archaeon MBS3175728.1
Archaea Nanobdellati Candidatus Woesearchaeota Candidatus Woesearchaeota archaeon MBW2970508.1
Archaea Nanobdellati Candidatus Woesearchaeota Candidatus Woesearchaeota archaeon MCX6708475.1
Archaea Nanobdellati Nanobdellota Nanobdellota archaeon MEK6823009.1
Archaea Nanobdellati Nanobdellota Candidatus Nanoarchaeia archaeon HLC47284.1
Archaea Nanobdellati Candidatus Woesearchaeota Candidatus Woesearchaeota archaeon MBI2147219.1
Archaea Nanobdellati Candidatus Woesearchaeota Candidatus Woesearchaeota archaeon MBI1970830.1
Archaea Nanobdellati unclassified Nanobdellati Nanobdellati archaeon MBS3060346.1
Archaea Nanobdellati Nanobdellota Nanobdellota archaeon MDH3352973.1
Archaea Nanobdellati Candidatus Pacearchaeota Candidatus Pacearchaeota archaeon MCK4650019.1
Archaea Nanobdellati Candidatus Pacearchaeota Candidatus Pacearchaeota archaeon HOA47349.1
Archaea Nanobdellati Candidatus Iainarchaeota Candidatus Iainarchaeum sp. MDO8428137.1
Archaea Nanobdellati Candidatus Aenigmatarchaeota Candidatus Aenigmatarchaeota archaeon MCK4634268.1
Archaea Nanobdellati Candidatus Nanohalarchaeota Candidatus Nanohalarchaeota archaeon RLG14450.1
Archaea Nanobdellati Candidatus Aenigmatarchaeota Candidatus Aenigmatarchaeota archaeon MCK5040324.1
Archaea Nanobdellati Candidatus Aenigmatarchaeota Candidatus Aenigmatarchaeota archaeon RLJ07317.1
Archaea Nanobdellati Nanobdellota Candidatus Nanoarchaeia archaeon MFQ5621736.1
Archaea Nanobdellati Candidatus Iainarchaeota Candidatus Iainarchaeum sp. MCD6229646.1
Archaea Nanobdellati Candidatus Aenigmatarchaeota Candidatus Aenigmatarchaeota archaeon MCD6398336.1
Archaea Nanobdellati Candidatus Aenigmatarchaeota Candidatus Aenigmatarchaeota archaeon MCD6478077.1
Archaea Nanobdellati Candidatus Undinarchaeota Candidatus Undinarchaeales archaeon MDP7282167.1
Archaea Nanobdellati Candidatus Undinarchaeota Candidatus Undinarchaeales archaeon MFO7872131.1
Archaea Nanobdellati Candidatus Undinarchaeota Candidatus Naiadarchaeales archaeon SRR2090159.bin1288 HIK02842.1
Archaea Nanobdellati Candidatus Undinarchaeota Candidatus Naiadarchaeum limnaeum HIK00041.1
Archaea Nanobdellati Candidatus Aenigmatarchaeota Candidatus Aenigmatarchaeota archaeon MBI4173980.1
Archaea Nanobdellati Candidatus Aenigmatarchaeota Candidatus Aenigmatarchaeota archaeon MDI6826702.1
Archaea Nanobdellati Nanobdellota Nanobdellota archaeon MBU4246383.1
Archaea Nanobdellati Candidatus Aenigmatarchaeota Candidatus Aenigmatarchaeota archaeon MFZ3077678.1
Archaea Nanobdellati Candidatus Aenigmatarchaeota Candidatus Aenigmatarchaeota archaeon MBI3413587.1
Archaea Nanobdellati Candidatus Aenigmatarchaeota Candidatus Aenigmatarchaeota archaeon MBI1971870.1
Archaea Nanobdellati Nanobdellota Candidatus Tiddalikarchaeum anstoanum VVB75522.1
Archaea Nanobdellati Nanobdellota Candidatus Jingweiarchaeum tengchongense MCW1296643.1
Archaea Nanobdellati Candidatus Aenigmatarchaeota Candidatus Aenigmatarchaeota archaeon RLI99801.1
Archaea Nanobdellati Candidatus Aenigmatarchaeota Candidatus Aenigmatarchaeota archaeon MBN2330239.1
Archaea Nanobdellati Candidatus Aenigmatarchaeota Candidatus Aenigmatarchaeota archaeon MFH0956421.1
Archaea Nanobdellati Candidatus Aenigmatarchaeota Candidatus Aenigmatarchaeota archaeon UCD02779.1
Archaea Nanobdellati Candidatus Aenigmatarchaeota Candidatus Aenigmatarchaeota archaeon MBN1896390.1
Archaea Nanobdellati Candidatus Aenigmatarchaeota Candidatus Aenigmatarchaeota archaeon MCD6496085.1
Archaea Nanobdellati Microcaldota Candidatus Micrarchaeia archaeon MEM4389723.1
Archaea Nanobdellati Microcaldota Candidatus Micrarchaeia archaeon MEM3030241.1
Archaea Nanobdellati Microcaldota Candidatus Micrarchaeia archaeon MEM3399454.1
Archaea Nanobdellati Microcaldota Microcaldota archaeon MBI5158772.1
Archaea Nanobdellati Candidatus Iainarchaeota Candidatus Iainarchaeum sp. MDD5318028.1
Archaea Nanobdellati Microcaldota Microcaldota archaeon MFH1285519.1
Archaea Nanobdellati Microcaldota Microcaldota archaeon MBI4399769.1
Archaea Nanobdellati Microcaldota Microcaldota archaeon MFH1448501.1
Archaea Nanobdellati Microcaldota Microcaldota archaeon MFH1470310.1
Archaea Nanobdellati Microcaldota Candidatus Micrarchaeia archaeon MEM3555566.1
Archaea Nanobdellati Microcaldota Candidatus Fermentimicrarchaeum limneticum QLJ53222.1
Archaea Nanobdellati Microcaldota Microcaldota archaeon MCX6776621.1
Archaea Nanobdellati Microcaldota Microcaldota archaeon MCX6775591.1
Archaea Nanobdellati Microcaldota Microcaldota archaeon NYZ76626.1
Archaea Nanobdellati Microcaldota Microcaldota archaeon NYZ75996.1
Archaea Nanobdellati Microcaldota Candidatus Micrarchaeota archaeon CG1 02 47 40 OIO21334.1
Archaea Nanobdellati Microcaldota Candidatus Anstonellales archaeon MEM2962918.1
Archaea Nanobdellati Microcaldota Candidatus Micrarchaeia archaeon MFA5105817.1
Archaea Nanobdellati Microcaldota Candidatus Micrarchaeia archaeon MFA6035515.1
Archaea Nanobdellati Microcaldota Candidatus Burarchaeum australiense VVC02643.1
Archaea Nanobdellati Microcaldota Candidatus Burarchaeum sp. MDO8339845.1
Archaea Nanobdellati Microcaldota Candidatus Micrarchaeia archaeon MEM3555561.1
Archaea Nanobdellati Microcaldota Microcaldota archaeon NYZ79186.1
Archaea Nanobdellati Microcaldota Microcaldota archaeon MEW6329167.1
Archaea Nanobdellati Microcaldota Candidatus Gugararchaeum adminiculabundum VVB66027.1
Archaea Nanobdellati Microcaldota Microcaldota archaeon MFH1470416.1
Archaea Nanobdellati Microcaldota Microcaldota archaeon MFH1448557.1
Archaea Nanobdellati Microcaldota Microcaldota archaeon MCX6778101.1
Archaea Nanobdellati Microcaldota Microcaldota archaeon MBS3069576.1
Archaea Nanobdellati Microcaldota Candidatus Anstonella stagnisolia VVB58905.1
Archaea Nanobdellati Microcaldota Candidatus Gugararchaeum adminiculabundum VVB66004.1
Archaea Nanobdellati Microcaldota Microcaldota archaeon MFH1306765.1
Archaea Nanobdellati Microcaldota Candidatus Anstonellaceae archaeon MFN3909861.1
Archaea Nanobdellati Microcaldota Microcaldota archaeon MCX8162963.1
Archaea Nanobdellati Microcaldota Candidatus Anstonellaceae archaeon MEM4137300.1
Archaea Nanobdellati Microcaldota Microcaldota archaeon MDE1797854.1
Archaea Nanobdellati Microcaldota Candidatus Micrarchaeota archaeon CG10 big fil rev 8 21 14 0 10 45 29 PIT83987.1
Archaea Nanobdellati Microcaldota Microcaldota archaeon MFH0927771.1
Archaea Nanobdellati Microcaldota Candidatus Micrarchaeia archaeon MFA5107917.1
Archaea Nanobdellati Microcaldota Candidatus Micrarchaeota archaeon CG08 land 8 20 14 0 20 49 17 PIU10141.1
Archaea Nanobdellati Microcaldota Microcaldota archaeon MCX8174674.1
Archaea Nanobdellati Microcaldota Microcaldota archaeon MCX8197432.1
Archaea Nanobdellati Microcaldota Candidatus Anstonellaceae archaeon MEM4554291.1
Archaea Nanobdellati Microcaldota Microcaldota archaeon MCX6772383.1
Archaea Nanobdellati Microcaldota Microcaldota archaeon MCX6769081.1
Archaea Nanobdellati Microcaldota Candidatus Micrarchaeia archaeon MFA6907297.1
Archaea Nanobdellati Microcaldota Candidatus Micrarchaeia archaeon MFA6489713.1
Archaea Nanobdellati Microcaldota Microcaldota archaeon MCX8197708.1
Archaea Nanobdellati Microcaldota Candidatus Anstonellaceae archaeon MEM4633580.1
Archaea Nanobdellati Microcaldota Candidatus Micrarchaeia archaeon MFA4983478.1
Archaea Nanobdellati Microcaldota Candidatus Anstonellaceae archaeon MEM2138305.1
Archaea Nanobdellati Microcaldota Microcaldota archaeon MCX8194562.1
Archaea Nanobdellati Microcaldota Microcaldota archaeon HIH19590.1
Archaea Nanobdellati Microcaldota Microcaldota archaeon MFH2106648.1
Archaea Nanobdellati Microcaldota Candidatus Bilamarchaeaceae archaeon HLD59553.1
Archaea Nanobdellati Microcaldota Microcaldota archaeon MEK6982203.1
Archaea Nanobdellati Microcaldota Microcaldota archaeon MDO8554337.1
Archaea Nanobdellati Microcaldota Candidatus Bilamarchaeaceae archaeon HLC68902.1
Archaea Nanobdellati Microcaldota Microcaldota archaeon MEW6528789.1
Archaea Nanobdellati Microcaldota Candidatus Micrarchaeia archaeon MFA5077077.1
Archaea Nanobdellati Microcaldota Microcaldota archaeon MBN2478559.1
Archaea Nanobdellati Microcaldota Candidatus Micrarchaeia archaeon MFA5049445.1
Archaea Nanobdellati Candidatus Iainarchaeota Candidatus Iainarchaeum sp. MDD5022602.1
Archaea Nanobdellati Microcaldota Microcaldota archaeon MBI5051619.1
Archaea Nanobdellati Microcaldota Microcaldota archaeon MBI2079931.1
Archaea Nanobdellati Microcaldota Microcaldota archaeon HIH22897.1
Archaea Nanobdellati Microcaldota Candidatus Micrarchaeia archaeon MFA6531136.1
Archaea Nanobdellati Microcaldota Candidatus Anstonellales archaeon MGB9719600.1
Archaea Nanobdellati Microcaldota Candidatus Anstonellales archaeon MEM2948226.1
Archaea Nanobdellati Microcaldota Microcaldota archaeon NYZ60544.1
Archaea Nanobdellati Microcaldota Candidatus Micrarchaeia archaeon MFA5412282.1
Archaea Nanobdellati Microcaldota Microcaldota archaeon MCK4319685.1
Archaea Nanobdellati Microcaldota Microcaldota archaeon MBN2122110.1
Archaea Nanobdellati Microcaldota Microcaldota archaeon MBD3397670.1
Archaea Nanobdellati Microcaldota Microcaldota archaeon MBD3389423.1
Archaea Nanobdellati Microcaldota Candidatus Micrarchaeia archaeon MFP3950176.1
Archaea Nanobdellati Microcaldota Microcaldota archaeon NYZ77710.1
Archaea Nanobdellati Microcaldota Candidatus Bilamarchaeaceae archaeon MEM2909231.1
Archaea Nanobdellati Microcaldota Microcaldota archaeon MFH1221747.1
Archaea Nanobdellati Microcaldota Candidatus Bilamarchaeaceae archaeon MEM3422550.1
Archaea Nanobdellati Microcaldota Microcaldota archaeon MBD3210889.1
Archaea Nanobdellati Microcaldota Microcaldota archaeon MBU0532434.1
Archaea Nanobdellati Microcaldota Microcaldota archaeon MFH1394026.1
Archaea Nanobdellati Microcaldota Microcaldota archaeon MFH1684751.1
Archaea Nanobdellati Microcaldota Microcaldota archaeon MBU0592097.1
Archaea Nanobdellati Microcaldota Microcaldota archaeon MBI5224173.1
Groups
Outgroup - Bacteria
Nanobdellati:Nanobdellota (12)
Nanobdellati:Microcaldota (96)
Nanobdellati:Other Nanobdellati (48)
Methanobacteriati:Thermoplasmatota (442)
Methanobacteriati:Methanobacteriota (550)
Methanobacteriati:ClassXII
Methanobacteriati:Other Methanobacteriati (44)
Promethearchaeati:Candidatus Heimdallarchaeota (21)
Promethearchaeati:Candidatus Helarchaeota (17)
Promethearchaeati:Candidatus Lokiarchaeota (100)
Promethearchaeati:Other Promethearchaeati (143)
Thermoproteati:Thermoproteota (304)
Thermoproteati:Nitrososphaerota (489)
Thermoproteati:Candidatus Bathyarchaeota (267)
Thermoproteati:Other Thermoproteati (13)

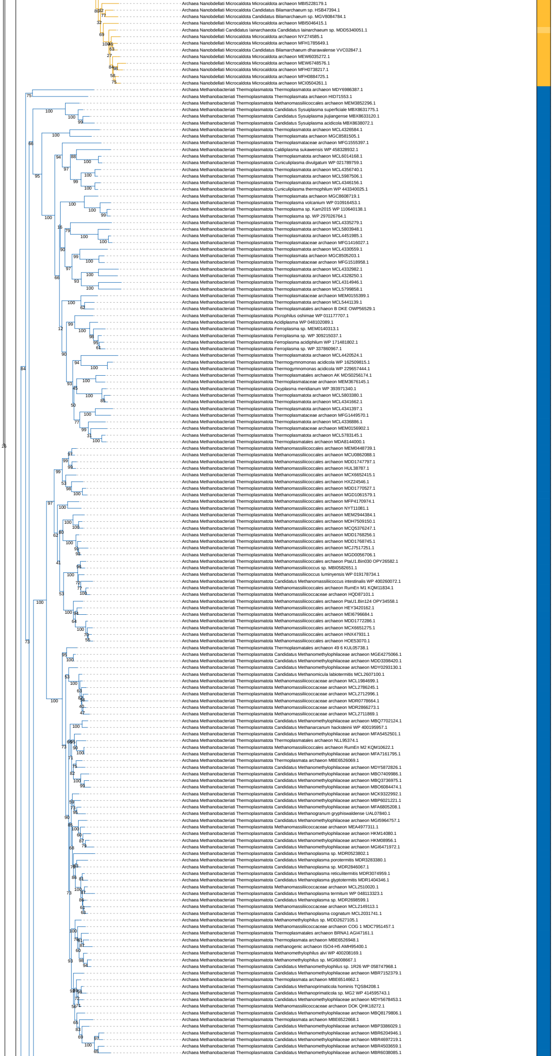
Archaea Nanobdellati Microcaldota Microcaldota archaeon MBI5228179.1
Archaea Nanobdellati Microcaldota Candidatus Bilamarchaeum sp. HSB47394.1
Archaea Nanobdellati Microcaldota Candidatus Bilamarchaeum sp. MGV8084784.1
Archaea Nanobdellati Microcaldota Microcaldota archaeon MBI5046415.1
Archaea Nanobdellati Candidatus Iainarchaeota Candidatus Iainarchaeum sp. MDD5340051.1
Archaea Nanobdellati Microcaldota Microcaldota archaeon NYZ74585.1
Archaea Nanobdellati Microcaldota Microcaldota archaeon MFH1785649.1
Archaea Nanobdellati Microcaldota Candidatus Bilamarchaeum dharawalense VVC02847.1
Archaea Nanobdellati Microcaldota Microcaldota archaeon MEW6035272.1
Archaea Nanobdellati Microcaldota Microcaldota archaeon MEW6748576.1
Archaea Nanobdellati Microcaldota Microcaldota archaeon MFH0738217.1
Archaea Nanobdellati Microcaldota Microcaldota archaeon MFH0884725.1
Archaea Nanobdellati Microcaldota Microcaldota archaeon MCI0504261.1
Archaea Methanobacteriati Thermoplasmatota Thermoplasmatota archaeon MDY6986387.1
Archaea Methanobacteriati Thermoplasmatota Thermoplasmata archaeon HID71553.1
Archaea Methanobacteriati Thermoplasmatota Methanomassiliicoccales archaeon MEM3852296.1
Archaea Methanobacteriati Thermoplasmatota Candidatus Sysuiplasma superficiale MBX8631775.1
Archaea Methanobacteriati Thermoplasmatota Candidatus Sysuiplasma jiujiangense MBX8633120.1
Archaea Methanobacteriati Thermoplasmatota Candidatus Sysuiplasma acidicola MBX8638072.1
Archaea Methanobacteriati Thermoplasmatota Thermoplasmatota archaeon MCL4326584.1
Archaea Methanobacteriati Thermoplasmatota Thermoplasmata archaeon MGC8581505.1
Archaea Methanobacteriati Thermoplasmatota Thermoplasmataceae archaeon MFG1555397.1
Archaea Methanobacteriati Thermoplasmatota Caldiplasma sukawensis WP 458328932.1
Archaea Methanobacteriati Thermoplasmatota Thermoplasmatota archaeon MCL6014168.1
Archaea Methanobacteriati Thermoplasmatota Cuniculiplasma divulgatum WP 021789759.1
Archaea Methanobacteriati Thermoplasmatota Thermoplasmatota archaeon MCL4356740.1
Archaea Methanobacteriati Thermoplasmatota Thermoplasmatota archaeon MCL5987506.1
Archaea Methanobacteriati Thermoplasmatota Thermoplasmatota archaeon MCL4346156.1
Archaea Methanobacteriati Thermoplasmatota Cuniculiplasma thermophilum WP 443340025.1
Archaea Methanobacteriati Thermoplasmatota Thermoplasmata archaeon MGC8608719.1
Archaea Methanobacteriati Thermoplasmatota Thermoplasma volcanium WP 010916453.1
Archaea Methanobacteriati Thermoplasmatota Thermoplasma sp. Kam2015 WP 110640138.1
Archaea Methanobacteriati Thermoplasmatota Thermoplasma sp. WP 297026764.1
Archaea Methanobacteriati Thermoplasmatota Thermoplasmatota archaeon MCL4335279.1
Archaea Methanobacteriati Thermoplasmatota Thermoplasmatota archaeon MCL5803948.1
Archaea Methanobacteriati Thermoplasmatota Thermoplasmatota archaeon MCL4451985.1
Archaea Methanobacteriati Thermoplasmatota Thermoplasmataceae archaeon MFG1416027.1
Archaea Methanobacteriati Thermoplasmatota Thermoplasmatota archaeon MCL4330559.1
Archaea Methanobacteriati Thermoplasmatota Thermoplasmata archaeon MGC8505203.1
Archaea Methanobacteriati Thermoplasmatota Thermoplasmataceae archaeon MFG1518958.1
Archaea Methanobacteriati Thermoplasmatota Thermoplasmatota archaeon MCL4332982.1
Archaea Methanobacteriati Thermoplasmatota Thermoplasmatota archaeon MCL4328250.1
Archaea Methanobacteriati Thermoplasmatota Thermoplasmatota archaeon MCL4314946.1
Archaea Methanobacteriati Thermoplasmatota Thermoplasmatota archaeon MCL5799858.1
Archaea Methanobacteriati Thermoplasmatota Thermoplasmataceae archaeon MEM0155399.1
Archaea Methanobacteriati Thermoplasmatota Thermoplasmatota archaeon MCL5441139.1
Archaea Methanobacteriati Thermoplasmatota Thermoplasmatales archaeon B DKE OWP56529.1
Archaea Methanobacteriati Thermoplasmatota Picrophilus oshimae WP 011177707.1
Archaea Methanobacteriati Thermoplasmatota Acidiplasma WP 048102089.1
Archaea Methanobacteriati Thermoplasmatota Ferroplasma sp. MEM0140313.1
Archaea Methanobacteriati Thermoplasmatota Ferroplasma sp. WP 309215037.1
Archaea Methanobacteriati Thermoplasmatota Ferroplasma acidiphilum WP 171481802.1
Archaea Methanobacteriati Thermoplasmatota Ferroplasma sp. WP 337860967.1
Archaea Methanobacteriati Thermoplasmatota Thermoplasmatota archaeon MCL4420524.1
Archaea Methanobacteriati Thermoplasmatota Thermogymnomonas acidicola WP 162509815.1
Archaea Methanobacteriati Thermoplasmatota Thermogymnomonas acidicola WP 229657444.1
Archaea Methanobacteriati Thermoplasmatota Thermoplasmatales archaeon AK MDS0256174.1
Archaea Methanobacteriati Thermoplasmatota Thermoplasmataceae archaeon MEM3676145.1
Archaea Methanobacteriati Thermoplasmatota Oxyplasma meridianum WP 393971340.1
Archaea Methanobacteriati Thermoplasmatota Thermoplasmatota archaeon MCL5803380.1
Archaea Methanobacteriati Thermoplasmatota Thermoplasmatota archaeon MCL4341662.1
Archaea Methanobacteriati Thermoplasmatota Thermoplasmatota archaeon MCL4341397.1
Archaea Methanobacteriati Thermoplasmatota Thermoplasmataceae archaeon MFG1449570.1
Archaea Methanobacteriati Thermoplasmatota Thermoplasmatota archaeon MCL4336886.1
Archaea Methanobacteriati Thermoplasmatota Thermoplasmataceae archaeon MEM0156902.1
Archaea Methanobacteriati Thermoplasmatota Thermoplasmatota archaeon MCL5783145.1
Archaea Methanobacteriati Thermoplasmatota Thermoplasmatales archaeon MDA8144000.1
Archaea Methanobacteriati Thermoplasmatota Methanomassiliicoccales archaeon MEM0448739.1
Archaea Methanobacteriati Thermoplasmatota Methanomassiliicoccales archaeon MCU0862088.1
Archaea Methanobacteriati Thermoplasmatota Methanomassiliicoccales archaeon MDD1747797.1
Archaea Methanobacteriati Thermoplasmatota Methanomassiliicoccales archaeon HUL38787.1
Archaea Methanobacteriati Thermoplasmatota Methanomassiliicoccales archaeon MCX6652415.1
Archaea Methanobacteriati Thermoplasmatota Methanomassiliicoccales archaeon HXZ24546.1
Archaea Methanobacteriati Thermoplasmatota Methanomassiliicoccales archaeon MDD1770527.1
Archaea Methanobacteriati Thermoplasmatota Methanomassiliicoccales archaeon MGD1061579.1
Archaea Methanobacteriati Thermoplasmatota Methanomassiliicoccales archaeon MFP4170974.1
Archaea Methanobacteriati Thermoplasmatota Methanomassiliicoccales archaeon NYT11081.1
Archaea Methanobacteriati Thermoplasmatota Methanomassiliicoccales archaeon MEM2944384.1
Archaea Methanobacteriati Thermoplasmatota Methanomassiliicoccales archaeon MDH7509150.1
Archaea Methanobacteriati Thermoplasmatota Methanomassiliicoccales archaeon MCQ5376247.1
Archaea Methanobacteriati Thermoplasmatota Methanomassiliicoccales archaeon MDD1768256.1
Archaea Methanobacteriati Thermoplasmatota Methanomassiliicoccales archaeon MDD1768745.1
Archaea Methanobacteriati Thermoplasmatota Methanomassiliicoccales archaeon MCJ7517251.1
Archaea Methanobacteriati Thermoplasmatota Methanomassiliicoccales archaeon MGD0056706.1
Archaea Methanobacteriati Thermoplasmatota Methanomassiliicoccales archaeon PtaU1.Bin030 OPY26582.1
Archaea Methanobacteriati Thermoplasmatota Methanomassiliicoccus sp. MBI0582651.1
Archaea Methanobacteriati Thermoplasmatota Methanomassiliicoccus luminyensis WP 019178734.1
Archaea Methanobacteriati Thermoplasmatota Candidatus Methanomassiliicoccus intestinalis WP 400260072.1
Archaea Methanobacteriati Thermoplasmatota Methanomassiliicoccales archaeon RumEn M1 KQM11834.1
Archaea Methanobacteriati Thermoplasmatota Methanomassiliicoccaceae archaeon HQD87101.1
Archaea Methanobacteriati Thermoplasmatota Methanomassiliicoccales archaeon PtaU1.Bin124 OPY34558.1
Archaea Methanobacteriati Thermoplasmatota Methanomassiliicoccales archaeon HEY3420162.1
Archaea Methanobacteriati Thermoplasmatota Methanomassiliicoccales archaeon MEI6796684.1
Archaea Methanobacteriati Thermoplasmatota Methanomassiliicoccales archaeon MDD1772286.1
Archaea Methanobacteriati Thermoplasmatota Methanomassiliicoccales archaeon MCX6651275.1
Archaea Methanobacteriati Thermoplasmatota Methanomassiliicoccales archaeon HNX47931.1
Archaea Methanobacteriati Thermoplasmatota Methanomassiliicoccales archaeon HOE53070.1
Archaea Methanobacteriati Thermoplasmatota Thermoplasmatales archaeon 49 6 KUL05738.1
Archaea Methanobacteriati Thermoplasmatota Candidatus Methanomethylophilaceae archaeon MGE4275066.1
Archaea Methanobacteriati Thermoplasmatota Candidatus Methanomethylophilaceae archaeon MDD3398420.1
Archaea Methanobacteriati Thermoplasmatota Candidatus Methanomethylophilaceae archaeon MDY0293130.1
Archaea Methanobacteriati Thermoplasmatota Candidatus Methanomicula labiotermitis MCL2607100.1
Archaea Methanobacteriati Thermoplasmatota Methanomassiliicoccaceae archaeon MCL1984699.1
Archaea Methanobacteriati Thermoplasmatota Methanomassiliicoccaceae archaeon MCL2786245.1
Archaea Methanobacteriati Thermoplasmatota Methanomassiliicoccaceae archaeon MCL2712996.1
Archaea Methanobacteriati Thermoplasmatota Methanomassiliicoccaceae archaeon MDR0778664.1
Archaea Methanobacteriati Thermoplasmatota Methanomassiliicoccaceae archaeon MDR2866273.1
Archaea Methanobacteriati Thermoplasmatota Methanomassiliicoccaceae archaeon MCL2711869.1
Archaea Methanobacteriati Thermoplasmatota Candidatus Methanomethylophilaceae archaeon MBQ7702124.1
Archaea Methanobacteriati Thermoplasmatota Candidatus Methanarcanum hacksteinii WP 400195957.1
Archaea Methanobacteriati Thermoplasmatota Candidatus Methanomethylophilaceae archaeon MFA5452501.1
Archaea Methanobacteriati Thermoplasmatota Thermoplasmatales archaeon NLL95374.1
Archaea Methanobacteriati Thermoplasmatota Methanomassiliicoccales archaeon RumEn M2 KQM10622.1
Archaea Methanobacteriati Thermoplasmatota Candidatus Methanomethylophilaceae archaeon MFA7161795.1
Archaea Methanobacteriati Thermoplasmatota Thermoplasmata archaeon MBE6526069.1
Archaea Methanobacteriati Thermoplasmatota Candidatus Methanomethylophilaceae archaeon MDY5872826.1
Archaea Methanobacteriati Thermoplasmatota Candidatus Methanomethylophilaceae archaeon MBO7409986.1
Archaea Methanobacteriati Thermoplasmatota Candidatus Methanomethylophilaceae archaeon MBQ3736975.1
Archaea Methanobacteriati Thermoplasmatota Candidatus Methanomethylophilaceae archaeon MBO6084474.1
Archaea Methanobacteriati Thermoplasmatota Candidatus Methanomethylophilaceae archaeon MCK9322992.1
Archaea Methanobacteriati Thermoplasmatota Candidatus Methanomethylophilaceae archaeon MBP6021221.1
Archaea Methanobacteriati Thermoplasmatota Candidatus Methanomethylophilaceae archaeon MFA6805208.1
Archaea Methanobacteriati Thermoplasmatota Candidatus Methanogranum gryphiswaldense UAL07840.1
Archaea Methanobacteriati Thermoplasmatota Candidatus Methanomethylophilaceae archaeon MGI5964757.1
Archaea Methanobacteriati Thermoplasmatota Methanomassiliicoccaceae archaeon MEA4977311.1
Archaea Methanobacteriati Thermoplasmatota Candidatus Methanomethylophilaceae archaeon HKM14080.1
Archaea Methanobacteriati Thermoplasmatota Candidatus Methanomethylophilaceae archaeon HKM08956.1
Archaea Methanobacteriati Thermoplasmatota Candidatus Methanomethylophilaceae archaeon MGI6471972.1
Archaea Methanobacteriati Thermoplasmatota Candidatus Methanoplasma sp. MDR0523802.1
Archaea Methanobacteriati Thermoplasmatota Candidatus Methanoplasma porotermitis MDR3283380.1
Archaea Methanobacteriati Thermoplasmatota Candidatus Methanoplasma sp. MDR2846067.1
Archaea Methanobacteriati Thermoplasmatota Candidatus Methanoplasma reticulitermitis MDR3074959.1
Archaea Methanobacteriati Thermoplasmatota Candidatus Methanoplasma glyptotermitis MDR1404346.1
Archaea Methanobacteriati Thermoplasmatota Methanomassiliicoccaceae archaeon MCL2510020.1
Archaea Methanobacteriati Thermoplasmatota Candidatus Methanoplasma termitum WP 048113323.1
Archaea Methanobacteriati Thermoplasmatota Candidatus Methanoplasma sp. MDR2698599.1
Archaea Methanobacteriati Thermoplasmatota Methanomassiliicoccaceae archaeon MCL2149113.1
Archaea Methanobacteriati Thermoplasmatota Candidatus Methanoplasma cognatum MCL2031741.1
Archaea Methanobacteriati Thermoplasmatota Methanomethylophilus sp. MDD2627105.1
Archaea Methanobacteriati Thermoplasmatota Methanomassiliicoccaceae archaeon COG 1 MDC7951457.1
Archaea Methanobacteriati Thermoplasmatota Thermoplasmatales archaeon BRNA1 AGI47161.1
Archaea Methanobacteriati Thermoplasmatota Thermoplasmata archaeon MBE6526948.1
Archaea Methanobacteriati Thermoplasmatota methanogenic archaeon ISO4-H5 AMH95400.1
Archaea Methanobacteriati Thermoplasmatota Methanomethylophilus alvi WP 400208169.1
Archaea Methanobacteriati Thermoplasmatota Methanomethylophilus sp. MGI6008667.1
Archaea Methanobacteriati Thermoplasmatota Candidatus Methanomethylophilus sp. 1R26 WP 058747968.1
Archaea Methanobacteriati Thermoplasmatota Candidatus Methanomethylophilaceae archaeon MBR7152379.1
Archaea Methanobacteriati Thermoplasmatota Thermoplasmata archaeon MBE6514662.1
Archaea Methanobacteriati Thermoplasmatota Candidatus Methanoprimaticola hominis TQS84208.1
Archaea Methanobacteriati Thermoplasmatota Candidatus Methanoprimaticola sp. MG2 WP 414595743.1
Archaea Methanobacteriati Thermoplasmatota Candidatus Methanomethylophilaceae archaeon MDY5678453.1
Archaea Methanobacteriati Thermoplasmatota Methanomassiliicoccaceae archaeon DOK QHK18272.1
Archaea Methanobacteriati Thermoplasmatota Candidatus Methanomethylophilaceae archaeon MBQ8179806.1
Archaea Methanobacteriati Thermoplasmatota Thermoplasmata archaeon MBE6522668.1
Archaea Methanobacteriati Thermoplasmatota Candidatus Methanomethylophilaceae archaeon MBP3386029.1
Archaea Methanobacteriati Thermoplasmatota Candidatus Methanomethylophilaceae archaeon MBR6204946.1
Archaea Methanobacteriati Thermoplasmatota Candidatus Methanomethylophilaceae archaeon MBR4697219.1
Archaea Methanobacteriati Thermoplasmatota Candidatus Methanomethylophilaceae archaeon MBR4503659.1
Archaea Methanobacteriati Thermoplasmatota Candidatus Methanomethylophilaceae archaeon MBR6038085.1

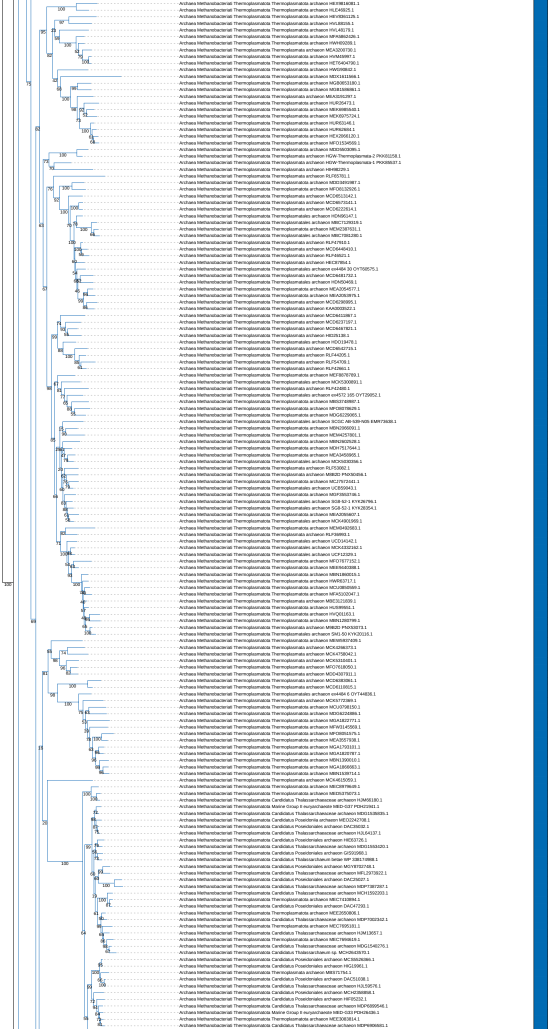
Archaea Methanobacteriati Thermoplasmatota Thermoplasmatota archaeon HEX9816081.1
Archaea Methanobacteriati Thermoplasmatota Thermoplasmatota archaeon HLE46925.1
Archaea Methanobacteriati Thermoplasmatota Thermoplasmatota archaeon HEV8361125.1
Archaea Methanobacteriati Thermoplasmatota Thermoplasmatota archaeon HVL88155.1
Archaea Methanobacteriati Thermoplasmatota Thermoplasmatota archaeon HVL48179.1
Archaea Methanobacteriati Thermoplasmatota Thermoplasmatota archaeon MFA5862426.1
Archaea Methanobacteriati Thermoplasmatota Thermoplasmatota archaeon HWH09289.1
Archaea Methanobacteriati Thermoplasmatota Thermoplasmata archaeon MEA3200730.1
Archaea Methanobacteriati Thermoplasmatota Thermoplasmatota archaeon HVM45997.1
Archaea Methanobacteriati Thermoplasmatota Thermoplasmatota archaeon HET6404790.1
Archaea Methanobacteriati Thermoplasmatota Thermoplasmatota archaeon HWG90842.1
Archaea Methanobacteriati Thermoplasmatota Thermoplasmatota archaeon MDX1611566.1
Archaea Methanobacteriati Thermoplasmatota Thermoplasmatota archaeon MGB0653180.1
Archaea Methanobacteriati Thermoplasmatota Thermoplasmatota archaeon MGB1586861.1
Archaea Methanobacteriati Thermoplasmatota Thermoplasmata archaeon MEA3191297.1
Archaea Methanobacteriati Thermoplasmatota Thermoplasmatota archaeon HUR26473.1
Archaea Methanobacteriati Thermoplasmatota Thermoplasmatota archaeon MEK6985540.1
Archaea Methanobacteriati Thermoplasmatota Thermoplasmatota archaeon MEK6975724.1
Archaea Methanobacteriati Thermoplasmatota Thermoplasmatota archaeon HUR63146.1
Archaea Methanobacteriati Thermoplasmatota Thermoplasmatota archaeon HUR62684.1
Archaea Methanobacteriati Thermoplasmatota Thermoplasmatota archaeon HEX2066120.1
Archaea Methanobacteriati Thermoplasmatota Thermoplasmatota archaeon MFO1534569.1
Archaea Methanobacteriati Thermoplasmatota Thermoplasmatota archaeon MDD5503095.1
Archaea Methanobacteriati Thermoplasmatota Thermoplasmata archaeon HGW-Thermoplasmata-2 PKK81158.1
Archaea Methanobacteriati Thermoplasmatota Thermoplasmata archaeon HGW-Thermoplasmata-1 PKK85537.1
Archaea Methanobacteriati Thermoplasmatota Thermoplasmata archaeon HIH98229.1
Archaea Methanobacteriati Thermoplasmatota Thermoplasmata archaeon RLF65781.1
Archaea Methanobacteriati Thermoplasmatota Thermoplasmatota archaeon MDD3491987.1
Archaea Methanobacteriati Thermoplasmatota Thermoplasmatota archaeon MFO8132926.1
Archaea Methanobacteriati Thermoplasmatota Thermoplasmata archaeon MCD6513142.1
Archaea Methanobacteriati Thermoplasmatota Thermoplasmata archaeon MCD6573141.1
Archaea Methanobacteriati Thermoplasmatota Thermoplasmata archaeon MCD6222614.1
Archaea Methanobacteriati Thermoplasmatota Thermoplasmatales archaeon HDN96147.1
Archaea Methanobacteriati Thermoplasmatota Thermoplasmatales archaeon MBC7129319.1
Archaea Methanobacteriati Thermoplasmatota Thermoplasmatota archaeon MEM2387631.1
Archaea Methanobacteriati Thermoplasmatota Thermoplasmatales archaeon MBC7081280.1
Archaea Methanobacteriati Thermoplasmatota Thermoplasmata archaeon RLF47910.1
Archaea Methanobacteriati Thermoplasmatota Thermoplasmata archaeon MCD6448410.1
Archaea Methanobacteriati Thermoplasmatota Thermoplasmata archaeon RLF46521.1
Archaea Methanobacteriati Thermoplasmatota Thermoplasmata archaeon HEC87854.1
Archaea Methanobacteriati Thermoplasmatota Thermoplasmatales archaeon ex4484 30 OYT60575.1
Archaea Methanobacteriati Thermoplasmatota Thermoplasmata archaeon MCD6481732.1
Archaea Methanobacteriati Thermoplasmatota Thermoplasmatales archaeon HDN50469.1
Archaea Methanobacteriati Thermoplasmatota Thermoplasmatota archaeon MEA2054577.1
Archaea Methanobacteriati Thermoplasmatota Thermoplasmatota archaeon MEA2053975.1
Archaea Methanobacteriati Thermoplasmatota Thermoplasmata archaeon MCD6298995.1
Archaea Methanobacteriati Thermoplasmatota Thermoplasmata archaeon KAA0003522.1
Archaea Methanobacteriati Thermoplasmatota Thermoplasmata archaeon MCD6411867.1
Archaea Methanobacteriati Thermoplasmatota Thermoplasmata archaeon MCD6237197.1
Archaea Methanobacteriati Thermoplasmatota Thermoplasmata archaeon MCD6467821.1
Archaea Methanobacteriati Thermoplasmatota Thermoplasmata archaeon HID25138.1
Archaea Methanobacteriati Thermoplasmatota Thermoplasmatales archaeon HDO19478.1
Archaea Methanobacteriati Thermoplasmatota Thermoplasmata archaeon MCD6542715.1
Archaea Methanobacteriati Thermoplasmatota Thermoplasmata archaeon RLF44205.1
Archaea Methanobacteriati Thermoplasmatota Thermoplasmata archaeon RLF54709.1
Archaea Methanobacteriati Thermoplasmatota Thermoplasmata archaeon RLF42661.1
Archaea Methanobacteriati Thermoplasmatota Thermoplasmatota archaeon MEF8878789.1
Archaea Methanobacteriati Thermoplasmatota Thermoplasmatales archaeon MCK5300891.1
Archaea Methanobacteriati Thermoplasmatota Thermoplasmata archaeon RLF42480.1
Archaea Methanobacteriati Thermoplasmatota Thermoplasmatales archaeon ex4572 165 OYT29052.1
Archaea Methanobacteriati Thermoplasmatota Thermoplasmatota archaeon MBS3748987.1
Archaea Methanobacteriati Thermoplasmatota Thermoplasmatota archaeon MFO8078629.1
Archaea Methanobacteriati Thermoplasmatota Thermoplasmatota archaeon MDG6229065.1
Archaea Methanobacteriati Thermoplasmatota Thermoplasmatales archaeon SCGC AB-539-N05 EMR73638.1
Archaea Methanobacteriati Thermoplasmatota Thermoplasmatota archaeon MBN2066091.1
Archaea Methanobacteriati Thermoplasmatota Thermoplasmatota archaeon MEM4257801.1
Archaea Methanobacteriati Thermoplasmatota Thermoplasmatota archaeon MBN2602528.1
Archaea Methanobacteriati Thermoplasmatota Thermoplasmatota archaeon MDH7517644.1
Archaea Methanobacteriati Thermoplasmatota Thermoplasmatota archaeon MEA3458965.1
Archaea Methanobacteriati Thermoplasmatota Thermoplasmatales archaeon MCK5030356.1
Archaea Methanobacteriati Thermoplasmatota Thermoplasmata archaeon RLF53082.1
Archaea Methanobacteriati Thermoplasmatota Thermoplasmata archaeon M8B2D PNX50456.1
Archaea Methanobacteriati Thermoplasmatota Thermoplasmatota archaeon MCJ7572441.1
Archaea Methanobacteriati Thermoplasmatota Thermoplasmatales archaeon UCB59043.1
Archaea Methanobacteriati Thermoplasmatota Thermoplasmatota archaeon MGF3553746.1
Archaea Methanobacteriati Thermoplasmatota Thermoplasmatales archaeon SG8-52-1 KYK26796.1
Archaea Methanobacteriati Thermoplasmatota Thermoplasmatales archaeon SG8-52-1 KYK28354.1
Archaea Methanobacteriati Thermoplasmatota Thermoplasmatota archaeon MEA2055607.1
Archaea Methanobacteriati Thermoplasmatota Thermoplasmatales archaeon MCK4901969.1
Archaea Methanobacteriati Thermoplasmatota Thermoplasmatota archaeon MEM0492683.1
Archaea Methanobacteriati Thermoplasmatota Thermoplasmata archaeon RLF36993.1
Archaea Methanobacteriati Thermoplasmatota Thermoplasmatales archaeon UCD14142.1
Archaea Methanobacteriati Thermoplasmatota Thermoplasmatales archaeon MCK4332162.1
Archaea Methanobacteriati Thermoplasmatota Thermoplasmatales archaeon UCF12329.1
Archaea Methanobacteriati Thermoplasmatota Thermoplasmatota archaeon MFO7677152.1
Archaea Methanobacteriati Thermoplasmatota Thermoplasmatota archaeon MEE9440388.1
Archaea Methanobacteriati Thermoplasmatota Thermoplasmatota archaeon MBN1860015.1
Archaea Methanobacteriati Thermoplasmatota Thermoplasmatota archaeon HWR63717.1
Archaea Methanobacteriati Thermoplasmatota Thermoplasmatota archaeon MCU0850559.1
Archaea Methanobacteriati Thermoplasmatota Thermoplasmatota archaeon MFA5102047.1
Archaea Methanobacteriati Thermoplasmatota Thermoplasmata archaeon MBE3121839.1
Archaea Methanobacteriati Thermoplasmatota Thermoplasmatota archaeon HUS99551.1
Archaea Methanobacteriati Thermoplasmatota Thermoplasmatota archaeon HVQ01163.1
Archaea Methanobacteriati Thermoplasmatota Thermoplasmatota archaeon MBN1280799.1
Archaea Methanobacteriati Thermoplasmatota Thermoplasmata archaeon M9B2D PNX53073.1
Archaea Methanobacteriati Thermoplasmatota Thermoplasmatales archaeon SM1-50 KYK20116.1
Archaea Methanobacteriati Thermoplasmatota Thermoplasmatota archaeon MEW5937409.1
Archaea Methanobacteriati Thermoplasmatota Thermoplasmata archaeon MCK4266373.1
Archaea Methanobacteriati Thermoplasmatota Thermoplasmata archaeon MCK4758042.1
Archaea Methanobacteriati Thermoplasmatota Thermoplasmata archaeon MCK5310401.1
Archaea Methanobacteriati Thermoplasmatota Thermoplasmata archaeon MFO7618050.1
Archaea Methanobacteriati Thermoplasmatota Thermoplasmata archaeon MDD4307911.1
Archaea Methanobacteriati Thermoplasmatota Thermoplasmata archaeon MCD6383061.1
Archaea Methanobacteriati Thermoplasmatota Thermoplasmata archaeon MCD6110815.1
Archaea Methanobacteriati Thermoplasmatota Thermoplasmatales archaeon ex4484 6 OYT44836.1
Archaea Methanobacteriati Thermoplasmatota Thermoplasmata archaeon MCK5772369.1
Archaea Methanobacteriati Thermoplasmatota Thermoplasmatota archaeon MCU0798150.1
Archaea Methanobacteriati Thermoplasmatota Thermoplasmatota archaeon MDG6224886.1
Archaea Methanobacteriati Thermoplasmatota Thermoplasmatota archaeon MGA1822771.1
Archaea Methanobacteriati Thermoplasmatota Thermoplasmatota archaeon MFW3145569.1
Archaea Methanobacteriati Thermoplasmatota Thermoplasmatota archaeon MFO8051575.1
Archaea Methanobacteriati Thermoplasmatota Thermoplasmatota archaeon MEA3557938.1
Archaea Methanobacteriati Thermoplasmatota Thermoplasmatota archaeon MGA1793101.1
Archaea Methanobacteriati Thermoplasmatota Thermoplasmatota archaeon MGA1820787.1
Archaea Methanobacteriati Thermoplasmatota Thermoplasmatota archaeon MBN1390010.1
Archaea Methanobacteriati Thermoplasmatota Thermoplasmatota archaeon MGA1866663.1
Archaea Methanobacteriati Thermoplasmatota Thermoplasmatota archaeon MBN1539714.1
Archaea Methanobacteriati Thermoplasmatota Thermoplasmata archaeon MCK4615059.1
Archaea Methanobacteriati Thermoplasmatota Thermoplasmatota archaeon MEC8979649.1
Archaea Methanobacteriati Thermoplasmatota Thermoplasmatota archaeon MED5375073.1
Archaea Methanobacteriati Thermoplasmatota Candidatus Thalassarchaeaceae archaeon HJM66180.1
Archaea Methanobacteriati Thermoplasmatota Marine Group II euryarchaeote MED-G37 PDH21941.1
Archaea Methanobacteriati Thermoplasmatota Candidatus Thalassarchaeaceae archaeon MDG1535835.1
Archaea Methanobacteriati Thermoplasmatota Candidatus Poseidoniia archaeon MEO2242708.1
Archaea Methanobacteriati Thermoplasmatota Candidatus Poseidoniales archaeon DAC35032.1
Archaea Methanobacteriati Thermoplasmatota Candidatus Thalassarchaeaceae archaeon HJL64137.1
Archaea Methanobacteriati Thermoplasmatota Candidatus Poseidoniales archaeon HIE63726.1
Archaea Methanobacteriati Thermoplasmatota Candidatus Thalassarchaeaceae archaeon MDG1553420.1
Archaea Methanobacteriati Thermoplasmatota Candidatus Poseidoniales archaeon GIS91968.1
Archaea Methanobacteriati Thermoplasmatota Candidatus Thalassarchaeum betae WP 338174988.1
Archaea Methanobacteriati Thermoplasmatota Candidatus Poseidoniales archaeon MGY8702748.1
Archaea Methanobacteriati Thermoplasmatota Candidatus Thalassarchaeaceae archaeon MFL2973922.1
Archaea Methanobacteriati Thermoplasmatota Candidatus Poseidoniales archaeon DAC25027.1
Archaea Methanobacteriati Thermoplasmatota Candidatus Thalassarchaeaceae archaeon MDP7387287.1
Archaea Methanobacteriati Thermoplasmatota Candidatus Thalassarchaeaceae archaeon MCH1592203.1
Archaea Methanobacteriati Thermoplasmatota Thermoplasmatota archaeon MEC7410894.1
Archaea Methanobacteriati Thermoplasmatota Candidatus Poseidoniales archaeon DAC47293.1
Archaea Methanobacteriati Thermoplasmatota Thermoplasmatota archaeon MEE2650806.1
Archaea Methanobacteriati Thermoplasmatota Candidatus Thalassarchaeaceae archaeon MDP7002342.1
Archaea Methanobacteriati Thermoplasmatota Thermoplasmatota archaeon MEC7695181.1
Archaea Methanobacteriati Thermoplasmatota Candidatus Thalassarchaeaceae archaeon HJM13657.1
Archaea Methanobacteriati Thermoplasmatota Thermoplasmatota archaeon MEC7694619.1
Archaea Methanobacteriati Thermoplasmatota Candidatus Thalassarchaeaceae archaeon MDG1540276.1
Archaea Methanobacteriati Thermoplasmatota Candidatus Thalassarchaeum sp. MCH2643570.1
Archaea Methanobacteriati Thermoplasmatota Candidatus Poseidoniales archaeon MCS5526366.1
Archaea Methanobacteriati Thermoplasmatota Candidatus Poseidoniales archaeon HIG19961.1
Archaea Methanobacteriati Thermoplasmatota Thermoplasmata archaeon MBS71754.1
Archaea Methanobacteriati Thermoplasmatota Candidatus Poseidoniales archaeon DAC51038.1
Archaea Methanobacteriati Thermoplasmatota Candidatus Thalassarchaeaceae archaeon HJL59576.1
Archaea Methanobacteriati Thermoplasmatota Candidatus Poseidoniales archaeon MCH2358858.1
Archaea Methanobacteriati Thermoplasmatota Candidatus Poseidoniales archaeon HIF05232.1
Archaea Methanobacteriati Thermoplasmatota Candidatus Thalassarchaeaceae archaeon MDP6899546.1
Archaea Methanobacteriati Thermoplasmatota Marine Group II euryarchaeote MED-G33 PDH26436.1
Archaea Methanobacteriati Thermoplasmatota Thermoplasmatota archaeon MEE3083814.1
Archaea Methanobacteriati Thermoplasmatota Candidatus Thalassarchaeaceae archaeon MDP6906581.1

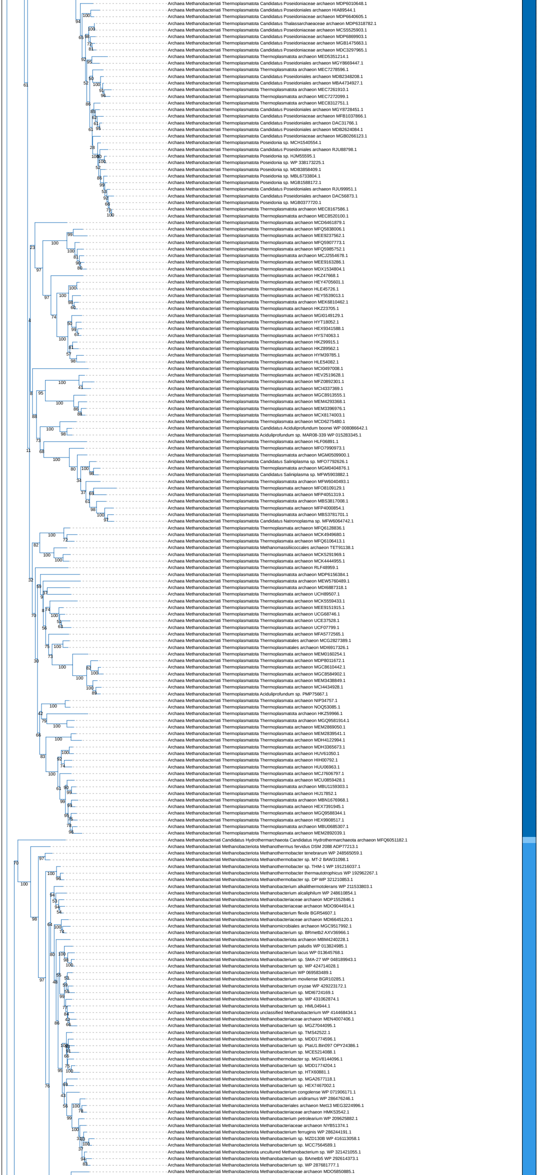

Archaea Methanobacteriati Thermoplasmatota Candidatus Poseidoniaceae archaeon MDP6010648.1
Archaea Methanobacteriati Thermoplasmatota Candidatus Poseidoniales archaeon HIA89544.1
Archaea Methanobacteriati Thermoplasmatota Candidatus Poseidoniaceae archaeon MDP6640605.1
Archaea Methanobacteriati Thermoplasmatota Candidatus Thalassarchaeaceae archaeon MDP6318782.1
Archaea Methanobacteriati Thermoplasmatota Candidatus Poseidoniaceae archaeon MCS5525903.1
Archaea Methanobacteriati Thermoplasmatota Candidatus Poseidoniaceae archaeon MDP6869903.1
Archaea Methanobacteriati Thermoplasmatota Candidatus Poseidoniaceae archaeon MGB1475663.1
Archaea Methanobacteriati Thermoplasmatota Candidatus Poseidoniaceae archaeon MDC3297965.1
Archaea Methanobacteriati Thermoplasmatota Thermoplasmatota archaeon MED5351214.1
Archaea Methanobacteriati Thermoplasmatota Candidatus Poseidoniales archaeon MGY8669447.1
Archaea Methanobacteriati Thermoplasmatota Thermoplasmatota archaeon MEC7278596.1
Archaea Methanobacteriati Thermoplasmatota Candidatus Poseidoniaceae archaeon MDB2348208.1
Archaea Methanobacteriati Thermoplasmatota Candidatus Poseidoniales archaeon MBA4734927.1
Archaea Methanobacteriati Thermoplasmatota Thermoplasmatota archaeon MEC7261910.1
Archaea Methanobacteriati Thermoplasmatota Thermoplasmatota archaeon MEC7272099.1
Archaea Methanobacteriati Thermoplasmatota Thermoplasmatota archaeon MEC8312751.1
Archaea Methanobacteriati Thermoplasmatota Candidatus Poseidoniales archaeon MGY8728451.1
Archaea Methanobacteriati Thermoplasmatota Candidatus Poseidoniaceae archaeon MFB1037866.1
Archaea Methanobacteriati Thermoplasmatota Candidatus Poseidoniales archaeon DAC31766.1
Archaea Methanobacteriati Thermoplasmatota Candidatus Poseidoniales archaeon MDB2624084.1
Archaea Methanobacteriati Thermoplasmatota Candidatus Poseidoniaceae archaeon MGB0266123.1
Archaea Methanobacteriati Thermoplasmatota Poseidonia sp. MCH1540554.1
Archaea Methanobacteriati Thermoplasmatota Candidatus Poseidoniales archaeon RJU88798.1
Archaea Methanobacteriati Thermoplasmatota Poseidonia sp. HJM55595.1
Archaea Methanobacteriati Thermoplasmatota Poseidonia sp. WP 338173225.1
Archaea Methanobacteriati Thermoplasmatota Poseidonia sp. MDB3858409.1
Archaea Methanobacteriati Thermoplasmatota Poseidonia sp. MBL6733804.1
Archaea Methanobacteriati Thermoplasmatota Poseidonia sp. MGB1588172.1
Archaea Methanobacteriati Thermoplasmatota Candidatus Poseidoniales archaeon RJU99951.1
Archaea Methanobacteriati Thermoplasmatota Candidatus Poseidoniales archaeon DAC56873.1
Archaea Methanobacteriati Thermoplasmatota Poseidonia sp. MGB0377720.1
Archaea Methanobacteriati Thermoplasmatota Thermoplasmatota archaeon MEC8167586.1
Archaea Methanobacteriati Thermoplasmatota Thermoplasmatota archaeon MEC8520100.1
Archaea Methanobacteriati Thermoplasmatota Thermoplasmata archaeon MCD6461879.1
Archaea Methanobacteriati Thermoplasmatota Thermoplasmata archaeon MFQ5838006.1
Archaea Methanobacteriati Thermoplasmatota Thermoplasmata archaeon MEE9237562.1
Archaea Methanobacteriati Thermoplasmatota Thermoplasmata archaeon MFQ5907773.1
Archaea Methanobacteriati Thermoplasmatota Thermoplasmata archaeon MFQ5985752.1
Archaea Methanobacteriati Thermoplasmatota Thermoplasmata archaeon MCJ2554678.1
Archaea Methanobacteriati Thermoplasmatota Thermoplasmata archaeon MEE9163286.1
Archaea Methanobacteriati Thermoplasmatota Thermoplasmata archaeon MDX1534804.1
Archaea Methanobacteriati Thermoplasmatota Thermoplasmata archaeon HKZ47668.1
Archaea Methanobacteriati Thermoplasmatota Thermoplasmata archaeon HEY4705601.1
Archaea Methanobacteriati Thermoplasmatota Thermoplasmata archaeon HLE45726.1
Archaea Methanobacteriati Thermoplasmatota Thermoplasmata archaeon HEY5539013.1
Archaea Methanobacteriati Thermoplasmatota Thermoplasmata archaeon MEK6810462.1
Archaea Methanobacteriati Thermoplasmatota Thermoplasmata archaeon HKZ23705.1
Archaea Methanobacteriati Thermoplasmatota Thermoplasmata archaeon MGI0149129.1
Archaea Methanobacteriati Thermoplasmatota Thermoplasmata archaeon HYT18052.1
Archaea Methanobacteriati Thermoplasmatota Thermoplasmata archaeon HEX9341588.1
Archaea Methanobacteriati Thermoplasmatota Thermoplasmata archaeon HYS74063.1
Archaea Methanobacteriati Thermoplasmatota Thermoplasmata archaeon HKZ99915.1
Archaea Methanobacteriati Thermoplasmatota Thermoplasmata archaeon HKZ89562.1
Archaea Methanobacteriati Thermoplasmatota Thermoplasmata archaeon HYM39785.1
Archaea Methanobacteriati Thermoplasmatota Thermoplasmata archaeon HLE54082.1
Archaea Methanobacteriati Thermoplasmatota Thermoplasmata archaeon MCI0497008.1
Archaea Methanobacteriati Thermoplasmatota Thermoplasmata archaeon HEV2519628.1
Archaea Methanobacteriati Thermoplasmatota Thermoplasmata archaeon MFZ0892301.1
Archaea Methanobacteriati Thermoplasmatota Thermoplasmata archaeon MCI4337369.1
Archaea Methanobacteriati Thermoplasmatota Thermoplasmata archaeon MGC8913555.1
Archaea Methanobacteriati Thermoplasmatota Thermoplasmata archaeon MEM4293368.1
Archaea Methanobacteriati Thermoplasmatota Thermoplasmata archaeon MEM3396976.1
Archaea Methanobacteriati Thermoplasmatota Thermoplasmata archaeon MCX8174003.1
Archaea Methanobacteriati Thermoplasmatota Thermoplasmata archaeon MCD6275480.1
Archaea Methanobacteriati Thermoplasmatota Candidatus Aciduliprofundum boonei WP 008086642.1
Archaea Methanobacteriati Thermoplasmatota Aciduliprofundum sp. MAR08-339 WP 015283345.1
Archaea Methanobacteriati Thermoplasmatota Thermoplasmata archaeon HLF06891.1
Archaea Methanobacteriati Thermoplasmatota Thermoplasmata archaeon MFO7990973.1
Archaea Methanobacteriati Thermoplasmatota Thermoplasmata archaeon MGM0509900.1
Archaea Methanobacteriati Thermoplasmatota Candidatus Salinipiasma sp. MFO7792626.1
Archaea Methanobacteriati Thermoplasmatota Thermoplasmata archaeon MGM0404876.1
Archaea Methanobacteriati Thermoplasmatota Candidatus Salinipiasma sp. MFW5903882.1
Archaea Methanobacteriati Thermoplasmatota Thermoplasmatota archaeon MFW6040493.1
Archaea Methanobacteriati Thermoplasmatota Thermoplasmata archaeon MFO8109129.1
Archaea Methanobacteriati Thermoplasmatota Thermoplasmata archaeon MFP4051319.1
Archaea Methanobacteriati Thermoplasmatota Thermoplasmata archaeon MBS3817008.1
Archaea Methanobacteriati Thermoplasmatota Thermoplasmata archaeon MFP4000854.1
Archaea Methanobacteriati Thermoplasmatota Thermoplasmata archaeon MBS3781701.1
Archaea Methanobacteriati Thermoplasmatota Candidatus Natronoplasma sp. MFW6064742.1
Archaea Methanobacteriati Thermoplasmatota Thermoplasmata archaeon MFQ6128836.1
Archaea Methanobacteriati Thermoplasmatota Thermoplasmata archaeon MCK4949680.1
Archaea Methanobacteriati Thermoplasmatota Thermoplasmata archaeon MFQ6106413.1
Archaea Methanobacteriati Thermoplasmatota Methanomassiliicoccales archaeon TET91138.1
Archaea Methanobacteriati Thermoplasmatota Thermoplasmata archaeon MCK5291969.1
Archaea Methanobacteriati Thermoplasmatota Thermoplasmata archaeon MCK4444955.1
Archaea Methanobacteriati Thermoplasmatota Thermoplasmata archaeon RLF48959.1
Archaea Methanobacteriati Thermoplasmatota Thermoplasmata archaeon MDP6156384.1
Archaea Methanobacteriati Thermoplasmatota Thermoplasmata archaeon MEW5760489.1
Archaea Methanobacteriati Thermoplasmatota Thermoplasmata archaeon MDI6887318.1
Archaea Methanobacteriati Thermoplasmatota Thermoplasmata archaeon UCH89507.1
Archaea Methanobacteriati Thermoplasmatota Thermoplasmata archaeon MCK5559433.1
Archaea Methanobacteriati Thermoplasmatota Thermoplasmata archaeon MEE9151915.1
Archaea Methanobacteriati Thermoplasmatota Thermoplasmata archaeon UCG68746.1
Archaea Methanobacteriati Thermoplasmatota Thermoplasmata archaeon UCE37528.1
Archaea Methanobacteriati Thermoplasmatota Thermoplasmata archaeon UCF07799.1
Archaea Methanobacteriati Thermoplasmatota Thermoplasmata archaeon MFA5772565.1
Archaea Methanobacteriati Thermoplasmatota Thermoplasmatales archaeon MCG2827389.1
Archaea Methanobacteriati Thermoplasmatota Thermoplasmatales archaeon MDI6917326.1
Archaea Methanobacteriati Thermoplasmatota Thermoplasmata archaeon MEM0160254.1
Archaea Methanobacteriati Thermoplasmatota Thermoplasmata archaeon MDP8011672.1
Archaea Methanobacteriati Thermoplasmatota Thermoplasmata archaeon MGC8610442.1
Archaea Methanobacteriati Thermoplasmatota Thermoplasmata archaeon MGC8584902.1
Archaea Methanobacteriati Thermoplasmatota Thermoplasmata archaeon MEM3438849.1
Archaea Methanobacteriati Thermoplasmatota Thermoplasmata archaeon MCI4434928.1
Archaea Methanobacteriati Thermoplasmatota Aciduliprofundum sp. PMP75667.1
Archaea Methanobacteriati Thermoplasmatota Thermoplasmata archaeon NIP34757.1
Archaea Methanobacteriati Thermoplasmatota Thermoplasmata archaeon NOQ53085.1
Archaea Methanobacteriati Thermoplasmatota Thermoplasmata archaeon HKZ59966.1
Archaea Methanobacteriati Thermoplasmatota Thermoplasmata archaeon MGQ9581914.1
Archaea Methanobacteriati Thermoplasmatota Thermoplasmata archaeon MEM2869050.1
Archaea Methanobacteriati Thermoplasmatota Thermoplasmata archaeon MEM2839541.1
Archaea Methanobacteriati Thermoplasmatota Thermoplasmata archaeon MDH4122994.1
Archaea Methanobacteriati Thermoplasmatota Thermoplasmata archaeon MDH3365673.1
Archaea Methanobacteriati Thermoplasmatota Thermoplasmata archaeon HUV61050.1
Archaea Methanobacteriati Thermoplasmatota Thermoplasmata archaeon HIH00792.1
Archaea Methanobacteriati Thermoplasmatota Thermoplasmata archaeon HUU06963.1
Archaea Methanobacteriati Thermoplasmatota Thermoplasmata archaeon MCJ7606797.1
Archaea Methanobacteriati Thermoplasmatota Thermoplasmata archaeon MCU0859428.1
Archaea Methanobacteriati Thermoplasmatota Thermoplasmata archaeon MBU1159303.1
Archaea Methanobacteriati Thermoplasmatota Thermoplasmata archaeon HIJ17852.1
Archaea Methanobacteriati Thermoplasmatota Thermoplasmata archaeon MBN1676968.1
Archaea Methanobacteriati Thermoplasmatota Thermoplasmata archaeon HEX7391945.1
Archaea Methanobacteriati Thermoplasmatota Thermoplasmata archaeon MGQ9588344.1
Archaea Methanobacteriati Thermoplasmatota Thermoplasmata archaeon HEX9908517.1
Archaea Methanobacteriati Thermoplasmatota Thermoplasmata archaeon MBU0685307.1
Archaea Methanobacteriati Thermoplasmatota Thermoplasmata archaeon MEM2892039.1
Archaea Methanobacteriati Candidatus Hydrothermarchaeota Candidatus Hydrothermarchaeota archaeon MFQ6051182.1
Archaea Methanobacteriati Methanobacteriota Methanothermus fervidus DSM 2088 ADP77213.1
Archaea Methanobacteriati Methanobacteriota Methanothermobacter tenebrarum WP 248565059.1
Archaea Methanobacteriati Methanobacteriota Methanothermobacter sp. MT-2 BAW31098.1
Archaea Methanobacteriati Methanobacteriota Methanothermobacter sp. THM-1 WP 191216037.1
Archaea Methanobacteriati Methanobacteriota Methanothermobacter thermautotrophicus WP 192962267.1
Archaea Methanobacteriati Methanobacteriota Methanothermobacter sp. DP WP 321210853.1
Archaea Methanobacteriati Methanobacteriota Methanobacterium alkalithermotolerans WP 211533803.1
Archaea Methanobacteriati Methanobacteriota Methanobacterium alcaliphilum WP 248610854.1
Archaea Methanobacteriati Methanobacteriota Methanobacteriaceae archaeon MDP1552846.1
Archaea Methanobacteriati Methanobacteriota Methanobacteriaceae archaeon MDO9044914.1
Archaea Methanobacteriati Methanobacteriota Methanobacterium flexile BGR54607.1
Archaea Methanobacteriati Methanobacteriota Methanobacteriaceae archaeon MDI6645120.1
Archaea Methanobacteriati Methanobacteriota Methanomicrobiales archaeon MGC9517992.1
Archaea Methanobacteriati Methanobacteriota Methanobacterium sp. BRmetb2 AXV36966.1
Archaea Methanobacteriati Methanobacteriota Methanobacteriota archaeon MBM4240228.1
Archaea Methanobacteriati Methanobacteriota Methanobacterium paludis WP 013824985.1
Archaea Methanobacteriati Methanobacteriota Methanobacterium lacus WP 013645768.1
Archaea Methanobacteriati Methanobacteriota Methanobacterium sp. SMA-27 WP 048189943.1
Archaea Methanobacteriati Methanobacteriota Methanobacterium sp. WP 424714028.1
Archaea Methanobacteriati Methanobacteriota Methanobacterium WP 069583489.1
Archaea Methanobacteriati Methanobacteriota Methanobacterium movilense BGR10285.1
Archaea Methanobacteriati Methanobacteriota Methanobacterium oryzae WP 429223172.1
Archaea Methanobacteriati Methanobacteriota Methanobacterium sp. MDI6724169.1
Archaea Methanobacteriati Methanobacteriota Methanobacterium sp. WP 431062874.1
Archaea Methanobacteriati Methanobacteriota Methanobacterium sp. HML04944.1
Archaea Methanobacteriati Methanobacteriota unclassified Methanobacterium WP 414468434.1
Archaea Methanobacteriati Methanobacteriota Methanobacteriaceae archaeon MEN4007406.1
Archaea Methanobacteriati Methanobacteriota Methanobacterium sp. MGZ7044095.1
Archaea Methanobacteriati Methanobacteriota Methanobacterium sp. TMS42522.1
Archaea Methanobacteriati Methanobacteriota Methanobacterium sp. MDD1774596.1
Archaea Methanobacteriati Methanobacteriota Methanobacterium sp. PtaU1.Bin097 OPY24386.1
Archaea Methanobacteriati Methanobacteriota Methanobacterium sp. MCE5214088.1
Archaea Methanobacteriati Methanobacteriota Methanothermobacter sp. MGV8144096.1
Archaea Methanobacteriati Methanobacteriota Methanobacterium sp. MDD1774204.1
Archaea Methanobacteriati Methanobacteriota Methanobacterium sp. HTX60881.1
Archaea Methanobacteriati Methanobacteriota Methanobacterium sp. MGA2677118.1
Archaea Methanobacteriati Methanobacteriota Methanobacterium sp. HEX7467002.1
Archaea Methanobacteriati Methanobacteriota Methanobacterium congolense WP 071906171.1
Archaea Methanobacteriati Methanobacteriota Methanobacterium aridiramus WP 286476246.1
Archaea Methanobacteriati Methanobacteriota Methanobacteriales archaeon Met13 MEG3224996.1
Archaea Methanobacteriati Methanobacteriota Methanobacteriaceae archaeon HMK53542.1
Archaea Methanobacteriati Methanobacteriota Methanobacterium petrolearium WP 209625882.1
Archaea Methanobacteriati Methanobacteriota Methanobacteriaceae archaeon NYB51374.1
Archaea Methanobacteriati Methanobacteriota Methanobacterium ferruginis WP 286244191.1
Archaea Methanobacteriati Methanobacteriota Methanobacterium sp. MZD130B WP 416113058.1
Archaea Methanobacteriati Methanobacteriota Methanobacterium sp. MCC7564589.1
Archaea Methanobacteriati Methanobacteriota uncultured Methanobacterium sp. WP 321421055.1
Archaea Methanobacteriati Methanobacteriota Methanobacterium sp. BAmetb5 WP 292614373.1
Archaea Methanobacteriati Methanobacteriota Methanobacterium sp. WP 287681777.1
Archaea Methanobacteriati Methanobacteriota Methanobacteriaceae archaeon MDO5850885.1

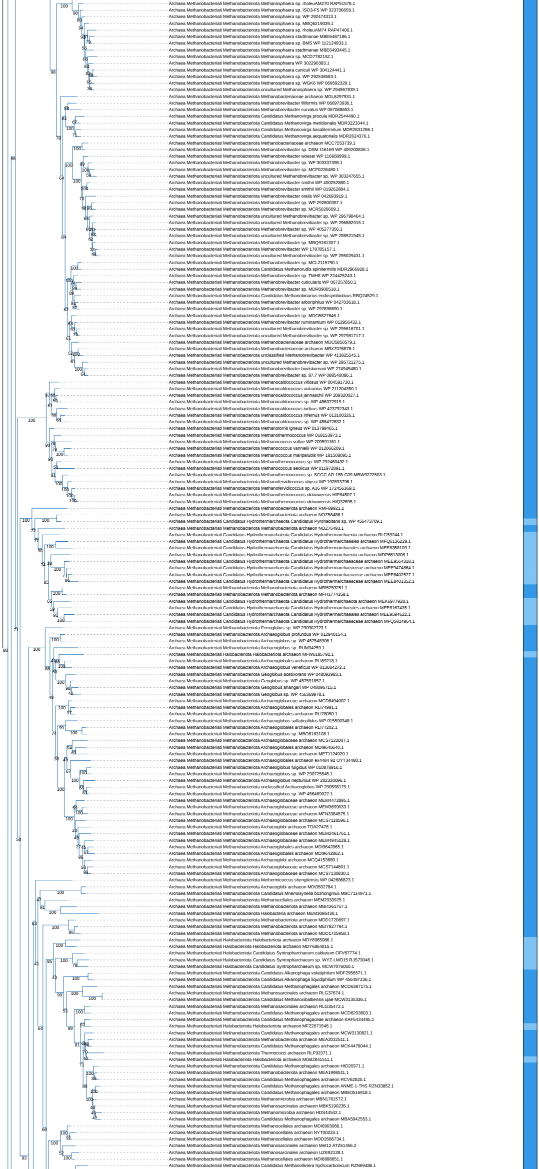
Archaea Methanobacteriati Methanobacteriota Methanosphaera sp. rholeuAM270 RAP51578.1
Archaea Methanobacteriati Methanobacteriota Methanosphaera sp. ISO3-F5 WP 323736659.1
Archaea Methanobacteriati Methanobacteriota Methanosphaera sp. WP 292474313.1
Archaea Methanobacteriati Methanobacteriota Methanosphaera sp. MBQ6219039.1
Archaea Methanobacteriati Methanobacteriota Methanosphaera sp. rholeuAM74 RAP47406.1
Archaea Methanobacteriati Methanobacteriota Methanosphaera stadtmanae MBE6487186.1
Archaea Methanobacteriati Methanobacteriota Methanosphaera sp. BMS WP 112124933.1
Archaea Methanobacteriati Methanobacteriota Methanosphaera stadtmanae MBE6493445.1
Archaea Methanobacteriati Methanobacteriota Methanosphaera sp. MCD7782152.1
Archaea Methanobacteriati Methanobacteriota Methanosphaera WP 302290383.1
Archaea Methanobacteriati Methanobacteriota Methanosphaera cuniculi WP 304124441.1
Archaea Methanobacteriati Methanobacteriota Methanosphaera sp. WP 292536583.1
Archaea Methanobacteriati Methanobacteriota Methanosphaera sp. WGK6 WP 069592329.1
Archaea Methanobacteriati Methanobacteriota uncultured Methanosphaera sp. WP 294967839.1
Archaea Methanobacteriati Methanobacteriota Methanobacteriaceae archaeon MGL6297931.1
Archaea Methanobacteriati Methanobacteriota Methanobrevibacter filiformis WP 066973936.1
Archaea Methanobacteriati Methanobacteriota Methanobrevibacter curvatus WP 067088653.1
Archaea Methanobacteriati Methanobacteriota Candidatus Methanovirga procula MDR2544490.1
Archaea Methanobacteriati Methanobacteriota Candidatus Methanovirga meridionalis MDR3223344.1
Archaea Methanobacteriati Methanobacteriota Candidatus Methanovirga basalitermitum MDR2831286.1
Archaea Methanobacteriati Methanobacteriota Candidatus Methanovirga aequatorialis MDR2624376.1
Archaea Methanobacteriati Methanobacteriota Methanobacteriaceae archaeon MCC7553739.1
Archaea Methanobacteriati Methanobacteriota Methanobrevibacter sp. DSM 116169 WP 409200836.1
Archaea Methanobacteriati Methanobacteriota Methanobrevibacter woesei WP 116668999.1
Archaea Methanobacteriati Methanobacteriota Methanobrevibacter sp. WP 303337398.1
Archaea Methanobacteriati Methanobacteriota Methanobrevibacter sp. MCF0226480.1
Archaea Methanobacteriati Methanobacteriota uncultured Methanobrevibacter sp. WP 303247655.1
Archaea Methanobacteriati Methanobacteriota Methanobrevibacter smithii WP 400252880.1
Archaea Methanobacteriati Methanobacteriota Methanobrevibacter smithii WP 019262884.1
Archaea Methanobacteriati Methanobacteriota Methanobrevibacter oralis WP 042693919.1
Archaea Methanobacteriati Methanobacteriota Methanobrevibacter sp. WP 292800357.1
Archaea Methanobacteriati Methanobacteriota Methanobrevibacter sp. MCR5026609.1
Archaea Methanobacteriati Methanobacteriota uncultured Methanobrevibacter sp. WP 296798464.1
Archaea Methanobacteriati Methanobacteriota uncultured Methanobrevibacter sp. WP 296862915.1
Archaea Methanobacteriati Methanobacteriota Methanobrevibacter sp. WP 405277356.1
Archaea Methanobacteriati Methanobacteriota uncultured Methanobrevibacter sp. WP 298521945.1
Archaea Methanobacteriati Methanobacteriota Methanobrevibacter sp. MBQ9161307.1
Archaea Methanobacteriati Methanobacteriota Methanobrevibacter WP 178785157.1
Archaea Methanobacteriati Methanobacteriota uncultured Methanobrevibacter sp. WP 295029431.1
Archaea Methanobacteriati Methanobacteriota Methanobrevibacter sp. MCL2115790.1
Archaea Methanobacteriati Methanobacteriota Candidatus Methanorudis spinitermitis MDR2966928.1
Archaea Methanobacteriati Methanobacteriota Methanobrevibacter sp. TMH8 WP 224425243.1
Archaea Methanobacteriati Methanobacteriota Methanobrevibacter cuticularis WP 067257850.1
Archaea Methanobacteriati Methanobacteriota Methanobrevibacter sp. MDR0900518.1
Archaea Methanobacteriati Methanobacteriota Candidatus Methanobinarius endosymbioticus RBQ24529.1
Archaea Methanobacteriati Methanobacteriota Methanobrevibacter arboriphilus WP 042703618.1
Archaea Methanobacteriati Methanobacteriota Methanobrevibacter sp. WP 297898690.1
Archaea Methanobacteriati Methanobacteriota Methanobrevibacter sp. MDO5827646.1
Archaea Methanobacteriati Methanobacteriota Methanobrevibacter ruminantium WP 012956432.1
Archaea Methanobacteriati Methanobacteriota uncultured Methanobrevibacter sp. WP 295616701.1
Archaea Methanobacteriati Methanobacteriota uncultured Methanobrevibacter sp. WP 297981717.1
Archaea Methanobacteriati Methanobacteriota Methanobacteriaceae archaeon MDO5850579.1
Archaea Methanobacteriati Methanobacteriota Methanobacteriaceae archaeon MBX7076876.1
Archaea Methanobacteriati Methanobacteriota unclassified Methanobrevibacter WP 413825545.1
Archaea Methanobacteriati Methanobacteriota uncultured Methanobrevibacter sp. WP 295721275.1
Archaea Methanobacteriati Methanobacteriota Methanobrevibacter boviskoreani WP 274945480.1
Archaea Methanobacteriati Methanobacteriota Methanobrevibacter sp. 87.7 WP 088540086.1
Archaea Methanobacteriati Methanobacteriota Methanocaldococcus villosus WP 004591730.1
Archaea Methanobacteriati Methanobacteriota Methanocaldococcus vulcanius WP 211204350.1
Archaea Methanobacteriati Methanobacteriota Methanocaldococcus jannaschii WP 209320027.1
Archaea Methanobacteriati Methanobacteriota Methanocaldococcus sp. WP 456372919.1
Archaea Methanobacteriati Methanobacteriota Methanocaldococcus indicus WP 423792343.1
Archaea Methanobacteriati Methanobacteriota Methanocaldococcus infernus WP 013100326.1
Archaea Methanobacteriati Methanobacteriota Methanocaldococcus sp. WP 456472632.1
Archaea Methanobacteriati Methanobacteriota Methanotorris igneus WP 013799465.1
Archaea Methanobacteriati Methanobacteriota Methanothermococcus WP 018153973.1
Archaea Methanobacteriati Methanobacteriota Methanococcus voltae WP 209591161.1
Archaea Methanobacteriati Methanobacteriota Methanococcus vannielii WP 012066209.1
Archaea Methanobacteriati Methanobacteriota Methanococcus maripaludis WP 181508095.1
Archaea Methanobacteriati Methanobacteriota Methanothermococcus sp. WP 292460432.1
Archaea Methanobacteriati Methanobacteriota Methanococcus aeolicus WP 011972891.1
Archaea Methanobacteriati Methanobacteriota Methanothermococcus sp. SCGC AD-155-C09 MBW9222503.1
Archaea Methanobacteriati Methanobacteriota Methanofervidicoccus abyssi WP 192893796.1
Archaea Methanobacteriati Methanobacteriota Methanofervidicoccus sp. A16 WP 172456369.1
Archaea Methanobacteriati Methanobacteriota Methanothermococcus okinawensis HIP84507.1
Archaea Methanobacteriati Methanobacteriota Methanothermococcus okinawensis HIQ32695.1
Archaea Methanobacteriati Methanobacteriota Methanobacteriota archaeon RMF89921.1
Archaea Methanobacteriati Methanobacteriota Methanobacteriota archaeon NOZ58486.1
Archaea Methanobacteriati Candidatus Hydrothermarchaeota Candidatus Pyrohabitans sp. WP 456473709.1
Archaea Methanobacteriati Methanobacteriota Methanobacteriota archaeon NOZ76493.1
Archaea Methanobacteriati Candidatus Hydrothermarchaeota Candidatus Hydrothermarchaeota archaeon RLG59244.1
Archaea Methanobacteriati Candidatus Hydrothermarchaeota Candidatus Hydrothermarchaeales archaeon MFQ6136229.1
Archaea Methanobacteriati Candidatus Hydrothermarchaeota Candidatus Hydrothermarchaeales archaeon MEE8358109.1
Archaea Methanobacteriati Candidatus Hydrothermarchaeota Candidatus Hydrothermarchaeota archaeon MDP6613008.1
Archaea Methanobacteriati Candidatus Hydrothermarchaeota Candidatus Hydrothermarchaeaceae archaeon MEE9564318.1
Archaea Methanobacteriati Candidatus Hydrothermarchaeota Candidatus Hydrothermarchaeaceae archaeon MEE9474864.1
Archaea Methanobacteriati Candidatus Hydrothermarchaeota Candidatus Hydrothermarchaeaceae archaeon MEE8402577.1
Archaea Methanobacteriati Candidatus Hydrothermarchaeota Candidatus Hydrothermarchaeaceae archaeon MEE8401352.1
Archaea Methanobacteriati Methanobacteriota Methanobacteriota archaeon MBI5253251.1
Archaea Methanobacteriati Methanobacteriota Methanobacteriota archaeon MFH1774359.1
Archaea Methanobacteriati Candidatus Hydrothermarchaeota Candidatus Hydrothermarchaeota archaeon MEK6977928.1
Archaea Methanobacteriati Candidatus Hydrothermarchaeota Candidatus Hydrothermarchaeales archaeon MEE8167435.1
Archaea Methanobacteriati Candidatus Hydrothermarchaeota Candidatus Hydrothermarchaeales archaeon MEE9594622.1
Archaea Methanobacteriati Candidatus Hydrothermarchaeota Candidatus Hydrothermarchaeaceae archaeon MFQ5814964.1
Archaea Methanobacteriati Methanobacteriota Ferroglobus sp. WP 290902723.1
Archaea Methanobacteriati Methanobacteriota Archaeoglobus profundus WP 012940154.1
Archaea Methanobacteriati Methanobacteriota Archaeoglobus sp. WP 457548906.1
Archaea Methanobacteriati Methanobacteriota Archaeoglobus sp. RUM34259.1
Archaea Methanobacteriati Halobacteriota Halobacteriota archaeon MFW6185792.1
Archaea Methanobacteriati Methanobacteriota Archaeoglobales archaeon RLI89218.1
Archaea Methanobacteriati Methanobacteriota Archaeoglobus veneficus WP 013684272.1
Archaea Methanobacteriati Methanobacteriota Geoglobus acetivorans WP 048092983.1
Archaea Methanobacteriati Methanobacteriota Geoglobus sp. WP 457591857.1
Archaea Methanobacteriati Methanobacteriota Geoglobus ahangari WP 048096715.1
Archaea Methanobacteriati Methanobacteriota Geoglobus sp. WP 456369978.1
Archaea Methanobacteriati Methanobacteriota Archaeoglobaceae archaeon MCD6494002.1
Archaea Methanobacteriati Methanobacteriota Archaeoglobales archaeon RLI74991.1
Archaea Methanobacteriati Methanobacteriota Archaeoglobales archaeon RLI78050.1
Archaea Methanobacteriati Methanobacteriota Archaeoglobus sulfaticallidus WP 015590348.1
Archaea Methanobacteriati Methanobacteriota Archaeoglobales archaeon RLI77202.1
Archaea Methanobacteriati Methanobacteriota Archaeoglobus sp. MBO8183108.1
Archaea Methanobacteriati Methanobacteriota Archaeoglobaceae archaeon MCS7122007.1
Archaea Methanobacteriati Methanobacteriota Archaeoglobales archaeon MDI9646640.1
Archaea Methanobacteriati Methanobacteriota Archaeoglobaceae archaeon MET1124920.1
Archaea Methanobacteriati Methanobacteriota Archaeoglobales archaeon ex4484 92 OYT34480.1
Archaea Methanobacteriati Methanobacteriota Archaeoglobus fulgidus WP 010878916.1
Archaea Methanobacteriati Methanobacteriota Archaeoglobus sp. WP 290725545.1
Archaea Methanobacteriati Methanobacteriota Archaeoglobus neptunius WP 202320096.1
Archaea Methanobacteriati Methanobacteriota unclassified Archaeoglobus WP 290598179.1
Archaea Methanobacteriati Methanobacteriota Archaeoglobus sp. WP 456469022.1
Archaea Methanobacteriati Methanobacteriota Archaeoglobaceae archaeon MEM4472895.1
Archaea Methanobacteriati Methanobacteriota Archaeoglobaceae archaeon MEM3699033.1
Archaea Methanobacteriati Methanobacteriota Archaeoglobaceae archaeon MFN3384575.1
Archaea Methanobacteriati Methanobacteriota Archaeoglobaceae archaeon MCS7118596.1
Archaea Methanobacteriati Methanobacteriota Archaeoglobi archaeon TDA27476.1
Archaea Methanobacteriati Methanobacteriota Archaeoglobaceae archaeon MEM2461761.1
Archaea Methanobacteriati Methanobacteriota Archaeoglobaceae archaeon MEM4945128.1
Archaea Methanobacteriati Methanobacteriota Archaeoglobales archaeon MDI9642865.1
Archaea Methanobacteriati Methanobacteriota Archaeoglobales archaeon MDI9642862.1
Archaea Methanobacteriati Methanobacteriota Archaeoglobi archaeon MCQ4153689.1
Archaea Methanobacteriati Methanobacteriota Archaeoglobaceae archaeon MCS7144601.1
Archaea Methanobacteriati Methanobacteriota Archaeoglobaceae archaeon MCS7130830.1
Archaea Methanobacteriati Methanobacteriota Methermicoccus shengliensis WP 042686823.1
Archaea Methanobacteriati Methanobacteriota Archaeoglobi archaeon MDI3502784.1
Archaea Methanobacteriati Methanobacteriota Candidatus Mnemosynella bozhongmuii MBC7114971.1
Archaea Methanobacteriati Methanobacteriota Methanocellales archaeon MEM2933925.1
Archaea Methanobacteriati Methanobacteriota Methanobacteriota archaeon MBI4361757.1
Archaea Methanobacteriati Methanobacteriota Halobacteria archaeon MEM3086430.1
Archaea Methanobacteriati Methanobacteriota Methanobacteriota archaeon MDD1720897.1
Archaea Methanobacteriati Methanobacteriota Methanobacteriota archaeon MEI7827794.1
Archaea Methanobacteriati Methanobacteriota Methanobacteriota archaeon MDD1725958.1
Archaea Methanobacteriati Halobacteriota Halobacteriota archaeon MDY6965086.1
Archaea Methanobacteriati Halobacteriota Halobacteriota archaeon MDY6864815.1
Archaea Methanobacteriati Halobacteriota Candidatus Syntropharchaeum caldarium OFV67774.1
Archaea Methanobacteriati Halobacteriota Candidatus Syntropharchaeum sp. WYZ-LMO15 RJS73046.1
Archaea Methanobacteriati Halobacteriota Candidatus Syntropharchaeum sp. MCW7076050.1
Archaea Methanobacteriati Methanobacteriota Candidatus Alkanophaga volatiphilum MDF2956571.1
Archaea Methanobacteriati Methanobacteriota Candidatus Alkanophaga liquidiphilum WP 456487236.1
Archaea Methanobacteriati Methanobacteriota Candidatus Methanophagales archaeon MCD6387175.1
Archaea Methanobacteriati Methanobacteriota Methanosarcinales archaeon RLG37674.1
Archaea Methanobacteriati Methanobacteriota Candidatus Methanoxibalbensis ujae MCW3135336.1
Archaea Methanobacteriati Methanobacteriota Methanosarcinales archaeon RLG35472.1
Archaea Methanobacteriati Methanobacteriota Candidatus Methanophagales archaeon MCD6203603.1
Archaea Methanobacteriati Methanobacteriota Candidatus Methanophagaceae archaeon KAF5434495.1
Archaea Methanobacteriati Halobacteriota Halobacteriota archaeon MFZ2071546.1
Archaea Methanobacteriati Methanobacteriota Candidatus Methanophagales archaeon MCW3130821.1
Archaea Methanobacteriati Methanobacteriota Methanobacteriota archaeon MEA2032511.1
Archaea Methanobacteriati Methanobacteriota Candidatus Methanophagales archaeon MCK4476044.1
Archaea Methanobacteriati Methanobacteriota Thermococci archaeon RLF92371.1
Archaea Methanobacteriati Halobacteriota Halobacteriota archaeon MGB2841511.1
Archaea Methanobacteriati Methanobacteriota Candidatus Methanophagales archaeon HID20371.1
Archaea Methanobacteriati Methanobacteriota Methanobacteriota archaeon MEA1998511.1
Archaea Methanobacteriati Methanobacteriota Candidatus Methanophagales archaeon RCV62825.1
Archaea Methanobacteriati Methanobacteriota Candidatus Methanophagales archaeon ANME-1-THS RZN33852.1
Archaea Methanobacteriati Methanobacteriota Candidatus Methanophagales archaeon MBE0516918.1
Archaea Methanobacteriati Methanobacteriota Methanomicrobia archaeon MBN1761572.1
Archaea Methanobacteriati Methanobacteriota Methanosarcinales archaeon MBK5190235.1
Archaea Methanobacteriati Methanobacteriota Methanomicrobia archaeon HDS44542.1
Archaea Methanobacteriati Methanobacteriota Candidatus Methanophagales archaeon MBA5942553.1
Archaea Methanobacteriati Methanobacteriota Methanocellales archaeon MDI6903066.1
Archaea Methanobacteriati Methanobacteriota Methanocellales archaeon NYT00224.1
Archaea Methanobacteriati Methanobacteriota Methanocellales archaeon MDD2666734.1
Archaea Methanobacteriati Methanobacteriota Methanosarcinales archaeon Met12 ATZ61456.2
Archaea Methanobacteriati Methanobacteriota Methanosarcinales archaeon UZE92128.1
Archaea Methanobacteriati Methanobacteriota Methanocellales archaeon MDI6888851.1
Archaea Methanobacteriati Methanobacteriota Candidatus Methanolliviera hydrocarbonicum RZN69486.1

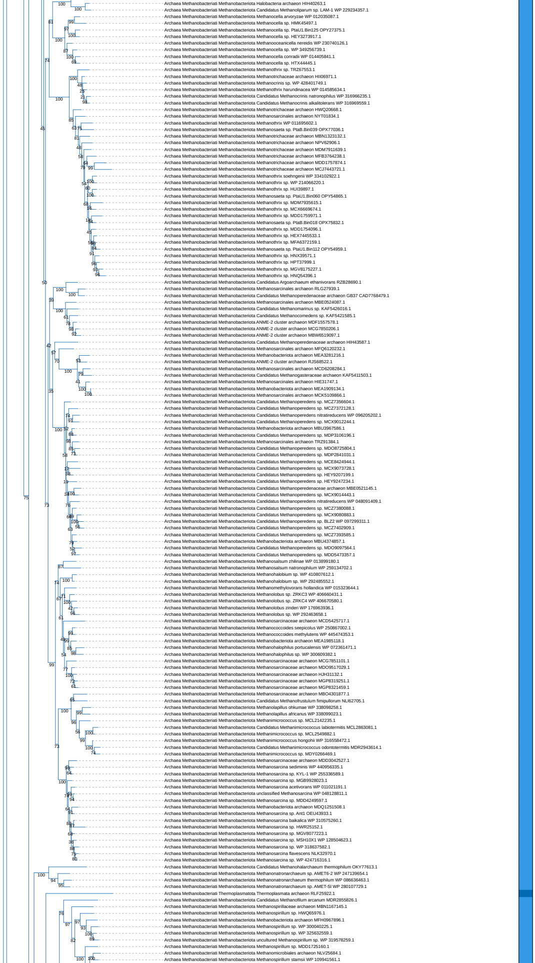
Archaea Methanobacteriati Methanobacteriota Halobacteria archaeon HIH40263.1
Archaea Methanobacteriati Methanobacteriota Candidatus Methanoliparum sp. LAM-1 WP 229234357.1
Archaea Methanobacteriati Methanobacteriota Methanocella arvoryzae WP 012035087.1
Archaea Methanobacteriati Methanobacteriota Methanocella sp. HMK45497.1
Archaea Methanobacteriati Methanobacteriota Methanocella sp. PtaU1.Bin125 OPY27375.1
Archaea Methanobacteriati Methanobacteriota Methanocella sp. HEY3273917.1
Archaea Methanobacteriati Methanobacteriota Methanooceanicella nereidis WP 230740126.1
Archaea Methanobacteriati Methanobacteriota Methanocella sp. WP 349256739.1
Archaea Methanobacteriati Methanobacteriota Methanocella conradii WP 014405841.1
Archaea Methanobacteriati Methanobacteriota Methanocella sp. HTX44445.1
Archaea Methanobacteriati Methanobacteriota Methanothrix sp. TRZ67553.1
Archaea Methanobacteriati Methanobacteriota Methanotrichaceae archaeon HII06971.1
Archaea Methanobacteriati Methanobacteriota Methanocrinis sp. WP 428401749.1
Archaea Methanobacteriati Methanobacteriota Methanothrix harundinacea WP 014585634.1
Archaea Methanobacteriati Methanobacteriota Candidatus Methanocrinis natronophilus WP 316966235.1
Archaea Methanobacteriati Methanobacteriota Candidatus Methanocrinis alkalitolerans WP 316969559.1
Archaea Methanobacteriati Methanobacteriota Methanotrichaceae archaeon HWQ20668.1
Archaea Methanobacteriati Methanobacteriota Methanosarcinales archaeon NYT01834.1
Archaea Methanobacteriati Methanobacteriota Methanothrix WP 011695602.1
Archaea Methanobacteriati Methanobacteriota Methanosaeta sp. PtaB.Bin039 OPX77036.1
Archaea Methanobacteriati Methanobacteriota Methanotrichaceae archaeon MBN1323132.1
Archaea Methanobacteriati Methanobacteriota Methanotrichaceae archaeon NPV62906.1
Archaea Methanobacteriati Methanobacteriota Methanotrichaceae archaeon MDM7911639.1
Archaea Methanobacteriati Methanobacteriota Methanotrichaceae archaeon MFB3764238.1
Archaea Methanobacteriati Methanobacteriota Methanotrichaceae archaeon MDD1757874.1
Archaea Methanobacteriati Methanobacteriota Methanotrichaceae archaeon MCJ7443721.1
Archaea Methanobacteriati Methanobacteriota Methanothrix soehngenii WP 334102922.1
Archaea Methanobacteriati Methanobacteriota Methanothrix sp. WP 214066220.1
Archaea Methanobacteriati Methanobacteriota Methanothrix sp. HUI39897.1
Archaea Methanobacteriati Methanobacteriota Methanosaeta sp. PtaU1.Bin060 OPY54865.1
Archaea Methanobacteriati Methanobacteriota Methanothrix sp. MDM7935615.1
Archaea Methanobacteriati Methanobacteriota Methanothrix sp. MCX6669674.1
Archaea Methanobacteriati Methanobacteriota Methanothrix sp. MDD1759971.1
Archaea Methanobacteriati Methanobacteriota Methanosaeta sp. PtaB.Bin018 OPX75832.1
Archaea Methanobacteriati Methanobacteriota Methanothrix sp. MDD1754096.1
Archaea Methanobacteriati Methanobacteriota Methanothrix sp. HEX7445533.1
Archaea Methanobacteriati Methanobacteriota Methanothrix sp. MFA6372159.1
Archaea Methanobacteriati Methanobacteriota Methanosaeta sp. PtaU1.Bin112 OPY54959.1
Archaea Methanobacteriati Methanobacteriota Methanothrix sp. HNX39571.1
Archaea Methanobacteriati Methanobacteriota Methanothrix sp. HPT37999.1
Archaea Methanobacteriati Methanobacteriota Methanothrix sp. MGV8175227.1
Archaea Methanobacteriati Methanobacteriota Methanothrix sp. HNQ54396.1
Archaea Methanobacteriati Methanobacteriota Candidatus Argoarchaeum ethanivorans RZB28690.1
Archaea Methanobacteriati Methanobacteriota Methanosarcinales archaeon RLG27939.1
Archaea Methanobacteriati Methanobacteriota Candidatus Methanoperedenaceae archaeon GB37 CAD7768479.1
Archaea Methanobacteriati Methanobacteriota Methanosarcinales archaeon MBE0524087.1
Archaea Methanobacteriati Methanobacteriota Candidatus Methanomarinus sp. KAF5426016.1
Archaea Methanobacteriati Methanobacteriota Candidatus Methanocomedens sp. KAF5421585.1
Archaea Methanobacteriati Methanobacteriota ANME-2 cluster archaeon MDF1557578.1
Archaea Methanobacteriati Methanobacteriota ANME-2 cluster archaeon MCG7850206.1
Archaea Methanobacteriati Methanobacteriota ANME-2 cluster archaeon MBW6519097.1
Archaea Methanobacteriati Methanobacteriota Candidatus Methanoperedenaceae archaeon HIH43587.1
Archaea Methanobacteriati Methanobacteriota Methanosarcinales archaeon MFQ6120232.1
Archaea Methanobacteriati Methanobacteriota Methanobacteriota archaeon MEA3281216.1
Archaea Methanobacteriati Methanobacteriota ANME-2 cluster archaeon RJS68522.1
Archaea Methanobacteriati Methanobacteriota Methanosarcinales archaeon MCD6208284.1
Archaea Methanobacteriati Methanobacteriota Candidatus Methanogasteraceae archaeon KAF5411503.1
Archaea Methanobacteriati Methanobacteriota Methanosarcinales archaeon HIE31747.1
Archaea Methanobacteriati Methanobacteriota Methanobacteriota archaeon MEA1909134.1
Archaea Methanobacteriati Methanobacteriota Methanosarcinales archaeon MCK5109866.1
Archaea Methanobacteriati Methanobacteriota Candidatus Methanoperedens sp. MCZ7356604.1
Archaea Methanobacteriati Methanobacteriota Candidatus Methanoperedens sp. MCZ7372128.1
Archaea Methanobacteriati Methanobacteriota Candidatus Methanoperedens nitratireducens WP 096205202.1
Archaea Methanobacteriati Methanobacteriota Candidatus Methanoperedens sp. MCX9012244.1
Archaea Methanobacteriati Methanobacteriota Methanobacteriota archaeon MBU3967586.1
Archaea Methanobacteriati Methanobacteriota Candidatus Methanoperedens sp. MDP3106196.1
Archaea Methanobacteriati Methanobacteriota Methanosarcinales archaeon TRZ91384.1
Archaea Methanobacteriati Methanobacteriota Candidatus Methanoperedens sp. MDO8725804.1
Archaea Methanobacteriati Methanobacteriota Candidatus Methanoperedens sp. MDP2841031.1
Archaea Methanobacteriati Methanobacteriota Candidatus Methanoperedens sp. MCE8424944.1
Archaea Methanobacteriati Methanobacteriota Candidatus Methanoperedens sp. MCX9073728.1
Archaea Methanobacteriati Methanobacteriota Candidatus Methanoperedens sp. HEY9207199.1
Archaea Methanobacteriati Methanobacteriota Candidatus Methanoperedens sp. HEY9247234.1
Archaea Methanobacteriati Methanobacteriota Candidatus Methanoperedenaceae archaeon MBE0521145.1
Archaea Methanobacteriati Methanobacteriota Candidatus Methanoperedens sp. MCX9014443.1
Archaea Methanobacteriati Methanobacteriota Candidatus Methanoperedens nitratireducens WP 048091409.1
Archaea Methanobacteriati Methanobacteriota Candidatus Methanoperedens sp. MCZ7380088.1
Archaea Methanobacteriati Methanobacteriota Candidatus Methanoperedens sp. MCX9080883.1
Archaea Methanobacteriati Methanobacteriota Candidatus Methanoperedens sp. BLZ2 WP 097299311.1
Archaea Methanobacteriati Methanobacteriota Candidatus Methanoperedens sp. MCZ7402909.1
Archaea Methanobacteriati Methanobacteriota Candidatus Methanoperedens sp. MCZ7393585.1
Archaea Methanobacteriati Methanobacteriota Methanobacteriota archaeon MBU4374857.1
Archaea Methanobacteriati Methanobacteriota Candidatus Methanoperedens sp. MDO9097564.1
Archaea Methanobacteriati Methanobacteriota Candidatus Methanoperedens sp. MDD5473357.1
Archaea Methanobacteriati Methanobacteriota Methanosalsum zhilinae WP 013899180.1
Archaea Methanobacteriati Methanobacteriota Methanosalsum natronophilum WP 259134702.1
Archaea Methanobacteriati Methanobacteriota Methanohalobium sp. WP 410807612.1
Archaea Methanobacteriati Methanobacteriota Methanohalobium sp. WP 292485552.1
Archaea Methanobacteriati Methanobacteriota Methanomethylovorans hollandica WP 015323644.1
Archaea Methanobacteriati Methanobacteriota Methanolobus sp. ZRKC3 WP 406660431.1
Archaea Methanobacteriati Methanobacteriota Methanolobus sp. ZRKC4 WP 406670580.1
Archaea Methanobacteriati Methanobacteriota Methanolobus zinderi WP 176963936.1
Archaea Methanobacteriati Methanobacteriota Methanolobus sp. WP 292463658.1
Archaea Methanobacteriati Methanobacteriota Methanosarcinaceae archaeon MCD5425717.1
Archaea Methanobacteriati Methanobacteriota Methanococcoides seepicolus WP 250867002.1
Archaea Methanobacteriati Methanobacteriota Methanococcoides methylutens WP 445474353.1
Archaea Methanobacteriati Methanobacteriota Methanobacteriota archaeon MEA1985118.1
Archaea Methanobacteriati Methanobacteriota Methanohalophilus portucalensis WP 072361471.1
Archaea Methanobacteriati Methanobacteriota Methanohalophilus sp. WP 300609382.1
Archaea Methanobacteriati Methanobacteriota Methanosarcinaceae archaeon MCG7851101.1
Archaea Methanobacteriati Methanobacteriota Methanosarcinaceae archaeon MDO9517029.1
Archaea Methanobacteriati Methanobacteriota Methanosarcinaceae archaeon HJH31132.1
Archaea Methanobacteriati Methanobacteriota Methanosarcinaceae archaeon MGP8319251.1
Archaea Methanobacteriati Methanobacteriota Methanosarcinaceae archaeon MGP8321459.1
Archaea Methanobacteriati Methanobacteriota Methanosarcinaceae archaeon MBO4301877.1
Archaea Methanobacteriati Methanobacteriota Candidatus Methanofrustulum fimipullorum NLI62705.1
Archaea Methanobacteriati Methanobacteriota Methanolapillus ohkumae WP 338098258.1
Archaea Methanobacteriati Methanobacteriota Methanolapillus africanus WP 338099023.1
Archaea Methanobacteriati Methanobacteriota Methanimicrococcus sp. MCL2142235.1
Archaea Methanobacteriati Methanobacteriota Candidatus Methanimicrococcus labiotermitis MCL2863081.1
Archaea Methanobacteriati Methanobacteriota Methanimicrococcus sp. MCL2549882.1
Archaea Methanobacteriati Methanobacteriota Methanimicrococcus hongohii WP 316558472.1
Archaea Methanobacteriati Methanobacteriota Candidatus Methanimicrococcus odontotermitis MDR2943614.1
Archaea Methanobacteriati Methanobacteriota Methanimicrococcus sp. MDY0266469.1
Archaea Methanobacteriati Methanobacteriota Methanosarcinaceae archaeon MDD3042527.1
Archaea Methanobacteriati Methanobacteriota Methanosarcina sediminis WP 440956335.1
Archaea Methanobacteriati Methanobacteriota Methanosarcina sp. KYL-1 WP 255336589.1
Archaea Methanobacteriati Methanobacteriota Methanosarcina sp. MGB9928023.1
Archaea Methanobacteriati Methanobacteriota Methanosarcina acetivorans WP 011021191.1
Archaea Methanobacteriati Methanobacteriota unclassified Methanosarcina WP 048128811.1
Archaea Methanobacteriati Methanobacteriota Methanosarcina sp. MDD4249597.1
Archaea Methanobacteriati Methanobacteriota Methanobacteriota archaeon MDQ1251508.1
Archaea Methanobacteriati Methanobacteriota Methanosarcina sp. Ant1 OEU43933.1
Archaea Methanobacteriati Methanobacteriota Methanosarcina baikalica WP 310575260.1
Archaea Methanobacteriati Methanobacteriota Methanosarcina sp. HWR25152.1
Archaea Methanobacteriati Methanobacteriota Methanosarcina sp. MGV8077223.1
Archaea Methanobacteriati Methanobacteriota Methanosarcina sp. MSH10X1 WP 128504623.1
Archaea Methanobacteriati Methanobacteriota Methanosarcina sp. WP 318637582.1
Archaea Methanobacteriati Methanobacteriota Methanosarcina flavescens NLK32970.1
Archaea Methanobacteriati Methanobacteriota Methanosarcina sp. WP 424716316.1
Archaea Methanobacteriati Methanobacteriota Candidatus Methanohalarchaeum thermophilum OKY77613.1
Archaea Methanobacteriati Methanobacteriota Methanonatronarchaeum sp. AMET6-2 WP 247139654.1
Archaea Methanobacteriati Methanobacteriota Methanonatronarchaeum thermophilum WP 086636463.1
Archaea Methanobacteriati Methanobacteriota Methanonatronarchaeum sp. AMET-Sl WP 280107729.1
Archaea Methanobacteriati Thermoplasmatota Thermoplasmata archaeon RLF25922.1
Archaea Methanobacteriati Methanobacteriota Candidatus Methanofilum arcanum MDR2855826.1
Archaea Methanobacteriati Methanobacteriota Methanospirillaceae archaeon MBN1167145.1
Archaea Methanobacteriati Methanobacteriota Methanospirillum sp. HWQ65976.1
Archaea Methanobacteriati Methanobacteriota Methanobacteriota archaeon MFH0967896.1
Archaea Methanobacteriati Methanobacteriota Methanospirillum sp. WP 300040225.1
Archaea Methanobacteriati Methanobacteriota Methanospirillum sp. WP 325632559.1
Archaea Methanobacteriati Methanobacteriota Methanospirillum uncultured Methanospirillum sp. WP 319578259.1
Archaea Methanobacteriati Methanobacteriota Methanospirillum sp. MDD1725160.1
Archaea Methanobacteriati Methanobacteriota Methanomicrobiales archaeon NLV25684.1
Archaea Methanobacteriati Methanobacteriota Methanospirillum stamsii WP 109941561.1

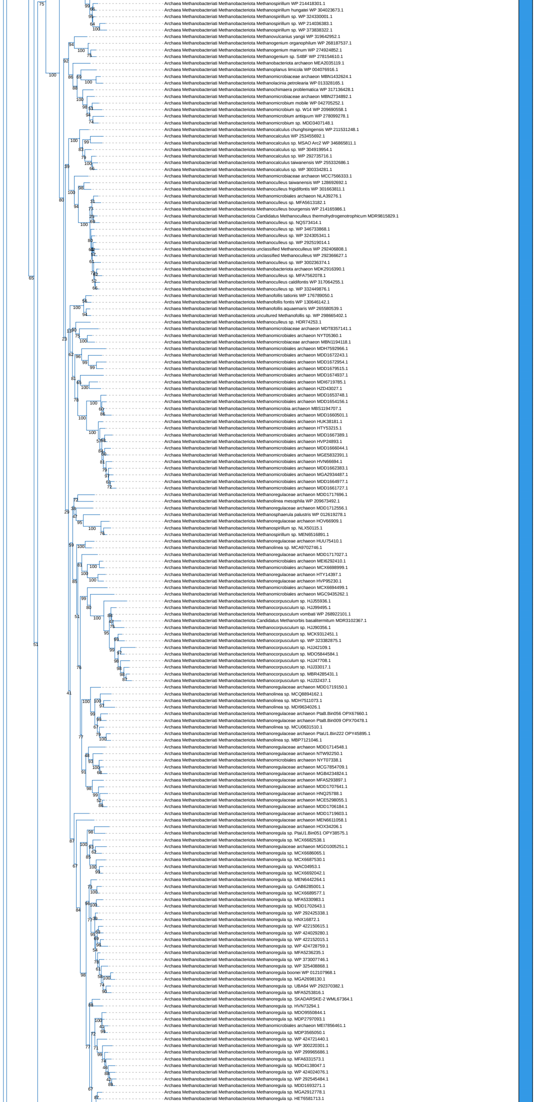

Archaea Methanobacteriati Methanobacteriota Methanospirillum WP 214418301.1
Archaea Methanobacteriati Methanobacteriota Methanospirillum hungatei WP 304023673.1
Archaea Methanobacteriati Methanobacteriota Methanospirillum sp. WP 324330001.1
Archaea Methanobacteriati Methanobacteriota Methanospirillum sp. WP 214036383.1
Archaea Methanobacteriati Methanobacteriota Methanospirillum sp. WP 373838322.1
Archaea Methanobacteriati Methanobacteriota Methanovulcanius yangii WP 319642952.1
Archaea Methanobacteriati Methanobacteriota Methanogenium organophilum WP 268187537.1
Archaea Methanobacteriati Methanobacteriota Methanogenium marinum WP 274924852.1
Archaea Methanobacteriati Methanobacteriota Methanogenium sp. S4BF WP 278154610.1
Archaea Methanobacteriati Methanobacteriota Methanobacteriota archaeon MEA2035119.1
Archaea Methanobacteriati Methanobacteriota Methanoplanus limicola WP 004076916.1
Archaea Methanobacteriati Methanobacteriota Methanomicrobiaceae archaeon MBN1432624.1
Archaea Methanobacteriati Methanobacteriota Methanolacinia petrolearia WP 013328165.1
Archaea Methanobacteriati Methanobacteriota Methanochimaera problematica WP 317136428.1
Archaea Methanobacteriati Methanobacteriota Methanomicrobiaceae archaeon MBN2734892.1
Archaea Methanobacteriati Methanobacteriota Methanomicrobium mobile WP 042705252.1
Archaea Methanobacteriati Methanobacteriota Methanomicrobium sp. W14 WP 209690558.1
Archaea Methanobacteriati Methanobacteriota Methanomicrobium antiquum WP 278099278.1
Archaea Methanobacteriati Methanobacteriota Methanomicrobium sp. MDD3407148.1
Archaea Methanobacteriati Methanobacteriota Methanocalculus chunghsingensis WP 211531248.1
Archaea Methanobacteriati Methanobacteriota Methanocalculus WP 253455692.1
Archaea Methanobacteriati Methanobacteriota Methanocalculus sp. MSAO Arc2 WP 346865811.1
Archaea Methanobacteriati Methanobacteriota Methanocalculus sp. WP 304919954.1
Archaea Methanobacteriati Methanobacteriota Methanocalculus sp. WP 292735716.1
Archaea Methanobacteriati Methanobacteriota Methanocalculus taiwanensis WP 255332686.1
Archaea Methanobacteriati Methanobacteriota Methanocalculus sp. WP 300334281.1
Archaea Methanobacteriati Methanobacteriota Methanomicrobiaceae archaeon MCC7566333.1
Archaea Methanobacteriati Methanobacteriota Methanoculleus taiwanensis WP 128692692.1
Archaea Methanobacteriati Methanobacteriota Methanoculleus frigidifontis WP 301663811.1
Archaea Methanobacteriati Methanobacteriota Methanomicrobiales archaeon NLA39276.1
Archaea Methanobacteriati Methanobacteriota Methanoculleus sp. MFA5613182.1
Archaea Methanobacteriati Methanobacteriota Methanoculleus bourgensis WP 214165986.1
Archaea Methanobacteriati Methanobacteriota Candidatus Methanoculleus thermohydrogenotrophicum MDR9815829.1
Archaea Methanobacteriati Methanobacteriota Methanoculleus sp. NQS73414.1
Archaea Methanobacteriati Methanobacteriota Methanoculleus sp. WP 346733868.1
Archaea Methanobacteriati Methanobacteriota Methanoculleus sp. WP 324305341.1
Archaea Methanobacteriati Methanobacteriota Methanoculleus sp. WP 292519014.1
Archaea Methanobacteriati Methanobacteriota unclassified Methanoculleus WP 292406808.1
Archaea Methanobacteriati Methanobacteriota unclassified Methanoculleus WP 292366627.1
Archaea Methanobacteriati Methanobacteriota Methanoculleus sp. WP 300236374.1
Archaea Methanobacteriati Methanobacteriota Methanobacteriota archaeon MDK2916390.1
Archaea Methanobacteriati Methanobacteriota Methanoculleus sp. MFA7562078.1
Archaea Methanobacteriati Methanobacteriota Methanoculleus caldifontis WP 317064255.1
Archaea Methanobacteriati Methanobacteriota Methanoculleus sp. WP 332449876.1
Archaea Methanobacteriati Methanobacteriota Methanofollis tationis WP 176789050.1
Archaea Methanobacteriati Methanobacteriota Methanofollis fontis WP 130646142.1
Archaea Methanobacteriati Methanobacteriota Methanofollis aquaemaris WP 265580539.1
Archaea Methanobacteriati Methanobacteriota uncultured Methanofollis sp. WP 298665402.1
Archaea Methanobacteriati Methanobacteriota Methanoculleus sp. HDR74253.1
Archaea Methanobacteriati Methanobacteriota Methanomicrobiaceae archaeon MDT8357141.1
Archaea Methanobacteriati Methanobacteriota Methanomicrobiales archaeon NYT05360.1
Archaea Methanobacteriati Methanobacteriota Methanomicrobiaceae archaeon MBN1194118.1
Archaea Methanobacteriati Methanobacteriota Methanomicrobiales archaeon MDH7592966.1
Archaea Methanobacteriati Methanobacteriota Methanomicrobiales archaeon MDD1672243.1
Archaea Methanobacteriati Methanobacteriota Methanomicrobiales archaeon MDD1672954.1
Archaea Methanobacteriati Methanobacteriota Methanomicrobiales archaeon MDD1679515.1
Archaea Methanobacteriati Methanobacteriota Methanomicrobiales archaeon MDD1674937.1
Archaea Methanobacteriati Methanobacteriota Methanomicrobiales archaeon MDI6719785.1
Archaea Methanobacteriati Methanobacteriota Methanomicrobiales archaeon HZD43027.1
Archaea Methanobacteriati Methanobacteriota Methanomicrobiales archaeon MDD1653748.1
Archaea Methanobacteriati Methanobacteriota Methanomicrobiales archaeon MDD1654156.1
Archaea Methanobacteriati Methanobacteriota Methanomicrobia archaeon MBS1194707.1
Archaea Methanobacteriati Methanobacteriota Methanomicrobiales archaeon MDD1660501.1
Archaea Methanobacteriati Methanobacteriota Methanomicrobiales archaeon HUK38181.1
Archaea Methanobacteriati Methanobacteriota Methanomicrobiales archaeon HTY53215.1
Archaea Methanobacteriati Methanobacteriota Methanomicrobiales archaeon MDD1667389.1
Archaea Methanobacteriati Methanobacteriota Methanomicrobiales archaeon HVP24893.1
Archaea Methanobacteriati Methanobacteriota Methanomicrobiales archaeon MDD1666044.1
Archaea Methanobacteriati Methanobacteriota Methanomicrobiales archaeon MGE5832391.1
Archaea Methanobacteriati Methanobacteriota Methanomicrobiales archaeon HVN66694.1
Archaea Methanobacteriati Methanobacteriota Methanomicrobiales archaeon MDD1662383.1
Archaea Methanobacteriati Methanobacteriota Methanomicrobiales archaeon MGA2934487.1
Archaea Methanobacteriati Methanobacteriota Methanomicrobiales archaeon MDD1664977.1
Archaea Methanobacteriati Methanobacteriota Methanomicrobiales archaeon MDD1661727.1
Archaea Methanobacteriati Methanobacteriota Methanoregulaceae archaeon MDD1717696.1
Archaea Methanobacteriati Methanobacteriota Methanolinea mesophila WP 209673492.1
Archaea Methanobacteriati Methanobacteriota Methanoregulaceae archaeon MDD1712556.1
Archaea Methanobacteriati Methanobacteriota Methanosphaerula palustris WP 012619278.1
Archaea Methanobacteriati Methanobacteriota Methanoregulaceae archaeon HOV66909.1
Archaea Methanobacteriati Methanobacteriota Methanospirillum sp. NLX50115.1
Archaea Methanobacteriati Methanobacteriota Methanospirillum sp. MEN6516891.1
Archaea Methanobacteriati Methanobacteriota Methanoregulaceae archaeon HUU75410.1
Archaea Methanobacteriati Methanobacteriota Methanolinea sp. MCA9702746.1
Archaea Methanobacteriati Methanobacteriota Methanoregulaceae archaeon MDD1717027.1
Archaea Methanobacteriati Methanobacteriota Methanomicrobiales archaeon MEI6292410.1
Archaea Methanobacteriati Methanobacteriota Methanomicrobiales archaeon MCX6698999.1
Archaea Methanobacteriati Methanobacteriota Methanoregulaceae archaeon HTY14397.1
Archaea Methanobacteriati Methanobacteriota Methanoregulaceae archaeon HVP95230.1
Archaea Methanobacteriati Methanobacteriota Methanomicrobiales archaeon MCX6694499.1
Archaea Methanobacteriati Methanobacteriota Methanomicrobiales archaeon MGC9435262.1
Archaea Methanobacteriati Methanobacteriota Methanocorpusculum sp. HJJ55936.1
Archaea Methanobacteriati Methanobacteriota Methanocorpusculum sp. HJJ99495.1
Archaea Methanobacteriati Methanobacteriota Methanocorpusculum vombati WP 268922101.1
Archaea Methanobacteriati Methanobacteriota Candidatus Methanorbis basalitermitum MDR3102367.1
Archaea Methanobacteriati Methanobacteriota Methanocorpusculum sp. HJJ90356.1
Archaea Methanobacteriati Methanobacteriota Methanocorpusculum sp. MCK9312451.1
Archaea Methanobacteriati Methanobacteriota Methanocorpusculum sp. WP 323382875.1
Archaea Methanobacteriati Methanobacteriota Methanocorpusculum sp. HJJ42109.1
Archaea Methanobacteriati Methanobacteriota Methanocorpusculum sp. MDO5844584.1
Archaea Methanobacteriati Methanobacteriota Methanocorpusculum sp. HJJ47708.1
Archaea Methanobacteriati Methanobacteriota Methanocorpusculum sp. HJJ33017.1
Archaea Methanobacteriati Methanobacteriota Methanocorpusculum sp. MBR4285431.1
Archaea Methanobacteriati Methanobacteriota Methanocorpusculum sp. HJJ32437.1
Archaea Methanobacteriati Methanobacteriota Methanoregulaceae archaeon MDD1719150.1
Archaea Methanobacteriati Methanobacteriota Methanolinea sp. MCQ8894162.1
Archaea Methanobacteriati Methanobacteriota Methanolinea sp. MDH7511073.1
Archaea Methanobacteriati Methanobacteriota Methanolinea sp. MDI9634026.1
Archaea Methanobacteriati Methanobacteriota Methanoregulaceae archaeon PtaB.Bin056 OPX67660.1
Archaea Methanobacteriati Methanobacteriota Methanoregulaceae archaeon PtaB.Bin009 OPX70478.1
Archaea Methanobacteriati Methanobacteriota Methanolinea sp. MCU0631510.1
Archaea Methanobacteriati Methanobacteriota Methanoregulaceae archaeon PtaU1.Bin222 OPY45895.1
Archaea Methanobacteriati Methanobacteriota Methanolinea sp. MBP7121046.1
Archaea Methanobacteriati Methanobacteriota Methanoregulaceae archaeon MDD1714548.1
Archaea Methanobacteriati Methanobacteriota Methanoregulaceae archaeon NTW92250.1
Archaea Methanobacteriati Methanobacteriota Methanomicrobiales archaeon NYT07338.1
Archaea Methanobacteriati Methanobacteriota Methanoregulaceae archaeon MCG7854709.1
Archaea Methanobacteriati Methanobacteriota Methanoregulaceae archaeon MGB4234824.1
Archaea Methanobacteriati Methanobacteriota Methanoregulaceae archaeon MFA5293897.1
Archaea Methanobacteriati Methanobacteriota Methanoregulaceae archaeon MDD1707641.1
Archaea Methanobacteriati Methanobacteriota Methanoregulaceae archaeon HNQ25788.1
Archaea Methanobacteriati Methanobacteriota Methanoregulaceae archaeon MCE5298055.1
Archaea Methanobacteriati Methanobacteriota Methanoregulaceae archaeon MDD1706184.1
Archaea Methanobacteriati Methanobacteriota Methanoregulaceae archaeon MDD1719603.1
Archaea Methanobacteriati Methanobacteriota Methanoregulaceae archaeon MEN6611058.1
Archaea Methanobacteriati Methanobacteriota Methanoregulaceae archaeon HOX34206.1
Archaea Methanobacteriati Methanobacteriota Methanoregula sp. PtaU1.Bin051 OPY38575.1
Archaea Methanobacteriati Methanobacteriota Methanoregula sp. MCX6682538.1
Archaea Methanobacteriati Methanobacteriota Methanoregulaceae archaeon MGD1005251.1
Archaea Methanobacteriati Methanobacteriota Methanoregula sp. MCX6686065.1
Archaea Methanobacteriati Methanobacteriota Methanoregula sp. MCX6687530.1
Archaea Methanobacteriati Methanobacteriota Methanoregula sp. WAC04953.1
Archaea Methanobacteriati Methanobacteriota Methanoregula sp. MCX6692042.1
Archaea Methanobacteriati Methanobacteriota Methanoregula sp. MEN6442264.1
Archaea Methanobacteriati Methanobacteriota Methanoregula sp. GAB6285001.1
Archaea Methanobacteriati Methanobacteriota Methanoregula sp. MCX6689577.1
Archaea Methanobacteriati Methanobacteriota Methanoregula sp. MFA5330983.1
Archaea Methanobacteriati Methanobacteriota Methanoregula sp. MDD1702643.1
Archaea Methanobacteriati Methanobacteriota Methanoregula sp. WP 292425338.1
Archaea Methanobacteriati Methanobacteriota Methanoregula sp. HNX16872.1
Archaea Methanobacteriati Methanobacteriota Methanoregula sp. WP 422150615.1
Archaea Methanobacteriati Methanobacteriota Methanoregula sp. WP 424029280.1
Archaea Methanobacteriati Methanobacteriota Methanoregula sp. WP 422152015.1
Archaea Methanobacteriati Methanobacteriota Methanoregula sp. WP 424728759.1
Archaea Methanobacteriati Methanobacteriota Methanoregula sp. MFA5236235.1
Archaea Methanobacteriati Methanobacteriota Methanoregula sp. WP 373007746.1
Archaea Methanobacteriati Methanobacteriota Methanoregula sp. WP 325408868.1
Archaea Methanobacteriati Methanobacteriota Methanoregula boonei WP 012107968.1
Archaea Methanobacteriati Methanobacteriota Methanoregula sp. MGA2698130.1
Archaea Methanobacteriati Methanobacteriota Methanoregula sp. UBA64 WP 292370382.1
Archaea Methanobacteriati Methanobacteriota Methanoregula sp. MFA5253816.1
Archaea Methanobacteriati Methanobacteriota Methanoregula sp. SKADARSKE-2 WML67364.1
Archaea Methanobacteriati Methanobacteriota Methanoregula sp. HVN73294.1
Archaea Methanobacteriati Methanobacteriota Methanoregula sp. MDO9550844.1
Archaea Methanobacteriati Methanobacteriota Methanoregula sp. MDP2797093.1
Archaea Methanobacteriati Methanobacteriota Methanomicrobiales archaeon MEI7856461.1
Archaea Methanobacteriati Methanobacteriota Methanoregula sp. MDP3565050.1
Archaea Methanobacteriati Methanobacteriota Methanoregula sp. WP 424721440.1
Archaea Methanobacteriati Methanobacteriota Methanoregula sp. WP 300220301.1
Archaea Methanobacteriati Methanobacteriota Methanoregula sp. WP 299965686.1
Archaea Methanobacteriati Methanobacteriota Methanoregula sp. MFA6331573.1
Archaea Methanobacteriati Methanobacteriota Methanoregula sp. MDD4138047.1
Archaea Methanobacteriati Methanobacteriota Methanoregula sp. WP 424024076.1
Archaea Methanobacteriati Methanobacteriota Methanoregula sp. WP 292545484.1
Archaea Methanobacteriati Methanobacteriota Methanoregula sp. MDD1693271.1
Archaea Methanobacteriati Methanobacteriota Methanoregula sp. MGA2912778.1
Archaea Methanobacteriati Methanobacteriota Methanoregula sp. HET6581713.1

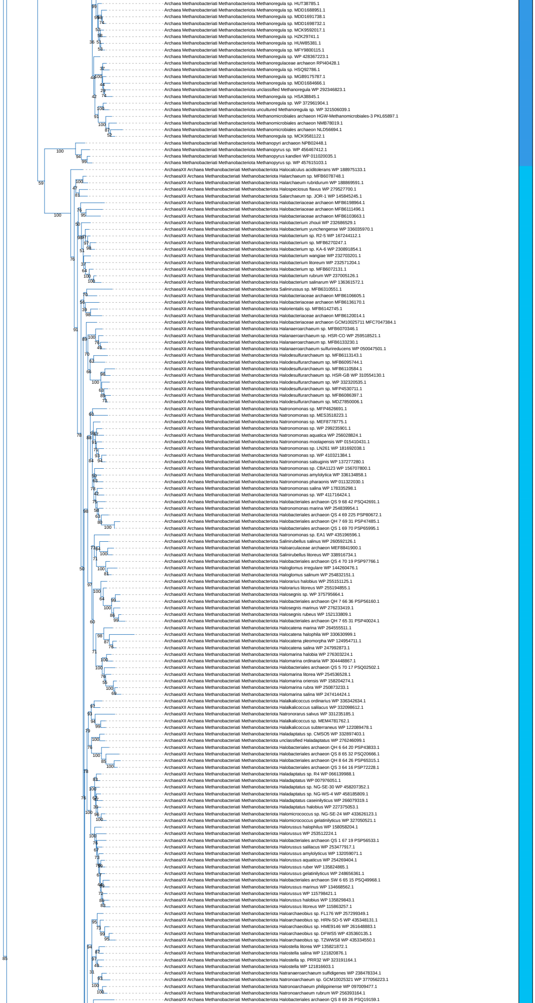
Archaea Methanobacteriati Methanobacteriota Methanoregula sp. HUT38785.1
Archaea Methanobacteriati Methanobacteriota Methanoregula sp. MDD1688951.1
Archaea Methanobacteriati Methanobacteriota Methanoregula sp. MDD1691738.1
Archaea Methanobacteriati Methanobacteriota Methanoregula sp. MDD1698732.1
Archaea Methanobacteriati Methanobacteriota Methanoregula sp. MCK9592017.1
Archaea Methanobacteriati Methanobacteriota Methanoregula sp. HZK29741.1
Archaea Methanobacteriati Methanobacteriota Methanoregula sp. HUW85381.1
Archaea Methanobacteriati Methanobacteriota Methanoregula sp. MFY9800115.1
Archaea Methanobacteriati Methanobacteriota Methanoregula sp. WP 428367223.1
Archaea Methanobacteriati Methanobacteriota Methanoregulaceae archaeon RPI40428.1
Archaea Methanobacteriati Methanobacteriota Methanoregula sp. HSQ92786.1
Archaea Methanobacteriati Methanobacteriota Methanoregula sp. MGB9175787.1
Archaea Methanobacteriati Methanobacteriota Methanoregula sp. MDD1684666.1
Archaea Methanobacteriati Methanobacteriota unclassified Methanoregula WP 292346823.1
Archaea Methanobacteriati Methanobacteriota Methanoregula sp. HSA38845.1
Archaea Methanobacteriati Methanobacteriota Methanoregula sp. WP 372961904.1
Archaea Methanobacteriati Methanobacteriota uncultured Methanoregula sp. WP 321506039.1
Archaea Methanobacteriati Methanobacteriota Methanomicrobiales archaeon HGW-Methanomicrobiales-3 PKL65897.1
Archaea Methanobacteriati Methanobacteriota Methanomicrobiales archaeon NMB78019.1
Archaea Methanobacteriati Methanobacteriota Methanomicrobiales archaeon NLD56694.1
Archaea Methanobacteriati Methanobacteriota Methanoregula sp. MCK9581122.1
Archaea Methanobacteriati Methanobacteriota Methanopyri archaeon NPB02448.1
Archaea Methanobacteriati Methanobacteriota Methanopyrus sp. WP 456467412.1
Archaea Methanobacteriati Methanobacteriota Methanopyrus kandleri WP 011020035.1
Archaea Methanobacteriati Methanobacteriota Methanopyrus sp. WP 457615103.1
ArchaeaXII Archaea Methanobacteriati Methanobacteriota Halocalculus aciditolerans WP 188975133.1
ArchaeaXII Archaea Methanobacteriati Methanobacteriota Halarchaeum sp. MFB6078748.1
ArchaeaXII Archaea Methanobacteriati Methanobacteriota Halarchaeum rubridurum WP 188869591.1
ArchaeaXII Archaea Methanobacteriati Methanobacteriota Halospeciosus flavus WP 279527700.1
ArchaeaXII Archaea Methanobacteriati Methanobacteriota Salarchaeum sp. JOR-1 WP 145845245.1
ArchaeaXII Archaea Methanobacteriati Methanobacteriota Halobacteriaceae archaeon MFB6198964.1
ArchaeaXII Archaea Methanobacteriati Methanobacteriota Halobacteriaceae archaeon MFB6111496.1
ArchaeaXII Archaea Methanobacteriati Methanobacteriota Halobacteriaceae archaeon MFB6103663.1
ArchaeaXII Archaea Methanobacteriati Methanobacteriota Halobacterium zhouii WP 232686529.1
ArchaeaXII Archaea Methanobacteriati Methanobacteriota Halobacterium yunchengense WP 336035970.1
ArchaeaXII Archaea Methanobacteriati Methanobacteriota Halobacterium sp. R2-5 WP 167244112.1
ArchaeaXII Archaea Methanobacteriati Methanobacteriota Halobacterium sp. MFB6270247.1
ArchaeaXII Archaea Methanobacteriati Methanobacteriota Halobacterium sp. KA-6 WP 230891854.1
ArchaeaXII Archaea Methanobacteriati Methanobacteriota Halobacterium wangiae WP 232703201.1
ArchaeaXII Archaea Methanobacteriati Methanobacteriota Halobacterium litoreum WP 232571204.1
ArchaeaXII Archaea Methanobacteriati Methanobacteriota Halobacterium sp. MFB6072131.1
ArchaeaXII Archaea Methanobacteriati Methanobacteriota Halobacterium rubrum WP 237005126.1
ArchaeaXII Archaea Methanobacteriati Methanobacteriota Halobacterium salinarum WP 136361572.1
ArchaeaXII Archaea Methanobacteriati Methanobacteriota Salinirussus sp. MFB6310551.1
ArchaeaXII Archaea Methanobacteriati Methanobacteriota Halobacteriaceae archaeon MFB6106605.1
ArchaeaXII Archaea Methanobacteriati Methanobacteriota Halobacteriaceae archaeon MFB6136170.1
ArchaeaXII Archaea Methanobacteriati Methanobacteriota Halorientalis sp. MFB6142745.1
ArchaeaXII Archaea Methanobacteriati Methanobacteriota Halobacteriaceae archaeon MFB6120014.1
ArchaeaXII Archaea Methanobacteriati Methanobacteriota Halobacteriaceae archaeon GCM10025711 MFC7047384.1
ArchaeaXII Archaea Methanobacteriati Methanobacteriota Halanaeroarchaeum sp. MFB6070346.1
ArchaeaXII Archaea Methanobacteriati Methanobacteriota Halanaeroarchaeum sp. HSR-CO WP 259518521.1
ArchaeaXII Archaea Methanobacteriati Methanobacteriota Halanaeroarchaeum sp. MFB6133230.1
ArchaeaXII Archaea Methanobacteriati Methanobacteriota Halanaeroarchaeum sulfurireducens WP 050047501.1
ArchaeaXII Archaea Methanobacteriati Methanobacteriota Halodesulfurarchaeum sp. MFB6113143.1
ArchaeaXII Archaea Methanobacteriati Methanobacteriota Halodesulfurarchaeum sp. MFB6095744.1
ArchaeaXII Archaea Methanobacteriati Methanobacteriota Halodesulfurarchaeum sp. MFB6110584.1
ArchaeaXII Archaea Methanobacteriati Methanobacteriota Halodesulfurarchaeum sp. HSR-GB WP 310554130.1
ArchaeaXII Archaea Methanobacteriati Methanobacteriota Halodesulfurarchaeum sp. WP 332320535.1
ArchaeaXII Archaea Methanobacteriati Methanobacteriota Halodesulfurarchaeum sp. MFP4530711.1
ArchaeaXII Archaea Methanobacteriati Methanobacteriota Halodesulfurarchaeum sp. MFB6086397.1
ArchaeaXII Archaea Methanobacteriati Methanobacteriota Halodesulfurarchaeum sp. MDZ7850006.1
ArchaeaXII Archaea Methanobacteriati Methanobacteriota Natronomonas sp. MFP4626691.1
ArchaeaXII Archaea Methanobacteriati Methanobacteriota Natronomonas sp. MES3518223.1
ArchaeaXII Archaea Methanobacteriati Methanobacteriota Natronomonas sp. MEF8778775.1
ArchaeaXII Archaea Methanobacteriati Methanobacteriota Natronomonas sp. WP 299235901.1
ArchaeaXII Archaea Methanobacteriati Methanobacteriota Natronomonas aquatica WP 256028824.1
ArchaeaXII Archaea Methanobacteriati Methanobacteriota Natronomonas moolapensis WP 015410431.1
ArchaeaXII Archaea Methanobacteriati Methanobacteriota Natronomonas sp. LN261 WP 181692038.1
ArchaeaXII Archaea Methanobacteriati Methanobacteriota Natronomonas sp. WP 410321384.1
ArchaeaXII Archaea Methanobacteriati Methanobacteriota Natronomonas salsuginis WP 137277280.1
ArchaeaXII Archaea Methanobacteriati Methanobacteriota Natronomonas sp. CBA1123 WP 156707800.1
ArchaeaXII Archaea Methanobacteriati Methanobacteriota Natronomonas amylolytica WP 336134858.1
ArchaeaXII Archaea Methanobacteriati Methanobacteriota Natronomonas pharaonis WP 011322030.1
ArchaeaXII Archaea Methanobacteriati Methanobacteriota Natronomonas salina WP 178335298.1
ArchaeaXII Archaea Methanobacteriati Methanobacteriota Natronomonas sp. WP 411716424.1
ArchaeaXII Archaea Methanobacteriati Methanobacteriota Halobacteriales archaeon QS 9 68 42 PSQ42691.1
ArchaeaXII Archaea Methanobacteriati Methanobacteriota Natronomonas marina WP 254839954.1
ArchaeaXII Archaea Methanobacteriati Methanobacteriota Halobacteriales archaeon QS 4 69 225 PSP80672.1
ArchaeaXII Archaea Methanobacteriati Methanobacteriota Halobacteriales archaeon QH 7 69 31 PSP47485.1
ArchaeaXII Archaea Methanobacteriati Methanobacteriota Halobacteriales archaeon QS 1 69 70 PSP65995.1
ArchaeaXII Archaea Methanobacteriati Methanobacteriota Natronomonas sp. EA1 WP 435196596.1
ArchaeaXII Archaea Methanobacteriati Methanobacteriota Salinirubellus salinus WP 260592126.1
ArchaeaXII Archaea Methanobacteriati Methanobacteriota Haloarculaceae archaeon MEF8841900.1
ArchaeaXII Archaea Methanobacteriati Methanobacteriota Salinirubellus litoreus WP 338916734.1
ArchaeaXII Archaea Methanobacteriati Methanobacteriota Halobacteriales archaeon QS 4 70 19 PSP97766.1
ArchaeaXII Archaea Methanobacteriati Methanobacteriota Haloglomus irregulare WP 144260476.1
ArchaeaXII Archaea Methanobacteriati Methanobacteriota Haloglomus salinum WP 254832151.1
ArchaeaXII Archaea Methanobacteriati Methanobacteriota Halorarius halobius WP 255151125.1
ArchaeaXII Archaea Methanobacteriati Methanobacteriota Halorarius litoreus WP 255194855.1
ArchaeaXII Archaea Methanobacteriati Methanobacteriota Halosegnis sp. WP 375795664.1
ArchaeaXII Archaea Methanobacteriati Methanobacteriota Halobacteriales archaeon QH 7 66 36 PSP56160.1
ArchaeaXII Archaea Methanobacteriati Methanobacteriota Halosegnis marinus WP 276233419.1
ArchaeaXII Archaea Methanobacteriati Methanobacteriota Halosegnis rubeus WP 152133809.1
ArchaeaXII Archaea Methanobacteriati Methanobacteriota Halobacteriales archaeon QH 7 65 31 PSP40024.1
ArchaeaXII Archaea Methanobacteriati Methanobacteriota Halocatena marina WP 264555511.1
ArchaeaXII Archaea Methanobacteriati Methanobacteriota Halocatena halophila WP 330630999.1
ArchaeaXII Archaea Methanobacteriati Methanobacteriota Halocatena pleomorpha WP 124954711.1
ArchaeaXII Archaea Methanobacteriati Methanobacteriota Halocatena salina WP 247992873.1
ArchaeaXII Archaea Methanobacteriati Methanobacteriota Halomarina halobia WP 276303224.1
ArchaeaXII Archaea Methanobacteriati Methanobacteriota Halomarina ordinaria WP 304448867.1
ArchaeaXII Archaea Methanobacteriati Methanobacteriota Halobacteriales archaeon QS 5 70 17 PSQ02502.1
ArchaeaXII Archaea Methanobacteriati Methanobacteriota Halomarina litorea WP 254536528.1
ArchaeaXII Archaea Methanobacteriati Methanobacteriota Halomarina oriensis WP 158204274.1
ArchaeaXII Archaea Methanobacteriati Methanobacteriota Halomarina rubra WP 250873233.1
ArchaeaXII Archaea Methanobacteriati Methanobacteriota Halomarina salina WP 247414424.1
ArchaeaXII Archaea Methanobacteriati Methanobacteriota Halalkalicoccus ordinarius WP 336342634.1
ArchaeaXII Archaea Methanobacteriati Methanobacteriota Halalkalicoccus salilacus WP 332098612.1
ArchaeaXII Archaea Methanobacteriati Methanobacteriota Natronorarus salvus WP 331235185.1
ArchaeaXII Archaea Methanobacteriati Methanobacteriota Halalkalicoccus sp. MEM4781762.1
ArchaeaXII Archaea Methanobacteriati Methanobacteriota Halalkalicoccus subterraneus WP 122089478.1
ArchaeaXII Archaea Methanobacteriati Methanobacteriota Haladaptatus sp. CMSO5 WP 332897403.1
ArchaeaXII Archaea Methanobacteriati Methanobacteriota unclassified Haladaptatus WP 276246099.1
ArchaeaXII Archaea Methanobacteriati Methanobacteriota Halobacteriales archaeon QH 6 64 20 PSP43833.1
ArchaeaXII Archaea Methanobacteriati Methanobacteriota Halobacteriales archaeon QS 8 65 32 PSQ20666.1
ArchaeaXII Archaea Methanobacteriati Methanobacteriota Halobacteriales archaeon QH 8 64 26 PSP65315.1
ArchaeaXII Archaea Methanobacteriati Methanobacteriota Halobacteriales archaeon QS 3 64 16 PSP72228.1
ArchaeaXII Archaea Methanobacteriati Methanobacteriota Haladaptatus sp. R4 WP 066139988.1
ArchaeaXII Archaea Methanobacteriati Methanobacteriota Haladaptatus WP 007976051.1
ArchaeaXII Archaea Methanobacteriati Methanobacteriota Haladaptatus sp. NG-SE-30 WP 458207352.1
ArchaeaXII Archaea Methanobacteriati Methanobacteriota Haladaptatus sp. NG-WS-4 WP 458185809.1
ArchaeaXII Archaea Methanobacteriati Methanobacteriota Haladaptatus caseinilyticus WP 266079319.1
ArchaeaXII Archaea Methanobacteriati Methanobacteriota Haladaptatus halobius WP 227375053.1
ArchaeaXII Archaea Methanobacteriati Methanobacteriota Halomicrococcus sp. NG-SE-24 WP 433626123.1
ArchaeaXII Archaea Methanobacteriati Methanobacteriota Halomicrococcus gelatinilyticus WP 327050521.1
ArchaeaXII Archaea Methanobacteriati Methanobacteriota Halorussus halophilus WP 158058204.1
ArchaeaXII Archaea Methanobacteriati Methanobacteriota Halorussus WP 253512224.1
ArchaeaXII Archaea Methanobacteriati Methanobacteriota Halobacteriales archaeon QS 1 67 19 PSP56533.1
ArchaeaXII Archaea Methanobacteriati Methanobacteriota Halorussus salilacus WP 253477917.1
ArchaeaXII Archaea Methanobacteriati Methanobacteriota Halorussus amylolyticus WP 132059071.1
ArchaeaXII Archaea Methanobacteriati Methanobacteriota Halorussus aquaticus WP 254269404.1
ArchaeaXII Archaea Methanobacteriati Methanobacteriota Halorussus ruber WP 135824865.1
ArchaeaXII Archaea Methanobacteriati Methanobacteriota Halorussus gelatinilyticus WP 248656361.1
ArchaeaXII Archaea Methanobacteriati Methanobacteriota Halobacteriales archaeon SW 6 65 15 PSQ49968.1
ArchaeaXII Archaea Methanobacteriati Methanobacteriota Halorussus marinus WP 134668562.1
ArchaeaXII Archaea Methanobacteriati Methanobacteriota Halorussus WP 115798421.1
ArchaeaXII Archaea Methanobacteriati Methanobacteriota Halorussus halobius WP 135829843.1
ArchaeaXII Archaea Methanobacteriati Methanobacteriota Halorussus litoreus WP 115863257.1
ArchaeaXII Archaea Methanobacteriati Methanobacteriota Haloarchaeobius sp. FL176 WP 257299349.1
ArchaeaXII Archaea Methanobacteriati Methanobacteriota Haloarchaeobius sp. HRN-SO-5 WP 435348131.1
ArchaeaXII Archaea Methanobacteriati Methanobacteriota Haloarchaeobius sp. HME9146 WP 261648883.1
ArchaeaXII Archaea Methanobacteriati Methanobacteriota Haloarchaeobius sp. DFWS5 WP 435360135.1
ArchaeaXII Archaea Methanobacteriati Methanobacteriota Haloarchaeobius sp. TZWWS8 WP 435334550.1
ArchaeaXII Archaea Methanobacteriati Methanobacteriota Halostella litorea WP 135821872.1
ArchaeaXII Archaea Methanobacteriati Methanobacteriota Halostella salina WP 121820876.1
ArchaeaXII Archaea Methanobacteriati Methanobacteriota Halostella sp. PRR32 WP 323191164.1
ArchaeaXII Archaea Methanobacteriati Methanobacteriota Halostella WP 121816603.1
ArchaeaXII Archaea Methanobacteriati Methanobacteriota Natranaeroarchaeum sulfidigenes WP 238478334.1
ArchaeaXII Archaea Methanobacteriati Methanobacteriota Natronoarchaeum sp. GCM10025321 WP 377056223.1
ArchaeaXII Archaea Methanobacteriati Methanobacteriota Natronoarchaeum philippinense WP 097009477.1
ArchaeaXII Archaea Methanobacteriati Methanobacteriota Natronoarchaeum rubrum WP 256393164.1
ArchaeaXII Archaea Methanobacteriati Methanobacteriota Halobacteriales archaeon QS 8 69 26 PSQ19159.1

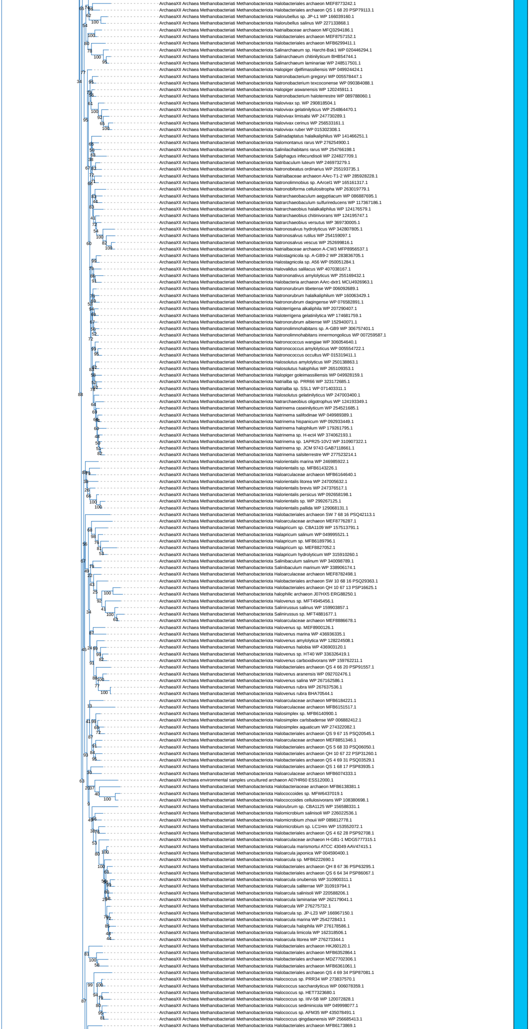
ArchaeaXII Archaea Methanobacteriati Methanobacteriota Halobacteriales archaeon MEF8773242.1
ArchaeaXII Archaea Methanobacteriati Methanobacteriota Halobacteriales archaeon QS 1 68 20 PSP79113.1
ArchaeaXII Archaea Methanobacteriati Methanobacteriota Halorubellus sp. JP-L1 WP 166039160.1
ArchaeaXII Archaea Methanobacteriati Methanobacteriota Halorubellus salinus WP 227133868.1
ArchaeaXII Archaea Methanobacteriati Methanobacteriota Natrialbaceae archaeon MFQ3294186.1
ArchaeaXII Archaea Methanobacteriati Methanobacteriota Halobacteriales archaeon MEF8757152.1
ArchaeaXII Archaea Methanobacteriati Methanobacteriota Halobacteriales archaeon MFB6299411.1
ArchaeaXII Archaea Methanobacteriati Methanobacteriota Salinarchaeum sp. Harcht-Bsk1 WP 020446294.1
ArchaeaXII Archaea Methanobacteriati Methanobacteriota Salinarchaeum chitinilyticum BHB54744.1
ArchaeaXII Archaea Methanobacteriati Methanobacteriota Salinarchaeum laminariae WP 248517501.1
ArchaeaXII Archaea Methanobacteriati Methanobacteriota Halopiger djelfimassiliensis WP 049924424.1
ArchaeaXII Archaea Methanobacteriati Methanobacteriota Natronobacterium gregoryi WP 005578447.1
ArchaeaXII Archaea Methanobacteriati Methanobacteriota Natronobacterium texcoconense WP 090384088.1
ArchaeaXII Archaea Methanobacteriati Methanobacteriota Halopiger aswanensis WP 120245911.1
ArchaeaXII Archaea Methanobacteriati Methanobacteriota Natronobacterium haloterrestre WP 089788060.1
ArchaeaXII Archaea Methanobacteriati Methanobacteriota Halovivax sp. WP 290818504.1
ArchaeaXII Archaea Methanobacteriati Methanobacteriota Halovivax gelatinilyticus WP 254864470.1
ArchaeaXII Archaea Methanobacteriati Methanobacteriota Halovivax limisalsi WP 247730289.1
ArchaeaXII Archaea Methanobacteriati Methanobacteriota Halovivax cerinus WP 256533161.1
ArchaeaXII Archaea Methanobacteriati Methanobacteriota Halovivax ruber WP 015302308.1
ArchaeaXII Archaea Methanobacteriati Methanobacteriota Salinadaptatus halalkaliphilus WP 141466251.1
ArchaeaXII Archaea Methanobacteriati Methanobacteriota Halomontanus rarus WP 276254900.1
ArchaeaXII Archaea Methanobacteriati Methanobacteriota Salinilacihabitans rarus WP 254766198.1
ArchaeaXII Archaea Methanobacteriati Methanobacteriota Saliphagus infecundisoli WP 224827709.1
ArchaeaXII Archaea Methanobacteriati Methanobacteriota Natribaculum luteum WP 246973279.1
ArchaeaXII Archaea Methanobacteriati Methanobacteriota Natronobeatus ordinarius WP 255193735.1
ArchaeaXII Archaea Methanobacteriati Methanobacteriota Natrialbaceae archaeon AArc-T1-2 WP 285928228.1
ArchaeaXII Archaea Methanobacteriati Methanobacteriota Natronolimnobius sp. AArcel1 WP 165161317.1
ArchaeaXII Archaea Methanobacteriati Methanobacteriota Natronobiforma cellulositropha WP 263019779.1
ArchaeaXII Archaea Methanobacteriati Methanobacteriota Natrarchaeobaculum aegyptiacum WP 086887695.1
ArchaeaXII Archaea Methanobacteriati Methanobacteriota Natrarchaeobaculum sulfurireducens WP 117367186.1
ArchaeaXII Archaea Methanobacteriati Methanobacteriota Natrarchaeobius halalkaliphilus WP 124176579.1
ArchaeaXII Archaea Methanobacteriati Methanobacteriota Natrarchaeobius chitinivorans WP 124195747.1
ArchaeaXII Archaea Methanobacteriati Methanobacteriota Natrarchaeobius versutus WP 369730005.1
ArchaeaXII Archaea Methanobacteriati Methanobacteriota Natronosalvus hydrolyticus WP 342807805.1
ArchaeaXII Archaea Methanobacteriati Methanobacteriota Natronosalvus rutilus WP 254159097.1
ArchaeaXII Archaea Methanobacteriati Methanobacteriota Natronosalvus vescus WP 252699816.1
ArchaeaXII Archaea Methanobacteriati Methanobacteriota Natrialbaceae archaeon A-CW3 MFP8956537.1
ArchaeaXII Archaea Methanobacteriati Methanobacteriota Halostagnicola sp. A-GB9-2 WP 283836705.1
ArchaeaXII Archaea Methanobacteriati Methanobacteriota Halostagnicola sp. A56 WP 050051284.1
ArchaeaXII Archaea Methanobacteriati Methanobacteriota Halovalidus salilacus WP 407038167.1
ArchaeaXII Archaea Methanobacteriati Methanobacteriota Natrononativus amylolyticus WP 255169432.1
ArchaeaXII Archaea Methanobacteriati Methanobacteriota Halobacteria archaeon AArc-dxtr1 MCU4926963.1
ArchaeaXII Archaea Methanobacteriati Methanobacteriota Natronorubrum tibetense WP 006092689.1
ArchaeaXII Archaea Methanobacteriati Methanobacteriota Natronorubrum halalkaliphilum WP 160063429.1
ArchaeaXII Archaea Methanobacteriati Methanobacteriota Natronorubrum daqingense WP 076582891.1
ArchaeaXII Archaea Methanobacteriati Methanobacteriota Haloterrigena alkaliphila WP 207290407.1
ArchaeaXII Archaea Methanobacteriati Methanobacteriota Haloterrigena gelatinilytica WP 174681759.1
ArchaeaXII Archaea Methanobacteriati Methanobacteriota Natronorubrum aibiense WP 152940071.1
ArchaeaXII Archaea Methanobacteriati Methanobacteriota Natronolimnohabitans sp. A-GB9 WP 306757401.1
ArchaeaXII Archaea Methanobacteriati Methanobacteriota Natronolimnohabitans innermongolicus WP 007259587.1
ArchaeaXII Archaea Methanobacteriati Methanobacteriota Natronococcus wangiae WP 306054640.1
ArchaeaXII Archaea Methanobacteriati Methanobacteriota Natronococcus amylolyticus WP 005554722.1
ArchaeaXII Archaea Methanobacteriati Methanobacteriota Natronococcus occultus WP 015319411.1
ArchaeaXII Archaea Methanobacteriati Methanobacteriota Halosolutus amylolyticus WP 250138863.1
ArchaeaXII Archaea Methanobacteriati Methanobacteriota Halosolutus halophilus WP 265109353.1
ArchaeaXII Archaea Methanobacteriati Methanobacteriota Halopiger goleimassiliensis WP 049928159.1
ArchaeaXII Archaea Methanobacteriati Methanobacteriota Natrialba sp. PRR66 WP 323172685.1
ArchaeaXII Archaea Methanobacteriati Methanobacteriota Natrialba sp. SSL1 WP 071403311.1
ArchaeaXII Archaea Methanobacteriati Methanobacteriota Halosolutus gelatinilyticus WP 247003400.1
ArchaeaXII Archaea Methanobacteriati Methanobacteriota Natrarchaeobius oligotrophus WP 124193349.1
ArchaeaXII Archaea Methanobacteriati Methanobacteriota Natrinema caseinilyticum WP 254521685.1
ArchaeaXII Archaea Methanobacteriati Methanobacteriota Natrinema salifodinae WP 049989389.1
ArchaeaXII Archaea Methanobacteriati Methanobacteriota Natrinema hispanicum WP 092933449.1
ArchaeaXII Archaea Methanobacteriati Methanobacteriota Natrinema halophilum WP 179261795.1
ArchaeaXII Archaea Methanobacteriati Methanobacteriota Natrinema sp. H-ect4 WP 374062193.1
ArchaeaXII Archaea Methanobacteriati Methanobacteriota Natrinema sp. 1APR25-10V2 WP 310907322.1
ArchaeaXII Archaea Methanobacteriati Methanobacteriota Natrinema sp. JCM 9743 GAB7118661.1
ArchaeaXII Archaea Methanobacteriati Methanobacteriota Natrinema salsiterrestre WP 277523214.1
ArchaeaXII Archaea Methanobacteriati Methanobacteriota Halorientalis marina WP 246985922.1
ArchaeaXII Archaea Methanobacteriati Methanobacteriota Halorientalis sp. MFB6143226.1
ArchaeaXII Archaea Methanobacteriati Methanobacteriota Haloarculaceae archaeon MFB6164640.1
ArchaeaXII Archaea Methanobacteriati Methanobacteriota Halorientalis litorea WP 247005632.1
ArchaeaXII Archaea Methanobacteriati Methanobacteriota Halorientalis brevis WP 247376517.1
ArchaeaXII Archaea Methanobacteriati Methanobacteriota Halorientalis persicus WP 092658198.1
ArchaeaXII Archaea Methanobacteriati Methanobacteriota Halorientalis sp. WP 299267125.1
ArchaeaXII Archaea Methanobacteriati Methanobacteriota Halorientalis pallida WP 129068131.1
ArchaeaXII Archaea Methanobacteriati Methanobacteriota Halobacteriales archaeon SW 7 68 16 PSQ42113.1
ArchaeaXII Archaea Methanobacteriati Methanobacteriota Haloarculaceae archaeon MEF8776287.1
ArchaeaXII Archaea Methanobacteriati Methanobacteriota Halapricum sp. CBA1109 WP 157513791.1
ArchaeaXII Archaea Methanobacteriati Methanobacteriota Halapricum salinum WP 049995521.1
ArchaeaXII Archaea Methanobacteriati Methanobacteriota Halapricum sp. MFB6189796.1
ArchaeaXII Archaea Methanobacteriati Methanobacteriota Halapricum sp. MEF8827052.1
ArchaeaXII Archaea Methanobacteriati Methanobacteriota Halapricum hydrolyticum WP 315910260.1
ArchaeaXII Archaea Methanobacteriati Methanobacteriota Salinibaculum salinum WP 340098789.1
ArchaeaXII Archaea Methanobacteriati Methanobacteriota Salinibaculum marinum WP 338906174.1
ArchaeaXII Archaea Methanobacteriati Methanobacteriota Haloarculaceae archaeon MEF8782498.1
ArchaeaXII Archaea Methanobacteriati Methanobacteriota Halobacteriales archaeon SW 10 68 16 PSQ29363.1
ArchaeaXII Archaea Methanobacteriati Methanobacteriota Halobacteriales archaeon QH 10 67 13 PSP16625.1
ArchaeaXII Archaea Methanobacteriati Methanobacteriota halophilic archaeon J07HX5 ERG88250.1
ArchaeaXII Archaea Methanobacteriati Methanobacteriota Halovenus sp. MFT4945456.1
ArchaeaXII Archaea Methanobacteriati Methanobacteriota Salinirussus salinus WP 159903857.1
ArchaeaXII Archaea Methanobacteriati Methanobacteriota Salinirussus sp. MFT4881677.1
ArchaeaXII Archaea Methanobacteriati Methanobacteriota Haloarculaceae archaeon MEF8886678.1
ArchaeaXII Archaea Methanobacteriati Methanobacteriota Halovenus sp. MEF8900126.1
ArchaeaXII Archaea Methanobacteriati Methanobacteriota Halovenus marina WP 436936335.1
ArchaeaXII Archaea Methanobacteriati Methanobacteriota Halovenus amylolytica WP 128224508.1
ArchaeaXII Archaea Methanobacteriati Methanobacteriota Halovenus halobia WP 436903120.1
ArchaeaXII Archaea Methanobacteriati Methanobacteriota Halovenus sp. HT40 WP 336326419.1
ArchaeaXII Archaea Methanobacteriati Methanobacteriota Halovenus carboxidivorans WP 159762211.1
ArchaeaXII Archaea Methanobacteriati Methanobacteriota Halobacteriales archaeon QS 4 66 20 PSP91557.1
ArchaeaXII Archaea Methanobacteriati Methanobacteriota Halovenus aranensis WP 092702476.1
ArchaeaXII Archaea Methanobacteriati Methanobacteriota Halovenus salina WP 267162586.1
ArchaeaXII Archaea Methanobacteriati Methanobacteriota Halovenus rubra WP 267637536.1
ArchaeaXII Archaea Methanobacteriati Methanobacteriota Halovenus rubra BHA70544.1
ArchaeaXII Archaea Methanobacteriati Methanobacteriota Haloarculaceae archaeon MFB6184221.1
ArchaeaXII Archaea Methanobacteriati Methanobacteriota Haloarculaceae archaeon MFB6151517.1
ArchaeaXII Archaea Methanobacteriati Methanobacteriota Halosimplex sp. MFB6140900.1
ArchaeaXII Archaea Methanobacteriati Methanobacteriota Halosimplex carlsbadense WP 006882412.1
ArchaeaXII Archaea Methanobacteriati Methanobacteriota Halosimplex aquaticum WP 274322082.1
ArchaeaXII Archaea Methanobacteriati Methanobacteriota Halobacteriales archaeon QS 9 67 15 PSQ20545.1
ArchaeaXII Archaea Methanobacteriati Methanobacteriota Haloarculaceae archaeon MEF8851346.1
ArchaeaXII Archaea Methanobacteriati Methanobacteriota Halobacteriales archaeon QS 5 68 33 PSQ06050.1
ArchaeaXII Archaea Methanobacteriati Methanobacteriota Halobacteriales archaeon QH 10 67 22 PSP31260.1
ArchaeaXII Archaea Methanobacteriati Methanobacteriota Halobacteriales archaeon QS 4 69 31 PSQ03529.1
ArchaeaXII Archaea Methanobacteriati Methanobacteriota Halobacteriales archaeon QS 1 68 17 PSP83935.1
ArchaeaXII Archaea Methanobacteriati Methanobacteriota Haloarculaceae archaeon MFB6074333.1
ArchaeaXII Archaea environmental samples uncultured archaeon A07HR60 ESS12000.1
ArchaeaXII Archaea Methanobacteriati Methanobacteriota Halobacteriaceae archaeon MFB6138381.1
ArchaeaXII Archaea Methanobacteriati Methanobacteriota Halococcoides sp. MFW6437019.1
ArchaeaXII Archaea Methanobacteriati Methanobacteriota Halococcoides cellulosivorans WP 108380698.1
ArchaeaXII Archaea Methanobacteriati Methanobacteriota Halorubrum sp. CBA1125 WP 156588331.1
ArchaeaXII Archaea Methanobacteriati Methanobacteriota Halomicrobium salinisoli WP 226022536.1
ArchaeaXII Archaea Methanobacteriati Methanobacteriota Halomicrobium zhouii WP 089812778.1
ArchaeaXII Archaea Methanobacteriati Methanobacteriota Halomicrobium sp. LC1Hm WP 153552072.1
ArchaeaXII Archaea Methanobacteriati Methanobacteriota Halobacteriales archaeon QS 4 62 28 PSP92708.1
ArchaeaXII Archaea Methanobacteriati Methanobacteriota Haloarculaceae archaeon H-GB1-1 MDG5777315.1
ArchaeaXII Archaea Methanobacteriati Methanobacteriota Haloarcula marismortui ATCC 43049 AAV47415.1
ArchaeaXII Archaea Methanobacteriati Methanobacteriota Haloarcula japonica WP 004590400.1
ArchaeaXII Archaea Methanobacteriati Methanobacteriota Haloarcula sp. MFB6222690.1
ArchaeaXII Archaea Methanobacteriati Methanobacteriota Halobacteriales archaeon QH 8 67 36 PSP63295.1
ArchaeaXII Archaea Methanobacteriati Methanobacteriota Halobacteriales archaeon QS 6 64 34 PSP86067.1
ArchaeaXII Archaea Methanobacteriati Methanobacteriota Haloarcula onubensis WP 310900311.1
ArchaeaXII Archaea Methanobacteriati Methanobacteriota Haloarcula saliterrae WP 310919794.1
ArchaeaXII Archaea Methanobacteriati Methanobacteriota Haloarcula salinisoli WP 220588206.1
ArchaeaXII Archaea Methanobacteriati Methanobacteriota Haloarcula laminariae WP 262179041.1
ArchaeaXII Archaea Methanobacteriati Methanobacteriota Haloarcula WP 276275732.1
ArchaeaXII Archaea Methanobacteriati Methanobacteriota Haloarcula sp. JP-L23 WP 166967150.1
ArchaeaXII Archaea Methanobacteriati Methanobacteriota Haloarcula marina WP 254272843.1
ArchaeaXII Archaea Methanobacteriati Methanobacteriota Haloarcula halophila WP 276178586.1
ArchaeaXII Archaea Methanobacteriati Methanobacteriota Haloarcula limicola WP 162318506.1
ArchaeaXII Archaea Methanobacteriati Methanobacteriota Haloarcula litorea WP 276273344.1
ArchaeaXII Archaea Methanobacteriati Methanobacteriota Halobacteriales archaeon HKJ60120.1
ArchaeaXII Archaea Methanobacteriati Methanobacteriota Halobacteriales archaeon MFB6352864.1
ArchaeaXII Archaea Methanobacteriati Methanobacteriota Halobacteriales archaeon MDZ7702306.1
ArchaeaXII Archaea Methanobacteriati Methanobacteriota Halobacteriales archaeon MFB6361061.1
ArchaeaXII Archaea Methanobacteriati Methanobacteriota Halobacteriales archaeon QS 4 69 34 PSP87081.1
ArchaeaXII Archaea Methanobacteriati Methanobacteriota Halococcus sp. PRR34 WP 273837570.1
ArchaeaXII Archaea Methanobacteriati Methanobacteriota Halococcus saccharolyticus WP 006078359.1
ArchaeaXII Archaea Methanobacteriati Methanobacteriota Halococcus sp. HET7323680.1
ArchaeaXII Archaea Methanobacteriati Methanobacteriota Halococcus sp. IIIV-5B WP 120072828.1
ArchaeaXII Archaea Methanobacteriati Methanobacteriota Halococcus sediminicola WP 049998077.1
ArchaeaXII Archaea Methanobacteriati Methanobacteriota Halococcus sp. AFM35 WP 435078491.1
ArchaeaXII Archaea Methanobacteriati Methanobacteriota Halococcus qingdaonensis WP 256685413.1
ArchaeaXII Archaea Methanobacteriati Methanobacteriota Halobacteriales archaeon MFB6173869.1

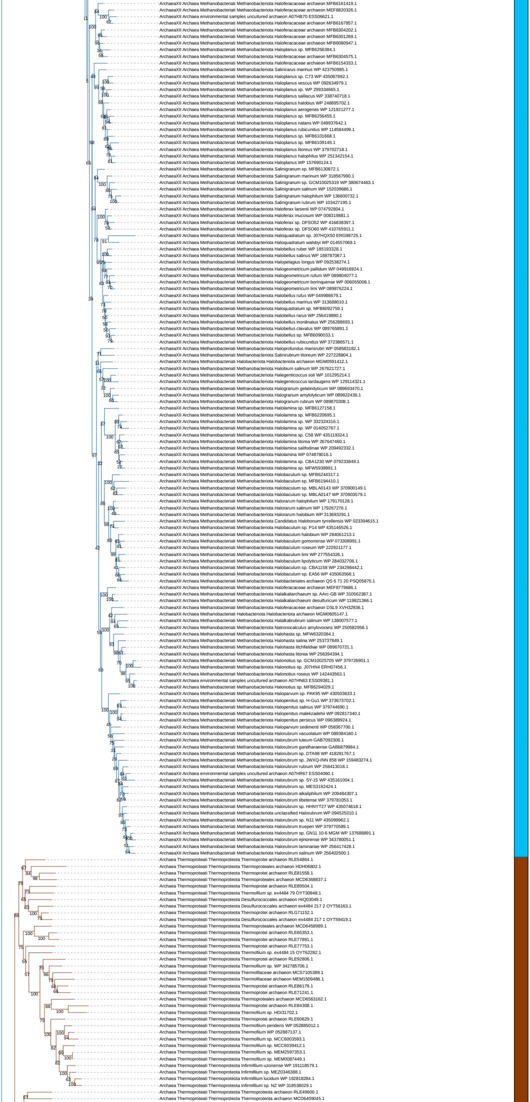
ArchaeaXII Archaea Methanobacteriati Methanobacteriota Haloferacaceae archaeon MFB6161419.1
ArchaeaXII Archaea Methanobacteriati Methanobacteriota Haloferacaceae archaeon MEF8820326.1
ArchaeaXII Archaea environmental samples uncultured archaeon A07HB70 ESS06621.1
ArchaeaXII Archaea Methanobacteriati Methanobacteriota Haloferacaceae archaeon MFB6167857.1
ArchaeaXII Archaea Methanobacteriati Methanobacteriota Haloferacaceae archaeon MFB6304202.1
ArchaeaXII Archaea Methanobacteriati Methanobacteriota Haloferacaceae archaeon MFB6301269.1
ArchaeaXII Archaea Methanobacteriati Methanobacteriota Haloferacaceae archaeon MFB6080947.1
ArchaeaXII Archaea Methanobacteriati Methanobacteriota Haloplanus sp. MFB6256384.1
ArchaeaXII Archaea Methanobacteriati Methanobacteriota Haloferacaceae archaeon MFB6304575.1
ArchaeaXII Archaea Methanobacteriati Methanobacteriota Haloferacaceae archaeon MFB6154333.1
ArchaeaXII Archaea Methanobacteriati Methanobacteriota Salinirarus marinus WP 423750985.1
ArchaeaXII Archaea Methanobacteriati Methanobacteriota Haloplanus sp. C73 WP 435067892.1
ArchaeaXII Archaea Methanobacteriati Methanobacteriota Haloplanus vescus WP 092634979.1
ArchaeaXII Archaea Methanobacteriati Methanobacteriota Haloplanus sp. WP 299334665.1
ArchaeaXII Archaea Methanobacteriati Methanobacteriota Haloplanus salilacus WP 338740718.1
ArchaeaXII Archaea Methanobacteriati Methanobacteriota Haloplanus halobius WP 248895702.1
ArchaeaXII Archaea Methanobacteriati Methanobacteriota Haloplanus aerogenes WP 121921277.1
ArchaeaXII Archaea Methanobacteriati Methanobacteriota Haloplanus sp. MFB6256455.1
ArchaeaXII Archaea Methanobacteriati Methanobacteriota Haloplanus natans WP 049937642.1
ArchaeaXII Archaea Methanobacteriati Methanobacteriota Haloplanus rubicundus WP 114584499.1
ArchaeaXII Archaea Methanobacteriati Methanobacteriota Haloplanus sp. MFB6101668.1
ArchaeaXII Archaea Methanobacteriati Methanobacteriota Haloplanus sp. MFB6109145.1
ArchaeaXII Archaea Methanobacteriati Methanobacteriota Haloplanus litoreus WP 379702718.1
ArchaeaXII Archaea Methanobacteriati Methanobacteriota Haloplanus halophilus WP 251342154.1
ArchaeaXII Archaea Methanobacteriati Methanobacteriota Haloplanus WP 157690124.1
ArchaeaXII Archaea Methanobacteriati Methanobacteriota Salinigranum sp. MFB6130872.1
ArchaeaXII Archaea Methanobacteriati Methanobacteriota Salinigranum marinum WP 318567990.1
ArchaeaXII Archaea Methanobacteriati Methanobacteriota Salinigranum sp. GCM10025319 WP 380674463.1
ArchaeaXII Archaea Methanobacteriati Methanobacteriota Salinigranum salinum WP 152039686.1
ArchaeaXII Archaea Methanobacteriati Methanobacteriota Salinigranum halophilum WP 136600732.1
ArchaeaXII Archaea Methanobacteriati Methanobacteriota Salinigranum rubrum WP 103427195.1
ArchaeaXII Archaea Methanobacteriati Methanobacteriota Haloferax larsenii WP 074792804.1
ArchaeaXII Archaea Methanobacteriati Methanobacteriota Haloferax mucosum WP 008319881.1
ArchaeaXII Archaea Methanobacteriati Methanobacteriota Haloferax sp. DFSO52 WP 416838397.1
ArchaeaXII Archaea Methanobacteriati Methanobacteriota Haloferax sp. DFSO60 WP 410765911.1
ArchaeaXII Archaea Methanobacteriati Methanobacteriota Haloquadratum sp. J07HQX50 ERG98725.1
ArchaeaXII Archaea Methanobacteriati Methanobacteriota Haloquadratum walsbyi WP 014557069.1
ArchaeaXII Archaea Methanobacteriati Methanobacteriota Halobellus ruber WP 185193328.1
ArchaeaXII Archaea Methanobacteriati Methanobacteriota Halobellus salinus WP 188787067.1
ArchaeaXII Archaea Methanobacteriati Methanobacteriota Halopelagius longus WP 092538274.1
ArchaeaXII Archaea Methanobacteriati Methanobacteriota Halogeometricum pallidum WP 049916924.1
ArchaeaXII Archaea Methanobacteriati Methanobacteriota Halogeometricum rufum WP 089804077.1
ArchaeaXII Archaea Methanobacteriati Methanobacteriota Halogeometricum borinquense WP 006055008.1
ArchaeaXII Archaea Methanobacteriati Methanobacteriota Halogeometricum limi WP 089876224.1
ArchaeaXII Archaea Methanobacteriati Methanobacteriota Halobellus rufus WP 049986679.1
ArchaeaXII Archaea Methanobacteriati Methanobacteriota Halobellus marinus WP 313688010.1
ArchaeaXII Archaea Methanobacteriati Methanobacteriota Haloquadratum sp. MFB6092759.1
ArchaeaXII Archaea Methanobacteriati Methanobacteriota Halobellus rarus WP 256419890.1
ArchaeaXII Archaea Methanobacteriati Methanobacteriota Halobellus inordinatus WP 256288693.1
ArchaeaXII Archaea Methanobacteriati Methanobacteriota Halobellus clavatus WP 089765891.1
ArchaeaXII Archaea Methanobacteriati Methanobacteriota Halobellus sp. MFB6090033.1
ArchaeaXII Archaea Methanobacteriati Methanobacteriota Halobellus rubicundus WP 372386571.1
ArchaeaXII Archaea Methanobacteriati Methanobacteriota Haloprofundus marisrubri WP 058583182.1
ArchaeaXII Archaea Methanobacteriati Methanobacteriota Salinirubrum litoreum WP 227228804.1
ArchaeaXII Archaea Methanobacteriati Halobacteriota Halobacteriota archaeon MGM0591412.1
ArchaeaXII Archaea Methanobacteriati Methanobacteriota Halobium salinum WP 267621727.1
ArchaeaXII Archaea Methanobacteriati Methanobacteriota Halegenticoccus soli WP 101295214.1
ArchaeaXII Archaea Methanobacteriati Methanobacteriota Halegenticoccus tardaugens WP 129114321.1
ArchaeaXII Archaea Methanobacteriati Methanobacteriota Halogranum gelatinilyticum WP 089693470.1
ArchaeaXII Archaea Methanobacteriati Methanobacteriota Halogranum amylolyticum WP 089822436.1
ArchaeaXII Archaea Methanobacteriati Methanobacteriota Halogranum rubrum WP 089870308.1
ArchaeaXII Archaea Methanobacteriati Methanobacteriota Halolamina sp. MFB6127158.1
ArchaeaXII Archaea Methanobacteriati Methanobacteriota Halolamina sp. MFB6220695.1
ArchaeaXII Archaea Methanobacteriati Methanobacteriota Halolamina sp. WP 332324316.1
ArchaeaXII Archaea Methanobacteriati Methanobacteriota Halolamina sp. WP 014052767.1
ArchaeaXII Archaea Methanobacteriati Methanobacteriota Halolamina sp. C58 WP 435119324.1
ArchaeaXII Archaea Methanobacteriati Methanobacteriota Halolamina litorea WP 267647460.1
ArchaeaXII Archaea Methanobacteriati Methanobacteriota Halolamina salifodinae WP 209492332.1
ArchaeaXII Archaea Methanobacteriati Methanobacteriota Halolamina WP 074878016.1
ArchaeaXII Archaea Methanobacteriati Methanobacteriota Halolamina sp. CBA1230 WP 079233849.1
ArchaeaXII Archaea Methanobacteriati Methanobacteriota Halolamina sp. MFW5938991.1
ArchaeaXII Archaea Methanobacteriati Methanobacteriota Halobaculum sp. MFB6244317.1
ArchaeaXII Archaea Methanobacteriati Methanobacteriota Halobaculum sp. MFB6194410.1
ArchaeaXII Archaea Methanobacteriati Methanobacteriota Halobaculum sp. MBLA0143 WP 370900149.1
ArchaeaXII Archaea Methanobacteriati Methanobacteriota Halobaculum sp. MBLA0147 WP 370903579.1
ArchaeaXII Archaea Methanobacteriati Methanobacteriota Halorarum halophilum WP 179170128.1
ArchaeaXII Archaea Methanobacteriati Methanobacteriota Halorarum salinum WP 179267276.1
ArchaeaXII Archaea Methanobacteriati Methanobacteriota Halorarum halobium WP 313693291.1
ArchaeaXII Archaea Methanobacteriati Methanobacteriota Candidatus Halobonum tyrrellensis WP 023394615.1
ArchaeaXII Archaea Methanobacteriati Methanobacteriota Halobaculum sp. P14 WP 435146526.1
ArchaeaXII Archaea Methanobacteriati Methanobacteriota Halobaculum halobium WP 284061213.1
ArchaeaXII Archaea Methanobacteriati Methanobacteriota Halobaculum gomorrense WP 073308991.1
ArchaeaXII Archaea Methanobacteriati Methanobacteriota Halobaculum roseum WP 222921177.1
ArchaeaXII Archaea Methanobacteriati Methanobacteriota Halobaculum limi WP 277554326.1
ArchaeaXII Archaea Methanobacteriati Methanobacteriota Halobaculum lipolyticum WP 284032706.1
ArchaeaXII Archaea Methanobacteriati Methanobacteriota Halobaculum sp. CBA1158 WP 234298442.1
ArchaeaXII Archaea Methanobacteriati Methanobacteriota Halobaculum sp. EA56 WP 435063568.1
ArchaeaXII Archaea Methanobacteriati Methanobacteriota Halobacteriales archaeon QS 6 71 20 PSQ05875.1
ArchaeaXII Archaea Methanobacteriati Methanobacteriota Haloferacaceae archaeon MEF8779686.1
ArchaeaXII Archaea Methanobacteriati Methanobacteriota Halalkaliarchaeum sp. AArc-GB WP 310562387.1
ArchaeaXII Archaea Methanobacteriati Methanobacteriota Halalkaliarchaeum desulfuricum WP 119821366.1
ArchaeaXII Archaea Methanobacteriati Methanobacteriota Haloferacaceae archaeon DSL9 XVH32936.1
ArchaeaXII Archaea Methanobacteriati Halobacteriota Halobacteriota archaeon MGM0605147.1
ArchaeaXII Archaea Methanobacteriati Methanobacteriota Halalkalirubrum salinum WP 138007577.1
ArchaeaXII Archaea Methanobacteriati Methanobacteriota Natronocalculus amylovorans WP 250582956.1
ArchaeaXII Archaea Methanobacteriati Methanobacteriota Halohasta sp. MFW6320384.1
ArchaeaXII Archaea Methanobacteriati Methanobacteriota Halohasta salina WP 253737849.1
ArchaeaXII Archaea Methanobacteriati Methanobacteriota Halohasta litchfieldiae WP 089670721.1
ArchaeaXII Archaea Methanobacteriati Methanobacteriota Halohasta litorea WP 256394394.1
ArchaeaXII Archaea Methanobacteriati Methanobacteriota Halonotius sp. GCM10025705 WP 379726901.1
ArchaeaXII Archaea Methanobacteriati Methanobacteriota Halonotius sp. J07HN4 ERH07456.1
ArchaeaXII Archaea Methanobacteriati Methanobacteriota Halonotius roseus WP 142443563.1
ArchaeaXII Archaea environmental samples uncultured archaeon A07HN63 ESS09381.1
ArchaeaXII Archaea Methanobacteriati Methanobacteriota Halonotius sp. MFB6294029.1
ArchaeaXII Archaea Methanobacteriati Methanobacteriota Haloparvum sp. PAK95 WP 430503633.1
ArchaeaXII Archaea Methanobacteriati Methanobacteriota Halopenitus sp. H-Gu1 WP 373673702.1
ArchaeaXII Archaea Methanobacteriati Methanobacteriota Halopenitus salinus WP 379744690.1
ArchaeaXII Archaea Methanobacteriati Methanobacteriota Halopenitus malekzadehii WP 092817340.1
ArchaeaXII Archaea Methanobacteriati Methanobacteriota Halopenitus persicus WP 096389924.1
ArchaeaXII Archaea Methanobacteriati Methanobacteriota Haloparvum sedimenti WP 058367700.1
ArchaeaXII Archaea Methanobacteriati Methanobacteriota Halorubrum vacuolatum WP 089384160.1
ArchaeaXII Archaea Methanobacteriati Methanobacteriota Halorubrum luteum GAB7092306.1
ArchaeaXII Archaea Methanobacteriati Methanobacteriota Halorubrum gandharaense GAB6879984.1
ArchaeaXII Archaea Methanobacteriati Methanobacteriota Halorubrum sp. DTA98 WP 418281767.1
ArchaeaXII Archaea Methanobacteriati Methanobacteriota Halorubrum sp. JWXQ-INN 858 WP 159483274.1
ArchaeaXII Archaea Methanobacteriati Methanobacteriota Halorubrum rubrum WP 256413018.1
ArchaeaXII Archaea environmental samples uncultured archaeon A07HR67 ESS04060.1
ArchaeaXII Archaea Methanobacteriati Methanobacteriota Halorubrum sp. SY-15 WP 435161004.1
ArchaeaXII Archaea Methanobacteriati Methanobacteriota Halorubrum sp. MES3162424.1
ArchaeaXII Archaea Methanobacteriati Methanobacteriota Halorubrum alkaliphilum WP 209484307.1
ArchaeaXII Archaea Methanobacteriati Methanobacteriota Halorubrum tibetense WP 379781053.1
ArchaeaXII Archaea Methanobacteriati Methanobacteriota Halorubrum sp. HHNYT27 WP 435074618.1
ArchaeaXII Archaea Methanobacteriati Methanobacteriota unclassified Halorubrum WP 094525310.1
ArchaeaXII Archaea Methanobacteriati Methanobacteriota Halorubrum sp. N11 WP 435099962.1
ArchaeaXII Archaea Methanobacteriati Methanobacteriota Halorubrum trueperi WP 379770589.1
ArchaeaXII Archaea Methanobacteriati Methanobacteriota Halorubrum sp. GN11 10-6 MGM WP 137688891.1
ArchaeaXII Archaea Methanobacteriati Methanobacteriota Halorubrum ejinorense WP 343780051.1
ArchaeaXII Archaea Methanobacteriati Methanobacteriota Halorubrum laminariae WP 256417428.1
ArchaeaXII Archaea Methanobacteriati Methanobacteriota Halorubrum salinum WP 256402500.1
Archaea Thermoproteati Thermoproteota Thermoprotei archaeon RLE54864.1
Archaea Thermoproteati Thermoproteota Thermoproteales archaeon HDH06802.1
Archaea Thermoproteati Thermoproteota Thermoprotei archaeon RLE81558.1
Archaea Thermoproteati Thermoproteota Thermoproteales archaeon MCD6368837.1
Archaea Thermoproteati Thermoproteota Thermoprotei archaeon RLE89504.1
Archaea Thermoproteati Thermoproteota Thermofilum sp. ex4484 79 OYT30948.1
Archaea Thermoproteati Thermoproteota Desulfurococcales archaeon HIQ03049.1
Archaea Thermoproteati Thermoproteota Desulfurococcales archaeon ex4484 217 2 OYT56163.1
Archaea Thermoproteati Thermoproteota Thermoprotei archaeon RLG71152.1
Archaea Thermoproteati Thermoproteota Desulfurococcales archaeon ex4484 217 1 OYT59419.1
Archaea Thermoproteati Thermoproteota Thermoproteales archaeon MCD6458989.1
Archaea Thermoproteati Thermoproteota Thermoprotei archaeon RLE65353.1
Archaea Thermoproteati Thermoproteota Thermoprotei archaeon RLE77891.1
Archaea Thermoproteati Thermoproteota Thermoprotei archaeon RLE77753.1
Archaea Thermoproteati Thermoproteota Thermofilum sp. ex4484 15 OYT62282.1
Archaea Thermoproteati Thermoproteota Thermoprotei archaeon RLE92806.1
Archaea Thermoproteati Thermoproteota Thermofilum sp. WP 342785706.1
Archaea Thermoproteati Thermoproteota Thermofilaceae archaeon MCS7105389.1
Archaea Thermoproteati Thermoproteota Thermofilaceae archaeon MEM1509486.1
Archaea Thermoproteati Thermoproteota Thermoprotei archaeon RLE86179.1
Archaea Thermoproteati Thermoproteota Thermoprotei archaeon RLE71241.1
Archaea Thermoproteati Thermoproteota Thermoproteales archaeon MCD6563162.1
Archaea Thermoproteati Thermoproteota Thermoprotei archaeon RLE84308.1
Archaea Thermoproteati Thermoproteota Thermofilum sp. HDI31702.1
Archaea Thermoproteati Thermoproteota Thermoprotei archaeon RLE60629.1
Archaea Thermoproteati Thermoproteota Thermofilum pendens WP 052885012.1
Archaea Thermoproteati Thermoproteota Thermofilum WP 052887137.1
Archaea Thermoproteati Thermoproteota Thermofilum sp. MCC6003593.1
Archaea Thermoproteati Thermoproteota Thermofilum sp. MCC6039412.1
Archaea Thermoproteati Thermoproteota Thermofilum sp. MEM2597353.1
Archaea Thermoproteati Thermoproteota Thermofilum sp. MEM0087449.1
Archaea Thermoproteati Thermoproteota Infirmifilum uzonense WP 191118579.1
Archaea Thermoproteati Thermoproteota Infirmifilum sp. MEZ0346388.1
Archaea Thermoproteati Thermoproteota Infirmifilum lucidum WP 192818284.1
Archaea Thermoproteati Thermoproteota Infirmifilum sp. NZ WP 318538029.1
Archaea Thermoproteati Thermoproteota Thermoproteota archaeon RLE49600.1
Archaea Thermoproteati Thermoproteota Thermoproteota archaeon MCD6409045.1

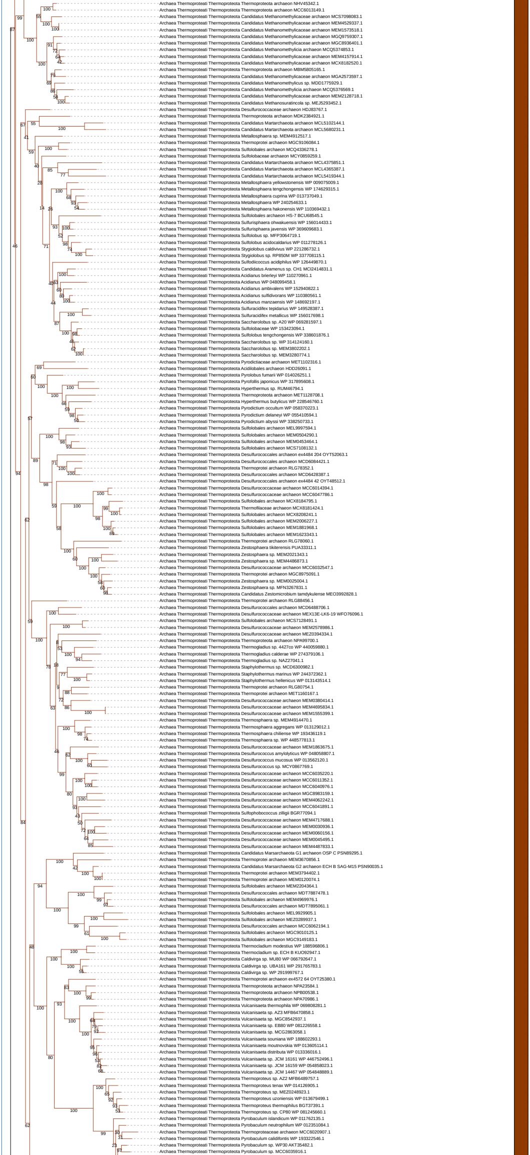
Archaea Thermoproteati Thermoproteota Thermoproteota archaeon NHV45342.1
Archaea Thermoproteati Thermoproteota Thermoproteota archaeon MCC6013149.1
Archaea Thermoproteati Thermoproteota Candidatus Methanomethylicaceae archaeon MCS7098083.1
Archaea Thermoproteati Thermoproteota Candidatus Methanomethylicaceae archaeon MEM4529337.1
Archaea Thermoproteati Thermoproteota Candidatus Methanomethylicaceae archaeon MEM1573518.1
Archaea Thermoproteati Thermoproteota Candidatus Methanomethylicaceae archaeon MGQ9759307.1
Archaea Thermoproteati Thermoproteota Candidatus Methanomethylicaceae archaeon MGC8936401.1
Archaea Thermoproteati Thermoproteota Candidatus Methanomethylicia archaeon MCQ5374853.1
Archaea Thermoproteati Thermoproteota Candidatus Methanomethylicaceae archaeon MEM4157914.1
Archaea Thermoproteati Thermoproteota Candidatus Methanomethylicaceae archaeon MCX8182520.1
Archaea Thermoproteati Thermoproteota Thermoproteota archaeon MBM5805165.1
Archaea Thermoproteati Thermoproteota Candidatus Methanomethylicaceae archaeon MGA2573597.1
Archaea Thermoproteati Thermoproteota Candidatus Methanomethylicus sp. MDD1775929.1
Archaea Thermoproteati Thermoproteota Candidatus Methanomethylicia archaeon MCQ5376569.1
Archaea Thermoproteati Thermoproteota Candidatus Methanomethylicaceae archaeon MEM2128718.1
Archaea Thermoproteati Thermoproteota Candidatus Methanosuratincola sp. MEJ5293452.1
Archaea Thermoproteati Thermoproteota Desulfurococcaceae archaeon HDJ83767.1
Archaea Thermoproteati Thermoproteota Thermoproteota archaeon MDK2384921.1
Archaea Thermoproteati Thermoproteota Candidatus Martarchaeota archaeon MCL5102144.1
Archaea Thermoproteati Thermoproteota Candidatus Martarchaeota archaeon MCL5680231.1
Archaea Thermoproteati Thermoproteota Metallosphaera sp. MEM4912517.1
Archaea Thermoproteati Thermoproteota Thermoprotei archaeon MGC9106084.1
Archaea Thermoproteati Thermoproteota Sulfolobales archaeon MCQ4336278.1
Archaea Thermoproteati Thermoproteota Sulfolobaceae archaeon MCY0859259.1
Archaea Thermoproteati Thermoproteota Candidatus Martarchaeota archaeon MCL4375851.1
Archaea Thermoproteati Thermoproteota Candidatus Martarchaeota archaeon MCL4365387.1
Archaea Thermoproteati Thermoproteota Candidatus Martarchaeota archaeon MCL5419344.1
Archaea Thermoproteati Thermoproteota Metallosphaera yellowstonensis WP 009075009.1
Archaea Thermoproteati Thermoproteota Metallosphaera tengchongensis WP 174629315.1
Archaea Thermoproteati Thermoproteota Metallosphaera cuprina WP 013737049.1
Archaea Thermoproteati Thermoproteota Metallosphaera WP 240254633.1
Archaea Thermoproteati Thermoproteota Metallosphaera hakonensis WP 110369432.1
Archaea Thermoproteati Thermoproteota Sulfolobales archaeon HS-7 BCU68545.1
Archaea Thermoproteati Thermoproteota Sulfurisphaera ohwakuensis WP 156014433.1
Archaea Thermoproteati Thermoproteota Sulfurisphaera javensis WP 369609683.1
Archaea Thermoproteati Thermoproteota Sulfolobus sp. MFP3064719.1
Archaea Thermoproteati Thermoproteota Sulfolobus acidocaldarius WP 011278126.1
Archaea Thermoproteati Thermoproteota Stygiolobus caldivivus WP 221286732.1
Archaea Thermoproteati Thermoproteota Stygiolobus sp. RP850M WP 337708115.1
Archaea Thermoproteati Thermoproteota Sulfodiicoccus acidiphilus WP 126449870.1
Archaea Thermoproteati Thermoproteota Candidatus Aramenus sp. CH1 MCI2414831.1
Archaea Thermoproteati Thermoproteota Acidianus brierleyi WP 110270961.1
Archaea Thermoproteati Thermoproteota Acidianus WP 048099458.1
Archaea Thermoproteati Thermoproteota Acidianus ambivalens WP 152940822.1
Archaea Thermoproteati Thermoproteota Acidianus sulfidivorans WP 110380561.1
Archaea Thermoproteati Thermoproteota Acidianus manzaensis WP 148692197.1
Archaea Thermoproteati Thermoproteota Sulfuracidifex tepidarius WP 149528387.1
Archaea Thermoproteati Thermoproteota Sulfuracidifex metallicus WP 156017698.1
Archaea Thermoproteati Thermoproteota Saccharolobus sp. A20 WP 069281597.1
Archaea Thermoproteati Thermoproteota Sulfolobaceae WP 153423094.1
Archaea Thermoproteati Thermoproteota Sulfolobus tengchongensis WP 338601876.1
Archaea Thermoproteati Thermoproteota Saccharolobus sp. WP 314124160.1
Archaea Thermoproteati Thermoproteota Saccharolobus sp. MEM3802202.1
Archaea Thermoproteati Thermoproteota Saccharolobus sp. MEM3280774.1
Archaea Thermoproteati Thermoproteota Pyrodictiaceae archaeon MET1102316.1
Archaea Thermoproteati Thermoproteota Acidilobales archaeon HDD26091.1
Archaea Thermoproteati Thermoproteota Pyrolobus fumarii WP 014026251.1
Archaea Thermoproteati Thermoproteota Pyrofollis japonicus WP 317895608.1
Archaea Thermoproteati Thermoproteota Hyperthermus sp. RUM46794.1
Archaea Thermoproteati Thermoproteota Thermoproteota archaeon MET1128708.1
Archaea Thermoproteati Thermoproteota Hyperthermus butylicus WP 228546760.1
Archaea Thermoproteati Thermoproteota Pyrodictium occultum WP 058370223.1
Archaea Thermoproteati Thermoproteota Pyrodictium delaneyi WP 055410594.1
Archaea Thermoproteati Thermoproteota Pyrodictium abyssi WP 338250733.1
Archaea Thermoproteati Thermoproteota Sulfolobales archaeon MEL9997594.1
Archaea Thermoproteati Thermoproteota Sulfolobales archaeon MEM0504290.1
Archaea Thermoproteati Thermoproteota Sulfolobales archaeon MEM0453464.1
Archaea Thermoproteati Thermoproteota Sulfolobales archaeon MCS7108132.1
Archaea Thermoproteati Thermoproteota Desulfurococcales archaeon ex4484 204 OYT52063.1
Archaea Thermoproteati Thermoproteota Desulfurococcales archaeon MCD6084421.1
Archaea Thermoproteati Thermoproteota Thermoprotei archaeon RLG78352.1
Archaea Thermoproteati Thermoproteota Desulfurococcales archaeon MCD6428387.1
Archaea Thermoproteati Thermoproteota Desulfurococcales archaeon ex4484 42 OYT48512.1
Archaea Thermoproteati Thermoproteota Desulfurococcaceae archaeon MCC6014394.1
Archaea Thermoproteati Thermoproteota Desulfurococcaceae archaeon MCC6047786.1
Archaea Thermoproteati Thermoproteota Sulfolobales archaeon MCX8184795.1
Archaea Thermoproteati Thermoproteota Thermofilaceae archaeon MCX8181424.1
Archaea Thermoproteati Thermoproteota Sulfolobales archaeon MCX8208241.1
Archaea Thermoproteati Thermoproteota Sulfolobales archaeon MEM2006227.1
Archaea Thermoproteati Thermoproteota Sulfolobales archaeon MEM1881968.1
Archaea Thermoproteati Thermoproteota Sulfolobales archaeon MEM1623343.1
Archaea Thermoproteati Thermoproteota Thermoprotei archaeon RLG78060.1
Archaea Thermoproteati Thermoproteota Zestosphaera tikiterensis PUA33311.1
Archaea Thermoproteati Thermoproteota Zestosphaera sp. MEM2021343.1
Archaea Thermoproteati Thermoproteota Zestosphaera sp. MEM4486873.1
Archaea Thermoproteati Thermoproteota Desulfurococcaceae archaeon MCC6032547.1
Archaea Thermoproteati Thermoproteota Thermoprotei archaeon MGC8975091.1
Archaea Thermoproteati Thermoproteota Zestosphaera sp. MEM0025004.1
Archaea Thermoproteati Thermoproteota Zestosphaera sp. MFN3267831.1
Archaea Thermoproteati Thermoproteota Candidatus Zestomicrobium tamdykulense MEO3992828.1
Archaea Thermoproteati Thermoproteota Thermoprotei archaeon RLG88456.1
Archaea Thermoproteati Thermoproteota Desulfurococcales archaeon MCD6488706.1
Archaea Thermoproteati Thermoproteota Desulfurococcaceae archaeon MEX13E-LK6-19 WFO76096.1
Archaea Thermoproteati Thermoproteota Sulfolobales archaeon MCS7128491.1
Archaea Thermoproteati Thermoproteota Desulfurococcaceae archaeon MEM2578986.1
Archaea Thermoproteati Thermoproteota Desulfurococcaceae archaeon MEZ0394334.1
Archaea Thermoproteati Thermoproteota Thermoproteota archaeon NPA99700.1
Archaea Thermoproteati Thermoproteota Thermogladius sp. 4427co WP 440059880.1
Archaea Thermoproteati Thermoproteota Thermogladius calderae WP 274379106.1
Archaea Thermoproteati Thermoproteota Thermogladius sp. NAZ27041.1
Archaea Thermoproteati Thermoproteota Staphylothermus sp. MCD6300982.1
Archaea Thermoproteati Thermoproteota Staphylothermus marinus WP 244372362.1
Archaea Thermoproteati Thermoproteota Staphylothermus hellenicus WP 013143514.1
Archaea Thermoproteati Thermoproteota Thermoprotei archaeon RLG80754.1
Archaea Thermoproteati Thermoproteota Thermoprotei archaeon MET1160167.1
Archaea Thermoproteati Thermoproteota Desulfurococcaceae archaeon MEM0380414.1
Archaea Thermoproteati Thermoproteota Desulfurococcaceae archaeon MEM4695834.1
Archaea Thermoproteati Thermoproteota Desulfurococcaceae archaeon MEM1555399.1
Archaea Thermoproteati Thermoproteota Thermosphaera sp. MEM4914470.1
Archaea Thermoproteati Thermoproteota Thermosphaera aggregans WP 013129012.1
Archaea Thermoproteati Thermoproteota Thermosphaera chiliense WP 193436119.1
Archaea Thermoproteati Thermoproteota Thermosphaera sp. WP 448577813.1
Archaea Thermoproteati Thermoproteota Desulfurococcaceae archaeon MEM1863675.1
Archaea Thermoproteati Thermoproteota Desulfurococcus amylolyticus WP 048058807.1
Archaea Thermoproteati Thermoproteota Desulfurococcus mucosus WP 013562120.1
Archaea Thermoproteati Thermoproteota Desulfurococcus sp. MCY0867769.1
Archaea Thermoproteati Thermoproteota Desulfurococcaceae archaeon MCC6035220.1
Archaea Thermoproteati Thermoproteota Desulfurococcaceae archaeon MCC6011352.1
Archaea Thermoproteati Thermoproteota Desulfurococcaceae archaeon MCC6040976.1
Archaea Thermoproteati Thermoproteota Desulfurococcaceae archaeon MGC8983159.1
Archaea Thermoproteati Thermoproteota Desulfurococcaceae archaeon MEM4062242.1
Archaea Thermoproteati Thermoproteota Desulfurococcaceae archaeon MCC6041891.1
Archaea Thermoproteati Thermoproteota Sulfophobococcus zilligii BGR77094.1
Archaea Thermoproteati Thermoproteota Desulfurococcaceae archaeon MEM4717688.1
Archaea Thermoproteati Thermoproteota Desulfurococcaceae archaeon MEM0030936.1
Archaea Thermoproteati Thermoproteota Desulfurococcaceae archaeon MEM0060156.1
Archaea Thermoproteati Thermoproteota Desulfurococcaceae archaeon MEM0045495.1
Archaea Thermoproteati Thermoproteota Desulfurococcaceae archaeon MEM4487833.1
Archaea Thermoproteati Thermoproteota Candidatus Marsarchaeota G1 archaeon OSP C PSN89295.1
Archaea Thermoproteati Thermoproteota Thermoprotei archaeon MEM3670856.1
Archaea Thermoproteati Thermoproteota Candidatus Marsarchaeota G2 archaeon ECH B SAG-M15 PSN90035.1
Archaea Thermoproteati Thermoproteota Thermoprotei archaeon MEM3794402.1
Archaea Thermoproteati Thermoproteota Thermoprotei archaeon MEM0120074.1
Archaea Thermoproteati Thermoproteota Sulfolobales archaeon MEM2204364.1
Archaea Thermoproteati Thermoproteota Desulfurococcales archaeon MDT7887478.1
Archaea Thermoproteati Thermoproteota Sulfolobales archaeon MEM4969976.1
Archaea Thermoproteati Thermoproteota Desulfurococcales archaeon MDT7895061.1
Archaea Thermoproteati Thermoproteota Sulfolobales archaeon MEL9929905.1
Archaea Thermoproteati Thermoproteota Sulfolobales archaeon MEZ0289937.1
Archaea Thermoproteati Thermoproteota Desulfurococcales archaeon MCC6062194.1
Archaea Thermoproteati Thermoproteota Sulfolobales archaeon MGC9010125.1
Archaea Thermoproteati Thermoproteota Sulfolobales archaeon MGC9149183.1
Archaea Thermoproteati Thermoproteota Thermocladium modestius WP 188596806.1
Archaea Thermoproteati Thermoproteota Thermocladium sp. ECH B KUO92947.1
Archaea Thermoproteati Thermoproteota Caldivirga sp. MU80 WP 066792647.1
Archaea Thermoproteati Thermoproteota Caldivirga sp. UBA161 WP 291765783.1
Archaea Thermoproteati Thermoproteota Caldivirga sp. WP 291999767.1
Archaea Thermoproteati Thermoproteota Thermoprotei archaeon ex4572 64 OYT25380.1
Archaea Thermoproteati Thermoproteota Thermoproteota archaeon NPA23584.1
Archaea Thermoproteati Thermoproteota Thermoproteota archaeon NPB00538.1
Archaea Thermoproteati Thermoproteota Thermoproteota archaeon NPA70986.1
Archaea Thermoproteati Thermoproteota Vulcanisaeta thermophila WP 069808281.1
Archaea Thermoproteati Thermoproteota Vulcanisaeta sp. AZ3 MFB6470858.1
Archaea Thermoproteati Thermoproteota Vulcanisaeta sp. MGC8542937.1
Archaea Thermoproteati Thermoproteota Vulcanisaeta sp. EB80 WP 081226558.1
Archaea Thermoproteati Thermoproteota Vulcanisaeta sp. MCG2863058.1
Archaea Thermoproteati Thermoproteota Vulcanisaeta souniana WP 188602293.1
Archaea Thermoproteati Thermoproteota Vulcanisaeta moutnovskia WP 013605114.1
Archaea Thermoproteati Thermoproteota Vulcanisaeta distributa WP 013336016.1
Archaea Thermoproteati Thermoproteota Vulcanisaeta sp. JCM 16161 WP 446752496.1
Archaea Thermoproteati Thermoproteota Vulcanisaeta sp. JCM 16159 WP 054858023.1
Archaea Thermoproteati Thermoproteota Vulcanisaeta sp. JCM 14467 WP 054848889.1
Archaea Thermoproteati Thermoproteota Thermoproteus sp. AZ2 MFB6489757.1
Archaea Thermoproteati Thermoproteota Thermoproteus tenax WP 014126905.1
Archaea Thermoproteati Thermoproteota Thermoproteus sp. MEZ0248923.1
Archaea Thermoproteati Thermoproteota Thermoproteus uzoniensis WP 013679499.1
Archaea Thermoproteati Thermoproteota Thermoproteus thermophilus BGT37391.1
Archaea Thermoproteati Thermoproteota Thermoproteus sp. CP80 WP 081245660.1
Archaea Thermoproteati Thermoproteota Pyrobaculum islandicum WP 011762135.1
Archaea Thermoproteati Thermoproteota Pyrobaculum neutrophilum WP 012351084.1
Archaea Thermoproteati Thermoproteota Thermoproteaceae archaeon MCC6020907.1
Archaea Thermoproteati Thermoproteota Pyrobaculum calidifontis WP 193322546.1
Archaea Thermoproteati Thermoproteota Pyrobaculum sp. WP30 AKT35482.1
Archaea Thermoproteati Thermoproteota Pyrobaculum sp. MCC6035916.1

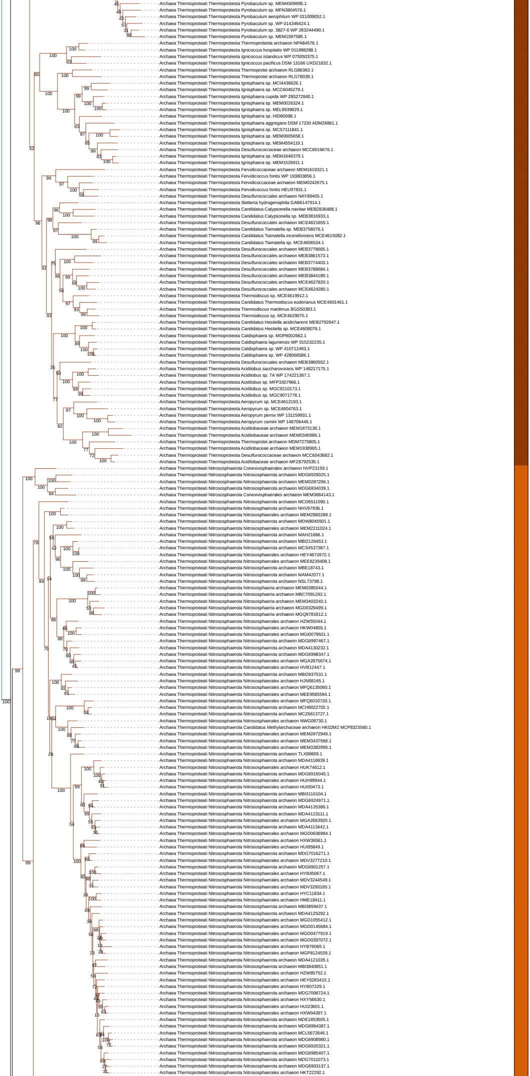
Archaea Thermoproteati Thermoproteota Pyrobaculum sp. MEM4509695.1
Archaea Thermoproteati Thermoproteota Pyrobaculum sp. MFN3804576.1
Archaea Thermoproteati Thermoproteota Pyrobaculum aerophilum WP 011009052.1
Archaea Thermoproteati Thermoproteota Pyrobaculum sp. WP 014346424.1
Archaea Thermoproteati Thermoproteota Pyrobaculum sp. 3827-6 WP 263244490.1
Archaea Thermoproteati Thermoproteota Pyrobaculum sp. MEM1597585.1
Archaea Thermoproteati Thermoproteota Thermoproteota archaeon NPA84576.1
Archaea Thermoproteati Thermoproteota Ignicoccus hospitalis WP 011998288.1
Archaea Thermoproteati Thermoproteota Ignicoccus islandicus WP 075050375.1
Archaea Thermoproteati Thermoproteota Ignicoccus pacificus DSM 13166 UXD21832.1
Archaea Thermoproteati Thermoproteota Thermoprotei archaeon RLG86363.1
Archaea Thermoproteati Thermoproteota Thermoprotei archaeon RLG78039.1
Archaea Thermoproteati Thermoproteota Ignisphaera sp. MCI4436626.1
Archaea Thermoproteati Thermoproteota Ignisphaera sp. MCC6045279.1
Archaea Thermoproteati Thermoproteota Ignisphaera cupida WP 285272840.1
Archaea Thermoproteati Thermoproteota Ignisphaera sp. MEM0026324.1
Archaea Thermoproteati Thermoproteota Ignisphaera sp. MEL9939829.1
Archaea Thermoproteati Thermoproteota Ignisphaera sp. HID80098.1
Archaea Thermoproteati Thermoproteota Ignisphaera aggregans DSM 17230 ADM26861.1
Archaea Thermoproteati Thermoproteota Ignisphaera sp. MCS7111841.1
Archaea Thermoproteati Thermoproteota Ignisphaera sp. MEM0005658.1
Archaea Thermoproteati Thermoproteota Ignisphaera sp. MEM4554119.1
Archaea Thermoproteati Thermoproteota Desulfurococcaceae archaeon MCC6016676.1
Archaea Thermoproteati Thermoproteota Ignisphaera sp. MEM1646379.1
Archaea Thermoproteati Thermoproteota Ignisphaera sp. MEM1526911.1
Archaea Thermoproteati Thermoproteota Fervidicoccaceae archaeon MEM1619321.1
Archaea Thermoproteati Thermoproteota Fervidicoccus fontis WP 193803856.1
Archaea Thermoproteati Thermoproteota Fervidicoccaceae archaeon MEM0242675.1
Archaea Thermoproteati Thermoproteota Fervidicoccus fontis HEU97831.1
Archaea Thermoproteati Thermoproteota Desulfurococcales archaeon NAY89405.1
Archaea Thermoproteati Thermoproteota Stetteria hydrogenophila GAB6147914.1
Archaea Thermoproteati Thermoproteota Candidatus Calypsonella navitae MEB2836488.1
Archaea Thermoproteati Thermoproteota Candidatus Calypsonella sp. MEB3816933.1
Archaea Thermoproteati Thermoproteota Desulfurococcales archaeon MCE4621655.1
Archaea Thermoproteati Thermoproteota Candidatus Tiamatella sp. MEB3758076.1
Archaea Thermoproteati Thermoproteota Candidatus Tiamatella incendiivivens MCE4615082.1
Archaea Thermoproteati Thermoproteota Candidatus Tiamatella sp. MCE4606534.1
Archaea Thermoproteati Thermoproteota Desulfurococcales archaeon MEB3778665.1
Archaea Thermoproteati Thermoproteota Desulfurococcales archaeon MEB3861573.1
Archaea Thermoproteati Thermoproteota Desulfurococcales archaeon MEB3774403.1
Archaea Thermoproteati Thermoproteota Desulfurococcales archaeon MEB3788684.1
Archaea Thermoproteati Thermoproteota Desulfurococcales archaeon MEB3844189.1
Archaea Thermoproteati Thermoproteota Desulfurococcales archaeon MCE4627820.1
Archaea Thermoproteati Thermoproteota Desulfurococcales archaeon MCE4624280.1
Archaea Thermoproteati Thermoproteota Thermodiscus sp. MCE4619912.1
Archaea Thermoproteati Thermoproteota Candidatus Thermodiscus eudorianus MCE4601461.1
Archaea Thermoproteati Thermoproteota Thermodiscus maritimus BGS50383.1
Archaea Thermoproteati Thermoproteota Thermodiscus sp. MCE4629075.1
Archaea Thermoproteati Thermoproteota Candidatus Hestiella acidicharens MEB2792647.1
Archaea Thermoproteati Thermoproteota Candidatus Hestiella sp. MCE4608078.1
Archaea Thermoproteati Thermoproteota Caldisphaera sp. MDP8002662.1
Archaea Thermoproteati Thermoproteota Caldisphaera lagunensis WP 015232235.1
Archaea Thermoproteati Thermoproteota Caldisphaera sp. WP 410712493.1
Archaea Thermoproteati Thermoproteota Caldisphaera sp. WP 428068586.1
Archaea Thermoproteati Thermoproteota Desulfurococcales archaeon MEB3860552.1
Archaea Thermoproteati Thermoproteota Acidilobus saccharovorans WP 148217175.1
Archaea Thermoproteati Thermoproteota Acidilobus sp. 7A WP 174221367.1
Archaea Thermoproteati Thermoproteota Acidilobus sp. MFP3307966.1
Archaea Thermoproteati Thermoproteota Acidilobus sp. MGC9210173.1
Archaea Thermoproteati Thermoproteota Acidilobus sp. MGC9071778.1
Archaea Thermoproteati Thermoproteota Aeropyrum sp. MCE4612193.1
Archaea Thermoproteati Thermoproteota Aeropyrum sp. MCE4604763.1
Archaea Thermoproteati Thermoproteota Aeropyrum pernix WP 131159931.1
Archaea Thermoproteati Thermoproteota Aeropyrum camini WP 148706446.1
Archaea Thermoproteati Thermoproteota Acidilobaceae archaeon MEM1873136.1
Archaea Thermoproteati Thermoproteota Acidilobaceae archaeon MEM0340986.1
Archaea Thermoproteati Thermoproteota Thermoprotei archaeon MDM7275805.1
Archaea Thermoproteati Thermoproteota Acidilobaceae archaeon MEM1938965.1
Archaea Thermoproteati Thermoproteota Desulfurococcaceae archaeon MCC6043682.1
Archaea Thermoproteati Thermoproteota Acidilobaceae archaeon MFZ8792535.1
Archaea Thermoproteati Nitrososphaerota Conexivisphaerales archaeon HVP23159.1
Archaea Thermoproteati Nitrososphaerota Nitrososphaerota archaeon MDG6929025.1
Archaea Thermoproteati Nitrososphaerota Nitrososphaerota archaeon MEM0287296.1
Archaea Thermoproteati Nitrososphaerota Nitrososphaerota archaeon MDG6934039.1
Archaea Thermoproteati Nitrososphaerota Conexivisphaerales archaeon MEM3684143.1
Archaea Thermoproteati Nitrososphaerota Nitrososphaerota archaeon MCD6511090.1
Archaea Thermoproteati Nitrososphaerota Nitrososphaerota archaeon NHV97936.1
Archaea Thermoproteati Nitrososphaerota Nitrososphaerales archaeon MEM2883289.1
Archaea Thermoproteati Nitrososphaerota Nitrososphaerota archaeon MDW8045501.1
Archaea Thermoproteati Nitrososphaerota Nitrososphaerales archaeon MEM2211024.1
Archaea Thermoproteati Nitrososphaerota Nitrososphaerota archaeon MAH21866.1
Archaea Thermoproteati Nitrososphaerota Nitrososphaerota archaeon MBI2126453.1
Archaea Thermoproteati Nitrososphaerota Nitrososphaerota archaeon MCS4537387.1
Archaea Thermoproteati Nitrososphaerota Nitrososphaerales archaeon HEY4672672.1
Archaea Thermoproteati Nitrososphaerota Nitrososphaerales archaeon MEE8239408.1
Archaea Thermoproteati Nitrososphaerota Nitrososphaerota archaeon MBE18743.1
Archaea Thermoproteati Nitrososphaerota Nitrososphaerota archaeon MAM42077.1
Archaea Thermoproteati Nitrososphaerota Nitrososphaerota archaeon NSL73798.1
Archaea Thermoproteati Nitrososphaerota Nitrososphaeria archaeon MEM0385544.1
Archaea Thermoproteati Nitrososphaerota Nitrososphaeria archaeon MBC7091292.1
Archaea Thermoproteati Nitrososphaerota Nitrososphaeria archaeon MEM3403240.1
Archaea Thermoproteati Nitrososphaerota Nitrososphaeria archaeon MGD0329499.1
Archaea Thermoproteati Nitrososphaerota Nitrososphaeria archaeon MGQ9781812.1
Archaea Thermoproteati Nitrososphaerota Nitrososphaerales archaeon HZW55044.1
Archaea Thermoproteati Nitrososphaerota Nitrososphaerales archaeon HKW04855.1
Archaea Thermoproteati Nitrososphaerota Nitrososphaerales archaeon MGI0079501.1
Archaea Thermoproteati Nitrososphaerota Nitrososphaerota archaeon MDG6997467.1
Archaea Thermoproteati Nitrososphaerota Nitrososphaerota archaeon MDA4130232.1
Archaea Thermoproteati Nitrososphaerota Nitrososphaerota archaeon MDG6998347.1
Archaea Thermoproteati Nitrososphaerota Nitrososphaerales archaeon MGA2875674.1
Archaea Thermoproteati Nitrososphaerota Nitrososphaerales archaeon HVB12447.1
Archaea Thermoproteati Nitrososphaerota Nitrososphaerota archaeon MBI2937531.1
Archaea Thermoproteati Nitrososphaerota Nitrososphaerales archaeon HJN58165.1
Archaea Thermoproteati Nitrososphaerota Nitrososphaerales archaeon MFQ6135060.1
Archaea Thermoproteati Nitrososphaerota Nitrososphaerales archaeon MEE9585594.1
Archaea Thermoproteati Nitrososphaerota Nitrososphaerales archaeon MFQ6010720.1
Archaea Thermoproteati Nitrososphaerota Nitrososphaerota archaeon MCH8022702.1
Archaea Thermoproteati Nitrososphaerota Nitrososphaerota archaeon MCZ6613727.1
Archaea Thermoproteati Nitrososphaerota Nitrososphaerales archaeon NWG08730.1
Archaea Thermoproteati Nitrososphaerota Candidatus Methylarchaceae archaeon HK02M2 MCP8323560.1
Archaea Thermoproteati Nitrososphaerota Nitrososphaerales archaeon MEM2872949.1
Archaea Thermoproteati Nitrososphaerota Nitrososphaerales archaeon MEM3437566.1
Archaea Thermoproteati Nitrososphaerota Nitrososphaerales archaeon MEM3382995.1
Archaea Thermoproteati Nitrososphaerota Nitrososphaerota archaeon TLX98809.1
Archaea Thermoproteati Nitrososphaerota Nitrososphaerota archaeon MDA4116939.1
Archaea Thermoproteati Nitrososphaerota Nitrososphaerales archaeon HUK74612.1
Archaea Thermoproteati Nitrososphaerota Nitrososphaerota archaeon MDG6915045.1
Archaea Thermoproteati Nitrososphaerota Nitrososphaerales archaeon HUH99944.1
Archaea Thermoproteati Nitrososphaerota Nitrososphaerales archaeon HUI00473.1
Archaea Thermoproteati Nitrososphaerota Nitrososphaerota archaeon MBI3116104.1
Archaea Thermoproteati Nitrososphaerota Nitrososphaerota archaeon MDG6924971.1
Archaea Thermoproteati Nitrososphaerota Nitrososphaerota archaeon MDA4135386.1
Archaea Thermoproteati Nitrososphaerota Nitrososphaerota archaeon MDA4123111.1
Archaea Thermoproteati Nitrososphaerota Nitrososphaerales archaeon MGA2663920.1
Archaea Thermoproteati Nitrososphaerota Nitrososphaerota archaeon MDA4113442.1
Archaea Thermoproteati Nitrososphaerota Nitrososphaerales archaeon MGD0636984.1
Archaea Thermoproteati Nitrososphaerota Nitrososphaerales archaeon HXW36561.1
Archaea Thermoproteati Nitrososphaerota Nitrososphaerales archaeon HUI85849.1
Archaea Thermoproteati Nitrososphaerota Nitrososphaerota archaeon MDG7016271.1
Archaea Thermoproteati Nitrososphaerota Nitrososphaerales archaeon MDV3277210.1
Archaea Thermoproteati Nitrososphaerota Nitrososphaerota archaeon MDG6901257.1
Archaea Thermoproteati Nitrososphaerota Nitrososphaerales archaeon HYB45067.1
Archaea Thermoproteati Nitrososphaerota Nitrososphaerales archaeon MDV3244549.1
Archaea Thermoproteati Nitrososphaerota Nitrososphaerales archaeon MDV3293165.1
Archaea Thermoproteati Nitrososphaerota Nitrososphaerales archaeon HYC11834.1
Archaea Thermoproteati Nitrososphaerota Nitrososphaerales archaeon HME18411.1
Archaea Thermoproteati Nitrososphaerota Nitrososphaerota archaeon MBI3859437.1
Archaea Thermoproteati Nitrososphaerota Nitrososphaerota archaeon MDA4125292.1
Archaea Thermoproteati Nitrososphaerota Nitrososphaerales archaeon MGD1055412.1
Archaea Thermoproteati Nitrososphaerota Nitrososphaerales archaeon MGD0145684.1
Archaea Thermoproteati Nitrososphaerota Nitrososphaerales archaeon MGD0477919.1
Archaea Thermoproteati Nitrososphaerota Nitrososphaerales archaeon MGD0397072.1
Archaea Thermoproteati Nitrososphaerota Nitrososphaerales archaeon HYB76065.1
Archaea Thermoproteati Nitrososphaerota Nitrososphaerales archaeon MGP8124539.1
Archaea Thermoproteati Nitrososphaerota Nitrososphaerota archaeon MDA4121035.1
Archaea Thermoproteati Nitrososphaerota Nitrososphaerota archaeon MBI3840851.1
Archaea Thermoproteati Nitrososphaerota Nitrososphaerales archaeon HZW85752.1
Archaea Thermoproteati Nitrososphaerota Nitrososphaerales archaeon HEY6283415.1
Archaea Thermoproteati Nitrososphaerota Nitrososphaerales archaeon HYB07229.1
Archaea Thermoproteati Nitrososphaerota Nitrososphaerota archaeon MDG7006724.1
Archaea Thermoproteati Nitrososphaerota Nitrososphaerales archaeon HXY56630.1
Archaea Thermoproteati Nitrososphaerota Nitrososphaerales archaeon HUI23601.1
Archaea Thermoproteati Nitrososphaerota Nitrososphaerales archaeon HXW94397.1
Archaea Thermoproteati Nitrososphaerota Nitrososphaerota archaeon MDE1853505.1
Archaea Thermoproteati Nitrososphaerota Nitrososphaerota archaeon MDG6964387.1
Archaea Thermoproteati Nitrososphaerota Nitrososphaerota archaeon MCL5672846.1
Archaea Thermoproteati Nitrososphaerota Nitrososphaerota archaeon MDG6908980.1
Archaea Thermoproteati Nitrososphaerota Nitrososphaerota archaeon MDG6920321.1
Archaea Thermoproteati Nitrososphaerota Nitrososphaerota archaeon MDG6985407.1
Archaea Thermoproteati Nitrososphaerota Nitrososphaerota archaeon MDG7011073.1
Archaea Thermoproteati Nitrososphaerota Nitrososphaerota archaeon MDG6903137.1
Archaea Thermoproteati Nitrososphaerota Nitrososphaerales archaeon HKT22292.1

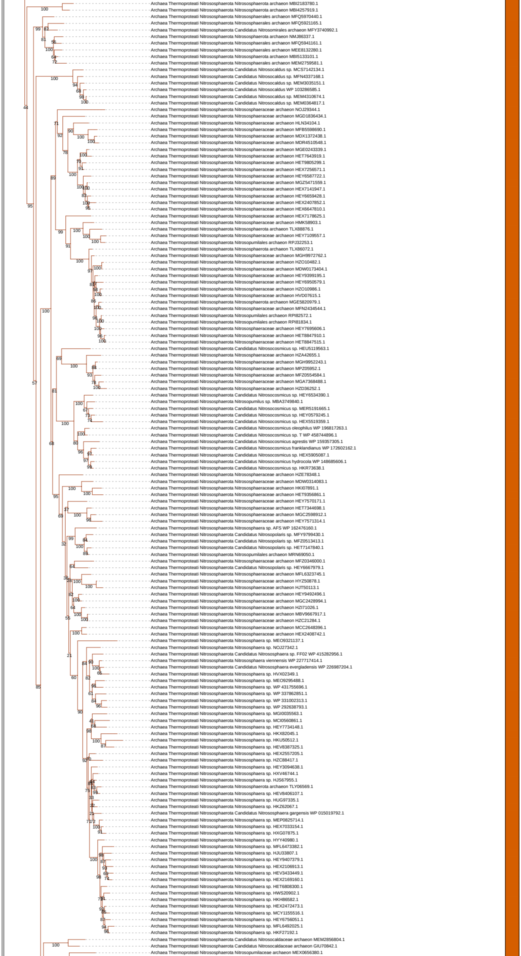
Archaea Thermoproteati Nitrososphaerota Nitrososphaerota archaeon MBI2183780.1
Archaea Thermoproteati Nitrososphaerota Nitrososphaerota archaeon MBI4257919.1
Archaea Thermoproteati Nitrososphaerota Nitrososphaerales archaeon MFQ5970440.1
Archaea Thermoproteati Nitrososphaerota Nitrososphaerales archaeon MFQ5921165.1
Archaea Thermoproteati Nitrososphaerota Candidatus Nitrosomirales archaeon MFY3740992.1
Archaea Thermoproteati Nitrososphaerota Nitrososphaerota archaeon NMJ86337.1
Archaea Thermoproteati Nitrososphaerota Nitrososphaerales archaeon MFQ5941161.1
Archaea Thermoproteati Nitrososphaerota Nitrososphaerales archaeon MEE8132280.1
Archaea Thermoproteati Nitrososphaerota Nitrososphaerota archaeon MBI5133101.1
Archaea Thermoproteati Nitrososphaerota Nitrososphaerales archaeon MEM2759581.1
Archaea Thermoproteati Nitrososphaerota Candidatus Nitrosocaldus sp. MCS7142134.1
Archaea Thermoproteati Nitrososphaerota Candidatus Nitrosocaldus sp. MFN4337168.1
Archaea Thermoproteati Nitrososphaerota Candidatus Nitrosocaldus sp. MEM3035151.1
Archaea Thermoproteati Nitrososphaerota Candidatus Nitrosocaldus WP 103286585.1
Archaea Thermoproteati Nitrososphaerota Candidatus Nitrosocaldus sp. MEM4310674.1
Archaea Thermoproteati Nitrososphaerota Candidatus Nitrosocaldus sp. MEM0364817.1
Archaea Thermoproteati Nitrososphaerota Nitrososphaeraceae archaeon NOJ29344.1
Archaea Thermoproteati Nitrososphaerota Nitrososphaeraceae archaeon MGD1836434.1
Archaea Thermoproteati Nitrososphaerota Nitrososphaeraceae archaeon HLN34104.1
Archaea Thermoproteati Nitrososphaerota Nitrososphaeraceae archaeon MFB5598690.1
Archaea Thermoproteati Nitrososphaerota Nitrososphaeraceae archaeon MDX1372438.1
Archaea Thermoproteati Nitrososphaerota Nitrososphaeraceae archaeon MDR4510548.1
Archaea Thermoproteati Nitrososphaerota Nitrososphaeraceae archaeon MGE0243339.1
Archaea Thermoproteati Nitrososphaerota Nitrososphaeraceae archaeon HET7643919.1
Archaea Thermoproteati Nitrososphaerota Nitrososphaeraceae archaeon HET9805299.1
Archaea Thermoproteati Nitrososphaerota Nitrososphaeraceae archaeon HEX7256571.1
Archaea Thermoproteati Nitrososphaerota Nitrososphaeraceae archaeon HEY6587722.1
Archaea Thermoproteati Nitrososphaerota Nitrososphaeraceae archaeon MGZ5471559.1
Archaea Thermoproteati Nitrososphaerota Nitrososphaeraceae archaeon HEX7141947.1
Archaea Thermoproteati Nitrososphaerota Nitrososphaeraceae archaeon HEY6659428.1
Archaea Thermoproteati Nitrososphaerota Nitrososphaeraceae archaeon HEX2407852.1
Archaea Thermoproteati Nitrososphaerota Nitrososphaeraceae archaeon HEX6647810.1
Archaea Thermoproteati Nitrososphaerota Nitrososphaeraceae archaeon HEX7178625.1
Archaea Thermoproteati Nitrososphaerota Nitrososphaeraceae archaeon HMK58903.1
Archaea Thermoproteati Nitrososphaerota Nitrososphaerota archaeon TLX88876.1
Archaea Thermoproteati Nitrososphaerota Nitrososphaeraceae archaeon HEY7109557.1
Archaea Thermoproteati Nitrososphaerota Nitrosopumilales archaeon RPJ32253.1
Archaea Thermoproteati Nitrososphaerota Nitrososphaerota archaeon TLX86072.1
Archaea Thermoproteati Nitrososphaerota Nitrososphaeraceae archaeon MGH9972762.1
Archaea Thermoproteati Nitrososphaerota Nitrososphaeraceae archaeon HZO10482.1
Archaea Thermoproteati Nitrososphaerota Nitrososphaeraceae archaeon MDW0173404.1
Archaea Thermoproteati Nitrososphaerota Nitrososphaeraceae archaeon HEY9399195.1
Archaea Thermoproteati Nitrososphaerota Nitrososphaeraceae archaeon HEY6950579.1
Archaea Thermoproteati Nitrososphaerota Nitrososphaeraceae archaeon HZO10986.1
Archaea Thermoproteati Nitrososphaerota Nitrososphaeraceae archaeon HVD07615.1
Archaea Thermoproteati Nitrososphaerota Nitrososphaerota archaeon MGE5820979.1
Archaea Thermoproteati Nitrososphaerota Nitrososphaeraceae archaeon MFN2434544.1
Archaea Thermoproteati Nitrososphaerota Nitrosopumilales archaeon RPI82572.1
Archaea Thermoproteati Nitrososphaerota Nitrosopumilales archaeon RPI81834.1
Archaea Thermoproteati Nitrososphaerota Nitrososphaeraceae archaeon HEY7695606.1
Archaea Thermoproteati Nitrososphaerota Nitrososphaeraceae archaeon HET8847910.1
Archaea Thermoproteati Nitrososphaerota Nitrososphaeraceae archaeon HET8847515.1
Archaea Thermoproteati Nitrososphaerota Candidatus Nitrosocosmicus sp. HEU5119563.1
Archaea Thermoproteati Nitrososphaerota Nitrososphaeraceae archaeon HZA42655.1
Archaea Thermoproteati Nitrososphaerota Nitrososphaeraceae archaeon MGH9952243.1
Archaea Thermoproteati Nitrososphaerota Nitrososphaeraceae archaeon MPZ05952.1
Archaea Thermoproteati Nitrososphaerota Nitrososphaeraceae archaeon MFZ0554584.1
Archaea Thermoproteati Nitrososphaerota Nitrososphaeraceae archaeon MGA7368488.1
Archaea Thermoproteati Nitrososphaerota Nitrososphaeraceae archaeon HZD36252.1
Archaea Thermoproteati Nitrososphaerota Candidatus Nitrosocosmicus sp. HEY6534390.1
Archaea Thermoproteati Nitrososphaerota Nitrosopumilus sp. MBA3749840.1
Archaea Thermoproteati Nitrososphaerota Candidatus Nitrosocosmicus sp. MER5191665.1
Archaea Thermoproteati Nitrososphaerota Candidatus Nitrosocosmicus sp. HEY0579245.1
Archaea Thermoproteati Nitrososphaerota Candidatus Nitrosocosmicus sp. HEX5519359.1
Archaea Thermoproteati Nitrososphaerota Candidatus Nitrosocosmicus oleophilus WP 196817263.1
Archaea Thermoproteati Nitrososphaerota Candidatus Nitrosocosmicus sp. T WP 458744896.1
Archaea Thermoproteati Nitrososphaerota Candidatus Nitrosocosmicus agrestis WP 159357305.1
Archaea Thermoproteati Nitrososphaerota Candidatus Nitrosocosmicus franklandianus WP 172602162.1
Archaea Thermoproteati Nitrososphaerota Candidatus Nitrosocosmicus sp. HEX5905087.1
Archaea Thermoproteati Nitrososphaerota Candidatus Nitrosocosmicus hydrocola WP 148685606.1
Archaea Thermoproteati Nitrososphaerota Candidatus Nitrosocosmicus sp. HKR73638.1
Archaea Thermoproteati Nitrososphaerota Nitrososphaeraceae archaeon HZE78348.1
Archaea Thermoproteati Nitrososphaerota Nitrososphaeraceae archaeon MDW0314083.1
Archaea Thermoproteati Nitrososphaerota Nitrososphaeraceae archaeon HKI07891.1
Archaea Thermoproteati Nitrososphaerota Nitrososphaeraceae archaeon HET9356861.1
Archaea Thermoproteati Nitrososphaerota Nitrososphaeraceae archaeon HEY7570171.1
Archaea Thermoproteati Nitrososphaerota Nitrososphaeraceae archaeon HET7344698.1
Archaea Thermoproteati Nitrososphaerota Nitrososphaeraceae archaeon MGC2598912.1
Archaea Thermoproteati Nitrososphaerota Nitrososphaeraceae archaeon HEY7571314.1
Archaea Thermoproteati Nitrososphaerota Nitrososphaera sp. AFS WP 162476160.1
Archaea Thermoproteati Nitrososphaerota Candidatus Nitrosopolaris sp. MFY9799430.1
Archaea Thermoproteati Nitrososphaerota Candidatus Nitrosopolaris sp. MFZ0513413.1
Archaea Thermoproteati Nitrososphaerota Candidatus Nitrosopolaris sp. HET7147840.1
Archaea Thermoproteati Nitrososphaerota Nitrosopumilales archaeon MRN69050.1
Archaea Thermoproteati Nitrososphaerota Nitrososphaeraceae archaeon MFZ0346000.1
Archaea Thermoproteati Nitrososphaerota Candidatus Nitrosopolaris sp. HEY6667979.1
Archaea Thermoproteati Nitrososphaerota Nitrososphaeraceae archaeon MFL6323745.1
Archaea Thermoproteati Nitrososphaerota Nitrososphaeraceae archaeon HYZ50878.1
Archaea Thermoproteati Nitrososphaerota Nitrososphaeraceae archaeon HJT50113.1
Archaea Thermoproteati Nitrososphaerota Nitrososphaeraceae archaeon HEY9492496.1
Archaea Thermoproteati Nitrososphaerota Nitrososphaeraceae archaeon MGC2428994.1
Archaea Thermoproteati Nitrososphaerota Nitrososphaeraceae archaeon HZI71026.1
Archaea Thermoproteati Nitrososphaerota Nitrososphaeraceae archaeon MBV9667917.1
Archaea Thermoproteati Nitrososphaerota Nitrososphaeraceae archaeon HZC21284.1
Archaea Thermoproteati Nitrososphaerota Nitrososphaeraceae archaeon MCC2648396.1
Archaea Thermoproteati Nitrososphaerota Nitrososphaeraceae archaeon HEX2408742.1
Archaea Thermoproteati Nitrososphaerota Nitrososphaera sp. MEO9321137.1
Archaea Thermoproteati Nitrososphaerota Nitrososphaera sp. NOJ27342.1
Archaea Thermoproteati Nitrososphaerota Candidatus Nitrososphaera sp. FF02 WP 415282956.1
Archaea Thermoproteati Nitrososphaerota Nitrososphaera viennensis WP 227717414.1
Archaea Thermoproteati Nitrososphaerota Candidatus Nitrososphaera evergladensis WP 226987204.1
Archaea Thermoproteati Nitrososphaerota Nitrososphaera sp. HVX02349.1
Archaea Thermoproteati Nitrososphaerota Nitrososphaera sp. MEO9295488.1
Archaea Thermoproteati Nitrososphaerota Nitrososphaera sp. WP 431755696.1
Archaea Thermoproteati Nitrososphaerota Nitrososphaera sp. WP 337862851.1
Archaea Thermoproteati Nitrososphaerota Nitrososphaera sp. WP 331002313.1
Archaea Thermoproteati Nitrososphaerota Nitrososphaera sp. WP 292638793.1
Archaea Thermoproteati Nitrososphaerota Nitrososphaera sp. MGI0035563.1
Archaea Thermoproteati Nitrososphaerota Nitrososphaera sp. MCI0560861.1
Archaea Thermoproteati Nitrososphaerota Nitrososphaera sp. HEY7734148.1
Archaea Thermoproteati Nitrososphaerota Nitrososphaera sp. HKX82045.1
Archaea Thermoproteati Nitrososphaerota Nitrososphaera sp. HKU50512.1
Archaea Thermoproteati Nitrososphaerota Nitrososphaera sp. HEV8387325.1
Archaea Thermoproteati Nitrososphaerota Nitrososphaera sp. HEX2557205.1
Archaea Thermoproteati Nitrososphaerota Nitrososphaera sp. HZC88417.1
Archaea Thermoproteati Nitrososphaerota Nitrososphaera sp. HEY3094638.1
Archaea Thermoproteati Nitrososphaerota Nitrososphaera sp. HXV46744.1
Archaea Thermoproteati Nitrososphaerota Nitrososphaera sp. HJS67955.1
Archaea Thermoproteati Nitrososphaerota Nitrososphaerota archaeon TLY06569.1
Archaea Thermoproteati Nitrososphaerota Nitrososphaera sp. HEV8406107.1
Archaea Thermoproteati Nitrososphaerota Nitrososphaera sp. HUG97335.1
Archaea Thermoproteati Nitrososphaerota Nitrososphaera sp. HKZ62067.1
Archaea Thermoproteati Nitrososphaerota Candidatus Nitrososphaera gargensis WP 015019792.1
Archaea Thermoproteati Nitrososphaerota Nitrososphaera sp. MEP0825714.1
Archaea Thermoproteati Nitrososphaerota Nitrososphaera sp. HEX7033154.1
Archaea Thermoproteati Nitrososphaerota Nitrososphaera sp. HXG07875.1
Archaea Thermoproteati Nitrososphaerota Nitrososphaera sp. HYY40980.1
Archaea Thermoproteati Nitrososphaerota Nitrososphaera sp. MFL6473382.1
Archaea Thermoproteati Nitrososphaerota Nitrososphaera sp. HJU33807.1
Archaea Thermoproteati Nitrososphaerota Nitrososphaera sp. HEY9407379.1
Archaea Thermoproteati Nitrososphaerota Nitrososphaera sp. HEX2106913.1
Archaea Thermoproteati Nitrososphaerota Nitrososphaera sp. HEV3433449.1
Archaea Thermoproteati Nitrososphaerota Nitrososphaera sp. HEX2169160.1
Archaea Thermoproteati Nitrososphaerota Nitrososphaera sp. HET6808300.1
Archaea Thermoproteati Nitrososphaerota Nitrososphaera sp. HWS20902.1
Archaea Thermoproteati Nitrososphaerota Nitrososphaera sp. HKH86582.1
Archaea Thermoproteati Nitrososphaerota Nitrososphaera sp. HEX2472473.1
Archaea Thermoproteati Nitrososphaerota Nitrososphaera sp. MCY1155516.1
Archaea Thermoproteati Nitrososphaerota Nitrososphaera sp. HEY6756051.1
Archaea Thermoproteati Nitrososphaerota Nitrososphaera sp. MFL6492025.1
Archaea Thermoproteati Nitrososphaerota Nitrososphaera sp. HKF27192.1
Archaea Thermoproteati Nitrososphaerota Candidatus Nitrosocaldaceae archaeon MEM2856804.1
Archaea Thermoproteati Nitrososphaerota Candidatus Nitrosocaldaceae archaeon GIU70842.1
Archaea Thermoproteati Nitrososphaerota Nitrosopumilaceae archaeon MEX0656380.1

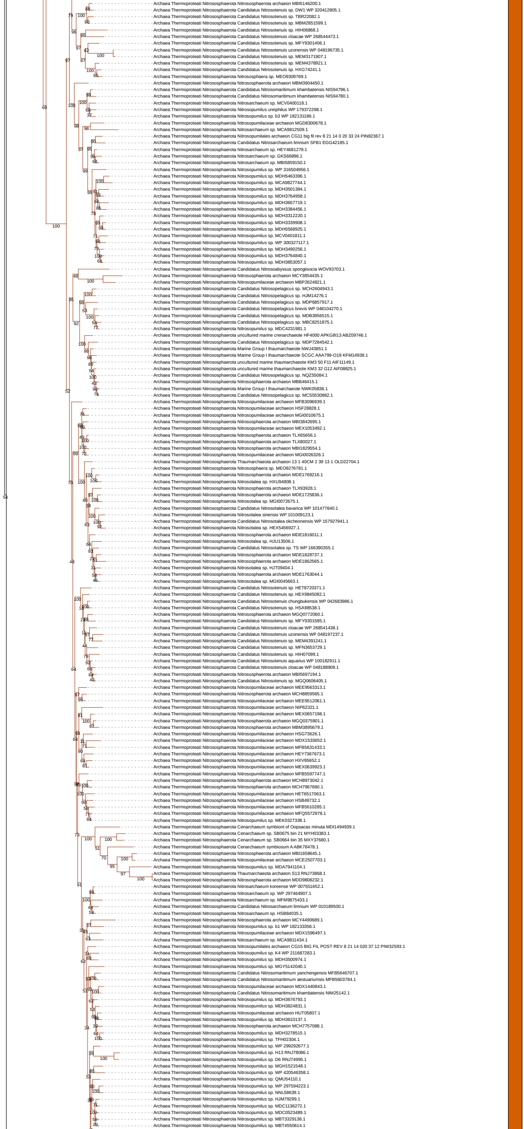
Archaea Thermoproteati Nitrososphaerota Nitrososphaerota archaeon MBI5146200.1
Archaea Thermoproteati Nitrososphaerota Candidatus Nitrosotenuis sp. DW1 WP 320412805.1
Archaea Thermoproteati Nitrososphaerota Candidatus Nitrosotenuis sp. TBR22082.1
Archaea Thermoproteati Nitrososphaerota Candidatus Nitrosotenuis sp. MBM2851599.1
Archaea Thermoproteati Nitrososphaerota Candidatus Nitrosotenuis sp. HIH06868.1
Archaea Thermoproteati Nitrososphaerota Candidatus Nitrosotenuis cloacae WP 268544473.1
Archaea Thermoproteati Nitrososphaerota Candidatus Nitrosotenuis sp. MFY9301406.1
Archaea Thermoproteati Nitrososphaerota Candidatus Nitrosotenuis uzonensis WP 048196735.1
Archaea Thermoproteati Nitrososphaerota Candidatus Nitrosotenuis sp. MEM3171907.1
Archaea Thermoproteati Nitrososphaerota Candidatus Nitrosotenuis sp. MEM4378921.1
Archaea Thermoproteati Nitrososphaerota Candidatus Nitrosotenuis sp. HXG74241.1
Archaea Thermoproteati Nitrososphaerota Nitrososphaera sp. MEO9309769.1
Archaea Thermoproteati Nitrososphaerota Nitrososphaerota archaeon MBM3904450.1
Archaea Thermoproteati Nitrososphaerota Candidatus Nitrosomaritimum khambatensis NIS94796.1
Archaea Thermoproteati Nitrososphaerota Candidatus Nitrosomaritimum khambatensis NIS94780.1
Archaea Thermoproteati Nitrososphaerota Nitrosarchaeum sp. MCV0400118.1
Archaea Thermoproteati Nitrososphaerota Nitrosopumilus ureiphilus WP 179372298.1
Archaea Thermoproteati Nitrososphaerota Nitrosopumilus sp. b3 WP 182131186.1
Archaea Thermoproteati Nitrososphaerota Nitrosopumilaceae archaeon MGD8300678.1
Archaea Thermoproteati Nitrososphaerota Nitrosarchaeum sp. MCA9812509.1
Archaea Thermoproteati Nitrososphaerota Nitrosopumilales archaeon CG11 big fil rev 8 21 14 0 20 33 24 PIN82367.1
Archaea Thermoproteati Nitrososphaerota Candidatus Nitrosarchaeum limnium SFB1 EGG42185.1
Archaea Thermoproteati Nitrososphaerota Nitrosarchaeum sp. HEY4681279.1
Archaea Thermoproteati Nitrososphaerota Nitrosarchaeum sp. GKS66896.1
Archaea Thermoproteati Nitrososphaerota Nitrosarchaeum sp. MBI5859150.1
Archaea Thermoproteati Nitrososphaerota Nitrosopumilus sp. WP 316504956.1
Archaea Thermoproteati Nitrososphaerota Nitrosopumilus sp. MDH5463396.1
Archaea Thermoproteati Nitrososphaerota Nitrosopumilus sp. MCA9827744.1
Archaea Thermoproteati Nitrososphaerota Nitrosopumilus sp. MDH3501384.1
Archaea Thermoproteati Nitrososphaerota Nitrosopumilus sp. MDH3764958.1
Archaea Thermoproteati Nitrososphaerota Nitrosopumilus sp. MDH3657719.1
Archaea Thermoproteati Nitrososphaerota Nitrosopumilus sp. MDH3384456.1
Archaea Thermoproteati Nitrososphaerota Nitrosopumilus sp. MDH3312220.1
Archaea Thermoproteati Nitrososphaerota Nitrosopumilus sp. MDH3339908.1
Archaea Thermoproteati Nitrososphaerota Nitrosopumilus sp. MDH5568925.1
Archaea Thermoproteati Nitrososphaerota Nitrosopumilus sp. MCV0401811.1
Archaea Thermoproteati Nitrososphaerota Nitrosopumilus sp. WP 300327117.1
Archaea Thermoproteati Nitrososphaerota Nitrosopumilus sp. MDH3490256.1
Archaea Thermoproteati Nitrososphaerota Nitrosopumilus sp. MDH3764840.1
Archaea Thermoproteati Nitrososphaerota Nitrosopumilus sp. MDH3853057.1
Archaea Thermoproteati Nitrososphaerota Candidatus Nitrosoabyssus spongiisocia WOV93703.1
Archaea Thermoproteati Nitrososphaerota Nitrososphaerota archaeon MCY3854435.1
Archaea Thermoproteati Nitrososphaerota Nitrosopumilaceae archaeon MBP2624821.1
Archaea Thermoproteati Nitrososphaerota Candidatus Nitrosopelagicus sp. MCH2604943.1
Archaea Thermoproteati Nitrososphaerota Candidatus Nitrosopelagicus sp. HJM14276.1
Archaea Thermoproteati Nitrososphaerota Candidatus Nitrosopelagicus sp. MDP6857917.1
Archaea Thermoproteati Nitrososphaerota Candidatus Nitrosopelagicus brevis WP 048104270.1
Archaea Thermoproteati Nitrososphaerota Candidatus Nitrosopelagicus sp. MDB3956515.1
Archaea Thermoproteati Nitrososphaerota Candidatus Nitrosopelagicus sp. MBC8251875.1
Archaea Thermoproteati Nitrososphaerota Nitrosopumilus sp. MDC4231981.1
Archaea Thermoproteati Nitrososphaerota uncultured marine crenarchaeote HF4000 APKG8I13 ABZ09746.1
Archaea Thermoproteati Nitrososphaerota Candidatus Nitrosopelagicus sp. MDP7284542.1
Archaea Thermoproteati Nitrososphaerota Marine Group I thaumarchaeote NWJ43851.1
Archaea Thermoproteati Nitrososphaerota Marine Group I thaumarchaeote SCGC AAA799-O18 KFM14938.1
Archaea Thermoproteati Nitrososphaerota uncultured marine thaumarchaeote KM3 50 F11 AIF11149.1
Archaea Thermoproteati Nitrososphaerota uncultured marine thaumarchaeote KM3 32 G12 AIF08825.1
Archaea Thermoproteati Nitrososphaerota Candidatus Nitrosopelagicus sp. NQZ55084.1
Archaea Thermoproteati Nitrososphaerota Nitrososphaerota archaeon MBB46415.1
Archaea Thermoproteati Nitrososphaerota Marine Group I thaumarchaeote NWK05836.1
Archaea Thermoproteati Nitrososphaerota Candidatus Nitrosopelagicus sp. MCS5530862.1
Archaea Thermoproteati Nitrososphaerota Nitrosopumilaceae archaeon MFB3096939.1
Archaea Thermoproteati Nitrososphaerota Nitrosopumilaceae archaeon HSF28828.1
Archaea Thermoproteati Nitrososphaerota Nitrosopumilaceae archaeon MGI0010675.1
Archaea Thermoproteati Nitrososphaerota Nitrososphaerota archaeon MBI3842695.1
Archaea Thermoproteati Nitrososphaerota Nitrosopumilaceae archaeon MEX1053492.1
Archaea Thermoproteati Nitrososphaerota Nitrososphaerota archaeon TLX65656.1
Archaea Thermoproteati Nitrososphaerota Nitrososphaerota archaeon TLX80027.1
Archaea Thermoproteati Nitrososphaerota Nitrososphaerota archaeon MBI1829554.1
Archaea Thermoproteati Nitrososphaerota Nitrosopumilaceae archaeon MGI0026326.1
Archaea Thermoproteati Nitrososphaerota Thaumarchaeota archaeon 13 1 40CM 2 39 13 1 OLD22704.1
Archaea Thermoproteati Nitrososphaerota Nitrososphaera sp. MEO9276781.1
Archaea Thermoproteati Nitrososphaerota Nitrososphaerota archaeon MDE1769216.1
Archaea Thermoproteati Nitrososphaerota Nitrosotalea sp. HXU94808.1
Archaea Thermoproteati Nitrososphaerota Nitrososphaerota archaeon TLX93928.1
Archaea Thermoproteati Nitrososphaerota Nitrososphaerota archaeon MDE1725836.1
Archaea Thermoproteati Nitrososphaerota Nitrosotalea sp. MGI0072675.1
Archaea Thermoproteati Nitrososphaerota Candidatus Nitrosotalea bavarica WP 101477840.1
Archaea Thermoproteati Nitrososphaerota Nitrosotalea sinensis WP 101009123.1
Archaea Thermoproteati Nitrososphaerota Candidatus Nitrosotalea okcheonensis WP 157927941.1
Archaea Thermoproteati Nitrososphaerota Nitrosotalea sp. HEX5456927.1
Archaea Thermoproteati Nitrososphaerota Nitrososphaerota archaeon MDE1816011.1
Archaea Thermoproteati Nitrososphaerota Nitrosotalea sp. HJU13506.1
Archaea Thermoproteati Nitrososphaerota Candidatus Nitrosotalea sp. TS WP 166390355.1
Archaea Thermoproteati Nitrososphaerota Nitrososphaerota archaeon MDE1828737.1
Archaea Thermoproteati Nitrososphaerota Nitrososphaerota archaeon MDE1862565.1
Archaea Thermoproteati Nitrososphaerota Nitrosotalea sp. HJT09404.1
Archaea Thermoproteati Nitrososphaerota Nitrososphaerota archaeon MDE1763044.1
Archaea Thermoproteati Nitrososphaerota Nitrosotalea sp. MGI0045663.1
Archaea Thermoproteati Nitrososphaerota Candidatus Nitrosotenuis sp. HET8720371.1
Archaea Thermoproteati Nitrososphaerota Candidatus Nitrosotenuis sp. HEX9845082.1
Archaea Thermoproteati Nitrososphaerota Candidatus Nitrosotenuis chungbukensis WP 042683986.1
Archaea Thermoproteati Nitrososphaerota Candidatus Nitrosotenuis sp. HSA98538.1
Archaea Thermoproteati Nitrososphaerota Nitrososphaerota archaeon MGQ0772060.1
Archaea Thermoproteati Nitrososphaerota Candidatus Nitrosotenuis sp. MFY9301585.1
Archaea Thermoproteati Nitrososphaerota Candidatus Nitrosotenuis cloacae WP 268541438.1
Archaea Thermoproteati Nitrososphaerota Candidatus Nitrosotenuis uzonensis WP 048197237.1
Archaea Thermoproteati Nitrososphaerota Candidatus Nitrosotenuis sp. MEM4391241.1
Archaea Thermoproteati Nitrososphaerota Candidatus Nitrosotenuis sp. MFN3653729.1
Archaea Thermoproteati Nitrososphaerota Candidatus Nitrosotenuis sp. HIH07099.1
Archaea Thermoproteati Nitrososphaerota Candidatus Nitrosotenuis aquarius WP 100182911.1
Archaea Thermoproteati Nitrososphaerota Candidatus Nitrosotenuis cloacae WP 048188909.1
Archaea Thermoproteati Nitrososphaerota Nitrososphaerota archaeon MBI5697194.1
Archaea Thermoproteati Nitrososphaerota Candidatus Nitrosotenuis sp. MGQ0606405.1
Archaea Thermoproteati Nitrososphaerota Nitrosopumilaceae archaeon MEE9563313.1
Archaea Thermoproteati Nitrososphaerota Nitrososphaerota archaeon MCH8859565.1
Archaea Thermoproteati Nitrososphaerota Nitrosopumilaceae archaeon MEE9512061.1
Archaea Thermoproteati Nitrososphaerota Nitrosopumilaceae archaeon NIP62321.1
Archaea Thermoproteati Nitrososphaerota Nitrosopumilaceae archaeon MEX0657198.1
Archaea Thermoproteati Nitrososphaerota Nitrososphaerota archaeon MGQ0375901.1
Archaea Thermoproteati Nitrososphaerota Nitrososphaerota archaeon MBM3895679.1
Archaea Thermoproteati Nitrososphaerota Nitrosopumilaceae archaeon HSG73626.1
Archaea Thermoproteati Nitrososphaerota Nitrosopumilaceae archaeon MDX1533652.1
Archaea Thermoproteati Nitrososphaerota Nitrosopumilaceae archaeon MFB5631433.1
Archaea Thermoproteati Nitrososphaerota Nitrosopumilaceae archaeon HEY7367673.1
Archaea Thermoproteati Nitrososphaerota Nitrosopumilaceae archaeon HXV65652.1
Archaea Thermoproteati Nitrososphaerota Nitrosopumilaceae archaeon MEX0639923.1
Archaea Thermoproteati Nitrososphaerota Nitrosopumilaceae archaeon MFB5597747.1
Archaea Thermoproteati Nitrososphaerota Nitrososphaerota archaeon MCH8973042.1
Archaea Thermoproteati Nitrososphaerota Nitrososphaerota archaeon MCH7967660.1
Archaea Thermoproteati Nitrososphaerota Nitrosopumilaceae archaeon HET6517063.1
Archaea Thermoproteati Nitrososphaerota Nitrosopumilaceae archaeon HSB49732.1
Archaea Thermoproteati Nitrososphaerota Nitrosopumilaceae archaeon MFB5610285.1
Archaea Thermoproteati Nitrososphaerota Nitrosopumilaceae archaeon MFQ5572978.1
Archaea Thermoproteati Nitrososphaerota Nitrosopumilus sp. MEK0327338.1
Archaea Thermoproteati Nitrososphaerota Cenarchaeum symbiont of Oopsacas minuta MDI1494939.1
Archaea Thermoproteati Nitrososphaerota Cenarchaeum sp. SB0675 bin 21 MYH03383.1
Archaea Thermoproteati Nitrososphaerota Cenarchaeum sp. SB0664 bin 35 MXY37680.1
Archaea Thermoproteati Nitrososphaerota Cenarchaeum symbiosum A ABK78478.1
Archaea Thermoproteati Nitrososphaerota Nitrososphaerota archaeon MBI1658645.1
Archaea Thermoproteati Nitrososphaerota Nitrosopumilaceae archaeon MCE2507703.1
Archaea Thermoproteati Nitrososphaerota Nitrosopumilus sp. MDA7941104.1
Archaea Thermoproteati Nitrososphaerota Thaumarchaeota archaeon S13 RNJ73868.1
Archaea Thermoproteati Nitrososphaerota Nitrososphaerota archaeon MDD9808232.1
Archaea Thermoproteati Nitrososphaerota Nitrosarchaeum koreense WP 007551652.1
Archaea Thermoproteati Nitrososphaerota Nitrosarchaeum sp. WP 297464907.1
Archaea Thermoproteati Nitrososphaerota Nitrosarchaeum sp. MFM9875433.1
Archaea Thermoproteati Nitrososphaerota Candidatus Nitrosarchaeum limnium WP 010189500.1
Archaea Thermoproteati Nitrososphaerota Nitrosarchaeum sp. HSB84035.1
Archaea Thermoproteati Nitrososphaerota Nitrososphaerota archaeon MCY4490689.1
Archaea Thermoproteati Nitrososphaerota Nitrosopumilus sp. b1 WP 182133356.1
Archaea Thermoproteati Nitrososphaerota Nitrosopumilaceae archaeon MDX1596497.1
Archaea Thermoproteati Nitrososphaerota Nitrosarchaeum sp. MCA9811434.1
Archaea Thermoproteati Nitrososphaerota Nitrosopumilales archaeon CG15 BIG FIL POST REV 8 21 14 020 37 12 PIW32593.1
Archaea Thermoproteati Nitrososphaerota Nitrosopumilus sp. K4 WP 211687283.1
Archaea Thermoproteati Nitrososphaerota Nitrosopumilus sp. MDH3500974.1
Archaea Thermoproteati Nitrososphaerota Nitrosopumilus sp. MGY5142040.1
Archaea Thermoproteati Nitrososphaerota Candidatus Nitrosomaritimum yanchengensis MFB5646707.1
Archaea Thermoproteati Nitrososphaerota Candidatus Nitrosomaritimum aestuariumsis MFB5603784.1
Archaea Thermoproteati Nitrososphaerota Nitrosopumilaceae archaeon MDX1440843.1
Archaea Thermoproteati Nitrososphaerota Candidatus Nitrosomaritimum khambatensis NIM25142.1
Archaea Thermoproteati Nitrososphaerota Nitrosopumilus sp. MDH3676793.1
Archaea Thermoproteati Nitrososphaerota Nitrosopumilus sp. MDH3824831.1
Archaea Thermoproteati Nitrososphaerota Nitrosopumilaceae archaeon HUT05807.1
Archaea Thermoproteati Nitrososphaerota Nitrosopumilus sp. MDH3610137.1
Archaea Thermoproteati Nitrososphaerota Nitrososphaerota archaeon MCH7757088.1
Archaea Thermoproteati Nitrososphaerota Nitrosopumilus sp. MDH3278515.1
Archaea Thermoproteati Nitrososphaerota Nitrosopumilus sp. TFH02304.1
Archaea Thermoproteati Nitrososphaerota Nitrosopumilus sp. WP 299292677.1
Archaea Thermoproteati Nitrososphaerota Nitrosopumilus sp. H13 RNJ78086.1
Archaea Thermoproteati Nitrososphaerota Nitrosopumilus sp. D6 RNJ74995.1
Archaea Thermoproteati Nitrososphaerota Nitrosopumilus sp. MGH1521548.1
Archaea Thermoproteati Nitrososphaerota Nitrosopumilus sp. WP 420546358.1
Archaea Thermoproteati Nitrososphaerota Nitrosopumilus sp. QMU54110.1
Archaea Thermoproteati Nitrososphaerota Nitrosopumilus sp. WP 297594223.1
Archaea Thermoproteati Nitrososphaerota Nitrosopumilus sp. NNL58639.1
Archaea Thermoproteati Nitrososphaerota Nitrosopumilus sp. HJM79299.1
Archaea Thermoproteati Nitrososphaerota Nitrosopumilus sp. MDC1136272.1
Archaea Thermoproteati Nitrososphaerota Nitrosopumilus sp. MDC0523489.1
Archaea Thermoproteati Nitrososphaerota Nitrosopumilus sp. MBT3329136.1
Archaea Thermoproteati Nitrososphaerota Nitrosopumilus sp. MBT4550614.1

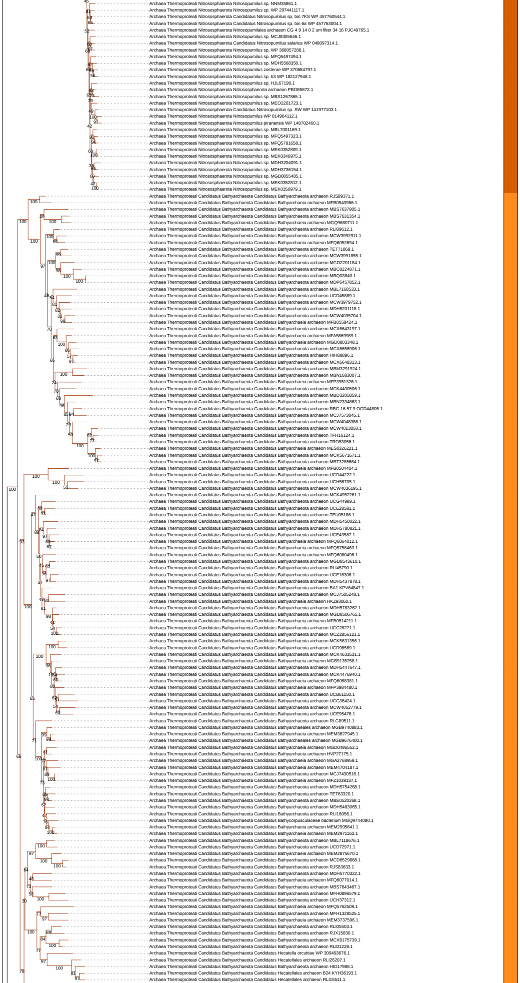

Archaea Thermoproteati Nitrososphaerota Nitrosopumilus sp. NNM35861.1
Archaea Thermoproteati Nitrososphaerota Nitrosopumilus sp. WP 297441117.1
Archaea Thermoproteati Nitrososphaerota Candidatus Nitrosopumilus sp. bin 7KS WP 457760544.1
Archaea Thermoproteati Nitrososphaerota Candidatus Nitrosopumilus sp. bin 6a WP 457763004.1
Archaea Thermoproteati Nitrososphaerota Nitrosopumilales archaeon CG 4 9 14 0 2 um filter 34 16 PJC49765.1
Archaea Thermoproteati Nitrososphaerota Nitrosopumilus sp. MCJ8305646.1
Archaea Thermoproteati Nitrososphaerota Candidatus Nitrosopumilus salarius WP 048097314.1
Archaea Thermoproteati Nitrososphaerota Nitrosopumilus sp. WP 368057288.1
Archaea Thermoproteati Nitrososphaerota Nitrosopumilus sp. MFQ5497494.1
Archaea Thermoproteati Nitrososphaerota Nitrosopumilus sp. MDH5568350.1
Archaea Thermoproteati Nitrososphaerota Nitrosopumilus zosterae WP 370684797.1
Archaea Thermoproteati Nitrososphaerota Nitrosopumilus sp. b3 WP 182127948.1
Archaea Thermoproteati Nitrososphaerota Nitrosopumilus sp. HJL67190.1
Archaea Thermoproteati Nitrososphaerota Nitrososphaerota archaeon PBO85872.1
Archaea Thermoproteati Nitrososphaerota Nitrosopumilus sp. MBS1267865.1
Archaea Thermoproteati Nitrososphaerota Nitrosopumilus sp. MEO2201723.1
Archaea Thermoproteati Nitrososphaerota Candidatus Nitrosopumilus sp. SW WP 141977103.1
Archaea Thermoproteati Nitrososphaerota Nitrosopumilus WP 014964112.1
Archaea Thermoproteati Nitrososphaerota Nitrosopumilus piranensis WP 148702460.1
Archaea Thermoproteati Nitrososphaerota Nitrosopumilus sp. MBL7001169.1
Archaea Thermoproteati Nitrososphaerota Nitrosopumilus sp. MFQ5497323.1
Archaea Thermoproteati Nitrososphaerota Nitrosopumilus sp. MFQ5781658.1
Archaea Thermoproteati Nitrososphaerota Nitrosopumilus sp. MEK0352809.1
Archaea Thermoproteati Nitrososphaerota Nitrosopumilus sp. MEK0346975.1
Archaea Thermoproteati Nitrososphaerota Nitrosopumilus sp. MDH3204091.1
Archaea Thermoproteati Nitrososphaerota Nitrosopumilus sp. MDH3736154.1
Archaea Thermoproteati Nitrososphaerota Nitrosopumilus sp. MGB0855485.1
Archaea Thermoproteati Nitrososphaerota Nitrosopumilus sp. MEK0352812.1
Archaea Thermoproteati Nitrososphaerota Nitrosopumilus sp. MEK0350979.1
Archaea Thermoproteati Candidatus Bathyarchaeota Candidatus Bathyarchaeota archaeon RJS89371.1
Archaea Thermoproteati Candidatus Bathyarchaeota Candidatus Bathyarchaeia archaeon MFB0543966.1
Archaea Thermoproteati Candidatus Bathyarchaeota Candidatus Bathyarchaeota archaeon MBS7637905.1
Archaea Thermoproteati Candidatus Bathyarchaeota Candidatus Bathyarchaeota archaeon MBS7631354.1
Archaea Thermoproteati Candidatus Bathyarchaeota Candidatus Bathyarchaeia archaeon MGQ9680711.1
Archaea Thermoproteati Candidatus Bathyarchaeota Candidatus Bathyarchaeota archaeon RLI09612.1
Archaea Thermoproteati Candidatus Bathyarchaeota Candidatus Bathyarchaeota archaeon MCW3992911.1
Archaea Thermoproteati Candidatus Bathyarchaeota Candidatus Bathyarchaeia archaeon MFQ6052694.1
Archaea Thermoproteati Candidatus Bathyarchaeota Candidatus Bathyarchaeota archaeon TET71868.1
Archaea Thermoproteati Candidatus Bathyarchaeota Candidatus Bathyarchaeota archaeon MCW3991855.1
Archaea Thermoproteati Candidatus Bathyarchaeota Candidatus Bathyarchaeota archaeon MGD2201184.1
Archaea Thermoproteati Candidatus Bathyarchaeota Candidatus Bathyarchaeota archaeon MBC8224871.1
Archaea Thermoproteati Candidatus Bathyarchaeota Candidatus Bathyarchaeota archaeon MBQ03840.1
Archaea Thermoproteati Candidatus Bathyarchaeota Candidatus Bathyarchaeota archaeon MDP6457852.1
Archaea Thermoproteati Candidatus Bathyarchaeota Candidatus Bathyarchaeota archaeon MBL7168533.1
Archaea Thermoproteati Candidatus Bathyarchaeota Candidatus Bathyarchaeota archaeon UCD45889.1
Archaea Thermoproteati Candidatus Bathyarchaeota Candidatus Bathyarchaeota archaeon MCW3979752.1
Archaea Thermoproteati Candidatus Bathyarchaeota Candidatus Bathyarchaeota archaeon MDH5201118.1
Archaea Thermoproteati Candidatus Bathyarchaeota Candidatus Bathyarchaeota archaeon MCW4035704.1
Archaea Thermoproteati Candidatus Bathyarchaeota Candidatus Bathyarchaeia archaeon MFB0558424.1
Archaea Thermoproteati Candidatus Bathyarchaeota Candidatus Bathyarchaeota archaeon MCX6643197.1
Archaea Thermoproteati Candidatus Bathyarchaeota Candidatus Bathyarchaeia archaeon MFA5869989.1
Archaea Thermoproteati Candidatus Bathyarchaeota Candidatus Bathyarchaeota archaeon MGD0803348.1
Archaea Thermoproteati Candidatus Bathyarchaeota Candidatus Bathyarchaeota archaeon MCX6656906.1
Archaea Thermoproteati Candidatus Bathyarchaeota Candidatus Bathyarchaeota archaeon HIH88896.1
Archaea Thermoproteati Candidatus Bathyarchaeota Candidatus Bathyarchaeota archaeon MCX6648313.1
Archaea Thermoproteati Candidatus Bathyarchaeota Candidatus Bathyarchaeota archaeon MBM3291924.1
Archaea Thermoproteati Candidatus Bathyarchaeota Candidatus Bathyarchaeota archaeon MBN1683007.1
Archaea Thermoproteati Candidatus Bathyarchaeota Candidatus Bathyarchaeia archaeon MFP3951326.1
Archaea Thermoproteati Candidatus Bathyarchaeota Candidatus Bathyarchaeota archaeon MCK4400506.1
Archaea Thermoproteati Candidatus Bathyarchaeota Candidatus Bathyarchaeota archaeon MBD3205859.1
Archaea Thermoproteati Candidatus Bathyarchaeota Candidatus Bathyarchaeota archaeon MBN2334863.1
Archaea Thermoproteati Candidatus Bathyarchaeota Candidatus Bathyarchaeota archaeon RBG 16 57 9 OGD44805.1
Archaea Thermoproteati Candidatus Bathyarchaeota Candidatus Bathyarchaeota archaeon MCJ7573045.1
Archaea Thermoproteati Candidatus Bathyarchaeota Candidatus Bathyarchaeota archaeon MCW4048386.1
Archaea Thermoproteati Candidatus Bathyarchaeota Candidatus Bathyarchaeota archaeon MCW4013050.1
Archaea Thermoproteati Candidatus Bathyarchaeota Candidatus Bathyarchaeota archaeon TFH16124.1
Archaea Thermoproteati Candidatus Bathyarchaeota Candidatus Bathyarchaeota archaeon TRO53056.1
Archaea Thermoproteati Candidatus Bathyarchaeota Candidatus Bathyarchaeia archaeon MES0326221.1
Archaea Thermoproteati Candidatus Bathyarchaeota Candidatus Bathyarchaeota archaeon MCK5671671.1
Archaea Thermoproteati Candidatus Bathyarchaeota Candidatus Bathyarchaeota archaeon MBT3285664.1
Archaea Thermoproteati Candidatus Bathyarchaeota Candidatus Bathyarchaeia archaeon MFB0504404.1
Archaea Thermoproteati Candidatus Bathyarchaeota Candidatus Bathyarchaeota archaeon UCD44222.1
Archaea Thermoproteati Candidatus Bathyarchaeota Candidatus Bathyarchaeota archaeon UCH56705.1
Archaea Thermoproteati Candidatus Bathyarchaeota Candidatus Bathyarchaeota archaeon MCW4036195.1
Archaea Thermoproteati Candidatus Bathyarchaeota Candidatus Bathyarchaeota archaeon MCK4952261.1
Archaea Thermoproteati Candidatus Bathyarchaeota Candidatus Bathyarchaeota archaeon UCG44989.1
Archaea Thermoproteati Candidatus Bathyarchaeota Candidatus Bathyarchaeota archaeon UCE28581.1
Archaea Thermoproteati Candidatus Bathyarchaeota Candidatus Bathyarchaeota archaeon TEU05186.1
Archaea Thermoproteati Candidatus Bathyarchaeota Candidatus Bathyarchaeota archaeon MDH5450022.1
Archaea Thermoproteati Candidatus Bathyarchaeota Candidatus Bathyarchaeota archaeon MDH5780821.1
Archaea Thermoproteati Candidatus Bathyarchaeota Candidatus Bathyarchaeota archaeon UCE43587.1
Archaea Thermoproteati Candidatus Bathyarchaeota Candidatus Bathyarchaeia archaeon MFQ6064012.1
Archaea Thermoproteati Candidatus Bathyarchaeota Candidatus Bathyarchaeia archaeon MFQ5758463.1
Archaea Thermoproteati Candidatus Bathyarchaeota Candidatus Bathyarchaeia archaeon MFQ6080496.1
Archaea Thermoproteati Candidatus Bathyarchaeota Candidatus Bathyarchaeota archaeon MGD8543610.1
Archaea Thermoproteati Candidatus Bathyarchaeota Candidatus Bathyarchaeota archaeon RLI45790.1
Archaea Thermoproteati Candidatus Bathyarchaeota Candidatus Bathyarchaeota archaeon UCE16306.1
Archaea Thermoproteati Candidatus Bathyarchaeota Candidatus Bathyarchaeota archaeon MDH5437878.1
Archaea Thermoproteati Candidatus Bathyarchaeota Candidatus Bathyarchaeota archaeon BA1 KPV64847.1
Archaea Thermoproteati Candidatus Bathyarchaeota Candidatus Bathyarchaeota archaeon MCJ7505248.1
Archaea Thermoproteati Candidatus Bathyarchaeota Candidatus Bathyarchaeota archaeon HKZ93060.1
Archaea Thermoproteati Candidatus Bathyarchaeota Candidatus Bathyarchaeota archaeon MDH5783262.1
Archaea Thermoproteati Candidatus Bathyarchaeota Candidatus Bathyarchaeota archaeon MGD8506765.1
Archaea Thermoproteati Candidatus Bathyarchaeota Candidatus Bathyarchaeia archaeon MFB0514211.1
Archaea Thermoproteati Candidatus Bathyarchaeota Candidatus Bathyarchaeota archaeon UCC28271.1
Archaea Thermoproteati Candidatus Bathyarchaeota Candidatus Bathyarchaeota archaeon MCZ2856121.1
Archaea Thermoproteati Candidatus Bathyarchaeota Candidatus Bathyarchaeota archaeon MCK5631356.1
Archaea Thermoproteati Candidatus Bathyarchaeota Candidatus Bathyarchaeota archaeon UCD96569.1
Archaea Thermoproteati Candidatus Bathyarchaeota Candidatus Bathyarchaeota archaeon MCK4633531.1
Archaea Thermoproteati Candidatus Bathyarchaeota Candidatus Bathyarchaeia archaeon MGB9135258.1
Archaea Thermoproteati Candidatus Bathyarchaeota Candidatus Bathyarchaeota archaeon MDH5447647.1
Archaea Thermoproteati Candidatus Bathyarchaeota Candidatus Bathyarchaeota archaeon MCK4476940.1
Archaea Thermoproteati Candidatus Bathyarchaeota Candidatus Bathyarchaeia archaeon MFQ6068381.1
Archaea Thermoproteati Candidatus Bathyarchaeota Candidatus Bathyarchaeia archaeon MFP3984480.1
Archaea Thermoproteati Candidatus Bathyarchaeota Candidatus Bathyarchaeota archaeon UCB61150.1
Archaea Thermoproteati Candidatus Bathyarchaeota Candidatus Bathyarchaeota archaeon UCG36424.1
Archaea Thermoproteati Candidatus Bathyarchaeota Candidatus Bathyarchaeota archaeon MCW4052779.1
Archaea Thermoproteati Candidatus Bathyarchaeota Candidatus Bathyarchaeota archaeon UCE95476.1
Archaea Thermoproteati Candidatus Bathyarchaeota Candidatus Bathyarchaeota archaeon RLG89511.1
Archaea Thermoproteati Candidatus Bathyarchaeota Candidatus Bathyarchaeales archaeon MGB9740883.1
Archaea Thermoproteati Candidatus Bathyarchaeota Candidatus Bathyarchaeia archaeon MEM3627945.1
Archaea Thermoproteati Candidatus Bathyarchaeota Candidatus Bathyarchaeales archaeon MGB9676400.1
Archaea Thermoproteati Candidatus Bathyarchaeota Candidatus Bathyarchaeia archaeon MGD0496552.1
Archaea Thermoproteati Candidatus Bathyarchaeota Candidatus Bathyarchaeia archaeon HVP27175.1
Archaea Thermoproteati Candidatus Bathyarchaeota Candidatus Bathyarchaeia archaeon MGA2768959.1
Archaea Thermoproteati Candidatus Bathyarchaeota Candidatus Bathyarchaeia archaeon MEM4704187.1
Archaea Thermoproteati Candidatus Bathyarchaeota Candidatus Bathyarchaeota archaeon MCJ7430518.1
Archaea Thermoproteati Candidatus Bathyarchaeota Candidatus Bathyarchaeia archaeon MFZ1039137.1
Archaea Thermoproteati Candidatus Bathyarchaeota Candidatus Bathyarchaeota archaeon MDH5754268.1
Archaea Thermoproteati Candidatus Bathyarchaeota Candidatus Bathyarchaeota archaeon TET63320.1
Archaea Thermoproteati Candidatus Bathyarchaeota Candidatus Bathyarchaeota archaeon MBE0520268.1
Archaea Thermoproteati Candidatus Bathyarchaeota Candidatus Bathyarchaeota archaeon MDH5483065.1
Archaea Thermoproteati Candidatus Bathyarchaeota Candidatus Bathyarchaeota archaeon RLI16056.1
Archaea Thermoproteati Candidatus Bathyarchaeota Candidatus Bathycorpusculaceae bacterium MGQ9744080.1
Archaea Thermoproteati Candidatus Bathyarchaeota Candidatus Bathyarchaeia archaeon MEM2995641.1
Archaea Thermoproteati Candidatus Bathyarchaeota Candidatus Bathyarchaeia archaeon MEM2971162.1
Archaea Thermoproteati Candidatus Bathyarchaeota Candidatus Bathyarchaeota archaeon MBL7118676.1
Archaea Thermoproteati Candidatus Bathyarchaeota Candidatus Bathyarchaeota archaeon UCD72971.1
Archaea Thermoproteati Candidatus Bathyarchaeota Candidatus Bathyarchaeia archaeon MEM2875670.1
Archaea Thermoproteati Candidatus Bathyarchaeota Candidatus Bathyarchaeota archaeon MCD6529668.1
Archaea Thermoproteati Candidatus Bathyarchaeota Candidatus Bathyarchaeota archaeon RJS83633.1
Archaea Thermoproteati Candidatus Bathyarchaeota Candidatus Bathyarchaeota archaeon MDH5770322.1
Archaea Thermoproteati Candidatus Bathyarchaeota Candidatus Bathyarchaeia archaeon MFQ6077014.1
Archaea Thermoproteati Candidatus Bathyarchaeota Candidatus Bathyarchaeota archaeon MBS7643467.1
Archaea Thermoproteati Candidatus Bathyarchaeota Candidatus Bathyarchaeia archaeon MFH0896579.1
Archaea Thermoproteati Candidatus Bathyarchaeota Candidatus Bathyarchaeota archaeon UCH37312.1
Archaea Thermoproteati Candidatus Bathyarchaeota Candidatus Bathyarchaeia archaeon MFQ5762509.1
Archaea Thermoproteati Candidatus Bathyarchaeota Candidatus Bathyarchaeota archaeon MFH1328525.1
Archaea Thermoproteati Candidatus Bathyarchaeota Candidatus Bathyarchaeia archaeon MEM3737596.1
Archaea Thermoproteati Candidatus Bathyarchaeota Candidatus Bathyarchaeota archaeon RLI05503.1
Archaea Thermoproteati Candidatus Bathyarchaeota Candidatus Bathyarchaeota archaeon RJX15830.1
Archaea Thermoproteati Candidatus Bathyarchaeota Candidatus Bathyarchaeota archaeon MCX8175739.1
Archaea Thermoproteati Candidatus Bathyarchaeota Candidatus Bathyarchaeota archaeon RLI01228.1
Archaea Thermoproteati Candidatus Bathyarchaeota Candidatus Hecatella orcuttiae WP 309493676.1
Archaea Thermoproteati Candidatus Bathyarchaeota Candidatus Hecatellales archaeon RLI26207.1
Archaea Thermoproteati Candidatus Bathyarchaeota Candidatus Bathyarchaeota archaeon HID17988.1
Archaea Thermoproteati Candidatus Bathyarchaeota Candidatus Hecatellales archaeon B24 KYH36193.1
Archaea Thermoproteati Candidatus Bathyarchaeota Candidatus Hecatellales archaeon RLI15511.1

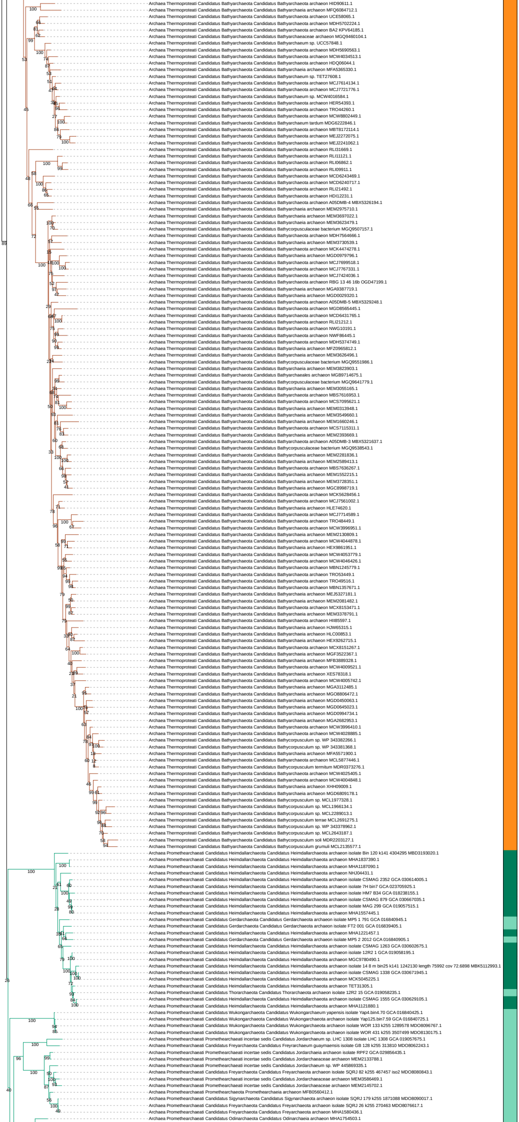
Archaea Thermoproteati Candidatus Bathyarchaeota Candidatus Bathyarchaeota archaeon HID90611.1
Archaea Thermoproteati Candidatus Bathyarchaeota Candidatus Bathyarchaeia archaeon MFQ6084712.1
Archaea Thermoproteati Candidatus Bathyarchaeota Candidatus Bathyarchaeota archaeon UCE58065.1
Archaea Thermoproteati Candidatus Bathyarchaeota Candidatus Bathyarchaeota archaeon MDH5702224.1
Archaea Thermoproteati Candidatus Bathyarchaeota Candidatus Bathyarchaeota archaeon BA2 KPV64185.1
Archaea Thermoproteati Candidatus Bathyarchaeota Candidatus Bathyarchaeaceae archaeon MGQ9460104.1
Archaea Thermoproteati Candidatus Bathyarchaeota Candidatus Bathyarchaeum sp. UCC57848.1
Archaea Thermoproteati Candidatus Bathyarchaeota Candidatus Bathyarchaeota archaeon MDH5690563.1
Archaea Thermoproteati Candidatus Bathyarchaeota Candidatus Bathyarchaeota archaeon MCW4034513.1
Archaea Thermoproteati Candidatus Bathyarchaeota Candidatus Bathyarchaeota archaeon HDQ06044.1
Archaea Thermoproteati Candidatus Bathyarchaeota Candidatus Bathyarchaeia archaeon MFA5365330.1
Archaea Thermoproteati Candidatus Bathyarchaeota Candidatus Bathyarchaeum sp. TET27608.1
Archaea Thermoproteati Candidatus Bathyarchaeota Candidatus Bathyarchaeota archaeon MCJ7614134.1
Archaea Thermoproteati Candidatus Bathyarchaeota Candidatus Bathyarchaeota archaeon MCJ7721776.1
Archaea Thermoproteati Candidatus Bathyarchaeota Candidatus Bathyarchaeum sp. MCW4016584.1
Archaea Thermoproteati Candidatus Bathyarchaeota Candidatus Bathyarchaeota archaeon HER54393.1
Archaea Thermoproteati Candidatus Bathyarchaeota Candidatus Bathyarchaeota archaeon TRO44260.1
Archaea Thermoproteati Candidatus Bathyarchaeota Candidatus Bathyarchaeota archaeon MCW8802449.1
Archaea Thermoproteati Candidatus Bathyarchaeota Candidatus Bathyarchaeum tardum MDG6222846.1
Archaea Thermoproteati Candidatus Bathyarchaeota Candidatus Bathyarchaeota archaeon MBT8172114.1
Archaea Thermoproteati Candidatus Bathyarchaeota Candidatus Bathyarchaeota archaeon MEJ2272075.1
Archaea Thermoproteati Candidatus Bathyarchaeota Candidatus Bathyarchaeota archaeon MEJ2241062.1
Archaea Thermoproteati Candidatus Bathyarchaeota Candidatus Bathyarchaeota archaeon RLI31669.1
Archaea Thermoproteati Candidatus Bathyarchaeota Candidatus Bathyarchaeota archaeon RLI11121.1
Archaea Thermoproteati Candidatus Bathyarchaeota Candidatus Bathyarchaeota archaeon RLI06862.1
Archaea Thermoproteati Candidatus Bathyarchaeota Candidatus Bathyarchaeota archaeon RLI09911.1
Archaea Thermoproteati Candidatus Bathyarchaeota Candidatus Bathyarchaeota archaeon MCD6243469.1
Archaea Thermoproteati Candidatus Bathyarchaeota Candidatus Bathyarchaeota archaeon MCD6240717.1
Archaea Thermoproteati Candidatus Bathyarchaeota Candidatus Bathyarchaeota archaeon RLI21492.1
Archaea Thermoproteati Candidatus Bathyarchaeota Candidatus Bathyarchaeota archaeon HDI12231.1
Archaea Thermoproteati Candidatus Bathyarchaeota Candidatus Bathyarchaeota archaeon A05DMB-4 MBX5326194.1
Archaea Thermoproteati Candidatus Bathyarchaeota Candidatus Bathyarchaeia archaeon MEM2975710.1
Archaea Thermoproteati Candidatus Bathyarchaeota Candidatus Bathyarchaeia archaeon MEM3697022.1
Archaea Thermoproteati Candidatus Bathyarchaeota Candidatus Bathyarchaeia archaeon MEM3623479.1
Archaea Thermoproteati Candidatus Bathyarchaeota Candidatus Bathycorpusculaceae bacterium MGQ9507157.1
Archaea Thermoproteati Candidatus Bathyarchaeota Candidatus Bathyarchaeota archaeon MDH7564666.1
Archaea Thermoproteati Candidatus Bathyarchaeota Candidatus Bathyarchaeia archaeon MEM3730539.1
Archaea Thermoproteati Candidatus Bathyarchaeota Candidatus Bathyarchaeota archaeon MCK4474278.1
Archaea Thermoproteati Candidatus Bathyarchaeota Candidatus Bathyarchaeia archaeon MGD0979796.1
Archaea Thermoproteati Candidatus Bathyarchaeota Candidatus Bathyarchaeota archaeon MCJ7699518.1
Archaea Thermoproteati Candidatus Bathyarchaeota Candidatus Bathyarchaeota archaeon MCJ7767331.1
Archaea Thermoproteati Candidatus Bathyarchaeota Candidatus Bathyarchaeota archaeon MCJ7424036.1
Archaea Thermoproteati Candidatus Bathyarchaeota Candidatus Bathyarchaeota archaeon RBG 13 46 16b OGD47199.1
Archaea Thermoproteati Candidatus Bathyarchaeota Candidatus Bathyarchaeia archaeon MGA9387719.1
Archaea Thermoproteati Candidatus Bathyarchaeota Candidatus Bathyarchaeia archaeon MGD0029320.1
Archaea Thermoproteati Candidatus Bathyarchaeota Candidatus Bathyarchaeota archaeon A05DMB-5 MBX5329248.1
Archaea Thermoproteati Candidatus Bathyarchaeota Candidatus Bathyarchaeota archaeon MGD8565445.1
Archaea Thermoproteati Candidatus Bathyarchaeota Candidatus Bathyarchaeota archaeon MCD6431765.1
Archaea Thermoproteati Candidatus Bathyarchaeota Candidatus Bathyarchaeota archaeon RLI21212.1
Archaea Thermoproteati Candidatus Bathyarchaeota Candidatus Bathyarchaeota archaeon NWG10191.1
Archaea Thermoproteati Candidatus Bathyarchaeota Candidatus Bathyarchaeota archaeon NWF86445.1
Archaea Thermoproteati Candidatus Bathyarchaeota Candidatus Bathyarchaeota archaeon MDH5374749.1
Archaea Thermoproteati Candidatus Bathyarchaeota Candidatus Bathyarchaeia archaeon MFZ0965812.1
Archaea Thermoproteati Candidatus Bathyarchaeota Candidatus Bathyarchaeia archaeon MEM3626496.1
Archaea Thermoproteati Candidatus Bathyarchaeota Candidatus Bathycorpusculaceae bacterium MGQ9551986.1
Archaea Thermoproteati Candidatus Bathyarchaeota Candidatus Bathyarchaeia archaeon MEM3823903.1
Archaea Thermoproteati Candidatus Bathyarchaeota Candidatus Bathyarchaeales archaeon MGB9714675.1
Archaea Thermoproteati Candidatus Bathyarchaeota Candidatus Bathycorpusculaceae bacterium MGQ9641779.1
Archaea Thermoproteati Candidatus Bathyarchaeota Candidatus Bathyarchaeia archaeon MEM3055165.1
Archaea Thermoproteati Candidatus Bathyarchaeota Candidatus Bathyarchaeota archaeon MBS7616953.1
Archaea Thermoproteati Candidatus Bathyarchaeota Candidatus Bathyarchaeota archaeon MCS7095621.1
Archaea Thermoproteati Candidatus Bathyarchaeota Candidatus Bathyarchaeia archaeon MEM0313948.1
Archaea Thermoproteati Candidatus Bathyarchaeota Candidatus Bathyarchaeia archaeon MEM3549660.1
Archaea Thermoproteati Candidatus Bathyarchaeota Candidatus Bathyarchaeia archaeon MEM1660246.1
Archaea Thermoproteati Candidatus Bathyarchaeota Candidatus Bathyarchaeota archaeon MCS7115311.1
Archaea Thermoproteati Candidatus Bathyarchaeota Candidatus Bathyarchaeia archaeon MEM2393669.1
Archaea Thermoproteati Candidatus Bathyarchaeota Candidatus Bathyarchaeota archaeon A05DMB-3 MBX5321637.1
Archaea Thermoproteati Candidatus Bathyarchaeota Candidatus Bathycorpusculaceae bacterium MGQ9538543.1
Archaea Thermoproteati Candidatus Bathyarchaeota Candidatus Bathyarchaeia archaeon MEM2281836.1
Archaea Thermoproteati Candidatus Bathyarchaeota Candidatus Bathyarchaeia archaeon MEM2589413.1
Archaea Thermoproteati Candidatus Bathyarchaeota Candidatus Bathyarchaeota archaeon MBS7636267.1
Archaea Thermoproteati Candidatus Bathyarchaeota Candidatus Bathyarchaeia archaeon MEM1552215.1
Archaea Thermoproteati Candidatus Bathyarchaeota Candidatus Bathyarchaeia archaeon MEM3728351.1
Archaea Thermoproteati Candidatus Bathyarchaeota Candidatus Bathyarchaeia archaeon MGC8998719.1
Archaea Thermoproteati Candidatus Bathyarchaeota Candidatus Bathyarchaeota archaeon MCK5628456.1
Archaea Thermoproteati Candidatus Bathyarchaeota Candidatus Bathyarchaeota archaeon MCJ7561002.1
Archaea Thermoproteati Candidatus Bathyarchaeota Candidatus Bathyarchaeia archaeon HLE74620.1
Archaea Thermoproteati Candidatus Bathyarchaeota Candidatus Bathyarchaeota archaeon MCJ7714589.1
Archaea Thermoproteati Candidatus Bathyarchaeota Candidatus Bathyarchaeota archaeon TRO48449.1
Archaea Thermoproteati Candidatus Bathyarchaeota Candidatus Bathyarchaeota archaeon MCW3996951.1
Archaea Thermoproteati Candidatus Bathyarchaeota Candidatus Bathyarchaeia archaeon MEM2130809.1
Archaea Thermoproteati Candidatus Bathyarchaeota Candidatus Bathyarchaeota archaeon MCW4044878.1
Archaea Thermoproteati Candidatus Bathyarchaeota Candidatus Bathyarchaeia archaeon HEX9861951.1
Archaea Thermoproteati Candidatus Bathyarchaeota Candidatus Bathyarchaeota archaeon MCW4053779.1
Archaea Thermoproteati Candidatus Bathyarchaeota Candidatus Bathyarchaeota archaeon MCW4046426.1
Archaea Thermoproteati Candidatus Bathyarchaeota Candidatus Bathyarchaeota archaeon MBN1245779.1
Archaea Thermoproteati Candidatus Bathyarchaeota Candidatus Bathyarchaeota archaeon TRO53449.1
Archaea Thermoproteati Candidatus Bathyarchaeota Candidatus Bathyarchaeota archaeon TRO49516.1
Archaea Thermoproteati Candidatus Bathyarchaeota Candidatus Bathyarchaeota archaeon MBN1357671.1
Archaea Thermoproteati Candidatus Bathyarchaeota Candidatus Bathyarchaeia archaeon MEJ5327181.1
Archaea Thermoproteati Candidatus Bathyarchaeota Candidatus Bathyarchaeia archaeon MEM2081482.1
Archaea Thermoproteati Candidatus Bathyarchaeota Candidatus Bathyarchaeota archaeon MCX8153471.1
Archaea Thermoproteati Candidatus Bathyarchaeota Candidatus Bathyarchaeia archaeon MEM3378791.1
Archaea Thermoproteati Candidatus Bathyarchaeota Candidatus Bathyarchaeota archaeon HII85597.1
Archaea Thermoproteati Candidatus Bathyarchaeota Candidatus Bathyarchaeia archaeon HJW65315.1
Archaea Thermoproteati Candidatus Bathyarchaeota Candidatus Bathyarchaeia archaeon HLC00853.1
Archaea Thermoproteati Candidatus Bathyarchaeota Candidatus Bathyarchaeia archaeon HEX9262715.1
Archaea Thermoproteati Candidatus Bathyarchaeota Candidatus Bathyarchaeota archaeon MCX8151267.1
Archaea Thermoproteati Candidatus Bathyarchaeota Candidatus Bathyarchaeia archaeon MGF3522367.1
Archaea Thermoproteati Candidatus Bathyarchaeota Candidatus Bathyarchaeia archaeon MFB3889328.1
Archaea Thermoproteati Candidatus Bathyarchaeota Candidatus Bathyarchaeota archaeon MCW4009521.1
Archaea Thermoproteati Candidatus Bathyarchaeota Candidatus Bathyarchaeia archaeon XES78318.1
Archaea Thermoproteati Candidatus Bathyarchaeota Candidatus Bathyarchaeota archaeon MCW4005742.1
Archaea Thermoproteati Candidatus Bathyarchaeota Candidatus Bathyarchaeia archaeon MGA3112485.1
Archaea Thermoproteati Candidatus Bathyarchaeota Candidatus Bathyarchaeia archaeon MGO8806472.1
Archaea Thermoproteati Candidatus Bathyarchaeota Candidatus Bathyarchaeia archaeon MGD0450063.1
Archaea Thermoproteati Candidatus Bathyarchaeota Candidatus Bathyarchaeia archaeon MGD0645023.1
Archaea Thermoproteati Candidatus Bathyarchaeota Candidatus Bathyarchaeia archaeon MGD0994734.1
Archaea Thermoproteati Candidatus Bathyarchaeota Candidatus Bathyarchaeia archaeon MGA2682953.1
Archaea Thermoproteati Candidatus Bathyarchaeota Candidatus Bathyarchaeota archaeon MCW3996410.1
Archaea Thermoproteati Candidatus Bathyarchaeota Candidatus Bathyarchaeota archaeon MCW4028885.1
Archaea Thermoproteati Candidatus Bathyarchaeota Candidatus Bathycorpusculum sp. WP 343382266.1
Archaea Thermoproteati Candidatus Bathyarchaeota Candidatus Bathycorpusculum sp. WP 343381368.1
Archaea Thermoproteati Candidatus Bathyarchaeota Candidatus Bathyarchaeia archaeon MFA5571900.1
Archaea Thermoproteati Candidatus Bathyarchaeota Candidatus Bathyarchaeota archaeon MCL5877446.1
Archaea Thermoproteati Candidatus Bathyarchaeota Candidatus Bathycorpusculum termitum MDR0373276.1
Archaea Thermoproteati Candidatus Bathyarchaeota Candidatus Bathyarchaeota archaeon MCW4025405.1
Archaea Thermoproteati Candidatus Bathyarchaeota Candidatus Bathyarchaeota archaeon MCW4004848.1
Archaea Thermoproteati Candidatus Bathyarchaeota Candidatus Bathyarchaeia archaeon XHH09009.1
Archaea Thermoproteati Candidatus Bathyarchaeota Candidatus Bathyarchaeia archaeon MGD6809178.1
Archaea Thermoproteati Candidatus Bathyarchaeota Candidatus Bathycorpusculum sp. MCL1977328.1
Archaea Thermoproteati Candidatus Bathyarchaeota Candidatus Bathycorpusculum sp. MCL1966134.1
Archaea Thermoproteati Candidatus Bathyarchaeota Candidatus Bathycorpusculum sp. MCL2289013.1
Archaea Thermoproteati Candidatus Bathyarchaeota Candidatus Bathycorpusculum terrae MCL2691275.1
Archaea Thermoproteati Candidatus Bathyarchaeota Candidatus Bathycorpusculum sp. WP 343378962.1
Archaea Thermoproteati Candidatus Bathyarchaeota Candidatus Bathycorpusculum sp. MCL2643187.1
Archaea Thermoproteati Candidatus Bathyarchaeota Candidatus Bathycorpusculum soli MDR2203127.1
Archaea Thermoproteati Candidatus Bathyarchaeota Candidatus Bathycorpusculum grumuli MCL2135577.1
Archaea Promethearchaeati Candidatus Heimdallarchaeota Candidatus Heimdallarchaeota archaeon isolate Bin 120 k141 4304295 MBD3193020.1
Archaea Promethearchaeati Candidatus Heimdallarchaeota Candidatus Heimdallarchaeota archaeon MHA1837390.1
Archaea Promethearchaeati Candidatus Heimdallarchaeota Candidatus Heimdallarchaeota archaeon MHA1187090.1
Archaea Promethearchaeati Candidatus Heimdallarchaeota Candidatus Heimdallarchaeota archaeon NHJ04431.1
Archaea Promethearchaeati Candidatus Heimdallarchaeota Candidatus Heimdallarchaeota archaeon isolate CSMAG 2352 GCA 030614005.1
Archaea Promethearchaeati Candidatus Heimdallarchaeota Candidatus Heimdallarchaeota archaeon isolate 7H bin7 GCA 023705925.1
Archaea Promethearchaeati Candidatus Heimdallarchaeota Candidatus Heimdallarchaeota archaeon isolate HM7 B34 GCA 018238155.1
Archaea Promethearchaeati Candidatus Heimdallarchaeota Candidatus Heimdallarchaeota archaeon isolate CSMAG 879 GCA 030667035.1
Archaea Promethearchaeati Candidatus Heimdallarchaeota Candidatus Heimdallarchaeota archaeon isolate MAG 299 GCA 019057515.1
Archaea Promethearchaeati Candidatus Heimdallarchaeota Candidatus Heimdallarchaeota archaeon MHA1557445.1
Archaea Promethearchaeati Candidatus Gerdarchaeota Candidatus Gerdarchaeota archaeon isolate MP5 1 791 GCA 016840945.1
Archaea Promethearchaeati Candidatus Gerdarchaeota Candidatus Gerdarchaeota archaeon isolate FT2 001 GCA 016839405.1
Archaea Promethearchaeati Candidatus Heimdallarchaeota Candidatus Heimdallarchaeota archaeon MHA1221457.1
Archaea Promethearchaeati Candidatus Gerdarchaeota Candidatus Gerdarchaeota archaeon isolate MP5 2 2012 GCA 016840905.1
Archaea Promethearchaeati Candidatus Heimdallarchaeota Candidatus Heimdallarchaeota archaeon isolate CSMAG 1263 GCA 030602675.1
Archaea Promethearchaeati Candidatus Heimdallarchaeota Candidatus Heimdallarchaeota archaeon isolate 12R2 1 GCA 019058195.1
Archaea Promethearchaeati Candidatus Heimdallarchaeota Candidatus Heimdallarchaeota archaeon MGC9780490.1
Archaea Promethearchaeati Candidatus Heimdallarchaeota Candidatus Heimdallarchaeota archaeon isolate 14 8 m bin25 k141 1242130 length 75992 cov 72.6898 MBK5112993.1
Archaea Promethearchaeati Candidatus Heimdallarchaeota Candidatus Heimdallarchaeota archaeon isolate CSMAG 1338 GCA 030671945.1
Archaea Promethearchaeati Candidatus Heimdallarchaeota Candidatus Heimdallarchaeota archaeon MCK5045225.1
Archaea Promethearchaeati Candidatus Heimdallarchaeota Candidatus Heimdallarchaeota archaeon TET31305.1
Archaea Promethearchaeati Candidatus Thorarchaeota Candidatus Thorarchaeota archaeon isolate 12R2 15 GCA 019058235.1
Archaea Promethearchaeati Candidatus Heimdallarchaeota Candidatus Heimdallarchaeota archaeon isolate CSMAG 1555 GCA 030629105.1
Archaea Promethearchaeati Candidatus Heimdallarchaeota Candidatus Heimdallarchaeota archaeon MHA1121880.1
Archaea Promethearchaeati Candidatus Wukongarchaeota Candidatus Wukongarchaeum yapensis isolate Yap4.bin4.70 GCA 016840425.1
Archaea Promethearchaeati Candidatus Wukongarchaeota Candidatus Wukongarchaeota archaeon isolate Yap125.bin7.59 GCA 016840725.1
Archaea Promethearchaeati Candidatus Wukongarchaeota Candidatus Wukongarchaeota archaeon isolate WOR 133 k255 1289578 MDO8096767.1
Archaea Promethearchaeati Candidatus Wukongarchaeota Candidatus Wukongarchaeota archaeon isolate WOR 431 k255 3507499 MDO8130175.1
Archaea Promethearchaeati Promethearchaeati incertae sedis Candidatus Jordarchaeum sp. LHC 1308 isolate LHC 1308 GCA 019057675.1
Archaea Promethearchaeati Candidatus Freyarchaeota Candidatus Freyrarchaeum guaymaensis isolate GB 128 k255 313810 MDO8062243.1
Archaea Promethearchaeati Promethearchaeati incertae sedis Candidatus Jordarchaeia archaeon isolate RPF2 GCA 029856435.1
Archaea Promethearchaeati Promethearchaeati incertae sedis Candidatus Jordarchaeaceae archaeon MEM2133788.1
Archaea Promethearchaeati Promethearchaeati incertae sedis Candidatus Jordarchaeum sp. WP 445869335.1
Archaea Promethearchaeati Candidatus Freyarchaeota Candidatus Freyarchaeota archaeon isolate SQRJ 82 k255 467457 iso2 MDO8080843.1
Archaea Promethearchaeati Promethearchaeati incertae sedis Candidatus Jordarchaeaceae archaeon MEM3586469.1
Archaea Promethearchaeati Promethearchaeati incertae sedis Candidatus Jordarchaeaceae archaeon MEM2145702.1
Archaea Promethearchaeati Promethearchaeota Promethearchaeia archaeon MFB0560412.1
Archaea Promethearchaeati Candidatus Sigynarchaeota Candidatus Sigynarchaeota archaeon isolate SQRJ 179 k255 1871088 MDO8090017.1
Archaea Promethearchaeati Candidatus Freyarchaeota Candidatus Freyarchaeota archaeon isolate SQRJ 26 k255 270463 MDO8076617.1
Archaea Promethearchaeati Candidatus Freyarchaeota Candidatus Freyarchaeota archaeon MHA1580436.1
Archaea Promethearchaeati Candidatus Odinarchaeota Candidatus Odinarchaeia archaeon MHA1754503.1

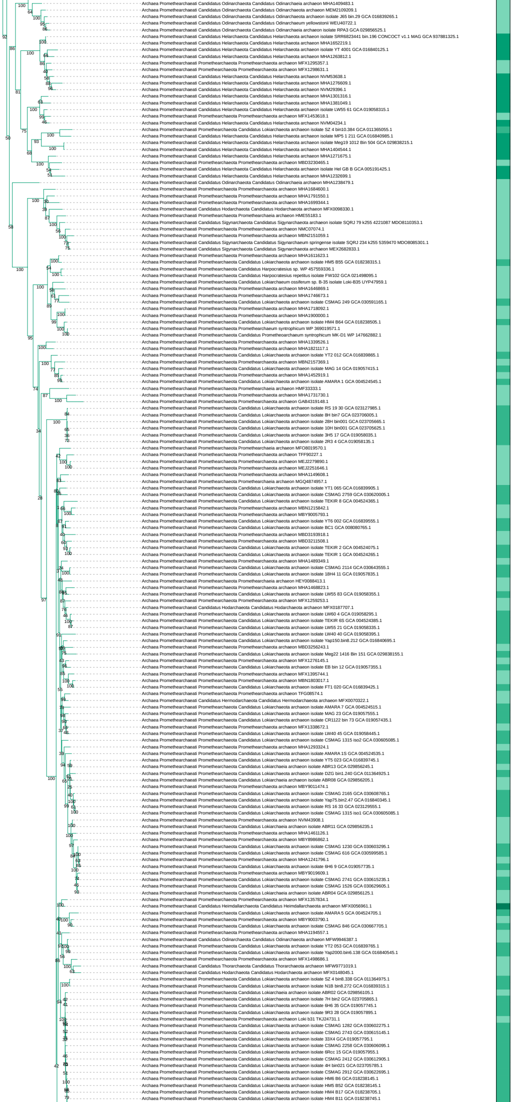
Archaea Promethearchaeati Candidatus Odinarchaeota Candidatus Odinarchaeia archaeon MHA1409483.1
Archaea Promethearchaeati Candidatus Odinarchaeota Candidatus Odinarchaeota archaeon MEM2109209.1
Archaea Promethearchaeati Candidatus Odinarchaeota Candidatus Odinarchaeota archaeon isolate J65 bin.29 GCA 016839265.1
Archaea Promethearchaeati Candidatus Odinarchaeota Candidatus Odinarchaeum yellowstonii WEU40722.1
Archaea Promethearchaeati Candidatus Odinarchaeota Candidatus Odinarchaeia archaeon isolate RPA3 GCA 029856525.1
Archaea Promethearchaeati Candidatus Helarchaeota Candidatus Helarchaeota archaeon isolate SRR6823441 bin.196 CONCOCT v1.1 MAG GCA 937881325.1
Archaea Promethearchaeati Candidatus Helarchaeota Candidatus Helarchaeota archaeon MHA1652219.1
Archaea Promethearchaeati Candidatus Helarchaeota Candidatus Helarchaeota archaeon isolate YT 4001 GCA 016840125.1
Archaea Promethearchaeati Candidatus Helarchaeota Candidatus Helarchaeota archaeon MHA1263812.1
Archaea Promethearchaeati Promethearchaeota Promethearchaeota archaeon MFX1295357.1
Archaea Promethearchaeati Promethearchaeota Promethearchaeota archaeon MFX1298631.1
Archaea Promethearchaeati Candidatus Helarchaeota Candidatus Helarchaeota archaeon NVM53638.1
Archaea Promethearchaeati Candidatus Helarchaeota Candidatus Helarchaeota archaeon MHA1276609.1
Archaea Promethearchaeati Candidatus Helarchaeota Candidatus Helarchaeota archaeon NVM29396.1
Archaea Promethearchaeati Candidatus Helarchaeota Candidatus Helarchaeota archaeon MHA1301316.1
Archaea Promethearchaeati Candidatus Helarchaeota Candidatus Helarchaeota archaeon MHA1381049.1
Archaea Promethearchaeati Candidatus Helarchaeota Candidatus Helarchaeota archaeon isolate LW55 61 GCA 019058315.1
Archaea Promethearchaeati Promethearchaeota Promethearchaeota archaeon MFX1453618.1
Archaea Promethearchaeati Candidatus Helarchaeota Candidatus Helarchaeota archaeon NVM04234.1
Archaea Promethearchaeati Promethearchaeota Candidatus Lokiarchaeota archaeon isolate SZ 4 bin10.384 GCA 011365055.1
Archaea Promethearchaeati Candidatus Helarchaeota Candidatus Helarchaeota archaeon isolate MP5 1 211 GCA 016840985.1
Archaea Promethearchaeati Candidatus Helarchaeota Candidatus Helarchaeota archaeon isolate Meg19 1012 Bin 504 GCA 029838215.1
Archaea Promethearchaeati Candidatus Helarchaeota Candidatus Helarchaeota archaeon MHA1404544.1
Archaea Promethearchaeati Candidatus Helarchaeota Candidatus Helarchaeota archaeon MHA1271675.1
Archaea Promethearchaeati Promethearchaeota Promethearchaeota archaeon MBD3230465.1
Archaea Promethearchaeati Candidatus Helarchaeota Candidatus Helarchaeota archaeon isolate Hel GB B GCA 005191425.1
Archaea Promethearchaeati Candidatus Helarchaeota Candidatus Helarchaeota archaeon MHA1232699.1
Archaea Promethearchaeati Candidatus Odinarchaeota Candidatus Odinarchaeia archaeon MHA1238479.1
Archaea Promethearchaeati Promethearchaeota Promethearchaeota archaeon MHA1684600.1
Archaea Promethearchaeati Promethearchaeota Promethearchaeota archaeon MHA1791550.1
Archaea Promethearchaeati Promethearchaeota Promethearchaeota archaeon MHA1699344.1
Archaea Promethearchaeati Candidatus Hodarchaeota Candidatus Hodarchaeota archaeon MFX0098330.1
Archaea Promethearchaeati Promethearchaeota Promethearchaeia archaeon HME55183.1
Archaea Promethearchaeati Candidatus Sigynarchaeota Candidatus Sigynarchaeota archaeon isolate SQRJ 79 k255 4221087 MDO8110353.1
Archaea Promethearchaeati Promethearchaeota Promethearchaeota archaeon NMC07074.1
Archaea Promethearchaeati Promethearchaeota Promethearchaeota archaeon MBN2151059.1
Archaea Promethearchaeati Candidatus Sigynarchaeota Candidatus Sigynarchaeum springense isolate SQRJ 234 k255 5359470 MDO8085301.1
Archaea Promethearchaeati Candidatus Sigynarchaeota Candidatus Sigynarchaeota archaeon MEX2682833.1
Archaea Promethearchaeati Promethearchaeota Promethearchaeota archaeon MHA1611623.1
Archaea Promethearchaeati Promethearchaeota Candidatus Lokiarchaeota archaeon isolate HM5 B55 GCA 018238315.1
Archaea Promethearchaeati Promethearchaeota Candidatus Harpocratesius sp. WP 457559336.1
Archaea Promethearchaeati Promethearchaeota Candidatus Harpocratesius repetitus isolate FW102 GCA 021498095.1
Archaea Promethearchaeati Promethearchaeota Candidatus Lokiarchaeum ossiferum sp. B-35 isolate Loki-B35 UYP47959.1
Archaea Promethearchaeati Promethearchaeota Promethearchaeota archaeon MHA1646869.1
Archaea Promethearchaeati Promethearchaeota Promethearchaeota archaeon MHA1746673.1
Archaea Promethearchaeati Promethearchaeota Candidatus Lokiarchaeota archaeon isolate CSMAG 249 GCA 030591165.1
Archaea Promethearchaeati Promethearchaeota Promethearchaeota archaeon MHA1718092.1
Archaea Promethearchaeati Promethearchaeota Promethearchaeota archaeon MHA1900000.1
Archaea Promethearchaeati Promethearchaeota Candidatus Lokiarchaeota archaeon isolate HM4 B64 GCA 018238505.1
Archaea Promethearchaeati Promethearchaeota Promethearchaeum syntrophicum WP 369019571.1
Archaea Promethearchaeati Promethearchaeota Candidatus Prometheoarchaeum syntrophicum MK-D1 WP 147662882.1
Archaea Promethearchaeati Promethearchaeota Promethearchaeota archaeon MHA1339526.1
Archaea Promethearchaeati Promethearchaeota Promethearchaeota archaeon MHA1821117.1
Archaea Promethearchaeati Promethearchaeota Candidatus Lokiarchaeota archaeon isolate YT2 012 GCA 016839865.1
Archaea Promethearchaeati Promethearchaeota Promethearchaeota archaeon MBN2157369.1
Archaea Promethearchaeati Promethearchaeota Candidatus Lokiarchaeota archaeon isolate MAG 14 GCA 019057415.1
Archaea Promethearchaeati Promethearchaeota Promethearchaeota archaeon MHA1452919.1
Archaea Promethearchaeati Promethearchaeota Candidatus Lokiarchaeota archaeon isolate AMARA 1 GCA 004524545.1
Archaea Promethearchaeati Promethearchaeota Promethearchaeia archaeon HMF33333.1
Archaea Promethearchaeati Promethearchaeota Promethearchaeota archaeon MHA1731730.1
Archaea Promethearchaeati Promethearchaeota Promethearchaeota archaeon GAB4319148.1
Archaea Promethearchaeati Promethearchaeota Candidatus Lokiarchaeota archaeon isolate RS 19 30 GCA 023127985.1
Archaea Promethearchaeati Promethearchaeota Candidatus Lokiarchaeota archaeon isolate 8H bin7 GCA 023706005.1
Archaea Promethearchaeati Promethearchaeota Candidatus Lokiarchaeota archaeon isolate 28H bin001 GCA 023705665.1
Archaea Promethearchaeati Promethearchaeota Candidatus Lokiarchaeota archaeon isolate 10H bin001 GCA 023705625.1
Archaea Promethearchaeati Promethearchaeota Candidatus Lokiarchaeota archaeon isolate 3H5 17 GCA 019058035.1
Archaea Promethearchaeati Promethearchaeota Candidatus Lokiarchaeota archaeon isolate 2R3 4 GCA 019058135.1
Archaea Promethearchaeati Promethearchaeota Promethearchaeia archaeon MFO8019570.1
Archaea Promethearchaeati Promethearchaeota Promethearchaeota archaeon TFF90227.1
Archaea Promethearchaeati Promethearchaeota Promethearchaeota archaeon MEJ2279890.1
Archaea Promethearchaeati Promethearchaeota Promethearchaeota archaeon MEJ2251646.1
Archaea Promethearchaeati Promethearchaeota Promethearchaeota archaeon MHA1149608.1
Archaea Promethearchaeati Promethearchaeota Promethearchaeia archaeon MGQ4874957.1
Archaea Promethearchaeati Promethearchaeota Candidatus Lokiarchaeota archaeon isolate YT1 065 GCA 016839905.1
Archaea Promethearchaeati Promethearchaeota Candidatus Lokiarchaeota archaeon isolate CSMAG 2759 GCA 030620005.1
Archaea Promethearchaeati Promethearchaeota Candidatus Lokiarchaeota archaeon isolate TEKIR 8 GCA 004524365.1
Archaea Promethearchaeati Promethearchaeota Promethearchaeota archaeon MBN1215842.1
Archaea Promethearchaeati Promethearchaeota Promethearchaeota archaeon MBY9005793.1
Archaea Promethearchaeati Promethearchaeota Candidatus Lokiarchaeota archaeon isolate YT6 002 GCA 016839555.1
Archaea Promethearchaeati Promethearchaeota Candidatus Lokiarchaeota archaeon isolate BC1 GCA 008080765.1
Archaea Promethearchaeati Promethearchaeota Promethearchaeota archaeon MBD3193918.1
Archaea Promethearchaeati Promethearchaeota Promethearchaeota archaeon MBD3211508.1
Archaea Promethearchaeati Promethearchaeota Candidatus Lokiarchaeota archaeon isolate TEKIR 2 GCA 004524075.1
Archaea Promethearchaeati Promethearchaeota Candidatus Lokiarchaeota archaeon isolate TEKIR 1 GCA 004524265.1
Archaea Promethearchaeati Promethearchaeota Promethearchaeota archaeon MHA1489349.1
Archaea Promethearchaeati Promethearchaeota Candidatus Lokiarchaeota archaeon isolate CSMAG 2114 GCA 030643555.1
Archaea Promethearchaeati Promethearchaeota Candidatus Lokiarchaeota archaeon isolate 18H4 11 GCA 019057835.1
Archaea Promethearchaeati Promethearchaeota Promethearchaeia archaeon HEY0088413.1
Archaea Promethearchaeati Promethearchaeota Promethearchaeota archaeon MHA1468823.1
Archaea Promethearchaeati Promethearchaeota Candidatus Lokiarchaeota archaeon isolate LW55 83 GCA 019058355.1
Archaea Promethearchaeati Promethearchaeota Promethearchaeota archaeon MFX1259253.1
Archaea Promethearchaeati Candidatus Hodarchaeota Candidatus Hodarchaeota archaeon MFX0187707.1
Archaea Promethearchaeati Promethearchaeota Candidatus Lokiarchaeota archaeon isolate LW60 4 GCA 019058295.1
Archaea Promethearchaeati Promethearchaeota Candidatus Lokiarchaeota archaeon isolate TEKIR 6S GCA 004524385.1
Archaea Promethearchaeati Promethearchaeota Candidatus Lokiarchaeota archaeon isolate LW55 21 GCA 019058335.1
Archaea Promethearchaeati Promethearchaeota Candidatus Lokiarchaeota archaeon isolate LW40 40 GCA 019058395.1
Archaea Promethearchaeati Promethearchaeota Candidatus Lokiarchaeota archaeon isolate Yap150.bin8.212 GCA 016840695.1
Archaea Promethearchaeati Promethearchaeota Promethearchaeota archaeon MBD3256243.1
Archaea Promethearchaeati Promethearchaeota Candidatus Lokiarchaeota archaeon isolate Meg22 1416 Bin 151 GCA 029838155.1
Archaea Promethearchaeati Promethearchaeota Promethearchaeota archaeon MFX1276145.1
Archaea Promethearchaeati Promethearchaeota Candidatus Lokiarchaeota archaeon isolate EB bin 12 GCA 019057355.1
Archaea Promethearchaeati Promethearchaeota Promethearchaeota archaeon MFX1395744.1
Archaea Promethearchaeati Promethearchaeota Promethearchaeota archaeon MBN1803017.1
Archaea Promethearchaeati Promethearchaeota Candidatus Lokiarchaeota archaeon isolate FT1 020 GCA 016839425.1
Archaea Promethearchaeati Promethearchaeota Promethearchaeota archaeon TFG08574.1
Archaea Promethearchaeati Candidatus Hermodarchaeota Candidatus Hermodarchaeota archaeon MFX0070322.1
Archaea Promethearchaeati Promethearchaeota Candidatus Lokiarchaeota archaeon isolate AMARA 7 GCA 004524515.1
Archaea Promethearchaeati Promethearchaeota Candidatus Lokiarchaeota archaeon isolate MAG 23 GCA 019057555.1
Archaea Promethearchaeati Promethearchaeota Candidatus Lokiarchaeota archaeon isolate CR1122 bin 73 GCA 019057435.1
Archaea Promethearchaeati Promethearchaeota Promethearchaeota archaeon MFX1338672.1
Archaea Promethearchaeati Promethearchaeota Candidatus Lokiarchaeota archaeon isolate LW40 45 GCA 019058445.1
Archaea Promethearchaeati Promethearchaeota Candidatus Lokiarchaeota archaeon isolate CSMAG 1315 iso2 GCA 030605085.1
Archaea Promethearchaeati Promethearchaeota Promethearchaeota archaeon MHA1293324.1
Archaea Promethearchaeati Promethearchaeota Candidatus Lokiarchaeota archaeon isolate AMARA 1S GCA 004524535.1
Archaea Promethearchaeati Promethearchaeota Candidatus Lokiarchaeota archaeon isolate YT5 023 GCA 016839745.1
Archaea Promethearchaeati Promethearchaeota Candidatus Lokiarchaeia archaeon isolate ABR13 GCA 029856245.1
Archaea Promethearchaeati Promethearchaeota Candidatus Lokiarchaeota archaeon isolate DZG bin1.240 GCA 011364925.1
Archaea Promethearchaeati Promethearchaeota Candidatus Lokiarchaeia archaeon isolate ABR08 GCA 029856205.1
Archaea Promethearchaeati Promethearchaeota Promethearchaeota archaeon MBY9011474.1
Archaea Promethearchaeati Promethearchaeota Candidatus Lokiarchaeota archaeon isolate CSMAG 2165 GCA 030608765.1
Archaea Promethearchaeati Promethearchaeota Candidatus Lokiarchaeota archaeon isolate Yap75.bin2.47 GCA 016840345.1
Archaea Promethearchaeati Promethearchaeota Candidatus Lokiarchaeota archaeon isolate RS 16 33 GCA 023129555.1
Archaea Promethearchaeati Promethearchaeota Candidatus Lokiarchaeota archaeon isolate CSMAG 1315 iso1 GCA 030605085.1
Archaea Promethearchaeati Promethearchaeota Promethearchaeota archaeon NVM43908.1
Archaea Promethearchaeati Promethearchaeota Candidatus Lokiarchaeia archaeon isolate ABR11 GCA 029856235.1
Archaea Promethearchaeati Promethearchaeota Promethearchaeota archaeon MHA1461126.1
Archaea Promethearchaeati Promethearchaeota Promethearchaeota archaeon MBY8986862.1
Archaea Promethearchaeati Promethearchaeota Candidatus Lokiarchaeota archaeon isolate CSMAG 1230 GCA 030603295.1
Archaea Promethearchaeati Promethearchaeota Candidatus Lokiarchaeota archaeon isolate CSMAG 616 GCA 030599585.1
Archaea Promethearchaeati Promethearchaeota Promethearchaeota archaeon MHA1241796.1
Archaea Promethearchaeati Promethearchaeota Candidatus Lokiarchaeota archaeon isolate 6H6 9 GCA 019057735.1
Archaea Promethearchaeati Promethearchaeota Promethearchaeota archaeon MBY9019609.1
Archaea Promethearchaeati Promethearchaeota Candidatus Lokiarchaeota archaeon isolate CSMAG 2741 GCA 030615235.1
Archaea Promethearchaeati Promethearchaeota Candidatus Lokiarchaeota archaeon isolate CSMAG 1526 GCA 030629605.1
Archaea Promethearchaeati Promethearchaeota Candidatus Lokiarchaeia archaeon isolate ABR04 GCA 029856125.1
Archaea Promethearchaeati Promethearchaeota Promethearchaeota archaeon MFX1357834.1
Archaea Promethearchaeati Candidatus Heimdallarchaeota Candidatus Heimdallarchaeota archaeon MFX0056961.1
Archaea Promethearchaeati Promethearchaeota Candidatus Lokiarchaeota archaeon isolate AMARA 5 GCA 004524705.1
Archaea Promethearchaeati Promethearchaeota Promethearchaeota archaeon MBY9003790.1
Archaea Promethearchaeati Promethearchaeota Candidatus Lokiarchaeota archaeon isolate CSMAG 846 GCA 030667705.1
Archaea Promethearchaeati Promethearchaeota Promethearchaeota archaeon MHA1194557.1
Archaea Promethearchaeati Candidatus Odinarchaeota Candidatus Odinarchaeota archaeon MFW9946387.1
Archaea Promethearchaeati Promethearchaeota Candidatus Lokiarchaeota archaeon isolate YT2 053 GCA 016839765.1
Archaea Promethearchaeati Promethearchaeota Candidatus Lokiarchaeota archaeon isolate Yap2000.bin6.138 GCA 016840545.1
Archaea Promethearchaeati Promethearchaeota Promethearchaeota archaeon MFX1498686.1
Archaea Promethearchaeati Candidatus Thorarchaeota Candidatus Thorarchaeota archaeon MFW9771019.1
Archaea Promethearchaeati Candidatus Hodarchaeota Candidatus Hodarchaeota archaeon MFX0148045.1
Archaea Promethearchaeati Promethearchaeota Candidatus Lokiarchaeota archaeon isolate SZ 4 bin8.338 GCA 011364975.1
Archaea Promethearchaeati Promethearchaeota Candidatus Lokiarchaeota archaeon isolate N1B bin8.272 GCA 016839315.1
Archaea Promethearchaeati Promethearchaeota Candidatus Lokiarchaeia archaeon isolate ABR02 GCA 029856105.1
Archaea Promethearchaeati Promethearchaeota Candidatus Lokiarchaeota archaeon isolate 7H bin2 GCA 023705865.1
Archaea Promethearchaeati Promethearchaeota Candidatus Lokiarchaeota archaeon isolate 6H6 35 GCA 019057745.1
Archaea Promethearchaeati Promethearchaeota Candidatus Lokiarchaeota archaeon isolate 9R3 28 GCA 019057895.1
Archaea Promethearchaeati Promethearchaeota Promethearchaeota archaeon Loki b31 TKJ24731.1
Archaea Promethearchaeati Promethearchaeota Candidatus Lokiarchaeota archaeon isolate CSMAG 1282 GCA 030602275.1
Archaea Promethearchaeati Promethearchaeota Candidatus Lokiarchaeota archaeon isolate CSMAG 2743 GCA 030615145.1
Archaea Promethearchaeati Promethearchaeota Candidatus Lokiarchaeota archaeon isolate 33X4 GCA 019057795.1
Archaea Promethearchaeati Promethearchaeota Candidatus Lokiarchaeota archaeon isolate CSMAG 2258 GCA 030606095.1
Archaea Promethearchaeati Promethearchaeota Candidatus Lokiarchaeota archaeon isolate 6Rcc 15 GCA 019057955.1
Archaea Promethearchaeati Promethearchaeota Candidatus Lokiarchaeota archaeon isolate CSMAG 2412 GCA 030612905.1
Archaea Promethearchaeati Promethearchaeota Candidatus Lokiarchaeota archaeon isolate 4H bin021 GCA 023705785.1
Archaea Promethearchaeati Promethearchaeota Candidatus Lokiarchaeota archaeon isolate CSMAG 2912 GCA 030622695.1
Archaea Promethearchaeati Promethearchaeota Candidatus Lokiarchaeota archaeon isolate HM6 B6 GCA 018238145.1
Archaea Promethearchaeati Promethearchaeota Candidatus Lokiarchaeota archaeon isolate HM5 B52 GCA 018238145.1
Archaea Promethearchaeati Promethearchaeota Candidatus Lokiarchaeota archaeon isolate HM4 B17 GCA 018238705.1
Archaea Promethearchaeati Promethearchaeota Candidatus Lokiarchaeota archaeon isolate HM4 B11 GCA 018238745.1

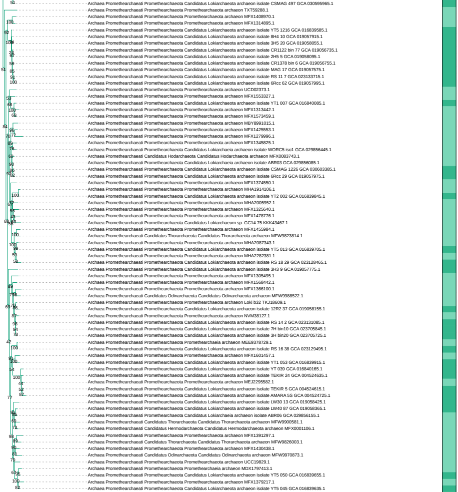
Archaea Promethearchaeati Promethearchaeota Candidatus Lokiarchaeota archaeon isolate CSMAG 497 GCA 030595965.1
Archaea Promethearchaeati Promethearchaeota Promethearchaeota archaeon TXT59288.1
Archaea Promethearchaeati Promethearchaeota Promethearchaeota archaeon MFX1408970.1
Archaea Promethearchaeati Promethearchaeota Promethearchaeota archaeon MFX1314895.1
Archaea Promethearchaeati Promethearchaeota Candidatus Lokiarchaeota archaeon isolate YT5 1216 GCA 016839585.1
Archaea Promethearchaeati Promethearchaeota Candidatus Lokiarchaeota archaeon isolate 8H4 10 GCA 019057915.1
Archaea Promethearchaeati Promethearchaeota Candidatus Lokiarchaeota archaeon isolate 3H5 20 GCA 019058055.1
Archaea Promethearchaeati Promethearchaeota Candidatus Lokiarchaeota archaeon isolate CR1122 bin 77 GCA 019056735.1
Archaea Promethearchaeati Promethearchaeota Candidatus Lokiarchaeota archaeon isolate 2H5 5 GCA 019058095.1
Archaea Promethearchaeati Promethearchaeota Candidatus Lokiarchaeota archaeon isolate CR1378 bin 6 GCA 019056755.1
Archaea Promethearchaeati Promethearchaeota Candidatus Lokiarchaeota archaeon isolate MAG 17 GCA 019057575.1
Archaea Promethearchaeati Promethearchaeota Candidatus Lokiarchaeota archaeon isolate RS 11 7 GCA 023133715.1
Archaea Promethearchaeati Promethearchaeota Candidatus Lokiarchaeota archaeon isolate 6Rcc 62 GCA 019057995.1
Archaea Promethearchaeati Promethearchaeota Promethearchaeota archaeon UCD02373.1
Archaea Promethearchaeati Promethearchaeota Promethearchaeota archaeon MFX1553327.1
Archaea Promethearchaeati Promethearchaeota Candidatus Lokiarchaeota archaeon isolate YT1 007 GCA 016840085.1
Archaea Promethearchaeati Promethearchaeota Promethearchaeota archaeon MFX1313442.1
Archaea Promethearchaeati Promethearchaeota Promethearchaeota archaeon MFX1573459.1
Archaea Promethearchaeati Promethearchaeota Promethearchaeota archaeon MBY8991015.1
Archaea Promethearchaeati Promethearchaeota Promethearchaeota archaeon MFX1425553.1
Archaea Promethearchaeati Promethearchaeota Promethearchaeota archaeon MFX1279996.1
Archaea Promethearchaeati Promethearchaeota Promethearchaeota archaeon MFX1345825.1
Archaea Promethearchaeati Promethearchaeota Candidatus Lokiarchaeia archaeon isolate WORC5 iso1 GCA 029856445.1
Archaea Promethearchaeati Candidatus Hodarchaeota Candidatus Hodarchaeota archaeon MFX0083743.1
Archaea Promethearchaeati Promethearchaeota Candidatus Lokiarchaeia archaeon isolate ABR03 GCA 029856085.1
Archaea Promethearchaeati Promethearchaeota Candidatus Lokiarchaeota archaeon isolate CSMAG 1226 GCA 030603385.1
Archaea Promethearchaeati Promethearchaeota Candidatus Lokiarchaeota archaeon isolate 6Rcc 29 GCA 019057975.1
Archaea Promethearchaeati Promethearchaeota Promethearchaeota archaeon MFX1374550.1
Archaea Promethearchaeati Promethearchaeota Promethearchaeota archaeon MHA1914106.1
Archaea Promethearchaeati Promethearchaeota Candidatus Lokiarchaeota archaeon isolate YT2 002 GCA 016839845.1
Archaea Promethearchaeati Promethearchaeota Promethearchaeota archaeon MHA2005952.1
Archaea Promethearchaeati Promethearchaeota Promethearchaeota archaeon MFX1325640.1
Archaea Promethearchaeati Promethearchaeota Promethearchaeota archaeon MFX1478776.1
Archaea Promethearchaeati Promethearchaeota Candidatus Lokiarchaeum sp. GC14 75 KKK43467.1
Archaea Promethearchaeati Promethearchaeota Promethearchaeota archaeon MFX1455984.1
Archaea Promethearchaeati Candidatus Thorarchaeota Candidatus Thorarchaeota archaeon MFW9823814.1
Archaea Promethearchaeati Promethearchaeota Promethearchaeota archaeon MHA2087343.1
Archaea Promethearchaeati Promethearchaeota Candidatus Lokiarchaeota archaeon isolate YT5 013 GCA 016839705.1
Archaea Promethearchaeati Promethearchaeota Promethearchaeota archaeon MHA2282381.1
Archaea Promethearchaeati Promethearchaeota Candidatus Lokiarchaeota archaeon isolate RS 18 29 GCA 023128465.1
Archaea Promethearchaeati Promethearchaeota Candidatus Lokiarchaeota archaeon isolate 3H3 9 GCA 019057775.1
Archaea Promethearchaeati Promethearchaeota Promethearchaeota archaeon MFX1305495.1
Archaea Promethearchaeati Promethearchaeota Promethearchaeota archaeon MFX1568442.1
Archaea Promethearchaeati Promethearchaeota Promethearchaeota archaeon MFX1366100.1
Archaea Promethearchaeati Candidatus Odinarchaeota Candidatus Odinarchaeota archaeon MFW9988522.1
Archaea Promethearchaeati Promethearchaeota Promethearchaeota archaeon Loki b32 TKJ18609.1
Archaea Promethearchaeati Promethearchaeota Candidatus Lokiarchaeota archaeon isolate 12R2 37 GCA 019058155.1
Archaea Promethearchaeati Promethearchaeota Promethearchaeota archaeon NVM38127.1
Archaea Promethearchaeati Promethearchaeota Candidatus Lokiarchaeota archaeon isolate RS 14 2 GCA 023131085.1
Archaea Promethearchaeati Promethearchaeota Candidatus Lokiarchaeota archaeon isolate 7H bin10 GCA 023705845.1
Archaea Promethearchaeati Promethearchaeota Candidatus Lokiarchaeota archaeon isolate 3H bin20 GCA 023705725.1
Archaea Promethearchaeati Promethearchaeota Promethearchaeia archaeon MEE9378729.1
Archaea Promethearchaeati Promethearchaeota Candidatus Lokiarchaeota archaeon isolate RS 16 38 GCA 023129495.1
Archaea Promethearchaeati Promethearchaeota Promethearchaeota archaeon MFX1601457.1
Archaea Promethearchaeati Promethearchaeota Candidatus Lokiarchaeota archaeon isolate YT1 053 GCA 016839915.1
Archaea Promethearchaeati Promethearchaeota Candidatus Lokiarchaeota archaeon isolate YT 039 GCA 016840165.1
Archaea Promethearchaeati Promethearchaeota Candidatus Lokiarchaeota archaeon isolate TEKIR 24 GCA 004524635.1
Archaea Promethearchaeati Promethearchaeota Promethearchaeota archaeon MEJ2295582.1
Archaea Promethearchaeati Promethearchaeota Candidatus Lokiarchaeota archaeon isolate TEKIR 5 GCA 004524615.1
Archaea Promethearchaeati Promethearchaeota Candidatus Lokiarchaeota archaeon isolate AMARA 5S GCA 004524725.1
Archaea Promethearchaeati Promethearchaeota Candidatus Lokiarchaeota archaeon isolate LW30 13 GCA 019058425.1
Archaea Promethearchaeati Promethearchaeota Candidatus Lokiarchaeota archaeon isolate LW40 87 GCA 019058365.1
Archaea Promethearchaeati Promethearchaeota Candidatus Lokiarchaeia archaeon isolate ABR06 GCA 029856155.1
Archaea Promethearchaeati Candidatus Thorarchaeota Candidatus Thorarchaeota archaeon MFW9900581.1
Archaea Promethearchaeati Candidatus Hermodarchaeota Candidatus Hermodarchaeota archaeon MFX0001106.1
Archaea Promethearchaeati Promethearchaeota Promethearchaeota archaeon MFX1391297.1
Archaea Promethearchaeati Candidatus Thorarchaeota Candidatus Thorarchaeota archaeon MFW9826003.1
Archaea Promethearchaeati Promethearchaeota Promethearchaeota archaeon MFX1430438.1
Archaea Promethearchaeati Candidatus Odinarchaeota Candidatus Odinarchaeota archaeon MFW9970873.1
Archaea Promethearchaeati Promethearchaeota Promethearchaeota archaeon UCC19829.1
Archaea Promethearchaeati Promethearchaeota Promethearchaeia archaeon MDX1797413.1
Archaea Promethearchaeati Promethearchaeota Candidatus Lokiarchaeota archaeon isolate YT5 050 GCA 016839655.1
Archaea Promethearchaeati Promethearchaeota Promethearchaeota archaeon MFX1379217.1
Archaea Promethearchaeati Promethearchaeota Candidatus Lokiarchaeota archaeon isolate YT5 045 GCA 016839635.1
