# Supplementary material for: Divergent evolution of the PRPS enzymes across the tree of life
Source: bioRxiv. 2026 Jun 3:2026.06.01.728777. Preprint. [Version 1] doi: 10.64898/2026.06.01.728777 (PMC13252144; doi:10.64898/2026.06.01.728777)

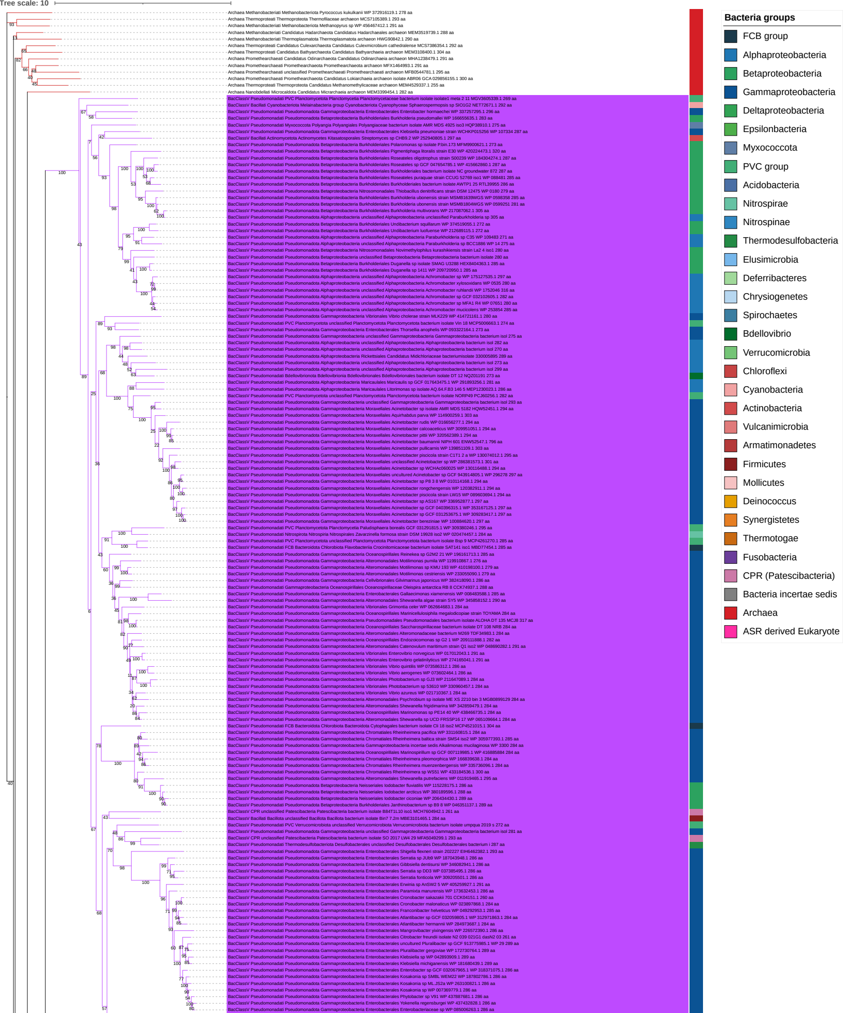

Tree scale: 10
Archaea Methanobacteriati Methanobacteriota Pyrococcus kukulkanii WP 372916119.1 278 aa
Archaea Thermoproteati Thermoproteota Thermofilaceae archaeon MCS7105389.1 293 aa
Archaea Methanobacteriati Methanobacteriota Methanopyrus sp WP 456467412.1 291 aa
Archaea Methanobacteriati Candidatus Hadarchaeota Candidatus Hadarchaeales archaeon MEM3519739.1 288 aa
Archaea Methanobacteriati Thermoplasmatota Thermoplasmatota archaeon HWG90842.1 290 aa
Archaea Thermoproteati Candidatus Culexarchaeota Candidatus Culexmicrobium cathedralense MCS7386354.1 292 aa
Archaea Thermoproteati Candidatus Bathyarchaeota Candidatus Bathyarchaeia archaeon MEM3108400.1 304 aa
Archaea Promethearchaeati Candidatus Odinarchaeota Candidatus Odinarchaeia archaeon MHA1238479.1 291 aa
Archaea Promethearchaeati Promethearchaeota Promethearchaeota archaeon MFX1464993.1 291 aa
Archaea Promethearchaeati unclassified Promethearchaeati archaeon MFB0544781.1 295 aa
Archaea Promethearchaeati Promethearchaeota Candidatus Lokiarchaeia archaeon isolate ABR06 GCA 029856155.1 300 aa
Archaea Thermoproteati Thermoproteota Candidatus Methanomethylicaceae archaeon MEM4529337.1 255 aa
Archaea Nanobdellati Microcaldota Candidatus Micrarchaeia archaeon MEM3399454.1 282 aa
BacClassV Pseudomonadati PVC Planctomycetota Planctomycetia Planctomycetaceae bacterium isolate isolate1 meta 2 11 MGV3605339.1 269 aa
BacClassV Bacillati Cyanobacteriota Melainabacteria group Cyanobacteriota Cyanophyceae Sphaerospermopsis sp SIO1G2 NET72671.1 292 aa
BacClassV Pseudomonadati Pseudomonadota Gammaproteobacteria Enterobacterales Enterobacter hormaechei WP 337257295.1 296 aa
BacClassV Pseudomonadati Pseudomonadota Betaproteobacteria Burkholderiales Burkholderia pseudomallei WP 166655635.1 283 aa
BacClassV Pseudomonadati Myxococcota Polyangia Polyangiales Polyangiaceae bacterium isolate AMR MDS 4925 iso3 HQP38910.1 275 aa
BacClassV Pseudomonadati Pseudomonadota Gammaproteobacteria Enterobacterales Klebsiella pneumoniae strain WCHKP015256 WP 107334 287 aa
BacClassV Bacillati Actinomycetota Actinomycetes Kitasatosporales Streptomyces sp CHB9.2 WP 252940805.1 297 aa
BacClassV Pseudomonadati Pseudomonadota Betaproteobacteria Burkholderiales Polaromonas sp isolate P.bin.173 MFM9900621.1 273 aa
BacClassV Pseudomonadati Pseudomonadota Betaproteobacteria Burkholderiales Pigmentiphaga litoralis strain E30 WP 420224473.1 320 aa
BacClassV Pseudomonadati Pseudomonadota Betaproteobacteria Burkholderiales Roseateles oligotrophus strain S00239 WP 184304274.1 287 aa
BacClassV Pseudomonadati Pseudomonadota Betaproteobacteria Burkholderiales Roseateles sp GCF 047654785.1 WP 415662860.1 287 aa
BacClassV Pseudomonadati Pseudomonadota Betaproteobacteria Burkholderiales Burkholderiales bacterium isolate NC groundwater 872 287 aa
BacClassV Pseudomonadati Pseudomonadota Betaproteobacteria Burkholderiales Roseateles puraquae strain CCUG 52769 iso1 WP 088481 285 aa
BacClassV Pseudomonadati Pseudomonadota Betaproteobacteria Burkholderiales Burkholderiales bacterium isolate AWTP1 25 RTL39955 286 aa
BacClassV Pseudomonadati Pseudomonadota Betaproteobacteria Nitrosomonadales Thiobacillus denitrificans strain DSM 12475 WP 0180 279 aa
BacClassV Pseudomonadati Pseudomonadota Betaproteobacteria Burkholderiales Burkholderia ubonensis strain MSMB1639WGS WP 0598358 285 aa
BacClassV Pseudomonadati Pseudomonadota Betaproteobacteria Burkholderiales Burkholderia ubonensis strain MSMB1804WGS WP 0599251 281 aa
BacClassV Pseudomonadati Pseudomonadota Betaproteobacteria Burkholderiales Burkholderia multivorans WP 217087062.1 305 aa
BacClassV Pseudomonadati Pseudomonadota Alphaproteobacteria unclassified Alphaproteobacteria unclassified Paraburkholderia sp 305 aa
BacClassV Pseudomonadati Pseudomonadota Betaproteobacteria Burkholderiales Undibacterium squillarum WP 374519055.1 272 aa
BacClassV Pseudomonadati Pseudomonadota Betaproteobacteria Burkholderiales Undibacterium luofuense WP 212689115.1 272 aa
BacClassV Pseudomonadati Pseudomonadota Alphaproteobacteria unclassified Alphaproteobacteria Paraburkholderia sp C35 WP 109483 271 aa
BacClassV Pseudomonadati Pseudomonadota Alphaproteobacteria unclassified Alphaproteobacteria Paraburkholderia sp BCC1886 WP 14 275 aa
BacClassV Pseudomonadati Pseudomonadota Betaproteobacteria Nitrosomonadales Novimethylophilus kurashikiensis strain La2 4 iso1 280 aa
BacClassV Pseudomonadati Pseudomonadota Betaproteobacteria unclassified Betaproteobacteria Betaproteobacteria bacterium isolate 280 aa
BacClassV Pseudomonadati Pseudomonadota Betaproteobacteria Burkholderiales Duganella sp isolate SMAG U3288 HEX8404363.1 285 aa
BacClassV Pseudomonadati Pseudomonadota Betaproteobacteria Burkholderiales Duganella sp 1411 WP 209720950.1 285 aa
BacClassV Pseudomonadati Pseudomonadota Alphaproteobacteria unclassified Alphaproteobacteria Achromobacter sp WP 175127535.1 297 aa
BacClassV Pseudomonadati Pseudomonadota Alphaproteobacteria unclassified Alphaproteobacteria Achromobacter xylosoxidans WP 0535 280 aa
BacClassV Pseudomonadati Pseudomonadota Alphaproteobacteria unclassified Alphaproteobacteria Achromobacter ruhlandii WP 1752046 316 aa
BacClassV Pseudomonadati Pseudomonadota Alphaproteobacteria unclassified Alphaproteobacteria Achromobacter sp GCF 032102605.1 282 aa
BacClassV Pseudomonadati Pseudomonadota Alphaproteobacteria unclassified Alphaproteobacteria Achromobacter sp MFA1 R4 WP 07651 280 aa
BacClassV Pseudomonadati Pseudomonadota Alphaproteobacteria unclassified Alphaproteobacteria Achromobacter mucicolens WP 253854 285 aa
BacClassV Pseudomonadati Pseudomonadota Gammaproteobacteria Vibrionales Vibrio cholerae strain MLK229 WP 414721161.1 280 aa
BacClassV Pseudomonadati PVC Planctomycetota unclassified Planctomycetota Planctomycetota bacterium isolate Vin 18 MCP5006663.1 274 aa
BacClassV Pseudomonadati Pseudomonadota Gammaproteobacteria Enterobacterales Thorsellia anophelis WP 093322164.1 273 aa
BacClassV Pseudomonadati Pseudomonadota Gammaproteobacteria unclassified Gammaproteobacteria Gammaproteobacteria bacterium isol 275 aa
BacClassV Pseudomonadati Pseudomonadota Alphaproteobacteria unclassified Alphaproteobacteria Alphaproteobacteria bacterium isol 282 aa
BacClassV Pseudomonadati Pseudomonadota Alphaproteobacteria unclassified Alphaproteobacteria Alphaproteobacteria bacterium isol 270 aa
BacClassV Pseudomonadati Pseudomonadota Alphaproteobacteria Rickettsiales Candidatus Midichloriaceae bacteriumisolate 330005895 289 aa
BacClassV Pseudomonadati Pseudomonadota Alphaproteobacteria unclassified Alphaproteobacteria Alphaproteobacteria bacterium isol 273 aa
BacClassV Pseudomonadati Pseudomonadota Alphaproteobacteria unclassified Alphaproteobacteria Alphaproteobacteria bacterium isol 299 aa
BacClassV Pseudomonadati Bdellovibrionota Bdellovibrionia Bdellovibrionales Bdellovibrionales bacterium isolate DT 12 NQZ01191 273 aa
BacClassV Pseudomonadati Pseudomonadota Alphaproteobacteria Maricaulales Maricaulis sp GCF 017643475.1 WP 291893256.1 281 aa
BacClassV Pseudomonadati Pseudomonadota Alphaproteobacteria Maricaulales Litorimonas sp isolate AQ.64.F.B3 146 5 MEP1230023.1 286 aa
BacClassV Pseudomonadati PVC Planctomycetota unclassified Planctomycetota Planctomycetota bacterium isolate NORP49 PCJ60256.1 282 aa
BacClassV Pseudomonadati Pseudomonadota Gammaproteobacteria unclassified Gammaproteobacteria Gammaproteobacteria bacterium isol 293 aa
BacClassV Pseudomonadati Pseudomonadota Gammaproteobacteria Moraxellales Acinetobacter sp isolate AMR MDS 5182 HQW52451.1 294 aa
BacClassV Pseudomonadati Pseudomonadota Gammaproteobacteria Moraxellales Aquirhabdus parva WP 114900259.1 303 aa
BacClassV Pseudomonadati Pseudomonadota Gammaproteobacteria Moraxellales Acinetobacter rudis WP 016656277.1 294 aa
BacClassV Pseudomonadati Pseudomonadota Gammaproteobacteria Moraxellales Acinetobacter calcoaceticus WP 309951051.1 294 aa
BacClassV Pseudomonadati Pseudomonadota Gammaproteobacteria Moraxellales Acinetobacter pittii WP 320562389.1 294 aa
BacClassV Pseudomonadati Pseudomonadota Gammaproteobacteria Moraxellales Acinetobacter baumannii NIPH 601 ENW52547.1 796 aa
BacClassV Pseudomonadati Pseudomonadota Gammaproteobacteria Moraxellales Acinetobacter pullicarnis WP 139851109.1 303 aa
BacClassV Pseudomonadati Pseudomonadota Gammaproteobacteria Moraxellales Acinetobacter piscicola strain C1T1 2 a WP 130074012.1 295 aa
BacClassV Pseudomonadati Pseudomonadota Gammaproteobacteria Moraxellales unclassified Acinetobacter sp WP 286381573.1 301 aa
BacClassV Pseudomonadati Pseudomonadota Gammaproteobacteria Moraxellales Acinetobacter sp WCHAc060025 WP 130116488.1 294 aa
BacClassV Pseudomonadati Pseudomonadota Gammaproteobacteria Moraxellales uncultured Acinetobacter sp GCF 943914805.1 WP 296278 297 aa
BacClassV Pseudomonadati Pseudomonadota Gammaproteobacteria Moraxellales Acinetobacter sp P8 3 8 WP 010114168.1 294 aa
BacClassV Pseudomonadati Pseudomonadota Gammaproteobacteria Moraxellales Acinetobacter rongchengensis WP 120382911.1 294 aa
BacClassV Pseudomonadati Pseudomonadota Gammaproteobacteria Moraxellales Acinetobacter piscicola strain LW15 WP 089603694.1 294 aa
BacClassV Pseudomonadati Pseudomonadota Gammaproteobacteria Moraxellales Acinetobacter sp AS167 WP 336952877.1 297 aa
BacClassV Pseudomonadati Pseudomonadota Gammaproteobacteria Moraxellales Acinetobacter sp GCF 040396315.1 WP 353167125.1 297 aa
BacClassV Pseudomonadati Pseudomonadota Gammaproteobacteria Moraxellales Acinetobacter sp GCF 031253675.1 WP 309283417.1 297 aa
BacClassV Pseudomonadati Pseudomonadota Gammaproteobacteria Moraxellales Acinetobacter bereziniae WP 100884620.1 297 aa
BacClassV Pseudomonadati PVC Planctomycetota Planctomycetia Paludisphaera borealis GCF 031291815.1 WP 309380246.1 295 aa
BacClassV Pseudomonadati Nitrospirota Nitrospiria Nitrospirales Zavarzinella formosa strain DSM 19928 iso2 WP 020474457.1 284 aa
BacClassV Pseudomonadati PVC Planctomycetota unclassified Planctomycetota Planctomycetota bacterium isolate Bsp 9 MCP4261270.1 285 aa
BacClassV Pseudomonadati FCB Bacteroidota Chlorobiota Flavobacteriia Crocinitomicaceae bacterium isolate SAT141 iso1 MBD77454.1 285 aa
BacClassV Pseudomonadati Pseudomonadota Gammaproteobacteria Oceanospirillales Reinekea sp G2M2 21 WP 196161713.1 285 aa
BacClassV Pseudomonadati Pseudomonadota Gammaproteobacteria Alteromonadales Motilimonas pumila WP 119910867.1 276 aa
BacClassV Pseudomonadati Pseudomonadota Gammaproteobacteria Alteromonadales Motilimonas sp KMU 193 WP 410198100.1 279 aa
BacClassV Pseudomonadati Pseudomonadota Gammaproteobacteria Alteromonadales Motilimonas cestriensis WP 233050090.1 279 aa
BacClassV Pseudomonadati Pseudomonadota Gammaproteobacteria Cellvibrionales Gilvimarinus japonicus WP 382418090.1 286 aa
BacClassV Pseudomonadati Gammaproteobacteria Oceanospirillales Oceanospirillaceae Oleispira antarctica RB 8 CCK74937.1 288 aa
BacClassV Pseudomonadati Pseudomonadota Gammaproteobacteria Enterobacterales Gallaecimonas xiamenensis WP 008483588.1 285 aa
BacClassV Pseudomonadati Pseudomonadota Gammaproteobacteria Alteromonadales Shewanella algae strain SY5 WP 345858152.1 290 aa
BacClassV Pseudomonadati Pseudomonadota Gammaproteobacteria Vibrionales Grimontia celer WP 062664683.1 284 aa
BacClassV Pseudomonadati Pseudomonadota Gammaproteobacteria Oceanospirillales Marinicellulosiphila megalodicopiae strain TOYAMA 284 aa
BacClassV Pseudomonadati Pseudomonadota Gammaproteobacteria Pseudomonadales Pseudomonadales bacterium isolate ALOHA DT 135 MCJ8 317 aa
BacClassV Pseudomonadati Pseudomonadota Gammaproteobacteria Oceanospirillales Saccharospirillaceae bacterium isolate DT 108 NRB 284 aa
BacClassV Pseudomonadati Pseudomonadota Gammaproteobacteria Alteromonadales Alteromonadaceae bacterium M269 TDF34983.1 284 aa
BacClassV Pseudomonadati Pseudomonadota Gammaproteobacteria Oceanospirillales Endozoicomonas sp G2 1 WP 209111888.1 282 aa
BacClassV Pseudomonadati Pseudomonadota Gammaproteobacteria Alteromonadales Catenovulum maritimum strain Q1 iso2 WP 048690282.1 291 aa
BacClassV Pseudomonadati Pseudomonadota Gammaproteobacteria Vibrionales Enterovibrio norvegicus WP 017012043.1 291 aa
BacClassV Pseudomonadati Pseudomonadota Gammaproteobacteria Vibrionales Enterovibrio gelatinilyticus WP 274165041.1 291 aa
BacClassV Pseudomonadati Pseudomonadota Gammaproteobacteria Vibrionales Vibrio quintilis WP 073586312.1 286 aa
BacClassV Pseudomonadati Pseudomonadota Gammaproteobacteria Vibrionales Vibrio aerogenes WP 073602464.1 286 aa
BacClassV Pseudomonadati Pseudomonadota Gammaproteobacteria Vibrionales Photobacterium sp GJ3 WP 211647089.1 284 aa
BacClassV Pseudomonadati Pseudomonadota Gammaproteobacteria Vibrionales Photobacterium sp 53610 WP 330960457.1 284 aa
BacClassV Pseudomonadati Pseudomonadota Gammaproteobacteria Vibrionales Vibrio azureus WP 021710367.1 284 aa
BacClassV Pseudomonadati Pseudomonadota Gammaproteobacteria Alteromonadales Psychrobium sp isolate ME XS 2210 bin 3 MGB0899129 284 aa
BacClassV Pseudomonadati Pseudomonadota Gammaproteobacteria Alteromonadales Shewanella frigidimarina WP 342859479.1 284 aa
BacClassV Pseudomonadati Pseudomonadota Gammaproteobacteria Oceanospirillales Marinomonas sp PE14 40 WP 438466735.1 284 aa
BacClassV Pseudomonadati Pseudomonadota Gammaproteobacteria Alteromonadales Shewanella sp UCD FRSSP16 17 WP 065109664.1 284 aa
BacClassV Pseudomonadati FCB Bacteroidota Chlorobiota Bacteroidota Cytophagales bacterium isolate Cli 18 iso2 MCP4521015.1 304 aa
BacClassV Pseudomonadati Pseudomonadota Gammaproteobacteria Chromatiales Rheinheimera pacifica WP 331160815.1 284 aa
BacClassV Pseudomonadati Pseudomonadota Gammaproteobacteria Chromatiales Rheinheimera baltica strain SMS4 iso2 WP 305977393.1 285 aa
BacClassV Pseudomonadati Pseudomonadota Gammaproteobacteria Gammaproteobacteria incertae sedis Alkalimonas mucilaginosa WP 3300 284 aa
BacClassV Pseudomonadati Pseudomonadota Gammaproteobacteria Oceanospirillales Marinospirillum sp GCF 007119985.1 WP 416885884 284 aa
BacClassV Pseudomonadati Pseudomonadota Gammaproteobacteria Chromatiales Rheinheimera pleomorphica WP 166839638.1 284 aa
BacClassV Pseudomonadati Pseudomonadota Gammaproteobacteria Chromatiales Rheinheimera muenzenbergensis WP 353736096.1 284 aa
BacClassV Pseudomonadati Pseudomonadota Gammaproteobacteria Chromatiales Rheinheimera sp WS51 WP 433184536.1 300 aa
BacClassV Pseudomonadati Pseudomonadota Gammaproteobacteria Alteromonadales Shewanella putrefaciens WP 011919465.1 295 aa
BacClassV Pseudomonadati Pseudomonadota Betaproteobacteria Neisseriales Iodobacter fluviatilis WP 115228175.1 286 aa
BacClassV Pseudomonadati Pseudomonadota Betaproteobacteria Neisseriales Iodobacter arcticus WP 380189596.1 288 aa
BacClassV Pseudomonadati Pseudomonadota Betaproteobacteria Neisseriales Iodobacter ciconiae WP 206434430.1 289 aa
BacClassV Pseudomonadati Pseudomonadota Betaproteobacteria Burkholderiales Janthinobacterium sp B9 8 WP 046351137.1 289 aa
BacClassV CPR unclassified Patescibacteria Patescibacteria bacterium isolate B84T1L10 iso1 MCH7604942.1 261 aa
BacClassV Bacillati Bacillota unclassified Bacillota Bacillota bacterium isolate Bin7 7.2m MBE3101465.1 284 aa
BacClassV Pseudomonadati PVC Verrucomicrobiota unclassified Verrucomicrobiota Verrucomicrobiota bacterium isolate umpqua 2019 s 272 aa
BacClassV Pseudomonadati Pseudomonadota Gammaproteobacteria unclassified Gammaproteobacteria Gammaproteobacteria bacterium isol 281 aa
BacClassV CPR unclassified Patescibacteria Patescibacteria bacterium isolate SO 2017 LW4 29 MFA5049299.1 293 aa
BacClassV Pseudomonadati Thermodesulfobacteriota Desulfobacterales unclassified Desulfobacterales Desulfobacterales bacterium i 287 aa
BacClassV Pseudomonadati Pseudomonadota Gammaproteobacteria Enterobacterales Shigella flexneri strain 202227 EIH6462382.1 293 aa
BacClassV Pseudomonadati Pseudomonadota Gammaproteobacteria Enterobacterales Serratia sp JUb9 WP 187043948.1 286 aa
BacClassV Pseudomonadati Pseudomonadota Gammaproteobacteria Enterobacterales Gibbsiella dentisursi WP 346082941.1 286 aa
BacClassV Pseudomonadati Pseudomonadota Gammaproteobacteria Enterobacterales Serratia sp DD3 WP 037385495.1 286 aa
BacClassV Pseudomonadati Pseudomonadota Gammaproteobacteria Enterobacterales Serratia fonticola WP 309205501.1 286 aa
BacClassV Pseudomonadati Pseudomonadota Gammaproteobacteria Enterobacterales Erwinia sp AnSW2 5 WP 405259927.1 291 aa
BacClassV Pseudomonadati Pseudomonadota Gammaproteobacteria Enterobacterales Paramixta manurensis WP 173632453.1 286 aa
BacClassV Pseudomonadati Pseudomonadota Gammaproteobacteria Enterobacterales Cronobacter sakazakii 701 CCK04151.1 260 aa
BacClassV Pseudomonadati Pseudomonadota Gammaproteobacteria Enterobacterales Cronobacter malonaticus WP 023897868.1 284 aa
BacClassV Pseudomonadati Pseudomonadota Gammaproteobacteria Enterobacterales Franconibacter helveticus WP 049292953.1 285 aa
BacClassV Pseudomonadati Pseudomonadota Gammaproteobacteria Enterobacterales Atlantibacter sp GCF 032059805.1 WP 312971863.1 284 aa
BacClassV Pseudomonadati Pseudomonadota Gammaproteobacteria Enterobacterales Atlantibacter hermannii WP 284973687.1 284 aa
BacClassV Pseudomonadati Pseudomonadota Gammaproteobacteria Enterobacterales Mangrovibacter yixingensis WP 226572390.1 286 aa
BacClassV Pseudomonadati Pseudomonadota Gammaproteobacteria Enterobacterales Citrobacter freundii isolate N2 039 021G1 dasN2 03 261 aa
BacClassV Pseudomonadati Pseudomonadota Gammaproteobacteria Enterobacterales uncultured Pluralibacter sp GCF 913775985.1 WP 29 289 aa
BacClassV Pseudomonadati Pseudomonadota Gammaproteobacteria Enterobacterales Pluralibacter gergoviae WP 172730764.1 289 aa
BacClassV Pseudomonadati Pseudomonadota Gammaproteobacteria Enterobacterales Klebsiella sp WP 042893909.1 289 aa
BacClassV Pseudomonadati Pseudomonadota Gammaproteobacteria Enterobacterales Klebsiella michiganensis WP 181680439.1 289 aa
BacClassV Pseudomonadati Pseudomonadota Gammaproteobacteria Enterobacterales Enterobacter sp GCF 032067965.1 WP 318371075.1 286 aa
BacClassV Pseudomonadati Pseudomonadota Gammaproteobacteria Enterobacterales Kosakonia sp SMBL WEM22 WP 187802786.1 286 aa
BacClassV Pseudomonadati Pseudomonadota Gammaproteobacteria Enterobacterales Kosakonia sp ML.JS2a WP 263100821.1 286 aa
BacClassV Pseudomonadati Pseudomonadota Gammaproteobacteria Enterobacterales Kosakonia sp WP 007369779.1 286 aa
BacClassV Pseudomonadati Pseudomonadota Gammaproteobacteria Enterobacterales Phytobacter sp V91 WP 437887681.1 286 aa
BacClassV Pseudomonadati Pseudomonadota Gammaproteobacteria Enterobacterales Yokenella regensburgei WP 437432828.1 286 aa
BacClassV Pseudomonadati Pseudomonadota Gammaproteobacteria Enterobacterales Enterobacteriaceae sp WP 085006263.1 286 aa
Bacteria groups
FCB group
Alphaproteobacteria
Betaproteobacteria
Gammaproteobacteria
Deltaproteobacteria
Epsilonbacteria
Myxococcota
PVC group
Acidobacteria
Nitrospirae
Nitrospinae
Thermodesulfobacteria
Elusimicrobia
Deferribacteres
Chrysiogenetes
Spirochaetes
Bdellovibrio
Verrucomicrobia
Chloroflexi
Cyanobacteria
Actinobacteria
Vulcanimicrobia
Armatimonadetes
Firmicutes
Mollicutes
Deinococcus
Synergistetes
Thermotogae
Fusobacteria
CPR (Patescibacteria)
Bacteria incertae sedis
Archaea
ASR derived Eukaryote

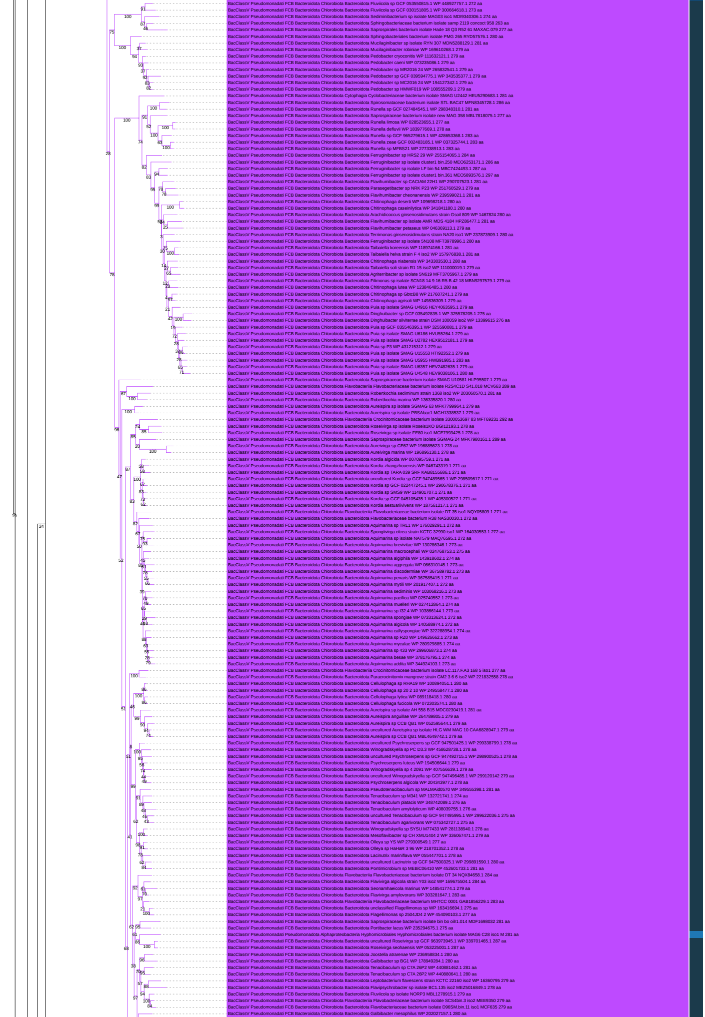
BacClassV Pseudomonadati FCB Bacteroidota Chlorobiota Bacteroidota Fluviicola sp GCF 053550815.1 WP 448927757.1 272 aa
BacClassV Pseudomonadati FCB Bacteroidota Chlorobiota Bacteroidota Fluviicola sp GCF 030151805.1 WP 300664618.1 273 aa
BacClassV Pseudomonadati FCB Bacteroidota Chlorobiota Bacteroidota Sediminibacterium sp isolate MAG03 iso1 MDI9340306.1 274 aa
BacClassV Pseudomonadati FCB Bacteroidota Chlorobiota Bacteroidota Sphingobacteriaceae bacterium isolate samp 2119 concoct 958 263 aa
BacClassV Pseudomonadati FCB Bacteroidota Chlorobiota Bacteroidota Saprospirales bacterium isolate Hade 18 Q3 R52 61 MAXAC.079 277 aa
BacClassV Pseudomonadati FCB Bacteroidota Chlorobiota Bacteroidota Sphingobacteriales bacterium isolate PMG 265 RYD57576.1 280 aa
BacClassV Pseudomonadati FCB Bacteroidota Chlorobiota Bacteroidota Mucilaginibacter sp isolate RYN 307 MDN5288129.1 281 aa
BacClassV Pseudomonadati FCB Bacteroidota Chlorobiota Bacteroidota Mucilaginibacter robiniae WP 169610268.1 279 aa
BacClassV Pseudomonadati FCB Bacteroidota Chlorobiota Bacteroidota Pedobacter cryoconitis WP 111632121.1 279 aa
BacClassV Pseudomonadati FCB Bacteroidota Chlorobiota Bacteroidota Pedobacter caeni WP 073235086.1 279 aa
BacClassV Pseudomonadati FCB Bacteroidota Chlorobiota Bacteroidota Pedobacter sp MR2016 24 WP 265832541.1 279 aa
BacClassV Pseudomonadati FCB Bacteroidota Chlorobiota Bacteroidota Pedobacter sp GCF 039594775.1 WP 343535377.1 279 aa
BacClassV Pseudomonadati FCB Bacteroidota Chlorobiota Bacteroidota Pedobacter sp MC2016 24 WP 194127342.1 279 aa
BacClassV Pseudomonadati FCB Bacteroidota Chlorobiota Bacteroidota Pedobacter sp HMWF019 WP 108555209.1 279 aa
BacClassV Pseudomonadati FCB Bacteroidota Chlorobiota Cytophagia Cyclobacteriaceae bacterium isolate SMAG U2442 HEU5290683.1 281 aa
BacClassV Pseudomonadati FCB Bacteroidota Chlorobiota Bacteroidota Spirosomataceae bacterium isolate STL BAC47 MFN8345728.1 286 aa
BacClassV Pseudomonadati FCB Bacteroidota Chlorobiota Bacteroidota Runella sp GCF 027484545.1 WP 298348310.1 281 aa
BacClassV Pseudomonadati FCB Bacteroidota Chlorobiota Bacteroidota Saprospiraceae bacterium isolate new MAG 358 MBL7818075.1 277 aa
BacClassV Pseudomonadati FCB Bacteroidota Chlorobiota Bacteroidota Runella limosa WP 028523655.1 277 aa
BacClassV Pseudomonadati FCB Bacteroidota Chlorobiota Bacteroidota Runella defluvii WP 183977669.1 278 aa
BacClassV Pseudomonadati FCB Bacteroidota Chlorobiota Bacteroidota Runella sp GCF 965279615.1 WP 428653368.1 283 aa
BacClassV Pseudomonadati FCB Bacteroidota Chlorobiota Bacteroidota Runella zeae GCF 002483185.1 WP 037325744.1 283 aa
BacClassV Pseudomonadati FCB Bacteroidota Chlorobiota Bacteroidota Runella sp MFBS21 WP 277338913.1 283 aa
BacClassV Pseudomonadati FCB Bacteroidota Chlorobiota Bacteroidota Ferruginibacter sp HRS2 29 WP 255154065.1 284 aa
BacClassV Pseudomonadati FCB Bacteroidota Chlorobiota Bacteroidota Ferruginibacter sp isolate cluster1 bin.250 MEO6253171.1 286 aa
BacClassV Pseudomonadati FCB Bacteroidota Chlorobiota Bacteroidota Ferruginibacter sp isolate LF bin 54 MBC7424493.1 287 aa
BacClassV Pseudomonadati FCB Bacteroidota Chlorobiota Bacteroidota Ferruginibacter sp isolate cluster1 bin.361 MEO5893576.1 297 aa
BacClassV Pseudomonadati FCB Bacteroidota Chlorobiota Bacteroidota Flavihumibacter sp CACIAM 22H1 WP 290707523.1 281 aa
BacClassV Pseudomonadati FCB Bacteroidota Chlorobiota Bacteroidota Parasegetibacter sp NRK P23 WP 251760529.1 279 aa
BacClassV Pseudomonadati FCB Bacteroidota Chlorobiota Bacteroidota Flavihumibacter cheonanensis WP 239599021.1 281 aa
BacClassV Pseudomonadati FCB Bacteroidota Chlorobiota Bacteroidota Chitinophaga deserti WP 109698218.1 280 aa
BacClassV Pseudomonadati FCB Bacteroidota Chlorobiota Bacteroidota Chitinophaga caseinilytica WP 341841180.1 280 aa
BacClassV Pseudomonadati FCB Bacteroidota Chlorobiota Bacteroidota Arachidicoccus ginsenosidimutans strain Gsoil 809 WP 1467824 280 aa
BacClassV Pseudomonadati FCB Bacteroidota Chlorobiota Bacteroidota Flavihumibacter sp isolate AMR MDS 4184 HPZ86477.1 281 aa
BacClassV Pseudomonadati FCB Bacteroidota Chlorobiota Bacteroidota Flavihumibacter petaseus WP 046369113.1 279 aa
BacClassV Pseudomonadati FCB Bacteroidota Chlorobiota Bacteroidota Terrimonas ginsenosidimutans strain NA20 iso1 WP 237873909.1 280 aa
BacClassV Pseudomonadati FCB Bacteroidota Chlorobiota Bacteroidota Ferruginibacter sp isolate 5N108 MFT3978996.1 280 aa
BacClassV Pseudomonadati FCB Bacteroidota Chlorobiota Bacteroidota Taibaiella koreensis WP 118974166.1 281 aa
BacClassV Pseudomonadati FCB Bacteroidota Chlorobiota Bacteroidota Taibaiella helva strain F 4 iso2 WP 157976838.1 281 aa
BacClassV Pseudomonadati FCB Bacteroidota Chlorobiota Bacteroidota Chitinophaga niabensis WP 343303530.1 280 aa
BacClassV Pseudomonadati FCB Bacteroidota Chlorobiota Bacteroidota Taibaiella soli strain R1 15 iso2 WP 111000019.1 279 aa
BacClassV Pseudomonadati FCB Bacteroidota Chlorobiota Bacteroidota Agriterribacter sp isolate 5N619 MFT3705967.1 279 aa
BacClassV Pseudomonadati FCB Bacteroidota Chlorobiota Bacteroidota Filimonas sp isolate SCN18 14 9 16 R5 B 42 18 MBN9297579.1 279 aa
BacClassV Pseudomonadati FCB Bacteroidota Chlorobiota Bacteroidota Chitinophaga lutea WP 123846485.1 280 aa
BacClassV Pseudomonadati FCB Bacteroidota Chlorobiota Bacteroidota Chitinophaga sp GbtcB8 WP 217607241.1 279 aa
BacClassV Pseudomonadati FCB Bacteroidota Chlorobiota Bacteroidota Chitinophaga agrisoli WP 149836309.1 279 aa
BacClassV Pseudomonadati FCB Bacteroidota Chlorobiota Bacteroidota Puia sp isolate SMAG U4916 HEY4063595.1 279 aa
BacClassV Pseudomonadati FCB Bacteroidota Chlorobiota Bacteroidota Dinghuibacter sp GCF 035492835.1 WP 325578205.1 275 aa
BacClassV Pseudomonadati FCB Bacteroidota Chlorobiota Bacteroidota Dinghuibacter silviterrae strain DSM 100059 iso2 WP 13399615 276 aa
BacClassV Pseudomonadati FCB Bacteroidota Chlorobiota Bacteroidota Puia sp GCF 035546395.1 WP 325590081.1 279 aa
BacClassV Pseudomonadati FCB Bacteroidota Chlorobiota Bacteroidota Puia sp isolate SMAG U6186 HVU55264.1 279 aa
BacClassV Pseudomonadati FCB Bacteroidota Chlorobiota Bacteroidota Puia sp isolate SMAG U2782 HEX9512181.1 279 aa
BacClassV Pseudomonadati FCB Bacteroidota Chlorobiota Bacteroidota Puia sp P3 WP 431215312.1 279 aa
BacClassV Pseudomonadati FCB Bacteroidota Chlorobiota Bacteroidota Puia sp isolate SMAG U15553 HTI92352.1 279 aa
BacClassV Pseudomonadati FCB Bacteroidota Chlorobiota Bacteroidota Puia sp isolate SMAG U5955 HWB91985.1 283 aa
BacClassV Pseudomonadati FCB Bacteroidota Chlorobiota Bacteroidota Puia sp isolate SMAG U6357 HEV2482635.1 279 aa
BacClassV Pseudomonadati FCB Bacteroidota Chlorobiota Bacteroidota Puia sp isolate SMAG U4548 HEV9038106.1 280 aa
BacClassV Pseudomonadati FCB Bacteroidota Chlorobiota Bacteroidota Saprospiraceae bacterium isolate SMAG U10581 HLP95507.1 279 aa
BacClassV Pseudomonadati FCB Bacteroidota Chlorobiota Flavobacteriia Flavobacteriaceae bacterium isolate R2S4C1D S41.018 MCV663 289 aa
BacClassV Pseudomonadati FCB Bacteroidota Chlorobiota Bacteroidota Robertkochia sediminum strain 1368 iso2 WP 203060570.1 281 aa
BacClassV Pseudomonadati FCB Bacteroidota Chlorobiota Bacteroidota Robertkochia marina WP 136335820.1 280 aa
BacClassV Pseudomonadati FCB Bacteroidota Chlorobiota Bacteroidota Aureispira sp isolate SGMAG 63 MFK7799964.1 279 aa
BacClassV Pseudomonadati FCB Bacteroidota Chlorobiota Bacteroidota Aureispira sp isolate PBSAbac1 MGH1338537.1 279 aa
BacClassV Pseudomonadati FCB Bacteroidota Chlorobiota Flavobacteriia Crocinitomicaceae bacterium isolate 3300053697 83 MFT69231 292 aa
BacClassV Pseudomonadati FCB Bacteroidota Chlorobiota Bacteroidota Roseivirga sp isolate Roseis1KO BGI12193.1 278 aa
BacClassV Pseudomonadati FCB Bacteroidota Chlorobiota Bacteroidota Roseivirga sp isolate FE80 iso1 MCE7993425.1 278 aa
BacClassV Pseudomonadati FCB Bacteroidota Chlorobiota Bacteroidota Saprospiraceae bacterium isolate SGMAG 24 MFK7980161.1 289 aa
BacClassV Pseudomonadati FCB Bacteroidota Chlorobiota Bacteroidota Aureivirga sp CE67 WP 196885623.1 278 aa
BacClassV Pseudomonadati FCB Bacteroidota Chlorobiota Bacteroidota Aureivirga marina WP 196896130.1 278 aa
BacClassV Pseudomonadati FCB Bacteroidota Chlorobiota Bacteroidota Kordia algicida WP 007095759.1 271 aa
BacClassV Pseudomonadati FCB Bacteroidota Chlorobiota Bacteroidota Kordia zhangzhouensis WP 046743319.1 271 aa
BacClassV Pseudomonadati FCB Bacteroidota Chlorobiota Bacteroidota Kordia sp TARA 039 SRF KAB8155686.1 271 aa
BacClassV Pseudomonadati FCB Bacteroidota Chlorobiota Bacteroidota uncultured Kordia sp GCF 947489565.1 WP 298509617.1 271 aa
BacClassV Pseudomonadati FCB Bacteroidota Chlorobiota Bacteroidota Kordia sp GCF 022447245.1 WP 290678376.1 271 aa
BacClassV Pseudomonadati FCB Bacteroidota Chlorobiota Bacteroidota Kordia sp SMS9 WP 114901707.1 271 aa
BacClassV Pseudomonadati FCB Bacteroidota Chlorobiota Bacteroidota Kordia sp GCF 045105435.1 WP 405300527.1 271 aa
BacClassV Pseudomonadati FCB Bacteroidota Chlorobiota Bacteroidota Kordia aestuariivivens WP 187561217.1 271 aa
BacClassV Pseudomonadati FCB Bacteroidota Chlorobiota Flavobacteriia Flavobacteriaceae bacterium isolate DT 35 iso1 NQY05809.1 271 aa
BacClassV Pseudomonadati FCB Bacteroidota Chlorobiota Bacteroidota Flavobacteriaceae bacterium R38 NAS30030.1 272 aa
BacClassV Pseudomonadati FCB Bacteroidota Chlorobiota Bacteroidota Aquimarina sp TRL1 WP 176029291.1 272 aa
BacClassV Pseudomonadati FCB Bacteroidota Chlorobiota Bacteroidota Spongiivirga citrea strain KCTC 32990 iso1 WP 164030553.1 272 aa
BacClassV Pseudomonadati FCB Bacteroidota Chlorobiota Bacteroidota Aquimarina sp isolate NAT579 MAQ76595.1 272 aa
BacClassV Pseudomonadati FCB Bacteroidota Chlorobiota Bacteroidota Aquimarina brevivitae WP 130286346.1 273 aa
BacClassV Pseudomonadati FCB Bacteroidota Chlorobiota Bacteroidota Aquimarina macrocephali WP 024768753.1 275 aa
BacClassV Pseudomonadati FCB Bacteroidota Chlorobiota Bacteroidota Aquimarina algiphila WP 143918602.1 274 aa
BacClassV Pseudomonadati FCB Bacteroidota Chlorobiota Bacteroidota Aquimarina aggregata WP 066310145.1 273 aa
BacClassV Pseudomonadati FCB Bacteroidota Chlorobiota Bacteroidota Aquimarina discodermiae WP 367589782.1 273 aa
BacClassV Pseudomonadati FCB Bacteroidota Chlorobiota Bacteroidota Aquimarina penaris WP 367585415.1 271 aa
BacClassV Pseudomonadati FCB Bacteroidota Chlorobiota Bacteroidota Aquimarina mytili WP 201917407.1 272 aa
BacClassV Pseudomonadati FCB Bacteroidota Chlorobiota Bacteroidota Aquimarina sediminis WP 103068216.1 273 aa
BacClassV Pseudomonadati FCB Bacteroidota Chlorobiota Bacteroidota Aquimarina pacifica WP 025740552.1 273 aa
BacClassV Pseudomonadati FCB Bacteroidota Chlorobiota Bacteroidota Aquimarina muelleri WP 027412864.1 274 aa
BacClassV Pseudomonadati FCB Bacteroidota Chlorobiota Bacteroidota Aquimarina sp I32.4 WP 103866144.1 273 aa
BacClassV Pseudomonadati FCB Bacteroidota Chlorobiota Bacteroidota Aquimarina spongiae WP 073313624.1 272 aa
BacClassV Pseudomonadati FCB Bacteroidota Chlorobiota Bacteroidota Aquimarina algicola WP 140588974.1 272 aa
BacClassV Pseudomonadati FCB Bacteroidota Chlorobiota Bacteroidota Aquimarina callyspongiae WP 322288954.1 274 aa
BacClassV Pseudomonadati FCB Bacteroidota Chlorobiota Bacteroidota Aquimarina sp RZD WP 149626662.1 273 aa
BacClassV Pseudomonadati FCB Bacteroidota Chlorobiota Bacteroidota Aquimarina mycalae WP 280929885.1 274 aa
BacClassV Pseudomonadati FCB Bacteroidota Chlorobiota Bacteroidota Aquimarina sp 433 WP 299606873.1 274 aa
BacClassV Pseudomonadati FCB Bacteroidota Chlorobiota Bacteroidota Aquimarina besae WP 378176795.1 274 aa
BacClassV Pseudomonadati FCB Bacteroidota Chlorobiota Bacteroidota Aquimarina addita WP 344924103.1 273 aa
BacClassV Pseudomonadati FCB Bacteroidota Chlorobiota Flavobacteriia Crocinitomicaceae bacterium isolate LC.117.F.A3 168 5 iso1 277 aa
BacClassV Pseudomonadati FCB Bacteroidota Chlorobiota Bacteroidota Paracrocinitomix mangrove strain GM2 3 6 6 iso2 WP 221832558 278 aa
BacClassV Pseudomonadati FCB Bacteroidota Chlorobiota Bacteroidota Cellulophaga sp RHA19 WP 100894051.1 280 aa
BacClassV Pseudomonadati FCB Bacteroidota Chlorobiota Bacteroidota Cellulophaga sp 20 2 10 WP 249558477.1 280 aa
BacClassV Pseudomonadati FCB Bacteroidota Chlorobiota Bacteroidota Cellulophaga lytica WP 089118418.1 280 aa
BacClassV Pseudomonadati FCB Bacteroidota Chlorobiota Bacteroidota Cellulophaga fucicola WP 072303574.1 280 aa
BacClassV Pseudomonadati FCB Bacteroidota Chlorobiota Bacteroidota Aureispira sp isolate AH 558 B15 MDC0230419.1 281 aa
BacClassV Pseudomonadati FCB Bacteroidota Chlorobiota Bacteroidota Aureispira anguillae WP 264789805.1 279 aa
BacClassV Pseudomonadati FCB Bacteroidota Chlorobiota Bacteroidota Aureispira sp CCB QB1 WP 052595644.1 279 aa
BacClassV Pseudomonadati FCB Bacteroidota Chlorobiota Bacteroidota uncultured Aureispira sp isolate HLG WM MAG 10 CAA6828947.1 279 aa
BacClassV Pseudomonadati FCB Bacteroidota Chlorobiota Bacteroidota Aureispira sp CCB QB1 MBL4649742.1 279 aa
BacClassV Pseudomonadati FCB Bacteroidota Chlorobiota Bacteroidota uncultured Psychroserpens sp GCF 947501425.1 WP 299338799.1 278 aa
BacClassV Pseudomonadati FCB Bacteroidota Chlorobiota Bacteroidota Winogradskyella sp PC D3.3 WP 458628738.1 278 aa
BacClassV Pseudomonadati FCB Bacteroidota Chlorobiota Bacteroidota uncultured Psychroserpens sp GCF 947492715.1 WP 298900525.1 278 aa
BacClassV Pseudomonadati FCB Bacteroidota Chlorobiota Bacteroidota Psychroserpens luteus WP 194506644.1 279 aa
BacClassV Pseudomonadati FCB Bacteroidota Chlorobiota Bacteroidota Winogradskyella sp 4 2091 WP 407556639.1 279 aa
BacClassV Pseudomonadati FCB Bacteroidota Chlorobiota Bacteroidota uncultured Winogradskyella sp GCF 947496485.1 WP 299120142 279 aa
BacClassV Pseudomonadati FCB Bacteroidota Chlorobiota Bacteroidota Psychroserpens algicola WP 204343977.1 278 aa
BacClassV Pseudomonadati FCB Bacteroidota Chlorobiota Bacteroidota Pseudotenacibaculum sp MALMAid0570 WP 349555398.1 281 aa
BacClassV Pseudomonadati FCB Bacteroidota Chlorobiota Bacteroidota Tenacibaculum sp M341 WP 132721741.1 274 aa
BacClassV Pseudomonadati FCB Bacteroidota Chlorobiota Bacteroidota Tenacibaculum platacis WP 348742089.1 276 aa
BacClassV Pseudomonadati FCB Bacteroidota Chlorobiota Bacteroidota Tenacibaculum amylolyticum WP 408039755.1 276 aa
BacClassV Pseudomonadati FCB Bacteroidota Chlorobiota Bacteroidota uncultured Tenacibaculum sp GCF 947495995.1 WP 299622036.1 275 aa
BacClassV Pseudomonadati FCB Bacteroidota Chlorobiota Bacteroidota Tenacibaculum agarivorans WP 075342727.1 275 aa
BacClassV Pseudomonadati FCB Bacteroidota Chlorobiota Bacteroidota Winogradskyella sp SYSU M77433 WP 281138940.1 278 aa
BacClassV Pseudomonadati FCB Bacteroidota Chlorobiota Bacteroidota Mesoflavibacter sp CH XMU1404 2 WP 336067471.1 279 aa
BacClassV Pseudomonadati FCB Bacteroidota Chlorobiota Bacteroidota Olleya sp YS WP 279300549.1 277 aa
BacClassV Pseudomonadati FCB Bacteroidota Chlorobiota Bacteroidota Olleya sp HaHaR 3 96 WP 218701352.1 278 aa
BacClassV Pseudomonadati FCB Bacteroidota Chlorobiota Bacteroidota Lacinutrix mariniflava WP 055447701.1 278 aa
BacClassV Pseudomonadati FCB Bacteroidota Chlorobiota Bacteroidota uncultured Lacinutrix sp GCF 947500325.1 WP 299891590.1 280 aa
BacClassV Pseudomonadati FCB Bacteroidota Chlorobiota Bacteroidota Pontimicrobium sp MEBiC06410 WP 452601733.1 281 aa
BacClassV Pseudomonadati FCB Bacteroidota Chlorobiota Flavobacteriia Flavobacteriaceae bacterium isolate DT 34 NQX84658.1 284 aa
BacClassV Pseudomonadati FCB Bacteroidota Chlorobiota Bacteroidota Flavivirga algicola strain Y03 iso2 WP 169675504.1 284 aa
BacClassV Pseudomonadati FCB Bacteroidota Chlorobiota Bacteroidota Seonamhaeicola marinus WP 148541774.1 279 aa
BacClassV Pseudomonadati FCB Bacteroidota Chlorobiota Bacteroidota Flavivirga amylovorans WP 303281647.1 283 aa
BacClassV Pseudomonadati FCB Bacteroidota Chlorobiota Flavobacteriia Flavobacteriaceae bacterium MHTCC 0001 GAB1856229.1 283 aa
BacClassV Pseudomonadati FCB Bacteroidota Chlorobiota Bacteroidota unclassified Flagellimonas sp WP 163416694.1 275 aa
BacClassV Pseudomonadati FCB Bacteroidota Chlorobiota Bacteroidota Flagellimonas sp 2504JD4 2 WP 454090103.1 277 aa
BacClassV Pseudomonadati FCB Bacteroidota Chlorobiota Bacteroidota Saprospiraceae bacterium isolate bin bo oilr1.014 MDF1698032 281 aa
BacClassV Pseudomonadati FCB Bacteroidota Chlorobiota Bacteroidota Portibacter lacus WP 235294675.1 275 aa
BacClassV Pseudomonadati Pseudomonadota Alphaproteobacteria Hyphomicrobiales Hyphomicrobiales bacterium isolate MAG6 C28 iso1 M 281 aa
BacClassV Pseudomonadati FCB Bacteroidota Chlorobiota Bacteroidota uncultured Roseivirga sp GCF 963973945.1 WP 339701465.1 287 aa
BacClassV Pseudomonadati FCB Bacteroidota Chlorobiota Bacteroidota Roseivirga seohaensis WP 053225001.1 287 aa
BacClassV Pseudomonadati FCB Bacteroidota Chlorobiota Bacteroidota Joostella atrarenae WP 236958834.1 280 aa
BacClassV Pseudomonadati FCB Bacteroidota Chlorobiota Bacteroidota Galbibacter sp BG1 WP 178949284.1 280 aa
BacClassV Pseudomonadati FCB Bacteroidota Chlorobiota Bacteroidota Tenacibaculum sp C7A 26P2 WP 440881462.1 281 aa
BacClassV Pseudomonadati FCB Bacteroidota Chlorobiota Bacteroidota Tenacibaculum sp C7A 26P2 WP 440880641.1 280 aa
BacClassV Pseudomonadati FCB Bacteroidota Chlorobiota Bacteroidota Leptobacterium flavescens strain KCTC 22160 iso2 WP 16360795 279 aa
BacClassV Pseudomonadati FCB Bacteroidota Chlorobiota Bacteroidota Flavipsychrobacter sp isolate BC1.135 iso2 MEZ5016849.1 278 aa
BacClassV Pseudomonadati FCB Bacteroidota Chlorobiota Bacteroidota Fluviicola sp isolate NORP3 MBL1278915.1 279 aa
BacClassV Pseudomonadati FCB Bacteroidota Chlorobiota Flavobacteriia Flavobacteriaceae bacterium isolate SCS4bin.3 iso2 MEE9350 279 aa
BacClassV Pseudomonadati FCB Bacteroidota Chlorobiota Bacteroidota Flavobacteraceae bacterium isolate D96SM.bin.11 iso1 MCF635 279 aa
BacClassV Pseudomonadati FCB Bacteroidota Chlorobiota Bacteroidota Galbibacter mesophilus WP 202027157.1 280 aa

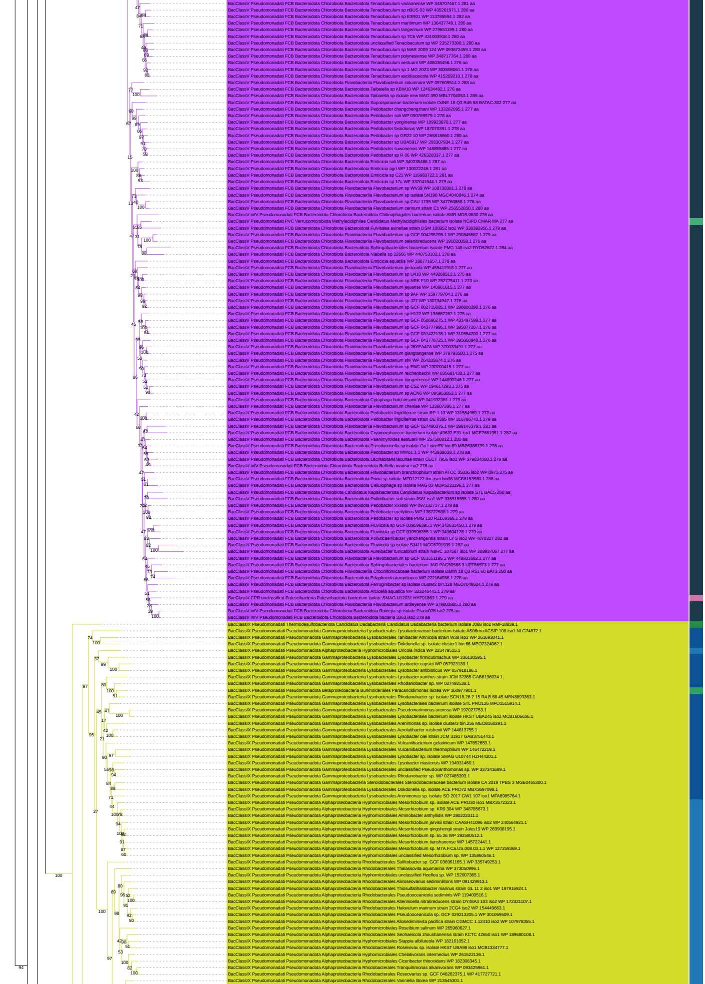

BacClassV Pseudomonadati FCB Bacteroidota Chlorobiota Bacteroidota Tenacibaculum vairaonense WP 348707467.1 281 aa
BacClassV Pseudomonadati FCB Bacteroidota Chlorobiota Bacteroidota Tenacibaculum sp nBUS 03 WP 435261971.1 280 aa
BacClassV Pseudomonadati FCB Bacteroidota Chlorobiota Bacteroidota Tenacibaculum sp E3R01 WP 113785594.1 282 aa
BacClassV Pseudomonadati FCB Bacteroidota Chlorobiota Bacteroidota Tenacibaculum maritimum WP 136437749.1 280 aa
BacClassV Pseudomonadati FCB Bacteroidota Chlorobiota Bacteroidota Tenacibaculum tangerinum WP 279651199.1 280 aa
BacClassV Pseudomonadati FCB Bacteroidota Chlorobiota Bacteroidota Tenacibaculum sp TC6 WP 431003918.1 280 aa
BacClassV Pseudomonadati FCB Bacteroidota Chlorobiota Bacteroidota unclassified Tenacibaculum sp WP 235273308.1 280 aa
BacClassV Pseudomonadati FCB Bacteroidota Chlorobiota Bacteroidota Tenacibaculum sp MAR 2009 124 WP 093672459.1 280 aa
BacClassV Pseudomonadati FCB Bacteroidota Chlorobiota Bacteroidota Tenacibaculum polynesiense WP 348717764.1 280 aa
BacClassV Pseudomonadati FCB Bacteroidota Chlorobiota Bacteroidota Tenacibaculum aestuarii WP 408036456.1 276 aa
BacClassV Pseudomonadati FCB Bacteroidota Chlorobiota Bacteroidota Tenacibaculum sp 1 MG 2023 WP 303508061.1 278 aa
BacClassV Pseudomonadati FCB Bacteroidota Chlorobiota Bacteroidota Tenacibaculum ascidiaceicola WP 415269210.1 278 aa
BacClassV Pseudomonadati FCB Bacteroidota Chlorobiota Flavobacteriia Flavobacterium columnare WP 097609514.1 283 aa
BacClassV Pseudomonadati FCB Bacteroidota Chlorobiota Bacteroidota Taibaiella sp KBW10 WP 124634482.1 276 aa
BacClassV Pseudomonadati FCB Bacteroidota Chlorobiota Bacteroidota Taibaiella sp isolate new MAG 390 MBL7704053.1 285 aa
BacClassV Pseudomonadati FCB Bacteroidota Chlorobiota Bacteroidota Saprospiraceae bacterium isolate OdNE 18 Q3 R46 58 BATAC.302 277 aa
BacClassV Pseudomonadati FCB Bacteroidota Chlorobiota Bacteroidota Pedobacter changchengzhani WP 133262095.1 277 aa
BacClassV Pseudomonadati FCB Bacteroidota Chlorobiota Bacteroidota Pedobacter soli WP 090769979.1 278 aa
BacClassV Pseudomonadati FCB Bacteroidota Chlorobiota Bacteroidota Pedobacter yonginense WP 109923870.1 277 aa
BacClassV Pseudomonadati FCB Bacteroidota Chlorobiota Bacteroidota Pedobacter fastidiosus WP 187070391.1 278 aa
BacClassV Pseudomonadati FCB Bacteroidota Chlorobiota Bacteroidota Pedobacter sp GR22 10 WP 265818660.1 280 aa
BacClassV Pseudomonadati FCB Bacteroidota Chlorobiota Bacteroidota Pedobacter sp UBA5917 WP 293307934.1 277 aa
BacClassV Pseudomonadati FCB Bacteroidota Chlorobiota Bacteroidota Pedobacter suwonensis WP 145855885.1 277 aa
BacClassV Pseudomonadati FCB Bacteroidota Chlorobiota Bacteroidota Pedobacter sp R 06 WP 426328337.1 277 aa
BacClassV Pseudomonadati FCB Bacteroidota Chlorobiota Bacteroidota Emticicia soli WP 340235486.1 287 aa
BacClassV Pseudomonadati FCB Bacteroidota Chlorobiota Bacteroidota Emticicia agri WP 130022246.1 281 aa
BacClassV Pseudomonadati FCB Bacteroidota Chlorobiota Bacteroidota Emticicia sp C21 WP 116983722.1 281 aa
BacClassV Pseudomonadati FCB Bacteroidota Chlorobiota Bacteroidota Emticicia sp 17c WP 337041644.1 279 aa
BacClassV Pseudomonadati FCB Bacteroidota Chlorobiota Flavobacteriia Flavobacterium sp WV39 WP 108738381.1 278 aa
BacClassV Pseudomonadati FCB Bacteroidota Chlorobiota Flavobacteriia Flavobacterium sp isolate 5N190 MGC4040846.1 274 aa
BacClassV Pseudomonadati FCB Bacteroidota Chlorobiota Flavobacteriia Flavobacterium sp CAU 1735 WP 347760868.1 278 aa
BacClassV Pseudomonadati FCB Bacteroidota Chlorobiota Flavobacteriia Flavobacterium cerinum strain C1 WP 256552850.1 280 aa
BacClassV inIV Pseudomonadati FCB Bacteroidota Chlorobiota Bacteroidota Chitinophagales bacterium isolate AMR MDS 0630 276 aa
BacClassV Pseudomonadati PVC Verrucomicrobiota Methylacidiphilae Candidatus Methylacidiphilales bacterium isolate NCIPD CMAR MA 277 aa
BacClassV Pseudomonadati FCB Bacteroidota Chlorobiota Bacteroidota Fulvitalea axinellae strain DSM 100852 iso2 WP 338392956.1 279 aa
BacClassV Pseudomonadati FCB Bacteroidota Chlorobiota Flavobacteriia Flavobacterium sp GCF 004295795.1 WP 290845587.1 279 aa
BacClassV Pseudomonadati FCB Bacteroidota Chlorobiota Flavobacteriia Flavobacterium selenitireducens WP 191020058.1 276 aa
BacClassV Pseudomonadati FCB Bacteroidota Chlorobiota Bacteroidota Sphingobacteriales bacterium isolate PMG 148 iso2 RYD52622.1 284 aa
BacClassV Pseudomonadati FCB Bacteroidota Chlorobiota Bacteroidota Niabella sp 22666 WP 440753102.1 278 aa
BacClassV Pseudomonadati FCB Bacteroidota Chlorobiota Bacteroidota Emticicia aquatilis WP 188771657.1 278 aa
BacClassV Pseudomonadati FCB Bacteroidota Chlorobiota Flavobacteriia Flavobacterium pedocola WP 455411918.1 277 aa
BacClassV Pseudomonadati FCB Bacteroidota Chlorobiota Flavobacteriia Flavobacterium sp U410 WP 449268512.1 275 aa
BacClassV Pseudomonadati FCB Bacteroidota Chlorobiota Flavobacteriia Flavobacterium sp NRK F10 WP 252775411.1 273 aa
BacClassV Pseudomonadati FCB Bacteroidota Chlorobiota Flavobacteriia Flavobacterium jejuense WP 140961615.1 277 aa
BacClassV Pseudomonadati FCB Bacteroidota Chlorobiota Flavobacteriia Flavobacterium sp 9AF WP 158779704.1 276 aa
BacClassV Pseudomonadati FCB Bacteroidota Chlorobiota Flavobacteriia Flavobacterium sp J27 WP 130734947.1 276 aa
BacClassV Pseudomonadati FCB Bacteroidota Chlorobiota Flavobacteriia Flavobacterium sp GCF 002715085.1 WP 290800290.1 276 aa
BacClassV Pseudomonadati FCB Bacteroidota Chlorobiota Flavobacteriia Flavobacterium sp H122 WP 136667262.1 275 aa
BacClassV Pseudomonadati FCB Bacteroidota Chlorobiota Flavobacteriia Flavobacterium sp GCF 050696275.1 WP 431497599.1 277 aa
BacClassV Pseudomonadati FCB Bacteroidota Chlorobiota Flavobacteriia Flavobacterium sp GCF 043777995.1 WP 395077207.1 276 aa
BacClassV Pseudomonadati FCB Bacteroidota Chlorobiota Flavobacteriia Flavobacterium sp GCF 031422135.1 WP 310554700.1 277 aa
BacClassV Pseudomonadati FCB Bacteroidota Chlorobiota Flavobacteriia Flavobacterium sp GCF 043778725.1 WP 395060940.1 276 aa
BacClassV Pseudomonadati FCB Bacteroidota Chlorobiota Flavobacteriia Flavobacterium sp 28YEA47A WP 370033491.1 277 aa
BacClassV Pseudomonadati FCB Bacteroidota Chlorobiota Flavobacteriia Flavobacterium qiangtangense WP 379793500.1 275 aa
BacClassV Pseudomonadati FCB Bacteroidota Chlorobiota Flavobacteriia Flavobacterium shii WP 264205874.1 276 aa
BacClassV Pseudomonadati FCB Bacteroidota Chlorobiota Flavobacteriia Flavobacterium sp ENC WP 230700415.1 277 aa
BacClassV Pseudomonadati FCB Bacteroidota Chlorobiota Flavobacteriia Flavobacterium reichenbachii WP 035681438.1 277 aa
BacClassV Pseudomonadati FCB Bacteroidota Chlorobiota Flavobacteriia Flavobacterium tiangeerense WP 144890246.1 277 aa
BacClassV Pseudomonadati FCB Bacteroidota Chlorobiota Flavobacteriia Flavobacterium sp CSZ WP 194617293.1 275 aa
BacClassV Pseudomonadati FCB Bacteroidota Chlorobiota Flavobacteriia Flavobacterium sp ACN6 WP 095953853.1 277 aa
BacClassV Pseudomonadati FCB Bacteroidota Chlorobiota Bacteroidota Cytophaga hutchinsonii WP 041932361.1 279 aa
BacClassV Pseudomonadati FCB Bacteroidota Chlorobiota Flavobacteriia Flavobacterium cheniae WP 133607396.1 277 aa
BacClassV Pseudomonadati FCB Bacteroidota Chlorobiota Bacteroidota Pedobacter frigiditerrae strain RP 1 13 WP 131554969.1 273 aa
BacClassV Pseudomonadati FCB Bacteroidota Chlorobiota Bacteroidota Pedobacter frigiditerrae strain DE 0385 WP 316786743.1 279 aa
BacClassV Pseudomonadati FCB Bacteroidota Chlorobiota Flavobacteriia Flavobacterium sp GCF 027490375.1 WP 298146379.1 281 aa
BacClassV Pseudomonadati FCB Bacteroidota Chlorobiota Bacteroidota Cryomorphaceae bacterium isolate 49632 E31 iso1 MCE2681951.1 282 aa
BacClassV Pseudomonadati FCB Bacteroidota Chlorobiota Bacteroidota Paenimyroides aestuarii WP 257500012.1 280 aa
BacClassV Pseudomonadati FCB Bacteroidota Chlorobiota Bacteroidota Pseudarcicella sp isolate Go LeineEff bin 69 MBP6386799.1 278 aa
BacClassV Pseudomonadati FCB Bacteroidota Chlorobiota Bacteroidota Pedobacter sp MW01 1 1 WP 443938038.1 278 aa
BacClassV Pseudomonadati FCB Bacteroidota Chlorobiota Bacteroidota Lacihabitans lacunae strain CECT 7956 iso1 WP 379834000.1 279 aa
BacClassV inIV Pseudomonadati FCB Bacteroidota Chlorobiota Bacteroidota Belliella marina iso2 278 aa
BacClassV Pseudomonadati FCB Bacteroidota Chlorobiota Bacteroidota Flavobacterium branchiophilum strain ATCC 35036 iso2 WP 0975 275 aa
BacClassV Pseudomonadati FCB Bacteroidota Chlorobiota Bacteroidota Pricia sp isolate MFD12122 ilm asm bin36 MGB6153560.1 286 aa
BacClassV Pseudomonadati FCB Bacteroidota Chlorobiota Bacteroidota Cellulophaga sp isolate MAG 03 MDP5231196.1 277 aa
BacClassV Pseudomonadati FCB Bacteroidota Chlorobiota Candidatus Kapaibacteriota Candidatus Kapaibacterium sp isolate STL BAC5 280 aa
BacClassV Pseudomonadati FCB Bacteroidota Chlorobiota Bacteroidota Pollutibacter soli strain JS81 iso1 WP 336515555.1 280 aa
BacClassV Pseudomonadati FCB Bacteroidota Chlorobiota Bacteroidota Pedobacter xixiisoli WP 097132737.1 278 aa
BacClassV Pseudomonadati FCB Bacteroidota Chlorobiota Bacteroidota Pedobacter ureilyticus WP 138722688.1 279 aa
BacClassV Pseudomonadati FCB Bacteroidota Chlorobiota Bacteroidota Pedobacter sp isolate PMG 120 RZL69366.1 279 aa
BacClassV Pseudomonadati FCB Bacteroidota Chlorobiota Bacteroidota Fluviicola sp GCF 039596395.1 WP 343631450.1 279 aa
BacClassV Pseudomonadati FCB Bacteroidota Chlorobiota Bacteroidota Fluviicola sp GCF 039596355.1 WP 343604178.1 279 aa
BacClassV Pseudomonadati FCB Bacteroidota Chlorobiota Bacteroidota Pollutibacter yanchengensis strain LY 5 iso2 WP 4070327 282 aa
BacClassV Pseudomonadati FCB Bacteroidota Chlorobiota Bacteroidota Fluviicola sp isolate SJ411 MCC6701939.1 282 aa
BacClassV Pseudomonadati FCB Bacteroidota Chlorobiota Bacteroidota Aureibacter tunicatorum strain NBRC 107587 iso1 WP 309937067 277 aa
BacClassV Pseudomonadati FCB Bacteroidota Chlorobiota Flavobacteriia Flavobacterium sp GCF 053551195.1 WP 448931682.1 277 aa
BacClassV Pseudomonadati FCB Bacteroidota Chlorobiota Bacteroidota Sphingobacteriales bacterium JAD PAG50586 3 UPT66573.1 277 aa
BacClassV Pseudomonadati FCB Bacteroidota Chlorobiota Flavobacteriia Crocinitomicaceae bacterium isolate Damh 18 Q3 R51 60 BAT3 280 aa
BacClassV Pseudomonadati FCB Bacteroidota Chlorobiota Bacteroidota Edaphocola aurantiacus WP 222164936.1 278 aa
BacClassV Pseudomonadati FCB Bacteroidota Chlorobiota Bacteroidota Ferruginibacter sp isolate cluster2 bin.128 MEO7048624.1 279 aa
BacClassV Pseudomonadati FCB Bacteroidota Chlorobiota Bacteroidota Arcicella aquatica WP 323246441.1 279 aa
BacClassV CPR unclassified Patescibacteria Patescibacteria bacterium isolate SMAG U12031 HYF01863.1 279 aa
BacClassV Pseudomonadati FCB Bacteroidota Chlorobiota Flavobacteriia Flavobacterium ardleyense WP 379803885.1 280 aa
BacClassV inIV Pseudomonadati FCB Bacteroidota Chlorobiota Bacteroidota Raineya sp isolate Prado078 iso2 275 aa
BacClassV inIV Pseudomonadati FCB Bacteroidota Chlorobiota Bacteroidota bacteria 3363 iso2 278 aa
BacClassIX Pseudomonadati Thermodesulfobacteriota Candidatus Dadabacteria Candidatus Dadabacteria bacterium isolate J088 iso2 RMF18839.1
BacClassIX Pseudomonadati Pseudomonadota Gammaproteobacteria Lysobacterales Lysobacteraceae bacterium isolate AS06rmzACSIP 108 iso1 NLG74672.1
BacClassIX Pseudomonadati Pseudomonadota Gammaproteobacteria Lysobacterales Tahibacter Amnicola strain W38 iso2 WP 261693041.1
BacClassIX Pseudomonadati Pseudomonadota Gammaproteobacteria Lysobacterales Dokdonella sp. isolate cluster1 bin.66 MEO7324062.1
BacClassIX Pseudomonadati Pseudomonadota Alphaproteobacteria Hyphomicrobiales Oricola indica WP 223479515.1
BacClassIX Pseudomonadati Pseudomonadota Gammaproteobacteria Lysobacterales Lysobacter firmicutimachus WP 336130595.1
BacClassIX Pseudomonadati Pseudomonadota Gammaproteobacteria Lysobacterales Lysobacter capsici WP 057923130.1
BacClassIX Pseudomonadati Pseudomonadota Gammaproteobacteria Lysobacterales Lysobacter antibioticus WP 057918186.1
BacClassIX Pseudomonadati Pseudomonadota Gammaproteobacteria Lysobacterales Lysobacter xanthus strain JCM 32365 GAB6196024.1
BacClassIX Pseudomonadati Pseudomonadota Gammaproteobacteria Lysobacterales Rhodanobacter sp. WP 027492538.1
BacClassIX Pseudomonadati Pseudomonadota Betaproteobacteria Burkholderiales Paracandidimonas lactea WP 160977901.1
BacClassIX Pseudomonadati Pseudomonadota Gammaproteobacteria Lysobacterales Rhodanobacter sp. isolate SCN18 26 2 15 R4 B 68 45 MBN8893363.1
BacClassIX Pseudomonadati Pseudomonadota Gammaproteobacteria Lysobacterales Lysobacterales bacterium isolate STL PRO126 MFO1515914.1
BacClassIX Pseudomonadati Pseudomonadota Gammaproteobacteria Lysobacterales Pseudomarimonas arenosa WP 192027753.1
BacClassIX Pseudomonadati Pseudomonadota Gammaproteobacteria Lysobacterales Lysobacterales bacterium isolate HKST UBA245 iso2 MCB1606636.1
BacClassIX Pseudomonadati Pseudomonadota Gammaproteobacteria Lysobacterales Arenimonas sp. isolate cluster3 bin.256 MEO8160291.1
BacClassIX Pseudomonadati Pseudomonadota Gammaproteobacteria Lysobacterales Aerolutibacter ruishenii WP 144813755.1
BacClassIX Pseudomonadati Pseudomonadota Gammaproteobacteria Lysobacterales Lysobacter olei strain JCM 31917 GAB3751443.1
BacClassIX Pseudomonadati Pseudomonadota Gammaproteobacteria Lysobacterales Vulcaniibacterium gelatinicum WP 147652653.1
BacClassIX Pseudomonadati Pseudomonadota Gammaproteobacteria Lysobacterales Vulcaniibacterium thermophilum WP 146472219.1
BacClassIX Pseudomonadati Pseudomonadota Gammaproteobacteria Lysobacterales Lysobacter sp. isolate SMAG U10744 HZH44201.1
BacClassIX Pseudomonadati Pseudomonadota Gammaproteobacteria Lysobacterales Lysobacter niastensis WP 194931460.1
BacClassIX Pseudomonadati Pseudomonadota Gammaproteobacteria Lysobacterales unclassified Pseudoxanthomonas sp. WP 337341689.1
BacClassIX Pseudomonadati Pseudomonadota Gammaproteobacteria Lysobacterales Rhodanobacter sp. WP 027485393.1
BacClassIX Pseudomonadati Pseudomonadota Gammaproteobacteria Steroidobacterales Steroidobacteraceae bacterium isolate CA 2019 TPBS 3 MGE0465300.1
BacClassIX Pseudomonadati Pseudomonadota Gammaproteobacteria Lysobacterales Dokdonella sp. isolate ACE PRO72 MBX3697098.1
BacClassIX Pseudomonadati Pseudomonadota Gammaproteobacteria Lysobacterales Arenimonas sp. isolate SO 2017 GW1 107 iso1 MFA6985764.1
BacClassIX Pseudomonadati Pseudomonadota Alphaproteobacteria Hyphomicrobiales Mesorhizobium sp. isolate ACE PRO30 iso1 MBX3572323.1
BacClassIX Pseudomonadati Pseudomonadota Alphaproteobacteria Hyphomicrobiales Mesorhizobium sp. KR9 304 WP 348785673.1
BacClassIX Pseudomonadati Pseudomonadota Alphaproteobacteria Hyphomicrobiales Aminobacter anthyllidis WP 280223311.1
BacClassIX Pseudomonadati Pseudomonadota Alphaproteobacteria Hyphomicrobiales Mesorhizobium jarvisii strain CAASH41096 iso2 WP 240564921.1
BacClassIX Pseudomonadati Pseudomonadota Alphaproteobacteria Hyphomicrobiales Mesorhizobium qingshengii strain Jales19 WP 269908195.1
BacClassIX Pseudomonadati Pseudomonadota Alphaproteobacteria Hyphomicrobiales Mesorhizobium sp. 65 26 WP 292580512.1
BacClassIX Pseudomonadati Pseudomonadota Alphaproteobacteria Hyphomicrobiales Mesorhizobium tianshanense WP 145722441.1
BacClassIX Pseudomonadati Pseudomonadota Alphaproteobacteria Hyphomicrobiales Mesorhizobium sp. M7A.F.Ca.US.008.03.1.1 WP 127259369.1
BacClassIX Pseudomonadati Pseudomonadota Alphaproteobacteria Hyphomicrobiales unclassified Mesorhizobium sp. WP 135860546.1
BacClassIX Pseudomonadati Pseudomonadota Alphaproteobacteria Rhodobacterales Sulfitobacter sp. GCF 036961165.1 WP 335749253.1
BacClassIX Pseudomonadati Pseudomonadota Alphaproteobacteria Rhodobacterales Thalassovita aquimarina WP 373050996.1
BacClassIX Pseudomonadati Pseudomonadota Alphaproteobacteria Hyphomicrobiales unclassified Hoeflea sp. WP 152007365.1
BacClassIX Pseudomonadati Pseudomonadota Alphaproteobacteria Rhodobacterales Aliiroseovarius sediminilitoris WP 091429913.1
BacClassIX Pseudomonadati Pseudomonadota Alphaproteobacteria Rhodobacterales Thiosulfatihalobacter marinus strain GL 11 2 iso1 WP 197916924.1
BacClassIX Pseudomonadati Pseudomonadota Alphaproteobacteria Rhodobacterales Pseudooceanicola sediminis WP 119400516.1
BacClassIX Pseudomonadati Pseudomonadota Alphaproteobacteria Rhodobacterales Alterinioella nitratireducens strain DY48A3 103 iso2 WP 172321107.1
BacClassIX Pseudomonadati Pseudomonadota Alphaproteobacteria Rhodobacterales Halovulum marinum strain 2CG4 iso2 WP 154449663.1
BacClassIX Pseudomonadati Pseudomonadota Alphaproteobacteria Rhodobacterales Pseudooceanicola sp. GCF 029213205.1 WP 301069509.1
BacClassIX Pseudomonadati Pseudomonadota Alphaproteobacteria Rhodobacterales Allosediminivita pacifica strain CGMCC 1.12410 iso2 WP 107978355.1
BacClassIX Pseudomonadati Pseudomonadota Alphaproteobacteria Hyphomicrobiales Roseibium salinum WP 265960627.1
BacClassIX Pseudomonadati Pseudomonadota Alphaproteobacteria Rhodobacterales Seohaeicola zhoushanensis strain KCTC 42650 iso1 WP 189680108.1
BacClassIX Pseudomonadati Pseudomonadota Alphaproteobacteria Hyphomicrobiales Stappia albiluteola WP 182161052.1
BacClassIX Pseudomonadati Pseudomonadota Alphaproteobacteria Rhodobacterales Roseivivax sp. isolate HKST UBA98 iso1 MCB1334777.1
BacClassIX Pseudomonadati Pseudomonadota Alphaproteobacteria Hyphomicrobiales Chelativorans intermedius WP 261522136.1
BacClassIX Pseudomonadati Pseudomonadota Alphaproteobacteria Hyphomicrobiales Ciceribacter thiooxidans WP 182308345.1
BacClassIX Pseudomonadati Pseudomonadota Alphaproteobacteria Rhodobacterales Tranquillimonas alkanivorans WP 093425961.1
BacClassIX Pseudomonadati Pseudomonadota Alphaproteobacteria Rhodobacterales Roseovarius sp. GCF 048262375.1 WP 417727721.1
BacClassIX Pseudomonadati Pseudomonadota Alphaproteobacteria Rhodobacterales Vannielia litorea WP 213545301.1

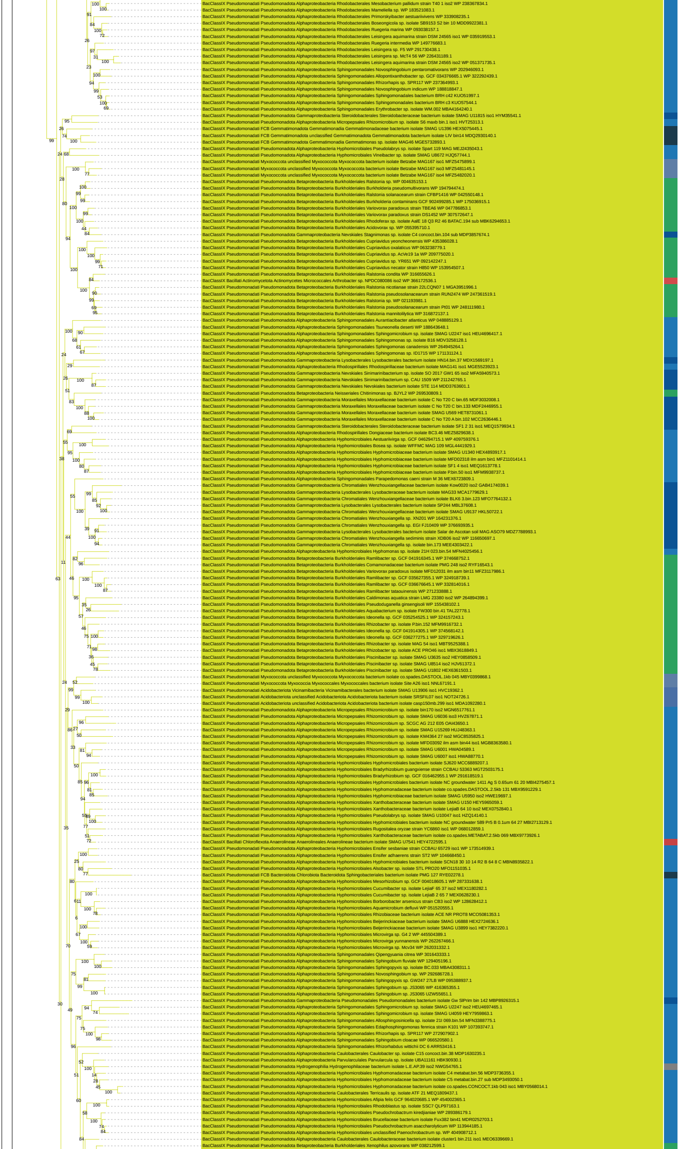

BacClassIX Pseudomonadati Pseudomonadota Alphaproteobacteria Rhodobacterales Mesobacterium pallidum strain T40 1 iso2 WP 238367834.1
BacClassIX Pseudomonadati Pseudomonadota Alphaproteobacteria Rhodobacterales Mameliella sp. WP 183521083.1
BacClassIX Pseudomonadati Pseudomonadota Alphaproteobacteria Rhodobacterales Primorskyibacter aestuariivivens WP 333908235.1
BacClassIX Pseudomonadati Pseudomonadota Alphaproteobacteria Rhodobacterales Boseongicola sp. isolate SB9153 S2 bin 10 MDD9922381.1
BacClassIX Pseudomonadati Pseudomonadota Alphaproteobacteria Rhodobacterales Ruegeria marina WP 093038157.1
BacClassIX Pseudomonadati Pseudomonadota Alphaproteobacteria Rhodobacterales Leisingera aquimarina strain DSM 24565 iso1 WP 035919553.1
BacClassIX Pseudomonadati Pseudomonadota Alphaproteobacteria Rhodobacterales Ruegeria intermedia WP 149776683.1
BacClassIX Pseudomonadati Pseudomonadota Alphaproteobacteria Rhodobacterales Leisingera sp. F5 WP 291730438.1
BacClassIX Pseudomonadati Pseudomonadota Alphaproteobacteria Rhodobacterales Leisingera sp. McT4 56 WP 226431189.1
BacClassIX Pseudomonadati Pseudomonadota Alphaproteobacteria Rhodobacterales Leisingera aquimarina strain DSM 24565 iso2 WP 051371735.1
BacClassIX Pseudomonadati Pseudomonadota Alphaproteobacteria Sphingomonadales Novosphingobium pentaromativorans WP 202946093.1
BacClassIX Pseudomonadati Pseudomonadota Alphaproteobacteria Sphingomonadales Allopontixanthobacter sp. GCF 034376665.1 WP 322292439.1
BacClassIX Pseudomonadati Pseudomonadota Alphaproteobacteria Sphingomonadales Rhizorhapis sp. SPR117 WP 237364993.1
BacClassIX Pseudomonadati Pseudomonadota Alphaproteobacteria Sphingomonadales Novosphingobium indicum WP 188818847.1
BacClassIX Pseudomonadati Pseudomonadota Alphaproteobacteria Sphingomonadales Sphingomonadales bacterium BRH c42 KUO51997.1
BacClassIX Pseudomonadati Pseudomonadota Alphaproteobacteria Sphingomonadales Sphingomonadales bacterium BRH c3 KUO57544.1
BacClassIX Pseudomonadati Pseudomonadota Alphaproteobacteria Sphingomonadales Erythrobacter sp. isolate WM.002 MBA4164240.1
BacClassIX Pseudomonadati Pseudomonadota Gammaproteobacteria Steroidobacterales Steroidobacteraceae bacterium isolate SMAG U11815 iso1 HYM35541.1
BacClassIX Pseudomonadati Pseudomonadota Alphaproteobacteria Micropepsales Rhizomicrobium sp. isolate S6 maxb bin.1 iso1 HVT25313.1
BacClassIX Pseudomonadati FCB Gemmatimonadota Gemmatimonadia Gemmatimonadaceae bacterium isolate SMAG U1396 HEX5075445.1
BacClassIX Pseudomonadati FCB Gemmatimonadota unclassified Gemmatimonadota Gemmatimonadota bacterium isolate LIV bin14 MDQ2930140.1
BacClassIX Pseudomonadati FCB Gemmatimonadota Gemmatimonadia Gemmatimonas sp. isolate MAG46 MGE5732893.1
BacClassIX Pseudomonadati Pseudomonadota Alphaproteobacteria Hyphomicrobiales Pseudolabrys sp. isolate Spart 119 MAG MEJ2435043.1
BacClassIX Pseudomonadati Pseudomonadota Alphaproteobacteria Hyphomicrobiales Vineibacter sp. isolate SMAG U8672 HJQ57744.1
BacClassIX Pseudomonadati Myxococcota unclassified Myxococcota Myxococcota bacterium isolate Betzabe MAG167 iso1 MFZ5475899.1
BacClassIX Pseudomonadati Myxococcota unclassified Myxococcota Myxococcota bacterium isolate Betzabe MAG167 iso3 MFZ5481145.1
BacClassIX Pseudomonadati Myxococcota unclassified Myxococcota Myxococcota bacterium isolate Betzabe MAG167 iso4 MFZ5482020.1
BacClassIX Pseudomonadati Pseudomonadota Betaproteobacteria Burkholderiales Ralstonia sp. WP 004635153.1
BacClassIX Pseudomonadati Pseudomonadota Betaproteobacteria Burkholderiales Burkholderia pseudomultivorans WP 194794474.1
BacClassIX Pseudomonadati Pseudomonadota Betaproteobacteria Burkholderiales Ralstonia solanacearum strain CFBP1416 WP 042550148.1
BacClassIX Pseudomonadati Pseudomonadota Betaproteobacteria Burkholderiales Burkholderia contaminans GCF 902499285.1 WP 175036915.1
BacClassIX Pseudomonadati Pseudomonadota Betaproteobacteria Burkholderiales Variovorax paradoxus strain TBEA6 WP 047786853.1
BacClassIX Pseudomonadati Pseudomonadota Betaproteobacteria Burkholderiales Variovorax paradoxus strain DS1452 WP 307572647.1
BacClassIX Pseudomonadati Pseudomonadota Betaproteobacteria Burkholderiales Rhodoferax sp. isolate AalE 18 Q3 R2 46 BATAC.194 sub MBK6294653.1
BacClassIX Pseudomonadati Pseudomonadota Betaproteobacteria Burkholderiales Acidovorax sp. WP 055395710.1
BacClassIX Pseudomonadati Pseudomonadota Gammaproteobacteria Nevskiales Stagnimonas sp. isolate C4 concoct.bin.104 sub MDP3857674.1
BacClassIX Pseudomonadati Pseudomonadota Betaproteobacteria Burkholderiales Cupriavidus yeoncheonensis WP 435386028.1
BacClassIX Pseudomonadati Pseudomonadota Betaproteobacteria Burkholderiales Cupriavidus oxalaticus WP 063238779.1
BacClassIX Pseudomonadati Pseudomonadota Betaproteobacteria Burkholderiales Cupriavidus sp. AcVe19 1a WP 209775020.1
BacClassIX Pseudomonadati Pseudomonadota Betaproteobacteria Burkholderiales Cupriavidus sp. YR651 WP 092142247.1
BacClassIX Pseudomonadati Pseudomonadota Betaproteobacteria Burkholderiales Cupriavidus necator strain H850 WP 153954507.1
BacClassIX Pseudomonadati Pseudomonadota Betaproteobacteria Burkholderiales Ralstonia condita WP 316655626.1
BacClassIX Bacillati Actinomycetota Actinomycetes Micrococcales Arthrobacter sp. NPDC080086 iso2 WP 366172536.1
BacClassIX Pseudomonadati Pseudomonadota Betaproteobacteria Burkholderiales Ralstonia nicotianae strain 22LCQN07 1 MGA3951996.1
BacClassIX Pseudomonadati Pseudomonadota Betaproteobacteria Burkholderiales Ralstonia pseudosolanacearum strain RUN2474 WP 247361519.1
BacClassIX Pseudomonadati Pseudomonadota Betaproteobacteria Burkholderiales Ralstonia sp. WP 021193981.1
BacClassIX Pseudomonadati Pseudomonadota Betaproteobacteria Burkholderiales Ralstonia pseudosolanacearum strain Pt01 WP 248111980.1
BacClassIX Pseudomonadati Pseudomonadota Betaproteobacteria Burkholderiales Ralstonia mannitolilytica WP 316872137.1
BacClassIX Pseudomonadati Pseudomonadota Alphaproteobacteria Sphingomonadales Aurantiacibacter atlanticus WP 048885129.1
BacClassIX Pseudomonadati Pseudomonadota Alphaproteobacteria Sphingomonadales Tsuneonella deserti WP 188643648.1
BacClassIX Pseudomonadati Pseudomonadota Alphaproteobacteria Sphingomonadales Sphingomicrobium sp. isolate SMAG U2247 iso1 HEU4696417.1
BacClassIX Pseudomonadati Pseudomonadota Alphaproteobacteria Sphingomonadales Sphingomonas sp. isolate B16 MDV3258128.1
BacClassIX Pseudomonadati Pseudomonadota Alphaproteobacteria Sphingomonadales Sphingomonas canadensis WP 264945264.1
BacClassIX Pseudomonadati Pseudomonadota Alphaproteobacteria Sphingomonadales Sphingomonas sp. ID1715 WP 171131124.1
BacClassIX Pseudomonadati Pseudomonadota Gammaproteobacteria Lysobacterales Lysobacterales bacterium isolate HN14.bin.37 MDX1569197.1
BacClassIX Pseudomonadati Pseudomonadota Alphaproteobacteria Rhodospirillales Rhodospirillaceae bacterium isolate MAG141 iso1 MGE5523923.1
BacClassIX Pseudomonadati Pseudomonadota Gammaproteobacteria Nevskiales Sinimarinibacterium sp. isolate SO 2017 GW1 65 iso2 MFA5940573.1
BacClassIX Pseudomonadati Pseudomonadota Gammaproteobacteria Nevskiales Sinimarinibacterium sp. CAU 1509 WP 211242765.1
BacClassIX Pseudomonadati Pseudomonadota Gammaproteobacteria Nevskiales Nevskiales bacterium isolate STE 114 MDD3763601.1
BacClassIX Pseudomonadati Pseudomonadota Betaproteobacteria Neisseriales Chitinimonas sp. BJYL2 WP 269530809.1
BacClassIX Pseudomonadati Pseudomonadota Gammaproteobacteria Moraxellales Moraxellaceae bacterium isolate C No T20 C bin.65 MDF3032008.1
BacClassIX Pseudomonadati Pseudomonadota Gammaproteobacteria Moraxellales Moraxellaceae bacterium isolate C No T20 C bin.133 MDF2446955.1
BacClassIX Pseudomonadati Pseudomonadota Gammaproteobacteria Moraxellales Moraxellaceae bacterium isolate SMAG U569 HET8731061.1
BacClassIX Pseudomonadati Pseudomonadota Gammaproteobacteria Moraxellales Moraxellaceae bacterium isolate C No T20 A bin.102 MCC2636446.1
BacClassIX Pseudomonadati Pseudomonadota Gammaproteobacteria Steroidobacterales Steroidobacteraceae bacterium isolate SF1 2 31 iso1 MEQ1579934.1
BacClassIX Pseudomonadati Pseudomonadota Alphaproteobacteria Rhodospirillales Dongiaceae bacterium isolate BC3.46 MEZ5829638.1
BacClassIX Pseudomonadati Pseudomonadota Alphaproteobacteria Hyphomicrobiales Aestuariivirga sp. GCF 046294715.1 WP 409759376.1
BacClassIX Pseudomonadati Pseudomonadota Alphaproteobacteria Hyphomicrobiales Bosea sp. isolate WFFMC MAG 109 MGL4441929.1
BacClassIX Pseudomonadati Pseudomonadota Alphaproteobacteria Hyphomicrobiales Hyphomicrobiaceae bacterium isolate SMAG U1340 HEX4893917.1
BacClassIX Pseudomonadati Pseudomonadota Alphaproteobacteria Hyphomicrobiales Hyphomicrobiaceae bacterium isolate MFD02318 ilm asm bin1 MFZ1101414.1
BacClassIX Pseudomonadati Pseudomonadota Alphaproteobacteria Hyphomicrobiales Hyphomicrobiaceae bacterium isolate SF1 4 iso1 MEQ1613778.1
BacClassIX Pseudomonadati Pseudomonadota Alphaproteobacteria Hyphomicrobiales Hyphomicrobiaceae bacterium isolate P.bin.50 iso1 MFM9938737.1
BacClassIX Pseudomonadati Pseudomonadota Alphaproteobacteria Sphingomonadales Parapedomonas caeni strain M 36 MEX6723809.1
BacClassIX Pseudomonadati Pseudomonadota Gammaproteobacteria Chromatiales Wenzhouxiangellaceae bacterium isolate Kow0020 iso2 GAB4174039.1
BacClassIX Pseudomonadati Pseudomonadota Gammaproteobacteria Lysobacterales Lysobacterales bacterium isolate MAG33 MCA1779629.1
BacClassIX Pseudomonadati Pseudomonadota Gammaproteobacteria Chromatiales Wenzhouxiangellaceae bacterium isolate BLK6 3.bin.123 MFO7764132.1
BacClassIX Pseudomonadati Pseudomonadota Gammaproteobacteria Lysobacterales Lysobacterales bacterium isolate SP244 MBL37608.1
BacClassIX Pseudomonadati Pseudomonadota Gammaproteobacteria Chromatiales Wenzhouxiangellaceae bacterium isolate SMAG U9137 HKL50722.1
BacClassIX Pseudomonadati Pseudomonadota Gammaproteobacteria Chromatiales Wenzhouxiangella sp. XN201 WP 164231376.1
BacClassIX Pseudomonadati Pseudomonadota Gammaproteobacteria Chromatiales Wenzhouxiangella sp. EGI FJ10409 WP 376693935.1
BacClassIX Pseudomonadati Pseudomonadota Gammaproteobacteria Lysobacterales Lysobacterales bacterium isolate Salar de Ascotan soil MAG ASO79 MDZ7788993.1
BacClassIX Pseudomonadati Pseudomonadota Gammaproteobacteria Chromatiales Wenzhouxiangella sediminis strain XDB06 iso2 WP 116650697.1
BacClassIX Pseudomonadati Pseudomonadota Gammaproteobacteria Chromatiales Wenzhouxiangella sp. isolate bin.173 MEE4303422.1
BacClassIX Pseudomonadati Pseudomonadota Alphaproteobacteria Hyphomicrobiales Hyphomonas sp. isolate 21H 023.bin.54 MFN4025456.1
BacClassIX Pseudomonadati Pseudomonadota Betaproteobacteria Burkholderiales Ramlibacter sp. GCF 041916345.1 WP 374668752.1
BacClassIX Pseudomonadati Pseudomonadota Betaproteobacteria Burkholderiales Comamonadaceae bacterium isolate PMG 248 iso2 RYF16543.1
BacClassIX Pseudomonadati Pseudomonadota Betaproteobacteria Burkholderiales Variovorax paradoxus isolate MFD12031 ilm asm bin11 MFZ3117986.1
BacClassIX Pseudomonadati Pseudomonadota Betaproteobacteria Burkholderiales Ramlibacter sp. GCF 035627355.1 WP 324918739.1
BacClassIX Pseudomonadati Pseudomonadota Betaproteobacteria Burkholderiales Ramlibacter sp. GCF 036676645.1 WP 332814016.1
BacClassIX Pseudomonadati Pseudomonadota Betaproteobacteria Burkholderiales Ramlibacter tataouinensis WP 271233888.1
BacClassIX Pseudomonadati Pseudomonadota Betaproteobacteria Burkholderiales Caldimonas aquatica strain LMG 23380 iso2 WP 264894399.1
BacClassIX Pseudomonadati Pseudomonadota Betaproteobacteria Burkholderiales Pseudoduganella ginsengisoli WP 155438102.1
BacClassIX Pseudomonadati Pseudomonadota Betaproteobacteria Burkholderiales Aquabacterium sp. isolate FW300 bin.41 TAL22778.1
BacClassIX Pseudomonadati Pseudomonadota Betaproteobacteria Burkholderiales Ideonella sp. GCF 035254525.1 WP 324157243.1
BacClassIX Pseudomonadati Pseudomonadota Betaproteobacteria Burkholderiales Rhizobacter sp. isolate P.bin.152 MFM9916732.1
BacClassIX Pseudomonadati Pseudomonadota Betaproteobacteria Burkholderiales Ideonella sp. GCF 041914305.1 WP 374568142.1
BacClassIX Pseudomonadati Pseudomonadota Betaproteobacteria Burkholderiales Ideonella sp. GCF 036277275.1 WP 329719626.1
BacClassIX Pseudomonadati Pseudomonadota Betaproteobacteria Burkholderiales Rhizobacter sp. isolate MAG 54 iso1 MBT9525388.1
BacClassIX Pseudomonadati Pseudomonadota Betaproteobacteria Burkholderiales Rhizobacter sp. isolate ACE PRO46 iso1 MBX3618849.1
BacClassIX Pseudomonadati Pseudomonadota Betaproteobacteria Burkholderiales Piscinibacter sp. isolate SMAG U3635 iso2 HEY0858509.1
BacClassIX Pseudomonadati Pseudomonadota Betaproteobacteria Burkholderiales Piscinibacter sp. isolate SMAG U8514 iso2 HJV61372.1
BacClassIX Pseudomonadati Pseudomonadota Betaproteobacteria Burkholderiales Piscinibacter sp. isolate SMAG U1802 HEX6361503.1
BacClassIX Pseudomonadati Myxococcota unclassified Myxococcota Myxococcota bacterium isolate co.spades.DASTOOL.1kb 045 MBY0399868.1
BacClassIX Pseudomonadati Myxococcota Myxococcia Myxococcales Myxococcales bacterium isolate Site A26 iso1 NNL67191.1
BacClassIX Pseudomonadati Acidobacteriota Vicinamibacteria Vicinamibacterales Vicinamibacterales bacterium isolate SMAG U13906 iso1 HVC19362.1
BacClassIX Pseudomonadati Acidobacteriota unclassified Acidobacteriota Acidobacteria bacterium isolate SRSFIL07 iso1 NOT24726.1
BacClassIX Pseudomonadati Acidobacteriota unclassified Acidobacteriota Acidobacteria bacterium isolate casp150mb.299 iso1 MDA1092280.1
BacClassIX Pseudomonadati Pseudomonadota Alphaproteobacteria Micropepsales Rhizomicrobium sp. isolate bin170 iso2 MGN6517761.1
BacClassIX Pseudomonadati Pseudomonadota Alphaproteobacteria Micropepsales Rhizomicrobium sp. isolate SMAG U6036 iso3 HVZ67871.1
BacClassIX Pseudomonadati Pseudomonadota Alphaproteobacteria Micropepsales Rhizomicrobium sp. SCGC AG 212 E05 OAI43650.1
BacClassIX Pseudomonadati Pseudomonadota Alphaproteobacteria Micropepsales Rhizomicrobium sp. isolate SMAG U15269 HUJ48363.1
BacClassIX Pseudomonadati Pseudomonadota Alphaproteobacteria Micropepsales Rhizomicrobium sp. isolate KM4364 27 iso2 MGC8535825.1
BacClassIX Pseudomonadati Pseudomonadota Alphaproteobacteria Micropepsales Rhizomicrobium sp. isolate MFD03092 ilm asm bin44 iso1 MGB8363580.1
BacClassIX Pseudomonadati Pseudomonadota Alphaproteobacteria Micropepsales Rhizomicrobium sp. isolate SMAG U6001 HWA04589.1
BacClassIX Pseudomonadati Pseudomonadota Alphaproteobacteria Micropepsales Rhizomicrobium sp. isolate SMAG U6007 iso1 HWA88770.1
BacClassIX Pseudomonadati Pseudomonadota Alphaproteobacteria Hyphomicrobiales Hyphomicrobiales bacterium isolate SJ620 MCC6889207.1
BacClassIX Pseudomonadati Pseudomonadota Alphaproteobacteria Hyphomicrobiales Bradyrhizobium guangxiense strain CCBAU 53363 MGT2503175.1
BacClassIX Pseudomonadati Pseudomonadota Alphaproteobacteria Hyphomicrobiales Bradyrhizobium sp. GCF 016462955.1 WP 291618519.1
BacClassIX Pseudomonadati Pseudomonadota Alphaproteobacteria Hyphomicrobiales Hyphomicrobiales bacterium isolate NC groundwater 1411 Ag S 0.65um 61 20 MBI4275457.1
BacClassIX Pseudomonadati Pseudomonadota Alphaproteobacteria Hyphomicrobiales Hyphomonadaceae bacterium isolate co.spades.DASTOOL.2.5kb 131 MBX9591229.1
BacClassIX Pseudomonadati Pseudomonadota Alphaproteobacteria Hyphomicrobiales Hyphomicrobiaceae bacterium isolate SMAG U5950 iso2 HWE19697.1
BacClassIX Pseudomonadati Pseudomonadota Alphaproteobacteria Hyphomicrobiales Xanthobacteraceae bacterium isolate SMAG U150 HEY5965059.1
BacClassIX Pseudomonadati Pseudomonadota Alphaproteobacteria Hyphomicrobiales Xanthobacteraceae bacterium isolate LejiaB 64 10 iso2 MEX0752840.1
BacClassIX Pseudomonadati Pseudomonadota Alphaproteobacteria Hyphomicrobiales Pseudolabrys sp. isolate SMAG U10047 iso1 HZQ14140.1
BacClassIX Pseudomonadati Pseudomonadota Alphaproteobacteria Hyphomicrobiales Hyphomicrobiales bacterium isolate NC groundwater 589 Pr5 B 0.1um 64 27 MBI2713129.1
BacClassIX Pseudomonadati Pseudomonadota Alphaproteobacteria Hyphomicrobiales Rugositalea oryzae strain YC6860 iso1 WP 068012859.1
BacClassIX Pseudomonadati Pseudomonadota Alphaproteobacteria Hyphomicrobiales Xanthobacteraceae bacterium isolate co.spades.METABAT.2.5kb 069 MBX9773926.1
BacClassIX Bacillati Chloroflexota Anaerolineae Anaerolineales Anaerolineae bacterium isolate SMAG U7541 HEY4722595.1
BacClassIX Pseudomonadati Pseudomonadota Alphaproteobacteria Hyphomicrobiales Ensifer sesbaniae strain CCBAU 65729 iso1 WP 173514939.1
BacClassIX Pseudomonadati Pseudomonadota Alphaproteobacteria Hyphomicrobiales Ensifer adhaerens strain ST2 WP 104668450.1
BacClassIX Pseudomonadati Pseudomonadota Alphaproteobacteria Hyphomicrobiales Hyphomicrobiales bacterium isolate SCN18 30 10 14 R2 B 64 8 C MBN8935822.1
BacClassIX Pseudomonadati Pseudomonadota Alphaproteobacteria Hyphomicrobiales Alsobacter sp. isolate STL PRO20 MFO1151035.1
BacClassIX Pseudomonadati FCB Bacteroidota Chlorobiota Bacteroidota Sphingobacteriales bacterium isolate PMG 127 RYE02278.1
BacClassIX Pseudomonadati Pseudomonadota Alphaproteobacteria Hyphomicrobiales Mesorhizobium sp. GCF 004018605.1 WP 287331638.1
BacClassIX Pseudomonadati Pseudomonadota Alphaproteobacteria Hyphomicrobiales Cucumibacter sp. isolate LejiaF 65 37 iso2 MEX1180282.1
BacClassIX Pseudomonadati Pseudomonadota Alphaproteobacteria Hyphomicrobiales Cucumibacter sp. isolate LejiaB 2 65 7 MEX0628230.1
BacClassIX Pseudomonadati Pseudomonadota Alphaproteobacteria Hyphomicrobiales Borborobacter arsenicus strain CB3 iso2 WP 128628412.1
BacClassIX Pseudomonadati Pseudomonadota Alphaproteobacteria Hyphomicrobiales Aquamicrobium defluvii WP 051520555.1
BacClassIX Pseudomonadati Pseudomonadota Alphaproteobacteria Hyphomicrobiales Rhizobiaceae bacterium isolate ACE NR PROT8 MCO5081353.1
BacClassIX Pseudomonadati Pseudomonadota Alphaproteobacteria Hyphomicrobiales Beijerinckiaceae bacterium isolate SMAG U6888 HEX2724636.1
BacClassIX Pseudomonadati Pseudomonadota Alphaproteobacteria Hyphomicrobiales Beijerinckiaceae bacterium isolate SMAG U3899 iso1 HEY7382220.1
BacClassIX Pseudomonadati Pseudomonadota Alphaproteobacteria Hyphomicrobiales Microvirga sp. G4 2 WP 445504389.1
BacClassIX Pseudomonadati Pseudomonadota Alphaproteobacteria Hyphomicrobiales Microvirga yunnanensis WP 262267466.1
BacClassIX Pseudomonadati Pseudomonadota Alphaproteobacteria Hyphomicrobiales Microvirga sp. Mcv34 WP 262031332.1
BacClassIX Pseudomonadati Pseudomonadota Alphaproteobacteria Sphingomonadales Qipengyuania citrea WP 301643333.1
BacClassIX Pseudomonadati Pseudomonadota Alphaproteobacteria Sphingomonadales Sphingobium fluviale WP 129405196.1
BacClassIX Pseudomonadati Pseudomonadota Alphaproteobacteria Sphingomonadales Sphingopyxis sp. isolate BC.033 MBA4308311.1
BacClassIX Pseudomonadati Pseudomonadota Alphaproteobacteria Sphingomonadales Novosphingobium sp. WP 292686728.1
BacClassIX Pseudomonadati Pseudomonadota Alphaproteobacteria Sphingomonadales Sphingopyxis sp. GW247 27LB WP 095388937.1
BacClassIX Pseudomonadati Pseudomonadota Alphaproteobacteria Sphingomonadales Sphingobium sp. JS3065 WP 416365355.1
BacClassIX Pseudomonadati Pseudomonadota Alphaproteobacteria Sphingomonadales Sphingobium sp. JS3065 UZW55651.1
BacClassIX Pseudomonadati Pseudomonadota Gammaproteobacteria Pseudomonadales Pseudomonadales bacterium isolate Gw SlPrim bin 142 MBP8926315.1
BacClassIX Pseudomonadati Pseudomonadota Alphaproteobacteria Sphingomonadales Sphingomicrobium sp. isolate SMAG U2247 iso2 HEU4697465.1
BacClassIX Pseudomonadati Pseudomonadota Alphaproteobacteria Sphingomonadales Sphingomicrobium sp. isolate SMAG U4059 HEY7959863.1
BacClassIX Pseudomonadati Pseudomonadota Alphaproteobacteria Sphingomonadales Allosphingosinicella sp. isolate 21I 069.bin.54 MFN3388775.1
BacClassIX Pseudomonadati Pseudomonadota Alphaproteobacteria Sphingomonadales Edaphosphingomonas fennica strain K101 WP 107393747.1
BacClassIX Pseudomonadati Pseudomonadota Alphaproteobacteria Sphingomonadales Rhizorhapis sp. SPR117 WP 272907902.1
BacClassIX Pseudomonadati Pseudomonadota Alphaproteobacteria Sphingomonadales Sphingobium cloacae WP 066520580.1
BacClassIX Pseudomonadati Pseudomonadota Alphaproteobacteria Sphingomonadales Rhizorhabdus wittichii DC 6 ARR53416.1
BacClassIX Pseudomonadati Pseudomonadota Alphaproteobacteria Caulobacterales Caulobacter sp. isolate C15 concoct.bin.38 MDP1630235.1
BacClassIX Pseudomonadati Pseudomonadota Alphaproteobacteria Parvularculales Parvularcula sp. isolate UBA11161 HBK90930.1
BacClassIX Pseudomonadati Pseudomonadota Hydrogenophilia Hydrogenophilaceae bacterium isolate L.E.AP.39 iso2 NWG54765.1
BacClassIX Pseudomonadati Pseudomonadota Alphaproteobacteria Hyphomicrobiales Hyphomonadaceae bacterium isolate C4 metabat.bin.56 MDP3736355.1
BacClassIX Pseudomonadati Pseudomonadota Alphaproteobacteria Hyphomicrobiales Hyphomonadaceae bacterium isolate C5 metabat.bin.27 sub MDP3493050.1
BacClassIX Pseudomonadati Pseudomonadota Alphaproteobacteria Hyphomicrobiales Hyphomonadaceae bacterium isolate co.spades.CONCOCT.1kb 043 iso1 MBY0568014.1
BacClassIX Pseudomonadati Pseudomonadota Alphaproteobacteria Caulobacterales Terricaulis sp. isolate ATF 21 MEQ1809437.1
BacClassIX Pseudomonadati Pseudomonadota Alphaproteobacteria Hyphomicrobiales Afipia felis GCF 964020685.1 WP 454002365.1
BacClassIX Pseudomonadati Pseudomonadota Alphaproteobacteria Hyphomicrobiales Rhodoblastus sp. isolate SSC7 QLP97163.1
BacClassIX Pseudomonadati Pseudomonadota Alphaproteobacteria Hyphomicrobiales Pseudochrobactrum kiredjianiae WP 289386179.1
BacClassIX Pseudomonadati Pseudomonadota Alphaproteobacteria Hyphomicrobiales Brucellaceae bacterium isolate Fux382 bin41 MDR0252703.1
BacClassIX Pseudomonadati Pseudomonadota Alphaproteobacteria Hyphomicrobiales Pseudochrobactrum asaccharolyticum WP 113944185.1
BacClassIX Pseudomonadati Pseudomonadota Alphaproteobacteria Hyphomicrobiales unclassified Paenochrobactrum sp. WP 404908712.1
BacClassIX Pseudomonadati Pseudomonadota Alphaproteobacteria Caulobacterales Caulobacteraceae bacterium isolate cluster1 bin.211 iso1 MEO6339669.1
BacClassIX Pseudomonadati Pseudomonadota Betaproteobacteria Burkholderiales Xenophilus azovorans WP 038212599.1

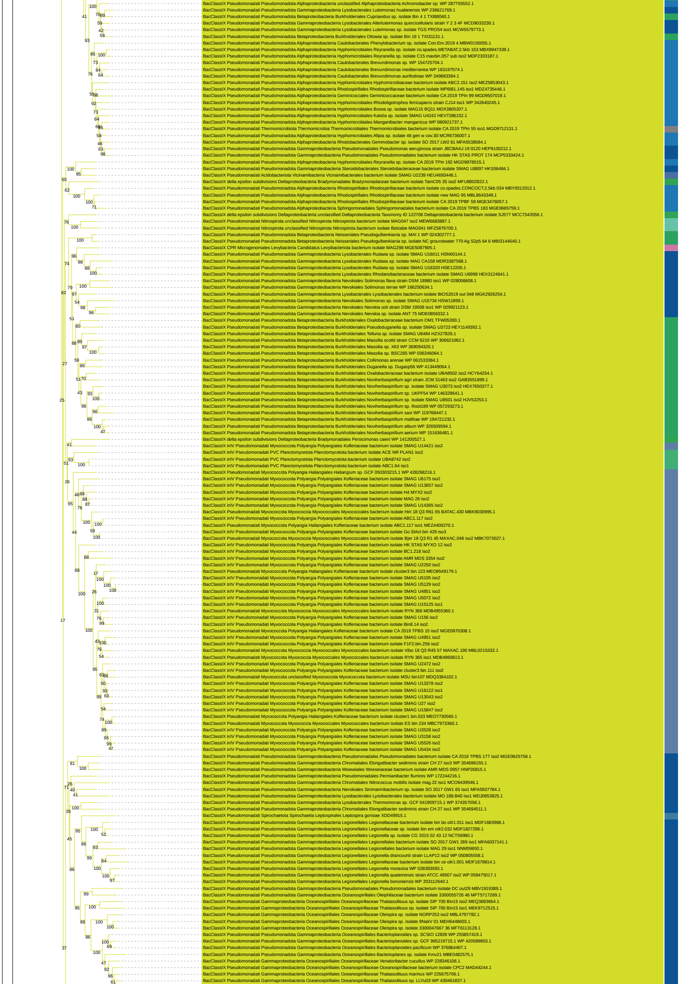

BacClassIX Pseudomonadati Pseudomonadota Alphaproteobacteria unclassified Alphaproteobacteria Achromobacter sp. WP 287755552.1
BacClassIX Pseudomonadati Pseudomonadota Gammaproteobacteria Lysobacterales Luteimonas huabeiensis WP 236621769.1
BacClassIX Pseudomonadati Pseudomonadota Betaproteobacteria Burkholderiales Cupriavidus sp. isolate Bin 4 1 TXI88040.1
BacClassIX Pseudomonadati Pseudomonadota Gammaproteobacteria Lysobacterales Alterluteimonas quercicellularis strain Y 2 3 4F MCD9033230.1
BacClassIX Pseudomonadati Pseudomonadota Gammaproteobacteria Lysobacterales Luteimonas sp. isolate TGS PRO54 iso1 MCW5579773.1
BacClassIX Pseudomonadati Pseudomonadota Betaproteobacteria Burkholderiales Ottowia sp. isolate Bin 19 1 TXI31131.1
BacClassIX Pseudomonadati Pseudomonadota Alphaproteobacteria Caulobacterales Phenylobacterium sp. isolate Con.Enr.2019 4 MBW0150055.1
BacClassIX Pseudomonadati Pseudomonadota Alphaproteobacteria Hyphomicrobiales Reyranella sp. isolate co.spades.METABAT.2.5kb 153 MBX9947339.1
BacClassIX Pseudomonadati Pseudomonadota Alphaproteobacteria Hyphomicrobiales Reyranella sp. isolate C15 maxbin.057 sub iso2 MDP2333187.1
BacClassIX Pseudomonadati Pseudomonadota Alphaproteobacteria Caulobacterales Brevundimonas sp. WP 154725704.1
BacClassIX Pseudomonadati Pseudomonadota Alphaproteobacteria Caulobacterales Brevundimonas mediterranea WP 183197574.1
BacClassIX Pseudomonadati Pseudomonadota Alphaproteobacteria Caulobacterales Brevundimonas aurifodinae WP 349683394.1
BacClassIX Pseudomonadati Pseudomonadota Alphaproteobacteria Hyphomicrobiales Hyphomicrobiaceae bacterium isolate ABC2.151 iso2 MEZ5853043.1
BacClassIX Pseudomonadati Pseudomonadota Alphaproteobacteria Rhodospirillales Rhodospirillaceae bacterium isolate MP6IB1.145 iso1 MDZ4735446.1
BacClassIX Pseudomonadati Pseudomonadota Alphaproteobacteria Geminicoccales Geminicoccaceae bacterium isolate CA 2019 TPIn 99 MGD9507019.1
BacClassIX Pseudomonadati Pseudomonadota Alphaproteobacteria Hyphomicrobiales Rhodoligotrophos ferricapiens strain CJ14 iso1 WP 342640245.1
BacClassIX Pseudomonadati Pseudomonadota Alphaproteobacteria Hyphomicrobiales Bosea sp. isolate MAG15 BQ11 MDX3805207.1
BacClassIX Pseudomonadati Pseudomonadota Alphaproteobacteria Hyphomicrobiales Kaistia sp. isolate SMAG U4242 HEV7286152.1
BacClassIX Pseudomonadati Pseudomonadota Alphaproteobacteria Hyphomicrobiales Manganibacter manganicus WP 080921737.1
BacClassIX Pseudomonadati Thermomicrobiota Thermomicrobia Thermomicrobiales Thermomicrobiales bacterium isolate CA 2019 TPIn 55 iso1 MGD9712131.1
BacClassIX Pseudomonadati Pseudomonadota Alphaproteobacteria Hyphomicrobiales Afipia sp. isolate 46 gen w cov.30 MCR6736007.1
BacClassIX Pseudomonadati Pseudomonadota Alphaproteobacteria Rhodobacterales Gemmobacter sp. isolate SO 2017 LW2 61 MFA5538584.1
BacClassIX Pseudomonadati Pseudomonadota Gammaproteobacteria Pseudomonadales Pseudomonas aeruginosa strain JBCBAAJ 19 0120 HEP9100212.1
BacClassIX Pseudomonadati Pseudomonadota Gammaproteobacteria Pseudomonadales Pseudomonadales bacterium isolate HK STAS PROT 174 MCP5333424.1
BacClassIX Pseudomonadati Pseudomonadota Alphaproteobacteria Hyphomicrobiales Reyranella sp. isolate CA 2019 TPIn 192 MGD9878515.1
BacClassIX Pseudomonadati Pseudomonadota Gammaproteobacteria Steroidobacterales Steroidobacteraceae bacterium isolate SMAG U8897 HKS56484.1
BacClassIX Pseudomonadati Acidobacteriota Vicinamibacteria Vicinamibacterales Vicinamibacteraceae bacterium isolate SMAG U2239 HEU4693446.1
BacClassIX delta epsilon subdivisions Deltaproteobacteria Bradymonadales Bradymonadaceae bacterium isolate TamC05 35 iso2 MFU8802822.1
BacClassIX Pseudomonadati Pseudomonadota Alphaproteobacteria Rhodospirillales Rhodospirillaceae bacterium isolate co.spades.CONCOCT.2.5kb 034 MBY0512012.1
BacClassIX Pseudomonadati Pseudomonadota Alphaproteobacteria Rhodospirillales Rhodospirillaceae bacterium isolate new MAG 95 MBL8643349.1
BacClassIX Pseudomonadati Pseudomonadota Alphaproteobacteria Rhodospirillales Rhodospirillaceae bacterium isolate CA 2019 TPBF 59 MGE3476057.1
BacClassIX Pseudomonadati Pseudomonadota Alphaproteobacteria Sphingomonadales Sphingomonadales bacterium isolate CA 2019 TPBS 183 MGE0665759.1
BacClassIX delta epsilon subdivisions Deltaproteobacteria unclassified Deltaproteobacteria Taxonomy ID 122706 Deltaproteobacteria bacterium isolate SJ577 MCC7343556.1
BacClassIX Pseudomonadati Nitrospirota unclassified Nitrospirota Nitrospirota bacterium isolate MAG047 iso2 MEW6683887.1
BacClassIX Pseudomonadati Nitrospirota unclassified Nitrospirota Nitrospirota bacterium isolate Betzabe MAG041 MFZ5876700.1
BacClassIX Pseudomonadati Pseudomonadota Betaproteobacteria Neisseriales Pseudogulbenkiania sp. MAI 1 WP 024302777.1
BacClassIX Pseudomonadati Pseudomonadota Betaproteobacteria Neisseriales Pseudogulbenkiania sp. isolate NC groundwater 770 Ag S2p5 64 6 MBI3144640.1
BacClassIX CPR Microgenomates Levybacteria Candidatus Levybacteriota bacterium isolate MAG298 MGE5087905.1
BacClassIX Pseudomonadati Pseudomonadota Gammaproteobacteria Lysobacterales Rudaea sp. isolate SMAG U16011 HSN00144.1
BacClassIX Pseudomonadati Pseudomonadota Gammaproteobacteria Lysobacterales Rudaea sp. isolate MAG CA158 MDR3387568.1
BacClassIX Pseudomonadati Pseudomonadota Gammaproteobacteria Lysobacterales Rudaea sp. isolate SMAG U16320 HSE12205.1
BacClassIX Pseudomonadati Pseudomonadota Gammaproteobacteria Lysobacterales Rhodanobacteraceae bacterium isolate SMAG U6998 HEX3124641.1
BacClassIX Pseudomonadati Pseudomonadota Gammaproteobacteria Nevskiales Solimonas flava strain DSM 18980 iso1 WP 028006608.1
BacClassIX Pseudomonadati Pseudomonadota Gammaproteobacteria Nevskiales Solimonas terrae WP 166250634.1
BacClassIX Pseudomonadati Pseudomonadota Gammaproteobacteria Lysobacterales Lysobacterales bacterium isolate BIOS2019 out 048 MGK2926254.1
BacClassIX Pseudomonadati Pseudomonadota Gammaproteobacteria Nevskiales Solimonas sp. isolate SMAG U15734 HSW11899.1
BacClassIX Pseudomonadati Pseudomonadota Gammaproteobacteria Nevskiales Nevskia soli strain DSM 19509 iso1 WP 029921123.1
BacClassIX Pseudomonadati Pseudomonadota Gammaproteobacteria Nevskiales Nevskia sp. isolate ANT 75 MDE0856332.1
BacClassIX Pseudomonadati Pseudomonadota Betaproteobacteria Burkholderiales Oxalobacteraceae bacterium OM1 TFW05393.1
BacClassIX Pseudomonadati Pseudomonadota Betaproteobacteria Burkholderiales Pseudoduganella sp. isolate SMAG U3723 HEY1149392.1
BacClassIX Pseudomonadati Pseudomonadota Betaproteobacteria Burkholderiales Telluria sp. isolate SMAG U8484 HZX27826.1
BacClassIX Pseudomonadati Pseudomonadota Betaproteobacteria Burkholderiales Massilia scottii strain CCM 9210 WP 306621062.1
BacClassIX Pseudomonadati Pseudomonadota Betaproteobacteria Burkholderiales Massilia sp. X63 WP 369094320.1
BacClassIX Pseudomonadati Pseudomonadota Betaproteobacteria Burkholderiales Massilia sp. BSC265 WP 036246064.1
BacClassIX Pseudomonadati Pseudomonadota Betaproteobacteria Burkholderiales Collimonas arenae WP 061533384.1
BacClassIX Pseudomonadati Pseudomonadota Betaproteobacteria Burkholderiales Duganella sp. Dugasp56 WP 413449064.1
BacClassIX Pseudomonadati Pseudomonadota Betaproteobacteria Burkholderiales Oxalobacteraceae bacterium isolate UBA8502 iso2 HCY64254.1
BacClassIX Pseudomonadati Pseudomonadota Betaproteobacteria Burkholderiales Noviherbaspirillum agri strain JCM 31463 iso2 GAB3551899.1
BacClassIX Pseudomonadati Pseudomonadota Betaproteobacteria Burkholderiales Noviherbaspirillum sp. isolate SMAG U3073 iso2 HEX7650377.1
BacClassIX Pseudomonadati Pseudomonadota Betaproteobacteria Burkholderiales Noviherbaspirillum sp. UKPF54 WP 146329641.1
BacClassIX Pseudomonadati Pseudomonadota Betaproteobacteria Burkholderiales Noviherbaspirillum sp. isolate SMAG U8501 iso2 HJV22253.1
BacClassIX Pseudomonadati Pseudomonadota Betaproteobacteria Burkholderiales Noviherbaspirillum sp. Root189 WP 057293273.1
BacClassIX Pseudomonadati Pseudomonadota Betaproteobacteria Burkholderiales Noviherbaspirillum saxi WP 119768447.1
BacClassIX Pseudomonadati Pseudomonadota Betaproteobacteria Burkholderiales Noviherbaspirillum malthae WP 194721232.1
BacClassIX Pseudomonadati Pseudomonadota Betaproteobacteria Burkholderiales Noviherbaspirillum album WP 326509594.1
BacClassIX Pseudomonadati Pseudomonadota Betaproteobacteria Burkholderiales Noviherbaspirillum aerium WP 151636481.1
BacClassIX delta epsilon subdivisions Deltaproteobacteria Bradymonadales Persicimonas caeni WP 141200527.1
BacClassIX inIV Pseudomonadati Myxococcota Polyangia Polyangiales Kofleriaceae bacterium isolate SMAG U14421 iso2
BacClassIX inIV Pseudomonadati PVC Planctomycetota Planctomycetota bacterium isolate ACE NR PLAN1 iso2
BacClassIX inIV Pseudomonadati PVC Planctomycetota Planctomycetota bacterium isolate UBA8742 iso2
BacClassIX inIV Pseudomonadati PVC Planctomycetota Planctomycetota bacterium isolate ABC1.64 iso1
BacClassIX Pseudomonadati Myxococcota Polyangia Haliangiales Haliangium sp. GCF 050303215.1 WP 428268216.1
BacClassIX inIV Pseudomonadati Myxococcota Polyangia Polyangiales Kofleriaceae bacterium isolate SMAG U6175 iso2
BacClassIX inIV Pseudomonadati Myxococcota Polyangia Polyangiales Kofleriaceae bacterium isolate SMAG U13657 iso2
BacClassIX inIV Pseudomonadati Myxococcota Polyangia Polyangiales Kofleriaceae bacterium isolate H4 MYX2 iso2
BacClassIX inIV Pseudomonadati Myxococcota Polyangia Polyangiales Kofleriaceae bacterium isolate MAG 26 iso2
BacClassIX inIV Pseudomonadati Myxococcota Polyangia Polyangiales Kofleriaceae bacterium isolate SMAG U14365 iso2
BacClassIX Pseudomonadati Myxococcota Myxococcia Myxococcales Myxococcales bacterium isolate Hirt 18 Q3 R61 65 BATAC.430 MBK9030995.1
BacClassIX inIV Pseudomonadati Myxococcota Polyangia Polyangiales Kofleriaceae bacterium isolate ABC1.117 iso2
BacClassIX Pseudomonadati Myxococcota Polyangia Haliangiales Kofleriaceae bacterium isolate ABC1.117 iso1 MEZ4400270.1
BacClassIX inIV Pseudomonadati Myxococcota Polyangia Polyangiales Kofleriaceae bacterium isolate Go SlAct bin 429 iso3
BacClassIX Pseudomonadati Myxococcota Myxococcia Myxococcales Myxococcales bacterium isolate Bjer 18 Q3 R1 45 MAXAC.048 iso2 MBK7073027.1
BacClassIX inIV Pseudomonadati Myxococcota Polyangia Polyangiales Kofleriaceae bacterium isolate HK STAS MYXO 12 iso2
BacClassIX inIV Pseudomonadati Myxococcota Polyangia Polyangiales Kofleriaceae bacterium isolate BC1.218 iso2
BacClassIX inIV Pseudomonadati Myxococcota Polyangia Polyangiales Kofleriaceae bacterium isolate AMR MDS 3354 iso2
BacClassIX inIV Pseudomonadati Myxococcota Polyangia Polyangiales Kofleriaceae bacterium isolate SMAG U2250 iso2
BacClassIX Pseudomonadati Myxococcota Polyangia Haliangiales Kofleriaceae bacterium isolate cluster3 bin.123 MEO8549179.1
BacClassIX inIV Pseudomonadati Myxococcota Polyangia Polyangiales Kofleriaceae bacterium isolate SMAG U5105 iso2
BacClassIX inIV Pseudomonadati Myxococcota Polyangia Polyangiales Kofleriaceae bacterium isolate SMAG U5129 iso2
BacClassIX inIV Pseudomonadati Myxococcota Polyangia Polyangiales Kofleriaceae bacterium isolate SMAG U4851 iso2
BacClassIX inIV Pseudomonadati Myxococcota Polyangia Polyangiales Kofleriaceae bacterium isolate SMAG U5072 iso2
BacClassIX inIV Pseudomonadati Myxococcota Polyangia Polyangiales Kofleriaceae bacterium isolate SMAG U15125 iso1
BacClassIX Pseudomonadati Myxococcota Myxococcia Myxococcales Myxococcales bacterium isolate RYN 366 MDB4955360.1
BacClassIX inIV Pseudomonadati Myxococcota Polyangia Polyangiales Kofleriaceae bacterium isolate SMAG U156 iso2
BacClassIX inIV Pseudomonadati Myxococcota Polyangia Polyangiales Kofleriaceae bacterium isolate Bin8.14 iso2
BacClassIX Pseudomonadati Myxococcota Polyangia Haliangiales Kofleriaceae bacterium isolate CA 2019 TPBS 15 iso2 MGE0870308.1
BacClassIX inIV Pseudomonadati Myxococcota Polyangia Polyangiales Kofleriaceae bacterium isolate SMAG U4951 iso2
BacClassIX inIV Pseudomonadati Myxococcota Polyangia Polyangiales Kofleriaceae bacterium isolate F1F2.bin.256 iso2
BacClassIX Pseudomonadati Myxococcota Myxococcia Myxococcales Myxococcales bacterium isolate Vibo 18 Q3 R45 57 MAXAC.190 MBL0215332.1
BacClassIX Pseudomonadati Myxococcota Myxococcia Myxococcales Myxococcales bacterium isolate RYN 365 iso1 MDB4960613.1
BacClassIX inIV Pseudomonadati Myxococcota Polyangia Polyangiales Kofleriaceae bacterium isolate SMAG U2472 iso2
BacClassIX inIV Pseudomonadati Myxococcota Polyangia Polyangiales Kofleriaceae bacterium isolate cluster3 bin.111 iso2
BacClassIX Pseudomonadati Myxococcota unclassified Myxococcota Myxococcota bacterium isolate MSU bin107 MDQ3364102.1
BacClassIX inIV Pseudomonadati Myxococcota Polyangia Polyangiales Kofleriaceae bacterium isolate SMAG U13378 iso2
BacClassIX inIV Pseudomonadati Myxococcota Polyangia Polyangiales Kofleriaceae bacterium isolate SMAG U16122 iso1
BacClassIX inIV Pseudomonadati Myxococcota Polyangia Polyangiales Kofleriaceae bacterium isolate SMAG U13043 iso2
BacClassIX inIV Pseudomonadati Myxococcota Polyangia Polyangiales Kofleriaceae bacterium isolate SMAG U27 iso2
BacClassIX inIV Pseudomonadati Myxococcota Polyangia Polyangiales Kofleriaceae bacterium isolate SMAG U15847 iso2
BacClassIX Pseudomonadati Myxococcota Polyangia Haliangiales Kofleriaceae bacterium isolate cluster1 bin.533 MEO7730565.1
BacClassIX Pseudomonadati Myxococcota Myxococcia Myxococcales Myxococcales bacterium isolate ES bin 234 MBC7973360.1
BacClassIX inIV Pseudomonadati Myxococcota Polyangia Polyangiales Kofleriaceae bacterium isolate SMAG U3528 iso2
BacClassIX inIV Pseudomonadati Myxococcota Polyangia Polyangiales Kofleriaceae bacterium isolate SMAG U3158 iso2
BacClassIX inIV Pseudomonadati Myxococcota Polyangia Polyangiales Kofleriaceae bacterium isolate SMAG U5526 iso2
BacClassIX inIV Pseudomonadati Myxococcota Polyangia Polyangiales Kofleriaceae bacterium isolate SMAG U5434 iso2
BacClassIX Pseudomonadati Pseudomonadota Gammaproteobacteria Pseudomonadales Pseudomonadales bacterium isolate CA 2019 TPBS 177 iso2 MGE0625758.1
BacClassIX Pseudomonadati Pseudomonadota Gammaproteobacteria Chromatiales Elongatibacter sediminis strain CH 27 iso3 WP 354696155.1
BacClassIX Pseudomonadati Pseudomonadota Gammaproteobacteria Woeseiales Woeseiaceae bacterium isolate AMR MDS 0957 HNP35815.1
BacClassIX Pseudomonadati Pseudomonadota Gammaproteobacteria Pseudomonadales Permianibacter fluminis WP 172244216.1
BacClassIX Pseudomonadati Pseudomonadota Gammaproteobacteria Chromatiales Nitrococcus mobilis isolate mag.22 iso1 MCO6439546.1
BacClassIX Pseudomonadati Pseudomonadota Gammaproteobacteria Nevskiales Sinimarinibacterium sp. isolate SO 2017 GW1 65 iso1 MFA5937784.1
BacClassIX Pseudomonadati Pseudomonadota Gammaproteobacteria Lysobacterales Lysobacterales bacterium isolate MO 188.B40 iso1 MDJ0653825.1
BacClassIX Pseudomonadati Pseudomonadota Gammaproteobacteria Lysobacterales Thermomonas sp. GCF 041909715.1 WP 374357056.1
BacClassIX Pseudomonadati Pseudomonadota Gammaproteobacteria Chromatiales Elongatibacter sediminis strain CH 27 iso1 WP 354694511.1
BacClassIX Pseudomonadati Spirochaetota Spirochaetia Leptospirales Leptospira gorisiae XDD49915.1
BacClassIX Pseudomonadati Pseudomonadota Gammaproteobacteria Legionellales Legionellaceae bacterium isolate bin bo oilr1.011 iso1 MDF1683968.1
BacClassIX Pseudomonadati Pseudomonadota Gammaproteobacteria Legionellales Legionellaceae sp. isolate bin em oilr2.032 MDF1827296.1
BacClassIX Pseudomonadati Pseudomonadota Gammaproteobacteria Legionellales Legionella sp. isolate CG 2015 02 43 12 NCT56980.1
BacClassIX Pseudomonadati Pseudomonadota Gammaproteobacteria Legionellales Legionellales bacterium isolate SO 2017 GW1 269 iso1 MFA6037141.1
BacClassIX Pseudomonadati Pseudomonadota Gammaproteobacteria Legionellales Legionellales bacterium isolate MAG 29 iso1 NNM59650.1
BacClassIX Pseudomonadati Pseudomonadota Gammaproteobacteria Legionellales Legionella drancourtii strain LLAP12 iso2 WP 050805558.1
BacClassIX Pseudomonadati Pseudomonadota Gammaproteobacteria Legionellales Legionellaceae bacterium isolate bin ce oilr1.001 MDF1678814.1
BacClassIX Pseudomonadati Pseudomonadota Gammaproteobacteria Legionellales Legionella moravica WP 028383593.1
BacClassIX Pseudomonadati Pseudomonadota Gammaproteobacteria Legionellales Legionella quateirensis strain ATCC 49507 iso2 WP 058475017.1
BacClassIX Pseudomonadati Pseudomonadota Gammaproteobacteria Legionellales Legionella bononiensis WP 203112640.1
BacClassIX Pseudomonadati Pseudomonadota Gammaproteobacteria Pseudomonadales Pseudomonadales bacterium isolate DC out29 MBV1919389.1
BacClassIX Pseudomonadati Pseudomonadota Gammaproteobacteria Oceanospirillales Oleiphilaceae bacterium isolate 3300055726 46 MFT5717289.1
BacClassIX Pseudomonadati Gammaproteobacteria Oceanospirillales Oceanospirillaceae Thalassolituus sp. isolate SIP 700 Bin15 iso2 MEQ3693654.1
BacClassIX Pseudomonadati Gammaproteobacteria Oceanospirillales Oceanospirillaceae Thalassolituus sp. isolate SIP 700 Bin15 iso1 MEK9712515.1
BacClassIX Pseudomonadati Gammaproteobacteria Oceanospirillales Oceanospirillaceae Oleispira sp. isolate NORP253 iso2 MBL4797782.1
BacClassIX Pseudomonadati Gammaproteobacteria Oceanospirillales Oceanospirillaceae Oleispira sp. isolate 9NakV 01 MEH6448603.1
BacClassIX Pseudomonadati Gammaproteobacteria Oceanospirillales Oceanospirillaceae Oleispira sp. isolate 3300047667 36 MFT6113128.1
BacClassIX Pseudomonadati Pseudomonadota Gammaproteobacteria Oceanospirillales Bacterioplanoides sp. SCSIO 12839 WP 255857419.1
BacClassIX Pseudomonadati Pseudomonadota Gammaproteobacteria Oceanospirillales Bacterioplanoides sp. GCF 965219715.1 WP 420589803.1
BacClassIX Pseudomonadati Pseudomonadota Gammaproteobacteria Oceanospirillales Bacterioplanoides pacificum WP 376864407.1
BacClassIX Pseudomonadati Pseudomonadota Gammaproteobacteria Oceanospirillales Bacterioplanes sp. isolate Kmv21 MBE0482575.1
BacClassIX Pseudomonadati Gammaproteobacteria Oceanospirillales Oceanospirillaceae Venatoribacter cucullus WP 228346106.1
BacClassIX Pseudomonadati Gammaproteobacteria Oceanospirillales Oceanospirillaceae Oceanospirillaceae bacterium isolate CPC2 MAD44244.1
BacClassIX Pseudomonadati Gammaproteobacteria Oceanospirillales Oceanospirillaceae Thalassolituus marinus WP 225675706.1
BacClassIX Pseudomonadati Gammaproteobacteria Oceanospirillales Oceanospirillaceae Thalassolituus sp. LLYu03 WP 430461837.1

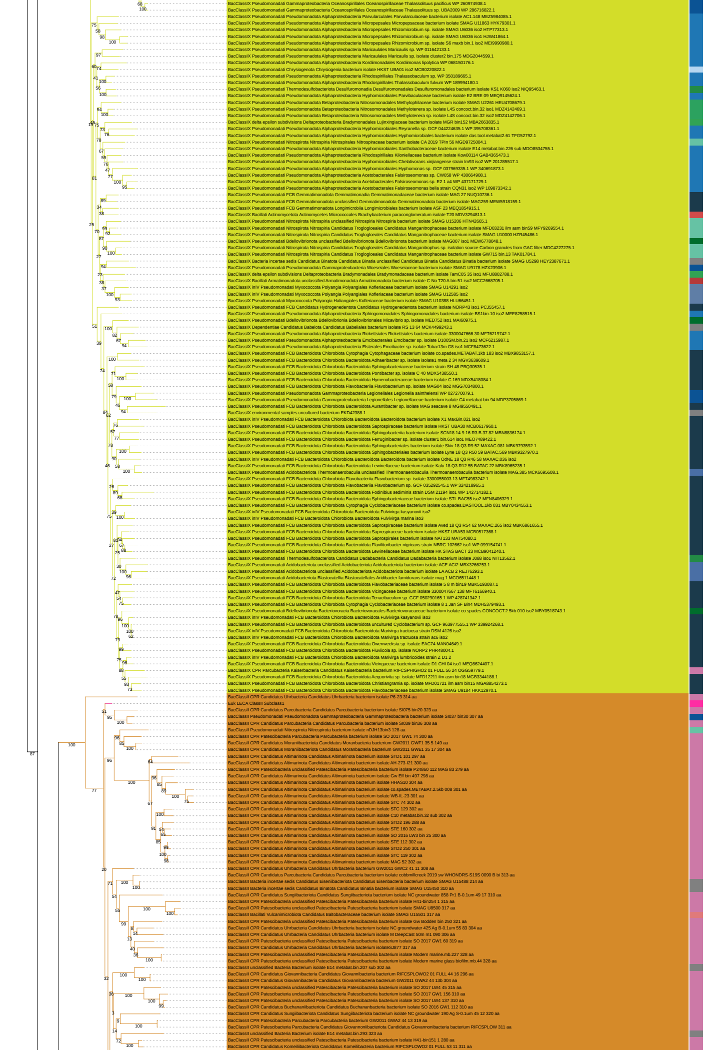
BacClassIX Pseudomonadati Gammaproteobacteria Oceanospirillales Oceanospirillaceae Thalassolituus pacificus WP 260974938.1
BacClassIX Pseudomonadati Gammaproteobacteria Oceanospirillales Oceanospirillaceae Thalassolituus sp. UBA2009 WP 286716822.1
BacClassIX Pseudomonadati Pseudomonadota Alphaproteobacteria Parvularculales Parvularculaceae bacterium isolate AC1.148 MEZ5984085.1
BacClassIX Pseudomonadati Pseudomonadota Alphaproteobacteria Micropepsales Micropepsaceae bacterium isolate SMAG U11863 HYK79301.1
BacClassIX Pseudomonadati Pseudomonadota Alphaproteobacteria Micropepsales Rhizomicrobium sp. isolate SMAG U6036 iso2 HTP77313.1
BacClassIX Pseudomonadati Pseudomonadota Alphaproteobacteria Micropepsales Rhizomicrobium sp. isolate SMAG U6036 iso1 HJW41864.1
BacClassIX Pseudomonadati Pseudomonadota Alphaproteobacteria Micropepsales Rhizomicrobium sp. isolate S6 maxb bin.1 iso2 MEI9990980.1
BacClassIX Pseudomonadati Pseudomonadota Alphaproteobacteria Maricaulales Maricaulis sp. WP 011642133.1
BacClassIX Pseudomonadati Pseudomonadota Alphaproteobacteria Maricaulales Maricaulis sp. isolate cluster2 bin.175 MDG2044599.1
BacClassIX Pseudomonadati Pseudomonadota Alphaproteobacteria Kordiimonadales Kordiimonas lipolytica WP 068150176.1
BacClassIX Pseudomonadati Chrysiogenota Chrysiogenia bacterium isolate HKST UBA01 iso2 MCB0220822.1
BacClassIX Pseudomonadati Pseudomonadota Alphaproteobacteria Rhodospirillales Thalassobaculum sp. WP 350189665.1
BacClassIX Pseudomonadati Pseudomonadota Alphaproteobacteria Rhodospirillales Thalassobaculum fulvum WP 189994180.1
BacClassIX Pseudomonadati Thermodesulfobacteriota Desulfuromonadia Desulfuromonadales Desulfuromonadales bacterium isolate KS1 K060 iso2 NIQ95463.1
BacClassIX Pseudomonadati Pseudomonadota Alphaproteobacteria Hyphomicrobiales Parvibaculaceae bacterium isolate E2 BRE 09 MEQ9145624.1
BacClassIX Pseudomonadati Pseudomonadota Betaproteobacteria Nitrosomonadales Methylophilaceae bacterium isolate SMAG U2261 HEU4708679.1
BacClassIX Pseudomonadati Pseudomonadota Betaproteobacteria Nitrosomonadales Methylotenera sp. isolate L4S concoct.bin.32 iso1 MDZ4142469.1
BacClassIX Pseudomonadati Pseudomonadota Betaproteobacteria Nitrosomonadales Methylotenera sp. isolate L4S concoct.bin.32 iso2 MDZ4142706.1
BacClassIX delta epsilon subdivisions Deltaproteobacteria Bradymonadales Lujinxingiaceae bacterium isolate MGR bin152 MBA2663835.1
BacClassIX Pseudomonadati Pseudomonadota Alphaproteobacteria Hyphomicrobiales Reyranella sp. GCF 044224635.1 WP 395708361.1
BacClassIX Pseudomonadati Pseudomonadota Alphaproteobacteria Hyphomicrobiales Hyphomicrobiales bacterium isolate das tool.metabat2.61 TFG52792.1
BacClassIX Pseudomonadati Nitrospirota Nitrospiria Nitrospirales Nitrospiraceae bacterium isolate CA 2019 TPIn 56 MGD9725004.1
BacClassIX Pseudomonadati Pseudomonadota Alphaproteobacteria Hyphomicrobiales Xanthobacteraceae bacterium isolate E14 metabat.bin.226 sub MDO8534755.1
BacClassIX Pseudomonadati Pseudomonadota Alphaproteobacteria Rhodospirillales Kiloniellaceae bacterium isolate Kow00114 GAB4365473.1
BacClassIX Pseudomonadati Pseudomonadota Alphaproteobacteria Hyphomicrobiales Chelativorans xinjiangense strain lm93 iso2 WP 201285517.1
BacClassIX Pseudomonadati Pseudomonadota Alphaproteobacteria Hyphomicrobiales Hyphomonas sp. GCF 037969335.1 WP 340691873.1
BacClassIX Pseudomonadati Pseudomonadota Alphaproteobacteria Acetobacterales Falsiroseomonas sp. CW058 WP 430664908.1
BacClassIX Pseudomonadati Pseudomonadota Alphaproteobacteria Acetobacterales Falsiroseomonas sp. E2 1 a4 WP 437171729.1
BacClassIX Pseudomonadati Pseudomonadota Alphaproteobacteria Acetobacterales Falsiroseomonas bella strain CQN31 iso2 WP 109873342.1
BacClassIX Pseudomonadati FCB Gemmatimonadota Gemmatimonadia Gemmatimonadaceae bacterium isolate MAG 27 NUQ10736.1
BacClassIX Pseudomonadati FCB Gemmatimonadota unclassified Gemmatimonadota Gemmatimonadota bacterium isolate MAG259 MEW5918159.1
BacClassIX Pseudomonadati FCB Gemmatimonadota Longimicrobiia Longimicrobiales bacterium isolate ASF 23 MEQ1854915.1
BacClassIX Bacillati Actinomycetota Actinomycetes Micrococcales Brachybacterium paraconglomeratum isolate T20 MDV3294813.1
BacClassIX Pseudomonadati Nitrospirota Nitrospiria unclassified Nitrospiria Nitrospiria bacterium isolate SMAG U15206 HTN42665.1
BacClassIX Pseudomonadati Nitrospirota Nitrospiria Candidatus Troglogloeales Candidatus Manganitrophaceae bacterium isolate MFD03231 ilm asm bin59 MFY9269554.1
BacClassIX Pseudomonadati Nitrospirota Nitrospiria Candidatus Troglogloeales Candidatus Manganitrophaceae bacterium isolate SMAG U10000 HZR45486.1
BacClassIX Pseudomonadati Bdellovibrionota unclassified Bdellovibrionota Bdellovibrionota bacterium isolate MAG007 iso1 MEW6778048.1
BacClassIX Pseudomonadati Nitrospirota Nitrospiria Candidatus Troglogloeales Candidatus Manganitrophus sp. isolation source Carbon granules from GAC filter MDC4227275.1
BacClassIX Pseudomonadati Nitrospirota Nitrospiria Candidatus Troglogloeales Candidatus Manganitrophaceae bacterium isolate GW715 bin.13 TAK01784.1
BacClassIX Bacteria incertae sedis Candidatus Binatota Candidatus Binatia unclassified Candidatus Binatia Candidatus Binatia bacterium isolate SMAG U5298 HEY2387671.1
BacClassIX Pseudomonadati Pseudomonadota Gammaproteobacteria Woeseiales Woeseiaceae bacterium isolate SMAG U9178 HZX23906.1
BacClassIX delta epsilon subdivisions Deltaproteobacteria Bradymonadales Bradymonadaceae bacterium isolate TamC05 35 iso1 MFU8802788.1
BacClassIX Bacillati Armatimonadota unclassified Armatimonadota Armatimonadota bacterium isolate C No T20 A bin.51 iso2 MCC2668705.1
BacClassIX inIV Pseudomonadati Myxococcota Polyangia Polyangiales Kofleriaceae bacterium isolate SMAG U14291 iso2
BacClassIX inIV Pseudomonadati Myxococcota Polyangia Polyangiales Kofleriaceae bacterium isolate SMAG U12585 iso2
BacClassIX Pseudomonadati Myxococcota Polyangia Haliangiales Kofleriaceae bacterium isolate SMAG U10388 HLU66451.1
BacClassIX Pseudomonadati FCB Candidatus Hydrogenedentota Candidatus Hydrogenedentota bacterium isolate NORP43 iso1 PCJ55457.1
BacClassIX Pseudomonadati Pseudomonadota Alphaproteobacteria Sphingomonadales Sphingomonadales bacterium isolate BS1bin.10 iso2 MEE8258515.1
BacClassIX Pseudomonadati Bdellovibrionota Bdellovibrionia Bdellovibrionales Micavibrio sp. isolate MED752 iso1 MAI60975.1
BacClassIX Dependentiae Candidatus Babelota Candidatus Babeliales bacterium isolate RS 13 64 MCK4499243.1
BacClassIX Pseudomonadati Pseudomonadota Alphaproteobacteria Rickettsiales Rickettsiales bacterium isolate 3300047666 30 MFT6219742.1
BacClassIX Pseudomonadati Pseudomonadota Alphaproteobacteria Emcibacterales Emcibacter sp. isolate D100SM.bin.21 iso2 MCF6215987.1
BacClassIX Pseudomonadati Pseudomonadota Alphaproteobacteria Elsterales Emcibacter sp. isolate Tobar13m G8 iso1 MCF8473622.1
BacClassIX Pseudomonadati FCB Bacteroidota Chlorobiota Cytophagia Cytophagaceae bacterium isolate co.spades.METABAT.1kb 183 iso2 MBX9853157.1
BacClassIX Pseudomonadati FCB Bacteroidota Chlorobiota Bacteroidota Adhaeribacter sp. isolate isolate1 meta 2 34 MGV3639609.1
BacClassIX Pseudomonadati FCB Bacteroidota Chlorobiota Bacteroidota Sphingobacteriaceae bacterium strain SH 48 PBQ30535.1
BacClassIX Pseudomonadati FCB Bacteroidota Chlorobiota Bacteroidota Pontibacter sp. isolate C 40 MDX5438550.1
BacClassIX Pseudomonadati FCB Bacteroidota Chlorobiota Bacteroidota Hymenobacteraceae bacterium isolate C 169 MDX5418084.1
BacClassIX Pseudomonadati FCB Bacteroidota Chlorobiota Flavobacteriia Flavobacterium sp. isolate MAG04 iso2 MGG7034800.1
BacClassIX Pseudomonadati Pseudomonadota Gammaproteobacteria Legionellales Legionella sainthelensi WP 027270079.1
BacClassIX Pseudomonadati Pseudomonadota Gammaproteobacteria Legionellales Legionellaceae bacterium isolate C4 metabat.bin.94 MDP3705869.1
BacClassIX Pseudomonadati FCB Bacteroidota Chlorobiota Bacteroidota Aurantibacter sp. isolate MAG seacave 8 MGI9550491.1
BacClassIX environmental samples uncultured bacterium EKD42388.1
BacClassIX inIV Pseudomonadati FCB Bacteroidota Chlorobiota Bacteroidota Bacteroidota bacterium isolate X1 MaxBin.021 iso2
BacClassIX Pseudomonadati FCB Bacteroidota Chlorobiota Bacteroidota Saprospiraceae bacterium isolate HKST UBA30 MCB0617960.1
BacClassIX Pseudomonadati FCB Bacteroidota Chlorobiota Bacteroidota Sphingobacteriia bacterium isolate SCN18 14 9 16 R3 B 37 82 MBN8836174.1
BacClassIX Pseudomonadati FCB Bacteroidota Chlorobiota Bacteroidota Ferruginibacter sp. isolate cluster1 bin.614 iso1 MEO7489422.1
BacClassIX Pseudomonadati FCB Bacteroidota Chlorobiota Bacteroidota Sphingobacteriales bacterium isolate Skiv 18 Q3 R9 52 MAXAC.081 MBK9793592.1
BacClassIX Pseudomonadati FCB Bacteroidota Chlorobiota Bacteroidota Sphingobacteriales bacterium isolate Lyne 18 Q3 R50 59 BATAC.569 MBK9327970.1
BacClassIX inIV Pseudomonadati FCB Bacteroidota Chlorobiota Bacteroidota Bacteroidota bacterium isolate OdNE 18 Q3 R46 58 MAXAC.036 iso2
BacClassIX Pseudomonadati FCB Bacteroidota Chlorobiota Bacteroidota Lewinellaceae bacterium isolate Kalu 18 Q3 R12 55 BATAC.22 MBK8965235.1
BacClassIX Pseudomonadati Acidobacteriota Thermoanaerobaculia unclassified Thermoanaerobaculia Thermoanaerobaculia bacterium isolate MAG.385 MCK6695608.1
BacClassIX Pseudomonadati FCB Bacteroidota Chlorobiota Flavobacteriia Flavobacterium sp. isolate 3300055003 13 MFT4983242.1
BacClassIX Pseudomonadati FCB Bacteroidota Chlorobiota Flavobacteriia Flavobacterium sp. GCF 035292545.1 WP 324218965.1
BacClassIX Pseudomonadati FCB Bacteroidota Chlorobiota Bacteroidota Fodinibius sediminis strain DSM 21194 iso1 WP 142714182.1
BacClassIX Pseudomonadati FCB Bacteroidota Chlorobiota Bacteroidota Sphingobacteriaceae bacterium isolate STL BAC55 iso2 MFN8406329.1
BacClassIX Pseudomonadati FCB Bacteroidota Chlorobiota Cytophagia Cyclobacteriaceae bacterium isolate co.spades.DASTOOL.1kb 031 MBY0434553.1
BacClassIX inIV Pseudomonadati FCB Bacteroidota Chlorobiota Bacteroidota Fulvivirga kasyanovii iso2
BacClassIX inIV Pseudomonadati FCB Bacteroidota Chlorobiota Bacteroidota Fulvivirga marina iso3
BacClassIX Pseudomonadati FCB Bacteroidota Chlorobiota Bacteroidota Saprospiraceae bacterium isolate Aved 18 Q3 R54 62 MAXAC.265 iso2 MBK6861655.1
BacClassIX Pseudomonadati FCB Bacteroidota Chlorobiota Bacteroidota Saprospiraceae bacterium isolate HKST UBA53 MCB0517368.1
BacClassIX Pseudomonadati FCB Bacteroidota Chlorobiota Bacteroidota Saprospirales bacterium isolate NAT133 MAT54080.1
BacClassIX Pseudomonadati FCB Bacteroidota Chlorobiota Bacteroidota Flavilitoribacter nigricans strain NBRC 102662 iso1 WP 099154741.1
BacClassIX Pseudomonadati FCB Bacteroidota Chlorobiota Bacteroidota Lewinellaceae bacterium isolate HK STAS BACT 23 MCB9041240.1
BacClassIX Pseudomonadati Thermodesulfobacteriota Candidatus Dadabacteria Candidatus Dadabacteria bacterium isolate J088 iso1 NIT13562.1
BacClassIX Pseudomonadati Acidobacteriota unclassified Acidobacteriota Acidobacteriota bacterium isolate ACE ACI2 MBX3266253.1
BacClassIX Pseudomonadati Acidobacteriota unclassified Acidobacteriota Acidobacteriota bacterium isolate LA ACB 2 REJ76293.1
BacClassIX Pseudomonadati Acidobacteriota Blastocatellia Blastocatellales Aridibacter famidurans isolate mag.1 MCO6511448.1
BacClassIX Pseudomonadati FCB Bacteroidota Chlorobiota Bacteroidota Flavobacteriaceae bacterium isolate 5 8 m bin19 MBK5193087.1
BacClassIX Pseudomonadati FCB Bacteroidota Chlorobiota Bacteroidota Vicingaceae bacterium isolate 3300047667 138 MFT6166940.1
BacClassIX Pseudomonadati FCB Bacteroidota Chlorobiota Bacteroidota Tenacibaculum sp. GCF 050290165.1 WP 428741342.1
BacClassIX Pseudomonadati FCB Bacteroidota Chlorobiota Cytophagia Cyclobacteriaceae bacterium isolate 8 1 Jan SF Bin4 MDH5379493.1
BacClassIX Pseudomonadati Bdellovibrionota Bacteriovoracia Bacteriovoracales Bacteriovoracaceae bacterium isolate co.spades.CONCOCT.2.5kb 010 iso2 MBY0518743.1
BacClassIX inIV Pseudomonadati FCB Bacteroidota Chlorobiota Bacteroidota Fulvivirga kasyanovii iso3
BacClassIX Pseudomonadati FCB Bacteroidota Chlorobiota Bacteroidota uncultured Cyclobacterium sp. GCF 963977555.1 WP 339924268.1
BacClassIX inIV Pseudomonadati FCB Bacteroidota Chlorobiota Bacteroidota Marivirga tractuosa strain DSM 4126 iso2
BacClassIX inIV Pseudomonadati FCB Bacteroidota Chlorobiota Bacteroidota Marivirga tractuosa strain ac6 iso2
BacClassIX Pseudomonadati FCB Bacteroidota Chlorobiota Bacteroidota Owenweeksia sp. isolate EAC74 MAN04649.1
BacClassIX Pseudomonadati FCB Bacteroidota Chlorobiota Bacteroidota Fluviicola sp. isolate NORP2 PHR48004.1
BacClassIX inIV Pseudomonadati FCB Bacteroidota Chlorobiota Bacteroidota Marivirga lumbricoides strain Z D1 2
BacClassIX Pseudomonadati FCB Bacteroidota Chlorobiota Bacteroidota Vicingaceae bacterium isolate D1 CHI 04 iso1 MEQ8624407.1
BacClassIX CPR Parcubacteria Kaiserbacteria Candidatus Kaiserbacteria bacterium RIFCSPHIGHO2 01 FULL 56 24 OGG59779.1
BacClassIX Pseudomonadati FCB Bacteroidota Chlorobiota Bacteroidota Aequorivita sp. isolate MFD12211 ilm asm bin18 MGB3344188.1
BacClassIX Pseudomonadati FCB Bacteroidota Chlorobiota Bacteroidota Christiangramia sp. isolate MFD01721 ilm asm bin15 MGA8854273.1
BacClassIX Pseudomonadati FCB Bacteroidota Chlorobiota Bacteroidota Flavobacteriaceae bacterium isolate SMAG U9184 HKK12970.1
BacClassII CPR Candidatus Uhrbacteria Candidatus Uhrbacteria bacterium isolate P6-23 314 aa
Euk LECA ClassII Subclass1
BacClassII CPR Candidatus Parcubacteria Candidatus Parcubacteria bacterium isolate SI075 bin20 323 aa
BacClassII Pseudomonadati Pseudomonadota Gammaproteobacteria Gammaproteobacteria bacterium isolate SI037 bin30 307 aa
BacClassII CPR Candidatus Parcubacteria Candidatus Parcubacteria bacterium isolate SI039 bin36 308 aa
BacClassII Pseudomonadati Nitrospirota Nitrospirota bacterium isolate nDJH13bin3 128 aa
BacClassII CPR Patescibacteria Parcubacteria Parcubacteria bacterium isolate SO 2017 GW1 74 300 aa
BacClassII CPR Candidatus Moraniibacteriota Candidatus Moranbacteria bacterium GW2011 GWF1 35 5 149 aa
BacClassII CPR Candidatus Moraniibacteriota Candidatus Moranbacteria bacterium GW2011 GWE1 35 17 304 aa
BacClassII CPR Candidatus Altimarinota Candidatus Altimarinota bacterium isolate STD1 101 297 aa
BacClassII CPR Candidatus Altimarinota Candidatus Altimarinota bacterium isolate AH-273-I21 300 aa
BacClassII CPR Patescibacteria unclassified Patescibacteria Patescibacteria bacterium isolate P24860 112 MAG 83 279 aa
BacClassII CPR Candidatus Altimarinota Candidatus Altimarinota bacterium isolate Gw Eff bin 497 298 aa
BacClassII CPR Candidatus Altimarinota Candidatus Altimarinota bacterium isolate HHAS10 304 aa
BacClassII CPR Candidatus Altimarinota Candidatus Altimarinota bacterium isolate co.spades.METABAT.2.5kb 008 301 aa
BacClassII CPR Candidatus Altimarinota Candidatus Altimarinota bacterium isolate WB-IL-23 301 aa
BacClassII CPR Candidatus Altimarinota Candidatus Altimarinota bacterium isolate STC 74 302 aa
BacClassII CPR Candidatus Altimarinota Candidatus Altimarinota bacterium isolate STC 129 302 aa
BacClassII CPR Candidatus Altimarinota Candidatus Altimarinota bacterium isolate C10 metabat.bin.32 sub 302 aa
BacClassII CPR Candidatus Altimarinota Candidatus Altimarinota bacterium isolate STD2 196 288 aa
BacClassII CPR Candidatus Altimarinota Candidatus Altimarinota bacterium isolate STE 160 302 aa
BacClassII CPR Candidatus Altimarinota Candidatus Altimarinota bacterium isolate SO 2016 LW3 bin 25 300 aa
BacClassII CPR Candidatus Altimarinota Candidatus Altimarinota bacterium isolate STE 112 302 aa
BacClassII CPR Candidatus Altimarinota Candidatus Altimarinota bacterium isolate STD2 250 301 aa
BacClassII CPR Candidatus Altimarinota Candidatus Altimarinota bacterium isolate STC 119 302 aa
BacClassII CPR Candidatus Altimarinota Candidatus Altimarinota bacterium isolate MAG 52 302 aa
BacClassII CPR Candidatus Uhrbacteria Candidatus Uhrbacteria bacterium GW2011 GWC2 41 11 308 aa
BacClassII CPR Candidatus Parcubacteria Candidatus Parcubacteria bacterium isolate cobbmillcreek 2019 sw WHONDRS-S19S 0090 B bi 313 aa
BacClassII Bacteria incertae sedis Candidatus Eiseniibacteriota Candidatus Eisenbacteria bacterium isolate SMAG U15488 214 aa
BacClassII Bacteria incertae sedis Candidatus Binatota Candidatus Binatia bacterium isolate SMAG U15450 310 aa
BacClassII CPR Candidatus Sungiibacteriota Candidatus Sungiibacteriota bacterium isolate NC groundwater 858 Pr1 B-0.1um 49 17 310 aa
BacClassII CPR Patescibacteria unclassified Patescibacteria Patescibacteria bacterium isolate H41-bin254 1 315 aa
BacClassII CPR Patescibacteria unclassified Patescibacteria Patescibacteria bacterium isolate SMAG U8500 317 aa
BacClassII Bacillati Vulcanimicrobiota Candidatus Baltobacteraceae bacterium isolate SMAG U15501 317 aa
BacClassII CPR Patescibacteria unclassified Patescibacteria Patescibacteria bacterium isolate Gw Bodden bin 250 321 aa
BacClassII CPR Candidatus Uhrbacteria Candidatus Uhrbacteria bacterium isolate NC groundwater 425 Ag B-0.1um 55 83 304 aa
BacClassII CPR Candidatus Uhrbacteria Candidatus Uhrbacteria bacterium isolate M DeepCast 50m m1 090 306 aa
BacClassII CPR Patescibacteria unclassified Patescibacteria Patescibacteria bacterium isolate SO 2017 GW1 60 319 aa
BacClassII CPR Candidatus Uhrbacteria Candidatus Uhrbacteria bacterium isolateSJ877 317 aa
BacClassII CPR Patescibacteria unclassified Patescibacteria Patescibacteria bacterium isolate Modern marine.mb.227 328 aa
BacClassII CPR Patescibacteria unclassified Patescibacteria Patescibacteria bacterium isolate Modern marine glass biofilm.mb.44 328 aa
BacClassII unclassified Bacteria Bacterium isolate E14 metabat.bin.207 sub 302 aa
BacClassII CPR Candidatus Giovannibacteria Candidatus Giovannibacteria bacterium RIFCSPLOWO2 01 FULL 44 16 296 aa
BacClassII CPR Candidatus Giovannibacteria Candidatus Giovannibacteria bacterium GW2011 GWA2 44 13b 304 aa
BacClassII CPR Patescibacteria unclassified Patescibacteria Patescibacteria bacterium isolate SO 2017 LW4 45 315 aa
BacClassII CPR Patescibacteria unclassified Patescibacteria Patescibacteria bacterium isolate SO 2017 GW1 156 310 aa
BacClassII CPR Patescibacteria unclassified Patescibacteria Patescibacteria bacterium isolate SO 2017 LW4 137 310 aa
BacClassII CPR Candidatus Buchananiibacteriota Candidatus Buchananbacteria bacterium isolate SO 2016 GW1 112 310 aa
BacClassII CPR Candidatus Sungiibacteriota Candidatus Sungiibacteriota bacterium isolate NC groundwater 190 Ag S-0.1um 45 12 320 aa
BacClassII CPR Patescibacteria Parcubacteria Parcubacteria bacterium GW2011 GWA2 44 13 319 aa
BacClassII CPR Patescibacteria Parcubacteria Candidatus Giovannoniibacteriota Candidatus Giovannonibacteria bacterium RIFCSPLOW 311 aa
BacClassII unclassified Bacteria Bacterium isolate E14 metabat.bin.293 323 aa
BacClassII CPR Patescibacteria unclassified Patescibacteria Patescibacteria bacterium isolate H41-bin151 1 280 aa
BacClassII CPR Candidatus Komeiliibacteriota Candidatus Komeilibacteria bacterium RIFCSPLOWO2 01 FULL 53 11 311 aa

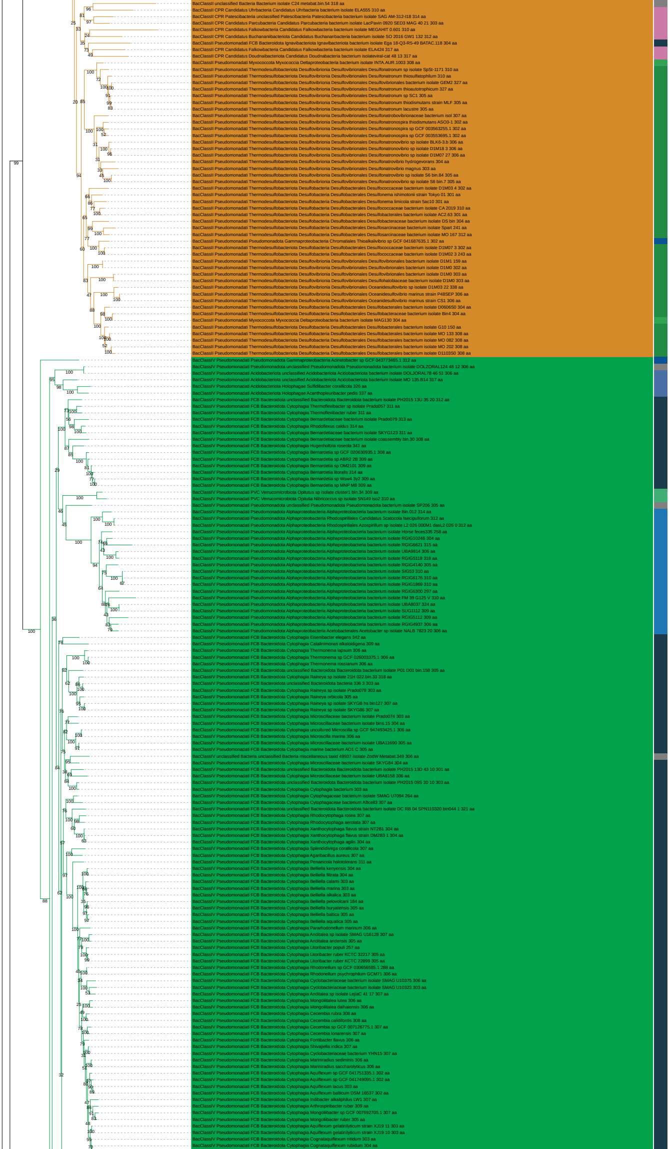
BacClassII unclassified Bacteria Bacterium isolate C24 metabat.bin.54 318 aa
BacClassII CPR Candidatus Uhrbacteria Candidatus Uhrbacteria bacterium isolate ELA555 310 aa
BacClassII CPR Patescibacteria unclassified Patescibacteria Patescibacteria bacterium isolate SAG AM-312-I18 314 aa
BacClassII CPR Candidatus Parcubacteria Candidatus Parcubacteria bacterium isolate LacPavin 0920 SED3 MAG 40 21 303 aa
BacClassII CPR Candidatus Falkowbacteria Candidatus Falkowbacteria bacterium isolate MEGAHIT 0.601 310 aa
BacClassII CPR Candidatus Buchananiibacteriota Candidatus Buchananbacteria bacterium isolate SO 2016 GW1 132 312 aa
BacClassII Pseudomonadati FCB Bacteroidota Ignavibacteriota Ignavibacteriota bacterium isolate Ega 18-Q3-R5-49 BATAC.118 304 aa
BacClassII CPR Candidatus Falkowbacteria Candidatus Falkowbacteria bacterium isolate ELA424 317 aa
BacClassII CPR Candidatus Doudnabacteria Candidatus Doudnabacteria bacterium isolateviral-cat 48 13 317 aa
BacClassII Pseudomonadati Myxococcota Myxococcota Deltaproteobacteria bacterium isolate INTA.AUR.1003 308 aa
BacClassII Pseudomonadati Thermodesulfobacteriota Desulfovibrionia Desulfovibrionales Desulfonatronum sp isolate SpSt-1171 310 aa
BacClassII Pseudomonadati Thermodesulfobacteriota Desulfovibrionia Desulfovibrionales Desulfonatronum thiosulfatophilum 310 aa
BacClassII Pseudomonadati Thermodesulfobacteriota Desulfovibrionia Desulfovibrionales Desulfovibrionales bacterium isolate GEM2 327 aa
BacClassII Pseudomonadati Thermodesulfobacteriota Desulfovibrionia Desulfovibrionales Desulfonatronum thioautotrophicum 327 aa
BacClassII Pseudomonadati Thermodesulfobacteriota Desulfovibrionia Desulfovibrionales Desulfonatronum sp SC1 305 aa
BacClassII Pseudomonadati Thermodesulfobacteriota Desulfovibrionia Desulfovibrionales Desulfonatronum thiodismutans strain MLF 305 aa
BacClassII Pseudomonadati Thermodesulfobacteriota Desulfovibrionia Desulfovibrionales Desulfonatronum lacustre 305 aa
BacClassII Pseudomonadati Thermodesulfobacteriota Desulfovibrionia Desulfovibrionales Desulfonatronovibrionaceae bacterium isol 307 aa
BacClassII Pseudomonadati Thermodesulfobacteriota Desulfovibrionia Desulfovibrionales Desulfonatronospira thiodismutans ASO3-1 302 aa
BacClassII Pseudomonadati Thermodesulfobacteriota Desulfovibrionia Desulfovibrionales Desulfonatronospira sp GCF 003563255.1 302 aa
BacClassII Pseudomonadati Thermodesulfobacteriota Desulfovibrionia Desulfovibrionales Desulfonatronospira sp GCF 003553695.1 302 aa
BacClassII Pseudomonadati Thermodesulfobacteriota Desulfovibrionia Desulfovibrionales Desulfonatronovibrio sp isolate BLK6-3.b 306 aa
BacClassII Pseudomonadati Thermodesulfobacteriota Desulfovibrionia Desulfovibrionales Desulfonatronovibrio sp isolate D1M18 3 306 aa
BacClassII Pseudomonadati Thermodesulfobacteriota Desulfovibrionia Desulfovibrionales Desulfonatronovibrio sp isolate D1M07 27 306 aa
BacClassII Pseudomonadati Thermodesulfobacteriota Desulfovibrionia Desulfovibrionales Desulfonatrovibrio hydrogevorans 304 aa
BacClassII Pseudomonadati Thermodesulfobacteriota Desulfovibrionia Desulfovibrionales Desulfonatrovibrio magnus 303 aa
BacClassII Pseudomonadati Thermodesulfobacteriota Desulfovibrionia Desulfovibrionales Desulfonatrovibrio sp isolate S6 bin.84 305 aa
BacClassII Pseudomonadati Thermodesulfobacteriota Desulfovibrionia Desulfovibrionales Desulfonatronovibrio sp isolate S6 bin.7 305 aa
BacClassII Pseudomonadati Thermodesulfobacteriota Desulfobacteria Desulfobacterales Desulfococcaceae bacterium isolate D1M03 4 302 aa
BacClassII Pseudomonadati Thermodesulfobacteriota Desulfobacteria Desulfobacterales Desulfonema ishimotonii strain Tokyo 01 301 aa
BacClassII Pseudomonadati Thermodesulfobacteriota Desulfobacteria Desulfobacterales Desulfonema limicola strain 5ac10 301 aa
BacClassII Pseudomonadati Thermodesulfobacteriota Desulfobacteria Desulfobacterales Desulfococcaceae bacterium isolate CA 2019 310 aa
BacClassII Pseudomonadati Thermodesulfobacteriota Desulfobacteria Desulfobacterales Desulfococcaceae bacterium isolate AC2.63 301 aa
BacClassII Pseudomonadati Thermodesulfobacteriota Desulfobacteria Desulfobacterales Desulfobacteraceae bacterium isolate DS bin 304 aa
BacClassII Pseudomonadati Thermodesulfobacteriota Desulfobacteria Desulfobacterales Desulfosarcinaceae bacterium isolate Spart 241 aa
BacClassII Pseudomonadati Thermodesulfobacteriota Desulfobacteria Desulfobacterales Desulfosarcinaceae bacterium isolate MO 167 312 aa
BacClassII Pseudomonadati Pseudomonadota Gammaproteobacteria Chromatiales Thioalkalivibrio sp GCF 041687635.1 302 aa
BacClassII Pseudomonadati Thermodesulfobacteriota Desulfobacteria Desulfobacterales Desulfococcaceae bacterium isolate D1M07 3 302 aa
BacClassII Pseudomonadati Thermodesulfobacteriota Desulfobacteria Desulfobacterales Desulfococcaceae bacterium isolate D1M02 3 243 aa
BacClassII Pseudomonadati Thermodesulfobacteriota Desulfovibrionia Desulfovibrionales Desulfovibrionales bacterium isolate D1M1 159 aa
BacClassII Pseudomonadati Thermodesulfobacteriota Desulfovibrionia Desulfovibrionales Desulfovibrionales bacterium isolate D1M0 302 aa
BacClassII Pseudomonadati Thermodesulfobacteriota Desulfovibrionia Desulfovibrionales Desulfovibrionales bacterium isolate D1M0 303 aa
BacClassII Pseudomonadati Thermodesulfobacteriota Desulfobacteria Desulfobacterales Desulfohalobiaceae bacterium isolate D1M0 303 aa
BacClassII Pseudomonadati Thermodesulfobacteriota Desulfovibrionia Desulfovibrionales Oceanidesulfovibrio sp isolate D1M03 22 338 aa
BacClassII Pseudomonadati Thermodesulfobacteriota Desulfovibrionia Desulfovibrionales Oceanidesulfovibrio marinus strain P48SEP 306 aa
BacClassII Pseudomonadati Thermodesulfobacteriota Desulfovibrionia Desulfovibrionales Oceanidesulfovibrio marinus strain CS1 306 aa
BacClassII Pseudomonadati Thermodesulfobacteriota Desulfobacteria Desulfobacterales Desulfobacterales bacterium isolate D0606S0 304 aa
BacClassII Pseudomonadati Thermodesulfobacteriota Desulfobacteria Desulfobacterales Desulfobacteraceae bacterium isolate Bin4 304 aa
BacClassII Pseudomonadati Myxococcota Myxococcia Deltaproteobacteria bacterium isolate MAG130 304 aa
BacClassII Pseudomonadati Thermodesulfobacteriota Desulfobacteria Desulfobacterales Desulfobacterales bacterium isolate G10 150 aa
BacClassII Pseudomonadati Thermodesulfobacteriota Desulfobacteria Desulfobacterales Desulfobacterales bacterium isolate MO 133 308 aa
BacClassII Pseudomonadati Thermodesulfobacteriota Desulfobacteria Desulfobacterales Desulfobacterales bacterium isolate MO 082 308 aa
BacClassII Pseudomonadati Thermodesulfobacteriota Desulfobacteria Desulfobacterales Desulfobacterales bacterium isolate MO 202 308 aa
BacClassII Pseudomonadati Thermodesulfobacteriota Desulfobacteria Desulfobacterales Desulfobacterales bacterium isolate D1103S0 308 aa
BacClassIV Pseudomonadati Pseudomonadota Gammaproteobacteria Acinetobacter sp GCF 043773465.1 312 aa
BacClassIV Pseudomonadati Pseudomonadota unclassified Pseudomonadota Pseudomonadota bacterium isolate DOLZORAL124 48 12 306 aa
BacClassIV Pseudomonadati Acidobacteriota unclassified Acidobacteriota Acidobacteriota bacterium isolate DOLJORAL78 46 51 306 aa
BacClassIV Pseudomonadati Acidobacteriota unclassified Acidobacteriota Acidobacteriota bacterium isolate MO 135.B14 317 aa
BacClassIV Pseudomonadati Acidobacteriota Holophagae Sulfidibacter corallicola 326 aa
BacClassIV Pseudomonadati Acidobacteriota Holophagae Acanthopleuribacter pedis 337 aa
BacClassIV Pseudomonadati FCB Bacteroidota unclassified Bacteroidota Bacteroidota bacterium isolate PH2015 13U 35 20 312 aa
BacClassIV Pseudomonadati FCB Bacteroidota Cytophagia Thermoflexibacter sp isolate Prado057 311 aa
BacClassIV Pseudomonadati FCB Bacteroidota Cytophagia Thermoflexibacter ruber 311 aa
BacClassIV Pseudomonadati FCB Bacteroidota Cytophagia Bernardetiaceae bacterium isolate Prado079 313 aa
BacClassIV Pseudomonadati FCB Bacteroidota Cytophagia Rhodoflexus caldus 314 aa
BacClassIV Pseudomonadati FCB Bacteroidota Cytophagia Bernardetiaceae bacterium isolate SKYG123 311 aa
BacClassIV Pseudomonadati FCB Bacteroidota Cytophagia Bernardetiaceae bacterium isolate coassembly bin.30 308 aa
BacClassIV Pseudomonadati FCB Bacteroidota Cytophagia Hugenholtzia roseola 341 aa
BacClassIV Pseudomonadati FCB Bacteroidota Cytophagia Bernardetia sp GCF 020630935.1 308 aa
BacClassIV Pseudomonadati FCB Bacteroidota Cytophagia Bernardetia sp ABR2 2B 309 aa
BacClassIV Pseudomonadati FCB Bacteroidota Cytophagia Bernardetia sp OM2101 309 aa
BacClassIV Pseudomonadati FCB Bacteroidota Cytophagia Bernardetia litoralis 314 aa
BacClassIV Pseudomonadati FCB Bacteroidota Cytophagia Bernardetia sp Wsw4 3y2 309 aa
BacClassIV Pseudomonadati FCB Bacteroidota Cytophagia Bernardetia sp MNP M8 309 aa
BacClassIV Pseudomonadati PVC Verrucomicrobiota Opitutus sp isolate cluster1 bin.34 309 aa
BacClassIV Pseudomonadati PVC Verrucomicrobiota Opitutia Nibricoccus sp isolate 5N149 iso2 310 aa
BacClassIV Pseudomonadati Pseudomonadota unclassified Pseudomonadota Pseudomonadota bacterium isolate SP206 305 aa
BacClassIV Pseudomonadati Pseudomonadota Alphaproteobacteria Alphaproteobacteria bacterium isolate Bin.012 314 aa
BacClassIV Pseudomonadati Pseudomonadota Alphaproteobacteria Rhodospirillales Candidatus Scatocola faecipullorum 312 aa
BacClassIV Pseudomonadati Pseudomonadota Alphaproteobacteria Rhodospirillales Azospirillum sp isolate L2 026 000M1 dasL2 026 0 312 aa
BacClassIV Pseudomonadati Pseudomonadota Alphaproteobacteria Alphaproteobacteria bacterium isolate Horse feces335 258 aa
BacClassIV Pseudomonadati Pseudomonadota Alphaproteobacteria Alphaproteobacteria bacterium isolate RGIG10246 304 aa
BacClassIV Pseudomonadati Pseudomonadota Alphaproteobacteria Alphaproteobacteria bacterium isolate RGIG6621 315 aa
BacClassIV Pseudomonadati Pseudomonadota Alphaproteobacteria Alphaproteobacteria bacterium isolate UBA9814 306 aa
BacClassIV Pseudomonadati Pseudomonadota Alphaproteobacteria Alphaproteobacteria bacterium isolate RGIG5118 318 aa
BacClassIV Pseudomonadati Pseudomonadota Alphaproteobacteria Alphaproteobacteria bacterium isolate RGIG4140 305 aa
BacClassIV Pseudomonadati Pseudomonadota Alphaproteobacteria Alphaproteobacteria bacterium isolate SIG53 310 aa
BacClassIV Pseudomonadati Pseudomonadota Alphaproteobacteria Alphaproteobacteria bacterium isolate RGIG6176 310 aa
BacClassIV Pseudomonadati Pseudomonadota Alphaproteobacteria Alphaproteobacteria bacterium isolate RGIG1869 310 aa
BacClassIV Pseudomonadati Pseudomonadota Alphaproteobacteria Alphaproteobacteria bacterium isolate RGIG6300 297 aa
BacClassIV Pseudomonadati Pseudomonadota Alphaproteobacteria Alphaproteobacteria bacterium isolate FM 39 G125 V 310 aa
BacClassIV Pseudomonadati Pseudomonadota Alphaproteobacteria Alphaproteobacteria bacterium isolate UBA8037 324 aa
BacClassIV Pseudomonadati Pseudomonadota Alphaproteobacteria Alphaproteobacteria bacterium isolate SUG1112 309 aa
BacClassIV Pseudomonadati Pseudomonadota Alphaproteobacteria Alphaproteobacteria bacterium isolate RGIG5112 309 aa
BacClassIV Pseudomonadati Pseudomonadota Alphaproteobacteria Alphaproteobacteria bacterium isolate RGIG4937 306 aa
BacClassIV Pseudomonadati Pseudomonadota Alphaproteobacteria Acetobacterales Acetobacter sp isolate NALB 7823 20 306 aa
BacClassIV Pseudomonadati FCB Bacteroidota Cytophagia Eisenibacter elegans 342 aa
BacClassIV Pseudomonadati FCB Bacteroidota Cytophagia Catalinimonas alkaloidigena 309 aa
BacClassIV Pseudomonadati FCB Bacteroidota Cytophagia Thermonema lapsum 306 aa
BacClassIV Pseudomonadati FCB Bacteroidota Cytophagia Thermonema sp GCF 026003375.1 306 aa
BacClassIV Pseudomonadati FCB Bacteroidota Cytophagia Thermonema rossianum 306 aa
BacClassIV Pseudomonadati FCB Bacteroidota unclassified Bacteroidota Bacteroidota bacterium isolate P01 D01 bin.158 305 aa
BacClassIV Pseudomonadati FCB Bacteroidota Cytophagia Raineya sp isolate 21H 022.bin.33 318 aa
BacClassIV Pseudomonadati FCB Bacteroidota unclassified Bacteroidota bacteria 336 3 303 aa
BacClassIV Pseudomonadati FCB Bacteroidota Cytophagia Raineya sp isolate Prado078 303 aa
BacClassIV Pseudomonadati FCB Bacteroidota Cytophagia Raineya orbicola 305 aa
BacClassIV Pseudomonadati FCB Bacteroidota Cytophagia Raineya sp isolate SKYG8 hs bin127 307 aa
BacClassIV Pseudomonadati FCB Bacteroidota Cytophagia Raineya sp isolate SKYG86 307 aa
BacClassIV Pseudomonadati FCB Bacteroidota Cytophagia Microscillaceae bacterium isolate Prado074 303 aa
BacClassIV Pseudomonadati FCB Bacteroidota Cytophagia Microscillaceae bacterium isolate bins.15 304 aa
BacClassIV Pseudomonadati FCB Bacteroidota Cytophagia uncultured Microscilla sp GCF 947493425.1 306 aa
BacClassIV Pseudomonadati FCB Bacteroidota Cytophagia Microscilla marina 306 aa
BacClassIV Pseudomonadati FCB Bacteroidota Cytophagia Microscillaceae bacterium isolate UBA11690 305 aa
BacClassIV Pseudomonadati FCB Bacteroidota Cytophagia marine bacterium AO1 C 305 aa
BacClassIV unclassified Bacteria unclassified Bacteria miscellaneous taxid 49937 isolate ZodW Metabat.349 306 aa
BacClassIV Pseudomonadati FCB Bacteroidota Cytophagia Microscillaceae bacterium isolate SKYG84 304 aa
BacClassIV Pseudomonadati FCB Bacteroidota unclassified Bacteroidota Bacteroidota bacterium isolate PH2015 13D 43 10 301 aa
BacClassIV Pseudomonadati FCB Bacteroidota Cytophagia Microscillaceae bacterium isolate UBA8158 306 aa
BacClassIV Pseudomonadati FCB Bacteroidota unclassified Bacteroidota Bacteroidota bacterium isolate PH2015 09S 30 10 303 aa
BacClassIV Pseudomonadati FCB Bacteroidota Cytophagia Cytophagia bacterium 303 aa
BacClassIV Pseudomonadati FCB Bacteroidota Cytophagia Cytophagaceae bacterium isolate SMAG U7094 264 aa
BacClassIV Pseudomonadati FCB Bacteroidota Cytophagia Cytophagaceae bacterium ABcell3 307 aa
BacClassIV Pseudomonadati FCB Bacteroidota unclassified Bacteroidota Bacteroidota bacterium isolate DC RB 04 SPN110320 bin044 1 321 aa
BacClassIV Pseudomonadati FCB Bacteroidota Cytophagia Rhodocytophaga rosea 307 aa
BacClassIV Pseudomonadati FCB Bacteroidota Cytophagia Rhodocytophaga aerolata 307 aa
BacClassIV Pseudomonadati FCB Bacteroidota Cytophagia Xanthocytophaga flavus strain NT2B1 304 aa
BacClassIV Pseudomonadati FCB Bacteroidota Cytophagia Xanthocytophaga flavus strain DM2B3 1 304 aa
BacClassIV Pseudomonadati FCB Bacteroidota Cytophagia Xanthocytophaga agilis 304 aa
BacClassIV Pseudomonadati FCB Bacteroidota Cytophagia Splendidivirga corallicola 307 aa
BacClassIV Pseudomonadati FCB Bacteroidota Cytophagia Agaribacillus aureus 307 aa
BacClassIV Pseudomonadati FCB Bacteroidota Cytophagia Penaeicola halotolerans 311 aa
BacClassIV Pseudomonadati FCB Bacteroidota Cytophagia Belliella kenyensis 304 aa
BacClassIV Pseudomonadati FCB Bacteroidota Cytophagia Belliella filtrata 304 aa
BacClassIV Pseudomonadati FCB Bacteroidota Cytophagia Belliella calami 303 aa
BacClassIV Pseudomonadati FCB Bacteroidota Cytophagia Belliella marina 303 aa
BacClassIV Pseudomonadati FCB Bacteroidota Cytophagia Belliella alkalica 303 aa
BacClassIV Pseudomonadati FCB Bacteroidota Cytophagia Belliella pelovolcani 184 aa
BacClassIV Pseudomonadati FCB Bacteroidota Cytophagia Belliella buryatensis 305 aa
BacClassIV Pseudomonadati FCB Bacteroidota Cytophagia Belliella baltica 305 aa
BacClassIV Pseudomonadati FCB Bacteroidota Cytophagia Belliella aquatica 305 aa
BacClassIV Pseudomonadati FCB Bacteroidota Cytophagia Pararhodonellum marinum 306 aa
BacClassIV Pseudomonadati FCB Bacteroidota Cytophagia Anditalea sp isolate SMAG U16128 307 aa
BacClassIV Pseudomonadati FCB Bacteroidota Cytophagia Anditalea andensis 305 aa
BacClassIV Pseudomonadati FCB Bacteroidota Cytophagia Litoribacter populi 257 aa
BacClassIV Pseudomonadati FCB Bacteroidota Cytophagia Litoribacter ruber KCTC 32217 305 aa
BacClassIV Pseudomonadati FCB Bacteroidota Cytophagia Litoribacter ruber KCTC 22899 305 aa
BacClassIV Pseudomonadati FCB Bacteroidota Cytophagia Rhodonellum sp GCF 030656585.1 288 aa
BacClassIV Pseudomonadati FCB Bacteroidota Cytophagia Rhodonellum psychrophilum GCM71 306 aa
BacClassIV Pseudomonadati FCB Bacteroidota Cytophagia Cyclobacteriaceae bacterium isolate SMAG U10375 306 aa
BacClassIV Pseudomonadati FCB Bacteroidota Cytophagia Cyclobacteriaceae bacterium isolate SMAG U10323 303 aa
BacClassIV Pseudomonadati FCB Bacteroidota Cytophagia Anditalea sp isolate LejiaC 41 17 307 aa
BacClassIV Pseudomonadati FCB Bacteroidota Cytophagia Mongoliitalea lutea 306 aa
BacClassIV Pseudomonadati FCB Bacteroidota Cytophagia Mongoliitalea daihaiensis 306 aa
BacClassIV Pseudomonadati FCB Bacteroidota Cytophagia Cecembia rubra 308 aa
BacClassIV Pseudomonadati FCB Bacteroidota Cytophagia Cecembia calidifontis 308 aa
BacClassIV Pseudomonadati FCB Bacteroidota Cytophagia Cecembia sp GCF 007126775.1 307 aa
BacClassIV Pseudomonadati FCB Bacteroidota Cytophagia Cecembia lonarensis 307 aa
BacClassIV Pseudomonadati FCB Bacteroidota Cytophagia Fontibacter flavus 306 aa
BacClassIV Pseudomonadati FCB Bacteroidota Cytophagia Shivajiella indica 307 aa
BacClassIV Pseudomonadati FCB Bacteroidota Cytophagia Cyclobacteriaceae bacterium YHN15 307 aa
BacClassIV Pseudomonadati FCB Bacteroidota Cytophagia Mariniradius sediminis 306 aa
BacClassIV Pseudomonadati FCB Bacteroidota Cytophagia Mariniradius saccharolyticus 306 aa
BacClassIV Pseudomonadati FCB Bacteroidota Cytophagia Aquiflexum sp GCF 041751335.1 302 aa
BacClassIV Pseudomonadati FCB Bacteroidota Cytophagia Aquiflexum sp GCF 041749095.1 302 aa
BacClassIV Pseudomonadati FCB Bacteroidota Cytophagia Aquiflexum lacus 303 aa
BacClassIV Pseudomonadati FCB Bacteroidota Cytophagia Aquiflexum balticum DSM 16537 302 aa
BacClassIV Pseudomonadati FCB Bacteroidota Cytophagia Indibacter alkaliphilus LW1 307 aa
BacClassIV Pseudomonadati FCB Bacteroidota Cytophagia Arthrospiribacter ruber 309 aa
BacClassIV Pseudomonadati FCB Bacteroidota Cytophagia Mongoliibacter sp GCF 007692705.1 307 aa
BacClassIV Pseudomonadati FCB Bacteroidota Cytophagia Mongoliibacter ruber 305 aa
BacClassIV Pseudomonadati FCB Bacteroidota Cytophagia Aquiflexum gelatinilyticum strain XJ19 11 303 aa
BacClassIV Pseudomonadati FCB Bacteroidota Cytophagia Aquiflexum gelatinilyticum strain XJ19 10 303 aa
BacClassIV Pseudomonadati FCB Bacteroidota Cytophagia Cognataquiflexum nitidum 303 aa
BacClassIV Pseudomonadati FCB Bacteroidota Cytophagia Cognataquiflexum rubidum 304 aa

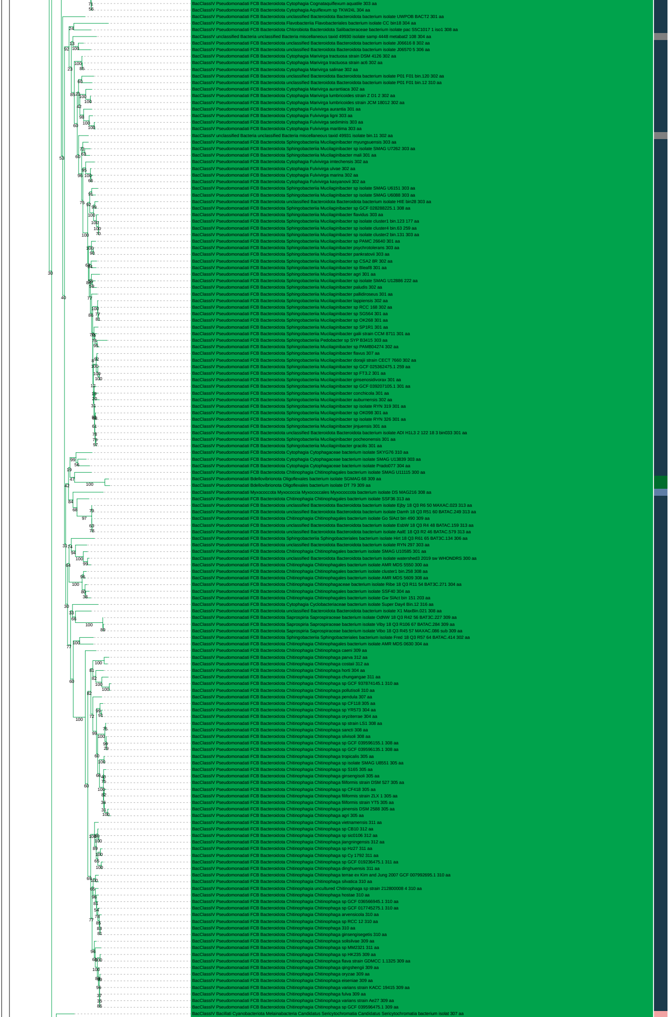

BacClassIV Pseudomonadati FCB Bacteroidota Cytophagia Cognataquiflexum aquatile 303 aa
BacClassIV Pseudomonadati FCB Bacteroidota Cytophagia Aquiflexum sp TKW24L 304 aa
BacClassIV Pseudomonadati FCB Bacteroidota unclassified Bacteroidota Bacteroidota bacterium isolate UWPOB BACT2 301 aa
BacClassIV Pseudomonadati FCB Bacteroidota Flavobacteriia Flavobacteriales bacterium isolate CC bin18 304 aa
BacClassIV Pseudomonadati FCB Chlorobiota Chlorobiota Salibacteraceae bacterium isolate pac S5C1017 1 iso1 308 aa
BacClassIV unclassified Bacteria unclassified Bacteria miscellaneous taxid 49930 isolate samp 4448 metabat2 108 304 aa
BacClassIV Pseudomonadati FCB Bacteroidota unclassified Bacteroidota Bacteroidota bacterium isolate J06616 8 302 aa
BacClassIV Pseudomonadati FCB Bacteroidota unclassified Bacteroidota Bacteroidota bacterium isolate J06570 5 306 aa
BacClassIV Pseudomonadati FCB Bacteroidota Cytophagia Marivirga tractuosa strain DSM 4126 302 aa
BacClassIV Pseudomonadati FCB Bacteroidota Cytophagia Marivirga tractuosa strain ac6 302 aa
BacClassIV Pseudomonadati FCB Bacteroidota Cytophagia Marivirga salinae 302 aa
BacClassIV Pseudomonadati FCB Bacteroidota unclassified Bacteroidota Bacteroidota bacterium isolate P01 F01 bin.120 302 aa
BacClassIV Pseudomonadati FCB Bacteroidota unclassified Bacteroidota Bacteroidota bacterium isolate P01 F01 bin.12 310 aa
BacClassIV Pseudomonadati FCB Bacteroidota Cytophagia Marivirga aurantiaca 302 aa
BacClassIV Pseudomonadati FCB Bacteroidota Cytophagia Marivirga lumbricoides strain Z D1 2 302 aa
BacClassIV Pseudomonadati FCB Bacteroidota Cytophagia Marivirga lumbricoides strain JCM 18012 302 aa
BacClassIV Pseudomonadati FCB Bacteroidota Cytophagia Fulvivirga aurantia 301 aa
BacClassIV Pseudomonadati FCB Bacteroidota Cytophagia Fulvivirga ligni 303 aa
BacClassIV Pseudomonadati FCB Bacteroidota Cytophagia Fulvivirga sediminis 303 aa
BacClassIV Pseudomonadati FCB Bacteroidota Cytophagia Fulvivirga maritima 303 aa
BacClassIV unclassified Bacteria unclassified Bacteria miscellaneous taxid 49931 isolate bin.11 302 aa
BacClassIV Pseudomonadati FCB Bacteroidota Sphingobacteriia Mucilaginibacter myungsuensis 303 aa
BacClassIV Pseudomonadati FCB Bacteroidota Sphingobacteriia Mucilaginibacter sp isolate SMAG U7262 303 aa
BacClassIV Pseudomonadati FCB Bacteroidota Sphingobacteriia Mucilaginibacter mali 301 aa
BacClassIV Pseudomonadati FCB Bacteroidota Cytophagia Fulvivirga imtechensis 302 aa
BacClassIV Pseudomonadati FCB Bacteroidota Cytophagia Fulvivirga ulvae 302 aa
BacClassIV Pseudomonadati FCB Bacteroidota Cytophagia Fulvivirga marina 302 aa
BacClassIV Pseudomonadati FCB Bacteroidota Cytophagia Fulvivirga kasyanovii 302 aa
BacClassIV Pseudomonadati FCB Bacteroidota Sphingobacteriia Mucilaginibacter sp isolate SMAG U6151 303 aa
BacClassIV Pseudomonadati FCB Bacteroidota Sphingobacteriia Mucilaginibacter sp isolate SMAG U6088 303 aa
BacClassIV Pseudomonadati FCB Bacteroidota unclassified Bacteroidota Bacteroidota bacterium isolate HIE bin28 303 aa
BacClassIV Pseudomonadati FCB Bacteroidota Sphingobacteriia Mucilaginibacter sp GCF 028288225.1 308 aa
BacClassIV Pseudomonadati FCB Bacteroidota Sphingobacteriia Mucilaginibacter flavidus 303 aa
BacClassIV Pseudomonadati FCB Bacteroidota Sphingobacteriia Mucilaginibacter sp isolate cluster1 bin.123 177 aa
BacClassIV Pseudomonadati FCB Bacteroidota Sphingobacteriia Mucilaginibacter sp isolate cluster4 bin.63 259 aa
BacClassIV Pseudomonadati FCB Bacteroidota Sphingobacteriia Mucilaginibacter sp isolate cluster2 bin.131 303 aa
BacClassIV Pseudomonadati FCB Bacteroidota Sphingobacteriia Mucilaginibacter sp PAMC 26640 301 aa
BacClassIV Pseudomonadati FCB Bacteroidota Sphingobacteriia Mucilaginibacter psychrotolerans 303 aa
BacClassIV Pseudomonadati FCB Bacteroidota Sphingobacteriia Mucilaginibacter pankratovii 303 aa
BacClassIV Pseudomonadati FCB Bacteroidota Sphingobacteriia Mucilaginibacter sp CSA2 8R 302 aa
BacClassIV Pseudomonadati FCB Bacteroidota Sphingobacteriia Mucilaginibacter sp Bleaf8 301 aa
BacClassIV Pseudomonadati FCB Bacteroidota Sphingobacteriia Mucilaginibacter agri 301 aa
BacClassIV Pseudomonadati FCB Bacteroidota Sphingobacteriia Mucilaginibacter sp isolate SMAG U12886 222 aa
BacClassIV Pseudomonadati FCB Bacteroidota Sphingobacteriia Mucilaginibacter paludis 302 aa
BacClassIV Pseudomonadati FCB Bacteroidota Sphingobacteriia Mucilaginibacter pallidiroseus 301 aa
BacClassIV Pseudomonadati FCB Bacteroidota Sphingobacteriia Mucilaginibacter lappiensis 302 aa
BacClassIV Pseudomonadati FCB Bacteroidota Sphingobacteriia Mucilaginibacter sp RCC 168 302 aa
BacClassIV Pseudomonadati FCB Bacteroidota Sphingobacteriia Mucilaginibacter sp SG564 301 aa
BacClassIV Pseudomonadati FCB Bacteroidota Sphingobacteriia Mucilaginibacter sp OK268 301 aa
BacClassIV Pseudomonadati FCB Bacteroidota Sphingobacteriia Mucilaginibacter sp SP1R1 301 aa
BacClassIV Pseudomonadati FCB Bacteroidota Sphingobacteriia Mucilaginibacter galii strain CCM 8711 301 aa
BacClassIV Pseudomonadati FCB Bacteroidota Sphingobacteriia Pedobacter sp SYP B3415 303 aa
BacClassIV Pseudomonadati FCB Bacteroidota Sphingobacteriia Mucilaginibacter sp PAMB04274 302 aa
BacClassIV Pseudomonadati FCB Bacteroidota Sphingobacteriia Mucilaginibacter flavus 307 aa
BacClassIV Pseudomonadati FCB Bacteroidota Sphingobacteriia Mucilaginibacter dorajii strain CECT 7660 302 aa
BacClassIV Pseudomonadati FCB Bacteroidota Sphingobacteriia Mucilaginibacter sp GCF 025362475.1 259 aa
BacClassIV Pseudomonadati FCB Bacteroidota Sphingobacteriia Mucilaginibacter sp FT3.2 301 aa
BacClassIV Pseudomonadati FCB Bacteroidota Sphingobacteriia Mucilaginibacter ginsenosidivorax 301 aa
BacClassIV Pseudomonadati FCB Bacteroidota Sphingobacteriia Mucilaginibacter sp GCF 039207105.1 301 aa
BacClassIV Pseudomonadati FCB Bacteroidota Sphingobacteriia Mucilaginibacter conchicola 301 aa
BacClassIV Pseudomonadati FCB Bacteroidota Sphingobacteriia Mucilaginibacter auburnensis 302 aa
BacClassIV Pseudomonadati FCB Bacteroidota Sphingobacteriia Mucilaginibacter sp isolate RYN 319 301 aa
BacClassIV Pseudomonadati FCB Bacteroidota Sphingobacteriia Mucilaginibacter sp OK098 301 aa
BacClassIV Pseudomonadati FCB Bacteroidota Sphingobacteriia Mucilaginibacter sp isolate RYN 326 301 aa
BacClassIV Pseudomonadati FCB Bacteroidota Sphingobacteriia Mucilaginibacter jinjuensis 301 aa
BacClassIV Pseudomonadati FCB Bacteroidota unclassified Bacteroidota Bacteroidota bacterium isolate ADI H1L3 2 122 18 3 bin033 301 aa
BacClassIV Pseudomonadati FCB Bacteroidota Sphingobacteriia Mucilaginibacter pocheonensis 301 aa
BacClassIV Pseudomonadati FCB Bacteroidota Sphingobacteriia Mucilaginibacter gracilis 301 aa
BacClassIV Pseudomonadati FCB Bacteroidota Cytophagia Cytophagaceae bacterium isolate SKYG76 310 aa
BacClassIV Pseudomonadati FCB Bacteroidota Cytophagia Cytophagaceae bacterium isolate SMAG U13839 303 aa
BacClassIV Pseudomonadati FCB Bacteroidota Cytophagia Cytophagaceae bacterium isolate Prado077 304 aa
BacClassIV Pseudomonadati FCB Bacteroidota Chitinophagia Chitinophagales bacterium isolate SMAG U11115 300 aa
BacClassIV Pseudomonadati Bdellovibrionota Oligoflexales bacterium isolate SGMAG 68 309 aa
BacClassIV Pseudomonadati Bdellovibrionota Oligoflexales bacterium isolate DT 79 309 aa
BacClassIV Pseudomonadati Myxococcota Myxococcia Myxococcales Myxococcota bacterium isolate DS MAG216 308 aa
BacClassIV Pseudomonadati FCB Bacteroidota Chitinophagia Chitinophagales bacterium isolate SSF36 313 aa
BacClassIV Pseudomonadati FCB Bacteroidota unclassified Bacteroidota Bacteroidota bacterium isolate Ejby 18 Q3 R6 50 MAXAC.023 313 aa
BacClassIV Pseudomonadati FCB Bacteroidota unclassified Bacteroidota Bacteroidota bacterium isolate Damh 18 Q3 R51 60 BATAC.249 313 aa
BacClassIV Pseudomonadati FCB Bacteroidota Chitinophagia Chitinophagales bacterium isolate Go SlAct bin 490 309 aa
BacClassIV Pseudomonadati FCB Bacteroidota unclassified Bacteroidota Bacteroidota bacterium isolate EsbW 18 Q3 R4 48 BATAC.159 313 aa
BacClassIV Pseudomonadati FCB Bacteroidota unclassified Bacteroidota Bacteroidota bacterium isolate AalE 18 Q3 R2 46 BATAC.579 313 aa
BacClassIV Pseudomonadati FCB Bacteroidota Sphingobacteriia Sphingobacteriales bacterium isolate Hirt 18 Q3 R61 65 BAT3C.134 306 aa
BacClassIV Pseudomonadati FCB Bacteroidota unclassified Bacteroidota Bacteroidota bacterium isolate RYN 297 303 aa
BacClassIV Pseudomonadati FCB Bacteroidota Chitinophagia Chitinophagales bacterium isolate SMAG U10585 301 aa
BacClassIV Pseudomonadati FCB Bacteroidota unclassified Bacteroidota Bacteroidota bacterium isolate watershed3 2019 sw WHONDRS 300 aa
BacClassIV Pseudomonadati FCB Bacteroidota Chitinophagia Chitinophagales bacterium isolate AMR MDS 5550 300 aa
BacClassIV Pseudomonadati FCB Bacteroidota Chitinophagia Chitinophagales bacterium isolate cluster1 bin.258 308 aa
BacClassIV Pseudomonadati FCB Bacteroidota Chitinophagia Chitinophagales bacterium isolate AMR MDS 5609 308 aa
BacClassIV Pseudomonadati FCB Bacteroidota Chitinophagia Chitinophagaceae bacterium isolate Ribe 18 Q3 R11 54 BAT3C.271 304 aa
BacClassIV Pseudomonadati FCB Bacteroidota Chitinophagia Chitinophagales bacterium isolate SSF40 304 aa
BacClassIV Pseudomonadati FCB Bacteroidota Chitinophagia Chitinophagales bacterium isolate Gw SlAct bin 151 203 aa
BacClassIV Pseudomonadati FCB Bacteroidota Cytophagia Cyclobacteriaceae bacterium isolate Super Day4 Bin.12 316 aa
BacClassIV Pseudomonadati FCB Bacteroidota unclassified Bacteroidota Bacteroidota bacterium isolate X1 MaxBin.021 308 aa
BacClassIV Pseudomonadati FCB Bacteroidota Saprospiria Saprospiraceae bacterium isolate OdNW 18 Q3 R42 56 BAT3C.227 309 aa
BacClassIV Pseudomonadati FCB Bacteroidota Saprospiria Saprospiraceae bacterium isolate Viby 18 Q3 R106 67 BATAC.284 309 aa
BacClassIV Pseudomonadati FCB Bacteroidota Saprospiria Saprospiraceae bacterium isolate Vibo 18 Q3 R45 57 MAXAC.086 sub 309 aa
BacClassIV Pseudomonadati FCB Bacteroidota Sphingobacteriia Sphingobacteriales bacterium isolate Fred 18 Q3 R57 64 BATAC.414 302 aa
BacClassIV Pseudomonadati FCB Bacteroidota Chitinophagia Chitinophagales bacterium isolate AMR MDS 0630 304 aa
BacClassIV Pseudomonadati FCB Bacteroidota Chitinophagia Chitinophaga caeni 309 aa
BacClassIV Pseudomonadati FCB Bacteroidota Chitinophagia Chitinophaga parva 312 aa
BacClassIV Pseudomonadati FCB Bacteroidota Chitinophagia Chitinophaga costaii 312 aa
BacClassIV Pseudomonadati FCB Bacteroidota Chitinophagia Chitinophaga horti 304 aa
BacClassIV Pseudomonadati FCB Bacteroidota Chitinophagia Chitinophaga chungangae 311 aa
BacClassIV Pseudomonadati FCB Bacteroidota Chitinophagia Chitinophaga sp GCF 937874145.1 310 aa
BacClassIV Pseudomonadati FCB Bacteroidota Chitinophagia Chitinophaga pollutisoli 310 aa
BacClassIV Pseudomonadati FCB Bacteroidota Chitinophagia Chitinophaga pendula 307 aa
BacClassIV Pseudomonadati FCB Bacteroidota Chitinophagia Chitinophaga sp CF118 305 aa
BacClassIV Pseudomonadati FCB Bacteroidota Chitinophagia Chitinophaga sp YR573 304 aa
BacClassIV Pseudomonadati FCB Bacteroidota Chitinophagia Chitinophaga oryziterrae 304 aa
BacClassIV Pseudomonadati FCB Bacteroidota Chitinophagia Chitinophaga sp strain LS1 308 aa
BacClassIV Pseudomonadati FCB Bacteroidota Chitinophagia Chitinophaga sancti 308 aa
BacClassIV Pseudomonadati FCB Bacteroidota Chitinophagia Chitinophaga silvisoli 308 aa
BacClassIV Pseudomonadati FCB Bacteroidota Chitinophagia Chitinophaga sp GCF 039596155.1 308 aa
BacClassIV Pseudomonadati FCB Bacteroidota Chitinophagia Chitinophaga sp GCF 039596135.1 308 aa
BacClassIV Pseudomonadati FCB Bacteroidota Chitinophagia Chitinophaga tropicalis 305 aa
BacClassIV Pseudomonadati FCB Bacteroidota Chitinophagia Chitinophaga sp isolate SMAG U8551 305 aa
BacClassIV Pseudomonadati FCB Bacteroidota Chitinophagia Chitinophaga sp S165 305 aa
BacClassIV Pseudomonadati FCB Bacteroidota Chitinophagia Chitinophaga ginsengisoli 305 aa
BacClassIV Pseudomonadati FCB Bacteroidota Chitinophagia Chitinophaga filiformis strain DSM 527 305 aa
BacClassIV Pseudomonadati FCB Bacteroidota Chitinophagia Chitinophaga sp CF418 305 aa
BacClassIV Pseudomonadati FCB Bacteroidota Chitinophagia Chitinophaga filiformis strain ZLX 1 305 aa
BacClassIV Pseudomonadati FCB Bacteroidota Chitinophagia Chitinophaga filiformis strain YT5 305 aa
BacClassIV Pseudomonadati FCB Bacteroidota Chitinophagia Chitinophaga pinensis DSM 2588 305 aa
BacClassIV Pseudomonadati FCB Bacteroidota Chitinophagia Chitinophaga agri 305 aa
BacClassIV Pseudomonadati FCB Bacteroidota Chitinophagia Chitinophaga vietnamensis 311 aa
BacClassIV Pseudomonadati FCB Bacteroidota Chitinophagia Chitinophaga sp CB10 312 aa
BacClassIV Pseudomonadati FCB Bacteroidota Chitinophagia Chitinophaga sp sic0106 312 aa
BacClassIV Pseudomonadati FCB Bacteroidota Chitinophagia Chitinophaga jiangningensis 312 aa
BacClassIV Pseudomonadati FCB Bacteroidota Chitinophagia Chitinophaga sp Hz27 311 aa
BacClassIV Pseudomonadati FCB Bacteroidota Chitinophagia Chitinophaga sp Cy 1792 311 aa
BacClassIV Pseudomonadati FCB Bacteroidota Chitinophagia Chitinophaga sp GCF 019236475.1 311 aa
BacClassIV Pseudomonadati FCB Bacteroidota Chitinophagia Chitinophaga dinghuensis 311 aa
BacClassIV Pseudomonadati FCB Bacteroidota Chitinophagia Chitinophaga terrae ex Kim and Jung 2007 GCF 007992695.1 310 aa
BacClassIV Pseudomonadati FCB Bacteroidota Chitinophagia Chitinophaga silvatica 310 aa
BacClassIV Pseudomonadati FCB Bacteroidota Chitinophagia uncultured Chitinophaga sp strain 212800008 4 310 aa
BacClassIV Pseudomonadati FCB Bacteroidota Chitinophagia Chitinophaga hostae 310 aa
BacClassIV Pseudomonadati FCB Bacteroidota Chitinophagia Chitinophaga sp GCF 036566945.1 310 aa
BacClassIV Pseudomonadati FCB Bacteroidota Chitinophagia Chitinophaga sp GCF 017745275.1 310 aa
BacClassIV Pseudomonadati FCB Bacteroidota Chitinophagia Chitinophaga arvensicola 310 aa
BacClassIV Pseudomonadati FCB Bacteroidota Chitinophagia Chitinophaga sp RCC 12 310 aa
BacClassIV Pseudomonadati FCB Bacteroidota Chitinophagia Chitinophaga 310 aa
BacClassIV Pseudomonadati FCB Bacteroidota Chitinophagia Chitinophaga ginsengisegetis 310 aa
BacClassIV Pseudomonadati FCB Bacteroidota Chitinophagia Chitinophaga solisilvae 309 aa
BacClassIV Pseudomonadati FCB Bacteroidota Chitinophagia Chitinophaga sp MM2321 311 aa
BacClassIV Pseudomonadati FCB Bacteroidota Chitinophagia Chitinophaga sp HK235 309 aa
BacClassIV Pseudomonadati FCB Bacteroidota Chitinophagia Chitinophaga flava strain GDMCC 1.1325 309 aa
BacClassIV Pseudomonadati FCB Bacteroidota Chitinophagia Chitinophaga qingshengii 309 aa
BacClassIV Pseudomonadati FCB Bacteroidota Chitinophagia Chitinophaga oryzae 309 aa
BacClassIV Pseudomonadati FCB Bacteroidota Chitinophagia Chitinophaga eiseniae 309 aa
BacClassIV Pseudomonadati FCB Bacteroidota Chitinophagia Chitinophaga varians strain KACC 19415 309 aa
BacClassIV Pseudomonadati FCB Bacteroidota Chitinophagia Chitinophaga fulva 309 aa
BacClassIV Pseudomonadati FCB Bacteroidota Chitinophagia Chitinophaga varians strain Ae27 309 aa
BacClassIV Pseudomonadati FCB Bacteroidota Chitinophagia Chitinophaga sp GCF 039596475.1 309 aa
BacClassIV Bacillati Cyanobacteriota Melainabacteria Candidatus Sericytochromatia Candidatus Sericytochromatia bacterium isolat 307 aa

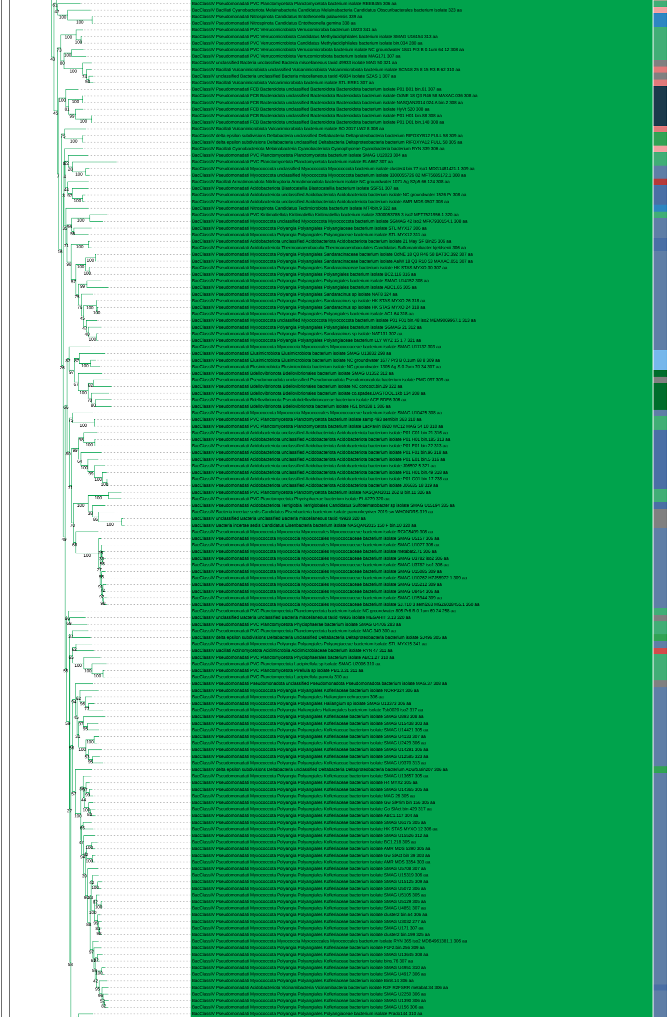
BacClassIV Pseudomonadati PVC Planctomycetota Planctomycetota bacterium isolate REEB455 306 aa
BacClassIV Bacillati Cyanobacteriota Melainabacteria Candidatus Melainabacteria Candidatus Obscuribacterales bacterium isolate 323 aa
BacClassIV Pseudomonadati Nitrospinota Candidatus Entotheonella palauensis 339 aa
BacClassIV Pseudomonadati Nitrospinota Candidatus Entotheonella gemina 338 aa
BacClassIV Pseudomonadati PVC Verrucomicrobiota Verrucomicrobia bacterium LW23 341 aa
BacClassIV Pseudomonadati PVC Verrucomicrobiota Candidatus Methylacidiphilales bacterium isolate SMAG U16154 313 aa
BacClassIV Pseudomonadati PVC Verrucomicrobiota Candidatus Methylacidiphilales bacterium isolate bin.034 280 aa
BacClassIV Pseudomonadati PVC Verrucomicrobiota Verrucomicrobiota bacterium isolate NC groundwater 1841 Pr3 B 0.1um 64 12 308 aa
BacClassIV Pseudomonadati PVC Verrucomicrobiota Verrucomicrobiota bacterium isolate MAG171 307 aa
BacClassIV unclassified Bacteria unclassified Bacteria miscellaneous taxid 49933 isolate MAG 50 321 aa
BacClassIV Bacillati Vulcanimicrobiota unclassified Vulcanimicrobiota bacterium isolate SCN18 25 8 15 R3 B 62 310 aa
BacClassIV unclassified Bacteria unclassified Bacteria miscellaneous taxid 49934 isolate SZAS 1 307 aa
BacClassIV Bacillati Vulcanimicrobiota Vulcanimicrobiota bacterium isolate STL ERE1 307 aa
BacClassIV Pseudomonadati FCB Bacteroidota unclassified Bacteroidota Bacteroidota bacterium isolate P01 B01 bin.61 307 aa
BacClassIV Pseudomonadati FCB Bacteroidota unclassified Bacteroidota Bacteroidota bacterium isolate OdNE 18 Q3 R46 58 MAXAC.036 308 aa
BacClassIV Pseudomonadati FCB Bacteroidota unclassified Bacteroidota Bacteroidota bacterium isolate NASQAN2014 024 A bin.2 308 aa
BacClassIV Pseudomonadati FCB Bacteroidota unclassified Bacteroidota Bacteroidota bacterium isolate HyVt 520 308 aa
BacClassIV Pseudomonadati FCB Bacteroidota unclassified Bacteroidota Bacteroidota bacterium isolate P01 H01 bin.88 308 aa
BacClassIV Pseudomonadati FCB Bacteroidota unclassified Bacteroidota Bacteroidota bacterium isolate P01 D01 bin.148 308 aa
BacClassIV Bacillati Vulcanimicrobiota Vulcanimicrobiota bacterium isolate SO 2017 LW2 8 308 aa
BacClassIV delta epsilon subdivisions Deltabacteria unclassified Deltabacteria Deltaproteobacteria bacterium RIFOXYB12 FULL 58 309 aa
BacClassIV delta epsilon subdivisions Deltabacteria unclassified Deltabacteria Deltaproteobacteria bacterium RIFOXYA12 FULL 58 305 aa
BacClassIV Bacillati Cyanobacteriota Melainabacteria Cyanobacteriota Cyanophyceae Cyanobacteria bacterium RYN 339 306 aa
BacClassIV Pseudomonadati PVC Planctomycetota Planctomycetota bacterium isolate SMAG U12023 304 aa
BacClassIV Pseudomonadati PVC Planctomycetota Planctomycetota bacterium isolate ELA667 307 aa
BacClassIV Pseudomonadati Myxococcota unclassified Myxococcota Myxococcota bacterium isolate cluster4 bin.77 iso1 MDG1481421.1 309 aa
BacClassIV Pseudomonadati Myxococcota unclassified Myxococcota Myxococcota bacterium isolate 3300055726 82 MFT5685172.1 308 aa
BacClassIV Bacillati Armatimonadota Nitriliruptoria Armatimonadota bacterium isolate NC groundwater 1071 Ag S2p5 66 124 308 aa
BacClassIV Pseudomonadati Acidobacteriota Blastocatellia Blastocatellia bacterium isolate SSF51 307 aa
BacClassIV Pseudomonadati Acidobacteriota unclassified Acidobacteriota Acidobacteriota bacterium isolate NC groundwater 1526 Pr 308 aa
BacClassIV Pseudomonadati Acidobacteriota unclassified Acidobacteriota Acidobacteriota bacterium isolate AMR MDS 0507 308 aa
BacClassIV Pseudomonadati Nitrospinota Candidatus Tectimicrobiota bacterium isolate MT4bin.9 322 aa
BacClassIV Pseudomonadati PVC Kiritimatiellota Kiritimatiellia Kiritimatiellia bacterium isolate 3300053785 3 iso2 MFT7521956.1 320 aa
BacClassIV Pseudomonadati Myxococcota unclassified Myxococcota Myxococcota bacterium isolate SGMAG 42 iso2 MFK7930154.1 308 aa
BacClassIV Pseudomonadati Myxococcota Polyangia Polyangiales Polyangiaceae bacterium isolate STL MYX17 306 aa
BacClassIV Pseudomonadati Myxococcota Polyangia Polyangiales Polyangiaceae bacterium isolate STL MYX12 311 aa
BacClassIV Pseudomonadati Acidobacteriota unclassified Acidobacteriota Acidobacteriota bacterium isolate 21 May SF Bin25 306 aa
BacClassIV Pseudomonadati Acidobacteriota Thermoanaerobaculia Thermoanaerobaculales Candidatus Sulfomarinibacter kjeldsenii 306 aa
BacClassIV Pseudomonadati Myxococcota Polyangia Polyangiales Sandaracinaceae bacterium isolate OdNE 18 Q3 R46 58 BAT3C.392 307 aa
BacClassIV Pseudomonadati Myxococcota Polyangia Polyangiales Sandaracinaceae bacterium isolate AalW 18 Q3 R10 53 MAXAC.051 307 aa
BacClassIV Pseudomonadati Myxococcota Polyangia Polyangiales Sandaracinaceae bacterium isolate HK STAS MYXO 30 307 aa
BacClassIV Pseudomonadati Myxococcota Polyangia Polyangiales Polyangiales bacterium isolate BC2.116 316 aa
BacClassIV Pseudomonadati Myxococcota Polyangia Polyangiales Polyangiales bacterium isolate SMAG U14152 308 aa
BacClassIV Pseudomonadati Myxococcota Polyangia Polyangiales Polyangiales bacterium isolate ABC1.65 305 aa
BacClassIV Pseudomonadati Myxococcota Polyangia Polyangiales Sandaracinus sp isolate NAT8 324 aa
BacClassIV Pseudomonadati Myxococcota Polyangia Polyangiales Sandaracinus sp isolate HK STAS MYXO 26 318 aa
BacClassIV Pseudomonadati Myxococcota Polyangia Polyangiales Sandaracinus sp isolate HK STAS MYXO 24 318 aa
BacClassIV Pseudomonadati Myxococcota Polyangia Polyangiales Polyangiales bacterium isolate AC1.64 318 aa
BacClassIV Pseudomonadati Myxococcota unclassified Myxococcota Myxococcota bacterium isolate P01 F01 bin.48 iso2 MEM9069967.1 313 aa
BacClassIV Pseudomonadati Myxococcota Polyangia Polyangiales Polyangiales bacterium isolate SGMAG 21 312 aa
BacClassIV Pseudomonadati Myxococcota Polyangia Polyangiales Sandaracinus sp isolate NAT131 302 aa
BacClassIV Pseudomonadati Myxococcota Polyangia Polyangiales Polyangiaceae bacterium LLY WYZ 15 1 7 321 aa
BacClassIV Pseudomonadati Myxococcota Myxococcia Myxococcales Myxococcaceae bacterium isolate SMAG U11132 303 aa
BacClassIV Pseudomonadati Elusimicrobiota Elusimicrobiota bacterium isolate SMAG U13832 298 aa
BacClassIV Pseudomonadati Elusimicrobiota Elusimicrobia Elusimicrobiota bacterium isolate NC groundwater 1677 Pr3 B 0.1um 68 8 309 aa
BacClassIV Pseudomonadati Elusimicrobiota Elusimicrobia Elusimicrobiota bacterium isolate NC groundwater 1305 Ag S 0.2um 70 34 307 aa
BacClassIV Pseudomonadati Bdellovibrionota Bdellovibrionales bacterium isolate SMAG U1352 312 aa
BacClassIV Pseudomonadati Pseudomonadota unclassified Pseudomonadota Pseudomonadota bacterium isolate PMG 097 309 aa
BacClassIV Pseudomonadati Bdellovibrionota Bdellovibrionales bacterium isolate NC concoct.bin.29 322 aa
BacClassIV Pseudomonadati Bdellovibrionota Bdellovibrionales bacterium isolate co.spades.DASTOOL.1kb 134 208 aa
BacClassIV Pseudomonadati Bdellovibrionota Pseudobdellovibrionaceae bacterium isolate ACE BDE6 306 aa
BacClassIV Pseudomonadati Bdellovibrionota Bdellovibrionota bacterium isolate H51 bin338 1 306 aa
BacClassIV Pseudomonadati Myxococcota Myxococcia Myxococcales Myxococcaceae bacterium isolate SMAG U10425 308 aa
BacClassIV Pseudomonadati PVC Planctomycetota Planctomycetota bacterium isolate samp 493 semibin 363 310 aa
BacClassIV Pseudomonadati PVC Planctomycetota Planctomycetota bacterium isolate LacPavin 0920 WC12 MAG 54 10 310 aa
BacClassIV Pseudomonadati Acidobacteriota unclassified Acidobacteriota Acidobacteriota bacterium isolate P01 C01 bin.21 316 aa
BacClassIV Pseudomonadati Acidobacteriota unclassified Acidobacteriota Acidobacteriota bacterium isolate P01 H01 bin.185 313 aa
BacClassIV Pseudomonadati Acidobacteriota unclassified Acidobacteriota Acidobacteriota bacterium isolate P01 E01 bin.22 313 aa
BacClassIV Pseudomonadati Acidobacteriota unclassified Acidobacteriota Acidobacteriota bacterium isolate P01 F01 bin.96 318 aa
BacClassIV Pseudomonadati Acidobacteriota unclassified Acidobacteriota Acidobacteriota bacterium isolate P01 E01 bin.5 316 aa
BacClassIV Pseudomonadati Acidobacteriota unclassified Acidobacteriota Acidobacteriota bacterium isolate J06592 5 321 aa
BacClassIV Pseudomonadati Acidobacteriota unclassified Acidobacteriota Acidobacteriota bacterium isolate P01 H01 bin.49 318 aa
BacClassIV Pseudomonadati Acidobacteriota unclassified Acidobacteriota Acidobacteriota bacterium isolate P01 G01 bin.17 238 aa
BacClassIV Pseudomonadati Acidobacteriota unclassified Acidobacteriota Acidobacteriota bacterium isolate J06635 18 319 aa
BacClassIV Pseudomonadati PVC Planctomycetota Planctomycetota bacterium isolate NASQAN2011 262 B bin.11 326 aa
BacClassIV Pseudomonadati PVC Planctomycetota Phycisphaerae bacterium isolate ELA279 320 aa
BacClassIV Pseudomonadati Acidobacteriota Terriglobia Terriglobales Candidatus Sulfotelmatobacter sp isolate SMAG U15194 335 aa
BacClassIV Bacteria incertae sedis Candidatus Eisenbacteria bacterium isolate pamunkeyriver 2019 sw WHONDRS 319 aa
BacClassIV unclassified Bacteria unclassified Bacteria miscellaneous taxid 49928 320 aa
BacClassIV Bacteria incertae sedis Candidatus Eisenbacteria bacterium isolate NASQAN2015 150 F bin.10 320 aa
BacClassIV Pseudomonadati Myxococcota Myxococcia Myxococcales Myxococcaceae bacterium isolate RGIG5499 308 aa
BacClassIV Pseudomonadati Myxococcota Myxococcia Myxococcales Myxococcaceae bacterium isolate SMAG U5157 306 aa
BacClassIV Pseudomonadati Myxococcota Myxococcia Myxococcales Myxococcaceae bacterium isolate SMAG U1027 306 aa
BacClassIV Pseudomonadati Myxococcota Myxococcia Myxococcales Myxococcaceae bacterium isolate metabat2.71 306 aa
BacClassIV Pseudomonadati Myxococcota Myxococcia Myxococcales Myxococcaceae bacterium isolate SMAG U3782 iso2 306 aa
BacClassIV Pseudomonadati Myxococcota Myxococcia Myxococcales Myxococcaceae bacterium isolate SMAG U3782 iso1 306 aa
BacClassIV Pseudomonadati Myxococcota Myxococcia Myxococcales Myxococcaceae bacterium isolate SMAG U15085 309 aa
BacClassIV Pseudomonadati Myxococcota Myxococcia Myxococcales Myxococcaceae bacterium isolate SMAG U10262 HZJ55972.1 309 aa
BacClassIV Pseudomonadati Myxococcota Myxococcia Myxococcales Myxococcaceae bacterium isolate SMAG U15212 309 aa
BacClassIV Pseudomonadati Myxococcota Myxococcia Myxococcales Myxococcaceae bacterium isolate SMAG U8464 306 aa
BacClassIV Pseudomonadati Myxococcota Myxococcia Myxococcales Myxococcaceae bacterium isolate SMAG U15944 309 aa
BacClassIV Pseudomonadati Myxococcota Myxococcia Myxococcales Myxococcaceae bacterium isolate SJ.T10 3 semi263 MGZ6028455.1 260 aa
BacClassIV Pseudomonadati PVC Planctomycetota Planctomycetota bacterium isolate NC groundwater 805 Pr6 B 0.1um 69 24 258 aa
BacClassIV unclassified Bacteria unclassified Bacteria miscellaneous taxid 49936 isolate MEGAHIT 3.13 320 aa
BacClassIV Pseudomonadati PVC Planctomycetota Phycisphaerae bacterium isolate SMAG U4706 283 aa
BacClassIV Pseudomonadati PVC Planctomycetota Planctomycetota bacterium isolate MAG.349 300 aa
BacClassIV delta epsilon subdivisions Deltabacteria unclassified Deltabacteria Deltaproteobacteria bacterium isolate SJ496 305 aa
BacClassIV Pseudomonadati Myxococcota Polyangia Polyangiales Polyangiaceae bacterium isolate STL MYX15 341 aa
BacClassIV Bacillati Actinomycetota Acidimicrobiia Acidimicrobiaceae bacterium isolate RYN 47 311 aa
BacClassIV Pseudomonadati PVC Planctomycetota Phycisphaerales bacterium isolate ABC1.27 310 aa
BacClassIV Pseudomonadati PVC Planctomycetota Lacipirellula sp isolate SMAG U2006 310 aa
BacClassIV Pseudomonadati PVC Planctomycetota Pirellula sp isolate PB1.3.31 311 aa
BacClassIV Pseudomonadati PVC Planctomycetota Lacipirellula parvula 310 aa
BacClassIV Pseudomonadati Pseudomonadota unclassified Pseudomonadota Pseudomonadota bacterium isolate MAG.37 308 aa
BacClassIV Pseudomonadati Myxococcota Polyangia Polyangiales Kofleriaceae bacterium isolate NORP324 306 aa
BacClassIV Pseudomonadati Myxococcota Polyangia Polyangiales Haliangium ochraceum 306 aa
BacClassIV Pseudomonadati Myxococcota Polyangia Polyangiales Haliangium sp isolate SMAG U13373 306 aa
BacClassIV Pseudomonadati Myxococcota Polyangia Haliangiales Haliangiales bacterium isolate Tsb0020 iso2 317 aa
BacClassIV Pseudomonadati Myxococcota Polyangia Polyangiales Kofleriaceae bacterium isolate SMAG U893 308 aa
BacClassIV Pseudomonadati Myxococcota Polyangia Polyangiales Kofleriaceae bacterium isolate SMAG U15438 303 aa
BacClassIV Pseudomonadati Myxococcota Polyangia Polyangiales Kofleriaceae bacterium isolate SMAG U14421 305 aa
BacClassIV Pseudomonadati Myxococcota Polyangia Polyangiales Kofleriaceae bacterium isolate SMAG U4133 307 aa
BacClassIV Pseudomonadati Myxococcota Polyangia Polyangiales Kofleriaceae bacterium isolate SMAG U2429 306 aa
BacClassIV Pseudomonadati Myxococcota Polyangia Polyangiales Kofleriaceae bacterium isolate SMAG U14291 306 aa
BacClassIV Pseudomonadati Myxococcota Polyangia Polyangiales Kofleriaceae bacterium isolate SMAG U12585 323 aa
BacClassIV Pseudomonadati Myxococcota Polyangia Polyangiales Kofleriaceae bacterium isolate SMAG U9370 313 aa
BacClassIV delta epsilon subdivisions Deltabacteria unclassified Deltabacteria Deltaproteobacteria bacterium ADurb.Bin207 306 aa
BacClassIV Pseudomonadati Myxococcota Polyangia Polyangiales Kofleriaceae bacterium isolate SMAG U13857 305 aa
BacClassIV Pseudomonadati Myxococcota Polyangia Polyangiales Kofleriaceae bacterium isolate H4 MYX2 305 aa
BacClassIV Pseudomonadati Myxococcota Polyangia Polyangiales Kofleriaceae bacterium isolate SMAG U14365 305 aa
BacClassIV Pseudomonadati Myxococcota Polyangia Polyangiales Kofleriaceae bacterium isolate MAG 26 305 aa
BacClassIV Pseudomonadati Myxococcota Polyangia Polyangiales Kofleriaceae bacterium isolate Gw SIPrim bin 156 305 aa
BacClassIV Pseudomonadati Myxococcota Polyangia Polyangiales Kofleriaceae bacterium isolate Go SIAct bin 429 317 aa
BacClassIV Pseudomonadati Myxococcota Polyangia Polyangiales Kofleriaceae bacterium isolate ABC1.117 304 aa
BacClassIV Pseudomonadati Myxococcota Polyangia Polyangiales Kofleriaceae bacterium isolate SMAG U6175 305 aa
BacClassIV Pseudomonadati Myxococcota Polyangia Polyangiales Kofleriaceae bacterium isolate HK STAS MYXO 12 306 aa
BacClassIV Pseudomonadati Myxococcota Polyangia Polyangiales Kofleriaceae bacterium isolate SMAG U15526 312 aa
BacClassIV Pseudomonadati Myxococcota Polyangia Polyangiales Kofleriaceae bacterium isolate BC1.218 305 aa
BacClassIV Pseudomonadati Myxococcota Polyangia Polyangiales Kofleriaceae bacterium isolate AMR MDS 5390 305 aa
BacClassIV Pseudomonadati Myxococcota Polyangia Polyangiales Kofleriaceae bacterium isolate Gw SIAct bin 39 303 aa
BacClassIV Pseudomonadati Myxococcota Polyangia Polyangiales Kofleriaceae bacterium isolate AMR MDS 3354 303 aa
BacClassIV Pseudomonadati Myxococcota Polyangia Polyangiales Kofleriaceae bacterium isolate SMAG U5708 307 aa
BacClassIV Pseudomonadati Myxococcota Polyangia Polyangiales Kofleriaceae bacterium isolate SMAG U15319 306 aa
BacClassIV Pseudomonadati Myxococcota Polyangia Polyangiales Kofleriaceae bacterium isolate SMAG U15125 309 aa
BacClassIV Pseudomonadati Myxococcota Polyangia Polyangiales Kofleriaceae bacterium isolate SMAG U5072 306 aa
BacClassIV Pseudomonadati Myxococcota Polyangia Polyangiales Kofleriaceae bacterium isolate SMAG U5105 305 aa
BacClassIV Pseudomonadati Myxococcota Polyangia Polyangiales Kofleriaceae bacterium isolate SMAG U5129 305 aa
BacClassIV Pseudomonadati Myxococcota Polyangia Polyangiales Kofleriaceae bacterium isolate SMAG U4851 307 aa
BacClassIV Pseudomonadati Myxococcota Polyangia Polyangiales Kofleriaceae bacterium isolate cluster2 bin.64 306 aa
BacClassIV Pseudomonadati Myxococcota Polyangia Polyangiales Kofleriaceae bacterium isolate SMAG U3032 277 aa
BacClassIV Pseudomonadati Myxococcota Polyangia Polyangiales Kofleriaceae bacterium isolate SMAG U171 307 aa
BacClassIV Pseudomonadati Myxococcota Polyangia Polyangiales Kofleriaceae bacterium isolate cluster2 bin.199 325 aa
BacClassIV Pseudomonadati Myxococcota Myxococcia Myxococcales Myxococcales bacterium isolate RYN 365 iso2 MDB4961381.1 306 aa
BacClassIV Pseudomonadati Myxococcota Polyangia Polyangiales Kofleriaceae bacterium isolate F1F2.bin.256 309 aa
BacClassIV Pseudomonadati Myxococcota Polyangia Polyangiales Kofleriaceae bacterium isolate SMAG U13645 308 aa
BacClassIV Pseudomonadati Myxococcota Polyangia Polyangiales Kofleriaceae bacterium isolate bins.76 307 aa
BacClassIV Pseudomonadati Myxococcota Polyangia Polyangiales Kofleriaceae bacterium isolate SMAG U4951 310 aa
BacClassIV Pseudomonadati Myxococcota Polyangia Polyangiales Kofleriaceae bacterium isolate SMAG U4917 306 aa
BacClassIV Pseudomonadati Myxococcota Polyangia Polyangiales Kofleriaceae bacterium isolate Bin8.14 306 aa
BacClassIV Pseudomonadati Acidobacteriota Vicinamibacteria Vicinamibacteria bacterium isolate R2F R2FSRR metabat.34 306 aa
BacClassIV Pseudomonadati Myxococcota Polyangia Polyangiales Kofleriaceae bacterium isolate SMAG U2250 306 aa
BacClassIV Pseudomonadati Myxococcota Polyangia Polyangiales Kofleriaceae bacterium isolate SMAG U1390 306 aa
BacClassIV Pseudomonadati Myxococcota Polyangia Polyangiales Kofleriaceae bacterium isolate SMAG U156 306 aa
BacClassIV Pseudomonadati Myxococcota Polyangia Polyangiales Polyangiaceae bacterium isolate Prado144 310 aa

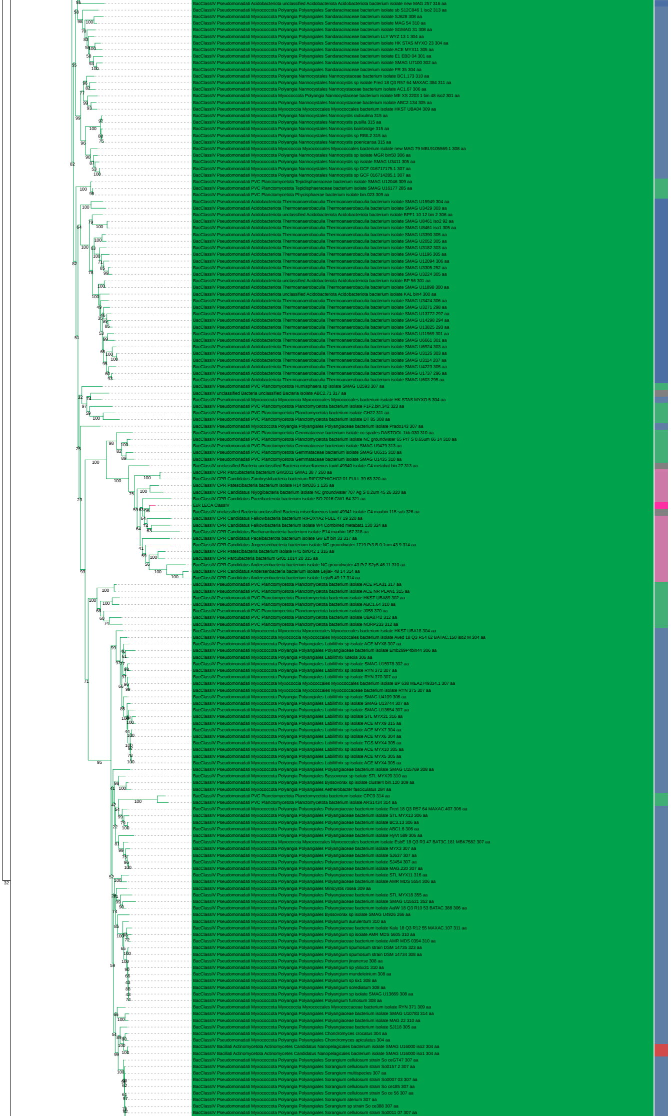
BacClassIV Pseudomonadati Acidobacteriota unclassified Acidobacteriota Acidobacteriota bacterium isolate new MAG 257 316 aa
BacClassIV Pseudomonadati Myxococcota Polyangia Polyangiales Sandaracinaceae bacterium isolate sb S12C646 1 iso2 313 aa
BacClassIV Pseudomonadati Myxococcota Polyangia Polyangiales Sandaracinaceae bacterium isolate SJ628 308 aa
BacClassIV Pseudomonadati Myxococcota Polyangia Polyangiales Sandaracinaceae bacterium isolate MAG 54 310 aa
BacClassIV Pseudomonadati Myxococcota Polyangia Polyangiales Sandaracinaceae bacterium isolate SGMAG 31 308 aa
BacClassIV Pseudomonadati Myxococcota Polyangia Polyangiales Sandaracinaceae bacterium LLY WYZ 13 1 304 aa
BacClassIV Pseudomonadati Myxococcota Polyangia Polyangiales Sandaracinaceae bacterium isolate HK STAS MYXO 23 304 aa
BacClassIV Pseudomonadati Myxococcota Polyangia Polyangiales Sandaracinaceae bacterium isolate ACE MYX11 305 aa
BacClassIV Pseudomonadati Myxococcota Polyangia Polyangiales Sandaracinaceae bacterium isolate E1 EBD 04 301 aa
BacClassIV Pseudomonadati Myxococcota Polyangia Polyangiales Sandaracinaceae bacterium isolate SMAG U7100 302 aa
BacClassIV Pseudomonadati Myxococcota Polyangia Polyangiales Sandaracinaceae bacterium isolate FR 35 304 aa
BacClassIV Pseudomonadati Myxococcota Polyangia Nannocystales Nannocystaceae bacterium isolate BC1.173 310 aa
BacClassIV Pseudomonadati Myxococcota Polyangia Nannocystales Nannocystis sp isolate Fred 18 Q3 R57 64 MAXAC.384 311 aa
BacClassIV Pseudomonadati Myxococcota Polyangia Nannocystales Nannocystaceae bacterium isolate AC1.67 306 aa
BacClassIV Pseudomonadati Myxococcota Myxococcota Polyangia Nannocystaceae bacterium isolate ME XS 2203 1 bin 48 iso2 301 aa
BacClassIV Pseudomonadati Myxococcota Polyangia Nannocystales Nannocystaceae bacterium isolate ABC2.134 305 aa
BacClassIV Pseudomonadati Myxococcota Myxococcia Myxococcales Myxococcales bacterium isolate HKST UBA04 309 aa
BacClassIV Pseudomonadati Myxococcota Polyangia Nannocystales Nannocystis radixulma 315 aa
BacClassIV Pseudomonadati Myxococcota Polyangia Nannocystales Nannocystis pusilla 315 aa
BacClassIV Pseudomonadati Myxococcota Polyangia Nannocystales Nannocystis bainbridge 315 aa
BacClassIV Pseudomonadati Myxococcota Polyangia Nannocystales Nannocystis sp RBIL2 315 aa
BacClassIV Pseudomonadati Myxococcota Polyangia Nannocystales Nannocystis poenicansa 315 aa
BacClassIV Pseudomonadati Myxococcota Myxococcia Myxococcales Myxococcales bacterium isolate new MAG 79 MBL9105569.1 308 aa
BacClassIV Pseudomonadati Myxococcota Polyangia Nannocystales Nannocystis sp isolate MGR bin50 306 aa
BacClassIV Pseudomonadati Myxococcota Polyangia Nannocystales Nannocystis sp isolate SMAG U3411 305 aa
BacClassIV Pseudomonadati Myxococcota Polyangia Nannocystales Nannocystis sp GCF 016717175.1 307 aa
BacClassIV Pseudomonadati Myxococcota Polyangia Nannocystales Nannocystis sp GCF 016714285.1 307 aa
BacClassIV Pseudomonadati PVC Planctomycetota Tepidisphaeraceae bacterium isolate SMAG U12046 309 aa
BacClassIV Pseudomonadati PVC Planctomycetota Tepidisphaeraceae bacterium isolate SMAG U16177 285 aa
BacClassIV Pseudomonadati PVC Planctomycetota Phycisphaerae bacterium isolate bin.023 309 aa
BacClassIV Pseudomonadati Acidobacteriota Thermoanaerobaculia Thermoanaerobaculia bacterium isolate SMAG U15949 304 aa
BacClassIV Pseudomonadati Acidobacteriota Thermoanaerobaculia Thermoanaerobaculia bacterium isolate SMAG U3429 303 aa
BacClassIV Pseudomonadati Acidobacteriota unclassified Acidobacteriota Acidobacteriota bacterium isolate BPF1 10 12 bin 2 306 aa
BacClassIV Pseudomonadati Acidobacteriota Thermoanaerobaculia Thermoanaerobaculia bacterium isolate SMAG U8461 iso2 92 aa
BacClassIV Pseudomonadati Acidobacteriota Thermoanaerobaculia Thermoanaerobaculia bacterium isolate SMAG U8461 iso1 305 aa
BacClassIV Pseudomonadati Acidobacteriota Thermoanaerobaculia Thermoanaerobaculia bacterium isolate SMAG U3390 305 aa
BacClassIV Pseudomonadati Acidobacteriota Thermoanaerobaculia Thermoanaerobaculia bacterium isolate SMAG U2052 305 aa
BacClassIV Pseudomonadati Acidobacteriota Thermoanaerobaculia Thermoanaerobaculia bacterium isolate SMAG U3182 303 aa
BacClassIV Pseudomonadati Acidobacteriota Thermoanaerobaculia Thermoanaerobaculia bacterium isolate SMAG U1196 305 aa
BacClassIV Pseudomonadati Acidobacteriota Thermoanaerobaculia Thermoanaerobaculia bacterium isolate SMAG U12094 306 aa
BacClassIV Pseudomonadati Acidobacteriota Thermoanaerobaculia Thermoanaerobaculia bacterium isolate SMAG U3305 252 aa
BacClassIV Pseudomonadati Acidobacteriota Thermoanaerobaculia Thermoanaerobaculia bacterium isolate SMAG U3224 305 aa
BacClassIV Pseudomonadati Acidobacteriota unclassified Acidobacteriota Acidobacteriota bacterium isolate BP 56 301 aa
BacClassIV Pseudomonadati Acidobacteriota Thermoanaerobaculia Thermoanaerobaculia bacterium isolate SMAG U11898 300 aa
BacClassIV Pseudomonadati Acidobacteriota unclassified Acidobacteriota Acidobacteriota bacterium isolate KAL bin4 300 aa
BacClassIV Pseudomonadati Acidobacteriota Thermoanaerobaculia Thermoanaerobaculia bacterium isolate SMAG U3424 306 aa
BacClassIV Pseudomonadati Acidobacteriota Thermoanaerobaculia Thermoanaerobaculia bacterium isolate SMAG U3271 298 aa
BacClassIV Pseudomonadati Acidobacteriota Thermoanaerobaculia Thermoanaerobaculia bacterium isolate SMAG U13772 297 aa
BacClassIV Pseudomonadati Acidobacteriota Thermoanaerobaculia Thermoanaerobaculia bacterium isolate SMAG U14298 294 aa
BacClassIV Pseudomonadati Acidobacteriota Thermoanaerobaculia Thermoanaerobaculia bacterium isolate SMAG U13825 293 aa
BacClassIV Pseudomonadati Acidobacteriota Thermoanaerobaculia Thermoanaerobaculia bacterium isolate SMAG U11969 301 aa
BacClassIV Pseudomonadati Acidobacteriota Thermoanaerobaculia Thermoanaerobaculia bacterium isolate SMAG U6661 301 aa
BacClassIV Pseudomonadati Acidobacteriota Thermoanaerobaculia Thermoanaerobaculia bacterium isolate SMAG U6924 303 aa
BacClassIV Pseudomonadati Acidobacteriota Thermoanaerobaculia Thermoanaerobaculia bacterium isolate SMAG U3126 303 aa
BacClassIV Pseudomonadati Acidobacteriota Thermoanaerobaculia Thermoanaerobaculia bacterium isolate SMAG U3114 207 aa
BacClassIV Pseudomonadati Acidobacteriota Thermoanaerobaculia Thermoanaerobaculia bacterium isolate SMAG U4223 305 aa
BacClassIV Pseudomonadati Acidobacteriota Thermoanaerobaculia Thermoanaerobaculia bacterium isolate SMAG U1737 296 aa
BacClassIV Pseudomonadati Acidobacteriota Thermoanaerobaculia Thermoanaerobaculia bacterium isolate SMAG U603 295 aa
BacClassIV Pseudomonadati PVC Planctomycetota Humisphaera sp isolate SMAG U2593 307 aa
BacClassIV unclassified Bacteria unclassified Bacteria isolate ABC2.71 317 aa
BacClassIV Pseudomonadati Myxococcota Myxococcia Myxococcales Myxococcales bacterium isolate HK STAS MYXO 5 304 aa
BacClassIV Pseudomonadati PVC Planctomycetota Planctomycetota bacterium isolate F1F2.bin.342 323 aa
BacClassIV Pseudomonadati PVC Planctomycetota Planctomycetota bacterium isolate GH22 311 aa
BacClassIV Pseudomonadati PVC Planctomycetota Planctomycetota bacterium isolate DT 85 308 aa
BacClassIV Pseudomonadati Myxococcota Polyangia Polyangiales Polyangiaceae bacterium isolate Prado143 307 aa
BacClassIV Pseudomonadati PVC Planctomycetota Gemmataceae bacterium isolate co.spades.DASTOOL.1kb 030 310 aa
BacClassIV Pseudomonadati PVC Planctomycetota Planctomycetota bacterium isolate NC groundwater 65 Pr7 S 0.65um 66 14 310 aa
BacClassIV Pseudomonadati PVC Planctomycetota Gemmataceae bacterium isolate SMAG U9479 313 aa
BacClassIV Pseudomonadati PVC Planctomycetota Gemmataceae bacterium isolate SMAG U6515 310 aa
BacClassIV Pseudomonadati PVC Planctomycetota Gemmataceae bacterium isolate SMAG U1435 310 aa
BacClassIV unclassified Bacteria unclassified Bacteria miscellaneous taxid 49940 isolate C4 metabat.bin.27 313 aa
BacClassIV CPR Parcubacteria bacterium GW2011 GWA1 38 7 260 aa
BacClassIV CPR Candidatus Zambryskibacteria bacterium RIFCSPHIGHO2 01 FULL 39 63 320 aa
BacClassIV CPR Patescibacteria bacterium isolate H14 bin026 1 126 aa
BacClassIV CPR Candidatus Niyogibacteria bacterium isolate NC groundwater 707 Ag S 0.2um 45 26 320 aa
BacClassIV CPR Candidatus Paceibacterota bacterium isolate SO 2016 GW1 64 321 aa
Euk LECA ClassIV
BacClassIV unclassified Bacteria unclassified Bacteria miscellaneous taxid 49941 isolate C4 maxbin.115 sub 326 aa
BacClassIV CPR Candidatus Falkowbacteria bacterium RIFOXYA2 FULL 47 19 320 aa
BacClassIV CPR Candidatus Falkowbacteria bacterium isolate W4 Combined metabat1 130 324 aa
BacClassIV CPR Candidatus Buchananbacteria bacterium isolate E14 maxbin.167 318 aa
BacClassIV CPR Candidatus Paceibacteria bacterium isolate Gw Eff bin 33 317 aa
BacClassIV CPR Candidatus Jorgensenbacteria bacterium isolate NC groundwater 1719 Pr3 B 0.1um 43 9 314 aa
BacClassIV CPR Patescibacteria bacterium isolate H41 bin042 1 316 aa
BacClassIV CPR Parcubacteria bacterium Gr01 1014 20 315 aa
BacClassIV CPR Candidatus Andersenbacteria bacterium isolate NC groundwater 43 Pr7 S2p5 46 11 310 aa
BacClassIV CPR Candidatus Andersenbacteria bacterium isolate LejiaF 48 14 314 aa
BacClassIV CPR Candidatus Andersenbacteria bacterium isolate LejiaB 49 17 314 aa
BacClassIV Pseudomonadati PVC Planctomycetota Planctomycetota bacterium isolate ACE PLA31 317 aa
BacClassIV Pseudomonadati PVC Planctomycetota Planctomycetota bacterium isolate ACE NR PLAN1 315 aa
BacClassIV Pseudomonadati PVC Planctomycetota Planctomycetota bacterium isolate HKST UBA89 302 aa
BacClassIV Pseudomonadati PVC Planctomycetota Planctomycetota bacterium isolate ABC1.64 310 aa
BacClassIV Pseudomonadati PVC Planctomycetota Planctomycetota bacterium isolate J058 370 aa
BacClassIV Pseudomonadati PVC Planctomycetota Planctomycetota bacterium isolate UBA8742 312 aa
BacClassIV Pseudomonadati PVC Planctomycetota Planctomycetota bacterium isolate NORP233 312 aa
BacClassIV Pseudomonadati Myxococcota Myxococcia Myxococcales Myxococcales bacterium isolate HKST UBA18 304 aa
BacClassIV Pseudomonadati Myxococcota Myxococcia Myxococcales Myxococcales bacterium isolate Aved 18 Q3 R54 62 BATAC.150 iso2 M 304 aa
BacClassIV Pseudomonadati Myxococcota Polyangia Polyangiales Labilithrix sp isolate ACE MYX8 307 aa
BacClassIV Pseudomonadati Myxococcota Polyangia Polyangiales Polyangiaceae bacterium isolate Emb289P4bin44 306 aa
BacClassIV Pseudomonadati Myxococcota Polyangia Polyangiales Labilithrix luteola 306 aa
BacClassIV Pseudomonadati Myxococcota Polyangia Polyangiales Labilithrix sp isolate SMAG U15978 302 aa
BacClassIV Pseudomonadati Myxococcota Polyangia Polyangiales Labilithrix sp isolate RYN 372 307 aa
BacClassIV Pseudomonadati Myxococcota Polyangia Polyangiales Labilithrix sp isolate RYN 370 307 aa
BacClassIV Pseudomonadati Myxococcota Myxococcia Myxococcales Myxococcales bacterium isolate BP 638 MEA2749334.1 307 aa
BacClassIV Pseudomonadati Myxococcota Myxococcia Myxococcales Myxococcaceae bacterium isolate RYN 375 307 aa
BacClassIV Pseudomonadati Myxococcota Polyangia Polyangiales Labilithrix sp isolate SMAG U4109 306 aa
BacClassIV Pseudomonadati Myxococcota Polyangia Polyangiales Labilithrix sp isolate SMAG U13744 307 aa
BacClassIV Pseudomonadati Myxococcota Polyangia Polyangiales Labilithrix sp isolate SMAG U13654 307 aa
BacClassIV Pseudomonadati Myxococcota Polyangia Polyangiales Labilithrix sp isolate STL MYX21 316 aa
BacClassIV Pseudomonadati Myxococcota Polyangia Polyangiales Labilithrix sp isolate ACE MYX9 315 aa
BacClassIV Pseudomonadati Myxococcota Polyangia Polyangiales Labilithrix sp isolate ACE MYX7 304 aa
BacClassIV Pseudomonadati Myxococcota Polyangia Polyangiales Labilithrix sp isolate ACE MYX6 304 aa
BacClassIV Pseudomonadati Myxococcota Polyangia Polyangiales Labilithrix sp isolate TGS MYX4 305 aa
BacClassIV Pseudomonadati Myxococcota Polyangia Polyangiales Labilithrix sp isolate ACE MYX10 305 aa
BacClassIV Pseudomonadati Myxococcota Polyangia Polyangiales Labilithrix sp isolate ACE MYX5 305 aa
BacClassIV Pseudomonadati Myxococcota Polyangia Polyangiales Labilithrix sp isolate ACE MYX4 305 aa
BacClassIV Pseudomonadati Myxococcota Polyangia Polyangiales Polyangiaceae bacterium isolate SMAG U15769 308 aa
BacClassIV Pseudomonadati Myxococcota Polyangia Polyangiales Byssovorax sp isolate STL MYX20 310 aa
BacClassIV Pseudomonadati Myxococcota Polyangia Polyangiales Byssovorax sp isolate cluster4 bin.120 309 aa
BacClassIV Pseudomonadati Myxococcota Polyangia Polyangiales Aetherobacter fasciculatus 284 aa
BacClassIV Pseudomonadati PVC Planctomycetota Planctomycetota bacterium isolate CPC9 314 aa
BacClassIV Pseudomonadati PVC Planctomycetota Planctomycetota bacterium isolate ARS1434 314 aa
BacClassIV Pseudomonadati Myxococcota Polyangia Polyangiales Polyangiaceae bacterium isolate Fred 18 Q3 R57 64 MAXAC.407 306 aa
BacClassIV Pseudomonadati Myxococcota Polyangia Polyangiales Polyangiaceae bacterium isolate STL MYX13 306 aa
BacClassIV Pseudomonadati Myxococcota Polyangia Polyangiales Polyangiaceae bacterium isolate BC3.13 306 aa
BacClassIV Pseudomonadati Myxococcota Polyangia Polyangiales Polyangiaceae bacterium isolate ABC1.6 306 aa
BacClassIV Pseudomonadati Myxococcota Polyangia Polyangiales Polyangiaceae bacterium isolate HyVt 589 306 aa
BacClassIV Pseudomonadati Myxococcota Myxococcia Myxococcales Myxococcales bacterium isolate EsbE 18 Q3 R3 47 BAT3C.181 MBK7582 307 aa
BacClassIV Pseudomonadati Myxococcota Polyangia Polyangiales Polyangiaceae bacterium isolate MYX3 307 aa
BacClassIV Pseudomonadati Myxococcota Polyangia Polyangiales Polyangiaceae bacterium isolate SJ637 307 aa
BacClassIV Pseudomonadati Myxococcota Polyangia Polyangiales Polyangiaceae bacterium isolate SJ454 307 aa
BacClassIV Pseudomonadati Myxococcota Polyangia Polyangiales Polyangiaceae bacterium isolate MAG.220 307 aa
BacClassIV Pseudomonadati Myxococcota Polyangia Polyangiales Polyangiaceae bacterium isolate STL MYX11 316 aa
BacClassIV Pseudomonadati Myxococcota Polyangia Polyangiales Polyangiaceae bacterium isolate AMR MDS 5554 306 aa
BacClassIV Pseudomonadati Myxococcota Polyangia Polyangiales Minicystis rosea 309 aa
BacClassIV Pseudomonadati Myxococcota Polyangia Polyangiales Polyangiaceae bacterium isolate STL MYX18 355 aa
BacClassIV Pseudomonadati Myxococcota Polyangia Polyangiales Polyangiaceae bacterium isolate SMAG U15521 352 aa
BacClassIV Pseudomonadati Myxococcota Polyangia Polyangiales Polyangiaceae bacterium isolate AalW 18 Q3 R10 53 BATAC.388 306 aa
BacClassIV Pseudomonadati Myxococcota Polyangia Polyangiales Byssovorax sp isolate SMAG U4926 266 aa
BacClassIV Pseudomonadati Myxococcota Polyangia Polyangiales Polyangium aurulentum 310 aa
BacClassIV Pseudomonadati Myxococcota Polyangia Polyangiales Polyangiaceae bacterium isolate Kalu 18 Q3 R12 55 MAXAC.107 311 aa
BacClassIV Pseudomonadati Myxococcota Polyangia Polyangiales Polyangium sp isolate AMR MDS 5605 310 aa
BacClassIV Pseudomonadati Myxococcota Polyangia Polyangiales Polyangiaceae bacterium isolate AMR MDS 0394 310 aa
BacClassIV Pseudomonadati Myxococcota Polyangia Polyangiales Polyangium spumosum strain DSM 14735 323 aa
BacClassIV Pseudomonadati Myxococcota Polyangia Polyangiales Polyangium spumosum strain DSM 14734 308 aa
BacClassIV Pseudomonadati Myxococcota Polyangia Polyangiales Polyangium jinanense 308 aa
BacClassIV Pseudomonadati Myxococcota Polyangia Polyangiales Polyangium sp y55x31 310 aa
BacClassIV Pseudomonadati Myxococcota Polyangia Polyangiales Polyangium mundeleinium 308 aa
BacClassIV Pseudomonadati Myxococcota Polyangia Polyangiales Polyangium sp 6x1 308 aa
BacClassIV Pseudomonadati Myxococcota Polyangia Polyangiales Polyangium sorediatum 308 aa
BacClassIV Pseudomonadati Myxococcota Polyangia Polyangiales Polyangium sp isolate SMAG U13669 308 aa
BacClassIV Pseudomonadati Myxococcota Polyangia Polyangiales Polyangium fumosum 308 aa
BacClassIV Pseudomonadati Myxococcota Myxococcia Myxococcales Myxococcaceae bacterium isolate RYN 371 309 aa
BacClassIV Pseudomonadati Myxococcota Polyangia Polyangiales Polyangiaceae bacterium isolate SMAG U10783 314 aa
BacClassIV Pseudomonadati Myxococcota Polyangia Polyangiales Polyangiaceae bacterium isolate MAG 22 310 aa
BacClassIV Pseudomonadati Myxococcota Polyangia Polyangiales Polyangiaceae bacterium isolate SJ118 305 aa
BacClassIV Pseudomonadati Myxococcota Polyangia Polyangiales Chondromyces crocatus 304 aa
BacClassIV Pseudomonadati Myxococcota Polyangia Polyangiales Chondromyces apiculatus 304 aa
BacClassIV Bacillati Actinomycetota Actinomycetes Candidatus Nanopelagicales bacterium isolate SMAG U16000 iso2 304 aa
BacClassIV Bacillati Actinomycetota Actinomycetes Candidatus Nanopelagicales bacterium isolate SMAG U16000 iso1 304 aa
BacClassIV Pseudomonadati Myxococcota Polyangia Polyangiales Sorangium cellulosum strain So ceGT47 307 aa
BacClassIV Pseudomonadati Myxococcota Polyangia Polyangiales Sorangium cellulosum strain So0157 2 307 aa
BacClassIV Pseudomonadati Myxococcota Polyangia Polyangiales Sorangium multispecies 307 aa
BacClassIV Pseudomonadati Myxococcota Polyangia Polyangiales Sorangium cellulosum strain So0007 03 307 aa
BacClassIV Pseudomonadati Myxococcota Polyangia Polyangiales Sorangium cellulosum strain So ce185 307 aa
BacClassIV Pseudomonadati Myxococcota Polyangia Polyangiales Sorangium cellulosum strain So ce 56 307 aa
BacClassIV Pseudomonadati Myxococcota Polyangia Polyangiales Sorangium aterium 307 aa
BacClassIV Pseudomonadati Myxococcota Polyangia Polyangiales Sorangium sp strain So ce388 307 aa
BacClassIV Pseudomonadati Myxococcota Polyangia Polyangiales Sorangium cellulosum strain So0011 07 307 aa

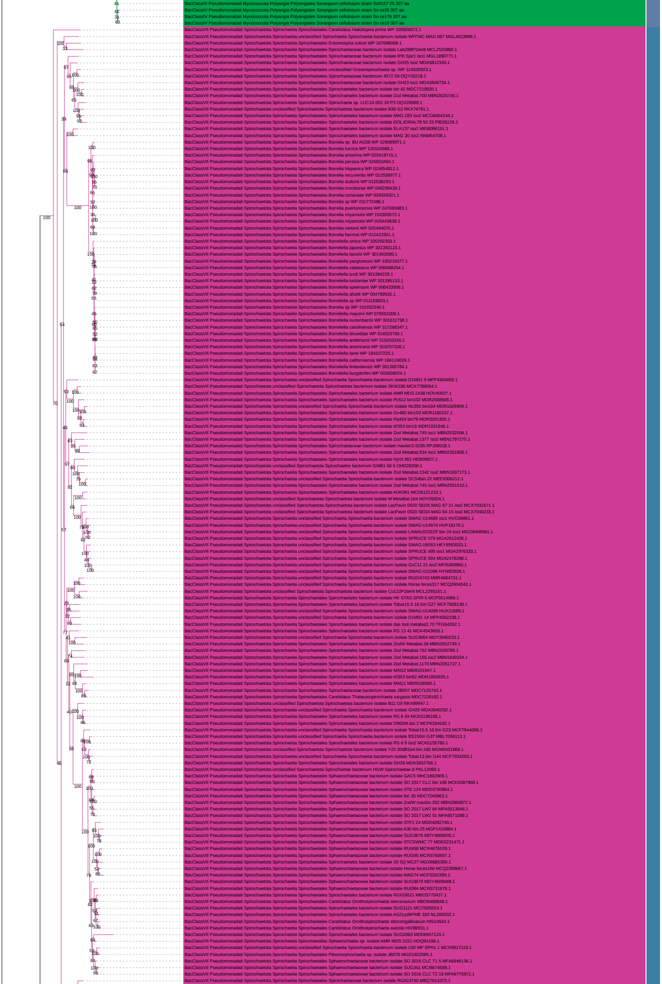

BacClassIV Pseudomonadati Myxococcota Polyangia Polyangiales Sorangium cellulosum strain So0157 25 307 aa
BacClassIV Pseudomonadati Myxococcota Polyangia Polyangiales Sorangium cellulosum strain So ce26 307 aa
BacClassIV Pseudomonadati Myxococcota Polyangia Polyangiales Sorangium cellulosum strain So ce176 307 aa
BacClassIV Pseudomonadati Myxococcota Polyangia Polyangiales Sorangium cellulosum strain So ce10 307 aa
BacClassVII Pseudomonadati Spirochaetota Spirochaetia Spirochaetales Candidatus Haliotispira prima WP 326926672.1
BacClassVII Pseudomonadati Spirochaetota Spirochaetia unclassified Spirochaetia Spirochaetia bacterium isolate WFFMC MAG 087 MGL4523898.1
BacClassVII Pseudomonadati Spirochaetota Spirochaetia Spirochaetales Entomospira culicis WP 167696068.1
BacClassVII Pseudomonadati Spirochaetota Spirochaetia Spirochaetales Spirochaetaceae bacterium isolate Lab288P1bin8 MCL2520860.1
BacClassVII Pseudomonadati Spirochaetota Spirochaetia Spirochaetales Spirochaetaceae bacterium isolate tPB Spir1 iso1 MGL1890771.1
BacClassVII Pseudomonadati Spirochaetota Spirochaetia Spirochaetales Spirochaetaceae bacterium isolate GH25 iso2 MDA3812343.1
BacClassVII Pseudomonadati Spirochaetota Spirochaetia Spirochaetales unclassified Oceanispirochaeta sp. WP 114630923.1
BacClassVII Pseudomonadati Spirochaetota Spirochaetia Spirochaetales Spirochaetaceae bacterium 4572 59 OQY35218.1
BacClassVII Pseudomonadati Spirochaetota Spirochaetia Spirochaetales Spirochaetaceae bacterium isolate GH23 iso1 MDA3849734.1
BacClassVII Pseudomonadati Spirochaetota Spirochaetia Spirochaetales Spirochaetales bacterium isolate bin 42 MDC7219820.1
BacClassVII Pseudomonadati Spirochaetota Spirochaetia Spirochaetales Spirochaetales bacterium isolate Zod Metabat.700 MBN2625745.1
BacClassVII Pseudomonadati Spirochaetota Spirochaetia Spirochaetales Spirochaeta sp. LUC14 002 19 P3 OQX28969.1
BacClassVII Pseudomonadati Spirochaetota unclassified Spirochaetota Spirochaetota bacterium isolate B38 G2 RKX74761.1
BacClassVII Pseudomonadati Spirochaetota Spirochaetia Spirochaetales Spirochaetales bacterium isolate MAG.283 iso2 MCG8454244.1
BacClassVII Pseudomonadati Spirochaetota Spirochaetia Spirochaetales Spirochaetales bacterium isolate DOLJORAL78 50 23 PIE05126.1
BacClassVII Pseudomonadati Spirochaetota Spirochaetia Spirochaetales Spirochaetales bacterium isolate ELA137 iso2 MEI8096151.1
BacClassVII Pseudomonadati Spirochaetota Spirochaetia Spirochaetales Spirochaetales bacterium isolate MAG 30 iso2 NNM54708.1
BacClassVII Pseudomonadati Spirochaetota Spirochaetia Spirochaetales Borrelia sp. BU AG58 WP 229089071.1
BacClassVII Pseudomonadati Spirochaetota Spirochaetia Spirochaetales Borrelia turcica WP 120104668.1
BacClassVII Pseudomonadati Spirochaetota Spirochaetia Spirochaetales Borrelia anserina WP 025419715.1
BacClassVII Pseudomonadati Spirochaetota Spirochaetia Spirochaetales Borrelia persica WP 024653494.1
BacClassVII Pseudomonadati Spirochaetota Spirochaetia Spirochaetales Borrelia hispanica WP 024654812.1
BacClassVII Pseudomonadati Spirochaetota Spirochaetia Spirochaetales Borrelia recurrentis WP 012538977.1
BacClassVII Pseudomonadati Spirochaetota Spirochaetia Spirochaetales Borrelia duttonii WP 012538293.1
BacClassVII Pseudomonadati Spirochaetota Spirochaetia Spirochaetales Borrelia crocidurae WP 040236438.1
BacClassVII Pseudomonadati Spirochaetota Spirochaetia Spirochaetales Borrelia coriaceae WP 028328321.1
BacClassVII Pseudomonadati Spirochaetota Spirochaetia Spirochaetales Borrelia sp WP 011772486.1
BacClassVII Pseudomonadati Spirochaetota Spirochaetia Spirochaetales Borrelia puertoricensis WP 247065683.1
BacClassVII Pseudomonadati Spirochaetota Spirochaetia Spirochaetales Borrelia miyamotoi WP 152300572.1
BacClassVII Pseudomonadati Spirochaetota Spirochaetia Spirochaetales Borrelia miyamotoi WP 025443638.1
BacClassVII Pseudomonadati Spirochaetota Spirochaetia Spirochaetales Borrelia nietonii WP 025434076.1
BacClassVII Pseudomonadati Spirochaetota Spirochaetia Spirochaetales Borrelia hermsii WP 012422301.1
BacClassVII Pseudomonadati Spirochaetota Spirochaetia Spirochaetales Borreliella sinica WP 320292303.1
BacClassVII Pseudomonadati Spirochaetota Spirochaetia Spirochaetales Borreliella japonica WP 301393123.1
BacClassVII Pseudomonadati Spirochaetota Spirochaetia Spirochaetales Borreliella tanukii WP 301403589.1
BacClassVII Pseudomonadati Spirochaetota Spirochaetia Spirochaetales Borreliella yangtzensis WP 183219377.1
BacClassVII Pseudomonadati Spirochaetota Spirochaetia Spirochaetales Borreliella valaisiana WP 006068254.1
BacClassVII Pseudomonadati Spirochaetota Spirochaetia Spirochaetales Borreliella turdi WP 301394229.1
BacClassVII Pseudomonadati Spirochaetota Spirochaetia Spirochaetales Borreliella lusitaniae WP 301395153.1
BacClassVII Pseudomonadati Spirochaetota Spirochaetia Spirochaetales Borreliella spielmanii WP 006433906.1
BacClassVII Pseudomonadati Spirochaetota Spirochaetia Spirochaetales Borreliella afzelii WP 004789531.1
BacClassVII Pseudomonadati Spirochaetota Spirochaetia Spirochaetales Borreliella sp WP 011193853.1
BacClassVII Pseudomonadati Spirochaetota Spirochaetia Spirochaetales Borrelia sp WP 151552340.1
BacClassVII Pseudomonadati Spirochaetota Spirochaetia Spirochaetales Borreliella mayonii WP 075552208.1
BacClassVII Pseudomonadati Spirochaetota Spirochaetia Spirochaetales Borreliella kurtenbachii WP 301611738.1
BacClassVII Pseudomonadati Spirochaetota Spirochaetia Spirochaetales Borreliella carolinensis WP 317268347.1
BacClassVII Pseudomonadati Spirochaetota Spirochaetia Spirochaetales Borreliella bissettiae WP 014023760.1
BacClassVII Pseudomonadati Spirochaetota Spirochaetia Spirochaetales Borreliella andersonii WP 316255340.1
BacClassVII Pseudomonadati Spirochaetota Spirochaetia Spirochaetales Borreliella americana WP 316257326.1
BacClassVII Pseudomonadati Spirochaetota Spirochaetia Spirochaetales Borreliella lanei WP 184107225.1
BacClassVII Pseudomonadati Spirochaetota Spirochaetia Spirochaetales Borreliella californiensis WP 184124039.1
BacClassVII Pseudomonadati Spirochaetota Spirochaetia Spirochaetales Borreliella finlandensis WP 301365784.1
BacClassVII Pseudomonadati Spirochaetota Spirochaetia Spirochaetales Borreliella burgdorferi WP 002658024.1
BacClassVII Pseudomonadati Spirochaetota Spirochaetia unclassified Spirochaetia Spirochaetia bacterium isolate D1M01 9 MFP4363455.1
BacClassVII Pseudomonadati Spirochaetota unclassified Spirochaetota Spirochaetota bacterium isolate SKW186 MCX7786064.1
BacClassVII Pseudomonadati Spirochaetota Spirochaetia Spirochaetales Spirochaetales bacterium isolate AMR MDS 2438 HOV40027.1
BacClassVII Pseudomonadati Spirochaetota Spirochaetia Spirochaetales Spirochaetales bacterium isolate Pc512 bin102 MDR2588608.1
BacClassVII Pseudomonadati Spirochaetota Spirochaetia unclassified Spirochaetia Spirochaetia bacterium isolate Nc350 bin164 MDR1626906.1
BacClassVII Pseudomonadati Spirochaetota Spirochaetia Spirochaetales Spirochaetales bacterium isolate Gx485 bin103 MDR1180137.1
BacClassVII Pseudomonadati Spirochaetota Spirochaetia Spirochaetales Spirochaetales bacterium isolate Pq454 bin79 MDR3201305.1
BacClassVII Pseudomonadati Spirochaetota Spirochaetia Spirochaetales Spirochaetales bacterium isolate Kf353 bin16 MDR1931846.1
BacClassVII Pseudomonadati Spirochaetota Spirochaetia Spirochaetales Spirochaetales bacterium isolate Zod Metabat.749 iso1 MBN2532594.1
BacClassVII Pseudomonadati Spirochaetota Spirochaetia Spirochaetales Spirochaetales bacterium isolate Zod Metabat.1377 iso2 MBN1797275.1
BacClassVII Pseudomonadati Spirochaetota Spirochaetia Spirochaetales Spirochaetaceae bacterium isolate maxbin2.0195 RPJ08018.1
BacClassVII Pseudomonadati Spirochaetota Spirochaetia Spirochaetales Spirochaetales bacterium isolate Zod Metabat.934 iso1 MBN2351856.1
BacClassVII Pseudomonadati Spirochaetota Spirochaetia Spirochaetales Spirochaetales bacterium isolate HyVt 362 HEB09927.1
BacClassVII Pseudomonadati Spirochaetota unclassified Spirochaetota Spirochaetes bacterium GWB1 66 5 OHD28208.1
BacClassVII Pseudomonadati Spirochaetota Spirochaetia Spirochaetales Spirochaetales bacterium isolate Zod Metabat.1342 iso2 MBN1837173.1
BacClassVII Pseudomonadati Spirochaetota Spirochaetia unclassified Spirochaetia Spirochaetia bacterium isolate SCS4bin.22 MEE9306212.1
BacClassVII Pseudomonadati Spirochaetota Spirochaetia Spirochaetales Spirochaetales bacterium isolate Zod Metabat.745 iso1 MBN2551510.1
BacClassVII Pseudomonadati Spirochaetota Spirochaetia Spirochaetales Spirochaetales bacterium isolate AUK091 MCD6121210.1
BacClassVII Pseudomonadati Spirochaetota unclassified Spirochaetota Spirochaetota bacterium isolate M MetaBat.164 NOY09304.1
BacClassVII Pseudomonadati Spirochaetota unclassified Spirochaetota Spirochaetota bacterium isolate LacPavin 0920 SED5 MAG 67 21 iso2 MCX7031671.1
BacClassVII Pseudomonadati Spirochaetota unclassified Spirochaetota Spirochaetota bacterium isolate LacPavin 0920 SED4 MAG 64 15 iso2 MCX7040233.1
BacClassVII Pseudomonadati Spirochaetota Spirochaetia unclassified Spirochaetia Spirochaetia bacterium isolate SMAG U14685 iso2 HVO38661.1
BacClassVII Pseudomonadati Spirochaetota Spirochaetia unclassified Spirochaetia Spirochaetia bacterium isolate SMAG U14674 HVP18170.1
BacClassVII Pseudomonadati Spirochaetota Spirochaetia unclassified Spirochaetia Spirochaetia bacterium isolate LAWAr2D2E2F bin 24 iso1 MGO9409961.1
BacClassVII Pseudomonadati Spirochaetota Spirochaetia unclassified Spirochaetia Spirochaetia bacterium isolate SPRUCE 579 MGA2612426.1
BacClassVII Pseudomonadati Spirochaetota Spirochaetia unclassified Spirochaetia Spirochaetia bacterium isolate SMAG U6053 HEY9593033.1
BacClassVII Pseudomonadati Spirochaetota Spirochaetia unclassified Spirochaetia Spirochaetia bacterium isolate SPRUCE 495 iso1 MGA2976333.1
BacClassVII Pseudomonadati Spirochaetota Spirochaetia unclassified Spirochaetia Spirochaetia bacterium isolate SPRUCE 594 MGA2478288.1
BacClassVII Pseudomonadati Spirochaetota Spirochaetia unclassified Spirochaetia Spirochaetia bacterium isolate GcC11 21 iso2 MFI5369860.1
BacClassVII Pseudomonadati Spirochaetota Spirochaetia unclassified Spirochaetia Spirochaetia bacterium isolate SMAG U11096 HYW82606.1
BacClassVII Pseudomonadati Spirochaetota Spirochaetia unclassified Spirochaetia Spirochaetia bacterium isolate RGIG6743 MBR4684731.1
BacClassVII Pseudomonadati Spirochaetota Spirochaetia unclassified Spirochaetia Spirochaetia bacterium isolate Horse feces317 MCQ2604042.1
BacClassVII Pseudomonadati Spirochaetota unclassified Spirochaetota Spirochaetota bacterium isolate Cu122P1bin9 MCL2295151.1
BacClassVII Pseudomonadati Spirochaetota Spirochaetia Spirochaetales Spirochaetales bacterium isolate HK STAS SPIR 6 MCP5514086.1
BacClassVII Pseudomonadati Spirochaetota Spirochaetia Spirochaetales Spirochaetales bacterium isolate Tobar15.5 16.5m G27 MCF7928139.1
BacClassVII Pseudomonadati Spirochaetota Spirochaetia unclassified Spirochaetia Spirochaetia bacterium isolate SMAG U14089 HUX21689.1
BacClassVII Pseudomonadati Spirochaetota Spirochaetia unclassified Spirochaetia Spirochaetia bacterium isolate D1M01 14 MFP4562238.1
BacClassVII Pseudomonadati Spirochaetota Spirochaetia Spirochaetales Spirochaetales bacterium isolate das tool.metabat2.70 TFG64262.1
BacClassVII Pseudomonadati Spirochaetota Spirochaetia Spirochaetales Spirochaetales bacterium isolate RS 13 41 MCK4543655.1
BacClassVII Pseudomonadati Spirochaetota Spirochaetia unclassified Spirochaetia Spirochaetia bacterium isolate SUG3664 MDY3940233.1
BacClassVII Pseudomonadati Spirochaetota Spirochaetia Spirochaetales Spirochaetales bacterium isolate ZodW Metabat.38 MBN2652749.1
BacClassVII Pseudomonadati Spirochaetota Spirochaetia Spirochaetales Spirochaetales bacterium isolate Zod Metabat.752 MBN2509789.1
BacClassVII Pseudomonadati Spirochaetota Spirochaetia Spirochaetales Spirochaetales bacterium isolate Zod Metabat.156 iso2 MBN1649324.1
BacClassVII Pseudomonadati Spirochaetota Spirochaetia Spirochaetales Spirochaetales bacterium isolate Zod Metabat.1170 MBN2051727.1
BacClassVII Pseudomonadati Spirochaetota Spirochaetia Spirochaetales Spirochaetales bacterium isolate MAG2 MBI9101947.1
BacClassVII Pseudomonadati Spirochaetota Spirochaetia Spirochaetales Spirochaetales bacterium isolate Kf353 bin62 MDR1893925.1
BacClassVII Pseudomonadati Spirochaetota Spirochaetia Spirochaetales Spirochaetales bacterium isolate MAG1 MBI9108565.1
BacClassVII Pseudomonadati Spirochaetota Spirochaetia Spirochaetales Spirochaetaceae bacterium isolate JB007 MDC7125743.1
BacClassVII Pseudomonadati Spirochaetota Spirochaetia Spirochaetales Candidatus Thalassospirochaeta sargassi MDC7228182.1
BacClassVII Pseudomonadati Spirochaetota unclassified Spirochaetota Spirochaetota bacterium isolate B11 G9 RKX89947.1
BacClassVII Pseudomonadati Spirochaetota Spirochaetia unclassified Spirochaetia Spirochaetia bacterium isolate GH26 MDA3940292.1
BacClassVII Pseudomonadati Spirochaetota Spirochaetia Spirochaetales Spirochaetales bacterium isolate RS 6 49 MCK5198168.1
BacClassVII Pseudomonadati Spirochaetota Spirochaetia Spirochaetales Spirochaetales bacterium isolate D96SM.bin.2 MCF6334542.1
BacClassVII Pseudomonadati Spirochaetota Spirochaetia unclassified Spirochaetia Spirochaetia bacterium isolate Tobar15.5 16.5m G23 MCF7944006.1
BacClassVII Pseudomonadati Spirochaetota Spirochaetia unclassified Spirochaetia Spirochaetia bacterium isolate BS150m G37 MBL7006113.1
BacClassVII Pseudomonadati Spirochaetota Spirochaetia Spirochaetales Spirochaetales bacterium isolate RS 6 9 iso2 MCK5155760.1
BacClassVII Pseudomonadati Spirochaetota unclassified Spirochaetota Spirochaetota bacterium isolate Y23 30dBSe4.bin.165 MGM0431868.1
BacClassVII Pseudomonadati Spirochaetota Spirochaetia unclassified Spirochaetia Spirochaetia bacterium isolate Tobar13.5m G44 MCF7934063.1
BacClassVII Pseudomonadati Spirochaetota Spirochaetia Spirochaetales Spirochaetales bacterium isolate GH28 MDA3832766.1
BacClassVII Pseudomonadati Spirochaetota unclassified Spirochaetota Spirochaetae bacterium HGW Spirochaetae 8 PKL12080.1
BacClassVII Pseudomonadati Spirochaetota Spirochaetia Spirochaetales Sphaerochaetaceae bacterium isolate GAC5 MHC1692909.1
BacClassVII Pseudomonadati Spirochaetota Spirochaetia Spirochaetales Sphaerochaetaceae bacterium isolate SO 2017 CLC bin 106 MCK9287908.1
BacClassVII Pseudomonadati Spirochaetota Spirochaetia Spirochaetales Sphaerochaetaceae bacterium isolate STE 124 MDD3790864.1
BacClassVII Pseudomonadati Spirochaetota Spirochaetia Spirochaetales Sphaerochaetaceae bacterium isolate bin 35 MDC7245863.1
BacClassVII Pseudomonadati Spirochaetota Spirochaetia Spirochaetales Sphaerochaetaceae bacterium isolate ZodW maxbin.202 MBN2860872.1
BacClassVII Pseudomonadati Spirochaetota Spirochaetia Spirochaetales Sphaerochaetaceae bacterium isolate SO 2017 LW2 64 MFA5513546.1
BacClassVII Pseudomonadati Spirochaetota Spirochaetia Spirochaetales Sphaerochaetaceae bacterium isolate SO 2017 LW2 51 MFA5571098.1
BacClassVII Pseudomonadati Spirochaetota Spirochaetia Spirochaetales Sphaerochaetaceae bacterium isolate STF1 24 MDD4082740.1
BacClassVII Pseudomonadati Spirochaetota Spirochaetia Spirochaetales Sphaerochaetaceae bacterium isolate A30 bin.25 MGP1419884.1
BacClassVII Pseudomonadati Spirochaetota Spirochaetia Spirochaetales Sphaerochaetaceae bacterium isolate SUG3675 MDY4889005.1
BacClassVII Pseudomonadati Spirochaetota Spirochaetia Spirochaetales Sphaerochaetaceae bacterium isolate STCSWMC 77 MDD2231471.1
BacClassVII Pseudomonadati Spirochaetota Spirochaetia Spirochaetales Sphaerochaetaceae bacterium isolate RUI458 MCR4676109.1
BacClassVII Pseudomonadati Spirochaetota Spirochaetia Spirochaetales Sphaerochaetaceae bacterium isolate RUI345 MCR5760697.1
BacClassVII Pseudomonadati Spirochaetota Spirochaetia Spirochaetales Spirochaetales bacterium isolate 20 SQ MC27 MGX8685260.1
BacClassVII Pseudomonadati Spirochaetota Spirochaetia Spirochaetales Sphaerochaetaceae bacterium isolate Horse feces196 MCQ2398667.1
BacClassVII Pseudomonadati Spirochaetota Spirochaetia Spirochaetales Sphaerochaetaceae bacterium isolate MAG74 MCF0262399.1
BacClassVII Pseudomonadati Spirochaetota Spirochaetia Spirochaetales Sphaerochaetaceae bacterium isolate SUG3679 MDY4609468.1
BacClassVII Pseudomonadati Spirochaetota Spirochaetia Spirochaetales Sphaerochaetaceae bacterium isolate RUI394 MCR5731978.1
BacClassVII Pseudomonadati Spirochaetota Spirochaetia Spirochaetales Spirochaetales bacterium isolate RGIG9521 MBO5770437.1
BacClassVII Pseudomonadati Spirochaetota Spirochaetia Spirochaetales Candidatus Ornithospirochaeta stercoravium MBO8468848.1
BacClassVII Pseudomonadati Spirochaetota Spirochaetia Spirochaetales Spirochaetales bacterium isolate SUG1121 MCI7605554.1
BacClassVII Pseudomonadati Spirochaetota Spirochaetia Spirochaetales Spirochaetales bacterium isolate AS21ysBPME 183 NLZ68202.1
BacClassVII Pseudomonadati Spirochaetota Spirochaetia Spirochaetales Candidatus Ornithospirochaeta stercorigallinarum HIS14524.1
BacClassVII Pseudomonadati Spirochaetota Spirochaetia Spirochaetales Candidatus Ornithospirochaeta avicola HIV99551.1
BacClassVII Pseudomonadati Spirochaetota Spirochaetia Spirochaetales Spirochaetales bacterium isolate SUG2063 MDD6957124.1
BacClassVII Pseudomonadati Spirochaetota Spirochaetia Spirochaetales Sphaerochaeta sp. isolate AMR MDS 2221 HOQ94156.1
BacClassVII Pseudomonadati Spirochaetota Spirochaetia unclassified Spirochaetia Spirochaetia bacterium isolate UW MP SPH1 1 MCH3917119.1
BacClassVII Pseudomonadati Spirochaetota Spirochaetia Spirochaetales Pleomorphochaeta sp. isolate JB076 MGD1822085.1
BacClassVII Pseudomonadati Spirochaetota Spirochaetia Spirochaetales Sphaerochaetaceae bacterium isolate SO 2016 CLC T1 5 MFA6846136.1
BacClassVII Pseudomonadati Spirochaetota Spirochaetia Spirochaetales Sphaerochaetaceae bacterium isolate SUG341 MCI6674589.1
BacClassVII Pseudomonadati Spirochaetota Spirochaetia Spirochaetales Sphaerochaetaceae bacterium isolate SO 2016 CLC T2 19 MFA6775972.1
BacClassVII Pseudomonadati Spirochaetota Spirochaetia Spirochaetales Sphaerochaetaceae bacterium isolate RGIG3740 MBQ7613373.1

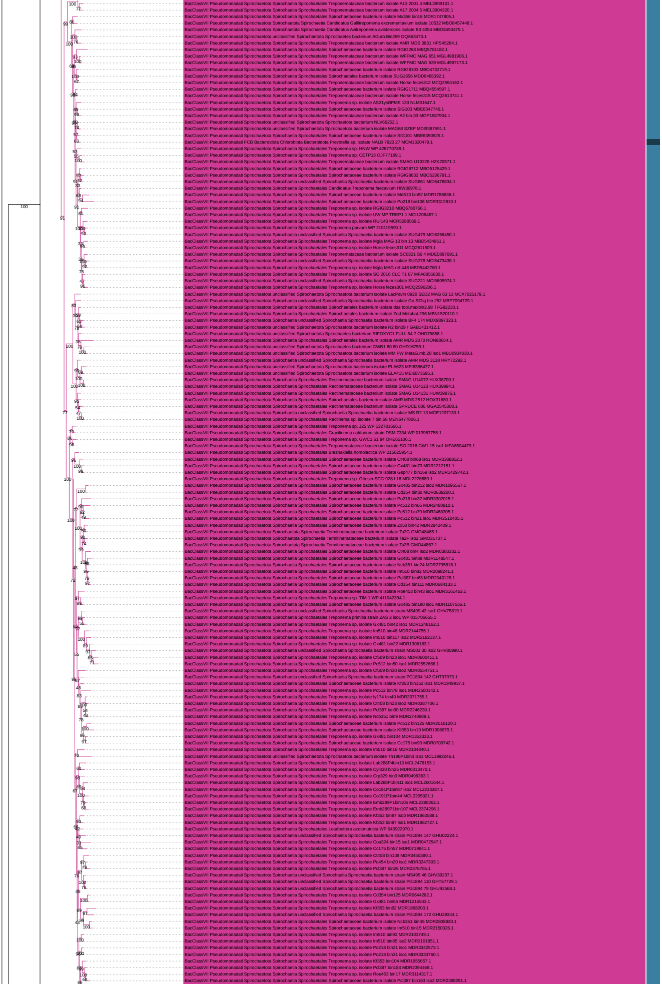
BacClassVII Pseudomonadati Spirochaetota Spirochaetia Spirochaetales Treponemataceae bacterium isolate A13 2001 4 MEL3909101.1
BacClassVII Pseudomonadati Spirochaetota Spirochaetia Spirochaetales Treponemataceae bacterium isolate A17 2004 5 MEL3904320.1
BacClassVII Pseudomonadati Spirochaetota Spirochaetia Spirochaetales Spirochaetaceae bacterium isolate Mx356 bin16 MDR1747805.1
BacClassVII Pseudomonadati Spirochaetota Spirochaetia Spirochaetota Spirochaetia Candidatus Gallitreponema excrementavium isolate 10532 MBO8457448.1
BacClassVII Pseudomonadati Spirochaetota Spirochaetia Spirochaetota Spirochaetia Candidatus Avitreponema avistercoris isolate B3 4054 MBO8450475.1
BacClassVII Pseudomonadati Spirochaetota unclassified Spirochaetota Spirochaetes bacterium ADurb.Bin269 OQA63473.1
BacClassVII Pseudomonadati Spirochaetota Spirochaetia Spirochaetales Treponemataceae bacterium isolate AMR MDS 3831 HPS45284.1
BacClassVII Pseudomonadati Spirochaetota Spirochaetia Spirochaetales Spirochaetaceae bacterium isolate RGIG268 MBQ5781182.1
BacClassVII Pseudomonadati Spirochaetota Spirochaetia Spirochaetales Treponemataceae bacterium isolate WFFMC MAG 651 MGL4981906.1
BacClassVII Pseudomonadati Spirochaetota Spirochaetia Spirochaetales Treponemataceae bacterium isolate WFFMC MAG 639 MGL4987173.1
BacClassVII Pseudomonadati Spirochaetota Spirochaetia Spirochaetales Spirochaetaceae bacterium isolate RGIG8103 MBO4732719.1
BacClassVII Pseudomonadati Spirochaetota Spirochaetia Spirochaetales Spirochaetales bacterium isolate SUG1656 MDD6485392.1
BacClassVII Pseudomonadati Spirochaetota Spirochaetia Spirochaetales Treponemataceae bacterium isolate Horse feces312 MCQ2594162.1
BacClassVII Pseudomonadati Spirochaetota Spirochaetia Spirochaetales Spirochaetaceae bacterium isolate RGIG1711 MBQ4554597.1
BacClassVII Pseudomonadati Spirochaetota Spirochaetia Spirochaetales Treponemataceae bacterium isolate Horse feces315 MCQ2613741.1
BacClassVII Pseudomonadati Spirochaetota Spirochaetia Spirochaetales Treponema sp. isolate AS21ysBPME 133 NLM01647.1
BacClassVII Pseudomonadati Spirochaetota Spirochaetia Spirochaetales Spirochaetaceae bacterium isolate SIG103 MBE6347746.1
BacClassVII Pseudomonadati Spirochaetota Spirochaetia Spirochaetales Treponemataceae bacterium isolate A2 bin.33 MGP1587904.1
BacClassVII Pseudomonadati Spirochaetota unclassified Spirochaetota Spirochaetota bacterium NLV66252.1
BacClassVII Pseudomonadati Spirochaetota unclassified Spirochaetota Spirochaetota bacterium isolate MAG68 SZBP MDI9387591.1
BacClassVII Pseudomonadati Spirochaetota Spirochaetia Spirochaetales Spirochaetaceae bacterium isolate SIG101 MBE6350525.1
BacClassVII Pseudomonadati FCB Bacteroidota Chlorobiota Bacteroidota Prevotella sp. isolate NALB 7823 27 MCM1320479.1
BacClassVII Pseudomonadati Spirochaetota Spirochaetia Spirochaetales Treponema sp. HNW WP 428770789.1
BacClassVII Pseudomonadati Spirochaetota Spirochaetia Spirochaetales Treponema sp. CETP13 OJF77189.1
BacClassVII Pseudomonadati Spirochaetota Spirochaetia Spirochaetales Treponemataceae bacterium isolate SMAG U10228 HZK20571.1
BacClassVII Pseudomonadati Spirochaetota Spirochaetia Spirochaetales Spirochaetaceae bacterium isolate RGIG8712 MBO5125429.1
BacClassVII Pseudomonadati Spirochaetota Spirochaetia Spirochaetales Spirochaetaceae bacterium isolate RGIG8632 MBO5236791.1
BacClassVII Pseudomonadati Spirochaetota Spirochaetia unclassified Spirochaetia Spirochaetia bacterium isolate SUG861 MCI6478834.1
BacClassVII Pseudomonadati Spirochaetota Spirochaetia Spirochaetales Candidatus Treponema faecavium HIW36978.1
BacClassVII Pseudomonadati Spirochaetota Spirochaetia Spirochaetales Spirochaetaceae bacterium isolate Md513 bin52 MDR1786636.1
BacClassVII Pseudomonadati Spirochaetota Spirochaetia Spirochaetales Spirochaetaceae bacterium isolate Po218 bin106 MDR3312810.1
BacClassVII Pseudomonadati Spirochaetota Spirochaetia Spirochaetales Treponema sp. isolate RGIG3210 MBQ6780766.1
BacClassVII Pseudomonadati Spirochaetota Spirochaetia Spirochaetales Treponema sp. isolate UW MP TREP1 1 MCI1208487.1
BacClassVII Pseudomonadati Spirochaetota Spirochaetia Spirochaetales Treponema sp. isolate RUI140 MCR5288568.1
BacClassVII Pseudomonadati Spirochaetota Spirochaetia Spirochaetales Treponema parvum WP 210119590.1
BacClassVII Pseudomonadati Spirochaetota Spirochaetia unclassified Spirochaetia Spirochaetia bacterium isolate SUG479 MCI6258450.1
BacClassVII Pseudomonadati Spirochaetota Spirochaetia Spirochaetales Treponema sp. isolate Mgla MAG 13 bin 13 MBD5434951.1
BacClassVII Pseudomonadati Spirochaetota Spirochaetia Spirochaetales Treponema sp. isolate Horse feces311 MCQ2611509.1
BacClassVII Pseudomonadati Spirochaetota Spirochaetia Spirochaetales Treponemataceae bacterium isolate SC0321 S6 4 MDE5897691.1
BacClassVII Pseudomonadati Spirochaetota Spirochaetia unclassified Spirochaetia Spirochaetia bacterium isolate SUG278 MCI5473438.1
BacClassVII Pseudomonadati Spirochaetota Spirochaetia Spirochaetales Treponema sp. isolate Mgla MAG ref 448 MBD5442760.1
BacClassVII Pseudomonadati Spirochaetota Spirochaetia Spirochaetales Treponema sp. isolate SO 2016 CLC T1 67 MFA6855630.1
BacClassVII Pseudomonadati Spirochaetota Spirochaetia unclassified Spirochaetia Spirochaetia bacterium isolate SUG221 MCI5605974.1
BacClassVII Pseudomonadati Spirochaetota Spirochaetia Spirochaetales Treponema sp. isolate Horse feces301 MCQ2596206.1
BacClassVII Pseudomonadati Spirochaetota unclassified Spirochaetota Spirochaetota bacterium isolate LacPavin 0920 SED2 MAG 63 13 MCX7025179.1
BacClassVII Pseudomonadati Spirochaetota Spirochaetia unclassified Spirochaetia Spirochaetia bacterium isolate Go SIDig bin 252 MBP7094729.1
BacClassVII Pseudomonadati Spirochaetota Spirochaetia Spirochaetales Spirochaetales bacterium isolate das tool.maxbin2.98 TFG82230.1
BacClassVII Pseudomonadati Spirochaetota Spirochaetia Spirochaetales Spirochaetales bacterium isolate Zod Metabat.296 MBN1520110.1
BacClassVII Pseudomonadati Spirochaetota Spirochaetia unclassified Spirochaetia Spirochaetia bacterium isolate BF4 174 MDX9897323.1
BacClassVII Pseudomonadati Spirochaetota unclassified Spirochaetota Spirochaetota bacterium isolate R2 bin29 r GAB1431412.1
BacClassVII Pseudomonadati Spirochaetota unclassified Spirochaetota Spirochaetes bacterium RIFOXYC1 FULL 54 7 OHD75858.1
BacClassVII Pseudomonadati Spirochaetota Spirochaetia Spirochaetales Spirochaetales bacterium isolate AMR MDS 2070 HON88654.1
BacClassVII Pseudomonadati Spirochaetota unclassified Spirochaetota Spirochaetes bacterium GWB1 60 80 OHD16759.1
BacClassVII Pseudomonadati Spirochaetota unclassified Spirochaetota Spirochaetota bacterium isolate MM PW MetaG.mb.28 iso1 MBU0934030.1
BacClassVII Pseudomonadati Spirochaetota Spirochaetia unclassified Spirochaetia Spirochaetia bacterium isolate AMR MDS 3138 HRY72292.1
BacClassVII Pseudomonadati Spirochaetota unclassified Spirochaetota Spirochaetota bacterium isolate ELA623 MEI6386477.1
BacClassVII Pseudomonadati Spirochaetota unclassified Spirochaetota Spirochaetota bacterium isolate ELA415 MEI6873595.1
BacClassVII Pseudomonadati Spirochaetota Spirochaetia Spirochaetales Rectinemataceae bacterium isolate SMAG U14072 HUX38700.1
BacClassVII Pseudomonadati Spirochaetota Spirochaetia Spirochaetales Rectinemataceae bacterium isolate SMAG U14123 HUX39994.1
BacClassVII Pseudomonadati Spirochaetota Spirochaetia Spirochaetales Rectinemataceae bacterium isolate SMAG U14131 HUW39876.1
BacClassVII Pseudomonadati Spirochaetota Spirochaetia Spirochaetales Spirochaetales bacterium isolate AMR MDS 2512 HOX31880.1
BacClassVII Pseudomonadati Spirochaetota Spirochaetia Spirochaetales Rectinemataceae bacterium isolate SPRUCE 606 MGA2545308.1
BacClassVII Pseudomonadati Spirochaetota Spirochaetia unclassified Spirochaetia Spirochaetia bacterium isolate MS R2 13 MCE1207130.1
BacClassVII Pseudomonadati Spirochaetota Spirochaetia Spirochaetales Rectinema sp. isolate 7 bin.68 MEN6477006.1
BacClassVII Pseudomonadati Spirochaetota Spirochaetia Spirochaetales Treponema sp. J25 WP 132781666.1
BacClassVII Pseudomonadati Spirochaetota Spirochaetia Spirochaetales Gracilinema caldarium strain DSM 7334 WP 013967755.1
BacClassVII Pseudomonadati Spirochaetota Spirochaetia Spirochaetales Treponema sp. GWC1 61 84 OHE65106.1
BacClassVII Pseudomonadati Spirochaetota Spirochaetia Spirochaetales Treponemataceae bacterium isolate SO 2016 GW1 15 iso1 MFA6504479.1
BacClassVII Pseudomonadati Spirochaetota Spirochaetia Spirochaetales Breznakiella homolactica WP 215625954.1
BacClassVII Pseudomonadati Spirochaetota Spirochaetia Spirochaetales Spirochaetaceae bacterium isolate Ct408 bin68 iso1 MDR0388852.1
BacClassVII Pseudomonadati Spirochaetota Spirochaetia Spirochaetales Spirochaetaceae bacterium isolate Gx481 bin73 MDR1212151.1
BacClassVII Pseudomonadati Spirochaetota Spirochaetia Spirochaetales Spirochaetaceae bacterium isolate Gsp477 bin169 iso2 MDR1429742.1
BacClassVII Pseudomonadati Spirochaetota Spirochaetia Spirochaetales Treponema sp. OttesenSCG 928 L16 MDL2228689.1
BacClassVII Pseudomonadati Spirochaetota Spirochaetia Spirochaetales Spirochaetaceae bacterium isolate Gx485 bin212 iso2 MDR1095567.1
BacClassVII Pseudomonadati Spirochaetota Spirochaetia Spirochaetales Spirochaetaceae bacterium isolate Cd354 bin30 MDR0638200.1
BacClassVII Pseudomonadati Spirochaetota Spirochaetia Spirochaetales Spirochaetaceae bacterium isolate Po218 bin37 MDR3302015.1
BacClassVII Pseudomonadati Spirochaetota Spirochaetia Spirochaetales Spirochaetaceae bacterium isolate Pc512 bin66 MDR2480810.1
BacClassVII Pseudomonadati Spirochaetota Spirochaetia Spirochaetales Spirochaetaceae bacterium isolate Pc512 bin79 MDR2468305.1
BacClassVII Pseudomonadati Spirochaetota Spirochaetia Spirochaetales Spirochaetaceae bacterium isolate Pc512 bin21 iso1 MDR2510405.1
BacClassVII Pseudomonadati Spirochaetota Spirochaetia Spirochaetales Spirochaetaceae bacterium isolate Zx50 bin42 MDR2842409.1
BacClassVII Pseudomonadati Spirochaetota Spirochaetia Spirochaetales Termitinemataceae bacterium isolate Ta2G GMO48465.1
BacClassVII Pseudomonadati Spirochaetota Spirochaetia Spirochaetales Termitinemataceae bacterium isolate Ta2F iso2 GMO31737.1
BacClassVII Pseudomonadati Spirochaetota Spirochaetota Spirochaetia Termitinemataceae bacterium isolate Ta2B GMO44867.1
BacClassVII Pseudomonadati Spirochaetota Spirochaetia Spirochaetales Spirochaetaceae bacterium isolate Ct408 bin4 iso2 MDR0383332.1
BacClassVII Pseudomonadati Spirochaetota Spirochaetia Spirochaetales Spirochaetaceae bacterium isolate Gx481 bin89 MDR1148547.1
BacClassVII Pseudomonadati Spirochaetota Spirochaetia Spirochaetales Spirochaetaceae bacterium isolate Ncb351 bin24 MDR2795616.1
BacClassVII Pseudomonadati Spirochaetota Spirochaetia Spirochaetales Spirochaetaceae bacterium isolate Im510 bin82 MDR2098241.1
BacClassVII Pseudomonadati Spirochaetota Spirochaetia Spirochaetales Spirochaetaceae bacterium isolate Pcl387 bin63 MDR2343128.1
BacClassVII Pseudomonadati Spirochaetota Spirochaetia Spirochaetales Spirochaetaceae bacterium isolate Cd354 bin111 MDR0684133.1
BacClassVII Pseudomonadati Spirochaetota Spirochaetia Spirochaetales Spirochaetaceae bacterium isolate Roe453 bin43 iso1 MDR3161463.1
BacClassVII Pseudomonadati Spirochaetota Spirochaetia Spirochaetales Treponema sp. TIM 1 WP 411042394.1
BacClassVII Pseudomonadati Spirochaetota Spirochaetia Spirochaetales Spirochaetaceae bacterium isolate Gx485 bin180 iso1 MDR1107556.1
BacClassVII Pseudomonadati Spirochaetota Spirochaetia unclassified Spirochaetia Spirochaetia bacterium strain MS499 42 iso1 GHV75819.1
BacClassVII Pseudomonadati Spirochaetota Spirochaetia Spirochaetales Treponema primitia strain ZAS 2 iso1 WP 015706655.1
BacClassVII Pseudomonadati Spirochaetota Spirochaetia Spirochaetales Treponema sp. isolate Gx481 bin42 iso1 MDR1248162.1
BacClassVII Pseudomonadati Spirochaetota Spirochaetia Spirochaetales Treponema sp. isolate Im510 bin48 MDR2144755.1
BacClassVII Pseudomonadati Spirochaetota Spirochaetia Spirochaetales Treponema sp. isolate Im510 bin117 iso2 MDR2182137.1
BacClassVII Pseudomonadati Spirochaetota Spirochaetia Spirochaetales Treponema sp. isolate Gx481 bin22 MDR1306183.1
BacClassVII Pseudomonadati Spirochaetota Spirochaetia unclassified Spirochaetia Spirochaetia bacterium strain MS502 30 iso2 GHV85990.1
BacClassVII Pseudomonadati Spirochaetota Spirochaetia Spirochaetales Treponema sp. isolate Cf509 bin23 iso1 MDR0600411.1
BacClassVII Pseudomonadati Spirochaetota Spirochaetia Spirochaetales Treponema sp. isolate Pc512 bin50 iso1 MDR2552668.1
BacClassVII Pseudomonadati Spirochaetota Spirochaetia Spirochaetales Treponema sp. isolate Cf509 bin30 iso2 MDR0554751.1
BacClassVII Pseudomonadati Spirochaetota Spirochaetia unclassified Spirochaetia Spirochaetia bacterium strain PG1894 142 GHT97973.1
BacClassVII Pseudomonadati Spirochaetota Spirochaetia Spirochaetales Spirochaetaceae bacterium isolate Kf353 bin152 iso1 MDR1949937.1
BacClassVII Pseudomonadati Spirochaetota Spirochaetia Spirochaetales Treponema sp. isolate Pc512 bin78 iso1 MDR2500142.1
BacClassVII Pseudomonadati Spirochaetota Spirochaetia Spirochaetales Treponema sp. isolate Iy174 bin49 MDR2071755.1
BacClassVII Pseudomonadati Spirochaetota Spirochaetia Spirochaetales Treponema sp. isolate Ct408 bin23 iso2 MDR0387706.1
BacClassVII Pseudomonadati Spirochaetota Spirochaetia Spirochaetales Treponema sp. isolate Pcl387 bin90 MDR2246230.1
BacClassVII Pseudomonadati Spirochaetota Spirochaetia Spirochaetales Treponema sp. isolate Ncb351 bin9 MDR2740888.1
BacClassVII Pseudomonadati Spirochaetota Spirochaetia Spirochaetales Spirochaetaceae bacterium isolate Pc512 bin125 MDR2516120.1
BacClassVII Pseudomonadati Spirochaetota Spirochaetia Spirochaetales Spirochaetaceae bacterium isolate Kf353 bin19 MDR1908879.1
BacClassVII Pseudomonadati Spirochaetota Spirochaetia Spirochaetales Treponema sp. isolate Gx481 bin104 MDR1353333.1
BacClassVII Pseudomonadati Spirochaetota Spirochaetia Spirochaetales Spirochaetaceae bacterium isolate Cc175 bin90 MDR0709742.1
BacClassVII Pseudomonadati Spirochaetota Spirochaetia Spirochaetales Treponema sp. isolate Im510 bin16 MDR2184840.1
BacClassVII Pseudomonadati Spirochaetota unclassified Spirochaetota Spirochaetota bacterium isolate Th196P1bin3 iso1 MCL1992046.1
BacClassVII Pseudomonadati Spirochaetota Spirochaetia Spirochaetales Treponema sp. isolate Lab288P4bin13 MCL2478153.1
BacClassVII Pseudomonadati Spirochaetota Spirochaetia Spirochaetales Treponema sp. isolate Cyl330 bin25 MDR0313470.1
BacClassVII Pseudomonadati Spirochaetota Spirochaetia Spirochaetales Treponema sp. isolate Crp329 bin3 MDR0496363.1
BacClassVII Pseudomonadati Spirochaetota Spirochaetia Spirochaetales Treponema sp. isolate Lab288P1bin11 iso1 MCL2601644.1
BacClassVII Pseudomonadati Spirochaetota Spirochaetia Spirochaetales Treponema sp. isolate Co191P1bin87 iso2 MCL2233387.1
BacClassVII Pseudomonadati Spirochaetota Spirochaetia Spirochaetales Treponema sp. isolate Co191P1bin44 MCL2205921.1
BacClassVII Pseudomonadati Spirochaetota Spirochaetia Spirochaetales Treponema sp. isolate Emb289P1bin105 MCL2380262.1
BacClassVII Pseudomonadati Spirochaetota Spirochaetia Spirochaetales Treponema sp. isolate Emb289P1bin107 MCL2374296.1
BacClassVII Pseudomonadati Spirochaetota Spirochaetia Spirochaetales Treponema sp. isolate Kf353 bin87 iso3 MDR1863588.1
BacClassVII Pseudomonadati Spirochaetota Spirochaetia Spirochaetales Treponema sp. isolate Kf353 bin87 iso1 MDR1862727.1
BacClassVII Pseudomonadati Spirochaetota Spirochaetia Spirochaetales Leadbettera azotonutricia WP 043922970.1
BacClassVII Pseudomonadati Spirochaetota Spirochaetia unclassified Spirochaetia Spirochaetia bacterium strain PG1894 147 GHU02224.1
BacClassVII Pseudomonadati Spirochaetota Spirochaetia Spirochaetales Treponema sp. isolate Coa324 bin15 iso1 MDR0472547.1
BacClassVII Pseudomonadati Spirochaetota Spirochaetia Spirochaetales Treponema sp. isolate Cc175 bin57 MDR0719841.1
BacClassVII Pseudomonadati Spirochaetota Spirochaetia Spirochaetales Treponema sp. isolate Ct408 bin138 MDR0450380.1
BacClassVII Pseudomonadati Spirochaetota Spirochaetia Spirochaetales Treponema sp. isolate Pq454 bin20 iso1 MDR3247303.1
BacClassVII Pseudomonadati Spirochaetota Spirochaetia Spirochaetales Treponema sp. isolate Pcl387 bin26 MDR2376755.1
BacClassVII Pseudomonadati Spirochaetota Spirochaetia unclassified Spirochaetia Spirochaetia bacterium strain MS495 46 GHV39237.1
BacClassVII Pseudomonadati Spirochaetota Spirochaetia unclassified Spirochaetia Spirochaetia bacterium strain PG1894 110 GHT67729.1
BacClassVII Pseudomonadati Spirochaetota Spirochaetia unclassified Spirochaetia Spirochaetia bacterium strain PG1894 79 GHU92568.1
BacClassVII Pseudomonadati Spirochaetota Spirochaetia Spirochaetales Treponema sp. isolate Cd354 bin125 MDR0644282.1
BacClassVII Pseudomonadati Spirochaetota Spirochaetia Spirochaetales Treponema sp. isolate Gx481 bin55 MDR1215543.1
BacClassVII Pseudomonadati Spirochaetota Spirochaetia Spirochaetales Treponema sp. isolate Kf353 bin92 MDR1868200.1
BacClassVII Pseudomonadati Spirochaetota Spirochaetia unclassified Spirochaetia Spirochaetia bacterium strain PG1894 172 GHU29344.1
BacClassVII Pseudomonadati Spirochaetota Spirochaetia Spirochaetales Spirochaetaceae bacterium isolate Ncb351 bin45 MDR2808830.1
BacClassVII Pseudomonadati Spirochaetota Spirochaetia Spirochaetales Spirochaetaceae bacterium isolate Im510 bin15 MDR2150326.1
BacClassVII Pseudomonadati Spirochaetota Spirochaetia Spirochaetales Treponema sp. isolate Im510 bin92 MDR2103749.1
BacClassVII Pseudomonadati Spirochaetota Spirochaetia Spirochaetales Treponema sp. isolate Im510 bin85 iso2 MDR2101851.1
BacClassVII Pseudomonadati Spirochaetota Spirochaetia Spirochaetales Treponema sp. isolate Po218 bin21 iso1 MDR3342573.1
BacClassVII Pseudomonadati Spirochaetota Spirochaetia Spirochaetales Treponema sp. isolate Po218 bin31 iso1 MDR3333760.1
BacClassVII Pseudomonadati Spirochaetota Spirochaetia Spirochaetales Spirochaetes bacterium isolate Kf353 bin104 MDR1955657.1
BacClassVII Pseudomonadati Spirochaetota Spirochaetia Spirochaetales Treponema sp. isolate Pcl387 bin184 MDR2394468.1
BacClassVII Pseudomonadati Spirochaetota Spirochaetia Spirochaetales Treponema sp. isolate Roe453 bin17 MDR3114317.1
BacClassVII Pseudomonadati Spirochaetota Spirochaetia Spirochaetales Spirochaetaceae bacterium isolate Pcl387 bin163 iso2 MDR2398291.1

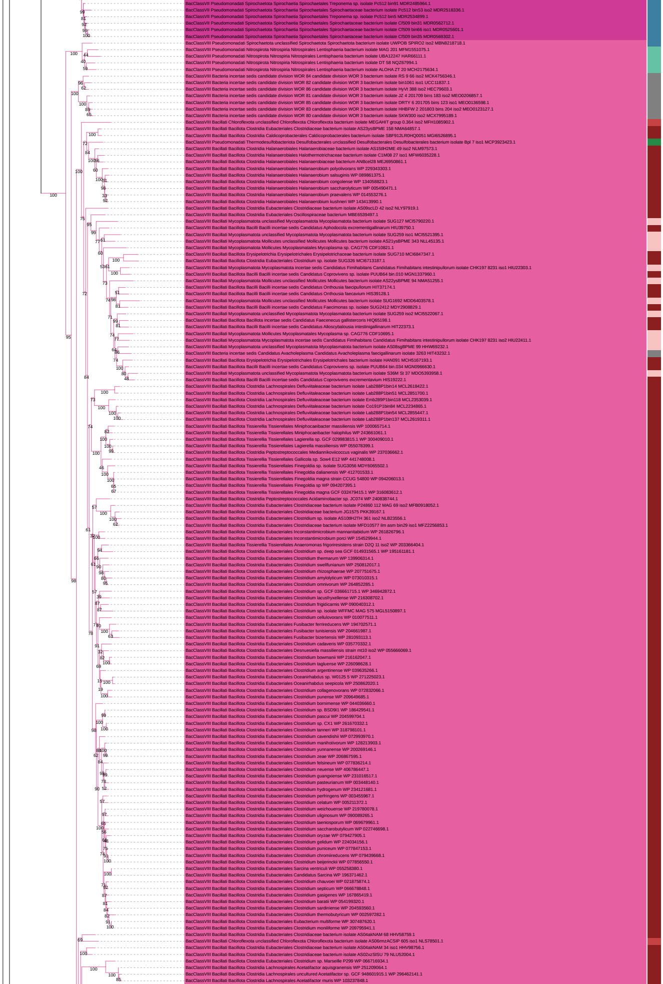
BacClassVII Pseudomonadati Spirochaetota Spirochaetia Spirochaetales Treponema sp. isolate Pc512 bin91 MDR2485964.1
BacClassVII Pseudomonadati Spirochaetota Spirochaetia Spirochaetales Spirochaetaceae bacterium isolate Pc512 bin53 iso2 MDR2518336.1
BacClassVII Pseudomonadati Spirochaetota Spirochaetia Spirochaetales Treponema sp. isolate Pc512 bin5 MDR2534899.1
BacClassVII Pseudomonadati Spirochaetota Spirochaetia Spirochaetales Spirochaetaceae bacterium isolate Cf509 bin31 MDR0562712.1
BacClassVII Pseudomonadati Spirochaetota Spirochaetia Spirochaetales Spirochaetaceae bacterium isolate Cf509 bin66 iso1 MDR0525601.1
BacClassVII Pseudomonadati Spirochaetota Spirochaetia Spirochaetales Spirochaetaceae bacterium isolate Cf509 bin35 MDR0569302.1
BacClassVIII Pseudomonadati Spirochaetota unclassified Spirochaetota Spirochaetota bacterium isolate UWPOB SPIRO2 iso2 MBN8218718.1
BacClassVIII Pseudomonadati Nitrospirota Nitrospiria Nitrospirales Lentisphaeria bacterium isolate MAG 201 MFM1551075.1
BacClassVIII Pseudomonadati Nitrospirota Nitrospiria Nitrospirales Lentisphaeria bacterium isolate UBA12247 HAR66111.1
BacClassVIII Pseudomonadati Nitrospirota Nitrospiria Nitrospirales Lentisphaeria bacterium isolate DT 58 NQZ67994.1
BacClassVIII Pseudomonadati Nitrospirota Nitrospiria Nitrospirales Lentisphaeria bacterium isolate ALOHA ZT 20 MCH2176634.1
BacClassVIII Bacteria incertae sedis candidate division WOR 84 candidate division WOR 3 bacterium isolate RS 9 66 iso2 MCK4756346.1
BacClassVIII Bacteria incertae sedis candidate division WOR 82 candidate division WOR 3 bacterium isolate bin1061 iso1 UCC11837.1
BacClassVIII Bacteria incertae sedis candidate division WOR 86 candidate division WOR 3 bacterium isolate HyVt 388 iso2 HEC79603.1
BacClassVIII Bacteria incertae sedis candidate division WOR 81 candidate division WOR 3 bacterium isolate JZ 4 201709 bins 183 iso2 MEO0206857.1
BacClassVIII Bacteria incertae sedis candidate division WOR 85 candidate division WOR 3 bacterium isolate DRTY 6 201705 bins 123 iso1 MEO0136598.1
BacClassVIII Bacteria incertae sedis candidate division WOR 83 candidate division WOR 3 bacterium isolate HHBFW 2 201803 bins 204 iso2 MEO0123127.1
BacClassVIII Bacteria incertae sedis candidate division WOR 80 candidate division WOR 3 bacterium isolate SKW300 iso2 MCX7995189.1
BacClassVIII Bacillati Chloroflexota unclassified Chloroflexota Chloroflexota bacterium isolate MEGAHIT group 0.364 iso2 MFH1085902.1
BacClassVIII Bacillati Bacillota Clostridia Eubacteriales Clostridiaceae bacterium isolate AS23ysBPME 158 NMA64857.1
BacClassVIII Bacillati Bacillota Clostridia Caldicoprobacterales Caldicoprobacteraceae bacterium isolate SBF912LR0HQ0051 MGI6526895.1
BacClassVIII Pseudomonadati Thermodesulfobacteriota Desulfobacterales unclassified Desulfobacterales Desulfobacterales bacterium isolate Bpl 7 iso1 MCP3923423.1
BacClassVIII Bacillati Bacillota Clostridia Halanaerobiales Halanaerobiaceae bacterium isolate AS15tIH2ME 49 iso2 NLM97573.1
BacClassVIII Bacillati Bacillota Clostridia Halanaerobiales Halothermotrichaceae bacterium isolate C1M08 27 iso1 MFW6035228.1
BacClassVIII Bacillati Bacillota Clostridia Halanaerobiales Halanaerobiaceae bacterium ANBcel28 MEJ6950861.1
BacClassVIII Bacillati Bacillota Clostridia Halanaerobiales Halanaerobium polyolivorans WP 229343303.1
BacClassVIII Bacillati Bacillota Clostridia Halanaerobiales Halanaerobium salsuginis WP 089861375.1
BacClassVIII Bacillati Bacillota Clostridia Halanaerobiales Halanaerobium congolense WP 134058823.1
BacClassVIII Bacillati Bacillota Clostridia Halanaerobiales Halanaerobium saccharolyticum WP 005490471.1
BacClassVIII Bacillati Bacillota Clostridia Halanaerobiales Halanaerobium praevalens WP 014553276.1
BacClassVIII Bacillati Bacillota Clostridia Halanaerobiales Halanaerobium kushneri WP 143413990.1
BacClassVIII Bacillati Bacillota Clostridia Eubacteriales Clostridiaceae bacterium isolate AS09scLD 42 iso2 NLY97919.1
BacClassVIII Bacillati Bacillota Clostridia Eubacteriales Oscillospiraceae bacterium MBE6539497.1
BacClassVIII Bacillati Mycoplasmatota unclassified Mycoplasmatota Mycoplasmatota bacterium isolate SUG127 MCI5790220.1
BacClassVIII Bacillati Bacillota Bacilli Bacilli incertae sedis Candidatus Aphodocola excrementigallinarum HIU39750.1
BacClassVIII Bacillati Mycoplasmatota unclassified Mycoplasmatota Mycoplasmatota bacterium isolate SUG259 iso1 MCI5521395.1
BacClassVIII Bacillati Mycoplasmatota Mollicutes unclassified Mollicutes Mollicutes bacterium isolate AS21ysBPME 343 NLL45135.1
BacClassVIII Bacillati Mycoplasmatota Mollicutes Mycoplasmatales Mycoplasma sp. CAG776 CDF10821.1
BacClassVIII Bacillati Bacillota Erysipelotrichia Erysipelotrichales Erysipelotrichaceae bacterium isolate SUG710 MCI6847347.1
BacClassVIII Bacillati Bacillota Clostridia Eubacteriales Clostridium sp. isolate SUG326 MCI6713187.1
BacClassVIII Bacillati Mycoplasmatota Mycoplasmatota incertae sedis Candidatus Fimihabitans Candidatus Fimihabitans intestinipullorum isolate CHK197 8231 iso1 HIU22303.1
BacClassVIII Bacillati Bacillota Bacilli Bacilli incertae sedis Candidatus Coprovivens sp. isolate PUUB64 bin.010 MGN1337960.1
BacClassVIII Bacillati Mycoplasmatota Mollicutes unclassified Mollicutes Mollicutes bacterium isolate AS22ysBPME 94 NMA51255.1
BacClassVIII Bacillati Bacillota Bacilli Bacilli incertae sedis Candidatus Onthousia faecipullorum HIT37174.1
BacClassVIII Bacillati Bacillota Bacilli Bacilli incertae sedis Candidatus Onthousia faecavium HIS39128.1
BacClassVIII Bacillati Mycoplasmatota Mollicutes unclassified Mollicutes Mollicutes bacterium isolate SUG1692 MDD6403578.1
BacClassVIII Bacillati Bacillota Bacilli Bacilli incertae sedis Candidatus Faecimonas sp. isolate SUG2412 MDY2908829.1
BacClassVIII Bacillati Mycoplasmatota unclassified Mycoplasmatota Mycoplasmatota bacterium isolate SUG259 iso2 MCI5522067.1
BacClassVIII Bacillati Bacillota Bacillota incertae sedis Candidatus Faecenecus gallistercoris HIQ65198.1
BacClassVIII Bacillati Bacillota Bacilli Bacilli incertae sedis Candidatus Alloscybalousia intestinigallinarum HIT22373.1
BacClassVIII Bacillati Mycoplasmatota Mollicutes Mycoplasmatales Mycoplasma sp. CAG776 CDF10695.1
BacClassVIII Bacillati Mycoplasmatota Mycoplasmatota incertae sedis Candidatus Fimihabitans Candidatus Fimihabitans intestinipullorum isolate CHK197 8231 iso2 HIU22411.1
BacClassVIII Bacillati Mycoplasmatota unclassified Mycoplasmatota Mycoplasmatota bacterium isolate AS08sgBPME 99 HHW69232.1
BacClassVIII Bacteria incertae sedis Candidatus Avacholeplasma Candidatus Avacholeplasma faecigallinarum isolate 3263 HIT43232.1
BacClassVIII Bacillati Bacillota Erysipelotrichia Erysipelotrichales Erysipelotrichales bacterium isolate HAN091 MCH5167193.1
BacClassVIII Bacillati Bacillota Bacilli Bacilli incertae sedis Candidatus Coprovivens sp. isolate PUUB64 bin.034 MGN0966630.1
BacClassVIII Bacillati Mycoplasmatota unclassified Mycoplasmatota Mycoplasmatota bacterium isolate S36M St 37 MDO5393958.1
BacClassVIII Bacillati Bacillota Bacilli Bacilli incertae sedis Candidatus Coprovivens excrementavium HIS19222.1
BacClassVIII Bacillati Bacillota Clostridia Lachnospirales Defluviitaleaceae bacterium isolate Lab288P1bin14 MCL2618422.1
BacClassVIII Bacillati Bacillota Clostridia Lachnospirales Defluviitaleaceae bacterium isolate Lab288P1bin51 MCL2851700.1
BacClassVIII Bacillati Bacillota Clostridia Lachnospirales Defluviitaleaceae bacterium isolate Emb289P1bin118 MCL2353039.1
BacClassVIII Bacillati Bacillota Clostridia Lachnospirales Defluviitaleaceae bacterium isolate Co191P1bin84 MCL2234865.1
BacClassVIII Bacillati Bacillota Clostridia Lachnospirales Defluviitaleaceae bacterium isolate Lab288P1bin54 MCL2855447.1
BacClassVIII Bacillati Bacillota Clostridia Lachnospirales Defluviitaleaceae bacterium isolate Lab288P1bin137 MCL2619311.1
BacClassVIII Bacillati Bacillota Tissierellia Tissierellales Miniphocaeibacter massiliensis WP 100065714.1
BacClassVIII Bacillati Bacillota Tissierellia Tissierellales Miniphocaeibacter halophilus WP 243661061.1
BacClassVIII Bacillati Bacillota Tissierellia Tissierellales Lagierella sp. GCF 029983815.1 WP 300409010.1
BacClassVIII Bacillati Bacillota Tissierellia Tissierellales Lagierella massiliensis WP 055078399.1
BacClassVIII Bacillati Bacillota Clostridia Peptostreptococcales Medianmikoviicoccus vaginalis WP 237036662.1
BacClassVIII Bacillati Bacillota Tissierellia Tissierellales Gallicola sp. Sow4 E12 WP 441748008.1
BacClassVIII Bacillati Bacillota Tissierellia Tissierellales Finegoldia sp. isolate SUG3056 MDY6065502.1
BacClassVIII Bacillati Bacillota Tissierellia Tissierellales Finegoldia dalianensis WP 412701533.1
BacClassVIII Bacillati Bacillota Tissierellia Tissierellales Finegoldia magna strain CCUG 54800 WP 094206013.1
BacClassVIII Bacillati Bacillota Tissierellia Tissierellales Finegoldia sp WP 094207395.1
BacClassVIII Bacillati Bacillota Tissierellia Tissierellales Finegoldia magna GCF 032479415.1 WP 316083612.1
BacClassVIII Bacillati Bacillota Clostridia Peptostreptococcales Acidaminobacter sp. JC074 WP 240838744.1
BacClassVIII Bacillati Bacillota Clostridia Eubacteriales Clostridiaceae bacterium isolate P24860 112 MAG 69 iso2 MFB0918052.1
BacClassVIII Bacillati Bacillota Clostridia Eubacteriales Clostridiaceae bacterium JG1575 PKK39167.1
BacClassVIII Bacillati Bacillota Clostridia Eubacteriales Clostridium sp. isolate AS10tIH2TH 361 iso2 NLB23556.1
BacClassVIII Bacillati Bacillota Clostridia Eubacteriales Clostridiaceae bacterium isolate MFD10577 ilm asm bin29 iso1 MFZ2256853.1
BacClassVIII Bacillati Bacillota Clostridia Eubacteriales Inconstantimicrobium mannanitabidum WP 261826796.1
BacClassVIII Bacillati Bacillota Clostridia Eubacteriales Inconstantimicrobium porci WP 154529944.1
BacClassVIII Bacillati Bacillota Tissierellia Tissierellales Anaeromonas frigoriresistens strain D2Q 11 iso2 WP 203366404.1
BacClassVIII Bacillati Bacillota Clostridia Eubacteriales Clostridium sp. deep sea GCF 014931565.1 WP 195161181.1
BacClassVIII Bacillati Bacillota Clostridia Eubacteriales Clostridium thermarum WP 139906314.1
BacClassVIII Bacillati Bacillota Clostridia Eubacteriales Clostridium swellfunianum WP 250812017.1
BacClassVIII Bacillati Bacillota Clostridia Eubacteriales Clostridium rhizosphaerae WP 207751675.1
BacClassVIII Bacillati Bacillota Clostridia Eubacteriales Clostridium amylolyticum WP 073010315.1
BacClassVIII Bacillati Bacillota Clostridia Eubacteriales Clostridium omnivorum WP 264852285.1
BacClassVIII Bacillati Bacillota Clostridia Eubacteriales Clostridium sp. GCF 036661715.1 WP 346942872.1
BacClassVIII Bacillati Bacillota Clostridia Eubacteriales Clostridium lacusfryxellense WP 216308702.1
BacClassVIII Bacillati Bacillota Clostridia Eubacteriales Clostridium frigidicarnis WP 090040312.1
BacClassVIII Bacillati Bacillota Clostridia Eubacteriales Clostridium sp. isolate WFFMC MAG 575 MGL5150897.1
BacClassVIII Bacillati Bacillota Clostridia Eubacteriales Clostridium cellulovorans WP 010077511.1
BacClassVIII Bacillati Bacillota Clostridia Eubacteriales Fusibacter ferrireducens WP 194702571.1
BacClassVIII Bacillati Bacillota Clostridia Eubacteriales Fusibacter tunisiensis WP 204661987.1
BacClassVIII Bacillati Bacillota Clostridia Eubacteriales Fusibacter bizertensis WP 281093113.1
BacClassVIII Bacillati Bacillota Clostridia Eubacteriales Clostridium cadaveris WP 035770332.1
BacClassVIII Bacillati Bacillota Clostridia Eubacteriales Desnuesiella massiliensis strain mt10 iso2 WP 055666069.1
BacClassVIII Bacillati Bacillota Clostridia Eubacteriales Clostridium bowmanii WP 216162047.1
BacClassVIII Bacillati Bacillota Clostridia Eubacteriales Clostridium tagluense WP 226098628.1
BacClassVIII Bacillati Bacillota Clostridia Eubacteriales Clostridium argentinense WP 039635266.1
BacClassVIII Bacillati Bacillota Clostridia Eubacteriales Oceanirhabdus sp. W0125 5 WP 271225023.1
BacClassVIII Bacillati Bacillota Clostridia Eubacteriales Oceanirhabdus seepicola WP 250862020.1
BacClassVIII Bacillati Bacillota Clostridia Eubacteriales Clostridium collagenovorans WP 072832066.1
BacClassVIII Bacillati Bacillota Clostridia Eubacteriales Clostridium punense WP 209649685.1
BacClassVIII Bacillati Bacillota Clostridia Eubacteriales Clostridium bornimense WP 044036660.1
BacClassVIII Bacillati Bacillota Clostridia Eubacteriales Clostridium sp. BSD9I1 WP 186429541.1
BacClassVIII Bacillati Bacillota Clostridia Eubacteriales Clostridium pascui WP 204599704.1
BacClassVIII Bacillati Bacillota Clostridia Eubacteriales Clostridium sp. CX1 WP 216670332.1
BacClassVIII Bacillati Bacillota Clostridia Eubacteriales Clostridium tanneri WP 318798101.1
BacClassVIII Bacillati Bacillota Clostridia Eubacteriales Clostridium cavendishii WP 072993970.1
BacClassVIII Bacillati Bacillota Clostridia Eubacteriales Clostridium manihotivorum WP 128213903.1
BacClassVIII Bacillati Bacillota Clostridia Eubacteriales Clostridium yunnanense WP 200269146.1
BacClassVIII Bacillati Bacillota Clostridia Eubacteriales Clostridium zeae WP 206867595.1
BacClassVIII Bacillati Bacillota Clostridia Eubacteriales Clostridium felsineum WP 077836214.1
BacClassVIII Bacillati Bacillota Clostridia Eubacteriales Clostridium neuense WP 406786447.1
BacClassVIII Bacillati Bacillota Clostridia Eubacteriales Clostridium guangxiense WP 231016517.1
BacClassVIII Bacillati Bacillota Clostridia Eubacteriales Clostridium pasteurianum WP 003448140.1
BacClassVIII Bacillati Bacillota Clostridia Eubacteriales Clostridium hydrogenum WP 234121681.1
BacClassVIII Bacillati Bacillota Clostridia Eubacteriales Clostridium perfringens WP 003455967.1
BacClassVIII Bacillati Bacillota Clostridia Eubacteriales Clostridium celatum WP 005211372.1
BacClassVIII Bacillati Bacillota Clostridia Eubacteriales Clostridium weizhouense WP 219780078.1
BacClassVIII Bacillati Bacillota Clostridia Eubacteriales Clostridium uliginosum WP 090089265.1
BacClassVIII Bacillati Bacillota Clostridia Eubacteriales Clostridium taeniosporum WP 069679961.1
BacClassVIII Bacillati Bacillota Clostridia Eubacteriales Clostridium saccharobutylicum WP 022746698.1
BacClassVIII Bacillati Bacillota Clostridia Eubacteriales Clostridium oryzae WP 079427905.1
BacClassVIII Bacillati Bacillota Clostridia Eubacteriales Clostridium gelidum WP 224034156.1
BacClassVIII Bacillati Bacillota Clostridia Eubacteriales Clostridium puniceum WP 077847153.1
BacClassVIII Bacillati Bacillota Clostridia Eubacteriales Clostridium chromiireducens WP 079439668.1
BacClassVIII Bacillati Bacillota Clostridia Eubacteriales Clostridium beijerinckii WP 077856550.1
BacClassVIII Bacillati Bacillota Clostridia Eubacteriales Sarcina ventriculi WP 055258380.1
BacClassVIII Bacillati Bacillota Clostridia Eubacteriales Candidatus Sarcina WP 196371462.1
BacClassVIII Bacillati Bacillota Clostridia Eubacteriales Clostridium chauvoei WP 021875874.1
BacClassVIII Bacillati Bacillota Clostridia Eubacteriales Clostridium septicum WP 066678848.1
BacClassVIII Bacillati Bacillota Clostridia Eubacteriales Clostridium gasigenes WP 167865419.1
BacClassVIII Bacillati Bacillota Clostridia Eubacteriales Clostridium baratii WP 054199320.1
BacClassVIII Bacillati Bacillota Clostridia Eubacteriales Clostridium sardiniense WP 204593560.1
BacClassVIII Bacillati Bacillota Clostridia Eubacteriales Clostridium thermobutyricum WP 002597282.1
BacClassVIII Bacillati Bacillota Clostridia Eubacteriales Eubacterium multiforme WP 307487620.1
BacClassVIII Bacillati Bacillota Clostridia Eubacteriales Clostridium moniliforme WP 209795941.1
BacClassVIII Bacillati Bacillota Clostridia Eubacteriales Clostridiaceae bacterium isolate AS04akNAM 68 HHV58759.1
BacClassVIII Bacillati Chloroflexota unclassified Chloroflexota Chloroflexota bacterium isolate AS06rmzACSIP 605 iso1 NLS78501.1
BacClassVIII Bacillati Bacillota Clostridia Eubacteriales Clostridiaceae bacterium isolate AS04akNAM 34 iso1 HHV98756.1
BacClassVIII Bacillati Bacillota Clostridia Eubacteriales Clostridiaceae bacterium isolate AS02xzSISU 79 NLU52004.1
BacClassVIII Bacillati Bacillota Clostridia Eubacteriales Clostridium sp. Marseille P299 WP 066716934.1
BacClassVIII Bacillati Bacillota Clostridia Lachnospirales Acetatifactor aquisgranensis WP 251209064.1
BacClassVIII Bacillati Bacillota Clostridia Lachnospirales uncultured Acetatifactor sp. GCF 948601915.1 WP 296462141.1
BacClassVIII Bacillati Bacillota Clostridia Lachnospirales Acetatifactor muris WP 103237848.1

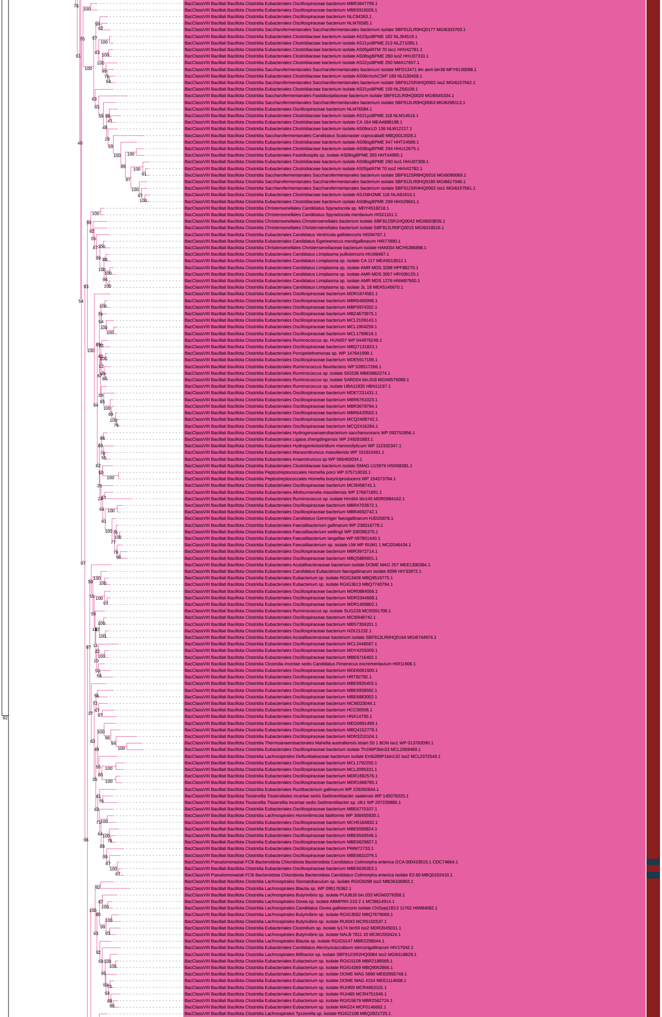

BacClassVIII Bacillati Bacillota Clostridia Eubacteriales Oscillospiraceae bacterium MBR3847799.1
BacClassVIII Bacillati Bacillota Clostridia Eubacteriales Oscillospiraceae bacterium MBE6916026.1
BacClassVIII Bacillati Bacillota Clostridia Eubacteriales Oscillospiraceae bacterium NLC84363.1
BacClassVIII Bacillati Bacillota Clostridia Eubacteriales Oscillospiraceae bacterium NLM76585.1
BacClassVIII Bacillati Bacillota Clostridia Saccharofermentanales Saccharofermentanales bacterium isolate SBF912LR0HQ0177 MGI6333703.1
BacClassVIII Bacillati Bacillota Clostridia Eubacteriales Clostridiaceae bacterium isolate AS23ysBPME 182 NLJ94519.1
BacClassVIII Bacillati Bacillota Clostridia Eubacteriales Clostridiaceae bacterium isolate AS21ysBPME 213 NLZ71085.1
BacClassVIII Bacillati Bacillota Clostridia Eubacteriales Clostridiaceae bacterium isolate AS05jafATM 70 iso1 HHV42781.1
BacClassVIII Bacillati Bacillota Clostridia Eubacteriales Clostridiaceae bacterium isolate AS08sgBPME 280 iso2 HHU07310.1
BacClassVIII Bacillati Bacillota Clostridia Eubacteriales Clostridiaceae bacterium isolate AS22ysBPME 250 NMA17657.1
BacClassVIII Bacillati Bacillota Clostridia Saccharofermentanales Saccharofermentanales bacterium isolate MFD13471 ilm asm bin38 MFY9130098.1
BacClassVIII Bacillati Bacillota Clostridia Eubacteriales Clostridiaceae bacterium isolate AS06rmzACSIP 189 NLG30458.1
BacClassVIII Bacillati Bacillota Clostridia Saccharofermentanales Saccharofermentanales bacterium isolate SBF912SR4HQ0002 iso2 MGI6157562.1
BacClassVIII Bacillati Bacillota Clostridia Eubacteriales Clostridiaceae bacterium isolate AS21ysBPME 159 NLZ56109.1
BacClassVIII Bacillati Bacillota Clostridia Saccharofermentanales Fastidiosipilaceae bacterium isolate SBF912LR0HQ0020 MGI6545334.1
BacClassVIII Bacillati Bacillota Clostridia Saccharofermentanales Saccharofermentanales bacterium isolate SBF912LR0HQ0063 MGI6298113.1
BacClassVIII Bacillati Bacillota Clostridia Eubacteriales Oscillospiraceae bacterium NLM76584.1
BacClassVIII Bacillati Bacillota Clostridia Eubacteriales Clostridiaceae bacterium isolate AS21ysBPME 118 NLM14516.1
BacClassVIII Bacillati Bacillota Clostridia Eubacteriales Clostridiaceae bacterium isolate CA 164 MEA4888198.1
BacClassVIII Bacillati Bacillota Clostridia Eubacteriales Clostridiaceae bacterium isolate AS09scLD 136 NLW12217.1
BacClassVIII Bacillati Bacillota Clostridia Saccharofermentanales Candidatus Scatonaster coprocaballi MBQ0012028.1
BacClassVIII Bacillati Bacillota Clostridia Eubacteriales Clostridiaceae bacterium isolate AS08sgBPME 347 HHT24589.1
BacClassVIII Bacillati Bacillota Clostridia Eubacteriales Clostridiaceae bacterium isolate AS08sgBPME 294 HHU12675.1
BacClassVIII Bacillati Bacillota Clostridia Eubacteriales Fastidiosipila sp. isolate AS08sgBPME 393 HHT44995.1
BacClassVIII Bacillati Bacillota Clostridia Eubacteriales Clostridiaceae bacterium isolate AS08sgBPME 280 iso1 HHU07309.1
BacClassVIII Bacillati Bacillota Clostridia Eubacteriales Clostridiaceae bacterium isolate AS05jafATM 70 iso2 HHV42782.1
BacClassVIII Bacillati Bacillota Clostridia Saccharofermentanales Saccharofermentanales bacterium isolate SBF912SR6HQ0016 MGI6090069.1
BacClassVIII Bacillati Bacillota Clostridia Saccharofermentanales Saccharofermentanales bacterium isolate SBF912LR0HQ0190 MGI6617046.1
BacClassVIII Bacillati Bacillota Clostridia Saccharofermentanales Saccharofermentanales bacterium isolate SBF912SR4HQ0002 iso1 MGI6157561.1
BacClassVIII Bacillati Bacillota Clostridia Eubacteriales Clostridiaceae bacterium isolate AS15tlH2ME 116 NLA81810.1
BacClassVIII Bacillati Bacillota Clostridia Eubacteriales Clostridiaceae bacterium isolate AS08sgBPME 299 HHX29641.1
BacClassVIII Bacillati Bacillota Clostridia Christensenellales Candidatus Spyradocola sp. MDY4518218.1
BacClassVIII Bacillati Bacillota Clostridia Christensenellales Candidatus Spyradocola merdavium HIS21101.1
BacClassVIII Bacillati Bacillota Clostridia Christensenellales Christensenellales bacterium isolate SBF912SR1HQ0042 MGI6003835.1
BacClassVIII Bacillati Bacillota Clostridia Christensenellales Christensenellales bacterium isolate SBF912LR0FQ0015 MGI6316016.1
BacClassVIII Bacillati Bacillota Clostridia Eubacteriales Candidatus Ventricola gallistercoris HIS94767.1
BacClassVIII Bacillati Bacillota Clostridia Eubacteriales Candidatus Egerieenecus merdigallinarum HIR77890.1
BacClassVIII Bacillati Bacillota Clostridia Christensenellales Christensenellaceae bacterium isolate HAN034 MCH5286898.1
BacClassVIII Bacillati Bacillota Clostridia Eubacteriales Candidatus Limiplasma pullistercoris HIU08487.1
BacClassVIII Bacillati Bacillota Clostridia Eubacteriales Candidatus Limiplasma sp. isolate CA 117 MEA5013012.1
BacClassVIII Bacillati Bacillota Clostridia Eubacteriales Candidatus Limiplasma sp. isolate AMR MDS 3298 HPF88270.1
BacClassVIII Bacillati Bacillota Clostridia Eubacteriales Candidatus Limiplasma sp. isolate AMR MDS 3057 HRX09120.1
BacClassVIII Bacillati Bacillota Clostridia Eubacteriales Candidatus Limiplasma sp. isolate AMR MDS 1276 HNW87500.1
BacClassVIII Bacillati Bacillota Clostridia Eubacteriales Candidatus Limiplasma sp. isolate 3L 18 MEA5145670.1
BacClassVIII Bacillati Bacillota Clostridia Eubacteriales Oscillospiraceae bacterium MDR1674581.1
BacClassVIII Bacillati Bacillota Clostridia Eubacteriales Oscillospiraceae bacterium MBR0485996.1
BacClassVIII Bacillati Bacillota Clostridia Eubacteriales Oscillospiraceae bacterium MBP0974332.1
BacClassVIII Bacillati Bacillota Clostridia Eubacteriales Oscillospiraceae bacterium MBZ4670975.1
BacClassVIII Bacillati Bacillota Clostridia Eubacteriales Oscillospiraceae bacterium MCL2109143.1
BacClassVIII Bacillati Bacillota Clostridia Eubacteriales Oscillospiraceae bacterium MCL1904259.1
BacClassVIII Bacillati Bacillota Clostridia Eubacteriales Oscillospiraceae bacterium MCL1789616.1
BacClassVIII Bacillati Bacillota Clostridia Eubacteriales Ruminococcus sp. HUN007 WP 044976248.1
BacClassVIII Bacillati Bacillota Clostridia Eubacteriales Oscillospiraceae bacterium MBQ7131823.1
BacClassVIII Bacillati Bacillota Clostridia Eubacteriales Porcipelethomonas sp. WP 147641999.1
BacClassVIII Bacillati Bacillota Clostridia Eubacteriales Oscillospiraceae bacterium MDE5917189.1
BacClassVIII Bacillati Bacillota Clostridia Eubacteriales Ruminococcus flavefaciens WP 028517266.1
BacClassVIII Bacillati Bacillota Clostridia Eubacteriales Ruminococcus sp. isolate SIG536 MBE6862274.1
BacClassVIII Bacillati Bacillota Clostridia Eubacteriales Ruminococcus sp. isolate SARD04 bin.018 MGN0575089.1
BacClassVIII Bacillati Bacillota Clostridia Eubacteriales Ruminococcus sp. isolate UBA11835 HBN11197.1
BacClassVIII Bacillati Bacillota Clostridia Eubacteriales Oscillospiraceae bacterium MDE7231431.1
BacClassVIII Bacillati Bacillota Clostridia Eubacteriales Oscillospiraceae bacterium MBR6761023.1
BacClassVIII Bacillati Bacillota Clostridia Eubacteriales Oscillospiraceae bacterium MBR3678794.1
BacClassVIII Bacillati Bacillota Clostridia Eubacteriales Oscillospiraceae bacterium MBR6420502.1
BacClassVIII Bacillati Bacillota Clostridia Eubacteriales Oscillospiraceae bacterium MCQ2408742.1
BacClassVIII Bacillati Bacillota Clostridia Eubacteriales Oscillospiraceae bacterium MCQ2416284.1
BacClassVIII Bacillati Bacillota Clostridia Eubacteriales Hydrogenoanaerobacterium saccharovorans WP 092752656.1
BacClassVIII Bacillati Bacillota Clostridia Eubacteriales Ligaoa zhengdingensis WP 249281883.1
BacClassVIII Bacillati Bacillota Clostridia Eubacteriales Hydrogeniiclostridium mannosilyticum WP 112332347.1
BacClassVIII Bacillati Bacillota Clostridia Eubacteriales Marasmitruncus massiliensis WP 101910461.1
BacClassVIII Bacillati Bacillota Clostridia Eubacteriales Anaerotruncus sp WP 066460034.1
BacClassVIII Bacillati Bacillota Clostridia Eubacteriales Clostridiaceae bacterium isolate SMAG U15976 HSN58381.1
BacClassVIII Bacillati Bacillota Clostridia Peptostreptococcales Hornefia porci WP 075713033.1
BacClassVIII Bacillati Bacillota Clostridia Peptostreptococcales Hornefia butyriciproducens WP 154573764.1
BacClassVIII Bacillati Bacillota Clostridia Eubacteriales Oscillospiraceae bacterium MCI9458741.1
BacClassVIII Bacillati Bacillota Clostridia Eubacteriales Allofournierella massiliensis WP 276871691.1
BacClassVIII Bacillati Bacillota Clostridia Eubacteriales Ruminococcus sp. isolate Hm464 bin145 MDR0984162.1
BacClassVIII Bacillati Bacillota Clostridia Eubacteriales Oscillospiraceae bacterium MBR4702672.1
BacClassVIII Bacillati Bacillota Clostridia Eubacteriales Oscillospiraceae bacterium MBR4692742.1
BacClassVIII Bacillati Bacillota Clostridia Eubacteriales Candidatus Gemmiger faecigallinarum HJD20876.1
BacClassVIII Bacillati Bacillota Clostridia Eubacteriales Faecalibacterium gallinarum WP 238316778.1
BacClassVIII Bacillati Bacillota Clostridia Eubacteriales Faecalibacterium wellingii WP 339395375.1
BacClassVIII Bacillati Bacillota Clostridia Eubacteriales Faecalibacterium langellae WP 097801440.1
BacClassVIII Bacillati Bacillota Clostridia Eubacteriales Faecalibacterium sp. isolate UW MP RUM1 1 MCI2046434.1
BacClassVIII Bacillati Bacillota Clostridia Eubacteriales Oscillospiraceae bacterium MBR3972714.1
BacClassVIII Bacillati Bacillota Clostridia Eubacteriales Oscillospiraceae bacterium MBQ5865601.1
BacClassVIII Bacillati Bacillota Clostridia Eubacteriales Acutalibacteraceae bacterium isolate DOME MAG 257 MEE1300384.1
BacClassVIII Bacillati Bacillota Clostridia Eubacteriales Candidatus Eubacterium faecigallinarum isolate 8396 HIY33972.1
BacClassVIII Bacillati Bacillota Clostridia Eubacteriales Eubacterium sp. isolate RGIG3408 MBQ9516775.1
BacClassVIII Bacillati Bacillota Clostridia Eubacteriales Eubacterium sp. isolate RGIG3613 MBQ7740794.1
BacClassVIII Bacillati Bacillota Clostridia Eubacteriales Oscillospiraceae bacterium MDR0884056.1
BacClassVIII Bacillati Bacillota Clostridia Eubacteriales Oscillospiraceae bacterium MDR3344608.1
BacClassVIII Bacillati Bacillota Clostridia Eubacteriales Oscillospiraceae bacterium MDR1409802.1
BacClassVIII Bacillati Bacillota Clostridia Eubacteriales Ruminococcus sp. isolate SUG228 MCI5591708.1
BacClassVIII Bacillati Bacillota Clostridia Eubacteriales Oscillospiraceae bacterium MCI5948742.1
BacClassVIII Bacillati Bacillota Clostridia Eubacteriales Oscillospiraceae bacterium MBS7359201.1
BacClassVIII Bacillati Bacillota Clostridia Eubacteriales Oscillospiraceae bacterium HZK21232.1
BacClassVIII Bacillati Bacillota Clostridia Eubacteriales Acutalibacteraceae bacterium isolate SBF912LR0HQ0164 MGI6744974.1
BacClassVIII Bacillati Bacillota Clostridia Eubacteriales Oscillospiraceae bacterium MCL2446587.1
BacClassVIII Bacillati Bacillota Clostridia Eubacteriales Oscillospiraceae bacterium MDY4255009.1
BacClassVIII Bacillati Bacillota Clostridia Eubacteriales Oscillospiraceae bacterium MBE6716402.1
BacClassVIII Bacillati Bacillota Clostridia Clostridia incertae sedis Candidatus Fimenecus excrementavium HIR11906.1
BacClassVIII Bacillati Bacillota Clostridia Eubacteriales Oscillospiraceae bacterium MDD6061500.1
BacClassVIII Bacillati Bacillota Clostridia Eubacteriales Oscillospiraceae bacterium HRT82782.1
BacClassVIII Bacillati Bacillota Clostridia Eubacteriales Oscillospiraceae bacterium MBE6935403.1
BacClassVIII Bacillati Bacillota Clostridia Eubacteriales Oscillospiraceae bacterium MBE6938592.1
BacClassVIII Bacillati Bacillota Clostridia Eubacteriales Oscillospiraceae bacterium MBE6883002.1
BacClassVIII Bacillati Bacillota Clostridia Eubacteriales Oscillospiraceae bacterium MCI6023044.1
BacClassVIII Bacillati Bacillota Clostridia Eubacteriales Oscillospiraceae bacterium HCC00506.1
BacClassVIII Bacillati Bacillota Clostridia Eubacteriales Oscillospiraceae bacterium HNX14750.1
BacClassVIII Bacillati Bacillota Clostridia Eubacteriales Oscillospiraceae bacterium MEG0691499.1
BacClassVIII Bacillati Bacillota Clostridia Eubacteriales Oscillospiraceae bacterium MBQ4152779.1
BacClassVIII Bacillati Bacillota Clostridia Eubacteriales Oscillospiraceae bacterium MDR3210104.1
BacClassVIII Bacillati Bacillota Clostridia Thermoanaerobacterales Mahella australiensis strain 50 1 BON iso1 WP 013782090.1
BacClassVIII Bacillati Bacillota Clostridia Eubacteriales Oscillospiraceae bacterium isolate Th196P3bin33 MCL2059469.1
BacClassVIII Bacillati Bacillota Clostridia Lachnospirales Defluviitaleaceae bacterium isolate Emb289P1bin132 iso2 MCL2372543.1
BacClassVIII Bacillati Bacillota Clostridia Eubacteriales Oscillospiraceae bacterium MCL1792250.1
BacClassVIII Bacillati Bacillota Clostridia Eubacteriales Oscillospiraceae bacterium MCL2095331.1
BacClassVIII Bacillati Bacillota Clostridia Eubacteriales Oscillospiraceae bacterium MDR1692576.1
BacClassVIII Bacillati Bacillota Clostridia Eubacteriales Oscillospiraceae bacterium MDR1668785.1
BacClassVIII Bacillati Bacillota Clostridia Eubacteriales Ructibacterium gallinarum WP 226392644.1
BacClassVIII Bacillati Bacillota Tissierellia Tissierellales incertae sedis Sedimentibacter saalensis WP 145079325.1
BacClassVIII Bacillati Bacillota Tissierellia Tissierellales incertae sedis Sedimentibacter sp. zth1 WP 207235885.1
BacClassVIII Bacillati Bacillota Clostridia Eubacteriales Oscillospiraceae bacterium MBE6770107.1
BacClassVIII Bacillati Bacillota Clostridia Lachnospirales Hominilimicola fabiformis WP 308455920.1
BacClassVIII Bacillati Bacillota Clostridia Eubacteriales Oscillospiraceae bacterium MCH5184832.1
BacClassVIII Bacillati Bacillota Clostridia Eubacteriales Oscillospiraceae bacterium MBE6599824.1
BacClassVIII Bacillati Bacillota Clostridia Eubacteriales Oscillospiraceae bacterium MBE6549546.1
BacClassVIII Bacillati Bacillota Clostridia Eubacteriales Oscillospiraceae bacterium MBE6629827.1
BacClassVIII Bacillati Bacillota Clostridia Eubacteriales Oscillospiraceae bacterium PWM72733.1
BacClassVIII Bacillati Bacillota Clostridia Eubacteriales Oscillospiraceae bacterium MBE6631079.1
BacClassVIII Pseudomonadati FCB Bacteroidota Chlorobiota Bacteroidota Candidatus Colimorpha enterica GCA 000433515.1 CDC74664.1
BacClassVIII Bacillati Bacillota Clostridia Eubacteriales Oscillospiraceae bacterium MBE6636353.1
BacClassVIII Pseudomonadati FCB Bacteroidota Chlorobiota Bacteroidota Candidatus Colimorpha enterica isolate E3 60 MBQ0102410.1
BacClassVIII Bacillati Bacillota Clostridia Lachnospirales Stomatobaculum sp. isolate RGIG9298 iso2 MBO6106902.1
BacClassVIII Bacillati Bacillota Clostridia Lachnospirales Blautia sp. WP 095176362.1
BacClassVIII Bacillati Bacillota Clostridia Lachnospirales Butyrivibrio sp. isolate PUUB16 bin.033 MGN0379358.1
BacClassVIII Bacillati Bacillota Clostridia Lachnospirales Dorea sp. isolate ABMPRH 210 2 1 MCI9614914.1
BacClassVIII Bacillati Bacillota Clostridia Lachnospirales Candidatus Dorea gallistercoris isolate ChiSxjej1B13 11762 HIW84082.1
BacClassVIII Bacillati Bacillota Clostridia Lachnospirales Butyrivibrio sp. isolate RGIG3582 MBQ7679069.1
BacClassVIII Bacillati Bacillota Clostridia Lachnospirales Butyrivibrio sp. isolate RUI043 MCR5102537.1
BacClassVIII Bacillati Bacillota Clostridia Eubacteriales Clostridium sp. isolate ly174 bin59 iso2 MDR2045031.1
BacClassVIII Bacillati Bacillota Clostridia Lachnospirales Butyrivibrio sp. isolate NALB 7811 10 MCM1553424.1
BacClassVIII Bacillati Bacillota Clostridia Lachnospirales Blautia sp. isolate RGIG5147 MBR2258044.1
BacClassVIII Bacillati Bacillota Clostridia Eubacteriales Candidatus Alectryocaccobium stercorigallinarum HIV17042.1
BacClassVIII Bacillati Bacillota Clostridia Lachnospirales Bilifractor sp. isolate SBF912SR2HQ0084 iso2 MGI6118829.1
BacClassVIII Bacillati Bacillota Clostridia Eubacteriales Eubacterium sp. isolate RGIG5108 MBR2188569.1
BacClassVIII Bacillati Bacillota Clostridia Eubacteriales Eubacterium sp. isolate RGIG4369 MBQ9062866.1
BacClassVIII Bacillati Bacillota Clostridia Eubacteriales Eubacterium sp. isolate DOME MAG 5690 MEE0955748.1
BacClassVIII Bacillati Bacillota Clostridia Eubacteriales Eubacterium sp. isolate DOME MAG 4154 MEE1114508.1
BacClassVIII Bacillati Bacillota Clostridia Eubacteriales Eubacterium sp. isolate RUI459 MCR4653101.1
BacClassVIII Bacillati Bacillota Clostridia Eubacteriales Eubacterium sp. isolate RUI485 MCR4751945.1
BacClassVIII Bacillati Bacillota Clostridia Eubacteriales Eubacterium sp. isolate RGIG5679 MBR2562724.1
BacClassVIII Bacillati Bacillota Clostridia Eubacteriales Eubacterium sp. isolate MAG24 MCF0146662.1
BacClassVIII Bacillati Bacillota Clostridia Lachnospirales Tyzzerella sp. isolate RGIG2108 MBQ2921725.1

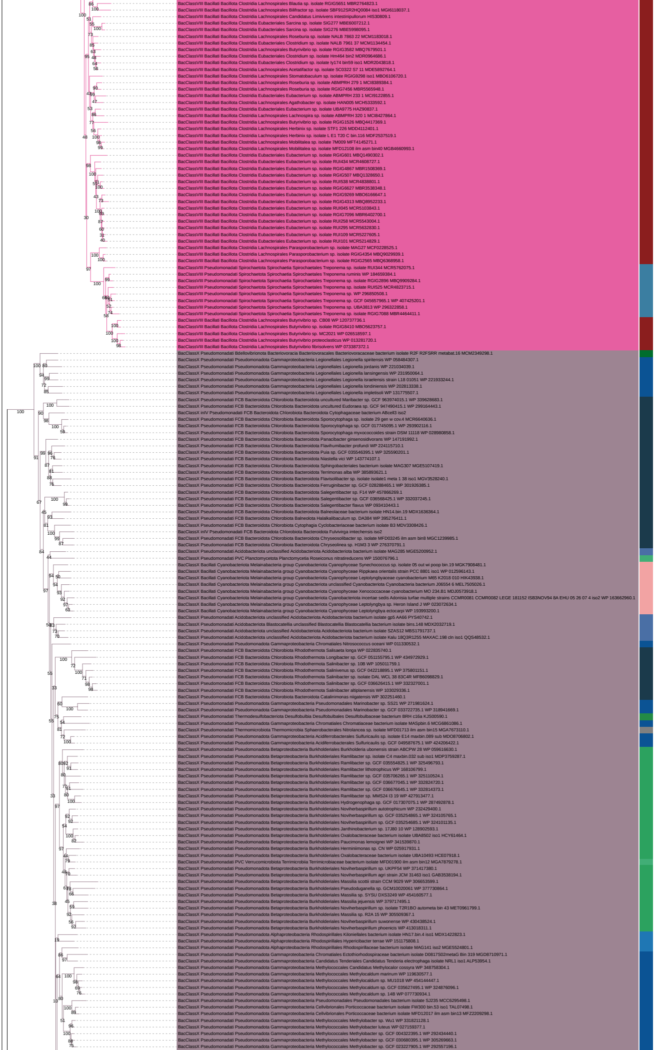
BacClassVIII Bacillati Bacillota Clostridia Lachnospirales Blautia sp. isolate RGIG5651 MBR2764823.1
BacClassVIII Bacillati Bacillota Clostridia Lachnospirales Bilifractor sp. isolate SBF912SR2HQ0084 iso1 MGI6118037.1
BacClassVIII Bacillati Bacillota Clostridia Lachnospirales Candidatus Limivivens intestinipullorum HIS30809.1
BacClassVIII Bacillati Bacillota Clostridia Eubacteriales Sarcina sp. isolate SIG277 MBE6007212.1
BacClassVIII Bacillati Bacillota Clostridia Eubacteriales Sarcina sp. isolate SIG276 MBE5998095.1
BacClassVIII Bacillati Bacillota Clostridia Lachnospirales Roseburia sp. isolate NALB 7863 22 MCM1183018.1
BacClassVIII Bacillati Bacillota Clostridia Eubacteriales Clostridium sp. isolate NALB 7961 37 MCM1134454.1
BacClassVIII Bacillati Bacillota Clostridia Lachnospirales Butyrivibrio sp. isolate RGIG3582 MBQ7679501.1
BacClassVIII Bacillati Bacillota Clostridia Eubacteriales Clostridium sp. isolate Hm464 bin2 MDR0964686.1
BacClassVIII Bacillati Bacillota Clostridia Eubacteriales Clostridium sp. isolate ly174 bin59 iso1 MDR2043818.1
BacClassVIII Bacillati Bacillota Clostridia Lachnospirales Acetatifactor sp. isolate SC0322 S7 11 MDE5892764.1
BacClassVIII Bacillati Bacillota Clostridia Lachnospirales Stomatobaculum sp. isolate RGIG9298 iso1 MBO6106720.1
BacClassVIII Bacillati Bacillota Clostridia Lachnospirales Roseburia sp. isolate ABMPRH 279 1 MCI8389384.1
BacClassVIII Bacillati Bacillota Clostridia Lachnospirales Roseburia sp. isolate RGIG7456 MBR5565948.1
BacClassVIII Bacillati Bacillota Clostridia Eubacteriales Eubacterium sp. isolate ABMPRH 233 1 MCI9122855.1
BacClassVIII Bacillati Bacillota Clostridia Lachnospirales Agathobacter sp. isolate HAN005 MCH5333592.1
BacClassVIII Bacillati Bacillota Clostridia Eubacteriales Eubacterium sp. isolate UBA9775 HAZ90837.1
BacClassVIII Bacillati Bacillota Clostridia Lachnospirales Lachnospira sp. isolate ABMPRH 320 1 MCI8427864.1
BacClassVIII Bacillati Bacillota Clostridia Lachnospirales Butyrivibrio sp. isolate RGIG1526 MBQ4417369.1
BacClassVIII Bacillati Bacillota Clostridia Lachnospirales Herbinix sp. isolate STF1 226 MDD4112401.1
BacClassVIII Bacillati Bacillota Clostridia Lachnospirales Herbinix sp. isolate L E1 T20 C bin.116 MDF2537519.1
BacClassVIII Bacillati Bacillota Clostridia Lachnospirales Mobilitalea sp. isolate 7M009 MFT4145271.1
BacClassVIII Bacillati Bacillota Clostridia Lachnospirales Mobilitalea sp. isolate MFD12108 ilm asm bin40 MGB4660993.1
BacClassVIII Bacillati Bacillota Clostridia Eubacteriales Eubacterium sp. isolate RGIG601 MBQ1490302.1
BacClassVIII Bacillati Bacillota Clostridia Eubacteriales Eubacterium sp. isolate RUI434 MCR4608727.1
BacClassVIII Bacillati Bacillota Clostridia Eubacteriales Eubacterium sp. isolate RGIG4867 MBR1508369.1
BacClassVIII Bacillati Bacillota Clostridia Eubacteriales Eubacterium sp. isolate RGIG507 MBQ1328650.1
BacClassVIII Bacillati Bacillota Clostridia Eubacteriales Eubacterium sp. isolate RUI538 MCR4838801.1
BacClassVIII Bacillati Bacillota Clostridia Eubacteriales Eubacterium sp. isolate RGIG6627 MBR3538348.1
BacClassVIII Bacillati Bacillota Clostridia Eubacteriales Eubacterium sp. isolate RGIG9269 MBO6166647.1
BacClassVIII Bacillati Bacillota Clostridia Eubacteriales Eubacterium sp. isolate RGIG4313 MBQ8952233.1
BacClassVIII Bacillati Bacillota Clostridia Eubacteriales Eubacterium sp. isolate RUI045 MCR5103843.1
BacClassVIII Bacillati Bacillota Clostridia Eubacteriales Eubacterium sp. isolate RGIG7096 MBR6402700.1
BacClassVIII Bacillati Bacillota Clostridia Eubacteriales Eubacterium sp. isolate RUI258 MCR5543004.1
BacClassVIII Bacillati Bacillota Clostridia Eubacteriales Eubacterium sp. isolate RUI295 MCR5632830.1
BacClassVIII Bacillati Bacillota Clostridia Eubacteriales Eubacterium sp. isolate RUI109 MCR5227605.1
BacClassVIII Bacillati Bacillota Clostridia Eubacteriales Eubacterium sp. isolate RUI101 MCR5214829.1
BacClassVIII Bacillati Bacillota Clostridia Lachnospirales Parasporobacterium sp. isolate MAG27 MCF0228525.1
BacClassVIII Bacillati Bacillota Clostridia Lachnospirales Parasporobacterium sp. isolate RGIG4354 MBQ9029939.1
BacClassVIII Bacillati Bacillota Clostridia Lachnospirales Parasporobacterium sp. isolate RGIG2565 MBQ6368958.1
BacClassVIII Pseudomonadati Spirochaetota Spirochaetia Spirochaetales Treponema sp. isolate RUI344 MCR5762075.1
BacClassVIII Pseudomonadati Spirochaetota Spirochaetia Spirochaetales Treponema ruminis WP 184659384.1
BacClassVIII Pseudomonadati Spirochaetota Spirochaetia Spirochaetales Treponema sp. isolate RGIG2896 MBQ9909284.1
BacClassVIII Pseudomonadati Spirochaetota Spirochaetia Spirochaetales Treponema sp. isolate RUI525 MCR4823715.1
BacClassVIII Pseudomonadati Spirochaetota Spirochaetia Spirochaetales Treponema sp. WP 296850508.1
BacClassVIII Pseudomonadati Spirochaetota Spirochaetia Spirochaetales Treponema sp. GCF 045657965.1 WP 407425201.1
BacClassVIII Pseudomonadati Spirochaetota Spirochaetia Spirochaetales Treponema sp. UBA3813 WP 296322858.1
BacClassVIII Pseudomonadati Spirochaetota Spirochaetia Spirochaetales Treponema sp. isolate RGIG7088 MBR4464411.1
BacClassVIII Bacillati Bacillota Clostridia Lachnospirales Butyrivibrio sp. CB08 WP 120737736.1
BacClassVIII Bacillati Bacillota Clostridia Lachnospirales Butyrivibrio sp. isolate RGIG8410 MBO5623757.1
BacClassVIII Bacillati Bacillota Clostridia Lachnospirales Butyrivibrio sp. MC2021 WP 026518597.1
BacClassVIII Bacillati Bacillota Clostridia Lachnospirales Butyrivibrio proteoclasticus WP 013281720.1
BacClassVIII Bacillati Bacillota Clostridia Lachnospirales Butyrivibrio fibrisolvens WP 073387372.1
BacClassX Pseudomonadati Bdellovibrionota Bacteriovoracia Bacteriovoracales Bacteriovoracaceae bacterium isolate R2F R2FSRR metabat.16 MCM2349298.1
BacClassX Pseudomonadati Pseudomonadota Gammaproteobacteria Legionellales Legionella spiritensis WP 058484307.1
BacClassX Pseudomonadati Pseudomonadota Gammaproteobacteria Legionellales Legionella jordanis WP 221034039.1
BacClassX Pseudomonadati Pseudomonadota Gammaproteobacteria Legionellales Legionella lansingensis WP 231950064.1
BacClassX Pseudomonadati Pseudomonadota Gammaproteobacteria Legionellales Legionella israelensis strain L18 01051 WP 221933244.1
BacClassX Pseudomonadati Pseudomonadota Gammaproteobacteria Legionellales Legionella londiniensis WP 202813338.1
BacClassX Pseudomonadati Pseudomonadota Gammaproteobacteria Legionellales Legionella impletisoli WP 131775507.1
BacClassX Pseudomonadati FCB Bacteroidota Chlorobiota Bacteroidota uncultured Maribacter sp. GCF 963974015.1 WP 339628683.1
BacClassX Pseudomonadati FCB Bacteroidota Chlorobiota Bacteroidota uncultured Eudoraea sp. GCF 947490415.1 WP 299164443.1
BacClassX inIV Pseudomonadati FCB Bacteroidota Chlorobiota Bacteroidota Cytophagaceae bacterium ABcell3 iso2
BacClassX Pseudomonadati FCB Bacteroidota Chlorobiota Bacteroidota Sporocytophaga sp. isolate 29 gen w cov.4 MCR6640636.1
BacClassX Pseudomonadati FCB Bacteroidota Chlorobiota Bacteroidota Sporocytophaga sp. GCF 017745095.1 WP 293902116.1
BacClassX Pseudomonadati FCB Bacteroidota Chlorobiota Bacteroidota Sporocytophaga myxococcoides strain DSM 11118 WP 028980858.1
BacClassX Pseudomonadati FCB Bacteroidota Chlorobiota Bacteroidota Panacibacter ginsenosidivorans WP 147191992.1
BacClassX Pseudomonadati FCB Bacteroidota Chlorobiota Bacteroidota Flavihumibacter profundi WP 224115710.1
BacClassX Pseudomonadati FCB Bacteroidota Chlorobiota Bacteroidota Puia sp. GCF 035546395.1 WP 325590201.1
BacClassX Pseudomonadati FCB Bacteroidota Chlorobiota Bacteroidota Niastella vici WP 143774107.1
BacClassX Pseudomonadati FCB Bacteroidota Chlorobiota Bacteroidota Sphingobacteriales bacterium isolate MAG307 MGE5107419.1
BacClassX Pseudomonadati FCB Bacteroidota Chlorobiota Bacteroidota Terrimonas alba WP 385893621.1
BacClassX Pseudomonadati FCB Bacteroidota Chlorobiota Bacteroidota Flavisolibacter sp. isolate isolate1 meta 1 38 iso1 MGV3528240.1
BacClassX Pseudomonadati FCB Bacteroidota Chlorobiota Bacteroidota Ferruginibacter sp. GCF 028288465.1 WP 301926385.1
BacClassX Pseudomonadati FCB Bacteroidota Chlorobiota Bacteroidota Salegentibacter sp. F14 WP 457866269.1
BacClassX Pseudomonadati FCB Bacteroidota Chlorobiota Bacteroidota Salegentibacter sp. GCF 036568425.1 WP 332037245.1
BacClassX Pseudomonadati FCB Bacteroidota Chlorobiota Bacteroidota Salegentibacter flavus WP 093410443.1
BacClassX Pseudomonadati FCB Bacteroidota Chlorobiota Bacteroidota Balneolaceae bacterium isolate HN14.bin.19 MDX1636364.1
BacClassX Pseudomonadati FCB Bacteroidota Chlorobiota Balneolota Halalkalibaculum sp. DA384 WP 395276411.1
BacClassX Pseudomonadati FCB Bacteroidota Chlorobiota Cytophagia Cyclobacteriaceae bacterium isolate B3 MDV3308426.1
BacClassX inIV Pseudomonadati FCB Bacteroidota Chlorobiota Bacteroidota Fulvivirga imtechensis iso2
BacClassX Pseudomonadati FCB Bacteroidota Chlorobiota Bacteroidota Chryseosolibacter sp. isolate MFD03245 ilm asm bin8 MGC1239985.1
BacClassX Pseudomonadati FCB Bacteroidota Chlorobiota Bacteroidota Chryseolinea sp. H1M3 3 WP 276370791.1
BacClassX Pseudomonadati Acidobacteriota unclassified Acidobacteriota Acidobacteriota bacterium isolate MAG285 MGE5200952.1
BacClassX Pseudomonadati PVC Planctomycetota Planctomycetia Roseiconus nitratireducens WP 150076796.1
BacClassX Bacillati Cyanobacteriota Melainabacteria group Cyanobacteriota Cyanophyceae Synechococcus sp. isolate 05 out wi poop bin.19 MGK7908481.1
BacClassX Bacillati Cyanobacteriota Melainabacteria group Cyanobacteriota Cyanophyceae Rippkaea orientalis strain PCC 8801 iso1 WP 012596143.1
BacClassX Bacillati Cyanobacteriota Melainabacteria group Cyanobacteriota Cyanophyceae Leptolyngbyaceae cyanobacterium M65 K2018 010 HIK43938.1
BacClassX Bacillati Cyanobacteriota Melainabacteria group Cyanobacteriota unclassified Cyanobacteriota Cyanobacteria bacterium J06554 6 MEL7505026.1
BacClassX Bacillati Cyanobacteriota Melainabacteria group Cyanobacteriota Cyanophyceae Xenococcaceae cyanobacterium MO 234.B1 MDJ0573918.1
BacClassX Bacillati Cyanobacteriota Melainabacteria group Cyanobacteriota Cyanobacteriota incertae sedis Adonisia turfae multiple strains CCMR0081 CCMR0082 LEGE 181152 ISB3NOV94 8A EHU 05 26 07 4 iso2 WP 163662960.1
BacClassX Bacillati Cyanobacteriota Melainabacteria group Cyanobacteriota Cyanophyceae Leptolyngbya sp. Heron Island J WP 023072634.1
BacClassX Bacillati Cyanobacteriota Melainabacteria group Cyanobacteriota Cyanophyceae Leptolyngbya ectocarpi WP 193993200.1
BacClassX Pseudomonadati Acidobacteriota unclassified Acidobacteriota Acidobacteriota bacterium isolate gp5 AA66 PYS40742.1
BacClassX Pseudomonadati Acidobacteriota Blastocatellia unclassified Blastocatellia Blastocatellia bacterium isolate bins.148 MDX2032719.1
BacClassX Pseudomonadati Acidobacteriota unclassified Acidobacteriota Acidobacteriota bacterium isolate SZAS12 MBS1791737.1
BacClassX Pseudomonadati Acidobacteriota unclassified Acidobacteriota Acidobacteriota bacterium isolate Kalu 18Q3R1255 MAXAC.198 cln iso1 QQS48532.1
BacClassX Pseudomonadati Pseudomonadota Gammaproteobacteria Chromatiales Nitrosococcus oceani WP 011330532.1
BacClassX Pseudomonadati FCB Bacteroidota Chlorobiota Rhodothermota Salisaeta longa WP 022835740.1
BacClassX Pseudomonadati FCB Bacteroidota Chlorobiota Rhodothermota Longibacter sp. GCF 051155795.1 WP 434972929.1
BacClassX Pseudomonadati FCB Bacteroidota Chlorobiota Rhodothermota Salinibacter sp. 10B WP 105011759.1
BacClassX Pseudomonadati FCB Bacteroidota Chlorobiota Rhodothermota Salinivenus sp. GCF 042218895.1 WP 375801151.1
BacClassX Pseudomonadati FCB Bacteroidota Chlorobiota Rhodothermota Salinibacter sp. isolate DAL WCL 38 83C4R MFB6098829.1
BacClassX Pseudomonadati FCB Bacteroidota Chlorobiota Rhodothermota Salinibacter sp. GCF 036626415.1 WP 332327001.1
BacClassX Pseudomonadati FCB Bacteroidota Chlorobiota Rhodothermota Salinibacter altiplanensis WP 103029336.1
BacClassX Pseudomonadati FCB Bacteroidota Chlorobiota Bacteroidota Catalinimonas niigatensis WP 302251460.1
BacClassX Pseudomonadati Pseudomonadota Gammaproteobacteria Pseudomonadales Marinobacter sp. SS21 WP 271981624.1
BacClassX Pseudomonadati Pseudomonadota Gammaproteobacteria Pseudomonadales Marinobacter sp. GCF 033722735.1 WP 318941669.1
BacClassX Pseudomonadati Thermodesulfobacteriota Desulfobulbia Desulfobulbales Desulfobulbaceae bacterium BRH c16a KJS00590.1
BacClassX Pseudomonadati Pseudomonadota Gammaproteobacteria Chromatiales Chromatiaceae bacterium isolate MASpbin.6 MCG6861086.1
BacClassX Pseudomonadati Thermomicrobiota Thermomicrobia Sphaerobacterales Nitrolancea sp. isolate MFD01713 ilm asm bin15 MGA7673110.1
BacClassX Pseudomonadati Pseudomonadota Gammaproteobacteria Acidiferrobacterales Sulfuricaulis sp. isolate E14 maxbin.089 sub MDO8706802.1
BacClassX Pseudomonadati Pseudomonadota Gammaproteobacteria Acidiferrobacterales Sulfuricaulis sp. GCF 049587675.1 WP 424206422.1
BacClassX Pseudomonadati Pseudomonadota Betaproteobacteria Burkholderiales Burkholderia ubonensis strain ABCPW 28 WP 059616630.1
BacClassX Pseudomonadati Pseudomonadota Betaproteobacteria Burkholderiales Ramlibacter sp. isolate C4 maxbin.032 sub iso1 MDP3759287.1
BacClassX Pseudomonadati Pseudomonadota Betaproteobacteria Burkholderiales Ramlibacter sp. GCF 035554825.1 WP 325496793.1
BacClassX Pseudomonadati Pseudomonadota Betaproteobacteria Burkholderiales Ramlibacter lithotrophicus WP 168106799.1
BacClassX Pseudomonadati Pseudomonadota Betaproteobacteria Burkholderiales Ramlibacter sp. GCF 035706265.1 WP 325110524.1
BacClassX Pseudomonadati Pseudomonadota Betaproteobacteria Burkholderiales Ramlibacter sp. GCF 036677045.1 WP 332824720.1
BacClassX Pseudomonadati Pseudomonadota Betaproteobacteria Burkholderiales Ramlibacter sp. GCF 036676645.1 WP 332814373.1
BacClassX Pseudomonadati Pseudomonadota Betaproteobacteria Burkholderiales Ramlibacter sp. MMS24 I3 19 WP 427913477.1
BacClassX Pseudomonadati Pseudomonadota Betaproteobacteria Burkholderiales Hydrogenophaga sp. GCF 017307075.1 WP 287492878.1
BacClassX Pseudomonadati Pseudomonadota Betaproteobacteria Burkholderiales Noviherbaspirillum autotrophicum WP 232429400.1
BacClassX Pseudomonadati Pseudomonadota Betaproteobacteria Burkholderiales Noviherbaspirillum sp. GCF 035254865.1 WP 324105765.1
BacClassX Pseudomonadati Pseudomonadota Betaproteobacteria Burkholderiales Noviherbaspirillum sp. GCF 035254685.1 WP 324101135.1
BacClassX Pseudomonadati Pseudomonadota Betaproteobacteria Burkholderiales Janthinobacterium sp. 17J80 10 WP 128902593.1
BacClassX Pseudomonadati Pseudomonadota Betaproteobacteria Burkholderiales Oxalobacteraceae bacterium isolate UBA8502 iso1 HCY61464.1
BacClassX Pseudomonadati Pseudomonadota Betaproteobacteria Burkholderiales Paucimonas lemoignei WP 341539870.1
BacClassX Pseudomonadati Pseudomonadota Betaproteobacteria Burkholderiales Herminiimonas sp. CN WP 025917931.1
BacClassX Pseudomonadati Pseudomonadota Betaproteobacteria Burkholderiales Oxalobacteraceae bacterium isolate UBA10493 HCE07918.1
BacClassX Pseudomonadati PVC Verrucomicrobiota Terrimicrobiia Terrimicrobiaceae bacterium isolate MFD01900 ilm asm bin12 MGA7879278.1
BacClassX Pseudomonadati Pseudomonadota Betaproteobacteria Burkholderiales Noviherbaspirillum sp. UKPF54 WP 371417380.1
BacClassX Pseudomonadati Pseudomonadota Betaproteobacteria Burkholderiales Noviherbaspirillum agri strain JCM 31463 iso1 GAB3538194.1
BacClassX Pseudomonadati Pseudomonadota Betaproteobacteria Burkholderiales Massilia scottii strain CCM 9029 WP 306653599.1
BacClassX Pseudomonadati Pseudomonadota Betaproteobacteria Burkholderiales Pseudoduganella sp. GCM10020061 WP 377730864.1
BacClassX Pseudomonadati Pseudomonadota Betaproteobacteria Burkholderiales Massilia sp. SYSU DXS3249 WP 454160577.1
BacClassX Pseudomonadati Pseudomonadota Betaproteobacteria Burkholderiales Massilia jejuensis WP 379717495.1
BacClassX Pseudomonadati Pseudomonadota Betaproteobacteria Burkholderiales Noviherbaspirillum sp. isolate T2R1BO autometa bin 43 MET0961799.1
BacClassX Pseudomonadati Pseudomonadota Betaproteobacteria Burkholderiales Massilia sp. R2A 15 WP 305509367.1
BacClassX Pseudomonadati Pseudomonadota Betaproteobacteria Burkholderiales Noviherbaspirillum suwonense WP 430438524.1
BacClassX Pseudomonadati Pseudomonadota Betaproteobacteria Burkholderiales Noviherbaspirillum phoenicis WP 413018311.1
BacClassX Pseudomonadati Pseudomonadota Alphaproteobacteria Rhodospirillales Kiloniellales bacterium isolate HN17.bin.4 iso1 MDX1422823.1
BacClassX Pseudomonadati Pseudomonadota Alphaproteobacteria Rhodospirillales Hypericibacter terrae WP 151175808.1
BacClassX Pseudomonadati Pseudomonadota Alphaproteobacteria Rhodospirillales Rhodospirillaceae bacterium isolate MAG141 iso2 MGE5524801.1
BacClassX Pseudomonadati Pseudomonadota Gammaproteobacteria Chromatiales Ectothiorhodospiraceae bacterium isolate D0817S02metaG Bin 319 MGD8710971.1
BacClassX Pseudomonadati Pseudomonadota Gammaproteobacteria Candidatus Tenderiales Candidatus Tenderia electrophaga isolate NRL1 iso1 ALP53954.1
BacClassX Pseudomonadati Pseudomonadota Gammaproteobacteria Methylococcales Candidatus Methylocalor cossyra WP 348758304.1
BacClassX Pseudomonadati Pseudomonadota Gammaproteobacteria Methylococcales Methylocaldum marinum WP 119630577.1
BacClassX Pseudomonadati Pseudomonadota Gammaproteobacteria Methylococcales Methylocaldum sp. MU1018 WP 454144447.1
BacClassX Pseudomonadati Pseudomonadota Gammaproteobacteria Methylococcales Methylocaldum sp. GCF 035627495.1 WP 324876096.1
BacClassX Pseudomonadati Pseudomonadota Gammaproteobacteria Methylococcales Methylocaldum sp. 14B WP 077730934.1
BacClassX Pseudomonadati Pseudomonadota Gammaproteobacteria Pseudomonadales Pseudomonadales bacterium isolate SJ235 MCC6295498.1
BacClassX Pseudomonadati Pseudomonadota Gammaproteobacteria Cellvibrionales Porticoccaceae bacterium isolate FW300 bin.53 iso1 TAL07498.1
BacClassX Pseudomonadati Pseudomonadota Gammaproteobacteria Cellvibrionales Porticoccaceae bacterium isolate MFD12017 ilm asm bin13 MFZ2209298.1
BacClassX Pseudomonadati Pseudomonadota Gammaproteobacteria Methylococcales Methylobacter sp. Wu1 WP 331821128.1
BacClassX Pseudomonadati Pseudomonadota Gammaproteobacteria Methylococcales Methylobacter luteus WP 027159377.1
BacClassX Pseudomonadati Pseudomonadota Gammaproteobacteria Methylococcales Methylobacter sp. GCF 004322395.1 WP 292434440.1
BacClassX Pseudomonadati Pseudomonadota Gammaproteobacteria Methylococcales Methylobacter sp. GCF 030680395.1 WP 305269663.1
BacClassX Pseudomonadati Pseudomonadota Gammaproteobacteria Methylococcales Methylobacter sp. GCF 023227905.1 WP 292557196.1

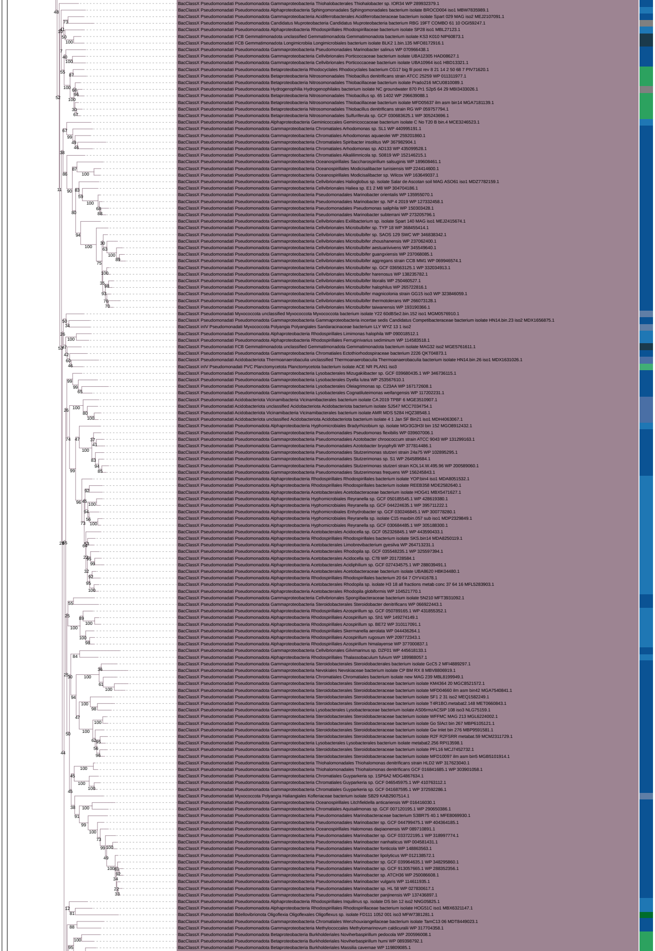
BacClassX Pseudomonadati Pseudomonadota Gammaproteobacteria Thiohalobacterales Thiohalobacter sp. IOR34 WP 289932379.1
BacClassX Pseudomonadati Pseudomonadota Alphaproteobacteria Sphingomonadales Sphingomonadales bacterium isolate BROCD004 iso1 MBW7835989.1
BacClassX Pseudomonadati Pseudomonadota Gammaproteobacteria Acidiferrobacterales Acidiferrobacteraceae bacterium isolate Spart 029 MAG iso2 MEJ2107091.1
BacClassX Pseudomonadati Pseudomonadota Candidatus Muproteobacteria Candidatus Muproteobacteria bacterium RBG 19FT COMBO 61 10 OGI59247.1
BacClassX Pseudomonadati Pseudomonadota Alphaproteobacteria Rhodospirillales Rhodospirillaceae bacterium isolate SP28 iso1 MBL27123.1
BacClassX Pseudomonadati FCB Gemmatimonadota unclassified Gemmatimonadota Gemmatimonadota bacterium isolate KS3 K010 NIP60873.1
BacClassX Pseudomonadati FCB Gemmatimonadota Longimicrobiia Longimicrobiales bacterium isolate BLK2 1.bin.135 MFO8172916.1
BacClassX Pseudomonadati Pseudomonadota Gammaproteobacteria Pseudomonadales Marinobacter salinus WP 070966438.1
BacClassX Pseudomonadati Pseudomonadota Gammaproteobacteria Cellvibrionales Porticoccaceae bacterium isolate UBA12305 HAD08627.1
BacClassX Pseudomonadati Pseudomonadota Gammaproteobacteria Cellvibrionales Porticoccaceae bacterium isolate UBA10964 iso1 HBD13321.1
BacClassX Pseudomonadati Pseudomonadota Betaproteobacteria Rhodocyclales Rhodocyclales bacterium CG17 big fil post rev 8 21 14 2 50 68 7 PIV71620.1
BacClassX Pseudomonadati Pseudomonadota Betaproteobacteria Nitrosomonadales Thiobacillus denitrificans strain ATCC 25259 WP 011311977.1
BacClassX Pseudomonadati Pseudomonadota Betaproteobacteria Nitrosomonadales Thiobacillaceae bacterium isolate Prado216 MCU0810089.1
BacClassX Pseudomonadati Pseudomonadota Hydrogenophilia Hydrogenophilales bacterium isolate NC groundwater 870 Pr1 S2p5 64 29 MBI3433026.1
BacClassX Pseudomonadati Pseudomonadota Betaproteobacteria Nitrosomonadales Thiobacillus sp. 65 1402 WP 296639088.1
BacClassX Pseudomonadati Pseudomonadota Betaproteobacteria Nitrosomonadales Thiobacillaceae bacterium isolate MFD05637 ilm asm bin14 MGA7181139.1
BacClassX Pseudomonadati Pseudomonadota Betaproteobacteria Nitrosomonadales Thiobacillus denitrificans strain RG WP 059757794.1
BacClassX Pseudomonadati Pseudomonadota Betaproteobacteria Nitrosomonadales Sulfuriferula sp. GCF 030683625.1 WP 305243696.1
BacClassX Pseudomonadati Pseudomonadota Alphaproteobacteria Geminicoccales Geminicoccaceae bacterium isolate C No T20 B bin.4 MCE3246523.1
BacClassX Pseudomonadati Pseudomonadota Gammaproteobacteria Chromatiales Arhodomonas sp. SL1 WP 440995191.1
BacClassX Pseudomonadati Pseudomonadota Gammaproteobacteria Chromatiales Arhodomonas aquaeolei WP 259201860.1
BacClassX Pseudomonadati Pseudomonadota Gammaproteobacteria Chromatiales Spiribacter insolitus WP 367982904.1
BacClassX Pseudomonadati Pseudomonadota Gammaproteobacteria Chromatiales Arhodomonas sp. AD133 WP 435099528.1
BacClassX Pseudomonadati Pseudomonadota Gammaproteobacteria Chromatiales Alkalilimnicola sp. S0819 WP 152146215.1
BacClassX Pseudomonadati Pseudomonadota Gammaproteobacteria Oceanospirillales Saccharospirillum salsuginis WP 189608461.1
BacClassX Pseudomonadati Pseudomonadota Gammaproteobacteria Oceanospirillales Modicisalibacter tunisiensis WP 224414600.1
BacClassX Pseudomonadati Pseudomonadota Gammaproteobacteria Oceanospirillales Modicisalibacter sp. Wilcox WP 163649037.1
BacClassX Pseudomonadati Pseudomonadota Gammaproteobacteria Cellvibrionales Halioglobus sp. isolate Salar de Ascotan soil MAG ASO61 iso1 MDZ7782159.1
BacClassX Pseudomonadati Pseudomonadota Gammaproteobacteria Cellvibrionales Haliea sp. E1 2 M8 WP 304704186.1
BacClassX Pseudomonadati Pseudomonadota Gammaproteobacteria Pseudomonadales Marinobacter orientalis WP 135955070.1
BacClassX Pseudomonadati Pseudomonadota Gammaproteobacteria Pseudomonadales Marinobacter sp. NP 4 2019 WP 127332458.1
BacClassX Pseudomonadati Pseudomonadota Gammaproteobacteria Pseudomonadales Pseudomonas saliphila WP 150303428.1
BacClassX Pseudomonadati Pseudomonadota Gammaproteobacteria Pseudomonadales Marinobacter subterrani WP 273205796.1
BacClassX Pseudomonadati Pseudomonadota Gammaproteobacteria Cellvibrionales Exilibacterium sp. isolate Spart 140 MAG iso1 MEJ2415674.1
BacClassX Pseudomonadati Pseudomonadota Gammaproteobacteria Cellvibrionales Microbulbifer sp. TYP 18 WP 368455414.1
BacClassX Pseudomonadati Pseudomonadota Gammaproteobacteria Cellvibrionales Microbulbifer sp. SAOS 129 SWC WP 346838342.1
BacClassX Pseudomonadati Pseudomonadota Gammaproteobacteria Cellvibrionales Microbulbifer zhoushanensis WP 237062400.1
BacClassX Pseudomonadati Pseudomonadota Gammaproteobacteria Cellvibrionales Microbulbifer aestuariivivens WP 345549640.1
BacClassX Pseudomonadati Pseudomonadota Gammaproteobacteria Cellvibrionales Microbulbifer guangxiensis WP 237068085.1
BacClassX Pseudomonadati Pseudomonadota Gammaproteobacteria Cellvibrionales Microbulbifer aggregans strain CCB MM1 WP 069946574.1
BacClassX Pseudomonadati Pseudomonadota Gammaproteobacteria Cellvibrionales Microbulbifer sp. GCF 036563125.1 WP 332034913.1
BacClassX Pseudomonadati Pseudomonadota Gammaproteobacteria Cellvibrionales Microbulbifer harenosus WP 138235782.1
BacClassX Pseudomonadati Pseudomonadota Gammaproteobacteria Cellvibrionales Microbulbifer litoralis WP 250460527.1
BacClassX Pseudomonadati Pseudomonadota Gammaproteobacteria Cellvibrionales Microbulbifer halophilus WP 265722816.1
BacClassX Pseudomonadati Pseudomonadota Gammaproteobacteria Cellvibrionales Microbulbifer magnicolonia strain GG15 iso3 WP 323846059.1
BacClassX Pseudomonadati Pseudomonadota Gammaproteobacteria Cellvibrionales Microbulbifer thermotolerans WP 266073128.1
BacClassX Pseudomonadati Pseudomonadota Gammaproteobacteria Cellvibrionales Microbulbifer taiwanensis WP 193190366.1
BacClassX Pseudomonadati Myxococcota unclassified Myxococcota Myxococcota bacterium isolate Y22 60dBSe2.bin.152 iso1 MGM0576910.1
BacClassX Pseudomonadati Pseudomonadota Gammaproteobacteria Gammaproteobacteria incertae sedis Candidatus Competibacteraceae bacterium isolate HN14.bin.23 iso2 MDX1656875.1
BacClassX inIV Pseudomonadati Myxococcota Polyangia Polyangiales Sandaracinaceae bacterium LLY WYZ 13 1 iso2
BacClassX Pseudomonadati Pseudomonadota Alphaproteobacteria Rhodospirillales Limimonas halophila WP 090018512.1
BacClassX Pseudomonadati Pseudomonadota Alphaproteobacteria Rhodospirillales Ferruginivarius sediminum WP 114583518.1
BacClassX Pseudomonadati FCB Gemmatimonadota unclassified Gemmatimonadota Gemmatimonadota bacterium isolate MAG32 iso2 MGE5761611.1
BacClassX Pseudomonadati Pseudomonadota Gammaproteobacteria Chromatiales Ectothiorhodospiraceae bacterium 2226 QKT04873.1
BacClassX Pseudomonadati Acidobacteriota Thermoanaerobaculia unclassified Thermoanaerobaculia Thermoanaerobaculia bacterium isolate HN14.bin.26 iso1 MDX1631026.1
BacClassX inIV Pseudomonadati PVC Planctomycetota Planctomycetota bacterium isolate ACE NR PLAN1 iso3
BacClassX Pseudomonadati Pseudomonadota Gammaproteobacteria Lysobacterales Mizugakiibacter sp. GCF 039680435.1 WP 346736115.1
BacClassX Pseudomonadati Pseudomonadota Gammaproteobacteria Lysobacterales Dyella lutea WP 253567610.1
BacClassX Pseudomonadati Pseudomonadota Gammaproteobacteria Lysobacterales Oleiagrimonas sp. C23AA WP 167172608.1
BacClassX Pseudomonadati Pseudomonadota Gammaproteobacteria Lysobacterales Cognatiluteimonas weifangensis WP 117202231.1
BacClassX Pseudomonadati Acidobacteriota Vicinamibacteria Vicinamibacterales bacterium isolate CA 2019 TPBF 6 MGE3510907.1
BacClassX Pseudomonadati Acidobacteriota unclassified Acidobacteriota Acidobacteriota bacterium isolate SJ547 MCC7034754.1
BacClassX Pseudomonadati Acidobacteriota Vicinamibacteria Vicinamibacterales bacterium isolate AMR MDS 5284 HQZ38548.1
BacClassX Pseudomonadati Acidobacteriota unclassified Acidobacteriota Acidobacteriota bacterium isolate 4 1 Jan SF Bin21 iso1 MDH4063067.1
BacClassX Pseudomonadati Pseudomonadota Alphaproteobacteria Hyphomicrobiales Bradyrhizobium sp. isolate MGr3G3H3I bin 152 MGO8912432.1
BacClassX Pseudomonadati Pseudomonadota Gammaproteobacteria Pseudomonadales Pseudomonas flexibilis WP 039607006.1
BacClassX Pseudomonadati Pseudomonadota Gammaproteobacteria Pseudomonadales Azotobacter chroococcum strain ATCC 9043 WP 131299163.1
BacClassX Pseudomonadati Pseudomonadota Gammaproteobacteria Pseudomonadales Azotobacter bryophylli WP 377814486.1
BacClassX Pseudomonadati Pseudomonadota Gammaproteobacteria Pseudomonadales Stutzerimonas stutzeri strain 24a75 WP 102895295.1
BacClassX Pseudomonadati Pseudomonadota Gammaproteobacteria Pseudomonadales Stutzerimonas sp. S1 WP 264589684.1
BacClassX Pseudomonadati Pseudomonadota Gammaproteobacteria Pseudomonadales Stutzerimonas stutzeri strain KOL14.W.495.96 WP 200589060.1
BacClassX Pseudomonadati Pseudomonadota Gammaproteobacteria Pseudomonadales Stutzerimonas frequens WP 156245843.1
BacClassX Pseudomonadati Pseudomonadota Alphaproteobacteria Rhodospirillales Rhodospirillales bacterium isolate YOP.bin4 iso1 MDA8051532.1
BacClassX Pseudomonadati Pseudomonadota Alphaproteobacteria Rhodospirillales Rhodospirillales bacterium isolate REEB358 MDE2582640.1
BacClassX Pseudomonadati Pseudomonadota Alphaproteobacteria Acetobacterales Acetobacteraceae bacterium isolate HOG41 MBX5471627.1
BacClassX Pseudomonadati Pseudomonadota Alphaproteobacteria Hyphomicrobiales Reyranella sp. GCF 050185545.1 WP 428619380.1
BacClassX Pseudomonadati Pseudomonadota Alphaproteobacteria Hyphomicrobiales Reyranella sp. GCF 044224635.1 WP 395711222.1
BacClassX Pseudomonadati Pseudomonadota Alphaproteobacteria Hyphomicrobiales Enhydrobacter sp. GCF 030246845.1 WP 300778280.1
BacClassX Pseudomonadati Pseudomonadota Alphaproteobacteria Hyphomicrobiales Reyranella sp. isolate C15 maxbin.057 sub iso1 MDP2329849.1
BacClassX Pseudomonadati Pseudomonadota Alphaproteobacteria Hyphomicrobiales Reyranella sp. GCF 030684485.1 WP 305188300.1
BacClassX Pseudomonadati Pseudomonadota Alphaproteobacteria Acetobacterales Acidocella sp. GCF 052326845.1 WP 443590433.1
BacClassX Pseudomonadati Pseudomonadota Alphaproteobacteria Rhodospirillales Rhodospirillales bacterium isolate SKS.bin14 MDA8250119.1
BacClassX Pseudomonadati Pseudomonadota Alphaproteobacteria Acetobacterales Limobrevibacterium gyesilva WP 264713231.1
BacClassX Pseudomonadati Pseudomonadota Alphaproteobacteria Acetobacterales Rhodopila sp. GCF 035548235.1 WP 325597394.1
BacClassX Pseudomonadati Pseudomonadota Alphaproteobacteria Acetobacterales Acidocella sp. C78 WP 201728584.1
BacClassX Pseudomonadati Pseudomonadota Alphaproteobacteria Acetobacterales Acidiphilium sp. GCF 027434575.1 WP 288039491.1
BacClassX Pseudomonadati Pseudomonadota Alphaproteobacteria Acetobacterales Acetobacteraceae bacterium isolate UBA8620 HBK04480.1
BacClassX Pseudomonadati Pseudomonadota Alphaproteobacteria Rhodospirillales Rhodospirillales bacterium 20 64 7 OYV41678.1
BacClassX Pseudomonadati Pseudomonadota Alphaproteobacteria Acetobacterales Rhodopila sp. isolate H3 18 all fractions metab conc 37 64 16 MFL5283903.1
BacClassX Pseudomonadati Pseudomonadota Alphaproteobacteria Acetobacterales Rhodopila globiformis WP 104521770.1
BacClassX Pseudomonadati Pseudomonadota Gammaproteobacteria Cellvibrionales Spongiibacteraceae bacterium isolate 5N210 MFT3931092.1
BacClassX Pseudomonadati Pseudomonadota Gammaproteobacteria Steroidobacterales Steroidobacter denitrificans WP 066922443.1
BacClassX Pseudomonadati Pseudomonadota Alphaproteobacteria Rhodospirillales Azospirillum sp. GCF 050789165.1 WP 431855352.1
BacClassX Pseudomonadati Pseudomonadota Alphaproteobacteria Rhodospirillales Azospirillum sp. Sh1 WP 149274149.1
BacClassX Pseudomonadati Pseudomonadota Alphaproteobacteria Rhodospirillales Azospirillum sp. BE72 WP 310117091.1
BacClassX Pseudomonadati Pseudomonadota Alphaproteobacteria Rhodospirillales Skermanella aerolata WP 044436264.1
BacClassX Pseudomonadati Pseudomonadota Alphaproteobacteria Rhodospirillales Azospirillum rugosum WP 209772343.1
BacClassX Pseudomonadati Pseudomonadota Alphaproteobacteria Rhodospirillales Azospirillum himalayense WP 377000837.1
BacClassX Pseudomonadati Pseudomonadota Gammaproteobacteria Cellvibrionales Gilvimarinus sp. DZF01 WP 445618133.1
BacClassX Pseudomonadati Pseudomonadota Alphaproteobacteria Rhodospirillales Thalassobaculum fulvum WP 189988057.1
BacClassX Pseudomonadati Pseudomonadota Gammaproteobacteria Steroidobacterales Steroidobacterales bacterium isolate GcC5 2 MFI4889297.1
BacClassX Pseudomonadati Pseudomonadota Gammaproteobacteria Nevskiales Nevskiaceae bacterium isolate CP BM RX 8 MBV8806919.1
BacClassX Pseudomonadati Pseudomonadota Gammaproteobacteria Chromatiales Chromatiales bacterium isolate new MAG 239 MBL8199949.1
BacClassX Pseudomonadati Pseudomonadota Gammaproteobacteria Steroidobacterales Steroidobacteraceae bacterium isolate KM4364 20 MGC8521572.1
BacClassX Pseudomonadati Pseudomonadota Gammaproteobacteria Steroidobacterales Steroidobacteraceae bacterium isolate MFD04660 ilm asm bin42 MGA7540841.1
BacClassX Pseudomonadati Pseudomonadota Gammaproteobacteria Steroidobacterales Steroidobacteraceae bacterium isolate SF1 2 31 iso2 MEQ1582249.1
BacClassX Pseudomonadati Pseudomonadota Gammaproteobacteria Steroidobacterales Steroidobacteraceae bacterium isolate T4R1BO.metabat2.148 MET0660843.1
BacClassX Pseudomonadati Pseudomonadota Gammaproteobacteria Lysobacterales Lysobacteraceae bacterium isolate AS06rmzACSIP 108 iso3 NLG75159.1
BacClassX Pseudomonadati Pseudomonadota Gammaproteobacteria Steroidobacterales Steroidobacteraceae bacterium isolate WFFMC MAG 213 MGL6224002.1
BacClassX Pseudomonadati Pseudomonadota Gammaproteobacteria Steroidobacterales Steroidobacteraceae bacterium isolate Go SlAct bin 267 MBP6105121.1
BacClassX Pseudomonadati Pseudomonadota Gammaproteobacteria Steroidobacterales Steroidobacteraceae bacterium isolate Gw Inlet bin 276 MBP9591581.1
BacClassX Pseudomonadati Pseudomonadota Gammaproteobacteria Steroidobacterales Steroidobacteraceae bacterium isolate R2F R2FSRR metabat.59 MCM2311729.1
BacClassX Pseudomonadati Pseudomonadota Gammaproteobacteria Lysobacterales Lysobacterales bacterium isolate metabat2.256 RPI13598.1
BacClassX Pseudomonadati Pseudomonadota Gammaproteobacteria Steroidobacterales Steroidobacteraceae bacterium isolate PFL16 MCJ7452732.1
BacClassX Pseudomonadati Pseudomonadota Gammaproteobacteria Steroidobacterales Steroidobacteraceae bacterium isolate MFD10097 ilm asm bin5 MGB5101914.1
BacClassX Pseudomonadati Pseudomonadota Gammaproteobacteria Thiohalomonadales Thiohalomonas denitrificans strain HLD2 WP 317623040.1
BacClassX Pseudomonadati Pseudomonadota Gammaproteobacteria Thiohalomonadales Thiohalomonas denitrificans GCF 016841685.1 WP 303901058.1
BacClassX Pseudomonadati Pseudomonadota Gammaproteobacteria Chromatiales Guyparkeria sp. 1SP6A2 MDG4867634.1
BacClassX Pseudomonadati Pseudomonadota Gammaproteobacteria Chromatiales Guyparkeria sp. GCF 046545975.1 WP 410763112.1
BacClassX Pseudomonadati Pseudomonadota Gammaproteobacteria Chromatiales Guyparkeria sp. GCF 041687595.1 WP 372592286.1
BacClassX Pseudomonadati Myxococcota Polyangia Haliangiales Kofleriaceae bacterium isolate SB29 KAB2907514.1
BacClassX Pseudomonadati Pseudomonadota Gammaproteobacteria Oceanospirillales Litchfieldella anticariensis WP 016416030.1
BacClassX Pseudomonadati Pseudomonadota Gammaproteobacteria Chromatiales Aquisalimonas sp. GCF 007120195.1 WP 290650386.1
BacClassX Pseudomonadati Pseudomonadota Gammaproteobacteria Pseudomonadales Marinobacteraceae bacterium S3BR75 40.1 MFE8069930.1
BacClassX Pseudomonadati Pseudomonadota Gammaproteobacteria Pseudomonadales Marinobacter sp. GCF 044799475.1 WP 404364185.1
BacClassX Pseudomonadati Pseudomonadota Gammaproteobacteria Oceanospirillales Halomonas daqiaonensis WP 089710891.1
BacClassX Pseudomonadati Pseudomonadota Gammaproteobacteria Pseudomonadales Marinobacter sp. GCF 033722195.1 WP 318997774.1
BacClassX Pseudomonadati Pseudomonadota Gammaproteobacteria Pseudomonadales Marinobacter nanhaiticus WP 004581431.1
BacClassX Pseudomonadati Pseudomonadota Gammaproteobacteria Pseudomonadales Marinobacter fonticola WP 148863563.1
BacClassX Pseudomonadati Pseudomonadota Gammaproteobacteria Pseudomonadales Marinobacter lipolyticus WP 012138572.1
BacClassX Pseudomonadati Pseudomonadota Gammaproteobacteria Pseudomonadales Marinobacter sp. GCF 039964635.1 WP 348295860.1
BacClassX Pseudomonadati Pseudomonadota Gammaproteobacteria Pseudomonadales Marinobacter sp. GCF 913057665.1 WP 288352356.1
BacClassX Pseudomonadati Pseudomonadota Gammaproteobacteria Pseudomonadales Marinobacter sp. ATCH36 WP 250086608.1
BacClassX Pseudomonadati Pseudomonadota Gammaproteobacteria Pseudomonadales Marinobacter vulgaris WP 114611935.1
BacClassX Pseudomonadati Pseudomonadota Gammaproteobacteria Pseudomonadales Marinobacter sp. HL 58 WP 027830617.1
BacClassX Pseudomonadati Pseudomonadota Gammaproteobacteria Pseudomonadales Marinobacter panjinensis WP 137436897.1
BacClassX Pseudomonadati Pseudomonadota Alphaproteobacteria Rhodospirillales Inquilinus sp. isolate DS bin 12 iso2 NNG05825.1
BacClassX Pseudomonadati Pseudomonadota Alphaproteobacteria Rhodospirillales Rhodospirillaceae bacterium isolate HOG51C iso1 MBX6321147.1
BacClassX Pseudomonadati Bdellovibrionota Oligoflexia Oligoflexales Oligoflexus sp. isolate FD111 1052 001 iso3 MFW7381281.1
BacClassX Pseudomonadati Pseudomonadota Gammaproteobacteria Chromatiales Wenzhouxiangellaceae bacterium isolate TamC13 06 MDT8449023.1
BacClassX Pseudomonadati Pseudomonadota Gammaproteobacteria Methylococcales Methylomarinovum caldicuralii WP 317704358.1
BacClassX Pseudomonadati Pseudomonadota Betaproteobacteria Burkholderiales Noviherbaspirillum pedocola WP 200596008.1
BacClassX Pseudomonadati Pseudomonadota Betaproteobacteria Burkholderiales Noviherbaspirillum humi WP 089398792.1
BacClassX Pseudomonadati Pseudomonadota Betaproteobacteria Burkholderiales Massilia cavernae WP 119809085.1

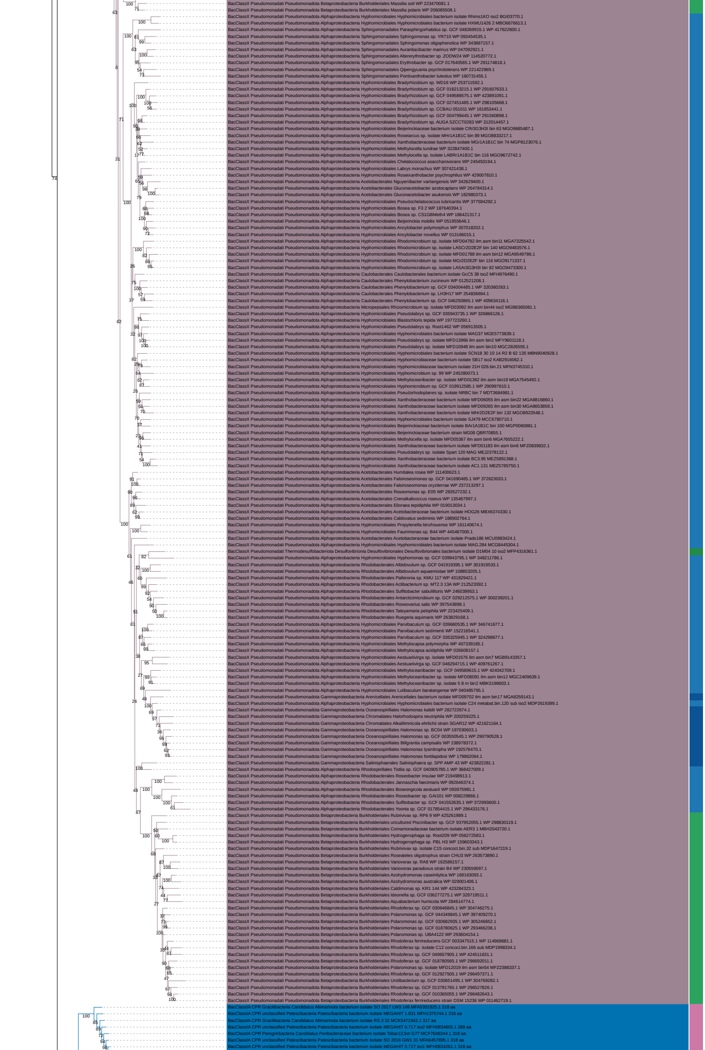

BacClassX Pseudomonadati Pseudomonadota Betaproteobacteria Burkholderiales Massilia soli WP 223470081.1
BacClassX Pseudomonadati Pseudomonadota Betaproteobacteria Burkholderiales Massilia polaris WP 206085508.1
BacClassX Pseudomonadati Pseudomonadota Alphaproteobacteria Hyphomicrobiales Hyphomicrobiales bacterium isolate Rhims1KO iso2 BGI03770.1
BacClassX Pseudomonadati Pseudomonadota Alphaproteobacteria Hyphomicrobiales Hyphomicrobiales bacterium isolate HXMU1426 2 MBO6676613.1
BacClassX Pseudomonadati Pseudomonadota Alphaproteobacteria Sphingomonadales Parasphingorhabdus sp. GCF 048269915.1 WP 417622600.1
BacClassX Pseudomonadati Pseudomonadota Alphaproteobacteria Sphingomonadales Sphingomonas sp. YR710 WP 093454535.1
BacClassX Pseudomonadati Pseudomonadota Alphaproteobacteria Sphingomonadales Sphingomonas oligophenolica WP 343887157.1
BacClassX Pseudomonadati Pseudomonadota Alphaproteobacteria Sphingomonadales Aurantiacibacter marinus WP 047092921.1
BacClassX Pseudomonadati Pseudomonadota Alphaproteobacteria Sphingomonadales Altererythrobacter sp. ZODW24 WP 114520772.1
BacClassX Pseudomonadati Pseudomonadota Alphaproteobacteria Sphingomonadales Erythrobacter sp. GCF 017640565.1 WP 291174818.1
BacClassX Pseudomonadati Pseudomonadota Alphaproteobacteria Sphingomonadales Qipengyuania psychrotolerans WP 221422969.1
BacClassX Pseudomonadati Pseudomonadota Alphaproteobacteria Sphingomonadales Pontixanthobacter luteolus WP 160731455.1
BacClassX Pseudomonadati Pseudomonadota Alphaproteobacteria Hyphomicrobiales Bradyrhizobium sp. WD16 WP 253711592.1
BacClassX Pseudomonadati Pseudomonadota Alphaproteobacteria Hyphomicrobiales Bradyrhizobium sp. GCF 016213215.1 WP 291607633.1
BacClassX Pseudomonadati Pseudomonadota Alphaproteobacteria Hyphomicrobiales Bradyrhizobium sp. GCF 049586575.1 WP 423891091.1
BacClassX Pseudomonadati Pseudomonadota Alphaproteobacteria Hyphomicrobiales Bradyrhizobium sp. GCF 027451485.1 WP 298105668.1
BacClassX Pseudomonadati Pseudomonadota Alphaproteobacteria Hyphomicrobiales Bradyrhizobium sp. CCBAU 051011 WP 161853441.1
BacClassX Pseudomonadati Pseudomonadota Alphaproteobacteria Hyphomicrobiales Bradyrhizobium sp. GCF 004799445.1 WP 291560898.1
BacClassX Pseudomonadati Pseudomonadota Alphaproteobacteria Hyphomicrobiales Bradyrhizobium sp. AUGA SZCCT0283 WP 312014457.1
BacClassX Pseudomonadati Pseudomonadota Alphaproteobacteria Hyphomicrobiales Beijerinckiaceae bacterium isolate CRr3G3H3I bin 63 MGO9685487.1
BacClassX Pseudomonadati Pseudomonadota Alphaproteobacteria Hyphomicrobiales Roseiarcus sp. isolate MHr1A1B1C bin 89 MGO8833217.1
BacClassX Pseudomonadati Pseudomonadota Alphaproteobacteria Hyphomicrobiales Xanthobacteraceae bacterium isolate MGr1A1B1C bin 74 MGP8123076.1
BacClassX Pseudomonadati Pseudomonadota Alphaproteobacteria Hyphomicrobiales Methylocella tundrae WP 322847400.1
BacClassX Pseudomonadati Pseudomonadota Alphaproteobacteria Hyphomicrobiales Methylocella sp. isolate LABRr1A1B1C bin 116 MGO9672742.1
BacClassX Pseudomonadati Pseudomonadota Alphaproteobacteria Hyphomicrobiales Chelatococcus asaccharovorans WP 245450194.1
BacClassX Pseudomonadati Pseudomonadota Alphaproteobacteria Hyphomicrobiales Labrys monachus WP 307421438.1
BacClassX Pseudomonadati Pseudomonadota Alphaproteobacteria Hyphomicrobiales Roseixanthobacter psychrophilus WP 429007810.1
BacClassX Pseudomonadati Pseudomonadota Alphaproteobacteria Acetobacterales Nguyenibacter vanlangensis WP 342629400.1
BacClassX Pseudomonadati Pseudomonadota Alphaproteobacteria Acetobacterales Gluconacetobacter azotocaptans WP 264784314.1
BacClassX Pseudomonadati Pseudomonadota Alphaproteobacteria Acetobacterales Gluconacetobacter asukensis WP 182980373.1
BacClassX Pseudomonadati Pseudomonadota Alphaproteobacteria Hyphomicrobiales Pseudochelatococcus lubricantis WP 377594292.1
BacClassX Pseudomonadati Pseudomonadota Alphaproteobacteria Hyphomicrobiales Bosea sp. F3 2 WP 187640394.1
BacClassX Pseudomonadati Pseudomonadota Alphaproteobacteria Hyphomicrobiales Bosea sp. CS1GBMeth4 WP 186421317.1
BacClassX Pseudomonadati Pseudomonadota Alphaproteobacteria Hyphomicrobiales Beijerinckia mobilis WP 051955646.1
BacClassX Pseudomonadati Pseudomonadota Alphaproteobacteria Hyphomicrobiales Ancylobacter polymorphus WP 307018202.1
BacClassX Pseudomonadati Pseudomonadota Alphaproteobacteria Hyphomicrobiales Ancylobacter novellus WP 013166015.1
BacClassX Pseudomonadati Pseudomonadota Alphaproteobacteria Hyphomicrobiales Rhodomicrobium sp. isolate MFD04782 ilm asm bin11 MGA7325542.1
BacClassX Pseudomonadati Pseudomonadota Alphaproteobacteria Hyphomicrobiales Rhodomicrobium sp. isolate LASCr2D2E2F bin 140 MGO9483576.1
BacClassX Pseudomonadati Pseudomonadota Alphaproteobacteria Hyphomicrobiales Rhodomicrobium sp. isolate MFD01788 ilm asm bin12 MGA9549786.1
BacClassX Pseudomonadati Pseudomonadota Alphaproteobacteria Hyphomicrobiales Rhodomicrobium sp. isolate MGr2D2E2F bin 116 MGO9171337.1
BacClassX Pseudomonadati Pseudomonadota Alphaproteobacteria Hyphomicrobiales Rhodomicrobium sp. isolate LASAr3G3H3I bin 82 MGO9473300.1
BacClassX Pseudomonadati Pseudomonadota Alphaproteobacteria Caulobacterales Caulobacterales bacterium isolate GcC5 38 iso2 MFI4976490.1
BacClassX Pseudomonadati Pseudomonadota Alphaproteobacteria Caulobacterales Phenylobacterium zucineum WP 012521208.1
BacClassX Pseudomonadati Pseudomonadota Alphaproteobacteria Caulobacterales Phenylobacterium sp. GCF 034004485.1 WP 320360263.1
BacClassX Pseudomonadati Pseudomonadota Alphaproteobacteria Caulobacterales Phenylobacterium sp. LH3H17 WP 254836694.1
BacClassX Pseudomonadati Pseudomonadota Alphaproteobacteria Caulobacterales Phenylobacterium sp. GCF 046293865.1 WP 409634116.1
BacClassX Pseudomonadati Pseudomonadota Alphaproteobacteria Micropepsales Rhizomicrobium sp. isolate MFD03092 ilm asm bin44 iso2 MGB8365081.1
BacClassX Pseudomonadati Pseudomonadota Alphaproteobacteria Hyphomicrobiales Pseudolabrys sp. GCF 035943735.1 WP 326866126.1
BacClassX Pseudomonadati Pseudomonadota Alphaproteobacteria Hyphomicrobiales Blastochloris tepida WP 197723260.1
BacClassX Pseudomonadati Pseudomonadota Alphaproteobacteria Hyphomicrobiales Pseudolabrys sp. Root1462 WP 056913505.1
BacClassX Pseudomonadati Pseudomonadota Alphaproteobacteria Hyphomicrobiales Hyphomicrobiales bacterium isolate MAG37 MGE5773839.1
BacClassX Pseudomonadati Pseudomonadota Alphaproteobacteria Hyphomicrobiales Pseudolabrys sp. isolate MFD13966 ilm asm bin2 MFY9601118.1
BacClassX Pseudomonadati Pseudomonadota Alphaproteobacteria Hyphomicrobiales Pseudolabrys sp. isolate MFD10948 ilm asm bin10 MGC2826555.1
BacClassX Pseudomonadati Pseudomonadota Alphaproteobacteria Hyphomicrobiales Hyphomicrobiales bacterium isolate SCN18 30 10 14 R2 B 62 135 MBN9040928.1
BacClassX Pseudomonadati Pseudomonadota Alphaproteobacteria Hyphomicrobiales Hyphomicrobiaceae bacterium isolate SB17 iso2 KAB2916062.1
BacClassX Pseudomonadati Pseudomonadota Alphaproteobacteria Hyphomicrobiales Hyphomicrobiaceae bacterium isolate 21H 026.bin.21 MFN3745310.1
BacClassX Pseudomonadati Pseudomonadota Alphaproteobacteria Hyphomicrobiales Hyphomicrobium sp. 99 WP 245280073.1
BacClassX Pseudomonadati Pseudomonadota Alphaproteobacteria Hyphomicrobiales Methyloceanibacter sp. isolate MFD01362 ilm asm bin19 MGA7545492.1
BacClassX Pseudomonadati Pseudomonadota Alphaproteobacteria Hyphomicrobiales Hyphomicrobium sp. GCF 019912585.1 WP 290997610.1
BacClassX Pseudomonadati Pseudomonadota Alphaproteobacteria Hyphomicrobiales Pseudorhodoplanes sp. isolate NRBC bin 7 MDT3684981.1
BacClassX Pseudomonadati Pseudomonadota Alphaproteobacteria Hyphomicrobiales Xanthobacteraceae bacterium isolate MFD06055 ilm asm bin22 MGA8816860.1
BacClassX Pseudomonadati Pseudomonadota Alphaproteobacteria Hyphomicrobiales Xanthobacteraceae bacterium isolate MFD09265 ilm asm bin30 MGA8653859.1
BacClassX Pseudomonadati Pseudomonadota Alphaproteobacteria Hyphomicrobiales Xanthobacteraceae bacterium isolate MHr2D2E2F bin 132 MGO8923548.1
BacClassX Pseudomonadati Pseudomonadota Alphaproteobacteria Hyphomicrobiales Hyphomicrobiales bacterium SJ479 MCC6780710.1
BacClassX Pseudomonadati Pseudomonadota Alphaproteobacteria Hyphomicrobiales Beijerinckiaceae bacterium isolate BAr1A1B1C bin 100 MGP0060881.1
BacClassX Pseudomonadati Pseudomonadota Alphaproteobacteria Hyphomicrobiales Beijerinckiaceae bacterium strain MG08 QBR70855.1
BacClassX Pseudomonadati Pseudomonadota Alphaproteobacteria Hyphomicrobiales Methylocella sp. isolate MFD05367 ilm asm bin6 MGA7655222.1
BacClassX Pseudomonadati Pseudomonadota Alphaproteobacteria Hyphomicrobiales Xanthobacteraceae bacterium isolate MFD01183 ilm asm bin6 MFZ0839832.1
BacClassX Pseudomonadati Pseudomonadota Alphaproteobacteria Hyphomicrobiales Pseudolabrys sp. isolate Spart 120 MAG MEJ2378122.1
BacClassX Pseudomonadati Pseudomonadota Alphaproteobacteria Hyphomicrobiales Xanthobacteraceae bacterium isolate BC3.95 MEZ5891368.1
BacClassX Pseudomonadati Pseudomonadota Alphaproteobacteria Hyphomicrobiales Xanthobacteraceae bacterium isolate AC1.131 MEZ5785750.1
BacClassX Pseudomonadati Pseudomonadota Alphaproteobacteria Acetobacterales Humitalea rosea WP 111400623.1
BacClassX Pseudomonadati Pseudomonadota Alphaproteobacteria Acetobacterales Falsiroseomonas sp. GCF 041690465.1 WP 372623033.1
BacClassX Pseudomonadati Pseudomonadota Alphaproteobacteria Acetobacterales Falsiroseomonas oryziterrae WP 237213297.1
BacClassX Pseudomonadati Pseudomonadota Alphaproteobacteria Acetobacterales Roseomonas sp. E05 WP 283527232.1
BacClassX Pseudomonadati Pseudomonadota Alphaproteobacteria Acetobacterales Crenalkalicoccus roseus WP 135467997.1
BacClassX Pseudomonadati Pseudomonadota Alphaproteobacteria Acetobacterales Elioraea tepidiphila WP 019013034.1
BacClassX Pseudomonadati Pseudomonadota Alphaproteobacteria Acetobacterales Acetobacteraceae bacterium isolate HOG26 MBX6374330.1
BacClassX Pseudomonadati Pseudomonadota Alphaproteobacteria Acetobacterales Caldovatus sediminis WP 188902764.1
BacClassX Pseudomonadati Pseudomonadota Alphaproteobacteria Hyphomicrobiales Propylenella binzhouense WP 161140674.1
BacClassX Pseudomonadati Pseudomonadota Alphaproteobacteria Hyphomicrobiales Faunimonas sp. B44 WP 445467000.1
BacClassX Pseudomonadati Pseudomonadota Alphaproteobacteria Acetobacterales Acetobacteraceae bacterium isolate Prado186 MCU0983424.1
BacClassX Pseudomonadati Pseudomonadota Alphaproteobacteria Hyphomicrobiales Hyphomicrobiales bacterium isolate MAG.284 MCG8445304.1
BacClassX Pseudomonadati Thermodesulfobacteriota Desulfovibrionia Desulfovibrionales Desulfovibrionales bacterium isolate D1M04 10 iso2 MFP4316361.1
BacClassX Pseudomonadati Pseudomonadota Alphaproteobacteria Hyphomicrobiales Hyphomonas sp. GCF 039943795.1 WP 348211786.1
BacClassX Pseudomonadati Pseudomonadota Alphaproteobacteria Rhodobacterales Albidovulum sp. GCF 041919395.1 WP 301919533.1
BacClassX Pseudomonadati Pseudomonadota Alphaproteobacteria Rhodobacterales Albidovulum aquaemixtae WP 108853205.1
BacClassX Pseudomonadati Pseudomonadota Alphaproteobacteria Rhodobacterales Palleronia sp. KMU 117 WP 431829421.1
BacClassX Pseudomonadati Pseudomonadota Alphaproteobacteria Rhodobacterales Actibacterium sp. MT2.3 13A WP 212523392.1
BacClassX Pseudomonadati Pseudomonadota Alphaproteobacteria Rhodobacterales Sulfitobacter sabulilitoris WP 246039953.1
BacClassX Pseudomonadati Pseudomonadota Alphaproteobacteria Rhodobacterales Antarcticimicrobium sp. GCF 029212575.1 WP 300239201.1
BacClassX Pseudomonadati Pseudomonadota Alphaproteobacteria Rhodobacterales Roseovarius salis WP 397543898.1
BacClassX Pseudomonadati Pseudomonadota Alphaproteobacteria Rhodobacterales Tateyamaria pelophila WP 223425409.1
BacClassX Pseudomonadati Pseudomonadota Alphaproteobacteria Rhodobacterales Ruegeria aquimaris WP 263829168.1
BacClassX Pseudomonadati Pseudomonadota Alphaproteobacteria Hyphomicrobiales Parvibaculum sp. GCF 039680535.1 WP 346741677.1
BacClassX Pseudomonadati Pseudomonadota Alphaproteobacteria Hyphomicrobiales Parvibaculum sedimenti WP 152216541.1
BacClassX Pseudomonadati Pseudomonadota Alphaproteobacteria Hyphomicrobiales Parvibaculum sp. GCF 035325945.1 WP 324298677.1
BacClassX Pseudomonadati Pseudomonadota Alphaproteobacteria Hyphomicrobiales Methylocapsa polymorpha WP 407339165.1
BacClassX Pseudomonadati Pseudomonadota Alphaproteobacteria Hyphomicrobiales Methylocapsa acidiphila WP 026608157.1
BacClassX Pseudomonadati Pseudomonadota Alphaproteobacteria Hyphomicrobiales Aestuariivirga sp. isolate MFD01576 ilm asm bin7 MGB9143357.1
BacClassX Pseudomonadati Pseudomonadota Alphaproteobacteria Hyphomicrobiales Aestuariivirga sp. GCF 046294715.1 WP 409761267.1
BacClassX Pseudomonadati Pseudomonadota Alphaproteobacteria Hyphomicrobiales Methyloceanibacter sp. GCF 049589615.1 WP 424042709.1
BacClassX Pseudomonadati Pseudomonadota Alphaproteobacteria Hyphomicrobiales Methyloceanibacter sp. isolate MFD06091 ilm asm bin12 MGC2409639.1
BacClassX Pseudomonadati Pseudomonadota Alphaproteobacteria Hyphomicrobiales Methyloceanibacter sp. isolate 5 8 m bin2 MBK5198603.1
BacClassX Pseudomonadati Pseudomonadota Alphaproteobacteria Hyphomicrobiales Lutibaculum baratangense WP 040485795.1
BacClassX Pseudomonadati Pseudomonadota Gammaproteobacteria Arenicellales Arenicellales bacterium isolate MFD09702 ilm asm bin17 MGA8259143.1
BacClassX Pseudomonadati Pseudomonadota Alphaproteobacteria Hyphomicrobiales Hyphomicrobiales bacterium isolate C24 metabat.bin.120 sub iso2 MDP2619399.1
BacClassX Pseudomonadati Pseudomonadota Gammaproteobacteria Oceanospirillales Halomonas kalidii WP 282722674.1
BacClassX Pseudomonadati Pseudomonadota Gammaproteobacteria Chromatiales Halorhodospira neutriphila WP 200259225.1
BacClassX Pseudomonadati Pseudomonadota Gammaproteobacteria Chromatiales Alkalilimnicola ehrlichii strain SGAR12 WP 421621164.1
BacClassX Pseudomonadati Pseudomonadota Gammaproteobacteria Oceanospirillales Halomonas sp. BC04 WP 197030603.1
BacClassX Pseudomonadati Pseudomonadota Gammaproteobacteria Oceanospirillales Halomonas sp. GCF 003550545.1 WP 290790528.1
BacClassX Pseudomonadati Pseudomonadota Gammaproteobacteria Oceanospirillales Billgrantia campisalis WP 238978372.1
BacClassX Pseudomonadati Pseudomonadota Gammaproteobacteria Oceanospirillales Halomonas lysinitropha WP 192576470.1
BacClassX Pseudomonadati Pseudomonadota Gammaproteobacteria Oceanospirillales Halomonas fontilapidosi WP 179862064.1
BacClassX Pseudomonadati Pseudomonadota Gammaproteobacteria Salinisphaerales Salinisphaera sp. SPP AMP 43 WP 423822281.1
BacClassX Pseudomonadati Pseudomonadota Alphaproteobacteria Rhodospirillales Tistlia sp. GCF 040905785.1 WP 368427009.1
BacClassX Pseudomonadati Pseudomonadota Alphaproteobacteria Rhodobacterales Roseobacter insulae WP 219498913.1
BacClassX Pseudomonadati Pseudomonadota Alphaproteobacteria Rhodobacterales Jannaschia faecimaris WP 092646374.1
BacClassX Pseudomonadati Pseudomonadota Alphaproteobacteria Rhodobacterales Boseongicola aestuarii WP 093975981.1
BacClassX Pseudomonadati Pseudomonadota Alphaproteobacteria Rhodobacterales Roseobacter sp. GAI101 WP 008229866.1
BacClassX Pseudomonadati Pseudomonadota Alphaproteobacteria Rhodobacterales Sulfitobacter sp. GCF 041552635.1 WP 372993600.1
BacClassX Pseudomonadati Pseudomonadota Alphaproteobacteria Rhodobacterales Yoonia sp. GCF 017854415.1 WP 296433176.1
BacClassX Pseudomonadati Pseudomonadota Betaproteobacteria Burkholderiales Rubrivivax sp. RP6 9 WP 425261989.1
BacClassX Pseudomonadati Pseudomonadota Betaproteobacteria Burkholderiales uncultured Piscinibacter sp. GCF 937952055.1 WP 298830119.1
BacClassX Pseudomonadati Pseudomonadota Betaproteobacteria Burkholderiales Comamonadaceae bacterium isolate AER3 1 MBH2043720.1
BacClassX Pseudomonadati Pseudomonadota Betaproteobacteria Burkholderiales Hydrogenophaga sp. Root209 WP 056272583.1
BacClassX Pseudomonadati Pseudomonadota Betaproteobacteria Burkholderiales Hydrogenophaga sp. PBL H3 WP 159603343.1
BacClassX Pseudomonadati Pseudomonadota Betaproteobacteria Burkholderiales Rubrivivax sp. isolate C15 concoct.bin.32 sub MDP1647219.1
BacClassX Pseudomonadati Pseudomonadota Betaproteobacteria Burkholderiales Roseateles oligotrophus strain CHU3 WP 263573890.1
BacClassX Pseudomonadati Pseudomonadota Betaproteobacteria Burkholderiales Variovorax sp. RA8 WP 162586157.1
BacClassX Pseudomonadati Pseudomonadota Betaproteobacteria Burkholderiales Variovorax paradoxus strain B4 WP 230559097.1
BacClassX Pseudomonadati Pseudomonadota Betaproteobacteria Burkholderiales Azohydromonas caseinilytica WP 169163093.1
BacClassX Pseudomonadati Pseudomonadota Betaproteobacteria Burkholderiales Azohydromonas australica WP 029001406.1
BacClassX Pseudomonadati Pseudomonadota Betaproteobacteria Burkholderiales Caldimonas sp. KR1 144 WP 423284323.1
BacClassX Pseudomonadati Pseudomonadota Betaproteobacteria Burkholderiales Ideonella sp. GCF 036277275.1 WP 329719511.1
BacClassX Pseudomonadati Pseudomonadota Betaproteobacteria Burkholderiales Aquabacterium humicola WP 284614774.1
BacClassX Pseudomonadati Pseudomonadota Betaproteobacteria Burkholderiales Rhodoferax sp. GCF 030646845.1 WP 304746275.1
BacClassX Pseudomonadati Pseudomonadota Betaproteobacteria Burkholderiales Polaromonas sp. GCF 044349845.1 WP 397409270.1
BacClassX Pseudomonadati Pseudomonadota Betaproteobacteria Burkholderiales Polaromonas sp. GCF 030682935.1 WP 305246652.1
BacClassX Pseudomonadati Pseudomonadota Betaproteobacteria Burkholderiales Polaromonas sp. GCF 018780625.1 WP 293466236.1
BacClassX Pseudomonadati Pseudomonadota Betaproteobacteria Burkholderiales Polaromonas sp. UBA4122 WP 293604154.1
BacClassX Pseudomonadati Pseudomonadota Betaproteobacteria Burkholderiales Rhodoferax ferrireducens GCF 003347515.1 WP 114968681.1
BacClassX Pseudomonadati Pseudomonadota Betaproteobacteria Burkholderiales Rhodoferax sp. isolate C12 concoct.bin.166 sub MDP1998334.1
BacClassX Pseudomonadati Pseudomonadota Betaproteobacteria Burkholderiales Rhodoferax sp. GCF 049657905.1 WP 424511631.1
BacClassX Pseudomonadati Pseudomonadota Betaproteobacteria Burkholderiales Rhodoferax sp. GCF 018780565.1 WP 296692011.1
BacClassX Pseudomonadati Pseudomonadota Betaproteobacteria Burkholderiales Polaromonas sp. isolate MFD12019 ilm asm bin54 MFZ2388337.1
BacClassX Pseudomonadati Pseudomonadota Betaproteobacteria Burkholderiales Rhodoferax sp. GCF 012927505.1 WP 296497371.1
BacClassX Pseudomonadati Pseudomonadota Betaproteobacteria Burkholderiales Undibacterium sp. GCF 030651495.1 WP 304769282.1
BacClassX Pseudomonadati Pseudomonadota Betaproteobacteria Burkholderiales Rhodoferax sp. GCF 013791765.1 WP 296527826.1
BacClassX Pseudomonadati Pseudomonadota Betaproteobacteria Burkholderiales Rhodoferax sp. GCF 010365055.1 WP 296482643.1
BacClassX Pseudomonadati Pseudomonadota Betaproteobacteria Burkholderiales Rhodoferax ferrireducens strain DSM 15236 WP 011462719.1
BacClassIA CPR Gracilibacteria Candidatus Altimarinota bacterium isolate SO 2017 LW3 148 MFA5351925.1 318 aa
BacClassIA CPR unclassified Patescibacteria Patescibacteria bacterium isolate MEGAHIT 1.831 MFH1375744.1 316 aa
BacClassIA CPR Gracilibacteria Candidatus Altimarinota bacterium isolate RS 3 32 MCK5471942.1 317 aa
BacClassIA CPR unclassified Patescibacteria Patescibacteria bacterium isolate MEGAHIT 0.717 iso2 MFH0834605.1 289 aa
BacClassIA CPR Peregrinbacteria Candidatus Peribacteraceae bacterium isolate Tobar13.5m G77 MCF7846344.1 318 aa
BacClassIA CPR unclassified Patescibacteria Patescibacteria bacterium isolate SO 2016 GW1 31 MFA6457895.1 318 aa
BacClassIA CPR unclassified Patescibacteria Patescibacteria bacterium isolate MEGAHIT 0.717 iso1 MFH0834261.1 318 aa

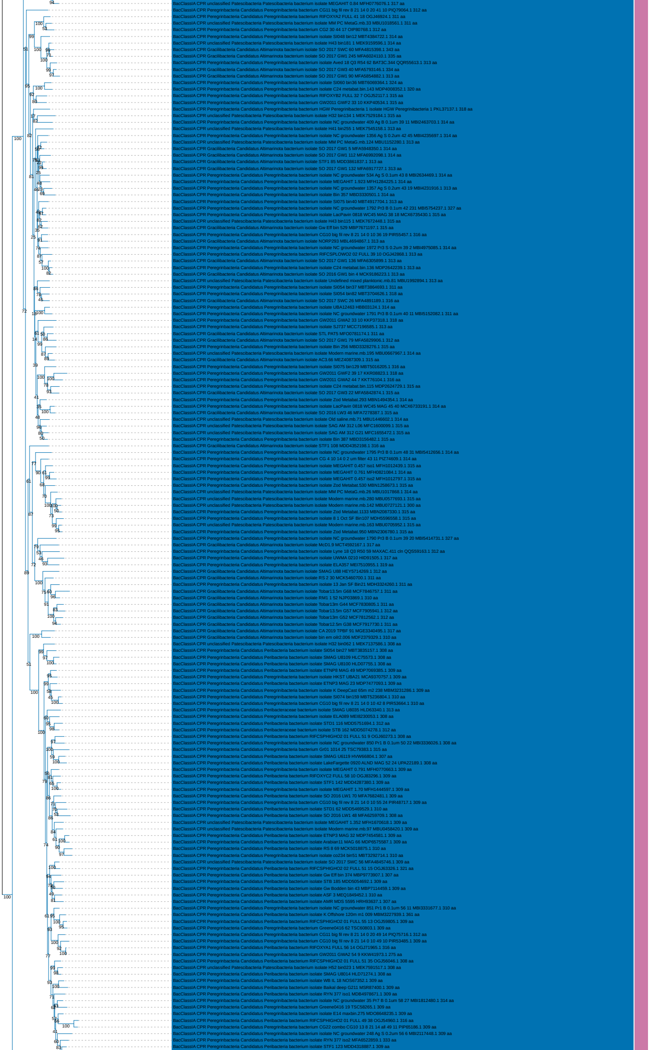

BacClassIA CPR unclassified Patescibacteria Patescibacteria bacterium isolate MEGAHIT 0.84 MFH0776076.1 317 aa
BacClassIA CPR Peregrinibacteria Candidatus Peregrinibacteria bacterium CG11 big fil rev 8 21 14 0 20 41 10 PIQ79064.1 312 aa
BacClassIA CPR Peregrinibacteria Candidatus Peregrinibacteria bacterium RIFOXYA2 FULL 41 18 OGJ46924.1 311 aa
BacClassIA CPR unclassified Patescibacteria Patescibacteria bacterium isolate MM PC MetaG.mb.33 MBU1018561.1 311 aa
BacClassIA CPR Peregrinibacteria Candidatus Peregrinibacteria bacterium CG2 30 44 17 OIP80768.1 312 aa
BacClassIA CPR Peregrinibacteria Candidatus Peregrinibacteria bacterium isolate SI048 bin12 MBT4384722.1 314 aa
BacClassIA CPR unclassified Patescibacteria Patescibacteria bacterium isolate H43 bin181 1 MEK9159596.1 314 aa
BacClassIA CPR Gracilibacteria Candidatus Altimarinota bacterium isolate SO 2017 SWC 60 MFA4815398.1 343 aa
BacClassIA CPR Gracilibacteria Candidatus Altimarinota bacterium isolate SO 2017 GW1 245 MFA6024110.1 335 aa
BacClassIA CPR Peregrinibacteria Candidatus Peregrinibacteria bacterium isolate Aved 18 Q3 R54 62 BAT3C.344 QQR55613.1 313 aa
BacClassIA CPR Gracilibacteria Candidatus Altimarinota bacterium isolate SO 2017 GW3 40 MFA5793146.1 334 aa
BacClassIA CPR Gracilibacteria Candidatus Altimarinota bacterium isolate SO 2017 GW1 90 MFA5854882.1 313 aa
BacClassIA CPR Peregrinibacteria Candidatus Peregrinibacteria bacterium isolate SI060 bin36 MBT6069364.1 324 aa
BacClassIA CPR Peregrinibacteria Candidatus Peregrinibacteria bacterium isolate C24 metabat.bin.143 MDP4008352.1 320 aa
BacClassIA CPR Peregrinibacteria Candidatus Peregrinibacteria bacterium RIFOXYB2 FULL 32 7 OGJ52117.1 315 aa
BacClassIA CPR Peregrinibacteria Candidatus Peregrinibacteria bacterium GW2011 GWF2 33 10 KKP40534.1 315 aa
BacClassIA CPR Peregrinibacteria Candidatus Peregrinibacteria bacterium HGW Peregrinibacteria 1 isolate HGW Peregrinibacteria 1 PKL37137.1 318 aa
BacClassIA CPR unclassified Patescibacteria Patescibacteria bacterium isolate H32 bin134 1 MEK7529184.1 315 aa
BacClassIA CPR Peregrinibacteria Candidatus Peregrinibacteria bacterium isolate NC groundwater 409 Ag B 0.1um 39 11 MBI2463703.1 314 aa
BacClassIA CPR unclassified Patescibacteria Patescibacteria bacterium isolate H41 bin255 1 MEK7545158.1 313 aa
BacClassIA CPR Peregrinibacteria Candidatus Peregrinibacteria bacterium isolate NC groundwater 1356 Ag S 0.2um 42 45 MBI4235697.1 314 aa
BacClassIA CPR unclassified Patescibacteria Patescibacteria bacterium isolate MM PC MetaG.mb.124 MBU1152280.1 313 aa
BacClassIA CPR Gracilibacteria Candidatus Altimarinota bacterium isolate SO 2017 GW1 5 MFA5948350.1 314 aa
BacClassIA CPR Gracilibacteria Candidatus Altimarinota bacterium isolate SO 2017 GW1 112 MFA6992098.1 314 aa
BacClassIA CPR Gracilibacteria Candidatus Altimarinota bacterium isolate STF1 85 MDD3861837.1 313 aa
BacClassIA CPR Gracilibacteria Candidatus Altimarinota bacterium isolate SO 2017 GW1 132 MFA6917727.1 313 aa
BacClassIA CPR Peregrinibacteria Candidatus Peregrinibacteria bacterium isolate NC groundwater 534 Ag S 0.1um 43 8 MBI2634469.1 314 aa
BacClassIA CPR Peregrinibacteria Candidatus Peregrinibacteria bacterium isolate MEGAHIT 1.923 MFH1284225.1 314 aa
BacClassIA CPR Peregrinibacteria Candidatus Peregrinibacteria bacterium isolate NC groundwater 1357 Ag S 0.2um 43 19 MBI4231916.1 313 aa
BacClassIA CPR Peregrinibacteria Candidatus Peregrinibacteria bacterium isolate Bin 357 MBD3330501.1 314 aa
BacClassIA CPR Peregrinibacteria Candidatus Peregrinibacteria bacterium isolate SI075 bin40 MBT4917704.1 313 aa
BacClassIA CPR Peregrinibacteria Candidatus Peregrinibacteria bacterium isolate NC groundwater 1792 Pr3 B 0.1um 42 231 MBI5754237.1 327 aa
BacClassIA CPR Peregrinibacteria Candidatus Peregrinibacteria bacterium isolate LacPavin 0818 WC45 MAG 38 18 MCX6735430.1 315 aa
BacClassIA CPR unclassified Patescibacteria Patescibacteria bacterium isolate H43 bin115 1 MEK7672448.1 315 aa
BacClassIA CPR Gracilibacteria Candidatus Altimarinota bacterium isolate Gw Eff bin 529 MBP7671197.1 315 aa
BacClassIA CPR Peregrinibacteria Candidatus Peregrinibacteria bacterium CG10 big fil rev 8 21 14 0 10 36 19 PIR55457.1 316 aa
BacClassIA CPR Gracilibacteria Candidatus Altimarinota bacterium isolate NORP293 MBL4694867.1 313 aa
BacClassIA CPR Peregrinibacteria Candidatus Peregrinibacteria bacterium isolate NC groundwater 1972 Pr3 S 0.2um 39 2 MBI4975085.1 314 aa
BacClassIA CPR Peregrinibacteria Candidatus Peregrinibacteria bacterium RIFCSPLOWO2 02 FULL 39 10 OGJ42868.1 313 aa
BacClassIA CPR Gracilibacteria Candidatus Altimarinota bacterium isolate SO 2017 GW1 136 MFA6305899.1 313 aa
BacClassIA CPR Peregrinibacteria Candidatus Peregrinibacteria bacterium isolate C24 metabat.bin.136 MDP2642239.1 313 aa
BacClassIA CPR Gracilibacteria Candidatus Altimarinota bacterium isolate SO 2016 GW1 bin 4 MCK9186223.1 313 aa
BacClassIA CPR unclassified Patescibacteria Patescibacteria bacterium isolate Undefined mixed planktonic.mb.81 MBU1992894.1 313 aa
BacClassIA CPR Peregrinibacteria Candidatus Peregrinibacteria bacterium isolate SI054 bin37 MBT3864693.1 311 aa
BacClassIA CPR Peregrinibacteria Candidatus Peregrinibacteria bacterium isolate SI054 bin82 MBT3704626.1 318 aa
BacClassIA CPR Gracilibacteria Candidatus Altimarinota bacterium isolate SO 2017 SWC 26 MFA4891189.1 316 aa
BacClassIA CPR Peregrinibacteria Candidatus Peregrinibacteria bacterium isolate UBA12463 HBB03124.1 314 aa
BacClassIA CPR Peregrinibacteria Candidatus Peregrinibacteria bacterium isolate NC groundwater 1791 Pr3 B 0.1um 40 11 MBI5152082.1 311 aa
BacClassIA CPR Peregrinibacteria Candidatus Peregrinibacteria bacterium GW2011 GWA2 33 10 KKP37318.1 318 aa
BacClassIA CPR Peregrinibacteria Candidatus Peregrinibacteria bacterium isolate SJ737 MCC7196585.1 313 aa
BacClassIA CPR Gracilibacteria Candidatus Altimarinota bacterium isolate STL PAT5 MFO0781174.1 311 aa
BacClassIA CPR Gracilibacteria Candidatus Altimarinota bacterium isolate SO 2017 GW1 79 MFA5829906.1 312 aa
BacClassIA CPR Peregrinibacteria Candidatus Peregrinibacteria bacterium isolate Bin 256 MBD3328276.1 315 aa
BacClassIA CPR unclassified Patescibacteria Patescibacteria bacterium isolate Modern marine.mb.195 MBU0667967.1 314 aa
BacClassIA CPR Gracilibacteria Candidatus Altimarinota bacterium isolate AC3.66 MEZ4087309.1 315 aa
BacClassIA CPR Peregrinibacteria Candidatus Peregrinibacteria bacterium isolate SI075 bin129 MBT5016205.1 316 aa
BacClassIA CPR Peregrinibacteria Candidatus Peregrinibacteria bacterium GW2011 GWF2 39 17 KKR08823.1 318 aa
BacClassIA CPR Peregrinibacteria Candidatus Peregrinibacteria bacterium GW2011 GWA2 44 7 KKT76104.1 316 aa
BacClassIA CPR Peregrinibacteria Candidatus Peregrinibacteria bacterium isolate C24 metabat.bin.115 MDP2624729.1 315 aa
BacClassIA CPR Gracilibacteria Candidatus Altimarinota bacterium isolate SO 2017 GW3 22 MFA5842874.1 315 aa
BacClassIA CPR Peregrinibacteria Candidatus Peregrinibacteria bacterium isolate Zod Metabat.293 MBN1494354.1 314 aa
BacClassIA CPR Peregrinibacteria Candidatus Peregrinibacteria bacterium isolate LacPavin 0818 WC45 MAG 45 40 MCX6733191.1 314 aa
BacClassIA CPR Gracilibacteria Candidatus Altimarinota bacterium isolate SO 2016 LW3 46 MFA7278387.1 315 aa
BacClassIA CPR unclassified Patescibacteria Patescibacteria bacterium isolate Old saline.mb.71 MBU1446602.1 314 aa
BacClassIA CPR unclassified Patescibacteria Patescibacteria bacterium isolate SAG AM 312 L06 MFC1600099.1 315 aa
BacClassIA CPR unclassified Patescibacteria Patescibacteria bacterium isolate SAG AM 312 G21 MFC1655472.1 315 aa
BacClassIA CPR Peregrinibacteria Candidatus Peregrinibacteria bacterium isolate Bin 387 MBD3156482.1 315 aa
BacClassIA CPR Gracilibacteria Candidatus Altimarinota bacterium isolate STF1 108 MDD4352198.1 316 aa
BacClassIA CPR Peregrinibacteria Candidatus Peregrinibacteria bacterium isolate NC groundwater 1795 Pr3 B 0.1um 48 31 MBI5412656.1 314 aa
BacClassIA CPR Peregrinibacteria Candidatus Peregrinibacteria bacterium CG 4 10 14 0 2 um filter 43 11 PIZ74609.1 314 aa
BacClassIA CPR Peregrinibacteria Candidatus Peregrinibacteria bacterium isolate MEGAHIT 0.457 iso1 MFH1012439.1 315 aa
BacClassIA CPR Peregrinibacteria Candidatus Peregrinibacteria bacterium isolate MEGAHIT 0.761 MFH0821084.1 314 aa
BacClassIA CPR Peregrinibacteria Candidatus Peregrinibacteria bacterium isolate MEGAHIT 0.457 iso2 MFH1012797.1 315 aa
BacClassIA CPR Peregrinibacteria Candidatus Peregrinibacteria bacterium isolate Zod Metabat.530 MBN1258673.1 315 aa
BacClassIA CPR unclassified Patescibacteria Patescibacteria bacterium isolate MM PC MetaG.mb.26 MBU1017868.1 314 aa
BacClassIA CPR unclassified Patescibacteria Patescibacteria bacterium isolate Modern marine.mb.280 MBU0577693.1 315 aa
BacClassIA CPR unclassified Patescibacteria Patescibacteria bacterium isolate Modern marine.mb.142 MBU0727121.1 300 aa
BacClassIA CPR Peregrinibacteria Candidatus Peregrinibacteria bacterium isolate Zod Metabat.1133 MBN2087330.1 315 aa
BacClassIA CPR Peregrinibacteria Candidatus Peregrinibacteria bacterium isolate 8 1 Oct SF Bin107 MDH5596558.1 315 aa
BacClassIA CPR unclassified Patescibacteria Patescibacteria bacterium isolate Modern marine.mb.163 MBU0705952.1 315 aa
BacClassIA CPR Peregrinibacteria Candidatus Peregrinibacteria bacterium isolate Zod Metabat.950 MBN2306780.1 315 aa
BacClassIA CPR Peregrinibacteria Candidatus Peregrinibacteria bacterium isolate NC groundwater 1790 Pr3 B 0.1um 39 20 MBI5414731.1 327 aa
BacClassIA CPR Gracilibacteria Candidatus Altimarinota bacterium isolate McD1 9 MCT4592167.1 317 aa
BacClassIA CPR Peregrinibacteria Candidatus Peregrinibacteria bacterium isolate Lyne 18 Q3 R50 59 MAXAC.411 cln QQS59163.1 312 aa
BacClassIA CPR Peregrinibacteria Candidatus Peregrinibacteria bacterium isolate UWMA 0210 HID91505.1 317 aa
BacClassIA CPR Peregrinibacteria Candidatus Peregrinibacteria bacterium isolate ELA357 MEI7510955.1 319 aa
BacClassIA CPR Gracilibacteria Candidatus Altimarinota bacterium isolate SMAG U88 HEY5714269.1 312 aa
BacClassIA CPR Gracilibacteria Candidatus Altimarinota bacterium isolate RS 2 30 MCK5460700.1 311 aa
BacClassIA CPR Peregrinibacteria Candidatus Peregrinibacteria bacterium isolate 13 Jan SF Bin21 MDH3324260.1 311 aa
BacClassIA CPR Gracilibacteria Candidatus Altimarinota bacterium isolate Tobar13.5m G68 MCF7846757.1 311 aa
BacClassIA CPR Gracilibacteria Candidatus Altimarinota bacterium isolate RM1 1 52 NJP03869.1 310 aa
BacClassIA CPR Gracilibacteria Candidatus Altimarinota bacterium isolate Tobar13m G44 MCF7830805.1 311 aa
BacClassIA CPR Gracilibacteria Candidatus Altimarinota bacterium isolate Tobar13.5m G57 MCF7905941.1 312 aa
BacClassIA CPR Gracilibacteria Candidatus Altimarinota bacterium isolate Tobar13m G52 MCF7812562.1 312 aa
BacClassIA CPR Gracilibacteria Candidatus Altimarinota bacterium isolate Tobar12.5m G38 MCF7917730.1 311 aa
BacClassIA CPR Gracilibacteria Candidatus Altimarinota bacterium isolate CA 2019 TPBF 91 MGE3340495.1 317 aa
BacClassIA CPR Gracilibacteria Candidatus Altimarinota bacterium isolate bin em oilr2.006 MDF2379329.1 310 aa
BacClassIA CPR unclassified Patescibacteria Patescibacteria bacterium isolate H32 bin062 1 MEK7137586.1 308 aa
BacClassIA CPR Peregrinibacteria Candidatus Peregrinibacteria bacterium isolate SI054 bin27 MBT3835157.1 308 aa
BacClassIA CPR Peregrinibacteria Candidatus Peribacteria bacterium isolate SMAG U8109 HLC75573.1 308 aa
BacClassIA CPR Peregrinibacteria Candidatus Peribacteria bacterium isolate SMAG U8100 HLD07755.1 308 aa
BacClassIA CPR Peregrinibacteria Candidatus Peribacteria bacterium isolate ETNP8 MAG 49 MDP7069385.1 309 aa
BacClassIA CPR Peregrinibacteria Candidatus Peribacteria bacterium isolate HKST UBA21 MCA9370757.1 309 aa
BacClassIA CPR Peregrinibacteria Candidatus Peribacteria bacterium isolate ETNP3 MAG 23 MDP7477093.1 309 aa
BacClassIA CPR Peregrinibacteria Candidatus Peregrinibacteria bacterium isolate K DeepCast 65m m2 238 MBM3231286.1 309 aa
BacClassIA CPR Peregrinibacteria Candidatus Peregrinibacteria bacterium isolate SI074 bin159 MBT5236804.1 310 aa
BacClassIA CPR Peregrinibacteria Candidatus Peregrinibacteria bacterium CG10 big fil rev 8 21 14 0 10 42 8 PIR53664.1 310 aa
BacClassIA CPR Peregrinibacteria Candidatus Peribacteraceae bacterium isolate SMAG U8035 HLD63340.1 313 aa
BacClassIA CPR Peregrinibacteria Candidatus Peregrinibacteria bacterium isolate ELA089 MEI8230053.1 308 aa
BacClassIA CPR Peregrinibacteria Candidatus Peribacteria bacterium isolate STD1 116 MDD5751694.1 312 aa
BacClassIA CPR Peregrinibacteria Candidatus Peribacteraceae bacterium isolate STB 162 MDD5074278.1 312 aa
BacClassIA CPR Peregrinibacteria Candidatus Peribacteria bacterium RIFCSPHIGHO2 01 FULL 51 9 OGJ60273.1 308 aa
BacClassIA CPR Peregrinibacteria Candidatus Peregrinibacteria bacterium isolate NC groundwater 850 Pr1 B 0.1um 50 22 MBI3336026.1 308 aa
BacClassIA CPR Peregrinibacteria Candidatus Peregrinibacteria bacterium Gr01 1014 25 TSC79383.1 315 aa
BacClassIA CPR Peregrinibacteria Candidatus Peribacteria bacterium isolate SMAG U6119 HVW66804.1 307 aa
BacClassIA CPR Peregrinibacteria Candidatus Peribacteria bacterium isolate LakeFargette 0920 ALND MAG 52 24 UPA22189.1 308 aa
BacClassIA CPR Peregrinibacteria Candidatus Peregrinibacteria bacterium isolate MEGAHIT 0.791 MFH0770663.1 309 aa
BacClassIA CPR Peregrinibacteria Candidatus Peribacteria bacterium RIFOXYC2 FULL 58 10 OGJ83296.1 309 aa
BacClassIA CPR Peregrinibacteria Candidatus Peribacteria bacterium isolate STF1 142 MDD4287380.1 309 aa
BacClassIA CPR Peregrinibacteria Candidatus Peregrinibacteria bacterium isolate MEGAHIT 1.70 MFH1444597.1 309 aa
BacClassIA CPR Peregrinibacteria Candidatus Peribacteria bacterium isolate SO 2016 LW1 70 MFA7682481.1 309 aa
BacClassIA CPR Peregrinibacteria Candidatus Peregrinibacteria bacterium CG10 big fil rev 8 21 14 0 10 55 24 PIR48717.1 309 aa
BacClassIA CPR Peregrinibacteria Candidatus Peribacteria bacterium isolate STD1 62 MDD5469529.1 310 aa
BacClassIA CPR Peregrinibacteria Candidatus Peribacteria bacterium isolate SO 2016 LW1 48 MFA6259709.1 308 aa
BacClassIA CPR unclassified Patescibacteria Patescibacteria bacterium isolate MEGAHIT 1.352 MFH1670618.1 309 aa
BacClassIA CPR unclassified Patescibacteria Patescibacteria bacterium isolate Modern marine.mb.97 MBU0458420.1 309 aa
BacClassIA CPR Peregrinibacteria Candidatus Peribacteria bacterium isolate ETNP3 MAG 32 MDP7454581.1 309 aa
BacClassIA CPR Peregrinibacteria Candidatus Peribacteria bacterium isolate Arabian11 MAG 66 MDP6575587.1 309 aa
BacClassIA CPR Peregrinibacteria Candidatus Peribacteria bacterium isolate RS 8 69 MCK5018875.1 310 aa
BacClassIA CPR Peregrinibacteria Candidatus Peregrinibacteria bacterium isolate co234 bin51 MBT3292714.1 310 aa
BacClassIA CPR unclassified Patescibacteria Patescibacteria bacterium isolate SO 2017 SWC 56 MFA4845746.1 309 aa
BacClassIA CPR Peregrinibacteria Candidatus Peregrinibacteria bacterium RIFCSPHIGHO2 02 FULL 51 15 OGJ63326.1 321 aa
BacClassIA CPR Peregrinibacteria Candidatus Peribacteria bacterium isolate Gw Eff bin 374 MBP9773907.1 307 aa
BacClassIA CPR Peregrinibacteria Candidatus Peribacteria bacterium isolate STB 185 MDD5054692.1 309 aa
BacClassIA CPR Peregrinibacteria Candidatus Peribacteria bacterium isolate Gw Bodden bin 43 MBP7114459.1 309 aa
BacClassIA CPR Peregrinibacteria Candidatus Peribacteria bacterium isolate ASF 3 MEQ1849452.1 310 aa
BacClassIA CPR Peregrinibacteria Candidatus Peribacteria bacterium isolate AMR MDS 5595 HRH93637.1 307 aa
BacClassIA CPR Peregrinibacteria Candidatus Peregrinibacteria bacterium isolate NC groundwater 851 Pr1 B 0.1um 56 11 MBI3331677.1 310 aa
BacClassIA CPR Peregrinibacteria Candidatus Peregrinibacteria bacterium isolate K Offshore 120m m1 009 MBM3227939.1 361 aa
BacClassIA CPR Peregrinibacteria Candidatus Peribacteria bacterium RIFCSPHIGHO2 01 FULL 55 13 OGJ59805.1 309 aa
BacClassIA CPR Peregrinibacteria Candidatus Peribacteria bacterium Greene0416 62 TSC60803.1 309 aa
BacClassIA CPR Peregrinibacteria Candidatus Peregrinibacteria bacterium CG11 big fil rev 8 21 14 0 20 49 14 PIQ75716.1 312 aa
BacClassIA CPR Peregrinibacteria Candidatus Peregrinibacteria bacterium CG10 big fil rev 8 21 14 0 10 49 10 PIR53485.1 309 aa
BacClassIA CPR Peregrinibacteria Candidatus Peribacteria bacterium RIFOXYA1 FULL 56 14 OGJ71965.1 316 aa
BacClassIA CPR Peregrinibacteria Candidatus Peregrinibacteria bacterium GW2011 GWA2 54 9 KKW41973.1 275 aa
BacClassIA CPR Peregrinibacteria Candidatus Peribacteria bacterium RIFCSPHIGHO2 01 FULL 51 35 OGJ56046.1 308 aa
BacClassIA CPR unclassified Patescibacteria Patescibacteria bacterium isolate H52 bin023 1 MEK7591517.1 308 aa
BacClassIA CPR Peregrinibacteria Candidatus Peribacteria bacterium isolate SMAG U8014 HLD71274.1 308 aa
BacClassIA CPR Peregrinibacteria Candidatus Peribacteria bacterium isolate WB IL 18 NOS67352.1 309 aa
BacClassIA CPR Peregrinibacteria Candidatus Peribacteria bacterium isolate Baikal deep G211 MSR87400.1 309 aa
BacClassIA CPR Peregrinibacteria Candidatus Peribacteria bacterium isolate RYN 377 iso1 MDB4978671.1 309 aa
BacClassIA CPR Peregrinibacteria Candidatus Peregrinibacteria bacterium isolate NC groundwater 35 Pr7 B 0.1um 58 27 MBI1812480.1 314 aa
BacClassIA CPR Peregrinibacteria Candidatus Peribacteria bacterium Greene0416 19 TSC58265.1 309 aa
BacClassIA CPR Peregrinibacteria Candidatus Peribacteria bacterium isolate E14 maxbin.275 MDO8648235.1 309 aa
BacClassIA CPR Peregrinibacteria Candidatus Peribacteria bacterium RIFCSPHIGHO2 01 FULL 49 38 OGJ54960.1 316 aa
BacClassIA CPR Peregrinibacteria Candidatus Peregrinibacteria bacterium CG22 combo CG10 13 8 21 14 all 49 11 PIP65186.1 309 aa
BacClassIA CPR Peregrinibacteria Candidatus Peregrinibacteria bacterium isolate NC groundwater 248 Ag S 0.2um 56 6 MBI2117448.1 309 aa
BacClassIA CPR Peregrinibacteria Candidatus Peribacteria bacterium isolate RYN 377 iso2 MFA6522859.1 333 aa
BacClassIA CPR Peregrinibacteria Candidatus Peribacteria bacterium isolate STF1 123 MDD4318887.1 309 aa

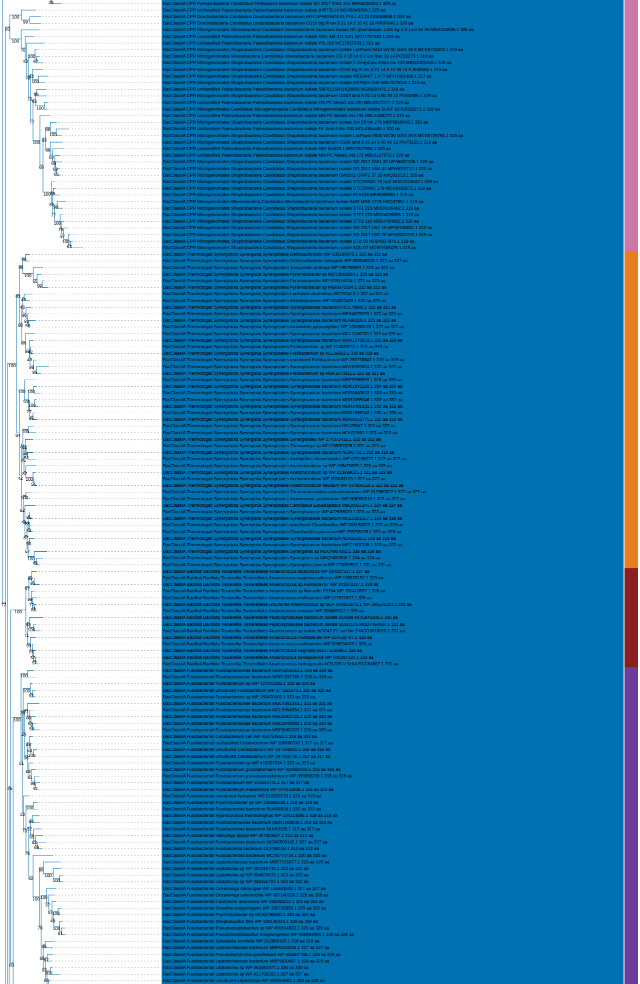

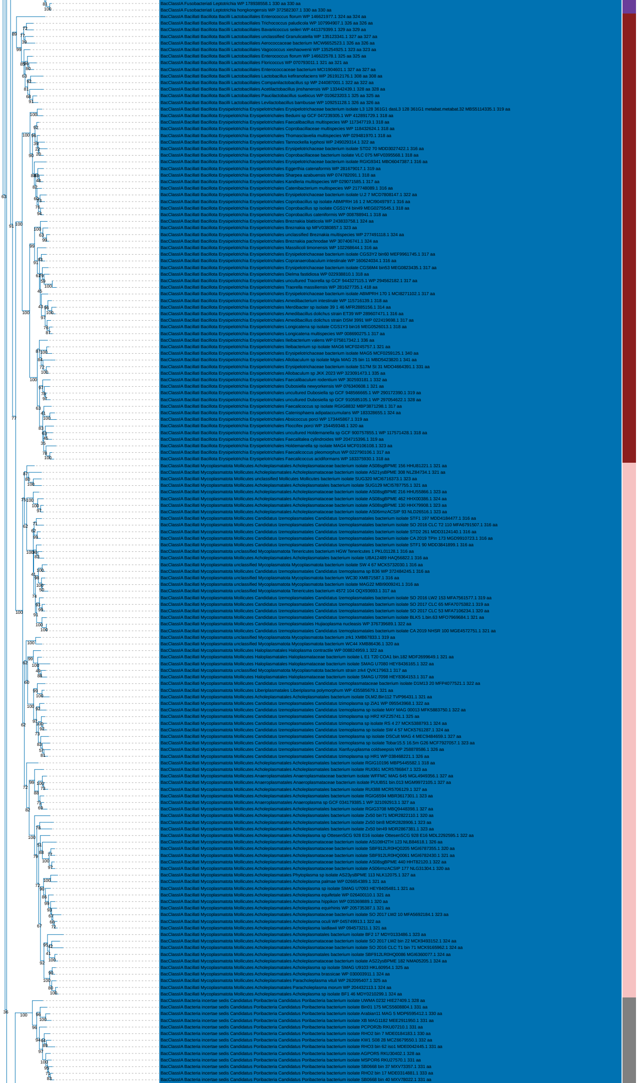
BacClassIA Fusobacteriati Leptotrichia WP 178938558.1 330 aa 330 aa
BacClassIA Fusobacteriati Leptotrichia hongkongensis WP 372582307.1 330 aa 330 aa
BacClassIA Bacillati Bacillota Bacilli Lactobacillales Enterococcus florum WP 146621977.1 324 aa 324 aa
BacClassIA Bacillati Bacillota Bacilli Lactobacillales Trichococcus paludicola WP 107994907.1 326 aa 326 aa
BacClassIA Bacillati Bacillota Bacilli Lactobacillales Bavariicoccus seileri WP 441379399.1 329 aa 329 aa
BacClassIA Bacillati Bacillota Bacilli Lactobacillales unclassified Granulicatella WP 135123341.1 327 aa 327 aa
BacClassIA Bacillati Bacillota Bacilli Lactobacillales Aerococcaceae bacterium MCW6652523.1 326 aa 326 aa
BacClassIA Bacillati Bacillota Bacilli Lactobacillales Vagococcus xieshaowenii WP 135254925.1 323 aa 323 aa
BacClassIA Bacillati Bacillota Bacilli Lactobacillales Enterococcus florum WP 146622578.1 325 aa 325 aa
BacClassIA Bacillati Bacillota Bacilli Lactobacillales Floricoccus WP 070793011.1 321 aa 321 aa
BacClassIA Bacillati Bacillota Bacilli Lactobacillales Enterococcaceae bacterium MCI1904601.1 327 aa 327 aa
BacClassIA Bacillati Bacillota Bacilli Lactobacillales Lactobacillus kefiranofaciens WP 261912176.1 308 aa 308 aa
BacClassIA Bacillati Bacillota Bacilli Lactobacillales Companilactobacillus sp WP 244087001.1 322 aa 322 aa
BacClassIA Bacillati Bacillota Bacilli Lactobacillales Acetilactobacillus jinshanensis WP 133442439.1 328 aa 328 aa
BacClassIA Bacillati Bacillota Bacilli Lactobacillales Paucilactobacillus suebicus WP 010623203.1 325 aa 325 aa
BacClassIA Bacillati Bacillota Bacilli Lactobacillales Levilactobacillus bambusae WP 109251128.1 326 aa 326 aa
BacClassIA Bacillati Bacillota Erysipelotrichia Erysipelotrichales Erysipelotrichaceae bacterium isolate L3 128 361G1 dasL3 128 361G1 metabat.metabat.32 MBS5114335.1 319 aa
BacClassIA Bacillati Bacillota Erysipelotrichia Erysipelotrichales Beduini sp GCF 047239305.1 WP 412891729.1 318 aa
BacClassIA Bacillati Bacillota Erysipelotrichia Erysipelotrichales Faecalibacillus multispecies WP 117347719.1 318 aa
BacClassIA Bacillati Bacillota Erysipelotrichia Erysipelotrichales Coprobacillaceae multispecies WP 118432624.1 318 aa
BacClassIA Bacillati Bacillota Erysipelotrichia Erysipelotrichales Thomasclavelia multispecies WP 029481970.1 318 aa
BacClassIA Bacillati Bacillota Erysipelotrichia Erysipelotrichales Tannockella kyphosi WP 249029314.1 322 aa
BacClassIA Bacillati Bacillota Erysipelotrichia Erysipelotrichales Erysipelotrichaceae bacterium isolate STD2 70 MDD3027422.1 316 aa
BacClassIA Bacillati Bacillota Erysipelotrichia Erysipelotrichales Coprobacillaceae bacterium isolate VLC 075 MFV0395568.1 318 aa
BacClassIA Bacillati Bacillota Erysipelotrichia Erysipelotrichales Erysipelotrichaceae bacterium isolate RGIG9341 MBO6047387.1 316 aa
BacClassIA Bacillati Bacillota Erysipelotrichia Erysipelotrichales Eggerthia catenaformis WP 281679017.1 319 aa
BacClassIA Bacillati Bacillota Erysipelotrichia Erysipelotrichales Sharpea azabuensis WP 074782091.1 318 aa
BacClassIA Bacillati Bacillota Erysipelotrichia Erysipelotrichales Kandleria multispecies WP 029071585.1 317 aa
BacClassIA Bacillati Bacillota Erysipelotrichia Erysipelotrichales Catenibacterium multispecies WP 217748089.1 316 aa
BacClassIA Bacillati Bacillota Erysipelotrichia Erysipelotrichales Erysipelotrichaceae bacterium isolate U.2 7 MCD7808147.1 322 aa
BacClassIA Bacillati Bacillota Erysipelotrichia Erysipelotrichales Coprobacillus sp isolate ABMPRH 16 1 2 MCI9049797.1 316 aa
BacClassIA Bacillati Bacillota Erysipelotrichia Erysipelotrichales Coprobacillus sp isolate CGS1Y4 bin49 MEG0275545.1 318 aa
BacClassIA Bacillati Bacillota Erysipelotrichia Erysipelotrichales Coprobacillus cateniformis WP 008788941.1 318 aa
BacClassIA Bacillati Bacillota Erysipelotrichia Erysipelotrichales Breznakia blatticola WP 243833758.1 324 aa
BacClassIA Bacillati Bacillota Erysipelotrichia Erysipelotrichales Breznakia sp MFV0380857.1 323 aa
BacClassIA Bacillati Bacillota Erysipelotrichia Erysipelotrichales unclassified Breznakia multispecies WP 277491118.1 324 aa
BacClassIA Bacillati Bacillota Erysipelotrichia Erysipelotrichales Breznakia pachnodae WP 307406741.1 324 aa
BacClassIA Bacillati Bacillota Erysipelotrichia Erysipelotrichales Massilicoli timonensis WP 102268644.1 316 aa
BacClassIA Bacillati Bacillota Erysipelotrichia Erysipelotrichales Erysipelotrichaceae bacterium isolate CGS3Y2 bin60 MEF9961745.1 317 aa
BacClassIA Bacillati Bacillota Erysipelotrichia Erysipelotrichales Copranaerobaculum intestinale WP 160624034.1 316 aa
BacClassIA Bacillati Bacillota Erysipelotrichia Erysipelotrichales Erysipelotrichaceae bacterium isolate CGS6M4 bin53 MEG0823435.1 317 aa
BacClassIA Bacillati Bacillota Erysipelotrichia Erysipelotrichales Dielma fastidiosa WP 022938810.1 318 aa
BacClassIA Bacillati Bacillota Erysipelotrichia Erysipelotrichales uncultured Traorella sp GCF 944327115.1 WP 294562182.1 317 aa
BacClassIA Bacillati Bacillota Erysipelotrichia Erysipelotrichales Traorella massiliensis WP 281627735.1 418 aa
BacClassIA Bacillati Bacillota Erysipelotrichia Erysipelotrichales Erysipelotrichaceae bacterium isolate ABMPRH 170 1 MCI8271102.1 317 aa
BacClassIA Bacillati Bacillota Erysipelotrichia Erysipelotrichales Amedibacterium intestinale WP 115716139.1 318 aa
BacClassIA Bacillati Bacillota Erysipelotrichia Erysipelotrichales Merdibacter sp isolate 39 1 46 MFR2885156.1 314 aa
BacClassIA Bacillati Bacillota Erysipelotrichia Erysipelotrichales Amedibacillus dolichus strain ET39 WP 289607471.1 316 aa
BacClassIA Bacillati Bacillota Erysipelotrichia Erysipelotrichales Amedibacillus dolichus strain DSM 3991 WP 022419698.1 317 aa
BacClassIA Bacillati Bacillota Erysipelotrichia Erysipelotrichales Longicatena sp isolate CGS1Y3 bin16 MEG0526013.1 318 aa
BacClassIA Bacillati Bacillota Erysipelotrichia Erysipelotrichales Longicatena multispecies WP 008690275.1 317 aa
BacClassIA Bacillati Bacillota Erysipelotrichia Erysipelotrichales Ileibacterium valens WP 075817342.1 336 aa
BacClassIA Bacillati Bacillota Erysipelotrichia Erysipelotrichales Ileibacterium sp isolate MAG6 MCF0245757.1 321 aa
BacClassIA Bacillati Bacillota Erysipelotrichia Erysipelotrichales Erysipelotrichaceae bacterium isolate MAG5 MCF0259125.1 340 aa
BacClassIA Bacillati Bacillota Erysipelotrichia Erysipelotrichales Allobaculum sp isolate Mgla MAG 25 bin 11 MBD5423820.1 341 aa
BacClassIA Bacillati Bacillota Erysipelotrichia Erysipelotrichales Erysipelotrichaceae bacterium isolate S17M St 31 MDO4664391.1 331 aa
BacClassIA Bacillati Bacillota Erysipelotrichia Erysipelotrichales Allobaculum sp JKK 2023 WP 323091473.1 335 aa
BacClassIA Bacillati Bacillota Erysipelotrichia Erysipelotrichales Faecalibaculum rodentium WP 302593181.1 332 aa
BacClassIA Bacillati Bacillota Erysipelotrichia Erysipelotrichales Dubosiella newyorkensis WP 076340608.1 321 aa
BacClassIA Bacillati Bacillota Erysipelotrichia Erysipelotrichales uncultured Dubosiella sp GCF 948566665.1 WP 290172390.1 319 aa
BacClassIA Bacillati Bacillota Erysipelotrichia Erysipelotrichales uncultured Dubosiella sp GCF 910585105.1 WP 297054622.1 328 aa
BacClassIA Bacillati Bacillota Erysipelotrichia Erysipelotrichales Faecalicoccus sp isolate RGIG8832 MBP3871298.1 317 aa
BacClassIA Bacillati Bacillota Erysipelotrichia Erysipelotrichales Catenisphaera adipataccumulans WP 183328655.1 324 aa
BacClassIA Bacillati Bacillota Erysipelotrichia Erysipelotrichales Absicoccus porci WP 173445867.1 319 aa
BacClassIA Bacillati Bacillota Erysipelotrichia Erysipelotrichales Floccifex porci WP 154459348.1 320 aa
BacClassIA Bacillati Bacillota Erysipelotrichia Erysipelotrichales uncultured Holdemanella sp GCF 900757855.1 WP 117571428.1 318 aa
BacClassIA Bacillati Bacillota Erysipelotrichia Erysipelotrichales Faecalitalea cylindroides WP 204715396.1 319 aa
BacClassIA Bacillati Bacillota Erysipelotrichia Erysipelotrichales Holdemanella sp isolate MAG4 MCF0106108.1 323 aa
BacClassIA Bacillati Bacillota Erysipelotrichia Erysipelotrichales Faecalicoccus pleomorphus WP 022790106.1 317 aa
BacClassIA Bacillati Bacillota Erysipelotrichia Erysipelotrichales Faecalicoccus acidiformans WP 183375930.1 318 aa
BacClassIA Bacillati Mycoplasmatota Mollicutes Acholeplasmatales Acholeplasmataceae bacterium isolate AS08sgBPME 156 HHU81221.1 321 aa
BacClassIA Bacillati Mycoplasmatota Mollicutes Acholeplasmatales Acholeplasmataceae bacterium isolate AS21ysBPME 308 NLZ84734.1 321 aa
BacClassIA Bacillati Mycoplasmatota Mollicutes unclassified Mollicutes Mollicutes bacterium isolate SUG320 MCI6716373.1 323 aa
BacClassIA Bacillati Mycoplasmatota Mollicutes Acholeplasmatales Acholeplasmataceae bacterium isolate SUG129 MCI5787755.1 321 aa
BacClassIA Bacillati Mycoplasmatota Mollicutes Acholeplasmatales Acholeplasmataceae bacterium isolate AS08sgBPME 216 HHU55866.1 323 aa
BacClassIA Bacillati Mycoplasmatota Mollicutes Acholeplasmatales Acholeplasmataceae bacterium isolate AS08sgBPME 462 HHX00386.1 324 aa
BacClassIA Bacillati Mycoplasmatota Mollicutes Acholeplasmatales Acholeplasmataceae bacterium isolate AS08sgBPME 130 HHX79906.1 323 aa
BacClassIA Bacillati Mycoplasmatota Mollicutes Acholeplasmatales Acholeplasmataceae bacterium isolate AS06rmzACSIP 93 NLD26516.1 323 aa
BacClassIA Bacillati Mycoplasmatota Mollicutes Candidatus Izemoplasmatales Candidatus Izemoplasmatales bacterium isolate STF1 197 MDD4184477.1 316 aa
BacClassIA Bacillati Mycoplasmatota Mollicutes Candidatus Izemoplasmatales Candidatus Izemoplasmatales bacterium isolate SO 2016 CLC T2 110 MFA6791507.1 316 aa
BacClassIA Bacillati Mycoplasmatota Mollicutes Candidatus Izemoplasmatales Candidatus Izemoplasmatales bacterium isolate STD2 261 MDD3124140.1 316 aa
BacClassIA Bacillati Mycoplasmatota Mollicutes Candidatus Izemoplasmatales Candidatus Izemoplasmatales bacterium isolate CA 2019 TPln 173 MGD9910723.1 316 aa
BacClassIA Bacillati Mycoplasmatota Mollicutes Candidatus Izemoplasmatales Candidatus Izemoplasmatales bacterium isolate STF1 90 MDD3841899.1 316 aa
BacClassIA Bacillati Mycoplasmatota unclassified Mycoplasmatota Tenericutes bacterium HGW Tenericutes 1 PKL01128.1 316 aa
BacClassIA Bacillati Mycoplasmatota Mollicutes Acholeplasmatales Acholeplasmataceae bacterium isolate UBA12489 HAQ56822.1 316 aa
BacClassIA Bacillati Mycoplasmatota unclassified Mycoplasmatota Mycoplasmatota bacterium isolate SW 4 67 MCK5732030.1 316 aa
BacClassIA Bacillati Mycoplasmatota Mollicutes Candidatus Izemoplasmatales Candidatus Izemoplasma sp B36 WP 372484245.1 316 aa
BacClassIA Bacillati Mycoplasmatota unclassified Mycoplasmatota Mycoplasmatota bacterium WC30 XMB71587.1 316 aa
BacClassIA Bacillati Mycoplasmatota unclassified Mycoplasmatota Mycoplasmatota bacterium isolate MAG22 MBI9009241.1 316 aa
BacClassIA Bacillati Mycoplasmatota unclassified Mycoplasmatota Tenericutes bacterium 4572 104 OQX93693.1 317 aa
BacClassIA Bacillati Mycoplasmatota Mollicutes Candidatus Izemoplasmatales Candidatus Izemoplasmatales bacterium isolate SO 2016 LW2 153 MFA7561577.1 319 aa
BacClassIA Bacillati Mycoplasmatota Mollicutes Candidatus Izemoplasmatales Candidatus Izemoplasmatales bacterium isolate SO 2017 CLC 65 MFA7075382.1 319 aa
BacClassIA Bacillati Mycoplasmatota Mollicutes Candidatus Izemoplasmatales Candidatus Izemoplasmatales bacterium isolate SO 2017 CLC 53 MFA7106234.1 320 aa
BacClassIA Bacillati Mycoplasmatota Mollicutes Candidatus Izemoplasmatales Candidatus Izemoplasmatales bacterium isolate BLKS 1.bin.63 MFO7969684.1 321 aa
BacClassIA Bacillati Mycoplasmatota Mollicutes Candidatus Izemoplasmatales Hujiaoplasma nucleasis WP 376739689.1 322 aa
BacClassIA Bacillati Mycoplasmatota Mollicutes Candidatus Izemoplasmatales Candidatus Izemoplasmatales bacterium isolate CA 2019 NHSR 100 MGE4572751.1 321 aa
BacClassIA Bacillati Mycoplasmatota unclassified Mycoplasmatota Mycoplasmatota bacterium zrk1 XMB67833.1 319 aa
BacClassIA Bacillati Mycoplasmatota unclassified Mycoplasmatota Mycoplasmatota bacterium WC44 XMB86436.1 320 aa
BacClassIA Bacillati Mycoplasmatota Mollicutes Haloplasmatales Haloplasma contractile WP 008824959.1 322 aa
BacClassIA Bacillati Mycoplasmatota Mollicutes Haloplasmatales Haloplasmataceae bacterium isolate L E1 T20 COA1 bin.182 MDF2699649.1 321 aa
BacClassIA Bacillati Mycoplasmatota Mollicutes Haloplasmatales Haloplasmatales bacterium isolate SMAG U7080 HEY8436165.1 322 aa
BacClassIA Bacillati Mycoplasmatota unclassified Mycoplasmatota Mycoplasmatota bacterium strain zrk4 QVK17963.1 317 aa
BacClassIA Bacillati Mycoplasmatota Mollicutes Haloplasmatales Haloplasmatales bacterium isolate SMAG U7098 HEY8364153.1 317 aa
BacClassIA Bacillati Mycoplasmatota Mollicutes Candidatus Izemoplasmatales Candidatus Izemoplasmataceae bacterium isolate D1M13 20 MFP4077521.1 322 aa
BacClassIA Bacillati Mycoplasmatota Mollicutes Liberiplasmatales Liberiplasma polymorphum WP 435585679.1 321 aa
BacClassIA Bacillati Mycoplasmatota Mollicutes Acholeplasmatales Acholeplasmatales bacterium isolate DLM2 Bin112 TVP96431.1 321 aa
BacClassIA Bacillati Mycoplasmatota Mollicutes Candidatus Izemoplasmatales Candidatus Izimoplasma sp ZiA1 WP 095543968.1 322 aa
BacClassIA Bacillati Mycoplasmatota Mollicutes Candidatus Izemoplasmatales Candidatus Izemoplasma sp isolate MAY MAG 00013 MFK5883750.1 322 aa
BacClassIA Bacillati Mycoplasmatota Mollicutes Candidatus Izemoplasmatales Candidatus Izimoplasma sp HR2 KFZ25741.1 325 aa
BacClassIA Bacillati Mycoplasmatota Mollicutes Candidatus Izemoplasmatales Candidatus Izimoplasma sp isolate RS 4 27 MCK5388793.1 324 aa
BacClassIA Bacillati Mycoplasmatota Mollicutes Candidatus Izemoplasmatales Candidatus Izemoplasma sp isolate SW 4 57 MCK5761287.1 324 aa
BacClassIA Bacillati Mycoplasmatota Mollicutes Candidatus Izemoplasmatales Candidatus Izemoplasma sp isolate DSCult MAG 4 MEC9484659.1 327 aa
BacClassIA Bacillati Mycoplasmatota Mollicutes Candidatus Izemoplasmatales Candidatus Izemoplasma sp isolate Tobar15.5 16.5m G26 MCF7927057.1 323 aa
BacClassIA Bacillati Mycoplasmatota Mollicutes Candidatus Izemoplasmatales Candidatus Xianfuyuplasma coldseepsis WP 258878586.1 326 aa
BacClassIA Bacillati Mycoplasmatota Mollicutes Candidatus Izemoplasmatales Candidatus Izimoplasma sp HR1 WP 038468221.1 326 aa
BacClassIA Bacillati Mycoplasmatota Mollicutes Acholeplasmatales Acholeplasmatales bacterium isolate RGIG10196 MBP5445582.1 318 aa
BacClassIA Bacillati Mycoplasmatota Mollicutes Acholeplasmatales Acholeplasmatales bacterium isolate RUI361 MCR5786847.1 323 aa
BacClassIA Bacillati Mycoplasmatota Mollicutes Anaeroplasmatales Anaeroplasmataceae bacterium isolate WFFMC MAG 645 MGL4949356.1 327 aa
BacClassIA Bacillati Mycoplasmatota Mollicutes Anaeroplasmatales Anaeroplasmataceae bacterium isolate PUUB51 bin.013 MGM9972105.1 327 aa
BacClassIA Bacillati Mycoplasmatota Mollicutes Acholeplasmatales Acholeplasmatales bacterium isolate RUI388 MCR5706129.1 327 aa
BacClassIA Bacillati Mycoplasmatota Mollicutes Acholeplasmatales Acholeplasmatales bacterium isolate RGIG6594 MBR3617301.1 323 aa
BacClassIA Bacillati Mycoplasmatota Mollicutes Anaeroplasmatales Anaeroplasma sp GCF 034179385.1 WP 321092913.1 327 aa
BacClassIA Bacillati Mycoplasmatota Mollicutes Acholeplasmatales Acholeplasmatales bacterium isolate RGIG3708 MBQ9448398.1 327 aa
BacClassIA Bacillati Mycoplasmatota Mollicutes Acholeplasmatales Acholeplasmatales bacterium isolate Zx50 bin71 MDR2822110.1 320 aa
BacClassIA Bacillati Mycoplasmatota Mollicutes Acholeplasmatales Acholeplasmatales bacterium isolate Zx50 bin8 MDR2828906.1 323 aa
BacClassIA Bacillati Mycoplasmatota Mollicutes Acholeplasmatales Acholeplasmatales bacterium isolate Zx50 bin49 MDR2867381.1 323 aa
BacClassIA Bacillati Mycoplasmatota Mollicutes Acholeplasmatales Acholeplasma sp OttesenSCG 928 E16 isolate OttesenSCG 928 E16 MDL2292595.1 322 aa
BacClassIA Bacillati Mycoplasmatota Mollicutes Acholeplasmatales Acholeplasmataceae bacterium isolate AS10tlH2TH 123 NLB84618.1 326 aa
BacClassIA Bacillati Mycoplasmatota Mollicutes Acholeplasmatales Acholeplasmataceae bacterium isolate SBF912LR0HQ0205 MGI6787355.1 320 aa
BacClassIA Bacillati Mycoplasmatota Mollicutes Acholeplasmatales Acholeplasmataceae bacterium isolate SBF912LR0HQ0061 MGI6782430.1 321 aa
BacClassIA Bacillati Mycoplasmatota Mollicutes Acholeplasmatales Acholeplasmataceae bacterium isolate AS08sgBPME 440 HHT82120.1 322 aa
BacClassIA Bacillati Mycoplasmatota Mollicutes Acholeplasmatales Acholeplasmataceae bacterium isolate AS06rmzACSIP 177 NLG31304.1 320 aa
BacClassIA Bacillati Mycoplasmatota Mollicutes Acholeplasmatales Phytoplasma sp isolate AS23ysBPME 113 NLK12075.1 327 aa
BacClassIA Bacillati Mycoplasmatota Mollicutes Acholeplasmatales Acholeplasma palmae WP 026654389.1 321 aa
BacClassIA Bacillati Mycoplasmatota Mollicutes Acholeplasmatales Acholeplasma sp isolate SMAG U7093 HEY8405481.1 321 aa
BacClassIA Bacillati Mycoplasmatota Mollicutes Acholeplasmatales Acholeplasma equifetale WP 026400110.1 321 aa
BacClassIA Bacillati Mycoplasmatota Mollicutes Acholeplasmatales Acholeplasma hippikon WP 035369889.1 320 aa
BacClassIA Bacillati Mycoplasmatota Mollicutes Acholeplasmatales Acholeplasma equirhinis WP 205735387.1 321 aa
BacClassIA Bacillati Mycoplasmatota Mollicutes Acholeplasmatales Acholeplasmataceae bacterium isolate SO 2017 LW2 10 MFA5692184.1 323 aa
BacClassIA Bacillati Mycoplasmatota Mollicutes Acholeplasmatales Acholeplasma oculi WP 045749913.1 322 aa
BacClassIA Bacillati Mycoplasmatota Mollicutes Acholeplasmatales Acholeplasma laidlawii WP 094573211.1 321 aa
BacClassIA Bacillati Mycoplasmatota Mollicutes Acholeplasmatales Acholeplasmatales bacterium isolate BF2 17 MDY0133486.1 323 aa
BacClassIA Bacillati Mycoplasmatota Mollicutes Acholeplasmatales Acholeplasmataceae bacterium isolate SO 2017 LW2 bin 22 MCK9493152.1 324 aa
BacClassIA Bacillati Mycoplasmatota Mollicutes Acholeplasmatales Acholeplasmataceae bacterium isolate SO 2016 CLC T1 bin 71 MCK9165962.1 324 aa
BacClassIA Bacillati Mycoplasmatota Mollicutes Acholeplasmatales Acholeplasmataceae bacterium isolate SBF912LR0HQ0086 MGI6360077.1 324 aa
BacClassIA Bacillati Mycoplasmatota Mollicutes Acholeplasmatales Acholeplasmataceae bacterium isolate AS22ysBPME 182 NMA05205.1 324 aa
BacClassIA Bacillati Mycoplasmatota Mollicutes Acholeplasmatales Acholeplasma sp isolate SMAG U9103 HKL60954.1 325 aa
BacClassIA Bacillati Mycoplasmatota Mollicutes Acholeplasmatales Acholeplasma brassicae WP 030003911.1 324 aa
BacClassIA Bacillati Mycoplasmatota Mollicutes Acholeplasmatales Paracholeplasma vituli WP 262095407.1 325 aa
BacClassIA Bacillati Mycoplasmatota Mollicutes Acholeplasmatales Paracholeplasma morum WP 204432113.1 324 aa
BacClassIA Bacillati Mycoplasmatota Mollicutes Acholeplasmatales Acholeplasma sp isolate BF1 46 MDY0210299.1 324 aa
BacClassIA Bacteria incertae sedis Candidatus Poribacteria Candidatus Poribacteria bacterium isolate UWMA 0232 HIE27409.1 328 aa
BacClassIA Bacteria incertae sedis Candidatus Poribacteria Candidatus Poribacteria bacterium isolate Bin01 175 MCS5608804.1 331 aa
BacClassIA Bacteria incertae sedis Candidatus Poribacteria Candidatus Poribacteria bacterium isolate Arabian11 MAG 5 MDP6595412.1 330 aa
BacClassIA Bacteria incertae sedis Candidatus Poribacteria Candidatus Poribacteria bacterium isolate XB MAG1182 MEE2911950.1 331 aa
BacClassIA Bacteria incertae sedis Candidatus Poribacteria Candidatus Poribacteria bacterium isolate PCPOR2b RKU07210.1 331 aa
BacClassIA Bacteria incertae sedis Candidatus Poribacteria Candidatus Poribacteria bacterium isolate RHO2 bin 7 MDE0184183.1 330 aa
BacClassIA Bacteria incertae sedis Candidatus Poribacteria Candidatus Poribacteria bacterium isolate KW1 S08 28 MCZ6679550.1 332 aa
BacClassIA Bacteria incertae sedis Candidatus Poribacteria Candidatus Poribacteria bacterium isolate RHO3 bin 62 iso1 MDE0042445.1 331 aa
BacClassIA Bacteria incertae sedis Candidatus Poribacteria Candidatus Poribacteria bacterium isolate AGPOR5 RKU30402.1 328 aa
BacClassIA Bacteria incertae sedis Candidatus Poribacteria Candidatus Poribacteria bacterium isolate MSPOR6 RKU27570.1 331 aa
BacClassIA Bacteria incertae sedis Candidatus Poribacteria Candidatus Poribacteria bacterium isolate SB0668 bin 37 MXV73357.1 331 aa
BacClassIA Bacteria incertae sedis Candidatus Poribacteria Candidatus Poribacteria bacterium isolate RHO2 bin 17 MDE0314881.1 333 aa
BacClassIA Bacteria incertae sedis Candidatus Poribacteria Candidatus Poribacteria bacterium isolate SB0668 bin 40 MXV78022.1 331 aa

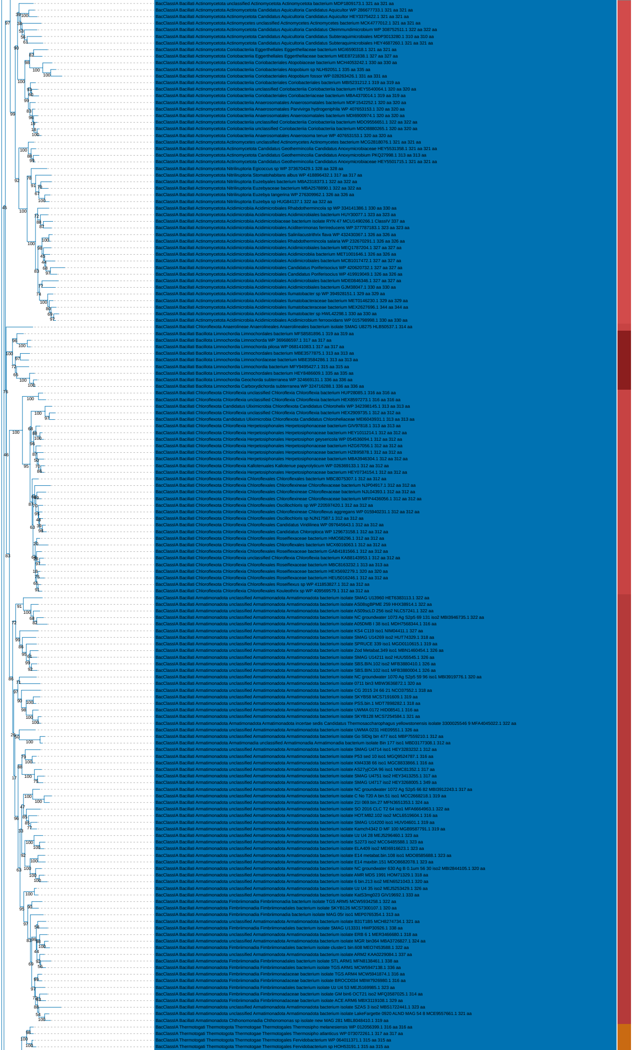

BacClassIA Bacillati Actinomycetota unclassified Actinomycetota Actinomycetota bacterium MDP1809173.1 321 aa 321 aa
BacClassIA Bacillati Actinomycetota Actinomycetes Candidatus Aquicultoria Candidatus Aquicultor WP 286677733.1 321 aa 321 aa
BacClassIA Bacillati Actinomycetota Actinomycetes Candidatus Aquicultoria Candidatus Aquicultor HEY3375422.1 321 aa 321 aa
BacClassIA Bacillati Actinomycetota Actinomycetes unclassified Actinomycetes Actinomycetes bacterium MCK4777012.1 321 aa 321 aa
BacClassIA Bacillati Actinomycetota Actinomycetes Candidatus Aquicultoria Candidatus Oleimmundimicrobium WP 308752511.1 322 aa 322 aa
BacClassIA Bacillati Actinomycetota Actinomycetes Candidatus Aquicultoria Candidatus Subterraquimicrobiales MDP3013280.1 310 aa 310 aa
BacClassIA Bacillati Actinomycetota Actinomycetes Candidatus Aquicultoria Candidatus Subterraquimicrobiales HEY4687260.1 321 aa 321 aa
BacClassIA Bacillati Actinomycetota Coriobacteriia Eggerthellales Eggerthellaceae bacterium MGI6590318.1 321 aa 321 aa
BacClassIA Bacillati Actinomycetota Coriobacteriia Eggerthellales Eggerthellaceae bacterium MEE8721838.1 327 aa 327 aa
BacClassIA Bacillati Actinomycetota Coriobacteriia Coriobacteriales Atopobiaceae bacterium MCH4053242.1 330 aa 330 aa
BacClassIA Bacillati Actinomycetota Coriobacteriia Coriobacteriales Atopobium sp NLH92051.1 335 aa 335 aa
BacClassIA Bacillati Actinomycetota Coriobacteriia Coriobacteriales Atopobium fossor WP 028263426.1 331 aa 331 aa
BacClassIA Bacillati Actinomycetota Coriobacteriia Coriobacteriales Coriobacteriales bacterium MBI5231212.1 319 aa 319 aa
BacClassIA Bacillati Actinomycetota Coriobacteriia unclassified Coriobacteriia Coriobacteriia bacterium HEY5540064.1 320 aa 320 aa
BacClassIA Bacillati Actinomycetota Coriobacteriia Coriobacteriales Coriobacteriaceae bacterium MBA4370014.1 319 aa 319 aa
BacClassIA Bacillati Actinomycetota Coriobacteriia Anaerosomatales Anaerosomatales bacterium MDF1542252.1 320 aa 320 aa
BacClassIA Bacillati Actinomycetota Coriobacteriia Anaerosomatales Parvivirga hydrogeniphila WP 407653153.1 320 aa 320 aa
BacClassIA Bacillati Actinomycetota Coriobacteriia Anaerosomatales Anaerosomatales bacterium MDI6900974.1 320 aa 320 aa
BacClassIA Bacillati Actinomycetota Coriobacteriia unclassified Coriobacteriia Coriobacteriia bacterium MDO9556651.1 322 aa 322 aa
BacClassIA Bacillati Actinomycetota Coriobacteriia unclassified Coriobacteriia Coriobacteriia bacterium MDO8880265.1 320 aa 320 aa
BacClassIA Bacillati Actinomycetota Coriobacteriia Anaerosomatales Anaerosoma tenue WP 407653153.1 320 aa 320 aa
BacClassIA Bacillati Actinomycetota Actinomycetes unclassified Actinomycetes Actinomycetes bacterium MCG2818076.1 321 aa 321 aa
BacClassIA Bacillati Actinomycetota Actinomycetes Candidatus Geothermincolia Candidatus Anoxymicrobiaceae HEY5531358.1 321 aa 321 aa
BacClassIA Bacillati Actinomycetota Actinomycetes Candidatus Geothermincolia Candidatus Anoxymicrobium PKQ27998.1 313 aa 313 aa
BacClassIA Bacillati Actinomycetota Actinomycetes Candidatus Geothermincolia Candidatus Anoxymicrobiaceae HEY5501715.1 321 aa 321 aa
BacClassIA Bacillati Actinomycetota Nitriliruptoria Egicoccus sp WP 373670429.1 328 aa 328 aa
BacClassIA Bacillati Actinomycetota Nitriliruptoria Stomatohabitans albus WP 418896432.1 317 aa 317 aa
BacClassIA Bacillati Actinomycetota Nitriliruptoria Euzebyales bacterium MBA2318373.1 322 aa 322 aa
BacClassIA Bacillati Actinomycetota Nitriliruptoria Euzebyaceae bacterium MBA2578890.1 322 aa 322 aa
BacClassIA Bacillati Actinomycetota Nitriliruptoria Euzebya tangerina WP 276309962.1 326 aa 326 aa
BacClassIA Bacillati Actinomycetota Nitriliruptoria Euzebya sp HUG84137.1 322 aa 322 aa
BacClassIA Bacillati Actinomycetota Acidimicrobiia Acidimicrobiales Rhabdothermincola sp WP 334141386.1 330 aa 330 aa
BacClassIA Bacillati Actinomycetota Acidimicrobiia Acidimicrobiales Acidimicrobiales bacterium HUY30077.1 323 aa 323 aa
BacClassIA Bacillati Actinomycetota Acidimicrobiia Acidimicrobiaceae bacterium isolate RYN 47 MCU1490266.1 ClassIV 337 aa
BacClassIA Bacillati Actinomycetota Acidimicrobiia Acidimicrobiales Aciditerrimonas ferrireducens WP 377787183.1 323 aa 323 aa
BacClassIA Bacillati Actinomycetota Acidimicrobiia Acidimicrobiales Salinilacustrithrix flava WP 432430367.1 326 aa 326 aa
BacClassIA Bacillati Actinomycetota Acidimicrobiia Acidimicrobiales Rhabdothermincola salaria WP 232670291.1 326 aa 326 aa
BacClassIA Bacillati Actinomycetota Acidimicrobiia Acidimicrobiales Acidimicrobiales bacterium MEQ1787204.1 327 aa 327 aa
BacClassIA Bacillati Actinomycetota Acidimicrobiia Acidimicrobiales Acidimicrobiia bacterium MET1001646.1 326 aa 326 aa
BacClassIA Bacillati Actinomycetota Acidimicrobiia Acidimicrobiales Acidimicrobiales bacterium MCB1017472.1 327 aa 327 aa
BacClassIA Bacillati Actinomycetota Acidimicrobiia Acidimicrobiales Candidatus Poriferisocius WP 420620732.1 327 aa 327 aa
BacClassIA Bacillati Actinomycetota Acidimicrobiia Acidimicrobiales Candidatus Poriferisocius WP 419919049.1 326 aa 326 aa
BacClassIA Bacillati Actinomycetota Acidimicrobiia Acidimicrobiales Acidimicrobiales bacterium MDE0846346.1 327 aa 327 aa
BacClassIA Bacillati Actinomycetota Acidimicrobiia Acidimicrobiales Acidimicrobiales bacterium GJM38047.1 330 aa 330 aa
BacClassIA Bacillati Actinomycetota Acidimicrobiia Acidimicrobiales Ilumatobacter sp WP 394928151.1 329 aa 329 aa
BacClassIA Bacillati Actinomycetota Acidimicrobiia Acidimicrobiales Ilumatobacteraceae bacterium MET0146230.1 329 aa 329 aa
BacClassIA Bacillati Actinomycetota Acidimicrobiia Acidimicrobiales Ilumatobacteraceae bacterium MEX2627696.1 344 aa 344 aa
BacClassIA Bacillati Actinomycetota Acidimicrobiia Acidimicrobiales Ilumatobacter sp HWL42298.1 330 aa 330 aa
BacClassIA Bacillati Actinomycetota Acidimicrobiia Acidimicrobiales Acidimicrobium ferrooxidans WP 015798998.1 330 aa 330 aa
BacClassIA Bacillati Chloroflexota Anaerolineae Anaerolineales Anaerolineales bacterium isolate SMAG U8275 HLB50537.1 314 aa
BacClassIA Bacillati Bacillota Limnochordia Limnochordales bacterium MFS8581896.1 319 aa 319 aa
BacClassIA Bacillati Bacillota Limnochordia Limnochorda WP 369686597.1 317 aa 317 aa
BacClassIA Bacillati Bacillota Limnochordia Limnochorda pilosa WP 068141083.1 317 aa 317 aa
BacClassIA Bacillati Bacillota Limnochordia Limnochordales bacterium MBE3577875.1 313 aa 313 aa
BacClassIA Bacillati Bacillota Limnochordia Limnochordaceae bacterium MBE3584286.1 313 aa 313 aa
BacClassIA Bacillati Bacillota Limnochordia Limnochorda bacterium MFY9495427.1 315 aa 315 aa
BacClassIA Bacillati Bacillota Limnochordia Limnochordales bacterium HEY8486609.1 335 aa 335 aa
BacClassIA Bacillati Bacillota Limnochordia Geochorda subterranea WP 324669131.1 336 aa 336 aa
BacClassIA Bacillati Bacillota Limnochordia Carboxydichorda subterranea WP 324716288.1 336 aa 336 aa
BacClassIA Bacillati Chloroflexota Chloroflexia unclassified Chloroflexia Chloroflexia bacterium HUP28085.1 316 aa 316 aa
BacClassIA Bacillati Chloroflexota Chloroflexia unclassified Chloroflexia Chloroflexia bacterium HEX8597273.1 316 aa 316 aa
BacClassIA Bacillati Chloroflexota Candidatus Uliximicrobia Chloroflexota Candidatus Chlorohelix WP 342398145.1 313 aa 313 aa
BacClassIA Bacillati Chloroflexota Chloroflexia unclassified Chloroflexia Chloroflexia bacterium HEX2909735.1 312 aa 312 aa
BacClassIA Bacillati Chloroflexota Candidatus Uliximicrobia Chloroflexota Candidatus Chloroheliaceae MEI6043931.1 313 aa 313 aa
BacClassIA Bacillati Chloroflexota Chloroflexia Herpetosiphonales Herpetosiphonaceae bacterium GIV97818.1 313 aa 313 aa
BacClassIA Bacillati Chloroflexota Chloroflexia Herpetosiphonales Herpetosiphonaceae bacterium HEY1011214.1 312 aa 312 aa
BacClassIA Bacillati Chloroflexota Chloroflexia Herpetosiphonales Herpetosiphon geysericola WP 054536094.1 312 aa 312 aa
BacClassIA Bacillati Chloroflexota Chloroflexia Herpetosiphonales Herpetosiphonaceae bacterium HZG67056.1 312 aa 312 aa
BacClassIA Bacillati Chloroflexota Chloroflexia Herpetosiphonales Herpetosiphonaceae bacterium HZB95878.1 312 aa 312 aa
BacClassIA Bacillati Chloroflexota Chloroflexia Herpetosiphonales Herpetosiphonaceae bacterium MBA3946304.1 312 aa 312 aa
BacClassIA Bacillati Chloroflexota Chloroflexia Kallotenuales Kallotenue papyrolyticum WP 026369133.1 312 aa 312 aa
BacClassIA Bacillati Chloroflexota Chloroflexia Herpetosiphonales Herpetosiphonaceae bacterium HEY0734154.1 312 aa 312 aa
BacClassIA Bacillati Chloroflexota Chloroflexia Chloroflexales Chloroflexales bacterium MBC8075307.1 312 aa 312 aa
BacClassIA Bacillati Chloroflexota Chloroflexia Chloroflexales Chloroflexineae Chloroflexaceae bacterium NJP04917.1 312 aa 312 aa
BacClassIA Bacillati Chloroflexota Chloroflexia Chloroflexales Chloroflexineae Chloroflexaceae bacterium NJL04393.1 312 aa 312 aa
BacClassIA Bacillati Chloroflexota Chloroflexia Chloroflexales Chloroflexineae Chloroflexaceae bacterium MFP4436056.1 312 aa 312 aa
BacClassIA Bacillati Chloroflexota Chloroflexia Chloroflexales Oscillochloris sp WP 220597420.1 312 aa 312 aa
BacClassIA Bacillati Chloroflexota Chloroflexia Chloroflexales Chloroflexineae Chloroflexus aggregans WP 015940231.1 312 aa 312 aa
BacClassIA Bacillati Chloroflexota Chloroflexia Chloroflexales Oscillochloris sp NJN17587.1 312 aa 312 aa
BacClassIA Bacillati Chloroflexota Chloroflexia Chloroflexales Candidatus Viridilinea WP 097645643.1 312 aa 312 aa
BacClassIA Bacillati Chloroflexota Chloroflexia Chloroflexales Candidatus Chloroploca WP 129673158.1 312 aa 312 aa
BacClassIA Bacillati Chloroflexota Chloroflexia Chloroflexales Roseiflexaceae bacterium HMO58296.1 312 aa 312 aa
BacClassIA Bacillati Chloroflexota Chloroflexia Chloroflexales Chloroflexales bacterium MCX6016063.1 312 aa 312 aa
BacClassIA Bacillati Chloroflexota Chloroflexia Chloroflexales Roseiflexaceae bacterium GAB4181566.1 312 aa 312 aa
BacClassIA Bacillati Chloroflexota Chloroflexia unclassified Chloroflexia Chloroflexia bacterium KAB8143953.1 312 aa 312 aa
BacClassIA Bacillati Chloroflexota Chloroflexia Chloroflexales Roseiflexaceae bacterium MBC8163232.1 313 aa 313 aa
BacClassIA Bacillati Chloroflexota Chloroflexia Chloroflexales Roseiflexaceae bacterium HEX5692279.1 320 aa 320 aa
BacClassIA Bacillati Chloroflexota Chloroflexia Chloroflexales Roseiflexaceae bacterium HEU5016246.1 312 aa 312 aa
BacClassIA Bacillati Chloroflexota Chloroflexia Chloroflexales Roseiflexus sp WP 411853827.1 312 aa 312 aa
BacClassIA Bacillati Chloroflexota Chloroflexia Chloroflexales Kouleothrix sp WP 409569579.1 312 aa 312 aa
BacClassIA Bacillati Armatimonadota unclassified Armatimonadota Armatimonadota bacterium isolate SMAG U13960 HET6383113.1 322 aa
BacClassIA Bacillati Armatimonadota unclassified Armatimonadota Armatimonadota bacterium isolate AS08sgBPME 259 HHX38914.1 322 aa
BacClassIA Bacillati Armatimonadota unclassified Armatimonadota Armatimonadota bacterium isolate AS09scLD 256 iso2 NLC57241.1 322 aa
BacClassIA Bacillati Armatimonadota unclassified Armatimonadota Armatimonadota bacterium isolate NC groundwater 1073 Ag S2p5 69 131 iso2 MBI3946735.1 322 aa
BacClassIA Bacillati Armatimonadota unclassified Armatimonadota Armatimonadota bacterium isolate A05DMB I 38 iso1 MDH7568344.1 316 aa
BacClassIA Bacillati Armatimonadota unclassified Armatimonadota Armatimonadota bacterium isolate KS4 C119 iso1 NIM04411.1 327 aa
BacClassIA Bacillati Armatimonadota unclassified Armatimonadota Armatimonadota bacterium isolate SMAG U14269 iso2 HUT74329.1 318 aa
BacClassIA Bacillati Armatimonadota unclassified Armatimonadota Armatimonadota bacterium isolate SPRUCE 339 iso1 MGD0110615.1 319 aa
BacClassIA Bacillati Armatimonadota unclassified Armatimonadota Armatimonadota bacterium isolate Zod Metabat.349 iso1 MBN1460454.1 326 aa
BacClassIA Bacillati Armatimonadota unclassified Armatimonadota Armatimonadota bacterium isolate SMAG U14211 iso2 HUU55545.1 326 aa
BacClassIA Bacillati Armatimonadota unclassified Armatimonadota Armatimonadota bacterium isolate SBS.BIN.102 iso2 MFB3880410.1 326 aa
BacClassIA Bacillati Armatimonadota unclassified Armatimonadota Armatimonadota bacterium isolate SBS.BIN.102 iso1 MFB3880004.1 326 aa
BacClassIA Bacillati Armatimonadota unclassified Armatimonadota Armatimonadota bacterium isolate NC groundwater 1070 Ag S2p5 59 96 iso1 MBI3919776.1 320 aa
BacClassIA Bacillati Armatimonadota unclassified Armatimonadota Armatimonadota bacterium isolate 0711 bin3 MBW3636872.1 320 aa
BacClassIA Bacillati Armatimonadota unclassified Armatimonadota Armatimonadota bacterium isolate CG 2015 24 66 21 NCO37552.1 318 aa
BacClassIA Bacillati Armatimonadota unclassified Armatimonadota Armatimonadota bacterium isolate SKYB58 MCS7191609.1 319 aa
BacClassIA Bacillati Armatimonadota unclassified Armatimonadota Armatimonadota bacterium isolate PSS.bin.1 MDT7898282.1 318 aa
BacClassIA Bacillati Armatimonadota unclassified Armatimonadota Armatimonadota bacterium isolate UWMA 0172 HID08541.1 316 aa
BacClassIA Bacillati Armatimonadota unclassified Armatimonadota Armatimonadota bacterium isolate SKYB128 MCS7254584.1 321 aa
BacClassIA Bacillati Armatimonadota Armatimonadota Armatimonadota incertae sedis Candidatus Thermosaccharophagus yellowstonensis isolate 3300025546 9 MFA4045022.1 322 aa
BacClassIA Bacillati Armatimonadota unclassified Armatimonadota Armatimonadota bacterium isolate UWMA 0231 HIE09551.1 326 aa
BacClassIA Bacillati Armatimonadota unclassified Armatimonadota Armatimonadota bacterium isolate Go SlDig bin 477 iso1 MBP7559210.1 312 aa
BacClassIA Bacillati Armatimonadota Armatimonadia unclassified Armatimonadia Armatimonadia bacterium isolate Bin 177 iso1 MBD3177308.1 312 aa
BacClassIA Bacillati Armatimonadota unclassified Armatimonadota Armatimonadota bacterium isolate SMAG U4714 iso1 HEY3283232.1 312 aa
BacClassIA Bacillati Armatimonadota unclassified Armatimonadota Armatimonadota bacterium isolate P53 sed 10 iso1 MGQ9524787.1 316 aa
BacClassIA Bacillati Armatimonadota unclassified Armatimonadota Armatimonadota bacterium isolate KM4338 66 iso1 MGC8833866.1 316 aa
BacClassIA Bacillati Armatimonadota unclassified Armatimonadota Armatimonadota bacterium isolate AS27yjCOA 96 iso1 NMC81352.1 317 aa
BacClassIA Bacillati Armatimonadota unclassified Armatimonadota Armatimonadota bacterium isolate SMAG U4751 iso2 HEY3413255.1 317 aa
BacClassIA Bacillati Armatimonadota unclassified Armatimonadota Armatimonadota bacterium isolate SMAG U4717 iso2 HEY3268005.1 349 aa
BacClassIA Bacillati Armatimonadota unclassified Armatimonadota Armatimonadota bacterium isolate NC groundwater 1072 Ag S2p5 66 82 MBI3912243.1 317 aa
BacClassIA Bacillati Armatimonadota unclassified Armatimonadota Armatimonadota bacterium isolate C No T20 A bin.51 iso1 MCC2668218.1 319 aa
BacClassIA Bacillati Armatimonadota unclassified Armatimonadota Armatimonadota bacterium isolate 21I 069.bin.27 MFN3651353.1 324 aa
BacClassIA Bacillati Armatimonadota unclassified Armatimonadota Armatimonadota bacterium isolate SO 2016 CLC T2 64 iso1 MFA6664963.1 322 aa
BacClassIA Bacillati Armatimonadota unclassified Armatimonadota Armatimonadota bacterium isolate HOT.MB2.102 iso2 MCL6519604.1 316 aa
BacClassIA Bacillati Armatimonadota unclassified Armatimonadota Armatimonadota bacterium isolate SMAG U14200 iso1 HUV04601.1 319 aa
BacClassIA Bacillati Armatimonadota unclassified Armatimonadota Armatimonadota bacterium isolate Kamch4342 D MF 100 MGB9587791.1 319 aa
BacClassIA Bacillati Armatimonadota unclassified Armatimonadota Armatimonadota bacterium isolate Uz U4 28 MEJ5296460.1 323 aa
BacClassIA Bacillati Armatimonadota unclassified Armatimonadota Armatimonadota bacterium isolate SJ273 iso2 MCC6485588.1 323 aa
BacClassIA Bacillati Armatimonadota unclassified Armatimonadota Armatimonadota bacterium isolate ELA409 iso2 MEI6916623.1 323 aa
BacClassIA Bacillati Armatimonadota unclassified Armatimonadota Armatimonadota bacterium isolate E14 metabat.bin.108 iso1 MDO8585668.1 323 aa
BacClassIA Bacillati Armatimonadota unclassified Armatimonadota Armatimonadota bacterium isolate E14 maxbin.151 MDO8682078.1 323 aa
BacClassIA Bacillati Armatimonadota unclassified Armatimonadota Armatimonadota bacterium isolate NC groundwater 630 Ag B 0.1um 56 30 iso2 MBI2844105.1 320 aa
BacClassIA Bacillati Armatimonadota unclassified Armatimonadota Armatimonadota bacterium isolate AMR MDS 1991 HOM71329.1 318 aa
BacClassIA Bacillati Armatimonadota unclassified Armatimonadota Armatimonadota bacterium isolate 6 bin.213 iso2 MEN6521043.1 320 aa
BacClassIA Bacillati Armatimonadota unclassified Armatimonadota Armatimonadota bacterium isolate Uz U4 35 iso2 MEJ5253429.1 326 aa
BacClassIA Bacillati Armatimonadota unclassified Armatimonadota Armatimonadota bacterium isolate KatS3mg023 GIV19692.1 333 aa
BacClassIA Bacillati Armatimonadota Fimbriimonadia Fimbriimonadia bacterium isolate TGS ARM5 MCW5934258.1 322 aa
BacClassIA Bacillati Armatimonadota Fimbriimonadia Fimbriimonadales bacterium isolate SKYB126 MCS7300107.1 320 aa
BacClassIA Bacillati Armatimonadota Fimbriimonadia Fimbriimonadia bacterium isolate MAG 05r iso1 MEP0765354.1 313 aa
BacClassIA Bacillati Armatimonadota unclassified Armatimonadota Armatimonadota bacterium isolate B31T1B5 MCH8274734.1 321 aa
BacClassIA Bacillati Armatimonadota Fimbriimonadia Fimbriimonadales bacterium isolate SMAG U13331 HWP30926.1 338 aa
BacClassIA Bacillati Armatimonadota unclassified Armatimonadota Armatimonadota bacterium isolate ERB 6 1 MER3466680.1 318 aa
BacClassIA Bacillati Armatimonadota unclassified Armatimonadota Armatimonadota bacterium isolate MGR bin364 MBA3726827.1 324 aa
BacClassIA Bacillati Armatimonadota Fimbriimonadia Fimbriimonadales bacterium isolate cluster1 bin.608 MEO7453588.1 322 aa
BacClassIA Bacillati Armatimonadota unclassified Armatimonadota Armatimonadota bacterium isolate ARM2 KAA0229084.1 337 aa
BacClassIA Bacillati Armatimonadota Fimbriimonadia Fimbriimonadales bacterium isolate STL ARM1 MFN8138461.1 338 aa
BacClassIA Bacillati Armatimonadota Fimbriimonadia Fimbriimonadales bacterium isolate TGS ARM1 MCW5947138.1 336 aa
BacClassIA Bacillati Armatimonadota Fimbriimonadia Fimbriimonadaceae bacterium isolate TGS ARM4 MCW5941874.1 316 aa
BacClassIA Bacillati Armatimonadota Fimbriimonadia Fimbriimonadaceae bacterium isolate BRDCD034 MBW7926980.1 316 aa
BacClassIA Bacillati Armatimonadota Fimbriimonadia Fimbriimonadales bacterium isolate Uz U4 53 MEJ5169985.1 323 aa
BacClassIA Bacillati Armatimonadota Fimbriimonadia Fimbriimonadaceae bacterium isolate GM bin6 OCT21 iso2 MFQ3587025.1 314 aa
BacClassIA Bacillati Armatimonadota Fimbriimonadia Fimbriimonadaceae bacterium isolate ACE ARM6 MBX3119108.1 329 aa
BacClassIA Bacillati Armatimonadota unclassified Armatimonadota Armatimonadota bacterium isolate SZAS 3 iso2 MBS1722441.1 323 aa
BacClassIA Bacillati Armatimonadota unclassified Armatimonadota Armatimonadota bacterium isolate LakeFargette 0920 ALND MAG 54 8 MCE9557661.1 321 aa
BacClassIA Bacillati Armatimonadota Chthonomonadia Chthonomonadales Chthonomonas sp isolate new MAG 281 MBL8048410.1 319 aa
BacClassIA Thermotogati Thermotogota Thermotogae Thermotogales Thermosipho melanesiensis WP 012056399.1 316 aa 316 aa
BacClassIA Thermotogati Thermotogota Thermotogae Thermotogales Thermosipho atlanticus WP 073072261.1 317 aa 317 aa
BacClassIA Thermotogati Thermotogota Thermotogae Thermotogales Fervidobacterium WP 064011371.1 315 aa 315 aa
BacClassIA Thermotogati Thermotogota Thermotogae Thermotogales Fervidobacterium sp HOH53191.1 315 aa 315 aa

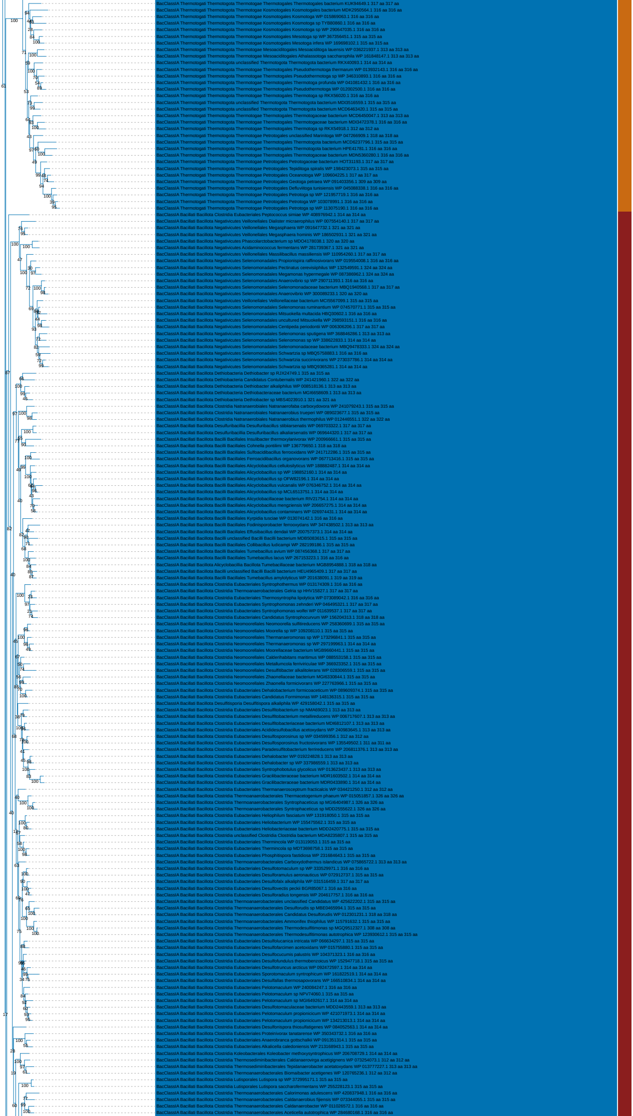
BacClassIA Thermotogati Thermotogota Thermotogae Thermotogales Thermotogales bacterium KUK94649.1 317 aa 317 aa
BacClassIA Thermotogati Thermotogota Thermotogae Kosmotogales Kosmotogales bacterium MDK2950564.1 316 aa 316 aa
BacClassIA Thermotogati Thermotogota Thermotogae Kosmotogales Kosmotoga WP 015869063.1 316 aa 316 aa
BacClassIA Thermotogati Thermotogota Thermotogae Kosmotogales Kosmotoga sp TYB80860.1 316 aa 316 aa
BacClassIA Thermotogati Thermotogota Thermotogae Kosmotogales Kosmotoga sp WP 290647035.1 316 aa 316 aa
BacClassIA Thermotogati Thermotogota Thermotogae Kosmotogales Mesotoga sp WP 367356451.1 315 aa 315 aa
BacClassIA Thermotogati Thermotogota Thermotogae Kosmotogales Mesotoga infera WP 169698102.1 315 aa 315 aa
BacClassIA Thermotogati Thermotogota Thermotogae Mesoaciditogales Mesoaciditoga lauensis WP 036221937.1 313 aa 313 aa
BacClassIA Thermotogati Thermotogota Thermotogae Mesoaciditogales Athalassotoga saccharophila WP 161848147.1 313 aa 313 aa
BacClassIA Thermotogati Thermotogota unclassified Thermotogota Thermotogota bacterium RKX40093.1 314 aa 314 aa
BacClassIA Thermotogati Thermotogota Thermotogae Thermotogales Pseudothermotoga thermarum WP 013932143.1 316 aa 316 aa
BacClassIA Thermotogati Thermotogota Thermotogae Thermotogales Pseudothermotoga sp WP 346310893.1 316 aa 316 aa
BacClassIA Thermotogati Thermotogota Thermotogae Thermotogales Thermotoga profunda WP 041081432.1 316 aa 316 aa
BacClassIA Thermotogati Thermotogota Thermotogae Thermotogales Pseudothermotoga WP 012002500.1 316 aa 316 aa
BacClassIA Thermotogati Thermotogota Thermotogae Thermotogales Thermotoga sp RKX56020.1 316 aa 316 aa
BacClassIA Thermotogati Thermotogota unclassified Thermotogota Thermotogota bacterium MDI3516559.1 315 aa 315 aa
BacClassIA Thermotogati Thermotogota unclassified Thermotogota Thermotogota bacterium MCD6463420.1 315 aa 315 aa
BacClassIA Thermotogati Thermotogota Thermotogae Thermotogales Thermotogaceae bacterium MCD6450047.1 313 aa 313 aa
BacClassIA Thermotogati Thermotogota Thermotogae Thermotogales Thermotogaceae bacterium MDI3472378.1 316 aa 316 aa
BacClassIA Thermotogati Thermotogota Thermotogae Thermotogales Thermotoga sp RKX54918.1 312 aa 312 aa
BacClassIA Thermotogati Thermotogota Thermotogae Petrotogales unclassified Marinitoga WP 047266909.1 318 aa 318 aa
BacClassIA Thermotogati Thermotogota Thermotogae Thermotogales Thermotogota bacterium MCD6237796.1 315 aa 315 aa
BacClassIA Thermotogati Thermotogota Thermotogae Thermotogales Thermotogota bacterium HPE41781.1 316 aa 316 aa
BacClassIA Thermotogati Thermotogota Thermotogae Thermotogales Thermotogaceae bacterium MDN5360280.1 316 aa 316 aa
BacClassIA Thermotogati Thermotogota Thermotogae Petrotogales Petrotogaceae bacterium HOT31193.1 317 aa 317 aa
BacClassIA Thermotogati Thermotogota Thermotogae Petrotogales Tepiditoga spiralis WP 198423073.1 315 aa 315 aa
BacClassIA Thermotogati Thermotogota Thermotogae Petrotogales Oceanotoga WP 109604225.1 317 aa 317 aa
BacClassIA Thermotogati Thermotogota Thermotogae Petrotogales Geotoga petraea WP 091403356.1 309 aa 309 aa
BacClassIA Thermotogati Thermotogota Thermotogae Petrotogales Defluviitoga tunisiensis WP 045088338.1 316 aa 316 aa
BacClassIA Thermotogati Thermotogota Thermotogae Petrotogales Petrotoga sp WP 121957719.1 316 aa 316 aa
BacClassIA Thermotogati Thermotogota Thermotogae Petrotogales Petrotoga WP 103078991.1 316 aa 316 aa
BacClassIA Thermotogati Thermotogota Thermotogae Petrotogales Petrotoga sp WP 113075190.1 316 aa 316 aa
BacClassIA Bacillati Bacillota Clostridia Eubacteriales Peptococcus simiae WP 408976942.1 314 aa 314 aa
BacClassIA Bacillati Bacillota Negativicutes Veillonellales Dialister micraerophilus WP 007554140.1 317 aa 317 aa
BacClassIA Bacillati Bacillota Negativicutes Veillonellales Megasphaera WP 091647732.1 321 aa 321 aa
BacClassIA Bacillati Bacillota Negativicutes Veillonellales Megasphaera hominis WP 186502931.1 321 aa 321 aa
BacClassIA Bacillati Bacillota Negativicutes Phascolarctobacterium sp MDO4178038.1 320 aa 320 aa
BacClassIA Bacillati Bacillota Negativicutes Acidaminococcus fermentans WP 281739367.1 321 aa 321 aa
BacClassIA Bacillati Bacillota Negativicutes Veillonellales Massilibacillus massiliensis WP 110954260.1 317 aa 317 aa
BacClassIA Bacillati Bacillota Negativicutes Selenomonadales Propionispira raffinosivorans WP 019554008.1 316 aa 316 aa
BacClassIA Bacillati Bacillota Negativicutes Selenomonadales Pectinatus cerevisiiphilus WP 132549591.1 324 aa 324 aa
BacClassIA Bacillati Bacillota Negativicutes Selenomonadales Megamonas hypermegale WP 087386962.1 324 aa 324 aa
BacClassIA Bacillati Bacillota Negativicutes Selenomonadales Anaerovibrio sp WP 290711393.1 316 aa 316 aa
BacClassIA Bacillati Bacillota Negativicutes Selenomonadales Selenomonadaceae bacterium MBQ1940568.1 317 aa 317 aa
BacClassIA Bacillati Bacillota Negativicutes Selenomonadales Anaerovibrio WP 300089233.1 320 aa 320 aa
BacClassIA Bacillati Bacillota Negativicutes Veillonellales Veillonellaceae bacterium MCI5567099.1 315 aa 315 aa
BacClassIA Bacillati Bacillota Negativicutes Selenomonadales Selenomonas ruminantium WP 074570771.1 315 aa 315 aa
BacClassIA Bacillati Bacillota Negativicutes Selenomonadales Mitsuokella multacida HBQ30602.1 316 aa 316 aa
BacClassIA Bacillati Bacillota Negativicutes Selenomonadales uncultured Mitsuokella WP 298593151.1 316 aa 316 aa
BacClassIA Bacillati Bacillota Negativicutes Selenomonadales Centipeda periodontii WP 006306206.1 317 aa 317 aa
BacClassIA Bacillati Bacillota Negativicutes Selenomonadales Selenomonas sputigena WP 368846286.1 313 aa 313 aa
BacClassIA Bacillati Bacillota Negativicutes Selenomonadales Selenomonas sp WP 338622833.1 314 aa 314 aa
BacClassIA Bacillati Bacillota Negativicutes Selenomonadales Selenomonadaceae bacterium MBQ9478333.1 324 aa 324 aa
BacClassIA Bacillati Bacillota Negativicutes Selenomonadales Schwartzia sp MBQ5758883.1 316 aa 316 aa
BacClassIA Bacillati Bacillota Negativicutes Selenomonadales Schwartzia succinivorans WP 273037786.1 314 aa 314 aa
BacClassIA Bacillati Bacillota Negativicutes Selenomonadales Schwartzia sp MBQ9365281.1 314 aa 314 aa
BacClassIA Bacillati Bacillota Dethiobacteria Dethiobacter sp RJX24749.1 315 aa 315 aa
BacClassIA Bacillati Bacillota Dethiobacteria Candidatus Contubernalis WP 241421960.1 322 aa 322 aa
BacClassIA Bacillati Bacillota Dethiobacteria Dethiobacter alkaliphilus WP 008518136.1 313 aa 313 aa
BacClassIA Bacillati Bacillota Dethiobacteria Dethiobacteraceae bacterium MGI6658609.1 313 aa 313 aa
BacClassIA Bacillati Bacillota Dethiobacteria Dethiobacter sp MBS4023910.1 321 aa 321 aa
BacClassIA Bacillati Bacillota Clostridia Natranaerobiales Natranaerofaba carboxydovora WP 241079243.1 315 aa 315 aa
BacClassIA Bacillati Bacillota Clostridia Natranaerobiales Natranaerobius trueperi WP 089023677.1 315 aa 315 aa
BacClassIA Bacillati Bacillota Clostridia Natranaerobiales Natranaerobius thermophilus WP 012446551.1 322 aa 322 aa
BacClassIA Bacillati Bacillota Desulfuribacillia Desulfuribacillus stibiiarsenatis WP 069703322.1 317 aa 317 aa
BacClassIA Bacillati Bacillota Desulfuribacillia Desulfuribacillus alkaliarsenatis WP 069644320.1 317 aa 317 aa
BacClassIA Bacillati Bacillota Bacilli Bacillales Insulibacter thermoxylanivorax WP 200966661.1 315 aa 315 aa
BacClassIA Bacillati Bacillota Bacilli Bacillales Cohnella pontilimi WP 136779650.1 318 aa 318 aa
BacClassIA Bacillati Bacillota Bacilli Bacillales Sulfoacidibacillus ferrooxidans WP 241712286.1 315 aa 315 aa
BacClassIA Bacillati Bacillota Bacilli Bacillales Ferroacidibacillus organovorans WP 067713416.1 315 aa 315 aa
BacClassIA Bacillati Bacillota Bacilli Bacillales Alicyclobacillus cellulosilyticus WP 188882487.1 314 aa 314 aa
BacClassIA Bacillati Bacillota Bacilli Bacillales Alicyclobacillus sp WP 198852160.1 314 aa 314 aa
BacClassIA Bacillati Bacillota Bacilli Bacillales Alicyclobacillus sp OFW82196.1 314 aa 314 aa
BacClassIA Bacillati Bacillota Bacilli Bacillales Alicyclobacillus vulcanalis WP 076346752.1 314 aa 314 aa
BacClassIA Bacillati Bacillota Bacilli Bacillales Alicyclobacillus sp MCL6513751.1 314 aa 314 aa
BacClassIA Bacillati Bacillota Bacilli Bacillales Alicyclobacillaceae bacterium RIV21754.1 314 aa 314 aa
BacClassIA Bacillati Bacillota Bacilli Bacillales Alicyclobacillus mengziensis WP 206657275.1 314 aa 314 aa
BacClassIA Bacillati Bacillota Bacilli Bacillales Alicyclobacillus contaminans WP 026974431.1 314 aa 314 aa
BacClassIA Bacillati Bacillota Bacilli Bacillales Kyrpidia tusciae WP 013074142.1 316 aa 316 aa
BacClassIA Bacillati Bacillota Bacilli Bacillales Fodinisporobacter ferrooxydans WP 347438502.1 313 aa 313 aa
BacClassIA Bacillati Bacillota Bacilli Bacillales Effusibacillus dendaii WP 200757373.1 314 aa 314 aa
BacClassIA Bacillati Bacillota Bacilli unclassified Bacilli Bacilli bacterium MDB5083615.1 315 aa 315 aa
BacClassIA Bacillati Bacillota Bacilli Bacillales Collibacillus ludicampi WP 282199186.1 315 aa 315 aa
BacClassIA Bacillati Bacillota Bacilli Bacillales Tumebacillus avium WP 087456368.1 317 aa 317 aa
BacClassIA Bacillati Bacillota Bacilli Bacillales Tumebacillus lacus WP 267153223.1 316 aa 316 aa
BacClassIA Bacillati Bacillota Alicyclobacillia Bacillota Tumebacillaceae bacterium MGB8954888.1 318 aa 318 aa
BacClassIA Bacillati Bacillota Bacilli unclassified Bacilli Bacilli bacterium HEU4965409.1 317 aa 317 aa
BacClassIA Bacillati Bacillota Bacilli Bacillales Tumebacillus amylolyticus WP 201638091.1 319 aa 319 aa
BacClassIA Bacillati Bacillota Clostridia Eubacteriales Syntrophothermus WP 013174309.1 316 aa 316 aa
BacClassIA Bacillati Bacillota Clostridia Thermoanaerobacterales Gelria sp HHV15827.1 317 aa 317 aa
BacClassIA Bacillati Bacillota Clostridia Eubacteriales Thermosyntropha lipolytica WP 073089042.1 316 aa 316 aa
BacClassIA Bacillati Bacillota Clostridia Eubacteriales Syntrophomonas zehnderi WP 046495321.1 317 aa 317 aa
BacClassIA Bacillati Bacillota Clostridia Eubacteriales Syntrophomonas wolfei WP 011639537.1 317 aa 317 aa
BacClassIA Bacillati Bacillota Clostridia Eubacteriales Candidatus Syntrophocurvum WP 156204313.1 318 aa 318 aa
BacClassIA Bacillati Bacillota Clostridia Neomoorellales Neomoorella sulfitireducens WP 258360699.1 315 aa 315 aa
BacClassIA Bacillati Bacillota Clostridia Neomoorellales Moorella sp WP 109208110.1 315 aa 315 aa
BacClassIA Bacillati Bacillota Clostridia Neomoorellales Thermanaeromonas sp WP 173296841.1 315 aa 315 aa
BacClassIA Bacillati Bacillota Clostridia Neomoorellales Thermanaeromonas sp WP 297199963.1 314 aa 314 aa
BacClassIA Bacillati Bacillota Clostridia Neomoorellales Moorellaceae bacterium MGB9660441.1 315 aa 315 aa
BacClassIA Bacillati Bacillota Clostridia Neomoorellales Calderihabitans maritimus WP 088553158.1 315 aa 315 aa
BacClassIA Bacillati Bacillota Clostridia Neomoorellales Metallumcola ferriviriculae WP 366923352.1 315 aa 315 aa
BacClassIA Bacillati Bacillota Clostridia Neomoorellales Desulfitibacter alkalitolerans WP 028306559.1 315 aa 315 aa
BacClassIA Bacillati Bacillota Clostridia Neomoorellales Zhaonellaceae bacterium MGI6330844.1 315 aa 315 aa
BacClassIA Bacillati Bacillota Clostridia Neomoorellales Zhaonella formicivorans WP 227763966.1 315 aa 315 aa
BacClassIA Bacillati Bacillota Clostridia Eubacteriales Dehalobacterium formicoaceticum WP 089609374.1 315 aa 315 aa
BacClassIA Bacillati Bacillota Clostridia Eubacteriales Candidatus Formimonas WP 148136315.1 315 aa 315 aa
BacClassIA Bacillati Bacillota Desulfitisporia Desulfitispora alkaliphila WP 429158042.1 315 aa 315 aa
BacClassIA Bacillati Bacillota Clostridia Eubacteriales Desulfitobacterium sp NMA69023.1 313 aa 313 aa
BacClassIA Bacillati Bacillota Clostridia Eubacteriales Desulfitobacterium metallireducens WP 006717607.1 313 aa 313 aa
BacClassIA Bacillati Bacillota Clostridia Eubacteriales Desulfitobacteriaceae bacterium MDI6812107.1 313 aa 313 aa
BacClassIA Bacillati Bacillota Clostridia Eubacteriales Acididesulfobacillus acetoxydans WP 240983645.1 313 aa 313 aa
BacClassIA Bacillati Bacillota Clostridia Eubacteriales Desulfosporosinus sp WP 034599356.1 312 aa 312 aa
BacClassIA Bacillati Bacillota Clostridia Eubacteriales Desulfosporosinus fructosivorans WP 135549502.1 311 aa 311 aa
BacClassIA Bacillati Bacillota Clostridia Eubacteriales Paradesulfitobacterium ferrireducens WP 206811376.1 313 aa 313 aa
BacClassIA Bacillati Bacillota Clostridia Eubacteriales Dehalobacter WP 019224828.1 313 aa 313 aa
BacClassIA Bacillati Bacillota Clostridia Eubacteriales Dehalobacter sp WP 337986559.1 313 aa 313 aa
BacClassIA Bacillati Bacillota Clostridia Eubacteriales Syntrophobotulus glycolicus WP 013623437.1 313 aa 313 aa
BacClassIA Bacillati Bacillota Clostridia Eubacteriales Gracilibacteraceae bacterium MDR1603502.1 314 aa 314 aa
BacClassIA Bacillati Bacillota Clostridia Eubacteriales Gracilibacteraceae bacterium MDR0433890.1 314 aa 314 aa
BacClassIA Bacillati Bacillota Clostridia Eubacteriales Thermanaerosceptrum fracticalcis WP 034421250.1 312 aa 312 aa
BacClassIA Bacillati Bacillota Clostridia Thermoanaerobacterales Thermacetogenium phaeum WP 015051857.1 326 aa 326 aa
BacClassIA Bacillati Bacillota Clostridia Thermoanaerobacterales Syntrophaceticus sp MGI6404987.1 326 aa 326 aa
BacClassIA Bacillati Bacillota Clostridia Thermoanaerobacterales Syntrophaceticus sp MDD2555622.1 326 aa 326 aa
BacClassIA Bacillati Bacillota Clostridia Eubacteriales Heliophilum fasciatum WP 131918050.1 315 aa 315 aa
BacClassIA Bacillati Bacillota Clostridia Eubacteriales Heliobacterium WP 155475562.1 315 aa 315 aa
BacClassIA Bacillati Bacillota Clostridia Eubacteriales Heliobacteriaceae bacterium MDD2420775.1 315 aa 315 aa
BacClassIA Bacillati Bacillota Clostridia unclassified Clostridia Clostridia bacterium MDA8235807.1 315 aa 315 aa
BacClassIA Bacillati Bacillota Clostridia Eubacteriales Thermincola WP 013119053.1 315 aa 315 aa
BacClassIA Bacillati Bacillota Clostridia Eubacteriales Thermincola sp MDT3698758.1 315 aa 315 aa
BacClassIA Bacillati Bacillota Clostridia Eubacteriales Phosphitispora fastidiosa WP 231684643.1 315 aa 315 aa
BacClassIA Bacillati Bacillota Clostridia Thermoanaerobacterales Carboxydothermus islandicus WP 075865722.1 313 aa 313 aa
BacClassIA Bacillati Bacillota Clostridia Eubacteriales Desulfotomaculum sp WP 333529971.1 316 aa 316 aa
BacClassIA Bacillati Bacillota Clostridia Eubacteriales Desulforamulus aeronauticus WP 072912737.1 315 aa 315 aa
BacClassIA Bacillati Bacillota Clostridia Eubacteriales Desulfofalx alkaliphila WP 031516459.1 317 aa 317 aa
BacClassIA Bacillati Bacillota Clostridia Eubacteriales Desulfovectis peckii BGR85067.1 316 aa 316 aa
BacClassIA Bacillati Bacillota Clostridia Eubacteriales Desulforadius tongensis WP 204617757.1 316 aa 316 aa
BacClassIA Bacillati Bacillota Clostridia Thermoanaerobacterales unclassified Candidatus WP 425622202.1 315 aa 315 aa
BacClassIA Bacillati Bacillota Clostridia Thermoanaerobacterales Desulforudis sp MBE0465994.1 315 aa 315 aa
BacClassIA Bacillati Bacillota Clostridia Thermoanaerobacterales Candidatus Desulforudis WP 012301231.1 318 aa 318 aa
BacClassIA Bacillati Bacillota Clostridia Thermoanaerobacterales Ammonifex thiophilus WP 115791632.1 315 aa 315 aa
BacClassIA Bacillati Bacillota Clostridia Thermoanaerobacterales Thermodesulfitimonas sp MGQ9512327.1 308 aa 308 aa
BacClassIA Bacillati Bacillota Clostridia Thermoanaerobacterales Thermodesulfitimonas autotrophica WP 123930612.1 315 aa 315 aa
BacClassIA Bacillati Bacillota Clostridia Eubacteriales Desulfolucanica intricata WP 066634297.1 315 aa 315 aa
BacClassIA Bacillati Bacillota Clostridia Eubacteriales Desulfofarcimen acetoxidans WP 015755880.1 315 aa 315 aa
BacClassIA Bacillati Bacillota Clostridia Eubacteriales Desulfocucumis palustris WP 104371323.1 316 aa 316 aa
BacClassIA Bacillati Bacillota Clostridia Eubacteriales Desulfofundulus thermobenzoicus WP 152947718.1 315 aa 315 aa
BacClassIA Bacillati Bacillota Clostridia Eubacteriales Desulfotruncus arcticus WP 092472597.1 314 aa 314 aa
BacClassIA Bacillati Bacillota Clostridia Eubacteriales Sporotomaculum syntrophicum WP 161822519.1 314 aa 314 aa
BacClassIA Bacillati Bacillota Clostridia Eubacteriales Desulfallas thermosapovorans WP 166510834.1 314 aa 314 aa
BacClassIA Bacillati Bacillota Clostridia Eubacteriales Pelotomaculum WP 240084247.1 316 aa 316 aa
BacClassIA Bacillati Bacillota Clostridia Eubacteriales Pelotomaculum sp NPV74060.1 315 aa 315 aa
BacClassIA Bacillati Bacillota Clostridia Eubacteriales Pelotomaculum sp MGI6492617.1 314 aa 314 aa
BacClassIA Bacillati Bacillota Clostridia Eubacteriales Desulfotomaculaceae bacterium MDD2443559.1 313 aa 313 aa
BacClassIA Bacillati Bacillota Clostridia Eubacteriales Pelotomaculum propionicicum WP 421071973.1 314 aa 314 aa
BacClassIA Bacillati Bacillota Clostridia Eubacteriales Pelotomaculum propionicicum WP 134213013.1 314 aa 314 aa
BacClassIA Bacillati Bacillota Clostridia Eubacteriales Desulfonispora thiosulfatigenes WP 084052563.1 314 aa 314 aa
BacClassIA Bacillati Bacillota Clostridia Eubacteriales Proteinivorax tanatarense WP 350343732.1 316 aa 316 aa
BacClassIA Bacillati Bacillota Clostridia Eubacteriales Anaerobranca gottschalkii WP 091351314.1 315 aa 315 aa
BacClassIA Bacillati Bacillota Clostridia Eubacteriales Alkalicella caledoniensis WP 213168943.1 315 aa 315 aa
BacClassIA Bacillati Bacillota Clostridia Koleobacterales Koleobacter methoxysyntrophicus WP 206708729.1 314 aa 314 aa
BacClassIA Bacillati Bacillota Clostridia Thermosediminibacterales Caldanaerovirga acetigignens WP 073254073.1 312 aa 312 aa
BacClassIA Bacillati Bacillota Clostridia Thermosediminibacterales Tepidanaerobacter acetatoxydans WP 013777227.1 313 aa 313 aa
BacClassIA Bacillati Bacillota Clostridia Thermoanaerobacterales Biomaibacter acetigenes WP 120765236.1 312 aa 312 aa
BacClassIA Bacillati Bacillota Clostridia Lutisporales Lutispora sp WP 372995171.1 315 aa 315 aa
BacClassIA Bacillati Bacillota Clostridia Lutisporales Lutispora saccharofermentans WP 255228123.1 315 aa 315 aa
BacClassIA Bacillati Bacillota Clostridia Thermoanaerobacterales Calorimonas adulescens WP 420837948.1 316 aa 316 aa
BacClassIA Bacillati Bacillota Clostridia Thermoanaerobacterales Caldanaerobius fijiensis WP 073344055.1 315 aa 315 aa
BacClassIA Bacillati Bacillota Clostridia Thermoanaerobacterales Caldanaerobacter WP 011026572.1 316 aa 316 aa
BacClassIA Bacillati Bacillota Clostridia Thermoanaerobacterales Aceticella autotrophica WP 284680168.1 316 aa 316 aa

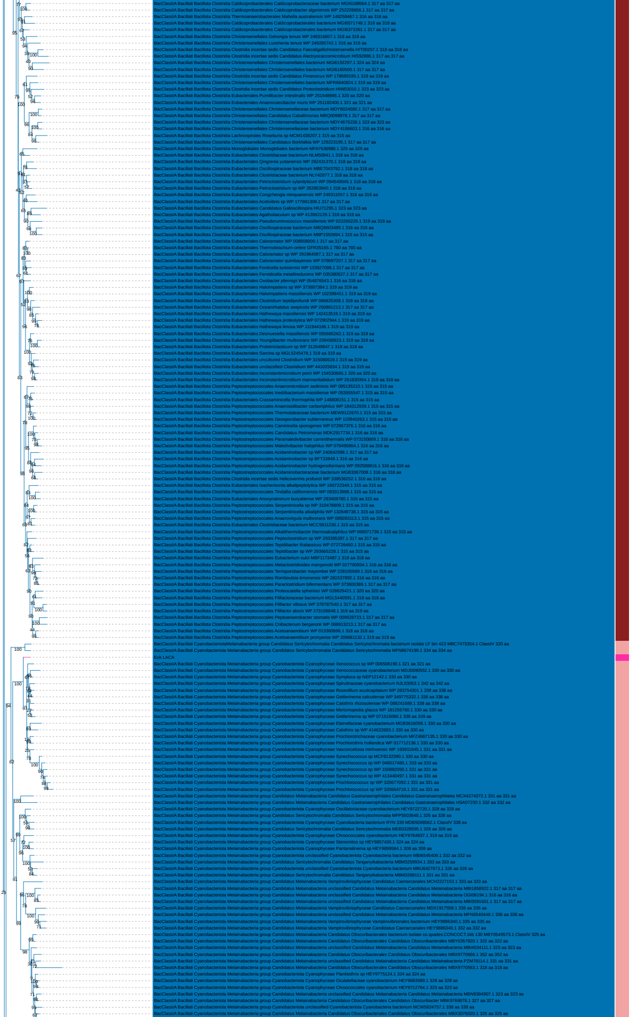

BacClassIA Bacilliati Bacillota Clostridia Caldicoprobacterales Caldicoprobacteraceae bacterium MGI6188564.1 317 aa 317 aa
BacClassIA Bacilliati Bacillota Clostridia Caldicoprobacterales Caldicoprobacter algeriensis WP 252228656.1 317 aa 317 aa
BacClassIA Bacilliati Bacillota Clostridia Thermoanaerobacterales Mahella australiensis WP 148258467.1 316 aa 316 aa
BacClassIA Bacilliati Bacillota Clostridia Caldicoprobacterales Caldicoprobacterales bacterium MGI6571748.1 318 aa 318 aa
BacClassIA Bacilliati Bacillota Clostridia Caldicoprobacterales Caldicoprobacterales bacterium MGI6373361.1 317 aa 317 aa
BacClassIA Bacilliati Bacillota Clostridia Christensenellales Gehongia tenuis WP 249316807.1 318 aa 318 aa
BacClassIA Bacilliati Bacillota Clostridia Christensenellales Luoshenia tenuis WP 249285742.1 316 aa 316 aa
BacClassIA Bacilliati Bacillota Clostridia Clostridia incertae sedis Candidatus Faecaligallichristensenella HIT00257.1 318 aa 318 aa
BacClassIA Bacilliati Bacillota Clostridia Clostridia incertae sedis Candidatus Alectryocaccomicrobium HIS92886.1 317 aa 317 aa
BacClassIA Bacilliati Bacillota Clostridia Christensenellales Christensenellales bacterium MGI6192297.1 324 aa 324 aa
BacClassIA Bacilliati Bacillota Clostridia Christensenellales Christensenellales bacterium MGI6160500.1 317 aa 317 aa
BacClassIA Bacilliati Bacillota Clostridia Clostridia incertae sedis Candidatus Fimenecus WP 178689195.1 319 aa 319 aa
BacClassIA Bacilliati Bacillota Clostridia Christensenellales Christensenellales bacterium MFR6640824.1 319 aa 319 aa
BacClassIA Bacilliati Bacillota Clostridia Clostridia incertae sedis Candidatus Protoclostridium HIW03010.1 323 aa 323 aa
BacClassIA Bacilliati Bacillota Clostridia Eubacteriales Pumilibacter intestinalis WP 251548665.1 320 aa 320 aa
BacClassIA Bacilliati Bacillota Clostridia Eubacteriales Anaerocaecibacter muris WP 251182400.1 321 aa 321 aa
BacClassIA Bacilliati Bacillota Clostridia Christensenellales Christensenellaceae bacterium MDY6024590.1 317 aa 317 aa
BacClassIA Bacilliati Bacillota Clostridia Christensenellales Candidatus Caballimonas MBQ0098978.1 317 aa 317 aa
BacClassIA Bacilliati Bacillota Clostridia Christensenellales Christensenellaceae bacterium MDY4676238.1 323 aa 323 aa
BacClassIA Bacilliati Bacillota Clostridia Christensenellales Christensenellaceae bacterium MDY4186603.1 316 aa 316 aa
BacClassIA Bacilliati Bacillota Clostridia Lachnospirales Roseburia sp MCM1438207.1 315 aa 315 aa
BacClassIA Bacilliati Bacillota Clostridia Christensenellales Candidatus Borkfalkia WP 129223195.1 317 aa 317 aa
BacClassIA Bacilliati Bacillota Clostridia Monoglobales Monoglobales bacterium MFA7636980.1 325 aa 325 aa
BacClassIA Bacilliati Bacillota Clostridia Eubacteriales Clostridiaceae bacterium NLM50941.1 318 aa 318 aa
BacClassIA Bacilliati Bacillota Clostridia Eubacteriales Qingrenia yutianensis WP 262431370.1 318 aa 318 aa
BacClassIA Bacilliati Bacillota Clostridia Eubacteriales Oscillospiraceae bacterium MBE7043792.1 318 aa 318 aa
BacClassIA Bacilliati Bacillota Clostridia Eubacteriales Clostridiaceae bacterium NLY42877.1 318 aa 318 aa
BacClassIA Bacilliati Bacillota Clostridia Eubacteriales Petroclostridium xylanilyticum WP 094549045.1 318 aa 318 aa
BacClassIA Bacilliati Bacillota Clostridia Eubacteriales Petroclostridium sp WP 282863940.1 318 aa 318 aa
BacClassIA Bacilliati Bacillota Clostridia Eubacteriales Congzhengia minquanensis WP 249311057.1 316 aa 316 aa
BacClassIA Bacilliati Bacillota Clostridia Eubacteriales Acetivibrio sp WP 177691308.1 317 aa 317 aa
BacClassIA Bacilliati Bacillota Clostridia Eubacteriales Candidatus Gallscillospira HIU71295.1 323 aa 323 aa
BacClassIA Bacilliati Bacillota Clostridia Eubacteriales Agathobaculum sp WP 412862129.1 319 aa 319 aa
BacClassIA Bacilliati Bacillota Clostridia Eubacteriales Pseudoruminococcus massiliensis WP 022265226.1 319 aa 319 aa
BacClassIA Bacilliati Bacillota Clostridia Eubacteriales Oscillospiraceae bacterium MBQ8603489.1 316 aa 316 aa
BacClassIA Bacilliati Bacillota Clostridia Eubacteriales Oscillospiraceae bacterium MBP1550694.1 315 aa 315 aa
BacClassIA Bacilliati Bacillota Clostridia Eubacteriales Caloramator WP 008908000.1 317 aa 317 aa
BacClassIA Bacilliati Bacillota Clostridia Eubacteriales Thermobrachium celere GFR35165.1 780 aa 780 aa
BacClassIA Bacilliati Bacillota Clostridia Eubacteriales Caloramator sp WP 291964087.1 317 aa 317 aa
BacClassIA Bacilliati Bacillota Clostridia Eubacteriales Caloramator quimbayensis WP 078697207.1 317 aa 317 aa
BacClassIA Bacilliati Bacillota Clostridia Eubacteriales Fonticella tunisiensis WP 133627098.1 317 aa 317 aa
BacClassIA Bacilliati Bacillota Clostridia Eubacteriales Fervidicella metallireducens WP 035380627.1 317 aa 317 aa
BacClassIA Bacilliati Bacillota Clostridia Eubacteriales Oxobacter pfennigii WP 054876543.1 316 aa 316 aa
BacClassIA Bacilliati Bacillota Clostridia Eubacteriales Haloimpatiens sp WP 373897384.1 319 aa 319 aa
BacClassIA Bacilliati Bacillota Clostridia Eubacteriales Haloimpatiens massiliensis WP 102399451.1 319 aa 319 aa
BacClassIA Bacilliati Bacillota Clostridia Eubacteriales Clostridium tepidiprofundi WP 066825308.1 318 aa 318 aa
BacClassIA Bacilliati Bacillota Clostridia Eubacteriales Oceanirhabdus seepicola WP 250861213.1 317 aa 317 aa
BacClassIA Bacilliati Bacillota Clostridia Eubacteriales Hathewaya massiliensis WP 142413519.1 319 aa 319 aa
BacClassIA Bacilliati Bacillota Clostridia Eubacteriales Hathewaya proteolytica WP 072902944.1 319 aa 319 aa
BacClassIA Bacilliati Bacillota Clostridia Eubacteriales Hathewaya limosa WP 111944166.1 319 aa 319 aa
BacClassIA Bacilliati Bacillota Clostridia Eubacteriales Desnuesiella massiliensis WP 055665262.1 319 aa 319 aa
BacClassIA Bacilliati Bacillota Clostridia Eubacteriales Youngiibacter multivorans WP 209458923.1 319 aa 319 aa
BacClassIA Bacilliati Bacillota Clostridia Eubacteriales Proteiniclasticum sp WP 312649647.1 318 aa 318 aa
BacClassIA Bacilliati Bacillota Clostridia Eubacteriales Sarcina sp MGL5245478.1 319 aa 319 aa
BacClassIA Bacilliati Bacillota Clostridia Eubacteriales uncultured Clostridium WP 315080619.1 319 aa 319 aa
BacClassIA Bacilliati Bacillota Clostridia Eubacteriales unclassified Clostridium WP 441025634.1 319 aa 319 aa
BacClassIA Bacilliati Bacillota Clostridia Eubacteriales Inconstantimicrobium porci WP 154530685.1 320 aa 320 aa
BacClassIA Bacilliati Bacillota Clostridia Eubacteriales Inconstantimicrobium mannanitabidum WP 261830364.1 318 aa 318 aa
BacClassIA Bacilliati Bacillota Clostridia Peptostreptococcales Anaeromicrobium sediminis WP 095135210.1 315 aa 315 aa
BacClassIA Bacilliati Bacillota Clostridia Peptostreptococcales Ineditibacterium massiliense WP 053955547.1 315 aa 315 aa
BacClassIA Bacilliati Bacillota Clostridia Eubacteriales Crassaminicella thermophila WP 148808151.1 315 aa 315 aa
BacClassIA Bacilliati Bacillota Clostridia Peptostreptococcales Anaerosolibacter carboniphilus WP 184312939.1 315 aa 315 aa
BacClassIA Bacilliati Bacillota Clostridia Peptostreptococcales Thermotaleaceae bacterium MEW9122670.1 315 aa 315 aa
BacClassIA Bacilliati Bacillota Clostridia Peptostreptococcales Geosporobacter subterraneus WP 110940263.1 315 aa 315 aa
BacClassIA Bacilliati Bacillota Clostridia Peptostreptococcales Caminicella sporogenes WP 072967376.1 316 aa 316 aa
BacClassIA Bacilliati Bacillota Clostridia Peptostreptococcales Candidatus Petromonas MDK2917734.1 316 aa 316 aa
BacClassIA Bacilliati Bacillota Clostridia Peptostreptococcales Paramaledivibacter caminithermalis WP 073150889.1 316 aa 316 aa
BacClassIA Bacilliati Bacillota Clostridia Peptostreptococcales Maledivibacter halophilus WP 079495864.1 316 aa 316 aa
BacClassIA Bacilliati Bacillota Clostridia Peptostreptococcales Acidaminobacter sp WP 240842998.1 317 aa 317 aa
BacClassIA Bacilliati Bacillota Clostridia Peptostreptococcales Acidaminobacter sp BFT33849.1 316 aa 316 aa
BacClassIA Bacilliati Bacillota Clostridia Peptostreptococcales Acidaminobacter hydrogenoformans WP 092588916.1 316 aa 316 aa
BacClassIA Bacilliati Bacillota Clostridia Peptostreptococcales Acidaminobacteraceae bacterium MGB3367008.1 316 aa 316 aa
BacClassIA Bacilliati Bacillota Clostridia Clostridia incertae sedis Helicovermis profundi WP 338536252.1 316 aa 316 aa
BacClassIA Bacilliati Bacillota Clostridia Peptostreptococcales Isachenkonia alkalipeptolytica WP 160722349.1 315 aa 315 aa
BacClassIA Bacilliati Bacillota Clostridia Peptostreptococcales Tindallia californiensis WP 093313988.1 315 aa 315 aa
BacClassIA Bacilliati Bacillota Clostridia Eubacteriales Anoxynatronum buryatiense WP 283408780.1 315 aa 315 aa
BacClassIA Bacilliati Bacillota Clostridia Peptostreptococcales Serpentinicella sp WP 310478809.1 315 aa 315 aa
BacClassIA Bacilliati Bacillota Clostridia Peptostreptococcales Serpentinicella alkaliphila WP 132848738.1 315 aa 315 aa
BacClassIA Bacilliati Bacillota Clostridia Peptostreptococcales Anaerovirgula multivorans WP 089283113.1 315 aa 315 aa
BacClassIA Bacilliati Bacillota Clostridia Eubacteriales Clostridiaceae bacterium MCC5911230.1 315 aa 315 aa
BacClassIA Bacilliati Bacillota Clostridia Peptostreptococcales Alkalithermobacter thermoalcaliphilus WP 066071736.1 315 aa 315 aa
BacClassIA Bacilliati Bacillota Clostridia Peptostreptococcales Peptoclostridium sp WP 293395397.1 317 aa 317 aa
BacClassIA Bacilliati Bacillota Clostridia Peptostreptococcales Tepidibacter thalassicus WP 072726460.1 315 aa 315 aa
BacClassIA Bacilliati Bacillota Clostridia Peptostreptococcales Tepidibacter sp WP 293665228.1 315 aa 315 aa
BacClassIA Bacilliati Bacillota Clostridia Peptostreptococcales Eubacterium sulci MBF1173487.1 318 aa 318 aa
BacClassIA Bacilliati Bacillota Clostridia Peptostreptococcales Metaclostridioides mangenotii WP 027700934.1 316 aa 316 aa
BacClassIA Bacilliati Bacillota Clostridia Peptostreptococcales Terrisporobacter mayombei WP 228105569.1 316 aa 316 aa
BacClassIA Bacilliati Bacillota Clostridia Peptostreptococcales Romboutsia timonensis WP 281537892.1 316 aa 316 aa
BacClassIA Bacilliati Bacillota Clostridia Peptostreptococcales Paraclostridium bifermentans WP 373600389.1 317 aa 317 aa
BacClassIA Bacilliati Bacillota Clostridia Peptostreptococcales Proteocatella sphenisci WP 028829421.1 320 aa 320 aa
BacClassIA Bacilliati Bacillota Clostridia Peptostreptococcales Filifactoraceae bacterium MGL5440591.1 318 aa 318 aa
BacClassIA Bacilliati Bacillota Clostridia Peptostreptococcales Filifactor villosus WP 379787540.1 317 aa 317 aa
BacClassIA Bacilliati Bacillota Clostridia Peptostreptococcales Filifactor alocis WP 273159848.1 319 aa 319 aa
BacClassIA Bacilliati Bacillota Clostridia Peptostreptococcales Peptoanaerobacter stomatis WP 009528723.1 317 aa 317 aa
BacClassIA Bacilliati Bacillota Clostridia Peptostreptococcales Cribacterium bergeronii WP 068913213.1 317 aa 317 aa
BacClassIA Bacilliati Bacillota Clostridia Peptostreptococcales Acetoanaerobium WP 013360696.1 318 aa 318 aa
BacClassIA Bacilliati Bacillota Clostridia Peptostreptococcales Acetoanaerobium pronyense WP 209661132.1 319 aa 319 aa
BacClassIA Bacilliati Cyanobacteriota Melainabacteria group Candidatus Sericytochromatia bacterium isolate LF bin 423 MBC7473354.1 ClassIV 320 aa
BacClassIA Bacilliati Cyanobacteriota Melainabacteria group Candidatus Sericytochromatia Candidatus Sericytochromatia MFN8674199.1 334 aa 334 aa
Euk LACA
BacClassIA Bacilliati Cyanobacteriota Melainabacteria group Cyanobacteriota Cyanophyceae Xenococcus sp WP 006508190.1 321 aa 321 aa
BacClassIA Bacilliati Cyanobacteriota Melainabacteria group Cyanobacteriota Cyanophyceae Xenococcaceae cyanobacterium MDJ0690552.1 330 aa 330 aa
BacClassIA Bacilliati Cyanobacteriota Melainabacteria group Cyanobacteriota Cyanophyceae Symploca sp NEP12142.1 330 aa 330 aa
BacClassIA Bacilliati Cyanobacteriota Melainabacteria group Cyanobacteriota Cyanophyceae Spirulinaceae cyanobacterium NJL03053.1 342 aa 342 aa
BacClassIA Bacilliati Cyanobacteriota Melainabacteria group Cyanobacteriota Cyanophyceae Roseofilum acuticapitatum WP 283754301.1 338 aa 338 aa
BacClassIA Bacilliati Cyanobacteriota Melainabacteria group Cyanobacteriota Cyanophyceae Geitlerinema calcuttense WP 349775332.1 338 aa 338 aa
BacClassIA Bacilliati Cyanobacteriota Melainabacteria group Cyanobacteriota Cyanophyceae Calothrix rhizosoleniae WP 088241689.1 338 aa 338 aa
BacClassIA Bacilliati Cyanobacteriota Melainabacteria group Cyanobacteriota Cyanophyceae Merismopedia glauca WP 181256760.1 330 aa 330 aa
BacClassIA Bacilliati Cyanobacteriota Melainabacteria group Cyanobacteriota Cyanophyceae Geitlerinema sp WP 071515090.1 339 aa 339 aa
BacClassIA Bacilliati Cyanobacteriota Melainabacteria group Cyanobacteriota Cyanophyceae Elainellaceae cyanobacterium MGB3616098.1 330 aa 330 aa
BacClassIA Bacilliati Cyanobacteriota Melainabacteria group Cyanobacteriota Cyanophyceae Calothrix sp WP 414622883.1 330 aa 330 aa
BacClassIA Bacilliati Cyanobacteriota Melainabacteria group Cyanobacteriota Cyanophyceae Prochlorotrichaceae cyanobacterium MFZ4667135.1 330 aa 330 aa
BacClassIA Bacilliati Cyanobacteriota Melainabacteria group Cyanobacteriota Cyanophyceae Prochlorothrix hollandica WP 017712136.1 330 aa 330 aa
BacClassIA Bacilliati Cyanobacteriota Melainabacteria group Cyanobacteriota Cyanophyceae Vasconcelosia minhoensis WP 193911645.1 331 aa 331 aa
BacClassIA Bacilliati Cyanobacteriota Melainabacteria group Cyanobacteriota Cyanophyceae Synechococcus sp MCF8132390.1 330 aa 330 aa
BacClassIA Bacilliati Cyanobacteriota Melainabacteria group Cyanobacteriota Cyanophyceae Synechococcus sp WP 048017480.1 333 aa 333 aa
BacClassIA Bacilliati Cyanobacteriota Melainabacteria group Cyanobacteriota Cyanophyceae Synechococcus sp WP 150882050.1 331 aa 331 aa
BacClassIA Bacilliati Cyanobacteriota Melainabacteria group Cyanobacteriota Cyanophyceae Synechococcus sp WP 413440497.1 331 aa 331 aa
BacClassIA Bacilliati Cyanobacteriota Melainabacteria group Cyanobacteriota Cyanophyceae Prochlorococcus sp WP 320677092.1 331 aa 331 aa
BacClassIA Bacilliati Cyanobacteriota Melainabacteria group Cyanobacteriota Cyanophyceae Prochlorococcus sp WP 320664718.1 331 aa 331 aa
BacClassIA Bacilliati Cyanobacteriota Melainabacteria group Candidatus Melainabacteria Candidatus Gastranaerophilales Candidatus Gastranaerophilales MCX4274372.1 331 aa 331 aa
BacClassIA Bacilliati Cyanobacteriota Melainabacteria group Candidatus Melainabacteria Candidatus Gastranaerophilales Candidatus Gastranaerophilales HSA07230.1 332 aa 332 aa
BacClassIA Bacilliati Cyanobacteriota Melainabacteria group Cyanobacteriota Cyanophyceae Oscillatoriaceae cyanobacterium HEY9722720.1 328 aa 328 aa
BacClassIA Bacilliati Cyanobacteriota Melainabacteria group Candidatus Sericytochromatia Candidatus Sericytochromatia MFP5503648.1 326 aa 326 aa
BacClassIA Bacilliati Cyanobacteriota Melainabacteria group Cyanobacteriota Cyanophyceae Cyanobacteria bacterium RYN 339 MDB5098562.1 ClassIV 328 aa
BacClassIA Bacilliati Cyanobacteriota Melainabacteria group Candidatus Sericytochromatia Candidatus Sericytochromatia MEB3328935.1 326 aa 326 aa
BacClassIA Bacilliati Cyanobacteriota Melainabacteria group Cyanobacteriota Cyanophyceae Chroococcales cyanobacterium HEY9764837.1 319 aa 319 aa
BacClassIA Bacilliati Cyanobacteriota Melainabacteria group Cyanobacteriota Cyanophyceae Stenomitos sp HEY9857430.1 324 aa 324 aa
BacClassIA Bacilliati Cyanobacteriota Melainabacteria group Cyanobacteriota Cyanophyceae Pantanalinema sp HEY9899584.1 308 aa 308 aa
BacClassIA Bacilliati Cyanobacteriota Melainabacteria group Cyanobacteriota unclassified Cyanobacteriota Cyanobacteria bacterium MBI6545409.1 332 aa 332 aa
BacClassIA Bacilliati Cyanobacteriota Melainabacteria group Candidatus Sericytochromatia Candidatus Tanganyikabacteria MBM3259934.1 333 aa 333 aa
BacClassIA Bacilliati Cyanobacteriota Melainabacteria group Cyanobacteriota unclassified Cyanobacteriota Cyanobacteria bacterium MBU6427673.1 326 aa 326 aa
BacClassIA Bacilliati Cyanobacteriota Melainabacteria group Candidatus Sericytochromatia Candidatus Tanganyikabacteria MBM3268111.1 331 aa 331 aa
BacClassIA Bacilliati Cyanobacteriota Melainabacteria group Candidatus Melainabacteria Vampirovibriophyceae Candidatus Caenarcanales MCH2227153.1 333 aa 333 aa
BacClassIA Bacilliati Cyanobacteriota Melainabacteria group Candidatus Melainabacteria unclassified Candidatus Melainabacteria Candidatus Melainabacteria MBI1858922.1 317 aa 317 aa
BacClassIA Bacilliati Cyanobacteriota Melainabacteria group Candidatus Melainabacteria unclassified Candidatus Melainabacteria Candidatus Melainabacteria OGI06194.1 316 aa 316 aa
BacClassIA Bacilliati Cyanobacteriota Melainabacteria group Candidatus Melainabacteria unclassified Candidatus Melainabacteria Candidatus Melainabacteria MBI3591601.1 317 aa 317 aa
BacClassIA Bacilliati Cyanobacteriota Melainabacteria group Candidatus Melainabacteria Vampirovibriophyceae Candidatus Caenarcanales MDX1917558.1 336 aa 336 aa
BacClassIA Bacilliati Cyanobacteriota Melainabacteria group Candidatus Melainabacteria unclassified Candidatus Melainabacteria Candidatus Melainabacteria MFN5540448.1 336 aa 336 aa
BacClassIA Bacilliati Cyanobacteriota Melainabacteria group Candidatus Melainabacteria Vampirovibriophyceae Vampirovibrionales bacterium HEY9886340.1 335 aa 335 aa
BacClassIA Bacilliati Cyanobacteriota Melainabacteria group Candidatus Melainabacteria Vampirovibriophyceae Candidatus Caenarcanales HEY9886340.1 332 aa 332 aa
BacClassIA Bacilliati Cyanobacteriota Melainabacteria group Candidatus Melainabacteria Candidatus Obscuribacterales bacterium isolate co.spades.CONCOCT.1kb 130 MBY0549573.1 ClassIV 325 aa
BacClassIA Bacilliati Cyanobacteriota Melainabacteria group Candidatus Melainabacteria Candidatus Obscuribacterales Candidatus Obscuribacterales MBY0357820.1 322 aa 322 aa
BacClassIA Bacilliati Cyanobacteriota Melainabacteria group Candidatus Melainabacteria unclassified Candidatus Melainabacteria Candidatus Melainabacteria MBI4534111.1 323 aa 323 aa
BacClassIA Bacilliati Cyanobacteriota Melainabacteria group Candidatus Melainabacteria Candidatus Obscuribacterales Candidatus Obscuribacterales MBX9770666.1 352 aa 352 aa
BacClassIA Bacilliati Cyanobacteriota Melainabacteria group Candidatus Melainabacteria unclassified Candidatus Melainabacteria Candidatus Melainabacteria PZM78114.1 331 aa 331 aa
BacClassIA Bacilliati Cyanobacteriota Melainabacteria group Candidatus Melainabacteria Candidatus Obscuribacterales Candidatus Obscuribacterales MBX9770563.1 318 aa 318 aa
BacClassIA Bacilliati Cyanobacteriota Melainabacteria group Cyanobacteriota Cyanophyceae Planktothrix sp HEY9775124.1 324 aa 324 aa
BacClassIA Bacilliati Cyanobacteriota Melainabacteria group Cyanobacteriota Cyanophyceae Oculatellaceae cyanobacterium HEY9683589.1 328 aa 328 aa
BacClassIA Bacilliati Cyanobacteriota Melainabacteria group Cyanobacteriota Cyanophyceae Chroococcales cyanobacterium HEY9712764.1 323 aa 323 aa
BacClassIA Bacilliati Cyanobacteriota Melainabacteria group Candidatus Melainabacteria unclassified Candidatus Melainabacteria Candidatus Melainabacteria MBN9394907.1 323 aa 323 aa
BacClassIA Bacilliati Cyanobacteriota Melainabacteria group Candidatus Melainabacteria Candidatus Obscuribacterales Candidatus Obscuribacter MBK9769878.1 327 aa 327 aa
BacClassIA Bacilliati Cyanobacteriota Melainabacteria group Cyanobacteriota unclassified Cyanobacteriota Cyanobacteria bacterium MCW5824757.1 338 aa 338 aa
BacClassIA Bacilliati Cyanobacteriota Melainabacteria group Candidatus Melainabacteria Candidatus Obscuribacterales Candidatus Obscuribacterales MBX3076020.1 325 aa 325 aa

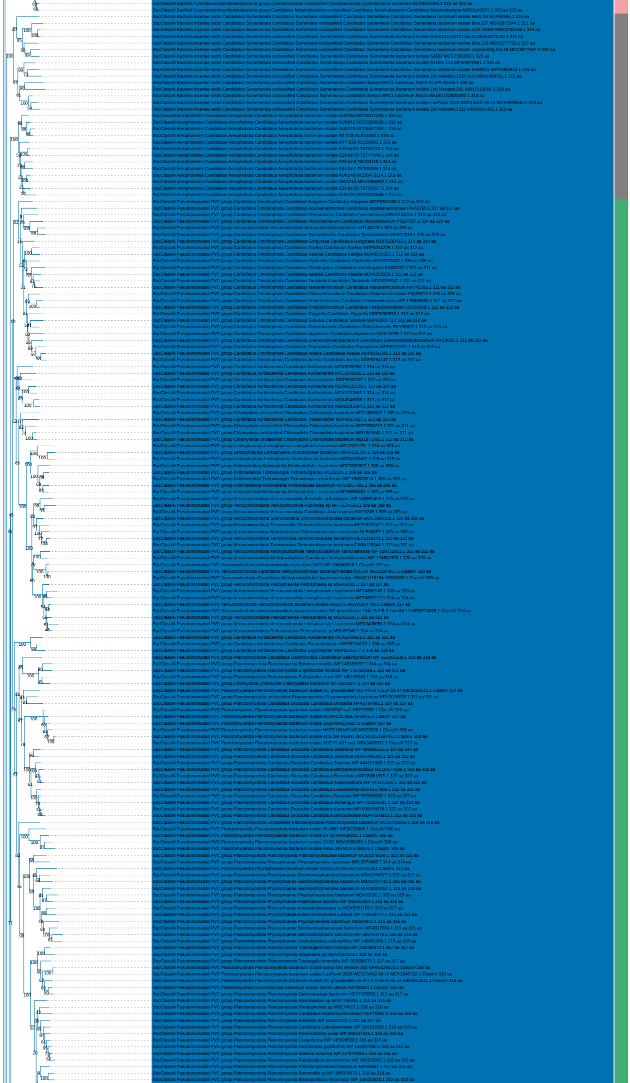

BacClassIA Bacillati Cyanobacteriota Melainabacteria group Cyanobacteriota unclassified Cyanobacteriota Cyanobacteria bacterium MCA9815495.1 323 aa 323 aa
BacClassIA Bacillati Cyanobacteriota Melainabacteria group Candidatus Melainabacteria unclassified Candidatus Melainabacteria Candidatus Melainabacteria MBK9143557.1 323 aa 323 aa
BacClassIA Bacteria incertae sedis Candidatus Sumerlaeota Candidatus Sumerlaeia unclassified Candidatus Sumerlaeia Candidatus Sumerlaeia bacterium isolate MAG 26 NUP89365.1 316 aa
BacClassIA Bacteria incertae sedis Candidatus Sumerlaeota Candidatus Sumerlaeia unclassified Candidatus Sumerlaeia Candidatus Sumerlaeia bacterium isolate bins.237 MDX1972544.1 321 aa
BacClassIA Bacteria incertae sedis Candidatus Sumerlaeota Candidatus Sumerlaeia unclassified Candidatus Sumerlaeia Candidatus Sumerlaeia bacterium isolate ACE SUM1 MBX3730105.1 319 aa
BacClassIA Bacteria incertae sedis Candidatus Sumerlaeota unclassified Candidatus Sumerlaeota Candidatus Sumerlaeota bacterium isolate DJKA124 ANT21 bin.23 MDK2973130.1 319 aa
BacClassIA Bacteria incertae sedis Candidatus Sumerlaeota Candidatus Sumerlaeia unclassified Candidatus Sumerlaeia Candidatus Sumerlaeia bacterium isolate bins.233 MDX2177720.1 337 aa
BacClassIA Bacteria incertae sedis Candidatus Sumerlaeota Candidatus Sumerlaeia unclassified Candidatus Sumerlaeia Candidatus Sumerlaeia bacterium isolate coassembly bin.29 MCC5877467.1 320 aa
BacClassIA Bacteria incertae sedis Candidatus Sumerlaeota unclassified Candidatus Sumerlaeota Candidatus Sumerlaeota bacterium isolate SJ802 MCC7391083.1 320 aa
BacClassIA Bacteria incertae sedis Candidatus Sumerlaeota unclassified Candidatus Sumerlaeota Candidatus Sumerlaeota bacterium isolate D1M01 123 MFW5870561.1 340 aa
BacClassIA Bacteria incertae sedis Candidatus Sumerlaeota Candidatus Sumerlaeia unclassified Candidatus Sumerlaeia Candidatus Sumerlaeia bacterium isolate D1M03 6 MFP4381518.1 315 aa
BacClassIA Bacteria incertae sedis Candidatus Sumerlaeota unclassified Candidatus Sumerlaeota Candidatus Sumerlaeota bacterium isolate Zod Metabat.1318 iso2 MBN1868781.1 315 aa
BacClassIA Bacteria incertae sedis Candidatus Sumerlaeota unclassified Candidatus Sumerlaeota candidate division BRC1 bacterium SM23 51 KPL06106.1 303 aa
BacClassIA Bacteria incertae sedis Candidatus Sumerlaeota unclassified Candidatus Sumerlaeota Candidatus Sumerlaeota bacterium isolate Zod Metabat.292 MBN1515566.1 318 aa
BacClassIA Bacteria incertae sedis Candidatus Sumerlaeota unclassified Candidatus Sumerlaeota candidate division BRC1 bacterium ADurb.Bin183 OQB20252.1 313 aa
BacClassIA Bacteria incertae sedis Candidatus Sumerlaeota unclassified Candidatus Sumerlaeota Candidatus Sumerlaeota bacterium isolate LacPavin 0920 SED5 MAG 50 10 MCX6999040.1 313 aa
BacClassIA Bacteria incertae sedis Candidatus Sumerlaeota unclassified Candidatus Sumerlaeota Candidatus Sumerlaeota bacterium isolate Zod Metabat.1312 MBN1901465.1 313 aa
BacClassIA Aerophobetes Candidatus Aerophobota Candidatus Aerophobota bacterium isolate AUK284 MCD6317493.1 313 aa
BacClassIA Aerophobetes Candidatus Aerophobota Candidatus Aerophobota bacterium isolate AUK042 MCD6256839.1 316 aa
BacClassIA Aerophobetes Candidatus Aerophobota Candidatus Aerophobota bacterium isolate AUK278 MCD6407389.1 316 aa
BacClassIA Aerophobetes Candidatus Aerophobota Candidatus Aerophobota bacterium isolate B3 G15 RLE14883.1 315 aa
BacClassIA Aerophobetes Candidatus Aerophobota Candidatus Aerophobota bacterium isolate B47 G16 RLE09081.1 313 aa
BacClassIA Aerophobetes Candidatus Aerophobota Candidatus Aerophobota bacterium isolate E29 bin52 TET62174.1 314 aa
BacClassIA Aerophobetes Candidatus Aerophobota Candidatus Aerophobota bacterium isolate E29 bin78 TET47946.1 314 aa
BacClassIA Aerophobetes Candidatus Aerophobota Candidatus Aerophobota bacterium isolate E26 bin6 TEU03116.1 314 aa
BacClassIA Aerophobetes Candidatus Aerophobota Candidatus Aerophobota bacterium isolate E44 bin7 TET08336.1 314 aa
BacClassIA Aerophobetes Candidatus Aerophobota Candidatus Aerophobota bacterium isolate AUK148 MCD6473114.1 319 aa
BacClassIA Aerophobetes Candidatus Aerophobota Candidatus Aerophobota bacterium isolate MAG210 MEA1964592.1 314 aa
BacClassIA Aerophobetes Candidatus Aerophobota Candidatus Aerophobota bacterium isolate E29 bin28 TET92807.1 314 aa
BacClassIA Aerophobetes Candidatus Aerophobota Candidatus Aerophobota bacterium isolate AUK101 MCD6231648.1 314 aa
BacClassIA Pseudomonadati PVC group Candidatus Omnitrophota Candidatus Kappaea Candidatus Kappaea MDP8261468.1 312 aa 312 aa
BacClassIA Pseudomonadati PVC group Candidatus Omnitrophota Candidatus Aquitaenariimonas Candidatus Aquitaenariimonas PIU42265.1 317 aa 317 aa
BacClassIA Pseudomonadati PVC group Candidatus Omnitrophota Candidatus Velesiimonas Candidatus Velesiimonas MBA2124132.1 313 aa 313 aa
BacClassIA Pseudomonadati PVC group Candidatus Omnitrophota Candidatus Abzuiibacterium Candidatus Abzuiibacterium PIQ87367.1 324 aa 324 aa
BacClassIA Pseudomonadati PVC group Verrucomicrobiota Verrucomicrobiia Verrucomicrobiia bacterium HTL48176.1 329 aa 329 aa
BacClassIA Pseudomonadati PVC group Candidatus Omnitrophota Candidatus Taenariivivens Candidatus Taenariivivens MSR77815.1 320 aa 320 aa
BacClassIA Pseudomonadati PVC group Candidatus Omnitrophota Candidatus Gorgyraea Candidatus Gorgyraea MDP8230415.1 314 aa 314 aa
BacClassIA Pseudomonadati PVC group Candidatus Omnitrophota Candidatus Saelkia Candidatus Saelkia MDP8234225.1 312 aa 312 aa
BacClassIA Pseudomonadati PVC group Candidatus Omnitrophota Candidatus Kaelpia Candidatus Kaelpia MDP8216352.1 312 aa 312 aa
BacClassIA Pseudomonadati PVC group Candidatus Omnitrophota Candidatus Orphnella Candidatus Orphnella MDP8292403.1 336 aa 336 aa
BacClassIA Pseudomonadati PVC group Candidatus Omnitrophota Candidatus Omnitrophus Candidatus Omnitrophus KJJ83732.1 311 aa 311 aa
BacClassIA Pseudomonadati PVC group Candidatus Omnitrophota Candidatus Aadella Candidatus Aadella MDP8259359.1 311 aa 311 aa
BacClassIA Pseudomonadati PVC group Candidatus Omnitrophota Candidatus Tantalella Candidatus Tantalella MDP8299942.1 311 aa 311 aa
BacClassIA Pseudomonadati PVC group Candidatus Omnitrophota Candidatus Makaraimicrobium Candidatus Makaraimicrobium RKY42360.1 311 aa 311 aa
BacClassIA Pseudomonadati PVC group Candidatus Omnitrophota Candidatus Ghiorseimicrobium Candidatus Ghiorseimicrobium PIQ89812.1 315 aa 315 aa
BacClassIA Pseudomonadati PVC group Candidatus Omnitrophota Candidatus Velamenicoccus Candidatus Velamenicoccus WP 128698980.1 317 aa 317 aa
BacClassIA Pseudomonadati PVC group Candidatus Omnitrophota Candidatus Phelpsiimicrobium Candidatus Phelpsiimicrobium OIO36009.1 312 aa 312 aa
BacClassIA Pseudomonadati PVC group Candidatus Omnitrophota Candidatus Gygaella Candidatus Gygaella MDP8260579.1 312 aa 312 aa
BacClassIA Pseudomonadati PVC group Candidatus Omnitrophota Candidatus Susulua Candidatus Susulua MDP8290171.1 312 aa 312 aa
BacClassIA Pseudomonadati PVC group Candidatus Omnitrophota Candidatus Duberdicusella Candidatus Duberdicusella RKY35609.1 313 aa 313 aa
BacClassIA Pseudomonadati PVC group Candidatus Omnitrophota Candidatus Aquivivens Candidatus Aquivivens OGX13235.1 312 aa 312 aa
BacClassIA Pseudomonadati PVC group Candidatus Omnitrophota Candidatus Sherwoodlollariibacterium Candidatus Sherwoodlollariibacterium PIP19068.1 312 aa 312 aa
BacClassIA Pseudomonadati PVC group Candidatus Omnitrophota Candidatus Zapsychrus Candidatus Zapsychrus MDP8212282.1 313 aa 313 aa
BacClassIA Pseudomonadati PVC group Candidatus Omnitrophota Candidatus Aceula Candidatus Aceula MDP8266330.1 316 aa 316 aa
BacClassIA Pseudomonadati PVC group Candidatus Omnitrophota Candidatus Aceula Candidatus Aceula MDP8264147.1 312 aa 312 aa
BacClassIA Pseudomonadati PVC group Candidatus Auribacterota Candidatus Auribacterota MCK5708350.1 314 aa 314 aa
BacClassIA Pseudomonadati PVC group Candidatus Auribacterota Candidatus Auribacterota MFC2140265.1 310 aa 310 aa
BacClassIA Pseudomonadati PVC group Candidatus Auribacterota Candidatus Ancaeobacter MDP8262107.1 312 aa 312 aa
BacClassIA Pseudomonadati PVC group Candidatus Auribacterota Candidatus Auribacterota MEW6535030.1 314 aa 314 aa
BacClassIA Pseudomonadati PVC group Candidatus Auribacterota Candidatus Auribacterota MDX9703562.1 314 aa 314 aa
BacClassIA Pseudomonadati PVC group Candidatus Auribacterota Candidatus Auribacterota MCK4946836.1 312 aa 312 aa
BacClassIA Pseudomonadati PVC group Candidatus Auribacterota Candidatus Auribacterota MBN2191094.1 312 aa 312 aa
BacClassIA Pseudomonadati PVC group Chlamydiota unclassified Chlamydiota Chlamydiota bacterium MFC1668500.1 289 aa 289 aa
BacClassIA Pseudomonadati PVC group Candidatus Auribacterota Candidatus Theseobacter MDP8217927.1 313 aa 313 aa
BacClassIA Pseudomonadati PVC group Chlamydiota unclassified Chlamydiota Chlamydiota bacterium MDP3980219.1 311 aa 311 aa
BacClassIA Pseudomonadati PVC group Chlamydiota unclassified Chlamydiota Chlamydiota bacterium MBI1882640.1 311 aa 311 aa
BacClassIA Pseudomonadati PVC group Chlamydiota unclassified Chlamydiota Chlamydiota bacterium MBI1871065.1 312 aa 312 aa
BacClassIA Pseudomonadati PVC group Lentisphaerota Lentisphaeria Lentisphaeria bacterium MFO7821251.1 324 aa 324 aa
BacClassIA Pseudomonadati PVC group Lentisphaerota Lentisphaeria Victivallaceae bacterium MFA7231787.1 319 aa 319 aa
BacClassIA Pseudomonadati PVC group Lentisphaerota Lentisphaeria Victivallaceae bacterium MDD4180402.1 315 aa 315 aa
BacClassIA Pseudomonadati PVC group Kiritimatiellota Kiritimatiellia Kiritimatiellales bacterium MCF7863302.1 308 aa 308 aa
BacClassIA Pseudomonadati PVC group Kiritimatiellota Tichowtungiia Tichowtungia sp HKL22605.1 309 aa 309 aa
BacClassIA Pseudomonadati PVC group Kiritimatiellota Tichowtungiia Tichowtungia aerotolerans WP 160626914.1 309 aa 309 aa
BacClassIA Pseudomonadati PVC group Kiritimatiellota Kiritimatiellia Pontiellaceae bacterium MCU0857081.1 309 aa 309 aa
BacClassIA Pseudomonadati PVC group Kiritimatiellota Kiritimatiellia Kiritimatiellales bacterium MFA5688481.1 309 aa 309 aa
BacClassIA Pseudomonadati PVC group Verrucomicrobiota Verrucomicrobiia Brevifollis gellanilyticus WP 146851429.1 310 aa 310 aa
BacClassIA Pseudomonadati PVC group Verrucomicrobiota Verrucomicrobiia Rubritalea sp MFT5634550.1 309 aa 309 aa
BacClassIA Pseudomonadati PVC group Verrucomicrobiota Verrucomicrobiia Candidatus Akkermansia HIX19245.1 309 aa 309 aa
BacClassIA Pseudomonadati PVC group Verrucomicrobiota Spartobacteria Chthoniobacteraceae bacterium MGC3990102.1 320 aa 320 aa
BacClassIA Pseudomonadati PVC group Verrucomicrobiota Terrimicrobiia Terrimicrobiaceae bacterium MFZ9920347.1 312 aa 312 aa
BacClassIA Pseudomonadati PVC group Verrucomicrobiota Spartobacteria Chthoniobacterales bacterium HLB34057.1 309 aa 309 aa
BacClassIA Pseudomonadati PVC group Verrucomicrobiota Terrimicrobiia Terrimicrobiaceae bacterium MEX1119343.1 310 aa 310 aa
BacClassIA Pseudomonadati PVC group Verrucomicrobiota Terrimicrobiia Terrimicrobiaceae bacterium GAB4175044.1 332 aa 332 aa
BacClassIA Pseudomonadati PVC group Verrucomicrobiota Methylacidiphilae Methylacidiphilum kamchatkense WP 039721852.1 322 aa 322 aa
BacClassIA Pseudomonadati PVC group Verrucomicrobiota Methylacidiphilae Candidatus Methylacidithermus WP 174582303.1 320 aa 320 aa
BacClassIA Pseudomonadati PVC Verrucomicrobiota Verrucomicrobia bacterium LW23 WP 108040016.1 ClassIV 329 aa
BacClassIA Pseudomonadati PVC Verrucomicrobiota Candidatus Methylacidiphilales bacterium isolate bin.034 MEZ0294857.1 ClassIV 328 aa
BacClassIA Pseudomonadati PVC Verrucomicrobiota Candidatus Methylacidiphilales bacterium isolate SMAG U16154 HSI83038.1 ClassIV 369 aa
BacClassIA Pseudomonadati PVC group Verrucomicrobiota Pedosphaerae Pedosphaera sp MSR66081.1 314 aa 314 aa
BacClassIA Pseudomonadati PVC group Verrucomicrobiota Verrucomicrobiia Limisphaerales bacterium MFT4688145.1 333 aa 333 aa
BacClassIA Pseudomonadati PVC group Verrucomicrobiota Verrucomicrobiia Limisphaerales bacterium MFT4587117.1 314 aa 314 aa
BacClassIA Pseudomonadati PVC Verrucomicrobiota Verrucomicrobiota bacterium isolate MAG171 MEW6304738.1 ClassIV 314 aa
BacClassIA Pseudomonadati PVC Verrucomicrobiota Verrucomicrobiota bacterium isolate NC groundwater 1841 Pr3 B 0.1um 64 12 MBI5773836.1 ClassIV 314 aa
BacClassIA Pseudomonadati PVC group Verrucomicrobiota Pedosphaerae Pedosphaera sp MSU05106.1 321 aa 321 aa
BacClassIA Pseudomonadati PVC group Verrucomicrobiota Verrucomicrobiia Limisphaerales bacterium MFN3408053.1 314 aa 314 aa
BacClassIA Pseudomonadati PVC group Verrucomicrobiota Pedosphaerae Pedosphaera sp MSU62946.1 314 aa 314 aa
BacClassIA Pseudomonadati PVC group Candidatus Auribacterota Candidatus Auribacterota MCX6349961.1 321 aa 321 aa
BacClassIA Pseudomonadati PVC group Candidatus Auribacterota Candidatus Euphemobacter MDP8214129.1 335 aa 335 aa
BacClassIA Pseudomonadati PVC group Candidatus Auribacterota Candidatus Erginobacter MDP8235677.1 339 aa 339 aa
BacClassIA Pseudomonadati PVC group Planctomycetota Candidatus Uabimicrobiia Candidatus Uabimicrobium WP 151966100.1 318 aa 318 aa
BacClassIA Pseudomonadati PVC group Planctomycetota Planctomycetia Rohdeia mirabilis WP 145189997.1 314 aa 314 aa
BacClassIA Pseudomonadati PVC group Planctomycetota Planctomycetia Engelhardtia mirabilis WP 145068299.1 322 aa 322 aa
BacClassIA Pseudomonadati PVC group Planctomycetota Planctomycetia Saltatorellus ferox WP 145196943.1 316 aa 316 aa
BacClassIA Pseudomonadati PVC group Chlamydiota Chlamydiia Chlamydiales bacterium MFT5050247.1 314 aa 314 aa
BacClassIA Pseudomonadati PVC Planctomycetota Planctomycetota bacterium isolate NC groundwater 805 Pr6 B 0.1um 69 24 MBI3268016.1 ClassIV 314 aa
BacClassIA Pseudomonadati PVC group Planctomycetota unclassified Planctomycetota Planctomycetota bacterium HEV3026528.1 311 aa 311 aa
BacClassIA Pseudomonadati PVC group Planctomycetota Candidatus Brocadiia Candidatus Brocadiia MFA5794992.1 314 aa 314 aa
BacClassIA Pseudomonadati PVC Planctomycetota Planctomycetota bacterium isolate UBA8742 iso1 HBP19558.1 ClassIV 313 aa
BacClassIA Pseudomonadati PVC Planctomycetota Planctomycetota bacterium isolate NORP233 MBL4849970.1 ClassIV 314 aa
BacClassIA Pseudomonadati PVC Planctomycetota Planctomycetota bacterium isolate J058 RMG14924.1 ClassIV 307 aa
BacClassIA Pseudomonadati PVC Planctomycetota Planctomycetota bacterium isolate HKST UBA89 MCA8923678.1 ClassIV 308 aa
BacClassIA Pseudomonadati PVC Planctomycetota Planctomycetota bacterium isolate ACE NR PLAN1 iso1 MCO5169746.1 ClassIV 308 aa
BacClassIA Pseudomonadati PVC Planctomycetota Planctomycetota bacterium isolate ACE PLA31 iso1 MBX3469592.1 ClassIV 317 aa
BacClassIA Pseudomonadati PVC group Planctomycetota Candidatus Brocadiia Candidatus Scalindua WP 096895555.1 324 aa 324 aa
BacClassIA Pseudomonadati PVC group Planctomycetota Candidatus Brocadiia Candidatus Scalindua MDR4496368.1 322 aa 322 aa
BacClassIA Pseudomonadati PVC group Planctomycetota Candidatus Brocadiia Candidatus Tripitaka WP 444201888.1 322 aa 322 aa
BacClassIA Pseudomonadati PVC group Planctomycetota Candidatus Brocadiia Candidatus Bathyanammoxibius MCQ4574680.1 322 aa 322 aa
BacClassIA Pseudomonadati PVC group Planctomycetota Candidatus Brocadiia Candidatus Brocadiales MFQ5861575.1 322 aa 322 aa
BacClassIA Pseudomonadati PVC group Planctomycetota Candidatus Brocadiia Candidatus Avalokitesvara WP 444141100.1 322 aa 322 aa
BacClassIA Pseudomonadati PVC group Planctomycetota Candidatus Brocadiia Candidatus Loosdrechtia MCF6157808.1 322 aa 322 aa
BacClassIA Pseudomonadati PVC group Planctomycetota Candidatus Brocadiia Candidatus Brocadia WP 052563005.1 323 aa 323 aa
BacClassIA Pseudomonadati PVC group Planctomycetota Candidatus Brocadiia Candidatus Wunengus WP 444203781.1 322 aa 322 aa
BacClassIA Pseudomonadati PVC group Planctomycetota Candidatus Brocadiia Candidatus Kuenenia WP 099326076.1 322 aa 322 aa
BacClassIA Pseudomonadati PVC group Planctomycetota Candidatus Brocadiia Candidatus Brocadiaceae MDR4506812.1 322 aa 322 aa
BacClassIA Pseudomonadati PVC group Planctomycetota unclassified Planctomycetota Planctomycetota bacterium MCZ6786860.1 319 aa 319 aa
BacClassIA Pseudomonadati PVC Planctomycetota Planctomycetota bacterium isolate ELA667 MEI6233809.1 ClassIV 580 aa
BacClassIA Pseudomonadati PVC Planctomycetota Planctomycetota bacterium isolate DT 85 NRA38281.1 ClassIV 306 aa
BacClassIA Pseudomonadati PVC Planctomycetota Planctomycetota bacterium isolate GH22 MDA3960989.1 ClassIV 306 aa
BacClassIA Pseudomonadati PVC Planctomycetota Planctomycetota bacterium isolate MAG.349 MCK6488046.1 ClassIV 306 aa
BacClassIA Pseudomonadati PVC group Planctomycetota Planctomycetia Planctomycetaceae bacterium MCE5278569.1 323 aa 323 aa
BacClassIA Pseudomonadati PVC group Planctomycetota Phycisphaerae Phycisphaerales bacterium MBL8879483.1 314 aa 314 aa
BacClassIA Pseudomonadati PVC Planctomycetota Phycisphaerae bacterium isolate SMAG U4706 HEY3244570.1 ClassIV 313 aa
BacClassIA Pseudomonadati PVC group Planctomycetota Phycisphaerae Sedimentisphaerales bacterium MBN1764577.1 317 aa 317 aa
BacClassIA Pseudomonadati PVC group Planctomycetota Phycisphaerae Sedimentisphaerales bacterium MBN1437745.1 326 aa 326 aa
BacClassIA Pseudomonadati PVC group Planctomycetota Phycisphaerae Sedimentisphaerales bacterium MDO8304087.1 318 aa 318 aa
BacClassIA Pseudomonadati PVC group Planctomycetota Phycisphaerae Phycisphaeraceae bacterium NQV33200.1 315 aa 315 aa
BacClassIA Pseudomonadati PVC group Planctomycetota Phycisphaerae Anaerobaca lacustris WP 349245464.1 318 aa 318 aa
BacClassIA Pseudomonadati PVC group Planctomycetota Phycisphaerae Anaerohalosphaera sp MCK5001153.1 317 aa 317 aa
BacClassIA Pseudomonadati PVC group Planctomycetota Phycisphaerae Anaerohalosphaera lusitana WP 146659477.1 315 aa 315 aa
BacClassIA Pseudomonadati PVC group Planctomycetota Phycisphaerae Phycisphaerales bacterium HDS84911.1 316 aa 316 aa
BacClassIA Pseudomonadati PVC group Planctomycetota Phycisphaerae Sedimentisphaeraceae bacterium MFI4912394.1 311 aa 311 aa
BacClassIA Pseudomonadati PVC group Planctomycetota Phycisphaerae Sedimentisphaera salicampi WP 085754476.1 316 aa 316 aa
BacClassIA Pseudomonadati PVC group Planctomycetota Phycisphaerae Limihaloglobus sulfuriphilus WP 146682386.1 315 aa 315 aa
BacClassIA Pseudomonadati PVC group Planctomycetota Planctomycetia Thermogemmata fonticola WP 228499572.1 357 aa 357 aa
BacClassIA Pseudomonadati PVC group Planctomycetota Planctomycetia Isosphaera sp MBA4062154.1 328 aa 328 aa
BacClassIA Pseudomonadati PVC group Planctomycetota Planctomycetia Tuwongella immobilis WP 162656579.1 317 aa 317 aa
BacClassIA Pseudomonadati PVC Planctomycetota Planctomycetota bacterium isolate samp 493 semibin 363 MFN5329423.1 ClassIV 316 aa
BacClassIA Pseudomonadati PVC Planctomycetota Planctomycetota bacterium isolate LacPavin 0920 WC12 MAG 54 10 MCY2947132.1 ClassIV 316 aa
BacClassIA Pseudomonadati PVC Planctomycetota Planctomycetota bacterium isolate NC groundwater 65 Pr7 S 0.65um 66 14 MBI1913511.1 ClassIV 318 aa
BacClassIA Pseudomonadati PVC Planctomycetota Gemmataceae bacterium isolate SMAG U6515 HZY88823.1 ClassIV 316 aa
BacClassIA Pseudomonadati PVC group Planctomycetota Planctomycetia Gemmataceae bacterium HEY7155055.1 317 aa 317 aa
BacClassIA Pseudomonadati PVC group Planctomycetota Planctomycetia Mariniblastus sp MFK7766035.1 315 aa 315 aa
BacClassIA Pseudomonadati PVC group Planctomycetota Planctomycetia Rhodopirellula sp MBE74617.1 316 aa 316 aa
BacClassIA Pseudomonadati PVC group Planctomycetota Planctomycetia Candidatus Anammoximicrobium NLF70262.1 319 aa 319 aa
BacClassIA Pseudomonadati PVC group Planctomycetota Planctomycetia Rosistilla WP 145123344.1 317 aa 317 aa
BacClassIA Pseudomonadati PVC group Planctomycetota Planctomycetia Candidatus Laterigemmans WP 164101656.1 316 aa 316 aa
BacClassIA Pseudomonadati PVC group Planctomycetota Planctomycetia Roseimaritima ulvae WP 068137345.1 316 aa 316 aa
BacClassIA Pseudomonadati PVC group Planctomycetota Planctomycetia Crateriforma WP 145300530.1 316 aa 316 aa
BacClassIA Pseudomonadati PVC group Planctomycetota Planctomycetia Novipirellula galeiformis WP 146597360.1 316 aa 316 aa
BacClassIA Pseudomonadati PVC group Planctomycetota Planctomycetia Stieleria maiorica WP 147870369.1 316 aa 316 aa
BacClassIA Pseudomonadati PVC group Planctomycetota Planctomycetia Rubripirellula lacrimiformis WP 145172666.1 316 aa 316 aa
BacClassIA Pseudomonadati PVC group Planctomycetota Planctomycetia Planctomycetaceae bacterium MBI83867.1 314 aa 314 aa
BacClassIA Pseudomonadati PVC group Planctomycetota Planctomycetia Bremerella sp WP 366974073.1 318 aa 318 aa
BacClassIA Pseudomonadati PVC group Planctomycetota Planctomycetia Blastopirellula retiformator WP 146432628.1 322 aa 322 aa

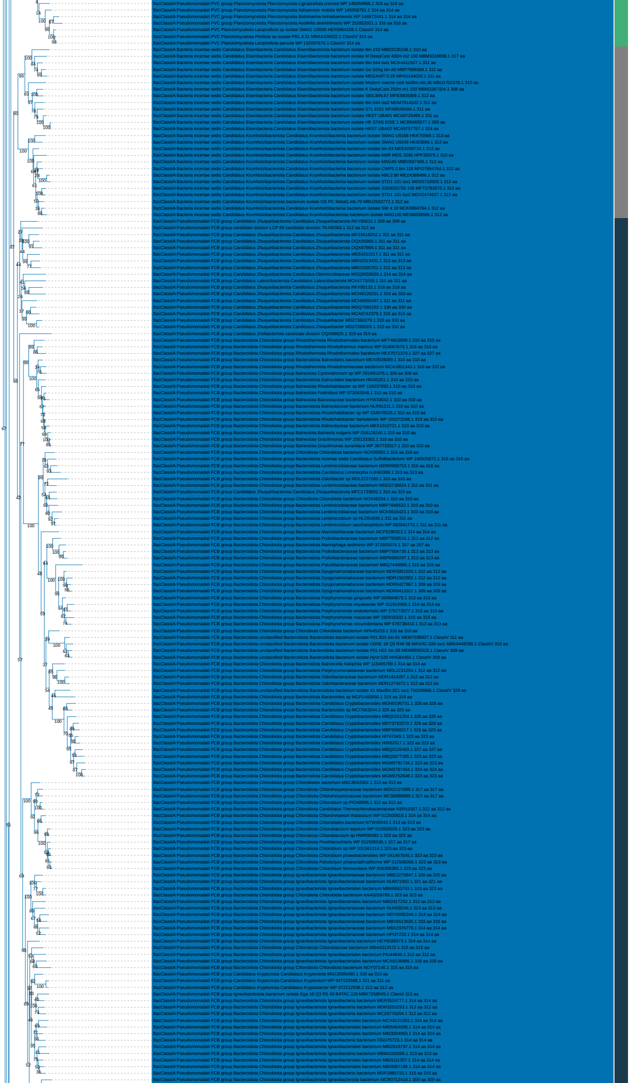

BacClassIA Pseudomonadati PVC group Planctomycetota Planctomycetia Lignipirellula cremea WP 145054966.1 318 aa 318 aa
BacClassIA Pseudomonadati PVC group Planctomycetota Planctomycetia Adhaeretor mobilis WP 145058791.1 314 aa 314 aa
BacClassIA Pseudomonadati PVC group Planctomycetota Planctomycetia Botrimarina hoheduenensis WP 146572441.1 314 aa 314 aa
BacClassIA Pseudomonadati PVC group Planctomycetota Planctomycetia Aeoliella straminimaris WP 252852021.1 316 aa 316 aa
BacClassIA Pseudomonadati PVC Planctomycetota Lacipirellula sp isolate SMAG U2006 HEX6964108.1 ClassIV 314 aa
BacClassIA Pseudomonadati PVC Planctomycetota Pirellula sp isolate PB1.3.31 MBA4106922.1 ClassIV 314 aa
BacClassIA Pseudomonadati PVC Planctomycetota Lacipirellula parvula WP 152097870.1 ClassIV 314 aa
BacClassIA Bacteria incertae sedis Candidatus Eisenbacteria Candidatus Eiseniibacteriota bacterium isolate Bin 233 MBD3235106.1 310 aa
BacClassIA Bacteria incertae sedis Candidatus Eisenbacteria Candidatus Eiseniibacteriota bacterium isolate M DeepCast 400m m2 100 MBM3318658.1 317 aa
BacClassIA Bacteria incertae sedis Candidatus Eisenbacteria Candidatus Eiseniibacteriota bacterium isolate Bin 044 iso1 MCK4412927.1 311 aa
BacClassIA Bacteria incertae sedis Candidatus Eisenbacteria Candidatus Eiseniibacteriota bacterium isolate Go SlDig bin 88 MBP7669369.1 311 aa
BacClassIA Bacteria incertae sedis Candidatus Eisenbacteria Candidatus Eiseniibacteriota bacterium isolate MEGAHIT 0.29 MFH1144039.1 311 aa
BacClassIA Bacteria incertae sedis Candidatus Eisenbacteria Candidatus Eiseniibacteriota bacterium isolate Modern marine rock biofilm.mb.36 MBU1702378.1 310 aa
BacClassIA Bacteria incertae sedis Candidatus Eisenbacteria Candidatus Eiseniibacteriota bacterium isolate K DeepCast 250m m1 333 MBM3287324.1 308 aa
BacClassIA Bacteria incertae sedis Candidatus Eisenbacteria Candidatus Eiseniibacteriota bacterium isolate SBS.BIN.67 MFB3909369.1 312 aa
BacClassIA Bacteria incertae sedis Candidatus Eisenbacteria Candidatus Eiseniibacteriota bacterium isolate Bin 044 iso2 MDM7914102.1 311 aa
BacClassIA Bacteria incertae sedis Candidatus Eisenbacteria Candidatus Eiseniibacteriota bacterium isolate STL EIS1 MFN8549584.1 311 aa
BacClassIA Bacteria incertae sedis Candidatus Eisenbacteria Candidatus Eiseniibacteriota bacterium isolate HKST UBA01 MCA9729495.1 311 aa
BacClassIA Bacteria incertae sedis Candidatus Eisenbacteria Candidatus Eiseniibacteriota bacterium isolate HK STAS EISE 1 MCB9463077.1 389 aa
BacClassIA Bacteria incertae sedis Candidatus Eisenbacteria Candidatus Eiseniibacteriota bacterium isolate HKST UBA02 MCA9757767.1 324 aa
BacClassIA Bacteria incertae sedis Candidatus Krumholzibacteriota Candidatus Krumholzibacteriia bacterium isolate SMAG U9168 HKK70569.1 313 aa
BacClassIA Bacteria incertae sedis Candidatus Krumholzibacteriota Candidatus Krumholzibacteriia bacterium isolate SMAG U9246 HKI83996.1 312 aa
BacClassIA Bacteria incertae sedis Candidatus Krumholzibacteriota Candidatus Krumholzibacteriia bacterium isolate bin.63 MEE4269724.1 312 aa
BacClassIA Bacteria incertae sedis Candidatus Krumholzibacteriota Candidatus Krumholzibacteriia bacterium isolate AMR MDS 3285 HPF35079.1 310 aa
BacClassIA Bacteria incertae sedis Candidatus Krumholzibacteriota Candidatus Krumholzibacteriia bacterium isolate MAG46 MBE0567406.1 312 aa
BacClassIA Bacteria incertae sedis Candidatus Krumholzibacteriota Candidatus Krumholzibacteriia bacterium isolate CWP5 2.bin.118 MFO7654764.1 312 aa
BacClassIA Bacteria incertae sedis Candidatus Krumholzibacteriota Candidatus Krumholzibacteriia bacterium isolate ABC2.80 MEZ4388496.1 312 aa
BacClassIA Bacteria incertae sedis Candidatus Krumholzibacteriota Candidatus Krumholzibacteriia bacterium isolate STD1 131 iso1 MDD5718505.1 312 aa
BacClassIA Bacteria incertae sedis Candidatus Krumholzibacteriota Candidatus Krumholzibacteriia bacterium isolate 3300055726 156 MFT5782875.1 312 aa
BacClassIA Bacteria incertae sedis Candidatus Krumholzibacteriota Candidatus Krumholzibacteriia bacterium isolate STD1 131 iso2 MDX2474627.1 312 aa
BacClassIA Bacteria incertae sedis Candidatus Krumholzibacteriota bacterium isolate OS PC MetaG.mb.70 MBU2502772.1 312 aa
BacClassIA Bacteria incertae sedis Candidatus Krumholzibacteriota Candidatus Krumholzibacteriia bacterium isolate SW 4 19 MCK9994784.1 312 aa
BacClassIA Bacteria incertae sedis Candidatus Krumholzibacteriota Candidatus Krumholzibacteriota bacterium isolate MAG120 MEN8008565.1 312 aa
BacClassIA Pseudomonadati FCB group Candidatus Zhuqueibacterota Candidatus Zhuqueibacterota RKY85612.1 309 aa 309 aa
BacClassIA Pseudomonadati FCB group candidate division LCP 89 candidate division TKJ40383.1 312 aa 312 aa
BacClassIA Pseudomonadati FCB group Candidatus Zhuqueibacterota Candidatus Zhuqueibacterota MFZ5518252.1 311 aa 311 aa
BacClassIA Pseudomonadati FCB group Candidatus Zhuqueibacterota Candidatus Zhuqueibacterota OQX95966.1 311 aa 311 aa
BacClassIA Pseudomonadati FCB group Candidatus Zhuqueibacterota Candidatus Zhuqueibacterota OQX87869.1 311 aa 311 aa
BacClassIA Pseudomonadati FCB group Candidatus Zhuqueibacterota Candidatus Zhuqueibacterota MEE4311517.1 311 aa 311 aa
BacClassIA Pseudomonadati FCB group Candidatus Zhuqueibacterota Candidatus Zhuqueibacterota MBN2013431.1 313 aa 313 aa
BacClassIA Pseudomonadati FCB group Candidatus Zhuqueibacterota Candidatus Zhuqueibacterota MBD3290701.1 312 aa 312 aa
BacClassIA Pseudomonadati FCB group Candidatus Zhuqueibacterota Candidatus Oleimicrobiaceae MGQ9559039.1 314 aa 314 aa
BacClassIA Pseudomonadati FCB group Candidatus Latescibacterota Candidatus Latescibacterota MCK4773059.1 311 aa 311 aa
BacClassIA Pseudomonadati FCB group Candidatus Zhuqueibacterota Candidatus Zhuqueibacterota RKY80133.1 318 aa 318 aa
BacClassIA Pseudomonadati FCB group Candidatus Zhuqueibacterota Candidatus Zhuqueibacterota MCH8126251.1 316 aa 316 aa
BacClassIA Pseudomonadati FCB group Candidatus Zhuqueibacterota Candidatus Zhuqueibacterota MCH8955497.1 311 aa 311 aa
BacClassIA Pseudomonadati FCB group Candidatus Zhuqueibacterota Candidatus Zhuqueibacterota MDQ7066152.1 330 aa 330 aa
BacClassIA Pseudomonadati FCB group Candidatus Zhuqueibacterota Candidatus Zhuqueibacterota MCA9742379.1 315 aa 315 aa
BacClassIA Pseudomonadati FCB group Candidatus Zhuqueibacterota Candidatus Zhuqueibacter MDZ7360279.1 310 aa 310 aa
BacClassIA Pseudomonadati FCB group Candidatus Zhuqueibacterota Candidatus Zhuqueibacter MDZ7289209.1 310 aa 310 aa
BacClassIA Pseudomonadati FCB group Candidatus Zixiibacteriota candidate division OQX89825.1 319 aa 319 aa
BacClassIA Pseudomonadati FCB group Bacteroidota Chlorobiota group Rhodothermota Rhodothermales bacterium MFT4603998.1 310 aa 310 aa
BacClassIA Pseudomonadati FCB group Bacteroidota Chlorobiota group Rhodothermota Rhodothermus marinus WP 014067879.1 316 aa 316 aa
BacClassIA Pseudomonadati FCB group Bacteroidota Chlorobiota group Rhodothermota Rhodothermales bacterium HEX7071370.1 327 aa 327 aa
BacClassIA Pseudomonadati FCB group Bacteroidota Chlorobiota group Bacteroidota Balneolales bacterium MEX0928089.1 310 aa 310 aa
BacClassIA Pseudomonadati FCB group Bacteroidota Chlorobiota group Rhodothermota Rhodothermaceae bacterium MCA1801343.1 310 aa 310 aa
BacClassIA Pseudomonadati FCB group Bacteroidota Chlorobiota group Balneolota Cyclonatronum sp WP 291481376.1 309 aa 309 aa
BacClassIA Pseudomonadati FCB group Bacteroidota Chlorobiota group Bacteroidota Balneolales bacterium HKI45251.1 310 aa 310 aa
BacClassIA Pseudomonadati FCB group Bacteroidota Chlorobiota group Balneolota Rhodohalobacter sp WP 116037893.1 310 aa 310 aa
BacClassIA Pseudomonadati FCB group Bacteroidota Chlorobiota group Balneolota Fodinibius WP 073063848.1 310 aa 310 aa
BacClassIA Pseudomonadati FCB group Bacteroidota Chlorobiota group Balneolota Balneolaceae bacterium HYW34042.1 310 aa 310 aa
BacClassIA Pseudomonadati FCB group Bacteroidota Chlorobiota group Bacteroidota Balneolaceae bacterium HLR91211.1 310 aa 310 aa
BacClassIA Pseudomonadati FCB group Bacteroidota Chlorobiota group Bacteroidota Rhodohalobacter sp WP 234570525.1 310 aa 310 aa
BacClassIA Pseudomonadati FCB group Bacteroidota Chlorobiota group Bacteroidota Rhodohalobacter barkolensis WP 101073166.1 310 aa 310 aa
BacClassIA Pseudomonadati FCB group Bacteroidota Chlorobiota group Bacteroidota Balneolaceae bacterium MEX1010721.1 310 aa 310 aa
BacClassIA Pseudomonadati FCB group Bacteroidota Chlorobiota group Balneolota Balneola vulgaris WP 018128240.1 310 aa 310 aa
BacClassIA Pseudomonadati FCB group Bacteroidota Chlorobiota group Balneolota Gracilimonas WP 255133262.1 310 aa 310 aa
BacClassIA Pseudomonadati FCB group Bacteroidota Chlorobiota group Balneolota Gracilimonas aurantiaca WP 367733317.1 310 aa 310 aa
BacClassIA Pseudomonadati FCB group Bacteroidota Chlorobiota group Chlorobiota Chlorobiota bacterium NOX86992.1 316 aa 316 aa
BacClassIA Pseudomonadati FCB group Bacteroidota Chlorobiota group Bacteroidota incertae sedis Candidatus Sulfidibacterium WP 240525872.1 316 aa 316 aa
BacClassIA Pseudomonadati FCB group Bacteroidota Chlorobiota group Bacteroidota Lentimicrobiaceae bacterium MDR0969753.1 316 aa 316 aa
BacClassIA Pseudomonadati FCB group Bacteroidota Chlorobiota group Bacteroidota Candidatus Limimorpha HJH60399.1 313 aa 313 aa
BacClassIA Pseudomonadati FCB group Bacteroidota Chlorobiota group Bacteroidota Odoribacter sp MDL2227280.1 310 aa 310 aa
BacClassIA Pseudomonadati FCB group Bacteroidota Chlorobiota group Bacteroidota Lentimicrobiaceae bacterium MDD3738824.1 311 aa 311 aa
BacClassIA Pseudomonadati FCB group Candidatus Zhuqueibacterota Candidatus Zhuqueibacterota MFC1733692.1 310 aa 310 aa
BacClassIA Pseudomonadati FCB group Bacteroidota Chlorobiota group Chlorobiota Chlorobiota bacterium NOX46294.1 310 aa 310 aa
BacClassIA Pseudomonadati FCB group Bacteroidota Chlorobiota group Bacteroidota Lentimicrobiaceae bacterium MBP7849632.1 310 aa 310 aa
BacClassIA Pseudomonadati FCB group Bacteroidota Chlorobiota group Bacteroidota Lentimicrobiaceae bacterium MCK9616423.1 310 aa 310 aa
BacClassIA Pseudomonadati FCB group Bacteroidota Chlorobiota group Bacteroidota Lentimicrobium sp HLO91846.1 311 aa 311 aa
BacClassIA Pseudomonadati FCB group Bacteroidota Chlorobiota group Bacteroidota Lentimicrobium saccharophilum WP 062041772.1 311 aa 311 aa
BacClassIA Pseudomonadati FCB group Bacteroidota Chlorobiota group Bacteroidota Marinilabiliaceae bacterium MCF0190312.1 314 aa 314 aa
BacClassIA Pseudomonadati FCB group Bacteroidota Chlorobiota group Bacteroidota Prolixibacteraceae bacterium MBP7506516.1 312 aa 312 aa
BacClassIA Pseudomonadati FCB group Bacteroidota Chlorobiota group Bacteroidota Mariniphaga sediminis WP 372935074.1 357 aa 357 aa
BacClassIA Pseudomonadati FCB group Bacteroidota Chlorobiota group Bacteroidota Prolixibacteraceae bacterium MBP7504730.1 313 aa 313 aa
BacClassIA Pseudomonadati FCB group Bacteroidota Chlorobiota group Bacteroidota Prolixibacteraceae bacterium MBP6969297.1 313 aa 313 aa
BacClassIA Pseudomonadati FCB group Bacteroidota Chlorobiota group Bacteroidota Paludibacteraceae bacterium MBQ7448886.1 315 aa 315 aa
BacClassIA Pseudomonadati FCB group Bacteroidota Chlorobiota group Bacteroidota Dysgonamonadaceae bacterium MDR3061020.1 312 aa 312 aa
BacClassIA Pseudomonadati FCB group Bacteroidota Chlorobiota group Bacteroidota Dysgonamonadaceae bacterium MDR1562902.1 312 aa 312 aa
BacClassIA Pseudomonadati FCB group Bacteroidota Chlorobiota group Bacteroidota Dysgonamonadaceae bacterium MDR0427867.1 309 aa 309 aa
BacClassIA Pseudomonadati FCB group Bacteroidota Chlorobiota group Bacteroidota Dysgonamonadaceae bacterium MDR0413327.1 309 aa 309 aa
BacClassIA Pseudomonadati FCB group Bacteroidota Chlorobiota group Bacteroidota Porphyromonas gingivalis WP 004584679.1 313 aa 313 aa
BacClassIA Pseudomonadati FCB group Bacteroidota Chlorobiota group Bacteroidota Porphyromonas miyakawae WP 411914969.1 314 aa 314 aa
BacClassIA Pseudomonadati FCB group Bacteroidota Chlorobiota group Bacteroidota Porphyromonas endodontalis WP 276773577.1 313 aa 313 aa
BacClassIA Pseudomonadati FCB group Bacteroidota Chlorobiota group Bacteroidota Porphyromonas macacae WP 282918332.1 315 aa 315 aa
BacClassIA Pseudomonadati FCB group Bacteroidota Chlorobiota group Bacteroidota Porphyromonas circumdentaria WP 078736414.1 313 aa 313 aa
BacClassIA Pseudomonadati FCB group Bacteroidota Chlorobiota group Chlorobiota Chlorobiota bacterium NPA45153.1 310 aa 310 aa
BacClassIA Pseudomonadati FCB group Bacteroidota unclassified Bacteroidota Bacteroidota bacterium isolate P01 B01 bin.61 MEM7036927.1 ClassIV 311 aa
BacClassIA Pseudomonadati FCB group Bacteroidota unclassified Bacteroidota Bacteroidota bacterium isolate OdNE 18 Q3 R46 58 MAXAC.036 iso1 MBK9449290.1 ClassIV 310 aa
BacClassIA Pseudomonadati FCB group Bacteroidota unclassified Bacteroidota Bacteroidota bacterium isolate P01 H01 bin.88 MEM0996328.1 ClassIV 309 aa
BacClassIA Pseudomonadati FCB group Bacteroidota unclassified Bacteroidota Bacteroidota bacterium isolate HyVt 520 HHG84484.1 ClassIV 309 aa
BacClassIA Pseudomonadati FCB group Bacteroidota Chlorobiota group Bacteroidota Balneicella halophila WP 116495789.1 314 aa 314 aa
BacClassIA Pseudomonadati FCB group Bacteroidota Chlorobiota group Bacteroidota Porphyromonadaceae bacterium MDL2231254.1 312 aa 312 aa
BacClassIA Pseudomonadati FCB group Bacteroidota Chlorobiota group Bacteroidota Odoribacteraceae bacterium MDR1414287.1 312 aa 312 aa
BacClassIA Pseudomonadati FCB group Bacteroidota Chlorobiota group Bacteroidota Odoribacteraceae bacterium MDR1274672.1 312 aa 312 aa
BacClassIA Pseudomonadati FCB group Bacteroidota unclassified Bacteroidota Bacteroidota bacterium isolate X1 MaxBin.021 iso1 TND09866.1 ClassIV 328 aa
BacClassIA Pseudomonadati FCB group Bacteroidota Chlorobiota group Bacteroidota Bacteroides sp MGP1460650.1 319 aa 319 aa
BacClassIA Pseudomonadati FCB group Bacteroidota Chlorobiota group Bacteroidota Candidatus Cryptobacteroides MGN0195731.1 328 aa 328 aa
BacClassIA Pseudomonadati FCB group Bacteroidota Chlorobiota group Bacteroidota Bacteroides sp MCI7663244.1 325 aa 325 aa
BacClassIA Pseudomonadati FCB group Bacteroidota Chlorobiota group Bacteroidota Candidatus Cryptobacteroides MBQ0151254.1 326 aa 326 aa
BacClassIA Pseudomonadati FCB group Bacteroidota Chlorobiota group Bacteroidota Candidatus Cryptobacteroides MDY3783370.1 328 aa 328 aa
BacClassIA Pseudomonadati FCB group Bacteroidota Chlorobiota group Bacteroidota Candidatus Cryptobacteroides MBP9998217.1 329 aa 329 aa
BacClassIA Pseudomonadati FCB group Bacteroidota Chlorobiota group Bacteroidota Candidatus Cryptobacteroides HIT47045.1 323 aa 323 aa
BacClassIA Pseudomonadati FCB group Bacteroidota Chlorobiota group Bacteroidota Candidatus Cryptobacteroides HIR82517.1 323 aa 323 aa
BacClassIA Pseudomonadati FCB group Bacteroidota Chlorobiota group Bacteroidota Candidatus Cryptobacteroides MBQ0126469.1 327 aa 327 aa
BacClassIA Pseudomonadati FCB group Bacteroidota Chlorobiota group Bacteroidota Candidatus Cryptobacteroides MBQ0077185.1 323 aa 323 aa
BacClassIA Pseudomonadati FCB group Bacteroidota Chlorobiota group Bacteroidota Candidatus Cryptobacteroides MGM9791734.1 323 aa 323 aa
BacClassIA Pseudomonadati FCB group Bacteroidota Chlorobiota group Bacteroidota Candidatus Cryptobacteroides MGM9787454.1 324 aa 324 aa
BacClassIA Pseudomonadati FCB group Bacteroidota Chlorobiota group Bacteroidota Candidatus Cryptobacteroides MGM9752648.1 323 aa 323 aa
BacClassIA Pseudomonadati FCB group Bacteroidota Chlorobiota group Chlorobiales bacterium MBC8043302.1 313 aa 313 aa
BacClassIA Pseudomonadati FCB group Bacteroidota Chlorobiota group Chlorobiota Chloroherpetonaceae bacterium MDX2127889.1 317 aa 317 aa
BacClassIA Pseudomonadati FCB group Bacteroidota Chlorobiota group Chlorobiota Chloroherpetonaceae bacterium MCS6989989.1 317 aa 317 aa
BacClassIA Pseudomonadati FCB group Bacteroidota Chlorobiota group Chlorobiota Chlorobium sp PIO48996.1 312 aa 312 aa
BacClassIA Pseudomonadati FCB group Bacteroidota Chlorobiota group Chlorobiota Candidatus Thermochlorobacteriaceae KER10327.1 312 aa 312 aa
BacClassIA Pseudomonadati FCB group Bacteroidota Chlorobiota group Chlorobiota Chloroherpeton thalassium WP 012500818.1 314 aa 314 aa
BacClassIA Pseudomonadati FCB group Bacteroidota Chlorobiota group Chlorobiota Chlorobiales bacterium NTW49043.1 313 aa 313 aa
BacClassIA Pseudomonadati FCB group Bacteroidota Chlorobiota group Chlorobiota Chlorobaculum tepidum WP 010933029.1 323 aa 323 aa
BacClassIA Pseudomonadati FCB group Bacteroidota Chlorobiota group Chlorobiota Chlorobaculum sp HWR00492.1 323 aa 323 aa
BacClassIA Pseudomonadati FCB group Bacteroidota Chlorobiota group Chlorobiota Prosthecochloris WP 012506045.1 317 aa 317 aa
BacClassIA Pseudomonadati FCB group Bacteroidota Chlorobiota group Chlorobiota Chlorobium sp WP 331561214.1 323 aa 323 aa
BacClassIA Pseudomonadati FCB group Bacteroidota Chlorobiota group Chlorobiota Chlorobium phaeobacteroides WP 041467645.1 323 aa 323 aa
BacClassIA Pseudomonadati FCB group Bacteroidota Chlorobiota group Chlorobiota Pelodictyon phaeoclathratiforme WP 012508366.1 323 aa 323 aa
BacClassIA Pseudomonadati FCB group Bacteroidota Chlorobiota group Chlorobiota Chlorobium ferrooxidans WP 006366385.1 323 aa 323 aa
BacClassIA Pseudomonadati FCB group Bacteroidota Chlorobiota group Ignavibacteriota Ignavibacteriaceae bacterium MBE2279847.1 320 aa 320 aa
BacClassIA Pseudomonadati FCB group Bacteroidota Chlorobiota group Ignavibacteriota Ignavibacteriaceae bacterium NUM71950.1 321 aa 321 aa
BacClassIA Pseudomonadati FCB group Bacteroidota Chlorobiota group Ignavibacteriota Ignavibacteriales bacterium MBK8663793.1 323 aa 323 aa
BacClassIA Pseudomonadati FCB group Bacteroidota Chlorobiota group Chlorobiota Chlorobiota bacterium KAA0259789.1 323 aa 323 aa
BacClassIA Pseudomonadati FCB group Bacteroidota Chlorobiota group Ignavibacteriota Ignavibacteriales bacterium MBI2417252.1 312 aa 312 aa
BacClassIA Pseudomonadati FCB group Bacteroidota Chlorobiota group Ignavibacteriota Ignavibacteriaceae bacterium NUN08246.1 313 aa 313 aa
BacClassIA Pseudomonadati FCB group Bacteroidota Chlorobiota group Ignavibacteriota Ignavibacteriaceae bacterium MDY0080344.1 314 aa 314 aa
BacClassIA Pseudomonadati FCB group Bacteroidota Chlorobiota group Ignavibacteriota Ignavibacteriaceae bacterium MBV6513635.1 333 aa 333 aa
BacClassIA Pseudomonadati FCB group Bacteroidota Chlorobiota group Ignavibacteriota Ignavibacteriaceae bacterium MBX2976779.1 314 aa 314 aa
BacClassIA Pseudomonadati FCB group Bacteroidota Chlorobiota group Ignavibacteriota Ignavibacteriaceae bacterium HPI37233.1 314 aa 314 aa
BacClassIA Pseudomonadati FCB group Bacteroidota Chlorobiota group Ignavibacteriota Ignavibacteria bacterium HEY9188373.1 314 aa 314 aa
BacClassIA Pseudomonadati FCB group Bacteroidota Chlorobiota group Chlorobiota Chlorobiaceae bacterium MBA4313572.1 315 aa 315 aa
BacClassIA Pseudomonadati FCB group Bacteroidota Chlorobiota group Ignavibacteriota Ignavibacteriales bacterium PIU44646.1 312 aa 312 aa
BacClassIA Pseudomonadati FCB group Bacteroidota Chlorobiota group Ignavibacteriota Ignavibacteriales bacterium MCX6136896.1 318 aa 318 aa
BacClassIA Pseudomonadati FCB group Bacteroidota Chlorobiota group Chlorobiota Chlorobiota bacterium NOY07146.1 315 aa 315 aa
BacClassIA Pseudomonadati FCB group Candidatus Kryptoniota Candidatus Kryptoniota MGC8595490.1 310 aa 310 aa
BacClassIA Pseudomonadati FCB group Candidatus Kryptoniota Candidatus Kryptonium WP 047133568.1 311 aa 311 aa
BacClassIA Pseudomonadati FCB group Candidatus Kryptoniota Candidatus Kryptobacter WP 072212938.1 312 aa 312 aa
BacClassIA Pseudomonadati FCB group Ignavibacteriota bacterium isolate Ega 18 Q3 R5 49 BATAC.118 MBK7258049.1 ClassII 312 aa
BacClassIA Pseudomonadati FCB group Bacteroidota Chlorobiota group Ignavibacteriota Ignavibacteria bacterium MER3524777.1 314 aa 314 aa
BacClassIA Pseudomonadati FCB group Bacteroidota Chlorobiota group Ignavibacteriota Ignavibacteria bacterium MDH3252293.1 312 aa 312 aa
BacClassIA Pseudomonadati FCB group Bacteroidota Chlorobiota group Ignavibacteriota Ignavibacteria bacterium MCZ6776204.1 312 aa 312 aa
BacClassIA Pseudomonadati FCB group Bacteroidota Chlorobiota group Ignavibacteriota Ignavibacteriales bacterium MCX6121593.1 314 aa 314 aa
BacClassIA Pseudomonadati FCB group Bacteroidota Chlorobiota group Ignavibacteriota Ignavibacteriales bacterium MBI5464098.1 314 aa 314 aa
BacClassIA Pseudomonadati FCB group Bacteroidota Chlorobiota group Ignavibacteriota Ignavibacteriales bacterium MBI3004963.1 314 aa 314 aa
BacClassIA Pseudomonadati FCB group Bacteroidota Chlorobiota group Ignavibacteriota Ignavibacteria bacterium OGU75723.1 314 aa 314 aa
BacClassIA Pseudomonadati FCB group Bacteroidota Chlorobiota group Ignavibacteriota Ignavibacteriales bacterium MBI2619797.1 314 aa 314 aa
BacClassIA Pseudomonadati FCB group Bacteroidota Chlorobiota group Ignavibacteriota Ignavibacteria bacterium MBM4169269.1 313 aa 313 aa
BacClassIA Pseudomonadati FCB group Bacteroidota Chlorobiota group Ignavibacteriota Ignavibacteriales bacterium MBI3111357.1 314 aa 314 aa
BacClassIA Pseudomonadati FCB group Bacteroidota Chlorobiota group Ignavibacteriota Ignavibacteriales bacterium MBI3587188.1 314 aa 314 aa
BacClassIA Pseudomonadati FCB group Bacteroidota Chlorobiota group Ignavibacteriota Ignavibacteria bacterium MDP2885723.1 315 aa 315 aa
BacClassIA Pseudomonadati FCB group Bacteroidota Chlorobiota group Ignavibacteriota Ignavibacteriota bacterium MCB0712418.1 309 aa 309 aa

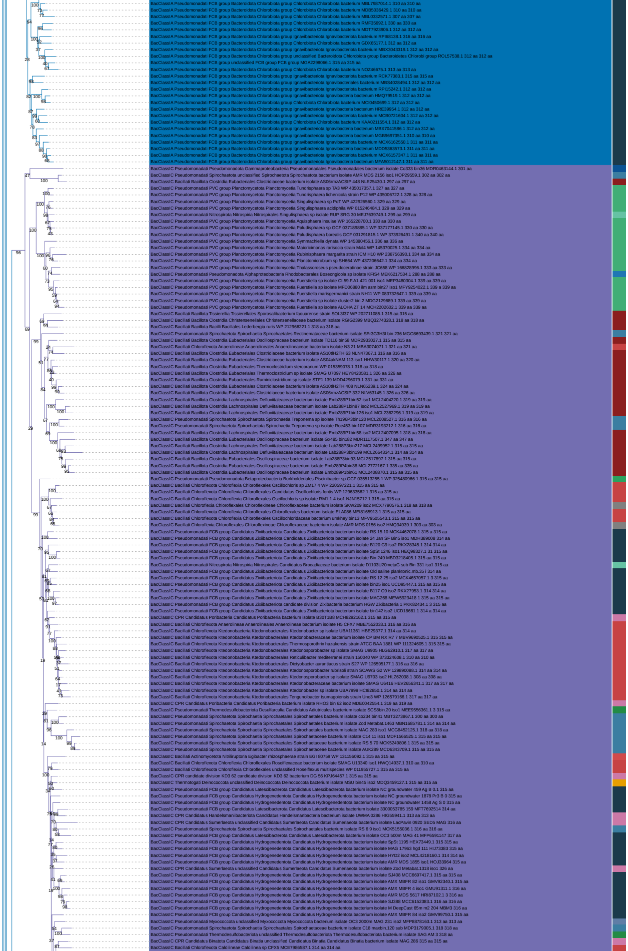

BacClassIA Pseudomonadati FCB group Bacteroidota Chlorobiota group Chlorobiota Chlorobiota bacterium MBL7987014.1 310 aa 310 aa
BacClassIA Pseudomonadati FCB group Bacteroidota Chlorobiota group Chlorobiota Chlorobiota bacterium MDB5036429.1 310 aa 310 aa
BacClassIA Pseudomonadati FCB group Bacteroidota Chlorobiota group Chlorobiota Chlorobiota bacterium MBL0332571.1 307 aa 307 aa
BacClassIA Pseudomonadati FCB group Bacteroidota Chlorobiota group Chlorobiota Chlorobiota bacterium RMF35692.1 330 aa 330 aa
BacClassIA Pseudomonadati FCB group Bacteroidota Chlorobiota group Chlorobiota Chlorobiota bacterium MDT7923906.1 312 aa 312 aa
BacClassIA Pseudomonadati FCB group Bacteroidota Chlorobiota group Ignavibacteriota Ignavibacteriota bacterium RPI68138.1 316 aa 316 aa
BacClassIA Pseudomonadati FCB group Bacteroidota Chlorobiota group Chlorobiota Chlorobiota bacterium GDX65177.1 312 aa 312 aa
BacClassIA Pseudomonadati FCB group Bacteroidota Chlorobiota group Ignavibacteriota Ignavibacteriota bacterium MBX3043319.1 312 aa 312 aa
BacClassIA Pseudomonadati FCB group Bacteroidota Chlorobiota group unclassified Bacteroidota Chlorobiota group Bacteroidetes Chlorobi group ROL57538.1 312 aa 312 aa
BacClassIA Pseudomonadati FCB group unclassified FCB group FCB group MGA2298066.1 315 aa 315 aa
BacClassIA Pseudomonadati FCB group Bacteroidota Chlorobiota group Chlorobiota Chlorobiota bacterium NOZ46675.1 313 aa 313 aa
BacClassIA Pseudomonadati FCB group Bacteroidota Chlorobiota group Ignavibacteriota Ignavibacteriota bacterium RCK77383.1 315 aa 315 aa
BacClassIA Pseudomonadati FCB group Bacteroidota Chlorobiota group Ignavibacteriota Ignavibacteriales bacterium MBS4028494.1 312 aa 312 aa
BacClassIA Pseudomonadati FCB group Bacteroidota Chlorobiota group Ignavibacteriota Ignavibacteriota bacterium RPI15242.1 312 aa 312 aa
BacClassIA Pseudomonadati FCB group Bacteroidota Chlorobiota group Ignavibacteriota Ignavibacteria bacterium HMQ79519.1 312 aa 312 aa
BacClassIA Pseudomonadati FCB group Bacteroidota Chlorobiota group Chlorobiota Chlorobiota bacterium MCI0450699.1 312 aa 312 aa
BacClassIA Pseudomonadati FCB group Bacteroidota Chlorobiota group Ignavibacteriota Ignavibacteria bacterium HRE39954.1 312 aa 312 aa
BacClassIA Pseudomonadati FCB group Bacteroidota Chlorobiota group Ignavibacteriota Ignavibacteriota bacterium MCB0721604.1 312 aa 312 aa
BacClassIA Pseudomonadati FCB group Bacteroidota Chlorobiota group Chlorobiota Chlorobiota bacterium KAA0211554.1 312 aa 312 aa
BacClassIA Pseudomonadati FCB group Bacteroidota Chlorobiota group Ignavibacteriota Ignavibacteria bacterium MBX7041586.1 312 aa 312 aa
BacClassIA Pseudomonadati FCB group Bacteroidota Chlorobiota group Ignavibacteriota Ignavibacteria bacterium MGB9697351.1 310 aa 310 aa
BacClassIA Pseudomonadati FCB group Bacteroidota Chlorobiota group Ignavibacteriota Ignavibacteriota bacterium MCX6162550.1 311 aa 311 aa
BacClassIA Pseudomonadati FCB group Bacteroidota Chlorobiota group Ignavibacteriota Ignavibacteria bacterium MDD5363573.1 311 aa 311 aa
BacClassIA Pseudomonadati FCB group Bacteroidota Chlorobiota group Ignavibacteriota Ignavibacteriota bacterium MCX6157347.1 311 aa 311 aa
BacClassIA Pseudomonadati FCB group Bacteroidota Chlorobiota group Ignavibacteriota Ignavibacteria bacterium MFA5012147.1 311 aa 311 aa
BacClassIC Pseudomonadati Pseudomonadota Gammaproteobacteria Pseudomonadales Pseudomonadales bacterium isolate Co333 bin36 MDR0463144.1 301 aa
BacClassIC Pseudomonadati Spirochaetota unclassified Spirochaetota Spirochaetota bacterium isolate AMR MDS 2156 iso1 HOP29559.1 302 aa 302 aa
BacClassIC Bacillati Bacillota Clostridia Eubacteriales Clostridiaceae bacterium isolate AS06rmzACSIP 448 NLE25430.1 297 aa 297 aa
BacClassIC Pseudomonadati PVC group Planctomycetota Planctomycetia Tundrisphaera sp TA3 WP 435017357.1 327 aa 327 aa
BacClassIC Pseudomonadati PVC group Planctomycetota Planctomycetia Tundrisphaera lichenicola strain P12 WP 435006722.1 328 aa 328 aa
BacClassIC Pseudomonadati PVC group Planctomycetota Planctomycetia Singulisphaera sp PoT WP 422926560.1 329 aa 329 aa
BacClassIC Pseudomonadati PVC group Planctomycetota Planctomycetia Singulisphaera acidiphila WP 015246484.1 329 aa 329 aa
BacClassIC Pseudomonadati Nitrospirota Nitrospiria Nitrospirales Singulisphaera sp isolate RUP SRG 30 MEJ7639749.1 299 aa 299 aa
BacClassIC Pseudomonadati PVC group Planctomycetota Planctomycetia Aquisphaera insulae WP 165228700.1 330 aa 330 aa
BacClassIC Pseudomonadati PVC group Planctomycetota Planctomycetia Paludisphaera sp GCF 037189885.1 WP 337177145.1 330 aa 330 aa
BacClassIC Pseudomonadati PVC group Planctomycetota Planctomycetia Paludisphaera borealis GCF 031291815.1 WP 373926491.1 340 aa 340 aa
BacClassIC Pseudomonadati PVC group Planctomycetota Planctomycetia Symmachiella dynata WP 145380456.1 336 aa 336 aa
BacClassIC Pseudomonadati PVC group Planctomycetota Planctomycetia Maioricimonas rarisocia strain Mal4 WP 145370025.1 334 aa 334 aa
BacClassIC Pseudomonadati PVC group Planctomycetota Planctomycetia Rubinisphaera margarita strain ICM H10 WP 238756390.1 334 aa 334 aa
BacClassIC Pseudomonadati PVC group Planctomycetota Planctomycetia Planctomicrobium sp SH664 WP 437206642.1 334 aa 334 aa
BacClassIC Pseudomonadati PVC group Planctomycetota Planctomycetia Thalassoroseus pseudoceratinae strain JC658 WP 166828996.1 333 aa 333 aa
BacClassIC Pseudomonadati Pseudomonadota Alphaproteobacteria Rhodobacterales Boseongicola sp isolate KFIS4 MEK6217534.1 288 aa 288 aa
BacClassIC Pseudomonadati PVC group Planctomycetota Planctomycetia Fuerstiella sp isolate CI.59.F.A1 421 001 iso1 MEP3480304.1 339 aa 339 aa
BacClassIC Pseudomonadati PVC group Planctomycetota Planctomycetia Fuerstiella sp isolate MFD06880 ilm asm bin27 iso1 MFY9254022.1 339 a 339 aa
BacClassIC Pseudomonadati PVC group Planctomycetota Planctomycetia Fuerstiella marisgermanici strain NH11 WP 083732647.1 339 aa 339 aa
BacClassIC Pseudomonadati PVC group Planctomycetota Planctomycetia Fuerstiella sp isolate cluster2 bin.2 MDG2129689.1 339 aa 339 aa
BacClassIC Pseudomonadati PVC group Planctomycetota Planctomycetia Fuerstiella sp isolate ALOHA ZT 14 MCH2202602.1 339 aa 339 aa
BacClassIC Bacillati Bacillota Tissierellia Tissierellales Sporosalibacterium faouarense strain SOL3f37 WP 202711085.1 315 aa 315 aa
BacClassIC Bacillati Bacillota Clostridia Christensenellales Christensenellaceae bacterium isolate RGIG2399 MBQ3274328.1 318 aa 318 aa
BacClassIC Bacillati Bacillota Bacilli Bacillales Lederbergia ruris WP 212966221.1 318 aa 318 aa
BacClassIC Pseudomonadati Spirochaetota Spirochaetia Spirochaetales Rectinemataceae bacterium isolate SEr3G3H3I bin 236 MGO8693439.1 321 321 aa
BacClassIC Bacillati Bacillota Clostridia Eubacteriales Oscillospiraceae bacterium isolate TD116 bin58 MDR2933027.1 315 aa 315 aa
BacClassIC Bacillati Chloroflexota Anaerolineae Anaerolineales Anaerolineaceae bacterium isolate N3 21 MBA3074071.1 321 aa 321 aa
BacClassIC Bacillati Bacillota Clostridia Eubacteriales Clostridiaceae bacterium isolate AS10tlH2TH 63 NLN47367.1 316 aa 316 aa
BacClassIC Bacillati Bacillota Clostridia Eubacteriales Clostridiaceae bacterium isolate AS04akNAM 113 iso1 HHW30117.1 320 aa 320 aa
BacClassIC Bacillati Bacillota Clostridia Eubacteriales Thermoclostridium stercorarium WP 015359078.1 318 aa 318 aa
BacClassIC Bacillati Bacillota Clostridia Eubacteriales Thermoclostridium sp isolate SMAG U7097 HEY8420581.1 326 aa 326 aa
BacClassIC Bacillati Bacillota Clostridia Eubacteriales Ruminiclostridium sp isolate STF1 139 MDD4296079.1 331 aa 331 aa
BacClassIC Bacillati Bacillota Clostridia Eubacteriales Clostridiaceae bacterium isolate AS10tlH2TH 408 NLN65239.1 324 aa 324 aa
BacClassIC Bacillati Bacillota Clostridia Eubacteriales Clostridiaceae bacterium isolate AS06rmzACSIP 332 NLV63145.1 326 aa 326 aa
BacClassIC Bacillati Bacillota Clostridia Lachnospirales Defluviitaleaceae bacterium isolate Emb289P1bin52 iso1 MCL2404220.1 319 aa 319 aa
BacClassIC Bacillati Bacillota Clostridia Lachnospirales Defluviitaleaceae bacterium isolate Lab288P1bin87 iso2 MCL2527969.1 319 aa 319 aa
BacClassIC Bacillati Bacillota Clostridia Lachnospirales Defluviitaleaceae bacterium isolate Emb289P1bin126 iso1 MCL2362296.1 319 aa 319 aa
BacClassIC Pseudomonadati Spirochaetota Spirochaetota Spirochaetia Treponema sp isolate Th196P3bin120 MCL2008527.1 316 aa 316 aa
BacClassIC Pseudomonadati Spirochaetota Spirochaetota Spirochaetia Treponema sp isolate Roe453 bin107 MDR3193212.1 316 aa 316 aa
BacClassIC Bacillati Bacillota Clostridia Lachnospirales Defluviitaleaceae bacterium isolate Emb289P1bin58 iso2 MCL2407095.1 318 aa 318 aa
BacClassIC Bacillati Bacillota Clostridia Eubacteriales Oscillospiraceae bacterium isolate Gx485 bin182 MDR1117507.1 347 aa 347 aa
BacClassIC Bacillati Bacillota Clostridia Lachnospirales Defluviitaleaceae bacterium isolate Lab288P3bin217 MCL2499952.1 315 aa 315 aa
BacClassIC Bacillati Bacillota Clostridia Lachnospirales Defluviitaleaceae bacterium isolate Lab288P3bin199 MCL2664334.1 314 aa 314 aa
BacClassIC Bacillati Bacillota Clostridia Eubacteriales Oscillospiraceae bacterium isolate Lab288P3bin93 MCL2517897.1 315 aa 315 aa
BacClassIC Bacillati Bacillota Clostridia Eubacteriales Oscillospiraceae bacterium isolate Emb289P4bin38 MCL2772167.1 335 aa 335 aa
BacClassIC Bacillati Bacillota Clostridia Eubacteriales Oscillospiraceae bacterium isolate Emb289P1bin61 MCL2408870.1 315 aa 315 aa
BacClassIC Pseudomonadati Pseudomonadota Betaproteobacteria Burkholderiales Piscinibacter sp GCF 035513255.1 WP 325480966.1 315 aa 315 aa
BacClassIC Bacillati Chloroflexota Chloroflexia Chloroflexales Oscillochloris sp ZM17 4 WP 220597221.1 315 aa 315 aa
BacClassIC Bacillati Chloroflexota Chloroflexia Chloroflexales Candidatus Oscillochloris fontis WP 129633562.1 315 aa 315 aa
BacClassIC Bacillati Chloroflexota Chloroflexia Chloroflexales Oscillochloris sp isolate RM1 1 4 iso1 NJN15712.1 315 aa 315 aa
BacClassIC Bacillati Chloroflexia Chloroflexales Chloroflexineae Chloroflexaceae bacterium isolate SKW209 iso2 MCX7790576.1 318 aa 318 aa
BacClassIC Bacillati Chloroflexota Chloroflexia Chloroflexales Chloroflexales bacterium isolate ELA086 MEI8165913.1 315 aa 315 aa
BacClassIC Bacillati Chloroflexota Chloroflexia Chloroflexales Oscillochloridaceae bacterium umkhey bin13 MFV9505543.1 315 aa 315 aa
BacClassIC Bacillati Chloroflexia Chloroflexales Chloroflexineae Chloroflexaceae bacterium isolate AMR MDS 0156 iso2 HMQ34939.1 303 aa 303 aa
BacClassIC Pseudomonadati FCB group Candidatus Zixiibacteriota Candidatus Zixiibacteriota bacterium isolate RS 15 10 MCK4462078.1 315 a 315 aa
BacClassIC Pseudomonadati FCB group Candidatus Zixiibacteriota Candidatus Zixiibacteriota bacterium isolate 24 Jan SF Bin5 iso1 MDH389008 314 aa
BacClassIC Pseudomonadati FCB group Candidatus Zixiibacteriota Candidatus Zixiibacteriota bacterium isolate B120 G9 iso2 RKX28345.1 314 314 aa
BacClassIC Pseudomonadati FCB group Candidatus Zixiibacteriota Candidatus Zixiibacteriota bacterium isolate SpSt 1246 iso1 HEQ98327.1 31 315 aa
BacClassIC Pseudomonadati FCB group Candidatus Zixiibacteriota Candidatus Zixiibacteriota bacterium isolate Bin 249 MBD3218405.1 315 aa 315 aa
BacClassIC Pseudomonadati Nitrospirota Nitrospiria Nitrospirales Candidatus Brocadiaceae bacterium isolate D1103U20metaG sub Bin 331 iso1 315 aa
BacClassIC Pseudomonadati FCB group Candidatus Zixiibacteriota Candidatus Zixiibacteriota bacterium isolate Old saline planktonic.mb.35 i 314 aa
BacClassIC Pseudomonadati FCB group Candidatus Zixiibacteriota Candidatus Zixiibacteriota bacterium isolate RS 12 25 iso2 MCK4657057.1 3 315 aa
BacClassIC Pseudomonadati FCB group Candidatus Zixiibacteriota Candidatus Zixiibacteriota bacterium isolate bin25 iso1 UCD95447.1 315 aa 315 aa
BacClassIC Pseudomonadati FCB group Candidatus Zixiibacteriota Candidatus Zixiibacteriota bacterium isolate B117 G9 iso2 RKX27953.1 314 314 aa
BacClassIC Pseudomonadati FCB group Candidatus Zixiibacteriota Candidatus Zixiibacteriota bacterium isolate MAG268 MEW5923418.1 315 aa 315 aa
BacClassIC Pseudomonadati FCB group Candidatus Zixiibacteriota candidate division Zixibacteria bacterium HGW Zixibacteria 1 PKK82434.1 3 315 aa
BacClassIC Pseudomonadati FCB group Candidatus Zixiibacteriota Candidatus Zixiibacteriota bacterium isolate bin142 iso2 UCD18661.1 314 a 314 aa
BacClassIC CPR Candidatus Poribacteria Candidatus Poribacteria bacterium isolate B30T1B8 MCH8292162.1 315 aa 315 aa
BacClassIC Bacillati Chloroflexota Anaerolineae Anaerolineales Anaerolineae bacterium isolate H5 CFX7 MBE7552033.1 316 aa 316 aa
BacClassIC Bacillati Chloroflexota Ktedonobacteria Ktedonobacterales Ktedonobacter sp isolate UBA11361 HBE29377.1 314 aa 314 aa
BacClassIC Bacillati Chloroflexota Ktedonobacteria Ktedonobacterales Ktedonobacteraceae bacterium isolate CP BM RX R7 7 MBV9690525.1 315 315 aa
BacClassIC Bacillati Chloroflexota Ktedonobacteria Ktedonobacterales Thermosporothrix hazakensis strain ATCC BAA 1881 WP 111324605.1 315 315 aa
BacClassIC Bacillati Chloroflexota Ktedonobacteria Ktedonobacterales Ktedonosporobacter sp isolate SMAG U9905 HLG62910.1 317 aa 317 aa
BacClassIC Bacillati Chloroflexota Ktedonobacteria Ktedonobacterales Reticulibacter mediterranei strain 150040 WP 373324608.1 310 aa 310 aa
BacClassIC Bacillati Chloroflexota Ktedonobacteria Ktedonobacterales Dictyobacter aurantiacus strain S27 WP 126595177.1 316 aa 316 aa
BacClassIC Bacillati Chloroflexota Ktedonobacteria Ktedonobacterales Ktedonosporobacter rubrisoli strain SCAWS G2 WP 129890088.1 314 aa 314 aa
BacClassIC Bacillati Chloroflexota Ktedonobacteria Ktedonobacterales Ktedonosporobacter sp isolate SMAG U9703 iso2 HLZ62038.1 308 aa 308 aa
BacClassIC Bacillati Chloroflexota Ktedonobacteria Ktedonobacterales Ktedonobacteraceae bacterium isolate SMAG U6416 HEV2656341.1 317 aa 317 aa
BacClassIC Bacillati Chloroflexota Ktedonobacteria Ktedonobacterales Ktedonobacter sp isolate UBA7999 HCI82850.1 314 aa 314 aa
BacClassIC Bacillati Chloroflexota Ktedonobacteria Ktedonobacterales Tengunoibacter tsumagoiensis strain Uno3 WP 126579166.1 317 aa 317 aa
BacClassIC CPR Candidatus Poribacteria Candidatus Poribacteria bacterium isolate RHO3 bin 62 iso2 MDE0042554.1 319 aa 319 aa
BacClassIC Pseudomonadati Thermodesulfobacteriota Desulfarculia Candidatus Adiutricales bacterium isolate SCS8bin.20 iso1 MEE9556361.1 3 315 aa
BacClassIC Pseudomonadati Spirochaetota Spirochaetia Spirochaetales Spirochaetales bacterium isolate co234 bin41 MBT3273867.1 300 aa 300 aa
BacClassIC Pseudomonadati Spirochaetota Spirochaetia Spirochaetales Spirochaetales bacterium isolate Zod Metabat.1463 MBN1685781.1 314 aa 314 aa
BacClassIC Pseudomonadati Spirochaetota Spirochaetia Spirochaetales Spirochaetales bacterium isolate MAG.283 iso1 MCG8452125.1 318 aa 318 aa
BacClassIC Pseudomonadati Spirochaetota Spirochaetia Spirochaetales Spirochaetaceae bacterium isolate C14 11 iso1 MDF1566525.1 315 aa 315 aa
BacClassIC Pseudomonadati Spirochaetota Spirochaetia Spirochaetales Spirochaetaceae bacterium isolate RS 5 70 MCK5249806.1 315 aa 315 aa
BacClassIC Pseudomonadati Spirochaetota Spirochaetia Spirochaetales Spirochaetaceae bacterium isolate AUK289 MCD6343709.1 315 aa 315 aa
BacClassIC Bacillati Actinomycetota Nitriliruptoria Egibacter rhizosphaerae strain EGI 80759 WP 131156092.1 315 aa 315 aa
BacClassIC Bacillati Chloroflexota Chloroflexia Chloroflexales Roseiflexaceae bacterium isolate SMAG U13340 iso1 HWQ14937.1 310 aa 310 aa
BacClassIC Bacillati Chloroflexota Chloroflexia Chloroflexales unclassified Roseiflexus multispecies WP 011955727.1 315 aa 315 aa
BacClassIC CPR candidate division KD3 62 candidate division KD3 62 bacterium DG 56 KPJ64457.1 315 aa 315 aa
BacClassIC Thermotogati Deinococcota unclassified Deinococcota Deinococcota bacterium isolate MSU bin45 iso2 MDQ3459127.1 315 aa 315 aa
BacClassIC Pseudomonadati FCB group Candidatus Latescibacterota Candidatus Latescibacterota bacterium isolate NC groundwater 459 Ag B 0.1 315 aa
BacClassIC Pseudomonadati FCB group Candidatus Hydrogenedentota Candidatus Hydrogenedentota bacterium isolate NC groundwater 1878 Pr3 B 0 315 aa
BacClassIC Pseudomonadati FCB group Candidatus Hydrogenedentota Candidatus Hydrogenedentota bacterium isolate NC groundwater 1458 Ag S 0 315 aa
BacClassIC Pseudomonadati FCB group Candidatus Latescibacterota Candidatus Latescibacterota bacterium isolate 3300053785 159 MFT7692514 314 aa
BacClassIC CPR Candidatus Handelsmaniibacteriota Candidatus Handelsmanbacteria bacterium isolate UWMA 0286 HIG55941.1 313 aa 313 aa
BacClassIC CPR Candidatus Sumerlaeota unclassified Candidatus Sumerlaeota Candidatus Sumerlaeota bacterium isolate LacPavin 0920 SED5 MAG 316 aa
BacClassIC Pseudomonadati Spirochaetota Spirochaetia Spirochaetales Spirochaetales bacterium isolate RS 6 9 iso1 MCK5155036.1 316 aa 316 aa
BacClassIC Pseudomonadati FCB group Candidatus Latescibacterota Candidatus Latescibacterota bacterium isolate OC3 500m MAG 41 MFP6591147 317 aa
BacClassIC Pseudomonadati FCB group Candidatus Hydrogenedentota Candidatus Hydrogenedentota bacterium isolate SpSt 1195 HEX73449.1 315 315 aa
BacClassIC Pseudomonadati FCB group Candidatus Hydrogenedentota Candidatus Hydrogenedentota bacterium isolate MAG 17963 hgd 111 HIJ73383 315 aa
BacClassIC Pseudomonadati FCB group Candidatus Hydrogenedentota Candidatus Hydrogenedentota bacterium isolate HYD2 iso2 MCL4218160.1 314 314 aa
BacClassIC Pseudomonadati FCB group Candidatus Hydrogenedentota Candidatus Hydrogenedentota bacterium isolate AMR MDS 1855 iso1 HOJ33964 315 aa
BacClassIC CPR Candidatus Sumerlaeota unclassified Candidatus Sumerlaeota Candidatus Sumerlaeota bacterium isolate Zod Metabat.1318 iso1 326 aa
BacClassIC Pseudomonadati FCB group Candidatus Hydrogenedentota Candidatus Hydrogenedentota bacterium isolate SJ408 MCC6697417.1 315 aa 315 aa
BacClassIC Pseudomonadati FCB group Candidatus Hydrogenedentota Candidatus Hydrogenedentota bacterium isolate AMX MBFR 82 iso1 GMV92340.1 315 aa
BacClassIC Pseudomonadati FCB group Candidatus Hydrogenedentota Candidatus Hydrogenedentota bacterium isolate AMX MBFR 4 iso1 GMU91311.1 316 aa
BacClassIC Pseudomonadati FCB group Candidatus Hydrogenedentota Candidatus Hydrogenedentota bacterium isolate AMR MDS 5617 HRI87102.1 3 316 aa
BacClassIC Pseudomonadati FCB group Candidatus Hydrogenedentota Candidatus Hydrogenedentota bacterium isolate SJ388 MCC6152383.1 316 aa 316 aa
BacClassIC Pseudomonadati FCB group Candidatus Hydrogenedentota Candidatus Hydrogenedentota bacterium isolate M DeepCast 65m m2 204 MBM3 316 aa
BacClassIC Pseudomonadati FCB group Candidatus Hydrogenedentota Candidatus Hydrogenedentota bacterium isolate AMX MBFR 84 iso2 GMV99750.1 315 aa
BacClassIC Pseudomonadati Myxococcota unclassified Myxococcota Myxococcota bacterium isolate OC3 2000m MAG 231 iso2 MFP8878163.1 313 aa 313 aa
BacClassIC Pseudomonadati Spirochaetota Spirochaetia Spirochaetales Spirochaetaceae bacterium isolate C18 maxbin.120 sub MDP3179005.1 318 318 aa
BacClassIC Pseudomonadati Thermodesulfobacteriota unclassified Thermodesulfobacteriota Thermodesulfobacteriota bacterium isolate SAG AM 3 318 aa
BacClassIC CPR Candidatus Binatota Candidatus Binatia unclassified Candidatus Binatia Candidatus Binatia bacterium isolate MAG.286 315 aa 315 aa
BacClassIC Bacillati Chloroflexota Caldilineae Caldilinea sp CFX5 MCE7986587.1 314 aa 314 aa

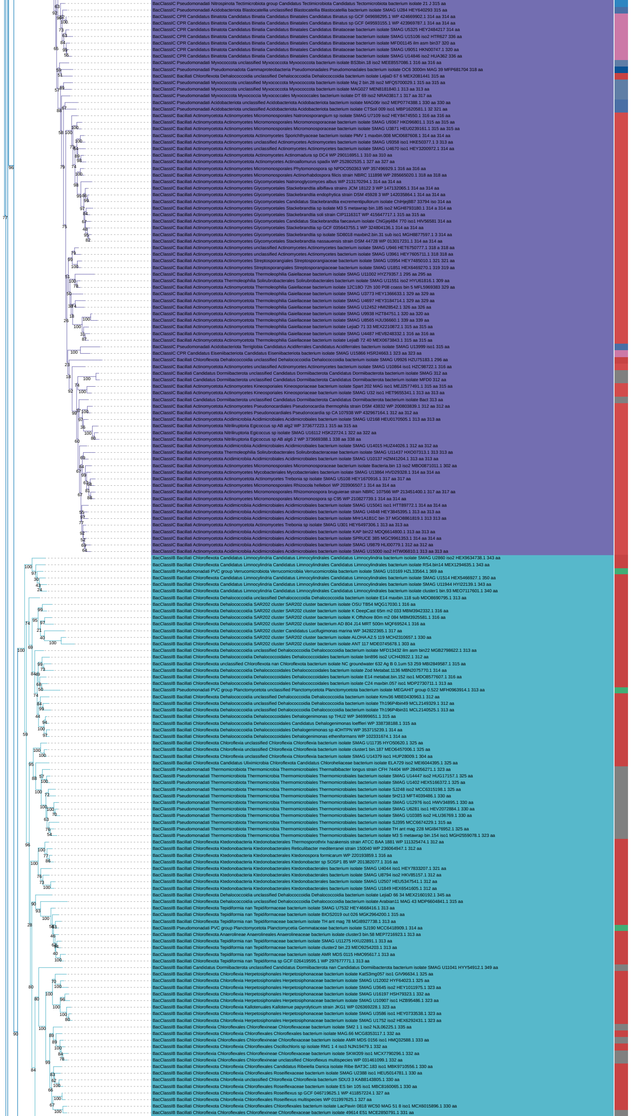

BacClassIC Pseudomonadati Nitrospinota Tectimicrobiota group Candidatus Tectimicrobiota Candidatus Tectomicrobia bacterium isolate 21 J 315 aa
BacClassIC Pseudomonadati Acidobacteriota Blastocatellia unclassified Blastocatellia Blastocatellia bacterium isolate SMAG U284 HEY640293 315 aa
BacClassIC CPR Candidatus Binatota Candidatus Binatia Candidatus Binatales Candidatus Binatus sp GCF 049698295.1 WP 424669902.1 314 aa 314 aa
BacClassIC CPR Candidatus Binatota Candidatus Binatia Candidatus Binatales Candidatus Binatus sp GCF 049593155.1 WP 423969787.1 314 aa 314 aa
BacClassIC CPR Candidatus Binatota Candidatus Binatia Candidatus Binatales Candidatus Binataceae bacterium isolate SMAG U5325 HEY2484217 314 aa
BacClassIC CPR Candidatus Binatota Candidatus Binatia Candidatus Binatales Candidatus Binataceae bacterium isolate SMAG U15106 iso2 HTR627 336 aa
BacClassIC CPR Candidatus Binatota Candidatus Binatia Candidatus Binatales Candidatus Binataceae bacterium isolate MFD01145 ilm asm bin37 320 aa
BacClassIC CPR Candidatus Binatota Candidatus Binatia Candidatus Binatales Candidatus Binataceae bacterium isolate SMAG U9051 HKN00747.1 320 aa
BacClassIC CPR Candidatus Binatota Candidatus Binatia Candidatus Binatales Candidatus Binataceae bacterium isolate SMAG U14846 iso2 HUA362 336 aa
BacClassIC Pseudomonadati Myxococcota unclassified Myxococcota Myxococcota bacterium isolate BS3bin.18 iso2 MEE8557086.1 316 aa 316 aa
BacClassIC Pseudomonadati Pseudomonadota Gammaproteobacteria Pseudomonadales Pseudomonadales bacterium isolate OC6 3000m MAG 39 MFP681704 318 aa
BacClassIC Bacillati Chloroflexota Dehalococcoidia unclassified Dehalococcoidia Dehalococcoidia bacterium isolate LejiaD 67 6 MEX2081441 315 aa
BacClassIC Pseudomonadati Myxococcota unclassified Myxococcota Myxococcota bacterium isolate Maj 2 bin.28 iso2 MFQ5700029.1 315 aa 315 aa
BacClassIC Pseudomonadati Myxococcota unclassified Myxococcota Myxococcota bacterium isolate MAG027 MEN8181840.1 313 aa 313 aa
BacClassIC Pseudomonadati Myxococcota Myxococcia Myxococcales Myxococcales bacterium isolate DT 69 iso2 NRA03817.1 317 aa 317 aa
BacClassIC Pseudomonadati Acidobacteriota unclassified Acidobacteriota Acidobacteriota bacterium isolate MAG06r iso2 MEP0774388.1 330 aa 330 aa
BacClassIC Pseudomonadati Acidobacteriota unclassified Acidobacteriota Acidobacteriota bacterium isolate CTSoil 009 iso1 MBP1620581.1 32 321 aa
BacClassIC Bacillati Actinomycetota Actinomycetes Micromonosporales Natronosporangium sp isolate SMAG U7109 iso2 HEY8474550.1 316 aa 316 aa
BacClassIC Bacillati Actinomycetota Actinomycetes Micromonosporales Micromonosporaceae bacterium isolate SMAG U9367 HKD96801.1 315 aa 315 aa
BacClassIC Bacillati Actinomycetota Actinomycetes Micromonosporales Micromonosporaceae bacterium isolate SMAG U3871 HEU0239161.1 315 aa 315 aa
BacClassIC Bacillati Actinomycetota Actinomycetota Actinomycetes Sporichthyaceae bacterium isolate PMV 1 maxbin.008 MCI0687608.1 314 aa 314 aa
BacClassIC Bacillati Actinomycetota Actinomycetes unclassified Actinomycetes Actinomycetes bacterium isolate SMAG U9358 iso1 HKE50377.1 3 313 aa
BacClassIC Bacillati Actinomycetota Actinomycetes unclassified Actinomycetes Actinomycetes bacterium isolate SMAG U4670 iso1 HEY3200972.1 314 aa
BacClassIC Bacillati Actinomycetota Actinomycetota Actinomycetota Actinomadura sp DC4 WP 290116951.1 310 aa 310 aa
BacClassIC Bacillati Actinomycetota Actinomycetota Actinomycetota Actinoallomurus spadix WP 252802535.1 327 aa 327 aa
BacClassIC Bacillati Actinomycetota Actinomycetes Micromonosporales Phytomonospora sp NPDC050363 WP 357496929.1 316 aa 316 aa
BacClassIC Bacillati Actinomycetota Actinomycetes Micromonosporales Actinorhabdospora filicis strain NBRC 111898 WP 285665020.1 318 aa 318 aa
BacClassIC Bacillati Actinomycetota Actinomycetes Glycomycetales Natronoglycomyces albus WP 213170294.1 314 aa 314 aa
BacClassIC Bacillati Actinomycetota Actinomycetes Glycomycetales Stackebrandtia albiflava strains JCM 18122 3 WP 147132065.1 314 aa 314 aa
BacClassIC Bacillati Actinomycetota Actinomycetes Glycomycetales Stackebrandtia endophytica strain DSM 45928 3 WP 142035864.1 314 aa 314 aa
BacClassIC Bacillati Actinomycetota Actinomycetes Glycomycetales Candidatus Stackebrandtia excrementipullorum isolate ChiHjej8B7 33794 iso 314 aa
BacClassIC Bacillati Actinomycetota Actinomycetes Glycomycetales Stackebrandtia sp isolate M3 S metawrap bin.185 iso2 MGH8793180.1 314 a 314 aa
BacClassIC Bacillati Actinomycetota Actinomycetes Glycomycetales Stackebrandtia soli strain CIP111631T WP 415647717.1 315 aa 315 aa
BacClassIC Bacillati Actinomycetota Actinomycetes Glycomycetales Candidatus Stackebrandtia faecavium isolate ChiGjej4B4 770 iso1 HIV56581 314 aa
BacClassIC Bacillati Actinomycetota Actinomycetes Glycomycetales Stackebrandtia sp GCF 035643755.1 WP 324804136.1 314 aa 314 aa
BacClassIC Bacillati Actinomycetota Actinomycetes Glycomycetales Stackebrandtia sp isolate SD8018 maxbin2.bin.31 sub iso1 MGH8877597.1 3 314 aa
BacClassIC Bacillati Actinomycetota Actinomycetes Glycomycetales Stackebrandtia nassauensis strain DSM 44728 WP 013017231.1 314 aa 314 aa
BacClassIC Bacillati Actinomycetota Actinomycetes unclassified Actinomycetes Actinomycetes bacterium isolate SMAG U946 HET6750777.1 318 a 318 aa
BacClassIC Bacillati Actinomycetota Actinomycetes unclassified Actinomycetes Actinomycetes bacterium isolate SMAG U3961 HEY7605711.1 318 318 aa
BacClassIC Bacillati Actinomycetota Actinomycetes Streptosporangiales Streptosporangiaceae bacterium isolate SMAG U3954 HEY7485010.1 321 321 aa
BacClassIC Bacillati Actinomycetota Actinomycetes Streptosporangiales Streptosporangiaceae bacterium isolate SMAG U1851 HEX6469270.1 319 319 aa
BacClassIC Bacillati Actinomycetota Actinomycetota Thermoleophilia Gaiellaceae bacterium isolate SMAG U11002 HYZ79357.1 295 aa 295 aa
BacClassIC Bacillati Actinomycetota Thermoleophilia Solirubrobacterales Solirubrobacterales bacterium isolate SMAG U11551 iso2 HYU61816.1 309 aa
BacClassIC Bacillati Actinomycetota Actinomycetota Thermoleophilia Gaiellaceae bacterium isolate 12C18O 72h 100 P08 coass bin 5 MFL5969383 329 aa
BacClassIC Bacillati Actinomycetota Actinomycetota Thermoleophilia Gaiellaceae bacterium isolate SMAG U3773 HEY1366633.1 329 aa 329 aa
BacClassIC Bacillati Actinomycetota Actinomycetota Thermoleophilia Gaiellaceae bacterium isolate SMAG U4697 HEY3184714.1 329 aa 329 aa
BacClassIC Bacillati Actinomycetota Actinomycetota Thermoleophilia Gaiellaceae bacterium isolate SMAG U12452 HMI28542.1 326 aa 326 aa
BacClassIC Bacillati Actinomycetota Actinomycetota Thermoleophilia Gaiellaceae bacterium isolate SMAG U9938 HZT84751.1 320 aa 320 aa
BacClassIC Bacillati Actinomycetota Actinomycetota Thermoleophilia Gaiellaceae bacterium isolate SMAG U8565 HJU36660.1 339 aa 339 aa
BacClassIC Bacillati Actinomycetota Actinomycetota Thermoleophilia Gaiellaceae bacterium isolate LejiaD 71 33 MEX2210872.1 315 aa 315 aa
BacClassIC Bacillati Actinomycetota Actinomycetota Thermoleophilia Gaiellaceae bacterium isolate SMAG U4487 HEV8248332.1 316 aa 316 aa
BacClassIC Bacillati Actinomycetota Actinomycetota Thermoleophilia Gaiellaceae bacterium isolate LejiaB 72 40 MEX0673843.1 315 aa 315 aa
BacClassIC Pseudomonadati Acidobacteriota Terriglobia Candidatus Acidiferrales Candidatus Acidiferrales bacterium isolate SMAG U13999 iso1 315 aa
BacClassIC CPR Candidatus Eiseniibacteriota Candidatus Eiseniibacteriota bacterium isolate SMAG U15866 HSR24663.1 323 aa 323 aa
BacClassIC Bacillati Chloroflexota Dehalococcoidia unclassified Dehalococcoidia Dehalococcoidia bacterium isolate SMAG U9926 HZU75183.1 296 aa
BacClassIC Bacillati Actinomycetota Actinomycetes unclassified Actinomycetes Actinomycetes bacterium isolate SMAG U10864 iso1 HZC98722.1 316 aa
BacClassIC Bacillati Candidatus Dormiibacterota unclassified Candidatus Dormiibacterota Candidatus Dormiibacterota bacterium isolate SMAG 312 aa
BacClassIC Bacillati Candidatus Dormiibacterota unclassified Candidatus Dormiibacterota Candidatus Dormiibacterota bacterium isolate MFD0 312 aa
BacClassIC Bacillati Actinomycetota Actinomycetes Kineosporiales Kineosporiales Kineosporiaceae bacterium isolate Spart 202 MAG iso1 MEJ2577491.1 315 aa 315 aa
BacClassIC Bacillati Actinomycetota Actinomycetes Kineosporiales Kineosporiaceae bacterium isolate SMAG U32 iso1 HET9655341.1 313 aa 313 aa
BacClassIC Bacillati Candidatus Dormiibacterota unclassified Candidatus Dormiibacterota Candidatus Dormiibacterota bacterium isolate Bact 313 aa
BacClassIC Bacillati Actinomycetota Actinomycetes Pseudonocardiales Pseudonocardia thermophila strain DSM 43832 WP 200803839.1 312 aa 312 aa
BacClassIC Bacillati Actinomycetota Actinomycetes Pseudonocardiales Pseudonocardia sp CA 107938 WP 432967164.1 312 aa 312 aa
BacClassIC Bacillati Actinomycetota Acidimicrobiia Acidimicrobiales Acidimicrobiales bacterium isolate SMAG U2168 HEU0170505.1 313 aa 313 aa
BacClassIC Bacillati Actinomycetota Nitriliruptoria Egicoccus sp AB alg2 WP 373677223.1 315 aa 315 aa
BacClassIC Bacillati Actinomycetota Nitriliruptoria Egicoccus sp isolate SMAG U16112 HSK22724.1 322 aa 322 aa
BacClassIC Bacillati Actinomycetota Nitriliruptoria Egicoccus sp AB alg6 2 WP 373669388.1 338 aa 338 aa
BacClassIC Bacillati Actinomycetota Acidimicrobiia Acidimicrobiales Acidimicrobiales bacterium isolate SMAG U14015 HUZ44026.1 312 aa 312 aa
BacClassIC Bacillati Actinomycetota Thermoleophilia Solirubrobacterales Solirubrobacteraceae bacterium isolate SMAG U11437 HXO07313.1 313 313 aa
BacClassIC Bacillati Actinomycetota Acidimicrobiia Acidimicrobiales Acidimicrobiales bacterium isolate SMAG U10137 HZM41204.1 313 aa 313 aa
BacClassIC Bacillati Actinomycetota Actinomycetes Micromonosporales Micromonosporaceae bacterium isolate Bacteria.bin 13 iso2 MBO0871011.1 302 aa
BacClassIC Bacillati Actinomycetota Actinomycetes Mycobacteriales Mycobacteriales bacterium isolate SMAG U13864 HVD29328.1 314 aa 314 aa
BacClassIC Bacillati Actinomycetota Actinomycetota Actinomycetes Trebonia sp isolate SMAG U5108 HEY1670916.1 317 aa 317 aa
BacClassIC Bacillati Actinomycetota Actinomycetes Micromonosporales Rhizocola hellebori WP 203906507.1 314 aa 314 aa
BacClassIC Bacillati Actinomycetota Actinomycetes Micromonosporales Rhizomonospora bruguierae strain NBRC 107566 WP 213451400.1 317 aa 317 aa
BacClassIC Bacillati Actinomycetota Actinomycetes Micromonosporales Micromonospora sp C95 WP 210827739.1 314 aa 314 aa
BacClassIC Bacillati Actinomycetota Acidimicrobiia Acidimicrobiales Acidimicrobiales bacterium isolate SMAG U15041 iso1 HTT89772.1 314 aa 314 aa
BacClassIC Bacillati Actinomycetota Acidimicrobiia Acidimicrobiales Acidimicrobiales bacterium isolate SMAG U4848 HEY3845395.1 313 aa 313 aa
BacClassIC Bacillati Actinomycetota Acidimicrobiia Acidimicrobiales Acidimicrobiales bacterium isolate MHr1A1B1C bin 37 MGO8861819.1 313 313 aa
BacClassIC Bacillati Actinomycetota Actinomycetota Actinomycetes Trebonia sp isolate SMAG U301 HEY6497306.1 313 aa 313 aa
BacClassIC Bacillati Actinomycetota Acidimicrobiia Acidimicrobiales Acidimicrobiales bacterium isolate KAP bin22 MDQ6614800.1 313 aa 313 aa
BacClassIC Bacillati Actinomycetota Acidimicrobiia Acidimicrobiales Acidimicrobiales bacterium isolate SPRUCE 385 MGC9961353.1 314 aa 314 aa
BacClassIC Bacillati Actinomycetota Acidimicrobiia Acidimicrobiales Acidimicrobiales bacterium isolate SMAG U9879 HLI00779.1 312 aa 312 aa
BacClassIC Bacillati Actinomycetota Acidimicrobiia Acidimicrobiales Acidimicrobiales bacterium isolate SMAG U15000 iso2 HTW06810.1 313 aa 313 aa
BacClassIB Bacillati Chloroflexota Candidatus Limnocylindria Candidatus Limnocylindrales Candidatus Limnocylindria bacterium isolate SMAG U2860 iso2 HEX9634738.1 343 aa
BacClassIB Bacillati Chloroflexota Candidatus Limnocylindria Candidatus Limnocylindrales Candidatus Limnocylindrales bacterium isolate RS4.bin14 MEX1294635.1 343 aa
BacClassIB Pseudomonadati PVC group Verrucomicrobiota Verrucomicrobiia Verrucomicrobiia bacterium isolate SMAG U10169 HZL33564.1 369 aa
BacClassIB Bacillati Chloroflexota Candidatus Limnocylindria Candidatus Limnocylindrales Candidatus Limnocylindrales bacterium isolate SMAG U1514 HEX5466927.1 350 aa
BacClassIB Bacillati Chloroflexota Candidatus Limnocylindria Candidatus Limnocylindrales Candidatus Limnocylindrales bacterium isolate SMAG U11944 HYI22139.1 343 aa
BacClassIB Bacillati Chloroflexota Candidatus Limnocylindria Candidatus Limnocylindrales Candidatus Limnocylindrales bacterium isolate cluster1 bin.93 MEO7117601.1 340 aa
BacClassIB Bacillati Chloroflexota Dehalococcoidia unclassified Dehalococcoidia Dehalococcoidia bacterium isolate E14 maxbin.118 sub MDO8690795.1 313 aa
BacClassIB Bacillati Chloroflexota Dehalococcoidia SAR202 cluster SAR202 cluster bacterium isolate OSU TB54 MQG17030.1 316 aa
BacClassIB Bacillati Chloroflexota Dehalococcoidia SAR202 cluster SAR202 cluster bacterium isolate K DeepCast 65m m2 033 MBM3942332.1 316 aa
BacClassIB Bacillati Chloroflexota Dehalococcoidia SAR202 cluster SAR202 cluster bacterium isolate K Offshore 80m m2 084 MBM3925581.1 316 aa
BacClassIB Bacillati Chloroflexota Dehalococcoidia SAR202 cluster SAR202 cluster bacterium AD 804 J14 MRT 500m MQF69524.1 316 aa
BacClassIB Bacillati Chloroflexota Dehalococcoidia SAR202 cluster Candidatus Lucifugimonas marina WP 342822365.1 317 aa
BacClassIB Bacillati Chloroflexota Dehalococcoidia SAR202 cluster SAR202 cluster bacterium isolate ALOHA A2.5 119 MCH2310657.1 330 aa
BacClassIB Bacillati Chloroflexota Dehalococcoidia SAR202 cluster SAR202 cluster bacterium isolate ANT 117 MDE0745678.1 303 aa
BacClassIB Bacillati Chloroflexota Dehalococcoidia unclassified Dehalococcoidia Dehalococcoidia bacterium isolate MFD13432 ilm asm bin22 MGB2798622.1 313 aa
BacClassIB Bacillati Chloroflexota Dehalococcoidia Dehalococcoidales Dehalococcoidales bacterium isolate bin896 iso2 UCH43922.1 312 aa
BacClassIB Bacillati Chloroflexota unclassified Chloroflexota nan Chloroflexota bacterium isolate NC groundwater 632 Ag B 0.1um 53 259 MBI2849587.1 315 aa
BacClassIB Bacillati Chloroflexota Dehalococcoidia Dehalococcoidales Dehalococcoidales bacterium isolate Zod Metabat.1136 MBN2075770.1 314 aa
BacClassIB Bacillati Chloroflexota Dehalococcoidia Dehalococcoidales Dehalococcoidales bacterium isolate E14 metabat.bin.152 iso1 MDO8577607.1 316 aa
BacClassIB Bacillati Chloroflexota Dehalococcoidia Dehalococcoidales Dehalococcoidales bacterium isolate C24 maxbin.057 iso1 MDP2730711.1 313 aa
BacClassIB Pseudomonadati PVC group Planctomycetota unclassified Planctomycetota Planctomycetota bacterium isolate MEGAHIT group 0.522 MFH0963914.1 313 aa
BacClassIB Bacillati Chloroflexota Dehalococcoidia unclassified Dehalococcoidia Dehalococcoidia bacterium isolate Kmv36 MBE0430963.1 312 aa
BacClassIB Bacillati Chloroflexota Dehalococcoidia unclassified Dehalococcoidia Dehalococcoidia bacterium isolate Th196P4bin49 MCL2149329.1 312 aa
BacClassIB Bacillati Chloroflexota Dehalococcoidia unclassified Dehalococcoidia Dehalococcoidia bacterium isolate Th196P4bin31 MCL2140525.1 313 aa
BacClassIB Bacillati Chloroflexota Dehalococcoidia Dehalococcoidales Dehalogenimonas sp THU2 WP 346999651.1 315 aa
BacClassIB Bacillati Chloroflexota Dehalococcoidia Dehalococcoidales Candidatus Dehalogenimonas loeffleri WP 338738188.1 315 aa
BacClassIB Bacillati Chloroflexota Dehalococcoidia Dehalococcoidales Dehalogenimonas sp 4OHTPN WP 353715239.1 314 aa
BacClassIB Bacillati Chloroflexota Dehalococcoidia Dehalococcoidales Dehalogenimonas etheniformans WP 102331674.1 314 aa
BacClassIB Bacillati Chloroflexota Chloroflexia unclassified Chloroflexia Chloroflexia bacterium isolate SMAG U11735 HYO50620.1 325 aa
BacClassIB Bacillati Chloroflexota Chloroflexia unclassified Chloroflexia Chloroflexia bacterium isolate cluster1 bin.187 MEO6457006.1 325 aa
BacClassIB Bacillati Chloroflexota Chloroflexia unclassified Chloroflexia Chloroflexia bacterium isolate SMAG U14379 iso1 HUP28009.1 304 aa
BacClassIB Bacillati Chloroflexota Candidatus Uliximicrobia Chloroflexota Candidatus Chloroheliaceae bacterium isolate ELA729 iso2 MEI6044395.1 325 aa
BacClassIB Pseudomonadati Thermomicrobiota Thermomicrobia Thermomicrobiales Thermalbibacter longus strain CFH 74404 WP 284056271.1 323 aa
BacClassIB Pseudomonadati Thermomicrobiota Thermomicrobia Thermomicrobiales Thermomicrobiales bacterium isolate SMAG U14447 iso2 HUG17157.1 325 aa
BacClassIB Pseudomonadati Thermomicrobiota Thermomicrobia Thermomicrobiales Thermomicrobiales bacterium isolate SMAG U1402 HEX5166372.1 325 aa
BacClassIB Pseudomonadati Thermomicrobiota Thermomicrobia Thermomicrobiales Thermomicrobiales bacterium isolate SJ248 iso2 MCC6315198.1 325 aa
BacClassIB Pseudomonadati Thermomicrobiota Thermomicrobia Thermomicrobiales Thermomicrobiales bacterium isolate SH213 MFT4039486.1 330 aa
BacClassIB Pseudomonadati Thermomicrobiota Thermomicrobia Thermomicrobiales Thermomicrobiales bacterium isolate SMAG U12976 iso1 HWV34895.1 330 aa
BacClassIB Pseudomonadati Thermomicrobiota Thermomicrobia Thermomicrobiales Thermomicrobiales bacterium isolate SMAG U6281 iso1 HEV2072884.1 330 aa
BacClassIB Pseudomonadati Thermomicrobiota Thermomicrobia Thermomicrobiales Thermomicrobiales bacterium isolate SMAG U10385 iso2 HLU36769.1 330 aa
BacClassIB Pseudomonadati Thermomicrobiota Thermomicrobia Thermomicrobiales Thermomicrobiales bacterium isolate SJ395 MCC6674229.1 315 aa
BacClassIB Pseudomonadati Thermomicrobiota Thermomicrobia Thermomicrobiales Thermomicrobiales bacterium isolate TH ant mag 228 MGI8476952.1 325 aa
BacClassIB Pseudomonadati Thermomicrobiota Thermomicrobia Thermomicrobiales Thermomicrobiales bacterium isolate M3 S metawrap bin.154 iso1 MGH2559078.1 323 aa
BacClassIB Bacillati Chloroflexota Ktedonobacteria Ktedonobacterales Thermosporothrix hazakensis strain ATCC BAA 1881 WP 111325474.1 312 aa
BacClassIB Bacillati Chloroflexota Ktedonobacteria Ktedonobacterales Reticulibacter mediterranei strain 150040 WP 236064947.1 312 aa
BacClassIB Bacillati Chloroflexota Ktedonobacteria Ktedonobacterales Ktedonospora formicarum WP 220193859.1 316 aa
BacClassIB Bacillati Chloroflexota Ktedonobacteria Ktedonobacterales Ktedonobacter sp SOSP1 85 WP 201382077.1 316 aa
BacClassIB Bacillati Chloroflexota Ktedonobacteria Ktedonobacterales Ktedonobacterales bacterium isolate SMAG U4044 iso1 HEY7833207.1 321 aa
BacClassIB Bacillati Chloroflexota Ktedonobacteria Ktedonobacterales Ktedonobacterales bacterium isolate SMAG U8794 iso2 HKV85157.1 312 aa
BacClassIB Bacillati Chloroflexota Ktedonobacteria Ktedonobacterales Ktedonobacterales bacterium isolate SMAG U2507 HEU5347541.1 312 aa
BacClassIB Bacillati Chloroflexota Ktedonobacteria Ktedonobacterales Ktedonobacterales bacterium isolate SMAG U1849 HEX6541605.1 312 aa
BacClassIB Bacillati Chloroflexota Dehalococcoidia unclassified Dehalococcoidia Dehalococcoidia bacterium isolate LejiaD 66 34 MEX2160192.1 345 aa
BacClassIB Bacillati Chloroflexota Dehalococcoidia unclassified Dehalococcoidia Dehalococcoidia bacterium isolate Arabian11 MAG 43 MDP6604841.1 315 aa
BacClassIB Bacillati Chloroflexota Tepidiformia nan Tepidiformaceae bacterium isolate SMAG U7532 HEY4668416.1 313 aa
BacClassIB Bacillati Chloroflexota Tepidiformia nan Tepidiformaceae bacterium isolate BIOS2019 out 026 MGK2964200.1 315 aa
BacClassIB Bacillati Chloroflexota Tepidiformia nan Tepidiformaceae bacterium isolate TH ant mag 78 MGI8927738.1 313 aa
BacClassIB Pseudomonadati PVC group Planctomycetota Planctomycetia Gemmataceae bacterium isolate SJ190 MCC6418909.1 314 aa
BacClassIB Bacillati Chloroflexota Anaerolineae Anaerolineales Anaerolineae bacterium isolate cluster3 bin.58 MEP7216923.1 313 aa
BacClassIB Bacillati Chloroflexota Tepidiformia nan Tepidiformaceae bacterium isolate SMAG U11275 HXU22891.1 313 aa
BacClassIB Bacillati Chloroflexota Tepidiformia nan Tepidiformaceae bacterium isolate cluster2 bin.23 MEO9254203.1 313 aa
BacClassIB Bacillati Chloroflexota Tepidiformia nan Tepidiformaceae bacterium isolate AMR MDS 0115 HMO95617.1 313 aa
BacClassIB Bacillati Chloroflexota Tepidiformia nan Tepidiforma sp GCF 026419595.1 WP 297677771.1 313 aa
BacClassIB Bacillati Candidatus Dormiibacterota unclassified Candidatus Dormiibacterota nan Candidatus Dormiibacterota bacterium isolate SMAG U11041 HYY54912.1 349 aa
BacClassIB Bacillati Chloroflexota Chloroflexia Herpetosiphonales Herpetosiphonaceae bacterium isolate KatS3mg057 iso1 GIV96634.1 325 aa
BacClassIB Bacillati Chloroflexota Chloroflexia Herpetosiphonales Herpetosiphonaceae bacterium isolate SMAG U12002 HYF64023.1 325 aa
BacClassIB Bacillati Chloroflexota Chloroflexia Herpetosiphonales Herpetosiphonaceae bacterium isolate SMAG U3645 iso2 HEY1011975.1 323 aa
BacClassIB Bacillati Chloroflexota Chloroflexia Herpetosiphonales Herpetosiphonaceae bacterium isolate SMAG U16197 HSH79323.1 332 aa
BacClassIB Bacillati Chloroflexota Chloroflexia Herpetosiphonales Herpetosiphonaceae bacterium isolate SMAG U10907 iso1 HZB95486.1 323 aa
BacClassIB Bacillati Chloroflexota Chloroflexia Kallotenuales Kallotenue papyrolyticum strain JKG1 WP 026369228.1 323 aa
BacClassIB Bacillati Chloroflexota Chloroflexia Herpetosiphonales Herpetosiphonaceae bacterium isolate SMAG U3586 iso1 HEY0733538.1 323 aa
BacClassIB Bacillati Chloroflexota Chloroflexia Herpetosiphonales Herpetosiphonaceae bacterium isolate SMAG U1752 iso2 HEX6292431.1 323 aa
BacClassIB Bacillati Chloroflexota Chloroflexia Chloroflexales Chloroflexineae Chloroflexaceae bacterium isolate SM2 1 1 iso2 NJL06225.1 335 aa
BacClassIB Bacillati Chloroflexota Chloroflexia Chloroflexales Chloroflexales bacterium isolate MAG.66 MCG8353117.1 332 aa
BacClassIB Bacillati Chloroflexota Chloroflexia Chloroflexales Chloroflexineae Chloroflexaceae bacterium isolate AMR MDS 0156 iso1 HMQ32588.1 333 aa
BacClassIB Bacillati Chloroflexota Chloroflexia Chloroflexales Oscillochloris sp isolate RM1 1 4 iso3 NJN19479.1 332 aa
BacClassIB Bacillati Chloroflexota Chloroflexia Chloroflexales Chloroflexineae Chloroflexaceae bacterium isolate SKW209 iso1 MCX7790296.1 332 aa
BacClassIB Bacillati Chloroflexota Chloroflexia Chloroflexales Chloroflexineae unclassified Chloroflexus multispecies WP 031461099.1 332 aa
BacClassIB Bacillati Chloroflexota Chloroflexia Chloroflexales Candidatus Ribeiella Danica isolate Ribe BAT3C.183 iso1 MBK9710556.1 330 aa
BacClassIB Bacillati Chloroflexota Chloroflexia Chloroflexales Roseiflexaceae bacterium isolate SMAG U2388 iso1 HEU5014781.1 330 aa
BacClassIB Bacillati Chloroflexota Chloroflexia Chloroflexales unclassified Chloroflexia Chloroflexia bacterium SDU3 3 KAB8143805.1 330 aa
BacClassIB Bacillati Chloroflexota Chloroflexia Chloroflexales Roseiflexaceae bacterium isolate ES bin 105 iso1 MBC8160065.1 330 aa
BacClassIB Bacillati Chloroflexota Chloroflexia Chloroflexales Roseiflexus sp GCF 046719625.1 WP 411857224.1 327 aa
BacClassIB Bacillati Chloroflexota Chloroflexia Chloroflexales Roseiflexus multispecies WP 011997625.1 327 aa
BacClassIB Bacillati Chloroflexota Chloroflexia Chloroflexales Chloroflexales bacterium isolate LacPavin 0818 WC50 MAG 51 8 iso1 MCX6015896.1 330 aa
BacClassIB Bacillati Chloroflexia Chloroflexales Chloroflexineae Chloroflexaceae bacterium isolate 49614 E51 MCE2850791.1 331 aa

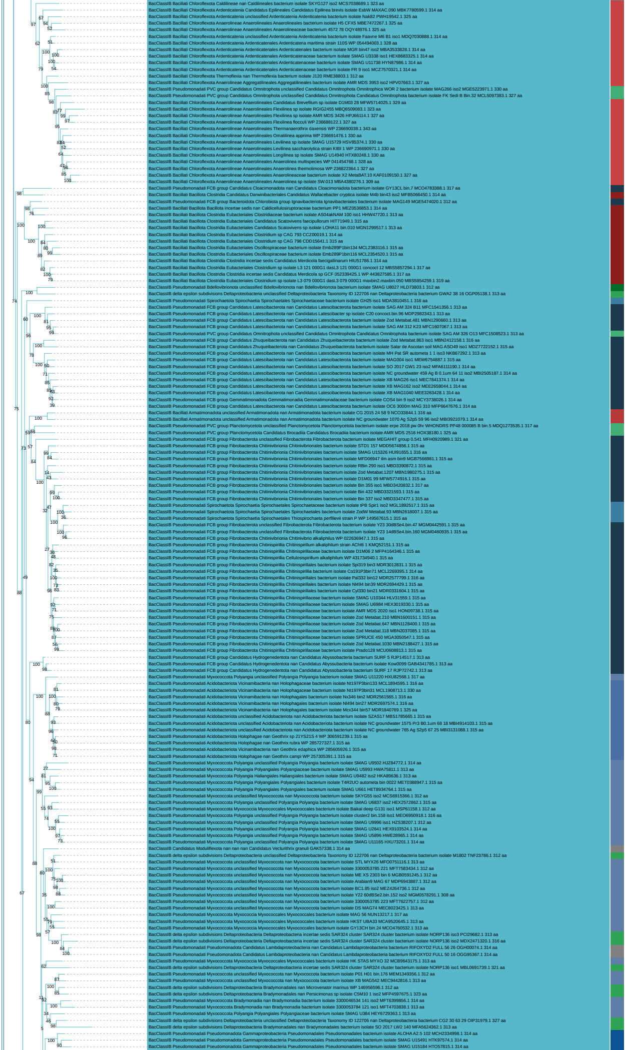
BacClassIB Bacillati Chloroflexota Caldilineae nan Caldilineales bacterium isolate SKYG127 iso2 MCS7038689.1 323 aa
BacClassIB Bacillati Chloroflexota Ardenticatenia Candidatus Epilineales Candidatus Epilinea brevis isolate EsbW MAXAC.090 MBK7780599.1 314 aa
BacClassIB Bacillati Chloroflexota Ardenticatenia unclassified Ardenticatenia Ardenticatenia bacterium isolate Nak82 PWH19542.1 325 aa
BacClassIB Bacillati Chloroflexota Anaerolineae Anaerolineales Anaerolineales bacterium isolate H5 CFX5 MBE7472267.1 325 aa
BacClassIB Bacillati Chloroflexota Anaerolineae Anaerolineales Anaerolineaceae bacterium 4572 78 OQY48976.1 325 aa
BacClassIB Bacillati Chloroflexota Ardenticatenia unclassified Ardenticatenia Ardenticatenia bacterium isolate Faavne M6 B1 iso1 MDQ7030888.1 314 aa
BacClassIB Bacillati Chloroflexota Ardenticatenia Ardenticatenales Ardenticatena maritima strain 110S WP 054494003.1 328 aa
BacClassIB Bacillati Chloroflexota Ardenticatenia Ardenticatenales Ardenticatenales bacterium isolate MGR bin47 iso2 MBA3533828.1 314 aa
BacClassIB Bacillati Chloroflexota Ardenticatenia Ardenticatenales Ardenticatenaceae bacterium isolate SMAG U3338 iso1 HEX8683325.1 314 aa
BacClassIB Bacillati Chloroflexota Ardenticatenia Ardenticatenales Ardenticatenaceae bacterium isolate SMAG U11738 HYN87986.1 314 aa
BacClassIB Bacillati Chloroflexota Ardenticatenia Ardenticatenales Ardenticatenaceae bacterium isolate FR 9 iso1 MCZ7570321.1 314 aa
BacClassIB Bacillati Chloroflexota Thermoflexia nan Thermoflexia bacterium isolate J120 RME38803.1 312 aa
BacClassIB Bacillati Chloroflexota Anaerolineae Aggregatilineales Aggregatilineales bacterium isolate AMR MDS 3953 iso2 HPV07663.1 327 aa
BacClassIB Pseudomonadati PVC group Candidatus Omnitrophota unclassified Candidatus Omnitrophota Omnitrophica WOR 2 bacterium isolate MAG266 iso2 MGE5223971.1 330 aa
BacClassIB Pseudomonadati PVC group Candidatus Omnitrophota unclassified Candidatus Omnitrophota Candidatus Omnitrophota bacterium isolate FK Sedi B Bin.32 MCL5097383.1 327 aa
BacClassIB Bacillati Chloroflexota Anaerolineae Anaerolineales Candidatus Brevefilum sp isolate D1M03 28 MFW5714025.1 329 aa
BacClassIB Bacillati Chloroflexota Anaerolineae Anaerolineales Flexilinea sp isolate RGIG2455 MBQ6509083.1 323 aa
BacClassIB Bacillati Chloroflexota Anaerolineae Anaerolineales Flexilinea sp isolate AMR MDS 3426 HPJ66114.1 327 aa
BacClassIB Bacillati Chloroflexota Anaerolineae Anaerolineales Flexilinea flocculi WP 236688122.1 327 aa
BacClassIB Bacillati Chloroflexota Anaerolineae Anaerolineales Thermanaerothrix daxensis WP 236690038.1 343 aa
BacClassIB Bacillati Chloroflexota Anaerolineae Anaerolineales Ornatilinea apprima WP 236691476.1 330 aa
BacClassIB Bacillati Chloroflexota Anaerolineae Anaerolineales Levilinea sp isolate SMAG U15729 HSV85374.1 330 aa
BacClassIB Bacillati Chloroflexota Anaerolineae Anaerolineales Levilinea saccharolytica strain KIBI 1 WP 236690971.1 330 aa
BacClassIB Bacillati Chloroflexota Anaerolineae Anaerolineales Longilinea sp isolate SMAG U14940 HTX80248.1 330 aa
BacClassIB Bacillati Chloroflexota Anaerolineae Anaerolineales Anaerolinea multispecies WP 041454768.1 328 aa
BacClassIB Bacillati Chloroflexota Anaerolineae Anaerolineales Anaerolinea thermolimosa WP 236822364.1 327 aa
BacClassIB Bacillati Chloroflexota Anaerolineae Anaerolineales Anaerolineaceae bacterium isolate X2 MetaBAT.10 KAF0109150.1 327 aa
BacClassIB Bacillati Chloroflexota Anaerolineae Anaerolineales Anaerolinea sp isolate SW.013 MBA4380276.1 309 aa
BacClassIB Pseudomonadati FCB group Candidatus Cloacimonadota Cloacimonadia nan Candidatus Cloacimonadota bacterium isolate GY13CL bin.7 MCO4783388.1 317 aa
BacClassIB Bacillati Bacillota Clostridia Candidatus Darwinibacteriales Candidatus Wallacebacter cryptica isolate M4b bin43 iso2 MFB5066450.1 314 aa
BacClassIB Pseudomonadati FCB group Bacteroidota Chlorobiota group Ignavibacteriota Ignavibacteria bacterium isolate MAG149 MGE5474020.1 312 aa
BacClassIB Bacillati Bacillota Bacillota incertae sedis nan Caldicellulosiruptoraceae bacterium PP1 MEZ0536853.1 314 aa
BacClassIB Bacillati Bacillota Clostridia Eubacteriales Clostridiaceae bacterium isolate AS04akNAM 100 iso1 HHW47720.1 313 aa
BacClassIB Bacillati Bacillota Clostridia Eubacteriales Candidatus Scatovivens faecipullorum HIT71949.1 315 aa
BacClassIB Bacillati Bacillota Clostridia Eubacteriales Candidatus Scatovivens sp isolate LOHA11 bin.010 MGN1299517.1 313 aa
BacClassIB Bacillati Bacillota Clostridia Eubacteriales Clostridium sp CAG 793 CCZ00019.1 314 aa
BacClassIB Bacillati Bacillota Clostridia Eubacteriales Clostridium sp CAG 798 CDD15641.1 315 aa
BacClassIB Bacillati Bacillota Clostridia Eubacteriales Oscillospiraceae bacterium isolate Emb289P1bin134 MCL2383116.1 315 aa
BacClassIB Bacillati Bacillota Clostridia Eubacteriales Oscillospiraceae bacterium isolate Emb289P1bin116 MCL2354520.1 315 aa
BacClassIB Bacillati Bacillota Clostridia Clostridia incertae sedis Candidatus Merdicola faecigallinarum HIU51786.1 314 aa
BacClassIB Bacillati Bacillota Clostridia Eubacteriales Clostridium sp isolate L3 121 000G1 dasL3 121 000G1 concoct 12 MBS5857294.1 317 aa
BacClassIB Bacillati Bacillota Clostridia Clostridia incertae sedis Candidatus Merdicola sp GCF 052339425.1 WP 443827585.1 317 aa
BacClassIB Bacillati Bacillota Clostridia Eubacteriales Clostridium sp isolate L3 079 000G1 dasL3 079 000G1 maxbin2.maxbin.050 MBS5854259.1 319 aa
BacClassIB Pseudomonadati Bdellovibrionota unclassified Bdellovibrionota nan Bdellovibrionota bacterium isolate SMAG U8027 HLD73803.1 312 aa
BacClassIB delta epsilon subdivisions Deltaproteobacteria unclassified Deltaproteobacteria Taxonomy ID 122706 nan Deltaproteobacteria bacterium GWA2 38 16 OGP05138.1 313 aa
BacClassIB Pseudomonadati Spirochaetota Spirochaetia Spirochaetales Spirochaetaceae bacterium isolate GH25 iso1 MDA3810451.1 316 aa
BacClassIB Pseudomonadati FCB group Candidatus Latescibacterota nan Candidatus Latescibacterota bacterium isolate SAG AM 324 B11 MFC1541356.1 313 aa
BacClassIB Pseudomonadati FCB group Candidatus Latescibacterota nan Candidatus Latescibacter sp isolate C20 concoct.bin.96 MDP2982343.1 313 aa
BacClassIB Pseudomonadati FCB group Candidatus Latescibacterota nan Candidatus Latescibacterota bacterium isolate Zod Metabat.481 MBN1290660.1 313 aa
BacClassIB Pseudomonadati FCB group Candidatus Latescibacterota nan Candidatus Latescibacterota bacterium isolate SAG AM 312 K23 MFC1607067.1 313 aa
BacClassIB Pseudomonadati PVC group Candidatus Omnitrophota unclassified Candidatus Omnitrophota Candidatus Omnitrophota bacterium isolate SAG AM 326 O13 MFC1508523.1 313 aa
BacClassIB Pseudomonadati FCB group Candidatus Zhuqueibacterota nan Candidatus Zhuqueibacterota bacterium isolate Zod Metabat.863 iso1 MBN2412158.1 316 aa
BacClassIB Pseudomonadati FCB group Candidatus Zhuqueibacterota nan Candidatus Zhuqueibacterota bacterium isolate Salar de Ascotan soil MAG ASO49 iso1 MDZ7722152.1 315 aa
BacClassIB Pseudomonadati FCB group Candidatus Latescibacterota nan Candidatus Latescibacterota bacterium isolate MH Pat SR autometa 1 1 iso3 NKB67292.1 313 aa
BacClassIB Pseudomonadati FCB group Candidatus Latescibacterota nan Candidatus Latescibacterota bacterium isolate MAG004 iso1 MEW6754887.1 315 aa
BacClassIB Pseudomonadati FCB group Candidatus Latescibacterota nan Candidatus Latescibacterota bacterium isolate SO 2017 GW1 23 iso2 MFA6111190.1 314 aa
BacClassIB Pseudomonadati FCB group Candidatus Latescibacterota nan Candidatus Latescibacterota bacterium isolate NC groundwater 459 Ag B 0.1um 64 11 iso2 MBI2505187.1 314 aa
BacClassIB Pseudomonadati FCB group Candidatus Latescibacterota nan Candidatus Latescibacterota bacterium isolate XB MAG26 iso1 MEC7841374.1 314 aa
BacClassIB Pseudomonadati FCB group Candidatus Latescibacterota nan Candidatus Latescibacterota bacterium isolate XB MAG162 iso2 MEE2658044.1 314 aa
BacClassIB Pseudomonadati FCB group Candidatus Latescibacterota nan Candidatus Latescibacterota bacterium isolate XB MAG1040 MEE3263428.1 314 aa
BacClassIB Pseudomonadati FCB group Gemmatimonadota Gemmatimonadia Gemmatimonadaceae bacterium isolate COS4 bin 9 iso2 MCY3738026.1 314 aa
BacClassIB Pseudomonadati FCB group Candidatus Latescibacterota nan Candidatus Latescibacterota bacterium isolate OC6 3000m MAG 310 MFP6647676.1 314 aa
BacClassIB Bacillati Armatimonadota unclassified Armatimonadota nan Armatimonadota bacterium isolate CG 2015 24 58 9 NCO33844.1 316 aa
BacClassIB Bacillati Armatimonadota unclassified Armatimonadota nan Armatimonadota bacterium isolate NC groundwater 1070 Ag S2p5 59 96 iso2 MBI3921079.1 314 aa
BacClassIB Pseudomonadati PVC group Planctomycetota unclassified Planctomycetota Planctomycetota bacterium isolate erpe 2018 pw 0hr WHONDRS PP48 000085 B bin.5 MDQ1273535.1 317 aa
BacClassIB Pseudomonadati PVC group Planctomycetota Candidatus Brocadiia Candidatus Brocadiia bacterium isolate AMR MDS 2516 HOX38180.1 325 aa
BacClassIB Pseudomonadati FCB group Fibrobacterota unclassified Fibrobacterota Fibrobacterota bacterium isolate MEGAHIT group 0.541 MFH0920989.1 321 aa
BacClassIB Pseudomonadati FCB group Fibrobacterota Chitinivibrionia Chitinivibrionales bacterium isolate STD1 157 MDD5674856.1 315 aa
BacClassIB Pseudomonadati FCB group Fibrobacterota Chitinivibrionia Chitinivibrionales bacterium isolate SMAG U15326 HUI91655.1 316 aa
BacClassIB Pseudomonadati FCB group Fibrobacterota Chitinivibrionia Chitinivibrionales bacterium isolate MFD06947 ilm asm bin9 MGB7566981.1 315 aa
BacClassIB Pseudomonadati FCB group Fibrobacterota Chitinivibrionia Chitinivibrionales bacterium isolate RBin 290 iso1 MBD3390872.1 315 aa
BacClassIB Pseudomonadati FCB group Fibrobacterota Chitinivibrionia Chitinivibrionales bacterium isolate Zod Metabat.1207 MBN1980275.1 315 aa
BacClassIB Pseudomonadati FCB group Fibrobacterota Chitinivibrionia Chitinivibrionales bacterium isolate D1M01 99 MFW5774916.1 315 aa
BacClassIB Pseudomonadati FCB group Fibrobacterota Chitinivibrionia Chitinivibrionales bacterium isolate Bin 355 iso1 MBD3420832.1 317 aa
BacClassIB Pseudomonadati FCB group Fibrobacterota Chitinivibrionia Chitinivibrionales bacterium isolate Bin 432 MBD3321593.1 315 aa
BacClassIB Pseudomonadati FCB group Fibrobacterota Chitinivibrionia Chitinivibrionales bacterium isolate Bin 337 iso2 MBD3347477.1 315 aa
BacClassIB Pseudomonadati Spirochaetota Spirochaetia Spirochaetales Spirochaetaceae bacterium isolate tPB Spir1 iso2 MGL1892517.1 315 aa
BacClassIB Pseudomonadati Spirochaetota Spirochaetia Spirochaetales Spirochaetales bacterium isolate ZodW Metabat.93 MBN2618007.1 315 aa
BacClassIB Pseudomonadati Spirochaetota Spirochaetia Spirochaetales Thiospirochaeta perfilievii strain P WP 149567615.1 315 aa
BacClassIB Pseudomonadati FCB group Fibrobacterota unclassified Fibrobacterota Fibrobacterota bacterium isolate Y23 30dBSe4.bin.47 MGM0442591.1 315 aa
BacClassIB Pseudomonadati FCB group Fibrobacterota unclassified Fibrobacterota Fibrobacterota bacterium isolate Y23 14dBSe4.bin.160 MGM0460935.1 315 aa
BacClassIB Pseudomonadati FCB group Fibrobacterota Chitinivibrionia Chitinivibrio alkaliphilus WP 022636947.1 315 aa
BacClassIB Pseudomonadati FCB group Fibrobacterota Chitinispirillia Chitinispirillum alkaliphilum strain ACht6 1 KMQ52151.1 315 aa
BacClassIB Pseudomonadati FCB group Fibrobacterota Chitinispirillia Chitinispirillaceae bacterium isolate D1M06 2 MFP4164346.1 315 aa
BacClassIB Pseudomonadati FCB group Fibrobacterota Chitinispirillia Cellulosispirillum alkaliphilum WP 431734940.1 315 aa
BacClassIB Pseudomonadati FCB group Fibrobacterota Chitinispirillia Chitinispirillales bacterium isolate Spi319 bin3 MDR3012831.1 315 aa
BacClassIB Pseudomonadati FCB group Fibrobacterota Chitinispirillia Chitinispirillia bacterium isolate Co191P3bin71 MCL2269395.1 314 aa
BacClassIB Pseudomonadati FCB group Fibrobacterota Chitinispirillia Chitinispirillales bacterium isolate Pal332 bin12 MDR2577799.1 316 aa
BacClassIB Pseudomonadati FCB group Fibrobacterota Chitinispirillia Chitinispirillales bacterium isolate Nl494 bin39 MDR2694429.1 315 aa
BacClassIB Pseudomonadati FCB group Fibrobacterota Chitinispirillia Chitinispirillales bacterium isolate Cyl330 bin21 MDR0331604.1 315 aa
BacClassIB Pseudomonadati FCB group Fibrobacterota Chitinispirillia Chitinispirillaceae bacterium isolate SMAG U10344 HLV31559.1 315 aa
BacClassIB Pseudomonadati FCB group Fibrobacterota Chitinispirillia Chitinispirillaceae bacterium isolate SMAG U6984 HEX3019330.1 315 aa
BacClassIB Pseudomonadati FCB group Fibrobacterota Chitinispirillia Chitinispirillaceae bacterium isolate AMR MDS 2020 iso1 HON09738.1 315 aa
BacClassIB Pseudomonadati FCB group Fibrobacterota Chitinispirillia Chitinispirillaceae bacterium isolate Zod Metabat.210 MBN1600151.1 315 aa
BacClassIB Pseudomonadati FCB group Fibrobacterota Chitinispirillia Chitinispirillaceae bacterium isolate Zod Metabat.647 MBN1128400.1 315 aa
BacClassIB Pseudomonadati FCB group Fibrobacterota Chitinispirillia Chitinispirillaceae bacterium isolate Zod Metabat.118 MBN2037085.1 315 aa
BacClassIB Pseudomonadati FCB group Fibrobacterota Chitinispirillia Chitinispirillaceae bacterium isolate SPRUCE 450 MGA3050547.1 315 aa
BacClassIB Pseudomonadati FCB group Fibrobacterota Chitinispirillia Chitinispirillaceae bacterium isolate Zod Metabat.1030 MBN2188427.1 315 aa
BacClassIB Pseudomonadati FCB group Fibrobacterota Chitinispirillia Chitinispirillaceae bacterium isolate Prado128 MCU0608813.1 315 aa
BacClassIB Pseudomonadati FCB group Candidatus Hydrogenedentota nan Candidatus Abyssobacteria bacterium SURF 5 RJP14517.1 313 aa
BacClassIB Pseudomonadati FCB group Candidatus Hydrogenedentota nan Candidatus Abyssobacteria bacterium isolate Kow0099 GAB4341785.1 313 aa
BacClassIB Pseudomonadati FCB group Candidatus Hydrogenedentota nan Candidatus Abyssobacteria bacterium SURF 17 RJP72742.1 313 aa
BacClassIB Pseudomonadati Myxococcota Polyangia unclassified Polyangia Polyangia bacterium isolate SMAG U11220 HXU82568.1 317 aa
BacClassIB Pseudomonadati Acidobacteriota Vicinamibacteria nan Holophagaceae bacterium isolate Nt197P3bin133 MCL1894595.1 316 aa
BacClassIB Pseudomonadati Acidobacteriota Vicinamibacteria nan Holophagaceae bacterium isolate Nt197P3bin31 MCL1908713.1 330 aa
BacClassIB Pseudomonadati Acidobacteriota Vicinamibacteria nan Holophagales bacterium isolate Nx346 bin2 MDR2561565.1 316 aa
BacClassIB Pseudomonadati Acidobacteriota Vicinamibacteria nan Holophagales bacterium isolate Nl494 bin27 MDR2697574.1 316 aa
BacClassIB Pseudomonadati Acidobacteriota Vicinamibacteria nan Holophagales bacterium isolate Mcx344 bin57 MDR1840769.1 325 aa
BacClassIB Pseudomonadati Acidobacteriota unclassified Acidobacteriota nan Acidobacteriota bacterium isolate SZAS17 MBS1785665.1 315 aa
BacClassIB Pseudomonadati Acidobacteriota unclassified Acidobacteriota nan Acidobacteriota bacterium isolate NC groundwater 1575 Pr3 B0.1um 68 18 MBI4914103.1 315 aa
BacClassIB Pseudomonadati Acidobacteriota unclassified Acidobacteriota nan Acidobacteriota bacterium isolate NC groundwater 765 Ag S2p5 67 25 MBI3131088.1 315 aa
BacClassIB Pseudomonadati Acidobacteriota Holophagae nan Geothrix sp 21YS21S 4 WP 306591239.1 315 aa
BacClassIB Pseudomonadati Acidobacteriota Holophagae nan Geothrix rubra WP 285727327.1 315 aa
BacClassIB Pseudomonadati Acidobacteriota Vicinamibacteria nan Geothrix edaphica WP 285605926.1 315 aa
BacClassIB Pseudomonadati Acidobacteriota Holophagae nan Geothrix campi WP 257305391.1 315 aa
BacClassIB Pseudomonadati Myxococcota Polyangia unclassified Polyangia Polyangia bacterium isolate SMAG U9502 HJZ84772.1 314 aa
BacClassIB Pseudomonadati Myxococcota Polyangia Polyangiales Polyangiaceae bacterium isolate SMAG U5993 HWA75811.1 313 aa
BacClassIB Pseudomonadati Myxococcota Polyangia Haliangiales Haliangiales bacterium isolate SMAG U9482 iso2 HKA89636.1 313 aa
BacClassIB Pseudomonadati Myxococcota Polyangia Polyangiales Polyangiales bacterium isolate T4R2UO autometa bin 0022 MET0388947.1 315 aa
BacClassIB Pseudomonadati Myxococcota Polyangia Polyangiales Polyangiales bacterium isolate SMAG U661 HET8934764.1 315 aa
BacClassIB Pseudomonadati Myxococcota unclassified Myxococcota nan Myxococcota bacterium isolate SKYG55 iso2 MCS6915366.1 312 aa
BacClassIB Pseudomonadati Myxococcota Polyangia unclassified Polyangia Polyangia bacterium isolate SMAG U6837 iso2 HEX2572862.1 315 aa
BacClassIB Pseudomonadati Myxococcota Myxococcia Myxococcales Myxococcales bacterium isolate Baikal deep G131 iso1 MSP61158.1 312 aa
BacClassIB Pseudomonadati Myxococcota Polyangia unclassified Polyangia Polyangia bacterium isolate cluster2 bin.158 iso1 MEO6950918.1 316 aa
BacClassIB Pseudomonadati Myxococcota Polyangia unclassified Polyangia Polyangia bacterium isolate SMAG U9996 iso1 HZS38207.1 312 aa
BacClassIB Pseudomonadati Myxococcota Polyangia unclassified Polyangia Polyangia bacterium isolate SMAG U2641 HEX9103524.1 314 aa
BacClassIB Pseudomonadati Myxococcota Polyangia unclassified Polyangia Polyangia bacterium isolate SMAG U5896 HWE28965.1 314 aa
BacClassIB Pseudomonadati Myxococcota Polyangia unclassified Polyangia Polyangia bacterium isolate SMAG U11165 HXU73201.1 314 aa
BacClassIB Candidatus Moduliflexota nan nan nan Candidatus Vecturithrix granuli GAK57338.1 314 aa
BacClassIB delta epsilon subdivisions Deltaproteobacteria unclassified Deltaproteobacteria Taxonomy ID 122706 nan Deltaproteobacteria bacterium isolate M1802 TNF23786.1 312 aa
BacClassIB Pseudomonadati Myxococcota unclassified Myxococcota nan Myxococcota bacterium isolate STL MYX26 MFO0751116.1 313 aa
BacClassIB Pseudomonadati Myxococcota unclassified Myxococcota nan Myxococcota bacterium isolate 3300053785 221 MFT7583434.1 312 aa
BacClassIB Pseudomonadati Myxococcota unclassified Myxococcota nan Myxococcota bacterium isolate ME XS 2303 bin 6 MGB0591245.1 312 aa
BacClassIB Pseudomonadati Myxococcota unclassified Myxococcota nan Myxococcota bacterium isolate Arabian9 MAG 67 MDP6943887.1 312 aa
BacClassIB Pseudomonadati Myxococcota unclassified Myxococcota nan Myxococcota bacterium isolate BC1.85 iso2 MEZ4264736.1 312 aa
BacClassIB Pseudomonadati Myxococcota unclassified Myxococcota nan Myxococcota bacterium isolate Y22 60dBSe2.bin.152 iso2 MGM0578291.1 308 aa
BacClassIB Pseudomonadati Myxococcota unclassified Myxococcota nan Myxococcota bacterium isolate 3300053785 223 MFT7622757.1 312 aa
BacClassIB Pseudomonadati Myxococcota unclassified Myxococcota nan Myxococcota bacterium isolate DS MAG74 MEC8023425.1 313 aa
BacClassIB Pseudomonadati Myxococcota Myxococcia Myxococcales Myxococcales bacterium isolate MAG 56 NUN13217.1 317 aa
BacClassIB Pseudomonadati Myxococcota Myxococcia Myxococcales Myxococcales bacterium isolate HKST UBA33 MCA9520645.1 313 aa
BacClassIB Pseudomonadati Myxococcota Myxococcia Myxococcales Myxococcales bacterium isolate GY13CH bin.24 MCO4760532.1 313 aa
BacClassIB delta epsilon subdivisions Deltaproteobacteria Deltaproteobacteria incertae sedis SAR324 cluster SAR324 cluster bacterium isolate NORP136 iso3 PCI29682.1 313 aa
BacClassIB delta epsilon subdivisions Deltaproteobacteria Deltaproteobacteria incertae sedis SAR324 cluster SAR324 cluster bacterium isolate NORP136 iso2 MDX2471320.1 316 aa
BacClassIB Pseudomonadati Pseudomonadota Candidatus Lambdaproteobacteria nan Candidatus Lambdaproteobacteria bacterium RIFOXYD2 FULL 56 26 OGH00074.1 314 aa
BacClassIB Pseudomonadati Pseudomonadota Candidatus Lambdaproteobacteria nan Candidatus Lambdaproteobacteria bacterium RIFOXYD2 FULL 50 16 OGG95367.1 314 aa
BacClassIB Pseudomonadati Myxococcota Myxococcia Myxococcales Myxococcales bacterium isolate HK STAS MYXO 32 MCB9643175.1 313 aa
BacClassIB delta epsilon subdivisions Deltaproteobacteria Deltaproteobacteria incertae sedis SAR324 cluster SAR324 cluster bacterium isolate NORP136 iso1 MBL0691739.1 321 aa
BacClassIB Pseudomonadati Myxococcota unclassified Myxococcota nan Myxococcota bacterium isolate P01 H01 bin.176 MEM1349356.1 312 aa
BacClassIB Pseudomonadati Myxococcota unclassified Myxococcota nan Myxococcota bacterium isolate XB MAG542 MEC9943816.1 313 aa
BacClassIB delta epsilon subdivisions Deltaproteobacteria Bradymonadales nan Microvenator marinus WP 146956596.1 312 aa
BacClassIB delta epsilon subdivisions Deltaproteobacteria Bradymonadales nan Persicimonas sp isolate C5M10 1 iso2 MFP4597675.1 323 aa
BacClassIB Pseudomonadati Myxococcota Bradymonadia nan Bradymonadia bacterium isolate 3300046534 141 iso2 MFT6399856.1 314 aa
BacClassIB Pseudomonadati Myxococcota Bradymonadia nan Bradymonadia bacterium isolate 3300053784 121 iso1 MFT4703838.1 313 aa
BacClassIB Pseudomonadati Myxococcota Polyangia Polyangiales Polyangiaceae bacterium isolate SMAG U384 HEY6729363.1 313 aa
BacClassIB delta epsilon subdivisions Deltaproteobacteria unclassified Deltaproteobacteria Taxonomy ID 122706 nan Deltaproteobacteria bacterium CG2 30 63 29 OIP31979.1 327 aa
BacClassIB delta epsilon subdivisions Deltaproteobacteria Bradymonadales nan Bradymonadales bacterium isolate SO 2017 LW2 140 MFA5624362.1 313 aa
BacClassIB Pseudomonadati Pseudomonadota Gammaproteobacteria Pseudomonadales Pseudomonadales bacterium isolate ALOHA A2.5 102 MCH2334998.1 314 aa
BacClassIB Pseudomonadati Pseudomonadota Gammaproteobacteria Pseudomonadales Pseudomonadales bacterium isolate SMAG U15491 HTK97574.1 314 aa
BacClassIB Pseudomonadati Pseudomonadota Gammaproteobacteria Pseudomonadales Pseudomonadales bacterium isolate SMAG U15184 HTO57815.1 314 aa

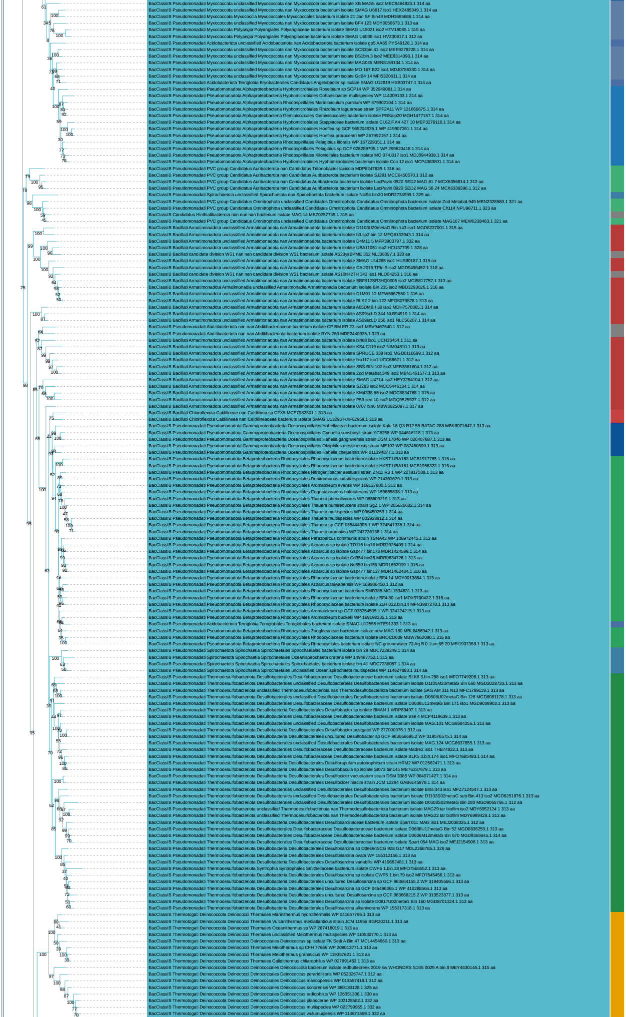
BacClassIB Pseudomonadati Myxococcota unclassified Myxococcota nan Myxococcota bacterium isolate XB MAG5 iso2 MEC9464823.1 314 aa
BacClassIB Pseudomonadati Myxococcota unclassified Myxococcota nan Myxococcota bacterium isolate SMAG U6817 iso1 HEX2485349.1 314 aa
BacClassIB Pseudomonadati Myxococcota Myxococcia Myxococcales Myxococcales bacterium isolate 21 Jan SF Bin49 MDH3685686.1 314 aa
BacClassIB Pseudomonadati Myxococcota unclassified Myxococcota nan Myxococcota bacterium isolate BF4 123 MDY0058673.1 313 aa
BacClassIB Pseudomonadati Myxococcota Polyangia Polyangiales Polyangiaceae bacterium isolate SMAG U15021 iso2 HTV18085.1 315 aa
BacClassIB Pseudomonadati Myxococcota Polyangia Polyangiales Polyangiaceae bacterium isolate SMAG U6038 iso1 HVZ30817.1 312 aa
BacClassIB Pseudomonadati Acidobacteriota unclassified Acidobacteriota nan Acidobacteriota bacterium isolate gp5 AA65 PYS49128.1 314 aa
BacClassIB Pseudomonadati Myxococcota unclassified Myxococcota nan Myxococcota bacterium isolate SCS3bin.41 iso2 MEE9279228.1 314 aa
BacClassIB Pseudomonadati Myxococcota unclassified Myxococcota nan Myxococcota bacterium isolate BS1bin.3 iso2 MEE8314390.1 314 aa
BacClassIB Pseudomonadati Myxococcota unclassified Myxococcota nan Myxococcota bacterium isolate MAG045 MEN8159134.1 314 aa
BacClassIB Pseudomonadati Myxococcota unclassified Myxococcota nan Myxococcota bacterium isolate MO 167.B22 iso1 MDJ0786330.1 314 aa
BacClassIB Pseudomonadati Myxococcota unclassified Myxococcota nan Myxococcota bacterium isolate GcB4 14 MFI5320611.1 314 aa
BacClassIB Pseudomonadati Acidobacteriota Terriglobia Bryobacterales Candidatus Angelobacter sp isolate SMAG U12819 HXB03747.1 314 aa
BacClassIB Pseudomonadati Pseudomonadota Alphaproteobacteria Hyphomicrobiales Roseibium sp SCP14 WP 352949081.1 314 aa
BacClassIB Pseudomonadati Pseudomonadota Alphaproteobacteria Hyphomicrobiales Cohaesibacter multispecies WP 114009133.1 314 aa
BacClassIB Pseudomonadati Pseudomonadota Alphaproteobacteria Rhodospirillales Marinibaculum pumilum WP 379902104.1 314 aa
BacClassIB Pseudomonadati Pseudomonadota Alphaproteobacteria Hyphomicrobiales Rhizobium laguerreae strain SPF2A11 WP 131666675.1 314 aa
BacClassIB Pseudomonadati Pseudomonadota Alphaproteobacteria Geminicoccales Geminicoccales bacterium isolate PBSalp20 MGH1477157.1 314 aa
BacClassIB Pseudomonadati Pseudomonadota Alphaproteobacteria Hyphomicrobiales Stappiaceae bacterium isolate CI.62.F.A4 427 10 MEP3279116.1 314 aa
BacClassIB Pseudomonadati Pseudomonadota Alphaproteobacteria Hyphomicrobiales Hoeflea sp GCF 965204935.1 WP 419907361.1 314 aa
BacClassIB Pseudomonadati Pseudomonadota Alphaproteobacteria Hyphomicrobiales Hoeflea prorocentri WP 267992157.1 314 aa
BacClassIB Pseudomonadati Pseudomonadota Alphaproteobacteria Rhodospirillales Pelagibius litoralis WP 167229351.1 314 aa
BacClassIB Pseudomonadati Pseudomonadota Alphaproteobacteria Rhodospirillales Pelagibius sp GCF 028289705.1 WP 299623418.1 314 aa
BacClassIB Pseudomonadati Pseudomonadota Alphaproteobacteria Rhodospirillales Kiloniellales bacterium isolate MO 074.B17 iso1 MDJ0944938.1 314 aa
BacClassIB Pseudomonadati Pseudomonadota Alphaproteobacteria Hyphomicrobiales Hyphomicrobiales bacterium isolate Cca 12 iso1 MCP4380801.1 314 aa
BacClassIB Pseudomonadati PVC group Candidatus Auribacterota nan Candidatus Tritonobacter lacicola MDP8247839.1 316 aa
BacClassIB Pseudomonadati PVC group Candidatus Auribacterota nan Candidatus Auribacterota bacterium isolate SJ281 MCC6450570.1 312 aa
BacClassIB Pseudomonadati PVC group Candidatus Auribacterota nan Candidatus Auribacterota bacterium isolate LacPavin 0920 SED2 MAG 61 7 MCX6356814.1 312 aa
BacClassIB Pseudomonadati PVC group Candidatus Auribacterota nan Candidatus Auribacterota bacterium isolate LacPavin 0920 SED2 MAG 56 24 MCX6339396.1 312 aa
BacClassIB Pseudomonadati Spirochaetota unclassified Spirochaetota nan Spirochaetota bacterium isolate NI494 bin20 MDR2734999.1 325 aa
BacClassIB Pseudomonadati PVC group Candidatus Omnitrophota unclassified Candidatus Omnitrophota Candidatus Omnitrophota bacterium isolate Zod Metabat.949 MBN2328580.1 321 aa
BacClassIB Pseudomonadati PVC group Candidatus Omnitrophota unclassified Candidatus Omnitrophota Candidatus Omnitrophota bacterium isolate Ch114 NPU98711.1 323 aa
BacClassIB Candidatus Hinthialibacterota nan nan nan bacterium isolate MAG 14 MBZ0257735.1 315 aa
BacClassIB Pseudomonadati PVC group Candidatus Omnitrophota unclassified Candidatus Omnitrophota Candidatus Omnitrophota bacterium isolate MAG167 MEW6238483.1 321 aa
BacClassIB Bacillati Armatimonadota unclassified Armatimonadota nan Armatimonadota bacterium isolate D1103U20metaG Bin 143 iso1 MGD8237001.1 315 aa
BacClassIB Bacillati Armatimonadota unclassified Armatimonadota nan Armatimonadota bacterium isolate b3.sp2 bin.12 MFQ6133943.1 314 aa
BacClassIB Bacillati Armatimonadota unclassified Armatimonadota nan Armatimonadota bacterium isolate D4M11 5 MFP3903797.1 332 aa
BacClassIB Bacillati Armatimonadota unclassified Armatimonadota nan Armatimonadota bacterium isolate UBA11051 iso2 HCU37709.1 328 aa
BacClassIB Bacillati candidate division WS1 nan nan candidate division WS1 bacterium isolate AS23ysBPME 352 NLJ36057.1 320 aa
BacClassIB Bacillati Armatimonadota unclassified Armatimonadota nan Armatimonadota bacterium isolate SMAG U14285 iso1 HUS80187.1 315 aa
BacClassIB Bacillati Armatimonadota unclassified Armatimonadota nan Armatimonadota bacterium isolate CA 2019 TPIn 9 iso2 MGD9498452.1 318 aa
BacClassIB Bacillati candidate division WS1 nan nan candidate division WS1 bacterium isolate AS10tlH2TH 342 iso1 NLO04253.1 316 aa
BacClassIB Bacillati Armatimonadota unclassified Armatimonadota nan Armatimonadota bacterium isolate SBF912SR3HQ0005 iso2 MGI5817757.1 313 aa
BacClassIB Bacillati Armatimonadota Armatimonadia unclassified Armatimonadia Armatimonadia bacterium isolate Bin 235 iso2 MBD3293026.1 316 aa
BacClassIB Bacillati Armatimonadota unclassified Armatimonadota nan Armatimonadota bacterium isolate D1M01 12 MFW5867550.1 316 aa
BacClassIB Bacillati Armatimonadota unclassified Armatimonadota nan Armatimonadota bacterium isolate BLK2 2.bin.122 MFO8079828.1 313 aa
BacClassIB Bacillati Armatimonadota unclassified Armatimonadota nan Armatimonadota bacterium isolate A05DMB I 38 iso2 MDH7570865.1 314 aa
BacClassIB Bacillati Armatimonadota unclassified Armatimonadota nan Armatimonadota bacterium isolate AS09scLD 344 NLB94919.1 314 aa
BacClassIB Bacillati Armatimonadota unclassified Armatimonadota nan Armatimonadota bacterium isolate AS09scLD 256 iso1 NLC56207.1 314 aa
BacClassIB Pseudomonadati Abditibacteriota nan nan Abditibacteriaceae bacterium isolate CP BM ER 23 iso1 MBV9467640.1 312 aa
BacClassIB Pseudomonadati Abditibacteriota nan nan Abditibacteriota bacterium isolate RYN 269 MDF2440935.1 323 aa
BacClassIB Bacillati Armatimonadota unclassified Armatimonadota nan Armatimonadota bacterium isolate bin88 iso1 UCH33454.1 311 aa
BacClassIB Bacillati Armatimonadota unclassified Armatimonadota nan Armatimonadota bacterium isolate KS4 C119 iso2 NIM04810.1 313 aa
BacClassIB Bacillati Armatimonadota unclassified Armatimonadota nan Armatimonadota bacterium isolate SPRUCE 339 iso2 MGD0110699.1 312 aa
BacClassIB Bacillati Armatimonadota unclassified Armatimonadota nan Armatimonadota bacterium isolate bin117 iso1 UCC68621.1 312 aa
BacClassIB Bacillati Armatimonadota unclassified Armatimonadota nan Armatimonadota bacterium isolate SBS.BIN.102 iso3 MFB3881804.1 312 aa
BacClassIB Bacillati Armatimonadota unclassified Armatimonadota nan Armatimonadota bacterium isolate Zod Metabat.349 iso2 MBN1461577.1 313 aa
BacClassIB Bacillati Armatimonadota unclassified Armatimonadota nan Armatimonadota bacterium isolate SMAG U4714 iso2 HEY3284104.1 312 aa
BacClassIB Bacillati Armatimonadota unclassified Armatimonadota nan Armatimonadota bacterium isolate SJ283 iso2 MCC6446134.1 314 aa
BacClassIB Bacillati Armatimonadota unclassified Armatimonadota nan Armatimonadota bacterium isolate KM4338 66 iso2 MGC8834768.1 315 aa
BacClassIB Bacillati Armatimonadota unclassified Armatimonadota nan Armatimonadota bacterium isolate P53 sed 10 iso2 MGQ9525927.1 312 aa
BacClassIB Bacillati Armatimonadota unclassified Armatimonadota nan Armatimonadota bacterium isolate 0707 bin6 MBW3625097.1 317 aa
BacClassIB Bacillati Chloroflexota Caldilineae nan Caldilinea sp CFX5 MCE7982801.1 313 aa
BacClassIB Bacillati Chloroflexota Caldilineae nan Caldilineaceae bacterium isolate SMAG U13295 HXF62959.1 313 aa
BacClassIB Pseudomonadati Pseudomonadota Gammaproteobacteria Oceanospirillales Hahellaceae bacterium isolate Kalu 18 Q3 R12 55 BATAC.288 MBK8971647.1 313 aa
BacClassIB Pseudomonadati Pseudomonadota Gammaproteobacteria Oceanospirillales Gynuella sunshinyii strain YC6258 WP 044616118.1 313 aa
BacClassIB Pseudomonadati Pseudomonadota Gammaproteobacteria Oceanospirillales Hahella ganghwensis strain DSM 17046 WP 020407887.1 313 aa
BacClassIB Pseudomonadati Pseudomonadota Gammaproteobacteria Oceanospirillales Oleiphilus messinensis strain ME102 WP 087460590.1 313 aa
BacClassIB Pseudomonadati Pseudomonadota Gammaproteobacteria Oceanospirillales Hahella chejuensis WP 011394877.1 313 aa
BacClassIB Pseudomonadati Pseudomonadota Betaproteobacteria Rhodocyclales Rhodocyclaceae bacterium isolate HKST UBA163 MCB1917765.1 315 aa
BacClassIB Pseudomonadati Pseudomonadota Betaproteobacteria Rhodocyclales Rhodocyclaceae bacterium isolate HKST UBA161 MCB1956323.1 315 aa
BacClassIB Pseudomonadati Pseudomonadota Betaproteobacteria Rhodocyclales Nitrogeniibacter aestuarii strain ZN11 R3 1 WP 227817508.1 313 aa
BacClassIB Pseudomonadati Pseudomonadota Betaproteobacteria Rhodocyclales Denitromonas iodatirespirans WP 214363629.1 313 aa
BacClassIB Pseudomonadati Pseudomonadota Betaproteobacteria Rhodocyclales Aromatoleum evansii WP 169127800.1 313 aa
BacClassIB Pseudomonadati Pseudomonadota Betaproteobacteria Rhodocyclales Cognatazoarcus halotolerans WP 159685838.1 313 aa
BacClassIB Pseudomonadati Pseudomonadota Betaproteobacteria Rhodocyclales Thauera phenolivorans WP 068809219.1 313 aa
BacClassIB Pseudomonadati Pseudomonadota Betaproteobacteria Rhodocyclales Thauera humireducens strain SgZ 1 WP 205626602.1 314 aa
BacClassIB Pseudomonadati Pseudomonadota Betaproteobacteria Rhodocyclales Thauera multispecies WP 096450253.1 314 aa
BacClassIB Pseudomonadati Pseudomonadota Betaproteobacteria Rhodocyclales Thauera multispecies WP 002928812.1 314 aa
BacClassIB Pseudomonadati Pseudomonadota Betaproteobacteria Rhodocyclales Thauera sp GCF 035444905.1 WP 324541336.1 314 aa
BacClassIB Pseudomonadati Pseudomonadota Betaproteobacteria Rhodocyclales Thauera aromatica WP 247736138.1 314 aa
BacClassIB Pseudomonadati Pseudomonadota Betaproteobacteria Rhodocyclales Parazoarcus communis strain TSNA42 WP 108972445.1 313 aa
BacClassIB Pseudomonadati Pseudomonadota Betaproteobacteria Rhodocyclales Azoarcus sp isolate TD116 bin18 MDR2926409.1 314 aa
BacClassIB Pseudomonadati Pseudomonadota Betaproteobacteria Rhodocyclales Azoarcus sp isolate Gsp477 bin173 MDR1424599.1 314 aa
BacClassIB Pseudomonadati Pseudomonadota Betaproteobacteria Rhodocyclales Azoarcus sp isolate Cd354 bin26 MDR0634726.1 313 aa
BacClassIB Pseudomonadati Pseudomonadota Betaproteobacteria Rhodocyclales Azoarcus sp isolate Nc350 bin159 MDR1662009.1 316 aa
BacClassIB Pseudomonadati Pseudomonadota Betaproteobacteria Rhodocyclales Azoarcus sp isolate Gsp477 bin127 MDR1462484.1 316 aa
BacClassIB Pseudomonadati Pseudomonadota Betaproteobacteria Rhodocyclales Rhodocyclaceae bacterium isolate BF4 14 MDY0013654.1 313 aa
BacClassIB Pseudomonadati Pseudomonadota Betaproteobacteria Rhodocyclales Azoarcus taiwanensis WP 168986450.1 312 aa
BacClassIB Pseudomonadati Pseudomonadota Betaproteobacteria Rhodocyclales Rhodocyclaceae bacterium SMB388 MGL1834831.1 313 aa
BacClassIB Pseudomonadati Pseudomonadota Betaproteobacteria Rhodocyclales Rhodocyclaceae bacterium isolate BF4 80 iso1 MDX9700422.1 316 aa
BacClassIB Pseudomonadati Pseudomonadota Betaproteobacteria Rhodocyclales Rhodocyclaceae bacterium isolate 21H 022.bin.14 MFN3987270.1 313 aa
BacClassIB Pseudomonadati Pseudomonadota Betaproteobacteria Rhodocyclales Aromatoleum sp GCF 035254505.1 WP 324124215.1 313 aa
BacClassIB Pseudomonadati Pseudomonadota Betaproteobacteria Rhodocyclales Aromatoleum buckelii WP 169198235.1 313 aa
BacClassIB Pseudomonadati Acidobacteriota Terriglobia Terriglobales Terriglobales bacterium isolate SMAG U12555 HTE91333.1 313 aa
BacClassIB Pseudomonadati Pseudomonadota Betaproteobacteria Rhodocyclales Zoogloeaceae bacterium isolate new MAG 180 MBL8456942.1 313 aa
BacClassIB Pseudomonadati Pseudomonadota Betaproteobacteria Rhodocyclales Rhodocyclaceae bacterium isolate BROCD009 MBW7862090.1 316 aa
BacClassIB Pseudomonadati Pseudomonadota Betaproteobacteria Rhodocyclales Rhodocyclales bacterium isolate NC groundwater 73 Ag B 0.1um 65 20 MBI1907358.1 313 aa
BacClassIB Pseudomonadati Spirochaetota Spirochaetia Spirochaetales Spirochaetales bacterium isolate bin 29 MDC7239249.1 314 aa
BacClassIB Pseudomonadati Spirochaetota Spirochaetia Spirochaetales Oceanispirochaeta crateris WP 149487752.1 313 aa
BacClassIB Pseudomonadati Spirochaetota Spirochaetia Spirochaetales Spirochaetales bacterium isolate bin 41 MDC7236067.1 314 aa
BacClassIB Pseudomonadati Spirochaetota Spirochaetia Spirochaetales unclassified Oceanispirochaeta multispecies WP 114627893.1 314 aa
BacClassIB Pseudomonadati Thermodesulfobacteriota Desulfobacterales Desulfobacteraceae Desulfobacteraceae bacterium isolate BLK6 3.bin.268 iso1 MFO7749206.1 313 aa
BacClassIB Pseudomonadati Thermodesulfobacteriota Desulfobacterales unclassified Desulfobacterales Desulfobacterales bacterium isolate D1105M20metaG Bin 660 MGD2028733.1 313 aa
BacClassIB Pseudomonadati Thermodesulfobacteriota unclassified Thermodesulfobacteriota nan Thermodesulfobacteriota bacterium isolate SAG AM 311 N13 MFC1789119.1 313 aa
BacClassIB Pseudomonadati Thermodesulfobacteriota Desulfobacterales unclassified Desulfobacterales Desulfobacterales bacterium isolate D0608U02metaG Bin 126 MGD8981178.1 313 aa
BacClassIB Pseudomonadati Thermodesulfobacteriota Desulfobacterales Desulfobacteraceae Desulfobacteraceae bacterium isolate D0608U12metaG Bin 171 iso1 MGD9009903.1 313 aa
BacClassIB Pseudomonadati Thermodesulfobacteriota Desulfobacteria Desulfobacterales Desulfobacter sp isolate BMAN 1 WDP89497.1 313 aa
BacClassIB Pseudomonadati Thermodesulfobacteriota Desulfobacterales Desulfobacteraceae Desulfobacteraceae bacterium isolate Bse 4 MCP4119639.1 313 aa
BacClassIB Pseudomonadati Thermodesulfobacteriota Desulfobacterales unclassified Desulfobacterales Desulfobacterales bacterium isolate MAG.101 MCG8684256.1 313 aa
BacClassIB Pseudomonadati Thermodesulfobacteriota Desulfobacteria Desulfobacterales Desulfobacter postgatei WP 277000976.1 312 aa
BacClassIB Pseudomonadati Thermodesulfobacteriota Desulfobacteria Desulfobacterales uncultured Desulfobacter sp GCF 963666695.2 WP 319576575.1 314 aa
BacClassIB Pseudomonadati Thermodesulfobacteriota Desulfobacterales unclassified Desulfobacterales Desulfobacterales bacterium isolate MAG.124 MCG8637855.1 313 aa
BacClassIB Pseudomonadati Thermodesulfobacteriota Desulfobacterales Desulfobacteraceae Desulfobacteraceae bacterium isolate Madre2 iso1 THB74832.1 313 aa
BacClassIB Pseudomonadati Thermodesulfobacteriota Desulfobacterales Desulfobacteraceae Desulfobacteraceae bacterium isolate BLK5 3.bin.174 iso1 MFO7885493.1 314 aa
BacClassIB Pseudomonadati Thermodesulfobacteriota Desulfobacteria Desulfobacterales Desulforapulum autotrophicum strain HRM2 WP 012662471.1 313 aa
BacClassIB Pseudomonadati Thermodesulfobacteriota Desulfobacteria Desulfobacterales Desulfobacula sp isolate SI073 bin145 MBT6337679.1 313 aa
BacClassIB Pseudomonadati Thermodesulfobacteriota Desulfobacteria Desulfobacterales Desulfocicer vacuolatum strain DSM 3385 WP 084071427.1 314 aa
BacClassIB Pseudomonadati Thermodesulfobacteriota Desulfobacteria Desulfobacterales Desulfocicer niacini strain JCM 12294 GAB6145979.1 314 aa
BacClassIB Pseudomonadati Thermodesulfobacteriota Desulfobacterales unclassified Desulfobacterales Desulfobacterales bacterium isolate Bins.043 iso1 MFZ7124547.1 313 aa
BacClassIB Pseudomonadati Thermodesulfobacteriota Desulfobacterales unclassified Desulfobacterales Desulfobacterales bacterium isolate D1103S02metaG sub Bin 413 iso2 MGD8251876.1 313 aa
BacClassIB Pseudomonadati Thermodesulfobacteriota Desulfobacterales unclassified Desulfobacterales Desulfobacterales bacterium isolate D0608S02metaG Bin 280 MGD9065756.1 312 aa
BacClassIB Pseudomonadati Thermodesulfobacteriota unclassified Thermodesulfobacteriota nan Thermodesulfobacteriota bacterium isolate MAG29 tar biofilm iso2 MDY6952124.1 313 aa
BacClassIB Pseudomonadati Thermodesulfobacteriota unclassified Thermodesulfobacteriota nan Thermodesulfobacteriota bacterium isolate MAG22 tar biofilm MDY6989428.1 313 aa
BacClassIB Pseudomonadati Thermodesulfobacteriota Desulfobacteria Desulfobacterales Desulfosarcinaceae bacterium isolate Spart 011 MAG iso1 MEJ2038335.1 312 aa
BacClassIB Pseudomonadati Thermodesulfobacteriota Desulfobacterales Desulfobacteraceae Desulfobacteraceae bacterium isolate D0608U12metaG Bin 52 MGD8836250.1 313 aa
BacClassIB Pseudomonadati Thermodesulfobacteriota Desulfobacterales Desulfobacteraceae Desulfobacteraceae bacterium isolate D0606M12metaG Bin 670 MGD9365645.1 314 aa
BacClassIB Pseudomonadati Thermodesulfobacteriota Desulfobacterales Desulfobacteraceae Desulfobacteraceae bacterium isolate Spart 054 MAG iso2 MEJ2154906.1 313 aa
BacClassIB Pseudomonadati Thermodesulfobacteriota Desulfobacteria Desulfobacterales Desulfosarcina sp OttesenSCG 928 G17 MDL2268785.1 328 aa
BacClassIB Pseudomonadati Thermodesulfobacteriota Desulfobacteria Desulfobacterales Desulfosarcina ovata WP 155312156.1 313 aa
BacClassIB Pseudomonadati Thermodesulfobacteriota Desulfobacteria Desulfobacterales Desulfosarcina variabilis WP 419662481.1 313 aa
BacClassIB Pseudomonadati Thermodesulfobacteriota Syntrophia Syntrophales Smithellaceae bacterium isolate CWP6 1.bin.28 MFO7568552.1 313 aa
BacClassIB Pseudomonadati Thermodesulfobacteriota Desulfobacteria Desulfobacterales Desulfosarcina sp isolate CWP5 1.bin.79 iso2 MFO7645456.1 313 aa
BacClassIB Pseudomonadati Thermodesulfobacteriota Desulfobacteria Desulfobacterales uncultured Desulfosarcina sp GCF 963664155.2 WP 319405566.1 313 aa
BacClassIB Pseudomonadati Thermodesulfobacteriota Desulfobacteria Desulfobacterales Desulfosarcina sp GCF 046496365.1 WP 410288566.1 313 aa
BacClassIB Pseudomonadati Thermodesulfobacteriota Desulfobacteria Desulfobacterales uncultured Desulfosarcina sp GCF 963668215.2 WP 319523377.1 313 aa
BacClassIB Pseudomonadati Thermodesulfobacteriota Desulfobacteria Desulfobacterales Desulfosarcina sp isolate D0817U02metaG Bin 160 MGD8701324.1 313 aa
BacClassIB Pseudomonadati Thermodesulfobacteriota Desulfobacteria Desulfobacterales Desulfosarcina alkanivorans WP 155317318.1 313 aa
BacClassIB Thermotogati Deinococcota Deinococci Thermales Marinithermus hydrothermalis WP 041657796.1 313 aa
BacClassIB Thermotogati Deinococcota Deinococci Thermales Vulcanithermus mediatlanticus strain JCM 11956 BGR20211.1 313 aa
BacClassIB Thermotogati Deinococcota Deinococci Thermales Oceanithermus sp WP 287418019.1 313 aa
BacClassIB Thermotogati Deinococcota Deinococci Thermales unclassified Meiothermus multispecies WP 110530770.1 313 aa
BacClassIB Thermotogati Deinococcota Deinococci Deinococcales Deinococcus sp isolate FK Sedi A Bin.47 MCL4454660.1 313 aa
BacClassIB Thermotogati Deinococcota Deinococci Thermales Meiothermus sp CFH 77666 WP 208013771.1 313 aa
BacClassIB Thermotogati Deinococcota Deinococci Thermales Meiothermus granaticius WP 119357621.1 313 aa
BacClassIB Thermotogati Deinococcota Deinococci Thermales Calidithermus chliarophilus WP 027891463.1 313 aa
BacClassIB Thermotogati Deinococcota Deinococci Deinococcales Deinococcota bacterium isolate redbuttecreek 2019 sw WHONDRS S19S 0029 A bin.8 MEY4530146.1 315 aa
BacClassIB Thermotogati Deinococcota Deinococci Deinococcales Deinococcus peraridilitoris WP 052326747.1 312 aa
BacClassIB Thermotogati Deinococcota Deinococci Deinococcales Deinococcus maricopensis WP 013557418.1 312 aa
BacClassIB Thermotogati Deinococcota Deinococci Deinococcales Deinococcus sonorensis WP 380130128.1 325 aa
BacClassIB Thermotogati Deinococcota Deinococci Deinococcales Deinococcus radiophilus WP 126351306.1 330 aa
BacClassIB Thermotogati Deinococcota Deinococci Deinococcales Deinococcus planocerae WP 102128582.1 332 aa
BacClassIB Thermotogati Deinococcota Deinococci Deinococcales Deinococcus multispecies WP 022799955.1 332 aa
BacClassIB Thermotogati Deinococcota Deinococci Deinococcales Deinococcus wulumuqiensis WP 114671559.1 332 aa

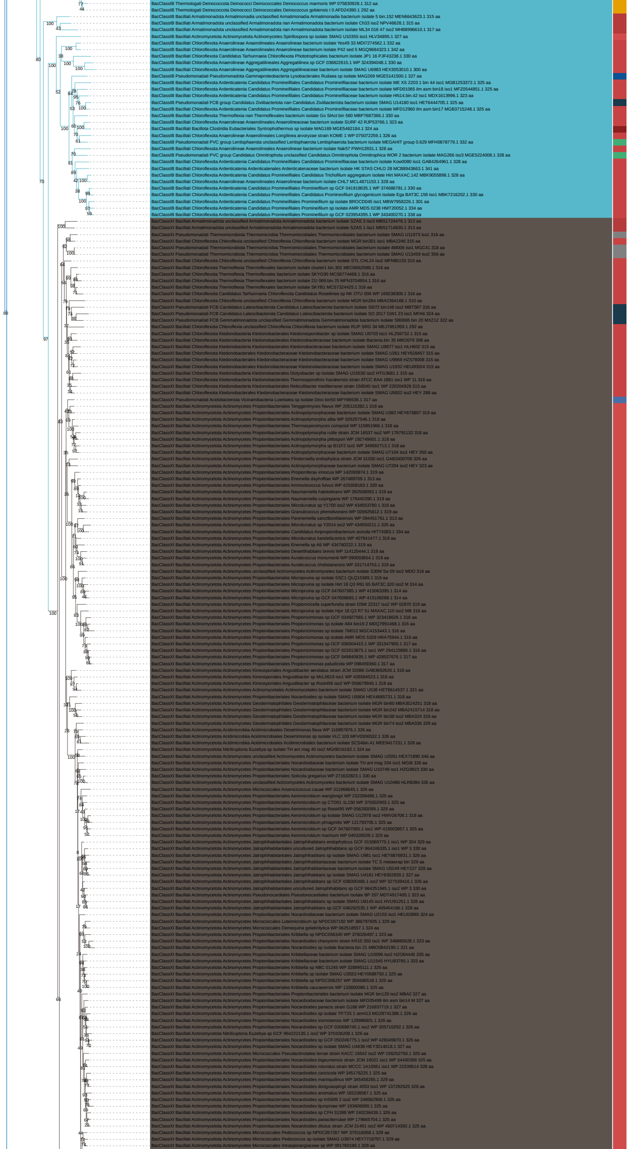
BacClassIB Thermotogati Deinococcota Deinococci Deinococcales Deinococcus marmoris WP 075830928.1 312 aa
BacClassIB Thermotogati Deinococcota Deinococci Deinococcales Deinococcus gobiensis I 0 AFD24390.1 292 aa
BacClassIB Bacillati Armatimonadota Armatimonadia unclassified Armatimonadia Armatimonadia bacterium isolate 5 bin.152 MEN6643623.1 315 aa
BacClassIB Bacillati Armatimonadota unclassified Armatimonadota nan Armatimonadota bacterium isolate Ch33 iso2 NPV46628.1 315 aa
BacClassIB Bacillati Armatimonadota unclassified Armatimonadota nan Armatimonadota bacterium isolate ML34 016 47 iso2 MHB8996610.1 317 aa
BacClassIB Bacillati Actinomycetota Actinomycetota Actinomycetes Spirillospora sp isolate SMAG U10355 iso1 HLV34895.1 327 aa
BacClassIB Bacillati Chloroflexota Anaerolineae Anaerolineales Anaerolineae bacterium isolate Yes49 33 MDI7274562.1 332 aa
BacClassIB Bacillati Chloroflexota Anaerolineae Anaerolineales Anaerolineae bacterium isolate P42 sed 5 MGQ9684323.1 342 aa
BacClassIB Bacillati Chloroflexota Candidatus Tarhunnaeia Chloroflexota Phototrophicales bacterium isolate JP1 16 PJF43238.1 330 aa
BacClassIB Bacillati Chloroflexota Anaerolineae Aggregatilineales Aggregatilinea sp GCF 036822615.1 WP 324394048.1 330 aa
BacClassIB Bacillati Chloroflexota Anaerolineae Aggregatilineales Aggregatilineaceae bacterium isolate SMAG U6983 HEX3053010.1 300 aa
BacClassIB Pseudomonadati Pseudomonadota Gammaproteobacteria Lysobacterales Rudaea sp isolate MAG269 MGE5141500.1 327 aa
BacClassIB Bacillati Chloroflexota Ardenticatenia Candidatus Promineifilales Candidatus Promineifilaceae bacterium isolate ME XS 2203 1 bin 44 iso1 MGB1253372.1 325 aa
BacClassIB Bacillati Chloroflexota Ardenticatenia Candidatus Promineifilales Candidatus Promineifilaceae bacterium isolate MFD01065 ilm asm bin18 iso1 MFZ0544851.1 325 aa
BacClassIB Bacillati Chloroflexota Ardenticatenia Candidatus Promineifilales Candidatus Promineifilaceae bacterium isolate HN14.bin.42 iso1 MDX1613996.1 323 aa
BacClassIB Pseudomonadati FCB group Candidatus Zixiibacteriota nan Candidatus Zixiibacteriota bacterium isolate SMAG U14180 iso1 HET6444705.1 325 aa
BacClassIB Bacillati Chloroflexota Ardenticatenia Candidatus Promineifilales Candidatus Promineifilaceae bacterium isolate MFD12960 ilm asm bin17 MGB3715248.1 325 aa
BacClassIB Bacillati Chloroflexota Thermoflexia nan Thermoflexales bacterium isolate Go SIAct bin 560 MBP7687366.1 330 aa
BacClassIB Bacillati Chloroflexota Anaerolineae Anaerolineales Anaerolineaceae bacterium isolate SURF 42 RJP53766.1 323 aa
BacClassIB Bacillati Bacillota Clostridia Eubacteriales Syntrophothermus sp isolate MAG169 MGE5462184.1 324 aa
BacClassIB Bacillati Chloroflexota Anaerolineae Anaerolineales Longilinea arvoryzae strain KOME 1 WP 075072259.1 326 aa
BacClassIB Pseudomonadati PVC group Lentisphaerota unclassified Lentisphaerota Lentisphaerota bacterium isolate MEGAHIT group 0.629 MFH0878779.1 332 aa
BacClassIB Bacillati Chloroflexota Anaerolineae Anaerolineales Anaerolineae bacterium isolate Nak57 PWH13931.1 328 aa
BacClassIB Pseudomonadati PVC group Candidatus Omnitrophota unclassified Candidatus Omnitrophota Omnitrophica WOR 2 bacterium isolate MAG266 iso3 MGE5224008.1 328 aa
BacClassIB Bacillati Chloroflexota Ardenticatenia Candidatus Promineifilales Candidatus Promineifilaceae bacterium isolate Kow0080 iso1 GAB4264961.1 328 aa
BacClassIB Bacillati Chloroflexota Ardenticatenia Ardenticatenales Ardenticatenaceae bacterium isolate HK STAS CHLO 28 MCB8943663.1 341 aa
BacClassIB Bacillati Chloroflexota Ardenticatenia Candidatus Promineifilales Candidatus Trichofilum aggregatum isolate Hirt MAXAC.142 MBK9055898.1 328 aa
BacClassIB Bacillati Chloroflexota Anaerolineae Anaerolineales Anaerolineae bacterium isolate CHL7 MCL4871153.1 328 aa
BacClassIB Bacillati Chloroflexota Ardenticatenia Candidatus Promineifilales Promineifilum sp GCF 041918635.1 WP 374686781.1 330 aa
BacClassIB Bacillati Chloroflexota Ardenticatenia Candidatus Promineifilales Candidatus Promineofilum glycogenicum isolate Ega BAT3C.159 iso1 MBK7216202.1 330 aa
BacClassIB Bacillati Chloroflexota Ardenticatenia Candidatus Promineifilales Promineifilum sp isolate BROCD045 iso1 MBW7958226.1 301 aa
BacClassIB Bacillati Chloroflexota Ardenticatenia Candidatus Promineifilales Promineifilum sp isolate AMR MDS 0238 HMT20052.1 334 aa
BacClassIB Bacillati Chloroflexota Ardenticatenia Candidatus Promineifilales Promineifilum sp GCF 023954395.1 WP 343400270.1 338 aa
BacClassXI Bacillati Armatimonadota unclassified Armatimonadota Armatimonadota bacterium isolate SZAS 3 iso3 MBS1724476.1 313 aa
BacClassXI Bacillati Armatimonadota unclassified Armatimonadota Armatimonadota bacterium isolate SZAS 1 iso1 MBS1714830.1 313 aa
BacClassXI Pseudomonadati Thermomicrobiota Thermomicrobia Thermomicrobiales Thermomicrobiales bacterium isolate SMAG U11973 iso1 316 aa
BacClassXI Bacillati Chloroflexota Chloroflexia unclassified Chloroflexia Chloroflexia bacterium isolate MGR bin301 iso1 MBA2246 315 aa
BacClassXI Pseudomonadati Thermomicrobiota Thermomicrobia Thermomicrobiales Thermomicrobiales bacterium isolate 4M006 iso1 MGC41 318 aa
BacClassXI Pseudomonadati Thermomicrobiota Thermomicrobia Thermomicrobiales Thermomicrobiales bacterium isolate SMAG U13459 iso2 356 aa
BacClassXI Bacillati Chloroflexota Chloroflexia unclassified Chloroflexia Chloroflexia bacterium isolate STL CHL24 iso2 MFN85153 315 aa
BacClassXI Bacillati Chloroflexota Thermoflexia Thermoflexales bacterium isolate cluster1 bin.301 MEO6062588.1 316 aa
BacClassXI Bacillati Chloroflexota Thermoflexia Thermoflexales bacterium isolate SKYG95 MCS6774468.1 316 aa
BacClassXI Bacillati Chloroflexota Thermoflexia Thermoflexales bacterium isolate 21l 069.bin.70 MFN3704954.1 316 aa
BacClassXI Bacillati Chloroflexota Thermoflexia Thermoflexales bacterium isolate SKYB1 MCS7324425.1 316 aa
BacClassXI Bacillati Chloroflexota Candidatus Tarhunnaeia Chloroflexota Candidatus Roseilinea sp NK OTU 006 WP 169238308.1 316 aa
BacClassXI Bacillati Chloroflexota Chloroflexia unclassified Chloroflexia Chloroflexia bacterium isolate MGR bin264 MBA2364168.1 310 aa
BacClassXI Pseudomonadati FCB Candidatus Latescibacterota Candidatus Latescibacterota bacterium isolate SI072 bin148 iso2 MBT587 316 aa
BacClassXI Pseudomonadati FCB Candidatus Latescibacterota Candidatus Latescibacterota bacterium isolate SO 2017 GW1 23 iso1 MFA6 324 aa
BacClassXI Pseudomonadati FCB Gemmatimonadota unclassified Gemmatimonadota Gemmatimonadota bacterium isolate SB0665 bin 20 MXZ12 322 aa
BacClassXI Bacillati Chloroflexota Chloroflexia unclassified Chloroflexia Chloroflexia bacterium isolate RUP SRG 34 MEJ7651959.1 292 aa
BacClassXI Bacillati Chloroflexota Ktedonobacteria Ktedonobacterales Ktedonosporobacter sp isolate SMAG U9703 iso1 HLZ56732.1 315 aa
BacClassXI Bacillati Chloroflexota Ktedonobacteria Ktedonobacterales Ktedonobacteraceae bacterium isolate Bacteria.bin 35 MBO079 308 aa
BacClassXI Bacillati Chloroflexota Ktedonobacteria Ktedonobacterales Ktedonobacteraceae bacterium isolate SMAG U9877 iso1 HLH602 315 aa
BacClassXI Bacillati Chloroflexota Ktedonobacterales Ktedonobacteraceae Ktedonobacteraceae bacterium isolate SMAG U261 HEY628457 315 aa
BacClassXI Bacillati Chloroflexota Ktedonobacterales Ktedonobacteraceae Ktedonobacteraceae bacterium isolate SMAG U9968 HZS78008 315 aa
BacClassXI Bacillati Chloroflexota Ktedonobacterales Ktedonobacteraceae Ktedonobacteraceae bacterium isolate SMAG U1932 HEU00024 315 aa
BacClassXI Bacillati Chloroflexota Ktedonobacteria Ktedonobacterales Dictyobacter sp isolate SMAG U15530 iso2 HTI13681.1 315 aa
BacClassXI Bacillati Chloroflexota Ktedonobacteria Ktedonobacterales Thermosporothrix hazakensis strain ATCC BAA 1881 iso1 WP 11 316 aa
BacClassXI Bacillati Chloroflexota Ktedonobacteria Ktedonobacterales Reticulibacter mediterranei strain 150040 iso1 WP 220204926 315 aa
BacClassXI Bacillati Chloroflexota Ktedonobacterales Ktedonobacteraceae Ktedonobacteraceae bacterium isolate SMAG U5002 iso2 HEY 288 aa
BacClassXI Pseudomonadati Acidobacteriota Vicinamibacteria Luteitalea sp isolate Dino bin50 MPY86536.1 317 aa
BacClassXI Bacillati Actinomycetota Actinomycetes Tenggerimyces flavus WP 205115382.1 318 aa
BacClassXI Bacillati Actinomycetota Actinomycetes Propionibacteriales Actinopolymorphaceae bacterium isolate SMAG U382 HEY673807 319 aa
BacClassXI Bacillati Actinomycetota Actinomycetes Propionibacteriales Actinopolymorpha alba WP 026257546.1 318 aa
BacClassXI Bacillati Actinomycetota Actinomycetes Propionibacteriales Thermasporomyces composti WP 115851966.1 318 aa
BacClassXI Bacillati Actinomycetota Actinomycetes Propionibacteriales Actinopolymorpha rutile strain JCM 16537 iso2 WP 179791132 318 aa
BacClassXI Bacillati Actinomycetota Actinomycetes Propionibacteriales Actinopolymorpha pittospori WP 192749601.1 318 aa
BacClassXI Bacillati Actinomycetota Actinomycetes Propionibacteriales Actinopolymorpha sp B11F2 iso1 WP 349692713.1 318 aa
BacClassXI Bacillati Actinomycetota Actinomycetes Propionibacteriales Actinopolymorphaceae bacterium isolate SMAG U7104 iso1 HEY 350 aa
BacClassXI Bacillati Actinomycetota Actinomycetes Propionibacteriales Flindersiella endophytica strain JCM 31030 iso1 GAB3400709 326 aa
BacClassXI Bacillati Actinomycetota Actinomycetes Propionibacteriales Actinopolymorphaceae bacterium isolate SMAG U7204 iso2 HEY 323 aa
BacClassXI Bacillati Actinomycetota Actinomycetes Propionibacteriales Propioniferax innocua WP 142093874.1 319 aa
BacClassXI Bacillati Actinomycetota Actinomycetes Propionibacteriales Enemella dayhoffiae WP 267489709.1 313 aa
BacClassXI Bacillati Actinomycetota Actinomycetes Propionibacteriales Ammonicoccus fulvus WP 425308183.1 320 aa
BacClassXI Bacillati Actinomycetota Actinomycetes Propionibacteriales Naumannella halotolerans WP 392508093.1 319 aa
BacClassXI Bacillati Actinomycetota Actinomycetes Propionibacteriales Naumannella cuiyingiana WP 179445390.1 319 aa
BacClassXI Bacillati Actinomycetota Actinomycetes Propionibacteriales Microlunatus sp Y1700 iso2 WP 434553780.1 319 aa
BacClassXI Bacillati Actinomycetota Actinomycetes Propionibacteriales Granulicoccus phenolivorans WP 026925812.1 319 aa
BacClassXI Bacillati Actinomycetota Actinomycetes Propionibacteriales Parenemella sanctibonifatiensis WP 094451761.1 313 aa
BacClassXI Bacillati Actinomycetota Actinomycetes Propionibacteriales Microlunatus sp Y2014 iso2 WP 434550211.1 325 aa
BacClassXI Bacillati Actinomycetota Actinomycetes Propionibacteriales Candidatus Avipropionibacterium avicola HIT74383.1 334 aa
BacClassXI Bacillati Actinomycetota Actinomycetes Propionibacteriales Microlunatus kandeliicorticis WP 407941477.1 319 aa
BacClassXI Bacillati Actinomycetota Actinomycetes Propionibacteriales Enemella sp A6 WP 434760222.1 319 aa
BacClassXI Bacillati Actinomycetota Actinomycetes Propionibacteriales Desertihabitans brevis WP 114125444.1 318 aa
BacClassXI Bacillati Actinomycetota Actinomycetes Propionibacteriales Auraticoccus monumenti WP 090593654.1 319 aa
BacClassXI Bacillati Actinomycetota Actinomycetes Propionibacteriales Auraticoccus cholistanensis WP 331714753.1 319 aa
BacClassXI Bacillati Actinomycetota Actinomycetes unclassified Actinomycetes Actinomycetes bacterium isolate S30M Sa 59 iso2 MDO 316 aa
BacClassXI Bacillati Actinomycetota Actinomycetes Propionibacteriales Micropruina sp isolate SSC1 QLQ15389.1 319 aa
BacClassXI Bacillati Actinomycetota Actinomycetes Propionibacteriales Micropruina sp isolate Hirt 18 Q3 R61 65 BAT3C.320 iso2 M 314 aa
BacClassXI Bacillati Actinomycetota Actinomycetes Propionibacteriales Micropruina sp GCF 047607385.1 WP 415083395.1 314 aa
BacClassXI Bacillati Actinomycetota Actinomycetes Propionibacteriales Micropruina sp GCF 047606665.1 WP 415108288.1 314 aa
BacClassXI Bacillati Actinomycetota Actinomycetes Propionibacteriales Propionicicella superfundia strain DSM 22317 iso2 WP 02870 319 aa
BacClassXI Bacillati Actinomycetota Actinomycetes Propionibacteriales Micropruina sp isolate Hjor 18 Q3 R7 51 MAXAC.115 iso2 MB 316 aa
BacClassXI Bacillati Actinomycetota Actinomycetes Propionibacteriales Propionicimonas sp GCF 034927565.1 WP 323418626.1 316 aa
BacClassXI Bacillati Actinomycetota Actinomycetes Propionibacteriales Propionicimonas sp isolate A84 bin19 2 MDQ7991468.1 316 aa
BacClassXI Bacillati Actinomycetota Actinomycetes Propionibacteriales Propionicimonas sp isolate 7M012 MGC4153443.1 316 aa
BacClassXI Bacillati Actinomycetota Actinomycetes Propionibacteriales Propionicimonas sp isolate AMR MDS 5329 HRA75944.1 316 aa
BacClassXI Bacillati Actinomycetota Actinomycetes Propionibacteriales Propionicimonas sp GCF 036504415.1 WP 331547905.1 317 aa
BacClassXI Bacillati Actinomycetota Actinomycetes Propionibacteriales Propionicimonas sp GCF 022013875.1 iso1 WP 294125886.1 316 aa
BacClassXI Bacillati Actinomycetota Actinomycetes Propionibacteriales Propionicimonas sp GCF 049840635.1 WP 428537676.1 317 aa
BacClassXI Bacillati Actinomycetota Actinomycetes Propionibacteriales Propionicimonas paludicola WP 098459360.1 317 aa
BacClassXI Bacillati Actinomycetota Actinomycetes Kineosporiales Angustibacter aerolatus strain JCM 32086 GAB3692626.1 318 aa
BacClassXI Bacillati Actinomycetota Actinomycetes Kineosporiales Angustibacter sp McL0619 iso1 WP 426564523.1 318 aa
BacClassXI Bacillati Actinomycetota Actinomycetes Kineosporiales Angustibacter sp Root456 iso2 WP 056679945.1 318 aa
BacClassXI Bacillati Actinomycetota Actinomycetes Actinomycetales Actinomycetales bacterium isolate SMAG U538 HET8614537.1 321 aa
BacClassXI Bacillati Actinomycetota Actinomycetes Propionibacteriales Nocardioides sp isolate SMAG U5804 HEX4685731.1 318 aa
BacClassXI Bacillati Actinomycetota Actinomycetes Geodermatophilales Geodermatophilaceae bacterium isolate MGR bin60 MBA3524251 318 aa
BacClassXI Bacillati Actinomycetota Actinomycetes Geodermatophilales Geodermatophilaceae bacterium isolate MGR bin242 MBA2415714 318 aa
BacClassXI Bacillati Actinomycetota Actinomycetes Geodermatophilales Geodermatophilaceae bacterium isolate MGR bin38 iso2 MBA324 319 aa
BacClassXI Bacillati Actinomycetota Actinomycetes Geodermatophilales Geodermatophilaceae bacterium isolate MGR bin74 iso2 MBA336 329 aa
BacClassXI Bacillati Actinomycetota Acidimicrobiia Acidimicrobiales Desertimonas flava WP 116997876.1 326 aa
BacClassXI Bacillati Actinomycetota Acidimicrobiia Acidimicrobiales Desertimonas sp isolate VLC 103 MFV0306532.1 326 aa
BacClassXI Bacillati Actinomycetota Acidimicrobiia Acidimicrobiales Acidimicrobiales bacterium isolate SCS4bin.41 MEE9417231.1 328 aa
BacClassXI Bacillati Actinomycetota Nitriliruptoria Euzebya sp isolate TH ant mag 40 iso2 MGI9016182.1 324 aa
BacClassXI Bacillati Actinomycetota Actinomycetes unclassified Actinomycetes Actinomycetes bacterium isolate SMAG U2051 HEX71890 346 aa
BacClassXI Bacillati Actinomycetota Actinomycetes Propionibacteriales Nocardioidaceae bacterium isolate TH ant mag 204 iso1 MGI8 326 aa
BacClassXI Bacillati Actinomycetota Actinomycetes Propionibacteriales Nocardioidaceae bacterium isolate SMAG U10749 iso1 HZG9923 330 aa
BacClassXI Bacillati Actinomycetota Actinomycetes Propionibacteriales Solicola gregarius WP 271632823.1 330 aa
BacClassXI Bacillati Actinomycetota Actinomycetes unclassified Actinomycetes Actinomycetes bacterium isolate SMAG U10486 HLR8394 335 aa
BacClassXI Bacillati Actinomycetota Actinomycetes Micrococcales Arsenicicoccus cauae WP 311966645.1 326 aa
BacClassXI Bacillati Actinomycetota Actinomycetes Propionibacteriales Aeromicrobium wangtongii WP 232398486.1 325 aa
BacClassXI Bacillati Actinomycetota Actinomycetes Propionibacteriales Aeromicrobium sp CTD01 1L150 WP 375002903.1 325 aa
BacClassXI Bacillati Actinomycetota Actinomycetes Propionibacteriales Aeromicrobium sp Root495 WP 056283099.1 325 aa
BacClassXI Bacillati Actinomycetota Actinomycetes Propionibacteriales Aeromicrobium sp isolate SMAG U12978 iso2 HWV26708.1 318 aa
BacClassXI Bacillati Actinomycetota Actinomycetes Propionibacteriales Aeromicrobium phragmitis WP 121793705.1 325 aa
BacClassXI Bacillati Actinomycetota Actinomycetes Propionibacteriales Aeromicrobium sp GCF 047607065.1 iso1 WP 415003957.1 325 aa
BacClassXI Bacillati Actinomycetota Actinomycetes Propionibacteriales Aeromicrobium marinum WP 040320029.1 325 aa
BacClassXI Bacillati Actinomycetota Actinomycetes Jatrophihabitantales Jatrophihabitans endophyticus GCF 015069775.1 iso1 WP 304 329 aa
BacClassXI Bacillati Actinomycetota Actinomycetes Jatrophihabitantales uncultured Jatrophihabitans sp GCF 964246335.1 iso1 WP 3 330 aa
BacClassXI Bacillati Actinomycetota Actinomycetes Jatrophihabitantales Jatrophihabitans sp isolate SMAG U981 iso1 HET6876931.1 326 aa
BacClassXI Bacillati Actinomycetota Actinomycetes Jatrophihabitantales Jatrophihabitantaceae bacterium isolate TC S metawrap bin 329 aa
BacClassXI Bacillati Actinomycetota Actinomycetes Jatrophihabitantales Jatrophihabitantaceae bacterium isolate SMAG U5249 HEY227 328 aa
BacClassXI Bacillati Actinomycetota Actinomycetes Jatrophihabitantales Jatrophihabitans sp isolate SMAG U4181 HEY8302839.1 327 aa
BacClassXI Bacillati Actinomycetota Actinomycetes Jatrophihabitantales Jatrophihabitans sp GCF 036000485.1 iso2 WP 327539416.1 326 aa
BacClassXI Bacillati Actinomycetota Actinomycetes Jatrophihabitantales uncultured Jatrophihabitans sp GCF 964251945.1 iso2 WP 3 330 aa
BacClassXI Bacillati Actinomycetota Actinomycetes Pseudonocardiales Pseudonocardiales bacterium isolate BP 207 MDT4917400.1 323 aa
BacClassXI Bacillati Actinomycetota Actinomycetes Jatrophihabitantales Jatrophihabitans sp isolate SMAG U6145 iso1 HVU91251.1 328 aa
BacClassXI Bacillati Actinomycetota Actinomycetes Jatrophihabitantales Jatrophihabitans sp GCF 046262535.1 WP 409454166.1 328 aa
BacClassXI Bacillati Actinomycetota Actinomycetes Propionibacteriales Nocardioidaceae bacterium isolate SMAG U2155 iso1 HEU02865 324 aa
BacClassXI Bacillati Actinomycetota Actinomycetes Micrococcales Luteimicrobium sp NPDC057192 WP 386797605.1 329 aa
BacClassXI Bacillati Actinomycetota Actinomycetes Micrococcales Demequina gelatinilytica WP 062518557.1 324 aa
BacClassXI Bacillati Actinomycetota Actinomycetes Propionibacteriales Kribbella sp NPDC056345 WP 376026497.1 323 aa
BacClassXI Bacillati Actinomycetota Actinomycetes Propionibacteriales Nocardioides cheoyonin strain KR10 350 iso1 WP 348860628.1 323 aa
BacClassXI Bacillati Actinomycetota Actinomycetes Propionibacteriales Nocardioides sp isolate Bacteria.bin 21 MBO0842190.1 321 aa
BacClassXI Bacillati Actinomycetota Actinomycetes Propionibacteriales Kribbellaceae bacterium isolate SMAG U10096 iso2 HZO64445 335 aa
BacClassXI Bacillati Actinomycetota Actinomycetes Propionibacteriales Kribbellaceae bacterium isolate SMAG U11545 HYU83765.1 333 aa
BacClassXI Bacillati Actinomycetota Actinomycetes Propionibacteriales Kribbella sp NBC 01245 WP 328995111.1 325 aa
BacClassXI Bacillati Actinomycetota Actinomycetes Propionibacteriales Kribbella sp isolate SMAG U3553 HEY0688750.1 325 aa
BacClassXI Bacillati Actinomycetota Actinomycetes Propionibacteriales Kribbella sp NPDC006257 WP 355686518.1 325 aa
BacClassXI Bacillati Actinomycetota Actinomycetes Propionibacteriales Kribbella caucasensis WP 133800080.1 325 aa
BacClassXI Bacillati Actinomycetota Actinomycetes Propionibacteriales Propionibacteriales bacterium isolate MGR bin129 iso2 MBA2 327 aa
BacClassXI Bacillati Actinomycetota Actinomycetes Propionibacteriales Nocardioidaceae bacterium isolate MFD05499 ilm asm bin14 M 327 aa
BacClassXI Bacillati Actinomycetota Actinomycetes Propionibacteriales Nocardioides panacis strain G188 WP 216937719.1 327 aa
BacClassXI Bacillati Actinomycetota Actinomycetes Propionibacteriales Nocardioides sp isolate TPT25 1 semi13 MGZ8741388.1 326 aa
BacClassXI Bacillati Actinomycetota Actinomycetes Propionibacteriales Nocardioides iriomotensis WP 129986801.1 326 aa
BacClassXI Bacillati Actinomycetota Actinomycetes Propionibacteriales Nocardioides sp GCF 030698745.1 iso2 WP 305710292.1 326 aa
BacClassXI Bacillati Actinomycetota Nitriliruptoria Euzebya sp GCF 964222135.1 iso2 WP 370326209.1 326 aa
BacClassXI Bacillati Actinomycetota Actinomycetes Propionibacteriales Nocardioides sp GCF 050246775.1 iso2 WP 428345870.1 326 aa
BacClassXI Bacillati Actinomycetota Actinomycetes Propionibacteriales Nocardioides sp isolate SMAG U4636 HEY3014018.1 327 aa
BacClassXI Bacillati Actinomycetota Actinomycetes Micrococcales Pseudactinotalea terrae strain KACC 16542 iso2 WP 156252750.1 325 aa
BacClassXI Bacillati Actinomycetota Actinomycetes Propionibacteriales Nocardioides bigeumensis strain JCM 16021 iso1 WP 34430399 325 aa
BacClassXI Bacillati Actinomycetota Actinomycetes Propionibacteriales Nocardioides rotundus strain MCCC 1A10561 iso1 WP 22339514 328 aa
BacClassXI Bacillati Actinomycetota Actinomycetes Propionibacteriales Nocardioides caricicola WP 345176225.1 325 aa
BacClassXI Bacillati Actinomycetota Actinomycetes Propionibacteriales Nocardioides marinquilinus WP 345458265.1 329 aa
BacClassXI Bacillati Actinomycetota Actinomycetes Propionibacteriales Nocardioides dongxiaopingii strain 4053 iso1 WP 137292525 328 aa
BacClassXI Bacillati Actinomycetota Actinomycetes Propionibacteriales Nocardioides anomalus WP 165238587.1 325 aa
BacClassXI Bacillati Actinomycetota Actinomycetes Propionibacteriales Nocardioides sp InS609 2 iso2 WP 248582908.1 325 aa
BacClassXI Bacillati Actinomycetota Actinomycetes Propionibacteriales Nocardioides lijunqiniae WP 193606699.1 325 aa
BacClassXI Bacillati Actinomycetota Actinomycetes Propionibacteriales Nocardioides sp CFH 31398 WP 240236439.1 326 aa
BacClassXI Bacillati Actinomycetota Actinomycetes Propionibacteriales Nocardioides panaciterrulae WP 179665704.1 325 aa
BacClassXI Bacillati Actinomycetota Actinomycetes Propionibacteriales Nocardioides dilutus strain JCM 31491 iso2 WP 460714392.1 325 aa
BacClassXI Bacillati Actinomycetota Actinomycetes Micrococcales Pedococcus sp NPDC057267 WP 379116058.1 329 aa
BacClassXI Bacillati Actinomycetota Actinomycetes Micrococcales Pedococcus sp isolate SMAG U3974 HEY7718797.1 329 aa
BacClassXI Bacillati Actinomycetota Actinomycetes Micrococcales Intrasporangiaceae sp WP 091760190.1 329 aa

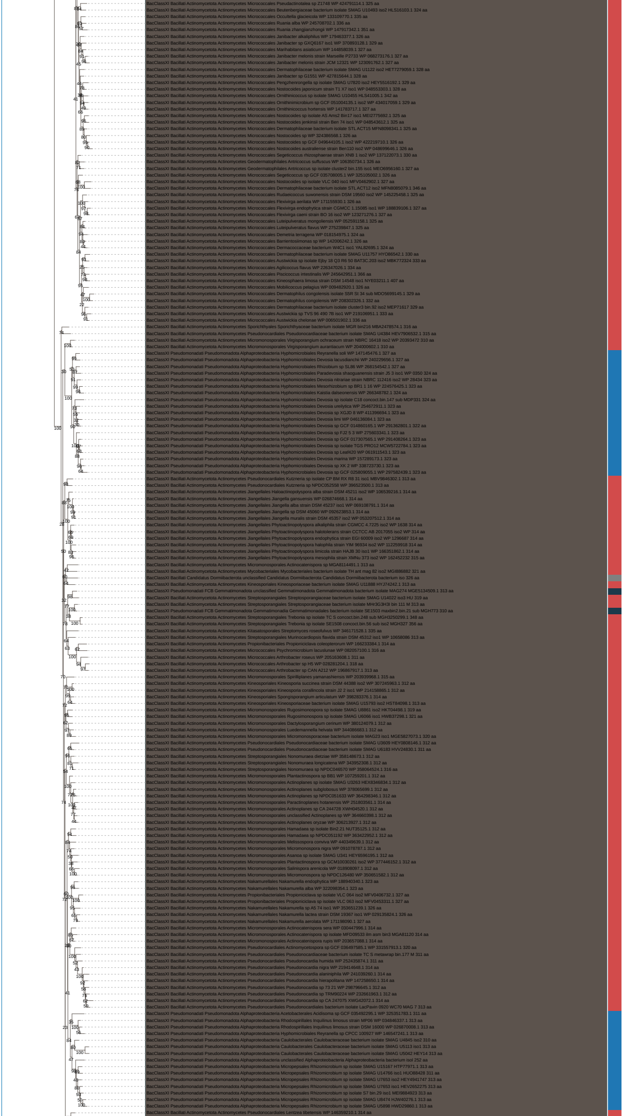
BacClassXI Bacillati Actinomycetota Actinomycetes Micrococcales Pseudactinotalea sp Z1748 WP 424791114.1 325 aa
BacClassXI Bacillati Actinomycetota Actinomycetes Micrococcales Beutenbergiaceae bacterium isolate SMAG U10493 iso2 HLS16103.1 324 aa
BacClassXI Bacillati Actinomycetota Actinomycetes Micrococcales Occultella glacieicola WP 133109770.1 335 aa
BacClassXI Bacillati Actinomycetota Actinomycetes Micrococcales Ruania alba WP 245708702.1 336 aa
BacClassXI Bacillati Actinomycetota Actinomycetes Micrococcales Ruania zhangjianzhongii WP 147917342.1 351 aa
BacClassXI Bacillati Actinomycetota Actinomycetes Micrococcales Janibacter alkaliphilus WP 179463377.1 326 aa
BacClassXI Bacillati Actinomycetota Actinomycetes Micrococcales Janibacter sp GXQ6167 iso1 WP 370893128.1 329 aa
BacClassXI Bacillati Actinomycetota Actinomycetes Micrococcales Marihabitans asiaticum WP 144858039.1 327 aa
BacClassXI Bacillati Actinomycetota Actinomycetes Micrococcales Janibacter melonis strain Marseille P2733 WP 068273176.1 327 aa
BacClassXI Bacillati Actinomycetota Actinomycetes Micrococcales Janibacter melonis strain JCM 12321 WP 123091762.1 327 aa
BacClassXI Bacillati Actinomycetota Actinomycetes Micrococcales Dermatophilaceae bacterium isolate SMAG U1122 iso2 HET7279059.1 328 aa
BacClassXI Bacillati Actinomycetota Actinomycetes Micrococcales Janibacter sp G1551 WP 427815644.1 328 aa
BacClassXI Bacillati Actinomycetota Actinomycetes Micrococcales Pengzhenrongella sp isolate SMAG U7820 iso2 HEY5516192.1 329 aa
BacClassXI Bacillati Actinomycetota Actinomycetes Micrococcales Nostocoides japonicum strain T1 X7 iso1 WP 048553303.1 328 aa
BacClassXI Bacillati Actinomycetota Actinomycetes Micrococcales Ornithinicoccus sp isolate SMAG U10455 HLS41005.1 342 aa
BacClassXI Bacillati Actinomycetota Actinomycetes Micrococcales Ornithinimicrobium sp GCF 051004135.1 iso2 WP 434017059.1 329 aa
BacClassXI Bacillati Actinomycetota Actinomycetes Micrococcales Ornithinicoccus hortensis WP 141783717.1 327 aa
BacClassXI Bacillati Actinomycetota Actinomycetes Micrococcales Nostocoides sp isolate AS Ams2 Bin17 iso1 MEI2775692.1 325 aa
BacClassXI Bacillati Actinomycetota Actinomycetes Micrococcales Nostocoides jenkinsii strain Ben 74 iso1 WP 048543612.1 325 aa
BacClassXI Bacillati Actinomycetota Actinomycetes Micrococcales Dermatophilaceae bacterium isolate STL ACT15 MFN8098341.1 325 aa
BacClassXI Bacillati Actinomycetota Actinomycetes Micrococcales Nostocoides sp WP 324386568.1 326 aa
BacClassXI Bacillati Actinomycetota Actinomycetes Micrococcales Nostocoides sp GCF 049644105.1 iso2 WP 422219710.1 326 aa
BacClassXI Bacillati Actinomycetota Actinomycetes Micrococcales Nostocoides australiense strain Ben110 iso2 WP 048699646.1 326 aa
BacClassXI Bacillati Actinomycetota Actinomycetes Micrococcales Segeticoccus rhizosphaerae strain XNB 1 iso2 WP 137122073.1 330 aa
BacClassXI Bacillati Actinomycetota Actinomycetes Geodermatophilales Antricoccus suffuscus WP 106350734.1 326 aa
BacClassXI Bacillati Actinomycetota Actinomycetes Geodermatophilales Antricoccus sp isolate cluster2 bin.155 iso1 MEO6956160.1 327 aa
BacClassXI Bacillati Actinomycetota Actinomycetes Micrococcales Segeticoccus sp GCF 035708005.1 WP 325105002.1 326 aa
BacClassXI Bacillati Actinomycetota Actinomycetes Micrococcales Nostocoides sp isolate VLC 040 iso1 MFV0462902.1 327 aa
BacClassXI Bacillati Actinomycetota Actinomycetes Micrococcales Dermatophilaceae bacterium isolate STL ACT12 iso2 MFN8085079.1 346 aa
BacClassXI Bacillati Actinomycetota Actinomycetes Micrococcales Rudaeicoccus suwonensis strain DSM 19560 iso2 WP 145225458.1 325 aa
BacClassXI Bacillati Actinomycetota Actinomycetes Micrococcales Flexivirga aerilata WP 171155930.1 326 aa
BacClassXI Bacillati Actinomycetota Actinomycetes Micrococcales Flexivirga endophytica strain CGMCC 1.15085 iso1 WP 188839106.1 327 aa
BacClassXI Bacillati Actinomycetota Actinomycetes Micrococcales Flexivirga caeni strain BO 16 iso2 WP 123271276.1 327 aa
BacClassXI Bacillati Actinomycetota Actinomycetes Micrococcales Luteipulveratus mongoliensis WP 052591158.1 325 aa
BacClassXI Bacillati Actinomycetota Actinomycetes Micrococcales Luteipulveratus flavus WP 275239847.1 325 aa
BacClassXI Bacillati Actinomycetota Actinomycetes Micrococcales Demetria terragena WP 018154975.1 324 aa
BacClassXI Bacillati Actinomycetota Actinomycetes Micrococcales Barrientosiimonas sp WP 142006242.1 326 aa
BacClassXI Bacillati Actinomycetota Actinomycetes Micrococcales Dermacoccaceae bacterium W4C1 iso1 YAL82695.1 324 aa
BacClassXI Bacillati Actinomycetota Actinomycetes Micrococcales Dermatophilaceae bacterium isolate SMAG U11757 HYO86542.1 330 aa
BacClassXI Bacillati Actinomycetota Actinomycetes Micrococcales Austwickia sp isolate Ejby 18 Q3 R6 50 BAT3C.203 iso2 MBK772324 333 aa
BacClassXI Bacillati Actinomycetota Actinomycetes Micrococcales Agilicoccus flavus WP 226347026.1 334 aa
BacClassXI Bacillati Actinomycetota Actinomycetes Micrococcales Piscicoccus intestinalis WP 245642951.1 366 aa
BacClassXI Bacillati Actinomycetota Actinomycetes Micrococcales Kineosphaera limosa strain DSM 14548 iso1 NYE03211.1 407 aa
BacClassXI Bacillati Actinomycetota Actinomycetes Micrococcales Mobilicoccus pelagius WP 009482920.1 326 aa
BacClassXI Bacillati Actinomycetota Actinomycetes Micrococcales Dermatophilus congolensis isolate S5R St 34 sub MDO5699145.1 329 aa
BacClassXI Bacillati Actinomycetota Actinomycetes Micrococcales Dermatophilus congolensis WP 208302326.1 332 aa
BacClassXI Bacillati Actinomycetota Actinomycetes Micrococcales Dermatophilaceae bacterium isolate cluster3 bin.92 iso2 MEP71617 329 aa
BacClassXI Bacillati Actinomycetota Actinomycetes Micrococcales Austwickia sp TVS 96 490 7B iso1 WP 219106951.1 333 aa
BacClassXI Bacillati Actinomycetota Actinomycetes Micrococcales Austwickia chelonae WP 006501902.1 336 aa
BacClassXI Bacillati Actinomycetota Actinomycetes Sporichthyales Sporichthyaceae bacterium isolate MGR bin216 MBA2478574.1 316 aa
BacClassXI Bacillati Actinomycetota Actinomycetes Pseudonocardiales Pseudonocardiaceae bacterium isolate SMAG U4384 HEV7906532.1 315 aa
BacClassXI Bacillati Actinomycetota Actinomycetes Micromonosporales Virgisporangium ochraceum strain NBRC 16418 iso2 WP 20393472 310 aa
BacClassXI Bacillati Actinomycetota Actinomycetes Micromonosporales Virgisporangium aurantiacum WP 204000602.1 310 aa
BacClassXI Pseudomonadati Pseudomonadota Alphaproteobacteria Hyphomicrobiales Reyranella soli WP 147145476.1 327 aa
BacClassXI Pseudomonadati Pseudomonadota Alphaproteobacteria Hyphomicrobiales Devosia lacusdianchii WP 240229656.1 327 aa
BacClassXI Pseudomonadati Pseudomonadota Alphaproteobacteria Hyphomicrobiales Rhizobium sp SL86 WP 268154542.1 327 aa
BacClassXI Pseudomonadati Pseudomonadota Alphaproteobacteria Hyphomicrobiales Paradevosia shaoguanensis strain J5 3 iso1 WP 0350 324 aa
BacClassXI Pseudomonadati Pseudomonadota Alphaproteobacteria Hyphomicrobiales Devosia nitrariae strain NBRC 112416 iso2 WP 28434 323 aa
BacClassXI Pseudomonadati Pseudomonadota Alphaproteobacteria Hyphomicrobiales Mesorhizobium sp BR1 1 16 WP 224576425.1 323 aa
BacClassXI Pseudomonadati Pseudomonadota Alphaproteobacteria Hyphomicrobiales Kaistia dalseonensis WP 266348782.1 324 aa
BacClassXI Pseudomonadati Pseudomonadota Alphaproteobacteria Hyphomicrobiales Devosia sp isolate C18 concoct.bin.147 sub MDP331 324 aa
BacClassXI Pseudomonadati Pseudomonadota Alphaproteobacteria Hyphomicrobiales Devosia ureilytica WP 254672911.1 323 aa
BacClassXI Pseudomonadati Pseudomonadota Alphaproteobacteria Hyphomicrobiales Devosia sp XGJD 8 WP 411396694.1 323 aa
BacClassXI Pseudomonadati Pseudomonadota Alphaproteobacteria Hyphomicrobiales Devosia limi WP 046136084.1 323 aa
BacClassXI Pseudomonadati Pseudomonadota Alphaproteobacteria Hyphomicrobiales Devosia sp GCF 014860165.1 WP 291362801.1 322 aa
BacClassXI Pseudomonadati Pseudomonadota Alphaproteobacteria Hyphomicrobiales Devosia sp FJ2 5 3 WP 275603341.1 323 aa
BacClassXI Pseudomonadati Pseudomonadota Alphaproteobacteria Hyphomicrobiales Devosia sp GCF 017307565.1 WP 291408264.1 323 aa
BacClassXI Pseudomonadati Pseudomonadota Alphaproteobacteria Hyphomicrobiales Devosia sp isolate TGS PRO12 MCW5722784.1 323 aa
BacClassXI Pseudomonadati Pseudomonadota Alphaproteobacteria Hyphomicrobiales Devosia sp Leaf420 WP 061911543.1 323 aa
BacClassXI Pseudomonadati Pseudomonadota Alphaproteobacteria Hyphomicrobiales Devosia marina WP 157289173.1 323 aa
BacClassXI Pseudomonadati Pseudomonadota Alphaproteobacteria Hyphomicrobiales Devosia sp XK 2 WP 338723730.1 323 aa
BacClassXI Pseudomonadati Pseudomonadota Alphaproteobacteria Hyphomicrobiales Devosia sp GCF 025809055.1 WP 297582439.1 323 aa
BacClassXI Bacillati Actinomycetota Actinomycetes Pseudonocardiales Kutzneria sp isolate CP BM RX R8 31 iso1 MBV9846302.1 313 aa
BacClassXI Bacillati Actinomycetota Actinomycetes Pseudonocardiales Kutzneria sp NPDC052558 WP 396523500.1 313 aa
BacClassXI Bacillati Actinomycetota Actinomycetes Jiangellales Haloactinopolyspora alba strain DSM 45211 iso2 WP 106539216.1 314 aa
BacClassXI Bacillati Actinomycetota Actinomycetes Jiangellales Jiangella gansuensis WP 026874668.1 314 aa
BacClassXI Bacillati Actinomycetota Actinomycetes Jiangellales Jiangella alba strain DSM 45237 iso1 WP 069108791.1 314 aa
BacClassXI Bacillati Actinomycetota Actinomycetes Jiangellales Jiangella sp DSM 45060 WP 092623853.1 314 aa
BacClassXI Bacillati Actinomycetota Actinomycetes Jiangellales Jiangella muralis strain DSM 45357 iso2 WP 053207512.1 314 aa
BacClassXI Bacillati Actinomycetota Actinomycetes Jiangellales Phytoactinopolyspora alkaliphila strain CGMCC 4.7225 iso2 WP 1638 314 aa
BacClassXI Bacillati Actinomycetota Actinomycetes Jiangellales Phytoactinopolyspora halotolerans strain CCTCC AB 2017055 iso2 WP 314 aa
BacClassXI Bacillati Actinomycetota Actinomycetes Jiangellales Phytoactinopolyspora endophytica strain EGI 60009 iso2 WP 1296687 314 aa
BacClassXI Bacillati Actinomycetota Actinomycetes Jiangellales Phytoactinopolyspora halophila strain YIM 96934 iso2 WP 112259918 314 aa
BacClassXI Bacillati Actinomycetota Actinomycetes Jiangellales Phytoactinopolyspora limicola strain HAJB 30 iso1 WP 166351862.1 314 aa
BacClassXI Bacillati Actinomycetota Actinomycetes Jiangellales Phytoactinopolyspora mesophila strain XMNu 373 iso2 WP 162452232 315 aa
BacClassXI Bacillati Actinomycetota Actinomycetes Micromonosporales Actinocatenispora sp MGA8114491.1 313 aa
BacClassXI Bacillati Actinomycetota Actinomycetes Mycobacteriales Mycobacteriales bacterium isolate TH ant mag 82 iso2 MGI886882 321 aa
BacClassXI Bacillati Candidatus Dormiibacterota unclassified Candidatus Dormiibacterota Candidatus Dormiibacterota bacterium iso 326 aa
BacClassXI Bacillati Actinomycetota Actinomycetes Kineosporiales Kineosporiaceae bacterium isolate SMAG U11888 HYJ74242.1 313 aa
BacClassXI Pseudomonadati FCB Gemmatimonadota unclassified Gemmatimonadota Gemmatimonadota bacterium isolate MAG274 MGE5134509.1 313 aa
BacClassXI Bacillati Actinomycetota Actinomycetes Streptosporangiales Streptosporangiaceae bacterium isolate SMAG U14022 iso3 HU 319 aa
BacClassXI Bacillati Actinomycetota Actinomycetes Streptosporangiales Streptosporangiaceae bacterium isolate MHr3G3H3I bin 111 M 313 aa
BacClassXI Pseudomonadati FCB Gemmatimonadota Gemmatimonadia Gemmatimonadales bacterium isolate SE1503 maxbin2.bin.21 sub MGH773 310 aa
BacClassXI Bacillati Actinomycetota Actinomycetes Streptosporangiales Trebonia sp isolate TC S concoct.bin.248 sub MGH3250299.1 348 aa
BacClassXI Bacillati Actinomycetota Actinomycetes Streptosporangiales Trebonia sp isolate SE1508 concoct.bin.56 sub iso2 MGH327 356 aa
BacClassXI Bacillati Actinomycetota Actinomycetes Kitasatosporales Streptomyces roseofulvus WP 346171528.1 335 aa
BacClassXI Bacillati Actinomycetota Actinomycetes Streptosporangiales Murinocardiopsis flavida strain DSM 45312 iso1 WP 10658086 313 aa
BacClassXI Bacillati Actinomycetota Actinomycetes Propionibacteriales Propioniciclava coleopterorum WP 166233384.1 314 aa
BacClassXI Bacillati Actinomycetota Actinomycetes Micrococcales Psychromicrobium lacuslunae WP 082057100.1 316 aa
BacClassXI Bacillati Actinomycetota Actinomycetes Micrococcales Arthrobacter roseus WP 205163608.1 311 aa
BacClassXI Bacillati Actinomycetota Actinomycetes Micrococcales Arthrobacter sp H5 WP 028281204.1 318 aa
BacClassXI Bacillati Actinomycetota Actinomycetes Micrococcales Arthrobacter sp CAN A212 WP 196867917.1 313 aa
BacClassXI Bacillati Actinomycetota Actinomycetes Micromonosporales Spirilliplanes yamanashiensis WP 203939968.1 315 aa
BacClassXI Bacillati Actinomycetota Actinomycetes Kineosporiales Kineosporia succinea strain DSM 44388 iso2 WP 307245963.1 312 aa
BacClassXI Bacillati Actinomycetota Actinomycetes Kineosporiales Kineosporia corallincola strain J2 2 iso1 WP 214158865.1 312 aa
BacClassXI Bacillati Actinomycetota Actinomycetes Kineosporiales Spongisporangium articulatum WP 398283376.1 314 aa
BacClassXI Bacillati Actinomycetota Actinomycetes Kineosporiales Kineosporiaceae bacterium isolate SMAG U15793 iso2 HST84098.1 313 aa
BacClassXI Bacillati Actinomycetota Actinomycetes Micromonosporales Rugosimonospora sp isolate SMAG U8861 iso2 HKT04498.1 319 aa
BacClassXI Bacillati Actinomycetota Actinomycetes Micromonosporales Rugosimonospora sp isolate SMAG U6066 iso1 HWB37298.1 321 aa
BacClassXI Bacillati Actinomycetota Actinomycetes Micromonosporales Dactylosporangium cerinum WP 380124079.1 312 aa
BacClassXI Bacillati Actinomycetota Actinomycetes Micromonosporales Luedemannella helvata WP 344086683.1 312 aa
BacClassXI Bacillati Actinomycetota Actinomycetes Micromonosporales Micromonosporaceae bacterium isolate MAG23 iso1 MGE5827073.1 320 aa
BacClassXI Bacillati Actinomycetota Actinomycetes Pseudonocardiales Pseudonocardiaceae bacterium isolate SMAG U3609 HEY0808146.1 312 aa
BacClassXI Bacillati Actinomycetota Actinomycetes Pseudonocardiales Pseudonocardiaceae bacterium isolate SMAG U6183 HVV24830.1 311 aa
BacClassXI Bacillati Actinomycetota Actinomycetes Streptosporangiales Nonomuraea dietziae WP 358148673.1 312 aa
BacClassXI Bacillati Actinomycetota Actinomycetes Streptosporangiales Nonomuraea longicatena WP 343952308.1 312 aa
BacClassXI Bacillati Actinomycetota Actinomycetes Streptosporangiales Nonomuraea sp NPDC046570 WP 358064524.1 316 aa
BacClassXI Bacillati Actinomycetota Actinomycetes Micromonosporales Plantactinospora sp BB1 WP 107259201.1 312 aa
BacClassXI Bacillati Actinomycetota Actinomycetes Micromonosporales Actinoplanes sp isolate SMAG U3263 HEX8346834.1 312 aa
BacClassXI Bacillati Actinomycetota Actinomycetes Micromonosporales Actinoplanes subglobosus WP 378065699.1 312 aa
BacClassXI Bacillati Actinomycetota Actinomycetes Micromonosporales Actinoplanes sp NPDC051633 WP 364298346.1 312 aa
BacClassXI Bacillati Actinomycetota Actinomycetes Micromonosporales Paractinoplanes hotanensis WP 251803561.1 314 aa
BacClassXI Bacillati Actinomycetota Actinomycetes Micromonosporales Actinoplanes sp CA 244728 XWH04520.1 312 aa
BacClassXI Bacillati Actinomycetota Actinomycetes Micromonosporales unclassified Actinoplanes sp WP 364660398.1 312 aa
BacClassXI Bacillati Actinomycetota Actinomycetes Micromonosporales Actinoplanes oryzae WP 306213927.1 312 aa
BacClassXI Bacillati Actinomycetota Actinomycetes Micromonosporales Hamadaea sp isolate Bin2.21 NUT35125.1 312 aa
BacClassXI Bacillati Actinomycetota Actinomycetes Micromonosporales Hamadaea sp NPDC051192 WP 363422952.1 312 aa
BacClassXI Bacillati Actinomycetota Actinomycetes Micromonosporales Melissospora conviva WP 440349639.1 312 aa
BacClassXI Bacillati Actinomycetota Actinomycetes Micromonosporales Micromonospora nigra WP 091078787.1 312 aa
BacClassXI Bacillati Actinomycetota Actinomycetes Micromonosporales Asanoa sp isolate SMAG U341 HEY6596195.1 312 aa
BacClassXI Bacillati Actinomycetota Actinomycetes Micromonosporales Plantactinospora sp GCM10030261 iso2 WP 377446152.1 312 aa
BacClassXI Bacillati Actinomycetota Actinomycetes Micromonosporales Salinispora arenicola WP 018908097.1 312 aa
BacClassXI Bacillati Actinomycetota Actinomycetes Micromonosporales Micromonospora sp NPDC126480 WP 350651582.1 312 aa
BacClassXI Bacillati Actinomycetota Actinomycetes Nakamurellales Nakamurella endophytica WP 188940340.1 323 aa
BacClassXI Bacillati Actinomycetota Actinomycetes Nakamurellales Nakamurella alba WP 322098354.1 323 aa
BacClassXI Bacillati Actinomycetota Actinomycetes Propionibacteriales Propioniciclava sp isolate VLC 064 iso2 MFV0406732.1 327 aa
BacClassXI Bacillati Actinomycetota Actinomycetes Propionibacteriales Propioniciclava sp isolate VLC 063 iso2 MFV0453311.1 327 aa
BacClassXI Bacillati Actinomycetota Actinomycetes Nakamurellales Nakamurella sp A5 74 iso1 WP 353651239.1 326 aa
BacClassXI Bacillati Actinomycetota Actinomycetes Nakamurellales Nakamurella lactea strain DSM 19367 iso1 WP 029135824.1 326 aa
BacClassXI Bacillati Actinomycetota Actinomycetes Nakamurellales Nakamurella aerolata WP 171198090.1 327 aa
BacClassXI Bacillati Actinomycetota Actinomycetes Micromonosporales Actinocatenispora sera WP 030447996.1 314 aa
BacClassXI Bacillati Actinomycetota Actinomycetes Micromonosporales Actinocatenispora sp isolate MFD09533 ilm asm bin3 MGA81120 314 aa
BacClassXI Bacillati Actinomycetota Actinomycetes Micromonosporales Actinocatenispora rupis WP 203657088.1 314 aa
BacClassXI Bacillati Actinomycetota Actinomycetes Pseudonocardiales Actinomycetospora sp GCF 036497585.1 WP 331557913.1 320 aa
BacClassXI Bacillati Actinomycetota Actinomycetes Pseudonocardiales Pseudonocardiaceae bacterium isolate TC S metawrap bin.177 M 311 aa
BacClassXI Bacillati Actinomycetota Actinomycetes Pseudonocardiales Pseudonocardia humida WP 252435874.1 311 aa
BacClassXI Bacillati Actinomycetota Actinomycetes Pseudonocardiales Pseudonocardia nigra WP 219414648.1 314 aa
BacClassXI Bacillati Actinomycetota Actinomycetes Pseudonocardiales Pseudonocardia alaniniphila WP 241039260.1 314 aa
BacClassXI Bacillati Actinomycetota Actinomycetes Pseudonocardiales Pseudonocardia hierapolitana WP 147258650.1 314 aa
BacClassXI Bacillati Actinomycetota Actinomycetes Pseudonocardiales Pseudonocardia sp 73 21 WP 298796645.1 312 aa
BacClassXI Bacillati Actinomycetota Actinomycetes Pseudonocardiales Pseudonocardia sp TRM90224 WP 232661963.1 312 aa
BacClassXI Bacillati Actinomycetota Actinomycetes Pseudonocardiales Pseudonocardia sp CA 247075 XWG42072.1 314 aa
BacClassXI Bacillati Actinomycetota Actinomycetes Pseudonocardiales Pseudonocardiales bacterium isolate LacPavin 0920 WC70 MAG 7 313 aa
BacClassXI Pseudomonadati Pseudomonadota Alphaproteobacteria Acetobacterales Acidisoma sp GCF 035492295.1 WP 325351783.1 311 aa
BacClassXI Pseudomonadati Pseudomonadota Alphaproteobacteria Rhodospirillales Inquilinus limosus strain MP06 WP 034846337.1 313 aa
BacClassXI Pseudomonadati Pseudomonadota Alphaproteobacteria Rhodospirillales Inquilinus limosus strain DSM 16000 WP 026870008.1 313 aa
BacClassXI Pseudomonadati Pseudomonadota Alphaproteobacteria Hyphomicrobiales Reyranella sp CPCC 100927 WP 146547241.1 313 aa
BacClassXI Pseudomonadati Pseudomonadota Alphaproteobacteria Caulobacterales Caulobacteraceae bacterium isolate SMAG U4845 iso2 310 aa
BacClassXI Pseudomonadati Pseudomonadota Alphaproteobacteria Caulobacterales Caulobacteraceae bacterium isolate SMAG U5113 iso1 313 aa
BacClassXI Pseudomonadati Pseudomonadota Alphaproteobacteria Caulobacterales Caulobacteraceae bacterium isolate SMAG U5042 HEY14 313 aa
BacClassXI Pseudomonadati Pseudomonadota Alphaproteobacteria unclassified Alphaproteobacteria Alphaproteobacteria bacterium isol 252 aa
BacClassXI Pseudomonadati Pseudomonadota Alphaproteobacteria Micropepsales Rhizomicrobium sp isolate SMAG U15167 HTP77971.1 313 aa
BacClassXI Pseudomonadati Pseudomonadota Alphaproteobacteria Micropepsales Rhizomicrobium sp isolate SMAG U14766 iso1 HUO88428 311 aa
BacClassXI Pseudomonadati Pseudomonadota Alphaproteobacteria Micropepsales Rhizomicrobium sp isolate SMAG U7653 iso2 HEY4941747 313 aa
BacClassXI Pseudomonadati Pseudomonadota Alphaproteobacteria Micropepsales Rhizomicrobium sp isolate SMAG U7653 iso1 HEV2652275 313 aa
BacClassXI Pseudomonadati Pseudomonadota Alphaproteobacteria Micropepsales Rhizomicrobium sp isolate S7 bin.29 iso1 MEI9884923 313 aa
BacClassXI Pseudomonadati Pseudomonadota Alphaproteobacteria Micropepsales Rhizomicrobium sp isolate SMAG U8474 HJW40276.1 313 aa
BacClassXI Pseudomonadati Pseudomonadota Alphaproteobacteria Micropepsales Rhizomicrobium sp isolate SMAG U5898 HWD29860.1 313 aa
BacClassXI Bacillati Actinomycetota Actinomycetes Pseudonocardiales Lentzea tibetensis WP 146359210.1 314 aa

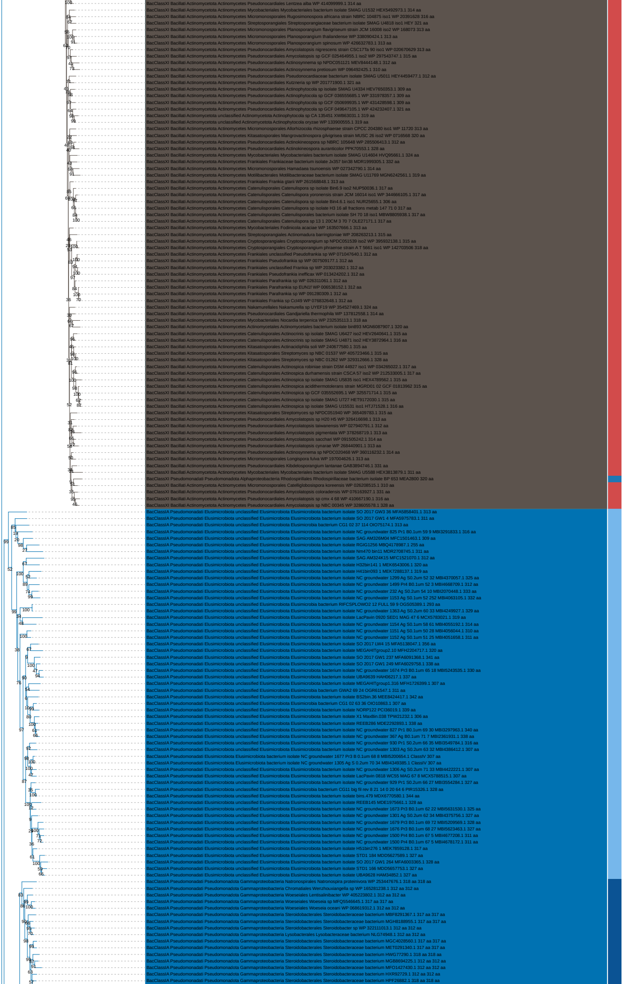

BacClassXI Bacillati Actinomycetota Actinomycetes Pseudonocardiales Lentzea alba WP 414099999.1 314 aa
BacClassXI Bacillati Actinomycetota Actinomycetes Mycobacteriales Mycobacteriales bacterium isolate SMAG U1532 HEX5492973.1 314 aa
BacClassXI Bacillati Actinomycetota Actinomycetes Micromonosporales Rugosimonospora africana strain NBRC 104875 iso1 WP 20391628 316 aa
BacClassXI Bacillati Actinomycetota Actinomycetes Streptosporangiales Streptosporangiaceae bacterium isolate SMAG U4818 iso1 HEY 321 aa
BacClassXI Bacillati Actinomycetota Actinomycetes Micromonosporales Planosporangium flavigriseum strain JCM 16008 iso2 WP 168073 313 aa
BacClassXI Bacillati Actinomycetota Actinomycetes Micromonosporales Planosporangium thailandense WP 338090424.1 313 aa
BacClassXI Bacillati Actinomycetota Actinomycetes Micromonosporales Planosporangium spinosum WP 426632783.1 313 aa
BacClassXI Bacillati Actinomycetota Actinomycetes Pseudonocardiales Amycolatopsis nigrescens strain CSC17Ta 90 iso1 WP 020670629 313 aa
BacClassXI Bacillati Actinomycetota Actinomycetes Pseudonocardiales Amycolatopsis sp GCF 025464955.1 iso2 WP 297543747.1 315 aa
BacClassXI Bacillati Actinomycetota Actinomycetes Pseudonocardiales Actinosynnema sp NPDC051121 MEV8444148.1 312 aa
BacClassXI Bacillati Actinomycetota Actinomycetes Pseudonocardiales Actinosynnema pretiosum WP 096492425.1 310 aa
BacClassXI Bacillati Actinomycetota Actinomycetes Pseudonocardiales Pseudonocardiaceae bacterium isolate SMAG U5011 HEY4459477.1 312 aa
BacClassXI Bacillati Actinomycetota Actinomycetes Pseudonocardiales Kutzneria sp WP 201771900.1 321 aa
BacClassXI Bacillati Actinomycetota Actinomycetes Pseudonocardiales Actinophytocola sp isolate SMAG U4334 HEV7650353.1 309 aa
BacClassXI Bacillati Actinomycetota Actinomycetes Pseudonocardiales Actinophytocola sp GCF 036555685.1 WP 331978357.1 309 aa
BacClassXI Bacillati Actinomycetota Actinomycetes Pseudonocardiales Actinophytocola sp GCF 050699935.1 WP 431428598.1 309 aa
BacClassXI Bacillati Actinomycetota Actinomycetes Pseudonocardiales Actinophytocola sp GCF 049647105.1 WP 424232407.1 321 aa
BacClassXI Bacillati Actinomycetota unclassified Actinomycetota Actinophytocola sp CA 135451 XWB63031.1 319 aa
BacClassXI Bacillati Actinomycetota unclassified Actinomycetota Actinophytocola oryzae WP 133900555.1 319 aa
BacClassXI Bacillati Actinomycetota Actinomycetes Micromonosporales Allorhizocola rhizosphaerae strain CPCC 204380 iso1 WP 11720 313 aa
BacClassXI Bacillati Actinomycetota Actinomycetes Kitasatosporales Mangrovactinospora gilvigrisea strain MUSC 26 iso2 WP 0716568 320 aa
BacClassXI Bacillati Actinomycetota Actinomycetes Pseudonocardiales Actinokineospora sp NBRC 105648 WP 285506413.1 312 aa
BacClassXI Bacillati Actinomycetota Actinomycetes Pseudonocardiales Actinokineospora auranticolor PPK70553.1 328 aa
BacClassXI Bacillati Actinomycetota Actinomycetes Mycobacteriales Mycobacteriales bacterium isolate SMAG U14604 HVQ95661.1 324 aa
BacClassXI Bacillati Actinomycetota Actinomycetes Frankiales Frankiaceae bacterium isolate Jx357 bin38 MDR1999305.1 332 aa
BacClassXI Bacillati Actinomycetota Actinomycetes Micromonosporales Hamadaea tsunoensis WP 027342790.1 314 aa
BacClassXI Bacillati Actinomycetota Actinomycetes Motilibacterales Motilibacteraceae bacterium isolate SMAG U11769 MGN6242561.1 319 aa
BacClassXI Bacillati Actinomycetota Actinomycetes Frankiales Frankia gtarii WP 261568848.1 313 aa
BacClassXI Bacillati Actinomycetota Actinomycetes Catenulisporales Catenulispora sp isolate Bin6.9 iso2 NUP50036.1 317 aa
BacClassXI Bacillati Actinomycetota Actinomycetes Catenulisporales Catenulispora yoronensis strain JCM 16014 iso1 WP 344666105.1 317 aa
BacClassXI Bacillati Actinomycetota Actinomycetes Catenulisporales Catenulispora sp isolate Bin4.6.1 iso1 NUR25655.1 306 aa
BacClassXI Bacillati Actinomycetota Actinomycetes Catenulisporales Catenulispora sp isolate H3 16 all fractions metab 147 71 0 317 aa
BacClassXI Bacillati Actinomycetota Actinomycetes Catenulisporales Catenulisporales bacterium isolate SH 70 18 iso1 MBW8805938.1 317 aa
BacClassXI Bacillati Actinomycetota Actinomycetes Catenulisporales Catenulispora sp 13 1 20CM 3 70 7 OLE27171.1 317 aa
BacClassXI Bacillati Actinomycetota Actinomycetes Mycobacteriales Fodinicola acaciae WP 163507666.1 313 aa
BacClassXI Bacillati Actinomycetota Actinomycetes Streptosporangiales Actinomadura barringtoniae WP 208263213.1 315 aa
BacClassXI Bacillati Actinomycetota Actinomycetes Cryptosporangiales Cryptosporangium sp NPDC051539 iso2 WP 395932138.1 315 aa
BacClassXI Bacillati Actinomycetota Actinomycetes Cryptosporangiales Cryptosporangium phraense strain A T 5661 iso1 WP 142703506 318 aa
BacClassXI Bacillati Actinomycetota Actinomycetes Frankiales unclassified Pseudofrankia sp WP 071047640.1 312 aa
BacClassXI Bacillati Actinomycetota Actinomycetes Frankiales Pseudofrankia sp WP 007509177.1 312 aa
BacClassXI Bacillati Actinomycetota Actinomycetes Frankiales unclassified Frankia sp WP 203023382.1 312 aa
BacClassXI Bacillati Actinomycetota Actinomycetes Frankiales Pseudofrankia inefficax WP 013424202.1 312 aa
BacClassXI Bacillati Actinomycetota Actinomycetes Frankiales Parafrankia sp WP 026311061.1 312 aa
BacClassXI Bacillati Actinomycetota Actinomycetes Frankiales Parafrankia sp EUN1f WP 006538152.1 312 aa
BacClassXI Bacillati Actinomycetota Actinomycetes Frankiales Parafrankia sp WP 091280309.1 312 aa
BacClassXI Bacillati Actinomycetota Actinomycetes Frankiales Frankia sp CcI49 WP 076832648.1 312 aa
BacClassXI Bacillati Actinomycetota Actinomycetes Nakamurellales Nakamurella sp UYEF19 WP 354527469.1 324 aa
BacClassXI Bacillati Actinomycetota Actinomycetes Pseudonocardiales Gandjariella thermophila WP 137812558.1 314 aa
BacClassXI Bacillati Actinomycetota Actinomycetes Mycobacteriales Nocardia terpenica WP 232535113.1 318 aa
BacClassXI Bacillati Actinomycetota Actinomycetes Actinomycetales Actinomycetales bacterium isolate bin893 MGN6087907.1 320 aa
BacClassXI Bacillati Actinomycetota Actinomycetes Catenulisporales Actinocrinis sp isolate SMAG U6427 iso2 HEV2640641.1 315 aa
BacClassXI Bacillati Actinomycetota Actinomycetes Catenulisporales Actinocrinis sp isolate SMAG U4871 iso2 HEY3872964.1 316 aa
BacClassXI Bacillati Actinomycetota Actinomycetes Kitasatosporales Actinacidiphila soli WP 240677580.1 315 aa
BacClassXI Bacillati Actinomycetota Actinomycetes Kitasatosporales Streptomyces sp NBC 01537 WP 405723466.1 315 aa
BacClassXI Bacillati Actinomycetota Actinomycetes Kitasatosporales Streptomyces sp NBC 01262 WP 329312666.1 328 aa
BacClassXI Bacillati Actinomycetota Actinomycetes Catenulisporales Actinospica robiniae strain DSM 44927 iso1 WP 034265022.1 317 aa
BacClassXI Bacillati Actinomycetota Actinomycetes Catenulisporales Actinospica durhamensis strain CSCA 57 iso2 WP 212533005.1 317 aa
BacClassXI Bacillati Actinomycetota Actinomycetes Catenulisporales Actinospica sp isolate SMAG U5835 iso1 HEX4789562.1 315 aa
BacClassXI Bacillati Actinomycetota Actinomycetes Catenulisporales Actinospica acidithermotolerans strain MGRD01 02 GCF 01813962 315 aa
BacClassXI Bacillati Actinomycetota Actinomycetes Catenulisporales Actinospica sp GCF 035552695.1 WP 325571714.1 315 aa
BacClassXI Bacillati Actinomycetota Actinomycetes Catenulisporales Actinospica sp isolate SMAG U727 HET9172030.1 315 aa
BacClassXI Bacillati Actinomycetota Actinomycetes Catenulisporales Actinospica sp isolate SMAG U15531 iso1 HTJ71528.1 316 aa
BacClassXI Bacillati Actinomycetota Actinomycetes Kitasatosporales Streptomyces sp NPDC051940 WP 365409783.1 315 aa
BacClassXI Bacillati Actinomycetota Actinomycetes Pseudonocardiales Amycolatopsis sp H20 H5 WP 326416698.1 313 aa
BacClassXI Bacillati Actinomycetota Actinomycetes Pseudonocardiales Amycolatopsis taiwanensis WP 027940791.1 312 aa
BacClassXI Bacillati Actinomycetota Actinomycetes Pseudonocardiales Amycolatopsis pigmentata WP 378268719.1 313 aa
BacClassXI Bacillati Actinomycetota Actinomycetes Pseudonocardiales Amycolatopsis sacchari WP 091505242.1 314 aa
BacClassXI Bacillati Actinomycetota Actinomycetes Pseudonocardiales Amycolatopsis cynarae WP 268440901.1 313 aa
BacClassXI Bacillati Actinomycetota Actinomycetes Pseudonocardiales Actinosynnema sp NPDC020468 WP 360116232.1 314 aa
BacClassXI Bacillati Actinomycetota Actinomycetes Micromonosporales Longispora fulva WP 197004626.1 313 aa
BacClassXI Bacillati Actinomycetota Actinomycetes Pseudonocardiales Kibdelosporangium lantanae GAB3894746.1 331 aa
BacClassXI Bacillati Actinomycetota Actinomycetes Mycobacteriales Mycobacteriales bacterium isolate SMAG U5588 HEX3813879.1 311 aa
BacClassXI Pseudomonadati Pseudomonadota Alphaproteobacteria Rhodospirillales Rhodospirillaceae bacterium isolate BP 653 MEA2800 320 aa
BacClassXI Bacillati Actinomycetota Actinomycetes Micromonosporales Catelliglobosispora koreensis WP 026208515.1 310 aa
BacClassXI Bacillati Actinomycetota Actinomycetes Pseudonocardiales Amycolatopsis coloradensis WP 076163927.1 331 aa
BacClassXI Bacillati Actinomycetota Actinomycetes Pseudonocardiales Amycolatopsis sp cmx 4 68 WP 410667190.1 316 aa
BacClassXI Bacillati Actinomycetota Actinomycetes Pseudonocardiales Amycolatopsis sp NBC 00345 WP 328605578.1 328 aa
BacClassIA Pseudomonadati Elusimicrobiota unclassified Elusimicrobiota Elusimicrobiota bacterium isolate SO 2017 GW3 36 MFA5858401.1 313 aa
BacClassIA Pseudomonadati Elusimicrobiota unclassified Elusimicrobiota Elusimicrobiota bacterium isolate SO 2017 GW1 4 MFA5975783.1 311 aa
BacClassIA Pseudomonadati Elusimicrobiota unclassified Elusimicrobiota Elusimicrobia bacterium CG1 02 37 114 OIO75174.1 313 aa
BacClassIA Pseudomonadati Elusimicrobiota unclassified Elusimicrobiota Elusimicrobiota bacterium isolate NC groundwater 825 Pr1 B0.1um 59 9 MBI3291833.1 316 aa
BacClassIA Pseudomonadati Elusimicrobiota unclassified Elusimicrobiota Elusimicrobiota bacterium isolate SAG AM326M04 MFC1501463.1 309 aa
BacClassIA Pseudomonadati Elusimicrobiota unclassified Elusimicrobiota Elusimicrobiota bacterium isolate RGIG1256 MBQ4178987.1 255 aa
BacClassIA Pseudomonadati Elusimicrobiota unclassified Elusimicrobiota Elusimicrobiota bacterium isolate Nm470 bin11 MDR2708745.1 311 aa
BacClassIA Pseudomonadati Elusimicrobiota unclassified Elusimicrobiota Elusimicrobiota bacterium isolate SAG AM324K15 MFC1521070.1 312 aa
BacClassIA Pseudomonadati Elusimicrobiota unclassified Elusimicrobiota Elusimicrobiota bacterium isolate H32bin141 1 MEK6543006.1 320 aa
BacClassIA Pseudomonadati Elusimicrobiota unclassified Elusimicrobiota Elusimicrobiota bacterium isolate H41bin093 1 MEK7288137.1 319 aa
BacClassIA Pseudomonadati Elusimicrobiota unclassified Elusimicrobiota Elusimicrobiota bacterium isolate NC groundwater 1299 Ag S0.2um 52 32 MBI4370057.1 325 aa
BacClassIA Pseudomonadati Elusimicrobiota unclassified Elusimicrobiota Elusimicrobiota bacterium isolate NC groundwater 1499 Pr4 B0.1um 52 3 MBI4668709.1 312 aa
BacClassIA Pseudomonadati Elusimicrobiota unclassified Elusimicrobiota Elusimicrobiota bacterium isolate NC groundwater 232 Ag S0.2um 54 10 MBI2070448.1 333 aa
BacClassIA Pseudomonadati Elusimicrobiota unclassified Elusimicrobiota Elusimicrobiota bacterium isolate NC groundwater 1153 Ag S0.1um 52 252 MBI4063105.1 332 aa
BacClassIA Pseudomonadati Elusimicrobiota unclassified Elusimicrobiota Elusimicrobia bacterium RIFCSPLOWO2 12 FULL 59 9 OGS05389.1 293 aa
BacClassIA Pseudomonadati Elusimicrobiota unclassified Elusimicrobiota Elusimicrobiota bacterium isolate NC groundwater 1363 Ag S0.2um 60 33 MBI4249927.1 329 aa
BacClassIA Pseudomonadati Elusimicrobiota unclassified Elusimicrobiota Elusimicrobiota bacterium isolate LacPavin 0920 SED1 MAG 47 6 MCX5783021.1 319 aa
BacClassIA Pseudomonadati Elusimicrobiota unclassified Elusimicrobiota Elusimicrobiota bacterium isolate NC groundwater 1154 Ag S0.1um 58 61 MBI4055192.1 314 aa
BacClassIA Pseudomonadati Elusimicrobiota unclassified Elusimicrobiota Elusimicrobiota bacterium isolate NC groundwater 1151 Ag S0.1um 50 28 MBI4056044.1 310 aa
BacClassIA Pseudomonadati Elusimicrobiota unclassified Elusimicrobiota Elusimicrobiota bacterium isolate NC groundwater 1152 Ag S0.1um 51 25 MBI4051658.1 311 aa
BacClassIA Pseudomonadati Elusimicrobiota unclassified Elusimicrobiota Elusimicrobiota bacterium isolate SO 2017 LW4 15 MFA5138047.1 356 aa
BacClassIA Pseudomonadati Elusimicrobiota unclassified Elusimicrobiota Elusimicrobiota bacterium isolate MEGAHITgroup2.10 MFH2204717.1 320 aa
BacClassIA Pseudomonadati Elusimicrobiota unclassified Elusimicrobiota Elusimicrobiota bacterium isolate SO 2017 GW1 237 MFA6091368.1 341 aa
BacClassIA Pseudomonadati Elusimicrobiota unclassified Elusimicrobiota Elusimicrobiota bacterium isolate SO 2017 GW1 249 MFA6029758.1 338 aa
BacClassIA Pseudomonadati Elusimicrobiota unclassified Elusimicrobiota Elusimicrobiota bacterium isolate NC groundwater 1674 Pr3 B0.1um 65 18 MBI5243535.1 330 aa
BacClassIA Pseudomonadati Elusimicrobiota unclassified Elusimicrobiota Elusimicrobiota bacterium isolate UBA9639 HAH06217.1 337 aa
BacClassIA Pseudomonadati Elusimicrobiota unclassified Elusimicrobiota Elusimicrobiota bacterium isolate MEGAHITgroup1.316 MFH1726399.1 307 aa
BacClassIA Pseudomonadati Elusimicrobiota unclassified Elusimicrobiota Elusimicrobia bacterium GWA2 69 24 OGR61547.1 311 aa
BacClassIA Pseudomonadati Elusimicrobiota unclassified Elusimicrobiota Elusimicrobiota bacterium isolate BS2bin.36 MEE8424417.1 342 aa
BacClassIA Pseudomonadati Elusimicrobiota unclassified Elusimicrobiota Elusimicrobia bacterium CG1 02 63 36 OIO10863.1 307 aa
BacClassIA Pseudomonadati Elusimicrobiota unclassified Elusimicrobiota Elusimicrobiota bacterium isolate NORP122 PCI36019.1 339 aa
BacClassIA Pseudomonadati Elusimicrobiota unclassified Elusimicrobiota Elusimicrobiota bacterium isolate X1 MaxBin.038 TPW21232.1 306 aa
BacClassIA Pseudomonadati Elusimicrobiota unclassified Elusimicrobiota Elusimicrobiota bacterium isolate REEB286 MDE2292893.1 338 aa
BacClassIA Pseudomonadati Elusimicrobiota unclassified Elusimicrobiota Elusimicrobiota bacterium isolate NC groundwater 827 Pr1 B0.1um 69 30 MBI3297963.1 340 aa
BacClassIA Pseudomonadati Elusimicrobiota unclassified Elusimicrobiota Elusimicrobiota bacterium isolate NC groundwater 367 Ag B0.1um 71 7 MBI2361931.1 338 aa
BacClassIA Pseudomonadati Elusimicrobiota unclassified Elusimicrobiota Elusimicrobiota bacterium isolate NC groundwater 930 Pr1 S0.2um 66 35 MBI3549784.1 316 aa
BacClassIA Pseudomonadati Elusimicrobiota unclassified Elusimicrobiota Elusimicrobiota bacterium isolate NC groundwater 1303 Ag S0.2um 63 32 MBI4386412.1 307 aa
BacClassIA Pseudomonadati Elusimicrobiota Elusimicrobiota bacterium isolate NC groundwater 1677 Pr3 B 0.1um 68 8 MBI5200654.1 ClassIV 307 aa
BacClassIA Pseudomonadati Elusimicrobiota Elusimicrobiota bacterium isolate NC groundwater 1305 Ag S 0.2um 70 34 MBI4349385.1 ClassIV 307 aa
BacClassIA Pseudomonadati Elusimicrobiota unclassified Elusimicrobiota Elusimicrobiota bacterium isolate NC groundwater 1306 Ag S0.2um 71 33 MBI4422221.1 307 aa
BacClassIA Pseudomonadati Elusimicrobiota unclassified Elusimicrobiota Elusimicrobiota bacterium isolate LacPavin 0818 WC55 MAG 67 8 MCX5788515.1 307 aa
BacClassIA Pseudomonadati Elusimicrobiota unclassified Elusimicrobiota Elusimicrobiota bacterium isolate NC groundwater 929 Pr1 S0.2um 66 27 MBI3554284.1 327 aa
BacClassIA Pseudomonadati Elusimicrobiota unclassified Elusimicrobiota Elusimicrobia bacterium CG11 big fil rev 8 21 14 0 20 64 6 PIR15326.1 328 aa
BacClassIA Pseudomonadati Elusimicrobiota unclassified Elusimicrobiota Elusimicrobiota bacterium isolate bins.479 MDX6770580.1 344 aa
BacClassIA Pseudomonadati Elusimicrobiota unclassified Elusimicrobiota Elusimicrobiota bacterium isolate REEB145 MDE1975661.1 328 aa
BacClassIA Pseudomonadati Elusimicrobiota unclassified Elusimicrobiota Elusimicrobiota bacterium isolate NC groundwater 1673 Pr3 B0.1um 62 22 MBI5631530.1 325 aa
BacClassIA Pseudomonadati Elusimicrobiota unclassified Elusimicrobiota Elusimicrobiota bacterium isolate NC groundwater 1301 Ag S0.2um 62 34 MBI4375756.1 327 aa
BacClassIA Pseudomonadati Elusimicrobiota unclassified Elusimicrobiota Elusimicrobiota bacterium isolate NC groundwater 1679 Pr3 B0.1um 69 72 MBI5209569.1 328 aa
BacClassIA Pseudomonadati Elusimicrobiota unclassified Elusimicrobiota Elusimicrobiota bacterium isolate NC groundwater 1676 Pr3 B0.1um 68 27 MBI5623463.1 327 aa
BacClassIA Pseudomonadati Elusimicrobiota unclassified Elusimicrobiota Elusimicrobiota bacterium isolate NC groundwater 1500 Pr4 B0.1um 67 5 MBI4677208.1 311 aa
BacClassIA Pseudomonadati Elusimicrobiota unclassified Elusimicrobiota Elusimicrobiota bacterium isolate NC groundwater 1500 Pr4 B0.1um 67 5 MBI4678172.1 311 aa
BacClassIA Pseudomonadati Elusimicrobiota unclassified Elusimicrobiota Elusimicrobiota bacterium isolate H51bin276 1 MEK7859128.1 317 aa
BacClassIA Pseudomonadati Elusimicrobiota unclassified Elusimicrobiota Elusimicrobiota bacterium isolate STD1 184 MDD5627589.1 327 aa
BacClassIA Pseudomonadati Elusimicrobiota unclassified Elusimicrobiota Elusimicrobiota bacterium isolate SO 2017 GW1 264 MFA6003365.1 328 aa
BacClassIA Pseudomonadati Elusimicrobiota unclassified Elusimicrobiota Elusimicrobiota bacterium isolate STD1 166 MDD5657753.1 327 aa
BacClassIA Pseudomonadati Elusimicrobiota unclassified Elusimicrobiota Elusimicrobiota bacterium isolate UBA9628 HAM34852.1 327 aa
BacClassIA Pseudomonadati Pseudomonadota Gammaproteobacteria Natronospirales Natronospira proteinivora WP 253447676.1 318 aa 318 aa
BacClassIA Pseudomonadati Pseudomonadota Gammaproteobacteria Chromatiales Wenzhouxiangella sp WP 165281238.1 312 aa 312 aa
BacClassIA Pseudomonadati Pseudomonadota Gammaproteobacteria Woeseiales Lentisalinibacter WP 405223802.1 312 aa 312 aa
BacClassIA Pseudomonadati Pseudomonadota Gammaproteobacteria Woeseiales Woeseia sp MFQ5546645.1 317 aa 317 aa
BacClassIA Pseudomonadati Pseudomonadota Gammaproteobacteria Woeseiales Woeseia oceani WP 068619312.1 312 aa 312 aa
BacClassIA Pseudomonadati Pseudomonadota Gammaproteobacteria Steroidobacterales Steroidobacteraceae bacterium MBF8291367.1 317 aa 317 aa
BacClassIA Pseudomonadati Pseudomonadota Gammaproteobacteria Steroidobacterales Steroidobacteraceae bacterium MGH8188955.1 317 aa 317 aa
BacClassIA Pseudomonadati Pseudomonadota Gammaproteobacteria Steroidobacterales Steroidobacter sp WP 322111013.1 312 aa 312 aa
BacClassIA Pseudomonadati Pseudomonadota Gammaproteobacteria Lysobacterales Lysobacteraceae bacterium NLG74948.1 312 aa 312 aa
BacClassIA Pseudomonadati Pseudomonadota Gammaproteobacteria Steroidobacterales Steroidobacteraceae bacterium MGC4028560.1 317 aa 317 aa
BacClassIA Pseudomonadati Pseudomonadota Gammaproteobacteria Steroidobacterales Steroidobacteraceae bacterium MET0291340.1 317 aa 317 aa
BacClassIA Pseudomonadati Pseudomonadota Gammaproteobacteria Steroidobacterales Steroidobacteraceae bacterium HWG77290.1 318 aa 318 aa
BacClassIA Pseudomonadati Pseudomonadota Gammaproteobacteria Steroidobacterales Steroidobacteraceae bacterium MGB8694225.1 312 aa 312 aa
BacClassIA Pseudomonadati Pseudomonadota Gammaproteobacteria Steroidobacterales Steroidobacteraceae bacterium MFO1427430.1 312 aa 312 aa
BacClassIA Pseudomonadati Pseudomonadota Gammaproteobacteria Steroidobacterales Steroidobacteraceae bacterium HXR92729.1 312 aa 312 aa
BacClassIA Pseudomonadati Pseudomonadota Gammaproteobacteria Steroidobacterales Steroidobacteraceae bacterium HPF26882.1 318 aa 318 aa

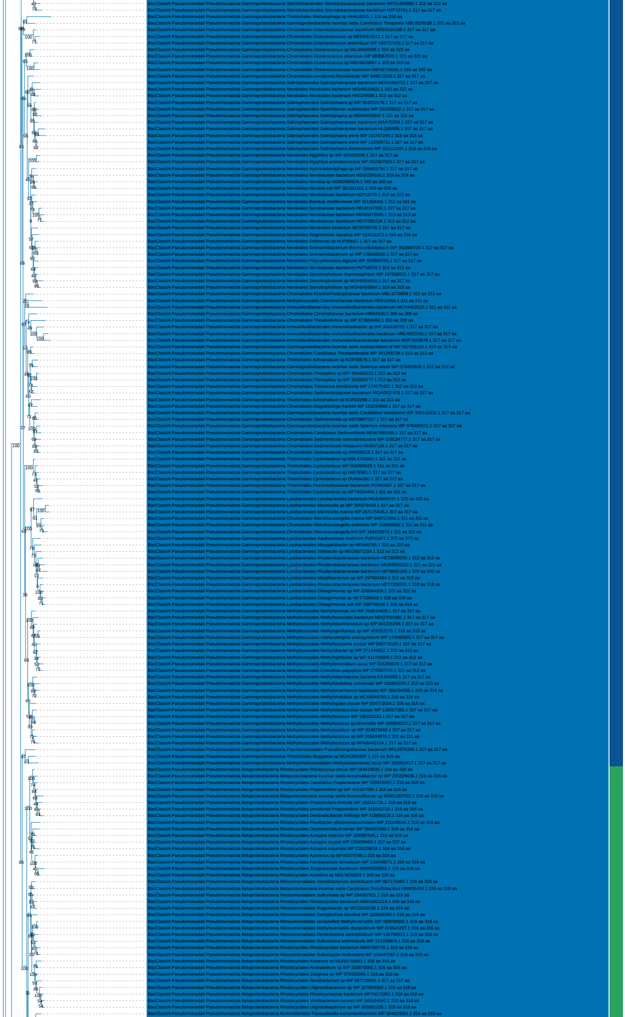
BacClassA Pseudomonadati Pseudomonadota Gammaproteobacteria Steroidobacterales Steroidobacteraceae bacterium MFO1455885.1 312 aa 312 aa
BacClassA Pseudomonadati Pseudomonadota Gammaproteobacteria Steroidobacterales Steroidobacteraceae bacterium HZF16761.1 317 aa 317 aa
BacClassA Pseudomonadati Pseudomonadota Gammaproteobacteria Thiotrichales Methylophaga sp HHA18315.1 318 aa 318 aa
BacClassA Pseudomonadati Pseudomonadota Gammaproteobacteria Gammaproteobacteria incertae sedis Candidatus Thiopontia MBC8520185.1 321 aa 321 aa
BacClassA Pseudomonadati Pseudomonadota Gammaproteobacteria Chromatiales Granulosicoccaceae bacterium MEE9334368.1 317 aa 317 aa
BacClassA Pseudomonadati Pseudomonadota Gammaproteobacteria Chromatiales Granulosicoccus sp MEE9319072.1 317 aa 317 aa
BacClassA Pseudomonadati Pseudomonadota Gammaproteobacteria Chromatiales Granulosicoccus antarcticus WP 169727439.1 317 aa 317 aa
BacClassA Pseudomonadati Pseudomonadota Gammaproteobacteria Chromatiales Oceanococcus sp MGJ8668338.1 322 aa 322 aa
BacClassA Pseudomonadati Pseudomonadota Gammaproteobacteria Chromatiales Oceanococcus atlanticus WP 083563508.1 321 aa 321 aa
BacClassA Pseudomonadati Pseudomonadota Gammaproteobacteria Chromatiales Oceanococcus sp MBV6629867.1 310 aa 310 aa
BacClassA Pseudomonadati Pseudomonadota Gammaproteobacteria Chromatiales Oceanococcaceae bacterium MEN8719581.1 292 aa 292 aa
BacClassA Pseudomonadati Pseudomonadota Gammaproteobacteria Chromatiales uncultured Abyssibacter WP 348673299.1 317 aa 317 aa
BacClassA Pseudomonadati Pseudomonadota Gammaproteobacteria Salinisphaerales Salinisphaeraceae bacterium MDX1496753.1 327 aa 327 aa
BacClassA Pseudomonadati Pseudomonadota Gammaproteobacteria Nevskiales Nevskiales bacterium MGH8529822.1 322 aa 322 aa
BacClassA Pseudomonadati Pseudomonadota Gammaproteobacteria Nevskiales Nevskiales bacterium HXG29939.1 312 aa 312 aa
BacClassA Pseudomonadati Pseudomonadota Gammaproteobacteria Salinisphaerales Salinisphaera sp WP 353252176.1 317 aa 317 aa
BacClassA Pseudomonadati Pseudomonadota Gammaproteobacteria Salinisphaerales Spectribacter acetivorans WP 311659832.1 317 aa 317 aa
BacClassA Pseudomonadati Pseudomonadota Gammaproteobacteria Salinisphaerales Salinisphaera sp MDN5938847.1 311 aa 311 aa
BacClassA Pseudomonadati Pseudomonadota Gammaproteobacteria Salinisphaerales Salinisphaeraceae bacterium MAA75934.1 317 aa 317 aa
BacClassA Pseudomonadati Pseudomonadota Gammaproteobacteria Salinisphaerales Salinisphaeraceae bacterium HLQ85995.1 317 aa 317 aa
BacClassA Pseudomonadati Pseudomonadota Gammaproteobacteria Salinisphaerales Salinisphaera orenii WP 111747299.1 318 aa 318 aa
BacClassA Pseudomonadati Pseudomonadota Gammaproteobacteria Salinisphaerales Salinisphaera orenii WP 123589711.1 317 aa 317 aa
BacClassA Pseudomonadati Pseudomonadota Gammaproteobacteria Salinisphaerales Salinisphaera dokdonensis WP 353111097.1 318 aa 318 aa
BacClassA Pseudomonadati Pseudomonadota Gammaproteobacteria Nevskiales Algiphilus sp WP 420426390.1 317 aa 317 aa
BacClassA Pseudomonadati Pseudomonadota Gammaproteobacteria Nevskiales Algiphilus aromaticivorans WP 052367563.1 317 aa 317 aa
BacClassA Pseudomonadati Pseudomonadota Gammaproteobacteria Nevskiales Hydrocarboniphaga sp WP 299693794.1 317 aa 317 aa
BacClassA Pseudomonadati Pseudomonadota Gammaproteobacteria Nevskiales Nevskiaceae bacterium MDI3259100.1 319 aa 319 aa
BacClassA Pseudomonadati Pseudomonadota Gammaproteobacteria Nevskiales Nevskia sp MDB5986829.1 340 aa 340 aa
BacClassA Pseudomonadati Pseudomonadota Gammaproteobacteria Nevskiales Nevskia soli WP 301331501.1 329 aa 329 aa
BacClassA Pseudomonadati Pseudomonadota Gammaproteobacteria Nevskiales Nevskiaceae bacterium HZP11078.1 312 aa 312 aa
BacClassA Pseudomonadati Pseudomonadota Gammaproteobacteria Nevskiales Banduia mediterranea WP 311364411.1 311 aa 311 aa
BacClassA Pseudomonadati Pseudomonadota Gammaproteobacteria Nevskiales Nevskiaceae bacterium HEU0197690.1 317 aa 317 aa
BacClassA Pseudomonadati Pseudomonadota Gammaproteobacteria Nevskiales Nevskiaceae bacterium MDN5873565.1 313 aa 313 aa
BacClassA Pseudomonadati Pseudomonadota Gammaproteobacteria Nevskiales Nevskiaceae bacterium HEX7382219.1 312 aa 312 aa
BacClassA Pseudomonadati Pseudomonadota Gammaproteobacteria Nevskiales Nevskiales bacterium MCI0749740.1 317 aa 317 aa
BacClassA Pseudomonadati Pseudomonadota Gammaproteobacteria Nevskiales Stagnimonas aquatica WP 123211372.1 316 aa 316 aa
BacClassA Pseudomonadati Pseudomonadota Gammaproteobacteria Nevskiales Solimonas sp HUP90667.1 317 aa 317 aa
BacClassA Pseudomonadati Pseudomonadota Gammaproteobacteria Nevskiales Sinimarinibacterium thermocellulolyticum WP 352889729.1 317 aa 317 aa
BacClassA Pseudomonadati Pseudomonadota Gammaproteobacteria Nevskiales Sinimarinibacterium sp WP 136848090.1 317 aa 317 aa
BacClassA Pseudomonadati Pseudomonadota Gammaproteobacteria Nevskiales Polycyclovorans algicola WP 029890786.1 317 aa 317 aa
BacClassA Pseudomonadati Pseudomonadota Gammaproteobacteria Nevskiales Nevskiaceae bacterium HVT34370.1 312 aa 312 aa
BacClassA Pseudomonadati Pseudomonadota Gammaproteobacteria Nevskiales Stenotrophobium rhamnosiphilum WP 107938531.1 317 aa 317 aa
BacClassA Pseudomonadati Pseudomonadota Gammaproteobacteria Nevskiales Stenotrophobium sp MGH8504930.1 317 aa 317 aa
BacClassA Pseudomonadati Pseudomonadota Gammaproteobacteria Nevskiales Stenotrophobium sp MGH8456894.1 318 aa 318 aa
BacClassA Pseudomonadati Pseudomonadota Gammaproteobacteria Chromatiales Ectothiorhodospiraceae bacterium MBL1276808.1 312 aa 312 aa
BacClassA Pseudomonadati Pseudomonadota Gammaproteobacteria Methylococcales Crenotrichaceae bacterium HFD10950.1 311 aa 311 aa
BacClassA Pseudomonadati Pseudomonadota Gammaproteobacteria Immundisolibacterales Immundisolibacterales bacterium MCY4453515.1 311 aa 311 aa
BacClassA Pseudomonadati Pseudomonadota Gammaproteobacteria Chromatiales Chromatiaceae bacterium HIB84240.1 306 aa 306 aa
BacClassA Pseudomonadati Pseudomonadota Gammaproteobacteria Chromatiales Thioalkalivibrio sp WP 373604464.1 350 aa 350 aa
BacClassA Pseudomonadati Pseudomonadota Gammaproteobacteria Immundisolibacterales Immundisolibacter sp WP 300126785.1 317 aa 317 aa
BacClassA Pseudomonadati Pseudomonadota Gammaproteobacteria Immundisolibacterales Immundisolibacterales bacterium MBL4622590.1 317 aa 317 aa
BacClassA Pseudomonadati Pseudomonadota Gammaproteobacteria Immundisolibacterales Immundisolibacteraceae bacterium MDF1819676.1 317 aa 317 aa
BacClassA Pseudomonadati Pseudomonadota Gammaproteobacteria Gammaproteobacteria incertae sedis endosymbiont of WP 057956152.1 319 aa 319 aa
BacClassA Pseudomonadati Pseudomonadota Gammaproteobacteria Chromatiales Candidatus Thiodiazotropha WP 201258728.1 312 aa 312 aa
BacClassA Pseudomonadati Pseudomonadota Gammaproteobacteria Thiotrichales Achromatium sp KOR30876.1 317 aa 317 aa
BacClassA Pseudomonadati Pseudomonadota Gammaproteobacteria Gammaproteobacteria incertae sedis Solemya velum WP 078460848.1 312 aa 312 aa
BacClassA Pseudomonadati Pseudomonadota Gammaproteobacteria Chromatiales Thiolapillus sp WP 456408222.1 312 aa 312 aa
BacClassA Pseudomonadati Pseudomonadota Gammaproteobacteria Chromatiales Thiolapillus sp WP 305800177.1 312 aa 312 aa
BacClassA Pseudomonadati Pseudomonadota Gammaproteobacteria Chromatiales Thiosocius teredinicola WP 174575421.1 312 aa 312 aa
BacClassA Pseudomonadati Pseudomonadota Gammaproteobacteria Chromatiales Sedimenticolaceae bacterium MGA0937478.1 317 aa 317 aa
BacClassA Pseudomonadati Pseudomonadota Gammaproteobacteria Thiotrichales Achromatium sp KOR32096.1 311 aa 311 aa
BacClassA Pseudomonadati Pseudomonadota Gammaproteobacteria Chromatiales Magnetovirga frankeli WP 153294868.1 317 aa 317 aa
BacClassA Pseudomonadati Pseudomonadota Gammaproteobacteria Gammaproteobacteria incertae sedis Candidatus Vondammii WP 320416332.1 317 aa 317 aa
BacClassA Pseudomonadati Pseudomonadota Gammaproteobacteria Chromatiales Sedimenticola sp MES9857537.1 317 aa 317 aa
BacClassA Pseudomonadati Pseudomonadota Gammaproteobacteria Gammaproteobacteria incertae sedis Solemya velesiana WP 078485571.1 317 aa 317 aa
BacClassA Pseudomonadati Pseudomonadota Gammaproteobacteria Chromatiales Candidatus Sedimenticola MEW7980195.1 317 aa 317 aa
BacClassA Pseudomonadati Pseudomonadota Gammaproteobacteria Chromatiales Sedimenticola selenatireducens WP 029134777.1 317 aa 317 aa
BacClassA Pseudomonadati Pseudomonadota Gammaproteobacteria Chromatiales Sedimenticola thiotaurini HEB97129.1 317 aa 317 aa
BacClassA Pseudomonadati Pseudomonadota Gammaproteobacteria Chromatiales Sedimenticola sp HHH39115.1 317 aa 317 aa
BacClassA Pseudomonadati Pseudomonadota Gammaproteobacteria Thiotrichales Cycloclasticus sp MBL4743992.1 311 aa 311 aa
BacClassA Pseudomonadati Pseudomonadota Gammaproteobacteria Thiotrichales Cycloclasticus WP 016390629.1 311 aa 311 aa
BacClassA Pseudomonadati Pseudomonadota Gammaproteobacteria Thiotrichales Cycloclasticus sp HIG78581.1 317 aa 317 aa
BacClassA Pseudomonadati Pseudomonadota Gammaproteobacteria Thiotrichales Cycloclasticus sp OUR84350.1 317 aa 317 aa
BacClassA Pseudomonadati Pseudomonadota Gammaproteobacteria Thiotrichales Piscirickettsiaceae bacterium PCH83567.1 317 aa 317 aa
BacClassA Pseudomonadati Pseudomonadota Gammaproteobacteria Thiotrichales Cycloclasticus sp MFT6624464.1 311 aa 311 aa
BacClassA Pseudomonadati Pseudomonadota Gammaproteobacteria Lysobacterales Lysobacteraceae bacterium MGE4069747.1 325 aa 325 aa
BacClassA Pseudomonadati Pseudomonadota Gammaproteobacteria Lysobacterales Marinicella sp WP 395374419.1 317 aa 317 aa
BacClassA Pseudomonadati Pseudomonadota Gammaproteobacteria Lysobacterales Marinicella marina WP 267175445.1 317 aa 317 aa
BacClassA Pseudomonadati Pseudomonadota Gammaproteobacteria Chromatiales Wenzhouxiangella marina WP 049727004.1 311 aa 311 aa
BacClassA Pseudomonadati Pseudomonadota Gammaproteobacteria Chromatiales Wenzhouxiangella sediminis WP 116649502.1 311 aa 311 aa
BacClassA Pseudomonadati Pseudomonadota Gammaproteobacteria Chromatiales Wenzhouxiangella limi WP 164210270.1 311 aa 311 aa
BacClassA Pseudomonadati Pseudomonadota Gammaproteobacteria Lysobacterales Xanthomonas hortorum PUF01471.1 372 aa 372 aa
BacClassA Pseudomonadati Pseudomonadota Gammaproteobacteria Lysobacterales Mizugakiibacter sp HEN46795.1 320 aa 320 aa
BacClassA Pseudomonadati Pseudomonadota Gammaproteobacteria Lysobacterales Tahibacter sp MEO8671324.1 312 aa 312 aa
BacClassA Pseudomonadati Pseudomonadota Gammaproteobacteria Lysobacterales Rhodanobacteraceae bacterium HET8899205.1 312 aa 312 aa
BacClassA Pseudomonadati Pseudomonadota Gammaproteobacteria Lysobacterales Rhodanobacteraceae bacterium MGB8634303.1 321 aa 321 aa
BacClassA Pseudomonadati Pseudomonadota Gammaproteobacteria Lysobacterales Rhodanobacteraceae bacterium HET6631309.1 320 aa 320 aa
BacClassA Pseudomonadati Pseudomonadota Gammaproteobacteria Lysobacterales Metallibacterium sp WP 297904264.1 312 aa 312 aa
BacClassA Pseudomonadati Pseudomonadota Gammaproteobacteria Lysobacterales Rhodanobacteraceae bacterium HET7358591.1 318 aa 318 aa
BacClassA Pseudomonadati Pseudomonadota Gammaproteobacteria Lysobacterales Oleiagrimonas sp WP 206064329.1 322 aa 322 aa
BacClassA Pseudomonadati Pseudomonadota Gammaproteobacteria Lysobacterales Oleiagrimonas sp HET7299428.1 328 aa 328 aa
BacClassA Pseudomonadati Pseudomonadota Gammaproteobacteria Lysobacterales Oleiagrimonas soli WP 268746046.1 318 aa 318 aa
BacClassA Pseudomonadati Pseudomonadota Gammaproteobacteria Methylococcales Methylomonas rivi WP 256614409.1 317 aa 317 aa
BacClassA Pseudomonadati Pseudomonadota Gammaproteobacteria Methylococcales Methylococcales bacterium MDQ7091881.1 317 aa 317 aa
BacClassA Pseudomonadati Pseudomonadota Gammaproteobacteria Methylococcales Methylotuvimicrobium sp WP 341326296.1 317 aa 317 aa
BacClassA Pseudomonadati Pseudomonadota Gammaproteobacteria Methylococcales Methyloprofundus sp WP 428353176.1 318 aa 318 aa
BacClassA Pseudomonadati Pseudomonadota Gammaproteobacteria Methylococcales methanotrophic endosymbiont WP 174482805.1 317 aa 317 aa
BacClassA Pseudomonadati Pseudomonadota Gammaproteobacteria Methylococcales Methylocucumis oryzae WP 045778185.1 317 aa 317 aa
BacClassA Pseudomonadati Pseudomonadota Gammaproteobacteria Methylococcales Methylobacter sp WP 371744921.1 312 aa 312 aa
BacClassA Pseudomonadati Pseudomonadota Gammaproteobacteria Methylococcales Methyloglobulus sp WP 411726599.1 312 aa 312 aa
BacClassA Pseudomonadati Pseudomonadota Gammaproteobacteria Methylococcales Methylomicrobium lacus WP 024298670.1 317 aa 317 aa
BacClassA Pseudomonadati Pseudomonadota Gammaproteobacteria Methylococcales Crenothrix polyspora WP 272867715.1 312 aa 312 aa
BacClassA Pseudomonadati Pseudomonadota Gammaproteobacteria Methylococcales Methylothermaceae bacteria KXJ41693.1 317 aa 317 aa
BacClassA Pseudomonadati Pseudomonadota Gammaproteobacteria Methylococcales Methylohalobius crimeensis WP 200861559.1 310 aa 310 aa
BacClassA Pseudomonadati Pseudomonadota Gammaproteobacteria Methylococcales Methylomarinovum tepidoasis WP 286294056.1 316 aa 316 aa
BacClassA Pseudomonadati Pseudomonadota Gammaproteobacteria Methylococcales Methylohalobius sp MCX8049700.1 316 aa 316 aa
BacClassA Pseudomonadati Pseudomonadota Gammaproteobacteria Methylococcales Methylogaea oryzae WP 054773534.1 316 aa 316 aa
BacClassA Pseudomonadati Pseudomonadota Gammaproteobacteria Methylococcales Methylotetracoccus oryzae WP 139557385.1 317 aa 317 aa
BacClassA Pseudomonadati Pseudomonadota Gammaproteobacteria Methylococcales Methylococcus WP 196323333.1 317 aa 317 aa
BacClassA Pseudomonadati Pseudomonadota Gammaproteobacteria Methylococcales Methylococcus geothermalis WP 169604023.1 317 aa 317 aa
BacClassA Pseudomonadati Pseudomonadota Gammaproteobacteria Methylococcales Methylocaldum sp WP 324873492.1 317 aa 317 aa
BacClassA Pseudomonadati Pseudomonadota Gammaproteobacteria Methylococcales Methylococcus sp WP 205434676.1 311 aa 311 aa
BacClassA Pseudomonadati Pseudomonadota Gammaproteobacteria Methylococcales Methylococcus sp MFM8443124.1 317 aa 317 aa
BacClassA Pseudomonadati Pseudomonadota Gammaproteobacteria Pseudomonadales Pseudohongiellaceae bacterium MFL2875395.1 317 aa 317 aa
BacClassA Pseudomonadati Pseudomonadota Gammaproteobacteria Thiotrichales Beggiatoa sp MCA1853437.1 315 aa 315 aa
BacClassA Pseudomonadati Pseudomonadota Gammaproteobacteria Methylohalomonadales Methylohalomonas lacus WP 259053417.1 317 aa 317 aa
BacClassA Pseudomonadati Pseudomonadota Betaproteobacteria Rhodocyclales Rhodocyclus tenuis WP 184414936.1 310 aa 310 aa
BacClassA Pseudomonadati Pseudomonadota Betaproteobacteria Betaproteobacteria incertae sedis Accumulibacter sp WP 295329436.1 316 aa 316 aa
BacClassA Pseudomonadati Pseudomonadota Betaproteobacteria Rhodocyclales Candidatus Propionivibrio WP 186410430.1 316 aa 316 aa
BacClassA Pseudomonadati Pseudomonadota Betaproteobacteria Rhodocyclales Propionivibrio sp WP 415167595.1 316 aa 316 aa
BacClassA Pseudomonadati Pseudomonadota Betaproteobacteria Betaproteobacteria incertae sedis Accumulibacter sp MDR1367582.1 316 aa 316 aa
BacClassA Pseudomonadati Pseudomonadota Betaproteobacteria Rhodocyclales Propionivibrio limicola WP 153111731.1 316 aa 316 aa
BacClassA Pseudomonadati Pseudomonadota Betaproteobacteria Rhodocyclales uncultured Propionivibrio WP 319242710.1 316 aa 316 aa
BacClassA Pseudomonadati Pseudomonadota Betaproteobacteria Rhodocyclales Dentiradicibacter hellwigii WP 418890110.1 316 aa 316 aa
BacClassA Pseudomonadati Pseudomonadota Betaproteobacteria Rhodocyclales Fluviibacter phosphoraccumulans WP 211149044.1 316 aa 316 aa
BacClassA Pseudomonadati Pseudomonadota Betaproteobacteria Rhodocyclales Oryzomicrobium terrae WP 054621930.1 318 aa 318 aa
BacClassA Pseudomonadati Pseudomonadota Betaproteobacteria Rhodocyclales Azospira restricta WP 203387545.1 316 aa 316 aa
BacClassA Pseudomonadati Pseudomonadota Betaproteobacteria Rhodocyclales Azospira oryzae WP 130459489.1 317 aa 317 aa
BacClassA Pseudomonadati Pseudomonadota Betaproteobacteria Rhodocyclales Azospira inquinata WP 216129618.1 316 aa 316 aa
BacClassA Pseudomonadati Pseudomonadota Betaproteobacteria Rhodocyclales Azonexus sp MFV0370786.1 316 aa 316 aa
BacClassA Pseudomonadati Pseudomonadota Betaproteobacteria Rhodocyclales Ferribacterium limneticum WP 226448571.1 316 aa 316 aa
BacClassA Pseudomonadati Pseudomonadota Betaproteobacteria Rhodocyclales Zoogloeaceae bacterium MDR0528858.1 316 aa 316 aa
BacClassA Pseudomonadati Pseudomonadota Betaproteobacteria Rhodocyclales Azovibrio sp MGL5630829.1 316 aa 316 aa
BacClassA Pseudomonadati Pseudomonadota Betaproteobacteria Nitrosomonadales Sterolibacterium denitrificans WP 067170962.1 316 aa 316 aa
BacClassA Pseudomonadati Pseudomonadota Betaproteobacteria Betaproteobacteria incertae sedis Candidatus Desulfobacillus HMM55493.1 316 aa 316 aa
BacClassA Pseudomonadati Pseudomonadota Betaproteobacteria Nitrosomonadales Sulfuritalea sp WP 294987821.1 316 aa 316 aa
BacClassA Pseudomonadati Pseudomonadota Betaproteobacteria Rhodocyclales Rhodocyclales bacterium MBH1962218.1 316 aa 316 aa
BacClassA Pseudomonadati Pseudomonadota Betaproteobacteria Nitrosomonadales Rugosibacter sp MFZ2629195.1 316 aa 316 aa
BacClassA Pseudomonadati Pseudomonadota Betaproteobacteria Nitrosomonadales Georgfuchsia toluolica WP 220636380.1 316 aa 316 aa
BacClassA Pseudomonadati Pseudomonadota Betaproteobacteria Nitrosomonadales unclassified Methyloversatilis WP 069039592.1 316 aa 316 aa
BacClassA Pseudomonadati Pseudomonadota Betaproteobacteria Nitrosomonadales Methyloversatilis discipulorum WP 419541197.1 316 aa 316 aa
BacClassA Pseudomonadati Pseudomonadota Betaproteobacteria Nitrosomonadales Denitratisoma oestradiolicum WP 145769512.1 316 aa 316 aa
BacClassA Pseudomonadati Pseudomonadota Betaproteobacteria Nitrosomonadales Sulfurisoma sediminicola WP 121239874.1 316 aa 316 aa
BacClassA Pseudomonadati Pseudomonadota Betaproteobacteria Rhodocyclales Rhodocyclales bacterium MBI5785778.1 316 aa 316 aa
BacClassA Pseudomonadati Pseudomonadota Betaproteobacteria Nitrosomonadales Sulfuricystis multivorans WP 126447087.1 316 aa 316 aa
BacClassA Pseudomonadati Pseudomonadota Betaproteobacteria Rhodocyclales Azoarcus sp MDR0716693.1 316 aa 316 aa
BacClassA Pseudomonadati Pseudomonadota Betaproteobacteria Rhodocyclales Aromatoleum sp WP 332670082.1 316 aa 316 aa
BacClassA Pseudomonadati Pseudomonadota Betaproteobacteria Rhodocyclales Zoogloea sp WP 079432586.1 316 aa 316 aa
BacClassA Pseudomonadati Pseudomonadota Betaproteobacteria Rhodocyclales Niveibacterium sp WP 267725931.1 317 aa 317 aa
BacClassA Pseudomonadati Pseudomonadota Betaproteobacteria Rhodocyclales Uliginosibacterium sp WP 327604363.1 318 aa 318 aa
BacClassA Pseudomonadati Pseudomonadota Betaproteobacteria Rhodocyclales Rhodocyclaceae bacterium MFT4173341.1 318 aa 318 aa
BacClassA Pseudomonadati Pseudomonadota Betaproteobacteria Rhodocyclales Viridibacterium curvum WP 345534040.1 318 aa 318 aa
BacClassA Pseudomonadati Pseudomonadota Betaproteobacteria Rhodocyclales Uliginosibacterium sp WP 305981235.1 318 aa 318 aa
BacClassA Pseudomonadati Pseudomonadota Betaproteobacteria Burkholderiales Parasutterella excrementihominis WP 304820081.1 316 aa 316 aa

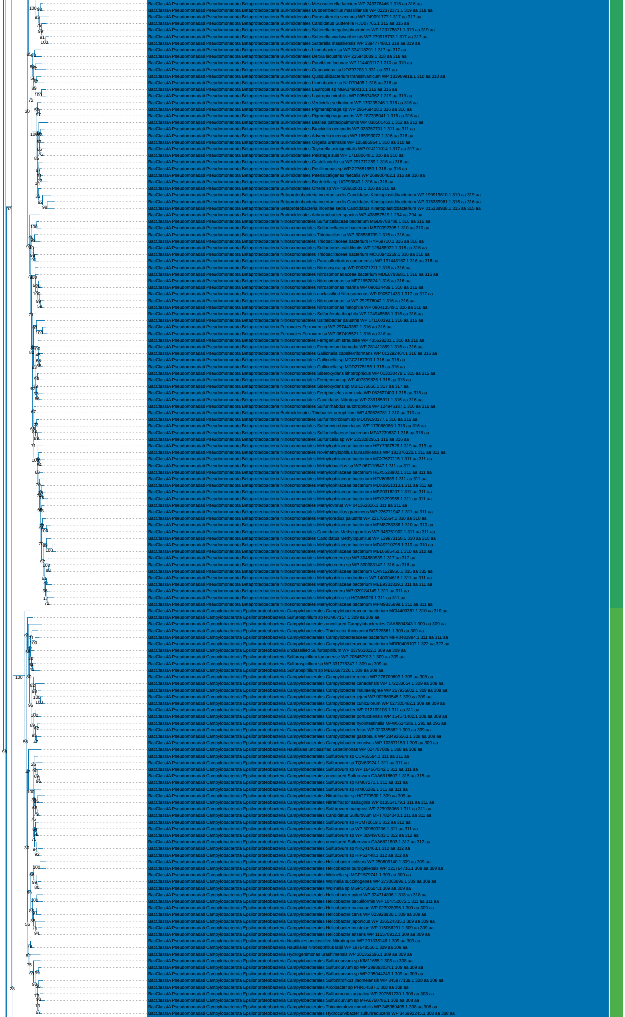
BacClassIA Pseudomonadati Pseudomonadota Betaproteobacteria Burkholderiales Mesosutterella faecium WP 243376446.1 316 aa 316 aa
BacClassIA Pseudomonadati Pseudomonadota Betaproteobacteria Burkholderiales Duodenibacillus massiliensis WP 022372371.1 319 aa 319 aa
BacClassIA Pseudomonadati Pseudomonadota Betaproteobacteria Burkholderiales Parasutterella secunda WP 249091777.1 317 aa 317 aa
BacClassIA Pseudomonadati Pseudomonadota Betaproteobacteria Burkholderiales Candidatus Sutterella HJD07765.1 315 aa 315 aa
BacClassIA Pseudomonadati Pseudomonadota Betaproteobacteria Burkholderiales Sutterella megalosphaeroides WP 120175871.1 319 aa 319 aa
BacClassIA Pseudomonadati Pseudomonadota Betaproteobacteria Burkholderiales Sutterella wadsworthensis WP 279015793.1 317 aa 317 aa
BacClassIA Pseudomonadati Pseudomonadota Betaproteobacteria Burkholderiales Sutterella massiliensis WP 239477488.1 319 aa 319 aa
BacClassIA Pseudomonadati Pseudomonadota Betaproteobacteria Burkholderiales Limnobacter sp WP 334118291.1 317 aa 317 aa
BacClassIA Pseudomonadati Pseudomonadota Betaproteobacteria Burkholderiales Derxia lacustris WP 235840039.1 318 aa 318 aa
BacClassIA Pseudomonadati Pseudomonadota Betaproteobacteria Burkholderiales Parvibium lacunae WP 114403117.1 310 aa 310 aa
BacClassIA Pseudomonadati Pseudomonadota Betaproteobacteria Burkholderiales Cupriavidus sp UOZ97203.1 331 aa 331 aa
BacClassIA Pseudomonadati Pseudomonadota Betaproteobacteria Burkholderiales Quisquiliibacterium transsilvanicum WP 183969918.1 310 aa 310 aa
BacClassIA Pseudomonadati Pseudomonadota Betaproteobacteria Burkholderiales Limnobacter sp NLD70408.1 316 aa 316 aa
BacClassIA Pseudomonadati Pseudomonadota Betaproteobacteria Burkholderiales Lautropia sp MBA3480010.1 316 aa 316 aa
BacClassIA Pseudomonadati Pseudomonadota Betaproteobacteria Burkholderiales Lautropia mirabilis WP 005674962.1 319 aa 319 aa
BacClassIA Pseudomonadati Pseudomonadota Betaproteobacteria Burkholderiales Verticiella sediminum WP 170235246.1 316 aa 316 aa
BacClassIA Pseudomonadati Pseudomonadota Betaproteobacteria Burkholderiales Pigmentiphaga sp WP 296468428.1 316 aa 316 aa
BacClassIA Pseudomonadati Pseudomonadota Betaproteobacteria Burkholderiales Pigmentiphaga aceris WP 187395041.1 316 aa 316 aa
BacClassIA Pseudomonadati Pseudomonadota Betaproteobacteria Burkholderiales Basilea psittacipulmonis WP 038501463.1 312 aa 312 aa
BacClassIA Pseudomonadati Pseudomonadota Betaproteobacteria Burkholderiales Brackiella oedipodis WP 028357701.1 311 aa 311 aa
BacClassIA Pseudomonadati Pseudomonadota Betaproteobacteria Burkholderiales Advenella incenata WP 165393072.1 316 aa 316 aa
BacClassIA Pseudomonadati Pseudomonadota Betaproteobacteria Burkholderiales Oligella urethralis WP 105885964.1 310 aa 310 aa
BacClassIA Pseudomonadati Pseudomonadota Betaproteobacteria Burkholderiales Taylorella asinigenitalis WP 014111014.1 317 aa 317 aa
BacClassIA Pseudomonadati Pseudomonadota Betaproteobacteria Burkholderiales Pelistega suis WP 171680648.1 316 aa 316 aa
BacClassIA Pseudomonadati Pseudomonadota Betaproteobacteria Burkholderiales Castellaniella sp WP 291771259.1 316 aa 316 aa
BacClassIA Pseudomonadati Pseudomonadota Betaproteobacteria Burkholderiales Pusillimonas sp WP 227681859.1 316 aa 316 aa
BacClassIA Pseudomonadati Pseudomonadota Betaproteobacteria Burkholderiales Paenalcaligenes faecalis WP 269900462.1 316 aa 316 aa
BacClassIA Pseudomonadati Pseudomonadota Betaproteobacteria Burkholderiales Bordetella sp UOF93843.1 316 aa 316 aa
BacClassIA Pseudomonadati Pseudomonadota Betaproteobacteria Burkholderiales Orrella sp WP 420662601.1 316 aa 316 aa
BacClassIA Pseudomonadati Pseudomonadota Betaproteobacteria Betaproteobacteria incertae sedis Candidatus Kinetoplastidibacterium WP 199919610.1 319 aa 319 aa
BacClassIA Pseudomonadati Pseudomonadota Betaproteobacteria Betaproteobacteria incertae sedis Candidatus Kinetoplastidibacterium WP 015389961.1 316 aa 316 aa
BacClassIA Pseudomonadati Pseudomonadota Betaproteobacteria Betaproteobacteria incertae sedis Candidatus Kinetoplastidibacterium WP 015238938.1 315 aa 315 aa
BacClassIA Pseudomonadati Pseudomonadota Betaproteobacteria Burkholderiales Achromobacter spanius WP 438857519.1 294 aa 294 aa
BacClassIA Pseudomonadati Pseudomonadota Betaproteobacteria Nitrosomonadales Sulfuricellaceae bacterium MGD9788788.1 316 aa 316 aa
BacClassIA Pseudomonadati Pseudomonadota Betaproteobacteria Nitrosomonadales Sulfuricellaceae bacterium MBZ0092305.1 310 aa 310 aa
BacClassIA Pseudomonadati Pseudomonadota Betaproteobacteria Nitrosomonadales Thiobacillus sp WP 305536709.1 316 aa 316 aa
BacClassIA Pseudomonadati Pseudomonadota Betaproteobacteria Nitrosomonadales Thiobacillaceae bacterium HYP68710.1 316 aa 316 aa
BacClassIA Pseudomonadati Pseudomonadota Betaproteobacteria Nitrosomonadales Sulfuritortus calidifontis WP 126458922.1 316 aa 316 aa
BacClassIA Pseudomonadati Pseudomonadota Betaproteobacteria Nitrosomonadales Thiobacillaceae bacterium MCU0842259.1 316 aa 316 aa
BacClassIA Pseudomonadati Pseudomonadota Betaproteobacteria Nitrosomonadales Parasulfuritortus cantonensis WP 131448162.1 316 aa 316 aa
BacClassIA Pseudomonadati Pseudomonadota Betaproteobacteria Nitrosomonadales Nitrosospira sp WP 090371211.1 316 aa 316 aa
BacClassIA Pseudomonadati Pseudomonadota Betaproteobacteria Nitrosomonadales Nitrosomonadaceae bacterium MDE0798681.1 316 aa 316 aa
BacClassIA Pseudomonadati Pseudomonadota Betaproteobacteria Nitrosomonadales Nitrosomonas sp MFZ1852824.1 316 aa 316 aa
BacClassIA Pseudomonadati Pseudomonadota Betaproteobacteria Nitrosomonadales Nitrosomonas marina WP 090634489.1 316 aa 316 aa
BacClassIA Pseudomonadati Pseudomonadota Betaproteobacteria Nitrosomonadales unclassified Nitrosomonas WP 090571433.1 317 aa 317 aa
BacClassIA Pseudomonadati Pseudomonadota Betaproteobacteria Nitrosomonadales Nitrosomonas sp WP 292976042.1 316 aa 316 aa
BacClassIA Pseudomonadati Pseudomonadota Betaproteobacteria Nitrosomonadales Nitrosomonas halophila WP 090413549.1 316 aa 316 aa
BacClassIA Pseudomonadati Pseudomonadota Betaproteobacteria Nitrosomonadales Sulfuriferula thiophila WP 124948568.1 316 aa 316 aa
BacClassIA Pseudomonadati Pseudomonadota Betaproteobacteria Nitrosomonadales Usitatibacter palustris WP 171160393.1 316 aa 316 aa
BacClassIA Pseudomonadati Pseudomonadota Betaproteobacteria Ferrovales Ferrovum sp WP 297449382.1 316 aa 316 aa
BacClassIA Pseudomonadati Pseudomonadota Betaproteobacteria Ferrovales Ferrovum sp WP 067495521.1 316 aa 316 aa
BacClassIA Pseudomonadati Pseudomonadota Betaproteobacteria Nitrosomonadales Ferrigenium straubiae WP 435628231.1 316 aa 316 aa
BacClassIA Pseudomonadati Pseudomonadota Betaproteobacteria Nitrosomonadales Ferrigenium kumadai WP 281411869.1 316 aa 316 aa
BacClassIA Pseudomonadati Pseudomonadota Betaproteobacteria Nitrosomonadales Gallionella capsiferriformans WP 013292464.1 316 aa 316 aa
BacClassIA Pseudomonadati Pseudomonadota Betaproteobacteria Nitrosomonadales Gallionella sp MGC2167390.1 316 aa 316 aa
BacClassIA Pseudomonadati Pseudomonadota Betaproteobacteria Nitrosomonadales Gallionella sp MDD2775156.1 316 aa 316 aa
BacClassIA Pseudomonadati Pseudomonadota Betaproteobacteria Nitrosomonadales Sideroxydans lithotrophicus WP 013030479.1 315 aa 315 aa
BacClassIA Pseudomonadati Pseudomonadota Betaproteobacteria Nitrosomonadales Ferrigenium sp WP 407899826.1 315 aa 315 aa
BacClassIA Pseudomonadati Pseudomonadota Betaproteobacteria Nitrosomonadales Sideroxydans sp MBI1175656.1 317 aa 317 aa
BacClassIA Pseudomonadati Pseudomonadota Betaproteobacteria Nitrosomonadales Ferriphaselus amnicola WP 062627400.1 315 aa 315 aa
BacClassIA Pseudomonadati Pseudomonadota Betaproteobacteria Nitrosomonadales Candidatus Nitrotoga WP 239185911.1 316 aa 316 aa
BacClassIA Pseudomonadati Pseudomonadota Betaproteobacteria Nitrosomonadales Sulfurirhabdus autotrophica WP 124946187.1 316 aa 316 aa
BacClassIA Pseudomonadati Pseudomonadota Betaproteobacteria Burkholderiales Thiobacter aerophilum WP 430626781.1 310 aa 310 aa
BacClassIA Pseudomonadati Pseudomonadota Betaproteobacteria Nitrosomonadales Sulfurimicrobium sp MDO9190177.1 316 aa 316 aa
BacClassIA Pseudomonadati Pseudomonadota Betaproteobacteria Nitrosomonadales Sulfurimicrobium lacus WP 173068086.1 316 aa 316 aa
BacClassIA Pseudomonadati Pseudomonadota Betaproteobacteria Nitrosomonadales Sulfuricellaceae bacterium MFA7239637.1 316 aa 316 aa
BacClassIA Pseudomonadati Pseudomonadota Betaproteobacteria Nitrosomonadales Sulfuricella sp WP 325328295.1 316 aa 316 aa
BacClassIA Pseudomonadati Pseudomonadota Betaproteobacteria Nitrosomonadales Methylophilaceae bacterium HEY7987528.1 319 aa 319 aa
BacClassIA Pseudomonadati Pseudomonadota Betaproteobacteria Nitrosomonadales Novimethylophilus kurashikiensis WP 181376320.1 311 aa 311 aa
BacClassIA Pseudomonadati Pseudomonadota Betaproteobacteria Nitrosomonadales Methylophilaceae bacterium MCX7627115.1 311 aa 311 aa
BacClassIA Pseudomonadati Pseudomonadota Betaproteobacteria Nitrosomonadales Methylobacillus sp WP 067110547.1 311 aa 311 aa
BacClassIA Pseudomonadati Pseudomonadota Betaproteobacteria Nitrosomonadales Methylophilaceae bacterium HEX5538902.1 311 aa 311 aa
BacClassIA Pseudomonadati Pseudomonadota Betaproteobacteria Nitrosomonadales Methylophilaceae bacterium HZV60889.1 311 aa 311 aa
BacClassIA Pseudomonadati Pseudomonadota Betaproteobacteria Nitrosomonadales Methylophilaceae bacterium MDX9951013.1 311 aa 311 aa
BacClassIA Pseudomonadati Pseudomonadota Betaproteobacteria Nitrosomonadales Methylophilaceae bacterium MEZ0316207.1 311 aa 311 aa
BacClassIA Pseudomonadati Pseudomonadota Betaproteobacteria Nitrosomonadales Methylophilaceae bacterium HEY3299955.1 311 aa 311 aa
BacClassIA Pseudomonadati Pseudomonadota Betaproteobacteria Nitrosomonadales Methylovorus WP 041362816.1 311 aa 311 aa
BacClassIA Pseudomonadati Pseudomonadota Betaproteobacteria Nitrosomonadales Methylobacillus gramineus WP 226771942.1 311 aa 311 aa
BacClassIA Pseudomonadati Pseudomonadota Betaproteobacteria Nitrosomonadales Methyloradius palustris WP 221765564.1 310 aa 310 aa
BacClassIA Pseudomonadati Pseudomonadota Betaproteobacteria Nitrosomonadales Methylophilaceae bacterium MFM8758386.1 310 aa 310 aa
BacClassIA Pseudomonadati Pseudomonadota Betaproteobacteria Nitrosomonadales Candidatus Methylopumilus WP 045751902.1 311 aa 311 aa
BacClassIA Pseudomonadati Pseudomonadota Betaproteobacteria Nitrosomonadales Candidatus Methylopumilus WP 139873150.1 310 aa 310 aa
BacClassIA Pseudomonadati Pseudomonadota Betaproteobacteria Nitrosomonadales Methylophilaceae bacterium MDA9210798.1 310 aa 310 aa
BacClassIA Pseudomonadati Pseudomonadota Betaproteobacteria Nitrosomonadales Methylophilaceae bacterium MBL6685450.1 310 aa 310 aa
BacClassIA Pseudomonadati Pseudomonadota Betaproteobacteria Nitrosomonadales Methylotenera sp WP 304889939.1 317 aa 317 aa
BacClassIA Pseudomonadati Pseudomonadota Betaproteobacteria Nitrosomonadales Methylotenera sp WP 300383147.1 316 aa 316 aa
BacClassIA Pseudomonadati Pseudomonadota Betaproteobacteria Nitrosomonadales Methylophilaceae bacterium CAN1528956.1 335 aa 335 aa
BacClassIA Pseudomonadati Pseudomonadota Betaproteobacteria Nitrosomonadales Methylophilus medardicus WP 140004616.1 311 aa 311 aa
BacClassIA Pseudomonadati Pseudomonadota Betaproteobacteria Nitrosomonadales Methylophilaceae bacterium MEE9331839.1 311 aa 311 aa
BacClassIA Pseudomonadati Pseudomonadota Betaproteobacteria Nitrosomonadales Methylotenera WP 020184149.1 311 aa 311 aa
BacClassIA Pseudomonadati Pseudomonadota Betaproteobacteria Nitrosomonadales Methylophilus sp HQN66026.1 311 aa 311 aa
BacClassIA Pseudomonadati Pseudomonadota Betaproteobacteria Nitrosomonadales Methylophilaceae bacterium MFM9835899.1 311 aa 311 aa
BacClassIA Pseudomonadati Campylobacterota Epsilonproteobacteria Campylobacterales Campylobacteraceae bacterium MCI4400361.1 310 aa 310 aa
BacClassIA Pseudomonadati Campylobacterota Epsilonproteobacteria Sulfurospirillum sp RUM67157.1 309 aa 309 aa
BacClassIA Pseudomonadati Campylobacterota Epsilonproteobacteria Campylobacterales uncultured Campylobacterales CAA6804343.1 309 aa 309 aa
BacClassIA Pseudomonadati Campylobacterota Epsilonproteobacteria Campylobacterales Thiofractor thiocamini BGR28561.1 309 aa 309 aa
BacClassIA Pseudomonadati Campylobacterota Epsilonproteobacteria Campylobacterales Campylobacteraceae bacterium MFV0481994.1 311 aa 311 aa
BacClassIA Pseudomonadati Campylobacterota Epsilonproteobacteria Campylobacterales Campylobacteraceae bacterium MDR0408107.1 322 aa 322 aa
BacClassIA Pseudomonadati Campylobacterota Epsilonproteobacteria unclassified Sulfurospirillum WP 037961822.1 309 aa 309 aa
BacClassIA Pseudomonadati Campylobacterota Epsilonproteobacteria Sulfurospirillum tamanense WP 205457913.1 309 aa 309 aa
BacClassIA Pseudomonadati Campylobacterota Epsilonproteobacteria Sulfurospirillum sp WP 331775347.1 309 aa 309 aa
BacClassIA Pseudomonadati Campylobacterota Epsilonproteobacteria Sulfurospirillum sp MBL0687226.1 309 aa 309 aa
BacClassIA Pseudomonadati Campylobacterota Epsilonproteobacteria Campylobacterales Campylobacter rectus WP 276709603.1 309 aa 309 aa
BacClassIA Pseudomonadati Campylobacterota Epsilonproteobacteria Campylobacterales Campylobacter canadensis WP 172233654.1 309 aa 309 aa
BacClassIA Pseudomonadati Campylobacterota Epsilonproteobacteria Campylobacterales Campylobacter insulaenigrae WP 257936802.1 309 aa 309 aa
BacClassIA Pseudomonadati Campylobacterota Epsilonproteobacteria Campylobacterales Campylobacter jejuni WP 002860545.1 309 aa 309 aa
BacClassIA Pseudomonadati Campylobacterota Epsilonproteobacteria Campylobacterales Campylobacter cuniculorum WP 027305482.1 309 aa 309 aa
BacClassIA Pseudomonadati Campylobacterota Epsilonproteobacteria Campylobacterales Campylobacter WP 012109108.1 311 aa 311 aa
BacClassIA Pseudomonadati Campylobacterota Epsilonproteobacteria Campylobacterales Campylobacter portucalensis WP 154571402.1 309 aa 309 aa
BacClassIA Pseudomonadati Campylobacterota Epsilonproteobacteria Campylobacterales Campylobacter hyointestinalis MFW5624386.1 295 aa 295 aa
BacClassIA Pseudomonadati Campylobacterota Epsilonproteobacteria Campylobacterales Campylobacter fetus WP 023385862.1 309 aa 309 aa
BacClassIA Pseudomonadati Campylobacterota Epsilonproteobacteria Campylobacterales Campylobacter gastrosuis WP 284936563.1 308 aa 308 aa
BacClassIA Pseudomonadati Campylobacterota Epsilonproteobacteria Campylobacterales Campylobacter concisus WP 103571153.1 309 aa 309 aa
BacClassIA Pseudomonadati Campylobacterota Epsilonproteobacteria Nautiliales unclassified Lebetimonas WP 024787089.1 308 aa 308 aa
BacClassIA Pseudomonadati Campylobacterota Epsilonproteobacteria Campylobacterales Sulfurovum sp CUV65594.1 311 aa 311 aa
BacClassIA Pseudomonadati Campylobacterota Epsilonproteobacteria Campylobacterales Sulfurovum sp TQV63924.1 311 aa 311 aa
BacClassIA Pseudomonadati Campylobacterota Epsilonproteobacteria Campylobacterales Sulfurovum sp WP 164664342.1 311 aa 311 aa
BacClassIA Pseudomonadati Campylobacterota Epsilonproteobacteria Campylobacterales uncultured Sulfurovum CAA6816607.1 315 aa 315 aa
BacClassIA Pseudomonadati Campylobacterota Epsilonproteobacteria Campylobacterales Sulfurovum sp KIM07271.1 311 aa 311 aa
BacClassIA Pseudomonadati Campylobacterota Epsilonproteobacteria Campylobacterales Sulfurovum sp KIM06286.1 311 aa 311 aa
BacClassIA Pseudomonadati Campylobacterota Epsilonproteobacteria Campylobacterales Nitratifractor sp HGZ70590.1 309 aa 309 aa
BacClassIA Pseudomonadati Campylobacterota Epsilonproteobacteria Campylobacterales Nitratifractor salsuginis WP 013554179.1 311 aa 311 aa
BacClassIA Pseudomonadati Campylobacterota Epsilonproteobacteria Campylobacterales Sulfurovum mangrovi WP 229938066.1 311 aa 311 aa
BacClassIA Pseudomonadati Campylobacterota Epsilonproteobacteria Campylobacterales Candidatus Sulfurovum MFT7824340.1 311 aa 311 aa
BacClassIA Pseudomonadati Campylobacterota Epsilonproteobacteria Campylobacterales Sulfurovum sp RUM70619.1 312 aa 312 aa
BacClassIA Pseudomonadati Campylobacterota Epsilonproteobacteria Campylobacterales Sulfurovum sp WP 309500236.1 311 aa 311 aa
BacClassIA Pseudomonadati Campylobacterota Epsilonproteobacteria Campylobacterales Sulfurovum sp WP 309497603.1 312 aa 312 aa
BacClassIA Pseudomonadati Campylobacterota Epsilonproteobacteria Campylobacterales uncultured Sulfurovum CAA6821803.1 312 aa 312 aa
BacClassIA Pseudomonadati Campylobacterota Epsilonproteobacteria Campylobacterales Sulfurovum sp NKQ41463.1 312 aa 312 aa
BacClassIA Pseudomonadati Campylobacterota Epsilonproteobacteria Campylobacterales Sulfurovum sp HIP62448.1 312 aa 312 aa
BacClassIA Pseudomonadati Campylobacterota Epsilonproteobacteria Campylobacterales Helicobacter colisuis WP 250608140.1 309 aa 309 aa
BacClassIA Pseudomonadati Campylobacterota Epsilonproteobacteria Campylobacterales Helicobacter burdigaliensis WP 121764716.1 309 aa 309 aa
BacClassIA Pseudomonadati Campylobacterota Epsilonproteobacteria Campylobacterales Wolinella sp MGP1579741.1 309 aa 309 aa
BacClassIA Pseudomonadati Campylobacterota Epsilonproteobacteria Campylobacterales Wolinella succinogenes WP 273063896.1 309 aa 309 aa
BacClassIA Pseudomonadati Campylobacterota Epsilonproteobacteria Campylobacterales Wolinella sp MGP1450504.1 309 aa 309 aa
BacClassIA Pseudomonadati Campylobacterota Epsilonproteobacteria Campylobacterales Helicobacter pylori WP 324714896.1 318 aa 318 aa
BacClassIA Pseudomonadati Campylobacterota Epsilonproteobacteria Campylobacterales Helicobacter baculiformis WP 104752072.1 311 aa 311 aa
BacClassIA Pseudomonadati Campylobacterota Epsilonproteobacteria Campylobacterales Helicobacter macacae WP 023928095.1 309 aa 309 aa
BacClassIA Pseudomonadati Campylobacterota Epsilonproteobacteria Campylobacterales Helicobacter canis WP 023928930.1 309 aa 309 aa
BacClassIA Pseudomonadati Campylobacterota Epsilonproteobacteria Campylobacterales Helicobacter japonicus WP 336524335.1 309 aa 309 aa
BacClassIA Pseudomonadati Campylobacterota Epsilonproteobacteria Campylobacterales Helicobacter mustelae WP 115056291.1 309 aa 309 aa
BacClassIA Pseudomonadati Campylobacterota Epsilonproteobacteria Campylobacterales Helicobacter anseris WP 115578912.1 309 aa 309 aa
BacClassIA Pseudomonadati Campylobacterota Epsilonproteobacteria Nautiliales unclassified Nitratiruptor WP 201338148.1 309 aa 309 aa
BacClassIA Pseudomonadati Campylobacterota Epsilonproteobacteria Nautiliales Nitrosophilus labii WP 187648556.1 309 aa 309 aa
BacClassIA Pseudomonadati Campylobacterota Epsilonproteobacteria Hydrogenimonas urashimensis WP 201352596.1 309 aa 309 aa
BacClassIA Pseudomonadati Campylobacterota Epsilonproteobacteria Campylobacterales Sulfuricurvum sp KIM11655.1 308 aa 308 aa
BacClassIA Pseudomonadati Campylobacterota Epsilonproteobacteria Campylobacterales Sulfuricurvum sp WP 299893034.1 309 aa 309 aa
BacClassIA Pseudomonadati Campylobacterota Epsilonproteobacteria Campylobacterales Sulfuricurvum sp WP 295044243.1 309 aa 309 aa
BacClassIA Pseudomonadati Campylobacterota Epsilonproteobacteria Campylobacterales Sulfonitrofixus jiaomeiensis WP 345977139.1 308 aa 308 aa
BacClassIA Pseudomonadati Campylobacterota Epsilonproteobacteria Campylobacterales Arcobacter sp PHR54587.1 308 aa 308 aa
BacClassIA Pseudomonadati Campylobacterota Epsilonproteobacteria Campylobacterales Sulfurimonas aquatica WP 207561230.1 308 aa 308 aa
BacClassIA Pseudomonadati Campylobacterota Epsilonproteobacteria Campylobacterales Sulfuricurvum sp MFA6760786.1 308 aa 308 aa
BacClassIA Pseudomonadati Campylobacterota Epsilonproteobacteria Campylobacterales Thiomicrolovo immobilis WP 345969405.1 308 aa 308 aa
BacClassIA Pseudomonadati Campylobacterota Epsilonproteobacteria Campylobacterales Hydrocurvibacter sulfurireducens WP 345992245.1 308 aa 308 aa

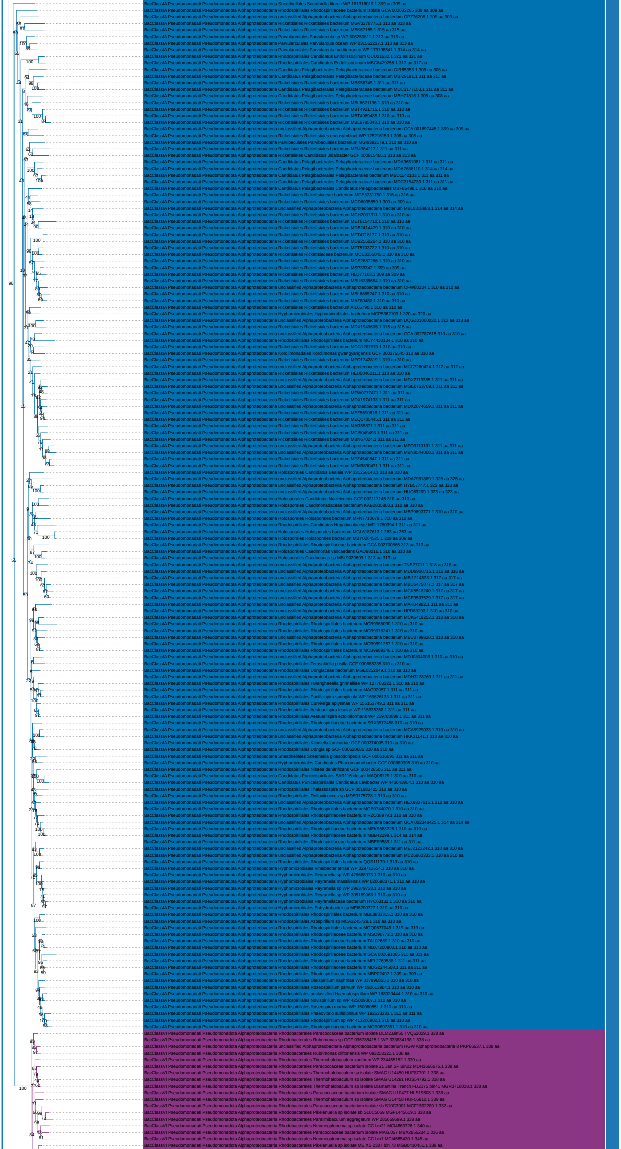

BacClassIA Pseudomonadati Pseudomonadota Alphaproteobacteria Sneathiellales Sneathiella litorea WP 161316019.1 309 aa 309 aa
BacClassIA Pseudomonadati Pseudomonadota Alphaproteobacteria Rhodospirillales Rhodospirillaceae bacterium isolate GCA 002632265 309 aa 309 aa
BacClassIA Pseudomonadati Pseudomonadota Alphaproteobacteria unclassified Alphaproteobacteria Alphaproteobacteria bacterium OPZ76208.1 309 aa 309 aa
BacClassIA Pseudomonadati Pseudomonadota Alphaproteobacteria Rickettsiales Rickettsiales bacterium MGV3278775.1 313 aa 313 aa
BacClassIA Pseudomonadati Pseudomonadota Alphaproteobacteria Rickettsiales Rickettsiales bacterium MBK67165.1 315 aa 315 aa
BacClassIA Pseudomonadati Pseudomonadota Alphaproteobacteria Parvularculales Parvularcula sp WP 306253811.1 313 aa 313 aa
BacClassIA Pseudomonadati Pseudomonadota Alphaproteobacteria Parvularculales Parvularcula oceani WP 031552237.1 313 aa 313 aa
BacClassIA Pseudomonadati Pseudomonadota Alphaproteobacteria Parvularculales Parvularcula mediterranea WP 173198541.1 314 aa 314 aa
BacClassIA Pseudomonadati Pseudomonadota Alphaproteobacteria Rhodospirillales Candidatus Endolissoclinum OUU21632.1 321 aa 321 aa
BacClassIA Pseudomonadati Pseudomonadota Alphaproteobacteria Rhodospirillales Candidatus Endolissoclinum MBC8429256.1 317 aa 317 aa
BacClassIA Pseudomonadati Pseudomonadota Alphaproteobacteria Candidatus Pelagibacterales Pelagibacteraceae bacterium GIR65353.1 308 aa 308 aa
BacClassIA Pseudomonadati Pseudomonadota Alphaproteobacteria Candidatus Pelagibacterales Pelagibacteraceae bacterium MBI29191.1 311 aa 311 aa
BacClassIA Pseudomonadati Pseudomonadota Alphaproteobacteria Rickettsiales Rickettsiales bacterium MBS56745.1 311 aa 311 aa
BacClassIA Pseudomonadati Pseudomonadota Alphaproteobacteria Candidatus Pelagibacterales Pelagibacteraceae bacterium MDC3177153.1 311 aa 311 aa
BacClassIA Pseudomonadati Pseudomonadota Alphaproteobacteria Candidatus Pelagibacterales Pelagibacteraceae bacterium MBH71818.1 308 aa 308 aa
BacClassIA Pseudomonadati Pseudomonadota Alphaproteobacteria Rickettsiales Rickettsiales bacterium MBL6621136.1 310 aa 310 aa
BacClassIA Pseudomonadati Pseudomonadota Alphaproteobacteria Rickettsiales Rickettsiales bacterium MBT4921715.1 310 aa 310 aa
BacClassIA Pseudomonadati Pseudomonadota Alphaproteobacteria Rickettsiales Rickettsiales bacterium MBT4989485.1 310 aa 310 aa
BacClassIA Pseudomonadati Pseudomonadota Alphaproteobacteria Rickettsiales Rickettsiales bacterium MBL6785043.1 310 aa 310 aa
BacClassIA Pseudomonadati Pseudomonadota Alphaproteobacteria unclassified Alphaproteobacteria Alphaproteobacteria bacterium GCA 001897445.1 309 aa 309 aa
BacClassIA Pseudomonadati Pseudomonadota Alphaproteobacteria Rickettsiales Rickettsiales endosymbiont WP 125216153.1 308 aa 308 aa
BacClassIA Pseudomonadati Pseudomonadota Alphaproteobacteria Parvibaculales Parvibaculales bacterium MGI9392179.1 310 aa 310 aa
BacClassIA Pseudomonadati Pseudomonadota Alphaproteobacteria Rickettsiales Rickettsiales bacterium MFI4984217.1 311 aa 311 aa
BacClassIA Pseudomonadati Pseudomonadota Alphaproteobacteria Rickettsiales Candidatus Jidaibacter GCF 000815465.1 313 aa 313 aa
BacClassIA Pseudomonadati Pseudomonadota Alphaproteobacteria Candidatus Pelagibacterales Pelagibacteraceae bacterium MDA9651081.1 311 aa 311 aa
BacClassIA Pseudomonadati Pseudomonadota Alphaproteobacteria Candidatus Pelagibacterales Pelagibacteraceae bacterium MDA7688110.1 314 aa 314 aa
BacClassIA Pseudomonadati Pseudomonadota Alphaproteobacteria Candidatus Pelagibacterales Pelagibacterales bacterium MBD1142103.1 311 aa 311 aa
BacClassIA Pseudomonadati Pseudomonadota Alphaproteobacteria Candidatus Pelagibacterales Pelagibacteraceae bacterium MDC3154733.1 311 aa 311 aa
BacClassIA Pseudomonadati Pseudomonadota Alphaproteobacteria Candidatus Pelagibacterales Candidatus Pelagibacterales MBF86498.1 310 aa 310 aa
BacClassIA Pseudomonadati Pseudomonadota Alphaproteobacteria Rickettsiales Rickettsiaceae bacterium MCE3231750.1 316 aa 316 aa
BacClassIA Pseudomonadati Pseudomonadota Alphaproteobacteria Rickettsiales Rickettsiales bacterium MCD6035659.1 309 aa 309 aa
BacClassIA Pseudomonadati Pseudomonadota Alphaproteobacteria unclassified Alphaproteobacteria Alphaproteobacteria bacterium MBL0318888.1 314 aa 314 aa
BacClassIA Pseudomonadati Pseudomonadota Alphaproteobacteria Rickettsiales Rickettsiales bacterium MCH2037311.1 310 aa 310 aa
BacClassIA Pseudomonadati Pseudomonadota Alphaproteobacteria Rickettsiales Rickettsiales bacterium MET0154710.1 310 aa 310 aa
BacClassIA Pseudomonadati Pseudomonadota Alphaproteobacteria Rickettsiales Rickettsiales bacterium MDB2414479.1 310 aa 310 aa
BacClassIA Pseudomonadati Pseudomonadota Alphaproteobacteria Rickettsiales Rickettsiales bacterium MFT4718177.1 310 aa 310 aa
BacClassIA Pseudomonadati Pseudomonadota Alphaproteobacteria Rickettsiales Rickettsiales bacterium MDB2550264.1 310 aa 310 aa
BacClassIA Pseudomonadati Pseudomonadota Alphaproteobacteria Rickettsiales Rickettsiales bacterium MFT5703722.1 310 aa 310 aa
BacClassIA Pseudomonadati Pseudomonadota Alphaproteobacteria Rickettsiales Rickettsiaceae bacterium MCE3255045.1 310 aa 310 aa
BacClassIA Pseudomonadati Pseudomonadota Alphaproteobacteria Rickettsiales Rickettsiales bacterium MCE2687266.1 310 aa 310 aa
BacClassIA Pseudomonadati Pseudomonadota Alphaproteobacteria Rickettsiales Rickettsiales bacterium MSP33342.1 309 aa 309 aa
BacClassIA Pseudomonadati Pseudomonadota Alphaproteobacteria Rickettsiales Rickettsiales bacterium HLD77163.1 309 aa 309 aa
BacClassIA Pseudomonadati Pseudomonadota Alphaproteobacteria Rickettsiales Rickettsiales bacterium MBU6338684.1 310 aa 310 aa
BacClassIA Pseudomonadati Pseudomonadota Alphaproteobacteria unclassified Alphaproteobacteria Alphaproteobacteria bacterium OFW80134.1 310 aa 310 aa
BacClassIA Pseudomonadati Pseudomonadota Alphaproteobacteria Rickettsiales Rickettsiales bacterium MBL6665247.1 310 aa 310 aa
BacClassIA Pseudomonadati Pseudomonadota Alphaproteobacteria Rickettsiales Rickettsiales bacterium MAZ80480.1 310 aa 310 aa
BacClassIA Pseudomonadati Pseudomonadota Alphaproteobacteria Rickettsiales Rickettsiales bacterium AIL65790.1 310 aa 310 aa
BacClassIA Pseudomonadati Pseudomonadota Alphaproteobacteria Hyphomicrobiales Hyphomicrobiales bacterium MCP5362109.1 320 aa 320 aa
BacClassIA Pseudomonadati Pseudomonadota Alphaproteobacteria unclassified Alphaproteobacteria Alphaproteobacteria bacterium DQGZ01000032.1 313 aa 313 aa
BacClassIA Pseudomonadati Pseudomonadota Alphaproteobacteria Rickettsiales Rickettsiales bacterium MDX1949605.1 315 aa 315 aa
BacClassIA Pseudomonadati Pseudomonadota Alphaproteobacteria unclassified Alphaproteobacteria Alphaproteobacteria bacterium GCA 002787615 310 aa 310 aa
BacClassIA Pseudomonadati Pseudomonadota Alphaproteobacteria Rhodospirillales Rhodospirillales bacterium MCY4430134.1 310 aa 310 aa
BacClassIA Pseudomonadati Pseudomonadota Alphaproteobacteria Rickettsiales Rickettsiales bacterium MDG1287970.1 310 aa 310 aa
BacClassIA Pseudomonadati Pseudomonadota Alphaproteobacteria Kordiimonadales Kordiimonas gwangyangensis GCF 000375545 310 aa 310 aa
BacClassIA Pseudomonadati Pseudomonadota Alphaproteobacteria Rickettsiales Rickettsiales bacterium MFO1242819.1 310 aa 310 aa
BacClassIA Pseudomonadati Pseudomonadota Alphaproteobacteria unclassified Alphaproteobacteria Alphaproteobacteria bacterium MCC7260424.1 312 aa 312 aa
BacClassIA Pseudomonadati Pseudomonadota Alphaproteobacteria Rickettsiales Rickettsiales bacterium HEU5046211.1 310 aa 310 aa
BacClassIA Pseudomonadati Pseudomonadota Alphaproteobacteria unclassified Alphaproteobacteria Alphaproteobacteria bacterium MDX2113385.1 311 aa 311 aa
BacClassIA Pseudomonadati Pseudomonadota Alphaproteobacteria unclassified Alphaproteobacteria Alphaproteobacteria bacterium MGE0753709.1 311 aa 311 aa
BacClassIA Pseudomonadati Pseudomonadota Alphaproteobacteria Rickettsiales Rickettsiales bacterium MFW0777471.1 311 aa 311 aa
BacClassIA Pseudomonadati Pseudomonadota Alphaproteobacteria Rickettsiales Rickettsiales bacterium MDX1974133.1 311 aa 311 aa
BacClassIA Pseudomonadati Pseudomonadota Alphaproteobacteria unclassified Alphaproteobacteria Alphaproteobacteria bacterium MDX2074659.1 311 aa 311 aa
BacClassIA Pseudomonadati Pseudomonadota Alphaproteobacteria Rickettsiales Rickettsiales bacterium MEZ5690616.1 311 aa 311 aa
BacClassIA Pseudomonadati Pseudomonadota Alphaproteobacteria Rickettsiales Rickettsiales bacterium MEQ1705445.1 311 aa 311 aa
BacClassIA Pseudomonadati Pseudomonadota Alphaproteobacteria Rickettsiales Rickettsiales bacterium MAR55871.1 311 aa 311 aa
BacClassIA Pseudomonadati Pseudomonadota Alphaproteobacteria Rickettsiales Rickettsiales bacterium MCI5049493.1 311 aa 311 aa
BacClassIA Pseudomonadati Pseudomonadota Alphaproteobacteria Rickettsiales Rickettsiales bacterium MBN67024.1 311 aa 311 aa
BacClassIA Pseudomonadati Pseudomonadota Alphaproteobacteria unclassified Alphaproteobacteria Alphaproteobacteria bacterium MFO0110101.1 311 aa 311 aa
BacClassIA Pseudomonadati Pseudomonadota Alphaproteobacteria unclassified Alphaproteobacteria Alphaproteobacteria bacterium MBN8544008.1 311 aa 311 aa
BacClassIA Pseudomonadati Pseudomonadota Alphaproteobacteria Rickettsiales Rickettsiales bacterium MFZ4540847.1 311 aa 311 aa
BacClassIA Pseudomonadati Pseudomonadota Alphaproteobacteria Rickettsiales Rickettsiales bacterium MFM9890471.1 311 aa 311 aa
BacClassIA Pseudomonadati Pseudomonadota Alphaproteobacteria Holosporales Candidatus Bealeia WP 331256143.1 310 aa 310 aa
BacClassIA Pseudomonadati Pseudomonadota Alphaproteobacteria unclassified Alphaproteobacteria Alphaproteobacteria bacterium MDA7981889.1 325 aa 325 aa
BacClassIA Pseudomonadati Pseudomonadota Alphaproteobacteria unclassified Alphaproteobacteria Alphaproteobacteria bacterium HYB57747.1 323 aa 323 aa
BacClassIA Pseudomonadati Pseudomonadota Alphaproteobacteria unclassified Alphaproteobacteria Alphaproteobacteria bacterium HUC63209.1 323 aa 323 aa
BacClassIA Pseudomonadati Pseudomonadota Alphaproteobacteria Holosporales Candidatus Nucleicultrix GCF 002117145 310 aa 310 aa
BacClassIA Pseudomonadati Pseudomonadota Alphaproteobacteria Holosporales Caedimonadaceae bacterium KAB2835803.1 310 aa 310 aa
BacClassIA Pseudomonadati Pseudomonadota Alphaproteobacteria unclassified Alphaproteobacteria Alphaproteobacteria bacterium MBP9693771.1 310 aa 310 aa
BacClassIA Pseudomonadati Pseudomonadota Alphaproteobacteria Holosporales Holosporales bacterium MFN7710079.1 310 aa 310 aa
BacClassIA Pseudomonadati Pseudomonadota Alphaproteobacteria Rhodospirillales Candidatus Hepatincolaceae MFL1780394.1 311 aa 311 aa
BacClassIA Pseudomonadati Pseudomonadota Alphaproteobacteria Holosporales Holosporales bacterium MGL6187613.1 263 aa 263 aa
BacClassIA Pseudomonadati Pseudomonadota Alphaproteobacteria Holosporales Holosporales bacterium MBY0264525.1 309 aa 309 aa
BacClassIA Pseudomonadati Pseudomonadota Alphaproteobacteria Rhodospirillales Rhodospirillaceae bacterium GCA 002700865 313 aa 313 aa
BacClassIA Pseudomonadati Pseudomonadota Alphaproteobacteria Holosporales Caedimonas varicaedens GAO98016.1 310 aa 310 aa
BacClassIA Pseudomonadati Pseudomonadota Alphaproteobacteria Holosporales Caedimonas sp MBL9029699.1 313 aa 313 aa
BacClassIA Pseudomonadati Pseudomonadota Alphaproteobacteria unclassified Alphaproteobacteria Alphaproteobacteria bacterium TNE27711.1 318 aa 318 aa
BacClassIA Pseudomonadati Pseudomonadota Alphaproteobacteria unclassified Alphaproteobacteria Alphaproteobacteria bacterium MDD9900716.1 316 aa 316 aa
BacClassIA Pseudomonadati Pseudomonadota Alphaproteobacteria unclassified Alphaproteobacteria Alphaproteobacteria bacterium MBI1214823.1 317 aa 317 aa
BacClassIA Pseudomonadati Pseudomonadota Alphaproteobacteria unclassified Alphaproteobacteria Alphaproteobacteria bacterium MBU6475077.1 317 aa 317 aa
BacClassIA Pseudomonadati Pseudomonadota Alphaproteobacteria unclassified Alphaproteobacteria Alphaproteobacteria bacterium MCK5518240.1 317 aa 317 aa
BacClassIA Pseudomonadati Pseudomonadota Alphaproteobacteria unclassified Alphaproteobacteria Alphaproteobacteria bacterium MCE9507529.1 317 aa 317 aa
BacClassIA Pseudomonadati Pseudomonadota Alphaproteobacteria unclassified Alphaproteobacteria Alphaproteobacteria bacterium MAH04982.1 311 aa 311 aa
BacClassIA Pseudomonadati Pseudomonadota Alphaproteobacteria unclassified Alphaproteobacteria Alphaproteobacteria bacterium HPD83293.1 310 aa 310 aa
BacClassIA Pseudomonadati Pseudomonadota Alphaproteobacteria unclassified Alphaproteobacteria Alphaproteobacteria bacterium MCK6418253.1 310 aa 310 aa
BacClassIA Pseudomonadati Pseudomonadota Alphaproteobacteria Rhodospirillales Rhodospirillales bacterium MCB9965090.1 310 aa 310 aa
BacClassIA Pseudomonadati Pseudomonadota Alphaproteobacteria Rhodospirillales Rhodospirillales bacterium MCB9978241.1 310 aa 310 aa
BacClassIA Pseudomonadati Pseudomonadota Alphaproteobacteria unclassified Alphaproteobacteria Alphaproteobacteria bacterium MBU0799820.1 310 aa 310 aa
BacClassIA Pseudomonadati Pseudomonadota Alphaproteobacteria Rhodospirillales Rhodospirillales bacterium MCB9991257.1 310 aa 310 aa
BacClassIA Pseudomonadati Pseudomonadota Alphaproteobacteria Rhodospirillales Rhodospirillales bacterium MCB9989346.1 310 aa 310 aa
BacClassIA Pseudomonadati Pseudomonadota Alphaproteobacteria unclassified Alphaproteobacteria Alphaproteobacteria bacterium MDJ0894008.1 310 aa 310 aa
BacClassIA Pseudomonadati Pseudomonadota Alphaproteobacteria Rhodospirillales Terasakiella pusilla GCF 000688235 310 aa 310 aa
BacClassIA Pseudomonadati Pseudomonadota Alphaproteobacteria Rhodospirillales Dongiaceae bacterium MGE0252049.1 310 aa 310 aa
BacClassIA Pseudomonadati Pseudomonadota Alphaproteobacteria unclassified Alphaproteobacteria Alphaproteobacteria bacterium MDH3228760.1 311 aa 311 aa
BacClassIA Pseudomonadati Pseudomonadota Alphaproteobacteria Rhodospirillales Hwanghaeella grinnelliae WP 127763323.1 310 aa 310 aa
BacClassIA Pseudomonadati Pseudomonadota Alphaproteobacteria Rhodospirillales Rhodospirillales bacterium MAO92057.1 311 aa 311 aa
BacClassIA Pseudomonadati Pseudomonadota Alphaproteobacteria Rhodospirillales Pacificispira spongiicola WP 169626133.1 311 aa 311 aa
BacClassIA Pseudomonadati Pseudomonadota Alphaproteobacteria Rhodospirillales Curvivirga aplysinae WP 155153749.1 311 aa 311 aa
BacClassIA Pseudomonadati Pseudomonadota Alphaproteobacteria Rhodospirillales Aestuariispira insulae WP 115935358.1 311 aa 311 aa
BacClassIA Pseudomonadati Pseudomonadota Alphaproteobacteria Rhodospirillales Aestuariispira ectoiniformans WP 259780899.1 311 aa 311 aa
BacClassIA Pseudomonadati Pseudomonadota Alphaproteobacteria Rhodospirillales Rhodospirillaceae bacterium SRX3572458 310 aa 310 aa
BacClassIA Pseudomonadati Pseudomonadota Alphaproteobacteria unclassified Alphaproteobacteria Alphaproteobacteria bacterium MCA8929033.1 310 aa 310 aa
BacClassIA Pseudomonadati Pseudomonadota Alphaproteobacteria unclassified Alphaproteobacteria Alphaproteobacteria bacterium HKK31141.1 310 aa 310 aa
BacClassIA Pseudomonadati Pseudomonadota Alphaproteobacteria Rhodospirillales Kiloniella laminariae GCF 000374005 310 aa 310 aa
BacClassIA Pseudomonadati Pseudomonadota Alphaproteobacteria Rhodospirillales Dongia sp GCF 000620685 310 aa 310 aa
BacClassIA Pseudomonadati Pseudomonadota Alphaproteobacteria Sneathiellales Sneathiella glossodoripedis GCF 000616095 311 aa 311 aa
BacClassIA Pseudomonadati Pseudomonadota Alphaproteobacteria Hyphomicrobiales Candidatus Phaeomarinobacter GCF 000689395 310 aa 310 aa
BacClassIA Pseudomonadati Pseudomonadota Alphaproteobacteria Rhodospirillales Nisaea denitrificans GCF 000426505 311 aa 311 aa
BacClassIA Pseudomonadati Pseudomonadota Alphaproteobacteria Candidatus Puniceispirillales SAR116 cluster MAQ06129.1 310 aa 310 aa
BacClassIA Pseudomonadati Pseudomonadota Alphaproteobacteria Candidatus Puniceispirillales Candidatus Levibacter WP 443643954.1 310 aa 310 aa
BacClassIA Pseudomonadati Pseudomonadota Alphaproteobacteria Rhodospirillales Thalassospira sp GCF 001983425 310 aa 310 aa
BacClassIA Pseudomonadati Pseudomonadota Alphaproteobacteria Rhodospirillales Defluviicoccus sp MDE0175728.1 310 aa 310 aa
BacClassIA Pseudomonadati Pseudomonadota Alphaproteobacteria unclassified Alphaproteobacteria Alphaproteobacteria bacterium HEX9837615.1 310 aa 310 aa
BacClassIA Pseudomonadati Pseudomonadota Alphaproteobacteria Rhodospirillales Rhodospirillales bacterium MGE0744070.1 310 aa 310 aa
BacClassIA Pseudomonadati Pseudomonadota Alphaproteobacteria Rhodospirillales Rhodospirillaceae bacterium RZO38979.1 310 aa 310 aa
BacClassIA Pseudomonadati Pseudomonadota Alphaproteobacteria unclassified Alphaproteobacteria Alphaproteobacteria bacterium GCA 002348425.1 314 aa 314 aa
BacClassIA Pseudomonadati Pseudomonadota Alphaproteobacteria Rhodospirillales Rhodospirillaceae bacterium MEK9683226.1 310 aa 310 aa
BacClassIA Pseudomonadati Pseudomonadota Alphaproteobacteria Rhodospirillales Rhodospirillaceae bacterium MBB42299.1 314 aa 314 aa
BacClassIA Pseudomonadati Pseudomonadota Alphaproteobacteria Rhodospirillales Rhodospirillaceae bacterium MBE89589.1 311 aa 311 aa
BacClassIA Pseudomonadati Pseudomonadota Alphaproteobacteria unclassified Alphaproteobacteria Alphaproteobacteria bacterium MEJ2122242.1 310 aa 310 aa
BacClassIA Pseudomonadati Pseudomonadota Alphaproteobacteria unclassified Alphaproteobacteria Alphaproteobacteria bacterium MCZ6862303.1 310 aa 310 aa
BacClassIA Pseudomonadati Pseudomonadota Alphaproteobacteria Rhodospirillales Rhodospirillales bacterium QQS11179.1 310 aa 310 aa
BacClassIA Pseudomonadati Pseudomonadota Alphaproteobacteria Hyphomicrobiales Vineibacter terrae WP 329713554.1 310 aa 310 aa
BacClassIA Pseudomonadati Pseudomonadota Alphaproteobacteria Hyphomicrobiales Reyranella sp WP 428668973.1 310 aa 310 aa
BacClassIA Pseudomonadati Pseudomonadota Alphaproteobacteria Hyphomicrobiales Reyranella massiliensis WP 020698371.1 310 aa 310 aa
BacClassIA Pseudomonadati Pseudomonadota Alphaproteobacteria Hyphomicrobiales Reyranella sp WP 296379723.1 310 aa 310 aa
BacClassIA Pseudomonadati Pseudomonadota Alphaproteobacteria Hyphomicrobiales Reyranella sp WP 305189063.1 310 aa 310 aa
BacClassIA Pseudomonadati Pseudomonadota Alphaproteobacteria Hyphomicrobiales Reyranellaceae bacterium HYC63132.1 310 aa 310 aa
BacClassIA Pseudomonadati Pseudomonadota Alphaproteobacteria Hyphomicrobiales Enhydrobacter sp MEI6200727.1 310 aa 310 aa
BacClassIA Pseudomonadati Pseudomonadota Alphaproteobacteria Rhodospirillales Rhodospirillales bacterium MBL8833315.1 310 aa 310 aa
BacClassIA Pseudomonadati Pseudomonadota Alphaproteobacteria Rhodospirillales Azospirillum sp MCA3245729.1 310 aa 310 aa
BacClassIA Pseudomonadati Pseudomonadota Alphaproteobacteria Rhodospirillales Rhodospirillales bacterium MGQ0677049.1 319 aa 319 aa
BacClassIA Pseudomonadati Pseudomonadota Alphaproteobacteria Rhodospirillales Rhodospirillaceae bacterium MSO96772.1 310 aa 310 aa
BacClassIA Pseudomonadati Pseudomonadota Alphaproteobacteria Rhodospirillales Rhodospirillaceae bacterium TAL02689.1 310 aa 310 aa
BacClassIA Pseudomonadati Pseudomonadota Alphaproteobacteria Rhodospirillales Rhodospirillaceae bacterium MBX7200800.1 310 aa 310 aa
BacClassIA Pseudomonadati Pseudomonadota Alphaproteobacteria Rhodospirillales Rhodospirillaceae bacterium GCA 002691085 311 aa 311 aa
BacClassIA Pseudomonadati Pseudomonadota Alphaproteobacteria Rhodospirillales Rhodospirillaceae bacterium MFL2769588.1 311 aa 311 aa
BacClassIA Pseudomonadati Pseudomonadota Alphaproteobacteria Rhodospirillales Rhodospirillaceae bacterium MDG2244508.1 311 aa 311 aa
BacClassIA Pseudomonadati Pseudomonadota Alphaproteobacteria Rhodospirillales Rhodospirillaceae bacterium MBP02497.1 309 aa 309 aa
BacClassIA Pseudomonadati Pseudomonadota Alphaproteobacteria Rhodospirillales Oleispirillum naphthae WP 337996855.1 310 aa 310 aa
BacClassIA Pseudomonadati Pseudomonadota Alphaproteobacteria Rhodospirillales Roseospirillum parvum WP 092613964.1 310 aa 310 aa
BacClassIA Pseudomonadati Pseudomonadota Alphaproteobacteria Rhodospirillales unclassified Haematospirillum WP 168020444.1 310 aa 310 aa
BacClassIA Pseudomonadati Pseudomonadota Alphaproteobacteria Rhodospirillales Novispirillum sp WP 425009307.1 310 aa 310 aa
BacClassIA Pseudomonadati Pseudomonadota Alphaproteobacteria Rhodospirillales Roseospira marina WP 150060551.1 310 aa 310 aa
BacClassIA Pseudomonadati Pseudomonadota Alphaproteobacteria Rhodospirillales Phaeovibrio sulfidiphilus WP 192533333.1 311 aa 311 aa
BacClassIA Pseudomonadati Pseudomonadota Alphaproteobacteria Rhodospirillales Rhodospirillum sp WP 413206902.1 310 aa 310 aa
BacClassIA Pseudomonadati Pseudomonadota Alphaproteobacteria Rhodospirillales Rhodospirillaceae bacterium MGB0697351.1 310 aa 310 aa
BacClassVI Pseudomonadati Pseudomonadota Alphaproteobacteria Rhodobacterales Paracoccaceae bacterium isolate DLM2.Bin65 TVQ52029.1 338 aa
BacClassVI Pseudomonadati Pseudomonadota Alphaproteobacteria Rhodobacterales Rubrimonas sp GCF 036788415.1 WP 333834198.1 338 aa
BacClassVI Pseudomonadati Pseudomonadota Alphaproteobacteria unclassified Alphaproteobacteria Alphaproteobacteria bacterium HGW Alphaproteobacteria 8 PKP66637.1 338 aa
BacClassVI Pseudomonadati Pseudomonadota Alphaproteobacteria Rhodobacterales Rubrimonas cliftonensis WP 093253121.1 338 aa
BacClassVI Pseudomonadati Pseudomonadota Alphaproteobacteria Rhodobacterales Thermohalobaculum xanthum WP 234455152.1 339 aa
BacClassVI Pseudomonadati Pseudomonadota Alphaproteobacteria Rhodobacterales Paracoccaceae bacterium isolate 21 Jan SF Bin22 MDH3666676.1 338 aa
BacClassVI Pseudomonadati Pseudomonadota Alphaproteobacteria Rhodobacterales Thermohalobaculum sp isolate SMAG U14450 HUF87701.1 338 aa
BacClassVI Pseudomonadati Pseudomonadota Alphaproteobacteria Rhodobacterales Thermohalobaculum sp isolate SMAG U14281 HUS54792.1 338 aa
BacClassVI Pseudomonadati Pseudomonadota Alphaproteobacteria Rhodobacterales Thermohalobaculum sp isolate Diamantina Trench FDZ175 bin41 MGR3718028.1 338 aa
BacClassVI Pseudomonadati Pseudomonadota Alphaproteobacteria Rhodobacterales Paracoccaceae bacterium isolate SMAG U10477 HLS19608.1 338 aa
BacClassVI Pseudomonadati Pseudomonadota Alphaproteobacteria Rhodobacterales Thermohalobaculum sp isolate SMAG U14458 HUF56810.1 339 aa
BacClassVI Pseudomonadati Pseudomonadota Alphaproteobacteria Rhodobacterales Paracoccaceae bacterium isolate sb S10C3901 MGF1502289.1 332 aa
BacClassVI Pseudomonadati Pseudomonadota Alphaproteobacteria Rhodobacterales Pikeienuella sp isolate sb S10C5069 MGF1445619.1 338 aa
BacClassVI Pseudomonadati Pseudomonadota Alphaproteobacteria Rhodobacterales Paralimibaculum aggregatum WP 285669699.1 338 aa
BacClassVI Pseudomonadati Pseudomonadota Alphaproteobacteria Rhodobacterales Neomegalonema sp isolate CC bin21 MCI4660726.1 340 aa
BacClassVI Pseudomonadati Pseudomonadota Alphaproteobacteria Rhodobacterales Paracoccaceae bacterium isolate MAG.057 MBX2856234.1 336 aa
BacClassVI Pseudomonadati Pseudomonadota Alphaproteobacteria Rhodobacterales Neomegalonema sp isolate CC bin1 MCI4665430.1 345 aa
BacClassVI Pseudomonadati Pseudomonadota Alphaproteobacteria Rhodobacterales Pikeienuella sp isolate ME XS 2307 bin 73 MGB0410451.1 338 aa

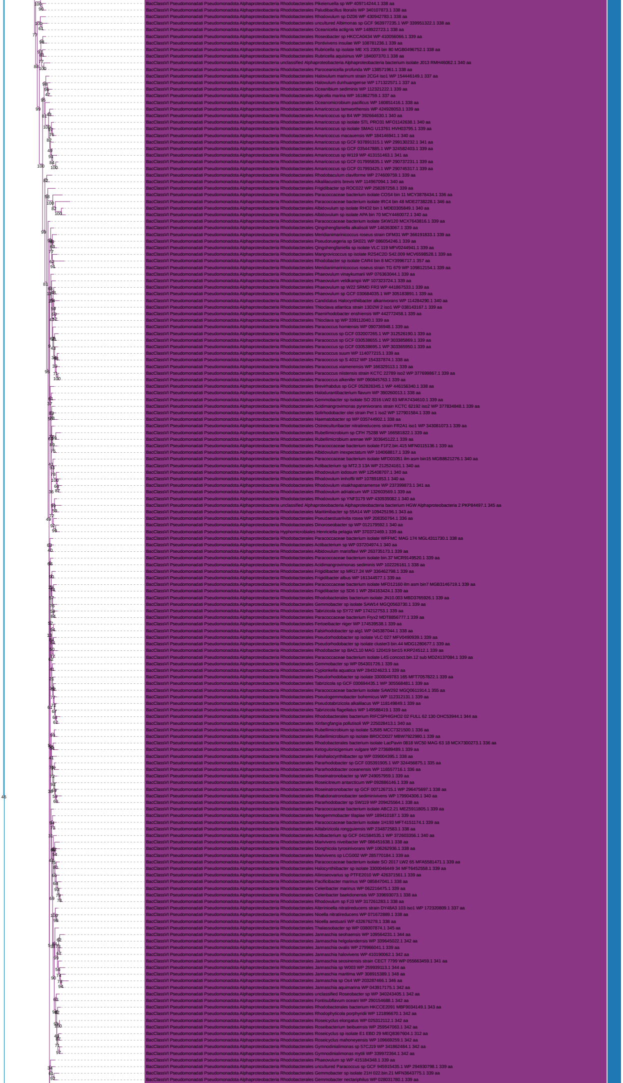
BacClassVI Pseudomonadati Pseudomonadota Alphaproteobacteria Rhodobacterales Pikeienuella sp WP 409714244.1 338 aa
BacClassVI Pseudomonadati Pseudomonadota Alphaproteobacteria Rhodobacterales Paludibacillus litoralis WP 340107873.1 338 aa
BacClassVI Pseudomonadati Pseudomonadota Alphaproteobacteria Rhodobacterales Rhodovulum sp DZ06 WP 430942783.1 338 aa
BacClassVI Pseudomonadati Pseudomonadota Alphaproteobacteria Rhodobacterales uncultured Albimonas sp GCF 963977235.1 WP 339951322.1 338 aa
BacClassVI Pseudomonadati Pseudomonadota Alphaproteobacteria Rhodobacterales Oceanicella actignis WP 148922723.1 338 aa
BacClassVI Pseudomonadati Pseudomonadota Alphaproteobacteria Rhodobacterales Roseobacter sp HKCCA0434 WP 410056066.1 339 aa
BacClassVI Pseudomonadati Pseudomonadota Alphaproteobacteria Rhodobacterales Pontivivens insulae WP 108781236.1 339 aa
BacClassVI Pseudomonadati Pseudomonadota Alphaproteobacteria Rhodobacterales Rubricella sp isolate ME XS 2305 bin 80 MGB0496752.1 338 aa
BacClassVI Pseudomonadati Pseudomonadota Alphaproteobacteria Rhodobacterales Rubricella aquisinus WP 184007370.1 338 aa
BacClassVI Pseudomonadati Pseudomonadota Alphaproteobacteria unclassified Alphaproteobacteria Alphaproteobacteria bacterium isolate J013 RMH46062.1 340 aa
BacClassVI Pseudomonadati Pseudomonadota Alphaproteobacteria Rhodobacterales Paroceanicella profunda WP 138571961.1 338 aa
BacClassVI Pseudomonadati Pseudomonadota Alphaproteobacteria Rhodobacterales Halovulum marinum strain 2CG4 iso1 WP 154446149.1 337 aa
BacClassVI Pseudomonadati Pseudomonadota Alphaproteobacteria Rhodobacterales Halovulum dunhuangense WP 171322571.1 337 aa
BacClassVI Pseudomonadati Pseudomonadota Alphaproteobacteria Rhodobacterales Oceanibium sediminis WP 112321222.1 339 aa
BacClassVI Pseudomonadati Pseudomonadota Alphaproteobacteria Rhodobacterales Algicella marina WP 161862759.1 337 aa
BacClassVI Pseudomonadati Pseudomonadota Alphaproteobacteria Rhodobacterales Oceanomicrobium pacificus WP 160851416.1 338 aa
BacClassVI Pseudomonadati Pseudomonadota Alphaproteobacteria Rhodobacterales Amaricoccus tamworthensis WP 424928053.1 339 aa
BacClassVI Pseudomonadati Pseudomonadota Alphaproteobacteria Rhodobacterales Amaricoccus sp B4 WP 392664630.1 340 aa
BacClassVI Pseudomonadati Pseudomonadota Alphaproteobacteria Rhodobacterales Amaricoccus sp isolate STL PRO31 MFO1142638.1 340 aa
BacClassVI Pseudomonadati Pseudomonadota Alphaproteobacteria Rhodobacterales Amaricoccus sp isolate SMAG U13761 HVH03795.1 339 aa
BacClassVI Pseudomonadati Pseudomonadota Alphaproteobacteria Rhodobacterales Amaricoccus macauensis WP 184146941.1 340 aa
BacClassVI Pseudomonadati Pseudomonadota Alphaproteobacteria Rhodobacterales Amaricoccus sp GCF 937891315.1 WP 299130232.1 341 aa
BacClassVI Pseudomonadati Pseudomonadota Alphaproteobacteria Rhodobacterales Amaricoccus sp GCF 035447885.1 WP 324582403.1 339 aa
BacClassVI Pseudomonadati Pseudomonadota Alphaproteobacteria Rhodobacterales Amaricoccus sp W119 WP 413151463.1 341 aa
BacClassVI Pseudomonadati Pseudomonadota Alphaproteobacteria Rhodobacterales Amaricoccus sp GCF 017995835.1 WP 290737231.1 339 aa
BacClassVI Pseudomonadati Pseudomonadota Alphaproteobacteria Rhodobacterales Amaricoccus sp GCF 017993425.1 WP 290745317.1 339 aa
BacClassVI Pseudomonadati Pseudomonadota Alphaproteobacteria Rhodobacterales Rhodobaculum claviforme WP 274609759.1 339 aa
BacClassVI Pseudomonadati Pseudomonadota Alphaproteobacteria Rhodobacterales Alkalilacustris brevis WP 114967094.1 340 aa
BacClassVI Pseudomonadati Pseudomonadota Alphaproteobacteria Rhodobacterales Frigidibacter sp ROC022 WP 258287258.1 339 aa
BacClassVI Pseudomonadati Pseudomonadota Alphaproteobacteria Rhodobacterales Paracoccaceae bacterium isolate COS4 bin 11 MCY3878434.1 336 aa
BacClassVI Pseudomonadati Pseudomonadota Alphaproteobacteria Rhodobacterales Paracoccaceae bacterium isolate IRC4 bin 48 MDE2738228.1 346 aa
BacClassVI Pseudomonadati Pseudomonadota Alphaproteobacteria Rhodobacterales Albidovulum sp isolate RHO2 bin 1 MDE0305849.1 340 aa
BacClassVI Pseudomonadati Pseudomonadota Alphaproteobacteria Rhodobacterales Albidovulum sp isolate APA bin 70 MCY4460072.1 340 aa
BacClassVI Pseudomonadati Pseudomonadota Alphaproteobacteria Rhodobacterales Paracoccaceae bacterium isolate SKW120 MCX7643816.1 339 aa
BacClassVI Pseudomonadati Pseudomonadota Alphaproteobacteria Rhodobacterales Qingshengfaniella alkalisoli WP 146363067.1 339 aa
BacClassVI Pseudomonadati Pseudomonadota Alphaproteobacteria Rhodobacterales Meridianimarinicoccus roseus strain DFM31 WP 366191833.1 339 aa
BacClassVI Pseudomonadati Pseudomonadota Alphaproteobacteria Rhodobacterales Pseudoruegeria sp SK021 WP 086054246.1 339 aa
BacClassVI Pseudomonadati Pseudomonadota Alphaproteobacteria Rhodobacterales Qingshengfaniella sp isolate VLC 119 MFV0244941.1 339 aa
BacClassVI Pseudomonadati Pseudomonadota Alphaproteobacteria Rhodobacterales Mangrovicoccus sp isolate R2S4C2D S42.009 MCV6598528.1 339 aa
BacClassVI Pseudomonadati Pseudomonadota Alphaproteobacteria Rhodobacterales Rhodobacter sp isolate CAR4 bin 8 MCY3996717.1 357 aa
BacClassVI Pseudomonadati Pseudomonadota Alphaproteobacteria Rhodobacterales Meridianimarinicoccus roseus strain TG 679 WP 109812154.1 339 aa
BacClassVI Pseudomonadati Pseudomonadota Alphaproteobacteria Rhodobacterales Phaeovulum vinaykumarii WP 076363044.1 339 aa
BacClassVI Pseudomonadati Pseudomonadota Alphaproteobacteria Rhodobacterales Phaeovulum veldkampii WP 107323724.1 339 aa
BacClassVI Pseudomonadati Pseudomonadota Alphaproteobacteria Rhodobacterales Phaeovulum sp W22 SRMD FR3 WP 441867533.1 339 aa
BacClassVI Pseudomonadati Pseudomonadota Alphaproteobacteria Rhodobacterales Phaeovulum sp GCF 030684035.1 WP 305183891.1 339 aa
BacClassVI Pseudomonadati Pseudomonadota Alphaproteobacteria Rhodobacterales Candidatus Halocynthiibacter alkanivorans WP 114284290.1 340 aa
BacClassVI Pseudomonadati Pseudomonadota Alphaproteobacteria Rhodobacterales Thioclava atlantica strain 13D2W 2 iso1 WP 038143167.1 339 aa
BacClassVI Pseudomonadati Pseudomonadota Alphaproteobacteria Rhodobacterales Paenirhodobacter enshiensis WP 442772458.1 339 aa
BacClassVI Pseudomonadati Pseudomonadota Alphaproteobacteria Rhodobacterales Thioclava sp WP 339112040.1 339 aa
BacClassVI Pseudomonadati Pseudomonadota Alphaproteobacteria Rhodobacterales Paracoccus homiensis WP 090736948.1 339 aa
BacClassVI Pseudomonadati Pseudomonadota Alphaproteobacteria Rhodobacterales Paracoccus sp GCF 032007265.1 WP 312526190.1 339 aa
BacClassVI Pseudomonadati Pseudomonadota Alphaproteobacteria Rhodobacterales Paracoccus sp GCF 030538655.1 WP 303385869.1 339 aa
BacClassVI Pseudomonadati Pseudomonadota Alphaproteobacteria Rhodobacterales Paracoccus sp GCF 030538695.1 WP 303365950.1 339 aa
BacClassVI Pseudomonadati Pseudomonadota Alphaproteobacteria Rhodobacterales Paracoccus suum WP 114077215.1 339 aa
BacClassVI Pseudomonadati Pseudomonadota Alphaproteobacteria Rhodobacterales Paracoccus sp S 4012 WP 154337874.1 338 aa
BacClassVI Pseudomonadati Pseudomonadota Alphaproteobacteria Rhodobacterales Paracoccus xiamenensis WP 166329113.1 339 aa
BacClassVI Pseudomonadati Pseudomonadota Alphaproteobacteria Rhodobacterales Paracoccus niistensis strain KCTC 22789 iso2 WP 377699867.1 339 aa
BacClassVI Pseudomonadati Pseudomonadota Alphaproteobacteria Rhodobacterales Paracoccus alkenifer WP 090845763.1 339 aa
BacClassVI Pseudomonadati Pseudomonadota Alphaproteobacteria Rhodobacterales Brevirhabdus sp GCF 052826345.1 WP 446156340.1 338 aa
BacClassVI Pseudomonadati Pseudomonadota Alphaproteobacteria Rhodobacterales Halodurantibacterium flavum WP 390260013.1 338 aa
BacClassVI Pseudomonadati Pseudomonadota Alphaproteobacteria Rhodobacterales Gemmobacter sp isolate SO 2016 LW2 83 MFA7434610.1 339 aa
BacClassVI Pseudomonadati Pseudomonadota Alphaproteobacteria Rhodobacterales Acidimangrovimonas pyrenivorans strain KCTC 62192 iso2 WP 377834848.1 339 aa
BacClassVI Pseudomonadati Pseudomonadota Alphaproteobacteria Rhodobacterales Solirhodobacter olei strain Pet 1 iso2 WP 127901584.1 339 aa
BacClassVI Pseudomonadati Pseudomonadota Alphaproteobacteria Rhodobacterales Haematobacter sp WP 035744902.1 338 aa
BacClassVI Pseudomonadati Pseudomonadota Alphaproteobacteria Rhodobacterales Ostreiculturibacter nitratireducens strain FR2A1 iso1 WP 343081073.1 339 aa
BacClassVI Pseudomonadati Pseudomonadota Alphaproteobacteria Rhodobacterales Rubellimicrobium sp CFH 75288 WP 166581822.1 339 aa
BacClassVI Pseudomonadati Pseudomonadota Alphaproteobacteria Rhodobacterales Rubellimicrobium arenae WP 303645122.1 339 aa
BacClassVI Pseudomonadati Pseudomonadota Alphaproteobacteria Rhodobacterales Paracoccaceae bacterium isolate F1F2.bin.415 MFN0115136.1 339 aa
BacClassVI Pseudomonadati Pseudomonadota Alphaproteobacteria Rhodobacterales Albidovulum inexpectatum WP 104068817.1 339 aa
BacClassVI Pseudomonadati Pseudomonadota Alphaproteobacteria Rhodobacterales Paracoccaceae bacterium isolate MFD01051 ilm asm bin15 MGB8621276.1 340 aa
BacClassVI Pseudomonadati Pseudomonadota Alphaproteobacteria Rhodobacterales Actibacterium sp MT2.3 13A WP 212524161.1 340 aa
BacClassVI Pseudomonadati Pseudomonadota Alphaproteobacteria Rhodobacterales Rhodovulum iodosum WP 125408707.1 340 aa
BacClassVI Pseudomonadati Pseudomonadota Alphaproteobacteria Rhodobacterales Rhodovulum imhoffii WP 107891853.1 340 aa
BacClassVI Pseudomonadati Pseudomonadota Alphaproteobacteria Rhodobacterales Rhodovulum visakhapatnamense WP 237399873.1 341 aa
BacClassVI Pseudomonadati Pseudomonadota Alphaproteobacteria Rhodobacterales Rhodovulum adriaticum WP 132603569.1 339 aa
BacClassVI Pseudomonadati Pseudomonadota Alphaproteobacteria Rhodobacterales Rhodovulum sp YNF3179 WP 430939082.1 340 aa
BacClassVI Pseudomonadati Pseudomonadota Alphaproteobacteria unclassified Alphaproteobacteria Alphaproteobacteria bacterium HGW Alphaproteobacteria 2 PKP84497.1 345 aa
BacClassVI Pseudomonadati Pseudomonadota Alphaproteobacteria Rhodobacterales Maritimibacter sp 55A14 WP 109425196.1 343 aa
BacClassVI Pseudomonadati Pseudomonadota Alphaproteobacteria Rhodobacterales Pseudaestuariivita rosea WP 208350764.1 336 aa
BacClassVI Pseudomonadati Pseudomonadota Alphaproteobacteria Rhodobacterales Dinoroseobacter sp WP 012179592.1 340 aa
BacClassVI Pseudomonadati Pseudomonadota Alphaproteobacteria Hyphomicrobiales Henriciella pelagia WP 370372469.1 339 aa
BacClassVI Pseudomonadati Pseudomonadota Alphaproteobacteria Rhodobacterales Paracoccaceae bacterium isolate WFFMC MAG 174 MGL4311730.1 338 aa
BacClassVI Pseudomonadati Pseudomonadota Alphaproteobacteria Rhodobacterales Actibacterium sp WP 037204974.1 340 aa
BacClassVI Pseudomonadati Pseudomonadota Alphaproteobacteria Rhodobacterales Albidovulum marisflavi WP 263735173.1 339 aa
BacClassVI Pseudomonadati Pseudomonadota Alphaproteobacteria Rhodobacterales Paracoccaceae bacterium isolate bin.37 MCR9149520.1 339 aa
BacClassVI Pseudomonadati Pseudomonadota Alphaproteobacteria Rhodobacterales Acidimangrovimonas sediminis WP 102226161.1 338 aa
BacClassVI Pseudomonadati Pseudomonadota Alphaproteobacteria Rhodobacterales Frigidibacter sp MR17.24 WP 336462798.1 339 aa
BacClassVI Pseudomonadati Pseudomonadota Alphaproteobacteria Rhodobacterales Frigidibacter albus WP 161344977.1 339 aa
BacClassVI Pseudomonadati Pseudomonadota Alphaproteobacteria Rhodobacterales Paracoccaceae bacterium isolate MFD12160 ilm asm bin7 MGB3146719.1 339 aa
BacClassVI Pseudomonadati Pseudomonadota Alphaproteobacteria Rhodobacterales Frigidibacter sp SD6 1 WP 284163424.1 339 aa
BacClassVI Pseudomonadati Pseudomonadota Alphaproteobacteria Rhodobacterales Rhodobacterales bacterium isolate JN10.003 MBD3765926.1 339 aa
BacClassVI Pseudomonadati Pseudomonadota Alphaproteobacteria Rhodobacterales Gemmobacter sp isolate SAW14 MGQ0563730.1 339 aa
BacClassVI Pseudomonadati Pseudomonadota Alphaproteobacteria Rhodobacterales Tabrizicola sp SY72 WP 174212753.1 339 aa
BacClassVI Pseudomonadati Pseudomonadota Alphaproteobacteria Rhodobacterales Paracoccaceae bacterium Fryx2 MDT8856777.1 339 aa
BacClassVI Pseudomonadati Pseudomonadota Alphaproteobacteria Rhodobacterales Fertoeibacter niger WP 174539538.1 339 aa
BacClassVI Pseudomonadati Pseudomonadota Alphaproteobacteria Rhodobacterales Falsirhodobacter sp alg1 WP 045387044.1 338 aa
BacClassVI Pseudomonadati Pseudomonadota Alphaproteobacteria Rhodobacterales Pseudorhodobacter sp isolate VLC 027 MFV0490939.1 339 aa
BacClassVI Pseudomonadati Pseudomonadota Alphaproteobacteria Rhodobacterales Pseudorhodobacter sp isolate cluster3 bin.44 MDG1280677.1 339 aa
BacClassVI Pseudomonadati Pseudomonadota Alphaproteobacteria Rhodobacterales Rhodobacter sp BACL10 MAG 120419 bin15 KRP24512.1 339 aa
BacClassVI Pseudomonadati Pseudomonadota Alphaproteobacteria Rhodobacterales Paracoccaceae bacterium isolate L4S concoct.bin.12 sub MDZ4137084.1 339 aa
BacClassVI Pseudomonadati Pseudomonadota Alphaproteobacteria Rhodobacterales Gemmobacter sp WP 054301726.1 339 aa
BacClassVI Pseudomonadati Pseudomonadota Alphaproteobacteria Rhodobacterales Cypionkella aquatica WP 284324623.1 339 aa
BacClassVI Pseudomonadati Pseudomonadota Alphaproteobacteria Rhodobacterales Pseudorhodobacter sp isolate 3300049783 165 MFT7057822.1 339 aa
BacClassVI Pseudomonadati Pseudomonadota Alphaproteobacteria Rhodobacterales Tabrizicola sp GCF 030694435.1 WP 305568481.1 339 aa
BacClassVI Pseudomonadati Pseudomonadota Alphaproteobacteria Rhodobacterales Paracoccaceae bacterium isolate SAW292 MGQ0611914.1 355 aa
BacClassVI Pseudomonadati Pseudomonadota Alphaproteobacteria Rhodobacterales Pseudogemmobacter bohemicus WP 112312131.1 339 aa
BacClassVI Pseudomonadati Pseudomonadota Alphaproteobacteria Rhodobacterales Pseudotabrizicola alkalilacus WP 118149849.1 339 aa
BacClassVI Pseudomonadati Pseudomonadota Alphaproteobacteria Rhodobacterales Tabrizicola flagellatus WP 149588419.1 339 aa
BacClassVI Pseudomonadati Pseudomonadota Alphaproteobacteria Rhodobacterales Rhodobacterales bacterium RIFCSPHIGHO2 02 FULL 62 130 OHC53944.1 344 aa
BacClassVI Pseudomonadati Pseudomonadota Alphaproteobacteria Rhodobacterales Xinfangfangia pollutisoli WP 225028413.1 340 aa
BacClassVI Pseudomonadati Pseudomonadota Alphaproteobacteria Rhodobacterales Rubellimicrobium sp isolate SJ585 MCC7321500.1 336 aa
BacClassVI Pseudomonadati Pseudomonadota Alphaproteobacteria Rhodobacterales Rubellimicrobium sp isolate BROCD027 MBW7922980.1 339 aa
BacClassVI Pseudomonadati Pseudomonadota Alphaproteobacteria Rhodobacterales Rhodobacterales bacterium isolate LacPavin 0818 WC50 MAG 63 18 MCX7300273.1 336 aa
BacClassVI Pseudomonadati Pseudomonadota Alphaproteobacteria Rhodobacterales Ketogulonicigenium vulgare WP 273689489.1 339 aa
BacClassVI Pseudomonadati Pseudomonadota Alphaproteobacteria Rhodobacterales Falsihalocynthiibacter sp WP 039004395.1 338 aa
BacClassVI Pseudomonadati Pseudomonadota Alphaproteobacteria Rhodobacterales Pararhodobacter sp GCF 035391905.1 WP 324456875.1 335 aa
BacClassVI Pseudomonadati Pseudomonadota Alphaproteobacteria Rhodobacterales Pararhodobacter oceanensis WP 116557716.1 336 aa
BacClassVI Pseudomonadati Pseudomonadota Alphaproteobacteria Rhodobacterales Roseinatronobacter sp WP 249057959.1 339 aa
BacClassVI Pseudomonadati Pseudomonadota Alphaproteobacteria Rhodobacterales Roseicitreum antarcticum WP 092886146.1 339 aa
BacClassVI Pseudomonadati Pseudomonadota Alphaproteobacteria Rhodobacterales Roseinatronobacter sp GCF 007126715.1 WP 296475697.1 338 aa
BacClassVI Pseudomonadati Pseudomonadota Alphaproteobacteria Rhodobacterales Rhabdonatronobacter sediminivivens WP 179904306.1 340 aa
BacClassVI Pseudomonadati Pseudomonadota Alphaproteobacteria Rhodobacterales Pararhodobacter sp SW119 WP 209425564.1 338 aa
BacClassVI Pseudomonadati Pseudomonadota Alphaproteobacteria Rhodobacterales Paracoccaceae bacterium isolate ABC2.21 MEZ5911805.1 339 aa
BacClassVI Pseudomonadati Pseudomonadota Alphaproteobacteria Rhodobacterales Neogemmobacter tilapiae WP 189410187.1 339 aa
BacClassVI Pseudomonadati Pseudomonadota Alphaproteobacteria Rhodobacterales Paracoccaceae bacterium isolate 1H193 MFT4151174.1 339 aa
BacClassVI Pseudomonadati Pseudomonadota Alphaproteobacteria Rhodobacterales Alitabrizicola rongguiensis WP 234872583.1 338 aa
BacClassVI Pseudomonadati Pseudomonadota Alphaproteobacteria Rhodobacterales Actibacterium sp GCF 041584535.1 WP 372603356.1 340 aa
BacClassVI Pseudomonadati Pseudomonadota Alphaproteobacteria Rhodobacterales Marivivens niveibacter WP 086451638.1 339 aa
BacClassVI Pseudomonadati Pseudomonadota Alphaproteobacteria Rhodobacterales Donghicola tyrosinivorans WP 106262936.1 338 aa
BacClassVI Pseudomonadati Pseudomonadota Alphaproteobacteria Rhodobacterales Marivivens sp LCG002 WP 285770184.1 339 aa
BacClassVI Pseudomonadati Pseudomonadota Alphaproteobacteria Rhodobacterales Paracoccaceae bacterium isolate SO 2017 LW2 65 MFA5581471.1 339 aa
BacClassVI Pseudomonadati Pseudomonadota Alphaproteobacteria Rhodobacterales Halocynthiibacter sp isolate 3300046449 34 MFT6452558.1 339 aa
BacClassVI Pseudomonadati Pseudomonadota Alphaproteobacteria Rhodobacterales Aliiroseovarius sp PTFE2010 WP 426371561.1 339 aa
BacClassVI Pseudomonadati Pseudomonadota Alphaproteobacteria Rhodobacterales Pacificibacter marinus WP 085847041.1 338 aa
BacClassVI Pseudomonadati Pseudomonadota Alphaproteobacteria Rhodobacterales Celeribacter marinus WP 062216475.1 339 aa
BacClassVI Pseudomonadati Pseudomonadota Alphaproteobacteria Rhodobacterales Celeribacter baekdonensis WP 339693073.1 338 aa
BacClassVI Pseudomonadati Pseudomonadota Alphaproteobacteria Rhodobacterales Rhodovulum sp FJ3 WP 317261283.1 338 aa
BacClassVI Pseudomonadati Pseudomonadota Alphaproteobacteria Rhodobacterales Alterinioella nitratireducens strain DY48A3 103 iso1 WP 172320809.1 337 aa
BacClassVI Pseudomonadati Pseudomonadota Alphaproteobacteria Rhodobacterales Nioella nitratireducens WP 071672889.1 338 aa
BacClassVI Pseudomonadati Pseudomonadota Alphaproteobacteria Rhodobacterales Nioella aestuarii WP 432676278.1 338 aa
BacClassVI Pseudomonadati Pseudomonadota Alphaproteobacteria Rhodobacterales Thalassobacter sp WP 038007874.1 345 aa
BacClassVI Pseudomonadati Pseudomonadota Alphaproteobacteria Rhodobacterales Jannaschia seohaensis WP 109564231.1 344 aa
BacClassVI Pseudomonadati Pseudomonadota Alphaproteobacteria Rhodobacterales Jannaschia helgolandensis WP 339645022.1 342 aa
BacClassVI Pseudomonadati Pseudomonadota Alphaproteobacteria Rhodobacterales Jannaschia ovalis WP 279966041.1 339 aa
BacClassVI Pseudomonadati Pseudomonadota Alphaproteobacteria Rhodobacterales Jannaschia halovivens WP 410190062.1 342 aa
BacClassVI Pseudomonadati Pseudomonadota Alphaproteobacteria Rhodobacterales Jannaschia seosinensis strain CECT 7799 WP 055663459.1 341 aa
BacClassVI Pseudomonadati Pseudomonadota Alphaproteobacteria Rhodobacterales Jannaschia sp W003 WP 259939113.1 344 aa
BacClassVI Pseudomonadati Pseudomonadota Alphaproteobacteria Rhodobacterales Jannaschia maritima WP 308915389.1 348 aa
BacClassVI Pseudomonadati Pseudomonadota Alphaproteobacteria Rhodobacterales Jannaschia sp Os4 WP 203287466.1 346 aa
BacClassVI Pseudomonadati Pseudomonadota Alphaproteobacteria Rhodobacterales Jannaschia aquimarina WP 043917175.1 342 aa
BacClassVI Pseudomonadati Pseudomonadota Alphaproteobacteria Rhodobacterales unclassified Roseobacter sp WP 340243405.1 342 aa
BacClassVI Pseudomonadati Pseudomonadota Alphaproteobacteria Rhodobacterales Fontisubflavum oceani WP 290154688.1 342 aa
BacClassVI Pseudomonadati Pseudomonadota Alphaproteobacteria Rhodobacterales Rhodobacterales bacterium HKCCE2091 MBF9034149.1 343 aa
BacClassVI Pseudomonadati Pseudomonadota Alphaproteobacteria Rhodobacterales Rhodophyticola porphyridii WP 121896670.1 342 aa
BacClassVI Pseudomonadati Pseudomonadota Alphaproteobacteria Rhodobacterales Roseicyclus elongatus WP 025312112.1 342 aa
BacClassVI Pseudomonadati Pseudomonadota Alphaproteobacteria Rhodobacterales Roseibacterium beibuensis WP 259547063.1 342 aa
BacClassVI Pseudomonadati Pseudomonadota Alphaproteobacteria Rhodobacterales Roseicyclus sp isolate E1 EBD 29 MEQ8367604.1 312 aa
BacClassVI Pseudomonadati Pseudomonadota Alphaproteobacteria Rhodobacterales Roseicyclus mahoneyensis WP 109669259.1 342 aa
BacClassVI Pseudomonadati Pseudomonadota Alphaproteobacteria Rhodobacterales Gymnodinialimonas sp 57CJ19 WP 341862484.1 342 aa
BacClassVI Pseudomonadati Pseudomonadota Alphaproteobacteria Rhodobacterales Gymnodinialimonas mytili WP 339972364.1 342 aa
BacClassVI Pseudomonadati Pseudomonadota Alphaproteobacteria Rhodobacterales Phaeovulum sp WP 415184348.1 339 aa
BacClassVI Pseudomonadati Pseudomonadota Alphaproteobacteria Rhodobacterales uncultured Paracoccus sp GCF 945915435.1 WP 294930798.1 339 aa
BacClassVI Pseudomonadati Pseudomonadota Alphaproteobacteria Rhodobacterales Gemmobacter sp isolate 21H 022.bin.21 MFN3643775.1 339 aa
BacClassVI Pseudomonadati Pseudomonadota Alphaproteobacteria Rhodobacterales Gemmobacter nectariphilus WP 028031780.1 339 aa

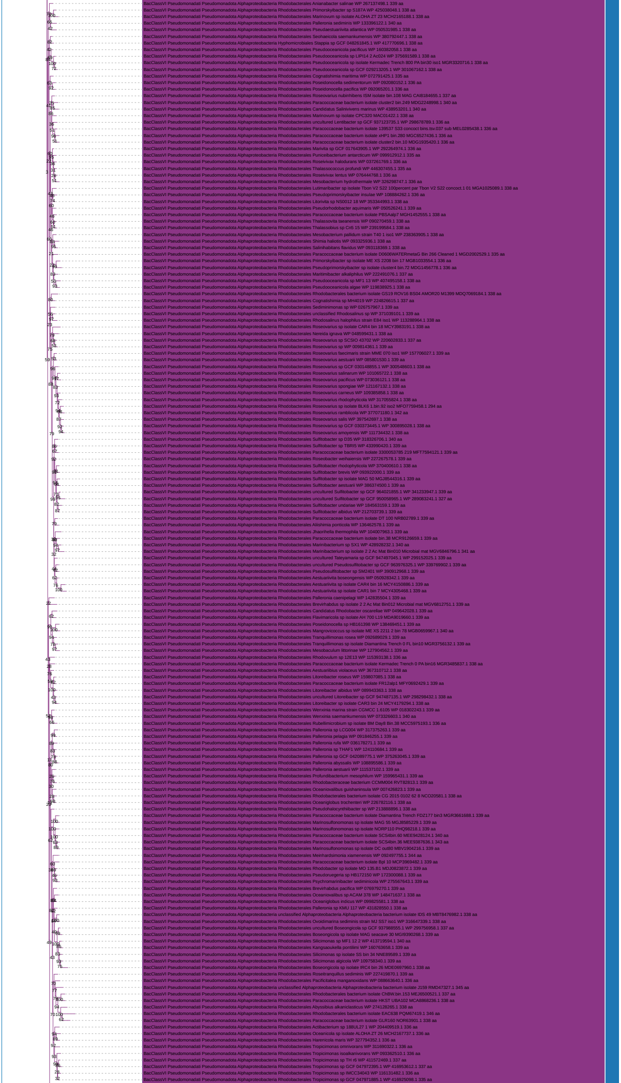

BacClassVI Pseudomonadati Pseudomonadota Alphaproteobacteria Rhodobacterales Anianabacter salinae WP 267137498.1 339 aa
BacClassVI Pseudomonadati Pseudomonadota Alphaproteobacteria Rhodobacterales Primorskyibacter sp S187A WP 425038048.1 338 aa
BacClassVI Pseudomonadati Pseudomonadota Alphaproteobacteria Rhodobacterales Marinovum sp isolate ALOHA ZT 23 MCH2165188.1 338 aa
BacClassVI Pseudomonadati Pseudomonadota Alphaproteobacteria Rhodobacterales Pallerionia sediminis WP 133396122.1 340 aa
BacClassVI Pseudomonadati Pseudomonadota Alphaproteobacteria Rhodobacterales Pseudaestuariivita atlantica WP 050531985.1 338 aa
BacClassVI Pseudomonadati Pseudomonadota Alphaproteobacteria Rhodobacterales Seohaeicola saemankumensis WP 380792447.1 338 aa
BacClassVI Pseudomonadati Pseudomonadota Alphaproteobacteria Hyphomicrobiales Stappia sp GCF 048261845.1 WP 417770696.1 338 aa
BacClassVI Pseudomonadati Pseudomonadota Alphaproteobacteria Rhodobacterales Pseudooceanicola pacificus WP 160382058.1 338 aa
BacClassVI Pseudomonadati Pseudomonadota Alphaproteobacteria Rhodobacterales Pseudooceanicola sp LIPI14 2 Ac024 WP 375691589.1 338 aa
BacClassVI Pseudomonadati Pseudomonadota Alphaproteobacteria Rhodobacterales Pseudooceanicola sp isolate Kermadec Trench 800 PA bin30 iso1 MGR3320716.1 338 aa
BacClassVI Pseudomonadati Pseudomonadota Alphaproteobacteria Rhodobacterales Pseudooceanicola sp GCF 029213205.1 WP 301067162.1 338 aa
BacClassVI Pseudomonadati Pseudomonadota Alphaproteobacteria Rhodobacterales Cognatishimia maritima WP 072791425.1 335 aa
BacClassVI Pseudomonadati Pseudomonadota Alphaproteobacteria Rhodobacterales Poseidonocella sedimentorum WP 092080152.1 336 aa
BacClassVI Pseudomonadati Pseudomonadota Alphaproteobacteria Rhodobacterales Poseidonocella pacifica WP 092065201.1 336 aa
BacClassVI Pseudomonadati Pseudomonadota Alphaproteobacteria Rhodobacterales Roseovarius nubinhibens ISM isolate bin.108 MAG CAI8184655.1 337 aa
BacClassVI Pseudomonadati Pseudomonadota Alphaproteobacteria Rhodobacterales Paracoccaceae bacterium isolate cluster2 bin.249 MDG2248998.1 340 aa
BacClassVI Pseudomonadati Pseudomonadota Alphaproteobacteria Rhodobacterales Candidatus Salinivivens marinus WP 438953201.1 340 aa
BacClassVI Pseudomonadati Pseudomonadota Alphaproteobacteria Rhodobacterales Marinovum sp isolate CPC320 MAC01422.1 338 aa
BacClassVI Pseudomonadati Pseudomonadota Alphaproteobacteria Rhodobacterales uncultured Lentibacter sp GCF 937123735.1 WP 298678789.1 336 aa
BacClassVI Pseudomonadati Pseudomonadota Alphaproteobacteria Rhodobacterales Paracoccaceae bacterium isolate 139537 S33 concoct bins.tsv.037 sub MEL0285438.1 336 aa
BacClassVI Pseudomonadati Pseudomonadota Alphaproteobacteria Rhodobacterales Paracoccaceae bacterium isolate xHP1 bin.280 MGC6527436.1 336 aa
BacClassVI Pseudomonadati Pseudomonadota Alphaproteobacteria Rhodobacterales Paracoccaceae bacterium isolate cluster2 bin.10 MDG1935420.1 338 aa
BacClassVI Pseudomonadati Pseudomonadota Alphaproteobacteria Rhodobacterales Marivita sp GCF 017643905.1 WP 292264974.1 336 aa
BacClassVI Pseudomonadati Pseudomonadota Alphaproteobacteria Rhodobacterales Puniceibacterium antarcticum WP 099912912.1 335 aa
BacClassVI Pseudomonadati Pseudomonadota Alphaproteobacteria Rhodobacterales Roseivivax halodurans WP 037261769.1 336 aa
BacClassVI Pseudomonadati Pseudomonadota Alphaproteobacteria Rhodobacterales Thalassococcus profundi WP 446307455.1 336 aa
BacClassVI Pseudomonadati Pseudomonadota Alphaproteobacteria Rhodobacterales Roseivivax lentus WP 076444768.1 336 aa
BacClassVI Pseudomonadati Pseudomonadota Alphaproteobacteria Rhodobacterales Mesobacterium hydrothermale WP 326298747.1 336 aa
BacClassVI Pseudomonadati Pseudomonadota Alphaproteobacteria Rhodobacterales Lutimaribacter sp isolate Tbon V2 S22 100percent par Tbon V2 S22 concoct.1 01 MGA1025089.1 338 aa
BacClassVI Pseudomonadati Pseudomonadota Alphaproteobacteria Rhodobacterales Pseudoprimorskyibacter insulae WP 108884262.1 336 aa
BacClassVI Pseudomonadati Pseudomonadota Alphaproteobacteria Rhodobacterales Litorivita sp NS0012 18 WP 353344993.1 338 aa
BacClassVI Pseudomonadati Pseudomonadota Alphaproteobacteria Rhodobacterales Pseudorhodobacter aquimaris WP 050526241.1 339 aa
BacClassVI Pseudomonadati Pseudomonadota Alphaproteobacteria Rhodobacterales Paracoccaceae bacterium isolate PBSAalp7 MGH1452555.1 338 aa
BacClassVI Pseudomonadati Pseudomonadota Alphaproteobacteria Rhodobacterales Thalassovita taeanensis WP 090270459.1 338 aa
BacClassVI Pseudomonadati Pseudomonadota Alphaproteobacteria Rhodobacterales Thalassobius sp Cn5 15 WP 239199584.1 338 aa
BacClassVI Pseudomonadati Pseudomonadota Alphaproteobacteria Rhodobacterales Mesobacterium pallidum strain T40 1 iso1 WP 238363905.1 338 aa
BacClassVI Pseudomonadati Pseudomonadota Alphaproteobacteria Rhodobacterales Shimia haliotis WP 093325936.1 338 aa
BacClassVI Pseudomonadati Pseudomonadota Alphaproteobacteria Rhodobacterales Salinihabitans flavidus WP 093118369.1 338 aa
BacClassVI Pseudomonadati Pseudomonadota Alphaproteobacteria Rhodobacterales Paracoccaceae bacterium isolate D0606WATERmetaG Bin 266 Cleaned 1 MGD2002529.1 335 aa
BacClassVI Pseudomonadati Pseudomonadota Alphaproteobacteria Rhodobacterales Primorskyibacter sp isolate ME XS 2208 bin 17 MGB1033554.1 336 aa
BacClassVI Pseudomonadati Pseudomonadota Alphaproteobacteria Rhodobacterales Pseudoprimorskyibacter sp isolate cluster4 bin.72 MDG1456778.1 336 aa
BacClassVI Pseudomonadati Pseudomonadota Alphaproteobacteria Rhodobacterales Maritimibacter alkaliphilus WP 222491076.1 337 aa
BacClassVI Pseudomonadati Pseudomonadota Alphaproteobacteria Rhodobacterales Pseudooceanicola sp MF1 13 WP 407495158.1 338 aa
BacClassVI Pseudomonadati Pseudomonadota Alphaproteobacteria Rhodobacterales Pseudooceanicola algae WP 119838925.1 338 aa
BacClassVI Pseudomonadati Pseudomonadota Alphaproteobacteria Rhodobacterales Rhodobacterales bacterium isolate GS19 ROV16 BS04 AMOR20 M1399 MDQ7069184.1 338 aa
BacClassVI Pseudomonadati Pseudomonadota Alphaproteobacteria Rhodobacterales Cognatishimia sp MH4019 WP 224826615.1 337 aa
BacClassVI Pseudomonadati Pseudomonadota Alphaproteobacteria Rhodobacterales Sediminimonas sp WP 026757967.1 339 aa
BacClassVI Pseudomonadati Pseudomonadota Alphaproteobacteria Rhodobacterales unclassified Rhodosalinus sp WP 371039101.1 339 aa
BacClassVI Pseudomonadati Pseudomonadota Alphaproteobacteria Rhodobacterales Rhodosalinus halophilus strain E84 iso1 WP 113288964.1 338 aa
BacClassVI Pseudomonadati Pseudomonadota Alphaproteobacteria Rhodobacterales Roseovarius sp isolate CAR4 bin 18 MCY3983191.1 338 aa
BacClassVI Pseudomonadati Pseudomonadota Alphaproteobacteria Rhodobacterales Nereida ignava WP 048599431.1 338 aa
BacClassVI Pseudomonadati Pseudomonadota Alphaproteobacteria Rhodobacterales Roseovarius sp SCSIO 43702 WP 220602833.1 337 aa
BacClassVI Pseudomonadati Pseudomonadota Alphaproteobacteria Rhodobacterales Roseovarius sp WP 009814361.1 339 aa
BacClassVI Pseudomonadati Pseudomonadota Alphaproteobacteria Rhodobacterales Roseovarius faecimaris strain MME 070 iso1 WP 157706027.1 339 aa
BacClassVI Pseudomonadati Pseudomonadota Alphaproteobacteria Rhodobacterales Roseovarius aestuarii WP 085801530.1 339 aa
BacClassVI Pseudomonadati Pseudomonadota Alphaproteobacteria Rhodobacterales Roseovarius sp GCF 030148855.1 WP 300548603.1 338 aa
BacClassVI Pseudomonadati Pseudomonadota Alphaproteobacteria Rhodobacterales Roseovarius salinarum WP 101065722.1 338 aa
BacClassVI Pseudomonadati Pseudomonadota Alphaproteobacteria Rhodobacterales Roseovarius pacificus WP 073036121.1 338 aa
BacClassVI Pseudomonadati Pseudomonadota Alphaproteobacteria Rhodobacterales Roseovarius spongiae WP 121167132.1 338 aa
BacClassVI Pseudomonadati Pseudomonadota Alphaproteobacteria Rhodobacterales Roseovarius carneus WP 109385858.1 338 aa
BacClassVI Pseudomonadati Pseudomonadota Alphaproteobacteria Rhodobacterales Roseovarius rhodophyticola WP 317055824.1 338 aa
BacClassVI Pseudomonadati Pseudomonadota Alphaproteobacteria Rhodobacterales Roseovarius sp isolate BLK6 1.bin.92 iso2 MFO7759458.1 294 aa
BacClassVI Pseudomonadati Pseudomonadota Alphaproteobacteria Rhodobacterales Roseovarius ramblicola WP 377071180.1 342 aa
BacClassVI Pseudomonadati Pseudomonadota Alphaproteobacteria Rhodobacterales Roseovarius salis WP 397542697.1 338 aa
BacClassVI Pseudomonadati Pseudomonadota Alphaproteobacteria Rhodobacterales Roseovarius sp GCF 030373445.1 WP 300895028.1 338 aa
BacClassVI Pseudomonadati Pseudomonadota Alphaproteobacteria Rhodobacterales Roseovarius amoyensis WP 111734432.1 338 aa
BacClassVI Pseudomonadati Pseudomonadota Alphaproteobacteria Rhodobacterales Sulfitobacter sp D35 WP 318326706.1 340 aa
BacClassVI Pseudomonadati Pseudomonadota Alphaproteobacteria Rhodobacterales Sulfitobacter sp TBRI5 WP 433990420.1 339 aa
BacClassVI Pseudomonadati Pseudomonadota Alphaproteobacteria Rhodobacterales Paracoccaceae bacterium isolate 3300053785 219 MFT7594121.1 339 aa
BacClassVI Pseudomonadati Pseudomonadota Alphaproteobacteria Rhodobacterales Roseobacter weihaiensis WP 227267578.1 339 aa
BacClassVI Pseudomonadati Pseudomonadota Alphaproteobacteria Rhodobacterales Sulfitobacter rhodophyticola WP 370400610.1 338 aa
BacClassVI Pseudomonadati Pseudomonadota Alphaproteobacteria Rhodobacterales Sulfitobacter brevis WP 093922000.1 339 aa
BacClassVI Pseudomonadati Pseudomonadota Alphaproteobacteria Rhodobacterales Sulfitobacter sp isolate MAG 50 MGJ8544316.1 339 aa
BacClassVI Pseudomonadati Pseudomonadota Alphaproteobacteria Rhodobacterales Sulfitobacter aestuarii WP 386374500.1 339 aa
BacClassVI Pseudomonadati Pseudomonadota Alphaproteobacteria Rhodobacterales uncultured Sulfitobacter sp GCF 964021855.1 WP 341233947.1 339 aa
BacClassVI Pseudomonadati Pseudomonadota Alphaproteobacteria Rhodobacterales uncultured Sulfitobacter sp GCF 950058965.1 WP 289083241.1 327 aa
BacClassVI Pseudomonadati Pseudomonadota Alphaproteobacteria Rhodobacterales Sulfitobacter undariae WP 184563159.1 339 aa
BacClassVI Pseudomonadati Pseudomonadota Alphaproteobacteria Rhodobacterales Sulfitobacter albidus WP 212703739.1 339 aa
BacClassVI Pseudomonadati Pseudomonadota Alphaproteobacteria Rhodobacterales Paracoccaceae bacterium isolate DT 100 NRB02789.1 339 aa
BacClassVI Pseudomonadati Pseudomonadota Alphaproteobacteria Rhodobacterales Aliishimia ponticola WP 136462578.1 339 aa
BacClassVI Pseudomonadati Pseudomonadota Alphaproteobacteria Rhodobacterales Jhaorihella thermophila WP 104007963.1 339 aa
BacClassVI Pseudomonadati Pseudomonadota Alphaproteobacteria Rhodobacterales Paracoccaceae bacterium isolate bin.38 MCR9126659.1 339 aa
BacClassVI Pseudomonadati Pseudomonadota Alphaproteobacteria Rhodobacterales Marinibacterium sp SX1 WP 428928232.1 340 aa
BacClassVI Pseudomonadati Pseudomonadota Alphaproteobacteria Rhodobacterales Marinibacterium sp isolate 2 2 Ac Mat Bin010 Microbial mat MGV6846796.1 341 aa
BacClassVI Pseudomonadati Pseudomonadota Alphaproteobacteria Rhodobacterales uncultured Tateyamaria sp GCF 947497045.1 WP 299152025.1 339 aa
BacClassVI Pseudomonadati Pseudomonadota Alphaproteobacteria Rhodobacterales uncultured Pseudosulfitobacter sp GCF 963976325.1 WP 339769902.1 339 aa
BacClassVI Pseudomonadati Pseudomonadota Alphaproteobacteria Rhodobacterales Pseudosulfitobacter sp SM2401 WP 390912968.1 339 aa
BacClassVI Pseudomonadati Pseudomonadota Alphaproteobacteria Rhodobacterales Aestuariivita boseongensis WP 050928342.1 339 aa
BacClassVI Pseudomonadati Pseudomonadota Alphaproteobacteria Rhodobacterales Aestuariivita sp isolate CAR4 bin 16 MCY4150886.1 339 aa
BacClassVI Pseudomonadati Pseudomonadota Alphaproteobacteria Rhodobacterales Aestuariivita sp isolate CAR1 bin 7 MCY4305468.1 339 aa
BacClassVI Pseudomonadati Pseudomonadota Alphaproteobacteria Rhodobacterales Pallerionia caenipelagi WP 142835504.1 339 aa
BacClassVI Pseudomonadati Pseudomonadota Alphaproteobacteria Rhodobacterales Brevirhabdus sp isolate 2 2 Ac Mat Bin012 Microbial mat MGV6812751.1 339 aa
BacClassVI Pseudomonadati Pseudomonadota Alphaproteobacteria Rhodobacterales Candidatus Rhodobacter oscarellae WP 049642028.1 339 aa
BacClassVI Pseudomonadati Pseudomonadota Alphaproteobacteria Rhodobacterales Flavimaricola sp isolate AH 700 L19 MDA9019660.1 339 aa
BacClassVI Pseudomonadati Pseudomonadota Alphaproteobacteria Rhodobacterales Poseidonocella sp HB161398 WP 138469451.1 339 aa
BacClassVI Pseudomonadati Pseudomonadota Alphaproteobacteria Rhodobacterales Mangrovicoccus sp isolate ME XS 2211 2 bin 78 MGB0659967.1 340 aa
BacClassVI Pseudomonadati Pseudomonadota Alphaproteobacteria Rhodobacterales Tranquillimonas rosea WP 092689029.1 339 aa
BacClassVI Pseudomonadati Pseudomonadota Alphaproteobacteria Rhodobacterales Tranquillimonas sp isolate Diamantina Trench 0 FL bin10 MGR3756132.1 339 aa
BacClassVI Pseudomonadati Pseudomonadota Alphaproteobacteria Rhodobacterales Mesobaculum littorinae WP 127904562.1 339 aa
BacClassVI Pseudomonadati Pseudomonadota Alphaproteobacteria Rhodobacterales Rhodovulum sp 12E13 WP 115393138.1 336 aa
BacClassVI Pseudomonadati Pseudomonadota Alphaproteobacteria Rhodobacterales Paracoccaceae bacterium isolate Kermadec Trench 0 PA bin16 MGR3485837.1 338 aa
BacClassVI Pseudomonadati Pseudomonadota Alphaproteobacteria Rhodobacterales Aestuariibius violaceus WP 367310712.1 338 aa
BacClassVI Pseudomonadati Pseudomonadota Alphaproteobacteria Rhodobacterales Litoreibacter roseus WP 159807085.1 338 aa
BacClassVI Pseudomonadati Pseudomonadota Alphaproteobacteria Rhodobacterales Paracoccaceae bacterium isolate FR12alp1 MFY0692429.1 339 aa
BacClassVI Pseudomonadati Pseudomonadota Alphaproteobacteria Rhodobacterales Litoreibacter albidus WP 089943363.1 338 aa
BacClassVI Pseudomonadati Pseudomonadota Alphaproteobacteria Rhodobacterales uncultured Litoreibacter sp GCF 947487135.1 WP 298298432.1 338 aa
BacClassVI Pseudomonadati Pseudomonadota Alphaproteobacteria Rhodobacterales Litoreibacter sp isolate CAR3 bin 24 MCY4179294.1 338 aa
BacClassVI Pseudomonadati Pseudomonadota Alphaproteobacteria Rhodobacterales Wenxinia marina strain CGMCC 1.6105 WP 018302243.1 339 aa
BacClassVI Pseudomonadati Pseudomonadota Alphaproteobacteria Rhodobacterales Wenxinia saemankumensis WP 073326603.1 340 aa
BacClassVI Pseudomonadati Pseudomonadota Alphaproteobacteria Rhodobacterales Rubellimicrobium sp isolate BM Day8 Bin.38 MCC5975193.1 336 aa
BacClassVI Pseudomonadati Pseudomonadota Alphaproteobacteria Rhodobacterales Pallerionia sp LCG004 WP 317375263.1 339 aa
BacClassVI Pseudomonadati Pseudomonadota Alphaproteobacteria Rhodobacterales Pallerionia pelagia WP 091846255.1 339 aa
BacClassVI Pseudomonadati Pseudomonadota Alphaproteobacteria Rhodobacterales Pallerionia rufa WP 036178271.1 339 aa
BacClassVI Pseudomonadati Pseudomonadota Alphaproteobacteria Rhodobacterales Pallerionia sp THAF1 WP 124110684.1 339 aa
BacClassVI Pseudomonadati Pseudomonadota Alphaproteobacteria Rhodobacterales Pallerionia sp GCF 042089775.1 WP 375263045.1 339 aa
BacClassVI Pseudomonadati Pseudomonadota Alphaproteobacteria Rhodobacterales Pallerionia abyssalis WP 108895586.1 339 aa
BacClassVI Pseudomonadati Pseudomonadota Alphaproteobacteria Rhodobacterales Pallerionia aestuarii WP 111537102.1 339 aa
BacClassVI Pseudomonadati Pseudomonadota Alphaproteobacteria Rhodobacterales Profundibacterium mesophilum WP 159965431.1 339 aa
BacClassVI Pseudomonadati Pseudomonadota Alphaproteobacteria Rhodobacterales Rhodobacteraceae bacterium CCMM004 RVT82813.1 339 aa
BacClassVI Pseudomonadati Pseudomonadota Alphaproteobacteria Rhodobacterales Oceaniovalibus guishaninsula WP 007426823.1 339 aa
BacClassVI Pseudomonadati Pseudomonadota Alphaproteobacteria Rhodobacterales Rhodobacterales bacterium isolate CG 2015 0102 62 8 NCO20581.1 338 aa
BacClassVI Pseudomonadati Pseudomonadota Alphaproteobacteria Rhodobacterales Oceaniglobus trochenteri WP 226782116.1 338 aa
BacClassVI Pseudomonadati Pseudomonadota Alphaproteobacteria Rhodobacterales Pseudohalocynthiibacter sp WP 213888896.1 338 aa
BacClassVI Pseudomonadati Pseudomonadota Alphaproteobacteria Rhodobacterales Paracoccaceae bacterium isolate Diamantina Trench FDZ177 bin3 MGR3661688.1 339 aa
BacClassVI Pseudomonadati Pseudomonadota Alphaproteobacteria Rhodobacterales Marinosulfonomonas sp isolate MAG 55 MGJ8585229.1 339 aa
BacClassVI Pseudomonadati Pseudomonadota Alphaproteobacteria Rhodobacterales Marinosulfonomonas sp isolate NORP110 PHQ98218.1 339 aa
BacClassVI Pseudomonadati Pseudomonadota Alphaproteobacteria Rhodobacterales Paracoccaceae bacterium isolate SCS4bin.60 MEE9428124.1 340 aa
BacClassVI Pseudomonadati Pseudomonadota Alphaproteobacteria Rhodobacterales Paracoccaceae bacterium isolate SCS4bin.36 MEE9387636.1 343 aa
BacClassVI Pseudomonadati Pseudomonadota Alphaproteobacteria Rhodobacterales Marinosulfonomonas sp isolate DC out80 MBV1904216.1 339 aa
BacClassVI Pseudomonadati Pseudomonadota Alphaproteobacteria Rhodobacterales Meinhardsimonia xiamenensis WP 092497755.1 344 aa
BacClassVI Pseudomonadati Pseudomonadota Alphaproteobacteria Rhodobacterales Paracoccaceae bacterium isolate Bpl 10 MCP3969482.1 339 aa
BacClassVI Pseudomonadati Pseudomonadota Alphaproteobacteria Rhodobacterales Rhodobacter sp isolate MO 135.B1 MDJ0823872.1 339 aa
BacClassVI Pseudomonadati Pseudomonadota Alphaproteobacteria Rhodobacterales Pseudoruegeria sp HB172150 WP 172300088.1 339 aa
BacClassVI Pseudomonadati Pseudomonadota Alphaproteobacteria Rhodobacterales Psychromarinibacter sediminicola WP 275567543.1 339 aa
BacClassVI Pseudomonadati Pseudomonadota Alphaproteobacteria Rhodobacterales Brevirhabdus pacifica WP 076979270.1 339 aa
BacClassVI Pseudomonadati Pseudomonadota Alphaproteobacteria Rhodobacterales Oceaniovalibus sp ACAM 378 WP 148471637.1 338 aa
BacClassVI Pseudomonadati Pseudomonadota Alphaproteobacteria Rhodobacterales Oceaniglobus indicus WP 099825581.1 338 aa
BacClassVI Pseudomonadati Pseudomonadota Alphaproteobacteria Rhodobacterales Pallerionia sp KMU 117 WP 431828550.1 338 aa
BacClassVI Pseudomonadati Pseudomonadota Alphaproteobacteria unclassified Alphaproteobacteria Alphaproteobacteria bacterium isolate IDS 49 MBT8476982.1 338 aa
BacClassVI Pseudomonadati Pseudomonadota Alphaproteobacteria Rhodobacterales Ovoidimarina sediminis strain MJ SS7 iso1 WP 316647339.1 338 aa
BacClassVI Pseudomonadati Pseudomonadota Alphaproteobacteria Rhodobacterales uncultured Boseongicola sp GCF 937988555.1 WP 299756958.1 337 aa
BacClassVI Pseudomonadati Pseudomonadota Alphaproteobacteria Rhodobacterales Boseongicola sp isolate MAG seacave 30 MGI9390268.1 339 aa
BacClassVI Pseudomonadati Pseudomonadota Alphaproteobacteria Rhodobacterales Silicimonas sp MF1 12 2 WP 413719594.1 340 aa
BacClassVI Pseudomonadati Pseudomonadota Alphaproteobacteria Rhodobacterales Kangsaoukella pontilimi WP 160763658.1 339 aa
BacClassVI Pseudomonadati Pseudomonadota Alphaproteobacteria Rhodobacterales Silicimonas sp isolate SS bin 34 NNE89589.1 339 aa
BacClassVI Pseudomonadati Pseudomonadota Alphaproteobacteria Rhodobacterales Silicimonas algicola WP 109758340.1 339 aa
BacClassVI Pseudomonadati Pseudomonadota Alphaproteobacteria Rhodobacterales Boseongicola sp isolate IRC4 bin 26 MDE0697960.1 338 aa
BacClassVI Pseudomonadati Pseudomonadota Alphaproteobacteria Rhodobacterales Roseitranquillus sediminis WP 227419870.1 339 aa
BacClassVI Pseudomonadati Pseudomonadota Alphaproteobacteria Rhodobacterales Pacificitalea manganoxidans WP 088663640.1 336 aa
BacClassVI Pseudomonadati Pseudomonadota Alphaproteobacteria unclassified Alphaproteobacteria Alphaproteobacteria bacterium isolate J159 RMD47327.1 345 aa
BacClassVI Pseudomonadati Pseudomonadota Alphaproteobacteria Rhodobacterales Rhodobacterales bacterium isolate ChBW.bin.153 MEJ6500521.1 337 aa
BacClassVI Pseudomonadati Pseudomonadota Alphaproteobacteria Rhodobacterales Paracoccaceae bacterium isolate HKST UBA102 MCA8868236.1 338 aa
BacClassVI Pseudomonadati Pseudomonadota Alphaproteobacteria Rhodobacterales Abyssibius alkaniclasticus WP 274128265.1 338 aa
BacClassVI Pseudomonadati Pseudomonadota Alphaproteobacteria Rhodobacterales Rhodobacterales bacterium isolate EAC638 PQM67419.1 346 aa
BacClassVI Pseudomonadati Pseudomonadota Alphaproteobacteria Rhodobacterales Paracoccaceae bacterium isolate GLR160 NOR63901.1 338 aa
BacClassVI Pseudomonadati Pseudomonadota Alphaproteobacteria Rhodobacterales Actibacterium sp 188UL27 1 WP 204409519.1 336 aa
BacClassVI Pseudomonadati Pseudomonadota Alphaproteobacteria Rhodobacterales Oceanicola sp isolate ALOHA ZT 26 MCH2167737.1 336 aa
BacClassVI Pseudomonadati Pseudomonadota Alphaproteobacteria Rhodobacterales Harenicola maris WP 327794352.1 336 aa
BacClassVI Pseudomonadati Pseudomonadota Alphaproteobacteria Rhodobacterales Tropicimonas omnivorans WP 311690322.1 336 aa
BacClassVI Pseudomonadati Pseudomonadota Alphaproteobacteria Rhodobacterales Tropicimonas isoalkanivorans WP 093362510.1 336 aa
BacClassVI Pseudomonadati Pseudomonadota Alphaproteobacteria Rhodobacterales Tropicimonas sp TH r6 WP 411572469.1 337 aa
BacClassVI Pseudomonadati Pseudomonadota Alphaproteobacteria Rhodobacterales Tropicimonas sp GCF 047972395.1 WP 416953612.1 337 aa
BacClassVI Pseudomonadati Pseudomonadota Alphaproteobacteria Rhodobacterales Tropicimonas sp IMCC34043 WP 116131482.1 336 aa
BacClassVI Pseudomonadati Pseudomonadota Alphaproteobacteria Rhodobacterales Tropicimonas sp GCF 047971885.1 WP 416925098.1 335 aa

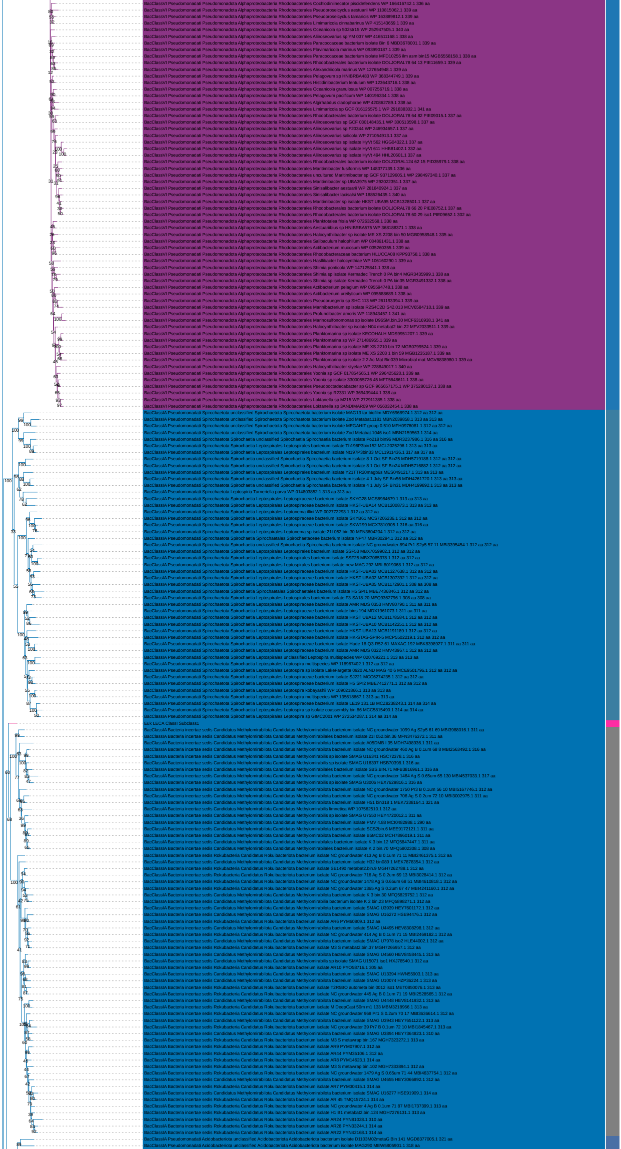
BacClassVI Pseudomonadati Pseudomonadota Alphaproteobacteria Rhodobacterales Cochlodiniinecator piscidefendens WP 166416742.1 336 aa
BacClassVI Pseudomonadati Pseudomonadota Alphaproteobacteria Rhodobacterales Pseudoroseicyclus aestuarii WP 110815062.1 339 aa
BacClassVI Pseudomonadati Pseudomonadota Alphaproteobacteria Rhodobacterales Pseudoroseicyclus tamaricis WP 163889812.1 339 aa
BacClassVI Pseudomonadati Pseudomonadota Alphaproteobacteria Rhodobacterales Limimaricola cinnabarinus WP 415143659.1 339 aa
BacClassVI Pseudomonadati Pseudomonadota Alphaproteobacteria Rhodobacterales Oceanicola sp 502str15 WP 252947505.1 340 aa
BacClassVI Pseudomonadati Pseudomonadota Alphaproteobacteria Rhodobacterales Aliiroseovarius sp YM 037 WP 416511168.1 338 aa
BacClassVI Pseudomonadati Pseudomonadota Alphaproteobacteria Rhodobacterales Paracoccaceae bacterium isolate Bin 6 MBD3678001.1 339 aa
BacClassVI Pseudomonadati Pseudomonadota Alphaproteobacteria Rhodobacterales Flavimaricola marinus WP 093990187.1 339 aa
BacClassVI Pseudomonadati Pseudomonadota Alphaproteobacteria Rhodobacterales Paracoccaceae bacterium isolate MFD10256 ilm asm bin15 MGB5558158.1 338 aa
BacClassVI Pseudomonadati Pseudomonadota Alphaproteobacteria Rhodobacterales Rhodobacterales bacterium isolate DOLJORAL78 64 13 PIE11659.1 339 aa
BacClassVI Pseudomonadati Pseudomonadota Alphaproteobacteria Rhodobacterales Alexandriicola marinus WP 127654948.1 339 aa
BacClassVI Pseudomonadati Pseudomonadota Alphaproteobacteria Rhodobacterales Pelagovum sp HNIBRBA483 WP 368344749.1 339 aa
BacClassVI Pseudomonadati Pseudomonadota Alphaproteobacteria Rhodobacterales Histidinibacterium lentulum WP 123643716.1 338 aa
BacClassVI Pseudomonadati Pseudomonadota Alphaproteobacteria Rhodobacterales Oceanicola granulosus WP 007256719.1 338 aa
BacClassVI Pseudomonadati Pseudomonadota Alphaproteobacteria Rhodobacterales Pelagovum pacificum WP 140196334.1 338 aa
BacClassVI Pseudomonadati Pseudomonadota Alphaproteobacteria Rhodobacterales Algirhabdus cladophorae WP 420862789.1 338 aa
BacClassVI Pseudomonadati Pseudomonadota Alphaproteobacteria Rhodobacterales Limimaricola sp GCF 016125575.1 WP 291838302.1 341 aa
BacClassVI Pseudomonadati Pseudomonadota Alphaproteobacteria Rhodobacterales Rhodobacterales bacterium isolate DOLJORAL78 64 82 PIE09015.1 337 aa
BacClassVI Pseudomonadati Pseudomonadota Alphaproteobacteria Rhodobacterales Aliiroseovarius sp GCF 030148435.1 WP 300513598.1 337 aa
BacClassVI Pseudomonadati Pseudomonadota Alphaproteobacteria Rhodobacterales Aliiroseovarius sp F20344 WP 246934657.1 337 aa
BacClassVI Pseudomonadati Pseudomonadota Alphaproteobacteria Rhodobacterales Aliiroseovarius salicola WP 271054913.1 337 aa
BacClassVI Pseudomonadati Pseudomonadota Alphaproteobacteria Rhodobacterales Aliiroseovarius sp isolate HyVt 562 HGG04322.1 337 aa
BacClassVI Pseudomonadati Pseudomonadota Alphaproteobacteria Rhodobacterales Aliiroseovarius sp isolate HyVt 611 HHB81402.1 332 aa
BacClassVI Pseudomonadati Pseudomonadota Alphaproteobacteria Rhodobacterales Aliiroseovarius sp isolate HyVt 494 HHL20601.1 337 aa
BacClassVI Pseudomonadati Pseudomonadota Alphaproteobacteria Rhodobacterales Rhodobacterales bacterium isolate DOLZORAL124 62 15 PID35979.1 338 aa
BacClassVI Pseudomonadati Pseudomonadota Alphaproteobacteria Rhodobacterales Maritimibacter fusiformis WP 148377139.1 336 aa
BacClassVI Pseudomonadati Pseudomonadota Alphaproteobacteria Rhodobacterales uncultured Maritimibacter sp GCF 937129605.1 WP 298497340.1 337 aa
BacClassVI Pseudomonadati Pseudomonadota Alphaproteobacteria Rhodobacterales Maritimibacter sp UBA3975 WP 292022351.1 337 aa
BacClassVI Pseudomonadati Pseudomonadota Alphaproteobacteria Rhodobacterales Sinisalibacter aestuarii WP 281840924.1 337 aa
BacClassVI Pseudomonadati Pseudomonadota Alphaproteobacteria Rhodobacterales Sinisalibacter lacisalsi WP 188526435.1 340 aa
BacClassVI Pseudomonadati Pseudomonadota Alphaproteobacteria Rhodobacterales Maritimibacter sp isolate HKST UBA95 MCB1328501.1 337 aa
BacClassVI Pseudomonadati Pseudomonadota Alphaproteobacteria Rhodobacterales Rhodobacterales bacterium isolate DOLJORAL78 66 20 PIE08752.1 337 aa
BacClassVI Pseudomonadati Pseudomonadota Alphaproteobacteria Rhodobacterales Rhodobacterales bacterium isolate DOLJORAL78 60 29 iso1 PIE09652.1 302 aa
BacClassVI Pseudomonadati Pseudomonadota Alphaproteobacteria Rhodobacterales Planktotalea frisia WP 072632568.1 338 aa
BacClassVI Pseudomonadati Pseudomonadota Alphaproteobacteria Rhodobacterales Aestuariibius sp HNIBRBA575 WP 368188371.1 338 aa
BacClassVI Pseudomonadati Pseudomonadota Alphaproteobacteria Rhodobacterales Halocynthiibacter sp isolate ME XS 2208 bin 50 MGB0958948.1 335 aa
BacClassVI Pseudomonadati Pseudomonadota Alphaproteobacteria Rhodobacterales Salibaculum halophilum WP 084861431.1 338 aa
BacClassVI Pseudomonadati Pseudomonadota Alphaproteobacteria Rhodobacterales Actibacterium mucosum WP 035260355.1 339 aa
BacClassVI Pseudomonadati Pseudomonadota Alphaproteobacteria Rhodobacterales Rhodobacteraceae bacterium HLUCCA08 KPP93758.1 338 aa
BacClassVI Pseudomonadati Pseudomonadota Alphaproteobacteria Rhodobacterales Hasllibacter halocynthiae WP 106160290.1 339 aa
BacClassVI Pseudomonadati Pseudomonadota Alphaproteobacteria Rhodobacterales Shimia ponticola WP 147125841.1 338 aa
BacClassVI Pseudomonadati Pseudomonadota Alphaproteobacteria Rhodobacterales Shimia sp isolate Kermadec Trench 0 PA bin4 MGR3435999.1 338 aa
BacClassVI Pseudomonadati Pseudomonadota Alphaproteobacteria Rhodobacterales Shimia sp isolate Kermadec Trench 0 PA bin35 MGR3491332.1 338 aa
BacClassVI Pseudomonadati Pseudomonadota Alphaproteobacteria Rhodobacterales Actibacterium pelagium WP 095594748.1 338 aa
BacClassVI Pseudomonadati Pseudomonadota Alphaproteobacteria Rhodobacterales Actibacterium ureilyticum WP 095588689.1 338 aa
BacClassVI Pseudomonadati Pseudomonadota Alphaproteobacteria Rhodobacterales Pseudoruegeria sp SHC 113 WP 261193394.1 339 aa
BacClassVI Pseudomonadati Pseudomonadota Alphaproteobacteria Rhodobacterales Marinibacterium sp isolate R2S4C2D S42.013 MCV6584710.1 339 aa
BacClassVI Pseudomonadati Pseudomonadota Alphaproteobacteria Rhodobacterales Profundibacter amoris WP 118943457.1 341 aa
BacClassVI Pseudomonadati Pseudomonadota Alphaproteobacteria Rhodobacterales Marinosulfonomonas sp isolate D96SM.bin.30 MCF6316938.1 341 aa
BacClassVI Pseudomonadati Pseudomonadota Alphaproteobacteria Rhodobacterales Halocynthiibacter sp isolate N04 metabat2 bin.22 MFV2033511.1 339 aa
BacClassVI Pseudomonadati Pseudomonadota Alphaproteobacteria Rhodobacterales Planktomarina sp isolate KECOHALH MDS9951207.1 339 aa
BacClassVI Pseudomonadati Pseudomonadota Alphaproteobacteria Rhodobacterales Planktomarina sp WP 271486955.1 339 aa
BacClassVI Pseudomonadati Pseudomonadota Alphaproteobacteria Rhodobacterales Planktomarina sp isolate ME XS 2210 bin 72 MGB0799524.1 339 aa
BacClassVI Pseudomonadati Pseudomonadota Alphaproteobacteria Rhodobacterales Planktomarina sp isolate ME XS 2203 1 bin 59 MGB1235187.1 339 aa
BacClassVI Pseudomonadati Pseudomonadota Alphaproteobacteria Rhodobacterales Planktomarina sp isolate 2 2 Ac Mat Bin039 Microbial mat MGV6838980.1 339 aa
BacClassVI Pseudomonadati Pseudomonadota Alphaproteobacteria Rhodobacterales Halocynthiibacter styelae WP 228849017.1 340 aa
BacClassVI Pseudomonadati Pseudomonadota Alphaproteobacteria Rhodobacterales Yoonia sp GCF 017854565.1 WP 296425620.1 339 aa
BacClassVI Pseudomonadati Pseudomonadota Alphaproteobacteria Rhodobacterales Yoonia sp isolate 3300055726 45 MFT5648611.1 338 aa
BacClassVI Pseudomonadati Pseudomonadota Alphaproteobacteria Rhodobacterales Pseudooctadecabacter sp GCF 965657175.1 WP 375280137.1 338 aa
BacClassVI Pseudomonadati Pseudomonadota Alphaproteobacteria Rhodobacterales Yoonia sp R2331 WP 369439444.1 338 aa
BacClassVI Pseudomonadati Pseudomonadota Alphaproteobacteria Rhodobacterales Loktanella sp M215 WP 272911385.1 338 aa
BacClassVI Pseudomonadati Pseudomonadota Alphaproteobacteria Rhodobacterales Loktanella sp 3ANDIMAR09 WP 056032454.1 338 aa
BacClassIA Pseudomonadati Spirochaetota unclassified Spirochaetota Spirochaetota bacterium isolate MAG13 tar biofilm MDY6968974.1 312 aa 312 aa
BacClassIA Pseudomonadati Spirochaetota unclassified Spirochaetota Spirochaetota bacterium isolate Zod Metabat.1181 MBN2039858.1 313 aa 313 aa
BacClassIA Pseudomonadati Spirochaetota unclassified Spirochaetota Spirochaetota bacterium isolate MEGAHIT group 0.510 MFH0976081.1 312 aa 312 aa
BacClassIA Pseudomonadati Spirochaetota unclassified Spirochaetota Spirochaetota bacterium isolate Zod Metabat.1046 iso1 MBN2159563.1 314 aa
BacClassIA Pseudomonadati Spirochaetota Spirochaetia unclassified Spirochaetia Spirochaetia bacterium isolate Po218 bin96 MDR3237986.1 316 aa 316 aa
BacClassIA Pseudomonadati Spirochaetota Spirochaetia Leptospirales Leptospirales bacterium isolate Th196P3bin152 MCL2025296.1 313 aa 313 aa
BacClassIA Pseudomonadati Spirochaetota Spirochaetia Leptospirales Leptospirales bacterium isolate Nt197P3bin33 MCL1911436.1 317 aa 317 aa
BacClassIA Pseudomonadati Spirochaetota Spirochaetia unclassified Spirochaetia Spirochaetia bacterium isolate 8 1 Oct SF Bin25 MDH5719188.1 312 aa 312 aa
BacClassIA Pseudomonadati Spirochaetota Spirochaetia unclassified Spirochaetia Spirochaetia bacterium isolate 8 1 Oct SF Bin24 MDH5716882.1 312 aa 312 aa
BacClassIA Pseudomonadati Spirochaetota Spirochaetia Leptospirales Leptospirales bacterium isolate Y21TTR20magb6s MES0491217.1 313 aa 313 aa
BacClassIA Pseudomonadati Spirochaetota Spirochaetia unclassified Spirochaetia Spirochaetia bacterium isolate 4 1 July SF Bin56 MDH4261720.1 313 aa 313 aa
BacClassIA Pseudomonadati Spirochaetota Spirochaetia unclassified Spirochaetia Spirochaetia bacterium isolate 4 1 July SF Bin31 MDH4199892.1 313 aa 313 aa
BacClassIA Pseudomonadati Spirochaetota Leptospiria Turneriella parva WP 014803852.1 313 aa 313 aa
BacClassIA Pseudomonadati Spirochaetota Spirochaetia Leptospirales Leptospiraceae bacterium isolate SKYG28 MCS6984679.1 313 aa 313 aa
BacClassIA Pseudomonadati Spirochaetota Spirochaetia Leptospirales Leptospiraceae bacterium isolate HKST-UBA14 MCB1200873.1 313 aa 313 aa
BacClassIA Pseudomonadati Spirochaetota Spirochaetia Leptospirales Leptonema illini WP 002772293.1 312 aa 312 aa
BacClassIA Pseudomonadati Spirochaetota Spirochaetia Leptospirales Leptospiraceae bacterium isolate SKYB61 MCS7206236.1 312 aa 312 aa
BacClassIA Pseudomonadati Spirochaetota Spirochaetia Leptospirales Leptospiraceae bacterium isolate SKW199 MCX7810905.1 316 aa 316 aa
BacClassIA Pseudomonadati Spirochaetota Spirochaetia Leptospirales Leptonema sp isolate 21I 052.bin.30 MFN3604204.1 312 aa 312 aa
BacClassIA Pseudomonadati Spirochaetota Spirochaetia Spirochaetales Spirochaetaceae bacterium isolate NP47 MBR30294.1 312 aa 312 aa
BacClassIA Pseudomonadati Spirochaetota Spirochaetia unclassified Spirochaetia Spirochaetia bacterium isolate NC groundwater 894 Pr1 S2p5 57 11 MBI3395454.1 312 aa 312 aa
BacClassIA Pseudomonadati Spirochaetota Spirochaetia Leptospirales Leptospirales bacterium isolate SSF53 MBX7059902.1 312 aa 312 aa
BacClassIA Pseudomonadati Spirochaetota Spirochaetia Leptospirales Leptospirales bacterium isolate SSF25 MBX7085378.1 312 aa 312 aa
BacClassIA Pseudomonadati Spirochaetota Spirochaetia Leptospirales Leptospirales bacterium isolate new MAG 292 MBL8019068.1 312 aa 312 aa
BacClassIA Pseudomonadati Spirochaetota Spirochaetia Leptospirales Leptospiraceae bacterium isolate HKST-UBA03 MCB1327638.1 312 aa 312 aa
BacClassIA Pseudomonadati Spirochaetota Spirochaetia Leptospirales Leptospiraceae bacterium isolate HKST-UBA02 MCB1307392.1 312 aa 312 aa
BacClassIA Pseudomonadati Spirochaetota Spirochaetia Leptospirales Leptospiraceae bacterium isolate HKST-UBA05 MCB1172901.1 308 aa 308 aa
BacClassIA Pseudomonadati Spirochaetota Spirochaetia Spirochaetales Spirochaetales bacterium isolate H5 SPI1 MBE7436846.1 312 aa 312 aa
BacClassIA Pseudomonadati Spirochaetota Spirochaetia Leptospirales Leptospirales bacterium isolate F3-SA18-20 MEQ9362796.1 308 aa 308 aa
BacClassIA Pseudomonadati Spirochaetota Spirochaetia Leptospirales Leptospiraceae bacterium isolate AMR MDS 0353 HMV80790.1 311 aa 311 aa
BacClassIA Pseudomonadati Spirochaetota Spirochaetia Leptospirales Leptospiraceae bacterium isolate bins.194 MDX1961073.1 311 aa 311 aa
BacClassIA Pseudomonadati Spirochaetota Spirochaetia Leptospirales Leptospiraceae bacterium isolate HKST UBA12 MCB1178584.1 312 aa 312 aa
BacClassIA Pseudomonadati Spirochaetota Spirochaetia Leptospirales Leptospiraceae bacterium isolate HKST-UBA10 MCB1142251.1 312 aa 312 aa
BacClassIA Pseudomonadati Spirochaetota Spirochaetia Leptospirales Leptospiraceae bacterium isolate HKST-UBA13 MCB1191189.1 312 aa 312 aa
BacClassIA Pseudomonadati Spirochaetota Spirochaetia Leptospirales Leptospiraceae bacterium isolate HK-STAS-SPIR-5 MCP5502219.1 312 aa 312 aa
BacClassIA Pseudomonadati Spirochaetota Spirochaetia Leptospirales Leptospiraceae bacterium isolate Hade 18-Q3-R52-61 MAXAC.192 MBK8398927.1 311 aa 311 aa
BacClassIA Pseudomonadati Spirochaetota Spirochaetia Leptospirales Leptospiraceae bacterium isolate AMR MDS 0322 HMV43967.1 312 aa 312 aa
BacClassIA Pseudomonadati Spirochaetota Spirochaetia Leptospirales unclassified Leptospira multispecies WP 020769221.1 313 aa 313 aa
BacClassIA Pseudomonadati Spirochaetota Spirochaetia Leptospirales Leptospira multispecies WP 118967402.1 312 aa 312 aa
BacClassIA Pseudomonadati Spirochaetota Spirochaetia Leptospirales Leptospira sp isolate LakeFargette 0920 ALND MAG 40 6 MCE9501796.1 312 aa 312 aa
BacClassIA Pseudomonadati Spirochaetota Spirochaetia Leptospirales Leptospiraceae bacterium isolate SJ221 MCC6274235.1 312 aa 312 aa
BacClassIA Pseudomonadati Spirochaetota Spirochaetia Leptospirales Leptospiraceae bacterium isolate H5 SPI2 MBE7412771.1 312 aa 312 aa
BacClassIA Pseudomonadati Spirochaetota Spirochaetia Leptospirales Leptospira kobayashii WP 109021866.1 313 aa 313 aa
BacClassIA Pseudomonadati Spirochaetota Spirochaetia Leptospirales Leptospira multispecies WP 135618667.1 313 aa 313 aa
BacClassIA Pseudomonadati Spirochaetota Spirochaetia Leptospirales Leptospiraceae bacterium isolate LE19 131.1B MCZ8238243.1 314 aa 314 aa
BacClassIA Pseudomonadati Spirochaetota Spirochaetia Leptospirales Leptospira sp isolate coassembly bin.86 MCC5815490.1 314 aa 314 aa
BacClassIA Pseudomonadati Spirochaetota Spirochaetia Leptospirales Leptospira sp GIMC2001 WP 272534287.1 314 aa 314 aa
Euk LECA ClassI Subclass1
BacClassIA Bacteria incertae sedis Candidatus Methylomirabilota Candidatus Methylomirabilota bacterium isolate NC groundwater 1099 Ag S2p5 61 69 MBI3988016.1 311 aa
BacClassIA Bacteria incertae sedis Candidatus Methylomirabilota Candidatus Methylomirabilales bacterium isolate 21I 052.bin.36 MFN3476372.1 311 aa
BacClassIA Bacteria incertae sedis Candidatus Methylomirabilota Candidatus Methylomirabilota bacterium isolate A05DMB I 35 MDH7498936.1 311 aa
BacClassIA Bacteria incertae sedis Candidatus Methylomirabilota Candidatus Methylomirabilota bacterium isolate NC groundwater 460 Ag B 0.1um 68 8 MBI2563492.1 316 aa
BacClassIA Bacteria incertae sedis Candidatus Methylomirabilota Candidatus Methylomirabilis sp isolate SMAG U16341 HSC72378.1 316 aa
BacClassIA Bacteria incertae sedis Candidatus Methylomirabilota Candidatus Methylomirabilis sp isolate SMAG U16397 HSB70398.1 316 aa
BacClassIA Bacteria incertae sedis Candidatus Methylomirabilota Candidatus Methylomirabilales bacterium isolate SBS.BIN.71 MFB3816961.1 316 aa
BacClassIA Bacteria incertae sedis Candidatus Methylomirabilota Candidatus Methylomirabilota bacterium isolate NC groundwater 1464 Ag S 0.65um 65 130 MBI4537033.1 317 aa
BacClassIA Bacteria incertae sedis Candidatus Methylomirabilota Candidatus Methylomirabilis sp isolate SMAG U3006 HEX7629816.1 316 aa
BacClassIA Bacteria incertae sedis Candidatus Methylomirabilota Candidatus Methylomirabilota bacterium isolate NC groundwater 1750 Pr3 B 0.1um 56 10 MBI5167746.1 312 aa
BacClassIA Bacteria incertae sedis Candidatus Methylomirabilota Candidatus Methylomirabilota bacterium isolate NC groundwater 706 Ag S 0.2um 72 10 MBI3002975.1 311 aa
BacClassIA Bacteria incertae sedis Candidatus Methylomirabilota Candidatus Methylomirabilota bacterium isolate H51 bin318 1 MEK7338164.1 321 aa
BacClassIA Bacteria incertae sedis Candidatus Methylomirabilota Candidatus Methylomirabilis limnetica WP 107562510.1 312 aa
BacClassIA Bacteria incertae sedis Candidatus Methylomirabilota Candidatus Methylomirabilis sp isolate SMAG U7550 HEY4720012.1 311 aa
BacClassIA Bacteria incertae sedis Candidatus Methylomirabilota Candidatus Methylomirabilota bacterium isolate PMV 4.88 MCI0482988.1 290 aa
BacClassIA Bacteria incertae sedis Candidatus Methylomirabilota Candidatus Methylomirabilota bacterium isolate SCS2bin.6 MEE9172121.1 311 aa
BacClassIA Bacteria incertae sedis Candidatus Methylomirabilota Candidatus Methylomirabilota bacterium isolate B5MC02 MCH7896019.1 311 aa
BacClassIA Bacteria incertae sedis Candidatus Methylomirabilota Candidatus Methylomirabilales bacterium isolate K 3 bin.12 MFQ5847447.1 311 aa
BacClassIA Bacteria incertae sedis Candidatus Methylomirabilota Candidatus Methylomirabilales bacterium isolate K 2 bin.70 MFQ5802308.1 308 aa
BacClassIA Bacteria incertae sedis Rokubacteria Candidatus Rokuibacteriota bacterium isolate NC groundwater 413 Ag B 0.1um 71 11 MBI2461375.1 312 aa
BacClassIA Bacteria incertae sedis Candidatus Methylomirabilota Candidatus Methylomirabilota bacterium isolate H32 bin089 1 MEK7878354.1 312 aa
BacClassIA Bacteria incertae sedis Rokubacteria Candidatus Rokuibacteriota bacterium isolate SE1490 metabat2.bin.9 MGH7262788.1 312 aa
BacClassIA Bacteria incertae sedis Rokubacteria Candidatus Rokuibacteriota bacterium isolate NC groundwater 716 Ag S 0.2um 69 13 MBI3028414.1 312 aa
BacClassIA Bacteria incertae sedis Rokubacteria Candidatus Rokuibacteriota bacterium isolate NC groundwater 1478 Ag S 0.65um 68 51 MBI4610818.1 312 aa
BacClassIA Bacteria incertae sedis Rokubacteria Candidatus Rokuibacteriota bacterium isolate NC groundwater 1365 Ag S 0.2um 67 47 MBI4241160.1 312 aa
BacClassIA Bacteria incertae sedis Candidatus Methylomirabilota Candidatus Methylomirabilota bacterium isolate K 3 bin.30 MFQ5829752.1 312 aa
BacClassIA Bacteria incertae sedis Candidatus Methylomirabilota Candidatus Methylomirabilia bacterium isolate K 2 bin.23 MFQ5898271.1 312 aa
BacClassIA Bacteria incertae sedis Candidatus Methylomirabilota Candidatus Methylomirabilota bacterium isolate SMAG U3939 HEY7601172.1 312 aa
BacClassIA Bacteria incertae sedis Candidatus Methylomirabilota Candidatus Methylomirabilota bacterium isolate SMAG U16272 HSE94476.1 312 aa
BacClassIA Bacteria incertae sedis Rokubacteria Candidatus Rokuibacteriota bacterium isolate AR6 PYM60809.1 312 aa
BacClassIA Bacteria incertae sedis Candidatus Methylomirabilota Candidatus Methylomirabilota bacterium isolate SMAG U4495 HEV8308298.1 312 aa
BacClassIA Bacteria incertae sedis Rokubacteria Candidatus Rokuibacteriota bacterium isolate NC groundwater 414 Ag B 0.1um 71 15 MBI2469182.1 312 aa
BacClassIA Bacteria incertae sedis Candidatus Methylomirabilota Candidatus Methylomirabilota bacterium isolate SMAG U7978 iso2 HLE44002.1 312 aa
BacClassIA Bacteria incertae sedis Rokubacteria Candidatus Rokuibacteriota bacterium isolate M3 S metabat2.bin.37 MGH7266957.1 312 aa
BacClassIA Bacteria incertae sedis Candidatus Methylomirabilota Candidatus Methylomirabilota bacterium isolate SMAG U4560 HEV8458445.1 313 aa
BacClassIA Bacteria incertae sedis Candidatus Methylomirabilota Candidatus Methylomirabilis sp isolate SMAG U15071 iso1 HXJ78540.1 312 aa
BacClassIA Bacteria incertae sedis Rokubacteria Candidatus Rokuibacteriota bacterium isolate AR10 PYO58716.1 305 aa
BacClassIA Bacteria incertae sedis Candidatus Methylomirabilota Candidatus Methylomirabilota bacterium isolate SMAG U13394 HWN55903.1 313 aa
BacClassIA Bacteria incertae sedis Candidatus Methylomirabilota Candidatus Methylomirabilota bacterium isolate SMAG U10074 HZP36224.1 313 aa
BacClassIA Bacteria incertae sedis Rokubacteria Candidatus Rokuibacteriota bacterium isolate T2R5BO autometa bin 0012 iso1 MET0850076.1 313 aa
BacClassIA Bacteria incertae sedis Rokubacteria Candidatus Rokuibacteriota bacterium isolate NC groundwater 445 Ag B 0.1um 71 19 MBI2528565.1 312 aa
BacClassIA Bacteria incertae sedis Candidatus Methylomirabilota Candidatus Methylomirabilota bacterium isolate SMAG U4448 HEV8141932.1 313 aa
BacClassIA Bacteria incertae sedis Rokubacteria Candidatus Rokuibacteriota bacterium isolate M DeepCast 50m m1 133 MBM3218966.1 313 aa
BacClassIA Bacteria incertae sedis Rokubacteria Candidatus Rokuibacteriota bacterium isolate NC groundwater 968 Pr1 S 0.2um 70 17 MBI3636614.1 312 aa
BacClassIA Bacteria incertae sedis Candidatus Methylomirabilota Candidatus Methylomirabilota bacterium isolate SMAG U3943 HEY7651122.1 313 aa
BacClassIA Bacteria incertae sedis Rokubacteria Candidatus Rokuibacteriota bacterium isolate NC groundwater 39 Pr7 B 0.1um 72 10 MBI1845467.1 313 aa
BacClassIA Bacteria incertae sedis Candidatus Methylomirabilota Candidatus Methylomirabilota bacterium isolate SMAG U3894 HEY7364823.1 310 aa
BacClassIA Bacteria incertae sedis Rokubacteria Candidatus Rokuibacteriota bacterium isolate M3 S metawrap bin.167 MGH7323272.1 313 aa
BacClassIA Bacteria incertae sedis Rokubacteria Candidatus Rokuibacteriota bacterium isolate AR9 PYM07907.1 312 aa
BacClassIA Bacteria incertae sedis Rokubacteria Candidatus Rokuibacteriota bacterium isolate AR44 PYM35106.1 312 aa
BacClassIA Bacteria incertae sedis Rokubacteria Candidatus Rokuibacteriota bacterium isolate AR8 PYM14623.1 314 aa
BacClassIA Bacteria incertae sedis Rokubacteria Candidatus Rokuibacteriota bacterium isolate M3 S metawrap bin.102 MGH7333894.1 312 aa
BacClassIA Bacteria incertae sedis Rokubacteria Candidatus Rokuibacteriota bacterium isolate NC groundwater 1479 Ag S 0.65um 71 44 MBI4637754.1 312 aa
BacClassIA Bacteria incertae sedis Candidatus Methylomirabilota Candidatus Methylomirabilota bacterium isolate SMAG U4655 HEY3066892.1 312 aa
BacClassIA Bacteria incertae sedis Rokubacteria Candidatus Rokuibacteriota bacterium isolate AR7 PYM30415.1 314 aa
BacClassIA Bacteria incertae sedis Candidatus Methylomirabilota Candidatus Methylomirabilota bacterium isolate SMAG U16277 HSE91909.1 314 aa
BacClassIA Bacteria incertae sedis Rokubacteria Candidatus Rokuibacteriota bacterium isolate AR 45 TMQ15724.1 314 aa
BacClassIA Bacteria incertae sedis Rokubacteria Candidatus Rokuibacteriota bacterium isolate NC groundwater 4 Ag B 0.1um 71 87 MBI1737399.1 313 aa
BacClassIA Bacteria incertae sedis Rokubacteria Candidatus Rokuibacteriota bacterium isolate H1 B1 metabat2.bin.124 MGH7276131.1 313 aa
BacClassIA Bacteria incertae sedis Rokubacteria Candidatus Rokuibacteriota bacterium isolate AR24 PYN81028.1 310 aa
BacClassIA Bacteria incertae sedis Rokubacteria Candidatus Rokuibacteriota bacterium isolate AR28 PYN33244.1 314 aa
BacClassIA Bacteria incertae sedis Rokubacteria Candidatus Rokuibacteriota bacterium isolate AR22 PYN42168.1 314 aa
BacClassIA Pseudomonadati Acidobacteriota unclassified Acidobacteriota Acidobacteriota bacterium isolate D1103M02metaG Bin 141 MGD8377005.1 321 aa
BacClassIA Pseudomonadati Acidobacteriota unclassified Acidobacteriota Acidobacteriota bacterium isolate MAG290 MEW5805901.1 318 aa

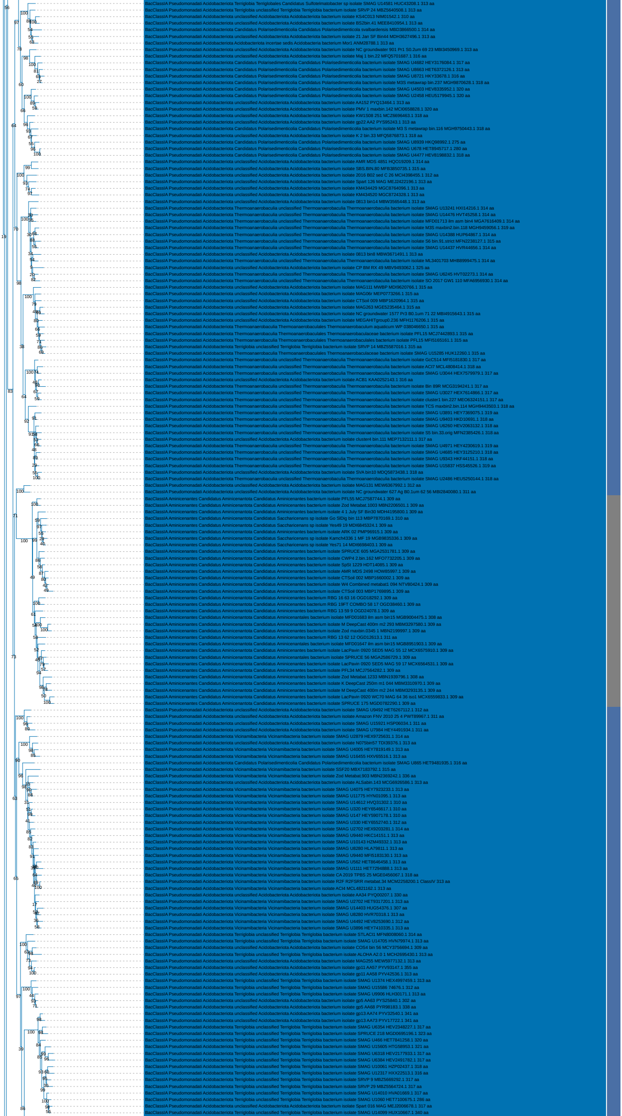

BacClassIA Pseudomonadati Acidobacteriota Terriglobia Terriglobales Candidatus Sulfotelmatobacter sp isolate SMAG U14581 HUC43208.1 313 aa
BacClassIA Pseudomonadati Acidobacteriota Terriglobia unclassified Terriglobia Terriglobia bacterium isolate SRVP 24 MBZ5640508.1 313 aa
BacClassIA Pseudomonadati Acidobacteriota unclassified Acidobacteriota Acidobacteriota bacterium isolate KS4C013 NIM01542.1 310 aa
BacClassIA Pseudomonadati Acidobacteriota unclassified Acidobacteriota Acidobacteriota bacterium isolate BS2bin.41 MEE8410954.1 313 aa
BacClassIA Pseudomonadati Acidobacteriota Candidatus Polarisedimenticolia Candidatus Polarisedimenticola svalbardensis MBD3866500.1 314 aa
BacClassIA Pseudomonadati Acidobacteriota unclassified Acidobacteriota Acidobacteriota bacterium isolate 21 Jan SF Bin44 MDH3627496.1 313 aa
BacClassIA Pseudomonadati Acidobacteriota Acidobacteriota incertae sedis Acidobacteriota bacterium Mor1 ANM28788.1 313 aa
BacClassIA Pseudomonadati Acidobacteriota unclassified Acidobacteriota Acidobacteriota bacterium isolate NC groundwater 901 Pr1 S0.2um 69 23 MBI3450969.1 313 aa
BacClassIA Pseudomonadati Acidobacteriota unclassified Acidobacteriota Acidobacteriota bacterium isolate Maj 1 bin.22 MFQ5701687.1 316 aa
BacClassIA Pseudomonadati Acidobacteriota Candidatus Polarisedimenticolia Candidatus Polarisedimenticolia bacterium isolate SMAG U4682 HEY3176084.1 317 aa
BacClassIA Pseudomonadati Acidobacteriota Candidatus Polarisedimenticolia Candidatus Polarisedimenticolia bacterium isolate SMAG U8663 HET6372126.1 313 aa
BacClassIA Pseudomonadati Acidobacteriota Candidatus Polarisedimenticolia Candidatus Polarisedimenticolia bacterium isolate SMAG U8721 HKY33678.1 316 aa
BacClassIA Pseudomonadati Acidobacteriota Candidatus Polarisedimenticolia Candidatus Polarisedimenticolia bacterium isolate M3S metawrap bin.237 MGH9870628.1 318 aa
BacClassIA Pseudomonadati Acidobacteriota Candidatus Polarisedimenticolia Candidatus Polarisedimenticolia bacterium isolate SMAG U4503 HEV8335952.1 320 aa
BacClassIA Pseudomonadati Acidobacteriota Candidatus Polarisedimenticolia Candidatus Polarisedimenticolia bacterium isolate SMAG U2458 HEU5179945.1 320 aa
BacClassIA Pseudomonadati Acidobacteriota unclassified Acidobacteriota Acidobacteriota bacterium isolate AA152 PYQ13464.1 313 aa
BacClassIA Pseudomonadati Acidobacteriota unclassified Acidobacteriota Acidobacteriota bacterium isolate PMV 1 maxbin.142 MCI0658828.1 320 aa
BacClassIA Pseudomonadati Acidobacteriota unclassified Acidobacteriota Acidobacteriota bacterium isolate KW1S08 251 MCZ6696463.1 318 aa
BacClassIA Pseudomonadati Acidobacteriota unclassified Acidobacteriota Acidobacteriota bacterium isolate gp22 AA2 PYS95243.1 313 aa
BacClassIA Pseudomonadati Acidobacteriota Candidatus Polarisedimenticolia Candidatus Polarisedimenticolia bacterium isolate M3 S metawrap bin.116 MGH9750443.1 318 aa
BacClassIA Pseudomonadati Acidobacteriota unclassified Acidobacteriota Acidobacteriota bacterium isolate K 2 bin.33 MFQ5876873.1 318 aa
BacClassIA Pseudomonadati Acidobacteriota Candidatus Polarisedimenticolia Candidatus Polarisedimenticolia bacterium isolate SMAG U8939 HKQ98992.1 275 aa
BacClassIA Pseudomonadati Acidobacteriota Candidatus Polarisedimenticolia Candidatus Polarisedimenticolia bacterium isolate SMAG U678 HET8945717.1 280 aa
BacClassIA Pseudomonadati Acidobacteriota Candidatus Polarisedimenticolia Candidatus Polarisedimenticolia bacterium isolate SMAG U4477 HEV8198832.1 318 aa
BacClassIA Pseudomonadati Acidobacteriota unclassified Acidobacteriota Acidobacteriota bacterium isolate AMR MDS 4851 HQO19209.1 314 aa
BacClassIA Pseudomonadati Acidobacteriota unclassified Acidobacteriota Acidobacteriota bacterium isolate SBS.BIN.80 MFB3850735.1 315 aa
BacClassIA Pseudomonadati Acidobacteriota unclassified Acidobacteriota Acidobacteriota bacterium isolate 2016 B02 sed C 26 MCI4398455.1 312 aa
BacClassIA Pseudomonadati Acidobacteriota unclassified Acidobacteriota Acidobacteriota bacterium isolate Spart 126 MAG MEJ2422196.1 313 aa
BacClassIA Pseudomonadati Acidobacteriota unclassified Acidobacteriota Acidobacteriota bacterium isolate KM434429 MGC8764096.1 313 aa
BacClassIA Pseudomonadati Acidobacteriota unclassified Acidobacteriota Acidobacteriota bacterium isolate KM434520 MGC8724328.1 313 aa
BacClassIA Pseudomonadati Acidobacteriota unclassified Acidobacteriota Acidobacteriota bacterium isolate 0813 bin14 MBW3565448.1 313 aa
BacClassIA Pseudomonadati Acidobacteriota Thermoanaerobaculia unclassified Thermoanaerobaculia Thermoanaerobaculia bacterium isolate SMAG U13241 HXI14216.1 314 aa
BacClassIA Pseudomonadati Acidobacteriota Thermoanaerobaculia unclassified Thermoanaerobaculia Thermoanaerobaculia bacterium isolate SMAG U14476 HVT45258.1 314 aa
BacClassIA Pseudomonadati Acidobacteriota Thermoanaerobaculia unclassified Thermoanaerobaculia Thermoanaerobaculia bacterium isolate MFD01713 llm asm bin4 MGA7616409.1 314 aa
BacClassIA Pseudomonadati Acidobacteriota Thermoanaerobaculia unclassified Thermoanaerobaculia Thermoanaerobaculia bacterium isolate M3S maxbin2.bin.118 MGH9459056.1 319 aa
BacClassIA Pseudomonadati Acidobacteriota Thermoanaerobaculia unclassified Thermoanaerobaculia Thermoanaerobaculia bacterium isolate SMAG U14388 HUP64867.1 314 aa
BacClassIA Pseudomonadati Acidobacteriota Thermoanaerobaculia unclassified Thermoanaerobaculia Thermoanaerobaculia bacterium isolate S6 bin.91.strict MFN2238127.1 315 aa
BacClassIA Pseudomonadati Acidobacteriota Thermoanaerobaculia unclassified Thermoanaerobaculia Thermoanaerobaculia bacterium isolate SMAG U14437 HVR44656.1 314 aa
BacClassIA Pseudomonadati Acidobacteriota unclassified Acidobacteriota Acidobacteriota bacterium isolate 0813 bin8 MBW3671491.1 313 aa
BacClassIA Pseudomonadati Acidobacteriota Thermoanaerobaculia unclassified Thermoanaerobaculia Thermoanaerobaculia bacterium isolate ML3401703 MHB8999475.1 314 aa
BacClassIA Pseudomonadati Acidobacteriota unclassified Acidobacteriota Acidobacteriota bacterium isolate CP BM RX 49 MBV9493062.1 325 aa
BacClassIA Pseudomonadati Acidobacteriota Thermoanaerobaculia unclassified Thermoanaerobaculia Thermoanaerobaculia bacterium isolate SMAG U6245 HVT02273.1 314 aa
BacClassIA Pseudomonadati Acidobacteriota Thermoanaerobaculia unclassified Thermoanaerobaculia Thermoanaerobaculia bacterium isolate SO 2017 GW1 110 MFA6956930.1 314 aa
BacClassIA Pseudomonadati Acidobacteriota unclassified Acidobacteriota Acidobacteriota bacterium isolate MAG111 MWBP MDI9620766.1 315 aa
BacClassIA Pseudomonadati Acidobacteriota unclassified Acidobacteriota Acidobacteriota bacterium isolate MAG06r MEP0773266.1 315 aa
BacClassIA Pseudomonadati Acidobacteriota unclassified Acidobacteriota Acidobacteriota bacterium isolate CTSoil 009 MBP1620964.1 315 aa
BacClassIA Pseudomonadati Acidobacteriota unclassified Acidobacteriota Acidobacteriota bacterium isolate MAG263 MGE5235464.1 315 aa
BacClassIA Pseudomonadati Acidobacteriota unclassified Acidobacteriota Acidobacteriota bacterium isolate NC groundwater 1577 Pr3 B0.1um 71 22 MBI4915643.1 315 aa
BacClassIA Pseudomonadati Acidobacteriota unclassified Acidobacteriota Acidobacteriota bacterium isolate MEGAHITgroup0.236 MFH1176206.1 315 aa
BacClassIA Pseudomonadati Acidobacteriota Thermoanaerobaculia Thermoanaerobaculales Thermoanaerobaculum aquaticum WP 038046650.1 315 aa
BacClassIA Pseudomonadati Acidobacteriota Thermoanaerobaculia Thermoanaerobaculales Thermoanaerobaculaceae bacterium isolate PFL15 MCJ7442893.1 315 aa
BacClassIA Pseudomonadati Acidobacteriota Thermoanaerobaculia Thermoanaerobaculales Thermoanaerobaculales bacterium isolate PFL15 MFI5165161.1 315 aa
BacClassIA Pseudomonadati Acidobacteriota Terriglobia unclassified Terriglobia Terriglobia bacterium isolate SRVP 14 MBZ5587016.1 315 aa
BacClassIA Pseudomonadati Acidobacteriota Thermoanaerobaculia Thermoanaerobaculales Thermoanaerobaculaceae bacterium isolate SMAG U15285 HUK12260.1 315 aa
BacClassIA Pseudomonadati Acidobacteriota Thermoanaerobaculia unclassified Thermoanaerobaculia Thermoanaerobaculia bacterium isolate GcC514 MFI5181830.1 317 aa
BacClassIA Pseudomonadati Acidobacteriota Thermoanaerobaculia unclassified Thermoanaerobaculia Thermoanaerobaculia bacterium isolate ACI7 MCL4808414.1 318 aa
BacClassIA Pseudomonadati Acidobacteriota Thermoanaerobaculia unclassified Thermoanaerobaculia Thermoanaerobaculia bacterium isolate SMAG U3044 HEX7579979.1 317 aa
BacClassIA Pseudomonadati Acidobacteriota unclassified Acidobacteriota Acidobacteriota bacterium isolate ACB1 KAA0252143.1 316 aa
BacClassIA Pseudomonadati Acidobacteriota Thermoanaerobaculia unclassified Thermoanaerobaculia Thermoanaerobaculia bacterium isolate Bin 89R MCG3194241.1 317 aa
BacClassIA Pseudomonadati Acidobacteriota Thermoanaerobaculia unclassified Thermoanaerobaculia Thermoanaerobaculia bacterium isolate SMAG U3027 HEX7614866.1 317 aa
BacClassIA Pseudomonadati Acidobacteriota Thermoanaerobaculia unclassified Thermoanaerobaculia Thermoanaerobaculia bacterium isolate cluster1 bin.227 MEO6324151.1 317 aa
BacClassIA Pseudomonadati Acidobacteriota Thermoanaerobaculia unclassified Thermoanaerobaculia Thermoanaerobaculia bacterium isolate TCS maxbin2.bin.114 MGH9443503.1 318 aa
BacClassIA Pseudomonadati Acidobacteriota Thermoanaerobaculia unclassified Thermoanaerobaculia Thermoanaerobaculia bacterium isolate SMAG U3891 HEY7369075.1 319 aa
BacClassIA Pseudomonadati Acidobacteriota Thermoanaerobaculia unclassified Thermoanaerobaculia Thermoanaerobaculia bacterium isolate SMAG U9403 HKD10691.1 318 aa
BacClassIA Pseudomonadati Acidobacteriota Thermoanaerobaculia unclassified Thermoanaerobaculia Thermoanaerobaculia bacterium isolate SMAG U6260 HEV2063132.1 318 aa
BacClassIA Pseudomonadati Acidobacteriota Thermoanaerobaculia unclassified Thermoanaerobaculia Thermoanaerobaculia bacterium isolate S5 bin.33.orig MFN2385426.1 318 aa
BacClassIA Pseudomonadati Acidobacteriota unclassified Acidobacteriota Acidobacteriota bacterium isolate cluster4 bin.111 MEP7132111.1 317 aa
BacClassIA Pseudomonadati Acidobacteriota Thermoanaerobaculia unclassified Thermoanaerobaculia Thermoanaerobaculia bacterium isolate SMAG U4971 HEY4230619.1 319 aa
BacClassIA Pseudomonadati Acidobacteriota Thermoanaerobaculia unclassified Thermoanaerobaculia Thermoanaerobaculia bacterium isolate SMAG U4685 HEY3125210.1 318 aa
BacClassIA Pseudomonadati Acidobacteriota Thermoanaerobaculia unclassified Thermoanaerobaculia Thermoanaerobaculia bacterium isolate SMAG U9343 HKF44151.1 318 aa
BacClassIA Pseudomonadati Acidobacteriota Thermoanaerobaculia unclassified Thermoanaerobaculia Thermoanaerobaculia bacterium isolate SMAG U15837 HSS45526.1 319 aa
BacClassIA Pseudomonadati Acidobacteriota unclassified Acidobacteriota Acidobacteriota bacterium isolate SVA bin10 MDQ5873438.1 318 aa
BacClassIA Pseudomonadati Acidobacteriota Thermoanaerobaculia unclassified Thermoanaerobaculia Thermoanaerobaculia bacterium isolate SMAG U2486 HEU5250144.1 318 aa
BacClassIA Pseudomonadati Acidobacteriota unclassified Acidobacteriota Acidobacteriota bacterium isolate MAG131 MEW6367992.1 312 aa
BacClassIA Pseudomonadati Acidobacteriota unclassified Acidobacteriota Acidobacteriota bacterium isolate NC groundwater 627 Ag B0.1um 62 56 MBI2840080.1 311 aa
BacClassIA Aminicenantes Candidatus Aminicenantota Candidatus Aminicenantes bacterium isolate PFL55 MCJ7587744.1 309 aa
BacClassIA Aminicenantes Candidatus Aminicenantota Candidatus Aminicenantes bacterium isolate Zod Metabat.1003 MBN2206501.1 309 aa
BacClassIA Aminicenantes Candidatus Aminicenantota Candidatus Aminicenantes bacterium isolate 4 1 July SF Bin30 MDH4195800.1 309 aa
BacClassIA Aminicenantes Candidatus Aminicenantota Candidatus Saccharicenans sp isolate Go SlDig bin 113 MBP7870169.1 310 aa
BacClassIA Aminicenantes Candidatus Aminicenantota Candidatus Saccharicenans sp isolate Yes49 19 MDI6845324.1 309 aa
BacClassIA Aminicenantes Candidatus Aminicenantota Candidatus Aminicenantes bacterium isolate ARK 02 PMP96915.1 309 aa
BacClassIA Aminicenantes Candidatus Aminicenantota Candidatus Saccharicenans sp isolate Kamch4336 1 MF 19 MGB9835336.1 309 aa
BacClassIA Aminicenantes Candidatus Aminicenantota Candidatus Saccharicenans sp isolate Yes71 14 MDI6698403.1 309 aa
BacClassIA Aminicenantes Candidatus Aminicenantota Candidatus Aminicenantes bacterium isolate SPRUCE 605 MGA2531781.1 309 aa
BacClassIA Aminicenantes Candidatus Aminicenantota Candidatus Aminicenantes bacterium isolate CWP4 2.bin.162 MFO7732205.1 309 aa
BacClassIA Aminicenantes Candidatus Aminicenantota Candidatus Aminicenantes bacterium isolate SpSt 1229 HDT14085.1 309 aa
BacClassIA Aminicenantes Candidatus Aminicenantota Candidatus Aminicenantes bacterium isolate AMR MDS 2498 HOW85997.1 309 aa
BacClassIA Aminicenantes Candidatus Aminicenantota Candidatus Aminicenantes bacterium isolate CTSoil 002 MBP1660002.1 309 aa
BacClassIA Aminicenantes Candidatus Aminicenantota Candidatus Aminicenantes bacterium isolate W4 Combined metabat1 094 NTV80424.1 309 aa
BacClassIA Aminicenantes Candidatus Aminicenantota Candidatus Aminicenantes bacterium isolate CTSoil 003 MBP1769895.1 309 aa
BacClassIA Aminicenantes Candidatus Aminicenantota Candidatus Aminicenantes bacterium RBG 16 63 16 OGD18292.1 309 aa
BacClassIA Aminicenantes Candidatus Aminicenantota Candidatus Aminicenantes bacterium RBG 19FT COMBO 58 17 OGD38460.1 309 aa
BacClassIA Aminicenantes Candidatus Aminicenantota Candidatus Aminicenantes bacterium RBG 13 59 9 OGD24078.1 309 aa
BacClassIA Aminicenantes Candidatus Aminicenantota Candidatus Aminicenantales bacterium isolate MFD01683 llm asm bin15 MGB9004475.1 308 aa
BacClassIA Aminicenantes Candidatus Aminicenantota Candidatus Aminicenantes bacterium isolate M DeepCast 400m m2 293 MBM3297580.1 309 aa
BacClassIA Aminicenantes Candidatus Aminicenantota Candidatus Aminicenantes bacterium isolate Zod maxbin.0345 1 MBN2199997.1 309 aa
BacClassIA Aminicenantes Candidatus Aminicenantota Candidatus Aminicenantes bacterium RBG 13 62 12 OGD12613.1 311 aa
BacClassIA Aminicenantes Candidatus Aminicenantota Candidatus Aminicenantales bacterium isolate MFD01647 llm asm bin15 MGB8951903.1 309 aa
BacClassIA Aminicenantes Candidatus Aminicenantota Candidatus Aminicenantes bacterium isolate LacPavin 0920 SED5 MAG 55 12 MCX6575910.1 309 aa
BacClassIA Aminicenantes Candidatus Aminicenantota Candidatus Aminicenantales bacterium isolate SPRUCE 56 MGA2586729.1 309 aa
BacClassIA Aminicenantes Candidatus Aminicenantota Candidatus Aminicenantes bacterium isolate LacPavin 0920 SED5 MAG 59 17 MCX6564531.1 309 aa
BacClassIA Aminicenantes Candidatus Aminicenantota Candidatus Aminicenantes bacterium isolate PFL34 MCJ7564282.1 309 aa
BacClassIA Aminicenantes Candidatus Aminicenantota Candidatus Aminicenantes bacterium isolate Zod Metabat.1233 MBN1939796.1 308 aa
BacClassIA Aminicenantes Candidatus Aminicenantota Candidatus Aminicenantes bacterium isolate K DeepCast 250m m1 044 MBM3310970.1 309 aa
BacClassIA Aminicenantes Candidatus Aminicenantota Candidatus Aminicenantes bacterium isolate M DeepCast 400m m2 244 MBM3293135.1 309 aa
BacClassIA Aminicenantes Candidatus Aminicenantota Candidatus Aminicenantes bacterium isolate LacPavin 0920 WC70 MAG 64 36 iso1 MCX6559833.1 309 aa
BacClassIA Aminicenantes Candidatus Aminicenantota Candidatus Aminicenantes bacterium isolate SPRUCE 175 MGD0782290.1 309 aa
BacClassIA Pseudomonadati Acidobacteriota unclassified Acidobacteriota Acidobacteriota bacterium isolate SMAG U9492 HET6267112.1 312 aa
BacClassIA Pseudomonadati Acidobacteriota unclassified Acidobacteriota Acidobacteriota bacterium isolate Amazon FNV 2010 25 4 PWT89967.1 311 aa
BacClassIA Pseudomonadati Acidobacteriota unclassified Acidobacteriota Acidobacteriota bacterium isolate SMAG U15921 HSP06034.1 311 aa
BacClassIA Pseudomonadati Acidobacteriota unclassified Acidobacteriota Acidobacteriota bacterium isolate SMAG U7984 HEY4491934.1 311 aa
BacClassIA Pseudomonadati Acidobacteriota Vicinamibacteria Vicinamibacteria bacterium isolate SMAG U2879 HEX9725631.1 314 aa
BacClassIA Pseudomonadati Acidobacteriota unclassified Acidobacteriota Acidobacteriota bacterium isolate N075bin57 TDI39376.1 313 aa
BacClassIA Pseudomonadati Acidobacteriota Vicinamibacteria Vicinamibacteria bacterium isolate SMAG U4005 HEY7819149.1 313 aa
BacClassIA Pseudomonadati Acidobacteriota Vicinamibacteria Vicinamibacteria bacterium isolate SMAG U16455 HXV65516.1 313 aa
BacClassIA Pseudomonadati Acidobacteriota Candidatus Polarisedimenticolia Candidatus Polarisedimenticolia bacterium isolate SMAG U865 HET9481935.1 316 aa
BacClassIA Pseudomonadati Acidobacteriota Vicinamibacteria Vicinamibacteria bacterium isolate SSF20 MBX7183792.1 315 aa
BacClassIA Pseudomonadati Acidobacteriota Vicinamibacteria Vicinamibacteria bacterium isolate Zod Metabat.903 MBN2369242.1 336 aa
BacClassIA Pseudomonadati Acidobacteriota unclassified Acidobacteriota Acidobacteriota bacterium isolate ALSabin.143 MCG6926586.1 313 aa
BacClassIA Pseudomonadati Acidobacteriota Vicinamibacteria Vicinamibacteria bacterium isolate SMAG U4075 HEY7923233.1 313 aa
BacClassIA Pseudomonadati Acidobacteriota Vicinamibacteria Vicinamibacteria bacterium isolate SMAG U11775 HYN01095.1 313 aa
BacClassIA Pseudomonadati Acidobacteriota Vicinamibacteria Vicinamibacteria bacterium isolate SMAG U14612 HVQ31302.1 310 aa
BacClassIA Pseudomonadati Acidobacteriota Vicinamibacteria Vicinamibacteria bacterium isolate SMAG U320 HEY6546617.1 310 aa
BacClassIA Pseudomonadati Acidobacteriota Vicinamibacteria Vicinamibacteria bacterium isolate SMAG U147 HEY5907178.1 310 aa
BacClassIA Pseudomonadati Acidobacteriota Vicinamibacteria Vicinamibacteria bacterium isolate SMAG U330 HEY6552740.1 312 aa
BacClassIA Pseudomonadati Acidobacteriota Vicinamibacteria Vicinamibacteria bacterium isolate SMAG U2702 HEX9203281.1 314 aa
BacClassIA Pseudomonadati Acidobacteriota Vicinamibacteria Vicinamibacteria bacterium isolate SMAG U9440 HKC14151.1 313 aa
BacClassIA Pseudomonadati Acidobacteriota Vicinamibacteria Vicinamibacteria bacterium isolate SMAG U10143 HZM49332.1 313 aa
BacClassIA Pseudomonadati Acidobacteriota Vicinamibacteria Vicinamibacteria bacterium isolate SMAG U8280 HLA79811.1 313 aa
BacClassIA Pseudomonadati Acidobacteriota Vicinamibacteria Vicinamibacteria bacterium isolate SMAG U9440 MFI5183130.1 313 aa
BacClassIA Pseudomonadati Acidobacteriota Vicinamibacteria Vicinamibacteria bacterium isolate SMAG U562 HET8646458.1 313 aa
BacClassIA Pseudomonadati Acidobacteriota Vicinamibacteria Vicinamibacteria bacterium isolate SMAG U1111 HET7294888.1 313 aa
BacClassIA Pseudomonadati Acidobacteriota Vicinamibacteria Vicinamibacteria bacterium isolate CA 2019 TPBS 25 MGE0456067.1 318 aa
BacClassIA Pseudomonadati Acidobacteriota Vicinamibacteria Vicinamibacteria bacterium isolate R2F R2FSRR metabat.34 MCM2258200.1 ClassIV 313 aa
BacClassIA Pseudomonadati Acidobacteriota Vicinamibacteria Vicinamibacteria bacterium isolate ACI4 MCL4821162.1 313 aa
BacClassIA Pseudomonadati Acidobacteriota unclassified Acidobacteriota Acidobacteriota bacterium isolate AA34 PYQ00207.1 330 aa
BacClassIA Pseudomonadati Acidobacteriota Vicinamibacteria Vicinamibacteria bacterium isolate SMAG U2702 HET9317201.1 313 aa
BacClassIA Pseudomonadati Acidobacteriota Vicinamibacteria Vicinamibacteria bacterium isolate SMAG U14403 HUG54376.1 307 aa
BacClassIA Pseudomonadati Acidobacteriota Vicinamibacteria Vicinamibacteria bacterium isolate SMAG U8280 HVR70318.1 313 aa
BacClassIA Pseudomonadati Acidobacteriota Vicinamibacteria Vicinamibacteria bacterium isolate SMAG U4492 HEV8253690.1 312 aa
BacClassIA Pseudomonadati Acidobacteriota Vicinamibacteria Vicinamibacteria bacterium isolate SMAG U3896 HEY7410335.1 313 aa
BacClassIA Pseudomonadati Acidobacteriota Terriglobia unclassified Terriglobia Terriglobia bacterium isolate STLACI1 MFN8008060.1 314 aa
BacClassIA Pseudomonadati Acidobacteriota Terriglobia unclassified Terriglobia Terriglobia bacterium isolate SMAG U14705 HVN79974.1 313 aa
BacClassIA Pseudomonadati Acidobacteriota unclassified Acidobacteriota Acidobacteriota bacterium isolate COS4 bin 56 MCY3756694.1 309 aa
BacClassIA Pseudomonadati Acidobacteriota Terriglobia unclassified Terriglobia Terriglobia bacterium isolate ALOHA A2.0 1 MCH2695430.1 313 aa
BacClassIA Pseudomonadati Acidobacteriota unclassified Acidobacteriota Acidobacteriota bacterium isolate MAG255 MEW5977132.1 313 aa
BacClassIA Pseudomonadati Acidobacteriota unclassified Acidobacteriota Acidobacteriota bacterium isolate gp11 AA57 PYV93147.1 355 aa
BacClassIA Pseudomonadati Acidobacteriota unclassified Acidobacteriota Acidobacteriota bacterium isolate gp11 AA58 PYV42536.1 313 aa
BacClassIA Pseudomonadati Acidobacteriota Terriglobia unclassified Terriglobia Terriglobia bacterium isolate SMAG U1374 HEX4997459.1 313 aa
BacClassIA Pseudomonadati Acidobacteriota Terriglobia unclassified Terriglobia Terriglobia bacterium isolate SMAG U15586 74676.1 312 aa
BacClassIA Pseudomonadati Acidobacteriota Terriglobia unclassified Terriglobia Terriglobia bacterium isolate SMAG U9906 HLH30171.1 313 aa
BacClassIA Pseudomonadati Acidobacteriota unclassified Acidobacteriota Acidobacteriota bacterium isolate gp5 AA63 PYS25840.1 302 aa
BacClassIA Pseudomonadati Acidobacteriota unclassified Acidobacteriota Acidobacteriota bacterium isolate gp5 AA68 PYR98183.1 338 aa
BacClassIA Pseudomonadati Acidobacteriota unclassified Acidobacteriota Acidobacteriota bacterium isolate gp13 AA74 PYV32540.1 341 aa
BacClassIA Pseudomonadati Acidobacteriota unclassified Acidobacteriota Acidobacteriota bacterium isolate gp13 AA73 PYV17722.1 341 aa
BacClassIA Pseudomonadati Acidobacteriota Terriglobia unclassified Terriglobia Terriglobia bacterium isolate SMAG U6354 HEV2348227.1 317 aa
BacClassIA Pseudomonadati Acidobacteriota Terriglobia unclassified Terriglobia Terriglobia bacterium isolate SPRUCE 218 MGD0695196.1 323 aa
BacClassIA Pseudomonadati Acidobacteriota Terriglobia unclassified Terriglobia Terriglobia bacterium isolate SMAG U466 HET7841258.1 320 aa
BacClassIA Pseudomonadati Acidobacteriota Terriglobia unclassified Terriglobia Terriglobia bacterium isolate SMAG U15605 HTG58953.1 321 aa
BacClassIA Pseudomonadati Acidobacteriota Terriglobia unclassified Terriglobia Terriglobia bacterium isolate SMAG U6318 HEV2177933.1 317 aa
BacClassIA Pseudomonadati Acidobacteriota Terriglobia unclassified Terriglobia Terriglobia bacterium isolate SMAG U6384 HEV2491782.1 317 aa
BacClassIA Pseudomonadati Acidobacteriota Terriglobia unclassified Terriglobia Terriglobia bacterium isolate SMAG U10061 HZP02437.1 318 aa
BacClassIA Pseudomonadati Acidobacteriota Terriglobia unclassified Terriglobia Terriglobia bacterium isolate SMAG U12317 HXX22513.1 316 aa
BacClassIA Pseudomonadati Acidobacteriota Terriglobia unclassified Terriglobia Terriglobia bacterium isolate SRVP 9 MBZ5669292.1 317 aa
BacClassIA Pseudomonadati Acidobacteriota Terriglobia unclassified Terriglobia Terriglobia bacterium isolate SRVP 29 MBZ5564724.1 317 aa
BacClassIA Pseudomonadati Acidobacteriota Terriglobia unclassified Terriglobia Terriglobia bacterium isolate SMAG U14010 HVA01669.1 317 aa
BacClassIA Pseudomonadati Acidobacteriota Terriglobia unclassified Terriglobia Terriglobia bacterium isolate SMAG U1060 HET7100675.1 286 aa
BacClassIA Pseudomonadati Acidobacteriota Terriglobia unclassified Terriglobia Terriglobia bacterium isolate Spart 016 MAG MEJ2006678.1 317 aa
BacClassIA Pseudomonadati Acidobacteriota Terriglobia unclassified Terriglobia Terriglobia bacterium isolate SMAG U14099 HUX10667.1 340 aa

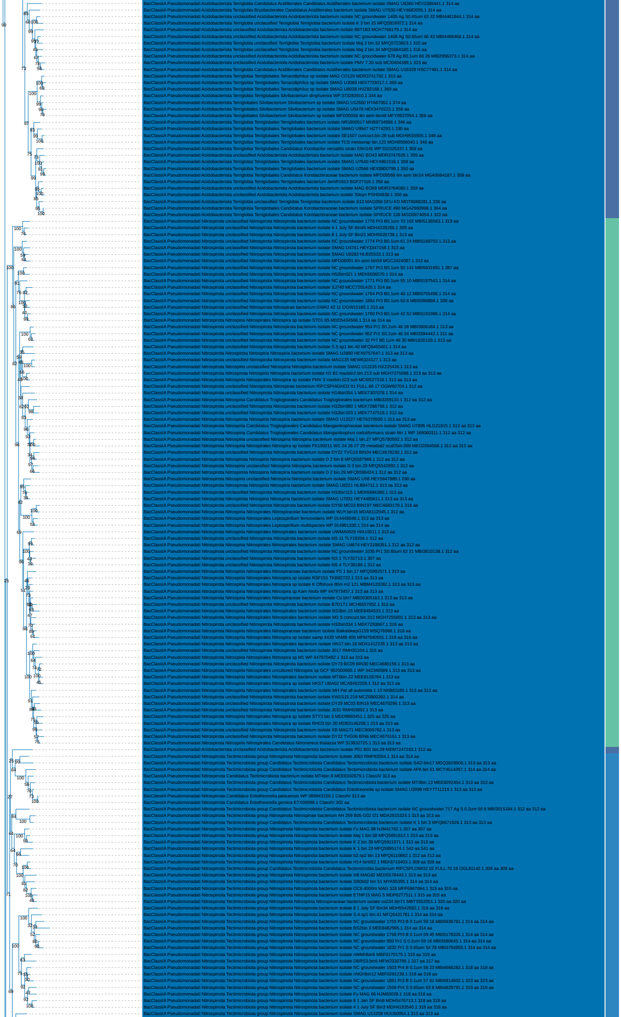

BacClassIA Pseudomonadati Acidobacteriota Terriglobia Candidatus Acidiferrales Candidatus Acidiferrales bacterium isolate SMAG U6360 HEV2386441.1 314 aa
BacClassIA Pseudomonadati Acidobacteriota Terriglobia Bryobacterales Candidatus Acidiferrales bacterium isolate SMAG U7530 HEY4682055.1 314 aa
BacClassIA Pseudomonadati Acidobacteriota unclassified Acidobacteriota Acidobacteriota bacterium isolate NC groundwater 1405 Ag S0.65um 63 32 MBI4461844.1 314 aa
BacClassIA Pseudomonadati Acidobacteriota Terriglobia unclassified Terriglobia Terriglobia bacterium isolate K 3 bin.15 MFQ5816972.1 314 aa
BacClassIA Pseudomonadati Acidobacteriota unclassified Acidobacteriota Acidobacteriota bacterium isolate B6T1B3 MCH7766179.1 314 aa
BacClassIA Pseudomonadati Acidobacteriota unclassified Acidobacteriota Acidobacteriota bacterium isolate NC groundwater 1408 Ag S0.65um 66 43 MBI4466466.1 314 aa
BacClassIA Pseudomonadati Acidobacteriota Terriglobia unclassified Terriglobia Terriglobia bacterium isolate Maj 2 bin.52 MFQ5723823.1 315 aa
BacClassIA Pseudomonadati Acidobacteriota Terriglobia unclassified Terriglobia Terriglobia bacterium isolate Maj 2 bin.34 MFQ5664180.1 316 aa
BacClassIA Pseudomonadati Acidobacteriota unclassified Acidobacteriota Acidobacteriota bacterium isolate NC groundwater 678 Ag B0.1um 66 26 MBI2956373.1 314 aa
BacClassIA Pseudomonadati Acidobacteriota unclassified Acidobacteriota Acidobacteriota bacterium isolate PMV 7.20 sub MCI0404369.1 323 aa
BacClassIA Pseudomonadati Acidobacteriota Terriglobia Candidatus Acidiferrales Candidatus Acidiferrales bacterium isolate SMAG U16329 HSC77491.1 314 aa
BacClassIA Pseudomonadati Acidobacteriota Terriglobia Terriglobales Terracidiphilus sp isolate MAG CO120 MDR3741702.1 315 aa
BacClassIA Pseudomonadati Acidobacteriota Terriglobia Terriglobales Terracidiphilus sp isolate SMAG U3069 HEX7729217.1 369 aa
BacClassIA Pseudomonadati Acidobacteriota Terriglobia Terriglobales Terracidiphilus sp isolate SMAG U6028 HVZ82168.1 369 aa
BacClassIA Pseudomonadati Acidobacteriota Terriglobia Terriglobales Silvibacterium dinghuense WP 373283910.1 344 aa
BacClassIA Pseudomonadati Acidobacteriota Terriglobales Silvibacterium Silvibacterium sp isolate SMAG U12660 HTA87062.1 374 aa
BacClassIA Pseudomonadati Acidobacteriota Terriglobia Terriglobales Silvibacterium Silvibacterium sp isolate SMAG U5478 HEX3470223.1 356 aa
BacClassIA Pseudomonadati Acidobacteriota Terriglobales Silvibacterium Silvibacterium sp isolate MFD00056 ilm asm bin48 MFY9937054.1 368 aa
BacClassIA Pseudomonadati Acidobacteriota Terriglobia Terriglobales Terriglobales bacterium isolate NR1800517 MHB8734986.1 346 aa
BacClassIA Pseudomonadati Acidobacteriota Terriglobia Terriglobales Terriglobales bacterium isolate SMAG U9947 HZT74293.1 330 aa
BacClassIA Pseudomonadati Acidobacteriota Terriglobia Terriglobales Terriglobales bacterium isolate SE1507 concoct.bin.28 sub MGH9535505.1 348 aa
BacClassIA Pseudomonadati Acidobacteriota Terriglobia Terriglobales Terriglobales bacterium isolate TCS metawrap bin.122 MGH9506040.1 346 aa
BacClassIA Pseudomonadati Acidobacteriota Terriglobia Terriglobales Candidatus Korobacter versatilis strain Ellin345 WP 011525337.1 359 aa
BacClassIA Pseudomonadati Acidobacteriota unclassified Acidobacteriota Acidobacteriota bacterium isolate MAG BO43 MDR3747528.1 359 aa
BacClassIA Pseudomonadati Acidobacteriota Terriglobia Terriglobales Terriglobales bacterium isolate SMAG U7640 HEY4961516.1 358 aa
BacClassIA Pseudomonadati Acidobacteriota Terriglobia Terriglobales Terriglobales bacterium isolate SMAG U2566 HEX8800799.1 350 aa
BacClassIA Pseudomonadati Acidobacteriota Terriglobia Terriglobales Candidatus Korobacteraceae bacterium isolate MFD09568 ilm asm bin24 MGA9564187.1 359 aa
BacClassIA Pseudomonadati Acidobacteriota Terriglobia Terriglobales Terriglobales bacterium JemR1613 BGF27116.1 356 aa
BacClassIA Pseudomonadati Acidobacteriota unclassified Acidobacteriota Acidobacteriota bacterium isolate MAG BO89 MDR3764060.1 359 aa
BacClassIA Pseudomonadati Acidobacteriota unclassified Acidobacteriota Acidobacteriota bacterium isolate Tolsyn PSH04838.1 356 aa
BacClassIA Pseudomonadati Acidobacteriota Terriglobia unclassified Terriglobia Terriglobia bacterium isolate S13 MAG056 SFU KD MDT8068281.1 336 aa
BacClassIA Pseudomonadati Acidobacteriota Terriglobia Terriglobales Candidatus Korobacteraceae bacterium isolate SPRUCE 490 MGA2960996.1 364 aa
BacClassIA Pseudomonadati Acidobacteriota Terriglobia Terriglobales Candidatus Korobacteraceae bacterium isolate SPRUCE 138 MGD0974054.1 322 aa
BacClassIA Pseudomonadati Nitrospirota unclassified Nitrospirota Nitrospirota bacterium isolate NC groundwater 1776 Pr3 B0.1um 70 102 MBI5136583.1 319 aa
BacClassIA Pseudomonadati Nitrospirota unclassified Nitrospirota Nitrospirota bacterium isolate 4 1 July SF Bin45 MDH4228266.1 309 aa
BacClassIA Pseudomonadati Nitrospirota unclassified Nitrospirota Nitrospirota bacterium isolate 8 1 July SF Bin21 MDH5526739.1 313 aa
BacClassIA Pseudomonadati Nitrospirota unclassified Nitrospirota Nitrospirota bacterium isolate NC groundwater 1774 Pr3 B0.1um 61 24 MBI5189703.1 313 aa
BacClassIA Pseudomonadati Nitrospirota unclassified Nitrospirota Nitrospirota bacterium isolate SMAG U4741 HEY3347158.1 313 aa
BacClassIA Pseudomonadati Nitrospirota unclassified Nitrospirota Nitrospirota bacterium isolate SMAG U8263 HLB25523.1 313 aa
BacClassIA Pseudomonadati Nitrospirota unclassified Nitrospirota Nitrospirota bacterium isolate MFD06091 ilm asm bin59 MGC2424087.1 313 aa
BacClassIA Pseudomonadati Nitrospirota unclassified Nitrospirota Nitrospirota bacterium isolate NC groundwater 1767 Pr3 B0.1um 50 141 MBI5631991.1 287 aa
BacClassIA Pseudomonadati Nitrospirota unclassified Nitrospirota Nitrospirota bacterium isolate H52bin021 1 MEK6656570.1 314 aa
BacClassIA Pseudomonadati Nitrospirota unclassified Nitrospirota Nitrospirota bacterium isolate NC groundwater 1771 Pr3 B0.1um 55 10 MBI5197543.1 314 aa
BacClassIA Pseudomonadati Nitrospirota unclassified Nitrospirota Nitrospirota bacterium isolate SJ740 MCC7201420.1 314 aa
BacClassIA Pseudomonadati Nitrospirota unclassified Nitrospirota Nitrospirota bacterium isolate NC groundwater 1764 Pr3 B0.1um 46 12 MBI5755498.1 314 aa
BacClassIA Pseudomonadati Nitrospirota unclassified Nitrospirota Nitrospirota bacterium isolate NC groundwater 1884 Pr3 B0.1um 50 8 MBI5096898.1 308 aa
BacClassIA Pseudomonadati Nitrospirota unclassified Nitrospirota Nitrospirae bacterium GWA2 42 11 OGW15165.1 313 aa
BacClassIA Pseudomonadati Nitrospirota unclassified Nitrospirota Nitrospirota bacterium isolate NC groundwater 1760 Pr3 B0.1um 42 52 MBI5191988.1 314 aa
BacClassIA Pseudomonadati Nitrospirota Nitrospiria Nitrospirales Nitrospira sp isolate STD1 85 MDD5434568.1 314 aa 314 aa
BacClassIA Pseudomonadati Nitrospirota unclassified Nitrospirota Nitrospirota bacterium isolate NC groundwater 954 Pr1 S0.2um 48 36 MBI3606184.1 313 aa
BacClassIA Pseudomonadati Nitrospirota unclassified Nitrospirota Nitrospirota bacterium isolate NC groundwater 952 Pr1 S0.2um 46 34 MBI3594442.1 311 aa
BacClassIA Pseudomonadati Nitrospirota unclassified Nitrospirota Nitrospirota bacterium isolate NC groundwater 32 Pr7 B0.1um 46 30 MBI1820103.1 313 aa
BacClassIA Pseudomonadati Nitrospirota unclassified Nitrospirota Nitrospirota bacterium isolate S.5.sp1 bin.40 MFQ5455401.1 314 aa
BacClassIA Pseudomonadati Nitrospirota Nitrospiria Nitrospiria Nitrospiria bacterium isolate SMAG U2890 HEX9757647.1 313 aa 313 aa
BacClassIA Pseudomonadati Nitrospirota unclassified Nitrospirota Nitrospirota bacterium isolate MAG135 MEW6324127.1 313 aa
BacClassIA Pseudomonadati Nitrospirota Nitrospiria unclassified Nitrospiria Nitrospiria bacterium isolate SMAG U12235 HXZ25428.1 313 aa
BacClassIA Pseudomonadati Nitrospirota Nitrospiria unclassified Nitrospiria Nitrospiria bacterium isolate H1 B1 maxbin2.bin.213 sub MGH7275068.1 313 aa 313 aa
BacClassIA Pseudomonadati Nitrospirota Nitrospiria Nitrospirales Nitrospira sp isolate PMV 3 maxbin.023 sub MCI0527316.1 313 aa 313 aa
BacClassIA Pseudomonadati Nitrospirota unclassified Nitrospirota Nitrospirae bacterium RIFCSPHIGHO2 01 FULL 66 17 OGW60704.1 312 aa
BacClassIA Pseudomonadati Nitrospirota unclassified Nitrospirota Nitrospirota bacterium isolate H14bin054 1 MEK7307078.1 314 aa
BacClassIA Pseudomonadati Nitrospirota Nitrospiria Candidatus Troglogloeales Candidatus Troglogloeales bacterium MBI3359133.1 312 aa 312 aa
BacClassIA Pseudomonadati Nitrospirota unclassified Nitrospirota Nitrospirota bacterium isolate H32bin080 1 MEK7286756.1 312 aa
BacClassIA Pseudomonadati Nitrospirota unclassified Nitrospirota Nitrospirota bacterium isolate H32bin103 1 MEK7747519.1 312 aa
BacClassIA Pseudomonadati Nitrospirota Nitrospiria Nitrospiria Nitrospiria bacterium isolate SMAG U13227 HET6370500.1 313 aa 313 aa
BacClassIA Pseudomonadati Nitrospirota Nitrospiria Candidatus Troglogloeales Candidatus Manganitrophaceae bacterium isolate SMAG U7895 HLG21915.1 312 aa 312 aa
BacClassIA Pseudomonadati Nitrospirota Nitrospiria Candidatus Troglogloeales Candidatus Manganitrophus noduliformans strain Mn 1 WP 168060311.1 312 aa 312 aa
BacClassIA Pseudomonadati Nitrospirota Nitrospiria unclassified Nitrospiria Nitrospiria bacterium isolate Maj 1 bin.27 MFQ5780592.1 312 aa
BacClassIA Pseudomonadati Nitrospirota Nitrospiria Nitrospirales Nitrospira sp isolate FK190211 WC 24 26 27 29 metabat2 scaf2bin.069 MEO2054568.1 312 aa 312 aa
BacClassIA Pseudomonadati Nitrospirota unclassified Nitrospirota Nitrospirota bacterium isolate DY22 TVG19 BIN34 MEC4678230.1 312 aa
BacClassIA Pseudomonadati Nitrospirota Nitrospiria Nitrospiria Nitrospiria bacterium isolate D 2 bin.8 MFQ5587968.1 312 aa 312 aa
BacClassIA Pseudomonadati Nitrospirota Nitrospiria unclassified Nitrospiria Nitrospiria bacterium isolate D 3 bin.29 MFQ5542892.1 312 aa
BacClassIA Pseudomonadati Nitrospirota Nitrospiria Nitrospiria Nitrospiria bacterium isolate D 2 bin.29 MFQ5596424.1 312 aa 312 aa
BacClassIA Pseudomonadati Nitrospirota Nitrospiria unclassified Nitrospiria Nitrospiria bacterium isolate SMAG U56 HEY5647889.1 290 aa
BacClassIA Pseudomonadati Nitrospirota Nitrospiria Nitrospiria Nitrospiria bacterium isolate SMAG U8221 HLB94711.1 313 aa 313 aa
BacClassIA Pseudomonadati Nitrospirota unclassified Nitrospirota Nitrospirota bacterium isolate H32bin115 1 MEK6684380.1 313 aa
BacClassIA Pseudomonadati Nitrospirota Nitrospiria Nitrospiria Nitrospiria bacterium isolate SMAG U7931 HEY4485611.1 313 aa 313 aa
BacClassIA Pseudomonadati Nitrospirota unclassified Nitrospirota Nitrospirota bacterium isolate DY50 MC03 BIN197 MEC4683178.1 316 aa
BacClassIA Pseudomonadati Nitrospirota Nitrospiria Nitrospirales Nitrospiraceae bacterium isolate WUY.bin15 MDA8112045.1 312 aa
BacClassIA Pseudomonadati Nitrospirota Nitrospiria Nitrospirales Leptospirillum ferrooxidans WP 014449048.1 313 aa 313 aa
BacClassIA Pseudomonadati Nitrospirota Nitrospiria Nitrospirales Leptospirillum multispecies WP 014961330.1 314 aa 314 aa
BacClassIA Pseudomonadati Nitrospirota Nitrospiria Nitrospirales Nitrospirales bacterium isolate UWMA0026 HIA15011.1 313 aa
BacClassIA Pseudomonadati Nitrospirota unclassified Nitrospirota Nitrospirota bacterium isolate NS 11 TLY19204.1 312 aa
BacClassIA Pseudomonadati Nitrospirota Nitrospiria Nitrospirales Nitrospirales bacterium isolate SMAG U4674 HEY3198351.1 312 aa 312 aa
BacClassIA Pseudomonadati Nitrospirota unclassified Nitrospirota Nitrospirota bacterium isolate NC groundwater 1036 Pr1 S0.65um 62 21 MBI3810138.1 312 aa
BacClassIA Pseudomonadati Nitrospirota unclassified Nitrospirota Nitrospirota bacterium isolate NS 1 TLY32713.1 307 aa
BacClassIA Pseudomonadati Nitrospirota unclassified Nitrospirota Nitrospirota bacterium isolate NS 4 TLY38186.1 312 aa
BacClassIA Pseudomonadati Nitrospirota Nitrospiria Nitrospirales Nitrospiraceae bacterium isolate FS 1 bin.17 MFQ5992571.1 313 aa
BacClassIA Pseudomonadati Nitrospirota Nitrospiria Nitrospirales Nitrospira sp isolate RSF151 TKB82722.1 313 aa 313 aa
BacClassIA Pseudomonadati Nitrospirota Nitrospiria Nitrospirales Nitrospira sp isolate K Offshore 80m m2 121 MBM4123262.1 313 aa 313 aa
BacClassIA Pseudomonadati Nitrospirota Nitrospiria Nitrospirales Nitrospira sp Kam Ns4a WP 447973457.1 313 aa 313 aa
BacClassIA Pseudomonadati Nitrospirota Nitrospiria Nitrospirales Nitrospiraceae bacterium isolate Co bin7 MBD0305163.1 313 aa 313 aa
BacClassIA Pseudomonadati Nitrospirota unclassified Nitrospirota Nitrospirota bacterium isolate B7D1T1 MCH6557952.1 313 aa
BacClassIA Pseudomonadati Nitrospirota Nitrospiria Nitrospirales Nitrospirales bacterium isolate BS3bin.15 MEE8494533.1 313 aa
BacClassIA Pseudomonadati Nitrospirota Nitrospiria Nitrospirales Nitrospirales bacterium isolate M3 5 concoct.bin.312 MGH7255601.1 313 aa 313 aa
BacClassIA Pseudomonadati Nitrospirota unclassified Nitrospirota Nitrospirota bacterium isolate H32bin034 1 MEK7293667.1 316 aa
BacClassIA Pseudomonadati Nitrospirota Nitrospiria Nitrospirales Nitrospiraceae bacterium isolate BaikaldeepG159 MSQ76966.1 316 aa
BacClassIA Pseudomonadati Nitrospirota Nitrospiria Nitrospirales Nitrospira sp isolate samp 4439 VAMB 406 MFM7840501.1 316 aa 316 aa
BacClassIA Pseudomonadati Nitrospirota Nitrospiria Nitrospirales Nitrospirales bacterium isolate HN17.bin.18 MDX1412226.1 313 aa 313 aa
BacClassIA Pseudomonadati Nitrospirota unclassified Nitrospirota Nitrospirota bacterium isolate J017 RMH31104.1 315 aa
BacClassIA Pseudomonadati Nitrospirota Nitrospiria Nitrospirales Nitrospira sp M1 WP 447970482.1 313 aa 313 aa
BacClassIA Pseudomonadati Nitrospirota unclassified Nitrospirota Nitrospirota bacterium isolate DY73 BC09 BIN30 MEC4680159.1 313 aa
BacClassIA Pseudomonadati Nitrospirota Nitrospiria Nitrospirales uncultured Nitrospira sp GCF 902500695.1 WP 342346569.1 313 aa 313 aa
BacClassIA Pseudomonadati Nitrospirota Nitrospiria Nitrospirales Nitrospirales bacterium isolate MT6bin.22 MEE8126794.1 313 aa
BacClassIA Pseudomonadati Nitrospirota Nitrospiria Nitrospirales Nitrospira sp isolate HKST UBA02 MCA9462208.1 312 aa 312 aa
BacClassIA Pseudomonadati Nitrospirota Nitrospiria Nitrospirales Nitrospirales bacterium isolate MH Pat all autometa 1 10 NKB81185.1 313 aa 313 aa
BacClassIA Pseudomonadati Nitrospirota unclassified Nitrospirota Nitrospirota bacterium isolate KW1S15 218 MCZ6800392.1 314 aa
BacClassIA Pseudomonadati Nitrospirota unclassified Nitrospirota Nitrospirota bacterium isolate DY29 MC03 BIN16 MEC4670296.1 313 aa
BacClassIA Pseudomonadati Nitrospirota unclassified Nitrospirota Nitrospirota bacterium isolate J031 RMH03892.1 313 aa
BacClassIA Pseudomonadati Nitrospirota Nitrospiria Nitrospirales Nitrospira sp isolate STY3 bin 3 MDO9860451.1 325 aa 325 aa
BacClassIA Pseudomonadati Nitrospirota Nitrospiria Nitrospirales Nitrospira sp isolate RHO3 bin 20 MDE0146208.1 313 aa 313 aa
BacClassIA Pseudomonadati Nitrospirota unclassified Nitrospirota Nitrospirota bacterium isolate XB MAG71 MEC9005762.1 313 aa
BacClassIA Pseudomonadati Nitrospirota unclassified Nitrospirota Nitrospirota bacterium isolate DY22 TVG06 BIN6 MEC4675161.1 313 aa
BacClassIA Pseudomonadati Nitrospirota Nitrospiria Nitrospirales Candidatus Nitronereus thalassa WP 313832725.1 313 aa 313 aa
BacClassIA Pseudomonadati Acidobacteriota unclassified Acidobacteriota Acidobacteriota bacterium isolate P01 B01 bin.29 MEM7247243.1 312 aa
BacClassIA Pseudomonadati Nitrospinota Tectimicrobiota group Nitrospinota Nitrospinota bacterium isolate J063 RMF92004.1 314 aa 314 aa
BacClassIA Pseudomonadati Nitrospinota Tectimicrobiota group Candidatus Tectimicrobiota Candidatus Tectomicrobia bacterium isolate SAD bin17 MDQ3829900.1 313 aa 313 aa
BacClassIA Pseudomonadati Nitrospinota Tectimicrobiota group Candidatus Tectimicrobiota Candidatus Tectomicrobia bacterium isolate APA bin 51 MCY4514367.1 314 aa 314 aa
BacClassIA Pseudomonadati Nitrospinota Candidatus Tectimicrobiota bacterium isolate MT4bin.9 MEE8302879.1 ClassIV 313 aa
BacClassIA Pseudomonadati Nitrospinota Tectimicrobiota group Candidatus Tectimicrobiota Candidatus Tectimicrobiota bacterium isolate MT4bin.13 MEE8292454.1 313 aa 313 aa
BacClassIA Pseudomonadati Nitrospinota Tectimicrobiota group Candidatus Tectimicrobiota Candidatus Entotheonella sp isolate SMAG U3999 HEY7711219.1 313 aa 313 aa
BacClassIA Pseudomonadati Nitrospinota Candidatus Entotheonella palauensis WP 089943156.1 ClassIV 313 aa
BacClassIA Pseudomonadati Nitrospinota Candidatus Entotheonella gemina ETX06998.1 ClassIV 302 aa
BacClassIA Pseudomonadati Nitrospinota Tectimicrobiota group Candidatus Tectimicrobiota Candidatus Tectimicrobiota bacterium isolate NC groundwater 717 Ag S 0.2um 59 8 MBI3015184.1 312 aa 312 aa
BacClassIA Pseudomonadati Nitrospinota Tectimicrobiota group Nitrospinota Nitrospinae bacterium AH 259 B05 G02 I21 MDA2915324.1 313 aa 313 aa
BacClassIA Pseudomonadati Nitrospinota Tectimicrobiota group Candidatus Tectimicrobiota Candidatus Tectimicrobiota bacterium isolate K 1 bin.3 MFQ6671826.1 313 aa 313 aa
BacClassIA Pseudomonadati Nitrospinota Tectimicrobiota group Nitrospinota Nitrospinota bacterium isolate Fu MAG 98 HJM41762.1 307 aa 307 aa
BacClassIA Pseudomonadati Nitrospinota Tectimicrobiota group Nitrospinota Nitrospinota bacterium isolate Maj 1 bin.58 MFQ5691612.1 313 aa 313 aa
BacClassIA Pseudomonadati Nitrospinota Tectimicrobiota group Nitrospinota Nitrospinota bacterium isolate K 2 bin.39 MFQ5911971.1 313 aa 313 aa
BacClassIA Pseudomonadati Nitrospinota Tectimicrobiota group Nitrospinota Nitrospinota bacterium isolate K 1 bin.23 MFQ5895174.1 542 aa 541 aa
BacClassIA Pseudomonadati Nitrospinota Tectimicrobiota group Nitrospinota Nitrospinota bacterium isolate b2.sp2 bin.13 MFQ6110862.1 312 aa 312 aa
BacClassIA Pseudomonadati Nitrospinota Tectimicrobiota group Nitrospinota Nitrospinota bacterium isolate H14 bin067 1 MEK6710403.1 309 aa 309 aa
BacClassIA Pseudomonadati Nitrospinota Tectimicrobiota group Candidatus Tectimicrobiota Candidatus Tectomicrobia bacterium RIFCSPLOWO2 02 FULL 70 19 OGL61142.1 309 aa 309 aa
BacClassIA Pseudomonadati Nitrospinota Tectimicrobiota group Nitrospinota Nitrospinota bacterium isolate XB MAG82 MED5578443.1 313 aa 313 aa
BacClassIA Pseudomonadati Nitrospinota Tectimicrobiota group Nitrospinota Nitrospinota bacterium isolate SB0662 bin 51 MYA95395.1 314 aa 314 aa
BacClassIA Pseudomonadati Nitrospinota Tectimicrobiota group Nitrospinota Nitrospinota bacterium isolate OC6 4000m MAG 328 MFP6867864.1 315 aa 315 aa
BacClassIA Pseudomonadati Nitrospinota Tectimicrobiota group Nitrospinota Nitrospinota bacterium isolate ETNP15 MAG 5 MDP6277511.1 315 aa 315 aa
BacClassIA Pseudomonadati Nitrospinota Tectimicrobiota group Nitrospinota Nitrospinia Nitrospinaceae bacterium isolate co234 bin71 MBT3352051.1 320 aa 320 aa
BacClassIA Pseudomonadati Nitrospinota Tectimicrobiota group Nitrospinota Nitrospinota bacterium isolate 8 1 July SF Bin34 MDH5542682.1 316 aa 316 aa
BacClassIA Pseudomonadati Nitrospinota Tectimicrobiota group Nitrospinota Nitrospinota bacterium isolate S.4.sp1 bin.41 MFQ5431781.1 314 aa 314 aa
BacClassIA Pseudomonadati Nitrospinota Tectimicrobiota group Nitrospinota Nitrospinota bacterium isolate NC groundwater 1755 Pr3 B 0.1um 59 18 MBI5636781.1 314 aa 314 aa
BacClassIA Pseudomonadati Nitrospinota Tectimicrobiota group Nitrospinota Nitrospinota bacterium isolate BS2bin.9 MEE8482995.1 314 aa 314 aa
BacClassIA Pseudomonadati Nitrospinota Tectimicrobiota group Nitrospinota Nitrospinota bacterium isolate NC groundwater 1756 Pr3 B 0.1um 59 45 MBI5178326.1 314 aa 314 aa
BacClassIA Pseudomonadati Nitrospinota Tectimicrobiota group Nitrospinota Nitrospinota bacterium isolate NC groundwater 950 Pr1 S 0.2um 59 16 MBI3580645.1 314 aa 314 aa
BacClassIA Pseudomonadati Nitrospinota Tectimicrobiota group Nitrospinota Nitrospinota bacterium isolate NC groundwater 1032 Pr1 S 0.65um 54 26 MBI3794850.1 314 aa 314 aa
BacClassIA Pseudomonadati Nitrospinota Tectimicrobiota group Nitrospinota Nitrospinota bacterium isolate nWMHbin6 MBF0170175.1 319 aa 319 aa
BacClassIA Pseudomonadati Nitrospinota Tectimicrobiota group Nitrospinota Nitrospinota bacterium isolate DBRS3.bin6 MFW2330795.1 317 aa 317 aa
BacClassIA Pseudomonadati Nitrospinota Tectimicrobiota group Nitrospinota Nitrospinota bacterium isolate NC groundwater 1503 Pr4 B 0.1um 56 23 MBI4666283.1 318 aa 318 aa
BacClassIA Pseudomonadati Nitrospinota Tectimicrobiota group Nitrospinota Nitrospinota bacterium isolate nNGHbin12 MBF0291239.1 318 aa 318 aa
BacClassIA Pseudomonadati Nitrospinota Tectimicrobiota group Nitrospinota Nitrospinota bacterium isolate NC groundwater 1881 Pr3 B 0.1um 57 61 MBI5814602.1 323 aa 323 aa
BacClassIA Pseudomonadati Nitrospinota Tectimicrobiota group Nitrospinota Nitrospinota bacterium isolate NC groundwater 1556 Pr4 S 0.65um 63 8 MBI4829791.1 319 aa 319 aa
BacClassIA Pseudomonadati Nitrospinota Tectimicrobiota group Nitrospinota Nitrospinota bacterium isolate Fu MAG 66 HJM83028.1 318 aa 318 aa
BacClassIA Pseudomonadati Nitrospinota Tectimicrobiota group Nitrospinota Nitrospinota bacterium isolate 8 1 Jan SF Bin9 MDH5476713.1 318 aa 318 aa
BacClassIA Pseudomonadati Nitrospinota Tectimicrobiota group Nitrospinota Nitrospinota bacterium isolate 4 1 July SF Bin3 MDH4183540.1 318 aa 318 aa
BacClassIA Pseudomonadati Nitrospinota Tectimicrobiota group Nitrospinota Nitrospinota bacterium isolate SMAG U14208 HUU50054.1 313 aa 313 aa

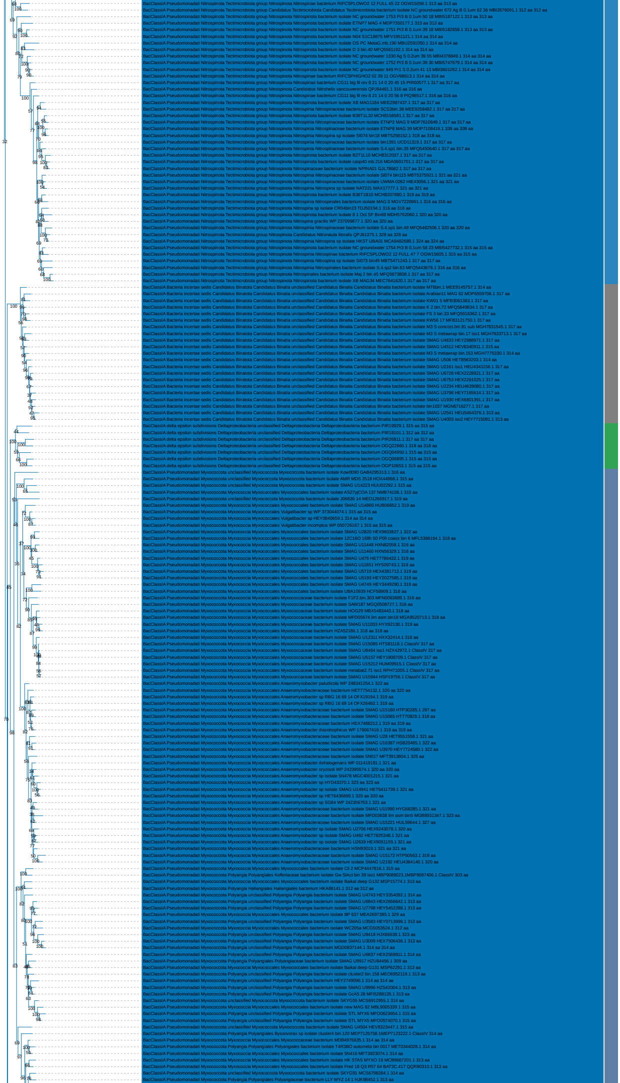
BacClassIA Pseudomonadati Nitrospinota Tectimicrobiota group Nitrospinota Nitrospinae bacterium RIFCSPLOWO2 12 FULL 45 22 OGW15056.1 313 aa 313 aa
BacClassIA Pseudomonadati Nitrospinota Tectimicrobiota group Candidatus Tectimicrobiota Candidatus Tectimicrobiota bacterium isolate NC groundwater 672 Ag B 0.1um 62 36 MBI2876091.1 312 aa 312 aa
BacClassIA Pseudomonadati Nitrospinota Tectimicrobiota group Nitrospinota Nitrospinota bacterium isolate NC groundwater 1753 Pr3 B 0.1um 50 18 MBI5187122.1 313 aa 313 aa
BacClassIA Pseudomonadati Nitrospinota Tectimicrobiota group Nitrospinota Nitrospinota bacterium isolate ETNP7 MAG 4 MDP7350177.1 313 aa 313 aa
BacClassIA Pseudomonadati Nitrospinota Tectimicrobiota group Nitrospinota Nitrospinota bacterium isolate NC groundwater 1751 Pr3 B 0.1um 39 18 MBI5182858.1 313 aa 313 aa
BacClassIA Pseudomonadati Nitrospinota Tectimicrobiota group Nitrospinota Nitrospinota bacterium isolate N04 S1C18675 MFV1951121.1 314 aa 314 aa
BacClassIA Pseudomonadati Nitrospinota Tectimicrobiota group Nitrospinota Nitrospinota bacterium isolate OS PC MetaG.mb.190 MBU2591050.1 314 aa 314 aa
BacClassIA Pseudomonadati Nitrospinota Tectimicrobiota group Nitrospinota Nitrospinota bacterium isolate D 3 bin.40 MFQ5561192.1 314 aa 314 aa
BacClassIA Pseudomonadati Nitrospinota Tectimicrobiota group Nitrospinota Nitrospinota bacterium isolate NC groundwater 1330 Ag S 0.2um 39 55 MBI4378849.1 314 aa 314 aa
BacClassIA Pseudomonadati Nitrospinota Tectimicrobiota group Nitrospinota Nitrospinota bacterium isolate NC groundwater 1752 Pr3 B 0.1um 39 30 MBI5747679.1 314 aa 314 aa
BacClassIA Pseudomonadati Nitrospinota Tectimicrobiota group Nitrospinota Nitrospinota bacterium isolate NC groundwater 949 Pr1 S 0.2um 41 13 MBI3601052.1 314 aa 314 aa
BacClassIA Pseudomonadati Nitrospinota Tectimicrobiota group Nitrospinota Nitrospinae bacterium RIFCSPHIGHO2 02 39 11 OGV98613.1 314 aa 314 aa
BacClassIA Pseudomonadati Nitrospinota Tectimicrobiota group Nitrospinota Nitrospinae bacterium CG11 big fil rev 8 21 14 0 20 45 15 PIR00577.1 317 aa 317 aa
BacClassIA Pseudomonadati Nitrospinota Tectimicrobiota group Nitrospinota Candidatus Nitrohelix vancouverensis QPJ64461.1 316 aa 316 aa
BacClassIA Pseudomonadati Nitrospinota Tectimicrobiota group Nitrospinota Nitrospinae bacterium CG11 big fil rev 8 21 14 0 20 56 8 PIQ86517.1 316 aa 316 aa
BacClassIA Pseudomonadati Nitrospinota Tectimicrobiota group Nitrospinota Nitrospinota bacterium isolate XB MAG1184 MEE2987437.1 317 aa 317 aa
BacClassIA Pseudomonadati Nitrospinota Tectimicrobiota group Nitrospinota Nitrospinia Nitrospinaceae bacterium isolate SCS3bin.38 MEE9258482.1 317 aa 317 aa
BacClassIA Pseudomonadati Nitrospinota Tectimicrobiota group Nitrospinota Nitrospinota bacterium isolate B39T1L10 MCH8156581.1 317 aa 317 aa
BacClassIA Pseudomonadati Nitrospinota Tectimicrobiota group Nitrospinota Nitrospinia Nitrospinaceae bacterium isolate ETNP2 MAG 9 MDP7610849.1 317 aa 317 aa
BacClassIA Pseudomonadati Nitrospinota Tectimicrobiota group Nitrospinota Nitrospinia Nitrospinaceae bacterium isolate ETNP8 MAG 39 MDP7108416.1 339 aa 339 aa
BacClassIA Pseudomonadati Nitrospinota Tectimicrobiota group Nitrospinota Nitrospinia Nitrospina sp isolate SI074 bin18 MBT5258152.1 318 aa 318 aa
BacClassIA Pseudomonadati Nitrospinota Tectimicrobiota group Nitrospinota Nitrospinia Nitrospinaceae bacterium isolate bin1391 UCD11319.1 317 aa 317 aa
BacClassIA Pseudomonadati Nitrospinota Tectimicrobiota group Nitrospinota Nitrospinia Nitrospinaceae bacterium isolate S.4.sp1 bin.35 MFQ5450640.1 317 aa 317 aa
BacClassIA Pseudomonadati Nitrospinota Tectimicrobiota group Nitrospinota Nitrospinota bacterium isolate B2T1L10 MCH8312037.1 317 aa 317 aa
BacClassIA Pseudomonadati Nitrospinota Tectimicrobiota group Nitrospinota Nitrospinia Nitrospinaceae bacterium isolate casp40 mb.216 MDA0691701.1 317 aa 317 aa
BacClassIA Pseudomonadati Nitrospinota Tectimicrobiota group Nitrospinota Nitrospinaceae bacterium isolate NPINA01 GJL78682.1 317 aa 317 aa
BacClassIA Pseudomonadati Nitrospinota Tectimicrobiota group Nitrospinota Nitrospinia Nitrospinaceae bacterium isolate SI074 bin115 MBT5375921.1 321 aa 321 aa
BacClassIA Pseudomonadati Nitrospinota Tectimicrobiota group Nitrospinota Nitrospinia Nitrospinaceae bacterium isolate UWMA 0252 HIE43056.1 321 aa 321 aa
BacClassIA Pseudomonadati Nitrospinota Tectimicrobiota group Nitrospinota Nitrospinia Nitrospina sp isolate NAT221 MAX17777.1 321 aa 321 aa
BacClassIA Pseudomonadati Nitrospinota Tectimicrobiota group Nitrospinota Nitrospinota bacterium isolate B36T1B10 MCH8207880.1 319 aa 319 aa
BacClassIA Pseudomonadati Nitrospinota Tectimicrobiota group Nitrospinota Nitrospinia Nitrospinales bacterium isolate MAG 0 MGV7220961.1 316 aa 316 aa
BacClassIA Pseudomonadati Nitrospinota Tectimicrobiota group Nitrospinota Nitrospinia Nitrospina sp isolate CR04bin23 TDJ50194.1 316 aa 316 aa
BacClassIA Pseudomonadati Nitrospinota Tectimicrobiota group Nitrospinota Nitrospinota bacterium isolate 8 1 Oct SF Bin69 MDH5762060.1 320 aa 320 aa
BacClassIA Pseudomonadati Nitrospinota Tectimicrobiota group Nitrospinota Nitrospinia Nitrospina gracilis WP 237099877.1 320 aa 320 aa
BacClassIA Pseudomonadati Nitrospinota Tectimicrobiota group Nitrospinota Nitrospinia Nitrospinaceae bacterium isolate S.4.sp1 bin.49 MFQ5482506.1 320 aa 320 aa
BacClassIA Pseudomonadati Nitrospinota Tectimicrobiota group Nitrospinota Candidatus Nitronauta litoralis QPJ61375.1 328 aa 328 aa
BacClassIA Pseudomonadati Nitrospinota Tectimicrobiota group Nitrospinota Nitrospinia Nitrospina sp isolate HKST UBA01 MCA9482689.1 324 aa 324 aa
BacClassIA Pseudomonadati Nitrospinota Tectimicrobiota group Nitrospinota Nitrospinota bacterium isolate NC groundwater 1754 Pr3 B 0.1um 58 23 MBI5427732.1 315 aa 315 aa
BacClassIA Pseudomonadati Nitrospinota Tectimicrobiota group Nitrospinota Nitrospinia Nitrospinae bacterium RIFCSPLOWO2 12 FULL 47 7 OGW15605.1 315 aa 315 aa
BacClassIA Pseudomonadati Nitrospinota Tectimicrobiota group Nitrospinota Nitrospinia Nitrospina sp isolate SI073 bin49 MBT5471243.1 317 aa 317 aa
BacClassIA Pseudomonadati Nitrospinota Tectimicrobiota group Nitrospinota Nitrospinia Nitrospinales bacterium isolate S.4.sp2 bin.63 MFQ5443676.1 316 aa 316 aa
BacClassIA Pseudomonadati Nitrospinota Tectimicrobiota group Nitrospinota Nitrospinales bacterium isolate Maj 2 bin.45 MFQ5673808.1 317 aa 317 aa
BacClassIA Pseudomonadati Nitrospinota Tectimicrobiota group Nitrospinota Nitrospinota bacterium isolate XB MAG34 MEC7641620.1 317 aa 317 aa
BacClassIA Bacteria incertae sedis Candidatus Binatota Candidatus Binatia unclassified Candidatus Binatia Candidatus Binatia bacterium isolate MT6bin.1 MEE9145757.1 314 aa
BacClassIA Bacteria incertae sedis Candidatus Binatota Candidatus Binatia unclassified Candidatus Binatia Candidatus Binatia bacterium isolate Arabian11 MAG 62 MDP6559706.1 317 aa
BacClassIA Bacteria incertae sedis Candidatus Binatota Candidatus Binatia unclassified Candidatus Binatia Candidatus Binatia bacterium isolate KW01 5 MFB3061383.1 317 aa
BacClassIA Bacteria incertae sedis Candidatus Binatota Candidatus Binatia unclassified Candidatus Binatia Candidatus Binatia bacterium isolate K 2 bin.72 MFQ5849634.1 317 aa
BacClassIA Bacteria incertae sedis Candidatus Binatota Candidatus Binatia unclassified Candidatus Binatia Candidatus Binatia bacterium isolate FS 3 bin.33 MFQ5916362.1 317 aa
BacClassIA Bacteria incertae sedis Candidatus Binatota Candidatus Binatia unclassified Candidatus Binatia Candidatus Binatia bacterium isolate KW56 17 MFB3121750.1 317 aa
BacClassIA Bacteria incertae sedis Candidatus Binatota Candidatus Binatia unclassified Candidatus Binatia Candidatus Binatia bacterium isolate M3 S concoct.bin.81 sub MGH7831545.1 317 aa
BacClassIA Bacteria incertae sedis Candidatus Binatota Candidatus Binatia unclassified Candidatus Binatia Candidatus Binatia bacterium isolate M3 S metawrap bin.17 iso1 MGH7833713.1 317 aa
BacClassIA Bacteria incertae sedis Candidatus Binatota Candidatus Binatia unclassified Candidatus Binatia Candidatus Binatia bacterium isolate SMAG U4633 HEY2988971.1 317 aa
BacClassIA Bacteria incertae sedis Candidatus Binatota Candidatus Binatia unclassified Candidatus Binatia Candidatus Binatia bacterium isolate SMAG U4512 HEV8340911.1 315 aa
BacClassIA Bacteria incertae sedis Candidatus Binatota Candidatus Binatia unclassified Candidatus Binatia Candidatus Binatia bacterium isolate M3 S metawrap bin.153 MGH7775330.1 314 aa
BacClassIA Bacteria incertae sedis Candidatus Binatota Candidatus Binatia unclassified Candidatus Binatia Candidatus Binatia bacterium isolate SMAG U506 HET8563203.1 314 aa
BacClassIA Bacteria incertae sedis Candidatus Binatota Candidatus Binatia unclassified Candidatus Binatia Candidatus Binatia bacterium isolate SMAG U2161 iso1 HEU4341156.1 317 aa
BacClassIA Bacteria incertae sedis Candidatus Binatota Candidatus Binatia unclassified Candidatus Binatia Candidatus Binatia bacterium isolate SMAG U6728 HEX2228921.1 317 aa
BacClassIA Bacteria incertae sedis Candidatus Binatota Candidatus Binatia unclassified Candidatus Binatia Candidatus Binatia bacterium isolate SMAG U6753 HEX2261025.1 317 aa
BacClassIA Bacteria incertae sedis Candidatus Binatota Candidatus Binatia unclassified Candidatus Binatia Candidatus Binatia bacterium isolate SMAG U2234 HEU4639080.1 317 aa
BacClassIA Bacteria incertae sedis Candidatus Binatota Candidatus Binatia unclassified Candidatus Binatia Candidatus Binatia bacterium isolate SMAG U3798 HEY7165614.1 317 aa
BacClassIA Bacteria incertae sedis Candidatus Binatota Candidatus Binatia unclassified Candidatus Binatia Candidatus Binatia bacterium isolate SMAG U1930 HEX6801391.1 317 aa
BacClassIA Bacteria incertae sedis Candidatus Binatota Candidatus Binatia unclassified Candidatus Binatia Candidatus Binatia bacterium isolate bin1037 MGN6716277.1 317 aa
BacClassIA Bacteria incertae sedis Candidatus Binatota Candidatus Binatia unclassified Candidatus Binatia Candidatus Binatia bacterium isolate SMAG U2541 HEU5464376.1 313 aa
BacClassIA Bacteria incertae sedis Candidatus Binatota Candidatus Binatia unclassified Candidatus Binatia Candidatus Binatia bacterium isolate SMAG U4003 iso2 HEY7715091.1 313 aa
BacClassIA delta epsilon subdivisions Deltaproteobacteria unclassified Deltaproteobacteria Deltaproteobacteria bacterium PIR19929.1 315 aa 315 aa
BacClassIA delta epsilon subdivisions Deltaproteobacteria unclassified Deltaproteobacteria Deltaproteobacteria bacterium PIR18101.1 312 aa 312 aa
BacClassIA delta epsilon subdivisions Deltaproteobacteria unclassified Deltaproteobacteria Deltaproteobacteria bacterium PIR26811.1 317 aa 317 aa
BacClassIA delta epsilon subdivisions Deltaproteobacteria unclassified Deltaproteobacteria Deltaproteobacteria bacterium OGQ22660.1 318 aa 318 aa
BacClassIA delta epsilon subdivisions Deltaproteobacteria unclassified Deltaproteobacteria Deltaproteobacteria bacterium OGQ04950.1 315 aa 315 aa
BacClassIA delta epsilon subdivisions Deltaproteobacteria unclassified Deltaproteobacteria Deltaproteobacteria bacterium OGQ06895.1 315 aa 315 aa
BacClassIA delta epsilon subdivisions Deltaproteobacteria unclassified Deltaproteobacteria Deltaproteobacteria bacterium OGP10653.1 315 aa 315 aa
BacClassIA Pseudomonadati Myxococcota unclassified Myxococcota Myxococcota bacterium isolate Kow0090 GAB4295313.1 316 aa
BacClassIA Pseudomonadati Myxococcota unclassified Myxococcota Myxococcota bacterium isolate AMR MDS 2518 HOX44968.1 315 aa
BacClassIA Pseudomonadati Myxococcota unclassified Myxococcota Myxococcota bacterium isolate SMAG U14223 HUU02292.1 315 aa
BacClassIA Pseudomonadati Myxococcota Myxococcia Myxococcales Myxococcales bacterium isolate AS27yjCOA 137 NMB74156.1 315 aa
BacClassIA Pseudomonadati Myxococcota unclassified Myxococcota Myxococcota bacterium isolate J06636 14 MEO1266917.1 319 aa
BacClassIA Pseudomonadati Myxococcota Myxococcia Myxococcales Myxococcales bacterium isolate SMAG U14860 HUB06652.1 319 aa
BacClassIA Pseudomonadati Myxococcota Myxococcia Myxococcales Vulgatibacter sp WP 373044074.1 315 aa 315 aa
BacClassIA Pseudomonadati Myxococcota Myxococcia Myxococcales Vulgatibacter sp HEY0840659.1 314 aa 314 aa
BacClassIA Pseudomonadati Myxococcota Myxococcia Myxococcales Vulgatibacter incomptus WP 050726167.1 315 aa 315 aa
BacClassIA Pseudomonadati Myxococcota Myxococcia Myxococcales Myxococcales bacterium isolate SMAG U2820 HEX9603627.1 322 aa
BacClassIA Pseudomonadati Myxococcota Myxococcia Myxococcales Myxococcales bacterium isolate 12C16O 168h 50 P09 coass bin 6 MFL5366164.1 318 aa
BacClassIA Pseudomonadati Myxococcota Myxococcia Myxococcales Myxococcales bacterium isolate SMAG U11448 HXN82058.1 316 aa
BacClassIA Pseudomonadati Myxococcota Myxococcia Myxococcales Myxococcales bacterium isolate SMAG U11460 HXN56329.1 316 aa
BacClassIA Pseudomonadati Myxococcota Myxococcia Myxococcales Myxococcales bacterium isolate SMAG U476 HET7786432.1 319 aa
BacClassIA Pseudomonadati Myxococcota Myxococcia Myxococcales Myxococcales bacterium isolate SMAG U11651 HYS09743.1 319 aa
BacClassIA Pseudomonadati Myxococcota Myxococcia Myxococcales Myxococcales bacterium isolate SMAG U5719 HEX4381713.1 319 aa
BacClassIA Pseudomonadati Myxococcota Myxococcia Myxococcales Myxococcales bacterium isolate SMAG U5193 HEY2027585.1 319 aa
BacClassIA Pseudomonadati Myxococcota Myxococcia Myxococcales Myxococcales bacterium isolate SMAG U4749 HEY3449290.1 319 aa
BacClassIA Pseudomonadati Myxococcota Myxococcia Myxococcales Myxococcales bacterium isolate UBA10939 HCF58909.1 318 aa
BacClassIA Pseudomonadati Myxococcota Myxococcia Myxococcales Myxococcaceae bacterium isolate F1F2.bin.303 MFN0063889.1 316 aa
BacClassIA Pseudomonadati Myxococcota Myxococcia Myxococcales Myxococcaceae bacterium isolate SAW187 MGQ0508727.1 316 aa
BacClassIA Pseudomonadati Myxococcota Myxococcia Myxococcales Myxococcaceae bacterium isolate HOG29 MBX5483443.1 318 aa
BacClassIA Pseudomonadati Myxococcota Myxococcia Myxococcales Myxococcaceae bacterium isolate MFD05674 ilm asm bin19 MGA9520713.1 318 aa
BacClassIA Pseudomonadati Myxococcota Myxococcia Myxococcales Myxococcaceae bacterium isolate SMAG U11033 HYX92130.1 319 aa
BacClassIA Pseudomonadati Myxococcota Myxococcia Myxococcales Myxococcaceae bacterium HZA52186.1 318 aa 318 aa
BacClassIA Pseudomonadati Myxococcota Myxococcia Myxococcales Myxococcaceae bacterium isolate SMAG U12311 HXX32414.1 316 aa
BacClassIA Pseudomonadati Myxococcota Myxococcia Myxococcales Myxococcaceae bacterium isolate SMAG U15085 HTS81118.1 ClassIV 317 aa
BacClassIA Pseudomonadati Myxococcota Myxococcia Myxococcales Myxococcaceae bacterium isolate SMAG U8464 iso1 HZX42972.1 ClassIV 317 aa
BacClassIA Pseudomonadati Myxococcota Myxococcia Myxococcales Myxococcaceae bacterium isolate SMAG U5157 HEY1908709.1 ClassIV 317 aa
BacClassIA Pseudomonadati Myxococcota Myxococcia Myxococcales Myxococcaceae bacterium isolate SMAG U15212 HUM09915.1 ClassIV 317 aa
BacClassIA Pseudomonadati Myxococcota Myxococcia Myxococcales Myxococcaceae bacterium isolate metabat2.71 iso1 RPH71005.1 ClassIV 317 aa
BacClassIA Pseudomonadati Myxococcota Myxococcia Myxococcales Myxococcaceae bacterium isolate SMAG U15944 HSP19756.1 ClassIV 317 aa
BacClassIA Pseudomonadati Myxococcota Myxococcia Myxococcales Anaeromyxobacter paludicola WP 248341254.1 322 aa
BacClassIA Pseudomonadati Myxococcota Myxococcia Myxococcales Anaeromyxobacteraceae bacterium HET7754132.1 320 aa 320 aa
BacClassIA Pseudomonadati Myxococcota Myxococcia Myxococcales Anaeromyxobacter sp RBG 16 69 14 OFX19194.1 319 aa
BacClassIA Pseudomonadati Myxococcota Myxococcia Myxococcales Anaeromyxobacter sp RBG 16 69 14 OFX26492.1 319 aa
BacClassIA Pseudomonadati Myxococcota Myxococcia Myxococcales Anaeromyxobacteraceae bacterium isolate SMAG U15160 HTP30285.1 297 aa
BacClassIA Pseudomonadati Myxococcota Myxococcia Myxococcales Anaeromyxobacteraceae bacterium isolate SMAG U15065 HTT70829.1 318 aa
BacClassIA Pseudomonadati Myxococcota Myxococcia Myxococcales Anaeromyxobacteraceae bacterium HEX7488212.1 319 aa 319 aa
BacClassIA Pseudomonadati Myxococcota Myxococcia Myxococcales Anaeromyxobacter diazotrophicus WP 176067416.1 319 aa 319 aa
BacClassIA Pseudomonadati Myxococcota Myxococcia Myxococcales Anaeromyxobacteraceae bacterium isolate SMAG U28 HET9551558.1 321 aa
BacClassIA Pseudomonadati Myxococcota Myxococcia Myxococcales Anaeromyxobacteraceae bacterium isolate SMAG U16387 HSB20485.1 322 aa
BacClassIA Pseudomonadati Myxococcota Myxococcia Myxococcales Anaeromyxobacteraceae bacterium isolate SMAG U3970 HEY7724580.1 322 aa
BacClassIA Pseudomonadati Myxococcota Myxococcia Myxococcales Anaeromyxobacteraceae bacterium isolate 5N017 MFT3913604.1 325 aa
BacClassIA Pseudomonadati Myxococcota Myxococcia Myxococcales Anaeromyxobacter dehalogenans WP 011419181.1 321 aa
BacClassIA Pseudomonadati Myxococcota Myxococcia Myxococcales Anaeromyxobacter oryzisoli WP 242395574.1 320 aa 320 aa
BacClassIA Pseudomonadati Myxococcota Myxococcia Myxococcales Anaeromyxobacter sp isolate 5N478 MGC4001215.1 321 aa
BacClassIA Pseudomonadati Myxococcota Myxococcia Myxococcales Anaeromyxobacter sp HYD43370.1 323 aa 323 aa
BacClassIA Pseudomonadati Myxococcota Myxococcia Myxococcales Anaeromyxobacter sp isolate SMAG U14941 HET6411739.1 321 aa
BacClassIA Pseudomonadati Myxococcota Myxococcia Myxococcales Anaeromyxobacter sp HET6436869.1 320 aa 320 aa
BacClassIA Pseudomonadati Myxococcota Myxococcia Myxococcales Anaeromyxobacter sp SG64 WP 242356763.1 321 aa
BacClassIA Pseudomonadati Myxococcota Myxococcia Myxococcales Anaeromyxobacteraceae bacterium isolate SMAG U11990 HYG68285.1 321 aa
BacClassIA Pseudomonadati Myxococcota Myxococcia Myxococcales Anaeromyxobacteraceae bacterium isolate MFD03638 ilm asm bin5 MGB8931347.1 323 aa
BacClassIA Pseudomonadati Myxococcota Myxococcia Myxococcales Anaeromyxobacteraceae bacterium isolate SMAG U15221 HUL59644.1 327 aa
BacClassIA Pseudomonadati Myxococcota Myxococcia Myxococcales Anaeromyxobacter sp isolate SMAG U2706 HEX9243078.1 320 aa
BacClassIA Pseudomonadati Myxococcota Myxococcia Myxococcales Anaeromyxobacter sp isolate SMAG U462 HET7825348.1 321 aa
BacClassIA Pseudomonadati Myxococcota Myxococcia Myxococcales Anaeromyxobacter sp isolate SMAG U2639 HEX9051159.1 321 aa
BacClassIA Pseudomonadati Myxococcota Myxococcia Myxococcales Anaeromyxobacteraceae bacterium HSN93019.1 321 aa 321 aa
BacClassIA Pseudomonadati Myxococcota Myxococcia Myxococcales Anaeromyxobacteraceae bacterium isolate SMAG U15172 HTP50563.1 319 aa
BacClassIA Pseudomonadati Myxococcota Myxococcia Myxococcales Anaeromyxobacteraceae bacterium isolate SMAG U2192 HEU4384140.1 320 aa
BacClassIA Pseudomonadati Myxococcota Myxococcia Myxococcales Myxococcales bacterium isolate Cli 2 MCP4447816.1 315 aa
BacClassIA Pseudomonadati Myxococcota Polyangia Polyangiales Kofleriaceae bacterium isolate Gw SIAct bin 39 iso1 MBP9089023 1MBP9087406.1 ClassIV 303 aa
BacClassIA Pseudomonadati Myxococcota Myxococcia Myxococcales Myxococcales bacterium isolate Baikal deep G132 MSP15774.1 313 aa
BacClassIA Pseudomonadati Myxococcota Polyangia Haliangiales Haliangiales bacterium HKA88141.1 312 aa 312 aa
BacClassIA Pseudomonadati Myxococcota Polyangia unclassified Polyangia Polyangia bacterium isolate SMAG U4743 HEY3354092.1 314 aa
BacClassIA Pseudomonadati Myxococcota Polyangia unclassified Polyangia Polyangia bacterium isolate SMAG U6643 HEX2656642.1 313 aa
BacClassIA Pseudomonadati Myxococcota Polyangia unclassified Polyangia Polyangia bacterium isolate SMAG U7798 HEY5452288.1 313 aa
BacClassIA Pseudomonadati Myxococcota Myxococcia Myxococcales Myxococcales bacterium isolate BP 637 MEA2697385.1 329 aa
BacClassIA Pseudomonadati Myxococcota Polyangia unclassified Polyangia Polyangia bacterium isolate SMAG U3583 HEY0713696.1 313 aa
BacClassIA Pseudomonadati Myxococcota Myxococcia Myxococcales Myxococcales bacterium isolate WC205a MCG5053524.1 312 aa
BacClassIA Pseudomonadati Myxococcota Polyangia unclassified Polyangia Polyangia bacterium isolate SMAG U8418 HJX66639.1 323 aa
BacClassIA Pseudomonadati Myxococcota Polyangia unclassified Polyangia Polyangia bacterium isolate SMAG U3009 HEX7506436.1 313 aa
BacClassIA Pseudomonadati Myxococcota Polyangia unclassified Polyangia Polyangia bacterium MGD0837144.1 314 aa 314 aa
BacClassIA Pseudomonadati Myxococcota Polyangia unclassified Polyangia Polyangia bacterium isolate SMAG U6837 HEX2568911.1 314 aa
BacClassIA Pseudomonadati Myxococcota Polyangia Polyangiales Polyangiaceae bacterium isolate SMAG U9917 HZU84456.1 309 aa
BacClassIA Pseudomonadati Myxococcota Myxococcia Myxococcales Myxococcales bacterium isolate Baikal deep G131 MSP62291.1 313 aa
BacClassIA Pseudomonadati Myxococcota Polyangia unclassified Polyangia Polyangia bacterium isolate cluster2 bin.158 MEO6952119.1 313 aa
BacClassIA Pseudomonadati Myxococcota Polyangia unclassified Polyangia Polyangia bacterium HEY2749096.1 314 aa 314 aa
BacClassIA Pseudomonadati Myxococcota Polyangia unclassified Polyangia Polyangia bacterium isolate SMAG U9996 HZS42004.1 313 aa
BacClassIA Pseudomonadati Myxococcota Polyangia unclassified Polyangia Polyangia bacterium isolate GcA5 28 MFI5288135.1 313 aa
BacClassIA Pseudomonadati Myxococcota unclassified Myxococcota Myxococcota bacterium isolate SKYG55 MCS6912955.1 314 aa
BacClassIA Pseudomonadati Myxococcota Myxococcia Myxococcales Myxococcales bacterium isolate new MAG 82 MBL9005339.1 315 aa
BacClassIA Pseudomonadati Myxococcota Polyangia unclassified Polyangia Polyangia bacterium isolate STL MYX6 MFO0623654.1 315 aa
BacClassIA Pseudomonadati Myxococcota Polyangia unclassified Polyangia Polyangia bacterium isolate STL MYX5 MFO0574070.1 315 aa
BacClassIA Pseudomonadati Myxococcota unclassified Myxococcota Myxococcota bacterium isolate SMAG U4504 HEV8323447.1 315 aa
BacClassIA Pseudomonadati Myxococcota Polyangia Polyangiales Byssovorax sp isolate cluster4 bin.120 MEP7125758.1MEP7123222.1 ClassIV 314 aa
BacClassIA Pseudomonadati Myxococcota Myxococcia Myxococcales Myxococcaceae bacterium MDB4976635.1 314 aa 314 aa
BacClassIA Pseudomonadati Myxococcota Polyangia Polyangiales Polyangiales bacterium isolate T4R3BO autometa bin 0017 MET0344028.1 314 aa
BacClassIA Pseudomonadati Myxococcota Myxococcia Myxococcales Myxococcales bacterium isolate 5N416 MFT3923074.1 314 aa
BacClassIA Pseudomonadati Myxococcota Myxococcia Myxococcales Myxococcales bacterium isolate HK STAS MYXO 19 MCB9867201.1 313 aa
BacClassIA Pseudomonadati Myxococcota Myxococcia Myxococcales Myxococcales bacterium isolate Fred 18 Q3 R57 64 BAT3C.417 QQR90310.1 313 aa
BacClassIA Pseudomonadati Myxococcota unclassified Myxococcota Myxococcota bacterium isolate SKYG91 MCS6798284.1 314 aa
BacClassIA Pseudomonadati Myxococcota Polyangia Polyangiales Polyangiaceae bacterium LLY WYZ 14.1 HJK98452.1 313 aa

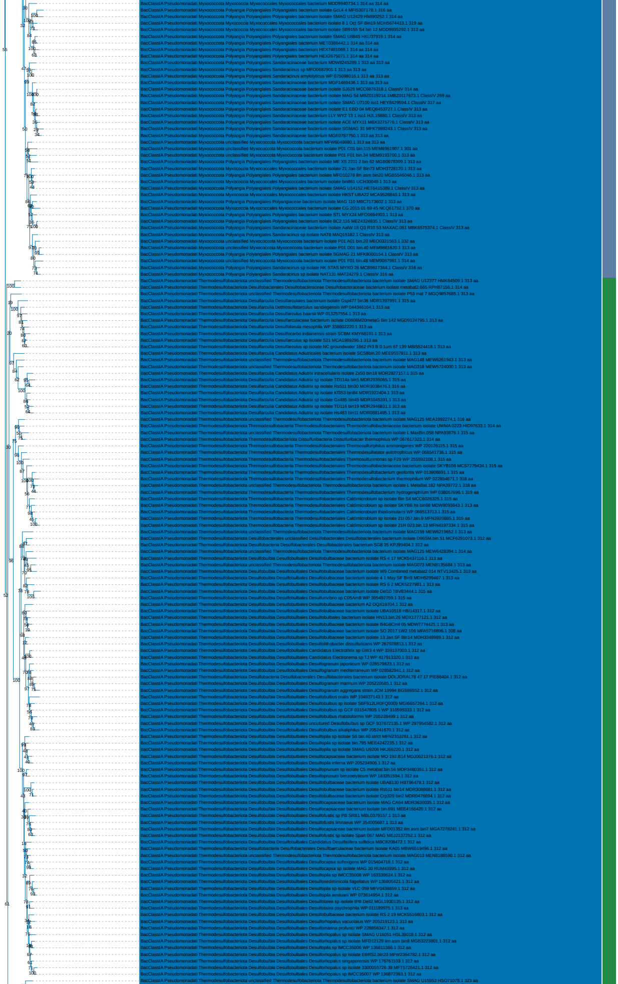
BacClassIA Pseudomonadati Myxococcota Myxococcia Myxococcales Myxococcales bacterium MDD9940734.1 314 aa 314 aa
BacClassIA Pseudomonadati Myxococcota Polyangia Polyangiales Polyangiales bacterium isolate GcL4 4 MFI5307178.1 316 aa
BacClassIA Pseudomonadati Myxococcota Polyangia Polyangiales Polyangiales bacterium isolate SMAG U12429 HMI90252.1 314 aa
BacClassIA Pseudomonadati Myxococcota Myxococcia Myxococcales Myxococcales bacterium isolate 8 1 Oct SF Bin19 MDH5674413.1 319 aa
BacClassIA Pseudomonadati Myxococcota Myxococcia Myxococcales Myxococcales bacterium isolate SB9155 S4 bin 12 MDD9935292.1 312 aa
BacClassIA Pseudomonadati Myxococcota Polyangia Polyangiales Polyangiales bacterium isolate SMAG U8845 HKU37919.1 314 aa
BacClassIA Pseudomonadati Myxococcota Polyangia Polyangiales Polyangiales bacterium MET0386442.1 314 aa 314 aa
BacClassIA Pseudomonadati Myxococcota Polyangia Polyangiales Polyangiales bacterium HEX7481069.1 314 aa 314 aa
BacClassIA Pseudomonadati Myxococcota Polyangia Polyangiales Polyangiales bacterium HEX2675071.1 314 aa 314 aa
BacClassIA Pseudomonadati Myxococcota Polyangia Polyangiales Sandaracinaceae bacterium MDW8245289.1 313 aa 313 aa
BacClassIA Pseudomonadati Myxococcota Polyangia Polyangiales Sandaracinus sp MFO0682901.1 313 aa 313 aa
BacClassIA Pseudomonadati Myxococcota Polyangia Polyangiales Sandaracinus amylolyticus WP 075098016.1 313 aa 313 aa
BacClassIA Pseudomonadati Myxococcota Polyangia Polyangiales Sandaracinaceae bacterium MGF1469436.1 313 aa 313 aa
BacClassIA Pseudomonadati Myxococcota Polyangia Polyangiales Sandaracinaceae bacterium isolate SJ628 MCC6876318.1 ClassIV 314 aa
BacClassIA Pseudomonadati Myxococcota Polyangia Polyangiales Sandaracinaceae bacterium isolate MAG 54 MBZ0119214.1MBZ0117673.1 ClassIV 269 aa
BacClassIA Pseudomonadati Myxococcota Polyangia Polyangiales Sandaracinaceae bacterium isolate SMAG U7100 iso1 HEY8429594.1 ClassIV 317 aa
BacClassIA Pseudomonadati Myxococcota Polyangia Polyangiales Sandaracinaceae bacterium isolate E1 EBD 04 MEQ8453727.1 ClassIV 313 aa
BacClassIA Pseudomonadati Myxococcota Polyangia Polyangiales Sandaracinaceae bacterium LLY WYZ 13 1 iso1 HJL15880.1 ClassIV 313 aa
BacClassIA Pseudomonadati Myxococcota Polyangia Polyangiales Sandaracinaceae bacterium isolate ACE MYX11 MBX3275776.1 ClassIV 313 aa
BacClassIA Pseudomonadati Myxococcota Polyangia Polyangiales Sandaracinaceae bacterium isolate SGMAG 31 MFK7989243.1 ClassIV 313 aa
BacClassIA Pseudomonadati Myxococcota Polyangia Polyangiales Sandaracinaceae bacterium MGE0787750.1 313 aa 313 aa
BacClassIA Pseudomonadati Myxococcota unclassified Myxococcota Myxococcota bacterium MFW6049880.1 313 aa 313 aa
BacClassIA Pseudomonadati Myxococcota unclassified Myxococcota Myxococcota bacterium isolate P01 C01 bin.115 MEM6961907.1 301 aa
BacClassIA Pseudomonadati Myxococcota unclassified Myxococcota Myxococcota bacterium isolate P01 F01 bin.34 MEM9193700.1 313 aa
BacClassIA Pseudomonadati Myxococcota Polyangia Polyangiales Polyangiales bacterium isolate ME XS 2211 2 bin 62 MGB0678309.1 313 aa
BacClassIA Pseudomonadati Myxococcota Myxococcia Myxococcales Myxococcales bacterium isolate 21 Jan SF Bin73 MDH3728120.1 313 aa
BacClassIA Pseudomonadati Myxococcota Polyangia Polyangiales Polyangiales bacterium isolate MFD10278 ilm asm bin20 MGB5546046.1 313 aa
BacClassIA Pseudomonadati Myxococcota Myxococcia Myxococcales Myxococcales bacterium isolate bin861 UCH30049.1 313 aa
BacClassIA Pseudomonadati Myxococcota Polyangia Polyangiales Polyangiales bacterium isolate SMAG U14152 HET6415389.1 ClassIV 313 aa
BacClassIA Pseudomonadati Myxococcota Myxococcia Myxococcales Myxococcales bacterium isolate HKST UBA22 MCA9528840.1 313 aa
BacClassIA Pseudomonadati Myxococcota Polyangia Polyangiales Polyangiaceae bacterium isolate MAG 110 MBC7173602.1 313 aa
BacClassIA Pseudomonadati Myxococcota Myxococcia Myxococcales Myxococcales bacterium isolate CG 2015 01 69 45 NCQ61732.1 370 aa
BacClassIA Pseudomonadati Myxococcota Polyangia Polyangiales Polyangiales bacterium isolate STL MYX24 MFO0694903.1 313 aa
BacClassIA Pseudomonadati Myxococcota Polyangia Polyangiales Polyangiales bacterium isolate BC2.116 MEZ4324835.1 ClassIV 313 aa
BacClassIA Pseudomonadati Myxococcota Polyangia Polyangiales Sandaracinaceae bacterium isolate AalW 18 Q3 R10 53 MAXAC.051 MBK6575374.1 ClassIV 313 aa
BacClassIA Pseudomonadati Myxococcota Polyangia Polyangiales Sandaracinus sp isolate NAT8 MAQ15182.1 ClassIV 313 aa
BacClassIA Pseudomonadati Myxococcota unclassified Myxococcota Myxococcota bacterium isolate P01 A01 bin.20 MEO0321563.1 332 aa
BacClassIA Pseudomonadati Myxococcota unclassified Myxococcota Myxococcota bacterium isolate P01 D01 bin.40 MEM9861620.1 313 aa
BacClassIA Pseudomonadati Myxococcota Polyangia Polyangiales Polyangiales bacterium isolate SGMAG 21 MFK8000154.1 ClassIV 313 aa
BacClassIA Pseudomonadati Myxococcota unclassified Myxococcota Myxococcota bacterium isolate P01 F01 bin.48 MEM9067961.1 314 aa
BacClassIA Pseudomonadati Myxococcota Polyangia Polyangiales Sandaracinus sp isolate HK STAS MYXO 26 MCB9617344.1 ClassIV 316 aa
BacClassIA Pseudomonadati Myxococcota Polyangia Polyangiales Sandaracinus sp isolate NAT131 MAT24279.1 ClassIV 316 aa
BacClassIA Pseudomonadati Thermodesulfobacteriota unclassified Thermodesulfobacteriota Thermodesulfobacteriota bacterium isolate SMAG U12377 HMK64509.1 313 aa
BacClassIA Pseudomonadati Thermodesulfobacteriota Desulfobacterales Desulfobacteraceae Desulfobacteraceae bacterium isolate metabat2.665 RPH87156.1 314 aa
BacClassIA Pseudomonadati Thermodesulfobacteriota unclassified Thermodesulfobacteriota Thermodesulfobacteriota bacterium isolate P53 mat 7 MGQ9857685.1 313 aa
BacClassIA Pseudomonadati Thermodesulfobacteriota Desulfarculia Desulfarculales bacterium isolate Gsp477 bin36 MDR1397991.1 315 aa
BacClassIA Pseudomonadati Thermodesulfobacteriota Desulfarculia Dethiosulfatarculus sandiegensis WP 044346164.1 313 aa
BacClassIA Pseudomonadati Thermodesulfobacteriota Desulfarculia Desulfarculus baarsii WP 013257554.1 313 aa
BacClassIA Pseudomonadati Thermodesulfobacteriota Desulfarculia Desulfarculaceae bacterium isolate D0608M20metaG Bin 142 MGD9124795.1 313 aa
BacClassIA Pseudomonadati Thermodesulfobacteriota Desulfarculia Desulfoferula mesophila WP 338602220.1 313 aa
BacClassIA Pseudomonadati Thermodesulfobacteriota Desulfarculia Desulfocarbo indianensis strain SCBM KMY68191.1 313 aa
BacClassIA Pseudomonadati Thermodesulfobacteriota Desulfarculia Desulfarculus sp isolate S21 MCA1989296.1 313 aa
BacClassIA Pseudomonadati Thermodesulfobacteriota Desulfarculia Desulfarculus sp isolate NC groundwater 1662 Pr3 B 0.1um 67 139 MBI5524418.1 313 aa
BacClassIA Pseudomonadati Thermodesulfobacteriota Desulfarculia Candidatus Adiutricales bacterium isolate SCS8bin.20 MEE9557811.1 313 aa
BacClassIA Pseudomonadati Thermodesulfobacteriota unclassified Thermodesulfobacteriota Thermodesulfobacteriota bacterium isolate MAG148 MEW6261943.1 313 aa
BacClassIA Pseudomonadati Thermodesulfobacteriota unclassified Thermodesulfobacteriota Thermodesulfobacteriota bacterium isolate MAG318 MEW5724000.1 313 aa
BacClassIA Pseudomonadati Thermodesulfobacteriota Desulfarculia Candidatus Adiutrix intracellularis isolate Zx50 bin18 MDR2827157.1 315 aa
BacClassIA Pseudomonadati Thermodesulfobacteriota Desulfarculia Candidatus Adiutrix sp isolate TD114a bin5 MDR2935065.1 315 aa
BacClassIA Pseudomonadati Thermodesulfobacteriota Desulfarculia Candidatus Adiutrix sp isolate Rs511 bin30 MDR3038476.1 316 aa
BacClassIA Pseudomonadati Thermodesulfobacteriota Desulfarculia Candidatus Adiutrix sp isolate Kf353 bin84 MDR1922404.1 313 aa
BacClassIA Pseudomonadati Thermodesulfobacteriota Desulfarculia Candidatus Adiutrix sp isolate Gx485 bin45 MDR1045391.1 313 aa
BacClassIA Pseudomonadati Thermodesulfobacteriota Desulfarculia Candidatus Adiutrix sp isolate TD116 bin19 MDR2946831.1 313 aa
BacClassIA Pseudomonadati Thermodesulfobacteriota Desulfarculia Candidatus Adiutrix sp isolate Hs463 bin11 MDR0881495.1 313 aa
BacClassIA Pseudomonadati Thermodesulfobacteriota unclassified Thermodesulfobacteriota Thermodesulfobacteriota bacterium isolate MAG125 MEA1992274.1 316 aa
BacClassIA Pseudomonadati Thermodesulfobacteriota Thermodesulfobacteria Thermodesulfobacteriales Thermodesulfobacteriaceae bacterium isolate UWMA 0223 HID97633.1 314 aa
BacClassIA Pseudomonadati Thermodesulfobacteriota unclassified Thermodesulfobacteriota Thermodesulfobacteriota bacterium isolate L MaxBin.058 NPA93876.1 315 aa
BacClassIA Pseudomonadati Thermodesulfobacteriota Thermodesulfobacteriota Dissulfuribacteria Dissulfuribacter thermophilus WP 067617323.1 314 aa
BacClassIA Pseudomonadati Thermodesulfobacteriota Thermodesulfobacteria Thermodesulfobacteriales Thermosulfuriphilus ammonigenes WP 220126115.1 315 aa
BacClassIA Pseudomonadati Thermodesulfobacteriota Thermodesulfobacteria Thermodesulfobacteriales Thermodesulfatator autotrophicus WP 068541736.1 315 aa
BacClassIA Pseudomonadati Thermodesulfobacteriota Thermodesulfobacteria Thermodesulfobacteriales Thermosulfurimonas sp F29 WP 255592108.1 315 aa
BacClassIA Pseudomonadati Thermodesulfobacteriota Thermodesulfobacteria Thermodesulfobacteriales Thermodesulfobacteriaceae bacterium isolate SKYB108 MCS7279434.1 315 aa
BacClassIA Pseudomonadati Thermodesulfobacteriota Thermodesulfobacteria Thermodesulfobacteriales Thermodesulfobacterium geofontis WP 013908891.1 315 aa
BacClassIA Pseudomonadati Thermodesulfobacteriota Thermodesulfobacteria Thermodesulfobacteriales Thermodesulfobacterium thermophilum WP 022854671.1 318 aa
BacClassIA Pseudomonadati Thermodesulfobacteriota unclassified Thermodesulfobacteriota Thermodesulfobacteriota bacterium isolate L MetaBat.182 NPA39772.1 318 aa
BacClassIA Pseudomonadati Thermodesulfobacteriota Thermodesulfobacteria Thermodesulfobacteriales Thermodesulfobacterium hydrogeniphilum WP 038057696.1 319 aa
BacClassIA Pseudomonadati Thermodesulfobacteriota Thermodesulfobacteria Thermodesulfobacteriales Caldimicrobium sp isolate Bin S4 MCC6026325.1 315 aa
BacClassIA Pseudomonadati Thermodesulfobacteriota Thermodesulfobacteria Thermodesulfobacteriales Caldimicrobium sp isolate SKYBB hs bin68 MDW8093843.1 313 aa
BacClassIA Pseudomonadati Thermodesulfobacteriota Thermodesulfobacteria Thermodesulfobacteriales Caldimicrobium thiodismutans WP 068513713.1 315 aa
BacClassIA Pseudomonadati Thermodesulfobacteriota Thermodesulfobacteria Thermodesulfobacteriales Caldimicrobium sp isolate 21I 057.bin.9 MFN3920885.1 315 aa
BacClassIA Pseudomonadati Thermodesulfobacteriota Thermodesulfobacteria Thermodesulfobacteriales Caldimicrobium sp isolate 21H 023.bin.13 MFN4197334.1 315 aa
BacClassIA Pseudomonadati Thermodesulfobacteriota unclassified Thermodesulfobacteriota Thermodesulfobacteriota bacterium isolate MAG159 MEW6219652.1 313 aa
BacClassIA Pseudomonadati Thermodesulfobacteriota Desulfobacterales unclassified Desulfobacterales Desulfobacterales bacterium isolate D96SM.bin.51 MCF6291073.1 312 aa
BacClassIA Pseudomonadati Thermodesulfobacteriota Desulfobacteria Desulfobacterales Desulfobacterales bacterium SG8 35 KPJ99404.1 312 aa
BacClassIA Pseudomonadati Thermodesulfobacteriota unclassified Thermodesulfobacteriota Thermodesulfobacteriota bacterium isolate MAG125 MEW6428394.1 314 aa
BacClassIA Pseudomonadati Thermodesulfobacteriota Desulfobulbia Desulfobulbales Desulfobulbaceae bacterium isolate RS 4 17 MCK5437116.1 313 aa
BacClassIA Pseudomonadati Thermodesulfobacteriota unclassified Thermodesulfobacteriota Thermodesulfobacteriota bacterium isolate MAG073 MEN8135684.1 313 aa
BacClassIA Pseudomonadati Thermodesulfobacteriota Desulfobulbia Desulfobulbales Desulfobulbaceae bacterium isolate W9 Combined metabat2 014 NTV13425.1 313 aa
BacClassIA Pseudomonadati Thermodesulfobacteriota Desulfobulbia Desulfobulbales Desulfobulbaceae bacterium isolate 4 1 May SF Bin9 MDH5299467.1 313 aa
BacClassIA Pseudomonadati Thermodesulfobacteriota Desulfobulbia Desulfobulbales Desulfobulbaceae bacterium isolate RS 6 2 MCK5227981.1 313 aa
BacClassIA Pseudomonadati Thermodesulfobacteriota Desulfobulbia Desulfobulbales Desulfobulbaceae bacterium isolate Del10 TBV83444.1 315 aa
BacClassIA Pseudomonadati Thermodesulfobacteriota Desulfobulbia Desulfobulbales Desulfurivibrio sp C05AmB WP 395492759.1 315 aa
BacClassIA Pseudomonadati Thermodesulfobacteriota Desulfobulbia Desulfobulbales Desulfobulbaceae bacterium A2 OQX19704.1 312 aa
BacClassIA Pseudomonadati Thermodesulfobacteriota Desulfobulbia Desulfobulbales Desulfobulbaceae bacterium isolate UBA10518 HBI14317.1 312 aa
BacClassIA Pseudomonadati Thermodesulfobacteriota Desulfobulbia Desulfobulbales Desulfobulbales bacterium isolate HN13.bin.26 MDX1777121.1 312 aa
BacClassIA Pseudomonadati Thermodesulfobacteriota Desulfobulbia Desulfobulbales Desulfobulbaceae bacterium isolate B4G8CH4 05 MDW7774421.1 313 aa
BacClassIA Pseudomonadati Thermodesulfobacteriota Desulfobulbia Desulfobulbales Desulfobulbaceae bacterium isolate SO 2017 LW2 106 MFA5716896.1 308 aa
BacClassIA Pseudomonadati Thermodesulfobacteriota Desulfobulbia Desulfobulbales Desulfobulbaceae bacterium isolate 13 Jan SF Bin14 MDH3348989.1 312 aa
BacClassIA Pseudomonadati Thermodesulfobacteriota Desulfobulbia Desulfobulbales Desulfolithobacter dissulfuricans WP 267928813.1 312 aa
BacClassIA Pseudomonadati Thermodesulfobacteriota Desulfobulbia Desulfobulbales Candidatus Electrothrix sp GW3 4 WP 339137003.1 312 aa
BacClassIA Pseudomonadati Thermodesulfobacteriota Desulfobulbia Desulfobulbales Candidatus Electronema sp TJ WP 417913320.1 313 aa
BacClassIA Pseudomonadati Thermodesulfobacteriota Desulfobulbia Desulfobulbales Desulfogranum japonicum WP 028579823.1 312 aa
BacClassIA Pseudomonadati Thermodesulfobacteriota Desulfobulbia Desulfobulbales Desulfogranum mediterraneum WP 028582941.1 312 aa
BacClassIA Pseudomonadati Thermodesulfobacteriota Desulfobacteria Desulfobacterales Desulfobacterales bacterium isolate DOLJORAL78 47 17 PIE66404.1 312 aa
BacClassIA Pseudomonadati Thermodesulfobacteriota Desulfobulbia Desulfobulbales Desulfogranum marinum WP 205220585.1 312 aa
BacClassIA Pseudomonadati Thermodesulfobacteriota Desulfobulbia Desulfobulbales Desulfogranum aggregans strain JCM 19994 BGS86552.1 312 aa
BacClassIA Pseudomonadati Thermodesulfobacteriota Desulfobulbia Desulfobulbales Desulfobulbus oralis WP 104937143.1 312 aa
BacClassIA Pseudomonadati Thermodesulfobacteriota Desulfobulbia Desulfobulbales Desulfobulbus sp isolate SBF912LR0FQ0009 MGI6657264.1 312 aa
BacClassIA Pseudomonadati Thermodesulfobacteriota Desulfobulbia Desulfobulbales Desulfobulbus sp GCF 031547805.1 WP 310599333.1 312 aa
BacClassIA Pseudomonadati Thermodesulfobacteriota Desulfobulbia Desulfobulbales Desulfobulbus rhabdoformis WP 205228499.1 312 aa
BacClassIA Pseudomonadati Thermodesulfobacteriota Desulfobulbia Desulfobulbales uncultured Desulfobulbus sp GCF 937872135.1 WP 297954582.1 312 aa
BacClassIA Pseudomonadati Thermodesulfobacteriota Desulfobulbia Desulfobulbales Desulfobulbus alkaliphilus WP 205241670.1 312 aa
BacClassIA Pseudomonadati Thermodesulfobacteriota Desulfobulbia Desulfobulbales Desulfopila sp isolate S6 bin.40.strict MFN2353261.1 312 aa
BacClassIA Pseudomonadati Thermodesulfobacteriota Desulfobulbia Desulfobulbales Desulfopila sp isolate bin.795 MEE4242235.1 312 aa
BacClassIA Pseudomonadati Thermodesulfobacteriota Desulfobulbia Desulfobulbales Desulfopila sp isolate SMAG U9206 HKJ66220.1 312 aa
BacClassIA Pseudomonadati Thermodesulfobacteriota Desulfobulbia Desulfobulbales Desulfocapsaceae bacterium isolate MO 192.B14 MDJ0621376.1 312 aa
BacClassIA Pseudomonadati Thermodesulfobacteriota Desulfobulbia Desulfobulbales Desulfopila inferna WP 205234906.1 312 aa
BacClassIA Pseudomonadati Thermodesulfobacteriota Desulfobulbia Desulfobulbales Desulfoprunum sp isolate C5 metabat.bin.56 MDP3480351.1 312 aa
BacClassIA Pseudomonadati Thermodesulfobacteriota Desulfobulbia Desulfobulbales Desulfoprunum benzoelyticum WP 183351594.1 312 aa
BacClassIA Pseudomonadati Thermodesulfobacteriota Desulfobulbia Desulfobulbales Desulfobulbaceae bacterium isolate UBA8130 HBT96479.1 312 aa
BacClassIA Pseudomonadati Thermodesulfobacteriota Desulfobulbia Desulfobulbales Desulfobulbaceae bacterium isolate Rs511 bin14 MDR3088681.1 312 aa
BacClassIA Pseudomonadati Thermodesulfobacteriota Desulfobulbia Desulfobulbales Desulfobulbaceae bacterium isolate Crp329 bin2 MDR0476694.1 312 aa
BacClassIA Pseudomonadati Thermodesulfobacteriota Desulfobulbia Desulfobulbales Desulfocapsaceae bacterium isolate MAG CA64 MDR3630035.1 312 aa
BacClassIA Pseudomonadati Thermodesulfobacteriota Desulfobulbia Desulfobulbales Desulfocapsaceae bacterium isolate bin.691 MEE4166420.1 312 aa
BacClassIA Pseudomonadati Thermodesulfobacteriota Desulfobulbia Desulfobulbales Desulfofustis sp PB SRB1 MBL0379157.1 313 aa
BacClassIA Pseudomonadati Thermodesulfobacteriota Desulfobulbia Desulfobulbales Desulfofustis limnaeus WP 354005687.1 313 aa
BacClassIA Pseudomonadati Thermodesulfobacteriota Desulfobulbia Desulfobulbales Desulfocapsaceae bacterium isolate MFD01352 ilm asm bin7 MGA7278241.1 312 aa
BacClassIA Pseudomonadati Thermodesulfobacteriota Desulfobulbia Desulfobulbales Desulfofustis sp isolate Spart 067 MAG MEJ2137252.1 312 aa
BacClassIA Pseudomonadati Thermodesulfobacteriota Desulfobulbia Desulfobulbales Candidatus Desulfatifera sulfidica MBC8208472.1 312 aa
BacClassIA Pseudomonadati Thermodesulfobacteriota Desulfobacteria Desulfobacterales Desulfoarculaceae bacterium isolate KA05 MBW6519496.1 312 aa
BacClassIA Pseudomonadati Thermodesulfobacteriota unclassified Thermodesulfobacteriota Thermodesulfobacteriota bacterium isolate MAG013 MEN8188590.1 312 aa
BacClassIA Pseudomonadati Thermodesulfobacteriota Desulfobulbia Desulfobulbales Desulfocapsa sulfexigens WP 015404718.1 312 aa
BacClassIA Pseudomonadati Thermodesulfobacteriota Desulfobulbia Desulfobulbales Desulfocapsa sp isolate MAG 30 RUM43995.1 312 aa
BacClassIA Pseudomonadati Thermodesulfobacteriota Desulfobulbia Desulfobulbales Desulfopila sp IMCC35008 WP 163339624.1 312 aa
BacClassIA Pseudomonadati Thermodesulfobacteriota Desulfobulbia Desulfobulbales Desulfosediminicola flagellatus WP 136805421.1 312 aa
BacClassIA Pseudomonadati Thermodesulfobacteriota Desulfobulbia Desulfobulbales Desulfopila sp isolate VLC 059 MFV0438659.1 312 aa
BacClassIA Pseudomonadati Thermodesulfobacteriota Desulfobulbia Desulfobulbales Desulfopila aestuarii WP 073614954.1 312 aa
BacClassIA Pseudomonadati Thermodesulfobacteriota Desulfobulbia Desulfobulbales Desulfotalea sp isolate tPB Delt2 MGL1930135.1 312 aa
BacClassIA Pseudomonadati Thermodesulfobacteriota Desulfobulbia Desulfobulbales Desulfotalea psychrophila WP 011189975.1 313 aa
BacClassIA Pseudomonadati Thermodesulfobacteriota Desulfobulbia Desulfobulbales Desulfobulbaceae bacterium isolate RS 2 19 MCK5516803.1 312 aa
BacClassIA Pseudomonadati Thermodesulfobacteriota Desulfobulbia Desulfobulbales Desulforhopalus vacuolatus WP 205219123.1 313 aa
BacClassIA Pseudomonadati Thermodesulfobacteriota Desulfobulbia Desulfobulbales Desulfomarina profundi WP 228856347.1 312 aa
BacClassIA Pseudomonadati Thermodesulfobacteriota Desulfobulbia Desulfobulbales Desulforhopalus sp isolate SMAG U16051 HSL39018.1 312 aa
BacClassIA Pseudomonadati Thermodesulfobacteriota Desulfobulbia Desulfobulbales Desulforhopalus sp isolate MFD12129 ilm asm bin8 MGB3223801.1 312 aa
BacClassIA Pseudomonadati Thermodesulfobacteriota Desulfobulbia Desulfobulbales Desulfopila sp IMCC35006 WP 136811386.1 312 aa
BacClassIA Pseudomonadati Thermodesulfobacteriota Desulfobulbia Desulfobulbales Desulforhopalus sp isolate EBRS2.bin23 MFW2364782.1 312 aa
BacClassIA Pseudomonadati Thermodesulfobacteriota Desulfobulbia Desulfobulbales Desulforhopalus singaporensis WP 176761103.1 312 aa
BacClassIA Pseudomonadati Thermodesulfobacteriota Desulfobulbia Desulfobulbales Desulforhopalus sp isolate 3300055726 39 MFT5726421.1 312 aa
BacClassIA Pseudomonadati Thermodesulfobacteriota Desulfobulbia Desulfobulbales Desulforhopalus sp IMCC35007 WP 136872363.1 312 aa
BacClassIA Pseudomonadati Thermodesulfobacteriota unclassified Thermodesulfobacteriota Thermodesulfobacteriota bacterium isolate SMAG U15953 HSO71078.1 323 aa

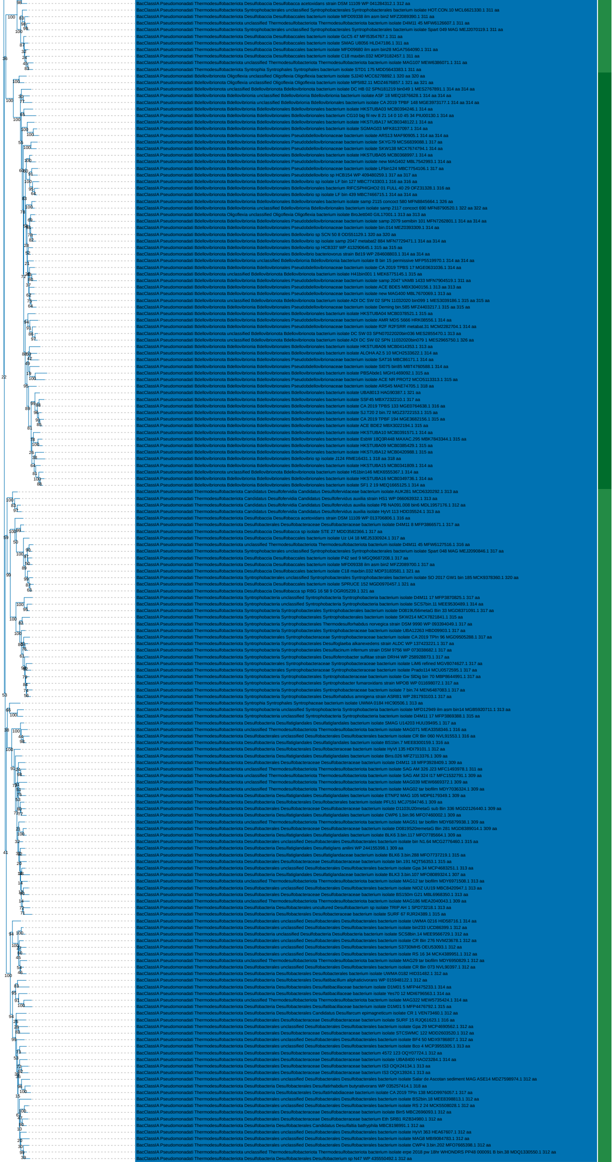

Supplement: Supplement 5 [file media-5.pdf]
